# Adaptive Evolution and Divergence of *SERPINB3*: A Young Duplicate in Great Apes

**DOI:** 10.1371/journal.pone.0104935

**Published:** 2014-08-18

**Authors:** Sílvia Gomes, Patrícia I. Marques, Rune Matthiesen, Susana Seixas

**Affiliations:** 1 Institute of Molecular Pathology and Immunology of the University of Porto (IPATIMUP), Porto, Portugal; 2 Institute of Biomedical Sciences Abel Salazar (ICBAS), University of Porto, Porto, Portugal; 3 National Health Institute Doutor Ricardo Jorge (INSA), Lisboa, Portugal; University of Bern, Switzerland

## Abstract

A series of duplication events led to an expansion of clade B Serine Protease Inhibitors (*SERPIN*), currently displaying a large repertoire of functions in vertebrates. Accordingly, the recent duplicates *SERPINB3* and *B4* located in human 18q21.3 *SERPIN* cluster control the activity of different cysteine and serine proteases, respectively. Here, we aim to assess SERPINB3 and B4 coevolution with their target proteases in order to understand the evolutionary forces shaping the accelerated divergence of these duplicates. Phylogenetic analysis of primate sequences placed the duplication event in a Hominoidae ancestor (∼30 Mya) and the emergence of *SERPINB3* in Homininae (∼9 Mya). We detected evidence of strong positive selection throughout *SERPINB4*/*B3* primate tree and target proteases, cathepsin L2 (*CTSL2*) and G (*CTSG*) and chymase (*CMA1*). Specifically, in the Homininae clade a perfect match was observed between the adaptive evolution of *SERPINB3* and cathepsin S (*CTSS*) and most of sites under positive selection were located at the inhibitor/protease interface. Altogether our results seem to favour a coevolution hypothesis for *SERPINB3*, *CTSS* and *CTSL2* and for *SERPINB4* and *CTSG* and *CMA1*. A scenario of an accelerated evolution driven by host-pathogen interactions is also possible since SERPINB3/B4 are potent inhibitors of exogenous proteases, released by infectious agents. Finally, similar patterns of expression and the sharing of many regulatory motifs suggest neofunctionalization as the best fitted model of the functional divergence of *SERPINB3* and *B4* duplicates.

## Introduction

Proteolysis is involved in the regulation of numerous biological processes being fundamental in every cell and organisms. The activity of proteases is regulated by a complex network of inhibitory molecules and different human pathologies such as arthritis, cancer, neurodegenerative and cardiovascular diseases can be associated with the deleterious effects of uncontrolled proteolysis. Thus, the regulation of endogenous proteases is crucial in the maintenance of organisms' homeostasis and health status [1], [2].

Serine protease inhibitors (SERPINs) are key elements in the regulation of proteolytic pathways, controlling the activity of serine proteases and helping to prevent from the pernicious effect of excessive proteolysis [1]. Some SERPINs can also inhibit cysteine proteases, acting as cross-class SERPINs, while others lost their inhibitory activity and developed other functions as serving as hormone carriers or chaperones [1], [3], [4]. SERPIN superfamily members share a conserved tertiary structure [5] with an exposed reactive center site loop (RCL), which carries the protease recognition site and acts as a pseudo-substrate determining protease specificity [6]. Inhibitory SERPINs regulate protease activity through a unique suicide mechanism where the RCL binds to the protease and is then cleaved between P1 and P1′ (scissile bond) residues resulting in the formation of a covalent complex that irreversibly locks both SERPIN and protease [5], [7].

Vertebrate SERPINs exhibit distinct exon-intron patterns [8] and segregate evolutionary into nine clades (A-I) [1]. The clade B SERPINs differ from other SERPINs by the absence of a signal peptide and by the occurrence of an additional polypeptide loop between helices C and D (CD-loop) present in most members [1]. Their localization in the cells is limited to cytoplasm and/or nuclear compartments where SERPINBs play a cytoprotective role through the inhibition of proteases involved in cell death [3], [4]. However, several SERPINBs (SERPINB2, B3, B5 and B7) [6] can be released from cells under certain conditions, which in most cases is thought to result from passive cell loss or lysis [1], [4]. Moreover, it has become apparent that these proteins participate alone or in concert with other molecules in the regulation of intricate proteolytic cascades implicated in tumor suppression, apoptosis, inflammation and angiogenesis, among others, through complex and still-obscure mechanisms [1], [9], [10].

At the gene level, *SERPINBs* share a similar structure comprising seven-eight exons with a translational starting site at exon II and the RCL located in the last exon [1]. In humans, *SERPINB* genes are organized in tandem at 6p25 (*SERPINB1*, *B6* and *B9*) [11], [12] and 18q21.3 (*SERPINB2*, *B3*, *B4*, *B5*, *B7*, *B8*, *B10*, *B11*, *B12* and *B13*) chromosomes [13], [14]. Comparative genomics of the human, mouse, chicken and zebrafish sequences indicates that *SERPINB* genes undergone an expansion throughout vertebrate evolution by a series of duplication events [15], [16].

In the SERPIN superfamily, events of gene duplication are likely to underlie the functional diversification of the inhibitory repertoire of these proteins [16]. Such phenomenon is well illustrated *in vitro* by mouse homologues *Serpinb3a-d*, while Serpinb3a inhibits both chymotrypsin-like serine proteases and papain-like cysteine proteases [17], Serpinb3b inhibits both papain-like cysteine proteases and trypsin-like serine proteases and no inhibitory activity was detected for Serpinb3c and Serpinb3d [16]. Likewise, the human homologs *SERPINB3* and *B4* (formerly known as squamous cell carcinoma antigen 1 (SCCA1) and 2 (SCCA2) respectively), share a sequence identity of 92% and regulate the activity of distinct proteases and *in vitro* experiments demonstrate that SERPINB3 targets cysteine proteases such as the cathepsins L1, L2, K and S (CTSL1, CTSL2, CTSK and CTSS) [18], [19] whereas SERPINB4 is a potent inhibitor of the serine proteases cathepsin G (CTSG) and mast cell chymase (CMA1) and a poor inhibitor of CTSS when compared with SERPINB3 (50 times less efficient) [20].

In a healthy state SERPINB3 and B4 play a major role in cell protection against cytotoxic molecules mainly through the inhibition of CTSS that may leak into the cytoplasm as a result of lysosome failure [4], [21], [22]. Conversely, in cancer disease SERPINB3 was found to inhibit apoptosis, circumventing the mechanism of cell death and favouring tumour growth and metastization [23]–[25]. Indeed, the overexpression of SERPINB3 in some types of squamous cell carcinomas, namely uterine cervix carcinoma, esophagus carcinoma, head and neck carcinomas, breast carcinoma and hepatocellular carcinoma is correlated with a poor prognosis [9]. For this reason, SERPINB3 and B4 have been regarded as important serum biomarkers used for the diagnostic and prognostic of squamous cell carcinomas [26]. Moreover, SERPINB3 is also up-regulated in patients suffering from systemic sclerosis, psoriasis, bronchitis and pneumonia [4], [27] and reduced in patients with hepatitis C infection and untraceable in patients with systemic lupus erythematosus [28].

Besides the role in cancer and autoimmunity, SERPINB3 and B4 have a dual role in the immune response to pathogens. Recent studies have shown that SERPINB3 may act as a surface receptor for the binding of hepatitis B virus to hepatocytes and to peripheral blood mononuclear cells [29]–[31]. In contrast, SERPINB3 and B4 can also target extrinsic proteases derived from several pathogens suggesting a protective role against the deleterious effects of several pathogenic organisms [32], [33].

Interestingly, *SERPINB3* and *B4* were previously identified as an example of young gene duplicates under positive selection in the hominid lineage [34]. Duplication events are regarded as an important source of innovation underlying the onset of gene families from a single ancestral gene and contributing to the increase of complexity in the eukaryotic genomes [35]. Two alternative models are frequently used to explain the evolution and retention of duplicate genes in the genomes. The neofunctionalization model [36] that claims the gain of a novel function by a gene copy as the main reason for the retention of duplicates in the genome [37]. The subfunctionalization model [38] on the other hand, predicts lower selective constraints affecting equally both duplicates in a way that neither copy is sufficient to perform the original function, and both copies are maintained in the genomes [37].

Here, we combine phylogenetic based tests and protein structural analysis to assess the evolution of *SERPINB3* and *B4* and their target proteases in the view of understanding the selective forces shaping the divergence of *SERPINB3* and *B4* duplicates and its potential implications for human health and disease. Results suggest that *SERPINB3* duplicate is evolving under positive selection supporting the functional divergence observed in several experimental studies.

## Materials and Methods

### Sequence data

Genomic DNA sequences for *SERPINB3*, *SERPINB4*, *CTSS* (Cathepsin S), *CTSL1* (Cathepsin L1), *CTSL2* (Cathepsin L2), *CTSK* (Cathepsin K), *CTSG* (Cathepsin G) and *CMA1* (Chymase) were retrieved from the National Center for Biotechnology Information database (NCBI) (http://www.ncbi.nlm.nih.gov) and University of California Santa Cruz (USCS) Genomic Bioinformatics database (http://genome.ucsc.edu/) for the following primate species: human (*Homo sapiens*), common chimpanzee (*Pan troglodytes*), gorilla (*Gorilla gorilla*), Sumatran orangutan (*Pongo abelli*), northern white-cheeked gibbon (*Nomascus leucogenys*), rhesus macaque (*Macaca mulatta*), olive baboon (*Papio anubis*), marmoset (*Callithrix jacchus*) and squirrel monkey (*Saimiri boliviensis*) (see Table S1). In the case of *G. gorilla*, to fill the large sequence gaps affecting *SERPINB4* and *CTSS* coding region, we amplified, by polymerase chain reaction (PCR), and sequenced a *G. gorilla* sample (EB(JC) from the primate DNA panel of the European Collection of Cell Cultures (ECACC). We used MultiPipMaker [39] to build multiple sequence alignments and the human *SERPINB3*, *SERPINB4*, *CTSS*, *CTSK*, *CTSL1*, *CTSL2*, *CTSG* and *CMA1* sequences were used to annotate for gene content in the collected sequences of other primate species. RepeatMasker (http://www.repeatmasker.org/) was used to detect repetitive sequences. Sequence editing and exon assembly were performed using Bioedit (7.0.9.1) [40].

### Phylogenetic analysis and selection tests

We used CLUSTALW [41] implemented in the MEGA5 [42] software to align the cDNA sequences of *SERPINB3*, *SERPINB4*, *CTSS*, *CTSL1*, *CTSL2*, *CTSK*, *CTSG* and *CMA1*. Phylogenetic trees were then constructed using neighbour-joining method with 10000 bootstraps implemented in MEGA5.

The nonsynonymous/synonymous substitution rate ratio (d_N_/d_S_ = ω) was estimated using the maximum likelihood (ML) framework implemented in the program CODEML of Phylogenetic Analysis by Maximum Likelihood (PAML) software [43]. We used ω values to investigate the selective pressures that have shaped the evolution of *SERPINB3* and *B4* duplicates and their known targets *CTSS*, *CTSL1*, *CTSL2*, *CTSK*, *CTSG* and *CMA1*. We used three likelihood ratio test (LTR) approaches to detect genes under positive selection: first the branch model evaluates the strength of natural selection in one or more phylogenetic clades and compares a single ω value obtained for all lineages (M0) with a model assuming different ω values for each lineage (free-ratio); second, the site models, which allows the ω values to vary among sites of the protein and compares the neutrality models M1a and M7 against the positive selection models M2a and M8, respectively; third, the branch-site model was used to identify codons under positive selection within a phylogenetic clade that compares the null model, with a fixed ω = 1 for all the sites in the background, with the alternative model, assuming a ω>1 for all the sites in the foreground [44]. In all cases, the significance of the models was carried out using the likelihood ratio test -2Δl with a χ^2^ distribution [43], [44]. The Bayes Empirical Bayes (BEB) approach is implemented to identify amino acids under positive selection [45]. For ω calculation, sequences associated with species-specific stop codons were removed.

### Protein modelling and docking

The three-dimensional (3D) structures of SERPINB3 (2ZV6), CTSS (2FQ9), CTSL1 (2XU3), CTSL2 (1FH0), CTSLK (3KWZ), CTSG (1CGH) and CMA1 (4AG1) proteins were obtained from Protein Data Base (PDB) (http://www.rcsb.org). In the case of SERPINB4, the 3D structure was predicted by homology modeling in MODELLER 9.10 software using SERPINB3 as template [46]. Structure validation was performed with PROCHECK [47] available in SWISS-MODEL web server [48]. After, to assess the possible functional significance of specific amino acids replacements between SERPINB3 and B4 in the target protease affinity, the obtained 3D structures were used to generate 3D structural models of inhibitor-protease complexes using the HADDOCK docking web server [49] (http://haddock.science.uu.nl). The published binding residue pairs, namely the P1 and P1′ residues, from SERPINB3 and B4, and the amino acids that form the catalytic triad of target proteases, at the interface region of the inhibitor-protease complex, were used to drive the docking process. Visualization of the 3D structures was performed in PyMol 0.99rc6 [50]. The models were evaluated according to the HADDOCK score [51], interface root mean square deviation (iRMSD) and ligand root mean square deviation (lRMSD) [52].

### Tissue expression screening of *SERPINB3* and *SERPINB4*

A set of 21 human cDNA samples from different healthy organs was used to study the tissue pattern of *SERPINB3* and *B4* expression. Except for the first-strand cDNA from leukocytes (Clontech), the RNA from the First Choice Human Total RNA Survey Panel (Ambion) was used as a template to generate cDNA by RT- PCR using a Superscript III system (Life Technologies). PCR amplification was performed using the primers 5′ – TGTAGGACTCCAGATAGCAC – 3′ and 5′- TGTAGGACTTTAGATACTGA – 3′, designed to be unique to the target *SERPINB3* and *B4* cDNA, respectively, and primer 5′ - TGGAAATACCATACAAAGGCA – 3′. *GAPDH* was employed as control using primers 5′ - TCAAGGCTGAGAACGGGAAG - 3′ and 5′ - AGAGGGGGCAGAGATGATGA - 3′ for amplification (see Fig. S1).

## Results

### Reconstructing the origin of *SERPINB3* and *SERPINB4* duplicates

**Figure 1:**
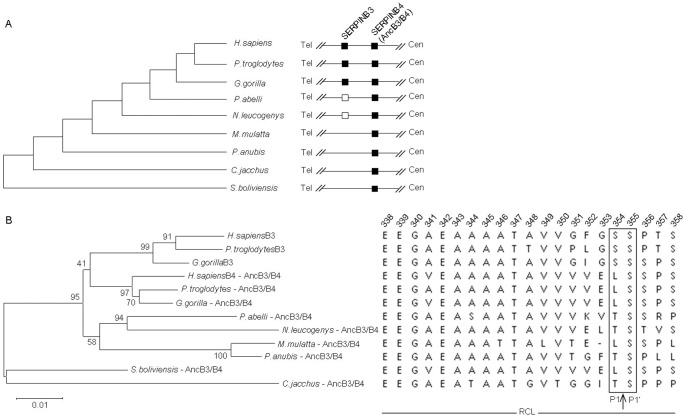
Origin of *SERPINB3* and *SERPINB4* duplicates. A) The organization of *SERPINB3* and *SERPINB4* loci in human and eight non-human primates. Relative position to telomere (Tel) and centromere (Cen) is shown. Solid boxes represent functional genes; open boxes represent pseudogenes. B) Phylogenetic tree of *SERPINB3* and *SERPINB4* genes with the bootstrap percentages shown at interior nodes and the alignment of RCL regions (P17-P4′). The canonical scissile bond is marked by an arrow and a standard P1 and P1′ nomenclature is used to number amino acid positions N- and C-terminal outward from the scissile bond. AncB3/B4: ancestral SERPINB3/B4 gene.

The chromosomal regions of *SERPINB3* and *B4* from *H. sapiens*, *P. troglodytes*, *G. gorilla*, *P. abelli*, *N. leucogenys*, *M. mulatta*, *P. anubis*, *C. jacchus* and *S. boliviensis* were downloaded from the USCS and NCBI databases or obtained by direct sequencing. The *SERPINB3* and *B4* sequences were retrieved from the human reference sequence of the chromosome 18 (assembly GRCh37,) in a large genomic segment delimited by *SERPINB7* and *SERPINB12* (chr18: c61429197-61222431) and aligned with the homologous sequences from non-human primates (see Table S1). Overall, sequence alignments revealed a conserved pattern of seven coding exons in primates for *SERPINB3* and *B4* (Fig. S2). However in *M. mulatta*, *P. anubis*, *C. jacchus* and *S. boliviensis* one of the duplicates was absent (Fig. 1A). In addition, the analysis of the predicted cDNA and protein sequences revealed that *P. abelli* and *N. leucogenys* telomeric duplicates have a premature stop codon in positions 60 and 19, respectively, causing any resulting protein to be abnormally shortened and suggesting that these duplicates are in fact pseudogenes.

The phylogenetic tree constructed using functional *SERPINB3* and *B4* sequences, places the duplication event before the divergence of *H. sapiens*, *P. troglodytes* and *G. gorilla* (Fig. 1B). However, the finding of non-functional gene copies in *P. abelli* and *N. leucogenys* species suggests that a duplication event occurred in a common ancestor of Hominoidae (great apes), after the separation from the Old World monkeys 29.6 million years (MY) ago. Interestingly, the protein alignments obtained for the RCL region in the different primate species suggest the existence of an ancestral SERPINB3/B4 (AncB3/4) with two possible scissile bond (P1-P1′) compositions either TS or LS (Fig. 1B). The presence of a SS scissile bond, suggests that the telomeric gene, named *SERPINB3* in humans, arose recently in evolution (about 9 MY ago in Hominidae) as the result of duplication and functional divergence. Noteworthy, SERPINB3 accumulated several other differences in the RCL region which are likely to have contributed to a shift in its protease affinity.

### Adaptive evolution of *SERPINB3*

We performed a maximum likelihood (ML) analysis, using codeml package in PAML software, to test whether the functional divergence of *SERPINB3* is a result of positive selection [43], [44]. Initially, we estimated the ω ratio for the entire phylogeny (M0 model) and the independent ω ratio for each branch to assess and characterize the selective pressures acting on *SERPINB3*/*B4* evolution. Overall, the M0 model shows a low value of ω for the entire phylogeny (ω≈0.67) suggesting a conserved evolution (ω<1). Also, the comparison of M0 versus the free-ratio (−2ΔlnL = 16.18, p>0.05) suggest that the different lineages experienced similar evolutionary rates. However, this result is not unexpected, since averaging across all sites is not a powerful test of adaptive evolution. Hence, we used likelihood ratio tests to compare nested models with and without positive selection to look for evidence of site-specific positive selection in *SERPINB3*/*B4* phylogeny. The comparisons of M1a (nearly neutral) versus M2a (positive selection) and M7 (beta) versus M8 (beta and ω>1) show significant (p<0.001) evidence of positive selection for *SERPINB3* and *B4* genes (Table 1). For M2a and M8 models, the BEB analysis identified the same 17 sites under adaptive evolution (ω>1) with high posterior probability (p>90%) (Table 1).

**Table 1:** Maximum likelihood estimates of positive selection for SERPINB3/B4 phylogeny.

Phylogeny	N	M1avs. M2a	M7vs. M8	Proportion of sites ω>1	Positively selected sites ^a^
*SERPINB3/B4*	9	88.97**	89.35**	ω = 7.12, p = 0.01	17Q, **24E**, 163I, 166G, 167N, 171N, **279R**, **321R**, **324V**, **351G**, **352F**, **353G**, **354S**, 356P, 357T, 358S, 364H

Likelihood ratio tests (−2Δ*l*) comparing a null and positive selection models (M1a vs M2a, M7 vs M8); N, number of primate species with sequences in alignment; p: proportion of sites under positive selection in M8 model; ω: estimate the dN/dS of the sites under selection in M8 model; ^a^ Amino acid sites found to be under positive selection with posterior probabilities greater than 90% (blank), 95% (underlined) or 99% (bold) in the BEB analysis. The reference sequence is human SERPINB3.

** Significance with p<0.001.

To test if this signal of positive selection could be connected with the appearance of *SERPINB3* we used the branch-site model test. This test allows the ω ratio to vary among sites in the protein and across branches in the tree to detect if positive selection was affecting sites along specific lineages. In the *SERPINB3*/*B4* tree the likelihood ratio tests, based on the branch-site models, were significant (p<0.01) only for the foreground branch 1 (Fig. S3), which includes the lineages from *H. sapiens*, *P. troglodytes* and *G. gorilla* for the *SERPINB3* duplicate (Table 2). Although most sites are under constrained evolution, the residues 327G, 351G and 352F were identified by the BEB analysis as being under positive selection (p>80%) in the *SERPINB3* clade (foreground branch 1).

**Table 2:** Likelihood ratio test for branch-site model for SERPINB3/B4 phylogeny.

Phylogeny	Parameter estimates Foreground vs. Background	−2Δ*l*	Positively selected sites
*SERPINB3/B4* Foreground 1	p_0_ = 0.612, p_1_ = 0.376, p_2a_ = 0.008, p_2b_ = 0.005, ω_0_ = 0.018, ω_1_ = 1.000, ω_2_ = 19.207	6.10**	327G, 351G, 352F
*SERPINB3/B4* Foreground 2	p_0_ = 0.630, p_1_ = 0.369, p_2a_ = 0.000, p_2b_ = 0.000, ω_0_ = 0.023, ω_1_ = 1.000, ω_2_ = 1.000	0	NA

−2Δ*L*, likelihood ratio test to detect positive selection with 1 degree of freedom; Foreground 1: *H. Sapiens* B3, *P. Troglodytes* B3 and *G. Gorilla* B3 lineages; Foreground 2: *H. Sapiens* B4, *P. Troglodytes* B4 and *G. Gorilla* B4 lineages. Amino acid sites found to be under positive selection with posterior probabilities greater than 80% (blank) are displayed; NA, not applicable because the neutral model fits better than positive selection.

** Significance with p<0.01.

**Figure 2:**
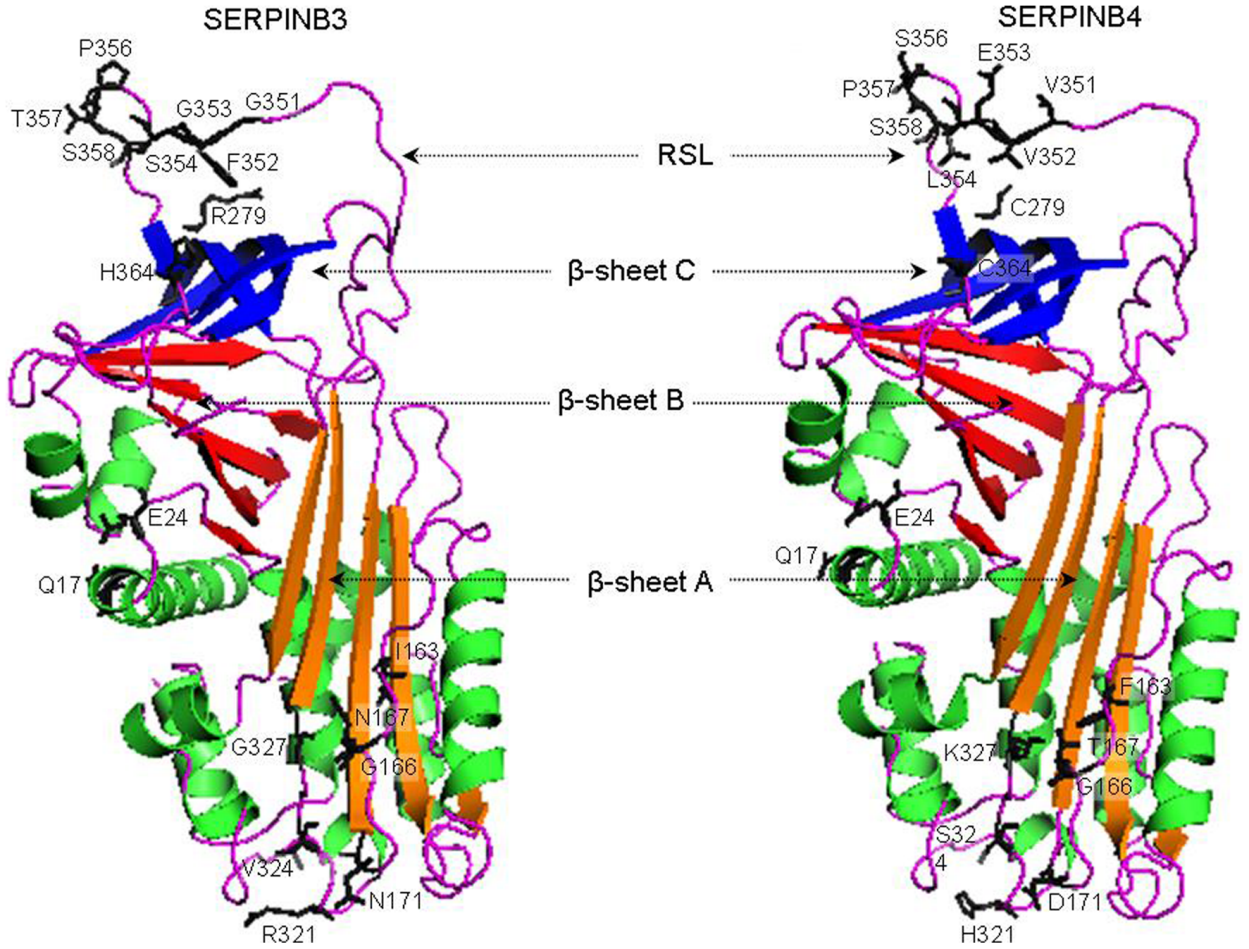
X-ray structure of SERPINB3 and predicted structure of SERPINB4. The A β-sheet (shutter) is in orange, B β-sheet (breach) is in red and C β-sheet (gate) is in blue. Helices are shown in green. RCL: reactive center loop. Sites under positive selection are in black.

Finally, to evaluate the structural basis of the positive selection signatures detected by the ML analyses, we compared SERPINB3 and B4 3D structures. However, since the SERPINB4 3D structure was not available in the surveyed databases, we used MODELLER software to calculate a homology model of SERPINB4 using the crystal structure coordinates of SERPINB3 as template (Fig. 2). Structural superimposition of the modelled SERPINB4 structure with the SERPINB3 template showed a very low root mean square deviation (RMSD) of 0.22 Å, which reveals a quite similar protein backbone.

From the 17 sites under positive selection identified by the site-model analysis, seven correspond to differences in the RCL from SERPINB3 and B4 mainly V351G, V352F, E353G, L354S, S356P, P357T and C364H (Fig. 2). As mentioned above, the RCL is a crucial region for the interaction with the target proteases being responsible for the functional SERPIN specificity, in which these 7 residues are likely to have a significant effect. Also, residue C279R is located at β-sheet C, in the gate domain (Fig. 2), a important region for the full insertion of RCL after protease cleavage [53]. Thus, amino acid alterations in this region could affect the RCL insertion and the SERPIN inhibitory mechanism. Finally, from the remaining eight sites under positive selection, six residues cluster together at the distal end of RCL (Fig. 2). Once inserted inside the molecule the RCL presses the target protease against the bottom of the SERPIN resulting in the distortion of the protease active site, greatly reducing the enzyme catalytic activity [5]. Consequently, amino acids positioned at the distal end of the RCL are in close proximity to the inhibited protease and substitutions in these sites are probably implicated in the stability of the inhibitor-protease complex.

Furthermore, branch-site model analysis identified the amino acid K327G and the RCL V351G and V352F residues as being under positive selection in *SERPINB3* duplicate for *H. sapiens*, *P. troglodytes* and *G. gorilla* lineage. In the case of SERPINB3, amino acids 351G and 352F are located in the RCL, very close to the 354S/355S scissile bond, and may have a relevant functional role in the specificity of SERPINB3 towards cysteine target proteases and in its functional divergence from SERPINB4. Amino acid 327G is located in the highly conserved β-sheet A in the shutter domain (Fig. 2) that has a key role in SERPIN suicide mechanism. Once cleaved by a protease the exposed RCL undergoes drastic conformational alterations ending inside of the SERPIN, inserted into the β-sheet A region. As a result, many of the RCL become buried with a major impact in the rate of RCL insertion [5]. Since the RCL of SERPINB3 and B4 differ in their amino acid compositions, the substitution of a polar residue, lysine (SERPINB4) by a stereochemically different glycine (SERPINB3) could be of crucial importance for an efficient insertion of SERPINB3 RCL.

### Target protease evolution

Furthermore, maximum likelihood approaches were used to address the evolutionary signatures of SERPINB3 and B4 target proteases and to check for similar evolutionary paths that could point to a possible coevolution process between inhibitor and target proteases mainly *CTSS*, *CTSL1*, *CTSL2*, *CTSK*, *CTSG* and *CMA1*. As for *SERPINB3*/*B4* phylogeny, the one ratio (M0) model tests reveal a ω<1 suggesting an overall conserved evolution for the *CTSS*, *CTSL1*, *CTSL2*, *CTSK* and *CMA1* phylogenies. However, *CTSG* shows higher ω ratios (ω≈0.98), which suggests a relaxation in the selective constrains. Also, the comparison of M0 versus the free-ratio model indicates that the different lineages experienced similar evolutionary rates, except for *CTSS* gene (Table 3) in which selective pressures may differ across *CTSS* tree branches. We then proceeded to more powerful and robust approaches to test for evidence of site-specific positive selection across the entire phylogeny or within a specific phylogenetic clade for *CTSS*, *CTSL1*, *CTSL2*, *CTSK*, *CTSG* and *CMA1*. The comparisons of M1a (nearly neutral) versus M2a (positive selection) and M7 (beta) versus M8 (beta and ω>1) show that *CTSL2*, *CTSG* and *CMA1* genes are under positive selection (Table 3) and several codons were identified as subject to positive selection. Interestingly, in a previous work both *CTSG* and *CMA1* were shown to be under positive selection in mammalians, possibly as a result of a trade-off between increased response to pathogens and decreased risk of autoimmunity by apoptosis related genes [54]. Furthermore, branch-site models were used to detect if positive selection was affecting sites along specific clades in *CTSS*, *CTSL1*, *CTSL2*, *CTSK*, *CTSG* and *CMA1* phylogeny and establish whether selective pressures varied in a similar way as for *SERPINB3*/*B4* gene tree suggesting inhibitor/target coevolution. Interestingly, we found evidence of positive selection (p<0.05) for *CTSS* gene (Table 4), when comparing the foreground *H. sapiens*, *P. troglodytes* and *G. gorilla* clade with the background phylogeny (Fig. S4) and we detected residue 255R as being under positive selection (p>90%). Therefore, positive selection might be acting in *SERPINB3* duplicate and *CTSS* for *H. sapiens*, *P. troglodytes* and *G. gorilla* lineage which can point to a possible coevolution between inhibitor and target protease. No statistical significance was obtained for the *H. sapiens*, *P. troglodytes* and *G. gorilla* clade (foreground) in the remaining branch-site tests (*CTSL1*, *CTSL2*, *CTSK*, *CTSG* and *CMA1*).

**Table 3:** Phylogenetic tests of positive selection for target proteases.

	Target	M0 ω^a^	M0vs. Free-ratio	M1avs. M2a	M7vs. M8	Proportion of sites ω>1	Positively selected sites^c^
*SERPINB3*	*CTSS*	0.22	22.35*	2.72 (2d.f.)	2.74 (2d.f.)	-	NA
	*CTSL1*	0.35	17.93	1.74 (2d.f.)	1.91 (2d.f.)	-	NA
	*CTSL2*	0.43	9.71	14.95** (2d.f.)	15.13** (2d.f.)	ω^b^ = 5.66 p = 0.02	171R, 185R, **226T**, 229A, 292N, 333N
	*CTSK*	0.19	18.89	3.64 (2d.f.)	4.76 (2d.f.)	-	NA
*SERPINB4*	*CTSG*	0.98	11.06	45.82** (2d.f.)	46.11** (2d.f.)	ω^b^ = 7.12 p = 0.02	25R, 44A, 46Q, 47S, **69N**, 141L, **152M**, **170R**, 173L, **177G**, 182P, 191R, 196A, 220K, **221S**
	*CMA1*	0.60	21.80	12.58* (2d.f.)	12.62* (2d.f.)	ω^b^ = 4.47 p = 28.58	12L, 50F, 131F, 158G, **222S**

−2Δ*L*, likelihood ratio test to detect positive selection; p: proportion of sites under positive selection for M8 model; ω^a^: dN/dS estimate for M0; ω^b^: dN/dS estimate for M8 model; ^c^Positively selected sites identified by M8 model: amino acid sites found to be under positive selection with posterior probabilities greater than 90% (blank), 95% (underlined) or 99% (bold) in the BEB analysis. ^a^ The reference sequence is human SERPINB3. NA, not applicable because the neutural model fits better than positive selection.

** Significance with p<0.001.

* Significance with p<0.05.

**Table 4:** Likelihood ratio test for branch-site model for target proteases using *H. sapiens*, *P. troglodytes* and *G. gorilla* lineage as foreground.

Gene	Parameter estimates Foreground vs. Background	-2ΔlnL	Positively selected sites
*CTSS*	p_0_ = 0.802, p_1_ = 0.188, p_2a_ = 0.008, p_2b_ = 0.002, ω_0_ = 0.001, ω_1_ = 1.000, ω_2_ = 48.657	5.38*	255R
*CTSL1*	p_0_ = 0.678, p_1_ = 0.321, p_2a_ = 0.000, p_2b_ = 0.000, ω_0_ = 0.051, ω_1_ = 1.000, ω_2_ = 1.000	0	NA
*CTSL2*	p_0_ = 0.679, p_1_ = 0.320, p_2a_ = 0.000, p_2b_ = 0.000, ω_0_ = 0.000, ω_1_ = 1.000, ω_2_ = 1.000	0	NA
*CTSK*	p_0_ = 0.549, p_1_ = 0.066, p_2a_ = 0.343, p_2b_ = 0.041, ω_0_ = 0.000, ω_1_ = 1.000, ω_2_ = 1.000	0	NA
*CTSG*	p_0_ = 0.421, p_1_ = 0.540, p_2a_ = 0.017, p_2b_ = 0.021, ω_0_ = 0.000, ω_1_ = 1.000, ω_2_ = 7.081	0.98	NA
*CMA1*	p_0_ = 0.331, p_1_ = 0.200, p_2a_ = 0.292, p_2b_ = 0.177, ω_0_ = 0.000, ω_1_ = 1.000, ω_2_ = 2.196	0.17	NA

−2Δ*L*, likelihood ratio test to detect positive selection; Foreground: *H. sapiens*, *P. troglodytes* and *G. gorilla* lineage. *Significance with p<0.05; Positively selected sites, amino acid sites found to be under positive selection with a posterior probabilities greater 90%; NA, not applicable because the neutral model fits better than positive selection.

Finally, to evaluate the functional impact of the sites identified as being under positive selection in SERPINB3/B4 and target proteases, we built 3D structures of human SERPINB3- and B4-target complexes. The HADDOCK outcomes for the best models (Table 5) are consistent with the known inhibitory activity for SERPINB3 and B4 published in previous studies [18], [19]. Except for SERPINB4/CTSS complex, HADDOCK generated good predictions with i-RMSD≤2 Å and l-RMSD≤5 Å [52]. Interestingly, the bad quality prediction for SERPINB4/CTSS complex (i-RMSD≥4 Å and l-RMSD≥10 Å) is consistent with previous *in vitro* results that show the low inhibitory activity of SERPINB4 towards CTSS, 50 times less than SERPINB3 [20].

**Table 5:** Inhibitor protein complexes tested by docking analysis.

Model	HADDOCK score	i-RMSD	l-RMSD
SERPINB3/CTSK	−88.3+/−2.3	0.58+/−0.39	1.30+/−0.87
SERPINB3/CTSL1	−62.0+/−15.7	1.04+/−0.91	3.09+/−3.18
SERPINB3/CTSL2	−75.0+/−4.2	0.38+/−0.25	0.778+/−0.59
SERPINB3/CTSS	−96.1+/−4.4	0.43+/−0.30	1.06+/−0.73
SERPINB4/CTSS	−74.1+/−7.0	16.23+/−0.35	35.47+/−1.05
SERPINB4/CMA1	−85.8+/−7.5	0.52+/−0.36	1.48+/−1.07
SERPINB4/CTSG	−74.5+/−5.6	0.47+/−0.32	1.02+/−0.75

i-RMSD: interfacial root mean square deviation; l-RMSD: ligand root mean square deviation; HADDOCK score is weighted sum of van der Waals, electrostatic, desolvation and restrained violation energies together with buried surface area.

**Figure 3:**
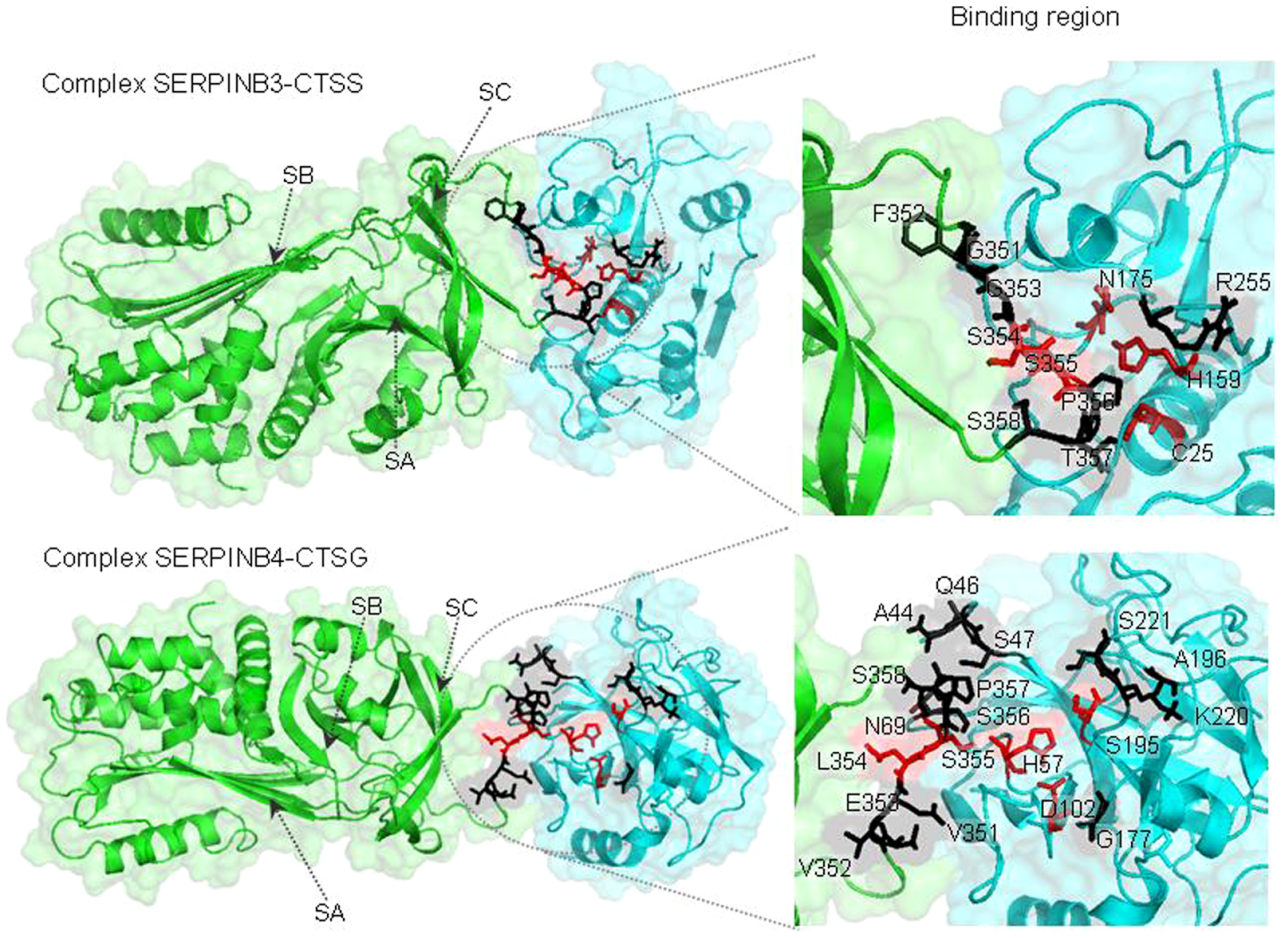
The best docking for SERPINB3 (green)/CTSS (blue) and SERPINB4 (green)/CTSG (blue) complex models generated using HADDOCK software. Amino acids under positive selection at the SERPIN/protease interface are in black. Amino acids at the inhibitor scissile bond and forming the proteases catalytic triad are depicted in red. Arrows point the location of β-sheet A (SA), β-sheet B (SB) and β-sheet C (SC). Binding regions are enlarged for a more detailed view (left panel).

Figure 3 shows the 3D structures of SERPINB3/CTSS and SERPINB4/CTSG complexes as representatives of inhibitor-proteases complexes. The seven RCL residues identified by the site-model tests as under positive selection for SERPINB3/B4 phylogeny (Table 1) (V351G, V352F, E353G, L354S, S356P, P357T and C364H), are in the inhibitor/protease interface, in close proximity to the activity site of the target protease (Fig. 3). Overall, the RCL plays a critical role in the inhibitory activity of SERPINs and some studies highlight this notion by showing that the target specificities of SERPINB3 and B4 could be reversed solely by swapping their RCL [18]. Moreover, as experimentally reported, single amino acid substitutions in the RCL region were unable to convert SERPINB4 in a more efficient cysteine protease inhibitor. In the particular case of CTSS inhibition, different combinations of mutations at SERPINB4 positions P2, P2′, P3′ and P10′ led to an increase in CTSS inhibition accounting for 80% of the difference in SERPINB3 and B4 activity [55]. Interestingly, the P2, P2′, P3′ and P10′ positions correspond to the residues E353G, S356P, P357T and C364H, respectively, which were found to be under strong positive selection in the present study. Furthermore, the residue V352F, in position P3, is a key residue for specificity and binding of papain-like cysteine proteases and in the case of CTSS the preferred P3 residues are bulky hydrophobic, as phenylalanine residue in SERPINB3 [18]. In addition, P1 position (L354S) was found to be under positive selection and several mutagenesis studies show that the P1 residue is usually the most important for SERPIN protease specificity [5].

The 3D structures of SERPINB3/CTSS (Fig. 3), SERPINB4/CMA1 and SERPINB4/CTSG (Fig. 3) reveal that several residues under positive selection (Table 3 and Table 4) are located in the loops surrounding the enzyme catalytic pocket, which have been shown to be involved in substrate specificity and in enzyme activation [54]. Also, the location of these residues in loops near to the enzyme catalytic pocket may suggest a possible role in the 3D conformation assumed by this region. Moreover, X-ray analysis of the SERPIN-protease inhibition complexes reveals that the distortion of protease activity is due to the compression of the loops surrounding the protease active site against the basis of the SERPIN. Hence, an amino acid substitution in the protease loops neighbouring the active site could have physical implications in the inhibition mechanism [5] and contribute for the functional divergence of SERPINB3 and B4.

### Tissue expression pattern of *SERPINB3* and *SERPINB4*

**Figure 4:**
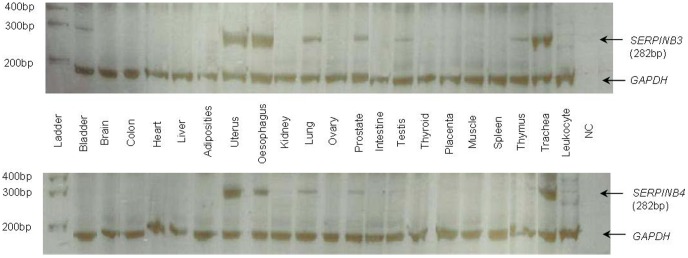
Expression patterns of SERPINB3 and SERPINB4 in human tissues. *GAPDH* amplification was used as a control. NC: negative control.

A panel of 21 tissues was used to determine the expression pattern of *SERPINB3* and *B4*. As shown in figure 4, *SERPINB3* and *B4* transcripts were found in uterus, esophagus, lung, prostate, testis and trachea tissues, whereas in bladder and thymus only the expression of SERPINB3 was detected (Fig. 4). These expression patterns are consistent with the ones obtained by Cataltepe and colleagues, who have shown that *SERPINB3* and *B4* are frequently co-expressed in several adult human tissues at both mRNA and the protein levels [27]. In addition, these findings fit the expectations of two recent duplicates being more likely to share cis-regulatory motifs and to display stronger co-expression patterns than two randomly selected genes [56]. The ENCODE annotation of transcript factors by CHIP-seq for *SERPINB3* and *B4* available in UCSC database (http://genome.ucsc.edu/) confirms that these duplicates still share several regulatory motifs, including STAT3, CEBPB, FOS and JUN (Fig. S5), which are associated to immunity and apoptosis pathways. Furthermore, upstream of *SERPINB3* there is an active regulatory region, identified by an H3K27Ac histone mark, and multiple transcripts factors which possibly affect both duplicates (Fig. S5). Therefore, the similar expression pattern of *SERPINB3* and *B4* is best explained by the low divergence in the cis-regulatory motifs contrasting with functional specialization into cysteine and serine inhibitors, respectively.

Finally the expression sequence tag (EST) profile of CTSS, CTSL1, CTSL2, CTSK, CTSG and CMA1 target proteases was assessed revealing an overlap with SERPINB3 and B4 expression pattern in several tissues (Fig. S6).

## Discussion

In the present work, we evaluate the evolutionary forces forging the recent duplicates *SERPINB3* and *B4* and address their functional impact in protein structure, inhibitor-protease interaction and gene expression regulation. Phylogenetic analysis reveals that a duplication event, at approximately 29.6 MY ago, gave rise to *SERPINB3* and *B4* paralogs, stably retained in *H. sapiens*, *P. troglodytes* and *G. gorilla* genomes, but not in *P. abelli* and *N. leucogenys* species, which carry a pseudogene and an ancestral gene (AncB3/B4) instead. In the *SERPINB3*/*B4* phylogeny, evolutionary tests disclosed a clear signature of positive selection in the substitution rates across the nine primate species studied, *H. sapiens*, *P. troglodytes*, *G. gorilla*, *P. abelli*, *N. leucogenys*, *M. mulatta*, *P. anubis*, *C. jacchus* and *S. boliviensis*. Also, the branch-site test shows that in the *H. sapiens*, *P. troglodytes* and *G. gorilla* clade, the *SERPINB3* copy is evolving under positive selection supporting the functional divergence observed in several experimental studies.

In this context we can consider two scenarios, either the duplication led to the acquisition of a complete new function by one of the duplicates or a subdivision of the ancestral function occurred to accommodate an improved inhibitory activity. Under a subfunctionalization hypothesis, after the duplication event both copies would maintain the original function and several degenerative mutations would be tolerated by *SERPINB3* and *SERPINB4,* due to a relaxation of selective constrains. However, this model fails to explain the different hits of positive selection detected for the entire SERPINB3/B4 phylogeny and for the *SERPINB3* clade alone. Likewise, the subfunctionalization theory predicts an expression diversification where duplicates sharing the same function become specialized in different tissues or developmental stages [38], which is not the case of *SERPINB3* and *B4*. Instead, the neofunctionalization model seems to fit better the evolutionary history of *SERPINB3* and *B4* duplicates. According to this model a copy is kept under purifying selection and retains the original function while the other is targeted by positive selection and experiences the accumulation of several amino acid substitutions ultimately leading to a novel function.

Several studies have demonstrated that positive selection frequently occurs in concert with duplication events in genes involved in brain function and cell growth [57], [58], reproduction [59], endurance running [60] and in xenobiotic recognition of macromolecules [61]. In addition, several gene families implicated in the immune system were proposed as targets of positive selection [62], [63]. There, gene duplications are considered a important mechanism in the enlargement of host defence repertoire, which is crucial for a rapid response to changing environments and to a increased burden of pathogens [64]. For instance, the tripartite motif (TRIM) protein family, a group of innate antiviral effectors, experienced several episodes of strong positive selection showing high levels of sequence divergence between paralogs and a wide range of antiviral activities possibly resulting from different attempts to counteract fast evolving viruses [65].

Similarly, evidence for positive selection was detected in several members of the SERPIN superfamily. *SERPINB11*, a highly conserved gene in primates, was lost and resurrected in humans where the accumulation of several mutations contributed to the appearance of a modified non-inhibitory SERPIN, probably linked to an adaptive response against the emergence of infectious diseases in recent human evolution [66]. Also, in *SERPINA2*, a 90 MY old duplicate of alpha1-antitrypsin (SERPINA1), several sites seem to be under positive selection in primates, contributing to the emergence of a new advantageous function, possible as a chymotrypsin-like inhibitor [67]. Conversely, a large deletion in *SERPINA2* was proposed to be selective advantageous in Africans through a potential role in fertility or in host–pathogen interactions (Seixas, et al 2007).

Such recent studies are in agreement with earlier assumptions based mostly in human and rodent sequences that established a link between RCL hypervariability, SERPIN superfamily functional diversity and positive selection acting after gene duplication [68]–[70]. Furthermore, Hill and Hastie postulate that these adaptive changes were fixated because SERPINs were challenged by exogenous proteases brought in by infectious agents, which may indicate an ongoing host-pathogen coevolution [69].

Likewise, we propose that the *SERPINB3*/*B4* selective signatures are the result of a coevolution process involving either endogenous or exogenous target proteases. Indeed, the structural and docking analyses are in line with previous biochemical studies [19], [55], showing that many of the putatively selected sites fall in regions important for the inhibitor function promoting functional divergence between *SERPINB3* and *B4*. Also, the ability of SERPINB4 to inhibit CTSS, as well as other papain-like cysteine proteases, at a rate 50-fold slower than that of SERPINB3 [55] may suggest that the functional divergence of these two inhibitors is still ongoing. Finally, the scenario of functional divergence is strengthened by the consistence of selective signatures of *SERPINB4* targets, *CMA1* and *CTSG* in the primates (our study) and mammalian phylogenies [54], [71]. Since CMA1 and CTSG are powerful proteases involved in programmed cell death (apoptosis) and in the immune response, an evolution of these molecules driving by host-defence is also likely. Hence, selective hallmarks observed throughout *SERPINB3*/*B4* phylogeny can result from an adaptive response to *CMA1* and *CTSG* evolution.

The overlap of *CTSS* and *SERPINB3* selective signatures in the *H. sapiens*, *P. troglodytes* and *G. gorilla* clade points as well for a possible coevolution of these molecules. Interestingly, both CTSS and SERPINB3 are found in endosome/lysosome structures in macrophage [72] and B cells [28] where CTSS is thought to be engaged in antigen presentation through the degradation of a major histocompatibility complex class II chain [73].

Aside from a role in innate immunity through the regulation of endogenous proteases, SERPINB3 may also be enrolled in the host-pathogen response by the inhibition of cysteine proteases released in the infectious processes by *Staphylococcus aureus* (staphopains) [33], *Leishmania Mexicana* (CPB2.8), *Trypanosoma cruzi* (cruzain), *T. brusei rhodesience* (rhodesain) and *Fasciola hepatica (*cathepsin L2) [32]. Worth to note, *SERPINB3* is expressed in squamous epithelium of mucous membranes, skin and the respiratory system, where it may act as a primary host-defence mechanism by preventing pathogens to cross and disrupt epithelial barriers. Moreover, the regulation of *SERPINB3* expression by the transcription factors STAT3, CEBPB and FOS/JUN AP-1 complex, which are involved in the development and modulation of the immune system, regulation of cell proliferation and differentiation, mediation of cytokine receptors signaling and control of genes involved in the immune and inflammatory responses [74]–[76], further supports the possible role of SERPINB3 in immune response.

In conclusion, the present work shows a positive selection signature throughout *SERPINB3*/*B4* phylogeny, which may be a major force driving the functional divergence of *SERPINB3* and *B4* duplicates. Ultimately, adaptive evolution led to different protease specificities providing *SERPINB3* and *B4* with the ability to inhibit a broader repertoire of endogenous and exogenous proteases. Furthermore, the retention of *SERPINB3* and *B4* duplicates in the *H. sapiens*, *P. troglodytes* and *G. gorilla* clade could have a selective advantage in host-pathogen interactions due to an adaptive response against infectious diseases in Africa, during the evolution of great apes. Also, our results show that *SERPINB3* duplicate is being subject to strong positive selection that could derive as well from ongoing host-pathogen coevolution. The interaction of host protease inhibitors with invasive proteases of pathogens can constitute a strong evolutionary pressure for the host to counteract by evolving new and effective inhibitors. Above all, the search for a positive selection signal among inhibitors and target proteases could contribute for a better understanding of the complex interactions involving both types of molecules and how its imbalance could lead to the onset of different types of carcinomas and immune diseases, having potential therapeutical implications.

## Supporting Information

Figure S1 **Primer annealing positions within** ***SERPINB3*** **and** ***SERPINB4*** **cDNA.** Underlined: 5′ - TGGAAATACCATACAAAGGCA – 3′ primer annealing position. Highlighted in red: unique 5′ – TGTAGGACTCCAGATAGCAC – 3′ and 5′- TGTAGGACTTTAGATACTGA – 3′ annealing positions. PCR was programmed as follows: initial denaturation at 95°C for 10 minutes, followed by 35 cycles of denaturation at 94°C for 30 seconds, annealing at 54°C for 30 seconds and extension at 72°C for 30 seconds and a final extension at 60°C for 30 minutes.
(TIF)

Figure S2 **Multipipemaker** ***SERPINB3*** **and** ***SERPINB4*** **alignment.** Hsapiens: *Homo sapiens*; Ptroglodytes: *Pan troglodytes*; Ggorilla: *Gorilla gorilla*; Pabelli: *Pongo abelli*; Nleucogenys: *Nomascus leucogenys*; Mmulatta: *Macaca mulatta*; Panubis: *Papio Anubis*; Cjacchus: *Callithrix jacchus*; Sboliviensis: *Saimiri boliviensis*
(PDF)

Figure S3 **Branch-site analysis for SERPINB3/B4 genes, foreground and background groups.**
(TIFF)

Figure S4 **Branch-site analysis for CTSS, foreground and background groups.**
(TIFF)

Figure S5 **UCSC ENCODE annotation of transcript factors obtained by CHIP-seq experiments for** ***SERPINB3*** **and** ***SERPINB4*** **.**
(TIF)

Figure S6 A) CTSG and CMA1 expression pattern showing an ubiquitous expression profile for CTSG. B) Heat map and hierarchical bi-clustering of the expression sequence tag (EST) data of SERPINB3/B4 and their target proteases. The data for 45 normal tissues were extracted from NCBI UNIGENE and normalized by total number of transcripts per library. Red and green correspond to the high and low expression levels, respectively. Black represents an average level of expression.
(TIF)

Table S1 **Genomic locations for the DNA sequences retrieved from the National Center for Biotechnology Information database (NCBI) and University of California Santa Cruz (USCS) Genomic Bioinformatics database for the nine primate species.**
(DOCX)
