# Supplementary figures and images for: Adaptive Evolution and Divergence of SERPINB3: A Young Duplicate in Great Apes
Source: PLoS One. 2014 Aug 18;9(8):e104935. doi: 10.1371/journal.pone.0104935 (PMC4136820; doi:10.1371/journal.pone.0104935)

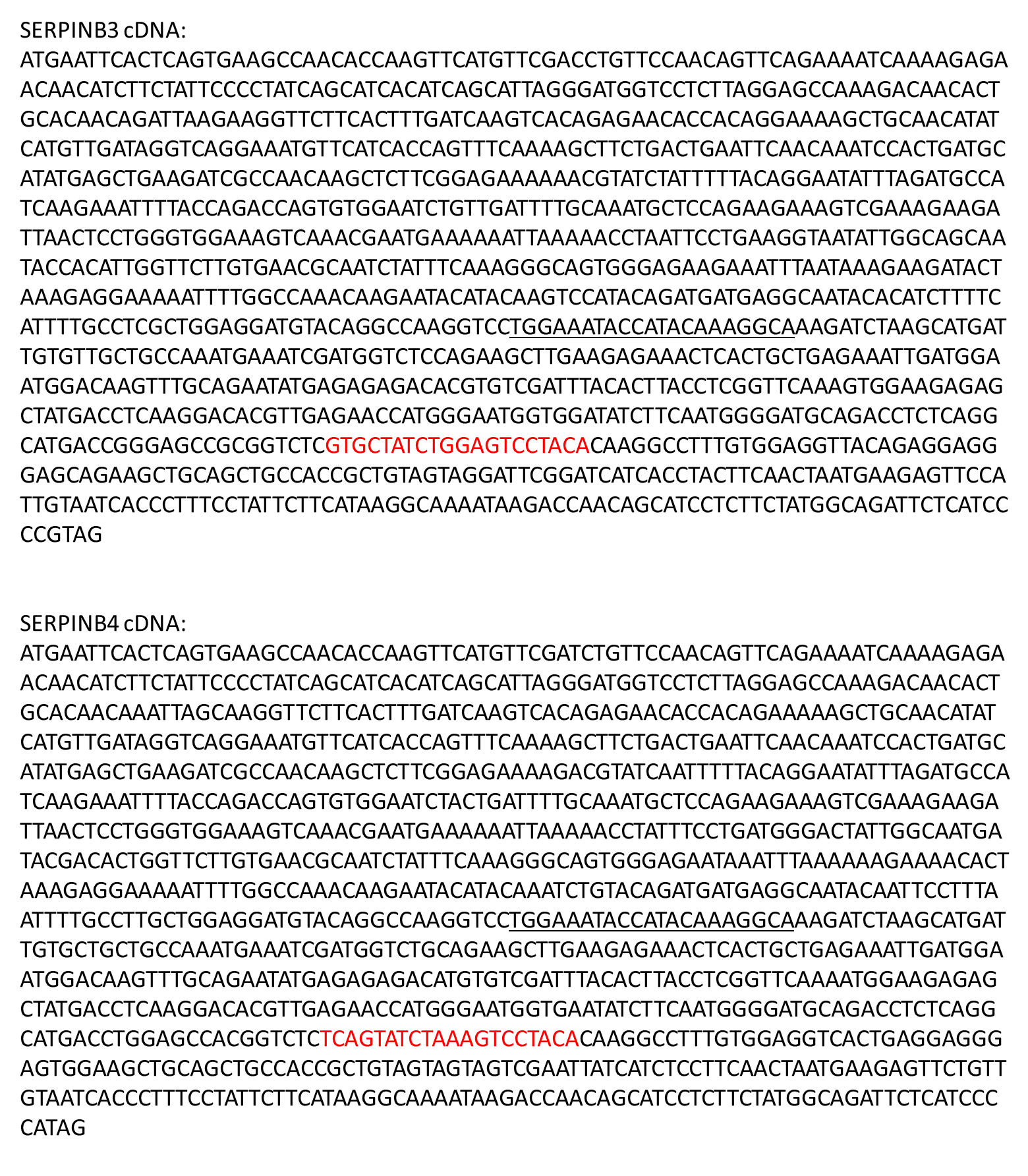

Supplement: Figure S1 — Primer annealing positions within SERPINB3 and SERPINB4 cDNA. Underlined: 5′ - TGGAAATACCATACAAAGGCA – 3′ primer annealing position. Highlighted in red: unique 5′ – TGTAGGACTCCAGATAGCAC – 3′ and 5′- TGTAGGACTTTAGATACTGA – 3′ annealing positions. PCR was programmed as follows: initial denaturation at 95°C for 10 minutes, followed by 35 cycles of denaturation at 94°C for 30 seconds, annealing at 54°C for 30 seconds and extension at 72°C for 30 seconds and a final extension at 60°C for 30 minutes. (TIF) [file pone.0104935.s001.tif]

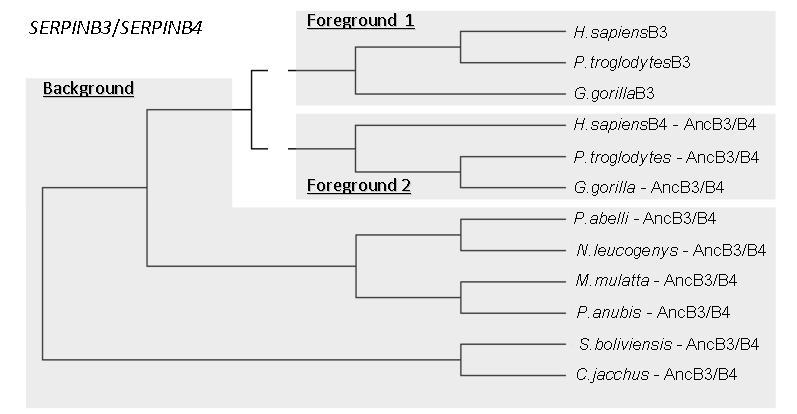

Supplement: Figure S3 — Branch-site analysis for SERPINB3/B4 genes, foreground and background groups. (TIFF) [file pone.0104935.s003.tiff]

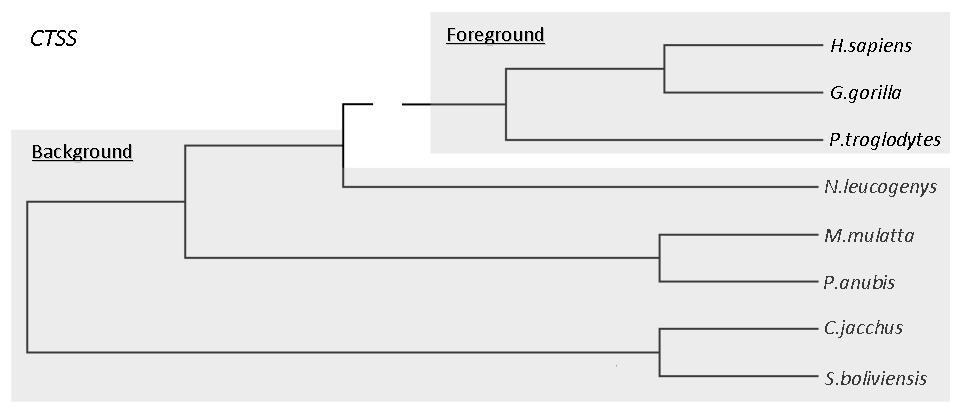

Supplement: Figure S4 — Branch-site analysis for CTSS, foreground and background groups. (TIFF) [file pone.0104935.s004.tiff]

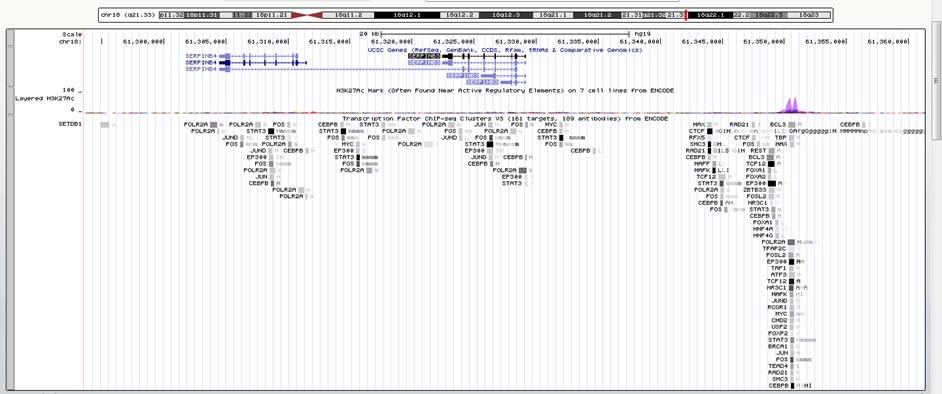

Supplement: Figure S5 — UCSC ENCODE annotation of transcript factors obtained by CHIP-seq experiments for SERPINB3 and SERPINB4 . (TIF) [file pone.0104935.s005.tif]

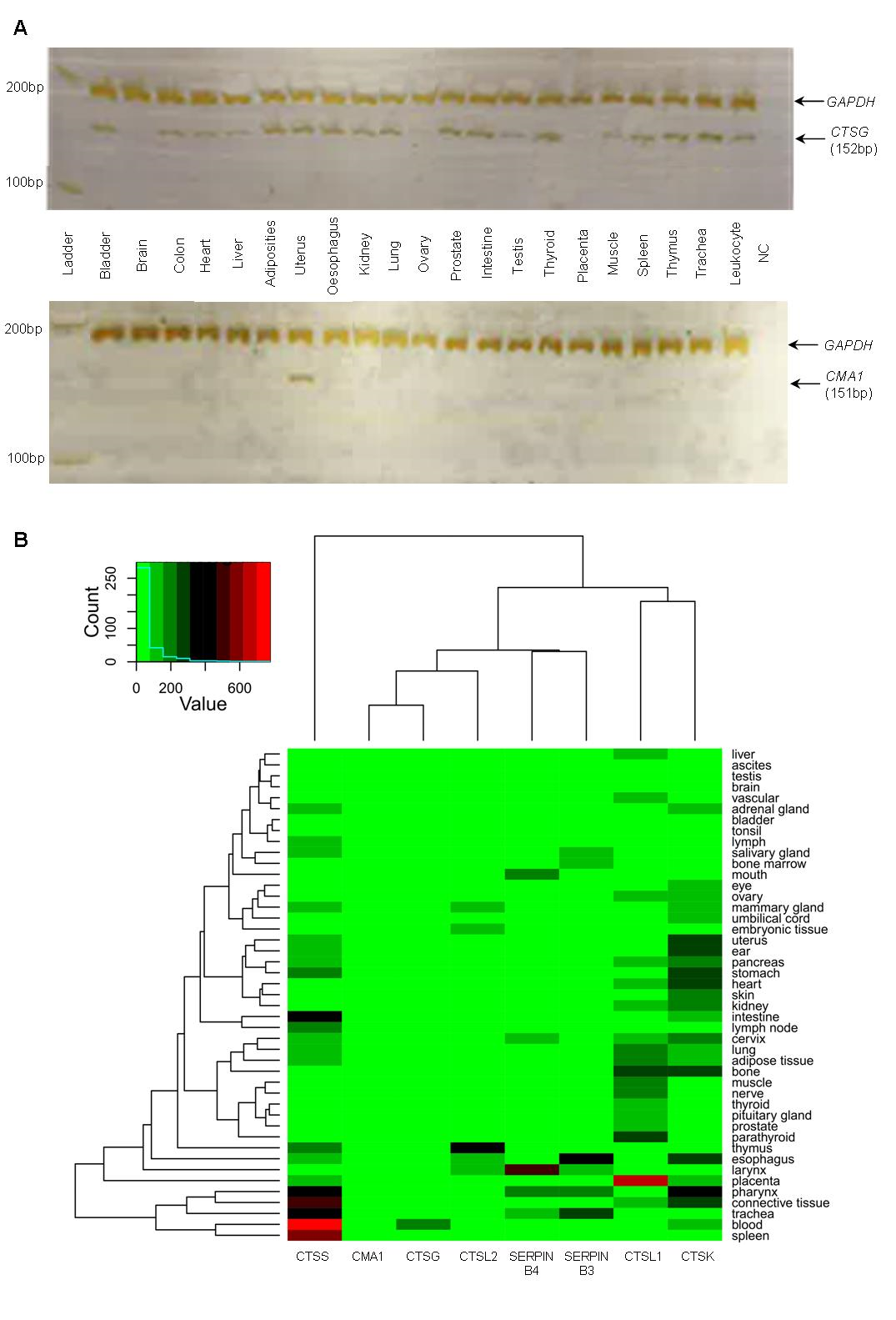

Supplement: Figure S6 — A) CTSG and CMA1 expression pattern showing an ubiquitous expression profile for CTSG. B) Heat map and hierarchical bi-clustering of the expression sequence tag (EST) data of SERPINB3/B4 and their target proteases. The data for 45 normal tissues were extracted from NCBI UNIGENE and normalized by total number of transcripts per library. Red and green correspond to the high and low expression levels, respectively. Black represents an average level of expression. (TIF) [file pone.0104935.s006.tif]
