# Supplementary material for: Adaptive Evolution and Divergence of SERPINB3: A Young Duplicate in Great Apes
Source: PLoS One. 2014 Aug 18;9(8):e104935. doi: 10.1371/journal.pone.0104935 (PMC4136820; doi:10.1371/journal.pone.0104935)
Supplement: Figure S2 — Multipipemaker SERPINB3 and SERPINB4 alignment. Hsapiens: Homo sapiens; Ptroglodytes: Pan troglodytes; Ggorilla: Gorilla gorilla; Pabelli: Pongo abelli; Nleucogenys: Nomascus leucogenys; Mmulatta: Macaca mulatta; Panubis: Papio Anubis; Cjacchus: Callithrix jacchus; Sboliviensis: Saimiri boliviensis (PDF) [file pone.0104935.s002.pdf]

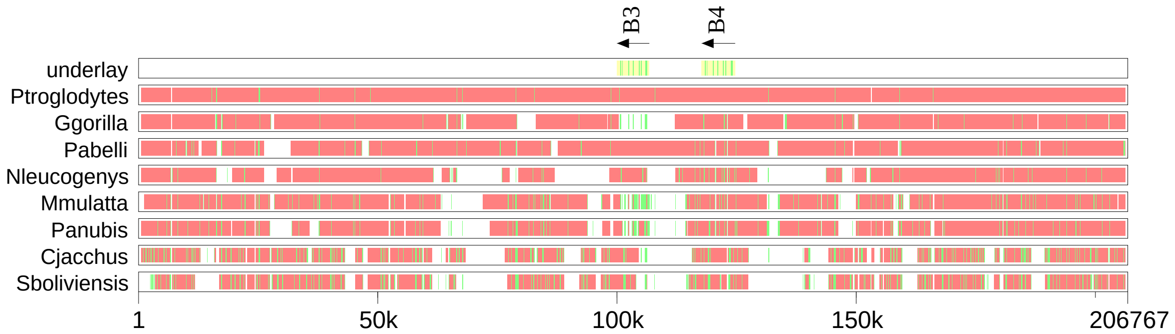
B3
B4
underlay
Ptroglodytes
Ggorilla
Pabelli
Nleucogenys
Mmulatta
Panubis
Cjacchus
Sboliviensis
1
50k
100k
150k
206767

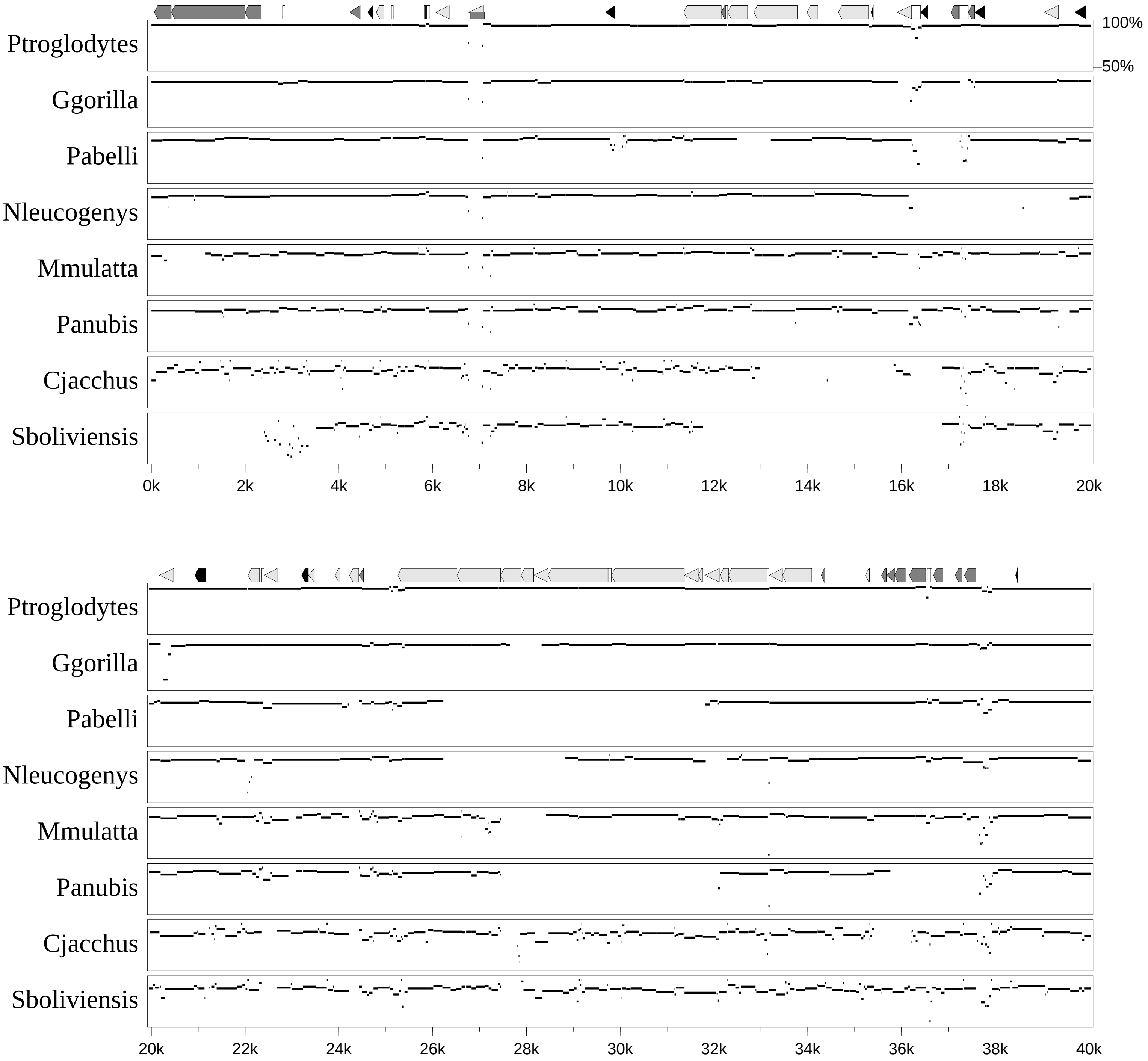

Ptroglodytes
Ggorilla
Pabelli
Nleucogenys
Mmulatta
Panubis
Cjacchus
Sboliviensis
100%
50%
0k
2k
4k
6k
8k
10k
12k
14k
16k
18k
20k
20k
22k
24k
26k
28k
30k
32k
34k
36k
38k
40k

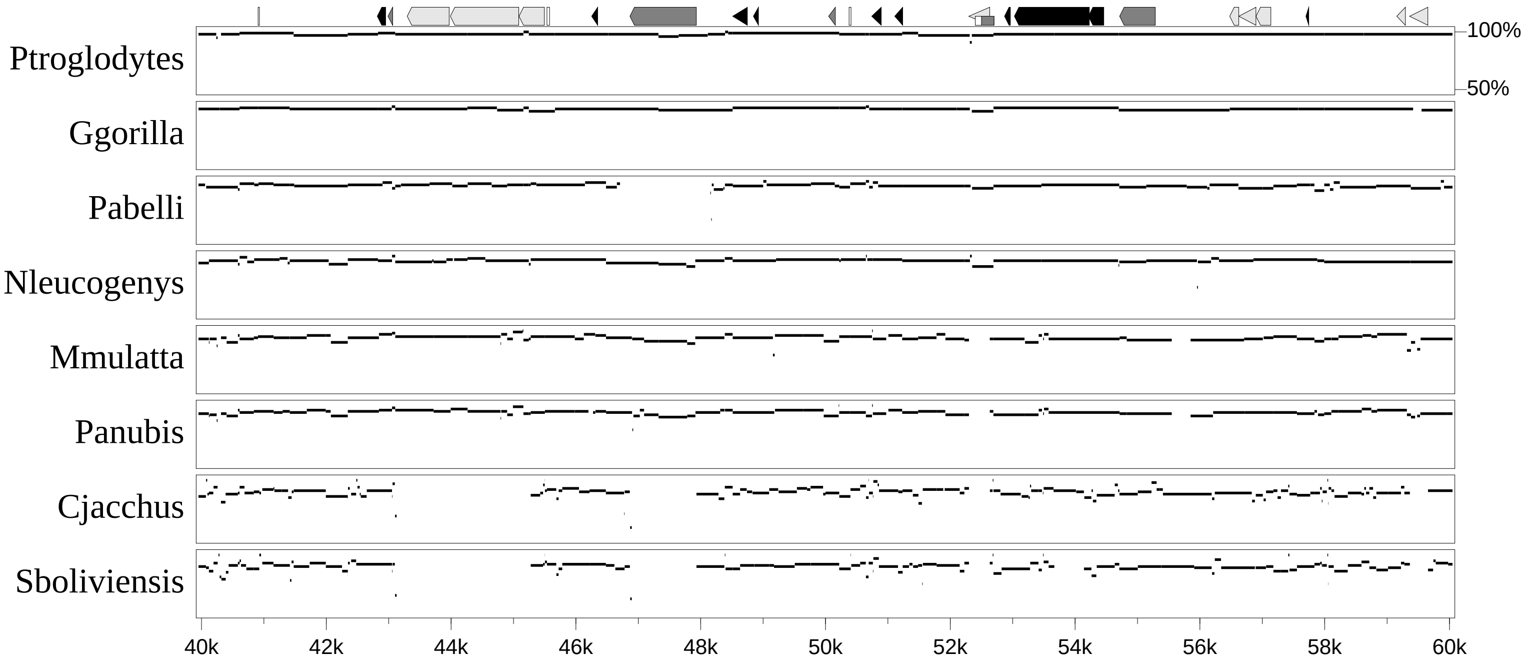

Ptroglodytes
Ggorilla
Pabelli
Nleucogenys
Mmulatta
Panubis
Cjacchus
Sboliviensis
100%
50%
40k
42k
44k
46k
48k
50k
52k
54k
56k
58k
60k

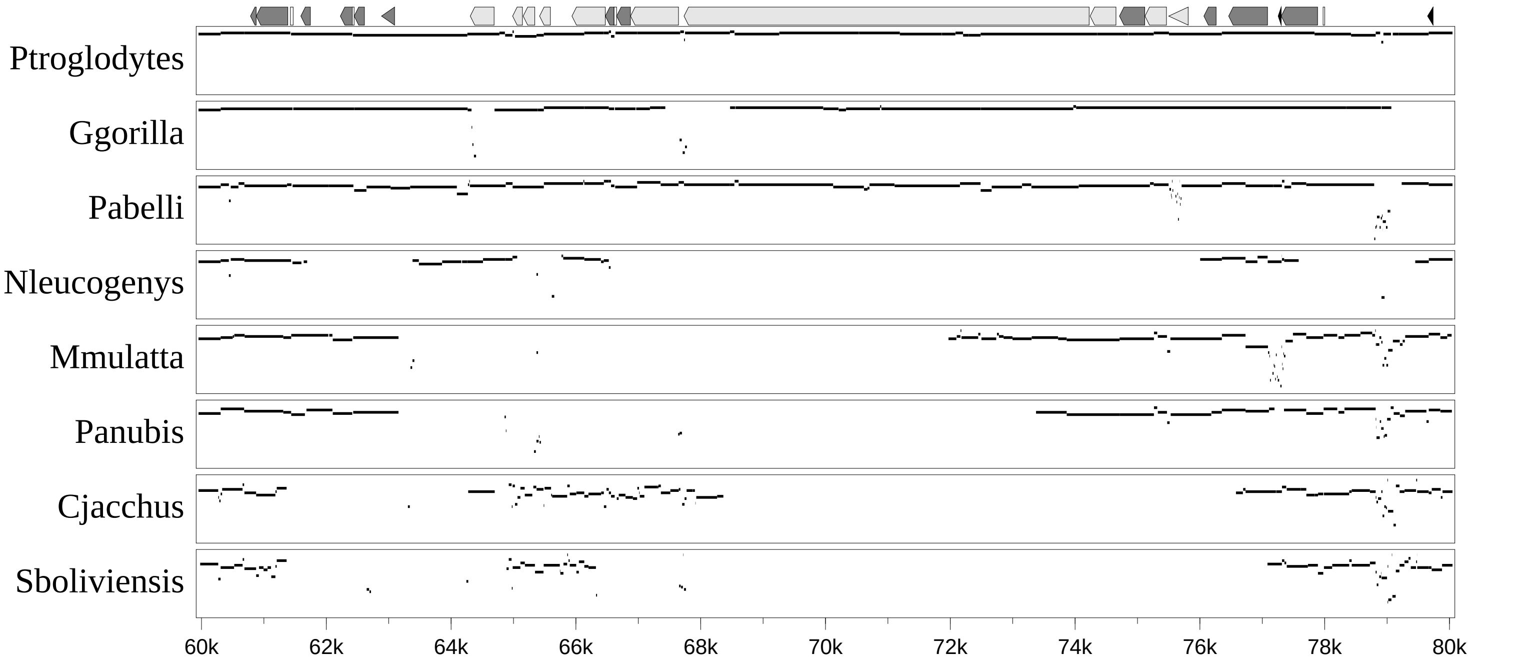

Ptroglodytes
Ggorilla
Pabelli
Nleucogenys
Mmulatta
Panubis
Cjacchus
Sboliviensis
60k
62k
64k
66k
68k
70k
72k
74k
76k
78k
80k

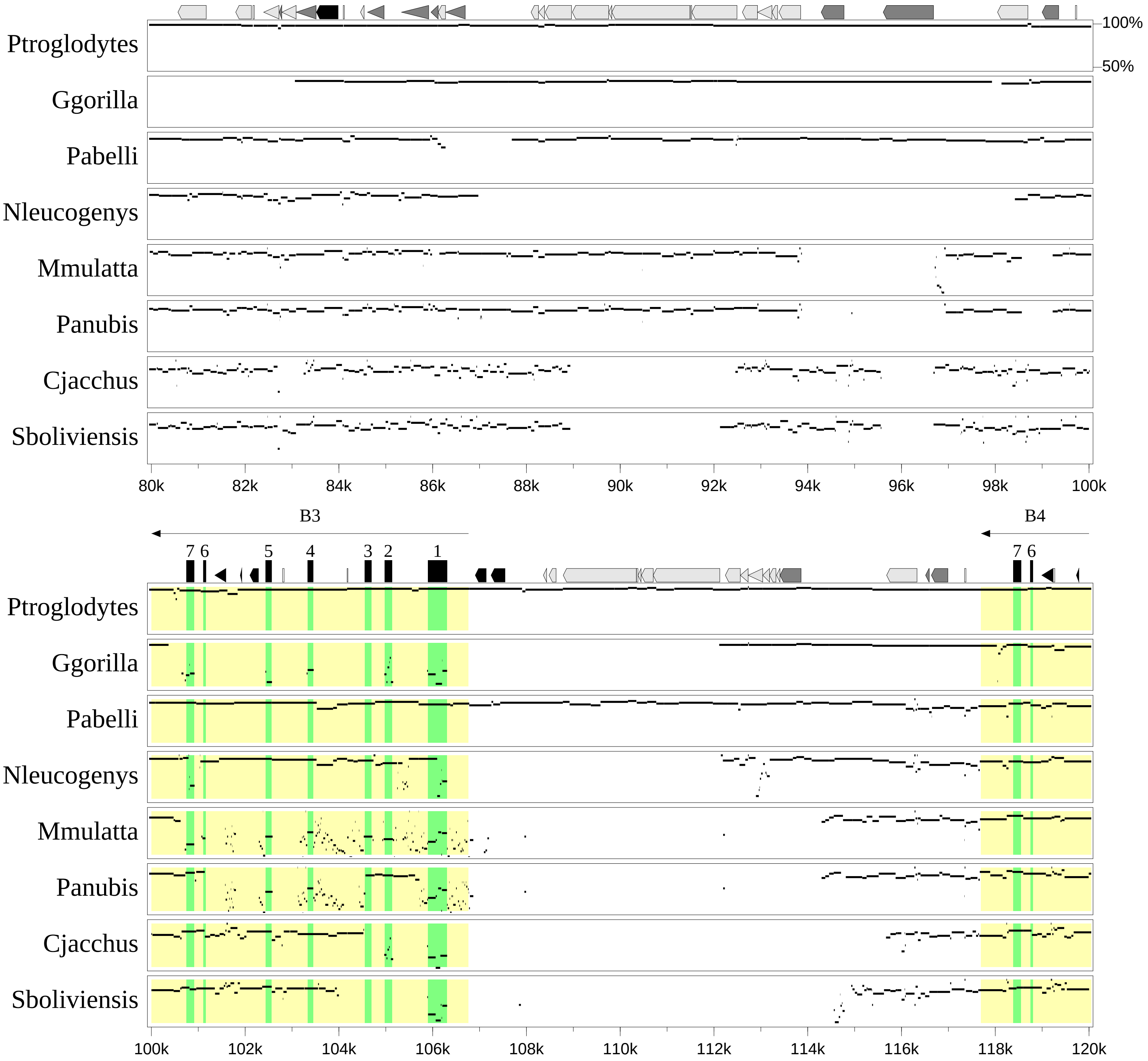

Ptroglodytes
Ggorilla
Pabelli
Nleucogenys
Mmulatta
Panubis
Cjacchus
Sboliviensis
100%
50%
80k
82k
84k
86k
88k
90k
92k
94k
96k
98k
100k
B3
B4
7 6 5 4 3 2 1
7 6
Ptroglodytes
Ggorilla
Pabelli
Nleucogenys
Mmulatta
Panubis
Cjacchus
Sboliviensis
100k
102k
104k
106k
108k
110k
112k
114k
116k
118k
120k

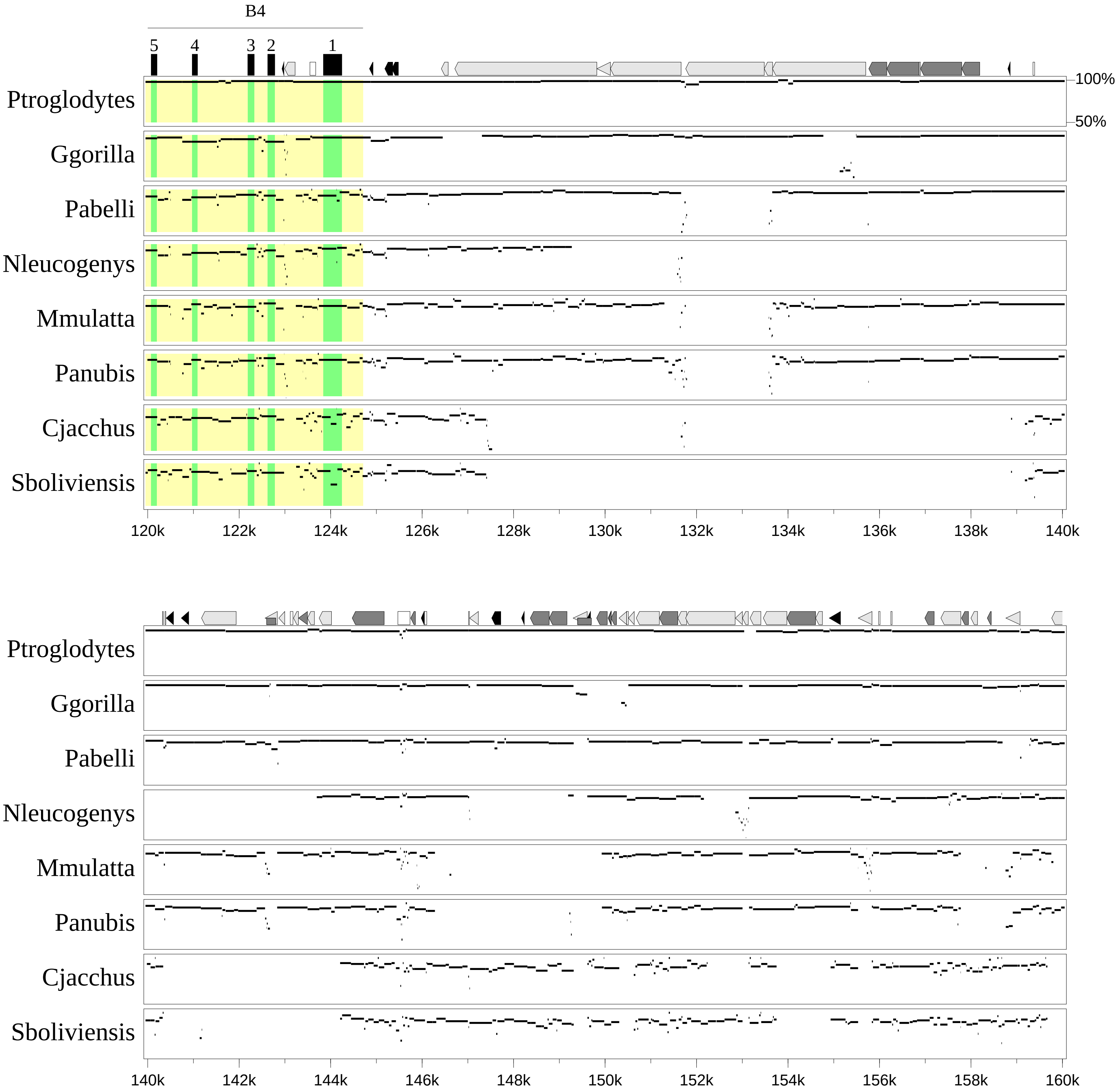

B4
5
4
3
2
1
Ptroglodytes
Ggorilla
Pabelli
Nleucogenys
Mmulatta
Panubis
Cjacchus
Sboliviensis
100%
50%
120k
122k
124k
126k
128k
130k
132k
134k
136k
138k
140k
142k
144k
146k
148k
150k
152k
154k
156k
158k
160k

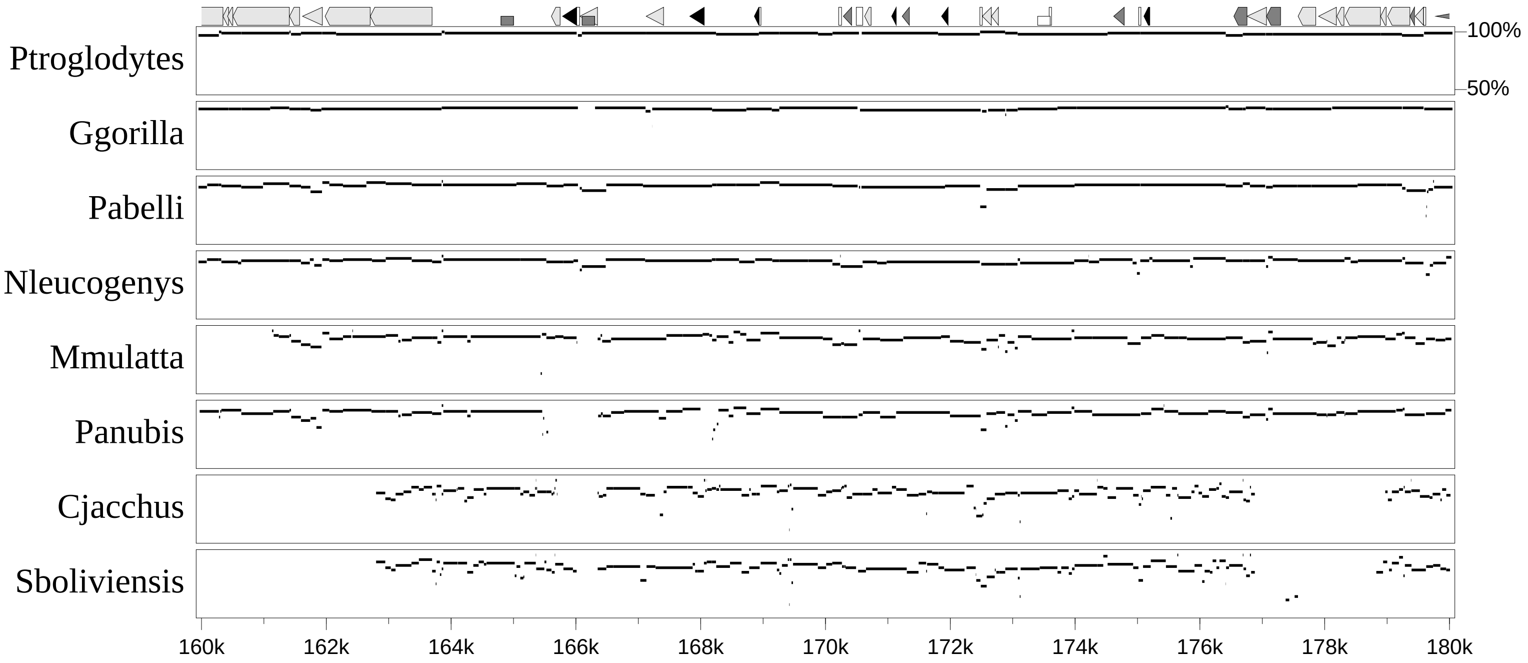

100%
50%
Ptroglodytes
Ggorilla
Pabelli
Nleucogenys
Mmulatta
Panubis
Cjacchus
Sboliviensis
160k
162k
164k
166k
168k
170k
172k
174k
176k
178k
180k

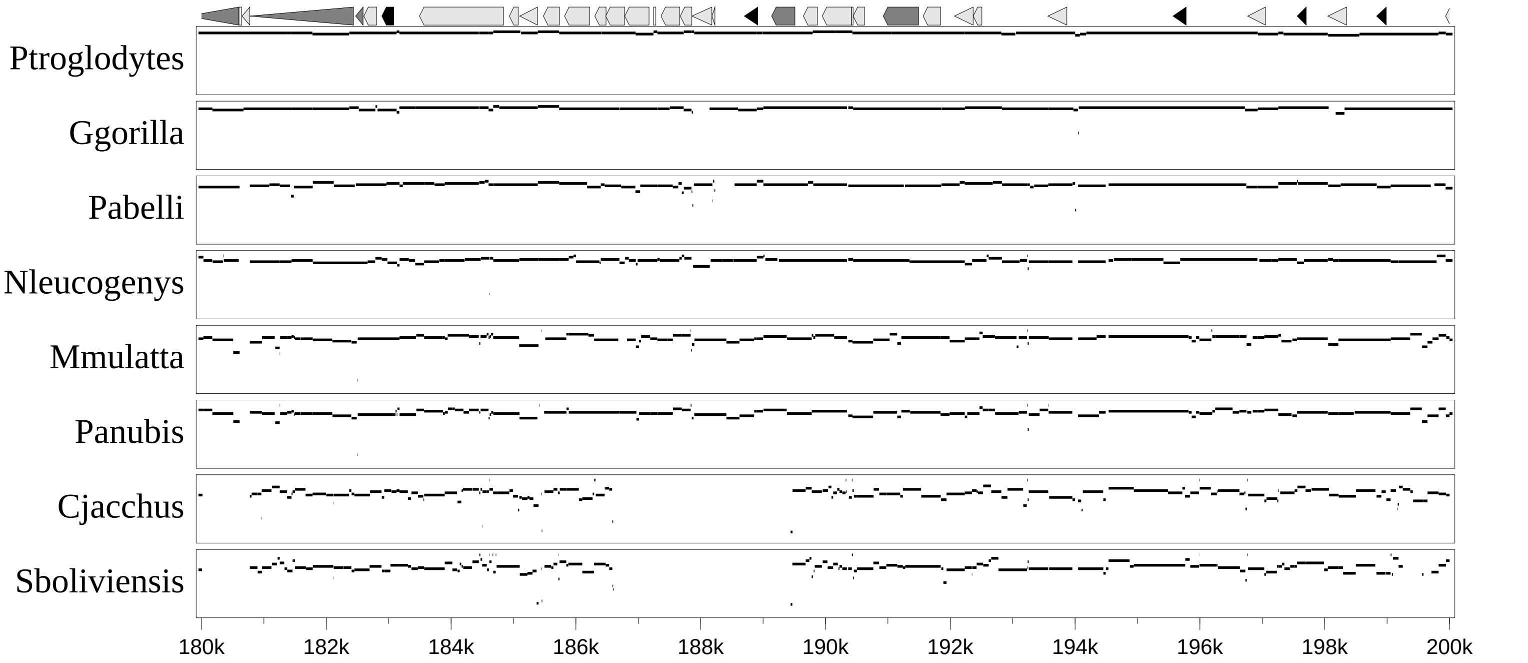

Ptroglodytes
Ggorilla
Pabelli
Nleucogenys
Mmulatta
Panubis
Cjacchus
Sboliviensis
180k
182k
184k
186k
188k
190k
192k
194k
196k
198k
200k

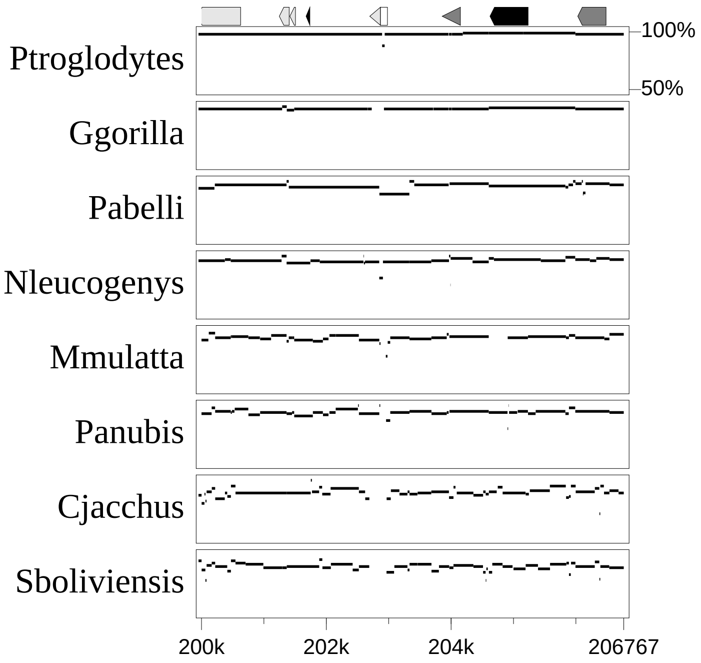
Ptroglodytes
Ggorilla
Pabelli
Nleucogenys
Mmulatta
Panubis
Cjacchus
Sboliviensis
100%
50%
200k
202k
204k
206767

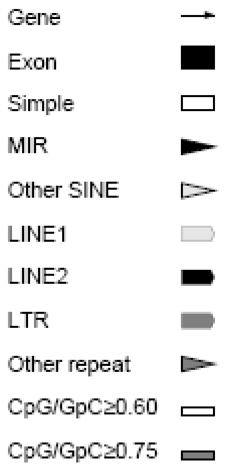
Gene
Exon
Simple
MIR
Other SINE
LINE1
LINE2
LTR
Other repeat
CpG/GpC≥0.60
CpG/GpC≥0.75

```
                   10        20        30        40        50
                    :         :         :         :         :
      1: AGATAAGTTGGATCAGGTCAGACCTAGAAGACAGAGAGGTGGCCCCAGCA  Hsapiens
 319901: ..............G......G............................  Ptroglodytes
 337157: .............TG......G............................  Ggorilla
 369635: .............TG......GT........T.................G  Pabelli
 294368: ..G..........TG......G.....G.....C...A...........G  Nleucogenys
 363872: .............TG......G.....G.C....................  Mmulatta
 316481: .............TG......G.....G.C....................  Panubis
 352813: .......AGA...TC......GAGC..G.A.TG...G.......A.....  Cjacchus

                                   LTR
                      ----------------------------------->
                   60        70        80        90       100
                    :         :         :         :         :
     51: GGACACGCCAGGTGTATTAGTCTGTTTTTGCACGGCTGATAAAGACATAC  Hsapiens
 319951: .T...T............................................  Ptroglodytes
 337207: ..................................................  Ggorilla
 369685: ................................T.................  Pabelli
 294418: ................................T.................  Nleucogenys
 363922: .....T.A...............A.........T................  Mmulatta
 316531: .....T.A...............A.........T................  Panubis
 352863: .....T.......T..............CATG.T................  Cjacchus

                              LTR
         -------------------------------------------------->
                  106       116       126       136       146
                    :         :         :         :         :
    101: CT----GAGATTGGGAAGAAAAAGAAAAAAAAGTTTAATTGAACTTACAG  Hsapiens
 320001: ..----.........................G..................  Ptroglodytes
 337257: ..----.........................GA.................  Ggorilla
 369735: ..----.......................G.G..................  Pabelli
 294468: .C----...................G.....G..................  Nleucogenys
 363972: ..----....C..................G.G.....G............  Mmulatta
 316581: ..----....C..................G.G.....G.....T......  Panubis
 352913: A.ACAC....C...................GGA......G.G.....T..  Cjacchus

                              LTR
         -------------------------------------------------->
                  156       166       176       186       196
                    :         :         :         :         :
    147: TTCCACATAGCTTGGGAGGCCTCATAATCATGGCAGGAGGTGAAAGGCAC  Hsapiens
 320047: ..................................................  Ptroglodytes
 337303: ..................................................  Ggorilla
 369781: ............G.....................................  Pabelli
 294514: ............G.....................................  Nleucogenys
 364018: .......G....G....................TG...............  Mmulatta
 316627: ............G.....................G...............  Panubis
 352963: ........G...G..........CG........TG...............  Cjacchus

                              LTR
         -------------------------------------------------->
                  206       216                 229       239
                    :         :                   :         :
    197: TTCTTACATGGCGGCAGCAAGAGAAAATG-------AGGAAGAAGCAAAA  Hsapiens
 320097: .............................-------..............  Ptroglodytes
 337353: ........A....................-------..............  Ggorilla
 369831: ...........T.................AGGAAGA..............  Pabelli
 294564: ...........T..A...C.........A-------..............  Nleucogenys
 364068: ...C.......TA................---------------------  Mmulatta
 316677: ...C........A................-------........A.....  Panubis
 353013: G...G......T.................-------.........G....  Cjacchus
```

```
                                 LTR
            ---------------------------------------------------->
                    249       259       269       279       289
   240: GCGGAAACCCCTGATAAATCCATCAGATCTTGTGAGACTTATTCACTATC  Hsapiens
320140: ..................................................  Ptroglodytes
337396: ........................................G.........  Ggorilla
369881: ...................................C..C...C.......  Pabelli
294607: ...........................C......................  Nleucogenys
364097: -------------------------.....CA................C.  Mmulatta
316720: ..A...............C....A........................C.  Panubis
353056: ..A.....T.........C........C.A.......TG...........  Cjacchus
```

```
                                 LTR
            ---------------------------------------------------->
                    299       309       319
   290: ACGAGAATAGCACAGGAAAGACTGGCCTCCATGATTCAA-----------  Hsapiens
320190: .......................................-----------  Ptroglodytes
337446: .T.....C....T................T.........-----------  Ggorilla
369931: .T.....C...........T...................-----------  Pabelli
294657: .T.....C...............................-----------  Nleucogenys
364122: .T....................CA....T.G........-----------  Mmulatta
316770: .T........T...........CA....T.G........-----------  Panubis
353106: .T...................T.........G.G....ACATGCCCAAA  Cjacchus
```

```
                                 LTR
            ---------------------------------------------------->
                    336       346       356       365       375
   329: --TTGCCTCTCCCTGGGTCCCTCCCACAACATG-TGGGAATTCTGGGAGA  Hsapiens
320229: --..A............................-................  Ptroglodytes
337485: --..A............................-................  Ggorilla
369970: --..A............................-................  Pabelli
294696: --..A...............---------....T..T.............  Nleucogenys
364161: --..AT....                                          Mmulatta
316809: --..AT.........T.................-..............T.  Panubis
353156: ACA.A.....T...............T......-...A............  Cjacchus
```

```
                                LTR
            ------------------------------------------------->
                    385       395       405       415       425
   376: TAAAATTTGAGTTGAGATTTGGATGGGGACACAGTCAAACCATATCATTT  Hsapiens
320276: ..................................................  Ptroglodytes
337532: .................................................C  Ggorilla
370017: ........C..C..........G..........................C  Pabelli
294735: C.................................C...........T..C  Nleucogenys
316856: .......C..............G...........C........G.....C  Panubis
353205: .......A..........................C........G.....C  Cjacchus
```

```
                                  LTR
             --------------------------------------------------->
                    435       445       455       465       475
   426: AGTCCCTGGCCCCTCCAAATCTCATGTCCTCACATTTTAAAACCAATCAC  Hsapiens
320326: ..................................................  Ptroglodytes
337582: ...G.......T......................................  Ggorilla
370067: ..C............................................A.T  Pabelli
294785: ..C.....A.....................T..................T  Nleucogenys
316906: T.C..............................................T  Panubis
353255: T.C............G.....................C...........T  Cjacchus
```

```
                              LTR
         ------------------------------------------------->
                  485       495       505       515       525
   476: GCCTTCCCAACAGTCCCCCAAAGTCTTAACTCATTTCAGCATTAACCCAA Hsapiens
320376: .................................................. Ptroglodytes
337632: A................................................. Ggorilla
370117: .........................G........................ Pabelli
294835: ........................................G......... Nleucogenys
316956: ................T..............................TGG Panubis
353305: ...---------A..................................... Cjacchus

                              LTR
         ------------------------------------------------->
                  535       545       555       565       575
   526: AAGTTCACAGTCCAAAGTCTCACTTGAGACAAGGCAAGTCCCTTCTGTCT Hsapiens
320426: .................................................. Ptroglodytes
337682: .......TG..............C.......................... Ggorilla
370167: .....T.................C.....T.................... Pabelli
294885: .......................C.......................... Nleucogenys
317006: .......................C.......................... Panubis
353346: ......................TC...............-....T..... Cjacchus

                              LTR
         ------------------------------------------------->
                  585       595       605       615       625
   576: ATGAGCCTGTAAAATCAAAAGCAAGTTAGTTACTTCCTAGATACAATGGT Hsapiens
320476: .................................................. Ptroglodytes
337732: .................................................. Ggorilla
370217: .................................................. Pabelli
294935: .................................................. Nleucogenys
317056: ..A......C.............G...................T.....G Panubis
353395: ..........T...C..........C......T...............CA Cjacchus

                              LTR
         ------------------------------------------------->
                  635       645       655       665       675
   626: GGCACAGGAATTGGGTAAATACAGCCATTCCAAATGGGAGAAATTGGACA Hsapiens
320526: .................................................. Ptroglodytes
337782: ...........................................C...... Ggorilla
370267: .....................................A............ Pabelli
294985: .......................................C.......... Nleucogenys
317106: .A..........T......................T..G........... Panubis
353445: ..T..................G.....C...........A...A...C.. Cjacchus

                              LTR
         ------------------------------------------------->
                  685       695       705       715       725
   676: AAACAAAGGGATTACAGGGCCCATGCAAGTTTGAAATCCAGCAGGGCAGT Hsapiens
320576: .................................................. Ptroglodytes
337832: .................................................. Ggorilla
370317: ....................T..C................T......... Pabelli
295035: ...T.............................................. Nleucogenys
317156: ..............................C...G..T.....ACA.... Panubis
353495: ..G.......G........A.A.CA...A.C.............-..... Cjacchus
```

```
                                     LTR
        ------------------------------------------------------->
                    735       745       755       765       775
    726: CAAATTTTAAAGCTCCAAAATAATCTCCTTTGCCTCCAGGTCTCACATCC  Hsapiens
 320626: ..................................................  Ptroglodytes
 337882: ..................................................  Ggorilla
 370367: ..................................................  Pabelli
 295085: ...........A.....C..............A.................  Nleucogenys
 317206: ................................A.................  Panubis
 353544: ....A.........T............T....A........T.......A  Cjacchus

                                     LTR
        ------------------------------------------------------->
                    785       795       805       815       825
    776: AGGTCATGCTGATGCAAGAGGTGGGTTTCCATGGTCTTGGACAGCTCTGC  Hsapiens
 320676: ..................................................  Ptroglodytes
 337932: ..................................................  Ggorilla
 370417: ..................................................  Pabelli
 295135: .......................C..........................  Nleucogenys
 317256: ..................................................  Panubis
 353594: .....G..............T...A..C............G.........  Cjacchus

                                     LTR
        ------------------------------------------------------->
                    835       845       855       865       875
    826: CCCTGTGGCTTTGCAGGATACAGCCTGCCTCCCAGCTGCTTTCATGGCCT  Hsapiens
 320726: ..................................................  Ptroglodytes
 337982: ............................................A.....  Ggorilla
 370467: .................G................................  Pabelli
 295185: .................G........................T.......  Nleucogenys
 317306: A................G........C.......................  Panubis
 353644: TT...............G..T.....C....................G..  Cjacchus

                                     LTR
        ------------------------------------------------------->
                    885       895       905       915       925
    876: GGTGTTGAGTGTGTGTGGCTTTTCCAGGCGCAAAGTACAAGCTGTCAGTG  Hsapiens
 320776: ..................................................  Ptroglodytes
 338032: ..................................................  Ggorilla
 370517: ............C................T....................  Pabelli
 295235: ............CC..............T.....-...........C...  Nleucogenys
 317356: ............C................A..C.................  Panubis
 353694: ..C.........C...............T...C...G.............  Cjacchus

                                     LTR
        ------------------------------------------------------->
                    935       945       955       965       975
    926: GATCTTCCCATTCTGGGGTCTGAAGGACAGTGGCCCTCTTCTCACAGCCC  Hsapiens
 320826: ..................................................  Ptroglodytes
 338082: .G................................................  Ggorilla
 370567: .....-A...........................................  Pabelli
 295284: .....-G.......C.......G...................T.......  Nleucogenys
 317406: .....-A..............AG....TG.....................  Panubis
 353744: .....-A...C...........G....TGA..............A.....  Cjacchus
```

```
                               LTR
         ---------------------------------------------------->
                985       995       1005      1015      1025
    976: CACTAGGCAGTAGCCCAGTAGGGACTCTATGTGGGGGCTTCAACCCCACA  Hsapiens
 320876: ............C..T.........C..G....A................  Ptroglodytes
 338132: ...........GC...............G.....................  Ggorilla
 370616: ...........GC...CA..........G.....................  Pabelli
 295333: ...........GC...............G..........C..........  Nleucogenys
 317455: ...........GC......G......T.G....T.....CTG.T......  Panubis
 353793: ...........GC........A......G....-.....C..........  Cjacchus

                               LTR
         ---------------------------------------------------->
               1035      1045      1055      1065      1075
   1026: TTTCCCTTCTGCACTGCCCTAGCAAAGGTTCTCCATGAGAGGACCACTCC  Hsapiens
 320926: .............................................G....  Ptroglodytes
 338182: ..........................A..................G....  Ggorilla
 370666: .G..........................................TG....  Pabelli
 295383: ...T.......A......................C...............  Nleucogenys
 317505: ..........................A.......................  Panubis
 353842: ......................................---.........  Cjacchus

                               LTR
         ---------------------------------------------------->
               1085      1095      1105      1115      1125
   1076: TACAGCAAACTTTTGCCTGGGCATCCAGGAATTTCTATACATCTTCTGAA  Hsapiens
 320976: ..................................................  Ptroglodytes
 338232: ..................................................  Ggorilla
 370716: ..................................................  Pabelli
 295433: .....T............................................  Nleucogenys
 317555: ..................................................  Panubis
 353889: .G...........................C......G.........CA..  Cjacchus

                               LTR
         ---------------------------------------------------->
               1135      1145      1155      1165      1175
   1126: ATCTAGGCAGAGGTTCCCAAACCTCAATTCTTGACTTCTGTGAACCTGCA  Hsapiens
 321026: ..................................................  Ptroglodytes
 338282: ..................................................  Ggorilla
 370766: ................................T.................  Pabelli
 295483: ..................................................  Nleucogenys
 365036:                              .............C.......  Mmulatta
 317605: .C........................................C.......  Panubis
 353939: .......TG...........C.....................C...CA..  Cjacchus

                               LTR
         ---------------------------------------------------->
               1185      1195      1205      1215      1225
   1176: GGCTCAACACCACATGGAAGCTGCCAAGGCTTGGGTCTTCCACCCTCTGA  Hsapiens
 321076: ...............................................C..  Ptroglodytes
 338332: ..................................................  Ggorilla
 370816: ..................................................  Pabelli
 295533: ..................................................  Nleucogenys
 365057: ......T..................T........................  Mmulatta
 317655: ......T...........................................  Panubis
 353989: .........T.......T.A..AG..........TG..............  Cjacchus
```

```
                                  LTR
         ---------------------------------------------------->
                   1235      1245      1255      1265      1275
   1226: AGCCATAGCCCGAGCTGTACATTGGCCCCTTTCAGCCACTGCTGGAGCAG  Hsapiens
 321126: .....C............................................  Ptroglodytes
 338382: .....C............................................  Ggorilla
 370866: .....C.........CA...T...............T.............  Pabelli
 295583: .....C.............TG.......T.....................  Nleucogenys
 365107: .....C....T.........G..............T............--  Mmulatta
 317705: .....C....T........TG..............T..............  Panubis
 354039: .....C....T.......................................  Cjacchus

                                  LTR
         ---------------------------------------------------->
                   1285      1295      1305      1315      1325
   1276: CTGGGACAGAGGGCACCAAGACCCTAGGCTGCACACAATACGAGGACCCT  Hsapiens
 321176: ....................T.................C..A........  Ptroglodytes
 338432: ....................T.................C..A........  Ggorilla
 370916: ....................T.................C..A.....T..  Pabelli
 295633: ....................T..............G.GC..A.....T..  Nleucogenys
 365155: -----.T.C...........T............T...GC..A.....T..  Mmulatta
 317755: ..A.....C...........T............T...GC..A.....T..  Panubis
 354089: ..A.A...T...........T......T.........GC..AG....T..  Cjacchus

                                  LTR
         ---------------------------------------------------->
                   1335      1345      1355      1365      1375
   1326: GGGCCCAGCCCACAAAACCACTTTTTCCTCCTGGGTCTCCAGGCCTGCAA  Hsapiens
 321226: ..................................................  Ptroglodytes
 338482: ..............C......G................G...........  Ggorilla
 370966: .....TG...................T..-..................T.  Pabelli
 295683: ................................................C.  Nleucogenys
 365200: ......G............................C...........TG.  Mmulatta
 317805: ...T.TG............................C...........TG.  Panubis
 354139: ...T...............................C..........TGG.  Cjacchus

                                  LTR
         ---------------------------------------------------->
                   1385      1395      1405      1415      1425
   1376: TGGGAGGGGCCACCATGAAGGTCTCTGACATGGTCTGGAGACATTTTCCC  Hsapiens
 321276: ..................................................  Ptroglodytes
 338532: ...........G.....................C................  Ggorilla
 371015: ....................T............C................  Pabelli
 295733: ...........T.....................C................  Nleucogenys
 365250: ...........G............TG.......C................  Mmulatta
 317855: ...........G.............G.......C................  Panubis
 354189: ..A.......TG.....................C.....A..........  Cjacchus

                                  LTR
         ---------------------------------------------------->
                   1435      1445      1455      1465      1475
   1426: TATGATCTTGGGGATTAACATTAGGCTCCTTGCTACTTATGCAAATTTCT  Hsapiens
 321326: ..................................................  Ptroglodytes
 338582: ..................................................  Ggorilla
 371065: ............C..............................C......  Pabelli
 295783: ................................................A.  Nleucogenys
 365300: ..................................................  Mmulatta
 317905: ..................................................  Panubis
 354239: ....G.............T.....--------------------.AA...  Cjacchus
```

```
                   LTR
              1485      1495      1505      1515      1525
  1476: GCAGCCAGCTTGAATTTCTCCCCAGAAAATGGGTTTTTCTTTTCTATCAC Hsapiens
321376: ..............................A................... Ptroglodytes
338632: .................................................. Ggorilla
371115: .................................................. Pabelli
295833: .................................................. Nleucogenys
365350: .................T..AT..........--....TA.......... Mmulatta
317955: .................T..AT..........--....TA.......... Panubis
354269: .............G.....T..........................CTG. Cjacchus

                   LTR
              1535      1545      1555      1564      1574
  1526: ATAGTCAGGCTGCAAATTTTCCAAACTTTT-TGCTCTGCTTCCCTTATAA Hsapiens
321426: ..............................-.....C............. Ptroglodytes
338682: .........T....................-................... Ggorilla
371165: .........T....................A............A...... Pabelli
295883: .........T.................C..A................... Nleucogenys
365398: .........T.....G.....T..G.....A................... Mmulatta
318003: ..----...T.....G.....T........A................... Panubis
354319: .........T....................A.....CA............ Cjacchus

                   LTR
              1584      1594      1604      1614      1624
  1575: AACAGAATGCCTTTAACAATACCCAAGTCACCTCTCGAATGCTTTGCTCC Hsapiens
321475: .................................................. Ptroglodytes
338731: .................................................. Ggorilla
371215: ................................G................. Pabelli
295933: ...T................................A............. Nleucogenys
365448: ...T.....T........G..T.TG...T....T.T.............. Mmulatta
318049: ...T.....T......T.G..T......T....T................ Panubis
354369: ...T........................T......T............GT Cjacchus

                   LTR
              1634      1644                1651      1661
  1625: TTAGAAATTTCTTCCACCAGATATCC-------------TAAATCATCAT Hsapiens
321525: ..........................-------------........... Ptroglodytes
338781: ..........................-------------........... Ggorilla
371265: ...................A......-------------........... Pabelli
295983: ..........................-------------....T..... Nleucogenys
365498: ..G....................C..-------------........... Mmulatta
318099: ..G....................CG.-------------........... Panubis
354419: .............TA.TT...A..TTATTTAACTGTTTAG....G...C. Cjacchus

                   LTR
                                            1673      1683
  1662: TT----------------------------TCAAGTTCAAAGTTTCACAA Hsapiens
321562: ..----------------------------.................... Ptroglodytes
338818: ..----------------------------...................G Ggorilla
371302: ..----------------------------.................... Pabelli
296020: ..----------------------------........G........... Nleucogenys
365535: ..----------------------------.................G.. Mmulatta
318136: ..----------------------------.................G.. Panubis
354469: ..CACTAGGATCATTCTCAATGATCCTAAA.................... Cjacchus
```

```
                              LTR
              1693      1703      1713      1723      1733
  1684: ATCTCTAGGGCAGGGGCAAAAAGCCACCAGTCTCTTTGCTAAAACATAAC  Hsapiens
321584: .....................T...........................T  Ptroglodytes
338840: .....................T............................  Ggorilla
371324: .....................T.T..........................  Pabelli
296042: .....................T.........................C..  Nleucogenys
365557: .....................T............................  Mmulatta
318158: .....................T............................  Panubis
354519: ...........---------------------------------------  Cjacchus

                              LTR
              1740      1750      1760      1770      1780
  1734: AAG---AGTCACCTTTGCTCCAGTTCCCAACAAGTTCCTCATCTCCATCA  Hsapiens
321634: ...---............................................  Ptroglodytes
338890: ...---...........................................G  Ggorilla
371374: ...---............................G..............G  Pabelli
296092: ...---................................C..........G  Nleucogenys
365607: ...TTTTT.T....C..................................T  Mmulatta
318208: ...---........C.....................T........T...T  Panubis
354530: ----------............A.....................T..C.T  Cjacchus

                              LTR
              1790      1800      1810      1820      1830
  1781: GAGATCACCTCAGCCTGGACTTTATTGTCCATATTGCTATCAGCATTTTG  Hsapiens
321681: ..................................................  Ptroglodytes
338937: ..................................................  Ggorilla
371421: ..................................................  Pabelli
296139: ..A.C.............................................  Nleucogenys
365657: ....C........G...............T....................  Mmulatta
318255: .............G...............T....................  Panubis
354570: ....................C.............CA.C............  Cjacchus

                              LTR
              1840      1850      1860      1870      1880
  1831: GGCAAAGCCATTCATCAAGTCTCTAGGAAGTTCCAAACTTTCCCACATTT  Hsapiens
321731: ..................................................  Ptroglodytes
338987: .......................................G..........  Ggorilla
371471: .....................................T............  Pabelli
296189: ........................G............T............  Nleucogenys
365707: .............G...T.......................T...T....  Mmulatta
318305: .............G...T.......................T...T....  Panubis
354620: .............G..C...................GT.........G..  Cjacchus

                              LTR
              1890      1900      1910      1920      1930
  1881: TCCTGTCTTCTGAGCCCTCCAAACTATTCCAACCTCTGCCTGTTACCCAG  Hsapiens
321781: ............................T.....................  Ptroglodytes
339037: ..................................................  Ggorilla
371521: ........................................C.........  Pabelli
296239: ..................................................  Nleucogenys
365757: .........................G......................CA  Mmulatta
318355: .........................G......................CA  Panubis
354670: .........................G........................  Cjacchus
```

```
                                    LTR
        ------------------------------------------------------>
                   1940      1950      1960      1970      1980
  1931: TTCCAAAGTCACTTCCACATTTTTGGGTATTTTTTCAGCAACACCCCACT  Hsapiens
321831: ..................................................  Ptroglodytes
339087: ..................................................  Ggorilla
371571: ..............................C...................  Pabelli
296289: ..............................C...C......T........  Nleucogenys
365807: ..............................C.......T...........  Mmulatta
318405: ..........G...................C.......T...........  Panubis
354720: ..........G......T.........C..G..........T...T..TC  Cjacchus

                LTR                      LTR
        ----------------> ------------------------------------>
                   1990      2000      2010      2020      2030
  1981: CCTGGTACCAATTTACTGTATTAGTCTGTTTTCACGCTGCTGATAAAGAC  Hsapiens
321881: ...................................A..............  Ptroglodytes
339137: ...........................A.......A..............  Ggorilla
371621: ..................................................  Pabelli
296339: ................................T.T............AG.  Nleucogenys
365857: .......TT........A................T...............  Mmulatta
318455: .......T....................-....GT...A...........  Panubis
354770: T.......................C..A.......A..............  Cjacchus

                                    LTR
        ------------------------------------------------------>
                   2040      2050      2060      2070      2079
  2031: ATACCTGAGACTGGGAAGAAAAAGAGGTTTCATTGGACTTA-CAGTTCCA  Hsapiens
321931: ................................C........-........  Ptroglodytes
339187: .....................................A...-........  Ggorilla
371671: .........................................---------  Pabelli
296389: ...........C.............................-........  Nleucogenys
365907: ....A....................................A.G......  Mmulatta
318504: .C..A..............................A.....A........  Panubis
354820: ..T...........................AG.........-........  Cjacchus

                                    LTR
        ------------------------------------------------------>
                   2089      2099      2109      2119      2129
  2080: CATGGCTGTGGAGGCCTCAGAATCATGGGGGGAGGTGAGAAGCACTTCTT  Hsapiens
321980: .................................C................  Ptroglodytes
339236: ..................................................  Ggorilla
371712: ------------................C.....................  Pabelli
296438: ............................C...........G.........  Nleucogenys
365957: ........CA..................TA........A.G.........  Mmulatta
318554: ........CA..................CA........A.G.........  Panubis
354869: ..............T.............CAA..A....A..T--.C....  Cjacchus

                                    LTR
        ------------------------------------------------------>
                   2139      2149      2159      2169      2179
  2130: ACATGGTAGTGACAAGAGAAAATGAGGAAGAAGCAAGAGCGGAAACCCCT  Hsapiens
322030: .......G...............................T..........  Ptroglodytes
339286: .......G........................................A.  Ggorilla
371750: .......G..........................................  Pabelli
296488: .......G...............................T..........  Nleucogenys
366007: .......G............................A..T...G..A...  Mmulatta
318604: .......G............................A..T..........  Panubis
354917: ..T....G..AG........................A..T......A...  Cjacchus
```

```
                                 LTR
        -------------------------------------------------->
                     2189      2199      2209      2219      2229
   2180: GATAAACTCATACGATCTCATGAGACTTACTCACTATGACAAGAATAGCA  Hsapiens
 322080: ...................G.........T....................  Ptroglodytes
 339336: ............T......G..............................  Ggorilla
 371800: ......A.....T.....TG..............................  Pabelli
 296538: .......C...CA......G.............G................  Nleucogenys
 366057: .......C...CA.C........A..........................  Mmulatta
 318654: .......C...CA.C...................................  Panubis
 354967: ........T..CA.G.----G..........AC....C............  Cjacchus

                                 LTR
        -------------------------------------------------->
                     2239      2249      2259      2269      2279
   2230: TGGGAAAGACCAACCACCTTGATTCAATTACCTCCCTCTGGGTCCTTCCA  Hsapiens
 322130: ..................................................  Ptroglodytes
 339386: ..................................................  Ggorilla
 371850: ..................................................  Pabelli
 296588: ....................................C.............  Nleucogenys
 366107: ...............C....................A.........C...  Mmulatta
 318704: ............T..CT...................A.........C...  Panubis
 355013: ........T...G..C.................T.TC.........C...  Cjacchus

                                 LTR
        -------------------------------------------------->
                     2289      2299      2309      2319      2329
   2280: CAACACATGGGAATTCTGGGAGATACAATTCAAGATGAGAATTTGGGTGG  Hsapiens
 322180: ..................................G...............  Ptroglodytes
 339436: ..................................G...............  Ggorilla
 371900: ......G........G........G.........G..G............  Pabelli
 296638: .....TG...........................G.....G.........  Nleucogenys
 366157: ..G...............................G.....-.........  Mmulatta
 318754: ..G..T............................G.....-......A..  Panubis
 355063: ....G..............A.............C......-.........  Cjacchus

               LTR
        --------------->
                     2339                2353      2363      2373
   2330: AGACACAGCCAAACC------CCACTTCAGGGGTAGGAGAGAGCATGGGA  Hsapiens
 322230: G..............------.............................  Ptroglodytes
 339486: GA.............------.............................  Ggorilla
 371950: G..............------.............................  Pabelli
 296688: G..............-------..G.....A...........AT......  Nleucogenys
 366206: G....T..T......-------..GG.........A....A.A.....A.  Mmulatta
 318803: G....T..T......-------..GG.........A....A.A.....A.  Panubis
 355112: G........TG....ATATCA.T.GG.......C.A............C.  Cjacchus

                     2383      2393      2403      2413
   2374: GCCCTTCCAGTTACCAGATGAGCTACTGGTAAAAGGCAGAGAGGGAGA--  Hsapiens
 322274: ................................................--  Ptroglodytes
 339530: ................................................--  Ggorilla
 371994: ...............T................................--  Pabelli
 296732: .G.............C.G..............................--  Nleucogenys
 366250: ..............................................C.--  Mmulatta
 318847: ................................................--  Panubis
 355162: ......-...G.......GT...............AT.....A..GA.--  Cjacchus
 201698:                                ....AG..........GGA  Sboliviensis
```

```
                        2431      2441      2451      2461
                           :         :         :         :
  2422: ----------AAACAAATAGAAAGAAAAAAATAATAATGGAAGAGGAAAG  Hsapiens
322322: ----------........................................  Ptroglodytes
339578: ----------.............T..........................  Ggorilla
372042: ----------....G...................................  Pabelli
296780: ----------.....................G..................  Nleucogenys
366298: ----------...TG...........................A.A.....  Mmulatta
318895: ----------...TG...........................A.A.....  Panubis
355209: ----------.......C............GC..C..A............  Cjacchus
201717: TGGAAGAAAG...GG..A............GA..G..G.A....A.....  Sboliviensis

              2471      2481      2491      2501      2511
                 :         :         :         :         :
  2462: CCTGGTACTACAATAAAAAAGAAAAGAAGGATTCTTAAAAATTGGTTTTG  Hsapiens
322362: ..................................................  Ptroglodytes
339618: ................................................G.  Ggorilla
372082: ..................................................  Pabelli
296820: .......................................G..........  Nleucogenys
366338: ........................................G.........  Mmulatta
318935: ........................................G.........  Panubis
355249: ..C.A.....T..............A..............T..A......  Cjacchus
258521:           ...A......A....A...TT..TG.G....C.A..      Sboliviensis

              2521      2528      2538      2548      2558
                 :         :         :         :         :
  2512: GAAGGAAAAA---AACCTACATATGCACCCAGAGAGGCCTGTGTGCTCTC  Hsapiens
322412: ..........---.....................................  Ptroglodytes
339668: A.G.A.....---.....................................  Ggorilla
372132: ..........---.............--............C.........  Pabelli
296870: ..........----.A..........--......................  Nleucogenys
366388: ..........-----...........--................AT....  Mmulatta
318985: ..........-----...........--................A.....  Panubis
355299: ....A.....GAA..T..........--......................  Cjacchus

              2568      2578      2588      2598      2608
                 :         :         :         :         :
  2559: AGTTAAGCTCCTTTGTCATGTTCTTCTGTGAAGGAAAATAGACATTATTT  Hsapiens
322459: ..................................................  Ptroglodytes
339715: ..................................................  Ggorilla
372177: .............................A...A............G...  Pabelli
296914: .................................A............G...  Nleucogenys
366431: G.....A...............T...................T...G...  Mmulatta
319028: G.....A...............T.C.................T...G...  Panubis
355347: .........T....A.......T.........T.........T.......  Cjacchus

              2618      2628      2638      2648      2658
                 :         :         :         :         :
  2609: TCCAATTGTCAATTTTAATTGGAAAATTTTTGAACAATTTTAATTCTTTA  Hsapiens
322509: ..................................................  Ptroglodytes
339765: ..................................................  Ggorilla
372227: ...............................T..................  Pabelli
296964: ...............................A...........C......  Nleucogenys
366481: .....C.........................A..................  Mmulatta
319078: .....C.........................A..................  Panubis
355397: C......-..........C......G...AAA.............A....  Cjacchus
260241:    ..C.A....A..ATT...C.....AG...T.T........A...     Sboliviensis
```

```
              2668      2678      2688      2698      2708
  2659: AACAGCAATGCTCCAGGTAATGGCATTACTGACCCTTGCCTACTCACTTT   Hsapiens
322559: ....A...........................................G.   Ptroglodytes
339815: ....A...........................................G.   Ggorilla
372277: ....A...........................................G.   Pabelli
297014: ....A...........................................G.   Nleucogenys
366531: ....A...........C.................................   Mmulatta
319128: ....A...........C.................................   Panubis
355446: ..-.A.......T...............................A...A.   Cjacchus
274772:                                               ....   Sboliviensis

                        2717      2727      2734      2744
  2709: TT-----------TTTTTTTAATTCAGATTACAAA---TCACTTGGAAAA   Hsapiens
322609: ..-----------......-...............---............   Ptroglodytes
339865: ..-----------......-...............---............   Ggorilla
372327: ..-----------.....A-...............---......A.....   Pabelli
297064: G.-----------.....A-...............---............   Nleucogenys
366581: ..-----------.AAA---...............---.........G..   Mmulatta
319178: ..-----------.AAA---...............---.........G..   Panubis
355495: A.ATATATATATA.A.A..-TT..T..........---....C...GG..   Cjacchus
274776: ..-----------......A.......                          Sboliviensis
401275:                               ..C..GTT.....GAA....   Sboliviensis

              2754      2764      2774      2784      2794
  2745: ACACAATACATTTAATAGTTAAACACCAGCTTTAAAAAACAAAAATGAGA   Hsapiens
322644: ..............G...........T.......................   Ptroglodytes
339900: ....T.........G.............................G.....   Ggorilla
372362: ..............G...................................   Pabelli
297099: ..............G...................................   Nleucogenys
366614: ..............G...................................   Mmulatta
319211: ..............G...................................   Panubis
355541: ...TG...T.....T.....GG...................G.....CA.   Cjacchus
401295: TT.T.............                                    Sboliviensis

                             Simple
              2804      2814      2824      2834      2844
  2795: TGCATTAAAATATAAGATAGGATAGGATACATCAGAAGAGTAGAATAGAA   Hsapiens
322694: ..................................................   Ptroglodytes
339950: ..............----...........G....................   Ggorilla
372412: ............C...........T..C.G....................   Pabelli
297149: .............................G....................   Nleucogenys
366664: ..A..........................G....................   Mmulatta
319261: ..A..........................G....................   Panubis
355591: ...............C...A.........G..........-----.....   Cjacchus

         Simple
              2854      2864      2874      2884
  2845: TAGGATAGACCATGAAGCCTACTCATAGCAGGAGCAAATATTCTTTTGT-   Hsapiens
322744: .........T.......................................-   Ptroglodytes
339996: .........T............................C..........-   Ggorilla
372462: .........T.......................................-   Pabelli
297199: .........T........A.......................G......-   Nleucogenys
366714: .........T.............................G..G......T   Mmulatta
319311: .........T.............................G..G......T   Panubis
355636: .........T..................T...G.....A...T......-   Cjacchus
418443:                                           .CCC..A-   Sboliviensis
```

```
                2899      2909      2919      2929      2939
                   :         :         :         :         :
  2894: ----GTGTATGCACCCATGCATATGATCAAGTAGAAACGTATTAGGAGGT  Hsapiens
322793: ----..........................................G...  Ptroglodytes
340045: ----..................................T.....A.G...  Ggorilla
372511: ----..................................T..A....G...  Pabelli
297248: ----A.A........A.....................GT.......G...  Nleucogenys
366764: TTGT..T..............................GT.......G...  Mmulatta
319361: TTGT..T..............................GT.......G...  Panubis
355685: ----..C..............................GT.......G...  Cjacchus
418450: ----A.A.G.A.T..T.AA.T..A..AA...G.....A------------  Sboliviensis

                2949      2959                2972      2982
                   :         :                   :         :
  2940: CATTATCAAAATTACTTGACAGCCACTC-------ACCAAGAAGTGGGTC  Hsapiens
322839: ..............G.............-------...............  Ptroglodytes
340091: ............................-------...............  Ggorilla
372557: ............C...............-------...............  Pabelli
297294: ..........G.................-------...............  Nleucogenys
366814: ............G...............-------...............  Mmulatta
319411: ............G...............-------...............  Panubis
355731: ....................T.......C------...............  Cjacchus
418484: .......TTT...G..A....AATG...TGATAAA.GG..T..AG.T..T  Sboliviensis

                          2998      3003      3013      3023
                             :         :         :         :
  2983: CACATTTA----GTGATGATT-----CCACTCAAGGCTTGACATCCCTTT  Hsapiens
322882: ........----.........-----........................  Ptroglodytes
340134: ........----.........-----........................  Ggorilla
372600: ........----.........-----........................  Pabelli
297337: ........----.........-----........................  Nleucogenys
366857: .....C..----.........-----........................  Mmulatta
319454: .....C..----.........-----........................  Panubis
355775: ........----.........-----.T.T..........G.....T...  Cjacchus
418534: ..T.CCC.GTAC.....C.C.AACCCTT....TCA...CA.A..T.A..-  Sboliviensis

                3033      3043      3053      3063      3073
                   :         :         :         :         :
  3024: AAATTTCTTCTTAAACATTTAAGGCTTAATATGAAAACTCTTGGACCCCA  Hsapiens
322923: ...............T..................................  Ptroglodytes
340175: ...A...........T..............................G...  Ggorilla
372641: ...A...........T........T.........................  Pabelli
297378: ...A...........T..................................  Nleucogenys
366898: ...A.............................G..............T.  Mmulatta
319495: ...A.............................G..............T.  Panubis
355816: ...A.G.........T..G..T.........C..C...............  Cjacchus
418583: ----....C.....G........                             Sboliviensis

                3083      3093      3103      3113      3123
                   :         :         :         :         :
  3074: CCACTCAGCACAGTTCTTGCTCTTCACGCTTTAGTACACTTGGACTCTGT  Hsapiens
322973: ..................................................  Ptroglodytes
340225: ..................................................  Ggorilla
372691: ..................T........A......................  Pabelli
297428: ...........................A....G.................  Nleucogenys
366948: ...TG.....................A.....G.................  Mmulatta
319545: ...TG...........................G.................  Panubis
355866: ....C....T..............T.TA......................  Cjacchus
```

```
              3132      3142      3152      3162      3172
                 :         :         :         :         :
  3124: GAGGATGC-TGTAGTGAGCTGGGCTTTCTGCCAAAGCAAAGAGTATAACT   Hsapiens
323023: ........C...G.....................................   Ptroglodytes
340275: ........C...G.....................................   Ggorilla
372741: ........C..C..............................C......C   Pabelli
297478: ........C...G....................................C   Nleucogenys
366998: .....A..C...GA........................G..........C   Mmulatta
319595: .....A..C...GA........................G..........C   Panubis
355916: ...A....C..C.....T.............TG.....G.........GC   Cjacchus
512592:  .A.....-....T.A.C.....T.C...A-------G.GA.A..G....   Sboliviensis

              3182      3192      3202      3212      3222
                 :         :         :         :         :
  3173: CTCAGAGTATTCATTCTTCTTGGTGAGGGGGTAGAGGGCTAGAAGGAGGT   Hsapiens
323073: ...T..............................................   Ptroglodytes
340325: ........G.........................................   Ggorilla
372791: ....C...G..................A......................   Pabelli
297528: ....C...G..................A......................   Nleucogenys
367048: ....C...G.............A....A......................   Mmulatta
319645: ....C...G.............A....C......................   Panubis
355966: ....T...G..................A....G...A......C......   Cjacchus
512633: TA....A.G.A.G.                                       Sboliviensis
608986:                       ...T....A.GTG...GGG..G..G...   Sboliviensis

              3231      3241      3251      3261      3271
                 :         :         :         :         :
  3223: GGGA-CCATCACATGCTATCACCAAGATTGTCGTGAGGATTTCCTTTTTA   Hsapiens
323123: ....-...........................C.................   Ptroglodytes
340375: ....-.............................................   Ggorilla
372841: ....-.T.............G.............................   Pabelli
297578: ....-......................C....A.................   Nleucogenys
367098: ...G-G...........G..............A..............G..   Mmulatta
319695: ...G-G....G......G..............A..............G..   Panubis
356016: ...GC........G...G..............A.................   Cjacchus
609014: ..T.-..T.TT.C....                                    Sboliviensis

              3281      3291      3301      3311      3321
                 :         :         :         :         :
  3272: AGCTTTATAGCAGAGACCCAGATAATATGACCTTCTGGTCATTGGAGGGA   Hsapiens
323172: ..................................................   Ptroglodytes
340424: ..................................................   Ggorilla
372890: .......C..T.........................T.............   Pabelli
297627: ......................................C.T.........   Nleucogenys
367147: ...................G.....................C........   Mmulatta
319744: .........................................C........   Panubis
356066: ....C.....--------..................-.............   Cjacchus
685991:                        .....TT..C.TACC.G...A....C.   Sboliviensis

              3331      3341      3351      3361      3371
                 :         :         :         :         :
  3322: GCAGGGCCTAAAGAATTCTTTTCTTATTATAAAAAATATCCCTGAAATAT   Hsapiens
323222: ..................................................   Ptroglodytes
340474: .........                                            Ggorilla
341084:          .........................................   Ggorilla
372940: ...A..............................................   Pabelli
297677: ...T.C..........................G.................   Nleucogenys
367197: .....C.T........................G.................   Mmulatta
319794: .....C.T....................G...G.................   Panubis
356107: A....C......T.......-----------.T..T.G.........C..   Cjacchus
686018: .TGA..TT..C.T......A..AA........                     Sboliviensis
```

```
               3381      3391                3404      3414
                  :         :                   :         :
   3372: CTTTCTACAGTCAACTGCTACCTCT-------CCAGGGGCCAGATACAGA  Hsapiens
 323272: .........................-------..................  Ptroglodytes
 341125: .........................-------..................  Ggorilla
 372990: ......G.................C-------..................  Pabelli
 297727: ............T............-------..................  Nleucogenys
 367247: .........A...............-------..................  Mmulatta
 319844: .........A...............CCAGGGG..................  Panubis
 356146: ....T..----------........-------..............T...  Cjacchus

               3424      3434      3444      3454      3464
                  :         :         :         :         :
   3415: GGATTTGAGACCAGCTGATAATAGACACATTATTCTTATTTAGAACCAGT  Hsapiens
 323315: ..................................................  Ptroglodytes
 341168: ..................................................  Ggorilla
 373033: .................................................C  Pabelli
 297770: .................G...C............................  Nleucogenys
 367290: ..................C..C............................  Mmulatta
 319894: ..................C..C............................  Panubis
 356179: ..........A.G..............AC.........C...........  Cjacchus

               3474      3484      3494      3504      3514
                  :         :         :         :         :
   3465: TTCTACACGTGGGTAGATTTCCCAGTGCTTCAGCGAATCAGAGAGCATCT  Hsapiens
 323365: .......T.........................T................  Ptroglodytes
 341218: ...C....T........................T.........G......  Ggorilla
 373083: .................................T................  Pabelli
 297820: ........A........................T................  Nleucogenys
 367340: ........A........................A.........--.T...  Mmulatta
 319944: ........A........................A.........--.T...  Panubis
 356229: ........A........C...T...........T.......G........  Cjacchus

               3524      3534      3544      3554      3564
                  :         :         :         :         :
   3515: TTAAGAAAAGGGTTCATGAATTTTTGCTTCACTTTATATTACCTGATCTT  Hsapiens
 323415: ..........A.......................................  Ptroglodytes
 341268: ..................................................  Ggorilla
 373133: ....................................A.............  Pabelli
 297870: ................C........................TA.......  Nleucogenys
 367388: .......G..........................................  Mmulatta
 319992: ..................G...............................  Panubis
 356279: ..........A.............A................T.......A  Cjacchus
1008277:   ........A..............................T.......A  Sboliviensis

               3574      3584      3594      3604      3614
                  :         :         :         :         :
   3565: TTCTAGACAGTAAAAAATTCAGTCACTATAAAATTGAAGTCCTATCAGGC  Hsapiens
 323465: ..................................................  Ptroglodytes
 341318: ....................G...........................A.  Ggorilla
 373183: ....................G.............................  Pabelli
 297920: ..................GTG.............................  Nleucogenys
 367438: ...............C...TG.......................A.....  Mmulatta
 320042: ...................TG.......................A.....  Panubis
 356329: ...................TG.G...........................  Cjacchus
1008325: ..................GTG.........................G...  Sboliviensis
```

```
               3624      3634      3644      3654      3664
                  :         :         :         :         :
   3615: AGAAAGTGAACAAGCATCTGAGGAAAAAGATATCTGTTCCATTGCTGTTG Hsapiens
 323515: .................................................. Ptroglodytes
 341368: .................................................. Ggorilla
 373233: ..............T..................T................ Pabelli
 297970: ..............T..................T................ Nleucogenys
 367488: ..............T......A....T......T................ Mmulatta
 320092: ..............T...........T......T.....T.......... Panubis
 356379: .AG..C........T.............C....T................ Cjacchus
1008375: .AG..C........T........G....A...CT................ Sboliviensis

               3674      3684      3694      3704      3714
                  :         :         :         :         :
   3665: TTGATGTCTGCTTCTGGCCATAATTAGTTATTGGGATAAGTCATGGTAAC Hsapiens
 323565: .........................G........................ Ptroglodytes
 341418: ......................G..G........................ Ggorilla
 373283: G.....................G..G........................ Pabelli
 298020: G................G....G..G........................ Nleucogenys
 367538: G..G.............G....G..-.C.T.................... Mmulatta
 320142: G..G.............G....G..-.C.T.................... Panubis
 356429: G..G.....CT.....AATG..G..G.......A.....C.T........ Cjacchus
1008425: G..G.....C......AATG..G..G.......A.....A.......... Sboliviensis

               3724      3734      3744      3754      3764
                  :         :         :         :         :
   3715: AACGCTACTAAAAATTTCTGGGGGTTTATATCTTTTAACATTTTAAGATC Hsapiens
 323615: .....................A............................ Ptroglodytes
 341468: ...................................A.............. Ggorilla
 373333: ..T.A..............................A...C.......... Pabelli
 298070: ..T................................G.............. Nleucogenys
 367587: ..T.........T......................G.............. Mmulatta
 320191: ..T.........T......................G.............. Panubis
 356479: ..T..C.................C...........A....C.....A... Cjacchus
1008475: ..T..C.................C...........G....C.....A... Sboliviensis

               3774      3784      3794      3804      3814
                  :         :         :         :         :
   3765: ACATTGATCATGCCTTTGTTATCTGCTATATAGGCCAAAGTTGGCAATGA Hsapiens
 323665: .................................................. Ptroglodytes
 341518: .................................................. Ggorilla
 373383: .................................A.............C.. Pabelli
 298120: ............................................T..... Nleucogenys
 367637: ..G............................................... Mmulatta
 320241: ..G...C..............................G............ Panubis
 356529: ..........C.........T...A.C.....A................T Cjacchus
1008525: .....................C....C.....A..G...........C.G Sboliviensis

               3824      3834      3844      3854      3864
                  :         :         :         :         :
   3815: CAATGATACAGTATGTGTGTTTGTGCATGCATGCATGTACTTTAGAGAGA Hsapiens
 323715: .................................................. Ptroglodytes
 341568: ........G.........T............................... Ggorilla
 373433: .................................................. Pabelli
 298170: .................................................. Nleucogenys
 367687: .................................................. Mmulatta
 320291: .................................................. Panubis
 356579: ..........T...A.......................G........... Cjacchus
1008575: .GG........C...............C..........G........... Sboliviensis
```

```
                    3874      3884      3894      3900      3910
                       :         :         :         :         :
   3865: GAAAGAGGGAGAGCAGGAGTGATAGAGAGAGA----GACACAGAGAGATA  Hsapiens
 323765: ................................----..............  Ptroglodytes
 341618: ......................C.........----..............  Ggorilla
 373483: ............A......C......C.A...--------------....  Pabelli
 298220: A..................CT...........----..G...........  Nleucogenys
 367737: .............AG....A......C.....------.........G..  Mmulatta
 320341: .............AG....A......A.....CAGA...........G..  Panubis
 356629: .........GA..AG....A..C..CC.....------.........--T  Cjacchus
1008625: .........G...AG....A.GC...C...C.--------.......--T  Sboliviensis

                    3920      3930      3940      3950      3960
                       :         :         :         :         :
   3911: CAGAGAAGCTACGCACCATGAGAATGTGGCAATGTGATAGTAACACAAAG  Hsapiens
 323811: ..................................C...............  Ptroglodytes
 341664: ...........T......................................  Ggorilla
 373519: ..............G......................C............  Pabelli
 298266: ...........A.........................C............  Nleucogenys
 367781: ...........A..G..................A...C............  Mmulatta
 320391: ...........A..G..................A...C............  Panubis
 356671: ...........A..G......................C........C...  Cjacchus
1008665: ...........A.TG......................C........C...  Sboliviensis

                    3970      3980      3990      4000      4010
                       :         :         :         :         :
   3961: TATGAAGAAATGCTTATTAAAATGGAAGGACAACACACCCTTCTGATCCT  Hsapiens
 323861: ..................................................  Ptroglodytes
 341714: ..................................................  Ggorilla
 373569: ...............G.............C....................  Pabelli
 298316: .......................A....................A.....  Nleucogenys
 367831: .......................A.......................T..  Mmulatta
 320441: .......................A.............-.........T..  Panubis
 356721: .....................GCA..........................  Cjacchus
1008715: .............---......CA..........................  Sboliviensis

                    4020      4030      4040      4050      4060
                       :         :         :         :         :
   4011: CCTGATGAAGCTACTTGTACTTGAGGGAGCAGATTAGGGATTCATAGGCA  Hsapiens
 323911: ................................................T.  Ptroglodytes
 341764: ......................................A...........  Ggorilla
 373619: ...........................C......................  Pabelli
 298366: .............T....................................  Nleucogenys
 367881: .........................A...........C...C..C.....  Mmulatta
 320490: -...................-................C...C........  Panubis
 356771: .........................-......C.C.T....-..C.-...  Cjacchus
1008762: ..................................CC........C.A...  Sboliviensis

                    4070      4080      4090      4100      4109
                       :         :         :         :         :
   4061: ATCAAGAAAGGAACCCTTGGCAAAACCTAGGAAGGTAAAC-TAGATTCTG  Hsapiens
 323961: ........................................-.........  Ptroglodytes
 341814: ........................................-.........  Ggorilla
 373669: ............................T...........-.........  Pabelli
 298416: ........................................-.........  Nleucogenys
 367931: .......................................G-.........  Mmulatta
 320538: .......................................G-.........  Panubis
 356818: ....GA..--.G.TT....A....                            Cjacchus
 357329:                         ..T.......A.....T....C....  Cjacchus
1008812: .........T................T......AA.....-.........  Sboliviensis
```

```
               4119      4129      4139      4149      4158
                  :         :         :         :        :
    4110: GACTTGAAGGGGTGCTCTGCTATTATTTTGTTTAGCATACTGCC-AGTGC  Hsapiens
  324010: ............................................-.....  Ptroglodytes
  341863: ............................................-.....  Ggorilla
  373718: .........-..................................-.....  Pabelli
  298465: .............T.............................T-.....  Nleucogenys
  367980: ...C-...............................G..G....-.....  Mmulatta
  320587: ...C-...............................G..G....-.....  Panubis
  357355: .........T............................CG....C..G..  Cjacchus
 1008861: .............A................C........G....C..G..  Sboliviensis

               4168      4178      4188      4198      4208
                  :         :         :         :         :
    4159: TTACAGTTAAATGAAAGATTAAGCAATGCATTGTAGAAGGTTACAGAATG  Hsapiens
  324059: ..............................C...................  Ptroglodytes
  341912: ..................................................  Ggorilla
  373766: .................G................................  Pabelli
  298514: ..................................................  Nleucogenys
  368028: ......................................A.........G.  Mmulatta
  320635: ......................................A...........  Panubis
  357405: .......................................C.........A  Cjacchus
 1008911: .......................................C.........A  Sboliviensis

                                                DNA
                                    ---------------------->
               4218      4228      4238      4248      4258
                  :         :         :         :         :
    4209: TGTTTCATTAGGAACTCCCAAGAGCAGAGATTGGTCAACTTTTTCTGCAA  Hsapiens
  324109: .A................................................  Ptroglodytes
  341962: ..................................................  Ggorilla
  373816: ......G.....................A.....................  Pabelli
  298564: ..................................................  Nleucogenys
  368078: ...................T..........G...C..........C....  Mmulatta
  320685: ...................T..........G...................  Panubis
  357455: ........................T..GT...A.C...............  Cjacchus
 1008961: ........................T.......A.C...........C...  Sboliviensis

                               DNA
          ------------------------------------------------->
               4268      4278      4288      4298      4308
                  :         :         :         :         :
    4259: GAAGCCAAATAGTAAATATGTCTGGCTTTGTGGGCCATGCATTCATCCAG  Hsapiens
  324159: ..T...............................................  Ptroglodytes
  342012: .................................................T  Ggorilla
  373866: ......C..C.......G...T................A..........T  Pabelli
  298614: ..C..................T................AT.........T  Nleucogenys
  368128: .........G...........T................AT..CT.....T  Mmulatta
  320735: .........G...........T................AT..CT.....T  Panubis
  357505: .G.....G...........T.T..A.............AT.G.......T  Cjacchus
 1009011: .G.....G..........GT.T..A.............AT.G.....TGT  Sboliviensis
```

DNA

```
                4318      4328      4338      4348      4358
   4309: CACAACTCAATTTTTCAGCAGTGACACTAAAGCAGACATAGGAAATATGT  Hsapiens
 324209: ..........................T................C......  Ptroglodytes
 342062: ..........................T.......................  Ggorilla
 373916: ................C.T.......T.......................  Pabelli
 298664: ..................T.A.....T.......................  Nleucogenys
 368178: ..................TT...G..T.......................  Mmulatta
 320785: .....T............TT...G..T.......................  Panubis
 357555: .........G........TG...GGCT..........G.........G..  Cjacchus
1009061: .................AT....G..T.............A......G..  Sboliviensis
```

DNA

```
                4368      4378      4388      4398      4408
   4359: AAGCAAATAGACATGGCTGTGTTTCAATAAAATTTTATTTATAAAATCAG  Hsapiens
 324259: ..................................................  Ptroglodytes
 342112: ................................A.................  Ggorilla
 373966: ..................................................  Pabelli
 298714: .........................G..............G.........  Nleucogenys
 368228: ..A.......G...............................G.....G.  Mmulatta
 320835: ..A.......G..............G................G.....G.  Panubis
 357605: ..A.....................TG...............C........  Cjacchus
1009111: ..A.....G...............TG........................  Sboliviensis
```

DNA

```
                4418      4428      4438      4448      4457
   4409: GTGTCCAGCCAGTTTTGGACCAATGACCAGAGTTTGCCAACCCCTAC-CC  Hsapiens
 324309: ...............................................-..  Ptroglodytes
 342162: A..............................................-..  Ggorilla
 374016: .........................GA....................-..  Pabelli
 298764: .......................C.G.....................-..  Nleucogenys
 368278: ........T..............G.G...........T.....T...-..  Mmulatta
 320885: .......................G.G...........T....T....-..  Panubis
 357655: ..T.....A...........A..A.G.....A.........T.TGTT-A.  Cjacchus
1009161: ........A.............---G...............T.TG.TAA.  Sboliviensis
```

```
                4467      4477      4487      4497      4507
   4458: TAAAGTCCTTGGCAAACAACACAGTTAAATCTTGACAGATCTGTAACTGA  Hsapiens
 324358: ..............................A...................  Ptroglodytes
 342211: .................................A...............G  Ggorilla
 374065: ..................................................  Pabelli
 298813: ...........T......................................  Nleucogenys
 368327: .........G.......G...TG...........................  Mmulatta
 320934: .........A...........TG...........................  Panubis
 357704: .......T........TG....C......C...C................  Cjacchus
1009208: ...G...T..........G..............C................  Sboliviensis
```

```
                     4517      4527      4537      4547      4557
                        :         :         :         :         :
    4508: CCTTACAACTTTATTGCACCAGCTAAATCCAGCAACAGATAATAGCAAAT  Hsapiens
  324408: ..................................................  Ptroglodytes
  342261: .T................................................  Ggorilla
  374115: .T..............T.....T...........................  Pabelli
  298863: .T......G.............T...........................  Nleucogenys
  368377: .T......-........G....T....C......................  Mmulatta
  320984: .T......-........G....T....C......................  Panubis
  357754: .T................................................  Cjacchus
 1009258: .T............................................C...  Sboliviensis

                     4567      4577      4587      4597      4607
                        :         :         :         :         :
    4558: TAGCATCTCTCTTGATTTTCACAAGCTTTGATATTATTAATACTACTTAG  Hsapiens
  324458: ..................................................  Ptroglodytes
  342311: .......................................G..........  Ggorilla
  374165: ..........................A............G..........  Pabelli
  298913: ...............C..........A..T.........G..........  Nleucogenys
  368426: ...............C..........A............G.....T..T.  Mmulatta
  321033: ...............C..........A............G.....T..T.  Panubis
  357804: C..............CC.........AA....G......G.....T...A  Cjacchus
 1009308: .........C.....C..........CA....G......G.....T....  Sboliviensis

                                   MIR
                ------------------------------------------------>
                     4617      4627      4637      4647      4657
                        :         :         :         :         :
    4608: TTTTTGATTCCCATTTTGCAGCTGAGGATTGAGATTTCAAGTTATTCAAT  Hsapiens
  324508: ..................................................  Ptroglodytes
  342361: ..........................AC.............C........  Ggorilla
  374215: ..........................A..............C........  Pabelli
  298963: ..........................A..............C........  Nleucogenys
  368476: .....T....................A..............C........  Mmulatta
  321083: .....T....................A..............C.......C  Panubis
  357854: .....A........................------.....C.......C  Cjacchus
 1009358: ..........................A...------.....C........  Sboliviensis

                              MIR
          -------------------------------------------------->
                     4667      4677      4687      4697      4707
                        :         :         :         :         :
    4658: TGCTTTCCCAGGGCTATACATGTTATAATGGATGAGAGAGGATTTACACA  Hsapiens
  324558: ......T...........................................  Ptroglodytes
  342411: ......T............C..............................  Ggorilla
  374265: ......T.................G..........C..............  Pabelli
  299013: ......TTG...............G....................G....  Nleucogenys
  368526: ......T...A.............G.............G...........  Mmulatta
  321133: ......T...A.A.....T.....G.............G...........  Panubis
  357898: ......T...A.A...C.T...........A.........A.........  Cjacchus
 1009402: ......T...A.A...C.......G....AA.........A.........  Sboliviensis
```

```
                MIR
         4717      4727      4737      4745      4755
   4708: CCATTTTTTGGCTTTATAAGAATCAGAAAACCTG--AGCTAAATTTTCAT  Hsapiens
 324608: ..................................--..............  Ptroglodytes
 342461: ..................................--..............  Ggorilla
 374315: ...A......A.......................--..............  Pabelli
 299063: ................C.................--..............  Nleucogenys
 368576: ..........A.......................GA..............  Mmulatta
 321183: ..........A.......C...............GA..............  Panubis
 357948: ........-.......................CAGA.....T.......C  Cjacchus
1009452: .T.G.G..-.......................CAGA.....TG......C  Sboliviensis

                                                LINE1
         4765      4775      4785      4795      4805
   4756: TTTGTGGCTGATTGAATTTCCTAGACAAAGAATGTGAAACTCAGAGTGAG  Hsapiens
 324656: ..................................................  Ptroglodytes
 342509: ......A.............T.............................  Ggorilla
 374363: ....................T.............................  Pabelli
 299111: ....................T.............................  Nleucogenys
 368626: .A............................G.......G.........G.  Mmulatta
 321233: ...........C..........................G.........G.  Panubis
 357997: .....T.TG.G.....................G............A..G.  Cjacchus
1009501: .....T..A.G.....C...............G...............G.  Sboliviensis

                                   LINE1
         4815      4825      4835      4845      4855
   4806: AGAAAATTTTTGCAATCTATCCATCTTACAAAGGTCTCATATCCAGAAAC  Hsapiens
 324706: ..................................................  Ptroglodytes
 342559: .................................A................  Ggorilla
 374413: ..................................................  Pabelli
 299161: .................................T................  Nleucogenys
 368676: .......G..................G..........A..........T.  Mmulatta
 321283: .......G..................G......A...A..........T.  Panubis
 358047: ..G....A..................GG.........A.......C.TTT  Cjacchus
1009551: ..G.......................GG.........A.........T.T  Sboliviensis

                                   LINE1
         4865      4874      4884
   4856: CACAAGGGAC-TTTAACAAATTTACAAGAAAAAAAA--------------  Hsapiens
 324756: ..........-.........................--------------  Ptroglodytes
 342609: ..........-.....T...................--------------  Ggorilla
 374463: ..........-.........................--------------  Pabelli
 299211: .......A..-.........................--------------  Nleucogenys
 368726: ..........-.........................--------------  Mmulatta
 321333: .......A..-....................T....--------------  Panubis
 358097: .......A..T.........................--------------  Cjacchus
1009601: .......A..-................A...C....ACAAAAAACAAACA  Sboliviensis
```

```
                                   LINE1
         ---------------------------------------------------------->
                                                        4898      4908
                                                           :         :
   4891: --------------------------------CAAACAACTCTATTAAAC  Hsapiens
 324791: --------------------------------..................  Ptroglodytes
 342644: --------------------------------..................  Ggorilla
 374498: ------------------------------AA....G.............  Pabelli
 299246: --------------------------------............A....A  Nleucogenys
 368761: ------------------------------AA.................A  Mmulatta
 321368: -------------------------------A.................A  Panubis
 358133: -------------------------------A........C........A  Cjacchus
1009650: AAACAAAACAAAACAAAACAAAACAAAACAAAA.......C........A  Sboliviensis

                                  LINE1
         -------------------------------------------------------->
                4918      4928      4938      4948      4958
                   :         :         :         :         :
   4909: AGTGGGCTAAGCACATGAACAGATACTTCTCAAAAGAAGACATTCATGTA  Hsapiens
 324809: ..................................................  Ptroglodytes
 342662: ..................................................  Ggorilla
 374518: .......A..........................................  Pabelli
 299264: .......A.....T.................................A..  Nleucogenys
 368781: .......-.......................................A..  Mmulatta
 321387: .......-........C.............................CA..  Panubis
 358152: .......A...............C............G.............  Cjacchus
1009700: .......A...............C..........................  Sboliviensis

                4968      4978      4988      4998      5008
                   :         :         :         :         :
   4959: CTACTAACTCTTAACATGGAATGGAAACTTTCAAAAATGGTGATAGCGAT  Hsapiens
 324859: ...............................................A..  Ptroglodytes
 342712: ..G............................................A..  Ggorilla
 374568: ...............................................A..  Pabelli
 299314: ......................A........................A.C  Nleucogenys
 368830: ......................A...............C...G....A..  Mmulatta
 321436: ......G...............A...............C...G....A..  Panubis
 358202: T........T.A..........A..........C.....T.......AG.  Cjacchus
1009750: T........T............A..........C.............A..  Sboliviensis

                5018      5028      5038      5048      5058
                   :         :         :         :         :
   5009: TGAAATAGGAAAAGGTATAGAATTTGAGTGAAAGAAGGCTTTTAATTCAC  Hsapiens
 324909: ..................................................  Ptroglodytes
 342762: ....................................A.............  Ggorilla
 374618: ................................G.................  Pabelli
 299364: ...........................A....G.................  Nleucogenys
 368880: ........................-.......G.................  Mmulatta
 321486: ..............C.........-.......G.................  Panubis
 358252: ...T.C...-...T..................G............A....  Cjacchus
1009800: ...T.C.......T..................G..............G..  Sboliviensis

                5068      5078      5088      5098      5108
                   :         :         :         :         :
   5059: TTTATTGCCAACTACAAAGAAATTAGAGTCTTTATAAGTTTCTTTATTTC  Hsapiens
 324959: ............................................C.....  Ptroglodytes
 342812: ............................................C.....  Ggorilla
 374668: ............................................C.....  Pabelli
 299414: .......................G....................C.....  Nleucogenys
 368929: .................................G.....G....C.....  Mmulatta
 321535: ............................C..........G....C.....  Panubis
 358301: ..................T..................C...T.CC.....  Cjacchus
1009850: ..................T..................C...T.CA.....  Sboliviensis
```

```
                                    Simple
                  5118                                          5130
     5109: TACTCACAGAACA----------------------------CACACACAC  Hsapiens
   325009: ..........--------------------------------........  Ptroglodytes
   342862: .............CACACACA--------------------.........  Ggorilla
   374718: ..........----------------------------------------  Pabelli
   299464: ..T.......--------------------------------........  Nleucogenys
   368979: .............GACACACACA------------------....TG.G.  Mmulatta
   321585: .............GA--------------------------.........  Panubis
   358351: ..T.....A.------------------------------------...G  Cjacchus
  1009900: ..T.....A....GACAGACAGACAGACAGACAGACACACA.........  Sboliviensis

                       Simple
                  5140      5150      5160      5170      5180
     5131: ACACACACACACACACACACACACACACAAGCTACCCCAAGACCCTGGAA  Hsapiens
   325027: ............................T.....................  Ptroglodytes
   342892: ..........................C.......................  Ggorilla
   374728: ------------...........T..........................  Pabelli
   299482: ..................................................  Nleucogenys
   369011: ............................................A.....  Mmulatta
   321609: ...............................A..................  Panubis
   358365: ........CT.T.................CA....A...G..........  Cjacchus
  1009950: .............................C.....A...G..........  Sboliviensis

                  5190      5200      5210      5220      5230
     5181: ACAACTGGCAAAACCCAAAATACACAATAGAAATATGGGAGGAAAATAAA  Hsapiens
   325077: .........................G........................  Ptroglodytes
   342942: ..................................................  Ggorilla
   374766: .........................G........................  Pabelli
   299532: .....................G..GG.C......................  Nleucogenys
   369061: .............T..........GG.C..................C...  Mmulatta
   321659: .............T..........GG.C..................C...  Panubis
   358415: ....T..T.......T......T.GG.C......C...A.......C...  Cjacchus
  1010000: ....T..........T......T.GG.C......C...A.......C...  Sboliviensis

                  5240      5250                5269      5279
     5231: GTGCCAGGCCTTCCCACCCTAGATGATCC-AGATGCTTATTAATGCCCTC  Hsapiens
   325127: .............................-....................  Ptroglodytes
   342992: .............................-....................  Ggorilla
   374816: .............................-....................  Pabelli
   299582: .........T...................-................A...  Nleucogenys
   369111: ......C.........T......GATC.T-.....T..........G...  Mmulatta
   321709: ......C.........T......GATC.T-.....T..............  Panubis
   358465: ...G....T...T.-.T....A.......T.................T..  Cjacchus
  1010050: ...G........T.-.T....A.......T.................T..  Sboliviensis

                  5289      5299      5309      5318      5328
     5280: TAATCTCACAGAATCTTCTTCTCAAGCTCCTC-AAATAATGCCGAGCATG  Hsapiens
   325176: ................................-.................  Ptroglodytes
   343041: ................................-.................  Ggorilla
   374865: ................................-.................  Pabelli
   299631: .......................----------.................  Nleucogenys
   369160: ................................-....T.....A......  Mmulatta
   321758: .........................T......-....T.....A......  Panubis
   358514: ..............A.........T.......A...A....A.A..G...  Cjacchus
  1010099: ..............A.................-...A....A..G.....  Sboliviensis
```

```
                5338      5348      5358      5368      5378
                   :         :         :         :         :
   5329: AAGGTGGGAACCTCTACCTATCCTCCACCCTGATCTCTACCAGGATGGAG  Hsapiens
 325225: ..................................................  Ptroglodytes
 343090: ..............C...................................  Ggorilla
 374914: ..............C...................................  Pabelli
 299671: ..........T...C...................................  Nleucogenys
 369209: ....A..A......C...........................A.......  Mmulatta
 321807: ....A..A......C...........................A.......  Panubis
 358564: ..............C.TT.....C................TG........  Cjacchus
1010148: ..........GG.TC.TT.....C...T......................  Sboliviensis

                5388      5398                5411      5421
                   :         :                   :         :
   5379: TGGAGACCTGGGCCCACTCTGTTGGATA-------AGAGTGAATGGCTGT  Hsapiens
 325275: ............................-------...............  Ptroglodytes
 343140: .......T....................-------...........T...  Ggorilla
 374964: ............................-------...............  Pabelli
 299721: ............................-------...C...........  Nleucogenys
 369259: ....................A.......-------...............  Mmulatta
 321857: ............................-------...............  Panubis
 358614: ...........A.............C..TTGGGAA...........T...  Cjacchus
1010198: .A..A......A.............C..TTGGGTA...........T...  Sboliviensis

                5431      5441      5451      5461      5471
                   :         :         :         :         :
   5422: GTGGCTTTAAAAAACCTTTAATAAGAGACTGGTTTGGGCTTATTGATGCA  Hsapiens
 325318: ..................................................  Ptroglodytes
 343183: ..................................................  Ggorilla
 375007: ............G.....C...............................  Pabelli
 299764: ...........GG............................TC......C  Nleucogenys
 369302: T...........G.T..............CA.............AC....  Mmulatta
 321900: T...........G.T..............C.....C........AT....  Panubis
 358664: ............G...C..............G....-------------  Cjacchus
1010248: A...........G...................G...........AC....  Sboliviensis

                5481      5491      5501      5511      5521
                   :         :         :         :         :
   5472: TTGCTGTGTCCTCATTTGATCCCTCTGCTGAGTCCTGCACATGAGTCTGG  Hsapiens
 325368: ..............C...................................  Ptroglodytes
 343233: ..............C.............C..........T..........  Ggorilla
 375057: ..............C...................................  Pabelli
 299814: ..............C................A..................  Nleucogenys
 369352: ..............C...................................  Mmulatta
 321950: ..............C...................................  Panubis
 358701: --............C.G..C...................TG......C..  Cjacchus
1010298: ..............C.G............A.........TG.....T...  Sboliviensis

                5531      5541      5551      5561      5571
                   :         :         :         :         :
   5522: AACTAAACCACTCACAAACTGGTATTCCGTGTTCCATGGACAGAAAGACA  Hsapiens
 325418: ..................T...............................  Ptroglodytes
 343283: ..................................................  Ggorilla
 375107: ..................................................  Pabelli
 299864: ............................A.....................  Nleucogenys
 369402: ..................................................  Mmulatta
 322000: ..................................................  Panubis
 358749: .......................GC........TTC..............  Cjacchus
1010348: .........G.............GC..T.....TT...............  Sboliviensis
```

```
               5581      5591      5601      5606      5616
   5572: AGTTACTCTGTGATATCCAGGAAAATCCATTCCA-----GTTTGCCCAAA  Hsapiens
 325468: ..............G...................-----...........  Ptroglodytes
 343333: ..................................-----...........  Ggorilla
 375157: ....G.............................-----...........  Pabelli
 299914: ..................................-----...........  Nleucogenys
 369452: ..............G.G.................-----...........  Mmulatta
 322050: ..............G.G.................-----...........  Panubis
 358799: .........A.A..G.G.................--------------..  Cjacchus
1010398: .........A.A..G.G.................AACCA...........  Sboliviensis

               5626      5636      5646      5656      5666
   5617: CATTTTACACACCCACACAGTCTAATGATTTTCTGGTATCTGGTACATGG  Hsapiens
 325513: ..................................................  Ptroglodytes
 343378: ..................................................  Ggorilla
 375202: ..................................................  Pabelli
 299959: ..................................................  Nleucogenys
 369497: .G...C............................................  Mmulatta
 322095: TG...C............................................  Panubis
 358835: .....................................G............  Cjacchus
1010448: ..G.....................................CA....G...  Sboliviensis

               5676      5686      5696      5706      5715
   5667: TAGAGTTTACAATTTCACATTGAAACAGGTAAAAACATGTGGAAG-AAAC  Hsapiens
 325563: .................G...........................A....  Ptroglodytes
 343428: ...............G.G...........................A....  Ggorilla
 375252: ..A............G.G...........................A....  Pabelli
 300009: ...............G.G...........................A....  Nleucogenys
 369547: ...............G.G.................-.........A....  Mmulatta
 322145: ...............G.G...........................A....  Panubis
 358885: ........G.G....GTG...........................A....  Cjacchus
1010498: ........G......GTG...........................A....  Sboliviensis

               5725      5735      5745      5755      5765
   5716: ATTTCTGGCCTTGGCTTTGTGTCTGCTAAGGCATGTTCTTGTTAGTTAAT  Hsapiens
 325613: ....................A.............................  Ptroglodytes
 343478: ....................A.............................  Ggorilla
 375302: ....................A.............................  Pabelli
 300059: ....................A.............................  Nleucogenys
 369596: ............A.......A.......G....................C  Mmulatta
 322195: ....................A.......G....................C  Panubis
 358935: .....C..T.....A.....A............................C  Cjacchus
1010548: ..............A.....A...................A........C  Sboliviensis

               5775      5785      5795      5805      5814
   5766: ACCTGTGCACATTGAGGAAAAGCACCTACTTTTAAACCTCAAAA-GTTCA  Hsapiens
 325663: .......T...C................................-.....  Ptroglodytes
 343528: .......T....................................-.....  Ggorilla
 375352: .......T....................................-.....  Pabelli
 300109: .......T....................................-.....  Nleucogenys
 369646: .......T..G...........................A.....-.....  Mmulatta
 322245: .......T..G...........................A.....-.....  Panubis
 358985: .......T...C................................A...T.  Cjacchus
1010598: .......T...C................................A.....  Sboliviensis
```

```
                       Simple                 Simple
               5824      5834      5844      5854      5864
   5815: TGTGTAAACACTAAATATATTACATATATGAGTGTGTGTGTGTGTGTGTG  Hsapiens
 325712: ...............................------------.......  Ptroglodytes
 343577: ...............................--.................  Ggorilla
 375401: ...............................------------.......  Pabelli
 300158: .A.............................------------.......  Nleucogenys
 369695: ......................AG.......--------------.....  Mmulatta
 322294: ......................AG...GA.T------------.......  Panubis
 359035: ......................A.A......----------------...  Cjacchus
1010648: ......................A.A......----------------...  Sboliviensis

          Simple             Simple
               5874      5884      5893      5903      5913
   5865: TGTGTGTAAACCCTATATATA-ATATTTATATTATATTGTATTATGCTGT  Hsapiens
 325750: .....................-............................  Ptroglodytes
 343625: ............A........-............................  Ggorilla
 375439: ...............A.....-............................  Pabelli
 300196: .....................-............................  Nleucogenys
 369731: .....................T........................T...  Mmulatta
 322332: .....................-........................T...  Panubis
 359069: ...A............G....--------.....--------------..  Cjacchus
1010682: .....................--...........--------------..  Sboliviensis

                       Simple
                                   5929      5939      5949
   5914: AAGTGATG--------------TATATATATATTTATATATCAATGAACA  Hsapiens
 325799: ........----------TATA.G..........................  Ptroglodytes
 343674: ........------------TA.....G......................  Ggorilla
 375488: ........--------------...G........................  Pabelli
 300245: ........--------TATATA............................  Nleucogenys
 369781: ........TGTGTGTGTATATA............................  Mmulatta
 322381: ........----TGTGTGTGTA...............G............  Panubis
 359097: ..---...--------------...........C..C.............  Cjacchus
1010716: ..A.....----------------......C..C..C.............  Sboliviensis

               5959      5969      5979      5989      5999
   5950: TTGTTACTATTTGTTTCCACCGTTCTTGACTCACTGTAATGACCTTATAT  Hsapiens
 325839: ..................................................  Ptroglodytes
 343712: ..................................................  Ggorilla
 375524: ...................T..............................  Pabelli
 300287: ....................T.C...........................  Nleucogenys
 369831: .....................T.....A.......A...A..........  Mmulatta
 322427: ............C........T.....A.......A...A..........  Panubis
 359130: .....................T.............A..............  Cjacchus
1010750: ..A..............T...T.............A......T.......  Sboliviensis
```

```
                 6009      6019      6029      6039      6049
                    :         :         :         :         :
   6000: AAGAACTTTATACTTTTTATTCACAAAATGATTCATTTAATGTCAACAGT  Hsapiens
 325889: ........................G.........................  Ptroglodytes
 343762: ........................G.........................  Ggorilla
 375574: .......................TG...C.....T...............  Pabelli
 300337: ..................................................  Nleucogenys
 369881: ...................G...TG.....................T...  Mmulatta
 322477: ...................G...TG.....................T...  Panubis
 359180: .....T.......C.....................A.......A..T...  Cjacchus
1010800: .....T.......C.........TG..........A.......A..T...  Sboliviensis

                                 Alu
                   ---------------------------------------->
                 6059      6069      6079      6089      6099
                    :         :         :         :         :
   6050: TGAATAGAAGTTTTTGTTTTAGAGACAGGGTCTCACTTTGTTACCTAGGC  Hsapiens
 325939: ..................................G...............  Ptroglodytes
 343812: ..................................G...............  Ggorilla
 375624: ..................................................  Pabelli
 300387: .................................TG...............  Nleucogenys
 369931: .................................TG..C...C...C....  Mmulatta
 322527: .................................TG..C...C...C....  Panubis
 359230: ..................................TT.C...C.....A..  Cjacchus
1010850: .............................A....TT.C...C........  Sboliviensis

                            Alu
         ---------------------------------------------------->
                 6109      6119      6129                6148
                    :         :         :                   :
   6100: TGGGGCACAGTGGCATCATCACAGCTCATTGCTGCCTCA-AACTTCTGGG  Hsapiens
 325989: .......................................-..........  Ptroglodytes
 343862: .......................................-....C.....  Ggorilla
 375674: .....................T....T............-....C.....  Pabelli
 300437: .....TG.............GT.................-....C.....  Nleucogenys
 369981: ......G..............T.................-....C.....  Mmulatta
 322577: ......G..............T.......G.........-....C.....  Panubis
 359280: .....TG..............T.........T.......A....C.....  Cjacchus
1010900: .....TG..............T......C....A.....G....C.....  Sboliviensis

                            Alu
         ---------------------------------------------------->
                 6158      6168      6178      6188      6198
                    :         :         :         :         :
   6149: TTCAAGCAATTCTCACACCTCAGCCTTCCAAGCAGCTGGGACTACAAGTA  Hsapiens
 326038: ..................................................  Ptroglodytes
 343911: ..................................................  Ggorilla
 375723: ..................................................  Pabelli
 300486: .....A............................................  Nleucogenys
 370030: .....................G..........T.......G.........  Mmulatta
 322626: .....................G..........T.......G......A..  Panubis
 359330: ..........................C.....T.................  Cjacchus
1010950: ..........C...............C.....T.................  Sboliviensis
```

```
                                Alu
           ------------------------------------------------->
                    6208            6225     6234      6244
                       :                :        :         :
   6199: TACACCACCATATCTAGC---TATTTTTTTTAA-TTTTTTGTAGAGACAG  Hsapiens
 326088: ..................---.......C..T.-A...............  Ptroglodytes
 343961: ....-.............---..A.........-................  Ggorilla
 375773: ..................---..A.........--...............  Pabelli
 300536: ........T.....C...---..A......A..-................  Nleucogenys
 370080: ..................---........AA..-.........C......  Mmulatta
 322676: ..................---........AA..-.........C......  Panubis
 359380: ..G....T..C.......TTT.T..........-..C.............  Cjacchus
1011000: .......T..C.......---........AA..A..C..C..........  Sboliviensis

                                Alu
           ------------------------------------------------->
                    6254      6264      6274      6284      6294
                       :         :         :         :         :
   6245: GGTCTTGCTATGTTGTCCAGGCTTGTCTCCAATTCCCGGGCTCAAGCAAT  Hsapiens
 326134: .......................G..........................  Ptroglodytes
 344006: ...................A...G..........................  Ggorilla
 375818: .......................G............T..A...T......  Pabelli
 300582: A......................G............T.........T...  Nleucogenys
 370126: ...............C.......G............T.A...........  Mmulatta
 322722: ...............C.......G............T.A...........  Panubis
 359429: ...............C.......G..G.........T.............  Cjacchus
1011047: ......A........C.......G..G.........T.............  Sboliviensis

                                Alu
           ------------------------------------------------->
                    6304      6314      6324      6334      6344
                       :         :         :         :         :
   6295: CCTCCCACCTCAGTCTCCCAAAGTGCTGTGGTTACAGGACTGAGTCACTG  Hsapiens
 326184: .......................................A..........  Ptroglodytes
 344056: ............A..........................A..........  Ggorilla
 375868: .......................................A..........  Pabelli
 300632: .......................................A..........  Nleucogenys
 370176: .............C........................CG....C.....  Mmulatta
 322772: .....TG......C........................CA....C.....  Panubis
 359479: ............AC.......G...T............TG....C....A  Cjacchus
1011097: .........................T...C........CA....C..T.T  Sboliviensis

             Alu
           -------->
                    6354      6364      6374      6384      6394
                       :         :         :         :         :
   6345: AACCTGGCCTAAATAGGGACTTAAAATATTCTTCAAATTGCAGATCTGTT  Hsapiens
 326234: ..................................................  Ptroglodytes
 344106: ..................................................  Ggorilla
 375918: ..........................C.......................  Pabelli
 300682: ..........................C........G..............  Nleucogenys
 370226: ............G.............CT......................  Mmulatta
 322822: ..........................CT......................  Panubis
 359529: ..G.......................C...............T.......  Cjacchus
1011147: .G-.......................G...............T.......  Sboliviensis
```

```
               6404      6414      6424      6434      6444
                  :         :         :         :         :
   6395: AAGCATAGCTTGCCTCACTAATTCACCTTTTAAAATGATACTTTTTCAAG  Hsapiens
 326284: ..............................................T...  Ptroglodytes
 344156: ..................................................  Ggorilla
 375968: ..........................A.......................  Pabelli
 300732: .........................T.........C..............  Nleucogenys
 370276: .............T....A......T.....T..................  Mmulatta
 322872: .............T....A......T.....T..................  Panubis
 359579: .......A............................A.............  Cjacchus
1011196: .........C........G.................A.............  Sboliviensis

               6454      6464      6474      6484      6494
                  :         :         :         :         :
   6445: CGAATTTTGCTCAGCTAAACCTCAAACTTCAGTGTCTGCTACTGAAACAA  Hsapiens
 326334: ..................................................  Ptroglodytes
 344206: ..................................................  Ggorilla
 376018: ..................................................  Pabelli
 300782: ....................T.............................  Nleucogenys
 370326: ......................................G...........  Mmulatta
 322922: .....................C................G...........  Panubis
 359629: A...C...................................G......T..  Cjacchus
1011246: A...C....................T..............G.........  Sboliviensis

               6504      6514      6524      6534      6544
                  :         :         :         :         :
   6495: ACTAGTTATGAAAAAAGAAGGCCAGTAGAAAGAAACTAAGCTCAGTTTCA  Hsapiens
 326384: G.......CA........................................  Ptroglodytes
 344256: G.......C.........................................  Ggorilla
 376068: G.......C.............T.A...................T.....  Pabelli
 300832: G.......C...................................T.....  Nleucogenys
 370376: G............C.......T..............G.....-.T.C...  Mmulatta
 322972: G............C.......T..............G.....-.T.C...  Panubis
 359679: G..G.........G..A....T......................T.C.T.  Cjacchus
1011296: G............C..A..-.T......................T.C...  Sboliviensis

               6554      6564      6574      6584      6594
                  :         :         :         :         :
   6545: TGTTTGTCTTTAATTTTTGTCCCTATTCATGCCTCCTGCAATCCAAAAAT  Hsapiens
 326434: ..................................................  Ptroglodytes
 344306: ...C..............................................  Ggorilla
 376118: ..................................................  Pabelli
 300882: ..................................................  Nleucogenys
 370425: .T..A......G.C............C.......................  Mmulatta
 323021: .T..A......G.C....................................  Panubis
 359729: .T......................G.....A.................C.  Cjacchus
1011345: .T.........-............GC....A.................C.  Sboliviensis

               6604      6613      6621      6631      6641
                  :         :         :         :         :
   6595: TGCTCTTGACTTCTCA-TAAAG--AAAAACTGAAAACAAGGAGGACACGA  Hsapiens
 326484: ................-.....--....G.....................  Ptroglodytes
 344356: ................-.....--G......................T..  Ggorilla
 376168: ................-.....--.....T.................T..  Pabelli
 300932: ................-.....--.....T.................T..  Nleucogenys
 370475: ................-.....--.....T.............C...T..  Mmulatta
 323071: ................-.....--.....T.............C...T..  Panubis
 359779: .......A........T....A--...C.T....---..........T..  Cjacchus
1011394: .......A........-....AAC.....T....---..........T..  Sboliviensis
```

```
                6651      6661      6671      6681
    6642: AATGAAAAGAATCCATGAAAAATGTATTTTTCATCCATTTCAGAAGAAA-  Hsapiens
  326531: .........G.......................................-  Ptroglodytes
  344403: .................................................-  Ggorilla
  376215: ............A..........TG........................-  Pabelli
  300979: ........................G..........T.............-  Nleucogenys
  370522: .......T.........................................-  Mmulatta
  323118: .......T.........................................-  Panubis
  359824: ...........G.AGCA....--.G.......                    Cjacchus
  360335:                                 .................-  Cjacchus
 1011440: ...........G.AGCC...---.G.......                    Sboliviensis
 1011954:                                 .........G...A...A  Sboliviensis

                6700      6705      6715      6725      6735
    6691: TAGAAGAAAAA-----GAAAGTAAAGGCCTCTAACTATAGCCTGAAACAC  Hsapiens
  326580: ...........-----A.................................  Ptroglodytes
  344452: ...........-----A.................................  Ggorilla
  376264: ...........-----A.................................  Pabelli
  301028: ...........------.........A.......................  Nleucogenys
  370571: ...........AAATAA.................................  Mmulatta
  323167: ......G....--AAAA.................................  Panubis
  360352: .........G.------....C....A..........C..TG.A......  Cjacchus
 1011972: .........G.------....................C..TG.A......  Sboliviensis

                                              Alu
                                   ------------------------>
                6745      6755                6770      6780
    6736: CACCTCAACTTAAGACCTCAGTTTAG-----GCCGGGCGCGGTGGCTCAC  Hsapiens
  326625: .........................AACAAT..T....A.            Ptroglodytes
  344497: ....................A....AACAAT..T....A.            Ggorilla
  376309: ....................A....AAC                        Pabelli
  301072: ................T........AACAAT..T....A.            Nleucogenys
  370621: ...............T.........AACATT..T....A.            Mmulatta
  323215: ...................C.....AACATT..T....A.            Panubis
  360396: ........G.......TG.......AACATT..T....A.            Cjacchus
 1012016: ...T............TA.......AACATT..T....A.            Sboliviensis

                                 Alu
          ------------------------------------------------->
                6790      6800      6810      6820      6830
    6781: GCCTGTAATCCCAGCACTTTGGGAGGCCGAGGCGGGTGGATCATGAGGTC  Hsapiens

                                 Alu
          ------------------------------------------------->
                6840      6850      6860      6870      6880
    6831: AGGAGATCGAGACCATCCTGGCTAACAAGGTGAAACCCCGTCTCTACTAA  Hsapiens

                                 Alu
          ------------------------------------------------->
                6890      6900      6910      6920      6930
    6881: AAAAAAAATACAAAAAATTAGCCGGGCGCGGTGGCGGGCGCCTGTAGTCC  Hsapiens

                                 Alu
          ------------------------------------------------->
                6940      6950      6960      6970      6980
    6931: CAGCTACTCGGGAGGCTGAGGCAGGAGAATGGCGTGAACCCGGGAAGCGG  Hsapiens
```

```
                                      Alu
         ------------------------------------------------------------------>
                     6990      7000      7010      7020      7030
   6981: AGCTTGCAGTGAGCCGAGATTGCGCCACTGCAGTCCGCAGTCCGGCCTGG  Hsapiens

                                      Alu
         ------------------------------------------------------------------>
                     7040      7050      7059      7069      7074
   7031: GCGACAGAGCGAGACTCCGTCTCAA-AAAAAAAAAAAAAAAA-----AAA  Hsapiens
 326564:                ...A.T...G-..G...T.G..G...------...  Ptroglodytes
 344436:                ...A.T...G-..G...T.G..G...------...  Ggorilla
 376248:                ...A.T...G-..G...T.G..G...------...  Pabelli
 301012:                ..TA.T...G-..G...T.G..G..-------...  Nleucogenys
 370555:                ...A.T...G-..G...T.G..G....AAAAT...  Mmulatta
 323151:                ...A.T...G-..G...T.G..GG...AAA--...  Panubis
 360336:                ...A.T...G-..G...T.G..G..-------G..  Cjacchus
1011951:            .A.A...A.T..GGA......T.G..G..-------G..  Sboliviensis

                                      Alu
         -------------------------------------------------------->
                                                             7088
   7075: AAAAAAA------------------------------------AAGACCT  Hsapiens
 326593: ..GT...GGCCTCTAACTATAGCCTGAAACACCACCTCAACTT.......  Ptroglodytes
 344465: ..GT...GGCCTCTAACTATAGCCTGAAACACCACCTCAACTT.......  Ggorilla
 376277: ..GT...GGCCTCTAACTATAGCCTGAAACACCACCTCAACTT.......  Pabelli
 301040: ..GT...GACCTCTAACTATAGCCTGAAACACCACCTCAACTT.....T.  Nleucogenys
 370589: ..GT...GGCCTCTAACTATAGCCTGAAACACCACCTCAACTT....T..  Mmulatta
 323183: ..GT...GGCCTCTAACTATAGCCTGAAACACCACCTCAACTT.......  Panubis
 360364: ..GC...GACCTCTAACTACAGTGTAAAACACCACCTCAAGTT.....TG  Cjacchus
1011984: ..GT...GGCCTCTAACTACAGTGTAAAACACCACTTCAACTT.....TA  Sboliviensis

                     7098      7108      7118      7128      7138
   7089: CAGTTTAAACAATGCTGGGCACTAGAAAATTGTTAGGACATTTTCCCTGC  Hsapiens
 326643: ..................................................  Ptroglodytes
 344515: ..A.........................T.....................  Ggorilla
 376327: ..A.............A......G..........................  Pabelli
 301090: ..................................................  Nleucogenys
 370639: ...........T......................................  Mmulatta
 323233: .C.........T......................................  Panubis
 360414: ...........T...................A......TT..C.......  Cjacchus
1012034: ...........T...................A......TT..C.......  Sboliviensis

                     7148      7158      7168      7178      7188
   7139: CTGTCCCTTCAAAGTCCGTGATCTAGAAATAAATTTGAAAATCAGACTAC  Hsapiens
 326693: ..................................................  Ptroglodytes
 344565: .................A................................  Ggorilla
 376377: T.................................................  Pabelli
 301140: ....T.............................................  Nleucogenys
 370689: ......T.C.............A........C..............G...  Mmulatta
 323283: ......T.C......................C..............G...  Panubis
 360464: .....T..C....T...A........................T.......  Cjacchus
1012084: ........C....T...A..C.............................  Sboliviensis
```

```
               7198      7208      7218      7228      7237
                  :         :         :         :         :
   7189: AGTGTGAAAACAGTGATGGTGGAAGTATTTCTAAAGTGCTT-GAAGAAGT   Hsapiens
 326743: .........................................-........   Ptroglodytes
 344615: ............................C............-........   Ggorilla
 376427: ..............................G..........-.....G..   Pabelli
 301190: G.............................G..........-.....GA.   Nleucogenys
 370739: .A...AGG......................G..........T.CG..G..   Mmulatta
 323333: .A...A.G......................G..........T.CG..G..   Panubis
 360514: .........G...A.......A..T.....G..........T..G..G..   Cjacchus
1012134: .A.......G..............T.....G..........T..G..G..   Sboliviensis

               7241      7251      7261      7271      7281
                  :         :         :         :         :
   7238: G------CTTGAGAAAGCTTCGGAGTGGAAATGATGCTGAAACTCCATCT   Hsapiens
 326792: .GAGGAGT.AC.........T.............................   Ptroglodytes
 344664: .GAGGAGT.AC.................................T.....   Ggorilla
 376476: .GAGGAGT.AC..........A......G.....................   Pabelli
 301239: .GAGGAGA.ACG..T...................................   Nleucogenys
 370789: .GAAGAGT.AC....G....-...A.........................   Mmulatta
 323383: .GAAGAGT.AC....G....-...A.........................   Panubis
 360564: .GAGGAGTCAC....G....T...A.........................   Cjacchus
1012184: .GAGGAGT.AC....G....T...A..A......................   Sboliviensis

               7291      7301      7311      7321      7331
                  :         :         :         :         :
   7282: GGCAGAGGGAAGGAGAGAGACTAGCAAGGTCTGCTGGGTGGAGGGTGGGG   Hsapiens
 326842: ..................................................   Ptroglodytes
 344714: ..................................................   Ggorilla
 376526: .............................................CA...   Pabelli
 301289: ........................GG...................C....   Nleucogenys
 370838: ........-.......C........G.....CA......A..........   Mmulatta
 323432: ........-.......C........G.....C.......A.....C....   Panubis
 360614: .........G...G.....G....TGG......A...........C....   Cjacchus
1012234: .........G...G.....G....TG.....C.A.-.........C....   Sboliviensis

               7341      7351      7361      7371      7381
                  :         :         :         :         :
   7332: CTCCAAGGGAGGGACTGATACCTGCGTGTCCTGCCCACCCTCCTAACCTG   Hsapiens
 326892: ..................................................   Ptroglodytes
 344764: ..................................................   Ggorilla
 376576: ...........A............AC...........G.........T..   Pabelli
 301339: .....................................G.........T..   Nleucogenys
 370887: ....................................TG...CT....T..   Mmulatta
 323481: ....................................TG...CT....T..   Panubis
 360664: .....G.....A......C.....T....TG....-.....T.....T..   Cjacchus
1012283: .....G.....A......C.....T....T.....-...........T..   Sboliviensis

               7391      7401      7411      7421      7431
                  :         :         :         :         :
   7382: AGTCTCTCTCGGGACCACCCTAATGACTAGTGTTACCTCTGACCAGCGTC   Hsapiens
 326942: ..................................................   Ptroglodytes
 344814: ..................................................   Ggorilla
 376626: ..................................................   Pabelli
 301389: ..............................................T...   Nleucogenys
 370937: ...................G........C.C...................   Mmulatta
 323531: ...................G........C.C...................   Panubis
 360713: .....A....A.......G........C.....C...C....T....A..   Cjacchus
1012332: .....A....A........T.......C.....C...C.........A..   Sboliviensis
```

```
                  7441      7451      7461      7471      7481
                     :         :         :         :         :
    7432: TTTGCTCCTCTAGGGACCACCCTTAGACGACAGGGCTCGTACCTACTAAA   Hsapiens
  326992: ................A.................................   Ptroglodytes
  344864: ................A.................................   Ggorilla
  376676: ................A..T........T.....................   Pabelli
  301439: ..........C.....A..T........T.....................   Nleucogenys
  370987: ................A..T........T.........A...........   Mmulatta
  323581: ................A..T........T.T.......A...........   Panubis
  360763: ......T..A......ATGTT..C....T.........A...........   Cjacchus
 1012382: ......T..A......A..TT..C....T...............G.....   Sboliviensis

                  7491      7501      7511      7521      7531
                     :         :         :         :         :
    7482: TGCCTCTTATGATTATCTGTTTACCGGTGTTCTCCTCAGCATTACCTATG   Hsapiens
  327042: ..................C...G.................G.........   Ptroglodytes
  344914: ..................C..........................T....   Ggorilla
  376726: C................GC.............................CA   Pabelli
  301489: C.................C...............................   Nleucogenys
  371037: ..................C.....T.A.......................   Mmulatta
  323631: .................GC.....T.A.......................   Panubis
  360813: ............-----AC......A..................T.....   Cjacchus
 1012432: ..................C......A........T...............   Sboliviensis

                  7541      7551      7561      7571      7581
                     :         :         :         :         :
    7532: GCAACGAGACCAAGTTCAGACAGGAAAACATAATCTGATTAGTTCTCCAA   Hsapiens
  327092: ..................................................   Ptroglodytes
  344964: ..................................................   Ggorilla
  376776: ..................................................   Pabelli
  301539: .............................--...................   Nleucogenys
  371087: .....A..............A.............................   Mmulatta
  323681: .....A..............A.........................T...   Panubis
  360858: ....T.............................................   Cjacchus
 1012482: ....T..........G.............G...................G   Sboliviensis

                                                7588      7598
                                                   :         :
    7582: GAAT---------------------------------GAAGAAGACAATC   Hsapiens
  327142: ....---------------------------------.............   Ptroglodytes
  345014: ....---------------------------------.............   Ggorilla
  376826: ....---------------------------------.............   Pabelli
  301587: ...C------------------------------------..........   Nleucogenys
  371137: ...C---------------------------------A............   Mmulatta
  323731: ...C---------------------------------A............   Panubis
  360908: ...CAAAGAAGACAAATCTGATTAGTTCTCCAAGAACA............   Cjacchus
 1012532: ...C---------------------------------A............   Sboliviensis

                  7608      7618      7628      7638      7648
                     :         :         :         :         :
    7599: TGTTTTCATTTTGTAACAAACCCGAACCCAGCCTTCATTCATTCAACATA   Hsapiens
  327159: ..................................................   Ptroglodytes
  345031: .......................A..........................   Ggorilla
  376843: ..................................................   Pabelli
  301601: .........-...............................G....TG..   Nleucogenys
  371154: ...................................T..............   Mmulatta
  323748: .....................T.............T..........T...   Panubis
  360958: .C....T..C...........-.A.G........................   Cjacchus
 1012549: .C....T..C.............A..........................   Sboliviensis
```

```
                7657      7667      7677      7687      7697
                   :         :         :         :         :
   7649: AATG-AAACTACATATTCCCTTGGCGTGAAATTTATTTCTTCCTAATTCT  Hsapiens
 327209: ....-.............................................  Ptroglodytes
 345081: ....-.............................................  Ggorilla
 376893: ....-......................................C...A..  Pabelli
 301650: ....-.............................................  Nleucogenys
 371204: .T.ATTG.A...........C...A.........................  Mmulatta
 323798: ....-...............C...A.........................  Panubis
 361007: ....-...................T.......G....C............  Cjacchus
1012599: ....-...........................A....C............  Sboliviensis

                7707      7717      7727      7737      7747
                   :         :         :         :         :
   7698: ATCCTCTATCAATCTCTAAACCACATTTCAGACAAATGGTAGACTTCATA  Hsapiens
 327258: ................................................C.  Ptroglodytes
 345130: ..................................................  Ggorilla
 376942: .C............................................T...  Pabelli
 301699: ..............................................T...  Nleucogenys
 371254: ..............................................T...  Mmulatta
 323847: ............C.................................T...  Panubis
 361056: ..............C.................T.....AC......T...  Cjacchus
1012648: ................................T.............T...  Sboliviensis

                7757      7765      7775      7785      7795
                   :         :         :         :         :
   7748: ACTCTTTAAAAAA--TCTTCCAGAATAGATTTATTTAAACTCTGTCAAGA  Hsapiens
 327308: .............--...........................C.......  Ptroglodytes
 345180: .............--...................................  Ggorilla
 376992: .............--...............A...................  Pabelli
 301749: .............--...........C............T..........  Nleucogenys
 371304: ....C........-A........C..........................  Mmulatta
 323897: ....C........-A...................................  Panubis
 361106: ...........GT-T.T.......................C.........  Cjacchus
1012698: ...........GTTT.T.......................C.........  Sboliviensis

                7805      7815      7825
                   :         :         :
   7796: AATCAATGCGATTTTATTTATTAACATAAGTAAA----------------  Hsapiens
 327356: ..................................----------------  Ptroglodytes
 345228: ..................................----------------  Ggorilla
 377040: ........T.........................----------------  Pabelli
 301797: .........................G...A....----------------  Nleucogenys
 371353: ........................TG........----------------  Mmulatta
 323946: .........A..............TG........----------------  Panubis
 361155: .....C...T........................TAAATAATGTAAGTAA  Cjacchus
1012748: ...A.....T....C...................TAAATAATGTAAGTAA  Sboliviensis

                7838      7848      7858      7868      7878
                   :         :         :         :         :
   7830: -CTACTATGCTTATATATTAGCATTAAAAACCCTGCCAAAGACTTTGGAT  Hsapiens
 327390: -.................................................  Ptroglodytes
 345262: -...........................................G.....  Ggorilla
 377074: -.----------------...........................C....  Pabelli
 301831: -.................................................  Nleucogenys
 371387: -..............A..........................G.......  Mmulatta
 323980: -........T.....A..........................G.......  Panubis
 361205: A..T..............................................  Cjacchus
1012798: A..T..............................................  Sboliviensis
```

```
                   7888      7898      7908      7918      7928
                      :         :         :         :         :
   7879: GAAAATCTTCCAGATACAACTATGATTAAATACAAGATGTTCTGCTGTTA  Hsapiens
 327439: ................T.......................C.........  Ptroglodytes
 345311: ........................................C.........  Ggorilla
 377107: ........................................C......-..  Pabelli
 301880: ........................................C.........  Nleucogenys
 371436: ........................................C.........  Mmulatta
 324029: ...................A....................C.........  Panubis
 361255: C.........T.......G............G........C...T.....  Cjacchus
1012848: C.........T....................G........C..TT.....  Sboliviensis

                   7938      7948      7958      7968      7978
                      :         :         :         :         :
   7929: CCATTTCTGTTCCATCAGCCACAGGTTTTAGAATCTTTTCCCTTCCCATG  Hsapiens
 327489: ...................................C..............  Ptroglodytes
 345361: ...................................C..............  Ggorilla
 377156: ..............C.........A..........C..............  Pabelli
 301930: .G...C..................A..........C......C.......  Nleucogenys
 371486: ..............A.........A......G...C..............  Mmulatta
 324079: ..............A.........A......G...C..............  Panubis
 361305: ..............A........TA.G....C...C..............  Cjacchus
1012898: ...........TA.A.........A.G....T...C..............  Sboliviensis

                   7988      7998      8008      8018      8028
                      :         :         :         :         :
   7979: AGCAGCCCACACTCTAGTTTGTTTATATTGAGGGCAAAAATATTAGCAGA  Hsapiens
 327539: ..................................................  Ptroglodytes
 345411: ..................................................  Ggorilla
 377206: ......................................C..........C  Pabelli
 301980: ..................................................  Nleucogenys
 371536: ...........T..........C...........................  Mmulatta
 324129: ...........T..........C...........................  Panubis
 361355: ....A......T.T..........T.......A.......C.........  Cjacchus
1012948: ....A......T.T..........T...............C.........  Sboliviensis

                   8038      8048      8058      8068      8078
                      :         :         :         :         :
   8029: GATGTCAAGGTTGTAAGTCATTGTAGAAGACAGTTATAAGATGGAGTTAT  Hsapiens
 327589: ................................A.................  Ptroglodytes
 345461: ................................A.................  Ggorilla
 377256: ............A.....................................  Pabelli
 302030: ................................A.............A...  Nleucogenys
 371586: ...................................G..........A...  Mmulatta
 324179: .................C...........G.....G..........A...  Panubis
 361405: ........................G......CA.............A...  Cjacchus
1012998: ................T.............TGA.G..G...CA...A...  Sboliviensis

                   8088      8098      8108      8118      8128
                      :         :         :         :         :
   8079: TCTAAAGCTCAGCTATACATAGTTTGGGATTTGTTGGAGAACCTCTGATT  Hsapiens
 327639: ..................................................  Ptroglodytes
 345511: .........A........................................  Ggorilla
 377306: ..................................................  Pabelli
 302080: .......A.....................................C.G..  Nleucogenys
 371636: .............CG.....G...........T...........A.....  Mmulatta
 324229: ..............G.....G.......................A.....  Panubis
 361455: G.....C......A..............................--....  Cjacchus
1013048: G.....C......A..............................------  Sboliviensis
```

```
                8138      8148      8158      8168      8177
   8129: CTGAATCATTCTATCAAGTACTCAAAAGTGCCTCATATTACTAAAAAA-T  Hsapiens
 327689: ................................................-.  Ptroglodytes
 345561: ..............................T.................T.  Ggorilla
 377356: ................................................A.  Pabelli
 302130: ................................................AA  Nleucogenys
 371686: G....C......C.........-........................--.  Mmulatta
 324279: A...........C.........-........................--.  Panubis
 361503: ......A........G......................CG.......--A  Cjacchus
1013092: ----------------------------------------.C......-A  Sboliviensis

                8187      8197      8207      8217      8227
   8178: TTTTTTGAAACCTCAAACCTCTCAAAATTGTTGCTAAAGTTGCCAAATAT  Hsapiens
 327738: ..................................................  Ptroglodytes
 345611: ..................................................  Ggorilla
 377406: ..................................................  Pabelli
 302180: .........G........................................  Nleucogenys
 371733: ...........................A.......C.....A........  Mmulatta
 324326: ...........................A.......C.....A........  Panubis
 361551: A..AC...........C..................G..............  Cjacchus
1013101: A..GC......T......................C...............  Sboliviensis

                          8236      8246      8256      8266
   8228: CATTGAT-----------TATAATCAACAAGGAATGTCCAACACATAGAA  Hsapiens
 327788: .....C.AAAAA------..............................C.  Ptroglodytes
 345661: .......AAAAA------..............................C.  Ggorilla
 377456: .......AAAAA------..............................C.  Pabelli
 302230: .......AAAAA------............................C.C.  Nleucogenys
 371783: .......AAAAATAAAAA......TG.......G.......T......C.  Mmulatta
 324376: .......AAAAATAAAAA......TG.......G.......T......C.  Panubis
 361601: TG.....AACATTTAAAA.........T...T.....T...T......C.  Cjacchus
1013151: TG.....AAAATTTAAAA.........T...T.....T...T.A...AC.  Sboliviensis

                8276      8286      8296      8306      8316
   8267: GACCTTTTAGGGAATTATCATCATGGTTGTATTGTGATCGACCTGGGAGT  Hsapiens
 327832: ..................................................  Ptroglodytes
 345705: ..................................................  Ggorilla
 377500: ......................................G...........  Pabelli
 302274: ................C.................................  Nleucogenys
 371833: .....C.................................A..........  Mmulatta
 324426: .....C.......G....................................  Panubis
 361651: .........................A...............T...A....  Cjacchus
1013201: ..................................................  Sboliviensis

                8326      8336      8346      8356
   8317: TGGCATGTAACTTGTCACCTGGGTTCTGGATCATTTGCTTAACAAAAAA-  Hsapiens
 327882: .................................................-  Ptroglodytes
 345755: .........G.....T.................................-  Ggorilla
 377550: ..........................................T......-  Pabelli
 302324: ..........................................T......-  Nleucogenys
 371883: .........................G..A.............T......-  Mmulatta
 324476: .........................G..A.............T......-  Panubis
 361701: ............A............T....C...........A......A  Cjacchus
1013251: .........................T....C...........A...G..A  Sboliviensis
```

```
                 8374      8384      8394      8404     8410
                    :         :         :         :        :
   8366: -GTAAAACTAGCTTGTAAATATCAATTCATTCACTCACATA----TATTT  Hsapiens
 327931: -........................................----.....  Ptroglodytes
 345804: -........................................----.....  Ggorilla
 377599: -T............A..........................----.....  Pabelli
 302373: -........G....A......C...................----.....  Nleucogenys
 371932: -.............A..................T.......----.....  Mmulatta
 324525: -.............A..................T.......----.....  Panubis
 361751: A...........C...........................GTATT.....  Cjacchus
1013301: A...........C...........................G----.....  Sboliviensis

                 8420      8430      8440      8450      8460
                    :         :         :         :         :
   8411: AATCAGTAGGTCTGTGACACCAGAGATAGAAAGCCCAGACACAGGTAGTA  Hsapiens
 327976: ..................................................  Ptroglodytes
 345849: ...............................................T..  Ggorilla
 377644: ..................................T.......G.......  Pabelli
 302418: ..........C...................................C...  Nleucogenys
 371977: ..................................................  Mmulatta
 324570: ..................................................  Panubis
 361801: .........T.......................G.....T..........  Cjacchus
1013347: .T.......T.......................G................  Sboliviensis

                 8470      8480      8490      8500      8510
                    :         :         :         :         :
   8461: GATATAGTTGGTGTATGAGCTATCTTGTATAAGCTTAGGGGTCTATTCAG  Hsapiens
 328026: ..........................T.......................  Ptroglodytes
 345899: ..........................T.......................  Ggorilla
 377694: ..............GC..........T.......................  Pabelli
 302468: ....C.........G...........T.......................  Nleucogenys
 372027: ..............G...........T.......................  Mmulatta
 324620: ..............G...........T.......................  Panubis
 361851: ..............G...AT......T..........A............  Cjacchus
1013397: ........C.....G...AT......T..........A....T.....C.  Sboliviensis

                 8520      8530      8538      8548      8558
                    :         :         :         :         :
   8511: CCGTCAGTAGGCACTTTTTTTT--CATAAGGTAAGGAATAAAGCTGAATT  Hsapiens
 328076: .A...G.......T........TT.G........................  Ptroglodytes
 345949: .A...G.......T........-T......A...................  Ggorilla
 377744: .A...G.......T........--..........................  Pabelli
 302518: .A...G.......T......---T...........A..............  Nleucogenys
 372077: .A...G.......T..C..C..-T........................C.  Mmulatta
 324670: .A...G.......T..C..C..-T........................C.  Panubis
 361901: .A...GT.............----..........................  Cjacchus
1013447: .A..TGT.............----..............G...........  Sboliviensis

                 8568      8578      8588      8598      8608
                    :         :         :         :         :
   8559: TTCTCTATTAAATATGTTGGCCAATGTTATAGATGCGGCCGTCAATTGTC  Hsapiens
 328126: ..................................................  Ptroglodytes
 345998: ..................................................  Ggorilla
 377792: ...................................AT.............  Pabelli
 302565: .C......................C...........T.............  Nleucogenys
 372126: ............................T.......T.......T.....  Mmulatta
 324719: ............................T.......T......GT.....  Panubis
 361947: .C...AG...G..................C......T...A.........  Cjacchus
1013493: .....A..G.G..................C...C..T...AC........  Sboliviensis
```

```
                   8618      8628      8638      8648      8658
                      :         :         :         :         :
    8609: ATTCTAATCCCCAAGATTAGTCTACAACAACCATGTGGAGAACAGAGATT  Hsapiens
  328176: ..................................................  Ptroglodytes
  346048: ..................................................  Ggorilla
  377842: .....................G........T..........G........  Pabelli
  302615: ....C.........T...................A...............  Nleucogenys
  372176: ..................................A...............  Mmulatta
  324769: ..................................................  Panubis
  361997: ...................A..C..........................C  Cjacchus
 1013543: ...................A..C............A..............  Sboliviensis

                   8668      8678      8688      8698      8708
                      :         :         :         :         :
    8659: GGCTGTGGTTACCCTGGAAAGGGGTATGTGTCTGCCAGAATCCCAGATGC  Hsapiens
  328226: ..................................................  Ptroglodytes
  346098: ..................................................  Ggorilla
  377892: ..............C...................................  Pabelli
  302665: ........................................C.........  Nleucogenys
  372226: .A...............................T................  Mmulatta
  324819: .A...............................T................  Panubis
  362047: A...A......T.........A...G....................G.T.  Cjacchus
 1013593: A...A..T...T.............G........................  Sboliviensis

                   8718      8728      8738      8748      8758
                      :         :         :         :         :
    8709: CAGAGGTAAAGCTGTTACTTTGACAGCAGGTAAATGCTCAGGGCATGCAG  Hsapiens
  328276: ..................................................  Ptroglodytes
  346148: ..................................................  Ggorilla
  377942: ..................................................  Pabelli
  302715: ..................................................  Nleucogenys
  372276: T...............GT.............G..................  Mmulatta
  324869: T.........-.....C..............G..................  Panubis
  362097: T..........T...........T.C.....G....T..G..........  Cjacchus
 1013643: T.........A............T.C.....G....T......T......  Sboliviensis

                   8768      8778      8788      8798      8808
                      :         :         :         :         :
    8759: CACCTCAGGCACTAGGGGCTGTCCATCCATCTTAGGGAATCTAAGCATAC  Hsapiens
  328326: .......A..........................................  Ptroglodytes
  346198: ...............................................C..  Ggorilla
  377992: ..................................C...............  Pabelli
  302765: ..................................................  Nleucogenys
  372326: ...............................C..........G.......  Mmulatta
  324918: ...............................C.G........G.......  Panubis
  362147: ...............................................C..  Cjacchus
 1013693: .............................................TCC..  Sboliviensis

                   8818      8828                 8839      8849
                      :         :                    :         :
    8809: TCAGAAGCCTAGAATAACCCAGCATAAG---------CACTCACCTCCTC  Hsapiens
  328376: ............................---------.............  Ptroglodytes
  346248: ...........................A---------.............  Ggorilla
  378042: ............................---------.............  Pabelli
  302815: ............................TAAGAATGC.............  Nleucogenys
  372376: ......................T.....TAAGAACGC.............  Mmulatta
  324968: ......................T.....TAAGAACGC.............  Panubis
  362197: .AG.......................G..TAAGAATGC.............  Cjacchus
 1013743: .AG.......................G..TAAGAATGC.............  Sboliviensis
```

```
                8859      8868      8878      8888      8898
                   :         :         :         :         :
    8850: TCACTGCAGT-CTGCGGCCTTCACAGCTGGGCTTCAGTTCCTCCTGCCTC  Hsapiens
  328417: ..........-...............................T.......  Ptroglodytes
  346289: ..........-.......................................  Ggorilla
  378083: ..........-...T...................................  Pabelli
  302865: ..........-...T...................................  Nleucogenys
  372426: ..........-....A...............................T..  Mmulatta
  325018: ..........-....A...............................T..  Panubis
  362247: ..........T.....A......................T...T......  Cjacchus
 1013793: ..........T.....A......................T...T......  Sboliviensis

                8908      8918      8928      8938      8948
                   :         :         :         :         :
    8899: CTACCTCCTAAGACTTCTCACTCCTCCCAGCACACCTATACCAAGGTTCA  Hsapiens
  328466: ..................................................  Ptroglodytes
  346338: .....................A............................  Ggorilla
  378132: ....G.............................................  Pabelli
  302914: ...................T..............................  Nleucogenys
  372475: .....................................C............  Mmulatta
  325067: .....................................C............  Panubis
  362297: ..C....--.....................TG..T..C.......A....  Cjacchus
 1013843: ..C...........................TG..T..C.......A....  Sboliviensis

                8958      8968      8978      8988      8998
                   :         :         :         :         :
    8949: GGGAAAATTCCCAAGACTTCCCTTTGGCTCAACTCCCAAGGGAGAGGTTG  Hsapiens
  328516: ...............................G..................  Ptroglodytes
  346388: ..................................................  Ggorilla
  378182: .........................................T........  Pabelli
  302964: ................................................GA  Nleucogenys
  372525: ....G.......C.....................................  Mmulatta
  325117: ............C.....................................  Panubis
  362345: .........G..TG........A...........................  Cjacchus
 1013893: .........G..C.........A...........................  Sboliviensis

                9008      9018      9028      9038      9048
                   :         :         :         :         :
    8999: ATGACAGCAGCAGCACTGGTGACAGCACTGAATGAAAAGTCCCTCCTTCA  Hsapiens
  328566: ..................................................  Ptroglodytes
  346438: ...........G......................................  Ggorilla
  378232: ...........................T......................  Pabelli
  303014: ..................................................  Nleucogenys
  372575: ...............G................................T.  Mmulatta
  325167: ..........G....G................................T.  Panubis
  362395: ...............G...........A..................C...  Cjacchus
 1013943: ...............G..A........A..................C...  Sboliviensis

                9058      9068      9077      9087      9097
                   :         :         :         :         :
    9049: TCCCAGACTGCACAAAGAGG-AAAATCATGGAGGGAACATCAGGAAGGGA  Hsapiens
  328616: ....................-.............................  Ptroglodytes
  346488: ....................-.............................  Ggorilla
  378282: ....................-............A.....G..........  Pabelli
  303064: ....................-.............................  Nleucogenys
  372625: ....................A........................C...T  Mmulatta
  325217: ....................-.............................  Panubis
  362445: ...........TT.......-.............................  Cjacchus
 1013993: ..........A.........-........A....................  Sboliviensis
```

```
                  9107      9117      9127      9137      9147
                     :         :         :         :         :
   9098: CTCCACAGGGGCTTTCCTGTCCAGCTCAGAAAGGCACTTGTCATCTCCCG  Hsapiens
 328665: ..................................................  Ptroglodytes
 346537: ........................................G.........  Ggorilla
 378331: ........................................G.........  Pabelli
 303113: .G.............T......................CAG.........  Nleucogenys
 372675: ......-.................................G....C...A  Mmulatta
 325266: ...T..-.................................G....C...A  Panubis
 362494: ...............T................TT.G....G.......T.  Cjacchus
1014042: .......C.......T................T.....-.GT....G.T.  Sboliviensis

                  9157      9167      9176      9186      9196
                     :         :         :         :         :
   9148: ACTCTGCAGGAAGCACAGAGGAGTG-CCCCATGGTGTCGTGTTGCTCATC  Hsapiens
 328715: .........................C........................  Ptroglodytes
 346587: .........................-...........G..........C.  Ggorilla
 378381: ...............T.........-........................  Pabelli
 303163: .........................-...........T............  Nleucogenys
 372724: .........................-.....G...A............C.  Mmulatta
 325315: .........................-.....G...A..A...........  Panubis
 362544: .....A...................-........................  Cjacchus
1014091: .........................-................C.......  Sboliviensis

                  9206      9216      9226      9236      9246
                     :         :         :         :         :
   9197: CTTCCATTGACCAACAAACCAGAGAAGTAAACATTCTAAAGATTGTCCTT  Hsapiens
 328765: ...........................C......................  Ptroglodytes
 346636: ...........................C......................  Ggorilla
 378430: ...........................C......................  Pabelli
 303212: ...........................C......................  Nleucogenys
 372773: ...........................C..............C.T.....  Mmulatta
 325364: ...........................C..............C.T.....  Panubis
 362593: T.........T...........GA...C..............C.......  Cjacchus
1014140: T.........T...........GA...C...T..........C.......  Sboliviensis

                  9256      9266      9276      9286      9296
                     :         :         :         :         :
   9247: TCACATTCCCTTTCCAACTGTGTGTGCCTGGACATATGGCAGGTTGTAGC  Hsapiens
 328815: ..................................................  Ptroglodytes
 346686: ..................................................  Ggorilla
 378480: .........................................A........  Pabelli
 303262: ........T...........................G....A........  Nleucogenys
 372823: ..........A......A...............G..G....A........  Mmulatta
 325414: ..........A......A..............TG..G....A........  Panubis
 362643: ..........A........A................G....A........  Cjacchus
1014190: ..........A.........................GA...A.....G.G  Sboliviensis

                  9306      9316      9326      9336      9346
                     :         :         :         :         :
   9297: CTTAGAGTCAGGAATCAGGGCCAGCAGGTTAGAACCAGGGAAGGACCCAG  Hsapiens
 328865: ..................................................  Ptroglodytes
 346736: ..................................................  Ggorilla
 378530: ..................A...........................A...  Pabelli
 303312: ..................A...........................A...  Nleucogenys
 372873: ..GG..............A..........................TG...  Mmulatta
 325464: ..GG..............A...........................G...  Panubis
 362693: ..G...............A.A......A....G.....A.......A...  Cjacchus
1014240: ..G...............A.A......A.......-----------A...  Sboliviensis
```

```
                  9356      9366      9376      9386      9396
                     :         :         :         :         :
   9347: GAGGGAGCCAGAGCCAGGAGAACAGACTTTGGACAATCCAATCCTCCACC  Hsapiens
 328915: ..................................................  Ptroglodytes
 346786: .................................................T  Ggorilla
 378580: ..................................................  Pabelli
 303362: ..........C.......................................  Nleucogenys
 372923: ......................G........A..................  Mmulatta
 325514: ................T.....G...........................  Panubis
 362743: ..A...T.....A.......G.T......G..G..G..............  Cjacchus
1014279: ............A.......G.T.........G..G..............  Sboliviensis

                  9406      9416      9426      9436      9446
                     :         :         :         :         :
   9397: GAGGACCAAGGTTAGATGCCTGGGTTTTATCTATTTCCATGGCAGTCATT  Hsapiens
 328965: ..................................................  Ptroglodytes
 346836: C.................................................  Ggorilla
 378630: C.................................................  Pabelli
 303412: C.................-................G....A.........  Nleucogenys
 372973: C....................A...................A.......G  Mmulatta
 325564: C....................A...................A.......G  Panubis
 362793: C.....T...............................GG.A........  Cjacchus
1014329: C.C.................CA.................G.A........  Sboliviensis

                  9456      9466      9476      9486      9496
                     :         :         :         :         :
   9447: GACTAGGGTTCAAATGGTCTCCCCAAAAGAGGAGGTGACTCAAGTCACAG  Hsapiens
 329015: ..................................................  Ptroglodytes
 346886: ........................G.....T...................  Ggorilla
 378680: ........................G.....T...................  Pabelli
 303461: ..............C........TG.....T......T............  Nleucogenys
 373023: ..........A............TG..C..T..A..A.............  Mmulatta
 325614: ..........A............TG..C..T..A..A.............  Panubis
 362843: A......................TG.....CA.A..........C.....  Cjacchus
1014379: A.A....................TG.....CA.A.....G..........  Sboliviensis

                  9506      9516      9526      9536      9546
                     :         :         :         :         :
   9497: ATCTCATGATAAGTTTGCAACATACAGATGGTTTTTACCAATATCAAGTG  Hsapiens
 329065: ..................................................  Ptroglodytes
 346936: ..................................................  Ggorilla
 378730: ...............C..................................  Pabelli
 303511: ..................................................  Nleucogenys
 373073: .............C..........T..-......................  Mmulatta
 325664: .............C..........T..-......................  Panubis
 362893: .............C..........................T..AG.....  Cjacchus
1014429: .............C..........................T..AG.....  Sboliviensis

                  9556      9566      9576      9586      9596
                     :         :         :         :         :
   9547: GCAATATACAGCTCTGGAGAAGCACTGACAGCCCACATACACAGCAGAGA  Hsapiens
 329115: ..................................................  Ptroglodytes
 346986: .....................A............................  Ggorilla
 378780: .....................A............................  Pabelli
 303561: .....G.........T.....A............................  Nleucogenys
 373122: .....................A...........------...........  Mmulatta
 325713: ................A....A...........------...........  Panubis
 362943: .....................A..-.........................  Cjacchus
1014479: ........T............A.............T..........C...  Sboliviensis
```

```
                                                MIR
                                            ---------->
           9606      9616      9625      9635      9645
   9597: CCAGATATTCTGAGACAGAC-TCAAGATATACCTTCCAGAAAGTATGCCC  Hsapiens
 329165: ....................-.............................  Ptroglodytes
 347036: ....................-.............................  Ggorilla
 378830: ......G.............-.......................G.....  Pabelli
 303611: ....................-C......................G.....  Nleucogenys
 373166: ..............C.....-.............A.........G.....  Mmulatta
 325757: ..............C.....-.......................G.....  Panubis
 362992: ..............C.....A......A...T..............A...  Cjacchus
1014529: ..............C.....A..........T..................  Sboliviensis
```

```
                                              MIR
                                           ---------->
           9655      9665      9675      9685     9694
   9646: TTGGATGCAAAATACATGAGGTGGGGTGTCACAGCTAAGGC-TTAGAGCC  Hsapiens
 329214: .........................................-........  Ptroglodytes
 347085: .........................................-........  Ggorilla
 378879: .........................................-........  Pabelli
 303660: .................A......A................-........  Nleucogenys
 373215: ..A......G............A........G.........-......G.  Mmulatta
 325806: ..A......G...............................-......G.  Panubis
 363042: ......................A...........T......T........  Cjacchus
1014579: ......................A..................T........  Sboliviensis
```

```
                            MIR
         -------------------------------------------------->
           9704      9714      9724      9734      9744
   9695: ACGCAAACCTGGGTTCCAATTCCAGCTCCCCATCTTGGACCCATGTGACC  Hsapiens
 329263: ..........................C..A....................  Ptroglodytes
 347134: ..................................................  Ggorilla
 378928: .T................................................  Pabelli
 303709: .T................................................  Nleucogenys
 373264: ..A...................T...........................  Mmulatta
 325855: ..A...................T...........................  Panubis
 363092: .A..............................G.........G.......  Cjacchus
1014629: .A......................A.......G.................  Sboliviensis
```

```
                            MIR
         -------------------------------------------------->
           9754      9764      9774      9784
   9745: TTTGAGAGGCTCTTCAACCTGTCTGTATCTTGCTTCACTTGTTT------  Hsapiens
 329313: ............................................------  Ptroglodytes
 347184: ............................................------  Ggorilla
 378978: ..C........................................CCTCTCT  Pabelli
 303759: ..CA........................................------  Nleucogenys
 373314: ..CA........................................------  Mmulatta
 325905: ..CA.A......................................------  Panubis
 363142: ..CA....A..G.......A..T.A.G.............A...------  Cjacchus
1014679: ..CA....A..G..........T...G.............A...------  Sboliviensis
```

```
                                     MIR
         -------------------------------------------------->
               9791      9801      9811      9821
    9789: -------GAAAAATGGGGAAATTTTTCATAACGTCATGGTTAAGAG----  Hsapiens
  329357: -------........................A..............----  Ptroglodytes
  347228: -------.......................................----  Ggorilla
  379028: GTTTATATT.GG..................................CCCT  Pabelli
  303803: -------.......................................----  Nleucogenys
  373358: -------.......T......................A........----  Mmulatta
  325949: -------..............................A........----  Panubis
  363186: -------...................T.....T.............----  Cjacchus
 1014723: -------...................T.....T..G..........----  Sboliviensis

                                     MIR
         -------------------------------------------------->
               9837      9847      9857      9866      9876
    9828: TATTATAGGTAACATGTAAAGTACTTAGTACACAGTA-ACCACTTGATAA  Hsapiens
  329396: .....................................-.G..........  Ptroglodytes
  347267: .....................................-.G..........  Ggorilla
  379078: .T....C.TAT.....................T....G.G..........  Pabelli
  303842: G....C..........A...........G...T....-.G..........  Nleucogenys
  373397: G.................G.............T....-.G..........  Mmulatta
  325988: G.................G.............T....-.G..........  Panubis
  363225: G......................A.............-.AT.........  Cjacchus
 1014762: G..........GG..........A.....G..TG...-.AT.........  Sboliviensis

                 MIR
         ---------------->
               9886      9896      9906      9916      9926
    9877: GTGTAGCTATTATTATAATAATAAGTTGAGACAGGAAAATCCAGGATGTA  Hsapiens
  329445: A.................................................  Ptroglodytes
  347316: ..................................................  Ggorilla
  379128: A........                                           Pabelli
  303891: A......................G..........................  Nleucogenys
  373446: A...........A..........G..........................  Mmulatta
  326037: A......................G..........................  Panubis
  363274: A......C...............G......G.......G...........  Cjacchus
 1014811: A.............G........G..............G......G....  Sboliviensis

               9936      9946      9956                9975
    9927: TGAGTACTCCACCTATCCATATATTGCCTATTACTCAAG-GCCACCCAAA  Hsapiens
  329495: ...............C.......................-..........  Ptroglodytes
  347366: .......................................-..........  Ggorilla
  303941: .......................................-..........  Nleucogenys
  373496: .......................................-......TG..  Mmulatta
  326087: .................................T.....-..........  Panubis
  363324: ....A..........G.T.C...................A..........  Cjacchus
 1014861: .................T.C...................A..........  Sboliviensis

               9985      9995     10005     10015     10025
    9976: CTTCCACCATTTCCATCATCTTCCATATTATTATGAGCCTATATTGAGTT  Hsapiens
  329544: G.................................................  Ptroglodytes
  347415: G.................................................  Ggorilla
  303990: G.................................................  Nleucogenys
  373545: G........................A...........A............  Mmulatta
  326136: G....G............C...............................  Panubis
  363374: T.....A...........................................  Cjacchus
 1014911: G......A...............T..........................  Sboliviensis
```

```
               10035     10045     10055     10065     10075
                   :         :         :         :         :
   10026: CTTTATTTCTTAATAATATTCTTTTATCATATGATTTTACTAAATATTTC  Hsapiens
  329594: ......................................G...........  Ptroglodytes
  347465: ..................................................  Ggorilla
  379421:                ......ATC...............-............  Pabelli
  304040: ...............................C..................  Nleucogenys
  373595: ...C................A...C..TG.....................  Mmulatta
  326186: ...C................A......TG.....................  Panubis
  363424: ----.......G.......G.....G.TG....-------..........  Cjacchus
 1014961: ...........G.......G.......T.....-------..........  Sboliviensis

               10085     10095     10105     10115     10125
                   :         :         :         :         :
   10076: CACTGCTGTATAATTTTAGATTGTCACTCATAAGTCACTACTTACGTCAC  Hsapiens
  329644: ............................................TA....  Ptroglodytes
  347515: .............................G..............T.....  Ggorilla
  379457: -...-...........-....-.....................-.T.....  Pabelli
  304090: ................C...........................T.....  Nleucogenys
  373645: ...................G..............................  Mmulatta
  326236: ............................................T.....  Panubis
  363463: .........................G.............----.T.....  Cjacchus
 1015004: ............................T................A....  Sboliviensis

               10135     10145     10155     10165     10175
                   :         :         :         :         :
   10126: TTATAAGTCATTTAGTACAAAATCCTTTCCTAAACACAAAAACCAAGCCA  Hsapiens
  329694: .............C...................G................  Ptroglodytes
  347565: .................................G................  Ggorilla
  379502: .-.....................AT....-...G....G...........  Pabelli
  304140: ...A....................T.............G...........  Nleucogenys
  373695: ..........C.A.A........AT........G....G...........  Mmulatta
  326286: ..........C.A.A.........T........G....G...........  Panubis
  363509: ..........C.A...........TG.....G.G....G...........  Cjacchus
 1015054: ..........C.A...........T......G.G....G...........  Sboliviensis

               10185     10195     10205     10214     10224
                   :         :         :         :         :
   10176: TGCTGTTTTAAAATCTACATTGTGTTGAACA-TACTACAAGAAACCTGGT  Hsapiens
  329744: ...............................T..................  Ptroglodytes
  347615: ...............................-..................  Ggorilla
  379550: ...............................-..................  Pabelli
  304190: ..........................A....-..................  Nleucogenys
  373745: ..................G............-..................  Mmulatta
  326336: ..................G............-..................  Panubis
  363559: ............G.G...............A-......C...........  Cjacchus
 1015104: ..T.........G.G...G...........A-.G....T...........  Sboliviensis

               10234     10244     10254     10264     10274
                   :         :         :         :         :
   10225: AAGCACTTGATAAGTAGGTGCTTAATTAATTGCATTTTAGGTACTATTTT  Hsapiens
  329794: ..................................................  Ptroglodytes
  347664: ..................................................  Ggorilla
  379599: ............................G...............CG....  Pabelli
  304239: ..........G.......................................  Nleucogenys
  373794: .........................A..........AC............  Mmulatta
  326385: ...................A................AC.......G....  Panubis
  363608: ........T........C.A.....-.G....T.C.AC............  Cjacchus
 1015153: .................C.A.....-.G....T...AC............  Sboliviensis
```

```
                    10284       10294       10304       10314       10324
                        :           :           :           :           :
   10275: GGGTTGGTCATGATGATAAATATCCGTTGTAAACACCTTAACAGTTCCTG  Hsapiens
  329844: ..................................................  Ptroglodytes
  347714: .........T........................................  Ggorilla
  379649: .........................A.........T..............  Pabelli
  304289: .........................A........................  Nleucogenys
  373844: -.......................T..........G..............  Mmulatta
  326435: -..........C..A..........A.........G..............  Panubis
  363657: .AA....-.................A.G........T.G.....G.....  Cjacchus
 1015202: ..A....-..........G.....TA.G........T.......A.....  Sboliviensis

                    10334       10343       10352       10362       10372
                        :           :           :           :           :
   10325: CATTACCTTTCAG-AAAAAGACCTTTCA-TTTTTTGCACTCCTCCATCAT  Hsapiens
  329894: .............-.......T......-.......T...G.........  Ptroglodytes
  347764: .............-..............-...........G.........  Ggorilla
  379699: .............-..............-...........G.........  Pabelli
  304339: .............A............A.-......T....G.........  Nleucogenys
  373893: .............-..............-...........G.........  Mmulatta
  326484: .............A..............-...........G.........  Panubis
  363706: .....G.......-..............C.......T...G.........  Cjacchus
 1015251: .....G.......-..............-.....C.T...G.........  Sboliviensis

                    10382       10392       10402       10412       10422
                        :           :           :           :           :
   10373: CTGTATGGAAAATACAAATTTTATCTATCTTATTAAAATATTGCCATTTT  Hsapiens
  329942: ..-...............................................  Ptroglodytes
  347812: ..................................................  Ggorilla
  379747: ...C..A.....C..........................G..........  Pabelli
  304388: ..................................................  Nleucogenys
  373941: .........G.........C...C.....--...............C...  Mmulatta
  326533: .........G.........C...C......C...............C...  Panubis
  363755: ......A............A......G...C...T...........C...  Cjacchus
 1015299: .....C...........G.A......G...C...............C...  Sboliviensis

                    10432       10442       10452       10462       10472
                        :           :           :           :           :
   10423: AACTTTGATATTGAATATGGTCATGGCTTCGTGAGTATGGAGACATAAAT  Hsapiens
  329991: .................CA...............................  Ptroglodytes
  347862: .................CAC..............................  Ggorilla
  379797: .................CA........C..A...................  Pabelli
  304438: ...G.............CA........C......................  Nleucogenys
  373989: .................CA........CCTC...................  Mmulatta
  326583: .................CA........CCTT...................  Panubis
  363805: .......G..C......CA...G....CCT......G..........G.A  Cjacchus
 1015349: .......G.........CA.......TCCT......G..........G..  Sboliviensis

                    10482       10492       10502       10512       10522
                        :           :           :           :           :
   10473: ATGAATCACTATACTCTCATATATTCACTAGTAAAACCATTTCAAATTGT  Hsapiens
  330041: ..A..........................................TC...  Ptroglodytes
  347912: ..............................................C...  Ggorilla
  379847: ..............................................C...  Pabelli
  304488: ..............................................C...  Nleucogenys
  374039: ..............................................CCTG  Mmulatta
  326633: .............--...............................CCTG  Panubis
  363855: .G............C....................T.T........CCTG  Cjacchus
 1015399: .....................................T........CCTG  Sboliviensis
```

```
              10532     10542     10552     10562     10572
  10523: GGGGGGAAGATGAGTTTTGGAAACACAAATGTAATTCTCACCCACCTCAT  Hsapiens
 330091: .........G.......G................................  Ptroglodytes
 347962: ..................................................  Ggorilla
 379897: ..............G......G...........G................  Pabelli
 304538: ..............G...................................  Nleucogenys
 374089: T.............G.....C......G....C..........G......  Mmulatta
 326681: T.............G.....C...........C..........G.....C  Panubis
 363905: T.......T.....G..........T...............T.....T..  Cjacchus
1015449: TA............G.......G..T....A..........T........  Sboliviensis

              10582     10592     10602     10612     10622
  10573: CCTATTCCTGATATAATGTTATTAGAAATGACATAAAATTTGTAATAAGG  Hsapiens
 330141: .......T..........................................  Ptroglodytes
 348012: ..................................................  Ggorilla
 379947: ..................................................  Pabelli
 304588: ..................................................  Nleucogenys
 374139: ........C...G...............................G.....  Mmulatta
 326731: ........CA........................................  Panubis
 363955: ........CA.G......................................  Cjacchus
1015499: ........CA.G......................................  Sboliviensis

              10632     10642     10652     10662     10672
  10623: GAAACTTCCATCACTGTTGATAAGGGAATAATGTCAGTACCAGAAATTTC  Hsapiens
 330191: ..................................................  Ptroglodytes
 348062: ..................................................  Ggorilla
 379997: ..........C.............A.........................  Pabelli
 304638: .C................C...............................  Nleucogenys
 374189: .....C..............................T.........G...  Mmulatta
 326781: .....C....CT.......G................T.........G...  Panubis
 364005: ....TC.....................G.T....A...............  Cjacchus
1015549: .....C.....................G.T....A...............  Sboliviensis

              10682     10692     10702     10712     10722
  10673: ATGGAAAGATAACCAGAGTTCACTTGCAAATCACCGTTACAGTAAGCAAT  Hsapiens
 330241: .C..........TT....................................  Ptroglodytes
 348112: ..................................................  Ggorilla
 380047: ....................------...............AG.......  Pabelli
 304688: ...................................A.....A........  Nleucogenys
 374239: ...................................A.....A.G......  Mmulatta
 326831: ...................................A.....A.G......  Panubis
 364055: ................G..................A.C...AGG......  Cjacchus
1015599: ................G..................A.C...AGG.....C  Sboliviensis

              10732     10742     10752     10762     10772
  10723: GACCTTGAGGGAGCAGGCGATGGAAGCCCTGCCCTCCACACCTGAACATG  Hsapiens
 330291: ..................................................  Ptroglodytes
 348162: ......C...........................................  Ggorilla
 380091: .................T..............T.................  Pabelli
 304738: ..................................................  Nleucogenys
 374289: .....................A.......C.................T..  Mmulatta
 326881: .........................CT.......................  Panubis
 364105: .................T........A.......................  Cjacchus
1015649: A.T..............T................................  Sboliviensis
```

```
                 10782     10792     10798     10808     10818
                     :         :         :         :         :
   10773: AAAACAGAAGGATGGGCGTGTGG----GATGGTGCAGGCAGCAAGACCGA  Hsapiens
  330341: .................A.....----.......................  Ptroglodytes
  348212: .......................----....................T..  Ggorilla
  380141: ...............A.......GGAA.....................A.  Pabelli
  304788: ...............A.A.....GGAA..........A..........A.  Nleucogenys
  374339: ...........G...A.......GGAA.....................A.  Mmulatta
  326931: ..G........G...A.......GGAA.....................A.  Panubis
  364155: ...............A...CA..GGAA..CA....G............A.  Cjacchus
 1015699: .....G........CAT..CG..GGAA.....................A.  Sboliviensis

                 10828     10838     10848     10858     10868
                     :         :         :         :         :
   10819: CCGAGAATAGCTTCATATGGAATCTCTTTTGCTTATAAAGTGATGGGATT  Hsapiens
  330387: .............................................A....  Ptroglodytes
  348258: ..................................................  Ggorilla
  380191: ..................................................  Pabelli
  304838: .T................................................  Nleucogenys
  374389: ..A..........T....................................  Mmulatta
  326981: ..AC...G..........................................  Panubis
  364205: ..T.....G.T..........G...G.........G..............  Cjacchus
 1015749: ..A.....G............G.............AT.....G.......  Sboliviensis

                 10878     10888     10898
                     :         :         :
   10869: TGAAGGCAGAGTGACAAGGGATCCTAGAAATTCAG---------------  Hsapiens
  330437: ...................................---------------  Ptroglodytes
  348308: .....................C.............---------------  Ggorilla
  380241: ...G.............T.................---------------  Pabelli
  304888: ...G.............T.................---------------  Nleucogenys
  374439: ...G.............T.................---------------  Mmulatta
  327031: ...G.............T.................---------------  Panubis
  364255: ...GA............T.T...............ATATATGTGTGTGTG  Cjacchus
 1015799: ...G.............T.T............T.T---------------  Sboliviensis

                           10908     10918     10928
                               :         :         :
   10904: ---------------ATAGATATAAAGGCAAAAAGCCCCTGAAGAGGGA-  Hsapiens
  330472: ---------------..........G.......................-  Ptroglodytes
  348343: ---------------..................................-  Ggorilla
  380276: ---------------..........G.........A.T...........-  Pabelli
  304923: ---------------..G.......G.....................T.-  Nleucogenys
  374474: ---------------..G.......G.......................-  Mmulatta
  327066: ---------------..G.......G.......................-  Panubis
  364305: TGTATATATATATAT...T.....TG.........-.............T  Cjacchus
 1015834: -------------AT...T.....TGA......................-  Sboliviensis

                 10947     10957     10967     10977     10986
                     :         :         :         :         :
   10938: TTTCCTGAGATGACAAGACATGTCTTTATAGTGAGTAGTTGACTGAC-AA  Hsapiens
  330506: ...............................................-..  Ptroglodytes
  348377: C..............................................-..  Ggorilla
  380310: ...............................................-..  Pabelli
  304957: ...............................................-..  Nleucogenys
  374508: .............T...........................G.....-..  Mmulatta
  327100: ........................C...C...A-----------...-..  Panubis
  364354: ...........-----............................ACA-..  Cjacchus
 1015870: ...........-----.....A.............A......G....A..  Sboliviensis
```

```
                 10996     11006     11016     11026     11036
   10987: AAGTGGCCTTGAAAACCAGATGTGCAAAGAAATTCATCATTGACAAAGTT  Hsapiens
  330555: ......................C...........................  Ptroglodytes
  348426: ..................................................  Ggorilla
  380359: .......................................C..G...G...  Pabelli
  305006: ..........................................G...GA..  Nleucogenys
  374557: ..........................................G...G...  Mmulatta
  327138: ..........................................G...G...  Panubis
  364398: .....T..........T...G.....................C.T.G...  Cjacchus
 1015915: .....A..........T...G.....................G...GA..  Sboliviensis

                 11046     11056     11066     11076     11086
   11037: TTGGAAGAATGAATTCCCAATGTGGCTTATTATAAATTAGTGAATAAAGC  Hsapiens
  330605: ............................G.....................  Ptroglodytes
  348476: ............................G.....................  Ggorilla
  380409: .......G....................G...................A.  Pabelli
  305056: ............................G............T........  Nleucogenys
  374607: ....................A.C.....G.....................  Mmulatta
  327188: ............................G.....................  Panubis
  364448: ....................C....T..G.........C..........T  Cjacchus
 1015965: ....................C....T.......................T  Sboliviensis

                 11096     11106     11116     11126     11136
   11087: AGGGTTGTTACAGCCAAAAGTGAACTGGATGACTAGCTACAGAGGACTCT  Hsapiens
  330655: ...............................C..................  Ptroglodytes
  348526: ............C...........A......C..................  Ggorilla
  380459: ...............-...............C..................  Pabelli
  305106: .............TT....C..........AC..................  Nleucogenys
  374657: .............T.................C..G........A......  Mmulatta
  327238: .............T.................C..G...............  Panubis
  364498: .-..........................----------------......  Cjacchus
 1016015: .-.............................T...TA.............  Sboliviensis

                 11146     11156     11166     11176     11184
   11137: GCACCTAGATCAAGGGTTTGGAAAACAACCAACACCACAAG--AAAAAAA  Hsapiens
  330705: .........................................--.......  Ptroglodytes
  348576: .......................G.................--.......  Ggorilla
  380508: .........................................---......  Pabelli
  305156: .........................................--.......  Nleucogenys
  374707: ..............T..........................---......  Mmulatta
  327288: ..............T..........................---......  Panubis
  364531: ...............C................AG.....G.GA.......  Cjacchus
 1016064: ...............C................AG.....G.---......  Sboliviensis

                 11194     11204     11214     11224     11234
   11185: GGAAAATAAAACAAAAACAATCAAAACAGAGAAGTGACTGCACAGAAACA  Hsapiens
  330753: ..................................................  Ptroglodytes
  348624: ..................................................  Ggorilla
  380555: ..................................................  Pabelli
  305204: ..................................................  Nleucogenys
  374754: .............................T....................  Mmulatta
  327335: ............-................T....................  Panubis
  364581: T.........................................T..T.C..  Cjacchus
 1016111: .................T.............................C..  Sboliviensis
```

```
                  11241     11251     11261     11271     11281
   11235: AATT---CTCCCTACTAGGCTTGCCCATGACCCTGAATCTCTTCTTCTGT  Hsapiens
  330803: ....CTC...........................................  Ptroglodytes
  348674: ....---...........................................  Ggorilla
  380605: ....---.........................T.................  Pabelli
  305254: ....---................................A..........  Nleucogenys
  374804: ....---.....................C.....................  Mmulatta
  327384: ....---.................T...C.....................  Panubis
  364631: ....---........C................-.................  Cjacchus
 1016161: ....---.........................T.................  Sboliviensis

                  11291     11301     11311     11321     11331
   11282: CCCGACATTTGCAACAGTGAATTAATAAAGTTTAATTACTTATGACATTT  Hsapiens
  330853: ..T.....................G.........................  Ptroglodytes
  348721: ..T.....................G.........................  Ggorilla
  380652: ..T.....................G.........................  Pabelli
  305301: .TT.....................G.........................  Nleucogenys
  374851: ..T.......A.............G..........G....G.........  Mmulatta
  327431: ..T.......A.............G..........G....G.........  Panubis
  364677: ..T..T..........C..G....G.......C..G...GG.........  Cjacchus
 1016208: ..T.............C..G...GGC.........G....G.........  Sboliviensis

                                            LINE1
                                    ------------------------>
                  11341     11344     11354     11364
   11332: ATAAAGTGATC-------AAAAAAAAATCAAAGTATGTGAGAGACCAG--  Hsapiens
  330903: ......G....-------..............................--  Ptroglodytes
  348771: ......C....--------.............................--  Ggorilla
  380702: ......G...A--------.............................-A  Pabelli
  305351: .....TG....--------.....................A...G...--  Nleucogenys
  374901: .C....G....-----AA..............................AA  Mmulatta
  327481: ......G....AAAAAAA..............................--  Panubis
  364727: ......G....------A...CC......G..................--  Cjacchus
 1016258: ......G....------A....C...G.....A...............--  Sboliviensis

                               LINE1
          ------------------------------------------------->
                  11382     11392     11402     11412     11422
   11373: AAAAAAAATTGAAGAATATAATTAACAAGAAGAAATGAAGGAACACTTAA  Hsapiens
  330944: ...........................G......................  Ptroglodytes
  348811: --.........................G...........C..........  Ggorilla
  380743: ...........................G......................  Pabelli
  305391: ...........................G......................  Nleucogenys
  374946: .................G.----....G......................  Mmulatta
  327529: .....................C.....G......................  Panubis
  364769: .C.G..G.C...G.........G..T.G........A......G......  Cjacchus
 1016300: .C.G..G.C...G............T.G......G...............  Sboliviensis
```

```
                                LINE1
         ------------------------------------------------->
              11432     11442     11452     11462
  11423: ACAGCAGGATAGATATATCATGTTTATGGAATGGAACACTCAATAT---- Hsapiens
 330994: ......................A.......................---- Ptroglodytes
 348859: ..............................................---- Ggorilla
 380793: .......A.............A........................---- Pabelli
 305441: .....................A........................---- Nleucogenys
 374992: ..............................................---- Mmulatta
 327579: ..............................................---- Panubis
 364819: .TG.........G.............................G...---- Cjacchus
1016350: .TG.....G...G.............C...................TATA Sboliviensis

                                LINE1
         ------------------------------------------------->
              11474     11484     11494     11504
  11469: ----TATAAACATGTCAATTTTCCCAAATTTTCCATCTTTTTTTT----- Hsapiens
 331040: ----................C..............A.........TTTTT Ptroglodytes
 348905: ----................C........................TTTTT Ggorilla
 380839: ----C...............C........................TTTTT Pabelli
 305487: ----................C.............G..........----- Nleucogenys
 375038: ----................C.G..............AC..--------- Mmulatta
 327625: ----................C.G..............ACG.--------- Panubis
 364865: ----....T.TG........C.................------------ Cjacchus
1016400: TATA....T.T....G....C.........G...G...------------ Sboliviensis

                                LINE1
         ------------------------------------------------->
              11518     11528     11538               11549
  11510: -TCTTTTTTGCTATTCCAATCAAAATTTAGCA-GGTT--------TTTTT Hsapiens
 331086: T.T.............................-....--------..... Ptroglodytes
 348951: ---.............................-....TT------..... Ggorilla
 380885: T--................C............G....--------..... Pabelli
 305528: ---.............................-....--------..... Nleucogenys
 375075: ---.............................-.T..--------....G Mmulatta
 327662: ---.............................-....--------....G Panubis
 364899: ---....C..................C.....-....TGTGTGCA.G.G. Cjacchus
1016438: ---....C.............-....C.....-....--------.G.G. Sboliviensis

                                LINE1
         ------------------------------------------------->
              11559                         11562     11572
  11550: GTGTATATGTG---------------------------TTGTAAATTGCC Hsapiens
 331127: ...........---------------------------............ Ptroglodytes
 348991: T..........---------------------------............ Ggorilla
 380925: .........C.--------------------------T............ Pabelli
 305566: ...........--------------------------T............ Nleucogenys
 375113: ...........--------------------------T............ Mmulatta
 327700: ..........A--------------------------T............ Panubis
 364945: ...........--------------------------T............ Cjacchus
1016475: ....G.G....TGTGTGTGTGTGTGTGTGTGTGTGTGT............ Sboliviensis
```

```
                              LINE1
          ------------------------------------------------->
                11582     11592     11602     11612     11622
    11573: AGGTTGATTCTAAAATTTTTATCAAAAGAAAGAAGGCCAAGAATAATGAA  Hsapiens
   331150: ..................................................  Ptroglodytes
   349014: ..................-...............................  Ggorilla
   380949: ..................................................  Pabelli
   305590: T.................A...............................  Nleucogenys
   375137: ..................A...............................  Mmulatta
   327724: ..................A...............................  Panubis
   364969: ...C.....T.C......A...G......................G....  Cjacchus
  1016525: ...C..............A...G....................C......  Sboliviensis
```

```
                              LINE1
          ------------------------------------------------->
                11632     11641     11651     11661     11671
    11623: GACAATCTTGAAGTTT-AAAAATGAAATTGGAGCCTTACATTAGCAGATG  Hsapiens
   331200: ................-................T................  Ptroglodytes
   349063: ................-.................................  Ggorilla
   380999: .G..............-.................................  Pabelli
   305640: ................-......A...C......................  Nleucogenys
   375187: ................-................T................  Mmulatta
   327774: ................-................T................  Panubis
   365019: ................A.........G....................---  Cjacchus
  1016575: ................-...G.....G.....................CA  Sboliviensis
```

```
                              LINE1
          ------------------------------------------------->
                11681     11691     11701     11711     11721
    11672: CCAACTATAAGGCTACAGTAATTAAGACTATGATATTGGTGCAAAGATAG  Hsapiens
   331249: ..................................................  Ptroglodytes
   349112: ..................................................  Ggorilla
   381048: ..................................................  Pabelli
   305689: .....................................C............  Nleucogenys
   375236: ..................................................  Mmulatta
   327823: ..................................................  Panubis
   365066: ---.......A....TGA.........T................G.....  Cjacchus
  1016624: ..........A.TCGTG..........TG...G....T......G.....  Sboliviensis
```

```
                              LINE1
          ------------------------------------------------->
                11731     11741     11751     11761     11771
    11722: GCAAACAGAGCAGCAGAATAGAAGAGGGCGGCCAAAAACAGACCAGCCAA  Hsapiens
   331299: ..................................................  Ptroglodytes
   349162: .............................A....................  Ggorilla
   381098: ............................T.A..C................  Pabelli
   305739: .......A........................................G.  Nleucogenys
   375286: .....T......................A.....................  Mmulatta
   327873: .....T....................A.A.....................  Panubis
   365113: A....T...................CC.A...A.............T...  Cjacchus
  1016674: AA...G.............G........A....C............      Sboliviensis
```

```
                               LINE1
      ---------------------------------------------------------->
                11781     11791     11801     11811     11821
 11772: TTTCATGTTGCTTTCAAACAAAGTCACCATTAAGTAAAGGAAGAGGGACA  Hsapiens
331349: ..................................................  Ptroglodytes
349212: ..................T...............................  Ggorilla
381148: ..................................................  Pabelli
305789: ........................G.........................  Nleucogenys
375336: ..............................C...C...............  Mmulatta
327923: ...................-..............................  Panubis
365163: .................G........................T-....T.  Cjacchus

                               LINE1
      ---------------------------------------------------------->
                11831     11841     11851     11861     11871
 11822: GAGAGTCTTTCCAGTAAATGGTCTGGATCTACTGGATATCCATACCTTTT  Hsapiens
331399: ..........T.......................................  Ptroglodytes
349262: ..........T.......................................  Ggorilla
381198: .....C....T.......................................  Pabelli
305839: ..........T......................T...........T....  Nleucogenys
375386: A..G......T..................A...............T....  Mmulatta
327972: A....C....T..................A.G.............T....  Panubis
365212: ..........T..A.........A.....A.......T....C..-....  Cjacchus

                               LINE1
      ---------------------------------------------------------->
                11880     11890     11900     11906     11916
 11872: TC-AAAAAATGAAGTTTAATCCATATTTTACAGG----GGTCACAAAAAT  Hsapiens
331449: ..-................C..............----............  Ptroglodytes
349312: ..-.......A.......................----............  Ggorilla
381248: ..-...............................----............  Pabelli
305889: ..-.............................A.----............  Nleucogenys
375436: ..-...............................----............  Mmulatta
328022: ..A...............................----............  Panubis
365261: ..-.........C.C...............T...GCTC....G.......  Cjacchus

                               LINE1
      ---------------------------------------------------------->
                11926     11936     11946     11956     11966
 11917: AAATTTCAGATGGCTTACAGATCTAAAGTAAAATGATTAAACAAAAAGAC  Hsapiens
331494: ..................................................  Ptroglodytes
349357: ..................................................  Ggorilla
381293: ............................G...............T.....  Pabelli
305934: ............................G...............T.....  Nleucogenys
375481: ..........C.T....T..........G.........T.....T.....  Mmulatta
328068: ..........A.T....T..........G...............T.....  Panubis
365310: .......CA........T..........AC..............T....T  Cjacchus

                               LINE1
      ---------------------------------------------------------->
                11976     11986     11996     12006     12016
 11967: TTCTAGAAGATAACAAAGGGGAATATCTTCATCAACTTGGTTTAGACAAA  Hsapiens
331544: ..................................................  Ptroglodytes
349407: ..................................................  Ggorilla
381343: ..T...............................................  Pabelli
305984: ..................................................  Nleucogenys
375531: ..-...........................G...................  Mmulatta
328118: ..............................G...................  Panubis
365360: C..C....A...........................A..A..........  Cjacchus
```

```
                            LINE1
        ------------------------------------------------->
                12026     12036     12046     12056     12066
                    :         :         :         :         :
 12017: AATGTTAATGGATCACAAAAAGCATTAACTATAAAGAAAAATATTAATAA   Hsapiens
331594: .........................................G........   Ptroglodytes
349457: ...............T.........................G........   Ggorilla
381393: ...............G.........................G........   Pabelli
306034: ...........................G.............G........   Nleucogenys
375580: .........................................G.AC.....   Mmulatta
328168: ...........................G.............G.AC.....   Panubis
365410: ........CA..........T....C.G.............G........   Cjacchus

                            LINE1
        ------------------------------------------------->
                12076     12086     12096     12102     12112
                    :         :         :         :         :
 12067: ACTGATTTCATTAAAATTAAGAACCTCCAGTCATC----AAAAAAATCTC   Hsapiens
331644: .....C.....................TG......----.T.........   Ptroglodytes
349507: .....C..........C..........TG......----...........   Ggorilla
381443: .....C..T...........A......TG......----...........   Pabelli
306084: .....C......G..............TG......---A........AA.   Nleucogenys
375630: .....C.....................TG..G...----.......A...   Mmulatta
328218: .....C.....................TG......---A.......A...   Panubis
365460: .....C..............A......TG.....AAAGA.......A...   Cjacchus

                      LINE1                     LTR
        ----------------------------------------->------->
                12122     12132     12142     12152     12162
                    :         :         :         :         :
 12113: CTTTAAGAAAAGAAAAAGACATACCACAGAGTGGTAGAAAATATTTGTAG   Hsapiens
331690: ..................................................   Ptroglodytes
349553: ..................................................   Ggorilla
381489: ............................................A.....   Pabelli
306131: ..................................................   Nleucogenys
375676: ........G.............G....................C......   Mmulatta
328265: .C......G.............G....................C......   Panubis
365510: ...........TG...GT....G......................G.C..   Cjacchus

                            LTR
        ------------------------------------------------->
                12172     12182     12192     12202     12212
                    :         :         :         :         :
 12163: AGCTCCCAGATAGCTTCTAGATGGTGGAGGTTCCAGGAGGTTGGCATGCC   Hsapiens
331740: ..................................................   Ptroglodytes
349603: G.................................................   Ggorilla
381539: ..........A..................A............CA......   Pabelli
306181: ....................G.............................   Nleucogenys
375726: ..................................................   Mmulatta
328315: ..................................................   Panubis
365560: ..T.T......................G.............CA.......   Cjacchus

                   LTR                         Simple
        -------------------------------->  ----------------->
                12222     12232     12242     12252     12262
                    :         :         :         :         :
 12213: CAGGGAGGACATGGAAGCTCCGTGACCCTTCCTATAAATAAATAAATAAA   Hsapiens
331790: ........................................--------..   Ptroglodytes
349653: ....................................------------..   Ggorilla
381589: ..........................A.....................G.   Pabelli
306231: ...........C........T.............................   Nleucogenys
375776: .....................A..........A.......--------..   Mmulatta
328365: .....................A..........G.....C...C...C...   Panubis
365610: ....................T...........------------------   Cjacchus
```

```
                               Simple                         LINE1
       ------------------------------------------------->---------->
                 12272                12285     12295     12305
                     :                    :         :         :
 12263: TAAATAAATAAATAAATA-------AATAAATAAATAATTTGCAATAAAT  Hsapiens
331832: ..C...............-------------------...G.........  Ptroglodytes
349691: ..................-------------------....C........  Ggorilla
381639: ..G...............-------C..G......AT.............  Pabelli
306281: ..................ATAACTA..........AT.............  Nleucogenys
375818: .................C---------------..AT.............  Mmulatta
328415: C...C.............----------------------------....  Panubis
365642: -.........G.......--------------------............  Cjacchus
```

```
                                  LINE1
       ------------------------------------------------------------>
                 12315     12325     12335     12345     12355
                     :         :         :         :         :
 12306: ATTTCTGAGAAAGATCTATTACCCAGAATGTATCAAGTAATTCCACAAAT  Hsapiens
331863: ........A..............................C..........  Ptroglodytes
349722: .......................................C..........  Ggorilla
381682: .....................T.................C..........  Pabelli
306331: .....................T...............A.C......G...  Nleucogenys
375853: ........A.........C..T..G............C.C..........  Mmulatta
328437: .......GA.........C..T...............C.C..........  Panubis
365671: ................C....T.A...............C.......T.G  Cjacchus
```

```
                                  LINE1
       ------------------------------------------------------------>
                 12365     12375     12385     12395     12405
                     :         :         :         :         :
 12356: CAATAATAAAAGAAAAGATAGCCCAGTAGAAAATGTCAAGCAGGAACTTC  Hsapiens
331913: ..................................................  Ptroglodytes
349772: ..................................................  Ggorilla
381732: ..................................................  Pabelli
306381: ..................................................  Nleucogenys
375903: ............T...A......................A..........  Mmulatta
328487: ............T..........................A..........  Panubis
365721: .....................T.................A..T......  Cjacchus
```

```
                                  LINE1
       ------------------------------------------------------------>
                 12415     12425     12435     12445     12455
                     :         :         :         :         :
 12406: ACAAACAATTTGAACAGAAACTTCACAAATGAGAAAATCCAAATGTCCAA  Hsapiens
331963: ..............T...................................  Ptroglodytes
349822: ..................................................  Ggorilla
381782: ..................................................  Pabelli
306431: ..................................................  Nleucogenys
375953: .....A...........G.............................G..  Mmulatta
328537: .....-............................................  Panubis
365771: .....-...........G...........G....................  Cjacchus
```

```
                                  LINE1
       ------------------------------------------------------------>
                 12465     12475     12485     12495     12505
                     :         :         :         :         :
 12456: AAGCTTATGAAAAAATTCTCAACATCATTAGTCACCAGAAAAATGCAAAT  Hsapiens
332013: ..................................................  Ptroglodytes
349872: ..-...............................................  Ggorilla
381832: .......................................G....  Pabelli
306481: .................A.....................G..........  Nleucogenys
376003: .......................................G..........  Mmulatta
328586: ...................T...................G..........  Panubis
365820: .......................................G..........  Cjacchus
```

```
                                   LINE1
             12515     12525     12535     12545     12555
  12506: TAAGACCACAGTAAGGTACCACTGCACACCCATTAAAGTGAGTATTTTTA Hsapiens
 332063: .................................................. Ptroglodytes
 349921: ...........................G...................... Ggorilla
 306531: .................................................. Nleucogenys
 376053: ..............A...........................C....... Mmulatta
 328636: ..........................................C....... Panubis
 365870: .............T..C.....G......T.....G......C....... Cjacchus

                                   LINE1
             12565     12575     12585     12595     12605
  12556: GTAAGACTGGTAATACAAGTGTTGCTGAGGATGTAGAGGAACTGGAACTC Hsapiens
 332113: .................................................. Ptroglodytes
 349971: .................................................. Ggorilla
 306581: ..................A............................... Nleucogenys
 376103: .................................................. Mmulatta
 328686: .................G................................ Panubis
 365920: .........A.........CA...........A........T.A....A. Cjacchus

                                   LINE1
             12615     12625     12635     12645     12655
  12606: TCATATATTGCTGATAGAAGTATAAATTACAGCAACTACTTTGGACTACT Hsapiens
 332163: .................................................. Ptroglodytes
 350021: ...............G........................C......... Ggorilla
 306631: .....C............................................ Nleucogenys
 376153: .......C.......................AG................. Mmulatta
 328736: .......C.......................A.................. Panubis
 365970: .T...G........A..............GCA...............T.. Cjacchus

                                   LINE1
             12665     12675     12685     12695     12705
  12656: CTTGAGAAGTATTTTCTAGAGCCAACCTTAGGTCTATGTTATAATCCAGT Hsapiens
 332213: .................................................. Ptroglodytes
 350071: .................................................. Ggorilla
 306681: .................................................. Nleucogenys
 376203: ............C..G.....T.............C.............C Mmulatta
 328786: ............C..G...................C.............C Panubis
 366020: ............C.....T......A....A..G.G.............C Cjacchus

             LINE1
             12715     12725     12735     12745     12755
  12706: TGTTTCAGTTCTCAATAGAAATGCGTACCAAATTAGCTGCATATTCTGCT Hsapiens
 332263: .................................................. Ptroglodytes
 350121: ..................................G.............T. Ggorilla
 306731: .................................................. Nleucogenys
 376253: .A................................................ Mmulatta
 328836: ........................A......................... Panubis
 366070: ..........T....CT.....ATAA........T..........G.... Cjacchus
```

```
                  12765      12774      12784      12794      12804
                      :          :          :          :          :
 12756: TCTTTCATGGTTTGCA-CCTGTATACTGAGTGTCAAATTTCAGAAATGGG  Hsapiens
332313: ................-.................................  Ptroglodytes
350171: ................-.................................  Ggorilla
306781: .....A..........-.................................  Nleucogenys
376303: .A..............C.................................  Mmulatta
328886: .A..............C.................................  Panubis
366120: .A...........T..-..------.C...G...................  Cjacchus
```

```
                                                         LINE1
                                                          -->
                  12813      12823      12833      12843      12853
                      :          :          :          :          :
 12805: AAGAA-CTAGTCTCCTTAAAATTCAGTATGTGCAAGCAAAATGTTGACTT  Hsapiens
332362: .....C....................................C.......  Ptroglodytes
350220: .....C....................................C.......  Ggorilla
306830: .....C....................................C.......  Nleucogenys
376353: .....C....................................C.......  Mmulatta
328936: .....C....................................C.......  Panubis
366163: .....C........TG..........C...CAG.......CAC.......  Cjacchus
```

```
                                  LINE1
        ------------------------------------------------->
                  12863      12871      12881      12891      12901
                      :          :          :          :          :
 12854: TCTTTTTTTAAAA--ATTATACTTTAAGTTCTGAGATACATATGCAGAAT  Hsapiens
332412: .........T...--...................................  Ptroglodytes
350270: .........TT..--...................................  Ggorilla
306880: .........C...--.......................T...........  Nleucogenys
376403: .........T--.--.........C................G..G.....  Mmulatta
328986: .T........TT.TT.........C................G....C...  Panubis
366213: .T.....AA....--.....-----........G.......G.A......  Cjacchus
```

```
                                  LINE1
        ------------------------------------------------->
                  12911      12921      12931      12941      12951
                      :          :          :          :          :
 12902: GTGTAGGTTTGTTACATAGGTATACATGTGCCATGGTGGTTTGCTGCACC  Hsapiens
332460: ..................................................  Ptroglodytes
350318: .................................C................  Ggorilla
306928: ..................................................  Nleucogenys
376449: ..........................C.................C.....  Mmulatta
329036: .......................C..........................  Panubis
366256: ...C.........T..................G.................  Cjacchus
```

```
                                  LINE1
        ------------------------------------------------->
                  12961      12971      12981      12991      13001
                      :          :          :          :          :
 12952: CCTCAATCCGTCATCTAGGTTTTAAGCCCCACATGCATTAGGTATCTCTC  Hsapiens
332510: ......C.................................A.........  Ptroglodytes
350368: ......C...........................................  Ggorilla
306978: ......C.T...............................AA........  Nleucogenys
376499: .A....C.T.........................................  Mmulatta
329086: .A....C.T.........................................  Panubis
366306: .A....C..A..............                            Cjacchus
```

```
                             LINE1
        ------------------------------------------------->
                 13011     13021     13031     13041     13051
                     :         :         :         :         :
 13002: CCAAAGTTATCCCTCCCCTTGCTCCCCACCCCCCAAACAGGCCCCAGTGT  Hsapiens
332560: ..................................-.......T...A..A  Ptroglodytes
350418: ........................G....A....-...........A...  Ggorilla
307028: ..............T.........T.........-...............  Nleucogenys
376549: .T........G.T.......A.C..........--......TG.......  Mmulatta
329136: .T..................A.C.T......T.--..G............  Panubis
```

```
                             LINE1
        ------------------------------------------------->
                 13061     13071     13081     13091     13101
                     :         :         :         :         :
 13052: GTGGTGTTCCCCTCCCTGTGCCCATGTGTTCTTATTGTTCAACTCCCACT  Hsapiens
332609: ........................G.........................  Ptroglodytes
350467: ..................................................  Ggorilla
307077: ........................C.........................  Nleucogenys
376597: ...................A........C...C........C....T...  Mmulatta
329184: ................................C........C.C......  Panubis
```

```
                             LINE1
        ------------------------------------------------->
                 13111     13121     13131     13141     13151
                     :         :         :         :         :
 13102: TGTGAGTGAGAACATGGCGGTGTTTGCTTTTCTGTTCCTGTGTTAGTTTG  Hsapiens
332659: ..................................A...............  Ptroglodytes
350517: ..................C...............................  Ggorilla
307127: .A................................................  Nleucogenys
376647: .A.A.....A................G...................A...  Mmulatta
329234: .A.A.....A........A.......G.......................  Panubis
```

```
                             LINE1
        ------------------------------------------------->
                 13161     13171     13181     13191     13201
                     :         :         :         :         :
 13152: CTGAGAATGATGGTTTCCAGCTTCATCCACGTCCCTGCAAAGGACATGAA  Hsapiens
332709: ..................................................  Ptroglodytes
350567: ............................................A.....  Ggorilla
307177: .............A................T...................  Nleucogenys
376697: ................A.................T...............  Mmulatta
329284: .............................T....T...........A...  Panubis
```

```
                             LINE1
        ------------------------------------------------->
                 13211     13221     13231     13241     13251
                     :         :         :         :         :
 13202: CTCATTCTTTTTTATGGCTGCATAGTATTCCACAGTGTATATGTGCCACA  Hsapiens
332759: ..................................................  Ptroglodytes
350617: ..................................................  Ggorilla
382661:          .......................TG................  Pabelli
307227: ................................TG................  Nleucogenys
376747: ......AC........................TGA...............  Mmulatta
329334: .......C........................TGA...............  Panubis
```

```
                          LINE1
         ------------------------------------------------->
                   13261     13271     13281     13291     13301
13252:   TTTTCTTTATCCAGTCTATTATTGGTGGGCATTTGGGTTGGTTTCAAGTC   Hsapiens
332809:  ..................................C...............   Ptroglodytes
350667:  ..................................................   Ggorilla
382702:  ...........................................C......   Pabelli
307277:  ........G..............T...................C...T..   Nleucogenys
376797:  ........................A..................C...A..   Mmulatta
329384:  ........................A..................C......   Panubis
```

```
                          LINE1
         ------------------------------------------------->
                   13311     13321     13331     13336     13346
13302:   TTTGCTATTGTAAATAGTGCTTCAATAAACATAC-----GTGTGCATGTG   Hsapiens
332859:  ..................................ATTATA..........   Ptroglodytes
350717:  ..................................-----...........   Ggorilla
382752:  .....................G............-----A..........   Pabelli
307327:  ..................A..G........C...-----...........   Nleucogenys
376847:  .....................G............-----.......A.CA   Mmulatta
329434:  .....................G............-----A........CA   Panubis
```

```
                          LINE1
         ------------------------------------------------->
                   13356     13366     13376     13386     13396
13347:   TCTTTATAATAGAATGATTTATAATCCTTTGGGTATATACTCAGTAATGG   Hsapiens
332909:  ........G.........................................   Ptroglodytes
350762:  ........G.........................................   Ggorilla
382797:  ........G.........................................   Pabelli
307372:  ........G.........................................   Nleucogenys
376892:  ........G.....C......C...A.......CG............C..   Mmulatta
329479:  ........G........C...C...A.......C................   Panubis
```

```
                          LINE1
         ------------------------------------------------->
                   13406     13416     13426     13436     13446
13397:   GATTTCTGGGTCAAATGGTATTTCTAGTTCTAGATCCTTGAAGAATCACT   Hsapiens
332959:  .T................................................   Ptroglodytes
350812:  ..................................................   Ggorilla
382847:  ..............G...................................   Pabelli
307422:  ..................................................   Nleucogenys
376942:  .........................G...............G.......C   Mmulatta
329529:  .........................G...............G.......C   Panubis
```

```
                          LINE1
         ------------------------------------------------->
                   13456     13466     13476     13486     13496
13447:   ACACTGTCTTCCACAATGGTTGAACTAATTTATACTCCCACCAATAATGT   Hsapiens
333009:  ..................................................   Ptroglodytes
350862:  ..................................................   Ggorilla
382897:  G...............................C...........C.GC..   Pabelli
307472:  G...............A...........A...C.............G...   Nleucogenys
376992:  ................................C.............G...   Mmulatta
329579:  .................A..............C.........T...G...   Panubis
```

```
                                      LINE1
                 -------------------------------------------------->
                       13506     13516     13526     13536     13546
   13497: AAAAGCATTCCTATTTCTCCACATCCTCTCCAGCATCAGTTGTTTCCTGA  Hsapiens
  333059: ..................................................  Ptroglodytes
  350912: ................................................A.  Ggorilla
  382947: ......G.....................C.....................  Pabelli
  307522: ......G................C..........................  Nleucogenys
  377042: ......                                              Mmulatta
  329629: ..............................T...................  Panubis

                                      LINE1
                 -------------------------------------------------->
                       13556     13566     13576     13586     13596
   13547: CTTTTTAATGATCACCATTCTAACTGGCGTGAGATGGTATCTCATTGTGA  Hsapiens
  333109: ..................................................  Ptroglodytes
  350962: ..................................................  Ggorilla
  382997: .....................T.A..........................  Pabelli
  307572: ....................C..............A..............  Nleucogenys
  377126:                                       ......C....G  Mmulatta
  329679: ...........................TA.....C.........C..A.G  Panubis

                                      LINE1
                 -------------------------------------------------->
                       13606     13616     13626     13636     13645
   13597: TTTTGATTTGCATTTCTCTAATGACCAGTGATGATGAGCTTTTTTT-TCA  Hsapiens
  333159: .............................A................-...  Ptroglodytes
  351012: ..............................................-...  Ggorilla
  383047: ..............................................T...  Pabelli
  307622: ......................................G.......-...  Nleucogenys
  377138: ..............................G.A......A.....--..  Mmulatta
  329729: .......................................A.....--..  Panubis

                                      LINE1
                 -------------------------------------------------->
                       13655     13665     13675     13685     13695
   13646: TGTTTGTTGGCTGCATACAAAAATGTCTTGTTTTGAGAAGTGTCTGTTCG  Hsapiens
  333208: ..................................................  Ptroglodytes
  351061: ....C.............................................  Ggorilla
  383097: ..................................................  Pabelli
  307671: ................................................T.  Nleucogenys
  377186: ...........C.................C..............C....A  Mmulatta
  329777: ...........G.................C...................A  Panubis

                                      LINE1
                 -------------------------------------------------->
                       13705     13715     13725     13735     13745
   13696: TATCCTTTGCCCACTTTATAATGGGGTTGTTTGTTACTGTTTTGGAAATC  Hsapiens
  333258: .................G.G................T...C.........  Ptroglodytes
  351111: .................G.G................T...C.........  Ggorilla
  383147: ..G..............G.G................T...C........T  Pabelli
  307721: .................G.G................T...C........T  Nleucogenys
  377236: ..........T......T.G...........----.T...C..AT....T  Mmulatta
  329827: ..........T......T.G...........----.T...C...T...--  Panubis
```

```
                    LINE1
        ------------------------------->
             13755     13765     13775     13785     13795
                 :         :         :         :         :
 13746: TGTTTAAGTTCCCTGTACAGTCTAGATATTAGACCTTTGTCAAGACTTCC  Hsapiens
333308: ............T....G.T..............................  Ptroglodytes
351161: ............T....G.T........C.....................  Ggorilla
383197: ............T....G.T..............................  Pabelli
307771: ............T....G.T..............................  Nleucogenys
377282: ............T....G.T.........C..........A.........  Mmulatta
329871: --..........T....G.T.........C....................  Panubis

             13805     13815     13825     13835     13845
                 :         :         :         :         :
 13796: AAGACTATTGACATGACTGTGAGTCTAGAAAATTGATTAAGAAACCTCAG  Hsapiens
333358: ..................................................  Ptroglodytes
351211: ..................................................  Ggorilla
383247: ..................................................  Pabelli
307821: ..................................................  Nleucogenys
377332: ..................................................  Mmulatta
329919: .......C..........................................  Panubis

             13855     13865     13875     13885     13895
                 :         :         :         :         :
 13846: TAGTGTCTCTGTTCCACATCCTATACACTTTCTTCATGAAGACAGTGTAC  Hsapiens
333408: ................G...........C.....................  Ptroglodytes
351261: ................G.................................  Ggorilla
383297: ........T.......G.................................  Pabelli
307871: ........T.......G.................................  Nleucogenys
377382: .G......T.......G........T.T......................  Mmulatta
329969: .G......T.......G..........T......................  Panubis

             13905     13915     13925     13935     13945
                 :         :         :         :         :
 13896: ATTGAAAGCCTAGAAGGTGGATATGAAGAATCAAGGAAGTAAGTAACCCT  Hsapiens
333458: ..................................................  Ptroglodytes
351311: ..................................................  Ggorilla
383347: ..................................................  Pabelli
307921: ...........G....A.................................  Nleucogenys
377432: ..GA..............................................  Mmulatta
330019: ..G...............A...............................  Panubis

                                                LINE1
                                              ------->
             13955     13965     13975     13985     13995
                 :         :         :         :         :
 13946: CAAATATTATTCCTAAATTTTGTCTCCTGCATTTCAATTTTAAAAATTAA  Hsapiens
333508: ..................................................  Ptroglodytes
351361: ..................................................  Ggorilla
383397: ...............................A.........T........  Pabelli
307971: ................G.............................C...  Nleucogenys
377482: ..................A...............................  Mmulatta
330069: ..................A...............................  Panubis
```

```
                               LINE1
         ------------------------------------------------------->
                  14005     14015     14025     14035     14045
13996: GCAAATAATAAATATACATTAATAAGGAATTATTTATATAAATCCTGCTG  Hsapiens
333558: ..................................................  Ptroglodytes
351411: ...................................G..............  Ggorilla
383447: .......................................G......T...  Pabelli
308021: ..................................................  Nleucogenys
377532: ..................................................  Mmulatta
330119: ..................................................  Panubis
```

```
                               LINE1
         ------------------------------------------------------->
                  14055     14065     14075     14085     14095
14046: TATCCTGTAAATGAATACAGTACAGCGCTTAATAATTATTTTATAGAGAA  Hsapiens
333608: ..........................A.......................  Ptroglodytes
351461: .........................T........................  Ggorilla
383497: ...........C..............A.................-.....  Pabelli
308071: ..C.......................A.......................  Nleucogenys
377582: .....CA...................A...............G.G.....  Mmulatta
330169: .....CA...................A...............G.G.....  Panubis
```

```
                               LINE1
         ------------------------------------------------------->
                  14105     14115     14125     14135     14145
14096: TGCTTATTGAGAGAGATATGCTCAGAATATGTTAAATACAAAAAGAAACT  Hsapiens
333658: ..................................................  Ptroglodytes
351511: .......................................T..........  Ggorilla
383546: ...............T..................................  Pabelli
308121: ..........A.........................C............-  Nleucogenys
377632: ......................A.........................T.  Mmulatta
330219: ......................A.........................T.  Panubis
```

```
                               LINE1
         ------------------------------------------------------->
                  14155     14165     14175     14185     14195
14146: TAACATGGTATATATAATATGATCCTTTCTAAAATACAAATTTAGATATA  Hsapiens
333708: ..................................................  Ptroglodytes
351561: ..................................................  Ggorilla
383596: ..................................................  Pabelli
308170: -...........-.....................................  Nleucogenys
377682: .............G....................................  Mmulatta
330269: .............G....................................  Panubis
```

```
                 LINE1
         --------------------->
                  14205     14215     14225     14235     14245
14196: TGTGAATATACACATAGGAAAATTCTGGGAGAATATTTGCATAATTTTAA  Hsapiens
333758: ..................................................  Ptroglodytes
351611: ..................................................  Ggorilla
383646: ............................A.....................  Pabelli
308218: ............................A.....................  Nleucogenys
377732: ............................A.....................  Mmulatta
330319: ............................A.....................  Panubis
```

```
                  14255     14265     14275     14285     14295
                      :         :         :         :         :
 14246: CAAAGTTTAGAGTTGAGGGAAGAATTATTAATAAGCTATTTCTTTGAGGT  Hsapiens
333808: ..................................................  Ptroglodytes
351661: ..................................................  Ggorilla
383696: ..................................................  Pabelli
308268: ..................................................  Nleucogenys
377782: ...............................C..............T...  Mmulatta
330369: ...............................C..............T...  Panubis

                  14305     14315     14325     14335     14345
                      :         :         :         :         :
 14296: TTTCTGTATTTTTACATGTTTATATCACTGTATAAATTTTTGTAAATAGG  Hsapiens
333858: ........................C.........................  Ptroglodytes
351711: ........................C.........................  Ggorilla
383746: ............C.....................................  Pabelli
308318: ........C...............C...........C............T  Nleucogenys
377832: .......................GC................A.......A  Mmulatta
330419: .......................GC................A.......A  Panubis

                  14355     14365     14375     14385     14395
                      :         :         :         :         :
 14346: CCTCTTTATATTTTCAATGAAATTAAGAGTGTAGAACCTATTCCATTAAT  Hsapiens
333908: ..................................................  Ptroglodytes
351761: G...............G.................................  Ggorilla
383796: ................................G...........T.....  Pabelli
308368: ..CT.......................................T......  Nleucogenys
377882: .............................................C....  Mmulatta
330469: .............................................C....  Panubis

                  14405     14415     14425     14435     14445
                      :         :         :         :         :
 14396: TATTGTTGGCATTTTCTCCCAGTATTTTCAGAGCATAGCTTCTCACTTTA  Hsapiens
333958: ..................................................  Ptroglodytes
351811: ..................................................  Ggorilla
383846: ..................................................  Pabelli
308418: ..................................................  Nleucogenys
377932: .........T.....T................A........T........  Mmulatta
330519: .........T.....T................A........T........  Panubis
 13765:           ..C....T.......G.....G.TT.C...            Cjacchus

                  14455     14465     14475     14485     14495
                      :         :         :         :         :
 14446: GAAATATTAAAACTGAGGTGAAGAAAGCTTTGGTCTGGGAAAATAATTAG  Hsapiens
334008: ..................................................  Ptroglodytes
351861: ..................................................  Ggorilla
383896: ...........................................C......  Pabelli
308468: ..................................................  Nleucogenys
377982: ....................C.A.......CA..........G...G...  Mmulatta
330569: ....................C.A.......CA..........G...G...  Panubis

                  14505     14514     14524     14534     14544
                      :         :         :         :         :
 14496: CCATCAGTAAAAAA-TATTATATCTAAAACTGGTCAACAGATAGTAGATG  Hsapiens
334058: ...........C..-...................................  Ptroglodytes
351911: ...........C..-...................................  Ggorilla
383946: ..............-...................................  Pabelli
308518: ..............-...................................  Nleucogenys
378032: ..............A...................................  Mmulatta
330619: ..............A...................................  Panubis
```

```
              14554     14564     14574     14584     14594
                  :         :         :         :         :
 14545: TAGAGTAGATGTACATTCTTGATCATCTAAGAATGAAAAATGAAGCCAGC  Hsapiens
334107: ..................................................  Ptroglodytes
351960: ..................................................  Ggorilla
383995: ..................C.T.............................  Pabelli
308567: ......................C...........................  Nleucogenys
378082: .............T....................................  Mmulatta
330669: .............T....................................  Panubis

              14604     14614     14624     14634     14644
                  :         :         :         :         :
 14595: AGAATAGCAATTTGGCCAAATAGATAACACAGCATAATTATAGAGCAAAA  Hsapiens
334157: .......................G..........................  Ptroglodytes
352010: .........G..........G.............................  Ggorilla
384045: ..........................G.......................  Pabelli
308617: ..................................................  Nleucogenys
378132: .A...............T....----.......G....C...........  Mmulatta
330719: .A...............T....----.......G................  Panubis

                                LINE1
                  ---------------------------------------->
              14654     14664               14678     14687
                  :         :                   :         :
 14645: ATAGAACCTGAAGTTTGAGGGCAAGAG------GAAAAGCAGAA-TTAGC  Hsapiens
334207: ...........................------...........-.....  Ptroglodytes
352060: ...........................------...........-.....  Ggorilla
384095: ..........................A------...........-.....  Pabelli
308667: .C.........................------...........T.....  Nleucogenys
378178: .C...T..............A......TTTGAG..G........-.....  Mmulatta
330765: .C...T..............A......TTTGAG..G........-.....  Panubis

                           LINE1
        ------------------------------------------------->
              14697     14707     14717     14727     14737
                  :         :         :         :         :
 14688: ATTGTCTCAGAGAATCTCCTCAAAGGTATTTATTAACTACAAAGTTGGGG  Hsapiens
334250: ................................................T.  Ptroglodytes
352103: .................................................A  Ggorilla
384138: G.................................................  Pabelli
308711: ..................................................  Nleucogenys
378227: ............................C.....................  Mmulatta
330814: ............................C.....................  Panubis

                           LINE1
        ------------------------------------------------->
              14747     14757     14767     14777     14787
                  :         :         :         :         :
 14738: GGAAGTAACTTGGCACTGGAGAAATCAGCAATCATCAAACTGACCAAGTG  Hsapiens
334300: ..................................................  Ptroglodytes
352153: ...............T..................................  Ggorilla
384188: ...............T.....C............................  Pabelli
308761: ...............T..................................  Nleucogenys
378277: .-.............T.........................A........  Mmulatta
330864: .-.............T.........................A.T......  Panubis
```

LINE1

```
             14797     14807     14817     14827     14837
 14788: ATCGAGTTAAACATCACCCACCATGAGACACACCAACATTGTAAATACCT Hsapiens
334350: ......G........................................... Ptroglodytes
352203: ......G........................................... Ggorilla
384238: ...A..G.......................-.......A........... Pabelli
308811: ..T...G........................................... Nleucogenys
378326: ...TT.G..............A..................A......... Mmulatta
330913: ...TT.G..............A..................A......... Panubis
```

LINE1

```
             14847     14857     14867     14877     14887
 14838: TGATGCGGTGCACTGAGGACACAATATTATGTCCTGAGTTTTTACCAAAA Hsapiens
334400: .................................................. Ptroglodytes
352253: .................................................. Ggorilla
384287: ...........................................G...... Pabelli
308861: ...........................................G...... Nleucogenys
378376: ......T.............................C......G...... Mmulatta
330963: ......T.............................C......G...... Panubis
```

LINE1

```
             14897     14907     14917     14927     14937
 14888: ATGCATACCCTCATTTATAATTGGATAAGTCAATACTAATGGACAATCTA Hsapiens
334450: .................................................. Ptroglodytes
352303: .................................................. Ggorilla
384337: .....C............................................ Pabelli
308911: ........T................C..T..................... Nleucogenys
378426: .................................................. Mmulatta
331013: .................................................. Panubis
```

LINE1

```
             14947     14957     14967     14977     14987
 14938: TAAAGTGCCTAATCAGTACTCTTCAGACTTGTCAAGGCGACAAAACACAA Hsapiens
334500: .....................................T............ Ptroglodytes
352353: .....................................T............ Ggorilla
384387: ............................C........T............ Pabelli
308961: ............................C........T............ Nleucogenys
378476: C...........................C.....G..T............ Mmulatta
331063: C...........................C........T............ Panubis
```

LINE1

```
             14997     15007     15017     15027     15037
 14988: AGACCAAGGAACTGTTAGAAAATGGAGGAGACTCAGAAGAAATAGTAACT Hsapiens
334550: .................................................. Ptroglodytes
352403: .................................................. Ggorilla
384437: ............C..................................... Pabelli
309011: .................................................. Nleucogenys
378526: ...............C.................................. Mmulatta
331113: .......A.......C.................................. Panubis
```

```
                         LINE1
        ------------------------------------------------->
                 15047     15057     15067     15077     15087
                     :         :         :         :         :
 15038: AAATGTAACGGGAAATTCTGGGTAAGATCCCAGAACCACAAAGGCGGCAT  Hsapiens
334600: ......................................G...........  Ptroglodytes
352453: ......................................G...........  Ggorilla
384487: .........A...................T........G......A....  Pabelli
309061: ......................................G...........  Nleucogenys
378576: .........C............................G.....T.....  Mmulatta
331163: .........C............................G.....T.....  Panubis
```

```
                         LINE1
        ------------------------------------------------->
                 15097     15107     15117     15127     15137
                     :         :         :         :         :
 15088: TAGTTGGTGGGTAGGGGGCACAAATGGGTACGATTCAAATAAGGTGTATA  Hsapiens
334650: C..............................................T..  Ptroglodytes
352503: ....................T........................-----  Ggorilla
384537: ...............................................T..  Pabelli
309111: ...............................A.............-----  Nleucogenys
378626: .....A.A.......................A...............T..  Mmulatta
331213: .....A.A.......................A...............T..  Panubis
```

```
                         LINE1
        ------------------------------------------------->
                 15147     15157     15167     15177     15187
                     :         :         :         :         :
 15138: GTTAAGTTAATACAGTTGTACCAATAGTATTTTTTACTCTAGATATTTGT  Hsapiens
334700: .......................G..........................  Ptroglodytes
352548: ..................................................  Ggorilla
384587: ..................................................  Pabelli
309156: ..................................................  Nleucogenys
378676: ...TCA.......................C....................  Mmulatta
331263: ...TCA.......................C....................  Panubis
```

```
                         LINE1
        ------------------------------------------------->
                 15197     15207     15217     15227     15237
                     :         :         :         :         :
 15188: ACTATGGTTATGTAAGATGTTAATGTTTTGGTTAATGGGCTGAAAGATAT  Hsapiens
334750: ..................................................  Ptroglodytes
352598: ..................................................  Ggorilla
384637: ..................................................  Pabelli
309206: ...........................G......................  Nleucogenys
378726: ..................................................  Mmulatta
331313: ..................................................  Panubis
```

```
                         LINE1
        ------------------------------------------------->
                 15247     15257     15267     15277     15287
                     :         :         :         :         :
 15238: AAGGCCACACTTCATACCATGCTTGCTTTTTTGTACGACAAAGACTCTTC  Hsapiens
334800: .............G.......T............................  Ptroglodytes
352648: .............G.......T............................  Ggorilla
384687: ................T....T..........A...........T.....  Pabelli
309256: .............G.......T..........A...A.......T.....  Nleucogenys
378776: ...A.........G.......T..........A...........T.....  Mmulatta
331363: ...A.........G.......T..........A...........T.....  Panubis
```

```
             LINE1
        ──────────►
                 15297     15307     15317     15327     15337
                     :         :         :         :         :
 15288: AAATTAAAATAGGCTTTTAAAAATGATGTGCTCAGAACATGAAAAAATAA  Hsapiens
334850: .....-......C.....................................  Ptroglodytes
352698: ............C.....................................  Ggorilla
384737: .......................C..........................  Pabelli
309306: ..................................................  Nleucogenys
378826: .........C....................A...................  Mmulatta
331413: .........C.......A................................  Panubis

                                     LINE2
                      ────────────────────────────────────►
                 15347     15357     15366     15376     15386
                     :         :         :         :         :
 15338: AGCGAATAAAATTTCAAATATC-TCGGGCCCCTACTGTAGGCCAGATACT  Hsapiens
334899: ......................T.T.........................  Ptroglodytes
352748: ......................T.T.........................  Ggorilla
384787: ......................T.T.A............A..........  Pabelli
309356: ....................C.T.T.A............A..........  Nleucogenys
378876: ...A..............C...T.T.A............A..........  Mmulatta
331463: ...A..............C...T.T.A............A..........  Panubis

               LINE2
        ─────────────►
                 15396     15406     15416     15426     15436
                     :         :         :         :         :
 15387: GTTCTAGATGTGGTCATGAATGAGATGGCCAAATTTCCTACTCTGTAATG  Hsapiens
334949: ..........CA............G.........................  Ptroglodytes
352798: ..........C.............G.........................  Ggorilla
384837: ..........CA............G.....................G...  Pabelli
309406: ..........C.............G.....................G...  Nleucogenys
378926: ..........A........G....GC............C......CG...  Mmulatta
331513: ..........A........G....GC............C......CG...  Panubis

                 15446     15456     15466     15476     15486
                     :         :         :         :         :
 15437: TATACTCCTTTTATAGATTTGGCAAACAATGAATATATAAAGGGGAAAAA  Hsapiens
334999: ........................G.........................  Ptroglodytes
352848: ........................G.........................  Ggorilla
384887: ........................G..........C..............  Pabelli
309456: ........................G....C...C.C..............  Nleucogenys
378976: ..................C.....C..........C.........G...-  Mmulatta
331563: ..................C.....C..........C.........G...-  Panubis

                 15496     15506     15516     15526     15536
                     :         :         :         :         :
 15487: TGGAGCTCTTACATTCATACTGAAAGAAAATTTAATCAGTAGAAATTTGT  Hsapiens
335049: ..................................................  Ptroglodytes
352898: ..................................................  Ggorilla
384937: ..................................................  Pabelli
309506: ..................................................  Nleucogenys
379025: ......A.............................G.............  Mmulatta
331612: ......G........T....................G.............  Panubis
```

```
             15546     15556     15566     15576     15586
 15537: AATGATAGATATTTATAATGCCTAAGTGAAATACCCCACAAACTATTTTA  Hsapiens
335099: ......................................T...........  Ptroglodytes
352948: ......................................T...........  Ggorilla
384987: ........G.............................T..---......  Pabelli
309556: ......................................TG..........  Nleucogenys
379075: ...A.............................G....A...........  Mmulatta
331662: ...A.............................G....A...T.......  Panubis

             15596     15606     15616     15626     15636
 15587: CCAGGAGAATCCAAGATAAGATTTAGAAATTAAATACCATATGCCTATCA  Hsapiens
335149: ..................................................  Ptroglodytes
352998: ..................................................  Ggorilla
385034: .......G.......................................C..  Pabelli
309606: .......G.......................................C..  Nleucogenys
379125: .......G......C..........T................A..C.C..  Mmulatta
331712: .......G......C..........T............G...A....C..  Panubis

             15646     15656     15666     15676     15686
 15637: CATCCTCCTCCCCCTCCTACAACTGAGTCTGCTATCTAGTGTTTGGATGG  Hsapiens
335199: .........A................T.......................  Ptroglodytes
353048: ..................................................  Ggorilla
385084: ............T.....................................  Pabelli
309656: ..................................................  Nleucogenys
379175: ..........T............C..........................  Mmulatta
331762: ..........T............C..........................  Panubis

             15696     15706     15716     15726     15736
 15687: TAAACTGCCATCCATAGTTCACTAGAATCTGTACAAAATCTTTCCACGGG  Hsapiens
335249: ................................................T.  Ptroglodytes
353098: ..............................................T.T.  Ggorilla
385134: ..............................................T...  Pabelli
309706: ..............................................T...  Nleucogenys
379225: ....................C.........................T...  Mmulatta
331812: ....................C.........................T...  Panubis

             15746     15756     15766     15776     15786
 15737: TTTTGACCTGCCAGTTAGCATGTATGAATTAAATATGTTCATTTTCTTAA  Hsapiens
335299: ..................................................  Ptroglodytes
353148: ..................................................  Ggorilla
385184: ............................................C.....  Pabelli
309756: ...............................C............C.....  Nleucogenys
379275: ...................G........................C.....  Mmulatta
331862: ....................C.......................C.....  Panubis

             15796     15806     15816     15826     15836
 15787: TCAGAAATATCAAGTGTTCTCTACAGCTTTCAAATAACATTCAAATTTAT  Hsapiens
335349: ..................................................  Ptroglodytes
353198: ..................................................  Ggorilla
385234: ..................................................  Pabelli
309806: ...............A..................................  Nleucogenys
379325: ........G.........................................  Mmulatta
331912: ........G.........................................  Panubis
366326:                                              .....  Cjacchus
```

```
                  15846     15856     15866     15876     15886
                      :         :         :         :         :
 15837: CGATGTGGGATTCAAATGAGTACACAATCTGGTCTCTGTCTATCGAGTGT  Hsapiens
335399: ..................................................  Ptroglodytes
353248: ..................................................  Ggorilla
385284: ...............................A............A.....  Pabelli
309856: .A..........................................A.....  Nleucogenys
379375: T....C...............G......................A.....  Mmulatta
331962: T...AC...............G......................A.....  Panubis
366331: T..............G....................-.....CGA.....  Cjacchus
```

```
                                         Alu
                              --------------------------->
                  15896     15906     15916     15926     15936
                      :         :         :         :         :
 15887: AAAGAAATAAGAACTCTGGGCTGGGCGCAGTGGCTCATGCCTGTAATCCC  Hsapiens
335449: .....................C......G.....................  Ptroglodytes
353298: .........C...........C......G.........              Ggorilla
385334: ................CA...C....A.G.....................  Pabelli
309906: ................C....C......G..A..................  Nleucogenys
379425: ---..................C............................  Mmulatta
332012: .....................C............................  Panubis
366380: ..............A.....T...............T.............  Cjacchus
```

```
                                 Alu
        ------------------------------------------------->
                  15946     15956     15966     15976     15986
                      :         :         :         :         :
 15937: AGCACTTTGGGAGGACGAAGTGGGCAGATCATCTGAGGTCAGGAGTTCGA  Hsapiens
335499: ........T.........................................  Ptroglodytes
385384: ..............C...G...............................  Pabelli
309956: ..............C...................................  Nleucogenys
379472: ..............C...G...............................  Mmulatta
332062: .............ACT..G...............................  Panubis
366430: .A...A.......TC.A.G.CA............................  Cjacchus
```

```
                                 Alu
        ------------------------------------------------->
                  15996     16006     16016     16026     16036
                      :         :         :         :         :
 15987: GACTAGCCTGGCCAGTATGGTGAAACCTCACCTCTACTAAAATACAAAA  Hsapiens
335549: ..................................................  Ptroglodytes
385434: .............................G....................  Pabelli
310006: .........................T...G....C...............  Nleucogenys
379522: ..............A.............................G.....  Mmulatta
332112: ..............A.............................G.....  Panubis
366480: ...C...T......A............C........T.............  Cjacchus
```

```
                                 Alu
        ------------------------------------------------->
                  16045     16055     16065     16075     16085
                      :         :         :         :         :
 16037: A-AATTAGCCAGGTGTGGTGGCGGGTGTCAGTAACCCCAGCTACTTGGGA  Hsapiens
335599: .A................................................  Ptroglodytes
385484: .-............................A...T...............  Pabelli
310056: .-...................T............T...............  Nleucogenys
379572: .-.........CA.............T.......T...............  Mmulatta
332162: .-........GCA.............T.......T...............  Panubis
366530: .---......TA........AT...C.C.T....T..T.......G....  Cjacchus
```

```
                                    Alu
         ------------------------------------------------->
               16095     16105     16115     16125     16135
  16086: GGCTGAGGCTGGAGAATCGCTTGAACCAGGGAGGTAGATGTTGCAGTGAG  Hsapiens
 335649: .........A.................C......................  Ptroglodytes
 385533: .........A.................C..........G...........  Pabelli
 310105: .........A.................CA.........G...........  Nleucogenys
 379621: .........A........A........TA.....CG..G..C........  Mmulatta
 332211: .........A........A........TA.....C...G..C........  Panubis
 366577: .........A................TCCTA...CG..G......A....  Cjacchus
```

```
                                    Alu
         ------------------------------------------------->
               16145     16154     16164     16174     16184
  16136: CCAAGATCGTGCCATTG-CACTCCAGCCTGGGCAATGAGAGCGAAACTCC  Hsapiens
 335699: .................-.................C......A.......  Ptroglodytes
 385583: T................-................................  Pabelli
 310155: .......T.........C........................A.......  Nleucogenys
 379671: ........                                            Mmulatta
 332261: ........T.---....---------..........A....T........  Panubis
 366627: .......T.....G...-.................C......A...----  Cjacchus
```

```
                                    Alu
         ------------------------------------------------->
               16193     16203     16213
  16185: AGCAAG-AAAGAAAGAAAGAAAGAAAGAAAGAA-----------------  Hsapiens
 335748: ..A...A.....................G...GAGAGAGAGAGAGAAAGA  Ptroglodytes
 354011:    .G.-..G...G...G...G...G...G...-----------------  Ggorilla
 385632: ..G..A-..G.......G.......G.......-----------------  Pabelli
 310205: ..G...-G.....G...G...G...G...G...-----------------  Nleucogenys
 332299: .T...A-...A...A.G.A.G...........G-----------------  Panubis
 366672: ---...-......GA.                                    Cjacchus
```

```
                      Alu                     Simple
         ------------------------------->  ---------------->
                                   16219     16229     16238
  16217: ---------------------------AGAAAGAAAGAAAGAAAGAA-AG  Hsapiens
 335798: AAGGAAGGAAGGAAGGAAGGAAGGAAGG...................-..  Ptroglodytes
 354040: ---------------------------G...G...G...G...G...GG.  Ggorilla
 385664: -----------------------------..GA..............-..  Pabelli
 310237: ---------------------------G...G...G...G...G...-GT  Nleucogenys
 332331: ---------------------------G..G...G...G.A..GA..-..  Panubis
```

```
                                   Simple
         ------------------------------------------------->
               16248     16258     16268     16278     16288
  16239: AAAGAAAGAAAGAAAGAAAGAAAGAAAGAAAGAAAGAAAGGAAGGAAGGA  Hsapiens
 335847: .................................G...G..AG..A...AG  Ptroglodytes
 354063: ........................................A...A...A.  Ggorilla
 385684: .G...G...G--...................C........A...A...A.  Pabelli
 310259: ..G...G...                                          Nleucogenys
 332353: ...AG..A.G.-.............G...........G..A...A...A.  Panubis
```

```
                             Simple
        ---------------------------------------------------->
                   16298                                  16309     16319
                       :                                      :         :
 16289: AGGAAGGAAGGAA-------------------GGAAGGAAGGAAGGAAGG  Hsapiens
335897: .............GGGAAAGAAAGAAAAGAAA....A...A...A.....  Ptroglodytes
354113: ..A...A...A..------------------A.A......A.......A.  Ggorilla
385732: ..A...A...A..--------------------...A...A...A...A.  Pabelli
332402: ..A...A...A..-----------------GAA...A...C...A.....  Panubis

                             Simple
        ---------------------------------------------------->
                   16329     16339     16349                    16363
                       :         :         :                        :
 16320: AAGGAAGGAAGGAAGGAAGGAAGGAAGGAAG-GAGGAAG-----GAGGAA  Hsapiens
335947: ..A...................A...A...A-A..A...CAG--......  Ptroglodytes
354145: ......................A...A...A-A..A...CAG--......  Ggorilla
385762: ..A...-----..T.A...A.GA...A...A-...AG..-----A.A..G  Pabelli
379873:                                                ...  Mmulatta
332435: ...................T.......T...TA..T...TAAGTA..T..  Panubis

                          Simple                        MIR
        ------------------------------------------------>--->
                   16373     16383     16391     16401
                       :         :         :         :
 16364: GGAAGGAGAGAGAAAGGAAGGAAGAAA--AAAGGAAGGAAGGAAGGAA--  Hsapiens
335994: ...................A.......--....A..............--  Ptroglodytes
354192: ...................A.......--....A..............GG  Ggorilla
385801: A...A.......GG....G...                              Pabelli
379876: .......---..G...........G..GG..G.A.G...G........--  Mmulatta
332485: .T.....---..G..........AG..GG..G.A.G...G...G...G--  Panubis

                             MIR
        ---------------------------------------------------->
                                                  16419     16429
                                                      :         :
 16410: ------------------------------GGAGAAAGAGACAGAGAGAA  Hsapiens
336040: --TGAAGGAAGGAAGGAACGAAGAAAGGAA..............T.....  Ptroglodytes
354240: AACGAAGGAAGGAAGGAACGAAGAAAGGAA..............T.....  Ggorilla
379921: --------------------------------.......A.G..G.....  Mmulatta
332530: ------------------------------------------.G.....  Panubis

                             MIR
        ---------------------------------------------------->
                   16437     16447     16457     16467     16477
                       :         :         :         :         :
 16430: A--AAGAATTCTGGAGTTTCTTCACCATTATATTTATTAACTTCCTATGT  Hsapiens
336088: .GG.......G.......................................  Ptroglodytes
354290: .GA.................C.............................  Ggorilla
379939: .GA.G...C......A............................G.....  Mmulatta
332537: .GA.G...C......A............................A.....  Panubis

                             MIR
        ---------------------------------------------------->
                   16487     16497     16507     16517     16527
                       :         :         :         :         :
 16478: GATCTTGGACAAATCTTTCAAATTTGTTGGGTTTTAGATGATTTATGTCT  Hsapiens
336138: ..................................C...............  Ptroglodytes
354340: ..........-.......................C...............  Ggorilla
379989: ....C....G........................................  Mmulatta
332587: ....C....G........................................  Panubis
```

MIR

```
              16537     16547     16557     16567     16577
                  :         :         :         :         :
   16528: AAACAAAGATGCTTAGACCAAATGATCTCAAAGAATTGTCATGGCCATAA  Hsapiens
  336188: ...................................C..............  Ptroglodytes
  354389: ...................................C..............  Ggorilla
  380039: ..........A........................C..........G...  Mmulatta
  332637: ..........A........................C..........G...  Panubis

              16587     16597     16607     16617     16627
                  :         :         :         :         :
   16578: GTATGATAAGCCCATATCTCTCTGAGCCCTTTTTCTCCTTCCCTAAGATC  Hsapiens
  336238: ..................................................  Ptroglodytes
  354439: ..................................................  Ggorilla
  380089: .........A..T....................A.C..............  Mmulatta
  332687: .........A..T....................A.C..............  Panubis

              16637     16647     16657     16667     16677
                  :         :         :         :         :
   16628: CTCCATTTGGGGTCTGACGCCCTCAACACCATCATCCCCTGTCCTAATGA  Hsapiens
  336288: .................T................................  Ptroglodytes
  354489: .............................................G....  Ggorilla
  380139: ..............C..T.......T....C..-................  Mmulatta
  332737: ..............C..T.......T....C...................  Panubis

              16687     16697     16707     16717     16727
                  :         :         :         :         :
   16678: TGTCCTGCTATGTTTGACCTCCTAATCCTCCTCATGCATCAAAACTAGCT  Hsapiens
  336338: ..................................................  Ptroglodytes
  354539: ...........................T...........T..........  Ggorilla
  380188: ..........C..................A......T........C....  Mmulatta
  332787: .............................A......T........C....  Panubis

              16737     16747     16757     16767     16777
                  :         :         :         :         :
   16728: AAAGTCCTAACCCCTCTACAAAGCCTTTCCTGACAAAAACTTCTTTTTCA  Hsapiens
  336388: ..................................................  Ptroglodytes
  354589: ..................................................  Ggorilla
  380238: .................................T........--......  Mmulatta
  332837: .................................T......A.--......  Panubis

              16787     16797     16807     16817     16827
                  :         :         :         :         :
   16778: TCTAAATTCCTATACTTACCTTGACTATATCGTTTAAACTCAACAGTAAG  Hsapiens
  336438: .................................C.....C...A......  Ptroglodytes
  354639: .................................C.....C...A......  Ggorilla
  380286: ................A..............A.C.....C...A....G.  Mmulatta
  332885: .................................C.....C...A....G.  Panubis

              16837     16847     16857     16867     16877
                  :         :         :         :         :
   16828: AAACATGAATTTGCAAAGAATTCTGACTTGTAAATATTACATAAAATATT  Hsapiens
  336488: ........................T..............T..........  Ptroglodytes
  354689: .......................................T..........  Ggorilla
  380336: ....................C........................C.--.  Mmulatta
  332935: ....................C........................C.--.  Panubis
  366800:                                    ......G........  Cjacchus
 1016927:                                ..T................  Sboliviensis
```

```
                   16887     16897     16907     16917     16927
   16878: TTAAAGGGCTAAGAAAGAACAACTTTTGGATATACAGTTAACCAATTACT  Hsapiens
  336538: .............................T....................  Ptroglodytes
  354739: .............................T....................  Ggorilla
  380384: .......................G.....T....................  Mmulatta
  332983: .............................T....T...............  Panubis
  366815: .............................T..................T.  Cjacchus
 1016946: .......A.....................T..................T.  Sboliviensis

                   16937     16947     16957     16967     16977
   16928: TTAACTTCATTAAAAAATACTATGATACATCAAAGCTAGAAAAATCAGTG  Hsapiens
  336588: ..................................................  Ptroglodytes
  354789: ..................................................  Ggorilla
  380434: ...................................T..............  Mmulatta
  333033: ..................................................  Panubis
  366865: ........................GC.......T................  Cjacchus
 1016996: .........................C.......T................  Sboliviensis

                   16987     16997     17007     17017     17027
   16978: TGTGGCTCCTGATGCCCCTTCAGGAAAATGATACAGCCTGGCGGTTATGT  Hsapiens
  336638: ....................................A....A........  Ptroglodytes
  354839: .........................................T........  Ggorilla
  380484: .........................................T........  Mmulatta
  333083: .........................................T........  Panubis
  366915: ........................G........T......T.C....A.  Cjacchus
 1017046: ........................G........T......T........  Sboliviensis

                                           LTR
                                   ------------------------->
                   17037     17047     17057     17067     17077
   17028: ACCTAGGCTTTGGACTCAAAAGATCTGTAGTAGATGTATTTTTCAAAGAC  Hsapiens
  336688: .................................................T  Ptroglodytes
  354889: .................................................T  Ggorilla
  380534: .......T.........................T...............T  Mmulatta
  333133: .......T.........................T...............T  Panubis
  366965: ........................T..........T.............T  Cjacchus
 1017096: ...........AC.......................T.............T  Sboliviensis

                               LTR
          ------------------------------------------------->
                   17087     17097     17107     17117     17127
   17078: ATTCACAACAAAATTTCCTATCCTACACAGTTTTCTAGAATTCTGCCACT  Hsapiens
  336738: ..................................................  Ptroglodytes
  354939: ..................................................  Ggorilla
  380584: ...T.........G.........---.TG.....................  Mmulatta
  333183: ...T.........G.........---.TG.....................  Panubis
  367015: .............G...TC.....G..TG.........-........T..  Cjacchus
 1017146: ..........G..G....C........TG..................T.C  Sboliviensis
```

```
                                        LTR
           ------------------------------------------------->
                   17137     17147     17157     17167     17177
                       :         :         :         :         :
   17128: TGCCCTTAAGAGTTAGAGTCTAATTAACATCCCCTTCAATCTGAGCAGGT  Hsapiens
  336788: ............................C.....................  Ptroglodytes
  354989: ............................C.....................  Ggorilla
  380631: ............................C.......G.............  Mmulatta
  333230: ............................C.......G.............  Panubis
  367064: ..........................T.C.......G.............  Cjacchus
 1017196: ..........................T.C.......G........T....  Sboliviensis

                                     LTR
           ------------------------------------------->
                   17187     17197     17207     17217     17227
                       :         :         :         :         :
   17178: TTGGGATTTATTTATAACCCATAGAATGTTGTATAATTGGAGCTGCTGCA  Hsapiens
  336838: .................................G................  Ptroglodytes
  355039: .................................G................  Ggorilla
  380681: ...........................A.....G..C.A...........  Mmulatta
  333280: ..........................G......G..C.......A.....  Panubis
  367114: ....A.........C....A.............G................  Cjacchus
 1017246: ..............C....A.......A.....G................  Sboliviensis

                                            Simple
                  ------------------------------------------>
                   17237     17247     17257
                       :         :         :
   17228: TGATTTCATATATATATGAAATATATATGAAATAT---------------  Hsapiens
  336888: ...................................GTATATATGAAATAT  Ptroglodytes
  355089: ......................                              Ggorilla
  387141:              ...C.............-....---------------  Pabelli
  380731: ......................---------.CC.---------------  Mmulatta
  333330: ......................---------..G.---------------  Panubis
  367164: ....A..TC........---.C.C....A.C....---------------  Cjacchus
 1017296: .T..---..........---...C......C.C..---------------  Sboliviensis


   17263: --------------------------------------------------  Hsapiens
  336938: GCATATATGAAATATATATGAAATATGTATATGTGAAATATATATGAAAT  Ptroglodytes
  387162: --------------------------------------------------  Pabelli
  380757: --------------------------------------------------  Mmulatta
  333356: --------------------------------------------------  Panubis
  367196: --------------------------------------------------  Cjacchus
 1017325: --------------------------------------------------  Sboliviensis

                                     Simple
           ------------------------------------------------->
                             17265     17275     17285    17290
                                 :         :         :        :
   17263: -----------------ATATGAAATGTATATGTGAAATAT-----ATGT  Hsapiens
  336988: ATGTATATATGAAATAT........................-----....  Ptroglodytes
  387162: --------ATTTTTCGT...C.....A......-A.T....TTTTCG.A.  Pabelli
  380757: ----------------------------------------------..A.  Mmulatta
  333356: ----------------------------------------------..A.  Panubis
  367196: -----------------T.CCC.T..A......-.C.....-----..A.  Cjacchus
 1017325: -----------------T.CCC.T.CA......-.G.....-----..A.  Sboliviensis
```

```
                                 Simple
              17300     17310     17320     17330     17340
   17291: ATGAAATATATATTTCATACATATATTTCATATGTGAAATACATATTTAT  Hsapiens
  337033: ..................................................  Ptroglodytes
  387203: .C...........---GA.T....T....G...AC......T..GAA...  Pabelli
  380761: ......--------------------------------------------  Mmulatta
  333360: ......--------------------------------------------  Panubis
  367223: ....G.----------..........G.--------.C...T....G...  Cjacchus
 1017352: ....G.----------..........G.--------GTG.GTG.G.G.G.  Sboliviensis

                                 Simple
              17344     17349     17359     17368     17378
   17341: TT------TAT-----ATATATTATTTTATATAT-TATATATATTTTATA  Hsapiens
  337083: ..------...-----..................-...............  Ptroglodytes
  387250: A.TTTTCG...ACGAA......G.A.A...T.T.-CG.....GAAA....  Pabelli
  380767: --------...-----..G.-----.....C...-........AC.....  Mmulatta
  333366: ----------.-----.....A.G......C...-........AC.....  Panubis
  367255: G.-----G...-----......ATA.A.....G.A.........CCCCC.  Cjacchus
 1017384: G.-----G...-----......ATA.AC......A......C.GCCCCC.  Sboliviensis

                                 Simple
              17386     17396     17406     17416
   17379: TATATT--ATATATATTTCATATATATTTCATATGTACATATTT--TA--  Hsapiens
  337121: ......--..........................A.........--..--  Ptroglodytes
  355278:                                       ......--..--  Ggorilla
  387299: ...GA---......T.CAT.........CAT.T.A..T.....GAA..--  Pabelli
  380798: ----------------------------------T..A..------A.--  Mmulatta
  333400: ----------------------------------T..A..------A.--  Panubis
  367295: AC.G..CA..CCC.C.G..C.TGC.C..C..GC.CAG..CCA.C--..AC  Cjacchus
 1017424: AC.G..CG..CCC.C.G..C.CGC.C..C..GC.CAGA.CCA.C--..GC  Sboliviensis

            Simple                   LTR
              17427     17437     17447     17456     17466
   17423: -----TATATAAAATATCCCTAGCCAGAACAACC-AATCAAGCCCTTCAC  Hsapiens
  337165: -----.............................-...............  Ptroglodytes
  355286: -----.............................-...............  Ggorilla
  387344: -----.............................-..............A  Pabelli
  380806: -----............................T-...............  Mmulatta
  333408: -----............................T-...............  Panubis
  367343: TGCAT.GC........C................TG.......T....T..  Cjacchus
 1017472: TGCAT.GC..........................CT......T.......  Sboliviensis

                                  LTR
              17476     17481     17491     17501     17511
   17467: AAAAAAAAAAAAA-----GAGAGAGATAATGAAATGACTGCTGCTCTCTT  Hsapiens
  337209: .............G-----...............................  Ptroglodytes
  355330: .............------.....A.........................  Ggorilla
  387388: .........----------...............................  Pabelli
  380850: .............AAAAAA.T.............................  Mmulatta
  333452: .............------.T.............................  Panubis
  367393: .........C.--------T........C........T.A.CA.......  Cjacchus
 1017522: .......C.----------T........C........T.A.CA.......  Sboliviensis
```

```
                                      LTR
                 17521     17531     17541     17551     17561
                     :         :         :         :         :
  17512: AAACCACTAAGGTTTGGGGTAGTTTGAATTGCAGCAATAGTCACTGAAAC  Hsapiens
 337254: ...........................T.............A........  Ptroglodytes
 355374: ..................------------...........A.......T  Ggorilla
 387428: ...........................TA............A.......T  Pabelli
 380900: ........C.......A..........TA.....T......A........  Mmulatta
 333496: ........C.......A..........TA.....T......A........  Panubis
 367435: .........................C.TA..G.........A........  Cjacchus
1017562: .........................A.TA..G....G....A........  Sboliviensis

                                      MIR
                 17571     17581     17591     17601     17611
                     :         :         :         :         :
  17562: GAAACTTGAGTTTAGATTCTGGTTCTTTGACTTACTGTGTGTCCTCAGGT  Hsapiens
 337304: ...........................C......................  Ptroglodytes
 355412: .....-.....................C...G..................  Ggorilla
 387478: ...........................C......................  Pabelli
 380950: A..........................CC.....................  Mmulatta
 333546: ...................C.......CC.....................  Panubis
 367485: A....C................A....CC.....................  Cjacchus
1017612: A...TCC...............A....CC.....................  Sboliviensis

                                      MIR
                 17621     17631     17641     17651     17661
                     :         :         :         :         :
  17612: AAATTGCTTAATCTCTCTGAGCCTGCATTGTCTAAACTTTAAAATATGGT  Hsapiens
 337354: ..................................................  Ptroglodytes
 355461: ..................................................  Ggorilla
 387528: .......................................G..........  Pabelli
 381000: ..............................C........G.....G....  Mmulatta
 333596: ..............................C........G.....G....  Panubis
 367535: ....C....................A...T.........G........A.  Cjacchus
1017662: ...C.....G...............A...T........CG......CA..  Sboliviensis

                                      MIR
                 17671     17681     17691     17701     17711
                     :         :         :         :         :
  17662: TGATTGTATCACCTACCCTGTTGGTTGCTGTTGCAGAACAAATGACAAAT  Hsapiens
 337404: ............................-.....................  Ptroglodytes
 355511: .....................C............................  Ggorilla
 387578: ..................................................  Pabelli
 381050: .......................-C.........G............G..  Mmulatta
 333646: .......................-C.........................  Panubis
 367585: ...CC.A......C...T...............T.....G.........C  Cjacchus
1017712: ...............T.T...............................C  Sboliviensis

                                      MIR
                 17721     17731     17741     17751     17761
                     :         :         :         :         :
  17712: AACTATAAAACACTTAACACAAGGCAAGCACAGAGTAAAACCTCAATAAA  Hsapiens
 337453: .......................C.........................G  Ptroglodytes
 355561: .......................C..........................  Ggorilla
 387628: .......................C..........................  Pabelli
 381099: .......................C..............G...........  Mmulatta
 333695: .......................C..............G...........  Panubis
 367635: ..TC.....G...-------...C..............G...........  Cjacchus
1017762: ..TC.........-------...CA.............G...........  Sboliviensis
```

```
                     MIR
               ----------------->
                    17771     17781     17790     17800     17810
                        :         :         :         :         :
   17762: TATCAACTATTATTATTAATAATTGGTA-TTTTTTATGATTCTCTGATGT  Hsapiens
  337503: ............................-........T............  Ptroglodytes
  355611: ......................C.....-........T............  Ggorilla
  387678: ...............C............-.....................  Pabelli
  381149: .......G....................-.....................  Mmulatta
  333745: ............................T.....................  Panubis
  367678: G..............A....C..------A....................  Cjacchus
 1017805: G..............A.......------...............-A....  Sboliviensis

                    17820     17830     17840     17850
                        :         :         :         :
   17811: AGCCCTTAGGATATTTGAGAACCTATGTTTTCCTTGCTTTATTTGGCC--  Hsapiens
  337552: ................................................--  Ptroglodytes
  355660: ................................................--  Ggorilla
  387727: .......................C........................--  Pabelli
  381198: .......................C......................A.AA  Mmulatta
  333795: .......................C..............G.......A.--  Panubis
  367722: ..............A........C.............C..........TT  Cjacchus
 1017848: ......G..T.............C........................TA  Sboliviensis

   17859: --------------------------------------------------  Hsapiens
  337600: --------------------------------------------------  Ptroglodytes
  355708: --------------------------------------------------  Ggorilla
  387775: --------------------------------------------------  Pabelli
  381248: AAGCTGTGACAACTGTAAGTTACCCATAGAACCCATGTTTTCCTTGCTTT  Mmulatta
  333843: --------------------------------------------------  Panubis
  367772: GGGAATAATC----------------------------------------  Cjacchus
 1017898: GGGAATAATC----------------------------------------  Sboliviensis

                    17860     17870     17880     17890     17900
                        :         :         :         :         :
   17859: --------AAAAACTTAAGTGACAACTGTAAGTTATACAGCTATGATCTC  Hsapiens
  337600: --------....G.....................................  Ptroglodytes
  355708: --------....G.....................................  Ggorilla
  387775: --------....G.....................................  Pabelli
  381298: ATTTGTAC....G....G.................CC.............  Mmulatta
  333843: --------....G......................CC.............  Panubis
  367782: --------....G...G.................................  Cjacchus
 1017908: --------........G..........A...A.................G  Sboliviensis

                    17910     17920     17930     17940     17950
                        :         :         :         :         :
   17901: CTACTGAGCAATAGGCACATGTCTTTGGCAATTGCTGTTACTTCCTCACT  Hsapiens
  337642: ..................................................  Ptroglodytes
  355750: ..................................................  Ggorilla
  387817: .............A....................................  Pabelli
  381348: ...........C................................T.....  Mmulatta
  333885: ...........C...T.....................-.G..........  Panubis
  367824: T..............TG...................A...........A.  Cjacchus
 1017950: ...............TG...................A.............  Sboliviensis
```

```
              17960     17969     17979     17989     17999
                  :         :         :         :         :
   17951: GTACCTTTTCTTTTA-TTTTAAATACAAATAAAATTTTATTTGTATATCA  Hsapiens
  337692: ...T...........-..................................  Ptroglodytes
  355800: ...............-..................................  Ggorilla
  387867: ...............-..................................  Pabelli
  381398: ...............-.......C............G.............  Mmulatta
  333934: ...............-.......C............G.............  Panubis
  367874: ...........C...T.....G.......G...T................  Cjacchus
 1018000: ...A.....A.-----.....G.......G....................  Sboliviensis

              18009     18019     18026     18036     18046
                  :         :         :         :         :
   18000: AAGACTCTATGAAATGATAACATAAT---GTTAACAGAGTTGATGTCAAA  Hsapiens
  337741: ..................G.......---.....................  Ptroglodytes
  355849: .........C........G.......---.....................  Ggorilla
  387916: ...G..............G.......---.....................  Pabelli
  381447: ...G.....CA....TG.G...C...---.....................  Mmulatta
  333983: ...G.....CA....T..G...C..A---.....................  Panubis
  367924: ....TC...A.......G........AAG.........A...........  Cjacchus
 1018045: ....T....A.......GG.......AAG.........A...........  Sboliviensis

              18056     18066     18076     18086     18096
                  :         :         :         :         :
   18047: ACACCAATAGGTTTGAAGTTATAGATGATAAATCACTTTGTTTCATTCAA  Hsapiens
  337788: ....A.............................................  Ptroglodytes
  355896: ....A.............................................  Ggorilla
  387963: ....A....................C........................  Pabelli
  381494: ....A............................................G  Mmulatta
  334030: ....A....................C.......................G  Panubis
  367974: ...TA.................G......C.................GG.  Cjacchus
 1018095: ....A.........C.......G......C....................  Sboliviensis

              18106     18116     18126     18136     18146
                  :         :         :         :         :
   18097: CCTTCCCTCGATTACATTATAGAGCATCCCTGGTATGCTCCCAGTTGAAT  Hsapiens
  337838: ...................G..............................  Ptroglodytes
  355946: ...................G..G...........................  Ggorilla
  388013: ........T..........G...A..........................  Pabelli
  381544: ........T......G...G..............................  Mmulatta
  334080: ........T......G...GC.........................A...  Panubis
  368024: ...C....T..........G.............C...G.TG..A......  Cjacchus
 1018145: ........T..........G....A........C...G.TG..A.G....  Sboliviensis

              18156     18166     18176     18186     18196
                  :         :         :         :         :
   18147: CTTAAGTATGATGTATCTCACTGTGTCTTTTCTACCCTGCTGCTGGGGAA  Hsapiens
  337888: ......................T.A.........................  Ptroglodytes
  355996: ......................T.A......T..................  Ggorilla
  388063: ........................A..........T..............  Pabelli
  381594: .............C........A.A.........................  Mmulatta
  334130: .............C........A.A.........................  Panubis
  368074: ......C..A...C..........AC....C..CT......T.......G  Cjacchus
 1018195: ......C......C..........AC........T......T....C..G  Sboliviensis
```

```
               18206     18215     18225     18235     18245
   18197: CTTATTTCCA-AGGCATCGACCTCTGCAGCCACCTGCATAAAAGGGCCAA  Hsapiens
  337938: ..........-.......A...................C..........G  Ptroglodytes
  356046: ..........-.......A...................C......A...G  Ggorilla
  388113: ..........-G...................G......C..........G  Pabelli
  381644: ........A.-...........................C..........G  Mmulatta
  334180: ........A.-...........................C..........G  Panubis
  368124: ..........TC.TT...T.......A....T......CG.........G  Cjacchus
 1018245: ..........-...T...T.......A...TG......C..........G  Sboliviensis

               18255     18265     18275     18285     18295
   18246: AATGGTTGTAATTTCTTTGAGGAAGCCTGTCCCTGGAGCTAAGGTGTAGC  Hsapiens
  337987: ..................................................  Ptroglodytes
  356095: ..................................................  Ggorilla
  388162: ..CA.....................A......................A.  Pabelli
  381693: ..CA.....................G.........A..............  Mmulatta
  334229: ..CA.......G.............G.........A..............  Panubis
  368174: .GC..A..-........CA......G...............C........  Cjacchus
 1018294: .GC..C..-.........A......G..............G....A....  Sboliviensis

               18305     18315     18325     18335     18345
   18296: TGTTGTTCTCATCGGCCCAAGCCACGGCCAATGGTGTTCTTGCCTGTCCT  Hsapiens
  338037: .............A..............T.....................  Ptroglodytes
  356145: .............A....................................  Ggorilla
  388212: .............A...........A....---------CA.........  Pabelli
  381743: .............A...........C........................  Mmulatta
  334279: .............A...........C........................  Panubis
  368223: ....---...C..A...........A........CA..............  Cjacchus
 1018343: ....---...C..A...........AA..........C............  Sboliviensis

               18355     18365     18375     18385     18395
   18346: CTCTCCTGGGGCCCTTTCCCCTGGAGCTGTCTACATAATGTGGAAATAAA  Hsapiens
  338087: .......A............T.............................  Ptroglodytes
  356195: ....................T.............................  Ggorilla
  388253: ........................................A.........  Pabelli
  381793: ............G.....A.....................A.......C.  Mmulatta
  334329: ............G.........C.................A.........  Panubis
  368270: ..........C.....................G.......A.........  Cjacchus
 1018390: ..........C.....................G.......A.........  Sboliviensis

               18405     18415     18425     18435     18445
   18396: AATCTTCAAAGGGAGCTATCCCACTTGGCATGGTATAGCTAAGCAAACAG  Hsapiens
  338137: .......................T.....T....................  Ptroglodytes
  356245: .......................T.....T....................  Ggorilla
  388303: .............................TA...................  Pabelli
  381843: ..................A..........T....................  Mmulatta
  334379: ..................A.T........T.....G..............  Panubis
  368320: C.....AGC..T---------------..T....................  Cjacchus
 1018440: .......G....---------------T.T....................  Sboliviensis
```

```
                 18455     18465     18474     18484     18494
                     :         :         :         :         :
  18446: GTTTCAGGGTACATTACAGGCT-CCCCACCTTCTCAGAGACTATTTAAAC  Hsapiens
 338187: ......................-..........................T  Ptroglodytes
 356295: ......................-...........................  Ggorilla
 388353: .....G................-...........................  Pabelli
 381893: ....TG................-...................G.......  Mmulatta
 334429: ....TG.....T........T.C...................G.......  Panubis
 368355: ....TG....G.....T.....-...........................  Cjacchus
1018475: ....TG....G....GT.....-..T........................  Sboliviensis

                 18504     18514     18524     18534     18544
                     :         :         :         :         :
  18495: CCTATCCCATTCCTGACTTGCTTGTAGACTAAGTCCTCCCCAGATCTACC  Hsapiens
 338236: ..................................................  Ptroglodytes
 356344: ..................................................  Ggorilla
 388402: ....C.............................................  Pabelli
 381942: .T..............T..T.......G..--........A.........  Mmulatta
 334479: .T..............T..T.......G..--........A.........  Panubis
 368404: ............A...........................A...A.....  Cjacchus
1018524: A...........A..G.........................G....C...  Sboliviensis

                 18554     18564     18574     18584     18594
                     :         :         :         :         :
  18545: CCAGAAAACTCCAATTCCAACTTCATCCTATAATAAAATATAGATGTAAT  Hsapiens
 338286: ......G...........................................  Ptroglodytes
 356394: ..................................................  Ggorilla
 388452: .............G....................................  Pabelli
  26545:                                  ......G..A......A  Nleucogenys
 381990: ...........................T..C.........C....A....  Mmulatta
 334527: ...........................T............C....A....  Panubis
 368454: ........................................C.........  Cjacchus
1018574: .............G.........T..........G.....C.........  Sboliviensis

                 18604     18614     18624     18634     18644
                     :         :         :         :         :
  18595: AATGTTAAATGGGACTTGTCTCATTTCTGAGTTTGTCTGCTGGTCCAAGG  Hsapiens
 338336: ..................................................  Ptroglodytes
 356444: .........C........................................  Ggorilla
 388502: .................................A................  Pabelli
  26562: .......CG..                                        Nleucogenys
 382040: .....................T.....C......................  Mmulatta
 334577: .....................T............................  Panubis
 368504: .C............................T.G.A...CA...C......  Cjacchus
1018624: .................T..............G.A...CA...C......  Sboliviensis

                 18654     18664     18674     18684     18694
                     :         :         :         :         :
  18645: CAGATATTAGCAGATAATAAGCATGCATCATGCTGTAAAGAGGGCTACCA  Hsapiens
 338386: .............G...........................A........  Ptroglodytes
 356494: .............G....................................  Ggorilla
 388552: .................C......A..............C..........  Pabelli
 382090: ....................A..C.....T.........C..........  Mmulatta
 334627: ....................A..C.....T.........C..........  Panubis
 368554: ........................AT..T..........C..A.......  Cjacchus
1018674: .............G..............T..........C..A..C....  Sboliviensis
```

```
              18704     18714     18724     18734     18744
                  :         :         :         :         :
  18695: GGGCATCCACTCAAGATAGGCCAAGACACCATGGGCCTGGTCTGATGACC Hsapiens
 338436: .................................................. Ptroglodytes
 356544: .................................................. Ggorilla
 388602: ...................A.A.........CA................. Pabelli
 382140: ..................A............................... Mmulatta
 334677: ..................A............................... Panubis
 368604: .....G..C.................TG....................T. Cjacchus
1018724: .A...G....................TG....................T. Sboliviensis

              18754     18764     18774     18784     18794
                  :         :         :         :         :
  18745: CTGACCTCAAGGCTCACATCCAAGATACTGGAGAGGATGCTGAGGATTTT Hsapiens
 338486: .................................................. Ptroglodytes
 356594: .................................................. Ggorilla
 388652: .....G.........................................G.. Pabelli
 382190: ......C.................G......................... Mmulatta
 334727: ......C....A.........G..G......................... Panubis
 368654: ..A.......T....G...T....T................A.A...G.. Cjacchus
1018774: ....T............G.T....T................A.A...G.. Sboliviensis

              18804     18814     18824     18834     18844
                  :         :         :         :         :
  18795: CCCCTGGAGGTTAGAGAATGGGGGTTTCTTGGGAGGAAAAGGGGAAAGGG Hsapiens
 338536: ..............................T................... Ptroglodytes
 356644: .......G......................T................... Ggorilla
 388702: ..............................T................... Pabelli
 382240: .............................CT.........A......C.. Mmulatta
 334777: ..............................T.........A......C.. Panubis
 368704: ...G...............-..........T.......G........... Cjacchus
1018824: .........A.................TA.T.............G..... Sboliviensis

              18854     18864     18874     18884     18894
                  :         :         :         :         :
  18845: ATGCTGCTTCTCTCCACATGAAGGAGAGGGGATAAATTCCCCATACCTT Hsapiens
 338586: ...............G.G................................ Ptroglodytes
 356694: ...............G......................A........... Ggorilla
 388752: .....T..........A..........................C...... Pabelli
 382290: ..A....C....................A...............C..... Mmulatta
 334827: .......C................................T...C..... Panubis
 368753: .......G......................A.G..............T.. Cjacchus
1018874: ................TG..........-.A................T.. Sboliviensis

              18904     18914     18924     18933     18943
                  :         :         :         :         :
  18895: ATACCATAGGCGGGGGAGCTGGATTTAAGAGAAAG-AAAACACACATAGA Hsapiens
 338636: ..........T........................-.............. Ptroglodytes
 356744: ...................................-......T....... Ggorilla
 388802: .............A................T....A.............. Pabelli
 382340: ........C.........................--............C. Mmulatta
 334877: ...................................A............C. Panubis
 368803: ...........A...A...........G.....G.T............C. Cjacchus
1018923: ......C....A...A...........G.......A.............. Sboliviensis
```

```
                18953     18963     18973     18983     18993
   18944: GAGGGAGGTACTTGCCACTAGGAGAAATCGACATCTCCCGGGCAACTGGA   Hsapiens
  338685: .......................................A..........   Ptroglodytes
  356793: ...............................TG.................   Ggorilla
  388852: ...........................C...........A.T........   Pabelli
  382388: ............                                         Mmulatta
  386056:             .......G.......CT........T.A..........   Mmulatta
  334927: ............                                         Panubis
  338837:             .......G.......CT........T.A..........   Panubis
  368853: ......C....................CT......CAA.A..........   Cjacchus
 1018973: ...........................CT......CAA.A..........   Sboliviensis

                                                       Alu
                                                        ►
                19003     19013     19023     19033     19043
   18994: TGTCTGCTGGGATGCAGAAACCTTGAGCCTTGTGAAATAAGGAGTTCAGG   Hsapiens
  338735: ...T........................................A.....   Ptroglodytes
  356843: ...T........................................A.....   Ggorilla
  388902: ...T..................................G.....A.....   Pabelli
  386094: ...T...........G............................A.....   Mmulatta
  338875: ...T...........G............................A.....   Panubis
  368903: ...T......T...............A.................A.....   Cjacchus
 1019023: ...T.............--...G...A.T...............A.....   Sboliviensis

                                 Alu
          ------------------------------------------------->
                19053     19063     19073     19083     19093
   19044: GCCGGGCACGGTGGCTCACACCTGTAATCCCAGCACTTTAGAAGGGCAAG   Hsapiens
  338785: ..................................................   Ptroglodytes
  356893: ..................................................   Ggorilla
  388952: ...A..............................................   Pabelli
  386144: ..T.............................................G.   Mmulatta
  338925: ..T.............................................G.   Panubis
  368953: ...A...G..A.......T......................G...C.GG.   Cjacchus
 1019071: A.AA..............T......................G...C.GG.   Sboliviensis

                                 Alu
          ------------------------------------------------->
                19103     19113     19123     19133     19143
   19094: GTGGGCAGATCGCTTGAGGTCATGGGCTCAAGACCAGCCTGGCCAACATG   Hsapiens
  338835: ........................A.T.......................   Ptroglodytes
  356943: ........................A.T.......................   Ggorilla
  389002: ..................A...G.A.T...................G...   Pabelli
  386194: ......................G.A.T......................A   Mmulatta
  338975: ...........A..........G.A.T......................A   Panubis
  369003: .CA...G....A..........G.A.T....A.G................   Cjacchus
 1019121: .CA..AG....A..........G.A...T..A.G.............G..   Sboliviensis

                                 Alu
          ------------------------------------------------->
                19153     19163     19173     19183
   19144: GAGAAACCCCGTCTCTACTAAAAATACACAAATTAGCTGGGCATGGTG--   Hsapiens
  338885: .C...................................C..........--   Ptroglodytes
  356993: .C.......T...........................C..........--   Ggorilla
  389052: .C...................................C....G.....--   Pabelli
  386244: .T........A..........................C....G.....--   Mmulatta
  339025: .T........A..........................C....G...G.GT   Panubis
  369053: .C.....T..A.................A........CA.T.C..T..--   Cjacchus
 1019171: .T.....T..A.................A........CA...C.....--   Sboliviensis
```

```
                                   Alu
              19193     19203     19213     19223     19232
   19192: --------GTCAGTGCCTATAATCCCAGCTATTCGGGAGGC-TGAGGTAC Hsapiens
  338933: --------................T................-........ Ptroglodytes
  357041: --------....C............................-........ Ggorilla
  389100: --------..............................T..-.....C.T Pabelli
  386292: --------...G......................A......-.....C.. Mmulatta
  339075: CGGTGCCA...G......................A......-.....C.. Panubis
  369101: --------..GG...A..G................A.....T.....C.. Cjacchus
 1019219: --------..GG......G...............AA...A.-.....C.. Sboliviensis

                                   Alu
              19240     19250     19260     19270     19280
   19233: GAGAA--TCCCTTGAACCCAGGAGGCAAAGATTGCAGTGATCTGAGATCA Hsapiens
  338974: .....--........................................... Ptroglodytes
  357082: .....--........................................... Ggorilla
  389141: .....--..........T......................C.C..A.... Pabelli
  386333: A....--.................................C.CT...... Mmulatta
  339124: .....--.................................C.CT...... Panubis
  369143: ....C--.T..C....TGTG..........G...........CA.....C Cjacchus
 1019260: ....GAC.T.......TGTG.........T...A........CA.....T Sboliviensis

                                   Alu
              19290     19300     19309     19318     19328
   19281: TGCCACTGCACTCCAGCCTGGGTGAC-AGCAAGA-CTCCATCTCAAAAAA Hsapiens
  339022: ..........................-.......-............... Ptroglodytes
  357130: ..........................-..AGT..GT.............. Ggorilla
  389189: ..........................-.......-............... Pabelli
  386381: ..........................-.......-............... Mmulatta
  339172: ..........................-.......-............... Panubis
  369191: CA....CA..G....C......C..TG...G.A.-..............T Cjacchus
 1019310: .A....CA..G..T........CA.TG.....A.-..T...........T Sboliviensis

                 Alu
              19338                         19354     19360
   19329: AAAAAAGAAAAAAAAAA--------------GACAGAGAGAGA----AAA Hsapiens
  339070: .....GA..........--------------............AAAG... Ptroglodytes
  357179: ......A.G........------------AA............AAAG... Ggorilla
  389237: ......AG.G.G.G.G.--------------------....T.AAAG... Pabelli
  386429: ....G.A....G.G.C.----------------------....----... Mmulatta
  339220: ...G..A...G.G.G..AGACTTAGTTATGG...T..A.....----.GC Panubis
  369240: ....T.A..T..T....--------------------------------. Cjacchus
 1019359: ....T.A..T..T....--------------------------------- Sboliviensis

              19370     19380     19390     19400     19410
   19361: GAAAGAGACAAGCACTAAGTCAACAATGACCTCCCAGCACAAAGGCTAGA Hsapiens
  339106: .................................................. Ptroglodytes
  357217: .................................................. Ggorilla
  389267: ........T......................................... Pabelli
  386453: ........T..............TG......................... Mmulatta
  339266: T.C.....                                           Panubis
  369258: ...G....TC........C..G.......T....T............... Cjacchus
 1019376: --.G....TC..............G....T...................G Sboliviensis
```

```
                    19420     19430     19440     19446     19456
                        :         :         :         :         :
   19411: GGAGTACTATCACACACAAAAGCTGAAACAGAGAG----GATGCATCTGT  Hsapiens
  339156: ............G......................----...........  Ptroglodytes
  357267: ............G......................----...........  Ggorilla
  389317: ............G......................----...........  Pabelli
  386503: ............G......................----...........  Mmulatta
  369308: ......T.G...G....-............A....AGAA.........TC  Cjacchus
 1019424: ............G.................AC...AGAA.........TC  Sboliviensis

                    19466     19476     19486     19496     19506
                        :         :         :         :         :
   19457: ATGGTGGGTGAGAAAGTTTGTAAAAGTCAAAGGTTCTAGGACCTGAGCCA  Hsapiens
  339202: ...........................................C......  Ptroglodytes
  357313: ........................C..................C......  Ggorilla
  389363: ..............................G............C......  Pabelli
  386549: .......A.............................------------.  Mmulatta
  369357: .....A.....................................C...A..  Cjacchus
 1019474: .....A...........C.........................C...A..  Sboliviensis

                    19516     19526     19536     19546     19556
                        :         :         :         :         :
   19507: GGAAAAAGCTTAGCCACAGGGAGGAGCTGATTTCTGCAGTGAGCAGTGCA  Hsapiens
  339252: .....................................C............  Ptroglodytes
  357363: .....................................CA...........  Ggorilla
  389413: .......-...................................T.C....  Pabelli
  386587: ..................................................  Mmulatta
  369407: ...................................TT.............  Cjacchus
 1019524: .......T...................C.......T..............  Sboliviensis

                    19566     19576     19586     19596     19606
                        :         :         :         :         :
   19557: GTGCTCCATCTAAACTAGTGTCGTTCATCTCAAAGGCTTAGTTGTGGGAC  Hsapiens
  339302: ..................................................  Ptroglodytes
  357413: ..................................................  Ggorilla
  389462: ..................................................  Pabelli
  310732:                                .G.T...............  Nleucogenys
  386637: ........A.....G......T.............A.......A......  Mmulatta
  339235:                                ....A.......A......  Panubis
  369457: .CT..................G.........G...........A...TTT  Cjacchus
 1019574: .C...................G.................T...A...TTT  Sboliviensis

                    19616     19626     19636     19646     19656
                        :         :         :         :         :
   19607: TGAAGGAGAAGCTATAGAGAATCAGAATTAGAACTCAGTTCTCTAAACTG  Hsapiens
  339352: ......................................G...........  Ptroglodytes
  357463: ......................................G...........  Ggorilla
  389512: ....A.................................G...........  Pabelli
  310751: ....A....C............................G....C......  Nleucogenys
  386687: ....A...................A........G....G...........  Mmulatta
  339254: ....A.........C.........A.............G...........  Panubis
  369507: ....A..........GA.A...................G....G......  Cjacchus
 1019624: ....A..........G..A...................G...........  Sboliviensis
```

```
                                                                      MIR
                                                          ----------------------->
               19666       19676       19686       19696       19706
19657: CAAATTTAACCCCACCCACAAATGTACTGGGTGTTACTACAGCAATAGCA  Hsapiens
339402: ..................................................  Ptroglodytes
357513: ..................................................  Ggorilla
389562: ..C...............................................  Pabelli
310801: .........................G...A....................  Nleucogenys
386737: .............................AC........T.....C....  Mmulatta
339304: .............................AC........T.....C....  Panubis
369557: ....G......A.........GCA..............TT..........  Cjacchus
1019674: ..................-...C...............TT..........  Sboliviensis

                                   MIR
        ------------------------------------------------->
               19716       19726       19736       19746       19756
19707: TGGAGGACAGGACATTGACTCTGAAGCTGGGGCTTCCTAGGCTCAAATCT  Hsapiens
339452: ........G.........................................  Ptroglodytes
357563: ..................................................  Ggorilla
389612: CA.........................C......................  Pabelli
310851: CA...................................C....C.......  Nleucogenys
386787: CA.......................A.C...A..................  Mmulatta
339354: CA.......................A.C...A..................  Panubis
369607: CA........A...CA..T........CA.....................  Cjacchus
1019723: CA........A...CG..T........CA......T..............  Sboliviensis

                                   MIR
        ------------------------------------------------->
                           19773       19781       19791       19801
19757: TGGCTCT---GCAACTAACACTG--ATCTAGGCAAACCACTGACATTGTC  Hsapiens
339502: .......---.............TG..................T......  Ptroglodytes
357613: .......---.............TG.........................  Ggorilla
389662: .......---.............TG...........T.............  Pabelli
310901: .......---...G.........TG....G................C...  Nleucogenys
386837: AACT..CTAG.............TG...C..........A..........  Mmulatta
339404: .A.....---.............TG...C..........A..........  Panubis
369657: C......---A....A.....C.TG......................C..  Cjacchus
1019773: .......---A..G..C......TG......................C..  Sboliviensis

                                   MIR
        ------------------------------------------------->
               19811       19821       19831       19841       19851
19802: CAATTCTCTGTCTTCTCATCTTGGACTGAAAATAGCAATAAGACATGCCA  Hsapiens
339549: ..................................................  Ptroglodytes
357660: ..................................................  Ggorilla
389709: ...................................T.....TG.......  Pabelli
310948: ........C.........................................  Nleucogenys
386887: ...G.....................................T........  Mmulatta
339451: ...G.......................A.............T........  Panubis
369704: ...G......C...............G.G............TG.......  Cjacchus
1019820: ...G...................C....G............T........  Sboliviensis
```

```
                                   MIR
          ------------------------------------------------------>
                 19861     19871     19881     19891     19901
   19852: CGTAGAGTTATGGGACCAAATTAGTGAGATATGTACAACGGTGCCTGGCA   Hsapiens
  339599: ..C.......C.......................................   Ptroglodytes
  357710: ..........C.......................................   Ggorilla
  389759: .A.................................A..T.........T.   Pabelli
  310998: .A...........A.....................A..T...........   Nleucogenys
  386937: .A.........................T.......A..T...........   Mmulatta
  339501: .A.........................T.......A..T...........   Panubis
  369754: .A..C...G.............G..AGA.......A..T...........   Cjacchus
 1019870: .A..C.........G.......G..A.A.......A..T...........   Sboliviensis

                         MIR
          -------------------------------------------->
                 19911     19921     19931     19941     19951
   19902: TGAAGTTATAATACTGCAATAAATGTTAGCTCTTGTTTCTGTTGTCATCC   Hsapiens
  339649: ..................................................   Ptroglodytes
  357760: ..................................................   Ggorilla
  389809: ..............A...G...............................   Pabelli
  311048: ..................................A...............   Nleucogenys
  386987: .................................................A   Mmulatta
  339551: .................................................A   Panubis
  369804: ..........GC.....G.............................C.G   Cjacchus
 1019920: ............G....................................G   Sboliviensis

                 19957     19967     19977     19987     19997
   19952: TGGG----CTTCCACAAGGACAGATTATATCTTTTATTCATCCCGTGTTA   Hsapiens
  339699: ....----..................G...T...................   Ptroglodytes
  357810: ....----..................G.......................   Ggorilla
  389859: ....----..................................T.......   Pabelli
  311098: ...A----.......-..........G.T..............T......   Nleucogenys
  387037: ...A----...................C...............T......   Mmulatta
  339601: ...A----...................C...............T......   Panubis
  369854: ..AACTTC.....T...................A.C........A.....   Cjacchus
 1019970: ..A.----A.................G......A.C........A.....   Sboliviensis

                 20007     20017     20027     20037     20047
   19998: CTGTCTCATCCACAAGGAGGAGCTCAATACACACTGGTTGACAAAAGGAT   Hsapiens
  339745: ..................................................   Ptroglodytes
  357856: ...............................................T..   Ggorilla
  389905: ...CT........................................G....   Pabelli
  311143: T..C..............................................   Nleucogenys
  387083: T..C.........................T...T..A.............   Mmulatta
  339647: T..C.........................T......A.............   Panubis
  369904: TA.................................A....G.........   Cjacchus
 1020016: TA.................................A..G.--C.......   Sboliviensis

                 20057     20067     20077     20087     20097
   20048: TATAACTTTAATGTAAAAGCTGAATAAAATTTCAATGAATAATGGGCAAA   Hsapiens
  339795: ..................................................   Ptroglodytes
  357906: ..................................................   Ggorilla
  389955: ......-.............C......................T......   Pabelli
  311193: ...........................................T......   Nleucogenys
  387133: .............................A.............T......   Mmulatta
  339697: ...........................................T......   Panubis
  369954: ....T............T................C........T......   Cjacchus
 1020064: .................T.......-........C........T......   Sboliviensis
```

```
                     20107     20117     20127     20137     20147
                         :         :         :         :         :
   20098: ACTCGTTACCTTATTTAATATTAGGAAACTTCATTTTGGAGATGTCAGAA  Hsapiens
  339845: ................................G.................  Ptroglodytes
  357956: ................................G.................  Ggorilla
  390004: ..............................--G.................  Pabelli
  311243: ...G...........................TG.................  Nleucogenys
  387183: ...G..............C............TG....A............  Mmulatta
  339747: ...G..............C............TG....A............  Panubis
  370004: ...G......G........G...........TGC......A......A..  Cjacchus
 1020113: ...G...............G...........TG.......A....T....  Sboliviensis

                                           Alu
                                 ----------------------------->
                     20157     20167     20177     20187
                         :         :         :         :
   20148: GATTCTTGATCATCCCTAACAATTCTTTTTTTTTTTTTTTTTTTTTTT--  Hsapiens
  339895: ................................................--  Ptroglodytes
  358006: ............................................C...TT  Ggorilla
  390052: ...........................................-------  Pabelli
  311293: ................................G..G...-----------  Nleucogenys
  387233: ................................................TT  Mmulatta
  339797: ................................................TT  Panubis
  370054: .............T..C.....A.T.......GAG---------------  Cjacchus
 1020163: .............T..........-----------...G...GAGAAG--  Sboliviensis

                              Alu
          ------------------------------------------------->
                                         20200     20210     20220
                                             :         :         :
   20196: -------------------------GAGACAGGGTCTTGCTATGTCACCC  Hsapiens
  339943: -------------------------................G........  Ptroglodytes
  358056: TTTTTTT---------------------------.T..TT.T...ATTTT  Ggorilla
  390095: -------------------------................G........  Pabelli
  311332: -------------------------................G........  Nleucogenys
  387283: TTTTTTTTTTTTTTTTTTTTTTTTT.......A........G...A....  Mmulatta
  339847: TTTTTTT------------------.......A........G...A....  Panubis
  370089: -------------------------...GAG.A....CA..C...TG...  Cjacchus
 1020200: -------------------------...GAG.A....CA..CC..TG...  Sboliviensis

                              Alu
          ------------------------------------------------->
                     20230     20240     20250             20263
                         :         :         :                 :
   20221: AGACTGGAATGCAGTGGTGGGATCTCGGCTCACTA-------CAACCTCT  Hsapiens
  339968: ..G................................-------........  Ptroglodytes
  358079: TA--------.T..A....A.G.T..AC.AT...GGCCAGGCT.GT...G  Ggorilla
  390120: ..G................A...............-------T.C.T...  Pabelli
  311357: ..G.......................A........-------........  Nleucogenys
  387333: ..G.......................A.......G-------........  Mmulatta
  339879: ..G.......................A.......G-------........  Panubis
  370114: ..G....C...........C..G.........A.G-------T..T....  Cjacchus
 1020225: ..G....CG........C.T..G.........A.G-------T.......  Sboliviensis
```

```
                                         Alu
           ------------------------------------------------------------------->
                  20273     20283     20293     20303     20313
                      :         :         :         :         :
   20264: GCGTCCCGGGTTCAAGCAATTCTCCTGCCTCAGCCTCCCGAGTAGCTGGG  Hsapiens
  340011: ..C...T...................................A.......  Ptroglodytes
  358121: AAC...T.ACC...GATG..CTG..CA.T..GT.....AA.AGT....A.  Ggorilla
  390163: ..C..............T................................  Pabelli
  311400: ..C.............TG.............................A..  Nleucogenys
  387376: ..C..............G....................T..A........  Mmulatta
  339922: ..C..............G.......................A........  Panubis
  370157: ..C......A.........G...................A.A........  Cjacchus
 1020268: ..C.........................-..........A.A........  Sboliviensis

                                         Alu
           ------------------------------------------------------------------->
                  20323     20333     20343     20353     20363
                      :         :         :         :         :
   20314: ATTACAGGCACCCGCCACCATGCCAGGCTAATTTTTGTATTTTTAGTAGA  Hsapiens
  340061: .........G..............C.........................  Ptroglodytes
  358171: .........GTGA.....A.C..TT...C..                     Ggorilla
  358067:                                ...................  Ggorilla
  390213: ........TG..............T..............G..........  Pabelli
  311450: .........G..............G.........................  Nleucogenys
  387426: ...........G........CA..CAA.......................  Mmulatta
  339972: ...........G........CA..CAA.......................  Panubis
  370207: .......ATGTGT..T.....T..T.......A....C...........C  Cjacchus
 1020317: .......TT.TGT..A....CA..T.......A....C............  Sboliviensis

                                         Alu
           ------------------------------------------------------------------->
                  20373     20383     20393     20403
                      :         :         :         :
   20364: GACAGGGTTTCACCATGTTGGCCAGGCTGGTCTCGATCTCCTGACCTC--  Hsapiens
  340111: .....................T..........................--  Ptroglodytes
  358086: .GTGA...........AC..........A.......A...........AG  Ggorilla
  390263: .....................A..........................--  Pabelli
  311500: .....................A...T.......T.....-----....--  Nleucogenys
  387476: .....................A............A.............--  Mmulatta
  340022: .....................A............A.............--  Panubis
  370257: ...T.A..........C.........A.......A.............--  Cjacchus
 1020367: ...C.A..........C.........A.......A.............--  Sboliviensis

                                         Alu
           ------------------------------------------------------------------->
                  20421     20431     20441     20451     20461
                      :         :         :         :         :
   20412: GTGATCTGCCTGTCTTGGCCTCCCAAAGTGCTGAGATTACAGGCGTGAGC  Hsapiens
  340159: .......................................A....A.....  Ptroglodytes
  358136: A.........CACT.C.T.....A..........................  Ggorilla
  390311: ......C.....C.....................................  Pabelli
  311543: A.....C....AC..C............................A.....  Nleucogenys
  387524: A..........TC..C............................A.....  Mmulatta
  340070: A..........TC..C............................A.....  Panubis
  370305: A.....G...CACT.CA..........................A......  Cjacchus
 1020415: A.........CAC..CA..........................A......  Sboliviensis
```

```
              Alu
          ------------->
              20471     20481     20491     20501     20511
                  :         :         :         :         :
  20462: CACAACGCTTGGCCAATCATCCCTAACAATTTTACAAGATTTGAATCGTA  Hsapiens
 340209: ..................................................  Ptroglodytes
 358186: ...............................................A..  Ggorilla
 390361: .....T.........................................T..  Pabelli
 311593: .....T.T...................T............G.....T.C.  Nleucogenys
 387574: .....T.....A..............................A.......  Mmulatta
 340120: .....T.....A..............................A.......  Panubis
 370355: ...CG.T.CCA....C..........T.........TA........T...  Cjacchus
1020465: ...CG...CCA...............T........GTA............  Sboliviensis

              20521     20531     20541     20551     20561
                  :         :         :         :         :
  20512: TCCTAACAAATCAGCAACCACTAAACAGATAAGCTCTCTGTTATCAACAC  Hsapiens
 340259: .......G..........................................  Ptroglodytes
 358236: ..................................................  Ggorilla
 390411: ..G...............................................  Pabelli
 311643: ..................................................  Nleucogenys
 387624: .........................T-.......................  Mmulatta
 340170: .........................T-........G..............  Panubis
 370405: G..C......C...............T.............C.T...G...  Cjacchus
1020515: ...C......................................T.......  Sboliviensis

              20571     20581     20591     20601     20611
                  :         :         :         :         :
  20562: CTTGAATTCAGTTAGACAGTCTGGGCTTCCCCTTTCTAAACTGTAGCTTA  Hsapiens
 340309: ..................................................  Ptroglodytes
 358286: ..................................................  Ggorilla
 390461: ................T.............G...................  Pabelli
 311693: .........................................C........  Nleucogenys
 387673: ..................................................  Mmulatta
 340219: ..................................................  Panubis
 370455: ..................A.....A....T..........T.........  Cjacchus
1020565: ........................A.........................  Sboliviensis

              20621     20631     20641     20651     20661
                  :         :         :         :         :
  20612: GATCTTCTGGGAAACCAGCTCAACACAAGTTGTATTTTTAACCATCTGAT  Hsapiens
 340359: ......................................A...........  Ptroglodytes
 358336: ......................................A...........  Ggorilla
 390511: ......................................A...........  Pabelli
 311743: .............C........................A...........  Nleucogenys
 387723: .............C........................A...........  Mmulatta
 340269: .............C........................A...........  Panubis
 370505: ...G....A....C.TG................G....A...........  Cjacchus
1020615: ...G.........C...................G....A...........  Sboliviensis

              20671     20681     20691     20701     20711
                  :         :         :         :         :
  20662: ATATGTAGTACACAGGCTTTTTCATCTCCTTGCTAAGAAGAGAATGTGTA  Hsapiens
 340409: ...............C..................................  Ptroglodytes
 358386: ..................................................  Ggorilla
 390561: .....C............C...............................  Pabelli
 311793: ............A.....C............T............C.....  Nleucogenys
 387773: ..........TG...A..C......T.....T..........C.C.....  Mmulatta
 340319: ..........TG...A..C......T.....T............C.....  Panubis
 370555: .C............A................TT..T........CA....  Cjacchus
1020665: ..............A................TT..T.......CCA....  Sboliviensis
```

```
               20721     20731     20741     20751     20761
                   :         :         :         :         :
   20712: TGTAGAAATAAAACATTTTTTTCTACACTGTAACCTGAAGCCACCTTAAC  Hsapiens
  340459: ..................................................  Ptroglodytes
  358436: ..............-...................................  Ggorilla
  390611: .................C........................G.......  Pabelli
  311843: ..............................C.G.................  Nleucogenys
  387823: .........................T....C.G.................  Mmulatta
  340369: .........................T....C.G.................  Panubis
  370605: ..............C...............C...................  Cjacchus
 1020715: ....................C.........C.G........TG.......  Sboliviensis

               20771     20781     20791     20801     20811
                   :         :         :         :         :
   20762: TTGCTTATAGAACAATCTTATTAGAATTGTCAGTGGAGCTCTGTCATGGG  Hsapiens
  340509: ......T...........................................  Ptroglodytes
  358485: ..................................................  Ggorilla
  390661: ..................................................  Pabelli
  311893: ..................................................  Nleucogenys
  387873: ..................................................  Mmulatta
  340419: ..................................................  Panubis
  370655: ...................G.C....................A...C...  Cjacchus
 1020765: ...................G......................AAT.CAA.  Sboliviensis

               20821     20831     20841     20851     20861
                   :         :         :         :         :
   20812: CCAAGTTCAGTGCACTAACTCCACCCAGCTTATCTCACTCAGGCCATTGC  Hsapiens
  340559: .........A........................................  Ptroglodytes
  358535: .........A.A..T....C..............................  Ggorilla
  390711: ...................CT.............................  Pabelli
  311943: ...................C..............................  Nleucogenys
  387923: ...................C.................T........C...  Mmulatta
  340469: ................G..C.................T............  Panubis
  370705: ....A..............C...T....AA....................  Cjacchus
 1020815: ...................C........AA....................  Sboliviensis

               20871     20881     20891     20897     20904
                   :         :         :         :         :
   20862: TCTTCACTTTGCAATTCTACAACATAGTAGGT----ACTAAAAGC---AT  Hsapiens
  340609: ................................----.........---..  Ptroglodytes
  358585: ................................----.........---..  Ggorilla
  390761: ........................C.......----.......T.---..  Pabelli
  311993: ...............................C----.........---..  Nleucogenys
  387973: ...................G....C......CTGGC.........---..  Mmulatta
  340519: ...................G....C......CAGGC.........---..  Panubis
  370755: ....G.........C.........C......C----.........CTAC.  Cjacchus
 1020865: ..............C.........CGA.G..C----.........---..  Sboliviensis

                                        LINE2
                                   ------------------------->
               20914     20924     20934     20944     20954
                   :         :         :         :         :
   20905: CAAAACAGTTAAAGAATGAATTCTGTCATTCATTCAACTGATATTTATTG  Hsapiens
  340652: ...........................G......................  Ptroglodytes
  358628: ..................................................  Ggorilla
  390804: ..G.............................................C.  Pabelli
  312036: ........G.......................................C.  Nleucogenys
  388020: .....G..........................................CA  Mmulatta
  340566: ................................................C.  Panubis
  370801: TT.................C....C.........................  Cjacchus
 1020908: T-..............C.......C.........................  Sboliviensis
```

```
                                  LINE2
          ------------------------------------------------------->
                 20964      20974      20984      20994      21004
   20955: AAGATCTACCATGCTCCCGGAATTGTTGTTGCTGAGACAACACAAAAATA  Hsapiens
  340702: ..................................................  Ptroglodytes
  358678: ..................................................  Ggorilla
  390854: .............T....................................  Pabelli
  312086: ..................C...............................  Nleucogenys
  388070: .................T................................  Mmulatta
  340616: .................T................................  Panubis
  370851: ...............TTT....---.......C..............G-.  Cjacchus
 1020957: ...............TTT....---...............T......G-.  Sboliviensis
```

```
                                  LINE2
          ------------------------------------------------------->
                 21014                  21033      21043      21053
   21005: TAAAACCCCTGCCCTCATG-AAACTTACATTCTAGTAGGGAGATATTGCC  Hsapiens
  340752: ...................-.......................G......  Ptroglodytes
  358728: ...................-.......................G......  Ggorilla
  390904: ...................A.......................G......  Pabelli
  312136: ...................-................G......G......  Nleucogenys
  388120: ...................-................G......G......  Mmulatta
  340666: ...................-................G......G......  Panubis
  370897: ..........C........-................G......GG..T.  Cjacchus
 1021003: ....G.....C........-.......T........G......G....T.  Sboliviensis
```

```
                                  LINE2
          ------------------------------------------------------->
                 21063      21073      21083      21093      21103
   21054: AGTAAACAAGATAAATAAGTAAAGTGTATGTCAAGAGAGAAGTAAATGCT  Hsapiens
  340801: ..G...............................................  Ptroglodytes
  358777: ..G...............................................  Ggorilla
  390954: ..................................................  Pabelli
  312185: ..C......................A........................  Nleucogenys
  388169: G.................A......A.......G................  Mmulatta
  340715: ..................A......A........................  Panubis
  370946: G........................A......G....GA...........  Cjacchus
 1021052: G...............................G...TGA...........  Sboliviensis
```

```
                                  LINE2
          ------------------------------------------------------->
                 21113      21123                  21142      21152
   21104: AATGAGAAAAGTAAAACAGCAAAGAGGGA-TTGAGAGTGTGTACTGAGGT  Hsapiens
  340851: .............................-.....G..............  Ptroglodytes
  358827: .............................-....................  Ggorilla
  391004: .G...........................-..--................  Pabelli
  312235: .............................-....................  Nleucogenys
  388219: G............................-........C...........  Mmulatta
  340765: G............................-....................  Panubis
  370996: ............C..............AG-C..........C......TG  Cjacchus
 1021102: ............C..............A.C..A...............TG  Sboliviensis
```

```
          LINE2

              21162     21172     21182     21192     21202
   21153: TGCAATTTAGATAGAAGCTGAAGCTCAGAGTCTGATATCTCTAAGTGGGA   Hsapiens
  340900: ..................................................   Ptroglodytes
  358876: ..................................................   Ggorilla
  391051: ..............................C...................   Pabelli
  312284: ...........C.......C..........C...G...............   Nleucogenys
  388268: ...................C..........C.............A.....   Mmulatta
  340814: ...................C..........C...........G.......   Panubis
  371045: ..TG----------------------------------------------   Cjacchus
 1021152: ..TG----------------------------------------------   Sboliviensis

              21212     21222     21228     21238     21248
   21203: ATTACGAGCATGGCCAGGGTCCCA----GATGGACCCCTGTGATTATGCA   Hsapiens
  340950: ....T...................----.....................T   Ptroglodytes
  358926: ....T...................----.......A.............T   Ggorilla
  391101: .....................T..GATG......................   Pabelli
  312334: ....T...................----..................C...   Nleucogenys
  388318: ....G...................----............G.........   Mmulatta
  340864: ....G...................----............G.........   Panubis
  371049: -------------------------------------.............   Cjacchus
 1021156: -------------------------------------.........C...   Sboliviensis

              21258     21268     21278     21288     21298
   21249: GGAATTCGGGTGGCATGGGAGCACATGAAGAAGTGGTGAGGGAGCCTAAT   Hsapiens
  340996: ..................A...............................   Ptroglodytes
  358972: ..................................................   Ggorilla
  391151: ..................................................   Pabelli
  312380: .......A............A.............................   Nleucogenys
  388364: .......A............A....G.........C..............   Mmulatta
  340910: .......A............A....G.........C..............   Panubis
  371062: .......A.----------------------------..........G..   Cjacchus
 1021169: .......A.----------------------------......T...G..   Sboliviensis

              21308     21318     21328     21337     21347
   21299: GGAGATTATTTGAATGAGGAAAAAAGAAACATCCTGG-AAAGAATTTATG   Hsapiens
  341046: ............C.........C..............-............   Ptroglodytes
  359022: ............C.........C..............-............   Ggorilla
  391201: .....C......C.........C......T.......-............   Pabelli
  312430: ..G.........C.........C...........G..-...T........   Nleucogenys
  388414: ............C.........C..............-.C........A.   Mmulatta
  340960: ............C.........C..............-.C........A.   Panubis
  371084: ...........TC........GC.G............A...A......C.   Cjacchus
 1021191: ............C........GC..............-............   Sboliviensis

              21357     21367     21377     21387     21396
   21348: CTCAAGTGCAGAGTCAGAGTCAGGATGTCCATGACCAAAGGAC-GTGGTT   Hsapiens
  341095: ........A..................................-A.....   Ptroglodytes
  359071: ........A..................................-...A..   Ggorilla
  391250: ........A..........................T.......-A.....   Pabelli
  312479: ........AG.................................A.G....   Nleucogenys
  388463: .A......A..................................A......   Mmulatta
  341009: ........A..................................A......   Panubis
  371134: ....G--.A..................................A....C.   Cjacchus
 1021240: ...--...A.............T....................A....C.   Sboliviensis
```

```
              21406     21416     21426     21436     21446
                  :         :         :         :         :
   21397: CAGGCTGGGAGATGAGGGGTGATTATGCAAGCAGCCCACCTGTCTGCTCA  Hsapiens
  341144: ..................................................  Ptroglodytes
  359120: ..................................T...............  Ggorilla
  391299: ..................................................  Pabelli
  312529: ..........A....A.................A........C.......  Nleucogenys
  388513: ...TT....................G.T.........-T..C........  Mmulatta
  341059: .........................G....A......-T..C........  Panubis
  371182: G.......A......A..................................  Cjacchus
 1021288: G.......A......A........................A.........  Sboliviensis

              21456     21466     21476     21486     21496
                  :         :         :         :         :
   21447: GGTCATGTTCCTCTCCCCACCACAGTCTGAATCTCAGAGAGAAAAGGCAC  Hsapiens
  341194: .....................................G............  Ptroglodytes
  359170: .....................................G........A...  Ggorilla
  391349: ....G................................G............  Pabelli
  312579: ...........-..G..........G...........G............  Nleucogenys
  388562: .....A.C.....C..AG...................GA...........  Mmulatta
  341108: .....A.C.....C..AG...................GA...........  Panubis
  371232: ............AC....................................  Cjacchus
 1021338: ............AC.T................T.....A...........  Sboliviensis

              21506     21516     21526     21536     21546
                  :         :         :         :         :
   21497: CATGGAACAGTTCAAAGAACTTCTGCAACATCTGAACAAGACTGGGAGGT  Hsapiens
  341244: ..................................................  Ptroglodytes
  359220: ..................................................  Ggorilla
  391399: ..................................................  Pabelli
  312628: ......................G....C......................  Nleucogenys
  388612: ...-.......................C......................  Mmulatta
  341158: ...........................C.....A................  Panubis
  371282: ...........................C......................  Cjacchus
 1021388: ..................G........C......................  Sboliviensis

              21556     21566     21576     21586     21596
                  :         :         :         :         :
   21547: CAAGTCAGCCCTGATTAGAATGACAGAATGGGATTACAGCCAACAGATGG  Hsapiens
  341294: .......................................A..........  Ptroglodytes
  359270: ..........................................G......A  Ggorilla
  391449: A.................................................  Pabelli
  312678: .............G.................................C..  Nleucogenys
  388661: ...........CAG..G...............GA................  Mmulatta
  341208: ...........CAG..G................A................  Panubis
  371332: ....G........G..G......T.........-...........AG...  Cjacchus
 1021438: ....G........G..G................A............G...  Sboliviensis

              21606     21616     21626     21636     21646
                  :         :         :         :         :
   21597: TGAGAGTCTTGCCACCCCAGCTTCTACAACCAGCCCAGAAATACCTGACA  Hsapiens
  341344: .......................................C..........  Ptroglodytes
  359320: ..................................................  Ggorilla
  391499: ..............................G...................  Pabelli
  312728: ..............................G...................  Nleucogenys
  388711: ..........C...................G...................  Mmulatta
  341258: ..........C...................G...................  Panubis
  371381: .....A....CT...T..........T...G...A............T..  Cjacchus
 1021488: ..........CT...T.............TG...A............T..  Sboliviensis
```

```
              21656     21666     21676     21686     21696
                  :         :         :         :         :
   21647: ACATCAAATTCTCAAGCCCTATGGGCAGGAGCAGCAGCTCCAGGAACTCA  Hsapiens
  341394: ....................G.............................  Ptroglodytes
  359370: ..................................................  Ggorilla
  391549: ....................G.............................  Pabelli
  312778: ..............................A...............A...  Nleucogenys
  388761: ..................T...............................  Mmulatta
  341308: ..................T........A...................A..  Panubis
  371431: ............T.......G.CA...A...T..................  Cjacchus
 1021538: ............T.......G.CA.......TC..........A......  Sboliviensis

              21706     21716     21726     21736     21746
                  :         :         :         :         :
   21697: GGGACATTTGGCCAGAAGCAGTTGTAGACATGGCAACATCAGGAGGGTCA  Hsapiens
  341444: ..................................................  Ptroglodytes
  359420: ..................................................  Ggorilla
  391599: .......................C..A......................G  Pabelli
  312828: .....................G.C..........................  Nleucogenys
  388811: ......CC...............C.........................G  Mmulatta
  341358: ......CC...............C.........................G  Panubis
  371481: ......CC..A...T........C............T.G...CG.CA.TG  Cjacchus
 1021588: ......CC..A...T........C..............G...CG.....G  Sboliviensis

              21756     21766     21776     21786     21796
                  :         :         :         :         :
   21747: GACCTTCATGAACCTGTCAGCTAAGTCATAGGCTTCACCATTGTTCTGTG  Hsapiens
  341494: ..................................................  Ptroglodytes
  359470: ..................................................  Ggorilla
  391649: ..................................................  Pabelli
  312878: ..................................................  Nleucogenys
  388861: .........AG................G......C...............  Mmulatta
  341408: ..........G..............G........................  Panubis
  371531: ..........T...........C.C...C......A..............  Cjacchus
 1021638: ..........T..............................C........  Sboliviensis

              21806     21816     21824     21834     21844
                  :         :         :         :         :
   21797: AGCTCAGGACATGGGAGCAGATTTT--ACACTGAGTAAAGCACGAGGAGT  Hsapiens
  341544: .....C...................--...............T.......  Ptroglodytes
  359520: .........................--...............T.......  Ggorilla
  391699: .........................--...............T.......  Pabelli
  312928: .C..............A........-T..............CT.......  Nleucogenys
  388911: ..............A..........--T..............T.......  Mmulatta
  341458: ..T...........A..........--T..............T.......  Panubis
  371581: .........................AA.A.............T.......  Cjacchus
 1021688: ...........C.A.......C...AA.A.............T.......  Sboliviensis

              21854     21864     21874     21884     21894
                  :         :         :         :         :
   21845: TCAGGAAGAGTGGTGAATCTCAGAGAGGGTTTGGGGGAACCAGAAGAAGG  Hsapiens
  341592: ..................................................  Ptroglodytes
  359568: ..................................................  Ggorilla
  391747: ..G..............................C..............A.  Pabelli
  312977: ..G........................................G......  Nleucogenys
  388959: ..........G...C...........................AG......  Mmulatta
  341506: ..G.......G...C............................G......  Panubis
  371631: ......C..A.................A.....A..C......G......  Cjacchus
 1021738: .........A................A......A.........G......  Sboliviensis
```

```
               21904     21914     21924     21934
  21895: AGGAGTGGGATTCAGAAGAACACACAGTTATCAGTGGGATCTAATC----  Hsapiens
 341642: .................A............................----  Ptroglodytes
 359618: ..............................................----  Ggorilla
 391797: ......................G.......................----  Pabelli
 313027: .A......................G.....................----  Nleucogenys
 389009: ...................--...................T.....----  Mmulatta
 341556: ...................--...................T.....----  Panubis
 371681: ......A..........---..........................AGAA  Cjacchus
1021788: ......A........C..............A...............AGAA  Sboliviensis

               21949     21959     21969     21979     21989
  21941: -CAGACTATTAGTTTGTTGTCATCTGGAAGACACAGGCTATAAGGGTGAT  Hsapiens
 341688: -......................................G..........  Ptroglodytes
 359664: -.................................................  Ggorilla
 391843: -.................................................  Pabelli
 313073: -..................CTG............................  Nleucogenys
 389053: -.....C.............TG.....................A......  Mmulatta
 341600: -......C........C...TG............................  Panubis
 371728: A...........C.........C............A..............  Cjacchus
1021838: A...........C......................A........A.....  Sboliviensis

               21999     22009     22019     22029     22039
  21990: ACAAACATTTAATTAACAAAAAGCAGAATTTACAAAAACAAATCTCTGGC  Hsapiens
 341737: .....G.......C.................G..................  Ptroglodytes
 359713: ..................................................  Ggorilla
 391892: .....G....................................-.....T.  Pabelli
 313122: .....G............................................  Nleucogenys
 389102: .A...G.....................................A......  Mmulatta
 341649: .....T......G.....................................  Panubis
 371778: .....G.......----........A...................G..A.  Cjacchus
1021888: .....G.......-----.......A...................G..A.  Sboliviensis

                                           LINE1
                                      ------------------->
               22043     22053     22063     22073     22081
  22040: TC------GGATATAGAACCTGGAGTCTCTAAATACTGCATGT--TCTCT  Hsapiens
 341787: ..---------------..........................--.....  Ptroglodytes
 359763: ..------...................................--.....  Ggorilla
 391941: ..------A.G...........................T....--.....  Pabelli
 313172: ..------A...G..................-------------------  Nleucogenys
 389152: ..------A......T...........................--.....  Mmulatta
 341699: ..------A..................................--.....  Panubis
 371824: ..AGATATA...........................CA...A.--.....  Cjacchus
1021933: ..AGATATA..C...............................TC.....  Sboliviensis

                          LINE1
         ------------------------------------------------->
               22091     22101     22111     22121     22131
  22082: TATAAATGGGAACTAAACAATGGGTCCACGTGAATGTAAAGATGGAAATA  Hsapiens
 341820: .....G............................................  Ptroglodytes
 359805: .....G.....................................A......  Ggorilla
 391983: .....G............................................  Pabelli
 313197: --------------------------------------------------  Nleucogenys
 389194: .........C........................................  Mmulatta
 341741: ........A.........................................  Panubis
 371872: .....G.....G...G...G.........A.........T..........  Cjacchus
1021983: .....G.....G...G.............A.A..................  Sboliviensis
```

```
           LINE1
          ------------------------------------------------->
                    22141     22151     22161     22171     22181
   22132: ATAGACACTGGGGACTCCAGAAAGGTGGAAGGGTAAGGGGGCAATGATTG   Hsapiens
  341870: .A..........................G.....................   Ptroglodytes
  359855: ............................G.....................   Ggorilla
  392033: ............................G.....................   Pabelli
  313197: --------------------------------------------------   Nleucogenys
  389244: .........................G..G........A............   Mmulatta
  341791: .........T...............-..G........A............   Panubis
  371922: ............A...........AG..T....C.G..............   Cjacchus
 1022033: ........................AG..T......T........G.....   Sboliviensis
```

```
           LINE1
          ------------------------------------------------->
                    22191     22201     22211     22221     22231
   22182: AAAAATTACCTATCAGGTACAATGTTCACTGTATGGGTCATGGGTACATT   Hsapiens
  341920: ..................................................   Ptroglodytes
  359905: ....................C........C....................   Ggorilla
  392083: ......C...........................................   Pabelli
  313197: --------..........................................   Nleucogenys
  389294: ......C.T---..................A.................C.   Mmulatta
  341840: ...........G..................A.................C.   Panubis
  371972: -..................T..........A....T............CC   Cjacchus
 1022083: .......G.....................CA.........C.......CC   Sboliviensis
```

```
           LINE1
          ------------------------------------------------->
                    22241     22251     22261     22271     22281
   22232: AGAAGCCCAATCCCCCAAAATTGGCAATATACTCTTTTAACCCCTGAATC   Hsapiens
  341970: ...............A.T................................   Ptroglodytes
  359955: ...............A..................................   Ggorilla
  392133: .....A...G.....A.C................................   Pabelli
  313239: ...............A.C.G.....G............--..........   Nleucogenys
  389341: -.G............A.C.....A......................G...   Mmulatta
  341890: -.G............A.C.....A......................G...   Panubis
  372021: G..........T..AA.C....T.............G.............   Cjacchus
 1022133: ...........T..AA.C....T..........G..G...A......G..   Sboliviensis
```

```
           LINE1
          ---------------------------->
                    22291     22299     22308     22318     22328
   22282: TGAAATAAATTTTT--AAGAG-AAAAAACCTGGAAACCCTGCCAAATCCC   Hsapiens
  342020: ..............--.....-............................   Ptroglodytes
  360005: ..............--.....-............................   Ggorilla
  392183: .A............--.....A...............G............   Pabelli
  313287: .A............--.....-........A...................   Nleucogenys
  389390: .A........G...AA.....-............................   Mmulatta
  341939: .A........G...AA.....-............................   Panubis
  372071: .A...........A--..A..-...G...G.................T..   Cjacchus
 1022183: .A............AA..AT.-...G.....................T..   Sboliviensis
```

```
                                        Simple
                                    ---------------------------->
                   22338     22348     22358     22368
                       :         :         :         :
   22329: AGTCTGGTAAATTCCCACCCAACAGTTTGCTTTTTTGTTTTGTTTT----  Hsapiens
  342067: ..................T...........................----  Ptroglodytes
  360052: ..............................................GTTT  Ggorilla
  392231: ....CA........................................----  Pabelli
  313334: ......................T..............C....A...----  Nleucogenys
  389439: .................A..........TT.G..............----  Mmulatta
  341988: .................A..........TT.G..............----  Panubis
  372118: ..........................                            Cjacchus
 1022232: ..........................                            Sboliviensis

                     Simple                        Alu
          ------------------------------------> ------------->
                   22375     22385     22395     22405     22415
                       :         :         :         :         :
   22375: ---------GTTTGTTTGTTTGTTTGTTTGTTTGTTCTGAGACAGAGTCT  Hsapiens
  342113: ------------......................................  Ptroglodytes
  360102: TGTTTGTTT....................T...A................  Ggorilla
  392277: ---------------......C..............T...A..G......  Pabelli
  313380: -------------------.................T.....TG......  Nleucogenys
  389485: -------------------...G..-----...T..T.....TG......  Mmulatta
  342034: -------------------......T..GT...T..T.....TG......  Panubis

                                  Alu
          -------------------------------------------------->
                   22425     22435     22445     22455     22465
                       :         :         :         :         :
   22416: CGCTCTGTCATCCAAGCTGGAGTGCACTGGCGCTATCTCGGCTCACCGCA  Hsapiens
  342151: .A............................T...............T...  Ptroglodytes
  360152: ..................................................  Ggorilla
  392312: T.........C...G...........G...T..G.....A......T...  Pabelli
  313411: ..........C...G...........G.....TG....T.......T...  Nleucogenys
  389511: .........GC...G...........G....CGG............T...  Mmulatta
  342065: .........GC...G...........G....CGG............T...  Panubis

                                  Alu
          -------------------------------------------------->
                   22475     22485     22495     22505     22515
                       :         :         :         :         :
   22466: AGCCCCACCTCCCAGGTTCACGCCATTCTCCTGCCTCAGCCTCCTGAGTA  Hsapiens
  342201: ....................T.............................  Ptroglodytes
  360202: ..................................................  Ggorilla
  392362: ...........T........T.....................T.......  Pabelli
  313461: .....................A.....................T...T..  Nleucogenys
  389561: ...T..G......G.....C........................C.....  Mmulatta
  342115: ...T..G......CA....C........................C.....  Panubis

                                  Alu
          -------------------------------------------------->
                   22525     22535     22544     22554     22564
                       :         :         :         :         :
   22516: GCTGGGACTACAGGCGCCCACCACCAC-CCCGGCTAATTTTTTGTATTTT  Hsapiens
  342251: ...........................-......................  Ptroglodytes
  360252: ..............A............-......................  Ggorilla
  392412: ...................G.......-......................  Pabelli
  313511: .A.............A...G.......-......................  Nleucogenys
  389611: ...............A...G.......G......................  Mmulatta
  342165: ...................G......TG......................  Panubis
```

```
                                          Alu
          ------------------------------------------------------------------>
                    22574     22583     22593     22603     22613
                        :         :         :         :         :
  22565: TAGTAGATAT-GGGTTTCACCGTGTTAGCCAGAATCGTCTCAATCTCCTG  Hsapiens
 342300: .....C...C-A.............................G........  Ptroglodytes
 360301: .........C-.........T....................G........  Ggorilla
 392461: .....A.G.CG.....................T.......TG........  Pabelli
 313560: .......G.CA.....................G.......TG........  Nleucogenys
 389661: .......G.CG.....................GT.G.....G.....TG.  Mmulatta
 342215: .......G.CG.....................G..G.....G.....TG.  Panubis

                                          Alu
          ------------------------------------------------------------------>
                    22623     22633     22643     22653     22663
                        :         :         :         :         :
  22614: ACCTCATGATCTGCCCGCCTCAGCCTCCCAAAGTGCTGGGATTACAGGTG  Hsapiens
 342349: ..................................................  Ptroglodytes
 360350: ...............T..................................  Ggorilla
 392511: ......C........T.....G............................  Pabelli
 313610: .....G.....CA........................A............  Nleucogenys
 389711: .....G..........A....G........G.................C.  Mmulatta
 342265: .....G...........G...G..........................C.  Panubis

                   Alu
          ----------------------->
                    22673     22683     22693     22703     22713
                        :         :         :         :         :
  22664: TGAGCCACCGCGCCCAGCCCCCCAACAGTCTTATTACCTCTTAACAAAGA  Hsapiens
 342399: ..........T....G..................................  Ptroglodytes
 360400: ........T...T..G.....G............................  Ggorilla
 392561: ..............TG.............G....................  Pabelli
 313660: ...A..G..A....TG..................................  Nleucogenys
 389761: .....T...AT.................C.....................  Mmulatta
 342315: .....T....T.................C.....................  Panubis
 372132:                  ..A..........G.................C.  Cjacchus
1022246:                  ..A..........G.................C.  Sboliviensis

                    22723     22733     22743     22753     22763
                        :         :         :         :         :
  22714: GACTTTTTAACAATGATGGTCAAAGATTCTCCAAGACCGTGCTGGGGATT  Hsapiens
 342449: ..................................................  Ptroglodytes
 360450: ..................................................  Ggorilla
 392611: .......C......T.C..........G...GG.................  Pabelli
 313710: .......C..................CG..TGG.................  Nleucogenys
 389811: .......C....G..............G..TGG....T........A.A.  Mmulatta
 342365: .......C....G..............G..TGG.A..T........A.A.  Panubis
 372165: .......C....C...........C..G..TGG....T..........A.  Cjacchus
1022279: .......C....T.....A.......CG..TGG....T..........A.  Sboliviensis

                    22773     22783     22793     22803     22813
                        :         :         :         :         :
  22764: TCTTGGCATGGTCACGTCATGGTTATTGTGGATGACTATGACTCATGTCC  Hsapiens
 342499: ..............TT...............G..................  Ptroglodytes
 360500: ..............T................G..................  Ggorilla
 392661: ........C.....T................G..................  Pabelli
 313760: ..............T................G.............C....  Nleucogenys
 389861: ..............T..............A.G................T.  Mmulatta
 342415: C.............T..............A.G................T.  Panubis
 372215: ...........C..T...............A..T................  Cjacchus
1022329: ..............T...............A..T................  Sboliviensis
```

```
                 22823     22833     22843     22853     22863
                     :         :         :         :         :
  22814: AAATTACTGGACAATTCCATCACCCAGTTAAGCACCTAATGGTGCCTGTC  Hsapiens
 342549: ..................................................  Ptroglodytes
 360550: ..................................................  Ggorilla
 392711: .........A........................................  Pabelli
 313810: ...............................A.............A....  Nleucogenys
 389911: ..................................................  Mmulatta
 342465: ..................................................  Panubis
 372265: ...............................A........A.........  Cjacchus
1022379: ......................T........A........A.........  Sboliviensis

                 22873     22883     22893     22903     22913
                     :         :         :         :         :
  22864: ACTGTGACACCTAAATTGTCAGAGATGCTGTCTACACATAACCAAAATAA  Hsapiens
 342599: ...........C......................................  Ptroglodytes
 360600: ...........C......................................  Ggorilla
 392761: .........T.C......................................  Pabelli
 313860: .....A.....C.....................G................  Nleucogenys
 389961: ...........C.............C...ACA..................  Mmulatta
 342515: ...........C.................ACA..................  Panubis
 372315: .T.........C.................A......T..G..........  Cjacchus
1022429: ...........C.................A....T.T.............  Sboliviensis

                 22923     22933     22943     22953     22963
                     :         :         :         :         :
  22914: ACCATGAATAGGTATTTTATCCCCACCTTCAAAATGTTCTAGTTCTGCCA  Hsapiens
 342649: ..................................................  Ptroglodytes
 360650: ..................................................  Ggorilla
 392811: ........C.........................................  Pabelli
 313910: ........C.........................................  Nleucogenys
 390011: ....CC..                                            Mmulatta
 342565: ....CC..                                            Panubis
 372365: ........C.........................................  Cjacchus
1022479: ...G....C..A......................................  Sboliviensis

                 22973     22983     22993     23003     23013
                     :         :         :         :         :
  22964: AACTGATTACTAGCATGTAAAATGCTCCTCTGTGATTTATTTCATGTAGT  Hsapiens
 342699: ..................................................  Ptroglodytes
 360700: ..................................................  Ggorilla
 392861: .................................T............C...  Pabelli
 313960: ..G......................C....................C...  Nleucogenys
 372415: ......--.........G....C....--.................C...  Cjacchus
1022529: G.....--.........G..............--............C...  Sboliviensis

                 23023     23033     23043     23053     23063
                     :         :         :         :         :
  23014: TTATAATGTACCTTTCCCGTTATAAATATGAGCATCTGGAAGCCCAAATG  Hsapiens
 342749: ..................A...............................  Ptroglodytes
 360750: ..................................................  Ggorilla
 392911: ........C.........A.......C................T......  Pabelli
 314010: ........C.........A.........C.....................  Nleucogenys
 372461: ........CC........T......G.....C..........G.......  Cjacchus
1022575: ........C.......T.T......G.....C..C...............  Sboliviensis
```

```
               23073     23083     23093     23103     23113
                   :         :         :         :         :
   23064: ACTTTTCTAGAATGCAAATCTGACCAAGTCCCTATCTTAGGAAGAGAACC  Hsapiens
  342799: ..................................................  Ptroglodytes
  360800: .............A....................................  Ggorilla
  392961: .............A...................C................  Pabelli
  314060: .............A............G.......................  Nleucogenys
  390014:                       ...........G...........C.G..  Mmulatta
  342568:                       .......................C.G..  Panubis
  372511: ..C..........A..............C..................G..  Cjacchus
 1022625: ..C..........A..C............T.................G..  Sboliviensis

               23123     23133     23143     23153     23163
                   :         :         :         :         :
   23114: CTCACTCTTTGAAATTGGGTCTACTAAAAGGACCCTGCCTATAACAGTCT  Hsapiens
  342849: ..................................................  Ptroglodytes
  360850: ..............C...................................  Ggorilla
  393011: .....................C............................  Pabelli
  314110: .....................C.....................G......  Nleucogenys
  390042: .....................C............................  Mmulatta
  342596: .....................C............................  Panubis
  372561: .....A...G..G...A....C.....................G....T.  Cjacchus
 1022675: .....A...G......AA...C..........................T.  Sboliviensis

                                                    LINE2
                                                  -------->
               23173     23183     23193     23203     23213
                   :         :         :         :         :
   23164: TCTCATGACACCGATTACCCTATTGTCATTACTACCCTAAAGCTGTCTTC  Hsapiens
  342899: .......................-....................C.....  Ptroglodytes
  360900: ....................A.............................  Ggorilla
  393061: ....................A....................A........  Pabelli
  314160: ....................A.............................  Nleucogenys
  390092: .........G..A.......A...........................G.  Mmulatta
  342646: ............A.......A...........................G.  Panubis
  372611: ......A.....A.....A.G..G.....................A....  Cjacchus
 1022725: ............A.....A.A.........G...T...............  Sboliviensis

                                LINE2
          ------------------------------------------------->
               23223     23233     23243     23253     23263
                   :         :         :         :         :
   23214: TCTGCCAAAATATAAATCCTCGCCTTATAAAGACAAGGACTGAGGCTTTG  Hsapiens
  342948: ..................................................  Ptroglodytes
  360950: ..................................................  Ggorilla
  393111: ...............G....T.......G.....................  Pabelli
  314210: ...............G....T.......G.....................  Nleucogenys
  390142: ..G...------------------....G.....................  Mmulatta
  342696: ......------------------....G......C..............  Panubis
  372661: ......G.T.....C.-------.....G....G................  Cjacchus
 1022775: ..............TG-------..C..G.....................  Sboliviensis
```

LINE2

```
                        23273     23283     23293     23303     23313
                            :         :         :         :         :
   23264: TGTTATTCACTGTATATCTCTGTGACTTAACATGGTATCTGGGCCAGAAT Hsapiens
  342998: .................................................. Ptroglodytes
  361000: .....G............................................ Ggorilla
  393161: .................................A............T... Pabelli
  314260: .............T................A...A...........T... Nleucogenys
  390174: ...........C.G................................T... Mmulatta
  342728: ..............................................T..C Panubis
  372704: A................T............................T... Cjacchus
 1022818: .T.....................C......................T... Sboliviensis
```

LINE2 / Alu

```
                        23323     23333     23343     23353     23363
                            :         :         :         :         :
   23314: TGGTGCTCAATAAATGTTTGTTGAAGAAATTAATCTGCTGGGTAGGGTGG Hsapiens
  343048: .................................................. Ptroglodytes
  361050: .................................................. Ggorilla
  393211: .................................................. Pabelli
  314310: ....................C............................. Nleucogenys
  390224: .................................................. Mmulatta
  342778: ................G................................. Panubis
  372754: ..........G.............................A...CAC... Cjacchus
 1022868: ..........G.............................A...CAC... Sboliviensis
```

Alu

```
                        23373     23383     23393     23403     23413
                            :         :         :         :         :
   23364: TGCACACCTGTAATCAGAGCTACTTGGGAGAATGAAGCAGGAGGATAGCT Hsapiens
  343098: .................................................. Ptroglodytes
  361100: .................................................. Ggorilla
  393261: .........A.....CC....G............................ Pabelli
  314360: ..T.............C............................C.... Nleucogenys
  390274: ...............CC........................G........ Mmulatta
  342828: ...............CC........................G........ Panubis
  372804: ....T..........CC................................. Cjacchus
 1022918: ....T..........CC.................G.............T. Sboliviensis
```

Alu

```
                        23423     23433     23443     23453     23463
                            :         :         :         :         :
   23414: TGAGCCCAGTAGTTTAAGACCAGCCTTGACAAAATAGTGAGCTCTTGTCT Hsapiens
  343148: ...........................................T...... Ptroglodytes
  361150: ................................................G. Ggorilla
  393311: .........................................A........ Pabelli
  314410: ...........................A...................... Nleucogenys
  390324: .A..T..............................G.....A........ Mmulatta
  342878: ....T..............................T.....A........ Panubis
  372854: .C........G.........TCA.....G......G.....A........ Cjacchus
 1022968: ..........G.........TCA.....G............A........ Sboliviensis
```

```
              Alu
          ----------->
               23473     23483     23493     23503     23513
                   :         :         :         :         :
   23464: CAAAAAATAAATGAAATTGAATAAGGGTGTCAATGAATTTTTAAAATGAG Hsapiens
  343198: .................................................. Ptroglodytes
  361200: ...........................A....................G. Ggorilla
  393361: .........................A...............A........ Pabelli
  314460: .................................................. Nleucogenys
  390374: .................................................A Mmulatta
  342928: .................................................A Panubis
  372904: ............A...........C.................GG...... Cjacchus
 1023018: ............A.............................G....... Sboliviensis

               23523     23533     23543     23553     23563
                   :         :         :         :         :
   23514: AATCTTTTAAAGTCATTTACTCTTGAGCAGTAAATCTATTGTTGGCTAGA Hsapiens
  343248: .................................................. Ptroglodytes
  361250: ..............G................................... Ggorilla
  393411: ............CTG..........G.......................G Pabelli
  314510: .......A.....T.............................A...... Nleucogenys
  390424: ..........G..............-T....................... Mmulatta
  342978: ..........G..............-T....................... Panubis
  372954: ...........A.............G..........--............ Cjacchus
 1023068: .--..C.....A.............G...T..C................. Sboliviensis

               23573     23583     23593     23603     23612
                   :         :         :         :         :
   23564: GGTGTATGTTCCTTTTTGGGTCCTGTTGAGGCCAAGCTGGGTACCTCA-T Hsapiens
  343298: ................................................-. Ptroglodytes
  361300: ................................................-. Ggorilla
  393461: .A..............................................-. Pabelli
  314560: .................................G..............-. Nleucogenys
  390473: ..........................C.....................T. Mmulatta
  343027: ................................................-. Panubis
  373002: ..CA.....                                          Cjacchus
  373986:          ........T...--....A..A........A........-. Cjacchus
 1023116: ...A..................T............C...A....T...-. Sboliviensis

               23622     23632     23642     23652     23662
                   :         :         :         :         :
   23613: TGATCTCTGCTTTCTCTGTTGACACAAAGAAGGAACAAGTGCCATCAGTG Hsapiens
  343347: .................................................. Ptroglodytes
  361349: .......C.......................................... Ggorilla
  393510: ...................C.............................. Pabelli
  314609: .................................................. Nleucogenys
  390523: .............T.................................... Mmulatta
  343076: .............T.................A...............C.. Panubis
  374024: .........................G......A................. Cjacchus
 1023165: G..........G.........................C............ Sboliviensis

               23672     23682     23692     23702     23712
                   :         :         :         :         :
   23663: GATAGGATAAAAGGGGAAGTTTCCTCTACAAAACTAGGGTACCAGAGGAA Hsapiens
  343397: ....................................A............. Ptroglodytes
  361399: ...................C................A............. Ggorilla
  393560: ............T......C......C.........A.......C..... Pabelli
  314659: ............C......C................A.......C..... Nleucogenys
  390573: ...................C................A.......T..... Mmulatta
  343126: ...................C................A.......T..... Panubis
  374074: .G.G...............C...........C....A.......TG.... Cjacchus
 1023215: .G.................C................A.......CG.... Sboliviensis
```

```
                   23722     23732                       23750
                       :         :                           :
   23713: GAAGACAGAGGTCAAGAGAGTGTGATGTG------------TAAAACTAC  Hsapiens
  343447: .............................------------.........  Ptroglodytes
  361449: .........A...................------------.........  Ggorilla
  393610: .............................------------.........  Pabelli
  314709: .....................A.......------------.......G.  Nleucogenys
  390623: ..........TG..............C..------------.........  Mmulatta
  343176: ...........G...........A..C..------------.........  Panubis
  374124: .C.......A.G......--.........GCAGCAAATCTG.........  Cjacchus
 1023265: ...........G...............G.-CAGCAAATCTG.........  Sboliviensis

                   23760     23770     23780     23790     23800
                       :         :         :         :         :
   23751: ATCAGTTTTCTGCATAAAGTGTTTCAAAACATGGAAGAAAATAATAAAAG  Hsapiens
  343485: ..................................................  Ptroglodytes
  361487: ..................................................  Ggorilla
  393648: ..A...............A...............................  Pabelli
  314747: ..................................................  Nleucogenys
  390661: ..............C...A....................T......G..A  Mmulatta
  343214: ..................A....................T......G...  Panubis
  374172: .........-....C.........A.........T...........G...  Cjacchus
 1023314: .........-.A...G........A...........T.........G...  Sboliviensis

                   23810     23820     23830     23840     23850
                       :         :         :         :         :
   23801: TGGAGAAAAATTAGAAAATCACTCATGTGTATAGATGCTACTGGCCTGAG  Hsapiens
  343535: ..................................................  Ptroglodytes
  361537: ..................................................  Ggorilla
  393698: .........G............................C......G....  Pabelli
  314797: ......................................C...........  Nleucogenys
  390711: ....A....T...............--...........C...........  Mmulatta
  343264: ....A....T...............--...........C...........  Panubis
  374221: .....................T......A......G..C...A.......  Cjacchus
 1023363: .....................T...........T....C...A.......  Sboliviensis

                   23860     23870     23880     23890     23900
                       :         :         :         :         :
   23851: TTTATGACAAAATGCGGTGGAGAACTGAGAAAATAAAGACTCCTAAATAT  Hsapiens
  343585: ..............A...................................  Ptroglodytes
  361587: ..............A...................................  Ggorilla
  393748: ..............A...........................T.......  Pabelli
  314847: ..............A........T................G.........  Nleucogenys
  390759: ..............AA..................................  Mmulatta
  343312: ..............AA..................................  Panubis
  374271: ..............AA..A..A..............T...-.........  Cjacchus
 1023413: C.............AA..A..A...--G............-.........  Sboliviensis

                                         LINE1
                               ------------------------------->
                   23910     23920     23930     23940     23950
                       :         :         :         :         :
   23901: CCTCACTTGTGTTATTGAGTTAAACGGAAGTTGGAAAAGATTCAGAATAG  Hsapiens
  343635: ........................G.........................  Ptroglodytes
  361637: ........................G.........................  Ggorilla
  393798: ........................A.........................  Pabelli
  314897: ........................G......................C..  Nleucogenys
  390809: ........................G.........................  Mmulatta
  343362: ........................G.........................  Panubis
  374320: ........................AA.......C.............C.C  Cjacchus
 1023460: ..............C.........AA..............C.........  Sboliviensis
```

```
                             LINE1
         ---------------------------------------------------->
                   23960     23970     23980     23990     24000
                       :         :         :         :         :
  23951: CCAAGATAATATTGAAGAAGAGCAAAGTCAGAGGATGCCACAACCTGATT   Hsapiens
 343685: ..................................................   Ptroglodytes
 361687: ......................G...........................   Ggorilla
 393848: ..................................................   Pabelli
 314947: .........C............TC..........................   Nleucogenys
 390859: ..................................................   Mmulatta
 343412: ............................A.....................   Panubis
 374370: ....A.C..............A..............C.............   Cjacchus
1023510: ....A.C..............A..............C.............   Sboliviensis

                   LINE1
         ------------------------------->
                   24010     24020     24030     24040     24050
                       :         :         :         :         :
  24001: TCGAAACTTACTATAAAGCTTCCTTATAAACACTGGCGTAGAATTGGGTG   Hsapiens
 343735: ..................................................   Ptroglodytes
 361737: .T................................................   Ggorilla
 393898: ..A...................T...C...........C...........   Pabelli
 314997: ..A...........-------------C..........C.C.........   Nleucogenys
 390909: ..A..............................C....C...........   Mmulatta
 343462: ..A.....................A........C....C....C......   Panubis
 374420: ..A..............A..................T.C...G.G.....   Cjacchus
1023560: ..A..................A..............T.C...GCG.....   Sboliviensis

                             24068     24078     24088     24098
                                 :         :         :         :
  24051: ACTAGTAGA--GTGTCAGGGGGCTGCTGTTGCCAAGATACCTAGGGCTTC   Hsapiens
 343785: .........--...........T................AG.........   Ptroglodytes
 361787: .........--...........T................A..........   Ggorilla
 393948: .........GT..........-T................A..........   Pabelli
 315034: .........--...........T................A..........   Nleucogenys
 390959: .........--..........-T...C........C...A..........   Mmulatta
 343512: .........--...........T...C............A..........   Panubis
 374470: .........--.........AAT....T.........A.A.C........   Cjacchus
1023610: .....C...--.......C..AT....T.........A.A..........   Sboliviensis

                   24108     24118     24128     24138     24148
                       :         :         :         :         :
  24099: TAACTAGCAGCCCTTTTTCCTGCTGCCTTTTGTAACCGATGGCCAATAGA   Hsapiens
 343833: ..........................................T.......   Ptroglodytes
 361835: .............................................G....   Ggorilla
 393997: .T...................................AG.T.........   Pabelli
 315082: .....................................A............   Nleucogenys
 391006: .T...................................A...A........   Mmulatta
 343560: .T...................................A...A........   Panubis
 374518: AT........T......C.T...............TTA.C..........   Cjacchus
1023658: AT...............C.T..........CA...T.A............   Sboliviensis

                   24158     24168     24178     24188     24198
                       :         :         :         :         :
  24149: TACAGCAGCCTCCAACCATGGCTCAATATCCAGTAGTATTTAGGTAAGGT   Hsapiens
 343883: ..................................................   Ptroglodytes
 361885: .......................................A..........   Ggorilla
 394047: ......................AT.........-------.....T....   Pabelli
 315132: ...T........T.........AT..........................   Nleucogenys
 391056: ........T.............AT..........................   Mmulatta
 343610: ........T.............AT..........................   Panubis
 374568: ....A..A....TG..TG....AT....................A....A   Cjacchus
1023708: C.A.A...G...TG..TG....AT..........G.........A....C   Sboliviensis
```

```
                                       LINE1
                          ------------------------>
               24208     24218     24228     24238     24248
   24199: GACCATATCATTTATCCTCCAAATATTGATCTCCTCCAAATATATTGATC  Hsapiens
  343933: ..................................................  Ptroglodytes
  361935: ........T.........................................  Ggorilla
  394090: .............C..........                            Pabelli
  315182: ..................................................  Nleucogenys
  391106: ........................                            Mmulatta
  343660: ..T.....................                            Panubis
  374618: ........................                            Cjacchus
 1023758: ........................                            Sboliviensis

                          LINE1
          ------------------------------------------------->
               24258     24268     24278     24288     24298
   24249: TCCTTTCTTTTGGGTAAATACCCAGTAGTGGAATTGTTGAGTCATATGGT  Hsapiens
  343983: ........................C.........................  Ptroglodytes
  361985: ...........A......................................  Ggorilla
  315232: ..........................G.......................  Nleucogenys

                          LINE1
          ------------------------------------------------->
               24308     24318     24328     24338     24348
   24299: AGCTCAATTTTTAGTTTTTTGAGGAACCTCCAAACTGTTCTCCATTGTTG  Hsapiens
  344033: ...................................A..............  Ptroglodytes
  362035: ..................................................  Ggorilla
  315282: ................C..................CA...........C.  Nleucogenys

                          LINE1
          ------------------------------------------------->
               24358     24368     24378     24388     24398
   24349: TACTAACTTACATTCTCACCAACAGTGTATGAGGATTCCCTTTTCTCCAT  Hsapiens
  344083: ...............C..................................  Ptroglodytes
  362085: ...............C..................................  Ggorilla
  315332: ...............C..................................  Nleucogenys

                LINE1                         DNA
          ------------------------->       ----------------->
               24408     24418     24428     24438     24444
   24399: ATCCTTGCCAGCATTTGTTATTGCCAGAATTTCACTTTTGAGAGT----G  Hsapiens
  344133: .............................................----.  Ptroglodytes
  362135: .............................................----.  Ggorilla
  394119:                                 .............----.  Pabelli
  315382: .........................T...................----.  Nleucogenys
  391135:                                 .............TTTC.  Mmulatta
  343689:                                 .............TTTC.  Panubis
  374647:                                 .............----A  Cjacchus
 1023787:                                 .....C.....A.----A  Sboliviensis
```

```
                                     DNA
         ───────────────────────────────────────────────────►
                    24451     24461     24471     24481     24491
                        :         :         :         :         :
  24445: AAAT---GGGAAGCTATTTATCATTACACCAGGACAACAGGAGTAGACCA  Hsapiens
 344179: ....---...........................................  Ptroglodytes
 362181: ....---.......G...................................  Ggorilla
 394133: ....---...........G...............................  Pabelli
 315428: ....---....G......G................C..............  Nleucogenys
 391153: C.T.TCA.C.........G...............................  Mmulatta
 343707: C.T.TCA.CA........G..................T............  Panubis
 374661: ....---...........G............T.G................  Cjacchus
1023801: ....---...........G............T..................  Sboliviensis

                             DNA
         ─────────────────────────────────────►
                    24500     24510     24520     24530     24540
                        :         :         :         :         :
  24492: G-ACGGAGGCAGGAATCCCAGGCATATAGTCACCTGTTAATTCAGATATT  Hsapiens
 344226: .G...........................................G....  Ptroglodytes
 362228: .G..C.............................................  Ggorilla
 394180: .G..CA............................................  Pabelli
 315475: .G..C.............................................  Nleucogenys
 391203: .G..T..................G....T.....................  Mmulatta
 343757: .G..CA.................T....T..G..................  Panubis
 374708: AG..CA.....A.GC..........GC.......................  Cjacchus
1023848: .G..CA.....A.G..........AGC.......................  Sboliviensis

                    24550     24560     24570     24580     24590
                        :         :         :         :         :
  24541: TCAAGGGACAACATGTGGGAAAGTAACTAGATTTTCCAAAGGCAGGATTT  Hsapiens
 344276: ...................G...........................A..  Ptroglodytes
 362278: ...................G...........................AC.  Ggorilla
 394230: ...................G...G...............G.......G..  Pabelli
 315525: ...................G...G..A....................G..  Nleucogenys
 391253: ..........G........G...G................C......G..  Mmulatta
 343807: ..........G........G...G.......................G..  Panubis
 374758: ..........G......AAG...G...........T..C...T....G..  Cjacchus
1023898: ......A...G...........AG...........T......T....G..  Sboliviensis

                    24598     24608     24618     24628     24638
                        :         :         :         :         :
  24591: TCTGAGG--TTTTAGTCCCCAGAAAGTTCTCCCCATTAAGTCCTCACTTT  Hsapiens
 344326: .......--.C.......................................  Ptroglodytes
 362328: .......--.........................................  Ggorilla
 394280: ..C....--....T.............G...T..................  Pabelli
 315575: .......--..........TG......G......................  Nleucogenys
 391303: .......--....T..T........A.GT.....................  Mmulatta
 343857: ......A--....C..T........A.GT.....................  Panubis
 374808: C......--....TTC...........GG.AT.......A...C.....C  Cjacchus
1023948: .......TT....TT............GG..T....G......C.....C  Sboliviensis

                                                        24645
                                                            :
  24639: TGCC-------------------------------------------ATT  Hsapiens
 344374: ....-------------------------------------------...  Ptroglodytes
 362376: ....-------------------------------------------...  Ggorilla
 394328: ....-------------------------------------------...  Pabelli
 315623: ....-------------------------------------------...  Nleucogenys
 391351: ....ATTCCATTATTTTCCTGAAAATTATTAAGTCCTCACTTTTGCC...  Mmulatta
 343905: ....-------------------------------------------...  Panubis
 374856: .C..---------------------------------------------C  Cjacchus
1023998: .C..---------------------------------------------C  Sboliviensis
```

```
                 24655     24665     24675                   24687
                     :         :         :                       :
  24646: CTATTCTGCTCCTGAAAATTTCTGAGAAAAAAAAAA--------GATGAC   Hsapiens
 344381: ....................................A-------......   Ptroglodytes
 362383: ...........T........................AAA-----......   Ggorilla
 394335: ....................................AAAAAAAA......   Pabelli
 315630: ..............................T.....AAAA----......   Nleucogenys
 391401: .C.....TT...........................AAAAAAAA......   Mmulatta
 343912: .C.....TT.................G....G....AAAAAAA-......   Panubis
 374861: TC......TA...A.........A..G....T....--------.T....   Cjacchus
1024003: .C.......A...A.................TC...--------.T....   Sboliviensis


                 24697     24707     24717     24727     24737
                     :         :         :         :         :
  24688: AGATCTAGAAGAAGTAGAAGGAAAAAACTAGACTGAGCAGTAAGTACACA   Hsapiens
 344424: ........................C.........................   Ptroglodytes
 362428: ..................................................   Ggorilla
 394385: .......A..................................G.......   Pabelli
 315676: .A.......---..............T.......................   Nleucogenys
 391451: ...........T..............-.......................   Mmulatta
 343961: ...........T..............-.......................   Panubis
 374903: .A................G......C..------A..TG..........G   Cjacchus
1024045: .A................G.....GC..------A..............G   Sboliviensis


                 24742     24748     24758     24768     24778
                     :         :         :         :         :
  24738: GACA-----CT----GTGCCACTGCCCAAATGGTGACTTCTTTTCCCTTT   Hsapiens
 344474: ....-----..----.....................T.............   Ptroglodytes
 362478: ....-----..TACT...................................   Ggorilla
 394435: ....-----..TACT..............................A....   Pabelli
 315723: ....-----..TACT..............................A....   Nleucogenys
 391500: ....-----..TACT........................A.....A....   Mmulatta
 344010: ..A.-----..TACT..............................A....   Panubis
 374947: ....AATCT..TCCT...T.........G......G.........A....   Cjacchus
1024089: ....AATCT..TCCT...T................G.........A....   Sboliviensis


                 24788     24798     24807     24817     24827
                     :         :         :         :         :
  24779: GTCTTTTAAATTTACATCAAGTTACCC-TGGAGGAGCCTCTGAGCAGAAA   Hsapiens
 344515: ...........................-......................   Ptroglodytes
 362523: ....................A......-......................   Ggorilla
 394480: ..............T......C.....-......................   Pabelli
 315768: ...........................-......................   Nleucogenys
 391545: ...................G.......C......................   Mmulatta
 344055: ...................G.......C......G...............   Panubis
 374997: ..........C................-.....A....A.A..T....G.   Cjacchus
1024139: ..........C................-.....A.C..A.A.......G.   Sboliviensis


                 24837                   24846     24856     24866
                     :                       :         :         :
  24828: CACCTCATTAA-----------AGTTACCTTTTGTGTGCACTATGCCCTT   Hsapiens
 344564: ...........-----------...................C........   Ptroglodytes
 362572: ...........-----------............................   Ggorilla
 394529: ...........-----------............................   Pabelli
 315817: ...........-----------............................   Nleucogenys
 391595: ...TC.T.G..-GTTTTCTTTA....GT....C....A............   Mmulatta
 344105: .........G.AGTTGTCTTTA....GT....C.................   Panubis
 375046: ....C......-----------....G...............CCTT....   Cjacchus
1024188: ....-..C...-----------....G...............C.......   Sboliviensis
```

```
                       24876     24886     24896     24906     24916
                           :         :         :         :         :
   24867: TCCATGACCCACTGGTGCCCTGTGGCTAAGAATCTGGTTTGATGCTGCTG   Hsapiens
  344603: ......C.................A.........................   Ptroglodytes
  362611: ...G..C.................A.................C.......   Ggorilla
  394568: ......C.T...............A.........................   Pabelli
  315856: ......C.................A.........................   Nleucogenys
  391644: ......C.................A.........C...........A...   Mmulatta
  344155: ......C.................A.....................A...   Panubis
  375085: ......T.......C..T......A.........................   Cjacchus
 1024226: ......C.......C.........A.....G...C...............   Sboliviensis

                       24926     24936     24946     24956     24966
                           :         :         :         :         :
   24917: ACTGCCTGTGGGAAAACACAGTGCTTCCTGTCCATTAGAGGTGTGGGTAT   Hsapiens
  344653: .............................................A....   Ptroglodytes
  362661: .................................G................   Ggorilla
  394618: .......AC..........C..................T.........G.   Pabelli
  315906: ...................C..............................   Nleucogenys
  391694: ..G.........GG.G...C.....................C......G.   Mmulatta
  344205: ..GC........GG.G...C.....................C......G.   Panubis
  375135: ......CA....GG.....CA................C..........G.   Cjacchus
 1024276: ...-........GT.....CA..............C.C..........G.   Sboliviensis

                       24976     24986     24996     25006     25016
                           :         :         :         :         :
   24967: GAGTGCTGTCACAGGGAAGCAGCAATTTTCCTGAGTCACTATAATATCAT   Hsapiens
  344703: ..................................................   Ptroglodytes
  362711: ..................................................   Ggorilla
  394668: ...........TG---------------.................G....   Pabelli
  315956: ....................G.............................   Nleucogenys
  391744: ..........................................G.......   Mmulatta
  344255: ..........................................G.......   Panubis
  375185: ........C.....AA..........G...............G.......   Cjacchus
 1024325: ..........................G...............G.......   Sboliviensis

                       25026     25036     25046     25056     25066
                           :         :         :         :         :
   25017: AGGTACCAAACATGGATTTGGGGGCTCAGTGTTTGTCCAGTAAGAAGGAA   Hsapiens
  344753: ........................................G.......--   Ptroglodytes
  362761: ................................................-.   Ggorilla
  394703: .T..............................................--   Pabelli
  316006: ......................................G.........--   Nleucogenys
  391794: ................................................--   Mmulatta
  344305: ......A.......................A.................--   Panubis
  375235: ......TG...............A...............A........--   Cjacchus
 1024375: .......G...............A...............A........--   Sboliviensis

                                 25081               25099     25109
                                     :                   :         :
   25067: AAAAAAA---ATT--TATTAATCAACTC--AAAAACTTTAGGTGTCAGAA   Hsapiens
  344801: .......---...--.............--....................   Ptroglodytes
  362810: .......---...--.............--....................   Ggorilla
  394751: -......---...--.......A.....--....................   Pabelli
  316054: -......---...--.......AG....--...........A........   Nleucogenys
  391842: -......---..G--.......A.....--....................   Mmulatta
  344353: -......---..G--.......A.....--....................   Panubis
  375283: -......---..A--.......A.....AG....G........A......   Cjacchus
 1024423: -......CAT...CA.......A.....AG....G........A......   Sboliviensis
```

```
                  25119     25129     25138     25148     25157
   25110: GAAGTAAGAACAATAGAGATAAAA-AACAAATACTATCCATGAAC-AATG  Hsapiens
  344844: .......-................-............T.......-T...  Ptroglodytes
  362853: ........................-....................-....  Ggorilla
  394793: ...A....................----.....T...T.......A....  Pabelli
  316096: ...A....................--...........T....C..-....  Nleucogenys
  391884: ...A.G..................-............T.......A....  Mmulatta
  344395: ...A.G.............C....A............T.......A....  Panubis
  375327: .G.AC....G............G.-............T...A...A.C..  Cjacchus
 1024472: ...A..G..G..............-............T...A...A.C..  Sboliviensis

                  25166     25176     25186     25196     25206
   25158: AGG-AAAAAAATAAAGACTTGTCTTTTGTTTTTTGAAATCCAGGTCAAGG  Hsapiens
  344891: ..--..............................................  Ptroglodytes
  362901: ...-..................G...........................  Ggorilla
  394839: ...-......................................A.......  Pabelli
  316143: ...-.......................................A......  Nleucogenys
  391933: ...A.........................G....................  Mmulatta
  344445: ...A.........................G....................  Panubis
  375376: ...A....................C........................A  Cjacchus
 1024521: ...A...............C....C.................C......A  Sboliviensis

                  25216     25226     25236     25246
   25207: AGACCTTCTCTCCACTTTCTCTCCAACTTCCTTTTCTAAATTTAAAAAA-  Hsapiens
  344939: .................................................A  Ptroglodytes
  362950: .................................................A  Ggorilla
  394888: ...A................T..............TA............A  Pabelli
  316192: ...G...............................T......A......-  Nleucogenys
  391983: ...T.....T..........G...........G..T.............A  Mmulatta
  344495: ...T.A...T..........G...........G..T.............A  Panubis
  375426: ...A......-----------.....A...T....T.--.C........-  Cjacchus
 1024571: ..................T.......A...T....T.TCC..AG.....-  Sboliviensis

                               LINE1
            ---------------------------------------------->
                  25264     25274     25284     25294     25304
   25256: -AATTGTGGGTACACAGTACTCATACGTTTTTATAGGGTACATGTGATAT  Hsapiens
  344989: -..................G..............................  Ptroglodytes
  363000: -..................G..............................  Ggorilla
  394938: -..................GG....T..A..C......C...........  Pabelli
  316241: -..A...............G.....T..A................A....  Nleucogenys
  392033: G........A.........GG....T..A..........G..........  Mmulatta
  344545: G........A......A..GG....T..A..........G..........  Panubis
  375462: -..A.....AC........GG....T-.A....C..T.C...........  Cjacchus
 1024620: -..AC....AC....C...GG....T-.A....C..T.............  Sboliviensis

                              LINE1
          ------------------------------------------------>
                  25314     25324     25331     25341
   25305: TTTGGTACAGGCATGCAGTGTGT---AAAAATCACATCATT------TT-  Hsapiens
  345038: ...................A...---..............G-AAAAA..G  Ptroglodytes
  363049: .......................---..............G-AAAAA..A  Ggorilla
  394987: .......................---........---...G-AAAAA..G  Pabelli
  316290: .......................---..............G-AAAAA..G  Nleucogenys
  392083: ......G................---......T...A...G-AAAAA..G  Mmulatta
  344595: ......G................---..........A...G-AAAAA..G  Panubis
  375510: .............CA..A..CA.--A......T.A.....GAAAAAAC.G  Cjacchus
 1024668: .................A..CA.AAA..............G-AAAAA..G  Sboliviensis
```

```
                              LINE1
              25352     25362     25372     25382     25392
                  :         :         :         :         :
  25345: --TATCTGTCCCCTCAAGCATTTATCCCTTCAATGCATAATGGATAAATG  Hsapiens
 345084: GG..........................C.G...................  Ptroglodytes
 363095: GG..........................C......A..............  Ggorilla
 395030: GG..........................C.....................  Pabelli
 316336: GG..........................C.....................  Nleucogenys
 392129: GA.......T..T.....T........AC.....................  Mmulatta
 344641: GA.......T..T...............C.....................  Panubis
 375558: GG.....A............-....A.T.-------------------..  Cjacchus
1024717: GG.....A.........A..-......T.-------------------..  Sboliviensis

                              LINE1
                        25408     25418     25428     25438
                            :         :         :         :
  25393: CACTAC----AAAAAATCCAGTTATATTATTTTAGTTATTATAAAATATA  Hsapiens
 345134: ......-----.......................................  Ptroglodytes
 363145: ......---A...............................C........  Ggorilla
 395080: ..T...----................G.......................  Pabelli
 316386: ......---A......................................A.  Nleucogenys
 392179: .TT..A----....T.............C.....................  Mmulatta
 344691: .TT..T----....T.............C...................C.  Panubis
 375588: ..T...----G...............C.C..CC..............G..  Cjacchus
1024747: ..T...AAAA................C.C..CC..............G..  Sboliviensis

                              LINE1
              25448     25458     25468     25478     25488
                  :         :         :         :         :
  25439: CAATGTAATTATTATTGACTATAGTCCCCCTGTTGTGCTTTCAAATAGTA  Hsapiens
 345179: ...............................................C..  Ptroglodytes
 363192: ...A...........................................C..  Ggorilla
 395126: ..................A...............................  Pabelli
 316433: .....C............A......A.....................C..  Nleucogenys
 392225: ....T............................C.............C..  Mmulatta
 344737: ....T............................C.............C..  Panubis
 375634: ....T...........A....C...-.............A.......C..  Cjacchus
1024797: T...T...........A....C..C-T............A.......C..  Sboliviensis

                              LINE1
              25498     25508     25518     25528     25538
                  :         :         :         :         :
  25489: GGTCCTATCTGTTTCTTCTAACTATATATTTTTGTACCTATTAATCATTC  Hsapiens
 345229: ............................................C.....  Ptroglodytes
 363242: .......................................G....C.....  Ggorilla
 395176: .............................C..............C.....  Pabelli
 316483: ............................................C.....  Nleucogenys
 392275: ..........A.................................C.....  Mmulatta
 344787: ..........A.................................C.....  Panubis
 375683: ........TG...C.............................CC.....  Cjacchus
1024846: ........T....C.......................G......C.....  Sboliviensis
```

```
                               LINE1
          ---------------------------------------------------->
                 25548     25558     25568                25587
                     :         :         :                    :
   25539: CACCTCCTCCCCAACCCCCCACTACCCTTCCCAGCCTTT-GGTCACCATC  Hsapiens
  345279: ...................T...................-..........  Ptroglodytes
  363292: ...................A...................-.A........  Ggorilla
  395226: ................................T......-..........  Pabelli
  316533: .....................................C.G..........  Nleucogenys
  392325: .G.................-.................C.-..........  Mmulatta
  344837: .G...................................C.-..........  Panubis
  375733: ..............T...T......T.....T.....GG-A.........  Cjacchus
 1024896: .C................T......T.....T......G-..........  Sboliviensis
```

```
                               LINE1
          ---------------------------------------------------->
                 25597     25607     25617     25627     25637
                     :         :         :         :         :
   25588: CTTCTATTCTCTATCTCCATAAGTTCAATTGTTTTGACTTTTAGATTCCA  Hsapiens
  345328: ......................................A...........  Ptroglodytes
  363341: ..................G...............................  Ggorilla
  395275: ..................................................  Pabelli
  316583: ..................................................  Nleucogenys
  392373: ...........................C...........C..........  Mmulatta
  344886: ............C..............C....C......C..........  Panubis
  375782: .......--...G..........A............G.C...G....T..  Cjacchus
 1024945: ............G..........AA...........G.C........T..  Sboliviensis
```

```
                               LINE1
          ---------------------------------------------------->
                 25647     25657     25667     25677     25687
                     :         :         :         :         :
   25638: CAAATAATTGAGAACATGCAATGTTTGTCTTTCTGTGCCTGGCTTATTTC  Hsapiens
  345378: ...G..............................................  Ptroglodytes
  363391: ..................................................  Ggorilla
  395325: ..................................................  Pabelli
  316633: ..G...................A...........................  Nleucogenys
  392423: ....................G.............................  Mmulatta
  344936: ....................G.............................  Panubis
  375830: ..G...G........G..............................C...  Cjacchus
 1024995: ..G...........................................C...  Sboliviensis
```

```
                               LINE1
          ---------------------------------------------------->
                 25697     25707     25717     25727     25737
                     :         :         :         :         :
   25688: ACTGAACATAGTAACCTCCTGTTCTATTCATGTTGCTGCAAGTGACAGAA  Hsapiens
  345428: ..................................................  Ptroglodytes
  363441: ...T....C.........................................  Ggorilla
  395375: ...T....C...............C..........T..............  Pabelli
  316683: ...T....C..........G....C.....A....T.C............  Nleucogenys
  392473: ...T...................AC..........T..............  Mmulatta
  344986: ...T..A................AC..........T..............  Panubis
  375880: G..T...............A....C..........T..............  Cjacchus
 1025045: ...T..TG...........A....C..........T..............  Sboliviensis
```

```
                                   LINE1
           ---------------------------------------------------->
                   25747     25757     25767     25777     25787
   25738: TCTCATCCTTTTTTATAGCTGAATGGTACTCCATTGTGTGTAAGTATCAC  Hsapiens
  345478: ..................................................  Ptroglodytes
  363491: ....................................C.............  Ggorilla
  395425: ......T........................T.......A..........  Pabelli
  316733: ......T................................A..........  Nleucogenys
  392523: ......T...........G................A...A....C..T..  Mmulatta
  345036: ......T...........G................A...A....C..T..  Panubis
  375930: ......T.............................GA.A..........  Cjacchus
 1025095: ......T.............................G..A..........  Sboliviensis
```

```
                                   LINE1
           ---------------------------------------------------->
                   25797     25807     25817     25827     25837
   25788: ATTTTCTTTATCTATTTATCTGTTGATGAATGCTTAGGTAGCTTCCAAAT  Hsapiens
  345528: .........G..C.....................................  Ptroglodytes
  363541: ............C.....................................  Ggorilla
  395475: ............C.....................................  Pabelli
  316783: ............C.....................................  Nleucogenys
  392573: ........A...C............G........................  Mmulatta
  345086: ........A...C.......C.............................  Panubis
  375980: ............C...C....C...G..........A..G..........  Cjacchus
 1025145: ............CG..C....C...G..........A..G.A........  Sboliviensis
```

```
                                   LINE1
           ---------------------------------------------------->
                   25847     25857     25867     25877     25887
   25838: CTTGGCTACTGTGAACAGTACTGCAACAAACATGAGAGTGGAGATATCTC  Hsapiens
  345578: ..................................................  Ptroglodytes
  363591: ..................................................  Ggorilla
  395525: ........T.....G............G..................C...  Pabelli
  316833: ........T.....................................C...  Nleucogenys
  392623: ........T...............................C.........  Mmulatta
  345136: ........T...............................C.........  Panubis
  376030: .......G--..A.GTG....A..............G.G.C.........  Cjacchus
 1025195: ........T...A..T......................G.C.........  Sboliviensis
```

```
                                   LINE1
           ---------------------------------------------------->
                   25896     25906     25916     25926     25936
   25888: TT-AGATACATTGATCTCCTTTCTTTTGGGTAAATACCCAGTAGTGGGAT  Hsapiens
  345628: ..-...............................................  Ptroglodytes
  363641: ..-......................C........................  Ggorilla
  395575: ..A............................................A..  Pabelli
  316883: ..-.................................T..........A..  Nleucogenys
  392673: ..-.....T............................T.........A..  Mmulatta
  345186: ..-.....T......................................A..  Panubis
  376078: .G-.....TG...-----.......................C....AA..  Cjacchus
 1025245: .G-.....TG...-----............................AA..  Sboliviensis
```

```
                              LINE1
         ------------------------------------------------->
              25946     25956     25966     25976     25986
                  :         :         :         :         :
  25937: TGTTGAGTCATAAGGTAGCTCAATTTTTAGTTTTTTGAGGAACCTCCAAA   Hsapiens
 345677: .......................C..........................   Ptroglodytes
 363690: ............T................A....................   Ggorilla
 395625: ............T.....................................   Pabelli
 316932: ............T.....................................   Nleucogenys
 392722: ............C.............................A.......   Mmulatta
 345235: ............T.............................A.......   Panubis
 376122: ............CA.....................C..............   Cjacchus
1025289: ...CAT......T................T....................   Sboliviensis
```

```
                              LINE1
         ------------------------------------------------->
              25996     26006     26016     26026     26036
                  :         :         :         :         :
  25987: CTGTTCTCCATTGTTGTACTAACTTACATTCCCACCAACAGTGTATGAGG   Hsapiens
 345727: ..................................................   Ptroglodytes
 363740: ..................................................   Ggorilla
 395675: ..................................................   Pabelli
 316982: ..................................................   Nleucogenys
 392772: ...................C.................G..-.........   Mmulatta
 345285: G....................................G..-.........   Panubis
 376172: ..A...................T...-........T....A....C....   Cjacchus
1025339: T.A...........A.......T...-.............A.......A.   Sboliviensis
```

```
                              LINE1
         ------------------------------------------------->
              26046     26056     26066     26076     26086
                  :         :         :         :         :
  26037: ATTCCCTTTTCTCCATATCCTTGCCAGTATTTGTTATTGCCTGAATTTTT   Hsapiens
 345777: ..................................................   Ptroglodytes
 363790: ..................................................   Ggorilla
 395725: ...........................C......................   Pabelli
 317032: ..........................AC...........A..........   Nleucogenys
 392821: ...............C..................................   Mmulatta
 345334: ..................................C...............   Panubis
 376221: ..........................AC.........A...........G   Cjacchus
1025388: ...........................C.....................G   Sboliviensis
```

```
                              LINE1
         ------------------------------------------------->
              26096     26106     26116     26126     26136
                  :         :         :         :         :
  26087: AATAAAAGCAATTTTAACTGGAATGAGATTTGATTTGCATTTCCCTGATG   Hsapiens
 345827: ..................G...............................   Ptroglodytes
 363840: ..................................................   Ggorilla
 395775: .......................................A..........   Pabelli
 317082: ...............G..A..............................A   Nleucogenys
 392871: ..................A....................C...T......   Mmulatta
 345384: ..................A.........................T.....   Panubis
 376271: ..................C..G............................   Cjacchus
1025438: .....................G.................A..........   Sboliviensis
```

LINE1

```
                26146     26156     26166     26176     26186
   26137: ATCAGTGATGTTGAGCACCTTTTCACATGTCTGTTTGCCATTTGTATGTC Hsapiens
  345877: .................................................. Ptroglodytes
  363890: .........................T........................ Ggorilla
  395825: .........................T........................ Pabelli
  317132: ........................GT........................ Nleucogenys
  392921: .....G...................T........................ Mmulatta
  345434: .....G.........T.........T........................ Panubis
  376321: .C.G..................GT.T...C............C....... Cjacchus
 1025488: ...G..............A....T.T.CTC.................... Sboliviensis
```

LINE1

```
                26196     26206     26216     26226     26236
   26187: TTCTTTTGAGAAATATCTGTTCGATTCTTTTACCCATTTTTAATCAGAGT Hsapiens
  345927: .................................................. Ptroglodytes
  363940: ......................A........................... Ggorilla
  395875: ......................AG.................          Pabelli
  317182: ......................AT.................          Nleucogenys
  392971: ......................A.............A............. Mmulatta
  345484: ........G....C........A.............A............. Panubis
  376371: ...................-..A....................A...G.. Cjacchus
 1025538: ...................-..A.....G..............A...G.. Sboliviensis
```

LINE1

```
                26246     26256     26266     26276     26286
   26237: ATTAGATTTTTTTCCTATAGAGCTGTTGGAGCTTCTTATATATTCTGGTT Hsapiens
  345977: ...................................C.............. Ptroglodytes
  363990: .................................................. Ggorilla
  393021: ...G..-.........G..........................G...... Mmulatta
  345534: G.....-........................................... Panubis
  376420: ....T............................................C Cjacchus
 1025587: ....T............................................C Sboliviensis
```

LINE1

```
                26296     26306     26316     26326     26336
   26287: ATCAATCCCTTGTCAGGTGGGTAGTTTGCAAACATTTTCTCCCATTCTGT Hsapiens
  346027: .................................................. Ptroglodytes
  364040: .................................................C Ggorilla
  393070: ..T..............C................................ Mmulatta
  345583: ..T..............C................................ Panubis
  376470: ..T.....T...C..AA.......C....................C.... Cjacchus
 1025637: .CT.....T...C..A.................................. Sboliviensis
```

LINE1

```
                26346     26356     26366     26376     26384
   26337: AGCTTATCTCTTCACTTTGTTGATAGTTTCTCTTGCTGTGCAGTAG--TT Hsapiens
  346077: .................................G............--.. Ptroglodytes
  364090: ..G..........G................C...............--.. Ggorilla
  393120: ..G....T................T.....................--.. Mmulatta
  345633: ..G..................T..T.....C...............--.. Panubis
  376520: G.G.....................T...............T.....--.. Cjacchus
 1025687: G.G.....................T.....C.........T.....CT.. Sboliviensis
```

```
                                    LINE1
           ---------------------------------------------------------->
                 26394     26404     26414     26424     26434
                     :         :         :         :         :
   26385: TTTTTAAGTTGATATGATCCCATTTGTCCATTTTTGCTTTGGTTGCCTGT  Hsapiens
  346125: ..................................................  Ptroglodytes
  364138: ............C.G...................................  Ggorilla
  393168: G...G.............................A.T.............  Mmulatta
  345681: G.................................................  Panubis
  376568: ............G............A......................A.  Cjacchus
 1025737: ............G............A....C...................  Sboliviensis

                                    LINE1
           ---------------------------------------------------------->
                 26444     26454     26464     26474     26484
                     :         :         :         :         :
   26435: AGTTGTAGAGTATTAATCAAGAAATTTTTGCCCACACAAATGTCCTGGAA  Hsapiens
  346175: ..................................................  Ptroglodytes
  364188: ......G...........................................  Ggorilla
  393218: ......G........C..............................A...  Mmulatta
  345731: ......G........C.......................T..........  Panubis
  376618: G.....G.G......C.............T....G.....AA........  Cjacchus
 1025787: ......G.G......C................T.G.....AA........  Sboliviensis

                          LINE1                      LINE1
           ------------------------------------>------------------>
                 26494     26504     26514     26524     26534
                     :         :         :         :         :
   26485: CATTTCCCCAATGTTTTCTTGTAGTAGTTTCATAGGAGCATATTGTTTAA  Hsapiens
  346225: ...........C......................................  Ptroglodytes
  364238: .................................G................  Ggorilla
  393268: ......TG..............................TG..........  Mmulatta
  345781: ......................................TG..........  Panubis
  376668: ............A....-...G...............A..C.........  Cjacchus
 1025837: .....T..TT.A.....-...A...............A..AG........  Sboliviensis

                                    LINE1
           ---------------------------------------------------------->
                 26544     26554     26564     26574     26584
                     :         :         :         :         :
   26535: TTTCCATGTGTTTGAATAGTTTCCAAAATTTCTCTTATTATTGATTTCTA  Hsapiens
  346275: ..................................................  Ptroglodytes
  364288: .........A........................................  Ggorilla
  393318: C..T...........................T..................  Mmulatta
  345831: C............T....................................  Panubis
  376717: ...T.......C...G.TT...........C......G............  Cjacchus
 1025886: ...T...........G.TT...........C...................  Sboliviensis

                                    LINE1
           ---------------------------------------------------------->
                 26594     26604                         26610
                     :         :                             :
   26585: GTTTTATTTCATTGTGGTCAGA------------------------GGAG  Hsapiens
  346325: ..................T...------------------------....  Ptroglodytes
  364338: ......................------------------------....  Ggorilla
  393368: ........C.-----------.NNNNNNNNNNNNNNNNNNNNNNNN.A.A  Mmulatta
  345881: ........C.............------------------------....  Panubis
  376767: ........CT............------------------------....  Cjacchus
 1025936: ........C......A......------------------------.A..  Sboliviensis
```

```
                         LINE1
            26620     26630     26640     26650     26660
   26611: ATGCTTGATATTATTTCAATTTTTTTGAATGTTTTAAGACTTGTTTTGTG  Hsapiens
  346351: ..................................................  Ptroglodytes
  364364: ..................................................  Ggorilla
  393407: ...-----------------------------..................  Mmulatta
  345907: ...T...................................TG.........  Panubis
  376793: ...................A--...G.......C...A............  Cjacchus
 1025962: ...-...............C................T.............  Sboliviensis
```

```
                         LINE1
            26670     26680     26690     26700     26710
   26661: ACCTAACACATGGTCTGTCCTTGAGAATAATCCACGTGCTGAGAAGAAGA  Hsapiens
  346401: ..................................................  Ptroglodytes
  364414: ...................................A..............  Ggorilla
  393428: ........T.........................T...............  Mmulatta
  345957: ........T.........................T...............  Panubis
  376841: ..A.....T.........................T...------------  Cjacchus
 1026011: ..A....GT...............A...-.....T...------------  Sboliviensis
```

```
                         LINE1
            26720     26730     26740     26750     26760
   26711: AAAATGTCCTGAAGCCATTGGAAAAAGTGTTCTCTAAATATTTATTAGGT  Hsapiens
  346451: ..................................................  Ptroglodytes
  364464: ..................................................  Ggorilla
  393478: .G........................................A.......  Mmulatta
  346007: .G................................................  Panubis
  376880: -----A..........G.....T.G.........................  Cjacchus
 1026049: -----A................T......................G....  Sboliviensis
```

```
                         LINE1
            26770     26780     26790     26800     26810
   26761: CCATTTGGTCTGTAGTGCAGATTAAGTCAGATATTTCTTTGTTGATTTTC  Hsapiens
  346501: ...........................................T......  Ptroglodytes
  364514: ..................................................  Ggorilla
  393528: T...........................C.....................  Mmulatta
  346057: T...............A...........CA......A.............  Panubis
  376925: .........................A..T...G.................  Cjacchus
 1026094: ...............C............T...G.................  Sboliviensis
```

```
                         LINE1
            26820     26829     26839     26849     26859
   26811: TGTCTGATAGTCTGTCC-GTGCTGAAAGTGGGGTGTTGAAGTCTCCAGCT  Hsapiens
  346551: .................-................................  Ptroglodytes
  364564: .................A................................  Ggorilla
  393578: ........----.....A.C.T........A...................  Mmulatta
  346107: .........T.......A.CA.........A...................  Panubis
  376975: .A..........CA...A................................  Cjacchus
 1026144: ............CA...AT.....................A.........  Sboliviensis
```

```
                                LINE1
              26869     26879     26889     26899     26909
   26860: ATTATTGTATTGGAGTCTATGTTTCTCTTCAGCTTTATATATCTGGGTGC  Hsapiens
  346600: ..................................................  Ptroglodytes
  364614: ......................................G...........  Ggorilla
  393624: ....C...............C.............................  Mmulatta
  346157: ....................C.....................T.......  Panubis
  377025: ..............A..C..C.....A......................T  Cjacchus
 1026194: ....................C.....A...............T.....AT  Sboliviensis
```

```
                                LINE1
              26919
   26910: CCCAGTGTTGGATGC-----------------------------------  Hsapiens
  346650: T..............-----------------------------------  Ptroglodytes
  364664: T..............-----------------------------------  Ggorilla
  393674: T..............-----------------------------------  Mmulatta
  346207: T...........G..-----------------------------------  Panubis
  377075: T........A...ATATATAATATATATGCATTCACTATATGTAAAATAT  Cjacchus
 1026244: T............ATATATAACATATATGCATTCA-TATATATGTAAAAT  Sboliviensis
```

```
                                LINE1
                        26931                         26940
   26925: -------------ATGTATATATTTA---------------------TAG  Hsapiens
  346665: -------------.............---------------------...  Ptroglodytes
  364679: -------------.............---------------------...  Ggorilla
  393689: -------------..A......--..---------------------...  Mmulatta
  346222: -------------..A.......A.GTGTGTGTGTATATATATATAT...  Panubis
  377125: ATATGTAAAA---..A..........---------------------C..  Cjacchus
 1026293: ATATATGTAAAAA..A.G........---------------------C.A  Sboliviensis
```

```
                                LINE1
              26950     26960     26970     26980     26990
   26941: TTGTTATATCTTCTTGCTGAATTGATCCCTTTACCATTATATAATAAGCT  Hsapiens
  346681: ..................................................  Ptroglodytes
  364695: ......C..............................G............  Ggorilla
  393703: ..T......................C............-...........  Mmulatta
  346259: ..T......................C............-...........  Panubis
  377151: .......................T.C.....................C..  Cjacchus
 1026322: .........................C........................  Sboliviensis
```

```
                                LINE1
              27000     27010     27020     27030     27040
   26991: TCTTTGTCTCTTCTTAAAGTTTTTGTCTTGAAGTCTATTTTGTCTGATAT  Hsapiens
  346731: ..................................................  Ptroglodytes
  364745: ..................................................  Ggorilla
  393752: ................C.A................C............G.  Mmulatta
  346308: ................C...................G.............  Panubis
  377201: .....A.....G....C.A....C.C......A.........C......C  Cjacchus
 1026372: .....A..........C........C......A.........C......C  Sboliviensis
```

```
                                    LINE1
         ------------------------------------------------------------>
                  27050     27060     27070     27080     27090
  27041: AAGTAGAGCTACTTCTGCTCTTTCTTGGTTTTCATTGGCATGGAATATCT  Hsapiens
 346781: ..................................................  Ptroglodytes
 364795: .............C............A.........C.T........C..  Ggorilla
 393802: .............G..A..G.....................A.....C..  Mmulatta
 346358: .............GT.A..............................C..  Panubis
 377251: .....C.......C....................C.........C.....  Cjacchus
1026422: .....C.......C.....G..............C...............  Sboliviensis

                                    LINE1
         ------------------------------------------------------------>
                  27100     27110     27120     27129     27139
  27091: TTTTCCATTCCTTTATTTTCGGTCTATATGTA-TCTTTATAGGTGAAGTG  Hsapiens
 346831: ................................-.................  Ptroglodytes
 364845: ................................-.................  Ggorilla
 393852: ........C..........T...A........T.T....C..A.......  Mmulatta
 346408: ........C..............A........-..............A..  Panubis
 377301: ..AC....CT..........A........A..-.G......TT.......  Cjacchus
1026472: ..A.....C..........--.......AA..-........TT.......  Sboliviensis

                                    LINE1
         ------------------------------------------------------------>
                  27149     27159     27169     27178     27188
  27140: TGTTTCTTGTAGGTGACAGATCATTGGGTCTTG-TTTTTCATCCATTCAT  Hsapiens
 346880: .................................-................  Ptroglodytes
 364894: .................................-................  Ggorilla
 393902: ........A....CA......TGG...T.....C.....TG...G....G  Mmulatta
 346457: .........................A.......-................  Panubis
 377350: ........................CA.......-....G........T..  Cjacchus
1026519: .................................-....G........T..  Sboliviensis

                                    LINE1
         ------------------------------------------------------------>
                            27202     27212     27222     27232
  27189: CCAC------TCTTTTGATTGAAGAGTTTGGTTCTTTTACATTCAATGTT  Hsapiens
 346929: ....------........................................  Ptroglodytes
 364943: ....------..................................G.....  Ggorilla
 393952: T...TCTGTG........C..G...-----C.C.A...............  Mmulatta
 346506: ....TCTATG.............A..C..................G....  Panubis
 377399: ....TCTCTAC.....A................TA..........G.C..  Cjacchus
1026568: ....TCTATA............C..........TA..........G....  Sboliviensis

                                    LINE1
         ------------------------------------------------------------>
                  27242     27252               27265     27275
  27233: ATTATTGATAAGTAGAGACTTAACTCC-------CACCATTTTGTTACTT  Hsapiens
 346973: ...........................-------................  Ptroglodytes
 364987: ...........................-------................  Ggorilla
 393997: .C.G.......C..AGA.TG.-.....TGACTAGTGT.....CAC..T..  Mmulatta
 346556: ...........................-------........CA......  Panubis
 377449: ...............G.....-.T..T-------................  Cjacchus
1026618: ...............G..T..-.T..T-------................  Sboliviensis
```

```
                                      LINE1
         --------------------------------------------------->
                27285     27295     27305     27315     27325
                    :         :         :         :         :
  27276: ATTTTTGGGTTGTTTTGTGCTCTTCTCTTCCTTCTTTCTTTCCTTCCTGT  Hsapiens
 347016: ..................................................  Ptroglodytes
 365030: ......................................C...........  Ggorilla
 394046: G.G.ACC...............................C...........  Mmulatta
 346599: C....CT...............................C...........  Panubis
 377491: G....CTCA..........G..................C..TT......C  Cjacchus
1026660: G....CTCA..........G..................C..TG.......  Sboliviensis

                                      LINE1
         --------------------------------------------------->
                27335     27345     27355     27365     27375
                    :         :         :         :         :
  27326: CTTCTTTTAGTGAAGATGATTTTCTCTTGTGGTATGCTTTAATTTCCTGA  Hsapiens
 347066: .........A................C.......................  Ptroglodytes
 365080: ..................................................  Ggorilla
 394096: ...................................A...........C..  Mmulatta
 346649: ..................................................  Panubis
 377541: ............................T.CT.G............T...  Cjacchus
1026710: ............................T...G.A..........T..G  Sboliviensis

                                      LINE1
         --------------------------------------------------->
                27385     27395
                    :         :
  27376: TTTTTATTTTTTGTGTATTCATTGTA------------------------  Hsapiens
 347116: ..........................------------------------  Ptroglodytes
 365130: ..........................------------------------  Ggorilla
 394146: ..................C.G.....------------------------  Mmulatta
 346699: ..................CTG....TT-----------------------  Panubis
 377591: ............-......TG.....TGGTTTT-----------------  Cjacchus
1026760: ...................TG.....TGGTTTTCTTTCCTTTTCTTTTTT  Sboliviensis

                                      LINE1
         --------------------------------------------------->
                          27406     27416     27426     27436
                              :         :         :         :
  27402: ---------------TTTTTTTTGAGGTTACCATGAGGCTTGCAAATACT  Hsapiens
 347142: ----------------..................................  Ptroglodytes
 365156: ---------------...................................  Ggorilla
 394172: ---------------........A..........................  Mmulatta
 346726: ---------------........A..........................  Panubis
 377623: ---------------....A...A..........................  Cjacchus
1026810: TTTTTTTTTTTTTTT..........CA.......................  Sboliviensis

                  LINE1                     LINE1
         ---------------------->---------------------------->
                          27447               27457     27467
                              :                   :         :
  27437: ATCT---------TTTTTTTAAATT----------ATACTTTAAGTTTTA  Hsapiens
 347176: ....---------.......T....----------...............  Ptroglodytes
 365191: ....---------.......T....TATTATTATT...............  Ggorilla
 394207: ....TATAACTCA..A........                            Mmulatta
 346761: ....TATAACTCA..A.........                           Panubis
 377658: ....TATAACTCA..A.........                           Cjacchus
1026860: ....TATAACTCA..A.........                           Sboliviensis
```

```
                                    LINE1
        ------------------------------------------------------>
                27477     27487     27497     27507     27517
                    :         :         :         :         :
 27468: GGGTACATGTGCACAATGTGCAGGTTAGTTACATATGTATACATGTGCCA  Hsapiens
347207: .....................................C............  Ptroglodytes
365232: ..................................................  Ggorilla

                                    LINE1
        ------------------------------------------------------>
                27527     27537     27547     27557     27567
                    :         :         :         :         :
 27518: TGCTGGTGCGCTGCACCCACTAACTCGTCATCTAGCATTAGGTATATCTC  Hsapiens
347257: ..................................................  Ptroglodytes
365282: .........A........................................  Ggorilla

                                    LINE1
        ------------------------------------------------------>
                27576     27586     27596     27606     27616
                    :         :         :         :         :
 27568: CCAA-TGCTATCCCTCCCTCCTCCCCCCACCCCACAACAGTCCCCAGAGT  Hsapiens
347307: ....-.............C...............................  Ptroglodytes
365332: ....T.............C............G..................  Ggorilla

                                    LINE1
        ------------------------------------------------------>
                27626     27636     27646     27656     27666
                    :         :         :         :         :
 27617: GTGATGTTCCCCTTCCTGTGTCCATGTGTTCTCATTGTTCAATTCCCACC  Hsapiens
347356: ..................................................  Ptroglodytes
365382: ....................................                Ggorilla

                                    LINE1
        ------------------------------------------------------>
                27676     27686     27696     27706     27716
                    :         :         :         :         :
 27667: TATGAGTGAGAACATGCAGTGTTTGGTTTTTCGTTCTTGCGATAGTTTAC  Hsapiens
347406: ...............................T..................  Ptroglodytes

                                    LINE1
        ------------------------------------------------------>
                27726     27736     27746     27756     27766
                    :         :         :         :         :
 27717: TGAGAATGATGATTTCCAATTTCATCCATGTCCCTACAAACGACATGAAC  Hsapiens
347456: ..................................................  Ptroglodytes

                                    LINE1
        ------------------------------------------------------>
                27776     27786     27796     27806     27816
                    :         :         :         :         :
 27767: TCATCATTTTTTATGGCTGCATAGTATTCCATGGTGTATATGTGCCACAT  Hsapiens
347506: ..................................................  Ptroglodytes
377573:                                         .....TTT.A  Cjacchus

                                    LINE1
        ------------------------------------------------------>
                27826     27836     27846     27854     27864
                    :         :         :         :         :
 27817: TTTCTTAATCCAGTCTATCATTGTTGGACATTTG--GGTTGGTTCCAAGT  Hsapiens
347556: ..................................--..............  Ptroglodytes
377583: ......G..------.T.T...T..TTGT.....TT.TA.....TTTTT.  Cjacchus
```

```
                  LINE1                     LINE1
         ------------------------->------------------------->
              27871     27881     27891     27901     27911
  27865: CTTT---GCTATTGTGAATAACGCAAATACTATCTTATAACTCATTATTT  Hsapiens
 347604: ....---...........................................  Ptroglodytes
 377627: A...AAG.T..CCA...GGCTT............................  Cjacchus
1026851:                       ............................  Sboliviensis

                                  LINE1
         -------------------------------------------------->
              27921     27931     27940     27950     27960
  27912: TAAATTGATGACAAGTCAACACTGG-TTGCATAAACAAACACCCACAAAA  Hsapiens
 347651: .........................-........................  Ptroglodytes
 377677: ................T...T....-.............TG..T.A....  Cjacchus
1026879: ................T........C....C.....T..TG....A...G  Sboliviensis

                                  LINE1
         -------------------------------------------------->
              27970     27980     27990     28000     28010
  27961: AAAACTAATAAAACTCTACACTTTCACTTTGTCCTCCTGCTTTTTAACTT  Hsapiens
 347700: ..................................................  Ptroglodytes
 377726: .....................C...............C.......-----  Cjacchus
1026929: .....................C..............T........-----  Sboliviensis

                                  LINE1
         -------------------------------------------------->
              28020     28030     28040     28050     28060
  28011: TTTGTTGTTTCTATTTATGTCTTATTGTACCACCTATGTCTTGAAAAGTT  Hsapiens
 347750: ..................................................  Ptroglodytes
 377771: ---.....A.................A....GT..G..............  Cjacchus
1026974: ---.....A.......................G..G..............  Sboliviensis

                                  LINE1
         -------------------------------------------------->
              28070     28080     28090     28100     28110
  28061: ATTATTTTTGATTGTTTGATCATTTGATCTTTCTACTTAAGAGAAAAGTG  Hsapiens
 347800: ..................................................  Ptroglodytes
 377818: .........A....G..C........C..............GC..G....  Cjacchus
1027021: .........T....G..A.......................GC..G....  Sboliviensis

                              LINE1                  Alu
         ------------------------------------------->------>
              28120     28130     28140     28150     28160
  28111: GTATACACACGACAATTACAGTGCTTTACTATTTTGGTTTTTTTTTTCTT  Hsapiens
 347850: ..................................................  Ptroglodytes
 377868: ..........T................C.....C..-.....C.......  Cjacchus
1027071: ..........C................C...C.C..-.....C.....C.  Sboliviensis

                                   Alu
         -------------------------------------------------->
              28170     28180                   28189     28199
  28161: TTTTTTCCTTTCTTTTCTTTTTTT-----------AGACAGAATCTTGCT  Hsapiens
 347900: C......................G-----------...............  Ptroglodytes
 377917: ...C.......T....T.......TTTTTTTTTTG....G..GA......  Cjacchus
1027120: ...C..T.........T.......----TTTTTTG.......G.......  Sboliviensis
```

```
                                      Alu
              28209     28219     28229     28239     28249
  28200: CTGTTGCCCAGGCTGGAGTGCAGTGGCACAATCTCAGCTCACTGCAACCT  Hsapiens
 347939: ..................................................  Ptroglodytes
 377967: ....CA....A.T..A.............G......C...C.........  Cjacchus
1027166: ....CA......T..A.............G......C...C.......A.  Sboliviensis
```

```
                                      Alu
              28259     28269     28279     28289     28299
  28250: CTGTCTCCCAGATTCAAGTGATTTTCTTGTCTCAGCCTCCCAAGTAGCTG  Hsapiens
 347989: ...C.....................T......................C.  Ptroglodytes
 378017: .CAC...A...G......C....C..C..C..........TG.A...G..  Cjacchus
1027216: .CAC...A...G......C....C..C..C..........TG.A......  Sboliviensis
```

```
                                      Alu
              28309     28319     28329                28345
  28300: GGACTACAGGCATGTGCCACCACCCCCAACTGATTTTT----TATATTTT  Hsapiens
 348039: .........................T............----........  Ptroglodytes
 366105:                        ...............----......C.  Ggorilla
 378067: A.........T.....T......AT...G..A......----.G......  Cjacchus
1027266: A.........TG....T......ATT..G..AT.....TGTG.G.G....  Sboliviensis
```

```
                                      Alu
              28355     28365     28375     28385     28395
  28346: TAGTGGAGACAGGGTTTCACCGTGTTAGCCAGGATGGTCTCAATCTCCTG  Hsapiens
 348085: ............C.....................................  Ptroglodytes
 366128: ..................................................  Ggorilla
 378113: ....A....TG..T.....T.A.....A.......C....GG....T...  Cjacchus
1027316: ....A....TG..T.....T.A.....A.......CA...G.........  Sboliviensis
```

```
                                      Alu
              28405     28415     28425     28435     28445
  28396: ACCTCCTGATCCTCCTACCTCAGCCTGCCAGAGTGGTGGGATTACAGGTG  Hsapiens
 348135: ..........................C.....C.................  Ptroglodytes
 366178: ..........................C...A.................C.  Ggorilla
 394749:                   ........C...A....C..............  Mmulatta
 378163: .....A......A...G.........C..TA....C...........TCA  Cjacchus
1027366: .....A.A....A...G.........CAA.A....C...........TCA  Sboliviensis
```

```
              Alu                     LINE1
              28455     28465     28475     28485     28495
  28446: TGAGCCACTATGCAGGGCACTATTCTGTTTTTCTTTGTGGTTACTATCAC  Hsapiens
 348185: ..................................................  Ptroglodytes
 366228: ..................................................  Ggorilla
 394781: ..................................................  Mmulatta
 378213: ........C.CA.CT......T...-...........CTC.......T..  Cjacchus
1027416: ........C.CA.CT...........A...........TC.......TG.  Sboliviensis
```

```
                                  LINE1
              28505     28515     28525     28535     28545
   28496: CAGTGAGTTTTGTGCCTTCAGATGATTTCTTCTTGCTCATTAACATCCAT Hsapiens
  348235: .................................................. Ptroglodytes
  366278: ....T............................................. Ggorilla
  394831: .................................................. Mmulatta
  378262: ...............................................TG. Cjacchus
 1027466: .G....A........................................TG. Sboliviensis

                                  LINE1
              28555     28565     28575     28585     28595
   28546: TTCTTTCAGATTGAAGAACTGCCTTTAGTATTTCTTGTAGAACAGGTTGG Hsapiens
  348285: ...................G.............................. Ptroglodytes
  366328: ........A...........................A............. Ggorilla
  394881: ........C...........C............................. Mmulatta
  378312: ..........A....T....C....C.............AG....C...A Cjacchus
 1027516: ..........A.........C...................G........A Sboliviensis

                                  LINE1
              28605     28615     28625     28635     28645
   28596: GTCTTGATGAAATCCCTCAGCTTTTGTTTGTCTAGGTAGGTCTTTATTTC Hsapiens
  348335: .................................................. Ptroglodytes
  366378: .................................................. Ggorilla
  394931: A................................G................ Mmulatta
  378362: ........A.............G..........G....A........... Cjacchus
 1027566: ........A........................G....A..........T Sboliviensis

                                  LINE1
              28655     28665     28675     28685     28695
   28646: TCCTTCATGCTTGAAGGACATTTTCATTGGATATATTATTCTAGAATAAA Hsapiens
  348385: .................................................. Ptroglodytes
  366428: .................................................. Ggorilla
  394981: ..........C....................................... Mmulatta
  378412: ..................T..........AG............AGG.... Cjacchus
 1027616: ..............................G............AGG.... Sboliviensis

                                  LINE1
              28705     28715     28725     28735     28745
   28696: AGATTTTTTTCCTTCAGCACTTTACATATGTCATGCCACTCTCTCCTGGC Hsapiens
  348435: .................................................. Ptroglodytes
  366478: .....-..................T......................... Ggorilla
  395031: ...........T............A...C..................A.. Mmulatta
  378462: ..G...........T.........A......................... Cjacchus
 1027666: .....G........T.........A......T.................. Sboliviensis
```

```
                                 LINE1
         ------------------------------------------------->
                   28755     28765     28775     28782     28792
                       :         :         :         :         :
   28746: CGGTAATGTTTCCATTGAAAAGTCTGCTGCCA---GACATTTTGGAGCTC  Hsapiens
  348485: ................................---...............  Ptroglodytes
  366527: ................................---...............  Ggorilla
  395081: .T...................C..........---..T............  Mmulatta
  378512: .TA..GA....TT...................TAA..............T  Cjacchus
 1027716: .T...GCA...TT...................TAA....-......A.GT  Sboliviensis

                                 LINE1
         ------------------------------------------------->
                   28802     28812     28822     28832     28842
                       :         :         :         :         :
   28793: CATTGTATGTTATTTGTTTCTTTACTCTTGCTGCTTTTAAGATTCTTTCT  Hsapiens
  348532: ..................................................  Ptroglodytes
  366574: ..................................................  Ggorilla
  320111:                                       ............  Nleucogenys
  395128: ...........................................G......  Mmulatta
  378562: ...............A.......T.......................C..  Cjacchus
 1027765: ...............A.......T.AA....................C..  Sboliviensis

                                 LINE1
         ------------------------------------------------->
                   28852     28862     28872     28882     28892
                       :         :         :         :         :
   28843: TTATCCTTGACATTTGGGAGTTTGATTATTAAATGCTTTGAGATAGTTTC  Hsapiens
  348582: ..................................................  Ptroglodytes
  366624: .....................C............................  Ggorilla
  320123: .......C...........................T..............  Nleucogenys
  395178: .................A.....................C..........  Mmulatta
  378612: .............A...AT......A.T........C.........CC..  Cjacchus
 1027815: .........G...A...AT........T........C.....G....C..  Sboliviensis

                                 LINE1
         ------------------------------------------------->
                   28902     28912     28921     28931     28941
                       :         :         :         :         :
   28893: CTTTGTGTTAAATCTGCTTGGTGT-CTATAACTTTGTACTTGAATGTTGC  Hsapiens
  348632: .....G..................-..T......................  Ptroglodytes
  366674: ........................T........................A  Ggorilla
  320173: .....G..................T........................A  Nleucogenys
  395228: .....G.........A.......CT.....G.........G........A  Mmulatta
  378662: .....G..............A...TA..C...C................A  Cjacchus
 1027865: T....G.............A....TA..C...C.....GC.......C.A  Sboliviensis

                                 LINE1
         ------------------------------------------------->
                   28951     28961     28971     28981     28991
                       :         :         :         :         :
   28942: TATCTTTCTGTAGATTTGAGAAGTTTTCTGATATTATCCCTTTGAATAAA  Hsapiens
  348681: ..................................................  Ptroglodytes
  366724: ...............G..................................  Ggorilla
  320223: ........G.........G...............................  Nleucogenys
  395278: .........A...G....G........T......................  Mmulatta
  378712: C............G...........C.....C.C....A...........  Cjacchus
 1027915: C............G...........C............AT..........  Sboliviensis
```

```
                               LINE1
         ------------------------------------------------->
                28999     29007     29017     29027     29037
                    :         :         :         :         :
   28992: CCCCTAT--CTCTT--TCTCTACCTTTTCTTTAAGGTTAATAACTCTTAG   Hsapiens
  348731: .......--.....--..................................   Ptroglodytes
  366774: .....G.--.....--..................T...............   Ggorilla
  320273: .......--.....--..................................   Nleucogenys
  395328: .......--.....--..................................   Mmulatta
  378762: ..G....TG....C--C.................A..C............   Cjacchus
 1027965: .......--.....TC.........C........A..C............   Sboliviensis

                               LINE1
         ------------------------------------------------->
                29047     29057     29067     29076     29086
                    :         :         :         :         :
   29038: ATTTGCCCTTTTGAGGCTATTTTCTAGATTG-GTATGCATGCTTCATTCT   Hsapiens
  348777: ...............................-..................   Ptroglodytes
  366820: ...............................-..................   Ggorilla
  320319: .......A.......................-............T.....   Nleucogenys
  395374: .A.............................-..................   Mmulatta
  378810: ..................CC..........TT..TG.A...T......GC   Cjacchus
 1028013: ........AC....................TT..TG.....T......GC   Sboliviensis

                               LINE1
         ------------------------------------------------->
                29096               29110     29120     29130
                    :                   :         :         :
   29087: TTTTTACTCTTTTTTT------TTTGTGTCCTCTGACTGTGTATTTTCAA   Hsapiens
  348826: ...............-------...A........................   Ptroglodytes
  366869: ................CT----............................   Ggorilla
  320368: ....C..........ACT----.....C.......G..............   Nleucogenys
  395423: ......T.........CT----.....C.........G.....-......   Mmulatta
  378860: ......T.........TTTTTC..G..C..............G.......   Cjacchus
 1028063: ......T.........GGGGGGAGG..C......................   Sboliviensis

                               LINE1
         ------------------------------------------------->
                29140     29150     29160     29170     29180
                    :         :         :         :         :
   29131: GCAGCCTGTCTTCAAGCTCACCAATTCTTTCTTCTATTAAATTGATCAAT   Hsapiens
  348869: .A................................................   Ptroglodytes
  366915: ..................................................   Ggorilla
  320414: ....T.............................................   Nleucogenys
  395468: ..................................................   Mmulatta
  378910: ....-................T.............G..----........   Cjacchus
 1028113: ....-................T.............G..----........   Sboliviensis

                               LINE1
         ------------------------------------------------->
                29190     29200     29210     29220     29230
                    :         :         :         :         :
   29181: TCTATTAAAAGGCTCTGATGCATTCTTCATGATGGCAATTGCATTTCTCG   Hsapiens
  348919: ....C.............................................   Ptroglodytes
  366965: ..........................................G.......   Ggorilla
  320464: ..............................................T..A   Nleucogenys
  395518: ..............................................T..A   Mmulatta
  378955: .-----.....A...--.......T.........TT....T.T...T..A   Cjacchus
 1028158: .-----.....A............T.........TT..........T..A   Sboliviensis
```

```
                               LINE1
         ------------------------------------------------->
              29240     29250     29260     29270     29280
  29231: ACTCAAGAATTTCTGCTTGATTCTTTTTCATAATTTCAATCTCTTTGTTA  Hsapiens
 348969: ..................................................  Ptroglodytes
 367015: ...............................T....T.............  Ggorilla
 320514: ....G..........................T..................  Nleucogenys
 395568: ...T...........................T..................  Mmulatta
 378998: ....T.............A----........T..................  Cjacchus
1028203: ....T..........T..A----........T..................  Sboliviensis

                               LINE1
         ------------------------------------------------->
              29290     29300     29310     29320     29330
  29281: AATTTATTGGATAGAATTCTTAATTCCTTTTCTAGACTATCTTGGATTTC  Hsapiens
 349019: ..................................................  Ptroglodytes
 367065: ...................................G..............  Ggorilla
 320564: .........................T....C....G........A.....  Nleucogenys
 395618: ...................................G........A.....  Mmulatta
 379044: ........T..G........A....A.........G........A.....  Cjacchus
1028249: ........T..G.T......G....A.........GT.......A.....  Sboliviensis

                               LINE1
         ------------------------------------------------->
              29340     29350     29360     29370     29380
  29331: TTTGATTTTCCTCAAAACAACTATTATGAATTATCTCTCTGAAAGGTCAC  Hsapiens
 349069: .....G...................T........................  Ptroglodytes
 367115: .....G...................T........T...............  Ggorilla
 320614: .....G...................T...................T....  Nleucogenys
 395668: .....G...................T................G..T....  Mmulatta
 379094: .....G.A..T..C...........T.........A........-T.TG.  Cjacchus
1028299: .....GC...T..............T.......C..........AT.TG.  Sboliviensis

                               LINE1
         ------------------------------------------------->
              29390     29400     29410     29420     29430
  29381: ATATGTCTCTTTCTCTAGAATTGGTCCCTGGTTCATTATTTAGTTCATTT  Hsapiens
 349119: ..................................................  Ptroglodytes
 367165: ..................................................  Ggorilla
 320664: ..........................T......T.......CA.......  Nleucogenys
 395718: ..................................................  Mmulatta
 379143: ...............C.T............AA..................  Cjacchus
1028349: ...............C.T............AA..................  Sboliviensis

                               LINE1
         ------------------------------------------------->
              29440     29450     29460     29470     29480
  29431: GGTGAGGTTATGCTTTCCTGGATGGTCTTGATGCTTATAGATATTTGTCA  Hsapiens
 349169: ..................................................  Ptroglodytes
 367215: ..................................................  Ggorilla
 320714: ...........................................G......  Nleucogenys
 395768: ...........A....................AT................  Mmulatta
 379193: A-------...A............C.........................  Cjacchus
1028399: A.......................C.........................  Sboliviensis
```

```
                                     LINE1
       ------------------------------------------------------------------>
                     29490     29500     29510     29520     29530
   29481: GTGTCTAAGCAATGAAAAGCTAGGTATTTACTGTAGTCTTTATAGTCTGA  Hsapiens
  349219: ..........................................G......G  Ptroglodytes
  367265: ...................................A..............  Ggorilla
  320764: ..........................................G.......  Nleucogenys
  395818: ...................................T......C.......  Mmulatta
  379236: .................T...G............C......G........  Cjacchus
 1028449: ..............................T...........C.......  Sboliviensis

                                     LINE1
       ------------------------------------------------------------------>
                     29540     29550     29557     29567     29577
   29531: TCTTATTTCTGGTTGTCCTT---GAGGAGGCTTTCAGGGTATTTGAAGGG  Hsapiens
  349269: ....................---...........................  Ptroglodytes
  367315: ....................---...........................  Ggorilla
  320814: G.......G...C.......---.....A.....................  Nleucogenys
  395868: G.......G...C.A.....---...A..........A............  Mmulatta
  379286: G......AG.C.C.A.....CTT.G.A.........A......CA.....  Cjacchus
 1028499: G......AG...C.A.....TTT.G.A.........A......CA.....  Sboliviensis

                                     LINE1
       ------------------------------------------------------------------>
                     29587     29597     29607     29617     29627
   29578: ATCGGACCTGAAGCCCCATAATGCTGTCAGTTTTGCAGACTCATAGAGGT  Hsapiens
  349316: ..................................................  Ptroglodytes
  367362: ..T...............................................  Ggorilla
  320861: ..T...................A..............A............  Nleucogenys
  395915: ..T...............................................  Mmulatta
  379336: ..T................G.CA...........................  Cjacchus
 1028549: ..T...........T....G...................T..........  Sboliviensis

                                     LINE1
       ------------------------------------------------------------------>
                     29637     29647     29657     29667     29677
   29628: ACCACTCTGGCAGTCTTGGATAAGACCTGGAAGAATTCTCTGAATTACCA  Hsapiens
  349366: ..................................................  Ptroglodytes
  367412: ....................C.............................  Ggorilla
  320911: .A...........................A....................  Nleucogenys
  395965: .......C..........................................  Mmulatta
  379386: G.........TG............GT....C...................  Cjacchus
 1028599: G.........TG.............T....C...................  Sboliviensis

                                     LINE1
       ------------------------------------------------------------------>
                     29687     29697     29707     29716     29726
   29678: GACAGAGGCTCTTGTTCTTTTCCCTTACGTTCTCCCAA-ACAAACAGAGT  Hsapiens
  349416: .............T........................-...........  Ptroglodytes
  367462: ......................................-...........  Ggorilla
  320961: ..........T...........................-.......A...  Nleucogenys
  396015: ............................A.AG......-...........  Mmulatta
  379436: .......A.A.................AA.....TT.CT....G......  Cjacchus
 1028649: .......A.......C.....G......A.....TT---....G......  Sboliviensis
```

```
          LINE1                   Simple
              29736     29746     29756     29766     29776
   29727: CTCTCTCTGTGTGTGTCTCTCTGTCTGTCTCTCTCTATCTACCTATCTAT  Hsapiens
  349465: ......G...........................................  Ptroglodytes
  367511: ..G.........................................G.....  Ggorilla
  321010: ......G...................C.........G..C.T..C...G.  Nleucogenys
  396064: ....G.....................C..C....G.C...CT..C...G.  Mmulatta
  379486: ........C.C.C.CC....G.C...C.........G...CT..C...C.  Cjacchus
 1028696: ...---------------------------------------..C...C.  Sboliviensis
```

```
                          Simple
                                                  29782
   29777: CTGTC--------------------------------------------T  Hsapiens
  349515: .....--------------------------------------------.  Ptroglodytes
  367561: .....--------------------------------------------.  Ggorilla
  321060: A.C.A------------------------------------TCTGTCTC.  Nleucogenys
  396114: ..C..--------------------------------TCTCTGTGTCTC.  Mmulatta
  379536: ..C..TGTGTGTGTGTGTGTGTGTGTGTGTGTGTGTGTGTGTCTG.  Cjacchus
 1028708: ..C..--------------------------------------------.  Sboliviensis
```

```
                  Simple                   LINE1
              29792     29802     29812     29822     29832
   29783: CTCTCTCTGTCTCTTTCTCTCGCTCTCTTTCTCTCTCTCTCTGTACTGAG  Hsapiens
  349521: ..........T.......................................  Ptroglodytes
  367567: ...........................--.T...................  Ggorilla
  321074: ..............C.T....T......C.T.T.................  Nleucogenys
  396132: ..T.....C.....C.T...GT......C.T.........T.........  Mmulatta
  379586: ..............C......T..G...C.....................  Cjacchus
 1028714: ........C.....C......TG.....C................A....  Sboliviensis
```

```
                          LINE1
              29842     29852     29862     29872     29882
   29833: CCATCTTAAACTGAGGGTGTGGTGATGCAAGCATCCCTGTGGCCACCACC  Hsapiens
  349571: ..................................................  Ptroglodytes
  367615: ..................................................  Ggorilla
  321124: ..................................................  Nleucogenys
  396182: ....................A.....................T.......  Mmulatta
  379636: ...C.............C........A...A...................  Cjacchus
 1028764: ..............................A...................  Sboliviensis
```

```
                          LINE1
              29892     29902     29912     29922     29932
   29883: ACTGAGACCGTGGTGGGTCAGCACCTGAAGCCAACATAGCACTGGGTATT  Hsapiens
  349621: ..................................................  Ptroglodytes
  367665: ..................................................  Ggorilla
  321174: .........A........................................  Nleucogenys
  396232: ........T....................................A....  Mmulatta
  379686: ....G..G....C.A.....................C..T...A......  Cjacchus
 1028814: ....G..GT...C..A....................C..T..........  Sboliviensis
```

```
                                   LINE1
          -------------------------------------------------->
                   29942     29952     29962     29972     29982
                       :         :         :         :         :
   29933: GCCCAAGGCCCTTTCCTTCACAGCAACAAGTTCCTCCAAGCTGCAGGCAT  Hsapiens
  349671: ..................................................  Ptroglodytes
  367715: ..................................................  Ggorilla
  321224: ............................................G.....  Nleucogenys
  396282: ..................................................  Mmulatta
  379736: ....................TGA...-.....T...T.....CT......  Cjacchus
 1028864: .................C..TGA.........T.........C.......  Sboliviensis
```

```
                                   LINE1
          -------------------------------------------------->
                   29992     30002     30012     30022     30032
                       :         :         :         :         :
   29983: GTCCATGGATGTTGTCTGAGAGCCAGAGATTGGAGTGAAAAACCTTAGCA  Hsapiens
  349721: ...........C......................................  Ptroglodytes
  367765: ...........C.........................G............  Ggorilla
  321274: ...........C......................................  Nleucogenys
  396332: ......A....C...................................C..  Mmulatta
  379785: ...T.......C..............G.......A.A.....-----A..  Cjacchus
 1028914: ...T.......C.....AG.......G.......A.A.....-----A..  Sboliviensis
```

```
                                   LINE1
          -------------------------------------------------->
                   30042     30047     30057     30067     30077
                       :         :         :         :         :
   30033: ACTTACTTGAT-----ATTCTACTGCTGCTAAGCTGTCATTCAAATCACA  Hsapiens
  349771: ...........-----..................................  Ptroglodytes
  367815: ...........-----..................................  Ggorilla
  321324: ....G......-----..........CA...G..................  Nleucogenys
  396382: ...........-----......................T...........  Mmulatta
  379830: .T......A..GTTAT..........A.......................  Cjacchus
 1028959: .T...T.....GTTAT..........A....................G..  Sboliviensis
```

```
                                   LINE1
          -------------------------------------------------->
                   30087     30096     30106     30116     30126
                       :         :         :         :         :
   30078: ATACAAAGAC-TTTCTTTCTCTTCCCTCCCCTTTCCACAGGCAGAGGAGG  Hsapiens
  349816: ..........-......G................................  Ptroglodytes
  367860: ..........-......G...............................-  Ggorilla
  321369: ..........-..C...-................................  Nleucogenys
  396427: ..........-......G................................  Mmulatta
  379880: ..........T......A...................TG.....T....-  Cjacchus
 1029009: ......G...-......A...............CT..T......T....-  Sboliviensis
```

```
                                   LINE1
          -------------------------------------------------->
                   30136     30146     30156     30166     30176
                       :         :         :         :         :
   30127: AGCCTCTCCCTGTGGTGACCACCACCACTGATCCACACAGGTTCTGCAAG  Hsapiens
  349865: ..................................................  Ptroglodytes
  367908: --.....................T..................C.......  Ggorilla
  321417: ................C..............................C..  Nleucogenys
  396476: ................C..............................C..  Mmulatta
  379929: --.....T........C..............................C..  Cjacchus
 1029057: --G..T.T........C.......G......................C..  Sboliviensis
```

```
                   LINE1
          ------------------------------------------------->
                   30186     30196     30206     30216     30226
                       :         :         :         :         :
  30177: GCCACTGCTGATGTTCACTTAAAGCCCAAGGGCTCATTTGTCCACTTGTG  Hsapiens
 349915: ..................................................  Ptroglodytes
 367956: ........................A.........................  Ggorilla
 321467: .......................A..........................  Nleucogenys
 396526: .....C............................................  Mmulatta
 379977: ....T.............................T......T........  Cjacchus
1029105: .............C...T......T.........T......T..-..A..  Sboliviensis

                   LINE1
          ------------------------------------------------->
                   30236     30246     30256     30266     30276
                       :         :         :         :         :
  30227: ATAAATGCTGCCAAGCCTGGGACTCATCTTTCAGGGCAGTCAAGTCCTGC  Hsapiens
 349965: ..................................................  Ptroglodytes
 368006: .................................................T  Ggorilla
 321517: ..........................C................C------  Nleucogenys
 396576: ..........................C...............GC.....T  Mmulatta
 380027: ..............A...........C...............GT...CTT  Cjacchus
1029154: ......A.......A...........C...............GC...CTT  Sboliviensis

                   LINE1
          ------------------------------------------------->
                   30286     30296     30306     30316     30326
                       :         :         :         :         :
  30277: CTGTCCTAGGGTAGATCCAGAAATGGTGTCCAAGAGCTTAGGCCTGGACT  Hsapiens
 350015: ..................................................  Ptroglodytes
 368056: ....................T.............................  Ggorilla
 321561: -----------------------........G..................  Nleucogenys
 396626: .....T......G.....................................  Mmulatta
 380077: .........................A.A......................  Cjacchus
1029204: ...........................AA....................G  Sboliviensis

                   LINE1
          ------------------------------------------------->
                   30336     30346     30356     30366     30376
                       :         :         :         :         :
  30327: CAGGGACCCCAAGAGCCTACTTGTTGTTCTACTCCACTGTGGCCAAGCTG  Hsapiens
 350065: ..................................................  Ptroglodytes
 368106: ..................................................  Ggorilla
 321588: ..............................G...................  Nleucogenys
 396676: ........T......................................T..  Mmulatta
 380127: TG....TG.......T..........C...CT..T...............  Cjacchus
1029254: TG.............T..........C....T..T...............  Sboliviensis

                   LINE1
          ------------------------------------------------->
                   30386     30396     30406     30416     30426
                       :         :         :         :         :
  30377: GTACCTAAGGTGCAAGACAAAGTCCCCCTTTACTTTCCCCTCTGCTTCTC  Hsapiens
 350115: ..................................................  Ptroglodytes
 368156: ..................................................  Ggorilla
 321638: ...........T........................T.............  Nleucogenys
 396726: ....................................T.............  Mmulatta
 380177: ...........................-.....C..T........C....  Cjacchus
1029304: .........................T.-...C....T.............  Sboliviensis
```

```
                                          LINE1
          ---------------------------------------------------------------->
                 30436     30446     30456                 30474
  30427: AAAAAGATGGAGCCTTTCACTATAGCCACCACAGCTGG--ACGTGCTTGG  Hsapiens
 350165: .....................................A--..........  Ptroglodytes
 368206: ......................................--..........  Ggorilla
 321688: ......G...............................--.T........  Nleucogenys
 396776: ...C..............................T...--..........  Mmulatta
 380226: ...C..G.....T..................T.A....GA..A....G..  Cjacchus
1029353: ...C........T..................T..T...GA....A..G..  Sboliviensis

                                          LINE1
          ---------------------------------------------------------------->
                 30484     30494     30504     30514     30524
  30475: GCTCACCTGAAGTCAGCACATTTCAGAGCCCAAGGCCCACAGCGTACTAC  Hsapiens
 350213: ..................................................  Ptroglodytes
 368254: ..................................................  Ggorilla
 321736: ........A.........................................  Nleucogenys
 396824: .....T............................................  Mmulatta
 380276: T....................C....................T.......  Cjacchus
1029403: T....................C....................T.......  Sboliviensis

                                          LINE1
          ---------------------------------------------------------------->
                 30534     30544     30554     30564     30574
  30525: CTGAGTATTGCTGCTGGTTATTCAGGAACCAAGGGCTCCTTAGTCAGCAG  Hsapiens
 350263: ..................................................  Ptroglodytes
 368304: ..................................................  Ggorilla
 321786: ..............C...................................  Nleucogenys
 396874: ..................................................  Mmulatta
 380326: ..................................AT..T..........C  Cjacchus
1029453: ......G...........................A...T.G.....A...  Sboliviensis

                                          LINE1
          ---------------------------------------------------------------->
                 30584     30594     30604     30614     30624
  30575: ATTATGAATTCTGCCAGGCACTGGTCCTTCCCTTCAAGGTAGCAGGTTTC  Hsapiens
 350313: ..................................................  Ptroglodytes
 368354: ..................................................  Ggorilla
 321836: .............A....AGTG............................  Nleucogenys
 396924: ..G.C.............AGTG........T...................  Mmulatta
 380376: ..G..T...........AAGTG...T...............T..CAC...  Cjacchus
1029503: ..G..T............AGTG...................A..CAC...  Sboliviensis

                                          LINE1
          ---------------------------------------------------------------->
                 30634     30644     30654     30664     30674
  30625: CTTTTGGCTCAGGTTGTCTCTAGAAATGTCCAGGAGCCAGGTCCTGGAAC  Hsapiens
 350363: ..................................................  Ptroglodytes
 368404: ..................................................  Ggorilla
 321886: ....C............................................T  Nleucogenys
 396974: ......T...................C......................T  Mmulatta
 380426: .................G.....................A....A.AT.T  Cjacchus
1029553: ...............T.G.....................A...TG.AT.T  Sboliviensis
```

```
                          LINE1
         ------------------------------------------------->
                   30684     30694     30704     30714     30724
                       :         :         :         :         :
  30675: GGGGGCCTCACAACTCTGCCTGGTGCCCTATCTTACTGTGGCTGAGCTGG  Hsapiens
 350413: ..................................................  Ptroglodytes
 368454: .....T............................................  Ggorilla
 321936: ............G.....................................  Nleucogenys
 397024: ..........T...................A.....G.............  Mmulatta
 380476: ..A.................G................A..A..T......  Cjacchus
1029603: ..A.............CA............C......A..A.........  Sboliviensis

                          LINE1
         ------------------------------------------------->
                   30734     30744     30754     30764     30774
                       :         :         :         :         :
  30725: TATCCAAGATGCAAGACAAAGTCCTCTTTGCTCTTTGTTCTCCTCTTGTT  Hsapiens
 350463: ..............C...................................  Ptroglodytes
 368504: ............................................T.....  Ggorilla
 321986: ............................C.....C.......T.......  Nleucogenys
 397074: ..................................................  Mmulatta
 380526: ................A............A............---.....  Cjacchus
1029653: ....T........................AT...........---.....  Sboliviensis

                          LINE1
         ------------------------------------------------->
                   30784     30794     30804     30814     30824
                       :         :         :         :         :
  30775: AAATAGAAAGAAAGAATCACTTTTATTGCTGTGAGCTTCACTGCCTGGGG  Hsapiens
 350513: ........G.........................................  Ptroglodytes
 368554: ........G.........................................  Ggorilla
 322036: ........G...............G............G............  Nleucogenys
 397124: ........................G............G.T..T.......  Mmulatta
 380573: .....AG.G...GA.G........G.CAT...A....G........T.T.  Cjacchus
1029700: ........G...G..G............T...A....G..........T.  Sboliviensis

                          LINE1
         ------------------------------------------------->
                   30834     30844     30854     30864     30874
                       :         :         :         :         :
  30825: TCGGGGGAGGGGTGACACCAGCACTCCCTTAGAAGCTAGTGTCTCCCTAG  Hsapiens
 350563: ..................................................  Ptroglodytes
 368604: ..................................................  Ggorilla
 322086: ..A........................T......................  Nleucogenys
 397174: ......................G.............A.............  Mmulatta
 380623: ......A...........A................T..............  Cjacchus
1029750: .T..A.A...........A....T...........TG.....T.......  Sboliviensis

                          LINE1
         ------------------------------------------------->
                   30884     30894     30904     30914     30924
                       :         :         :         :         :
  30875: GTCACGTGCCACTCTAGAACTCTGGCTCTGAGCCCAGCTTAGCACTAGGA  Hsapiens
 350613: ..................................................  Ptroglodytes
 368654: ..........................................T.......  Ggorilla
 322136: ...........TC.....C........G..........C...........  Nleucogenys
 397224: ............C.....C...................C...........  Mmulatta
 380673: .....A......C....T....................C...........  Cjacchus
1029800: .....A......C....TC......A......T.....C...........  Sboliviensis
```

```
                         LINE1
      ------------------------------------------------------->
                30934     30944     30954     30964     30974
  30925: ATTTCCTAGGAATTGCAGTCCTTGTGTCCTACATTGTCTTTCAAGCTTAT Hsapiens
 350663: .................................................. Ptroglodytes
 368704: ..............T................................... Ggorilla
 322186: ...........................T.....................C Nleucogenys
 397274: ...............A............T....................C Mmulatta
 380723: G..............................G.C...............C Cjacchus
1029850: G........C..........G..........G.C...............C Sboliviensis
```

```
                         LINE1
      ------------------------------------------------------->
                30984     30994     31004     31014     31024
  30975: CTAAAACCCCAAAGCACTTCAGCCTGTGGTGGCAAGGCTTTGCCAAGAAA Hsapiens
 350713: .................................................. Ptroglodytes
 368754: .................................................. Ggorilla
 322236: .................................................. Nleucogenys
 397324: ...............G...T.................T....T......G Mmulatta
 380773: ............G.TG...T........A..................G.. Cjacchus
1029900: T.............TG...T........A......T.............. Sboliviensis
```

```
                         LINE1
      ------------------------------------------------------->
                31034     31044     31054     31064     31074
  31025: CTCGAGTTCCAACCACTGGGATGGGTGAATCCCCTCTGGCTAGGGCTGGT Hsapiens
 350763: ...A.....................C..................C...T. Ptroglodytes
 368804: ...A.....................C............A........... Ggorilla
 322286: ...A.....................CA....................... Nleucogenys
 397374: ...T...............C.............................. Mmulatta
 380823: ...A......C.T...............G.........AT.......... Cjacchus
1029950: ...A.......GT...............G..T......AT....A..... Sboliviensis
```

```
                         LINE1
      ------------------------------------------------------->
                31084     31094     31104     31114     31124
  31075: CCAAATGCTCACTTCGGGCAAGGGTGAGCTGGCTGAGCCCAGCACGGCCT Hsapiens
 350813: .................................................. Ptroglodytes
 368854: .................................................. Ggorilla
 322336: ................T................................. Nleucogenys
 397424: ................T................................. Mmulatta
 380873: ..........C....TT........A......................T. Cjacchus
1030000: T.....T...C....TT.....................T.C....A..T. Sboliviensis
```

```
                         LINE1
      ------------------------------------------------------->
                31134               31145     31155     31165
  31125: TATTCTCTGCTAAG---------CTTTATTCTCTACTGTGATAGAGCAGT Hsapiens
 350863: ...........C..---------........................... Ptroglodytes
 368904: ..............---------........................... Ggorilla
 322386: ..............---------............T.............. Nleucogenys
 397474: .......C.....A---------........................... Mmulatta
 380923: ..............TCTGCTAAG.....C.............-......C Cjacchus
1030050: .G............TCTGCTAAG....C.............C-.....TC Sboliviensis
```

```
                          LINE1
         ------------------------------------------------->
              31175     31185     31194     31204     31214
  31166: ACTGAATTCAATGTAAAATCCC-AAGTTGCTGCACTCTCCCTCCCTCAAG  Hsapiens
 350904: ......................-...........................  Ptroglodytes
 368945: ..........G...........-...........................  Ggorilla
 322427: ......................-..........CT...............  Nleucogenys
 397515: .........C............-...........................  Mmulatta
 380972: .....C......A....G....C....CAA..........T.........  Cjacchus
1030099: .....C......A....G....C....CA...........T.........  Sboliviensis

                          LINE1
         ------------------------------------------------->
              31224     31234                         31246
  31215: GGCATAGACTCTTTCTCCATGTTGCATGG------------------GGA  Hsapiens
 350953: .............................------------------...  Ptroglodytes
 368994: ..........G........C.........------------------...  Ggorilla
 322476: .............................------------------...  Nleucogenys
 397564: .......................T.....CCACTGCGGAAGAGATGG...  Mmulatta
 381022: .T.......----.........CA.....CCGCTGCTGAAGAGATGG...  Cjacchus
1030149: .....G................C......CTGCTGCAGAAGAGATGG...  Sboliviensis

                          LINE1
         ------------------------------------------------->
              31256     31266     31276     31286     31296
  31247: AAGGGTGGCATCGACAATTCAAGATTGTCTCTCCTGCCCCCCTCAATTCC  Hsapiens
 350985: ..................................................  Ptroglodytes
 369026: ......................................A...........  Ggorilla
 322508: ............AG.G....G.............................  Nleucogenys
 397614: ...........T.G.GG.......................-....T....  Mmulatta
 381068: ...........T.GTG........C.A......G......T.........  Cjacchus
1030199: ...........T.GTG........C...............T.........  Sboliviensis

                          LINE1
         ------------------------------------------------->
              31306     31316     31326     31336     31346
  31297: TCTTTCCAGGATATGAAGTTAAAACCAGTTACTATGATTGCTCACCTGAT  Hsapiens
 351035: ...........C.....................G................  Ptroglodytes
 369076: .................................G..........G.....  Ggorilla
 322558: .................A...............G................  Nleucogenys
 397663: ........A..C.....................G.........C......  Mmulatta
 381118: ....................GC......G...CG................  Cjacchus
1030249: .....................C......G.....................  Sboliviensis

                LINE1                     Alu
         -------------------->---------------------------->
              31356     31366
  31347: TTTTGGTTCTCGTGACACTGCTTTT-------------------------  Hsapiens
 351085: ..........T..............-------------------------  Ptroglodytes
 369126: ..........T..............-------------------------  Ggorilla
 322608: ..........T......G.......-------------------------  Nleucogenys
 397713: ........T.T......G.......-------------------------  Mmulatta
 381168: ....-.....T......G.......CTTTCC-------------------  Cjacchus
1030299: ..G.......T......G..T....CTTTTCTTTTCTTTTCTTTTTTCTT  Sboliviensis
```

```
                                  Alu
           ------------------------------------------------------>
                   31380     31384     31394     31404     31414
                       :         :         :         :         :
   31372: -TTTTTTTTTTT------AGACTAAGTTTCACTCATGTCACCCAGGCTGG  Hsapiens
  351110: -...........TT----.......A........................  Ptroglodytes
  369151: -...........TTTTT-................................  Ggorilla
  322633: -...........------...........................A....  Nleucogenys
  397738: -...........TTTTAA....................T....CA.....  Mmulatta
  381198: -....C......TTTTT-...TG......TG..TT.......T.......  Cjacchus
 1030349: T...........TTTTT-...TG......GG..TT...............  Sboliviensis

                                  Alu
           ------------------------------------------------------>
                   31424     31434     31444     31454     31464
                       :         :         :         :         :
   31415: AGTGCAATGGTGCAATCTTGGCTAACTGCAACCTCCACCTCCCAGATTCA  Hsapiens
  351155: ..........CA.......................T..............  Ptroglodytes
  369199: ..........C.......................................  Ggorilla
  322676: ....T.....CA......................................  Nleucogenys
  397787: ..........C.TG....................................  Mmulatta
  381246: .A........CA.G.C.......C.....................G....  Cjacchus
 1030398: ...A......CA.G.........C.........C...........G....  Sboliviensis

                                  Alu
           ------------------------------------------------------>
                   31474     31484     31494     31504     31514
                       :         :         :         :         :
   31465: AGAGATTCTCCTGCTTCAGCCTCACGAGTAGCTGGGATTACAGGTGCCCA  Hsapiens
  351205: ..............C...................................  Ptroglodytes
  369249: ..............C...................................  Ggorilla
  322726: ..C...........C........C..........................  Nleucogenys
  397837: ..............C........C..........................  Mmulatta
  381296: ..CA......T...C.T......CT.........C........C.T..T  Cjacchus
 1030448: ..CA...A..T...C.T......CT........A..........C.....  Sboliviensis

                                  Alu
           ------------------------------------------------------>
                   31524     31534     31544     31554     31563
                       :         :         :         :         :
   31515: CTACTATACCCAGCTAATTTTGTGTATTTTTAGTAGAGAT-GGGGTTTCA  Hsapiens
  351255: ...............G........................-.........  Ptroglodytes
  369299: ...............C........................-.........  Ggorilla
  322776: ...........G............................G.........  Nleucogenys
  397887: ...........GA..........................C-.........  Mmulatta
  381346: .C....C......AA............A............-.........  Cjacchus
 1030498: .C....C......A.............A............-.........  Sboliviensis

                                  Alu
           ------------------------------------------------------>
                   31573     31583     31593     31603     31613
                       :         :         :         :         :
   31564: CCGTATTGGCCAGGCTGGTCTCGAACTCCTGACCTCCGGTGATCCACCGG  Hsapiens
  351304: ................................................A.  Ptroglodytes
  369348: .T................................................  Ggorilla
  322826: ..A...................A.............T...........A.  Nleucogenys
  397936: ..A.................................A...........C.  Mmulatta
  381395: ..A......T............A..........TG.A...-.......T.  Cjacchus
 1030547: ..A.G....T............A...A.........A...........T.  Sboliviensis
```

```
                                Alu
          -------------------------------------------------->
               31623     31633     31643     31653     31663
                   :         :         :         :         :
   31614: CCTCAGCCTCCCAAAGTACTGGGATTGCAGTTGTAAGCCACCACACCCAG  Hsapiens
  351354: ..................................................  Ptroglodytes
  369398: ..................................................  Ggorilla
  322876: .G........................A.......G.......GTG.....  Nleucogenys
  397986: ..........T...............A.......G.........G.....  Mmulatta
  381444: ....G.GT.........G........A...GCA...A.A....TG.....  Cjacchus
 1030597: ....G.GT.........G........A...GCA.....A....TG.....  Sboliviensis

          Alu                     LINE1
          -->----------------------------------------------->
               31673     31683     31693     31703     31713
                   :         :         :         :         :
   31664: CCATGACAGTGCTTTTCTATGTGCAGATAGTTATTAAAATCTGGTGTTCC  Hsapiens
  351404: ......T...........................................  Ptroglodytes
  369448: ..G...............................................  Ggorilla
  322926: G.......................................T.........  Nleucogenys
  398036: ....................A...............G...G.........  Mmulatta
  381494: .....G..................................T.........  Cjacchus
 1030647: ..G....T...................C.....C......T.......T.  Sboliviensis

                                LINE1
          -------------------------------------------------->
               31723     31733     31743     31753     31763
                   :         :         :         :         :
   31714: AGCAGGGGAAACAAGTGGTATAGGCTTCTATTCTGACATCTTGCTCCGCC  Hsapiens
  351454: ..............................................T...  Ptroglodytes
  369498: ..............................................T...  Ggorilla
  322976: ...................................C.....C....T...  Nleucogenys
  398086: ....................G..............C..........T...  Mmulatta
  381544: ...........TG...T.GG...............C.........T.-..  Cjacchus
 1030697: .......C...TG.....GG...............C..........T..T  Sboliviensis

                                                     Alu
                                                   ------->
               31773     31783     31793     31803     31813
                   :         :         :         :         :
   31764: CACACAAAACTCCCAATTTCCTTCTTTAAAAATCAATGTTCTTGGCCAGA  Hsapiens
  351504: ...................................G..............  Ptroglodytes
  369548: ..................................................  Ggorilla
  401106:                                            .......  Pabelli
  323026: .........T...........................A...........G  Nleucogenys
  398136: .....................................A...........G  Mmulatta
  381593: T..........T..................T....G.A...........G  Cjacchus
 1030747: ...C.C.....A.....................T...A........A..G  Sboliviensis

                                Alu
          -------------------------------------------------->
               31823     31833     31843     31853     31863
                   :         :         :         :         :
   31814: CACGGTGGCTCACGCCTGTAATCCCAGCGCTTTGGGAGGCCGAGAAGAGT  Hsapiens
  351554: .............A...........................A.....C..  Ptroglodytes
  369598: ..................................................  Ggorilla
  401113: .....C......T...............A............A........  Pabelli
  323076: T.........                                          Nleucogenys
  398186: ..T.........................A...............G.....  Mmulatta
  381643: ...A......T..A..............T............A..GT.G..  Cjacchus
 1030797: ...A..T......A..............T............A..GT.G..  Sboliviensis
```

```
                              Alu
          --------------------------------------------------->
             31873     31883     31893     31903     31913
    31864: GGATCATTTGAGGTCAGGAGTTTGAGACCAGCCTGATCAAGATGGTGAAA  Hsapiens
   351604: ....................................C.............  Ptroglodytes
   369648: .......................C............C.............  Ggorilla
   401163: ....................................C...C......---  Pabelli
   398236: ........................CA....AT....C...C.........  Mmulatta
   381693: ...........................G........C...T.....C...  Cjacchus
  1030847: .A..................................C...T.....C...  Sboliviensis

                              Alu
          --------------------------------------------------->
             31923     31933     31943     31953     31963
    31914: CCCCGTCTCTACTAAAAATACAGAAAAATTAGCCAGGCATGGTGGCACAC  Hsapiens
   351654: ..................................................  Ptroglodytes
   369698: ..................................................  Ggorilla
   401210: ----..............................................  Pabelli
   398286: ..................................----.C.....A....  Mmulatta
   381743: .T..A..........C......A.G............A...........T  Cjacchus
  1030897: ....A.................A.G......A..................  Sboliviensis

                              Alu
          --------------------------------------------------->
             31973     31983     31993     32003     32013
    31964: TCCTGTAATCCCAGCTACTTGGAGGCTGAGGCAGGAGAATCACTTGAACC  Hsapiens
   351704: ..................................................  Ptroglodytes
   369748: ..................................................  Ggorilla
   401256: ..................................................  Pabelli
   398332: .............A....................................  Mmulatta
   381793: G...............G............AA..C......T.T.......  Cjacchus
  1030947: G..........T....G..C.........A...T......T.T.......  Sboliviensis

                              Alu
          --------------------------------------------------->
             32023     32033
    32014: TGGGAGACGGAGGTTGCAGTGAGCCAAGA---------------------  Hsapiens
   351754: .............................---------------------  Ptroglodytes
   369798: .............................NNNNNNNNNNNNNNNNNNNNN  Ggorilla
   401306: .......T.....................---------------------  Pabelli
   398382: C.....G.....C................---------------------  Mmulatta
   381843: CA....GTA..AA......GA.......----------------------  Cjacchus
  1030997: CA....GTA..AA.......A.......----------------------  Sboliviensis

                              Alu
          --------------------------------------------------->
                                     32051     32061     32071
    32043: ---------------------ATCATGCAACTGCACTCCAGCCTGGGTGA  Hsapiens
   351783: ---------------------....C..C.....................  Ptroglodytes
   369848: NNNNNNNNNNNNNNNNNNNNN.----------------------------  Ggorilla
   401335: ---------------------....C..C.....................  Pabelli
   398411: ---------------------....C..CG..........CA........  Mmulatta
   381871: ---------------------..T....C..C.......G.......CAG  Cjacchus
  1031025: ---------------------....A..-------...AG.......CA.  Sboliviensis
```

```
                                  Alu
                        32090     32100     32110
 32072:  CAGAGTGAG-TTCCATCTCAAAAAAAAAAAAAGAAAAGAAAA--------  Hsapiens
351812:  .........-.......G.........G..............GAAAA---  Ptroglodytes
369870:  -------------------.......................GAAAA---  Ggorilla
401364:  .........AC..................G..A.----------------  Pabelli
398440:  .........AC.....T.............G.A..G.A....AAGAAAAA  Mmulatta
351123:                     ......G...G..A....AG...--------  Panubis
381900:  .........TC..TG.........T...                        Cjacchus
382216:                              .T..A.T..A.T..GTAAATAA  Cjacchus
1031047: .........TC..TG.........T...TT--------------------  Sboliviensis

                Alu                           LINE1
                        32115     32125     32135     32145
 32113:  -----------------AATTAGTATTCTTAATTTTTTTGAAGAAGTTTT  Hsapiens
351858:  -----------------...........................C.....  Ptroglodytes
369898:  -----------------.................................  Ggorilla
401398:  -----------------.................................  Pabelli
398490:  -----------------......C......G.......-A.....A....  Mmulatta
351146:  -----------------......C..--C.........-..........-  Panubis
382238:  ATAAATTAAATTAAATT.....A....T......................  Cjacchus
1031077: -----------------.....A...........................  Sboliviensis

                                 LINE1
              32155     32165     32175     32185     32195
 32146:  AGGTTTAAAGAAAAATAGACCAGAGAGTATAGGGTTCCCATATACTCCCT  Hsapiens
351891:  ...................G..............................  Ptroglodytes
369931:  ...................G..............................  Ggorilla
401431:  ...................G..............................  Pabelli
398522:  ..T................G......A.................T...--  Mmulatta
351175:  -A.................G......A.................T.....  Panubis
382288:  .......C...........G....A..C...A......T........T..  Cjacchus
1031110: .......C...........G....A..C...A......T........T..  Sboliviensis

                                 LINE1
              32205     32215     32225     32235     32245
 32196:  TAGTCCTTCCCTCCCAGTTTTTCCTGCTATTAACATTTTTCATTAGTGTT  Hsapiens
351941:  .......................................G..........  Ptroglodytes
369981:  ..................................................  Ggorilla
401481:  .......................................G..........  Pabelli
398570:  ......C..............................C.G..........  Mmulatta
351224:  ......C................................G..G.......  Panubis
382338:  .....TC...T..............A.......T.....G..........  Cjacchus
1031160: ....ATC...T.....A........A.AC....T.....G.....T....  Sboliviensis
```

```
                                       LINE1
         -------------------------------------------------->
                  32255     32265     32275     32285     32295
                      :         :         :         :         :
   32246: GTACATTTGTTATAATTGATGAACTAATATTATTATTAATTTAGATAGCT Hsapiens
  351991: .................................................. Ptroglodytes
  370031: .................................................. Ggorilla
  401531: ......A........................................... Pabelli
  323084:                        ............G.............. Nleucogenys
  398620: ......................G.C.......................T. Mmulatta
  351274: A...................A.G.........................T. Panubis
  382388: A.....................T............---......----T. Cjacchus
 1031210: A...............G...---............---......----T. Sboliviensis

               LINE1                 LINE1
         --------------->----------------------------------->
                  32305     32315     32325     32335     32345
                      :         :         :         :         :
   32296: ATTAACTAAATACAGTATTGTTGACTAAATTCTATAGTTTACATTAGAGT Hsapiens
  352041: .......................................C.......... Ptroglodytes
  370081: .................................................. Ggorilla
  401581: .................................................. Pabelli
  323111: .................................................. Nleucogenys
  398670: C...........G....................G................ Mmulatta
  351324: ............G....................G................ Panubis
  382431: ............T......A...........T.........T........ Cjacchus
 1031250: ............T......A.............G.......A........ Sboliviensis

                                       LINE1
         -------------------------------------------------->
                  32355     32365     32375     32385     32395
                      :         :         :         :         :
   32346: TCAATCTTTCTGTTGTATATCCTATGGGTTTTAACAAATGTACAATGGCG Hsapiens
  352091: ....................-....................G.......A Ptroglodytes
  370131: .................................................. Ggorilla
  401631: .................................................A Pabelli
  323161: .........G.....................................TTA Nleucogenys
  398720: ...........C.....C................................ Mmulatta
  351374: .................C...............................A Panubis
  382481: ...G.......................CG.........CA.........A Cjacchus
 1031300: ...G........................................G....A Sboliviensis

                                       LINE1
         -------------------------------------------------->
                  32405     32415     32425     32435     32445
                      :         :         :         :         :
   32396: TGCATTTTCCATTACAATATCATAGAAAATAGTTTCACTGCCCTATAAAT Hsapiens
  352140: .................................................. Ptroglodytes
  370181: ..........G....................................... Ggorilla
  401681: ................G................................. Pabelli
  323211: ................G............G.................... Nleucogenys
  398770: ................GC..........G.........C......G.... Mmulatta
  351424: ................G.....................C......G.... Panubis
  382531: .....CC...........G..........................G.... Cjacchus
 1031350: .....C.......................................G.... Sboliviensis
```

```
                                    LINE1
           ------------------------------------------------------------->
                    32455     32465     32475     32485     32495
   32446: CCTCTGTGCTTCACCTATTCATCCCTCCCTTCCTCTCCCTAGTCCCCTGA  Hsapiens
  352190: ..................................................  Ptroglodytes
  370231: ......................................G...........  Ggorilla
  401731: ..................................................  Pabelli
  323261: .......................................C..........  Nleucogenys
  398820: .......................................C..........  Mmulatta
  351474: ......................................GC..........  Panubis
  382581: ..........-.........................T..C...T....C.  Cjacchus
 1031400: ..........-.........................T..C...T.A..C.  Sboliviensis

                                    LINE1
           ------------------------------------------------------------->
                    32505     32515     32525     32535     32544
   32496: CAACCACTAATCTTTATATTGTCTCCACAGTTTTGTCCTTCCC-GAATGT  Hsapiens
  352240: ...........................T...............-......  Ptroglodytes
  370281: ...........................T...............-......  Ggorilla
  401781: ...........................T.........T.....-......  Pabelli
  323311: ...T.......................T..-............-......  Nleucogenys
  398870: ......................T....................A......  Mmulatta
  351524: ................C.....T....................A......  Panubis
  382630: .............................A.............A.....C  Cjacchus
 1031449: .......C.G.................................A......  Sboliviensis

                                    LINE1
           ------------------------------------------------------------->
                    32554     32564     32574     32584     32594
   32545: TTTAAGTTGGGATCATATAGTATGCAGCTTTTCAGACTGGCTTCTTTTAC  Hsapiens
  352289: ..................................................  Ptroglodytes
  370330: ..................................................  Ggorilla
  401830: .C................................................  Pabelli
  323359: .C.............................-............-.....  Nleucogenys
  398920: .C...A.................................A..........  Mmulatta
  351574: .C...A.................................A..........  Panubis
  382680: .C........A.............T........CA...............  Cjacchus
 1031499: .C...T....A.............T..T...C..-...............  Sboliviensis

                                    LINE1
           ------------------------------------------------------------->
                    32604     32614     32624     32634     32644
   32595: TTAGTAATATGCATTTAAGTTCCTCCATGTCTTTTCATGGCTAGGTAGTT  Hsapiens
  352339: ..................................................  Ptroglodytes
  370380: ..................................................  Ggorilla
  401880: ....................................G.............  Pabelli
  323407: ..............C...................................  Nleucogenys
  398970: .........................T.............T..G.A.....  Mmulatta
  351624: .......................................T..G.A.....  Panubis
  382730: ...........................CA..................T..  Cjacchus
 1031548: ..................A..............................C  Sboliviensis
```

```
                                LINE1
         ------------------------------------------------------>
                   32654     32664     32674     32684     32694
                       :         :         :         :         :
  32645: CATTTCTTTTTACTGCTGAATAATATTCCATTGCATAAATGTACCACAGT  Hsapiens
 352389: ..........C.......................................  Ptroglodytes
 370430: ..................................................  Ggorilla
 401930: ................................A.................  Pabelli
 323457: ................................A.................  Nleucogenys
 399020: .......................C.........T........G.......  Mmulatta
 351674: .......................C..................G.......  Panubis
 382780: G.....................G............C...........T..  Cjacchus
1031598: .....A.........................................T..  Sboliviensis

                                LINE1
         ------------------------------------------------------>
                   32704     32714     32724     32734     32744
                       :         :         :         :         :
  32695: TTTTTAATCATTCATGTATTGAAGGACATTTGGTTGCTTCCAAGTTTTTG  Hsapiens
 352439: ..................................................  Ptroglodytes
 370480: ..................................................  Ggorilla
 401980: ..................................................  Pabelli
 323507: ..............A....C..............................  Nleucogenys
 399070: ............A...C.................................  Mmulatta
 351724: .G..........A...C.................................  Panubis
 382830: A...A.TC.G.......................G.........-......  Cjacchus
1031648: ....A.TC.................................G.T......  Sboliviensis

                                LINE1
         ------------------------------------------------------>
                   32754     32764     32774     32784     32794
                       :         :         :         :         :
  32745: GCAATAATGAATAAAGCTGCTATAAACATTTGTGTGCAGGTTTTTGTGTA  Hsapiens
 352489: ..................................................  Ptroglodytes
 370530: ...............A..................................  Ggorilla
 402030: ..................................................  Pabelli
 323557: .................................A...............G  Nleucogenys
 399120: .................................A................  Mmulatta
 351774: ................................C.................  Panubis
 382879: .............G.....................A..............  Cjacchus
1031698: .............G.....................A..............  Sboliviensis

                                LINE1
         ------------------------------------------------------>
                   32804     32814     32824     32834     32844
                       :         :         :         :         :
  32795: GACATAAATTTTCAGGTCATCTGGGTAAATACCAAAGAGTGAAACTGCTG  Hsapiens
 352539: ..................G.T.............................  Ptroglodytes
 370580: ....................T....................C........  Ggorilla
 402080: ....................T........C...........C........  Pabelli
 323607: ....................T...A................C........  Nleucogenys
 399170: ....................T...................AC........  Mmulatta
 351824: ....................T................C..ACC.......  Panubis
 382929: ...............C....T.C.T..........T.GA..C.....T..  Cjacchus
1031748: CC.............C....T..............T.GA..C....AT..  Sboliviensis
```

```
                          LINE1
          ------------------------------------------------->
                 32854     32864     32874     32884
   32845: GATTGTATGGCAATAGCATGTTTAGTTTTATAAGATACTGAC--------  Hsapiens
  352589: ..........................................--------  Ptroglodytes
  370630: ....................C.....................--------  Ggorilla
  402130: .......................C.........A........--------  Pabelli
  323657: ........................A.................--------  Nleucogenys
  399220: ........A..........................C....C.--------  Mmulatta
  351874: ........A....................G.....C....C.--------  Panubis
  382979: .............A.A..........................AAGCTATC  Cjacchus
 1031798: .....C.......C.A..........................AAGCTGTC  Sboliviensis

                          LINE1
          ------------------------------------------------->
                 32894     32903     32913     32923     32933
   32887: --CCAAAATACCACCTT-TTATACCCATAAGCTATGAGTGACATAGTTTC  Hsapiens
  352631: --...............-..............A.................  Ptroglodytes
  370672: --...............-..............A.................  Ggorilla
  402172: --...............-..............A.................  Pabelli
  323699: --.......T.......-..............A.................  Nleucogenys
  399262: --............T..-..............A.................  Mmulatta
  351916: --............T..-..............A.................  Panubis
  383029: TT............A.--....T.........A.....C..G.G......  Cjacchus
 1031848: TT...........TT..T....T...G.....A....ACC...G......  Sboliviensis

                          LINE1
          ------------------------------------------------->
                 32943     32953     32963     32973     32983
   32934: GGTTGCTCCACATGCTCACCAGCATTTGATGTTGTCAGTACTTTAGATTT  Hsapiens
  352678: ..................T........................C......  Ptroglodytes
  370719: .....T............................................  Ggorilla
  402219: .......................................G....G.....  Pabelli
  323746: .......................................G....G.....  Nleucogenys
  399309: .............C....A....................G....G.....  Mmulatta
  351963: .............C....A...............G....G....G.....  Panubis
  383077: T............C.................CC......G....G.....  Cjacchus
 1031898: T............CT................C.......G....G.....  Sboliviensis

                          LINE1
          ------------------------------------------------->
                 32992     33002     33012     33022     33032
   32984: TAGCCATG-CTAATAGGTTTTAATTCGCAATTCTCTAATGACAGATAATG  Hsapiens
  352728: ........-.........................................  Ptroglodytes
  370769: ........-.........................................  Ggorilla
  402269: .......C-.................T.......................  Pabelli
  323796: .......C-.........................T...............  Nleucogenys
  399359: .......C-................T........................  Mmulatta
  352013: G......C-................T........................  Panubis
  383127: .......CAT...............T......T.....C...........  Cjacchus
 1031948: .......C-................T......T.....CA..........  Sboliviensis
```

```
                                LINE1
         ------------------------------------------------->
              33042     33052     33062     33072
    33033: TACAGCATGTTTTCATTTGTTTATTTGCCATCTATGTACCTTCTCT----  Hsapiens
   352777: ..............................................----  Ptroglodytes
   370818: ..............................................----  Ggorilla
   402318: ........A.....................................----  Pabelli
   323845: ........A.....................................----  Nleucogenys
   399408: ........A.....................................----  Mmulatta
   352062: ........A.....................................----  Panubis
   383177: .TG.....A...............C.............T.......TTGC  Cjacchus
  1031997: .CA.....A........................G....T.......----  Sboliviensis
```

```
                                LINE1
         ------------------------------------------------->
                                                      33087
    33079: -----------------------------------------GGTATGGTG  Hsapiens
   352823: -----------------------------------------.........  Ptroglodytes
   370864: -----------------------------------------.........  Ggorilla
   402364: -----------------------------------------....C....  Pabelli
   323891: -----------------------------------------....CA...  Nleucogenys
   399454: -----------------------------------------.A.......  Mmulatta
   352108: -----------------------------------------.A.......  Panubis
   383227: CATCTATGTATCTTCTCTATGTATCTTCTTTATGTGCCATCTA.G.ATCT  Cjacchus
  1032043: -----------------------------------------.........  Sboliviensis
```

```
                              LINE1                  Simple
         ---------------------------------------------> --->
              33097     33107     33117     33127     33137
    33088: TCCATATATTTTGCCCATTTTTAACTAACAGTTTTCTTATTGTTAATTGT  Hsapiens
   352832: .............................T....................  Ptroglodytes
   370873: .............................T....................  Ggorilla
   402373: ................G......T.....T...........T......T.  Pabelli
   323900: ...........................G.T..................T.  Nleucogenys
   399463: .G...............G.........G.T.................A..  Mmulatta
   352117: .G...............G.........G.T.................A..  Panubis
   383277: ..........................GG.T.........A....--....  Cjacchus
  1032052: .........................AGG.T.........A.....T..T.  Sboliviensis
```

```
                                Simple
         ------------------------------------------------->
              33147     33156     33166     33176
    33138: GTGTGTGTGTGT-GTGTGTGTGTGTGTGTGTGTGTGTGTG----------  Hsapiens
   352882: ............-........----.......GT.AG.GA----------  Ptroglodytes
   370923: ............-...........................TGTGGTTAGG  Ggorilla
   402423: ............-------------.......GT..G.G.----------  Pabelli
   323950: .........G.G-.G.G.G.-----.......GC..A.G.----------  Nleucogenys
   399513: .........G.GC.G.G.G.G.G.G.G.GAG..TA.G...----------  Mmulatta
   352167: .........G.G-.G.G.G.-----.C.G.G..TA.G...----------  Panubis
   383325: T.T...T.TGA.-.G-------------------..G.G.----------  Cjacchus
  1032102: ..T.T...T..G-TG.------------------..GTG.----------  Sboliviensis
```

```
           Simple                          Alu
                         33184     33190     33200     33210     33220
   33177: --TTTAGGGACT----CTGTCTCCTGGGCTGAAGTGCAGTGCTGTGATCT  Hsapiens
  352917: --GAC..T.T..TTCT....A.............................  Ptroglodytes
  370972: GAGAC..T.T..TTCT..................................  Ggorilla
  402450: --GAC....T..TTCT..................................  Pabelli
  323984: --GAC....T..TTCT......G...........................  Nleucogenys
  399553: --GAC..A.T..TTCT..................................  Mmulatta
  352201: --GAC..A.T..TTCT..................................  Panubis
  383345: --GAC..A.T..TTCT.............C.G.................A  Cjacchus
 1032123: --GGC..A.T..T----..............G.................A  Sboliviensis

                                     Alu
                         33230     33240     33250     33260     33270
   33221: TAGTTCACTGCAGCCCCTACCTCCTGGGCTCAACTCATCCTCCCACCTCA  Hsapiens
  352965: ..................................................  Ptroglodytes
  371022: ..................................................  Ggorilla
  402498: .....................................G............  Pabelli
  324032: ..................................................  Nleucogenys
  399601: ....................T.............................  Mmulatta
  352249: ....................T.................G...........  Panubis
  383393: C-...T.T.........C.......A.........T..............  Cjacchus
 1032167: C......T.........C.......A.........T.G..........T.  Sboliviensis

                                     Alu
                         33280     33290     33300     33310     33320
   33271: GTCCCCCAAGCAGCTAGGACTAAGGCAGGTACCACAATGCCTGGCTAATT  Hsapiens
  353015: ....T.........................G...................  Ptroglodytes
  371072: ..............................G...................  Ggorilla
  402548: ..............................G......C............  Pabelli
  324082: ........................T.....GT..................  Nleucogenys
  399651: ..........................T...G....C........T...C.  Mmulatta
  352299: ..............................G....C..............  Panubis
  383442: ..........T................A..G....C........T.....  Cjacchus
 1032217: .G........T................A..G....C........T.....  Sboliviensis

                                     Alu
                         33328     33338     33342     33352     33362
   33321: TTTT--TTGGTGGGGGGCGGGG------GATGGGAGACAGCGTTTTTCTC  Hsapiens
  353065: ....--................------.............A.......G  Ptroglodytes
  371122: ....--............A..-------.....................G  Ggorilla
  402598: ....--................------..............C......G  Pabelli
  324132: ....--................------.....................G  Nleucogenys
  399701: ....--................------.....................G  Mmulatta
  352349: ....--................------.....................G  Panubis
  383492: ...G--....G.A..A..A...GACAGG..C.........T....CA.CA  Cjacchus
 1032267: ....TG....A.A..AA.AA..------............T....CA.CA  Sboliviensis
```

```
                                   Alu
          ---------------------------------------------------------->
                      33372     33382     33392     33402     33412
    33363: CTTGCCCAGGCTGGTCTCAAACTCCTGAGCTCAAGCAATTTGCCTGCCTT  Hsapiens
   353107: ..................................................  Ptroglodytes
   371163: .......................T..........................  Ggorilla
   402640: ..C.T.....................................T.......  Pabelli
   324174: ..............................................T...  Nleucogenys
   399743: ..........A.....................C.................  Mmulatta
   352391: ..........A.....................C........A........  Panubis
   383540: T....................T.T...G...................A..  Cjacchus
  1032311: T......T.............T.T...G........G..........A..  Sboliviensis

                                  Alu                       LINE1
          ------------------------------------------------->-->
                      33422     33432     33442     33452     33462
    33413: AACCTCCCAAAGTGCTGAGATTATAGGCATAAGCCACCATGCCTGGCCTC  Hsapiens
   353157: .............................................T....  Ptroglodytes
   371213: ..................................................  Ggorilla
   402690: ..................................................  Pabelli
   324224: ..................................................  Nleucogenys
   399793: .............................................A....  Mmulatta
   352441: ..................................................  Panubis
   383590: .G.....................C..A..C.......A........T...  Cjacchus
  1032361: .G.............C..........AA.........A..A.....T...  Sboliviensis

                                  LINE1
          ---------------------------------------------------------->
                      33472     33482     33492     33502     33512
    33463: TTATTGTTGAATTTTAAGAATTCTTTGTATATTTTAAATACCAGCTTTGT  Hsapiens
   353207: ........................................T..A......  Ptroglodytes
   371263: ..................................................  Ggorilla
   402740: ..................................................  Pabelli
   324274: ......................G...........................  Nleucogenys
   399843: .....................................-............  Mmulatta
   352491: .....................................-............  Panubis
   383640: ............................G.......GG...TG.....T.  Cjacchus
  1032411: ................................C...GG...T.....GTC  Sboliviensis

                                  LINE1
          ---------------------------------------------------------->
                      33520     33530                 33549     33559
    33513: TTTT--ATTAGACATATGTTTTGTAAATA-TTTTTTCCACTCTGTTGCTT  Hsapiens
   353257: ....--.......................-....................  Ptroglodytes
   371313: ....--............C..........-............C.......  Ggorilla
   402790: ....--.......................-....................  Pabelli
   324324: ....--........CG.............-...................C  Nleucogenys
   399892: ....--..C.......--------------...............G....  Mmulatta
   352540: ....--..C....................-........G......G....  Panubis
   383690: ....--..C....................-.............A.GA...  Cjacchus
  1032461: ....TA..C....................T...............C....  Sboliviensis
```

```
                                    LINE1
          ------------------------------------------------------>
                33569      33579      33588      33598      33608
   33560: GTGTTTTTATTCTCACGGTGTCTTTTG-AGAGCAGAAGTTTTTAATTTTA  Hsapiens
  353304: ...........................-......................  Ptroglodytes
  371360: ...............T...........-......................  Ggorilla
  402837: .............A..A..........-......................  Pabelli
  324371: A............A..A--........-......................  Nleucogenys
  399926: ..........C..----..........-......................  Mmulatta
  352587: ..........C..----..........-......................  Panubis
  383737: .............A..AA......CCAC...T.......C..........  Cjacchus
 1032511: .C...........A..CA...G.....C...........C..........  Sboliviensis

                                    LINE1
          ------------------------------------------------------>
                33618                  33631      33641      33651
   33609: ATTAAGTCCAAATTATCCA-------TTTTTTTATGACTCATACCTTTGG  Hsapiens
  353353: ...................-------...........A............  Ptroglodytes
  371409: ...................-------...........A............  Ggorilla
  402886: ...................-------...........A...........A  Pabelli
  324418: ...............C...-------...........A............  Nleucogenys
  399971: ...................-------......A...GA....G.......  Mmulatta
  352632: ...................-------......A...GA...C........  Panubis
  383787: ...G...............-----TT......C...GA.T..........  Cjacchus
 1032561: ..................GGGGTTTT.....CC.C.GA.T..........  Sboliviensis

                                    LINE1
          ------------------------------------------------------>
                33661      33671      33681      33691      33701
   33652: TATTGTATCTAAAAAGGCATTGCTAAACCAAAGGTCACCTAGATTTTCTC  Hsapiens
  353396: ..................................................  Ptroglodytes
  371452: ..................................................  Ggorilla
  402929: ..................................................  Pabelli
  324461: C...T.............................................  Nleucogenys
  400014: ..................................................  Mmulatta
  352675: .....................................T............  Panubis
  383832: .G.................C.....C............T...........  Cjacchus
 1032611: .G............T.A.................................  Sboliviensis

                                    LINE1
          ------------------------------------------------------>
                33711      33721      33731      33741      33751
   33702: CCATGTCATCTTCTAGAAGTTTTGTAGTTTTGCATGTTATACTTAGGTGA  Hsapiens
  353446: ..................................................  Ptroglodytes
  371502: ..................................................  Ggorilla
  402979: .............................................A....  Pabelli
  324511: ........................................G.........  Nleucogenys
  400064: ......T........................C..................  Mmulatta
  352725: ...............................T..................  Panubis
  383882: T..........................-...................C..  Cjacchus
 1032661: ..........---------------------------------.......  Sboliviensis
```

```
                                           LINE1
          ------------------------------------------------------------------>
                     33761     33771     33781     33791     33801
                         :         :         :         :         :
  33752: ATAATCAATTTTGAGATAATTTTTGTGAGTCATGTAAAATCAGATTCTAG  Hsapiens
 353496: .............................G....................  Ptroglodytes
 371552: .............................G....................  Ggorilla
 403029: ..G..........................G....................  Pabelli
 324561: ..G.....................C...TG....................  Nleucogenys
 400114: ..G...............T.........AG....................  Mmulatta
 352775: ..T...............T.........AG....................  Panubis
 383931: ..G..............G..........AG..........TG....A...  Cjacchus
1032678: ..G....................C....AG..........TG....A...  Sboliviensis

                                           LINE1
          ------------------------------------------------------------------>
                     33810     33820     33830     33840     33850
                         :         :         :         :         :
  33802: A-TTATTTTTGACATGTGGAGATCCAGCTTTTCTACCACCATTTGTTGAA  Hsapiens
 353546: .-.........................T......................  Ptroglodytes
 371602: .-.........................T......................  Ggorilla
 403079: .-..................T......T.G....................  Pabelli
 324611: .-.....CA..G........T......T.G........TG..........  Nleucogenys
 400164: .-............C.....T......T.G....................  Mmulatta
 352825: .-..................T......T.G....................  Panubis
 383981: .-..T...............T......T.G....................  Cjacchus
1032728: .T..T...............T......T.G....................  Sboliviensis

                                           LINE1
          ------------------------------------------------------------------>
                     33860     33870     33880     33890     33900
                         :         :         :         :         :
  33851: AAGATTATTCTTTCTCCATTGAATTGGCTTCGCACCTTTACCAAAGACCA  Hsapiens
 353595: ..............................T...................  Ptroglodytes
 371651: ..............................T...................  Ggorilla
 403128: ...G...........T...........T..T...................  Pabelli
 324660: ....G.........................T...................  Nleucogenys
 400213: ..........................A...T....G..............  Mmulatta
 352874: ..................A...........T...................  Panubis
 384030: ..........C..T................T...................  Cjacchus
1032778: ...T......C...G...............TA..................  Sboliviensis

                                           LINE1
          ------------------------------------------------------------------>
                     33910     33920     33930     33940     33950
                         :         :         :         :         :
  33901: ATTGATTGTATTTGTGTGGGTCTCTTTCTGGGTTTCTTATTCTGTTCCAC  Hsapiens
 353645: ..................................C...............  Ptroglodytes
 371701: ..................................C...............  Ggorilla
 403178: .......T..C.......................CT..............  Pabelli
 324710: .......T..........................CT..............  Nleucogenys
 400263: .......T..-.....................C.CT..............  Mmulatta
 352924: .......T........................C.CT..............  Panubis
 384080: G......T.......T................C.CT..C...-.......  Cjacchus
1032828: G......T.......T................CACT..C...-A......  Sboliviensis
```

```
                                LINE1
          ------------------------------------------------->
                   33960     33970     33980     33990     34000
                       :         :         :         :         :
   33951: TGACCTATTTGTTATTCTTTCAACAATATGACACTGTCTTGATTATTGTG  Hsapiens
  353695: .............................................C....  Ptroglodytes
  371751: .............................................C....  Ggorilla
  403228: .............................................C....  Pabelli
  324760: .............................................C....  Nleucogenys
  400312: ...T..........................T..............C....  Mmulatta
  352974: ...T..........................T..............C....  Panubis
  384129: ................T.A..................T.......C....  Cjacchus
 1032877: ....T.....T.....T........G.............C.....C....  Sboliviensis

                                LINE1
          ------------------------------------------------->
                   34010     34020     34026     34036     34046
                       :         :         :         :         :
   34001: GCTTTATGTAAGTCTTGAAATC----AGGCGGTAGCCTTCCTCTGGCTTT  Hsapiens
  353745: ......................----...T.......A.......A....  Ptroglodytes
  371801: ......................----...T.......A.......A....  Ggorilla
  403278: ......................----...T.......A......AA.G..  Pabelli
  324810: .T....................AGGT...T.......A.......A....  Nleucogenys
  400362: ......................----.T.T.....A.A.......A....  Mmulatta
  353024: ......................----...T.....A.A.......A....  Panubis
  384179: A..............G......----...AA...AG.A.......A....  Cjacchus
 1032927: .......A.......G......----...AA......AC......A....  Sboliviensis

                           LINE1
          ----------------------------------------->
                   34056     34066     34076     34086     34096
                       :         :         :         :         :
   34047: ATTCTTCTTCTTCAATATTGTGCGGGCTATTCTGAATCTTTCCCGGCTTC  Hsapiens
  353791: .......................A....................A.....  Ptroglodytes
  371847: ............................................A.....  Ggorilla
  403324: ............................................A.....  Pabelli
  324860: ............................................A.....  Nleucogenys
  400408: C.....................T........TG.........T.A.....  Mmulatta
  353070: C....................AT...................T.A.....  Panubis
  384225: .......C.....T........T.....................AA...G  Cjacchus
 1032973: ......................T..............T......AA....  Sboliviensis

                   34106     34116     34126     34136     34146
                       :         :         :         :         :
   34097: CTTTTAACTCAATAACGCAAGTGAGGATATTTAGAAGTCTCTTTTCTCAG  Hsapiens
  353841: ..................................................  Ptroglodytes
  371897: ..................................G...............  Ggorilla
  403374: TC..............A.................G.A......C......  Pabelli
  324910: ................A............C....G...............  Nleucogenys
  400458: .C.........G....A.................G...............  Mmulatta
  353120: .C..............A.................G...............  Panubis
  384275: ...............TA...C.............................  Cjacchus
 1033023: ...............T....C..............T..G......A....  Sboliviensis
```

```
                   34156     34166     34176     34186     34196
   34147: TCCTCCACTTCTCTTGTCATGAACTCAGGCCAGTGGCATCTATACACATG   Hsapiens
  353891: ..................................................   Ptroglodytes
  371947: ......G...........................................   Ggorilla
  403424: ............................T.....................   Pabelli
  324960: C........................................G........   Nleucogenys
  400508: ..........................................G.......   Mmulatta
  353170: ..........................................G.......   Panubis
  384325: .........C.......................CA...........--..   Cjacchus
 1033073: .........C.............................G.......--.   Sboliviensis
```

```
                   34206     34215     34225     34235     34245
   34197: AGTGATTTCTTAATTTT-TCCCCATTTCTATCATTTTTCTTCCATATTTT   Hsapiens
  353941: .................-................................   Ptroglodytes
  371997: .................-...........G....................   Ggorilla
  403474: ..C..............-.A.......G......................   Pabelli
  325010: .................T................................   Nleucogenys
  400558: ....C............-....T..................T........   Mmulatta
  353220: .................-.......................T........   Panubis
  384373: .........C.-.....-....G....A.............A........   Cjacchus
 1033121: .........C.......--....G.................A........   Sboliviensis
```

```
                                                      DNA
                                                   -------->
                   34255     34265     34275     34285     34295
   34246: AAAAGATTGTATCCACAAAACTGCCTTTGTTTCACAGATTTGCTTCATCA   Hsapiens
  353990: ..................................................   Ptroglodytes
  372046: ...........C......................................   Ggorilla
  403523: ........T..................A......................   Pabelli
  325060: ........TC........................................   Nleucogenys
  400607: ....T...T.........................................   Mmulatta
  353269: ....T...T...T.....................................   Panubis
  384421: G.......T.GC..................................T...   Cjacchus
 1033169: GT.....GT.....................................T...   Sboliviensis
```

```
                                  DNA
          ------------------------------------------------->
                   34305     34315     34325     34335     34345
   34296: AAGATATCTATAACTTCTGGTCAGAGATTCCAATCAGCATGAGATATCAT   Hsapiens
  354040: ..................................................   Ptroglodytes
  372096: ..................................................   Ggorilla
  403573: ......................................G..........C   Pabelli
  325110: ...................................G..............   Nleucogenys
  400657: ..................................................   Mmulatta
  353319: ..................................................   Panubis
  384471: .GC...........................................CT..   Cjacchus
 1033219: .GC...........................................AT..   Sboliviensis
```

```
          DNA
          ------>
               34355     34365     34374     34384     34394
   34346: TGTTACTGACGATTTTGATATGTCTGCC-ACAAAAGTAAACAGCATTATG  Hsapiens
  354090: ............................-...G.................  Ptroglodytes
  372146: ............................-.....................  Ggorilla
  403623: ............................-.....................  Pabelli
  325160: ............................-..G.........G.....G..  Nleucogenys
  400707: .......A..A.................-................G....  Mmulatta
  353369: .......A..A.................-................G....  Panubis
  384521: .C........A..C..............T.A.....C........G....  Cjacchus
 1033269: ..........A.................A.......C.............  Sboliviensis

               34404     34414     34424     34434     34444
   34395: CAACCTAATGCCAAGTCTAACGTGATGCCAAATTAAGTTTAAGTGAGGCA  Hsapiens
  354139: ..................................................  Ptroglodytes
  372195: ....G.............................................  Ggorilla
  403672: ..................................................  Pabelli
  325209: ....................TA.....A......................  Nleucogenys
  400756: ...........................................C......  Mmulatta
  353418: ...........................................C......  Panubis
  384571: .G..................T........T..C.....C...........  Cjacchus
 1033319: ....-.................................C...........  Sboliviensis

               34454     34464     34474     34484     34494
   34445: CCAAAATAAATTAGGTGCTGGCTTCCGAATGAGACAGTGGACTATATGTA  Hsapiens
  354189: ...............C....................T.A...........  Ptroglodytes
  372245: .........................T..........T.A...........  Ggorilla
  403722: .....................T....A.........T.A...........  Pabelli
  325259: ..........................A.........TCA...........  Nleucogenys
  400806: T............----------------.....T.T.A...........  Mmulatta
  353468: T............----------------.....T.T.A...........  Panubis
  384621: ....................C.....A......GT.T.A...C.C.....  Cjacchus
 1033368: ....................C.....A......GT.T.A.....C.....  Sboliviensis

               34504     34514     34524     34534     34544
   34495: GGTAATCCAACAAAACTTGGTATGAGACTCCTTAAAACTTGTGCAGGTCA  Hsapiens
  354239: ..................................................  Ptroglodytes
  372295: ..........G.......A...........T...................  Ggorilla
  403772: ..............................T...................  Pabelli
  325309: ..C...........................T...................  Nleucogenys
  400840: .A.G...G..T......G....G.......T...................  Mmulatta
  353502: .A.G..TG..T......G....G.......T...................  Panubis
  384671: ......G...A....                                     Cjacchus
  385987:                        ..T..AT...TT.GA............  Cjacchus
 1033418: ......G...A....                                     Sboliviensis
 1034730:                        ..T..AT...TT.GA............  Sboliviensis

               34554     34564     34574     34584     34594
   34545: TAAGGCAAAACATACTTCAATAAACTCTTACCTTGTCCCTCCCCATTTGC  Hsapiens
  354289: ...........G................................G.....  Ptroglodytes
  372345: ...................T........................G.....  Ggorilla
  403822: .....................T............................  Pabelli
  325359: ............................................G.....  Nleucogenys
  400890: ...........................................TG.....  Mmulatta
  353552: ...........................................TG.....  Panubis
  386014: .............G.....G--------..................C...  Cjacchus
 1034757: .............G.........G......................C...  Sboliviensis
```

```
                 34604     34614     34624     34634     34644
   34595: CCATAGGCCATCTTGGGAGGTGACCCACTTTGTGCCCAGCTGCCACCAGC  Hsapiens
  354339: ..................................................  Ptroglodytes
  372395: ............................A.....................  Ggorilla
  403872: ...........................T......................  Pabelli
  325409: ........T........................................T  Nleucogenys
  400940: ....................................T.............  Mmulatta
  353602: ....................................T.............  Panubis
  386056: ..............................G...................  Cjacchus
 1034807: .........C....................G...................  Sboliviensis

                 34654     34664     34674     34684     34694
   34645: TGGAAACATTTTTGGAATGAAGAATGACCTGTCCAAGGACTAGGGACCAG  Hsapiens
  354389: ...............................C.......G..........  Ptroglodytes
  372445: ...............................C.......G..........  Ggorilla
  403922: ........C...................G..C.......G..........  Pabelli
  325459: ...............................C.......G..........  Nleucogenys
  400990: ...............................CT......GG.........  Mmulatta
  353652: ...............................C.......GG.........  Panubis
  386106: ............................T..C.....A.GG.........  Cjacchus
 1034857: .......G..C..........A.......C.C.....A.GG.AA......  Sboliviensis

                 34704     34714     34724     34734     34744
   34695: GTGGGCCAGGTAGGCAGGTGGGAAGGGGTGCGACCACCTGGGTCAGTTTC  Hsapiens
  354439: ........A.........................................  Ptroglodytes
  372495: .......................................T..........  Ggorilla
  403972: ..................................................  Pabelli
  325509: .......G..........................................  Nleucogenys
  401040: ................A.................................  Mmulatta
  353702: ................A............................A....  Panubis
  386156: ...T...........................T..................  Cjacchus
 1034907: ...T.....A.....................T..T.TG............  Sboliviensis

                 34754     34764     34774     34783     34793
   34745: TCTCTCAGCCCCAACGACATTCAGATCTTTTC-TCCTCCTGGAGGGTGTG  Hsapiens
  354489: ...............A................-.................  Ptroglodytes
  372545: ...............AG...............-..T..............  Ggorilla
  404022: ...............A................-.......C.........  Pabelli
  325559: .........T.....A................-.................  Nleucogenys
  401090: .....A.........A...C......T.....-.................  Mmulatta
  353752: .....A.........A...C......T.....-.................  Panubis
  386206: ...............-...C...........T-...........AT....  Cjacchus
 1034957: ....--.........A................T...........AT....  Sboliviensis

                 34803     34813     34823     34833     34843
   34794: GTTGCCTCTGGTAAGTCACAGGTTCATAGACTGAGATTCTGCCACATAAT  Hsapiens
  354538: .C................................................  Ptroglodytes
  372594: .C................................................  Ggorilla
  404071: .C..T.........T..G.C..............................  Pabelli
  325608: .C..T..............CA.............................  Nleucogenys
  401139: .C..T.....C........T.A.....G.................G....  Mmulatta
  353801: .C..T.....C........T.A.....G.................G....  Panubis
  386254: .C..T..T...........T.......G..............TG...C..  Cjacchus
 1035005: .C..T.........A....T.......G..............TG...C..  Sboliviensis
```

```
              34853     34863     34873     34883     34893
                  :         :         :         :         :
  34844: GTGGTTCCTGAGGAAGCAGGCACTGGGGATGGGGAGCAGAGAGGAGAACT   Hsapiens
 354588: ..................................................   Ptroglodytes
 372644: ..................................................   Ggorilla
 404121: ...........T......................................   Pabelli
 325658: ..................................................   Nleucogenys
 401189: .....G...................T...C....................   Mmulatta
 353851: .....G...................C...C....................   Panubis
 386304: A....G.......................C.....A............G.   Cjacchus
1035055: A....G.......................C..A..........A..---.   Sboliviensis

              34903     34913     34923     34933     34943
                  :         :         :         :         :
  34894: GAGAGTGGAGGGAACACCAGAGTGTGTCTTAGCAGCTCACAGTGCTATTC   Hsapiens
 354638: ..................................................   Ptroglodytes
 372694: ..................................................   Ggorilla
 404171: ..........A.....................TG................   Pabelli
 325708: .................................G................   Nleucogenys
 401239: ..................G......A.......G.............C..   Mmulatta
 353901: .........................A.......G.............C..   Panubis
 386354: ...................CG...........TG.....A...C..G...   Cjacchus
1035102: A...................G...........TG.T....C.....G.G.   Sboliviensis

              34953     34963     34973     34983     34993
                  :         :         :         :         :
  34944: CTTCCAAGTGCTGACCACTGGCCACACAAAACCAGTGGTAGCTTCAAAAC   Hsapiens
 354688: ..................................................   Ptroglodytes
 372744: ..................................................   Ggorilla
 404221: ..................................................   Pabelli
 325758: ........A.........................................   Nleucogenys
 401289: ..........................G.......................   Mmulatta
 353951: ..................................................   Panubis
 386404: ......T.................T.........................   Cjacchus
1035152: ..............G.........T..........CA.............   Sboliviensis

              35003     35013     35023     35033     35043
                  :         :         :         :         :
  34994: CATACAAAACTAGGTGGAAGGCAGGACTTGGCCGTCAGGCTGCAGTGTGC   Hsapiens
 354738: .................................C................   Ptroglodytes
 372794: .................................C................   Ggorilla
 404271: .................................C...........G....   Pabelli
 325808: T..T.............................C................   Nleucogenys
 401339: .................................C................   Mmulatta
 354001: .................................C................   Panubis
 386454: ..C..........................A.A.C...C..G.........   Cjacchus
1035202: ......G.......CA...........C.C...-................   Sboliviensis

              35053     35063     35073     35083     35093
                  :         :         :         :         :
  35044: TGGCCACAGCCGTCAAATAACAGCTGATCTAGATCCACCCTAGCAAAGCC   Hsapiens
 354788: ...........C..G...................................   Ptroglodytes
 372844: ...........C..G...................................   Ggorilla
 404321: ......TG...C..G..........A........................   Pabelli
 325858: .......C...C.........-...A...........T............   Nleucogenys
 401389: .......G...C........G....A...........T.G..........   Mmulatta
 354051: .......G...C..G.....G....A.............G..........   Panubis
 386504: ..A...TG.G.C..C.G...G....AC.....G.....A...........   Cjacchus
1035251: ..AG...G...C..C.G...G....AC...........G...........   Sboliviensis
```

```
              35102     35112     35122     35132     35141
                  :         :         :         :         :
   35094: CCAGAA-CCATGCTTCCATGGGATCAGGCTCTGTCGCCAGTAAAATA-TT  Hsapiens
  354838: ......-............................A...........-..  Ptroglodytes
  372894: ......-............................T...........-..  Ggorilla
  404371: ......-...........................T............-..  Pabelli
  325907: ......-........................................-..  Nleucogenys
  401439: ......-........................................-..  Mmulatta
  354101: ......-............A...........................-..  Panubis
  386554: ......-...........CA...........A........C......C..  Cjacchus
 1035301: ......C...........CA...........................T..  Sboliviensis

              35151     35161     35171     35181     35190
                  :         :         :         :         :
   35142: TTTAAAAAGAAAAATGTACTTAAGTTGGTGCAAAAGTAATCTTGGT-TTT  Hsapiens
  354886: ..............................................-...  Ptroglodytes
  372942: ...................................A..........-...  Ggorilla
  404419: ..............C.......................G...C...-...  Pabelli
  325955: ..................T.......................C...-...  Nleucogenys
  401487: ...............A..T...GT................TGC...-...  Mmulatta
  354149: ...T...........A..T...GT................TGCA..-...  Panubis
  386603: ...............ACTT...G..................GCA..G...  Cjacchus
 1035351: ...............CCTT...GT..............G..GCA.--...  Sboliviensis

                                                     LINE1
                                                 --------->
              35200     35210     35219     35229     35239
                  :         :         :         :         :
   35191: TGACTTTAGCAAAAACATACGTACAT-ACACACACATGAATATATTTAAG  Hsapiens
  354935: ..........................-......................A  Ptroglodytes
  372991: ...................T......-......................A  Ggorilla
  404468: ...................T......-....................C..  Pabelli
  326004: ...............T...T......-......................A  Nleucogenys
  401536: ..C................T......-......................A  Mmulatta
  354198: ..C...............GT......-......................A  Panubis
  386653: ..C.G...........G..T......G.T....................A  Cjacchus
 1035399: ..C................T......A.T.......C............A  Sboliviensis

                                 LINE1
          ------------------------------------------------->
              35249     35259     35269     35279     35289
                  :         :         :         :         :
   35240: GAAATAATGACTGAATGCTCTATAAATCTGATGAAAAATATTAACCAATA  Hsapiens
  354984: .................................................T  Ptroglodytes
  373040: ......G...........................................  Ggorilla
  404517: ...............CA.................................  Pabelli
  326053: ...............CA.................................  Nleucogenys
  401585: ...............CA.....-.....CA..A..............G..  Mmulatta
  354247: ...........C...CA.....-......A..A..............G..  Panubis
  386703: ...............CA.....C..........G...........T....  Cjacchus
 1035449: ...............C-------..........G...........T....  Sboliviensis
```

```
                  LINE1
         ---------------------------->
              35299     35309     35319     35329     35339
                  :         :         :         :         :
  35290: ATGCCAAGAAATTCAAAAAACCTCAAGCCAGAGGAAGAGATCCTGCCAAA  Hsapiens
 355034: ..................................................  Ptroglodytes
 373090: ............................A.....................  Ggorilla
 404567: ............................A..................C..  Pabelli
 326103: ............................A.....................  Nleucogenys
 401634: .............A...T..T.......A...............TA....  Mmulatta
 354296: .............A......T.......A................A....  Panubis
 386753: ...........G....-T...A....--........-...C....A.-..  Cjacchus
1035492: ...........G...GT...........A...........C.........  Sboliviensis

              35349     35359     35369     35379     35389
                  :         :         :         :         :
  35340: AAGAGAAGGGAACTTAATCATTACGGTTGTGGGGAATGTTACTGGGGCCA  Hsapiens
 355084: ..................................................  Ptroglodytes
 373140: ..................................................  Ggorilla
 404617: ...GA..................T..........................  Pabelli
 326153: ...G....................A.........................  Nleucogenys
 401684: ...G........T..........T....A.....................  Mmulatta
 354346: ...G........T..........T....A.....................  Panubis
 386798: ...GT......G...................A.-TG..............  Cjacchus
1035542: ...G...................G.........ATG..............  Sboliviensis

              35399     35409               35412     35422
                  :         :                   :         :
  35390: GCAGCTCACCAGCAGGAAGA-----------------GAGCAGAGAGGCC  Hsapiens
 355134: ....................-----------------.............  Ptroglodytes
 373190: ....................-----------------.............  Ggorilla
 404667: ....................-----------------.............  Pabelli
 326203: ....................-----------------.............  Nleucogenys
 401734: ....................GCACTGCCCATGGGAGG........G....  Mmulatta
 354396: ....................GCACTGCCCATGGGAGG........G....  Panubis
 386847: .T..................G                              Cjacchus
1035592: ....................GCACTGCCTATGGGAGG..C....T.A...  Sboliviensis

              35432     35442     35452     35462     35472
                  :         :         :         :         :
  35423: CTCTGTCCAGGGAACCCACAACAGCTCCTGCAGGCAAAATGTCAGGTCCA  Hsapiens
 355167: ..................................................  Ptroglodytes
 373223: .............................A....................  Ggorilla
 404700: .............................A....................  Pabelli
 326236: .............................A....................  Nleucogenys
 401784: .............................A....................  Mmulatta
 354446: .............................A....................  Panubis
1035642: .....C.......................A..CA................  Sboliviensis

              35482     35492     35502     35512     35522
                  :         :         :         :         :
  35473: GAGCATAACACCAGCTTGTAAATCTGCAGGACTGTCTCATTCCCTTGTCA  Hsapiens
 355217: ..................................................  Ptroglodytes
 373273: ..................C...............C...............  Ggorilla
 404750: ......G...........................................  Pabelli
 326286: ......G..........................A................  Nleucogenys
 401834: ......G...........................................  Mmulatta
 354496: ......G...........................................  Panubis
1035692: ..A...G..........C..G............A.TC........G....  Sboliviensis
```

```
                      35532     35542     35552     35562     35572
                          :         :         :         :         :
  35523: ATTTCTCCCTTACTGCTTATCCTTAGGAGTCAGAAATAGCAGGTGGGTGA  Hsapiens
 355267: ..................................................  Ptroglodytes
 373323: G................C................................  Ggorilla
 404800: ..................C...........................C...  Pabelli
 326336: ..................C...............................  Nleucogenys
 401884: ..................C...............................  Mmulatta
 354546: ..................C...............................  Panubis
1035742: .C................T...........------G........A...-  Sboliviensis
```

```
                                   LTR
          ------------------------------------------------>
                      35582     35592     35602     35612     35622
                          :         :         :         :         :
  35573: GGGAACAACCACCCCTCTCTTCTAGATCCCAGGCTTCTCCCTGCACCTGT  Hsapiens
 355317: ..................................................  Ptroglodytes
 373373: ................G................A................  Ggorilla
 404850: ..A..C............................................  Pabelli
 326386: A.................................................  Nleucogenys
 401934: .....G.G..............C...........................  Mmulatta
 354596: .......G..............C...........................  Panubis
1035785: -....G...................................G.......C  Sboliviensis
```

```
                                   LTR
         ------------------------------------------------->
                      35632     35642     35652     35662     35672
                          :         :         :         :         :
  35623: TGCAGTTGGTGGGAGGGAAGAAGATACACTATTAAATTAGGCTTGCAGTT  Hsapiens
 355367: ..................................................  Ptroglodytes
 373423: ..................................................  Ggorilla
 404900: .....C............................................  Pabelli
 326436: .....C..........A..A.........C....................  Nleucogenys
 401984: .....C.......C...............................TG...  Mmulatta
 354646: .....C.......C...............................TG...  Panubis
1035834: CC...C...G..............C.T.....A............T....  Sboliviensis
```

```
          LTR                          DNA
         ----><------------------------------------------->
                      35682     35692     35702     35712     35722
                          :         :         :         :         :
  35673: AAGATCAGTGCTTCTCAAACTGTCGTGTGCACACAAATCAACTGGGGAGC  Hsapiens
 355417: ........................A.........................  Ptroglodytes
 373473: ........................A......G..................  Ggorilla
 404950: .G......................A.........................  Pabelli
 326486: .G......................A.........................  Nleucogenys
 402034: .G........TG............A.........................  Mmulatta
 354696: .G......................A.........................  Panubis
1035884: .G...................A..A..........G....G.........  Sboliviensis
```

```
                                   DNA
         ------------------------------------------------->
                      35732     35742     35752     35762     35772
                          :         :         :         :         :
  35723: TTGTTAAACTGCAGGCTCTGATTCCTTCTGTGGATTTGGGTTGGAGCCAG  Hsapiens
 355467: .................................G................  Ptroglodytes
 373523: ...........A.....................G................  Ggorilla
 405000: .................................G................  Pabelli
 326536: ................................AG................  Nleucogenys
 402084: .......................A.........G................  Mmulatta
 354746: ............................C....G........          Panubis
1035934: ............................C...AG...............A  Sboliviensis
```

```
                                    DNA
         -------------------------------------------------->
                   35782     35792     35802     35810     35820
                       :         :         :         :         :
  35773: AGATTTTGCCTTTCCATTTCTAACAAGTTCCC--ATGAGACCAGCATTAT  Hsapiens
 355517: ................................--................  Ptroglodytes
 373573: ................................--................  Ggorilla
 405050: ...........................C....--..........T...G.  Pabelli
 326586: ...........................C....--................  Nleucogenys
 402134: ...C.......................C....--..........T...G.  Mmulatta
1035984: ...C.....T....T.....A......C....AT..........T...T.  Sboliviensis

                    DNA                       LTR
         ------------------------------>------------------->
                   35830     35840     35850     35860     35870
                       :         :         :         :         :
  35821: GGTCCCCAGACCACCCGGGGAATGGTAAGGGTTTAGATTACTATCTGGGC  Hsapiens
 355565: ..................................................  Ptroglodytes
 373621: ................A.................................  Ggorilla
 405098: ...................................A..............  Pabelli
 326634: ..................................................  Nleucogenys
 402182: .................A................................  Mmulatta
1036034: .....T.........T.ATA...T......A...G......C....A...  Sboliviensis

                                    LTR
         -------------------------------------------------->
                   35880     35890     35900     35910     35920
                       :         :         :         :         :
  35871: CCAAATGTTTTAATTCCTGAATCTCATTATGTTTGTGATATGGAACAATC  Hsapiens
 355615: ......C.......C...................................  Ptroglodytes
 373671: ......C...........................................  Ggorilla
 405148: ......C.................G..............G..........  Pabelli
 326684: ......C................................G..........  Nleucogenys
 402232: ......C.................G.....C........G..........  Mmulatta
1036084: TT....C............T....G........A...G.G.....T....  Sboliviensis

                                    LTR
         -------------------------------------------------->
                   35930     35940     35950     35960     35969
                       :         :         :         :         :
  35921: ACAGATTTTCTAGATTTTAGGTGTAACCAGAAAGCCATGAGGACTGCC-A  Hsapiens
 355665: ................................................-.  Ptroglodytes
 373721: ................................................-.  Ggorilla
 405198: ....................T.-.........................-.  Pabelli
 326734: ...........................T....................-.  Nleucogenys
 402282: ................................................A.  Mmulatta
1036134: ......................A..................A.....T-G  Sboliviensis

                                    LTR
         -------------------------------------------------->
                   35979     35989     35999     36009     36019
                       :         :         :         :         :
  35970: GAGTTTTCATGCTGGGGCTAAGGATGGGCCTCCCTCTAAGCAGCTTTATA  Hsapiens
 355714: ....................G.......T.....................  Ptroglodytes
 373770: ....................G.......T.....................  Ggorilla
 405246: ...............C....G.......T.....................  Pabelli
 326783: ....................G..T....T.....................  Nleucogenys
 402332: ....................G......AT................C..C.  Mmulatta
1036183: ..........T.........G......AT..TT.................  Sboliviensis
```

```
                                       LTR
         ---------------------------------------------------->
                36029     36039     36049     36059     36069
  36020: GGGACAATAGGAGGGGAATGCAAAGTTGCTTTTGTGGCTTATTTCCATGA  Hsapiens
 355764: ..................................................  Ptroglodytes
 373820: ..................................................  Ggorilla
 405296: .......................G..........................  Pabelli
 326833: .............A.........G.........................G  Nleucogenys
 402382: ...............A...A...G.C........................  Mmulatta
1036233: ...............A...A...G.C...........G..-.......A.  Sboliviensis

              LTR
         ------------>
                36079     36089     36099     36109     36119
  36070: GTCTTGCTGGTTTGTTACATTGTTTATACTGGCAGAGGCAGAGCTTCTGT  Hsapiens
 355814: ..................................................  Ptroglodytes
 373870: ........T.........................................  Ggorilla
 405346: ...........................................T......  Pabelli
 326883: .C.........................................T......  Nleucogenys
 402432: ...........................................T......  Mmulatta
1036282: .................T........C.............C..T......  Sboliviensis

                36129     36139               36149     36159
  36120: TAGCTCTTTGAAGGGTGAAA----------ATTCACCTCCCTGCCCCCTT  Hsapiens
 355864: ....................----------....................  Ptroglodytes
 373920: ....................----------....................  Ggorilla
 405396: ....................----------.........T......T...  Pabelli
 326933: ....................----------............A.......  Nleucogenys
 402482: .....G..............----------...................C  Mmulatta
1036332: ........CA....A.....AATAGCTTGC..........TC........  Sboliviensis

                                          LTR
               ---------------------------------------------->
                36169     36178     36188     36198     36208
  36160: GGTGCCTGTGATGGTT-AATTCCATGTGTCAACTTCGCTAGGTCCACAGG  Hsapiens
 355904: ................-....G.............G..............  Ptroglodytes
 373960: ....A...........-..................G..............  Ggorilla
 405436: ................-..................G..............  Pabelli
 326973: ................-..................G.....A........  Nleucogenys
 402522: .......C........-..................G......C.......  Mmulatta
 389290:                                            ...T...  Cjacchus
1036382: ...............CA......G...........G......C...T..A  Sboliviensis

                                       LTR
         ---------------------------------------------------->
                36218     36227     36237     36246     36256
  36209: TATTTGGTCAAACACCA-GTCTGGATGTTGCTGTG-AAGGCAATTTTTGA  Hsapiens
 355953: .................-.................-...........A..  Ptroglodytes
 374009: .................-.................-...........A..  Ggorilla
 405485: ......A..........-...C.............-.......C...A..  Pabelli
 327022: .................-...............CA-...........A..  Nleucogenys
 402571: .................-....C............-...........A..  Mmulatta
 389297: ..-...........T..G..GG.A..A........A...........A..  Cjacchus
1036432: ............TC...-..G..............-...........A..  Sboliviensis
```

```
                                  LTR
         ------------------------------------------------------->
               36266     36276     36286     36296     36305
                   :         :         :         :        :
  36257: TAAAATTAACATTTAAATCAGTACACTTTGAGTAAAGCAGATTACC-TCC  Hsapiens
 356001: .......................T......................C...  Ptroglodytes
 374057: .......................G......................C...  Ggorilla
 405533: ...................C..........................C...  Pabelli
 327070: ..............................................C...  Nleucogenys
 402619: .............................................TC...  Mmulatta
 389346: .G.............C.......G.....A.....GC.G.......C...  Cjacchus
1036480: .G.....................G.....A.....G..CA......C...  Sboliviensis
```

```
                                  LTR
         ------------------------------------------------------->
               36315     36325     36335     36344     36354
                   :         :         :         :        :
  36306: ATAGTGTGGGTAGACCTCATGCAATCAGTTGAAGG-CCTTAAGAGAAGAT  Hsapiens
 356051: ...................................-..............  Ptroglodytes
 374107: ...................................-..............  Ggorilla
 405583: ...................................-..............  Pabelli
 327120: ....................TT.............-..............  Nleucogenys
 402669: ......................C.......A..A.-..............  Mmulatta
 389396: .C.........GAC.T............CA...A.C............CC  Cjacchus
1036530: ...T........................C......-.T..........CC  Sboliviensis
```

```
                                  LTR
         ------------------------------------------------------->
               36364     36374     36384     36394     36404
                   :         :         :         :        :
  36355: AGATTAAGGTCTCTTGAGGAAGAGGGCACTCTGCCTCCAGACTGCCTTCA  Hsapiens
 356100: ..................................................  Ptroglodytes
 374156: ..................................................  Ggorilla
 405632: ..........G......A...........................T....  Pabelli
 327169: .............A....................................  Nleucogenys
 402718: ....C........A....................................  Mmulatta
 389446: .............A...A................................  Cjacchus
1036579: .............A....................................  Sboliviensis
```

```
                                  LTR
         ------------------------------------------------------->
               36414     36424     36434     36444     36454
                   :         :         :         :        :
  36405: GACTCAAGCTGCAGGATCAATTCTTCCCTGGGTATCAAGACTGCCCTGCA  Hsapiens
 356150: ..................................................  Ptroglodytes
 374206: ..................................................  Ggorilla
 405682: .......................................C..........  Pabelli
 327219: .......................................C..........  Nleucogenys
 402768: ..............T..................C...G.CT.........  Mmulatta
 389496: ..................C.C.........CA.......C..........  Cjacchus
1036629: ..................C.C....T....CA.......C..........  Sboliviensis
```

```
                                  LTR
         ------------------------------------------------------->
               36464     36474     36484     36494     36504
                   :         :         :         :        :
  36455: GATTTTGCACTTGCCAACCTCCACAATTGCATGAGCCATTTCCTTTAATT  Hsapiens
 356200: ..................................................  Ptroglodytes
 374256: ..........................C.......................  Ggorilla
 405732: ...................................T..............  Pabelli
 327269: ..................................................  Nleucogenys
 402818: ......................................A...........  Mmulatta
 389546: .....C...T.............T....A.........C.........A.  Cjacchus
1036679: .........T.............T..C...........C.........A.  Sboliviensis
```

```
                      LTR
                 36514     36524                           36530
   36505: AAACTCTCTCTCTCTCTCTCT------------------------GCCTC  Hsapiens
  356250: .............G...G...----------------------------.  Ptroglodytes
  374306: .....................------------------------.T...  Ggorilla
  405782: .....................------------------------.T...  Pabelli
  327319: .....................CTCTCTCTCTCTCTCTCTCTGTCT.T...  Nleucogenys
  402868: ...............T.....--------------------------...  Mmulatta
  389596: .G...T......C.......C-----------------------------  Cjacchus
 1036729: .....T......C.......C-----------------------------  Sboliviensis
```

```
                                               Simple
                 36540     36550     36560                 36573
   36531: TCCCCACCCCTAATATATGGAGATAGATAGATAGATAGA-------TAGA  Hsapiens
  356272: .......................G...G...G...G...-----------  Ptroglodytes
  374332: .......................................-------..T.  Ggorilla
  405808: ................G......G.T.............TGATAGA....  Pabelli
  327369: .......................G.T.............-------....  Nleucogenys
  402892: C...T....G.............G.C.............-------....  Mmulatta
  389617: ---.......C............G.T.......A.C...-------....  Cjacchus
 1036750: ---.......C............G.C.......A.C.A.-------....  Sboliviensis
```

```
                                Simple                        Simple
                 36583     36593     36600     36610       36618
   36574: TAGATAGATAGATAGATAGAGAGA---TATATGAAGATATATAGAT--AT  Hsapiens
  356311: ---------------------------G.G................--..  Ptroglodytes
  374375: G..------------------------....G..............--..  Ggorilla
  405858: ....................T...---..G..A.............--..  Pabelli
  327412: G...........G...G.......---G..................--..  Nleucogenys
  402935: ..........-----------------G...GAT.....G..T...TG..  Mmulatta
  389657: ......T.G..----------------..C..AT.T.......T..--..  Cjacchus
 1036790: ....G...............T.T.GAT....CAC.C.C.C.C.C.C--.C  Sboliviensis
```

```
                                 Simple
                 36627     36637
   36619: AGAT-TATATAAAGATATATATAT--------------------------  Hsapiens
  356332: ....-...................--------------------------  Ptroglodytes
  374400: ....-...................--------------------------  Ggorilla
  405903: ....-...................--------------------------  Pabelli
  327457: ....-..........G........--------------------------  Nleucogenys
  402968: T...-............G......--------------------------  Mmulatta
  389689: ...--G.G.G.G...G.G.G.G.G--------------------------  Cjacchus
 1036838: .C.CAC.C.C.C.C..........AGAGAGAGAGAGAGGGAGAGAGAAAG  Sboliviensis
```

```
                     Simple                                  LTR
                           36647     36657     36667     36677
   36642: --------------ATGTTTTATAATTGACAATGGTGAACATACATATAG  Hsapiens
  356355: ----------------..................................  Ptroglodytes
  374423: ----------------..................................  Ggorilla
  405926: ----------------...............G...........T......  Pabelli
  327480: ------------AT.................G..........--......  Nleucogenys
  402991: ------------GT.................G...........T......  Mmulatta
  389711: ----------------A.....C........G.A...GC...GT......  Cjacchus
 1036888: AGAGAGAGAGAGAG......C..........G.A...GC....T......  Sboliviensis
```

```
                                   LTR
         ------------------------------------------------->
                 36687     36697     36707     36717
                     :         :         :         :
  36678: TGGGAACTTTAAGAAAAAGTCTGACATAATAAAAACCACAACAC------ Hsapiens
 356389: ............................................------ Ptroglodytes
 374457: ............................................------ Ggorilla
 405960: ............................................------ Pabelli
 327516: .........C..................................------ Nleucogenys
 403029: .......A....................................AGGACT Mmulatta
 389745: C.A.........T...............................------ Cjacchus
1036938: ............T...............................------ Sboliviensis

                                   LTR
         ------------------------------------------------->
                                                      36723
                                                         :
  36722: ------------------------------------------------AG Hsapiens
 356433: ------------------------------------------------.. Ptroglodytes
 374501: ------------------------------------------------.. Ggorilla
 406004: ------------------------------------------------.. Pabelli
 327560: ------------------------------------------------.. Nleucogenys
 403079: CTGTGTCAGGAAAACCCATATCTCTCTTGGCGTGTACTCTCATTCAAT.. Mmulatta
 389789: ------------------------------------------------.A Cjacchus
1036982: ------------------------------------------------.. Sboliviensis

                                   LTR
         ------------------------------------------------->
                 36733     36743     36753     36763     36773
                     :         :         :         :         :
  36724: GACTATATGGGCTGTTTTCCAGTCAGGACAACCCACATCTCTCTTAGCGT Hsapiens
 356435: ...............................................T.. Ptroglodytes
 374503: ................................................A. Ggorilla
 406006: .................................................. Pabelli
 327562: A...C.................................G........... Nleucogenys
 403129: ....C.......................A......T......T..G.... Mmulatta
 389791: ....C.T..............A.....T...G.............G.A.. Cjacchus
1036984: ....C...-..T..........C...AT...G.............G.A.. Sboliviensis

                                   LTR
         ------------------------------------------------->
                 36783     36793     36803     36813     36823
                     :         :         :         :         :
  36774: GTACTCTCATTCAATAAACTCTCTGGTCTCTTAGCTAAATTGTCTCTTGG Hsapiens
 356485: .................................................. Ptroglodytes
 374553: .................................................. Ggorilla
 406056: .......................-...A..C..........T........ Pabelli
 327612: ........................A..A...................... Nleucogenys
 403179: .......................C.C........................ Mmulatta
 389841: .........................C...T..T................. Cjacchus
1037033: .............G...........C...T..T................. Sboliviensis

                                   LTR
         ------------------------------------------------->
                 36833     36843     36848     36858     36867
                     :         :         :         :         :
  36824: CCAAATTCTTTCTCTCAAAAAA-----GACAGAAACGGAGGA-CCCCCAC Hsapiens
 356535: ......................-----...............-....... Ptroglodytes
 374603: ......................-----........T..G...-....... Ggorilla
 406105: ......................-----.........A.....-....... Pabelli
 327662: ......C...............-----............A..C....... Nleucogenys
 403229: .....................T-----.........A.....-.T..... Mmulatta
 389891: ......................AAAAA....A....C.....-.T..... Cjacchus
1037083: ......C...............----A....A....CA....-.T..... Sboliviensis
```

```
              LTR
         ────────────►
              36877     36887     36897     36907    36916
  36868: ACTTCCAGGTGACATCCTCAAGATGTTTAGGGTGAAAAGTAGATATC-AA  Hsapiens
 356579: ....................C..........................-..  Ptroglodytes
 374647: ....................C..........................-..  Ggorilla
 406149: ....................CA.........A.............C.-..  Pabelli
 327707: ....................CA.......................C.-..  Nleucogenys
 403273: ..........A...A.....CA....................G..C.A..  Mmulatta
 389940: G...................CA...............CA......C.-..  Cjacchus
1037128: ....................CA...C..........GT....G..C.A..  Sboliviensis

              36926     36936     36946     36956     36966
  36917: AAACCATTCTTACTTTTCCTACACCCATCATTGTTGCTTCATGTCTCTCC  Hsapiens
 356628: ........................................G.........  Ptroglodytes
 374696: ..................................................  Ggorilla
 406198: ............T.....................................  Pabelli
 327756: ............T.....................................  Nleucogenys
 403323: ............T.......................T...G.........  Mmulatta
 389989: ..GA..--A...T.............TA......................  Cjacchus
1037178: .G..T.......T........T..A.T...C...................  Sboliviensis

              36976     36986     36996     37006     37016
  36967: ATGTTACCACAGTTAAACCCACTGTGCCTGTGGAGTCTTGGGTTATCCTG  Hsapiens
 356678: ..................................C......A........  Ptroglodytes
 374746: ..................................C......A........  Ggorilla
 406248: ..................................C......A........  Pabelli
 327806: ..................................C......A........  Nleucogenys
 403373: ..................................C......A........  Mmulatta
 390037: ..................................C..C...A........  Cjacchus
1037228: ..................................C..C...A........  Sboliviensis

              37026     37036     37046     37056     37066
  37017: TGATGTTGGCCAAGACAGCTTCTTGTCTTTATGCCATGTGACGAATGGGC  Hsapiens
 356728: .......A..................................A.......  Ptroglodytes
 374796: .............................C.....G......A....A..  Ggorilla
 406298: .....................G....................A.......  Pabelli
 327856: ............G.............................A.......  Nleucogenys
 403423: ...C...A......G..A........................AG......  Mmulatta
 390087: ...CA.........G.........A.................A.....A.  Cjacchus
1037278: ...CAC........G...........................A.......  Sboliviensis

              37076     37086     37096     37106     37116
  37067: CTGGTAGACAATCACTGGGACAACCACGTGCAACAAGACAGCCATTCAGT  Hsapiens
 356778: ...........................A......................  Ptroglodytes
 374846: ....................................C.............  Ggorilla
 406348: ..................................................  Pabelli
 327906: .............G....................................  Nleucogenys
 403473: ...........................A......................  Mmulatta
 390137: ....G............T........T.G......T..............  Cjacchus
1037328: ....G.............A..G.............T..............  Sboliviensis
```

```
                                                   LTR
                                      ---------------------->
              37126     37136     37146     37156     37166
                  :         :         :         :         :
  37117: GCAGCAAGGAAGTTGCATGCTATTATAAGTAGGTACTCTTCAGTCTTACA  Hsapiens
 356828: ..................................................  Ptroglodytes
 374896: ..................................................  Ggorilla
 406398: ...................................T..............  Pabelli
 327956: .........................................T........  Nleucogenys
 403523: ..C.............................A..T..............  Mmulatta
 390187: ........A...C..T........G.......A..T..............  Cjacchus
1037378: ............C..T........G.......A..T.......C......  Sboliviensis

                                LTR
         ------------------------------------------------->
              37176     37186     37196     37206     37216
                  :         :         :         :         :
  37167: CTTTGCTGAGATTGTTGCTTGCTTGCAATTTTATAATATTTTTATTGAAG  Hsapiens
 356878: ..................................................  Ptroglodytes
 374946: ..................................................  Ggorilla
 406448: .......................................C..........  Pabelli
 328006: ..................................................  Nleucogenys
 403573: .................T...T.................C.A........  Mmulatta
 390237: ..................AA..........C........CC.........  Cjacchus
1037428: .............A....A.....AA.............CC.........  Sboliviensis

                                LTR
         ------------------------------------------------->
              37226     37236     37246     37256     37266
                  :         :         :         :         :
  37217: ATTAAAGTAAGCCACATTCTTCAATGCCCCAAGGAGAAAGGAAAAGATTT  Hsapiens
 356928: ..............T...................................  Ptroglodytes
 374996: ..............T...T...............................  Ggorilla
 406498: ..............T....................C..............  Pabelli
 328056: ..............T..............................T....  Nleucogenys
 403623: ..............T.......................G...........  Mmulatta
 390287: .........GA...T............T..-...G........C......  Cjacchus
1037478: .......C.GA...T............T......G........C......  Sboliviensis

                    LTR
         ------------------------>
              37276     37286     37296     37306     37316
                  :         :         :         :         :
  37267: CATTAGCTGCCACCGCTGTCTATCCTAAAAGAATGTTGCCCTGTCACCTA  Hsapiens
 356978: .........T....A...................................  Ptroglodytes
 375046: ..............A...................................  Ggorilla
 406548: ..............A.-..T....................-.........  Pabelli
 328106: ..............A....................C....-.........  Nleucogenys
 403673: T...........G.A.........................-.A.......  Mmulatta
 390336: ..G.GA........A..A...G..................-.........  Cjacchus
1037528: T.G.GA........A......G..................-.........  Sboliviensis

                                                 LTR
                                    ------------------------>
              37326     37336     37346     37356     37366
                  :         :         :         :         :
  37317: GGAGGGAAGCAATGGCTGACCTCCAGCCACCTCTTCTAAGATGGGGCTAG  Hsapiens
 357028: ..................................................  Ptroglodytes
 375096: ..................................................  Ggorilla
 406596: ...........G......................................  Pabelli
 328155: .........A........................................  Nleucogenys
 403722: ..................................................  Mmulatta
 390385: ....................-..A.....G..T..G..C.....AA....  Cjacchus
1037577: ....................-..AG....G.....G.........A....  Sboliviensis
```

```
                                  LTR
          ------------------------------------------------->
                   37376     37386     37396     37406     37416
                       :         :         :         :         :
  37367: GGAAGGTAGAAGTGGGGGACTCCTGCTCTACTCAGACTTCTGAGGAAGTT  Hsapiens
 357078: ..................................................  Ptroglodytes
 375146: ..................................................  Ggorilla
 406646: ...........................................A......  Pabelli
 328205: .....T..........A....T.CA...............C.......C.  Nleucogenys
 403772: .....T........-----------.......A.................  Mmulatta
 390434: ........T..C..A....T......A..........C........C...  Cjacchus
1037626: ........T..C.......A......A.....G....CC...........  Sboliviensis

                                  LTR
          ------------------------------------------------->
                   37426     37436     37446     37456     37466
                       :         :         :         :         :
  37417: CCAGGAGAAAATATTCACAAAGAAGGCACTGAGAAGGCAGTAAATGTTTA  Hsapiens
 357128: ..................................................  Ptroglodytes
 375196: .................-................................  Ggorilla
 406696: ..................C...............................  Pabelli
 328255: ..................C................A..............  Nleucogenys
 403811: ..................C...GC......................C..T  Mmulatta
 390484: ..............G...C.......................C......T  Cjacchus
1037676: ..................C.................T.....C......T  Sboliviensis

                                  LTR
          ------------------------------------------------->
                   37475     37485     37495     37505     37515
                       :         :         :         :         :
  37467: ATAGCCAC-TCTTATCTGATTACTGCTCCATGTTATCCTGAGTATAAAAG  Hsapiens
 357178: ........-.......................A.................  Ptroglodytes
 375245: ........-.........................................  Ggorilla
 406746: ......G.-.........................................  Pabelli
 328305: ........-.........................................  Nleucogenys
 403861: ..T.A...T.T...........T...........................  Mmulatta
 390534: ........-.............T....T......................  Cjacchus
1037726: ........-.............T....T....C.................  Sboliviensis

                                  LTR
          ------------------------------------------------->
                   37525     37535     37545     37555     37565
                       :         :         :         :         :
  37516: GAGGCTTCCTTTAAAACAAAAATCTTCTGATTGACTTATGCCTTTTTCTG  Hsapiens
 357227: ..................................................  Ptroglodytes
 375294: ..................................................  Ggorilla
 406795: ..................................................  Pabelli
 328354: ..................................A...............  Nleucogenys
 403911: ..................................................  Mmulatta
 390583: ..A....T................................T.....G..A  Cjacchus
1037775: ..A....T..............A..................T.....G...  Sboliviensis
```

```
           LTR
         ------------------->
                    37575     37585     37595     37605     37615
                        :         :         :         :         :
   37566: TTTTGAACCTTTACTTGCTGAAGAATAAGATGGGTAGGAGAAGGAGGGAG  Hsapiens
  357277: .........................G........................  Ptroglodytes
  375344: .........................G........................  Ggorilla
  406845: .......A.................G.............              Pabelli
  406886:                                          .........  Pabelli
  328404: .........................G........................  Nleucogenys
  403961: .......T................TG...G........GA..........  Mmulatta
  390633: ....AC..............G....A.G.G.......A..G...T.A...  Cjacchus
 1037825: ....A...............G..---------------------------  Sboliviensis

                    37625     37635     37645     37655
                        :         :         :         :
   37616: AGAAGGAGGAGCAGAAGGATGAGGAGGATTGGGGAGAAGAGGAAGAA---  Hsapiens
  357327: ...............................................---  Ptroglodytes
  375394: ......-........................................GAA  Ggorilla
  406895: ..............................A................---  Pabelli
  328454: .......A...........CA............A..........T..---  Nleucogenys
  101684:                                   .A..A.AA..A..---  Mmulatta
  404011: ................................A.                  Mmulatta
   32198:                                 ...A..A.AA..A.G---  Panubis
  390683: ---------------------------------------------..---  Cjacchus
 1037848: ---------------------------------------------..---  Sboliviensis

                    37666     37676
                        :         :
   37663: ------GAAAAAGAAGAGGAAGAAGAA-----------------------  Hsapiens
  357374: ------...G.................-----------------------  Ptroglodytes
  375443: GACGAG...G..........C......-----------------------  Ggorilla
  406942: ------...G.......A..G.G.--------------------------  Pabelli
  328501: ------...GC.....CA....C....-----------------------  Nleucogenys
  101697: ------A...........AG...                             Mmulatta
  404076:                        T...CTTGTTCATCATATGCAACTGGG  Mmulatta
   32213: ------A........A..A..A..                            Panubis
  390685: --------------------------------------------------  Cjacchus
 1037850: --------------------------------------------------  Sboliviensis

                    37689                37703              37712
                        :                    :                  :
   37684: ----GAGGACAGAGGA------GGAGGAGGAGGAAG-----------GGG  Hsapiens
  357395: ----............------.........T..G.-----------A..  Ptroglodytes
  375470: ----......G.....GGTGGT..............-----------...  Ggorilla
  406960: ----.GA.GAG.....------...A.G....A.GA-----------A.A  Pabelli
  328522: ----..A..AG.....------.....GA...A.G.-----------A..  Nleucogenys
  404103: TCTT......GAGCC.------...A..AT..A.TATCTTTTCTCCT...  Mmulatta
  390685: ----.G..GAGT.CA.------.A.A...C..A...-----------...  Cjacchus
 1037850: ----....GAG.....------..T..GA...A.G.-----------...  Sboliviensis

                    37713     37723     37733     37743     37753
                        :         :         :         :         :
   37713: ---------AGAAGAAGAAGGAGGAGGAGAAGAAGAAGAAGAAGAGGAAG  Hsapiens
  357424: AAGGGGAGA....................G..G.................  Ptroglodytes
  375505: ---------...........A....AA..G..G.................  Ggorilla
  406989: ---------..G..G..G..A..A..A.........G........A....  Pabelli
   70265:                                        ...G.....GA  Nleucogenys
  328551: ---------..G.AG..G...................A.            Nleucogenys
  404147: ---------G.CCAG...GA...AGA...--------...A......G..  Mmulatta
   41361:                                           .A......  Panubis
  390714: ---------..G..G....A.....A...G..G.--..........A...  Cjacchus
 1037879: ---------..T.......A...TG.A..G.------....G..GC....  Sboliviensis
```

```
              37763     37773     37783     37793     37803
                  :         :         :         :         :
  37754: AGGGAGGAGGAGGAAGAGGAGGAGAAGAGGAAGAAGAAGAAGAGGAGGAG  Hsapiens
 357474: ............A................................G....  Ptroglodytes
 375546: ............................C.....................  Ggorilla
 407030: .........A....G..A........A................A......  Pabelli
  70276: .............G...                                  Nleucogenys
 328597:                                            .......  Nleucogenys
 404181: .A............G.-------------------..G..GC........  Mmulatta
  41369: .A...........                                      Panubis
 354838:                                      ...G.GA..A...  Panubis
 390753: .A.A..A.....A.....A.........--........AGGA..A....A  Cjacchus
1037914: GA...A........G..A........-.TA.GA.G...A.....A.....  Sboliviensis
```

```
              37813               37826     37836     37846
                  :                   :         :         :
  37804: GAGGAAGAGAAGGAAGTAG-------AAGAAGGGGGAGGAGGAGGAAGAG  Hsapiens
 357524: ................A..GAAGAAG........................  Ptroglodytes
 375596: ................A..GAAGAAG........T...............  Ggorilla
 407080: .....G..AG..A.G.A..-------...G.A......A.......G...  Pabelli
 328604: .....GA.....A.G--..-------...G...AA.........A.GAG.  Nleucogenys
 404212: A....G.GAG.A....G..-------...-.......A....G.......  Mmulatta
 354851: .-.......GG....AGG.-------..AG.A.....A.....T..G...  Panubis
  37885:                                   .AG.T..TG.......  Cjacchus
 390801: .....G...G..A...A..-------.....A                    Cjacchus
1037963: A..A....AG.-A.G.A..-------..AG...AA..............A  Sboliviensis
```

```
  37847: GA------------------------------------------------  Hsapiens
 357574: ..GAAGAAGAAGGAGGAGGAGGAGAAGAAGAAGAAAAGGAAGAGGGAGGA  Ptroglodytes
 375646: ..------------------------------------------------  Ggorilla
 407123: A.                                                  Pabelli
 328645: A.------------------------------------------------  Nleucogenys
 404254: .G------------------------------------------------  Mmulatta
 354893: ..------------------------------------------------  Panubis
  37901: ..------------------------------------------------  Cjacchus
1038005: ..------------------------------------------------  Sboliviensis
```

```
              37849     37859     37869     37878     37888
                  :         :         :         :         :
  37849: ---------GGAGAAGGGGAAGAAGAAGAAGAAGAG-GAGGAGGAGGAAG  Hsapiens
 357624: GGAGTAAGA....G..AA..G...............-.............  Ptroglodytes
 375648: ---------............---............-.............  Ggorilla
 407050:          ......AA...................-.............  Pabelli
 328647: ---------.A.....AA..G..G..G..G......-...C.......G.  Nleucogenys
 404256: ---------...A..A.....--.....GG......-..C........G.  Mmulatta
 354895: ---------.......--------.......G.AG.---....A..C.G.  Panubis
  37903: ---------..G..GT.....                               Cjacchus
 390849:                      .G.AGG....G..G.-AG....AGAA.G.  Cjacchus
1038007: ---------A..A..AA.A.G..G..G..G.G..G.A...A.A.G...G.  Sboliviensis
```

```
              37898               37915     37925     37932
                  :                   :         :         :
  37889: AGAAGGAAGAAGGAAGGG---GAGAAGGAGGAGGAGAAAA-T--ATTATT  Hsapiens
 357673: ..................---.......G...........-.CT......  Ptroglodytes
 375685: ..................---...................-.CT......  Ggorilla
 407090: ..................---...................-.CT......  Pabelli
 328687: ..................---...................-.CT......  Nleucogenys
 404294: ................A.GAG...................-.CT...G..  Mmulatta
 354925: ................A.GAG...................-.CT...G..  Panubis
 390877: G....A...G........CCG...................A.CT......  Cjacchus
1038048: .....A...G........CAG...................-.CT......  Sboliviensis
```

```
               37942     37947     37952     37962     37972
   37933: CAATTGAGTAGTAT-----AATTC-----AATTCAATTCAAGCAATTAGC  Hsapiens
  357719: ..............-----.....-----..................G..  Ptroglodytes
  375731: ..............-----.....-----..................G..  Ggorilla
  407136: -.............-----.....-----..................G..  Pabelli
  328733: .....C........-----.....-----..................G..  Nleucogenys
  404343: T....C........-----.....AATTA..................G..  Mmulatta
  354974: .....C........CATTC.....-----.......C..........G..  Panubis
  390927: .....C......G.-----.....-----.......G.....G....G..  Cjacchus
 1038097: .....T.....CG.-----.....-----.......G.....G....G..  Sboliviensis

               37982     37992     38002     38012     38022
   37973: CAAAAATTCCACTGAAATATTGACTGAATTCAATTTATCCACATTCAAAG  Hsapiens
  357759: ...............................G..................  Ptroglodytes
  375771: ..............................TG..................  Ggorilla
  407175: ..............................AG....C.............  Pabelli
  328773: ..............................AG....C.............  Nleucogenys
  404388: ..............................AG.C..C.....G.......  Mmulatta
  355019: ..............................AG.C..C.....G.......  Panubis
  390967: .............A................TG.C..C.............  Cjacchus
 1038137: .............A.................G.C..C.............  Sboliviensis

               38032     38042     38052     38060     38070
   38023: TATCCTTTTATAAGCCATTTGATTGCTGCCAT--GTTACAAGTCTTCACA  Hsapiens
  357809: ................................--................  Ptroglodytes
  375821: ................................--.....G..........  Ggorilla
  407225: ................................AT................  Pabelli
  328823: ................................AT................  Nleucogenys
  404438: .........G......................AT................  Mmulatta
  355069: ................................AT................  Panubis
  391017: ........C.......................ATA............G..  Cjacchus
 1038187: ........C...C...............T...ATA...............  Sboliviensis

               38080     38090     38100     38109     38119
   38071: GAGCTTTAAGGTATTTCATGCATTTATTATTCT-CAACAGAGAATTTTGA  Hsapiens
  357857: .................................-................  Ptroglodytes
  375869: ..A..............................-................  Ggorilla
  407275: ........................C........-................  Pabelli
  328873: .......G.........................-.....C..........  Nleucogenys
  404488: ...............C.................-.....C......C...  Mmulatta
  355119: ...............C.................-.....C..........  Panubis
  391067: .....-..............GG.........T.T..G..C..........  Cjacchus
 1038237: .GC..--.............GG.........T.T.....C..........  Sboliviensis

               38129     38139     38149     38159     38169
   38120: ACTAAAACTCACCACATTCTATTTACATATGCAAGGAAAATATTTAAAAA  Hsapiens
  357906: ..................................................  Ptroglodytes
  375918: ..................................................  Ggorilla
  407324: ..................................................  Pabelli
  328922: ..............T................A.............T....  Nleucogenys
  404537: .........T........T............T.............-....  Mmulatta
  355168: .............G.................T..................  Panubis
  391116: ..............TGC..................T....C.........  Cjacchus
 1038285: ..............TG...................T....C....T....  Sboliviensis
```

```
              38179     38189     38199     38209     38219
   38170: TCCAAGCAAAGTTGAAGCCCTTCAGCTGGTTTTCTTTAAGCAGAGATGTC   Hsapiens
  357956: ..................................................   Ptroglodytes
  375968: ..................................................   Ggorilla
  407374: ..................................................   Pabelli
  328972: ..................................................   Nleucogenys
  404586: .................T................................   Mmulatta
  355218: ...........C......................................   Panubis
  391166: .T......................................T.........   Cjacchus
 1038335: ........................................T.........   Sboliviensis

              38229     38239     38249     38257     38267
   38220: AGGTCACTTATTCAAGATCTCACAGATACAT--ACAAGTCAAGAAAATCT   Hsapiens
  358006: ...............................--.................   Ptroglodytes
  376018: ...................G........T.C--.................   Ggorilla
  407424: .A.............................--.G...............   Pabelli
  329022: ...............................--.................   Nleucogenys
  404636: .C......................A...T..--.................   Mmulatta
  355268: .C......................A...T..--.................   Panubis
  391216: ..A..G.................T....T..GC.................   Cjacchus
 1038385: .....G.................T....T..--.........A.......   Sboliviensis

              38277     38287     38297     38307
   38268: AATCCCTTAAAATTTTACTCATTGACTTGATCATGTCACTGG--------   Hsapiens
  358054: .............CC...........................--------   Ptroglodytes
  376066: ..........................................--------   Ggorilla
  407472: ....................--.......T..G.........--------   Pabelli
  329070: ..........................................--------   Nleucogenys
  404684: ..C.A..............T......................--------   Mmulatta
  355316: ..........................................--------   Panubis
  391266: ........................G....C.....C.....ATTATGTTC   Cjacchus
 1038433: .....................C..G....C.....CTG...A-TTATGTT   Sboliviensis

                        38318     38328     38338     38348
   38310: -----------ACAGCACTCAAATGAGACACCACAGATAAGAAAGCCAGA   Hsapiens
  358096: -----------.......................................   Ptroglodytes
  376108: -----------.......................................   Ggorilla
  407512: -----------.......................................   Pabelli
  329112: -----------.......................................   Nleucogenys
  404726: -----------......A.....C..........................   Mmulatta
  355358: -----------......A.....C..........................   Panubis
  391316: TTGATCATGTT..........GT...........................   Cjacchus
 1038482: TTGATCATGTT..................G....................   Sboliviensis

              38358     38368     38378     38388     38398
   38349: AAGGCTTTCTGCATAAATCTACCTTTTTCCATGATTAACAAAGCCTTAGC   Hsapiens
  358135: ..................................................   Ptroglodytes
  376147: ..................................................   Ggorilla
  407551: ..................................................   Pabelli
  329151: .........................C........................   Nleucogenys
  404765: ..-................C.....A...T....................   Mmulatta
  355397: ..-................C.....A...T....................   Panubis
  391366: ..........---------C.....C...T................C..G   Cjacchus
 1038532: ..........---------C.........T................C..G   Sboliviensis
```

```
                                                           MIR
                                        -------------->
               38408     38418     38428     38438     38448
   38399: TTCACAACTCATGAAATGGTTGGAAGAAATCCTAGACCTATTTTGCAGAT Hsapiens
  358185: .................................................. Ptroglodytes
  376197: .................................................. Ggorilla
  407601: .................................................. Pabelli
  329201: ...........................G.........A............ Nleucogenys
  404814: .................T.........G......C....G.......... Mmulatta
  355446: .................T.........G......C....G.......... Panubis
  391407: .................T.........G...................... Cjacchus
 1038573: ..TG.............T.........G.................T..G. Sboliviensis

                      MIR
          ------------------------>
               38458     38468     38478     38487     38497
   38449: GAGCAAACTGAGGCACAGAGGTGTGTGAGGTGTGAACA-GCTTTTTGGCC Hsapiens
  358235: ............A.........................-.........T Ptroglodytes
  376247: ......................................-.........A Ggorilla
  407651: ..........................A.....C.....-........... Pabelli
  329251: ...G..................................-........... Nleucogenys
  404864: ..................................---.-........... Mmulatta
  355496: ..................................---.-........... Panubis
  391457: ......................................A.......A.T. Cjacchus
 1038623: .....................C................A.....C...T. Sboliviensis

               38507     38517     38527     38537     38547
   38498: GTGGGAAAGTCACAGGATTGTCTCTGCACCTCATCTGCAACTCTGAGCCT Hsapiens
  358284: .................................................. Ptroglodytes
  376296: A..............................T.................. Ggorilla
  407700: A................................................. Pabelli
  329300: A................................................. Nleucogenys
  404910: A.................A.........T.................CA.. Mmulatta
  355542: A...........................T.................CA.. Panubis
  391507: ..A...T.........................C................. Cjacchus
 1038673: A...A.T.........................C................. Sboliviensis

               38557     38567     38577     38587     38597
   38548: TGCTCAACCCCATCTGATTAGGGAGAGGCAAGCTTGCCACAGAATAGGAT Hsapiens
  358334: ......................................G........... Ptroglodytes
  376346: .................................................. Ggorilla
  407750: .................................................. Pabelli
  329350: .................................................. Nleucogenys
  404960: ......C........................................A.. Mmulatta
  355592: ......C........................................A.. Panubis
  391557: ..........G....................................A.. Cjacchus
 1038723: ...............................................A.. Sboliviensis

               38607     38617     38627     38637     38647
   38598: CGTGTTGGTATGAGTGTGCCTTATAAAGAAAAGGAAGGGGTGGTTCGCCT Hsapiens
  358384: ..............................C................... Ptroglodytes
  376396: ..............................C................... Ggorilla
  407800: ..C...........................C...........A....... Pabelli
  329400: ..............................C................... Nleucogenys
  405010: TACA..........................C.A................. Mmulatta
  355642: TACA.G........................C.A................. Panubis
  391607: .A.........C..................C..................A Cjacchus
 1038773: T..........C..................C..................A Sboliviensis
```

```
                    38657     38667     38677     38687     38697
                        :         :         :         :         :
  38648: TGAACTGAGCTCTCATTGGTAGGCTTTTTACAGCGATGCTGTCCCCAGTG   Hsapiens
 358434: ............G...G.................................   Ptroglodytes
 376446: ............G...G.................................   Ggorilla
 407850: ............G...G.................................   Pabelli
 329450: ............G...G.................................   Nleucogenys
 405060: ............G...G...........C.....................   Mmulatta
 355692: ............G...G...........C.....................   Panubis
 391657: ............G...G.....A.....C.....................   Cjacchus
1038823: ............G...G.....A.......G...................   Sboliviensis

                    38707     38717     38727     38737     38747
                        :         :         :         :         :
  38698: GCTGCTGCTGCCTCCGTGCCCTCTTCGCTGACATCCAGGTATGACTTGTG   Hsapiens
 358484: ..................................................   Ptroglodytes
 376496: ..........................A.......................   Ggorilla
 407900: ..................................................   Pabelli
 329500: ..................................................   Nleucogenys
 405110: ..G...............................................   Mmulatta
 355742: ..G...............................................   Panubis
 391707: ...........................T......................   Cjacchus
1038873: ..................................................   Sboliviensis

                    38757     38767     38777     38787     38797
                        :         :         :         :         :
  38748: GATGGCTTTTGATAAATATAGGCCCTTGGTTGGTGACATTCCAGAAAGAT   Hsapiens
 358534: ................................A.................   Ptroglodytes
 376546: .......C..........................................   Ggorilla
 407950: ....................T.............................   Pabelli
 329550: ....A...................T.........................   Nleucogenys
 405160: .....................A............................   Mmulatta
 355792: .....................A............................   Panubis
 391757: ..............G..............C....................   Cjacchus
1038923: ..............G...C..........C....................   Sboliviensis

                    38807     38817     38827     38837     38847
                        :         :         :         :         :
  38798: CAGCTTTGACCTGGTTGAAGAGATCTGTCACCCCTAGAGATTTTAACAGG   Hsapiens
 358584: ..................................................   Ptroglodytes
 376596: ..................................................   Ggorilla
 408000: ..................................................   Pabelli
 329600: .....................T............................   Nleucogenys
 405210: ..................................................   Mmulatta
 355842: ..............................T...................   Panubis
 391807: .....................T...........................A   Cjacchus
1038973: .....................T............................   Sboliviensis

                    38857     38867     38877     38887     38897
                        :         :         :         :         :
  38848: GAATTTAGCTCATACTTAGTTTCAAGTTTGAATCGGGGGAGGTGTACTTC   Hsapiens
 358634: ..................A...............................   Ptroglodytes
 376646: ..................A...............................   Ggorilla
 408050: ..................A......................A........   Pabelli
 329650: ............C.....A......................A........   Nleucogenys
 405260: ..................A...................A..A........   Mmulatta
 355892: ..................A...................A..A........   Panubis
 391857: .................CA.............C........A........   Cjacchus
1039023: .................CA.............C..A.....A........   Sboliviensis
```

```
              38907     38917     38927     38937     38947
                  :         :         :         :         :
  38898: AACTTCTCTTTCCATCATGTTAGAAGAGCTTGTCCACTCATGAAACGTCC  Hsapiens
 358684: ..................................................  Ptroglodytes
 376696: ..................................................  Ggorilla
 408100: ..............................................T...  Pabelli
 329700: ..............................G..............T....  Nleucogenys
 405310: ..............................G...................  Mmulatta
 355942: ..............................G...................  Panubis
 391907: ............................T.G...............A..T  Cjacchus
1039073: ..............................G........G......A..T  Sboliviensis

              38957     38967     38977     38987     38997
                  :         :         :         :         :
  38948: CCGAATTCAGCTGCTTTTCTATCTAAGAGACAAAACACAGACAAGCCCAC  Hsapiens
 358734: ..................................................  Ptroglodytes
 376746: ..................................................  Ggorilla
 408150: ..A...............................................  Pabelli
 329750: .........A........................................  Nleucogenys
 405360: T....................................G............  Mmulatta
 355992: T.................................................  Panubis
 391957: T..................................T.....TG.......  Cjacchus
1039123: T..................................T.....TG.......  Sboliviensis

              39007     39017     39027     39036     39046
                  :         :         :         :         :
  38998: ATGAGTCAAACACACGACCTCTACATTAAATATCATAT-GTTTTTATAAT  Hsapiens
 358784: ...............................G......-...........  Ptroglodytes
 376796: ......................................-...........  Ggorilla
 408200: ..............T................G......-...........  Pabelli
 329800: ..............T................G......-.....C....C  Nleucogenys
 405410: ..............T................G......G.....CG....  Mmulatta
 356042: ..............T................G......-...........  Panubis
 392007: .-...C........T.......G.....G..G....T.-......-....  Cjacchus
1039173: .....C........T.......G.....G..G....T.-......T.C..  Sboliviensis

              39056     39066     39076     39086     39096
                  :         :         :         :         :
  39047: TCTCAGTGTGAGTGGTTCAACTTCATCACTTTTTCTACCTTTAATACAGT  Hsapiens
 358833: ..................................................  Ptroglodytes
 376845: ..................................................  Ggorilla
 408249: ..................................................  Pabelli
 329849: ..................................................  Nleucogenys
 405460: ..............T...................................  Mmulatta
 356091: ...........A..T....G..............................  Panubis
 392054: .....A.....A.....T..T..T..........................  Cjacchus
1039222: .................T..T..------------.C....---------  Sboliviensis

              39106     39116     39126     39136     39146
                  :         :         :         :         :
  39097: CTACTTACCTGGGAACAAAATCATACCATTAGCCACATTTTCCCTTTTCT  Hsapiens
 358883: ..................................................  Ptroglodytes
 376895: ..................................................  Ggorilla
 408299: ..................................................  Pabelli
 329899: ................G.................................  Nleucogenys
 405510: ..................................................  Mmulatta
 356141: ..................................................  Panubis
 392104: .......T......................CA..................  Cjacchus
1039251: ----------------.....T.-G.........................  Sboliviensis
```

```
              39156     39166     39176     39186     39196
                  :         :         :         :         :
  39147: GTTGTCTGTTTCTCATCTAAATTTCATAATTAAGCTTCTCAAGTAGGTAT  Hsapiens
 358933: ..C...........G...................................  Ptroglodytes
 376945: ..C......................G........................  Ggorilla
 408349: ..CA.......................CG.....................  Pabelli
 329949: ..................................................  Nleucogenys
 405560: ..CA..........C...................................  Mmulatta
 356191: ..CA..........C...................................  Panubis
 392154: ...T....G........C......G......GGT...T.G..........  Cjacchus
1039284: .................G......G.......GT.....G....TT....  Sboliviensis

              39206     39216     39226     39236     39246
                  :         :         :         :         :
  39197: CCTACAGTCTTTTCCCTCAATTCCCTCAGCATACATGCTTCACTGCACTT  Hsapiens
 358983: ..................................................  Ptroglodytes
 376995: ..................................................  Ggorilla
 408399: ..................G...............................  Pabelli
 329999: .....G......................................C.....  Nleucogenys
 405610: ...............................C..................  Mmulatta
 356241: ...............................C..................  Panubis
 392204: ...G.......C......G...T.........G...A.....A..G....  Cjacchus
1039334: ...........C......G...T.........G.........AC......  Sboliviensis

              39256     39266     39276     39286     39296
                  :         :         :         :         :
  39247: CTCCCACTCTCGCCTGTGAAGTTGTCATGGTCCTGTTTCGTGGATGGACT  Hsapiens
 359033: ........A.........................................  Ptroglodytes
 377045: ........A.........................................  Ggorilla
 408449: ........A.T............................A..........  Pabelli
 330049: ........A.........................................  Nleucogenys
 405660: ........A.........................................  Mmulatta
 356291: ........A.........................................  Panubis
 392254: .....T..A..AT.........CA...............C.CAG......  Cjacchus
1039384: .....TG.A...T..C...G..CA.....C.........C..A...A...  Sboliviensis

              39306     39316     39326     39336     39346
                  :         :         :         :         :
  39297: CAACCATTTCTTCTGGGTTCTCATATCTGAAACATTTCATGATACCCTCC  Hsapiens
 359083: ..................................................  Ptroglodytes
 377095: ..................................................  Ggorilla
 408499: .....................G.................C..C.......  Pabelli
 330099: .......................................C..C.......  Nleucogenys
 405710: ..............T.....................C..C..C.......  Mmulatta
 356341: ..............T.....................C..C..C.......  Panubis
 392304: ....T.........T................CA.........C......A  Cjacchus
1039434: ....T.........T........................C..C......A  Sboliviensis

              39356     39366     39376     39386     39396
                  :         :         :         :         :
  39347: TTTATGAGCGTGATTCTTGTTTGGCATCCCTGATGCCAGGATCCCCATGC  Hsapiens
 359133: .......A......................................G...  Ptroglodytes
 377145: ..............................................G...  Ggorilla
 408549: .C............................................G...  Pabelli
 330149: ..............................................G...  Nleucogenys
 405760: .................C...........................TG..A  Mmulatta
 356391: .................C...........................TG..A  Panubis
 392354: .......ATT................................T.......  Cjacchus
1039484: .......A.T.......C................................  Sboliviensis
```

```
                    39406                39425     39435     39445
                        :                  :         :         :
   39397: ACTCTGATAGCTTTCATCC-TCTCGTGCCCTTCTCTGTCACTGTTTCTGG  Hsapiens
  359183: ...................-..............................  Ptroglodytes
  377195: ...G...............-..............................  Ggorilla
  408599: ...................-............................A.  Pabelli
  330199: .......C...........-...T..........................  Nleucogenys
  405810: .T.................T....A...................C.....  Mmulatta
  356441: .T.................T....A...................C.....  Panubis
  392404: ...................-....A.............TG....C.....  Cjacchus
 1039534: ......G.....C......-....A.............TG....C.....  Sboliviensis

                    39455     39465     39475     39485     39495
                        :         :         :         :         :
   39446: TAGCTGCTCCTGAAGCTGAACTCTGGGCCACCTGTGCCTCTGCCCAGCAT  Hsapiens
  359232: ...............................T..................  Ptroglodytes
  377244: ..................................................  Ggorilla
  408648: ..................................................  Pabelli
  330248: .......................C..........................  Nleucogenys
  405860: .............G....................................  Mmulatta
  356491: ..................................................  Panubis
  392453: ......................T...........C..............G  Cjacchus
 1039583: .G...........................T....C...............  Sboliviensis

                    39505     39515     39525     39535     39545
                        :         :         :         :         :
   39496: GTCAGCTCCCACATCAATGCTCAGCTTTCCCAAAGCAGCCTCAGCACACT  Hsapiens
  359282: ...............................................G..  Ptroglodytes
  377294: ...............................................G..  Ggorilla
  408698: ......................................A.......TG..  Pabelli
  330298: ..............................................TG..  Nleucogenys
  405910: ........................G......................T..  Mmulatta
  356541: .......G................G....T.................T..  Panubis
  392503: ........................G.....................AG..  Cjacchus
 1039633: ...........TG...........G.....................TG..  Sboliviensis

                              39562     39572     39582     39592
                                  :         :         :         :
   39546: GACTTCTCA---TTCTCCATCCAGTTTCATCATTTGTTTGTAAACCTTTC  Hsapiens
  359332: .........---......................................  Ptroglodytes
  377344: .........---......................................  Ggorilla
  408748: .........---......................................  Pabelli
  330348: .........---......................................  Nleucogenys
  405960: .........TTT..................G.............T.....  Mmulatta
  356591: .........TTT................................T.....  Panubis
  392553: A........---......................................  Cjacchus
 1039683: A........---...C..................T..C............  Sboliviensis

                    39602     39612     39622     39632
                        :         :         :         :
   39593: AAACGCCTCCCTCGGGCATTGAACTCTCCAGCCAGCCCTGTCCA------  Hsapiens
  359379: .......C....................................------  Ptroglodytes
  377391: ............................................------  Ggorilla
  408795: .......C.....A..............................------  Pabelli
  330395: .......C...............................A....------  Nleucogenys
  406010: .......C.....A....C..................G......CAGCTC  Mmulatta
  356641: .......C.....A....C.........................CAGCTC  Panubis
  392600: ...T...-....T.A...C.........................------  Cjacchus
 1039730: .......-......A...C................G........------  Sboliviensis
```

```
                 39645     39655     39665     39675     39685
                     :         :         :         :         :
  39637: -CAGCTGCAAACCACCATGCTGTGTCACATCCCATCTTTTTAATCTGAGA  Hsapiens
 359423: -.....C...........................................  Ptroglodytes
 377435: -.....C...................................G.......  Ggorilla
 408839: -.....C...........................................  Pabelli
 330439: -.....C...........................................  Nleucogenys
 406060: G.....C.......................................A...  Mmulatta
 356691: C.....C......................................CA...  Panubis
 392643: -.....C................................C..C.TG....  Cjacchus
1039773: -.....C..........C...A..............C..C..C..G....  Sboliviensis

                 39695     39705     39715     39725     39735
                     :         :         :         :         :
  39686: ATTTAAAACAGTTTTGACAACTACTGCCTACCATGACAATATAAAAGCCA  Hsapiens
 359472: ..........A...................................T...  Ptroglodytes
 377484: ..............................................T...  Ggorilla
 408888: ..............................................T...  Pabelli
 330488: ..............................................T...  Nleucogenys
 406110: .....................CT....................T..T...  Mmulatta
 356741: ..................C..CG....................T..T...  Panubis
 392692: G.......TTA.....G........C....................TT..  Cjacchus
1039822: G.......TGA.....G........C....T...............TT..  Sboliviensis

                 39745     39755     39765     39775     39784
                     :         :         :         :         :
  39736: CCAAAAAGCAAAGGTGGTTTTTCTTCCAAAGTATCTTTTCCTTTTGTC-A  Hsapiens
 359522: ...T...AT.......................................-C  Ptroglodytes
 377534: ...T...AT....A..................................-.  Ggorilla
 408938: ...T...AT.......................................-C  Pabelli
 330538: ...T...AT........C....-.C.......................-C  Nleucogenys
 406160: ...T...AT...T...................G...............-C  Mmulatta
 356791: ...T...AT...T...................GG..............-C  Panubis
 392742: ...TG...T.......................G...............-T  Cjacchus
1039872: ...TG...T.......................G...............TT  Sboliviensis

                 39794     39804     39814     39824     39834
                     :         :         :         :         :
  39785: TTTTCTGCCCACATCCAAGAGACTATGACTTCATGCCACAGCTCATGCTG  Hsapiens
 359571: ..........G.......................................  Ptroglodytes
 377583: ..........G.......................................  Ggorilla
 408987: ..........G.......................................  Pabelli
 330586: .........TG.........A..C..........................  Nleucogenys
 406209: .......................C.....................C....  Mmulatta
 356840: ...........................................G.C....  Panubis
 392791: .......................C.......TG.TG..............  Cjacchus
1039922: .......................C.......TGCA...............  Sboliviensis

                 39844     39854     39857     39867     39877
                     :         :         :         :         :
  39835: CCTTGCTTCTAAGGCATTTGCA-------CTGCTCTTTGCTCTGCTCAGT  Hsapiens
 359621: ......................-------.............G.......  Ptroglodytes
 377633: ......................-------.....................  Ggorilla
 409037: ..C...................-------............G.....G..  Pabelli
 330636: ..C...................-------..................G..  Nleucogenys
 406259: ..C.T.................-------.....................  Mmulatta
 356890: ..C.T.................-------.....................  Panubis
 392841: T.......-.............GTGCTGTT....G...........TG..  Cjacchus
1039972: ........-.............-------.....G..C........TG..  Sboliviensis
```

```
                   39887     39897
                       :         :
  39878: TTAGCAGGGCCCCTGTGATTC-----------------------------  Hsapiens
 359664: .....................-----------------------------  Ptroglodytes
 377676: .....................-----------------------------  Ggorilla
 409080: ..........T..........-----------------------------  Pabelli
 330679: .....................-----------------------------  Nleucogenys
 406302: .....................-----------------------------  Mmulatta
 356933: .....................-----------------------------  Panubis
 392890: G........A.....C...G.GATTCTATTCTATTCTATTCTATTCTATT  Cjacchus
1040014: G..............CA...TGATTCTATTTTATTCTGTAGAATTCTATT  Sboliviensis


  39899: --------------------------------------------------  Hsapiens
 359685: --------------------------------------------------  Ptroglodytes
 377697: --------------------------------------------------  Ggorilla
 409101: --------------------------------------------------  Pabelli
 330700: --------------------------------------------------  Nleucogenys
 406323: --------------------------------------------------  Mmulatta
 356954: --------------------------------------------------  Panubis
 392940: CTATTCTATTCTATTCTATTCTATTCTATTCTATTCTATTCTATTCTATT  Cjacchus
1040064: CACC----------------------------------------------  Sboliviensis


                   39904     39914     39924     39934     39944
                       :         :         :         :         :
  39899: ----TCCTATTCATCCAGCAAGGCTCGGACTGAAATTGCTGTACTAGGAA  Hsapiens
 359685: ----..............................................  Ptroglodytes
 377697: ----..............................................  Ggorilla
 409101: ----.T............................................  Pabelli
 330700: ----.......T.............T.............C..........  Nleucogenys
 406323: ----....G......................A...G....C.G.......  Mmulatta
 356954: ----....G......................A...G....C.G.......  Panubis
 392990: CTAT..TA..........G..A....AC............C.........  Cjacchus
1040068: ----.T.....................C............C.........  Sboliviensis


                   39954     39964     39974     39984     39994
                       :         :         :         :         :
  39945: TTCCTTTCTGACCCATTCATTCTAATCCCATAGTCTACTTCCAGAAAACT  Hsapiens
 359731: A...C.............................................  Ptroglodytes
 377743: A..................................A.T............  Ggorilla
 409147: A.............C...................................  Pabelli
 330746: A.............C.........G.........................  Nleucogenys
 406369: A...C.........C...............A...................  Mmulatta
 357000: A.............C...............A...................  Panubis
 393040: AC......C...A.C.....................G..G.T........  Cjacchus
1040114: AC............C.....................G....T........  Sboliviensis


                   40004     40014     40024     40034     40044
                       :         :         :         :         :
  39995: TTCTGCAGAATCTAAACTGCTCCCAAACTGCATTATGCCTCCAAATGTCT  Hsapiens
 359781: .........G........................................  Ptroglodytes
 377793: .........G........................................  Ggorilla
 409197: .........G........................................  Pabelli
 330796: ........GG........................................  Nleucogenys
 406419: .........G.............A.......T............C.....  Mmulatta
 357050: .........G.....................T............C.....  Panubis
 393090: .CT......G.....................T.....T........A...  Cjacchus
1040164: .CT......G.........T...........T.....T........A...  Sboliviensis
```

```
              40054     40064     40074     40084     40094
                  :         :         :         :         :
   40045: TTCACTCCAGTCAACCACAGGCCCTTAGGGACCTGATTCCCTCTGACTTA  Hsapiens
  359831: .................................................G  Ptroglodytes
  377843: .................................................G  Ggorilla
  409247: .............-----------------...................G  Pabelli
  330846: ......................................G..........G  Nleucogenys
  406469: .........................C.......................G  Mmulatta
  357100: .........................C.......................G  Panubis
  393140: ......G...G...........T..C.-...................---  Cjacchus
 1040214: ..........A........A.....C.-.....................G  Sboliviensis

              40104     40114     40121     40131     40141
                  :         :         :         :         :
   40095: ACCACAACCGTAAGCATACCTGTT---ATGGCCACCGCTGCACTTCTGTG  Hsapiens
  359881: ........................---........A.............T  Ptroglodytes
  377893: ........................---........A.............T  Ggorilla
  409280: G........A..............-------.T..A.............T  Pabelli
  330896: G......T.A..............CTC..A.....A.............T  Nleucogenys
  406519: G........A..............CTC........A.-...........T  Mmulatta
  357150: G........A..............CTC........A..-..........T  Panubis
  393186: .........AG..T..........TTC........A........G....T  Cjacchus
 1040263: .......T.AC....G........CTC........A........G....T  Sboliviensis

              40151     40161     40171     40181     40191
                  :         :         :         :         :
   40142: ACTACAGCTCCTTTTCAGTTGGCACCCGTCTCACTTCTTAATCCAAATGC  Hsapiens
  359928: .............C...C................................  Ptroglodytes
  377940: .............C.............A......................  Ggorilla
  409327: ....T......C.C....................................  Pabelli
  330946: ...........C.C...A........T.......................  Nleucogenys
  406568: ...........C.C...A....................G...........  Mmulatta
  357199: ...........C.C...A................G...G...........  Panubis
  393236: .............C...A....T....A.................G..--  Cjacchus
 1040313: ...........C.C...A....T....A...T.............G..--  Sboliviensis

              40201     40211     40221     40231     40241
                  :         :         :         :         :
   40192: GTACGTCCTGACCCTGCTCTTTCAAGAAGCCTTCCCTACCACCCGACACA  Hsapiens
  359978: ........C.........................................  Ptroglodytes
  377990: ..................................................  Ggorilla
  409377: ...T.......................................T...GG.  Pabelli
  330996: ............T.....................................  Nleucogenys
  406618: ....A..................CG.........................  Mmulatta
  357249: ....A..................CG.........................  Panubis
  393284: -..T.......................................T......  Cjacchus
 1040361: -..T........................................A.....  Sboliviensis

              40249     40259     40269     40279     40289
                  :         :         :         :         :
   40242: CA--CACACTTTGCTCACCTGTGCCACAGTGTTCCTTCCCTACCACCCAA  Hsapiens
  360028: ..CA.....C.........-------------------------------  Ptroglodytes
  378040: ..--............................................G-  Ggorilla
  409427: ..--............................................G.  Pabelli
  331046: ..--..............................................  Nleucogenys
  406668: ..TG.....C.......T.-------------------------------  Mmulatta
  357299: ..TG.....C.......T.-------------------------------  Panubis
  393333: ..--.....CC........-------------------------------  Cjacchus
 1040410: ..--T....CC.........                                Sboliviensis
 1040389:                                  ................-  Sboliviensis
```

```
               40299     40309     40319     40329     40339
                   :         :         :         :         :
  40290: CACACACACACACCTTGCTCACCTGTGCCACAGTGTTTTCATCTCAAGCC  Hsapiens
 360047: -----------------------...........................  Ptroglodytes
 378087: -.................................................  Ggorilla
 409475: ..................................................  Pabelli
 331094: ..................................................  Nleucogenys
 406687: -----------------------.................G.........  Mmulatta
 357318: -----------------------.................G.........  Panubis
 393350: -----------------------.A...A..........TGC........  Cjacchus
1040405: -.......T.....C.........ACA.                        Sboliviensis
1040433:                              ..........TGC........  Sboliviensis

               40349     40359     40369     40379     40389
                   :         :         :         :         :
  40340: AGCACTCCCTGGCTTAGAAGCCCCTAACTGTTAGGCTATATCACCCATAT  Hsapiens
 360074: ................................G.................  Ptroglodytes
 378136: ................................G...............G.  Ggorilla
 409525: ...................A............G...C.............  Pabelli
 331144: ................................G...C.............  Nleucogenys
 406714: ................C...............G...C......T......  Mmulatta
 357345: ...........................G....G...C......T..C...  Panubis
 393377: ....................TT.....T...GT...C..CC..T.--.GC  Cjacchus
1040454: ....................TTT..C.T...GT...C..CC..T...C.-  Sboliviensis

               40399     40409     40419     40429     40439
                   :         :         :         :         :
  40390: GTGTGTCAGCTTCTGCAGGCTTCCAGACGTCCAGCTGTTAGAAGCACAGC  Hsapiens
 360124: ............................T.T.......G...........  Ptroglodytes
 378186: ..............................T...................  Ggorilla
 409575: ...........................A..T...................  Pabelli
 331194: .....................C.....A..T.............T.....  Nleucogenys
 406764: ............-........C.....A..T.......C...........  Mmulatta
 357395: ............-........C.....A..T.......C...........  Panubis
 393425: ....A................C.A...A..T.............G.....  Cjacchus
1040503: -...A................C.....A..T.....A....G---.....  Sboliviensis

               40449     40459     40469     40479     40489
                   :         :         :         :         :
  40440: CTGCTCTATGCTGGGCAGCTTTGGCTGCAGCTGGGGAAAGGGGGTTGGAT  Hsapiens
 360174: .................................A................  Ptroglodytes
 378236: ..................................................  Ggorilla
 409625: ..........................A.......................  Pabelli
 331244: ..................................................  Nleucogenys
 406813: .......G.C...........C.T....................A.....  Mmulatta
 357444: .......G.C...........C......................A.....  Panubis
 393475: .C.T...G.....................T..............A.....  Cjacchus
1040549: ...T...G....................................A.....  Sboliviensis

               40499     40509     40519     40529     40539
                   :         :         :         :         :
  40490: CCAAACTCCTTTCACGGCCATGACCACTTCTTAATGTGTGTGGTGCCCAT  Hsapiens
 360224: ....................................G.............  Ptroglodytes
 378286: ..................................C.G.............  Ggorilla
 409675: ...............T....................G.............  Pabelli
 331294: ...............T..........T.........G.............  Nleucogenys
 406863: ...............T..................C.G.........A...  Mmulatta
 357494: ...............T..................C.G.........A...  Panubis
 393525: .T.............CA..C................GC............  Cjacchus
1040599: .T.............CA.TG.........A......A.............  Sboliviensis
```

```
                 40549     40559     40569     40579     40584
                     :         :         :         :         :
    40540: TTCTCCACAAAACTGAGATGTTAAAAATGGTTAGCCTGTGAAAG-----T  Hsapiens
   360274: ............................................-----.  Ptroglodytes
   378336: ....................................C.......-----.  Ggorilla
   409725: ............T...............................TACAT.  Pabelli
   331344: ....................................C.......TACAT.  Nleucogenys
   406913: ........................T.....C.....CAC.....TACAT.  Mmulatta
   357544: ........................T.....C.....CA......TACAT.  Panubis
   393575: ...........G..........G.............CA......TACAT.  Cjacchus
  1040649: ......................G.............CA......TACAT.  Sboliviensis

                 40594     40604     40613     40623
                     :         :         :         :
    40585: ACAATTGGCTCAGAAATACTGCCCCC-AGAGTAAAATGGCTTAATCAGA-  Hsapiens
   360319: .........................TC......................-  Ptroglodytes
   378381: .........................TC......................-  Ggorilla
   409775: ....................T....TG....................T.-  Pabelli
   331394: ........................TTG......................-  Nleucogenys
   406963: .........................TG.CG.....C.....G....G..-  Mmulatta
   357594: .........................TG..G.....C.....GG......-  Panubis
   393625: ....A...........C........TG.......G..............-  Cjacchus
  1040699: ....A....................TG.......G..............T  Sboliviensis

                 40641     40651     40661     40671     40681
                     :         :         :         :         :
    40633: -TTTCTGCACACTAGGAGCCCAAATAGCATTATAACTTCATTACTATAGA  Hsapiens
   360368: -.................................................  Ptroglodytes
   378430: -.................................................  Ggorilla
   409824: -.................................................  Pabelli
   331443: -.................................................  Nleucogenys
   407012: -................T...........................G....  Mmulatta
   357643: -............................................G....  Panubis
   393674: -............G...............................GC...  Cjacchus
  1040749: C.....A.G.............................T......G....  Sboliviensis

                 40691     40701     40711     40721     40731
                     :         :         :         :         :
    40682: AAGATGTCCCCCGACTATCAACCAACCAGTACTTGTGTGTATTTGTACAA  Hsapiens
   360417: ..................................................  Ptroglodytes
   378479: ...........T........G.............................  Ggorilla
   409873: ...................................C..............  Pabelli
   331492: ...........T......................................  Nleucogenys
   407061: ............A.TC..................................  Mmulatta
   357692: ............A.TC..................................  Panubis
   393723: .C.........-A........A.........T......A...........  Cjacchus
  1040799: ............A........A.........T.----.C...........  Sboliviensis

                 40740     40750     40760     40770     40780
                     :         :         :         :         :
    40732: A-ATATCACTTCTGCTTCCATGTGCCTTTCTCATGTAAGAATAGCTTGTT  Hsapiens
   360467: .-................................................  Ptroglodytes
   378529: .-................................................  Ggorilla
   409923: .-...C............................................  Pabelli
   331542: .TT.............................G.................  Nleucogenys
   407111: .-.C...........C..................................  Mmulatta
   357742: .-.C...........C..................................  Panubis
   393772: .-.............A.T......G....T....................  Cjacchus
  1040845: .-.............A.T......G....TC...................  Sboliviensis
```

```
                40790     40800     40810     40820     40830
                    :         :         :         :         :
  40781: CTCCATAAACACACTGTCCTGAAAGGCAGAGGCCATGTGTCTCCCTGCTA  Hsapiens
 360516: ..................................................  Ptroglodytes
 378578: ..........G.......................................  Ggorilla
 409972: ..................................................  Pabelli
 331592: .........................A......A...A.............  Nleucogenys
 407160: .............T.....................C..............  Mmulatta
 357791: .............T.....................C..............  Panubis
 393821: .........T.T.T.........G.....G.......CA......CA...  Cjacchus
1040894: .........T...T...............G.A.......C.....CA.C.  Sboliviensis

                40839     40847     40857     40867     40877
                    :         :         :         :         :
  40831: CTTTCACA-CCCC--CGGGACAATCCTACTAAGGCTTAGATTGTGAATCA  Hsapiens
 360566: ........-....--...............G...................  Ptroglodytes
 378628: ........-....--...................................  Ggorilla
 410022: ........-....AGTA.................................  Pabelli
 331642: ........C....--T...........C.C....................  Nleucogenys
 407210: .....G..G....--T...G..........G...................  Mmulatta
 357841: .....G..G....--T...G..........G...................  Panubis
 393871: ........G....--T...G....T..........C..............  Cjacchus
1040944: ........G....--T...G....T..........C..............  Sboliviensis

                                                Simple
                                       ------------------------->
                40887     40897     40907     40917     40927
                    :         :         :         :         :
  40878: ATGACTACGCTAATGTCATGAGATACACAGTGTGTCTGTGTGTGTGTGTG  Hsapiens
 360613: ........A.........................................  Ptroglodytes
 378675: ........A.........................................  Ggorilla
 410071: ........AT........................................  Pabelli
 331690: ....T...A.........................................  Nleucogenys
 407258: ........A...................T......G..............  Mmulatta
 357889: ........A...................T......G..............  Panubis
 393919: ........A..G..........T.....T......G..............  Cjacchus
1040992: .......AA..G..........T.....T......G.......A......  Sboliviensis

                  Simple
         ------------------------->
                                          40933     40943     40953
                                              :         :         :
  40928: ------------------------TTAACATGCACACATGCTTTAACTGC  Hsapiens
 360663: ------------------------..........................  Ptroglodytes
 378725: TGTGTCTGTGTGTGTGTGTG----..........................  Ggorilla
 410121: TGTG--------------------..........................  Pabelli
 331740: ------------------------..........................  Nleucogenys
 407308: TGTG--------------------.G......................T.  Mmulatta
 357939: ------------------------.G........................  Panubis
 393969: TGTGTGCGCGCGTGCGTGTGCGCA.C...........G...........T  Cjacchus
1041042: TGTGTTTGTGTCTGTGCG------..........................  Sboliviensis

                40963     40973     40983     40993     41003
                    :         :         :         :         :
  40954: ATTATAGCATTCTTATAATTTTACCTGTTTCAGATTAGCTATGCCTACTG  Hsapiens
 360689: ........................................G.A.......  Ptroglodytes
 378771: ........................................G.........  Ggorilla
 410151: .....................................A.CG.........  Pabelli
 331766: ................................C.......G.........  Nleucogenys
 407338: T..C............................C.......G.........  Mmulatta
 357965: T..C............................C.......G.........  Panubis
 394019: .-------------------..........T......T..G.....G...  Cjacchus
1041086: .-------------------..........T......T..G.........  Sboliviensis
```

```
               41013     41023     41033     41043     41053
                   :         :         :         :         :
   41004: GAAGCAGAATAATCATGCTTAATTTGTTGTTAACGTAGGGCAGCTCAAGA   Hsapiens
  360739: ..................................................   Ptroglodytes
  378821: ..................................................   Ggorilla
  410201: ..................................A...............   Pabelli
  331816: ..................................A...............   Nleucogenys
  407388: ..................................................   Mmulatta
  358015: ..................................................   Panubis
  394050: .....................G.........C..A..T....A.......   Cjacchus
 1041117: .....................G.........C..A..C....A.......   Sboliviensis

               41063     41073     41083     41093     41103
                   :         :         :         :         :
   41054: ACTTGCATCTGCGGCTCCTTTACAAAGGCCAGTTTAAATGTTCCAATTTG   Hsapiens
  360789: ............A.....................................   Ptroglodytes
  378871: ..................................................   Ggorilla
  410251: ..................................................   Pabelli
  331866: ...........T...........G..........................   Nleucogenys
  407438: ......................GG.....................G....   Mmulatta
  358065: ...........T..........GG..........................   Panubis
  394100: ..C........T...........C..A....................C..   Cjacchus
 1041167: ..C....................G..A....................C..   Sboliviensis

               41113     41123     41133     41143     41153
                   :         :         :         :         :
   41104: ATACATCATTTCCACAGTTACATTTTTACCCTGTTAAGAAGAAAAAGATT   Hsapiens
  360839: .........A........................................   Ptroglodytes
  378921: ..................................................   Ggorilla
  410301: ..................................................   Pabelli
  331916: ..................................................   Nleucogenys
  407488: ..................................G..........G....   Mmulatta
  358115: .............................G....G..........G....   Panubis
  394150: .......................................G..........   Cjacchus
 1041217: .................................C................   Sboliviensis

               41155               41168     41178     41188
                   :                   :         :         :
   41154: --------ATAGTATAC----TTACA---TATGTGAGTGCACATGTGTGT   Hsapiens
  360889: --------.........----.....---.....................   Ptroglodytes
  378971: --------.........----.....---.....................   Ggorilla
  410351: GTAATTAGG........----.....---.....................   Pabelli
  331966: ATAATTAGG........----.....---.....................   Nleucogenys
  407538: ATAATTAGG.......TATAG.....---........CA...........   Mmulatta
  358165: ATAATTAGG.......TATAG.....---.....................   Panubis
  394200: TTAATTAGG........----.....TAT....................A   Cjacchus
 1041267: ATAATTAG.....G..T----....C---..C.................A   Sboliviensis

               41198     41208     41218     41228     41238
                   :         :         :         :         :
   41189: GTACTGATTACCTTGCATAACACGGATATCTGCATGAACACTCTAAAGGC   Hsapiens
  360924: A.....................T...........................   Ptroglodytes
  379006: A.....................T...........................   Ggorilla
  410394: A.G...G...............T...........................   Pabelli
  332009: A.....................T...........................   Nleucogenys
  407585: A..........T..........T.A.......................A.   Mmulatta
  358212: A..........T..........T.A.......................A.   Panubis
  394246: A........G............T.A..........AC............G   Cjacchus
 1041310: A........G............T.A..........AC...........A.   Sboliviensis
```

```
              41248     41258     41268     41278     41288
                  :         :         :         :         :
   41239: ATGGGTAAAATAGAAACTACAAAGTAGATTCCTTATTACTTCCCTTTGCA   Hsapiens
  360974: ...........G......................................   Ptroglodytes
  379056: ..................................................   Ggorilla
  410444: ..........................C.......................   Pabelli
  332059: .G..........-.........G...........................   Nleucogenys
  407635: ..................................................   Mmulatta
  358262: ..................................................   Panubis
  394296: G............................G....G...............   Cjacchus
 1041360: .............................G....G...............   Sboliviensis

              41298     41306     41316     41326     41336
                  :         :         :         :         :
   41289: AAGGTTATCCA--TTTTTTTAATTTAAAATAAGTCATGGTTAGAGACTGA   Hsapiens
  361024: ...........--.....................................   Ptroglodytes
  379106: ...........--.....................................   Ggorilla
  410494: ...........--..................................C..   Pabelli
  332108: .G.........--.....................................   Nleucogenys
  407685: ..AA.......--.....................................   Mmulatta
  358312: ..AA.......TT.............................T.......   Panubis
  394346: .-.........--......A......G.......................   Cjacchus
 1041410: ...........--......A..............................   Sboliviensis

              41346     41356     41366     41376     41386
                  :         :         :         :         :
   41337: ATTGTTCTAATAATATATAGAGATAGCTTGGTCTACCAAAAACAAAGTCA   Hsapiens
  361072: ..................................................   Ptroglodytes
  379154: ..................................................   Ggorilla
  410542: ..................................................   Pabelli
  332156: ...........................................-......   Nleucogenys
  407733: ...A...............T.....................G........   Mmulatta
  358362: ...A...............T.....................G........   Panubis
  394393: ...............CG..T..GG.A..............C.........   Cjacchus
 1041458: ................G..T..GG.A........................   Sboliviensis

              41396     41406                    41421     41431
                  :         :                        :         :
   41387: GGATTTTTAGCTAACATGATTTTAGTGG-----GAAGAAAAAGGATATAT   Hsapiens
  361122: .......................G....-----...A.............   Ptroglodytes
  379204: ............................----G...A.............   Ggorilla
  410592: ......C.....................----G...A....G........   Pabelli
  332205: ......C................T....------..A.............   Nleucogenys
  407783: ......C............C........AAAAAA..A..........C..   Mmulatta
  358412: ......C............C........--AAAA..A..........C..   Panubis
  394443: ..-...C.........G......T....-----A..A.....T......G   Cjacchus
 1041508: ..G...C................T....-------GA.....AT.....G   Sboliviensis

              41441     41451     41461     41471     41480
                  :         :         :         :         :
   41432: CATTAAATCATTTTCTTTTTAATCTTTCCTCAAGAAACTCTCTAG-AAAA   Hsapiens
  361167: .............................................A....   Ptroglodytes
  379250: .............................................-....   Ggorilla
  410638: .............................................A....   Pabelli
  332249: ............................................T-....   Nleucogenys
  407833: .............................................-....   Mmulatta
  358460: .............................................-....   Panubis
  394487: T............--....A..........G.......C......---..   Cjacchus
 1041551: T.......T....-..............T.............C..--...   Sboliviensis
```

```
                  41490     41500     41510     41520     41530
                      :         :         :         :         :
   41481: AAAAATAACACAGATAGCCTTAGTGTGCTGCTGGAGAACATAACCACAGT  Hsapiens
  361217: ..................................................  Ptroglodytes
  379299: .........T........................................  Ggorilla
  410688: ....T.........AT..................................  Pabelli
  332298: ..............G...................................  Nleucogenys
  407882: ................A.T...................A...........  Mmulatta
  358509: ................A.T...................A...........  Panubis
  394532: .........G........T...........A.......A.......TT..  Cjacchus
 1041598: ..................T...................A.......TC..  Sboliviensis

                  41540     41550     41560     41570     41580
                      :         :         :         :         :
   41531: ATATATGGAAAGAATAAGCCAAGAGAGCATTAAGAAAAGAATGGGTTTTC  Hsapiens
  361267: ..........................A......T................  Ptroglodytes
  379349: .......A.....G...A....T...A.......................  Ggorilla
  410738: .................A....T...A.......................  Pabelli
  332348: G................A........A.................C.....  Nleucogenys
  407932: G..C.............A....T...A.......................  Mmulatta
  358559: G..C.............A....T...A.......................  Panubis
  394582: ......A....T...GCA..C.T...A.......GG..............  Cjacchus
 1041648: .....GA....T...GCA..C.T...A.......G...C...........  Sboliviensis

                  41590     41600     41610     41620     41630
                      :         :         :         :         :
   41581: TCCTCTGCCAGAGAATCTTAGTATCCCAAGTCTCCAGTTAGGTGCTCTGT  Hsapiens
  361317: .T................................................  Ptroglodytes
  379399: .T................................................  Ggorilla
  410788: .T.....................................G..........  Pabelli
  332398: .T..........................G..........G..........  Nleucogenys
  407982: .T..T..................................G..........  Mmulatta
  358609: .T..T..................................G..........  Panubis
  394632: .T...................................GCG..........  Cjacchus
 1041698: .T.....T.T....................A......G.G..........  Sboliviensis

                  41640     41650     41660     41670     41680
                      :         :         :         :         :
   41631: GATAAAAGTTATTGTCACTTAATAAAGGAAAAATGAAAATCTGTAGAAAA  Hsapiens
  361367: .................T................................  Ptroglodytes
  379449: ..................................................  Ggorilla
  410838: ............................G.....................  Pabelli
  332448: ........................................G.A.......  Nleucogenys
  408032: ........................G.........T.....G.........  Mmulatta
  358659: ........................G.........T.....G.........  Panubis
  394682: ........C......T.....G..................T.........  Cjacchus
 1041748: ...........C...T........................T.........  Sboliviensis

                  41690     41700     41710     41720     41730
                      :         :         :         :         :
   41681: TACAATAAAAAGAACCAACAGAAACCTAGACCAATATTTTCACTAAATCA  Hsapiens
  361417: ..................................................  Ptroglodytes
  379499: ..................................................  Ggorilla
  410888: ..................G...............................  Pabelli
  332498: ...........A......................................  Nleucogenys
  408082: ......-...........................................  Mmulatta
  358709: ......-........................T..................  Panubis
  394732: ..............................T...................  Cjacchus
 1041798: ................................................--  Sboliviensis
```

```
                 41740     41750     41760     41770     41780
                     :         :         :         :         :
  41731: CTACATCTTTCTAAATTGTCCTTAAATGATTATTGTATCCTCCAACATTT  Hsapiens
 361467: ...A..................................T...........  Ptroglodytes
 379549: ......................................T...........  Ggorilla
 410938: ...A...........C......................T...........  Pabelli
 332548: T..A..................................T...........  Nleucogenys
 408131: ...A..........G.................C.....T.G.........  Mmulatta
 358758: ...A..........G.................C.....T...........  Panubis
 394782: ...A.C..........................C.....T...........  Cjacchus
1041846: ------.C............................G.T...........  Sboliviensis

                 41790     41800     41810     41820     41830
                     :         :         :         :         :
  41781: GTCATGAGTCTGAAAATAAAATACTTACCTCACTTAGCTGAAAAGGACTT  Hsapiens
 361517: ..................................................  Ptroglodytes
 379599: ..................................................  Ggorilla
 410988: .....C............................................  Pabelli
 332598: .....C......................................A.....  Nleucogenys
 408181: .....C............................................  Mmulatta
 358808: .....C............................................  Panubis
 394832: .....C....CA..................................GG..  Cjacchus
1041890: .....C....CA...................................G..  Sboliviensis

                 41840     41850     41860     41870     41880
                     :         :         :         :         :
  41831: TTAACTGTCTCTCTTACTTGAAATTTATTTTGCCATTGTCCTTTGAAATA  Hsapiens
 361567: ...............T..................................  Ptroglodytes
 379649: ...............T..................................  Ggorilla
 411038: ...............T..................................  Pabelli
 332648: ........G.....GT..................................  Nleucogenys
 408231: ...............T..................................  Mmulatta
 358858: ...............T..................................  Panubis
 394882: ....TA.........T..................................  Cjacchus
1041940: ....TC.........T..................................  Sboliviensis

                 41890     41900     41910     41920     41930
                     :         :         :         :         :
  41881: TATGGCATTCACCAGGACCATTACAGATGAAGGGTCAATTGTGCTCTTTC  Hsapiens
 361617: ............T...............................C.....  Ptroglodytes
 379699: ............................................C.....  Ggorilla
 411088: C..........................C................C.....  Pabelli
 332698: ............................................C.....  Nleucogenys
 408281: ............................................C.....  Mmulatta
 358908: ............................................C.....  Panubis
 394932: G........A....A.....G......................TC.....  Cjacchus
1041990: G........A....A.....G......................TC.....  Sboliviensis

                 41940     41950     41960     41970     41980
                     :         :         :         :         :
  41931: CAAAGAGATTTGCGACTTTTCCTTGTATAAAAAATAAGATAATTATATTT  Hsapiens
 361667: .............C..............................G.....  Ptroglodytes
 379749: ............T.....................................  Ggorilla
 411138: ............T...............................G.....  Pabelli
 332748: ............T...............................G.....  Nleucogenys
 408331: ............T.............G................CG.....  Mmulatta
 358958: ............T.............G.................G.....  Panubis
 394982: .......G....T...............................G.....  Cjacchus
1042040: ............T...............................G.....  Sboliviensis
```

```
                  41990     41999     42009     42019     42029
  41981: GGATTTGAAC-TCCTGATAATAGAGAGGTGAAGAGTCATGAGAAATTTAG  Hsapiens
 361717: ..........-..................A....................  Ptroglodytes
 379799: ..........-.......................................  Ggorilla
 411188: ..........-......................................A  Pabelli
 332798: ..........-.......................................  Nleucogenys
 408381: ..........T.......G....G.........................C  Mmulatta
 359008: ..........T..T....G....G.........................C  Panubis
 395032: ......T...T.AT...C.G.G.....T.........C.A..........  Cjacchus
1042090: ..........T.AT.....G.G.....T.........C.A..........  Sboliviensis

                  42039     42049     42059     42069     42079
  42030: AGCCATTTCTTTTTTAAGACTAATTGTAAATTTGAGATACCAGAAGAACT  Hsapiens
 361766: ..................................................  Ptroglodytes
 379848: ..................................................  Ggorilla
 411237: ..................................................  Pabelli
 332847: .........-........................................  Nleucogenys
 408431: .........................................---......  Mmulatta
 359058: .........................................---......  Panubis
 395082: ....G..............T....C..........A..............  Cjacchus
1042140: ..................................................  Sboliviensis

                  42089     42099     42109     42119     42129
  42080: CCTTTCATCTCCTGGCCATGGAGCCTTCTTTGTCAAATGAAGTTTATTTC  Hsapiens
 361816: ..................................................  Ptroglodytes
 379898: ....................................C.............  Ggorilla
 411287: ..................................................  Pabelli
 332896: ........T..............G..........................  Nleucogenys
 408478: ..................C...............................  Mmulatta
 359105: ..................................................  Panubis
 395132: ...........T............A.........................  Cjacchus
1042190: A..........T......................................  Sboliviensis

                  42139     42149     42159     42169     42179
  42130: AGGGAATATTTAATGCTTTATTATTTAATCATAAGACGGCACCCAGGAAG  Hsapiens
 361866: ...............................G....T.............  Ptroglodytes
 379948: ...............................G..................  Ggorilla
 411337: ...................G...........G.........T........  Pabelli
 332946: ...................G.......G...G.........T........  Nleucogenys
 408528: ...................G........C..G.....A...T........  Mmulatta
 359155: ...................G........C..G...G.A...T........  Panubis
 395182: ..A.C.....G........G...........GT...T....TT....G..  Cjacchus
1042240: ..A.......G.......AG...........G....T..........G..  Sboliviensis

                  42189     42199     42209     42219     42229
  42180: TAGCCAGCATATTCGTGATGGATCAATTTGTCTTATGCCTAGTTACATCG  Hsapiens
 361916: .......T..........................................  Ptroglodytes
 379998: .......T..........................................  Ggorilla
 411387: .......T...........................C..T......T...A  Pabelli
 332996: .....G.T..........C................C...G.....T....  Nleucogenys
 408578: .T.....T......A....................C....CA...T...A  Mmulatta
 359205: .T.....T......A....................C....CA...T....  Panubis
 395232: ....T..T.....A.....AA........................T...C  Cjacchus
1042290: ....T..T.....A.....AAG.......................T...C  Sboliviensis
```

```
                   42239     42249     42259     42269     42279
                       :         :         :         :         :
  42230: AGTAATTTCTTATACATCACTGCTTATTTAGATATTATGTAATCATTTCT  Hsapiens
 361966: ...GG.......C....T................C...............  Ptroglodytes
 380048: ...G.............T................................  Ggorilla
 411437: ...G.............T....................T...........  Pabelli
 333046: ...G.............T...........C........T...........  Nleucogenys
 408628: ...G..........T..T............A...C........A......  Mmulatta
 359255: ...G.......C..G..T............A............A......  Panubis
 395282: ..GGC......G.....T.T.C........A......CT....A......  Cjacchus
1042340: ...GC......G.....T.....-......A......CT....A......  Sboliviensis

                   42289     42299     42309     42319     42329
                       :         :         :         :         :
  42280: TGTGGCTTACTTTTAATATTTACAAGACTTTATACTTACCAGCAGTTCAC  Hsapiens
 362016: ............................................C.....  Ptroglodytes
 380098: ............................................C.....  Ggorilla
 411487: ....................................C.......C.....  Pabelli
 333096: ...A........................................C.....  Nleucogenys
 408678: .................................G..........C....T  Mmulatta
 359305: .................................G..........C.....  Panubis
 395332: ..............C..T.....T..........G.........C....G  Cjacchus
1042389: .......................T..........G......C..C....G  Sboliviensis

                   42339     42345     42355     42365     42375
                       :         :         :         :         :
  42330: TACACACAAATATAT----TATTTTAAAATATTTATTCTTTCAATGCACT  Hsapiens
 362066: .....G........GTAAG...............................  Ptroglodytes
 380148: ...............----...............................  Ggorilla
 411537: ....TG.........TAAG...........GC..................  Pabelli
 333146: ....TG.........TAAG............C.....T............  Nleucogenys
 408728: ....TG.......G.TAAG...............................  Mmulatta
 359355: ....TG.......G.TAAG............C..................  Panubis
 395382: G...TG.......G.TAAG...                              Cjacchus
 395639:                       .........C.................A  Cjacchus
1042439: ...GTG.......G.TAAGC..                              Sboliviensis
1042699:                       .........C.................A  Sboliviensis

                   42385     42395     42405     42415     42425
                       :         :         :         :         :
  42376: AGAATTAGTGTATTACAAATATATATACTAAAGCACTTGAAGTAAACACA  Hsapiens
 362116: ............................A.....................  Ptroglodytes
 380194: ............................A.....................  Ggorilla
 411587: ............................A.....................  Pabelli
 333196: ............................A.....................  Nleucogenys
 408778: .C....................G.....A...A.....C...........  Mmulatta
 359405: .C......C.............G.....A.....................  Panubis
 395667: .---------------------......A.TGA.....A...........  Cjacchus
1042727: .---------------------......A.T.......A...........  Sboliviensis

                   42435     42445     42455     42465     42475
                       :         :         :         :         :
  42426: TATCTGAAAATCAAGATTCATAAAATTATGCCAGGAATTGCTTTGTAAAT  Hsapiens
 362166: ........................T.........................  Ptroglodytes
 380244: ..................................................  Ggorilla
 411637: .........G...G.C..................................  Pabelli
 333246: ................G...C.............................  Nleucogenys
 408828: ...........................................G......  Mmulatta
 359455: ............................C..............G......  Panubis
 395696: ........T....T............................C.A...C.  Cjacchus
1042756: ........T.........................................  Sboliviensis
```

```
                   42485     42495     42505     42515     42525
                       :         :         :         :         :
  42476: AAAACTGATCAAAATTAAACATCCAGCCCTCTTGGTGCATTTTGGTACAT  Hsapiens
 362216: .................................................C  Ptroglodytes
 380294: ..................................................  Ggorilla
 411687: ............................T......A..............  Pabelli
 333296: ..................C...............................  Nleucogenys
 408878: ............................G................C.T..  Mmulatta
 359505: ....................................A..........T..  Panubis
 395746: ...-...................-......G...................  Cjacchus
1042806: ...-...................T......G......A............  Sboliviensis

                             42544               42555     42565
                                 :                   :         :
  42526: TGCAATTTA-TAAAACCTTTTTGGAATGG---------AATCTGTTATAC  Hsapiens
 362266: .........-...................---------............  Ptroglodytes
 380344: .........-......T...........T---------............  Ggorilla
 411737: .........-..................T---------............  Pabelli
 333346: .........-........G........AT---------............  Nleucogenys
 408928: ...G.....-................G.T---------............  Mmulatta
 359555: .........-................G.T---------............  Panubis
 395794: ......G..T...C............C.TAGAATTGTA..AT.C..T...  Cjacchus
1042855: ....G....-...C..............T---------.....A..T...  Sboliviensis

                   42575     42585     42595     42605     42615
                       :         :         :         :         :
  42566: AATATGAAACATAGGAATATCTGAACATCAGCTTTAAGCTTCATGAAGGT  Hsapiens
 362306: ..................................................  Ptroglodytes
 380384: ..................................................  Ggorilla
 411777: ..................................................  Pabelli
 333386: ...........................A......................  Nleucogenys
 408968: ...........C......................................  Mmulatta
 359595: ...........C.......G..............................  Panubis
 395844: ...TC....................AGA..A...................  Cjacchus
1042895: ...T....T.................GA..A...................  Sboliviensis

                   42625     42635     42645     42655     42665
                       :         :         :         :         :
  42616: TTTAATTTTCATTGAAATTCTAGGCCTAACACAATCCTAGCATGTGATAG  Hsapiens
 362356: ..................................................  Ptroglodytes
 380434: ..........G..................T....................  Ggorilla
 411827: ..................................................  Pabelli
 333436: .................................G................  Nleucogenys
 409018: ................C.................................  Mmulatta
 359645: ....C......C....C.................................  Panubis
 395894: ................C.....C.....--..................G.  Cjacchus
1042945: ................C...............................G.  Sboliviensis

                   42675     42685     42695     42705     42715
                       :         :         :         :         :
  42666: CAATACCTAACCTTAAGCATACAATGTATATTTTTGGATGAAACAGTAAA  Hsapiens
 362406: ..........A.......G...............................  Ptroglodytes
 380484: ..................................................  Ggorilla
 411877: ..................G...............................  Pabelli
 333486: ..........A.......G........................G......  Nleucogenys
 409068: .................TG........G.........T..G.........  Mmulatta
 359695: .................TG...C..............T............  Panubis
 395942: .....T.....T.....TG....G............A..T...TG.....  Cjacchus
1042995: .....T.....T.....TG.................A......TG.....  Sboliviensis
```

```
              42725     42735     42745     42755     42765
                  :         :         :         :         :
  42716: TAAAGATATAAACAAAAGATGAAATAACATTAATATTCTACATAAATATA  Hsapiens
 362456: .................................C................  Ptroglodytes
 380534: ................T........................G........  Ggorilla
 411927: ................T.......................TG........  Pabelli
 333536: ................T...................C.............  Nleucogenys
 409118: ......C.........T..........................G......  Mmulatta
 359745: ................T.................................  Panubis
 395992: ....A...........T............G....................  Cjacchus
1043045: ....A...........T............G....................  Sboliviensis

              42775     42785     42795     42805     42815
                  :         :         :         :         :
  42766: TAACATGTGGATCCCTAATGCACACCCATGACAGACAACACTAATCATCT  Hsapiens
 362506: ..................................................  Ptroglodytes
 380584: ..................................................  Ggorilla
 411977: ..................................................  Pabelli
 333586: ..................................................  Nleucogenys
 409168: .........A.....A..............G...................  Mmulatta
 359795: .........A.....G..............G...................  Panubis
 396042: .........AC........A..T.......T....T..............  Cjacchus
1043095: .........A.........................T..............  Sboliviensis

                                 LINE2
          ------------------------------------------------>
              42825     42834                   42853     42863
                  :         :                       :         :
  42816: TTTCATTCGTGCC-AAAAAATTTTAGGCA-TTTTCTAAATGCTGGGCACT  Hsapiens
 362556: ........A....A...............-....................  Ptroglodytes
 380634: ........A....A...............-....................  Ggorilla
 412027: .............A...............-....................  Pabelli
 333636: .............A...............-.................T..  Nleucogenys
 409218: ........A....-....G.A........T.......G..T.........  Mmulatta
 359845: ........A....-....G.A........T.......G..T.........  Panubis
 396092: ........A....-.....CAA...A...-....................  Cjacchus
1043145: ...T.C..A...T-......AA...A...-....................  Sboliviensis

                                LINE2
         ------------------------------------------------->
              42873     42883     42893     42902     42912
                  :         :         :         :         :
  42864: GTTCTAGGCACTGTAGCACTAAATTCAGACTATAACAC-AAAACTAAACA  Hsapiens
 362605: .......................C..............-...........  Ptroglodytes
 380683: .................G.....C..............-...........  Ggorilla
 412076: ....................C..C..............A...........  Pabelli
 333685: .......................C..............-...........  Nleucogenys
 409267: .......A...............C..............-...........  Mmulatta
 359894: .......A...............C............G.-...........  Panubis
 396140: ....C.C..........C......C.............-....TG.....  Cjacchus
1043193: ....C.T............A...C.....A........-........G..  Sboliviensis
```

```
                                  LINE2
          ------------------------------------------------------>
                    42922     42932     42942     42952     42962
                        :         :         :         :         :
   42913: GACAAAAATCTCTACTGTCACAGAGTTAGTGTCTACTGGCTCAATTGTGG  Hsapiens
  362654: ..................................................  Ptroglodytes
  380732: ..................................G...............  Ggorilla
  412126: ..........C.......................................  Pabelli
  333734: .........................G....................A...  Nleucogenys
  409316: ....................................C.............  Mmulatta
  359943: ....................................C.............  Panubis
  396189: ......................................A...........  Cjacchus
 1043242: ...............................A..................  Sboliviensis

                                                     DNA
                                   ------------------------->
                    42972     42982     42992     43002     43012
                        :         :         :         :         :
   42963: GCTTTTTCAAGTAACCTAAGTTTAAGCCTCAGACTCTCAATACAGATTTC  Hsapiens
  362704: ..........................T.......................  Ptroglodytes
  380782: ..................................................  Ggorilla
  412176: ..................................................  Pabelli
  333784: .........................................G........  Nleucogenys
  409366: .................................................T  Mmulatta
  359993: .................................................T  Panubis
  396239: .......................................G...G..C...  Cjacchus
 1043292: .......................................G......C...  Sboliviensis

                                     DNA
          -------------------------------------------------->
                    43022     43032     43042     43052     43061
                        :         :         :         :         :
   43013: CCAAGCAGTCACTACCAGTGGATTAGAGTTAGCACAAGAGA-GAGGCATA  Hsapiens
  362754: .......................................C.-........  Ptroglodytes
  380832: ..................................TG...---........  Ggorilla
  412226: ........C................................T........  Pabelli
  333834: ....A...C........C.......................T........  Nleucogenys
  409416: .........G...............................T........  Mmulatta
  360043: ........CG...............................T........  Panubis
  396289: ........CT........CA.....................CA......-  Cjacchus
 1043342: ........CT........CA.....................T.....G.-  Sboliviensis

          DNA
          --->
                    43071     43081     43091     43101     43110
                        :         :         :         :         :
   43062: TTTTTACACCACTGTGCTAAGTAAATTAACAGATATGAAAAAA-AAATAC  Hsapiens
  362803: ...........................................T.T....  Ptroglodytes
  380879: ...........................................T.T....  Ggorilla
  412276: ......T.........T.............T............T.TG...  Pabelli
  333884: ...........................................T.T....  Nleucogenys
  409466: ...................G.......................T.T....  Mmulatta
  360093: ...................G.......................T.T....  Panubis
  396338: ....G....................................T.T.T...-  Cjacchus
 1043391: ....A....T.....A.........................T.TGT...-  Sboliviensis
```

```
                    43120     43130     43140     43150     43160
                        :         :         :         :         :
  43111: ACCTTTCAAAGCATTTTCTGACTTAATACAAATAATATAATGGATATATA  Hsapiens
 362853: .................A...................G............  Ptroglodytes
 380929: .....................................G.........G..  Ggorilla
 412326: .....................................G...A........  Pabelli
 333934: ............G.......................GG...A....C...  Nleucogenys
 409516: .....................................G...A....C...  Mmulatta
 360143: .....................................G...A....C...  Panubis
 396387: ---..C.T.G.T.....                                   Cjacchus
1043440: ---..C.T.G.T.....                                   Sboliviensis

                    43170     43180     43190     43200     43210
                        :         :         :         :         :
  43161: TTTTAATCTGGTCACCTATTACTAAACTTGACATCTGTCAGTTCCTCAAT  Hsapiens
 362903: ..................................................  Ptroglodytes
 380979: ..................................................  Ggorilla
 412376: .................................----.............  Pabelli
 333984: ..................................................  Nleucogenys
 409566: .G...........G....................................  Mmulatta
 360193: .G................................................  Panubis

                    43220     43230     43240     43250     43260
                        :         :         :         :         :
  43211: CAAAAAGATACTATTGAGAGCTCATCACTGTTAAGTGATATAATGGGAGA  Hsapiens
 362953: ..................................................  Ptroglodytes
 381029: ..................................................  Ggorilla
 412422: .........G...................C......AG...........G  Pabelli
 334034: ......................TG..........A...............  Nleucogenys
 409616: ..................................................  Mmulatta
 360243: ..................................................  Panubis

                                                       LINE1
                                                  ------------->
                    43270     43280     43290     43300     43310
                        :         :         :         :         :
  43261: AAAATATATTAGAAAGCATTGTCCTTATGCTAAAAGAAATCTAAAATAAT  Hsapiens
 363003: .................G.....T..........................  Ptroglodytes
 381079: .......................T..........................  Ggorilla
 412472: C..............A.......T..........................  Pabelli
 334084: C..............A.......T..........................  Nleucogenys
 409666: C..............A....T..T..G.......................  Mmulatta
 360293: C..............A....T..T..........................  Panubis

                                  LINE1
         ------------------------------------------------->
                    43320     43330     43340     43350     43360
                        :         :         :         :         :
  43311: ATAAAGCTAAACTAGAATTATTAAGTGAATTCAGCAATGTTAATATACAA  Hsapiens
 363053: ..................................................  Ptroglodytes
 381129: ..................................................  Ggorilla
 412522: ..................................................  Pabelli
 334134: ..................................................  Nleucogenys
 409716: .......................................C..........  Mmulatta
 360343: ..................................................  Panubis
```

```
                               LINE1
         ------------------------------------------------------------>
                 43370     43380     43390     43400     43410
                     :         :         :         :         :
 43361: AAGTAAAGTATATACTTATACATCAACAACAAATAAACCAAAATAATAGA  Hsapiens
363103: ..................................................  Ptroglodytes
381179: ..................................................  Ggorilla
412572: ..................................................  Pabelli
334184: ..................................................  Nleucogenys
409766: ......................................T...........  Mmulatta
360393: ..................................................  Panubis

                               LINE1
         ------------------------------------------------------------>
                 43420     43430     43440     43450     43460
                     :         :         :         :         :
 43411: TACCAGCTAAAATTCACCAAACACCTCAAATACCACCTAGGATCAGTTTA  Hsapiens
363153: .........................................G........  Ptroglodytes
381229: .........................................G........  Ggorilla
412622: ..................................................  Pabelli
334234: ..................................................  Nleucogenys
409816: ........................................A.........  Mmulatta
360443: ........................................A.........  Panubis

                               LINE1
         ------------------------------------------------------------>
                 43470     43480     43490     43500     43510
                     :         :         :         :         :
 43461: TCAAAAAATGTACAAAACTTCAACATAGGAAAATACAAAATGTTATCAAA  Hsapiens
363203: ............T.................................TG..  Ptroglodytes
381279: ..............................................TG..  Ggorilla
412672: ..............................................TG..  Pabelli
334284: .........A...................................CTG..  Nleucogenys
409866: ..........C..................................C....  Mmulatta
360493: ..........C...............................C..CT...  Panubis

                               LINE1
         ------------------------------------------------------------>
                 43520     43530     43540     43550     43560
                     :         :         :         :         :
 43511: AGAAAAAATTAAGGAAGACCTGTATAGAGGTAAATTTCATGGTCATGGAT  Hsapiens
363253: ...................T..............................  Ptroglodytes
381329: ............................................G.....  Ggorilla
412722: ..................................................  Pabelli
334334: ............A.....................................  Nleucogenys
409916: ............A........A................G...........  Mmulatta
360543: ............A...............................C.....  Panubis

                               LINE1
         ------------------------------------------------------------>
                 43570     43580     43590     43600     43610
                     :         :         :         :         :
 43561: TAGAATTTGAATAAAGATGTATTTTCTCTCCAAATCTATAGATTCTATAT  Hsapiens
363303: .............G...............T....................  Ptroglodytes
381379: .............G...............T....................  Ggorilla
412772: ..............................................G...  Pabelli
334384: .....C.....A....................................G.  Nleucogenys
409966: ....G...........................................G.  Mmulatta
360593: ....G...........................................G.  Panubis
```

```
                              LINE1
         ------------------------------------------------------>
                  43620     43630     43640     43650     43660
                      :         :         :         :         :
  43611: AAATCCTATTAACATTTCAACTGATATATTCTATGGAAGTTAACAAGCTA  Hsapiens
 363353: ................................G.................  Ptroglodytes
 381429: ....................T...........G........G........  Ggorilla
 412822: ...................G........................-.....  Pabelli
 334434: ............A..............GA..C......T...........  Nleucogenys
 410016: ............A...G.........C...................A...  Mmulatta
 360643: ............A.............C.......................  Panubis

                              LINE1
         ------------------------------------------------------>
                  43670     43680     43690     43700     43710
                      :         :         :         :         :
  43661: ATTTTAAAACGTATATGGAAATACAAAGGATCAAGATTTGGCAAGGCAAC  Hsapiens
 363403: .........................C........................  Ptroglodytes
 381479: .........................C........................  Ggorilla
 412871: .........T..............G..A......................  Pabelli
 334484: .........T...........C..G..........-C.............  Nleucogenys
 410066: .........T.......A......G.........................  Mmulatta
 360693: .........T..............G.........................  Panubis

                              LINE1
         ------------------------------------------------------>
                            43729     43739     43749     43759
                                :         :         :         :
  43711: CCTAGAAGA-GATCAACATTGGAGGGATTACACTTCCAGCTTCCAAGAGT  Hsapiens
 363453: .........-........................................  Ptroglodytes
 381529: .........-........................................  Ggorilla
 412921: .........-........................................  Pabelli
 334533: .........G......................................C.  Nleucogenys
 410116: .........G....................T.................C.  Mmulatta
 360743: .........G....................T.................C.  Panubis

                              LINE1
         ------------------------------------------------------>
                  43769     43779     43789     43799     43809
                      :         :         :         :         :
  43760: TATTATAAAGCTACTATAATTGTGGCATCATCATGAGAATAAACAAATTG  Hsapiens
 363502: ..................................................  Ptroglodytes
 381578: ..........G...........................G...........  Ggorilla
 412970: ..................................................  Pabelli
 334583: ..................................................  Nleucogenys
 410166: ..................................................  Mmulatta
 360793: ................A.......A.........................  Panubis

                              LINE1
         ------------------------------------------------------>
                  43819     43829     43839     43849     43859
                      :         :         :         :         :
  43810: ACGAATGAAAGAGAATGGAAAACCTAGGAGCAGATTCACACACATATGGT  Hsapiens
 363552: ..........................................T.......  Ptroglodytes
 381628: ..........................................T.......  Ggorilla
 413020: ........................C.................T.......  Pabelli
 334633: ...............C........C..A..............T.C.....  Nleucogenys
 410216: ........................C.................G.......  Mmulatta
 360843: ........................C.................G.......  Panubis
```

```
                               LINE1
        ------------------------------------------------->
                  43869     43879     43889     43899     43909
                      :         :         :         :         :
 43860: CATCTAATTTATGATAAAATTTACATTGCAGTGCAATAATGAAAGGATTC  Hsapiens
363602: ..................................................  Ptroglodytes
381678: ..................................................  Ggorilla
413070: .................................T................  Pabelli
334683: .................C...............T...........A....  Nleucogenys
410266: .................................T................  Mmulatta
360893: .................................T................  Panubis

                               LINE1
        ------------------------------------------------->
                  43919     43928     43938     43948     43958
                      :         :         :         :         :
 43910: TTTGTTCAATAAGTA-GTGCCATAGCAATTAGACATCCACATGGAATGAA  Hsapiens
363652: ..........C....-..................................  Ptroglodytes
381728: ...............-..................................  Ggorilla
413120: ...A...........-..................................  Pabelli
334733: ...A...........TA.................................  Nleucogenys
410316: ...A........A..-A.......TA........G.....G.........  Mmulatta
360943: ...A........A..-A.......TA..............G.........  Panubis

          LINE1                         LINE1
        ---------->                  ------------------->
                  43968     43978     43988     43998     44008
                      :         :         :         :         :
 43959: ACTAATTTTAAAATGAATCCTGACTTTAATAAAACAATAAAACTTCTAGA  Hsapiens
363701: ..................................................  Ptroglodytes
381777: ..................................................  Ggorilla
413169: ..................................................  Pabelli
334783: ......................................T...........  Nleucogenys
410365: ..................................................  Mmulatta
360992: ..................................-...............  Panubis

                               LINE1
        ------------------------------------------------->
                  44018     44028     44038     44048     44058
                      :         :         :         :         :
 44009: AAATAACATACGAAAATATTTTCACAAACTAGAAAATACTTTCACGACAA  Hsapiens
363751: ..........T..............G............T...........  Ptroglodytes
381827: .........................G............T...........  Ggorilla
413219: .........--.............TG.G..........T....G......  Pabelli
334833: ..........T.............--------------------......  Nleucogenys
410415: ..........A.............TG............T........TG.  Mmulatta
361041: ..........T.............TG............T...........  Panubis

                               LINE1
        ------------------------------------------------->
                  44068     44078     44088     44098     44108
                      :         :         :         :         :
 44059: GATTTCTTTAAAATCGTCCAAAAAATGTGTGCAAAGACACTAACTATAAA  Hsapiens
363801: ...................................A..............  Ptroglodytes
381877: ...................................A..............  Ggorilla
413267: ..................................................  Pabelli
334863: ...............A................G.................  Nleucogenys
410465: T..............A................G.................  Mmulatta
361091: T..............A..................................  Panubis
```

```
                                    LINE1
                 44118     44128     44138     44148     44158
 44109: GGAAAAGATCATAAATTTTATTCCTTTGAAATTAGAAACTTCCATTCATC  Hsapiens
363851: ..................................................  Ptroglodytes
381927: ..................................................  Ggorilla
413317: ...............................................G..  Pabelli
334913: .......................T..........................  Nleucogenys
410515: ..........................................T.......  Mmulatta
361141: ..................................................  Panubis
```

```
                                    LINE1
                 44168     44178     44188     44198     44208
 44159: TAAGACACCACTAAGAGATGGGAAAGATAAGATGCAAAATAGTAGAAGTT  Hsapiens
363901: ..................................................  Ptroglodytes
381977: ...........................................G......  Ggorilla
413367: ..........................................C..C....  Pabelli
334963: ....................A..................C..........  Nleucogenys
410565: .......T..............................T...........  Mmulatta
361191: .......T..........................................  Panubis
```

```
                                    LINE1
                 44218     44228     44238     44248     44258
 44209: ACTTGCAACGTAGGTATCCAGTATGTGCAAAGAACTCCTAAAAATTTATC  Hsapiens
363951: .........A........................................  Ptroglodytes
382027: .........A..C..................................G..  Ggorilla
413417: .........A........................................  Pabelli
335013: .........A..........................T.............  Nleucogenys
410615: .........A........................................  Mmulatta
361241: .........A........................................  Panubis
```

```
                                    LINE1
                           44274     44284     44294     44304
 44259: AG-AAAA---AAAAACAGACAAACCATTCTTACAGGTGGGCAAAAGATTT  Hsapiens
364001: ..A....---........................................  Ptroglodytes
382077: ..A....---........................................  Ggorilla
413467: ..A....---T..........................A............  Pabelli
335063: ..A...GAA-...........................A............  Nleucogenys
410665: ..A....AAG.......A................A...............  Mmulatta
361291: ..A....AAG.......A................................  Panubis
```

```
                                    LINE1
                 44314     44324     44334     44344     44354
 44305: AAACAGAAACTTCACAAAGGAGGATATGCAGCATATGAAAAATTGTTCAA  Hsapiens
364048: ................................TG................  Ptroglodytes
382124: ................................TG................  Ggorilla
413514: ..................................................  Pabelli
335112: .........................................G........  Nleucogenys
410715: ..................................C...............  Mmulatta
361341: .....................................G............  Panubis
```

```
                                    LINE1
         ------------------------------------------------->
              44364     44374     44384     44394     44404
  44355: CCATATAATTCATGAAGGAAATGCAAATTAAAATTAAAATAACTACAGTG  Hsapiens
 364098: ..................................................  Ptroglodytes
 382174: ..................................................  Ggorilla
 413564: ..........A.......................................  Pabelli
 335162: ..................................................  Nleucogenys
 410765: ....G........A....................................  Mmulatta
 361391: ....G........A....................................  Panubis
```

```
                                    LINE1
         ------------------------------------------------->
              44414     44424     44434     44444     44454
  44405: GCTAAAGTTAACAAGCCTTACACTACCAAGTGTTGGCAAGGAGGAGGGAC  Hsapiens
 364148: ..................................................  Ptroglodytes
 382224: ..................................................  Ggorilla
 413614: .T..G.............................................  Pabelli
 335212: ...............A..................................  Nleucogenys
 410815: ...............................................A..  Mmulatta
 361441: ...............................................A..  Panubis
```

```
                                    LINE1
         ------------------------------------------------->
              44464     44474     44484     44494     44504
  44455: AAATGAAATTCTCATTTCTTGTTTGGGCAAATAAATTGACACAACCACTT  Hsapiens
 364198: ..................................................  Ptroglodytes
 382274: ...C..............................................  Ggorilla
 413664: ....................................A.............  Pabelli
 335262: ..................................................  Nleucogenys
 410865: ..T...............................................  Mmulatta
 361491: ..T............................................T..  Panubis
```

```
                                    LINE1
         ------------------------------------------------->
              44514     44524     44534     44544     44554
  44505: AAGAAAATTGCTTGGCATCTACTACAATTGAATGTATATATGCCTTATGA  Hsapiens
 364248: ..................................................  Ptroglodytes
 382324: ..................................................  Ggorilla
 413714: .............................T....................  Pabelli
 335312: ..........................G......A..........-.....  Nleucogenys
 410915: ......G...........................................  Mmulatta
 361541: ......G...........................................  Panubis
```

```
                                    LINE1
         ------------------------------------------------->
              44564     44574     44584     44594     44604
  44555: CTAGCAATTCCATTCCAAGGTATACACCCAATAAAAGTGCATACATATGT  Hsapiens
 364298: .............C..................................A.  Ptroglodytes
 382374: .............C..................................A.  Ggorilla
 413764: ..................................................  Pabelli
 335361: ............................................G....C  Nleucogenys
 410965: .C...................G............................  Mmulatta
 361591: .C...................G............................  Panubis
```

```
                              LINE1
         ------------------------------------------------->
                  44614     44624     44634     44644     44654
 44605: GTACCAGGGATATGTATCAAAAATATTCACAGCAGCACTATTTATAAGAG  Hsapiens
364348: ..................................A...............  Ptroglodytes
382424: ..................................................  Ggorilla
413814: .....G.A.....................................--...  Pabelli
335411: .......A..........G...............................  Nleucogenys
411015: ...T...A..................................G.....G.  Mmulatta
361641: ...T...A.....C............................G.....G.  Panubis

                              LINE1
         ------------------------------------------------->
                  44664     44674     44684     44694     44704
 44655: ATCCAAACTAAAAATGTCCTAAGTATTTACTAACAGTAAAATGGATAAGT  Hsapiens
364398: .....G............................................  Ptroglodytes
382474: .....G............................................  Ggorilla
413862: .............................T....................  Pabelli
335461: .............GC..............T....................  Nleucogenys
411065: .............................T..............G.....  Mmulatta
361691: .............................T..............G.....  Panubis

                              LINE1
         ------------------------------------------------->
                  44714     44724     44734     44744     44754
 44705: ATATTTGGAATATTCTGAGAATGAAATGTATATTAATAAAAATGAACAAA  Hsapiens
364448: ............................C.....................  Ptroglodytes
382524: ............................C.---.................  Ggorilla
413912: ............................C.....................  Pabelli
335511: ............................C.....................  Nleucogenys
411115: ......A....................AC...C.................  Mmulatta
361741: ......A....................AC...C.................  Panubis

                              LINE1
         ------------------------------------------------->
                  44764     44774     44784               44801
 44755: CCATGCAATGTGTAACTACATGGATGACTCTCACAAAT---TATTGACCT  Hsapiens
364498: .....................................G---........A  Ptroglodytes
382571: ...........C.......T.................G---.........  Ggorilla
413962: ........CT...........T...............G---.........  Pabelli
335561: ........A............................G---.........  Nleucogenys
411165: ........C.........TG.......A.........CATA.....G...  Mmulatta
361791: ........CA........TG.......A.........CATA.....G...  Panubis

                              LINE1
         ------------------------------------------------->
                  44811     44821     44831     44841     44851
 44802: AAAAAAAACCCACATAACTTATGATTCCAATTACATGAGATTTAAAAACA  Hsapiens
364545: .................T................................  Ptroglodytes
382618: .........T.......T................................  Ggorilla
414009: ......T..........T..........................G.....  Pabelli
335608: .................T.....................T..........  Nleucogenys
411215: -T...............T................................  Mmulatta
361841: -T...............T................................  Panubis
```

```
                               LINE1
        ---------------------------------------------------->
                 44861     44871     44881     44891     44900
                     :         :         :         :        :
 44852: GGCAGAACTAATAAGTGGTAACAGAAGTCAGAGAAGCCTTAACATTT-GG  Hsapiens
364595: ...............................................-..  Ptroglodytes
382668: .....G.........................................-..  Ggorilla
414059: ...............................................C..  Pabelli
335658: ...G...........................................-..  Nleucogenys
411264: ...................................A...........G..  Mmulatta
361890: ...................................A......TG...G..  Panubis
```

```
                               LINE1
        ---------------------------------------------------->
                 44910     44920     44930     44940     44950
                     :         :         :         :         :
 44901: GAAAAAAGGGGCTTATTTCCAGAAGAGGATTTAAGGGGGACCTTGCAGTG  Hsapiens
364644: .G.................T..............................  Ptroglodytes
382717: .........TT........T.......................G......  Ggorilla
414109: A..................T..............................  Pabelli
335707: ........A.....C....T....A.........................  Nleucogenys
411314: .......A...........T...................C..........  Mmulatta
361940: .G.....A...........T...................C..........  Panubis
```

```
                               LINE1
        ---------------------------------------------------->
                 44960     44970     44980     44990     44993
                     :         :         :         :         :
 44951: CCTCTTTCTAACAACTAATGTCTTATTTTGCTGACCTGAG-------TCA  Hsapiens
364694: .........C.T............................-------...  Ptroglodytes
382767: ...........T............................-------...  Ggorilla
414159: ...........T........C................A..-------...  Pabelli
335757: ...........T............................-------...  Nleucogenys
411364: .........G.T....T.......................TAGTGAT...  Mmulatta
361990: .........G.T....T.......................TAGTGAT...  Panubis
```

```
                               LINE1
        ---------------------------------------------------->
                 45003     45013     45023     45033     45043
                     :         :         :         :         :
 44994: TGAGTATATTCACTTTGTGATAATTTATCAAATAGCACACATACAATAAT  Hsapiens
364737: ..................................................  Ptroglodytes
382810: ..................................................  Ggorilla
414202: ................................C.................  Pabelli
335800: ................................C.................  Nleucogenys
411414: ................................C.................  Mmulatta
362040: ................................C.................  Panubis
```

```
                           LINE1                   LINE1
        ----------------------------------------->  --------->
                 45053     45063     45073     45083     45093
                     :         :         :         :         :
 45044: TTACTTTTCTATATGTATGTATATTTAAATCATAAAGTTTATTTTTAAAA  Hsapiens
364787: ...........A......................................  Ptroglodytes
382860: ..........G.......................................  Ggorilla
414252: ................................................G.  Pabelli
335850: ...................C.......G......................  Nleucogenys
411464: ..................................................  Mmulatta
362090: ..................................................  Panubis
```

LINE1

```
             45103     45113     45123     45133     45143
   45094: TCAAGATTTAAGGGATGTATTAACTAATTGTGATATGTCAATGTTATTTG  Hsapiens
  364837: .............................................G....  Ptroglodytes
  382910: ...........A......G...............................  Ggorilla
  414302: ...........................C......................  Pabelli
  335900: ..................................................  Nleucogenys
  411514: ....A.............................................  Mmulatta
  362140: ..................................................  Panubis
```

LINE1

```
             45150           45167     45177     45187
   45144: GT---TCCTGATTCAAAC---AAAAAAAAAAGAGCCAACTGGGGAAAAAT  Hsapiens
  364887: ..---.............-----...........................  Ptroglodytes
  382960: ..---.............----............................  Ggorilla
  414352: ..---.............----C...........................  Pabelli
  335950: ..---......-......--A.............................  Nleucogenys
  411564: ..TCC.............AAG..........A.......A..........  Mmulatta
  362190: ..---.............AAG..................A..........  Panubis
```

LINE1

```
             45197     45207     45217     45227     45237
   45188: GAAAACACACTGAATATTTAACATTAAAGGATTATTTTTAATTTTTCAGA  Hsapiens
  364929: ..................................................  Ptroglodytes
  383003: ............................................C.....  Ggorilla
  414395: ..................................................  Pabelli
  335994: ................G....T.......T....................  Nleucogenys
  411614: ........................G...T.....................  Mmulatta
  362237: ........................G...T..C..................  Panubis
```

LINE1

```
             45246     45256     45266               45285
   45238: AGTAATGA-ATTGTAGCGGTTGTATTAAAATTGTCCATA-TTTTTAGAAA  Hsapiens
  364979: ........G.......T......................-..........  Ptroglodytes
  383053: ........G..............................-..........  Ggorilla
  414445: T.....A.T.......T......................C..........  Pabelli
  336044: T.....A.T.......TT.....................T..........  Nleucogenys
  411664: T.C...A.T.G.....T......................T..........  Mmulatta
  362287: T.....A.T.G.....T......................T.......G..  Panubis
  396917:                                         ....G.....  Cjacchus
 1043972:                                         ....G.....  Sboliviensis
```

LINE1

```
             45295     45305     45315     45325     45335
   45286: AACAAAGTGAAATATGTGAAGGAAAATGATAGAATATCTAGAATTTGCTG  Hsapiens
  365028: ...............A..T...............................  Ptroglodytes
  383102: ..............GA..T.........G.....................  Ggorilla
  414495: ..................T............T..................  Pabelli
  336094: ......A.......CA..T............T..................  Nleucogenys
  411714: ..................T............T..................  Mmulatta
  362337: ..................T............T..................  Panubis
  396927: ..................T..........G.T.G............A...  Cjacchus
 1043982: ..................T............T...G..........A...  Sboliviensis
```

LINE1

```
            45345     45355     45365     45375     45385
   45336: CAATATAATCTTAAAGAAGTGTTGGAGAAAGGGTGAGAGATTACATGAAT  Hsapiens
  365078: ..................................................  Ptroglodytes
  383152: ..............................T...................  Ggorilla
  414545: ..............................-...................  Pabelli
  336144: ..................................................  Nleucogenys
  411764: .................G................................  Mmulatta
  362387: .................G..A.............................  Panubis
  396977: ....G.........G..G................................  Cjacchus
 1044032: .................G................................  Sboliviensis
```

LINE1

```
            45395     45405     45415                45431
   45386: AAAGCCAGCCATTAGTGAGAAATGTTGCAATTGAGT----GATGGATAAA  Hsapiens
  365128: ....................................----..........  Ptroglodytes
  383202: ....................................------C.......  Ggorilla
  414594: ..G.................................----.....G....  Pabelli
  336194: ..G.................................----.....G....  Nleucogenys
  411814: ..G.................................----.....G..T.  Mmulatta
  362437: ..G.............................C...----.....G..T.  Panubis
  397027: G.GA..C...C.........GC.A..A..G......GACG..C..G....  Cjacchus
 1044082: G.G.......C..........C....A..G......----.....GA...  Sboliviensis
```

LINE1

```
            45441     45451     45461     45471     45480
   45432: TAGGGTTCATTATATATTCTCTTTACCTTTGTATATCCTTAAA-TTTTTC  Hsapiens
  365174: ......................C...T................-......  Ptroglodytes
  383248: ......................C...T....C...........-......  Ggorilla
  414640: ....................G.C...T................-......  Pabelli
  336240: ......................C...T................-......  Nleucogenys
  411860: .........C............C...T................-......  Mmulatta
  362483: .........C............C...T.......................  Panubis
  397077: C...........C.........C............G..C....A......  Cjacchus
 1044128: ............C.........C........C......C....A......  Sboliviensis
```

LINE1

```
            45490     45500                          45501
   45481: CTTAACCAAATGTTTCTAAG-----------------------------A  Hsapiens
  365223: ....................-----------------------------.  Ptroglodytes
  383297: ....................-----------------------------.  Ggorilla
  414689: ...................A-----------------------------.  Pabelli
  336289: ...........A........-----------------------------.  Nleucogenys
  411909: ..C................A-----------------------------T  Mmulatta
  362532: ...................A-----------------------------T  Panubis
  397127: ...................ATTTTAAAAAATAAATATATCCTTCGTAAC.  Cjacchus
 1044178: ...........A.......ATTTAAAAAAATAAATATATCCTTGGTAAC.  Sboliviensis
```

```
                                                 Simple
                                             45504     45514
   45502: AA-------------------------------------CATTTAAGAAA  Hsapiens
  365244: ..-------------------------------------...........  Ptroglodytes
  383318: ..-------------------------------------...........  Ggorilla
  414710: ..-------------------------------------...........  Pabelli
  336310: ..-------------------------------------...........  Nleucogenys
  411930: ..AAATGAATATATCCTTGGTACCAAATATTAAATGTGC...........  Mmulatta
  362553: ..AAATGAATATATCCTTGGTACCAAATATTAAATGTGC...........  Panubis
  397177: ..TATATAGTGTGC-------------------------...........  Cjacchus
 1044228: ..TATTTAACGTGT-------------------------...........  Sboliviensis
```

```
                                      Simple
                               ---------------------------->
               45524     45534     45543     45553     45563
   45515: TGTTTAGCAATATATATGGGT-TTTTAATTGTGAAAAGTAAATTTAAATT  Hsapiens
  365257: .A...................-............................  Ptroglodytes
  383331: .A...................-............................  Ggorilla
  414723: .A...................-............................  Pabelli
  336323: .A......T............-............................  Nleucogenys
  411980: .A................A..-............................  Mmulatta
  362603: .A...................-............................  Panubis
  397202: .A....T..........A...G............................  Cjacchus
 1044253: .A....T..............G............................  Sboliviensis
```

```
               Simple
        --------------->
               45573     45583     45593     45603     45613
   45564: TAATTAATTTTAATTGCAGAATGTGCCACACATTTATGAAACAGAAATGG  Hsapiens
  365306: .......................CA................T........  Ptroglodytes
  383380: .......................CA................T......A.  Ggorilla
  414772: .......................CA................T.C......  Pabelli
  336372: .......................CA................T........  Nleucogenys
  412029: .......................CA...T............T........  Mmulatta
  362652: .......................CA...T............T........  Panubis
  397252: .......................GA.........................  Cjacchus
 1044303: .......................GA......G..................  Sboliviensis
```

```
               45623     45633     45643     45653     45663
   45614: TGTTCATTCGCAGAGTCACTTTGTATAGCTTCTAGAACAACTGATTGTAA  Hsapiens
  365356: ..................................................  Ptroglodytes
  383430: .........A........................................  Ggorilla
  414822: ..................................................  Pabelli
  336422: ........T...A.............................A.......  Nleucogenys
  412079: .........A.................TA..T..................  Mmulatta
  362702: .........A........A........TA.....................  Panubis
  397302: C.....C..T..A...............A..A.......C..........  Cjacchus
 1044353: ......C..T..A...............A..A.......G..........  Sboliviensis
```

```
               45673     45683     45693     45703     45713
   45664: GGGGTATTCCAAAATATGGCCCCATTCCTTTCTTGCTGATCTGCCTATGT  Hsapiens
  365406: -..................T..............................  Ptroglodytes
  383480: -..................T..............................  Ggorilla
  414872: ................C.A...............................  Pabelli
  336472: ................C.................................  Nleucogenys
  412129: A.................................................  Mmulatta
  362752: A.................................................  Panubis
  397352: ....G...TT............-............A..........GC..  Cjacchus
 1044403: ...A....TT............-............A....T......CA.  Sboliviensis
```

```
              45723     45732     45742     45752     45762
  45714: CAGGTGCAGA-CTTCAGCCAGCTTCCTCTTTAGCAAGTGGACTCAGGTTG   Hsapiens
 365455: ..........-.................G........C............   Ptroglodytes
 383529: .........G-................TG........C............   Ggorilla
 414922: ..........-.................A.....................   Pabelli
 336522: ..........-.................A........C...T........   Nleucogenys
 412179: .......G..-.................G...............G.A...   Mmulatta
 362802: .......G..-......T..........G...............G.A...   Panubis
 397401: .....A.G.GG.................G........CCA........A.   Cjacchus
1044452: .....A.G.GA.........A.......G...A....CCA........A.   Sboliviensis

              45772               45788     45798     45808
  45763: CTTATCAACACAACACAGA----GTTTAGAGACACATATTGACTTATCCT   Hsapiens
 365504: ...................----...........TG..............   Ptroglodytes
 383578: ...................----............G..............   Ggorilla
 414971: ..........T........----......................T....   Pabelli
 336571: ...................----...........................   Nleucogenys
 412228: ........T..........----A...........C..............   Mmulatta
 362851: ........T..........----A...........C..............   Panubis
 397451: ...................CAGA.A..G......................   Cjacchus
1044502: ...................CAGA.A..G......................   Sboliviensis

              45818     45828     45838     45848     45858
  45809: TACCATTAGTTTTATTTTCAACCCAAGCATTAATCGTTTTCCTCGTTTCT   Hsapiens
 365550: ..................................................   Ptroglodytes
 383624: ..................................................   Ggorilla
 415017: ..................................................   Pabelli
 336617: ..................................T...............   Nleucogenys
 412274: .............G....................T...............   Mmulatta
 362897: .............G....................T...............   Panubis
 397501: ..................................T...............   Cjacchus
1044552: ..............C...................T...............   Sboliviensis

              45868     45878     45888     45898     45908
  45859: TCTGTAGACTGTTCAAAATCCACAGTTTGCAACCTGGCTTGATACCATTT   Hsapiens
 365600: ...............................G..................   Ptroglodytes
 383674: ..................................................   Ggorilla
 415067: ..................................................   Pabelli
 336667: ..................................................   Nleucogenys
 412324: .............................T....................   Mmulatta
 362947: .........A........................................   Panubis
 397551: ..................................................   Cjacchus
1044602: ..................................................   Sboliviensis

              45918     45928     45938     45948     45958
  45909: CTCAGAACAGCTTAAATATTGCTATGAAAGACATGATAATTGAACAACAA   Hsapiens
 365650: .................................................T   Ptroglodytes
 383724: .................................................T   Ggorilla
 415117: .........................A........................   Pabelli
 336717: .........................A........................   Nleucogenys
 412374: .........................A........................   Mmulatta
 362997: .........................A........................   Panubis
 397601: .......................G.A.....A..............G...   Cjacchus
1044652: .........A.............G.......A..............G...   Sboliviensis
```

```
              45968     45978     45988     45998     46008
                  :         :         :         :         :
  45959: TTCAAAAGCAGTGTAAGGAAAACAAAAATCTACAGCTCAATGTATTCTAA   Hsapiens
 365700: ..................................................   Ptroglodytes
 383774: ..................................................   Ggorilla
 415167: ...........C......................................   Pabelli
 336767: ..................................A...............   Nleucogenys
 412424: .......A...............-........T....TG...........   Mmulatta
 363047: .......................-........T.....G...........   Panubis
 397651: .......A..........................T..TG....G......   Cjacchus
1044702: .......A........A.................T..TT....G......   Sboliviensis

              46018     46028     46038     46048     46058
                  :         :         :         :         :
  46009: AATTCTAATGCAGAGAAAAAAGCAGTTCCCATAAACAAGTCTGAGAGGAA   Hsapiens
 365750: .............................T....................   Ptroglodytes
 383824: .............................T....................   Ggorilla
 415217: .............................TG........C..........   Pabelli
 336817: .............................T...............C....   Nleucogenys
 412473: .............................T....................   Mmulatta
 363096: .............................T....................   Panubis
 397701: .T.............GC..........T.T.C.........-...G....   Cjacchus
1044752: ................C............T............T.......   Sboliviensis

              46068     46078     46088     46098     46108
                  :         :         :         :         :
  46059: ACAGGATCCATATGTACTTATTTTTAATAAAACAAATAAATTTAACAAAA   Hsapiens
 365800: G.................................................   Ptroglodytes
 383874: G.................................................   Ggorilla
 415267: G...A...T.........................................   Pabelli
 336867: G...A.............................................   Nleucogenys
 412523: G...A..G....................G...T.................   Mmulatta
 363146: G...A..GTG..................G...T.................   Panubis
 397750: G........G...A....................................   Cjacchus
1044802: T...A.....A.....T.......A.........................   Sboliviensis

              46118     46128     46138     46148     46158
                  :         :         :         :         :
  46109: TCTGAAACCCTTCCAAAAGGAATGTTCAGAAATGTTTTGTTGTTACTTCT   Hsapiens
 365850: ..................................................   Ptroglodytes
 383924: ..................................................   Ggorilla
 415317: ........................................---.......   Pabelli
 336917: ..................................................   Nleucogenys
 412573: ..G...............-...............................   Mmulatta
 363196: ..........................................G.......   Panubis
 397800: ..CA....TT...T.............G....CC................   Cjacchus
1044852: ..CA....T....T.........T.G......CC................   Sboliviensis

              46168     46178     46188     46198     46208
                  :         :         :         :         :
  46159: GTCATATGATTGGCTTTCTTAGTTTTAAAGTGTTCAAAGCAAAATGACAA   Hsapiens
 365900: ..................................................   Ptroglodytes
 383974: ..................................................   Ggorilla
 415364: ..................................................   Pabelli
 336967: .....................A.......................A....   Nleucogenys
 412622: ....C..........G...........G......................   Mmulatta
 363246: ...............G...........................          Panubis
 397850: ..........C.........G...C.........................   Cjacchus
1044902: ....A.....A.......................A...............   Sboliviensis
```

```
                                                                  MIR
                                                              ------->
                  46218     46227     46237     46247     46257
                      :        :         :         :         :
   46209: ATTGCAATAC-ATGAGCACCTTCTGACTCTATTCCTACTCTTTCCTCTTC  Hsapiens
  365950: ..........-.......................................  Ptroglodytes
  384024: ..........-.......................................  Ggorilla
  415414: ..........-.......................T...............  Pabelli
  337017: ..........-.......................................  Nleucogenys
  412672: ..........A......................A................  Mmulatta
  397900: .C........A.G.....................................  Cjacchus
 1044952: .C..T.....A.G......T..............................  Sboliviensis

                                  MIR
          ------------------------------------------------->
                  46267     46277     46287     46297     46307
                      :         :         :         :         :
   46258: AAAGTAGACATGGAAGTTACTGCTCTCAGATTATAGACAGGAAAACTGAG  Hsapiens
  365999: .....................TG..........................A  Ptroglodytes
  384073: ..................................................  Ggorilla
  415463: ..................................................  Pabelli
  337066: .........................A........................  Nleucogenys
  412722: ................C..................A..............  Mmulatta
  363340:                  ....................TG...........  Panubis
  397950: ....C........G...............G...C................  Cjacchus
 1045002: ....C........G...............G....................  Sboliviensis

                              MIR
          ---------------------------------------->
                  46317     46327     46337     46347     46357
                      :         :         :         :         :
   46308: AAGCAGAGTGGTTAAGTGACTTGCTTTTCTGAGGTCTAACAGAAGGTTGC  Hsapiens
  366049: ........C....................C....................  Ptroglodytes
  384123: ..................................................  Ggorilla
  415513: ...................................T..............  Pabelli
  337116: .........................G..................A..C..  Nleucogenys
  412772: .---.......................C.....................T  Mmulatta
  363373: .---..G....................C...............G.....T  Panubis
  398000: ...TC....A...C..........C........A..............T.  Cjacchus
 1045052: ...T...A.....C..................................T.  Sboliviensis

                  46367     46377     46387     46397     46407
                      :         :         :         :         :
   46358: TCCCATGATATATATCAATCTCCTCACAAGCTGCCATAAATATAATTAAT  Hsapiens
  366099: .T................................................  Ptroglodytes
  384173: ..................................................  Ggorilla
  415563: .......G..........................................  Pabelli
  337166: ..................................................  Nleucogenys
  412819: ...........G......................................  Mmulatta
  363420: ...........G......................................  Panubis
  398050: ..........................A.....A.........G.......  Cjacchus
 1045102: ........C.......................A.A.......G.......  Sboliviensis

                  46417     46427     46437     46447     46457
                      :         :         :         :         :
   46408: AATAAAAACATTCTGTGATGTAGATAGTTGGCATTTCTGTCAAATTTATG  Hsapiens
  366149: ..................................................  Ptroglodytes
  384223: .................T................................  Ggorilla
  415613: ..............C.................................C.  Pabelli
  337216: ..............C..........G........................  Nleucogenys
  412869: ..............C.......A..........................A  Mmulatta
  363470: ..............C.......A..........................A  Panubis
  398100: ...........GA.C...........C..................G....  Cjacchus
 1045152: ..G....T...GA.C.....A.A...C.......................  Sboliviensis
```

```
                46467     46477     46486     46496     46506
                    :         :         :         :         :
  46458: TAAAGTGTGAACAAAAATATCTTAA-GAATTTATTATTTCGTTCACCTAT  Hsapiens
 366199: .........................-....G.........A.........  Ptroglodytes
 384273: ..........G..............-........................  Ggorilla
 415663: .........................G........................  Pabelli
 337266: .........................G..............AC....T...  Nleucogenys
 412919: ..........G..............G........................  Mmulatta
 363520: ..........G..............G..............A.........  Panubis
 398150: ...GT...........T....C...AA...G........T.........A  Cjacchus
1045202: ................G........A....G........T..........  Sboliviensis

                46516     46526     46536     46546     46556
                    :         :         :         :         :
  46507: AACATATTATCTAAGTGAAAGTGGTGTGCAAACTCAGTATAAAGAACTTA  Hsapiens
 366248: ............-.....................................  Ptroglodytes
 384322: ..................................................  Ggorilla
 415713: ..................................................  Pabelli
 337316: ..................................................  Nleucogenys
 412969: ...........C..................G...................  Mmulatta
 363570: ...........C......................................  Panubis
 398200: ...........C.........................C.....T..A...  Cjacchus
1045252: .......C...C..................................A...  Sboliviensis

                46566     46576     46586     46596     46606
                    :         :         :         :         :
  46557: CCCTTTCTTTTAACCAAAAATTTTTACTCCGGAGGCCCTCCTGTGTAGCA  Hsapiens
 366297: ...............................A..C...............  Ptroglodytes
 384372: ..............................AA..C.....T.........  Ggorilla
 415763: ......................C.......CA..C...............  Pabelli
 337366: ......................C.......CA..C......CA.......  Nleucogenys
 413019: ......................C.......CA..C.......A.....T.  Mmulatta
 363620: .T....................C.......CA..C.......A.......  Panubis
 398250: ..T...................C.......CA..C...............  Cjacchus
1045302: ..T...................C.......CA.AC...............  Sboliviensis

                46616     46626     46636     46646     46656
                    :         :         :         :         :
  46607: TTCTGGAGCAACAATTTATGACCACCCATGGCCCTAGATTTCTATAGAAC  Hsapiens
 366347: ............G...G.................................  Ptroglodytes
 384422: ............G...G...........A.....................  Ggorilla
 415813: ................G............................AG.A  Pabelli
 337416: ............T...G...........................C.....  Nleucogenys
 413069: ............T...G...........................C.....  Mmulatta
 363670: ............T...G.....G...........................  Panubis
 398300: .........C..C...G.............A..........T....C...  Cjacchus
1045352: .........C.--------------................T......G.  Sboliviensis

                46663     46673     46683     46693     46703
                    :         :         :         :         :
  46657: ---TGGAGACTTTCCGTTGTCTTTCTGCCAAGACAACAGTGATATCTACA  Hsapiens
 366397: ---............A...............................T..  Ptroglodytes
 384472: ---............A..................................  Ggorilla
 415863: GCG............A..................................  Pabelli
 337466: ---....A.......T............G.....................  Nleucogenys
 413119: ---...........T............T......................  Mmulatta
 363720: ---............A...........T......................  Panubis
 398350: ---G.....T....TA...C.......T...A..........A.......  Cjacchus
1045388: ---A.....T...AT....C.......T..............A......G  Sboliviensis
```

```
                46713     46723     46733     46743     46753
                    :         :         :         :         :
   46704: TGGACCCAAATGAGTTTACCATGATGCAATTCCTGAGACCTAAGGAGCAT  Hsapiens
  366444: ............T.....................................  Ptroglodytes
  384519: ..................................................  Ggorilla
  415913: ....                                                Pabelli
  337513: .........................................T........  Nleucogenys
  413166: .............A...........................T........  Mmulatta
  363767: .........................................T........  Panubis
  398397: ..............G..G.......T...............C.......C  Cjacchus
 1045435: ..............A..G.......T...............T........  Sboliviensis

                46763     46773     46783     46793     46803
                    :         :         :         :         :
   46754: ACCTCCACAGCCCAGTGGGACTCAATGCCCCAACATGAACTGAATAGAAC  Hsapiens
  366494: .....................C............................  Ptroglodytes
  384569: .....................C..GC........................  Ggorilla
  337563: ......G..T...........C...........................T  Nleucogenys
  413216: .....T...............C..........G................T  Mmulatta
  363817: .....................CTG.........................T  Panubis
  398447: .......T...........-.C..G..-....T........T.....G.T  Cjacchus
 1045485: .......T......C......C..G..-....T........T.....G.T  Sboliviensis

                46813     46823     46833     46843     46853
                    :         :         :         :         :
   46804: AATACAAGACTTTGAAATATGGAAGATGTGCCTAGCGTAGAAGAAAAACA  Hsapiens
  366544: ..................................................  Ptroglodytes
  384619: ....................................A.............  Ggorilla
  337613: G.............T...........................T.......  Nleucogenys
  413266: G..............G....................A.............  Mmulatta
  363867: G...........C..G....................A.............  Panubis
  398495: G..........................A..T.....A.............  Cjacchus
 1045534: G.............................T.....A.....T.......  Sboliviensis

                              LTR
                     ----------------------------------->
                46863     46873     46883     46893     46903
                    :         :         :         :         :
   46854: AAGCTTAACTAATGTTGGGGGAAGTCAGGGACCCCGAACGGAGGGACTGA  Hsapiens
  366594: .................C................T............C..  Ptroglodytes
  384669: .................CA............................C..  Ggorilla
  337663: .................C.A..................G........C..  Nleucogenys
  413316: ..T.............AT.................A........----..  Mmulatta
  363917: ..T.............AT.................A..T.....----..  Panubis
  398545: ...........C----.A.CA.....T...C.T.A.C.T.A.          Cjacchus
 1045584: ............----.A.CA.....T...C.T.A.C.T.A.          Sboliviensis

                        LTR
          ------------------------------------------------->
                46913     46923     46933     46943     46953
                    :         :         :         :         :
   46904: CCTGCTGAAGCCGTGACAGAAGAACATAAATTGTGAAGAATTCATGGGCA  Hsapiens
  366644: ..................................................  Ptroglodytes
  384719: ..................................................  Ggorilla
  337713: ............A.............................T....A..  Nleucogenys
  413362: ...........T...........................T.......A..  Mmulatta
  363963: ..........TT..T---...............A.....T.......A..  Panubis
```

```
                                    LTR
             46963     46973     46983     46993     47003
 46954: TTTATTAGTTCCTCAAATTAATACTTTTATAATTTCTTACACCTGTCTTT  Hsapiens
366694: ...G........C.....................................  Ptroglodytes
384769: ............C.....................................  Ggorilla
337763: ............C..........................TG.........  Nleucogenys
413412: ............C.............C.C....A.....TG.........  Mmulatta
364010: ............C.............C......A.....TG.........  Panubis

                                    LTR
             47013               47031     47041     47051
 47004: ATTGCAATCTCTGAACAT--AAATTGTGAAGATTTCATGGACATTTATCA  Hsapiens
366744: .C................--..............................  Ptroglodytes
384819: .C................--..............................  Ggorilla
337813: .C...............C--..............................  Nleucogenys
413462: .C................--..............................  Mmulatta
364060: .C................AA..............................  Panubis

                                    LTR
             47061     47071     47081     47091     47099
 47052: CTTCCCTAATCAATACTCTTGTGATTTTCTATGCCTGTCTT--CTTTAAT  Hsapiens
366792: ...........................C.............--.......  Ptroglodytes
384867: ...........................C.............--.......  Ggorilla
337861: ...........................C...G.........--.......  Nleucogenys
413510: .......G.......G...........C..G..........TA.......  Mmulatta
364110: .......G...................C..G..........TA.......  Panubis

                                    LTR
             47109     47119     47129     47139     47149
 47100: CTCTTAATCCCATCATCTTCGTAAGCTGAGGATGTATATCACCTCAGGAC  Hsapiens
366840: ..................................................  Ptroglodytes
384915: ...................................T..............  Ggorilla
337909: .................C...................G............  Nleucogenys
413560: ......................G..............G.TG.........  Mmulatta
364160: ....................A.G..............G.TG.........  Panubis

                                    LTR
             47159     47169     47179     47189     47199
 47150: CCTGTGATGATTGCGTTAACTGCACAAATTGTTCGTAAAGCATGTGTGTT  Hsapiens
366890: .............T....................................  Ptroglodytes
384965: .............T....................................  Ggorilla
337959: ............................C.C...A......C........  Nleucogenys
413610: .............AT.............C.....A...............  Mmulatta
364210: .....A.......AT.............C.....A...............  Panubis
```

```
                           LTR
        ------------------------------------------------->
             47209     47219     47229     47239     47249
 47200: TAAACAATATGAAATCTGGGCAACTTGAAAAAAGAACAGGATAACAGCGA  Hsapiens
366940: ..................................................  Ptroglodytes
385015: ..................................................  Ggorilla
338009: ..................................................  Nleucogenys
413660: ....T.................T...........................  Mmulatta
364260: ......................C...........................  Panubis

                           LTR
        ------------------------------------------------->
             47259     47269     47279     47289     47299
 47250: TGTTCAGGGAACTAGGGAGATAACCATTAGATCTGGCTGCCTGAGAGCCA  Hsapiens
366990: ............A.................G..G............A...  Ptroglodytes
385065: ............A.................G..................G  Ggorilla
338059: ............A.................G..................G  Nleucogenys
413710: ...........GA.A...............G.............A....G  Mmulatta
364310: ............A............T....G.............A...TG  Panubis

                           LTR
        ------------------------------------------------->
             47309     47319     47327               47340
 47300: GGCAGAACAGAGCCATATTTCTCT--TTTCAAAAAC-------AAATAGG  Hsapiens
367040: ........................TC..........-------.......  Ptroglodytes
385115: ....................N...TC..........-------.......  Ggorilla
338109: ..TG....................TC........G.-------.......  Nleucogenys
413760: ..AG......G.............TC........C.GAAAAGTG..G...  Mmulatta
364360: ..AG....................TC........C.-------G.G....  Panubis

                           LTR
        ------------------------------------------------->
             47350     47360     47370     47380     47390
 47341: AGAAATATCGTTGAATTCTTTTTCTCAGCAAGGAACAGCCCTGAGAAAGA  Hsapiens
367083: .........A........................................  Ptroglodytes
385158: ...............................A..................  Ggorilla
338152: ....G....AC.......................................  Nleucogenys
413810: .A...C....C...............G.......................  Mmulatta
364403: ........T.C........CC..T..G.......................  Panubis

                           LTR
        ------------------------------------------------->
             47400     47410     47420     47430     47440
 47391: GAATGCGTTCCTAGGGGGAGGTCTCTGAAATGGCTGCTCTGGGAATGCCT  Hsapiens
367133: ...............................................T..  Ptroglodytes
385208: ...............................................T..  Ggorilla
338202: .........G....T.A...T.............C............T..  Nleucogenys
413860: ......A...........................C............T..  Mmulatta
364453: ......A........................................T..  Panubis
```

```
                                  LTR
        ------------------------------------------------------>
                 47450     47460     47470     47480     47490
                     :         :         :         :         :
 47441: GTCTTATACAGTTGTAGATAAGGGATGAAATAAGCCCCGGTCTCCCGTAG  Hsapiens
367183: .....................................T.....T......  Ptroglodytes
385258: .........................A...........T.........C..  Ggorilla
338252: ...................................T..........A...  Nleucogenys
413910: ........TG....C.......................A...C.T.A...  Mmulatta
364503: ........TG....C.......................A...C...A...  Panubis
```

```
                                  LTR
        ------------------------------------------------------>
                 47500     47510     47520     47530     47540
                     :         :         :         :         :
 47491: CACTCCCAGGCCTATCAGGACGAGGAAATTCCTGACTAGTAAATTTTAGT  Hsapiens
367233: .G................................C...............  Ptroglodytes
385308: TG..................T.............C...............  Ggorilla
338302: .....................A.......C....C...............  Nleucogenys
413960: TG...........G......T.............C...............  Mmulatta
364553: TG...........G.T....T...........C.C...............  Panubis
```

```
                                  LTR
        ------------------------------------------------------>
                 47550     47560     47570     47580     47590
                     :         :         :         :         :
 47541: CAGACCGGTTGTCTGCTCTCAAACCCTGCCTCCTGATAAGATATTATCAA  Hsapiens
367283: .....T......................T.....CG..............  Ptroglodytes
385358: ............................T.....................  Ggorilla
338352: ......AA....................T.............G.......  Nleucogenys
414010: .....T...C..............T...T.............G.......  Mmulatta
364603: ........................TG..T.............GC......  Panubis
```

```
                                  LTR
        ------------------------------------------------------>
                 47600     47610     47620     47630     47640
                     :         :         :         :         :
 47591: TGACAATGCATGCCCGAAACTTCATTAGCAATTTTAATTTTGCCCCGATG  Hsapiens
367333: .......................G................C.........  Ptroglodytes
385408: ........................................CA......A.  Ggorilla
338402: ...........C..T.........................CA...T.G..  Nleucogenys
414060: ..G...........T.........................C......G..  Mmulatta
364653: ..G.....TG....T.........................CA.....G..  Panubis
```

```
                                  LTR
        ------------------------------------------------------>
                 47650     47660     47670     47680     47690
                     :         :         :         :         :
 47641: CTCTGCCCCCATTTGCCTTGTGATATTTTATTGCCTTGTGAAGCATGTGA  Hsapiens
367383: ........-.........................................  Ptroglodytes
385458: ............................................G.....  Ggorilla
338452: ..G...................G...........................  Nleucogenys
414110: ........T.....A...................................  Mmulatta
364703: ........T.....A.T.........................A..G....  Panubis
```

```
                                LTR
              47700     47710     47720     47730     47740
   47691: TCTCTGTGACCCACACCCTATTCGTACACTCCCTCCCCTTTGAAAATCAC  Hsapiens
  367432: ..................................................  Ptroglodytes
  385508: ......................TC..........................  Ggorilla
  338502: ...........................G......................  Nleucogenys
  414160: ..................................................  Mmulatta
  364753: ..........................T.......................  Panubis

                                LTR
              47750     47760     47770     47780     47790
   47741: TAATAAAAAATTGCTGGTTTTGCGGCTTGGGGGGCTTCATGGAACCTGCC  Hsapiens
  367482: .........................T........................  Ptroglodytes
  385558: ..................................................  Ggorilla
  338552: ...........C................---....A........A.....  Nleucogenys
  414210: .......................A....T......A...CA.-.......  Mmulatta
  364803: .......................A....T......A...CA.-.......  Panubis

                                LTR
              47800     47810     47820     47830     47840
   47791: GACATGTGATGTCTCTCCCGGGCACCCAGCTTTAAAATTTCTCTCTGTTG  Hsapiens
  367532: .............C.C..T..A........................T...  Ptroglodytes
  385608: ...............C.....A........................T...  Ggorilla
  338599: .....................A........................T...  Nleucogenys
  414259: A..............CT....A........................T...  Mmulatta
  364852: A..............CT....A........................T...  Panubis

                                LTR
              47850     47860     47870     47880     47890
   47841: TACTCTTTCCCTTTATTTCTCAGACCAGCCGACACATAGGGAAAACAGAA  Hsapiens
  367582: ..................................................  Ptroglodytes
  385658: ..................................................  Ggorilla
  338649: ..T.A...........G........TG..T...............T....  Nleucogenys
  414309: .C................A.......GC..A....T..............  Mmulatta
  364902: .C.........................C..A....T..............  Panubis

                           LTR
              47900     47910     47919     47929     47939
   47891: AAGAACCTACATTGAATTATCA-GGGCGGGTTCCCCCAATATACTATGAG  Hsapiens
  367632: ............A........G-....A.............A....A...  Ptroglodytes
  385708: ................A....G-...T..........G...A....A...  Ggorilla
  338699: .........TG...........G..............G...A....A...  Nleucogenys
  414359: .....T...TG...........G...T.......T.TG...A........  Mmulatta
  364952: .........TG..........GG...T.......T.TG...A....C...  Panubis
  398552:                                           ....C...  Cjacchus
 1045591:                                           ....A...  Sboliviensis
```

```
              47949     47959     47969     47979     47989
                  :         :         :         :         :
  47940: CAAAATCTGGGCCTCAGAATGAATCTTGTCCTGAGCTGGATCCAATTGTG  Hsapiens
 367681: ...........................C......................  Ptroglodytes
 385757: ..................................................  Ggorilla
 338749: ..C.G...................T..A......................  Nleucogenys
 414409: .............................................C....  Mmulatta
 365002: .............................................C....  Panubis
 398560: ....G............C................................  Cjacchus
1045599: ....G............C................................  Sboliviensis

              47999     48009     48019     48029     48039
                  :         :         :         :         :
  47990: GTTGAAACAACCTTAAGTAACTCTCCATAGCCCAGGAGATGGACTTTTTC  Hsapiens
 367731: ..................................................  Ptroglodytes
 385807: ..................................................  Ggorilla
 338799: ............G..............................TG.....  Nleucogenys
 414459: ...........................................TG....T  Mmulatta
 365052: .......T...................................TG....T  Panubis
 398610: A.C.....G..........................A.A...A.AA....T  Cjacchus
1045649: A........................................A..G....T  Sboliviensis

              48049     48059     48069     48079     48089
                  :         :         :         :         :
  48040: CCAGCCAGAATCCTGAAATAACAAGGTCCCTCTCATGACATAGTTCAAGA  Hsapiens
 367781: ..................................................  Ptroglodytes
 385857: ..................................................  Ggorilla
 338849: ..................................................  Nleucogenys
 414509: ..................................................  Mmulatta
 365102: ..................................................  Panubis
 398660: ............................T.....................  Cjacchus
1045699: .................G..........T.......T.............  Sboliviensis

              48099     48109     48119     48129     48139
                  :         :         :         :         :
  48090: CAATCTTCATGATGCTAATGAGGACCTTCTCACTTCTGGAACAGATGTCA  Hsapiens
 367831: .....................T.---........................  Ptroglodytes
 385907: ..............................T...................  Ggorilla
 338899: ..................................................  Nleucogenys
 414559: ....G...............................A..........C..  Mmulatta
 365152: ....T...............................A..........C..  Panubis
 398710: .......T......TG..............G....TA......A...C..  Cjacchus
1045749: ..............TG..............G.....AA.........G..  Sboliviensis

              48149     48159     48169     48179     48189
                  :         :         :         :         :
  48140: TCATGATCATCCCAAATCCACATTTGTCTACTGTTATATCTGGGTCACTG  Hsapiens
 367878: .........................A...........G............  Ptroglodytes
 385957: .........................A...........G............  Ggorilla
 417214:               ..A.......--A...-.....-G.........T..  Pabelli
 338949: .........................A..A....G...G............  Nleucogenys
 414609: .A.......................A...........G.G..........  Mmulatta
 365202: .A.......................A...........G............  Panubis
 398760: ..................T......A...........G............  Cjacchus
1045799: .........................AA..........G.....T......  Sboliviensis
```

```
                  48199     48209     48219     48229     48239
                      :         :         :         :         :
  48190: TTTTGAGAAGGCAAAGTCAAGAGAGCGGGCATACCAGCTCAAAGGACCGT  Hsapiens
 367928: .........................T......C...............A.  Ptroglodytes
 386007: ................................C...............A.  Ggorilla
 417246: ...................-............CG.....TG.......A.  Pabelli
 338999: ...................T............C...............A.  Nleucogenys
 414659: ...................T.....G.A.T..C...A...G.......A.  Mmulatta
 365252: ...................C......A.....C...A...G.......A.  Panubis
 398810: ...................T.....AA....CCT..A......A....A.  Cjacchus
1045849: ......A............C.....AA....CC...A...G..A....A.  Sboliviensis

                  48249     48259     48269     48279     48289
                      :         :         :         :         :
  48240: GATTGTTAATCCCTGCCACTCTAGACACTCTCCATACATCGTTCCCTATA  Hsapiens
 367978: ........................................A.........  Ptroglodytes
 386057: ........................................A.........  Ggorilla
 417295: ...C.........C..........................A........G  Pabelli
 339049: ........................................A.........  Nleucogenys
 414709: ........................................A.........  Mmulatta
 365302: ........................................A.........  Panubis
 398860: ..........T..CA..C.........T......C.A...A.......--  Cjacchus
1045899: .............CA...............C...C.A...A.........  Sboliviensis

                  48299                                   48304
                      :                                       :
  48290: TGCTTCCAGGGCA-----------------------------------GT  Hsapiens
 368028: .............-----------------------------------..  Ptroglodytes
 386107: .............-----------------------------------..  Ggorilla
 417345: ......T......-----------------------------------..  Pabelli
 339099: .............-----------------------------------..  Nleucogenys
 414759: .............-----------------------------------..  Mmulatta
 365352: .............GTAAAATAAATCATTCCCTATATGCTTCCAGGGCG..  Panubis
 398908: ..T..A...A...-----------------------------------..  Cjacchus
1045949: ..T..A...A...-----------------------------------..  Sboliviensis

                  48314     48324     48334     48344     48354
                      :         :         :         :         :
  48305: AAAAGAAAATACTCTCTGAATGTCTATAATACCTACCCCAAGTTGCAAAT  Hsapiens
 368043: ..................................................  Ptroglodytes
 386122: ..................................................  Ggorilla
 417360: ...........................G......................  Pabelli
 339114: ..................................................  Nleucogenys
 414774: ....T.......C....A................................  Mmulatta
 365402: ....T.......C....A................................  Panubis
 398923: ............A....A..CA........GT..........C..T....  Cjacchus
1045964: ............A........A.......................T....  Sboliviensis

                  48364     48374     48384     48394     48404
                      :         :         :         :         :
  48355: ATTCTGACTATCAGAAGCTCATCACATAGGTTATCCAAGACATAACCTGA  Hsapiens
 368093: ....................................-.............  Ptroglodytes
 386172: ................................G.................  Ggorilla
 417410: ...----.............T....-------..................  Pabelli
 339164: ...................TG....-------..................  Nleucogenys
 414824: ...T.....................-------..................  Mmulatta
 365452: ...................T.....-------..................  Panubis
 398973: .........G......A..T.G...-------..................  Cjacchus
1046014: .........G......A..T.....-------.........--..T....  Sboliviensis
```

```
                   48414     48424     48434     48444     48454
                       :         :         :         :         :
   48405: TCTACCAGGCCAGGGAAGAACATTAGAAACTCATGTTTTCATCCAGCTTC  Hsapiens
  368142: ...................................-..............  Ptroglodytes
  386222: ....TG.............................G..............  Ggorilla
  417449: ...................................G..............  Pabelli
  339207: ...................................G..............  Nleucogenys
  414867: ...................................G..............  Mmulatta
  365495: .............A.....................G..............  Panubis
  399016: .AAG.........................T.....G..............  Cjacchus
 1046055: .TAG......A...C........G.C...T.....A..............  Sboliviensis

                   48464     48474     48484     48494     48504
                       :         :         :         :         :
   48455: ACCCAGATAGCATTTAAATAACCCTGGTCAAACAAAAGCTTGCATTTCAC  Hsapiens
  368191: ........................C.........................  Ptroglodytes
  386272: ..................................................  Ggorilla
  417499: ........GC.......................................T  Pabelli
  339257: ........G.........................................  Nleucogenys
  414917: ..G.....G................A........................  Mmulatta
  365545: ..G.....G................A........................  Panubis
  399066: ......C.G.................................T.......  Cjacchus
 1046105: ......C.G............T....................T.......  Sboliviensis

                                   MIR
                 ------------------------------------------->
                             48521     48531     48541     48551
                                 :         :         :         :
   48505: CTCAATCA---CAAGATGATGTAGCAATTAAAACCAGGAAGACAGATTTG  Hsapiens
  368241: ........CAA.............................A.........  Ptroglodytes
  386322: ........CAA.......................................  Ggorilla
  417549: ........CAA......................A.CT.....TG......  Pabelli
  339307: ....G...TAA...A..................A........TG......  Nleucogenys
  414967: ........TAG......C...............A........TG......  Mmulatta
  365595: ........TAG......C...............A........TG......  Panubis
  399116: ........CAG..G...................A.....G..TG......  Cjacchus
 1046155: ........CAG..G.T.................A.....G..TG.C....  Sboliviensis

                               MIR
          ------------------------------------------------->
                   48561     48571     48581     48591     48601
                       :         :         :         :         :
   48552: CATAGGGGTTAAATCCAGGTTTTGCCATTTAAATATGTCTGTGACCCTTA  Hsapiens
  368291: G...................................C.............  Ptroglodytes
  386372: G........................G..........C.............  Ggorilla
  417599: G........G........T.................C.............  Pabelli
  339357: G........C.....T..A.................C.............  Nleucogenys
  415017: G........C...................C......C........T...G  Mmulatta
  365645: G........C...................C......C.......TT...G  Panubis
  399166: G........C.........C............G...C.........T..G  Cjacchus
 1046205: G........C.........C................C.........T..G  Sboliviensis
```

```
                               MIR
         ------------------------------------------------->
              48611     48621     48631     48637     48647
   48602: TCAGCCTGACTTTTCAGAATCTGCTTCACA----GTCTGTAGAATTAATG  Hsapiens
  368341: ..............................----................  Ptroglodytes
  386422: ..............................----................  Ggorilla
  417649: ..............................----................  Pabelli
  339407: ..............................----................  Nleucogenys
  415067: ..............................----.....C..........  Mmulatta
  365695: ..............................----.....C..........  Panubis
  399216: ...AG.........................GTGT..A.............  Cjacchus
 1046255: ...A..........................GTGT..A.............  Sboliviensis

                               MIR
         ------------------------------------------------->
              48657     48667     48677     48687     48697
   48648: GAAATTTTATACCAATATCTTAGCACAGTGTGAGGATTATAGGAGATAAT  Hsapiens
  368387: .............C....................................  Ptroglodytes
  386468: .............C.............A......................  Ggorilla
  417695: .............C.............AC.....................  Pabelli
  339453: .............C.............A..................C...  Nleucogenys
  415113: ..C......C...C.............A......................  Mmulatta
  365741: ..T......C...C.............A......................  Panubis
  399266: C............C..........G..A.....................A  Cjacchus
 1046305: ............GC.............A.....................A  Sboliviensis

                               MIR
         ------------------------------------------------->
              48707     48717     48727     48737     48747
   48698: GCTTGTAATGAGCTAAGCAGAATGACTGGCACAGAGTAAATACTCAATAA  Hsapiens
  368437: ........................................C.........  Ptroglodytes
  386518: ..C...............................................  Ggorilla
  417745: ..C...............................................  Pabelli
  339503: ..C.......................................A..C....  Nleucogenys
  415163: ..C...............................................  Mmulatta
  365791: ..C...............................................  Panubis
  399316: ..C.......T.T...........................----......  Cjacchus
 1046355: ..C.......T.T...........................C.........  Sboliviensis

        MIR
         >
              48757     48767     48777     48787     48797
   48748: AACTACATAGGACCATGGTGTTGGGAAAGGGCATAAATGGTGTTAGAATA  Hsapiens
  368487: ..T.........................A.....................  Ptroglodytes
  386568: ..................................................  Ggorilla
  417795: ....G...........A.........................G.......  Pabelli
  339553: .........T..............................G.........  Nleucogenys
  415213: ...........G.......T..................A...........  Mmulatta
  365841: ...........G...C..................................  Panubis
  399362: .G............G..C......T........A................  Cjacchus
 1046405: .GG....A.........C......T..........T..............  Sboliviensis
```

```
                                                          MIR
                                                        ------>
              48807     48817     48827     48837     48847
   48798: TTGGAGCTACAAAGAACTAAGCCTAATCCTCTCATTTTAAAGACAGAAGT Hsapiens
  368537: ...........................................T...... Ptroglodytes
  386618: .................................G.........T...... Ggorilla
  417845: ...........................................T...G.. Pabelli
  339603: ...........................................T...... Nleucogenys
  415263: ...........T...............................T...... Mmulatta
  365891: ...........................................T...... Panubis
  399412: .........G.C.T..............------.C.......T.....C Cjacchus
 1046455: .........G.C.T..................T..........T...... Sboliviensis

                                   MIR
          ------------------------------------------------->
              48857     48867     48877     48887     48897
   48848: GACTTGCCCAAAGTTATATATTCTAAATGGAAACAGAACTCCTGTCTCTT Hsapiens
  368587: .................................................. Ptroglodytes
  386668: .................................................. Ggorilla
  417895: ................A................................. Pabelli
  339653: .G...............................................C Nleucogenys
  415313: .................G..............G................. Mmulatta
  365941: .................G...............G...........T.... Panubis
  399456: A................G........T................T...... Cjacchus
 1046505: .........--......G.........................T...... Sboliviensis

                         MIR
          ----------------------------->
              48907     48917     48927     48937     48947
   48898: GGCTCACAGTCCAATGTAATTTCAACTACTTCTTGATACTCCACTATTAA Hsapiens
  368637: .................................................. Ptroglodytes
  386718: .................................................. Ggorilla
  417945: .................................................. Pabelli
  339703: A................................................. Nleucogenys
  415363: .......G.......................................... Mmulatta
  365991: .......G..........................T............... Panubis
  399506: ........A.......G.C......TG......A....T........... Cjacchus
 1046553: ........A.......G.C......TG..G.................... Sboliviensis

              48957     48967     48977     48987     48997
   48948: GAACCTCTGAAAAAGATATGAAAAATTGATTTTGTTTTTCTTCTGGTCCA Hsapiens
  368687: .................................................. Ptroglodytes
  386768: .................................................. Ggorilla
  417995: ......................................C........... Pabelli
  339753: ..............................C................... Nleucogenys
  415413: .............T..............G..................... Mmulatta
  366041: .............T.........C....G..................... Panubis
  399556: ..C.T.............CA..G.......C................... Cjacchus
 1046603: ......T...........CA..G........................... Sboliviensis
```

```
                  49007     49017     49027     49037     49047
                      :         :         :         :         :
   48998: CAGGGATGATTCTTACCTACTTTGTTTTGTGCTTCATCCCTGACCTTGGT  Hsapiens
  368737: ..................................................  Ptroglodytes
  386818: ..................................................  Ggorilla
  418045: ..-...............................................  Pabelli
  339803: A.................................................  Nleucogenys
  415463: ..................................................  Mmulatta
  366091: ..................................................  Panubis
  399606: ........G............G............................  Cjacchus
 1046653: TG......G.......T..........A......................  Sboliviensis

                  49057     49067     49077     49087     49097
                      :         :         :         :         :
   49048: TCTTAGACAGCATTAGGGATCTTGAAGAAGTCATTTTTATGTGATATTTG  Hsapiens
  368787: .................................................T  Ptroglodytes
  386868: .................................................T  Ggorilla
  418094: ...----..........................................T  Pabelli
  339853: .......A.........................................T  Nleucogenys
  415513: ....G.........................C...........A......T  Mmulatta
  366141: ....G.....................A...............A......T  Panubis
  399656: ....G....C.C..........C.C.A..................G...-  Cjacchus
 1046703: ....G......C..G.A.......C........................-  Sboliviensis

                  49107     49117     49127     49137     49147
                      :         :         :         :         :
   49098: GGAACTTTACATGAGAGTTTCAATAAATCCATTTCACTGAAACTGTAAGT  Hsapiens
  368837: ..................................................  Ptroglodytes
  386918: .........T........................................  Ggorilla
  418140: ................................................A.  Pabelli
  339903: .............................A....................  Nleucogenys
  415563: AT........C.......................................  Mmulatta
  366191: AT................................................  Panubis
  399705: A.....................G....A......................  Cjacchus
 1046752: A.....................G....A......................  Sboliviensis

                  49157     49167     49177     49183     49193
                      :         :         :         :         :
   49148: TAGCACAAAGTCAATCTCTTAATGATAAATG----TCATGTGACTCTTCT  Hsapiens
  368887: ...............................----...............  Ptroglodytes
  386968: ...............................----...............  Ggorilla
  418190: C..............................----....A.....G....  Pabelli
  339953: C..............................----........T.....C  Nleucogenys
  129244:           ...G..AT.C..........T----A......            Mmulatta
  415613: C...T....T                                          Mmulatta
  415644:                                               ....  Mmulatta
  366241: C...T..G.T...GC..............C.---------------....  Panubis
  399755: C...T........G.................----...............  Cjacchus
 1046802: C..TG..........G...............AATG...............  Sboliviensis

                  49203     49213     49223     49233     49243
                      :         :         :         :         :
   49194: CACTCTTCATCAATTTTGGGGTATGAAATTCAGAGGACGCCAAGAAAGTT  Hsapiens
  368933: ..................T...................C...........  Ptroglodytes
  387014: ..................T...G.........................A.  Ggorilla
  418236: ..T...............T..................T............  Pabelli
  339999: ......C.......-..AT..................T............  Nleucogenys
  415648: ..................T..................T............  Mmulatta
  366276: ..................T..................T............  Panubis
  399801: ..............G...T................AGT...........-  Cjacchus
 1046852: ............G.G...T.................GT............  Sboliviensis
```

```
                   49253     49263     49273     49283     49293
                       :         :         :         :         :
  49244: GTTGATCACTCTTCCAAATGGACTTACCTGATGAAATGCCATCGTCTTTG   Hsapiens
 368983: ......................................T...........   Ptroglodytes
 387064: ..................................................   Ggorilla
 418286: .....C............................................   Pabelli
 340048: ....GC...........................G........T.......   Nleucogenys
 415698: .....C..T........................G........T.......   Mmulatta
 366326: ....GC..T........................G........T.......   Panubis
 399850: ------..........................AG..CA.....A......   Cjacchus
1046902: .....G...............G...........G..G......A......   Sboliviensis

                   49303     49313     49323     49333     49343
                       :         :         :         :         :
  49294: TCCCGTAGAGCCTGTTGGCAATGCTGAGGGTACAGTTAGAGTCTGGCTGG   Hsapiens
 369033: ..................................................   Ptroglodytes
 387114: G.............................C...................   Ggorilla
 418336: ....A.............................................   Pabelli
 340098: ................................T.................   Nleucogenys
 415748: ................................T.................   Mmulatta
 366376: ................................T.................   Panubis
 399894: .......A..T..........C........C.T.................   Cjacchus
1046952: .......A..T...................C.T.................   Sboliviensis

                   49353     49363     49373     49383     49393
                       :         :         :         :         :
  49344: TTGATTTGAGAGAATTAGACACCAAACTCGGAATGAATTCTTCCAGCTTG   Hsapiens
 369083: ...............A..................................   Ptroglodytes
 387164: ...............A..................................   Ggorilla
 418386: ...............A..................................   Pabelli
 340148: ...............A.............A....................   Nleucogenys
 415798: ...............A............................G.....   Mmulatta
 366426: ...............A............................G.....   Panubis
 399944: ...............AG....T.......A..........C.........   Cjacchus
1047002: ..............CAG....T.......A....................   Sboliviensis

                   49403     49413     49423     49433     49443
                       :         :         :         :         :
  49394: GCTGCACTGAAGCATCAGTAAACAAAGAATGATGTATTAAATTGCTTGAT   Hsapiens
 369133: ..................................................   Ptroglodytes
 387214: ..................................................   Ggorilla
 418436: ...............................G...............T..   Pabelli
 340198: ...............................G..................   Nleucogenys
 415848: ...............T...............G..................   Mmulatta
 366476: ...............T...............G..................   Panubis
 399994: AGA............T...............G..................   Cjacchus
1047052: AGA............T...............G..................   Sboliviensis

                   49453     49463     49473     49483     49493
                       :         :         :         :         :
  49444: TTTTGCCATTGGAGAATGATAACTATCTAGAGAGCAAGGTGTTTGCCTCT   Hsapiens
 369183: ..................................................   Ptroglodytes
 387264: ..................................................   Ggorilla
 418486: ..................................................   Pabelli
 340248: .....................C............................   Nleucogenys
 415898: ........................G....T.C..................   Mmulatta
 366526: ...............................................A..   Panubis
 400044: .......G.......GC................C................   Cjacchus
1047102: ......T..C.....G.........C...C...A....AC..........   Sboliviensis
```

```
              49503     49513     49523     49533     49542
                  :         :         :         :         :
  49494: GAATAAAAGGGCTGTGAATACAAGGAAATTAGTTTAAAGCATTTTCTT-C  Hsapiens
 369233: ................................................-.  Ptroglodytes
 387314: ................................................-.  Ggorilla
 418536: ................................................-.  Pabelli
 340298: .............A..................................-.  Nleucogenys
 415948: ................................................-.  Mmulatta
 366576: ................................................-.  Panubis
 400094: .......T............T........G.....C.........T..C.  Cjacchus
1047152: .......C....--......T........G.....C.........T..-.  Sboliviensis

              49552     49562     49572     49582     49592
                  :         :         :         :         :
  49543: CTTGGATTTTTGCTAGCACATTAATTTCACCACTCTGTAACTTTAGTTGC  Hsapiens
 369282: ..................................................  Ptroglodytes
 387363: ...................G..............................  Ggorilla
 418585: ..........CA......................................  Pabelli
 340347: ..........CA...............................C......  Nleucogenys
 415997: T.........C.......................................  Mmulatta
 366625: T.........C.......................................  Panubis
 400144: ..........GA............................G...G.....  Cjacchus
1047199: ..........GA............................G...G.....  Sboliviensis

              49602     49612     49622     49632     49642
                  :         :         :         :         :
  49593: AGTCTGTGTGCAGAATTGTAAGTTACTGCATAATACATGTGTACTTGTTG  Hsapiens
 369332: ..........................C.......................  Ptroglodytes
 387413: ......C......................C....................  Ggorilla
 418635: ...................................T..............  Pabelli
 340397: ..................................................  Nleucogenys
 416047: ...............................................-..  Mmulatta
 366675: ......C.....................T..................-..  Panubis
 400194: .................A.................A....T.....A...  Cjacchus
1047249: .................A........................G..C....  Sboliviensis

              49652     49662     49672     49682     49692
                  :         :         :         :         :
  49643: GTCCTAATGATTTAATTCAAAGGCAGTTTTCATAAATAAAGAGATCCTAG  Hsapiens
 369382: ..................................................  Ptroglodytes
 387463: ..................................................  Ggorilla
 418685: ..................................................  Pabelli
 340447: ..................................................  Nleucogenys
 416096: .......C................................C.........  Mmulatta
 366724: .......C................................C.........  Panubis
 400244: ..A..........G.................................C..  Cjacchus
1047299: .........................A.....................C..  Sboliviensis

              49702     49712     49722     49732     49742
                  :         :         :         :         :
  49693: TGGGGTATTCAGGGCAATAATTCCAGTGTTATTGCTGCCTTTGTACTTAC  Hsapiens
 369432: ..................................................  Ptroglodytes
 387513: ..................................................  Ggorilla
 418735: ..................................................  Pabelli
 340497: ................................................G.  Nleucogenys
 416146: ...............................C.......C..........  Mmulatta
 366774: .......................................C.........T  Panubis
 400294: ......G....-.................A........T......G....  Cjacchus
1047349: ......G......................A.C......T......G....  Sboliviensis
```

```
                   49752     49762     49772     49782     49792
                       :         :         :         :         :
   49743: AGAATCGTTTCTCTTTTCTTTCACACTTTTTTCTACATCTCCATTTTTCA  Hsapiens
  369482: ..................................................  Ptroglodytes
  387563: ..................................................  Ggorilla
  418785: ......A........C.T......-.C......C..G.............  Pabelli
  340547: ......A........C....................G.............  Nleucogenys
  416196: ...............C....................G.............  Mmulatta
  366824: ............T..C....................G.............  Panubis
  400343: ......A........C----...........C....GC............  Cjacchus
 1047399: ...............G.--..................G.............  Sboliviensis

                   49802     49812     49822     49832     49842
                       :         :         :         :         :
   49793: TGTGGTCATTTAATCAAATACCTCAGCCCTTCACTGAACTGACTTCACAC  Hsapiens
  369532: ..................................................  Ptroglodytes
  387613: ..................................................  Ggorilla
  418834: .....................................G.........G..  Pabelli
  340597: ..........................................T.......  Nleucogenys
  416246: ...........G............................T.G..T....  Mmulatta
  366874: ...........G............................T.G.......  Panubis
  400389: ....................G........C....................  Cjacchus
 1047447: .A..................G........................T....  Sboliviensis

                   49852     49862     49872     49882     49892
                       :         :         :         :         :
   49843: ACTAATTTGCTTCTCAGTGGTAGCAATTCTGCTAAGCTGCAGCTTTGTTG  Hsapiens
  369582: ..................................................  Ptroglodytes
  387663: ....C.............................................  Ggorilla
  418884: ..................................................  Pabelli
  340647: ..................................................  Nleucogenys
  416296: ..................................................  Mmulatta
  366924: ...............................T..................  Panubis
  400439: ...........C...................................A..  Cjacchus
 1047497: .....C.....C...................................A..  Sboliviensis

                   49902     49912     49922     49932     49942
                       :         :         :         :         :
   49893: CATTTGAAAGATGGCTAAGAATTTATGGACATTATAGGCAATATTCCTAT  Hsapiens
  369632: .........A........................................  Ptroglodytes
  387713: ..................................................  Ggorilla
  418934: ..................................................  Pabelli
  340697: ..................................................  Nleucogenys
  416346: ......................................G.....G.....  Mmulatta
  366974: .....T................................G.....G.....  Panubis
  400489: T.....................................A...G.G.....  Cjacchus
 1047547: ......................................G.....G.....  Sboliviensis

                   49952     49962     49972     49982     49992
                       :         :         :         :         :
   49943: ATTCCCTAAAGCATTCCCTAAAGCAAATGGTTCCTAAACTATGGACTTTG  Hsapiens
  369682: .............G....................................  Ptroglodytes
  387763: ..................................................  Ggorilla
  418984: ......C...........................................  Pabelli
  340747: .........................G........................  Nleucogenys
  416396: ............G..............C-....A..............A.  Mmulatta
  367024: ............................-....A..............A.  Panubis
  400539: .A.................-..T................C..........  Cjacchus
 1047597: .......................A.............C.C..........  Sboliviensis
```

```
                                  DNA
              50002     50012     50022     50032     50042
   49993: AACTTTTAGTAGTAAGTTGGGTTCCGTGAAGCTGGTATTCTGTGAGTCTA   Hsapiens
  369732: .........................A........................   Ptroglodytes
  387813: .........................AG.......................   Ggorilla
  419034: ....................................G.............   Pabelli
  340797: ....................................G........A....   Nleucogenys
  416445: ........A....C..C..............T....G.........G...   Mmulatta
  367073: ........A....C..C..............T....G............G   Panubis
  400588: ..A....                                              Cjacchus
  400901:        .A......A........G...........G....A.......G   Cjacchus
 1047647: ........A...............A...........G....A........   Sboliviensis

                                   DNA
                 ------------------------------------------>
              50052     50062     50072     50082     50092
   50043: TATGCATTTATGTATTTTCTCAACCAATCGTAATCAAACAGAACTATTTT   Hsapiens
  369782: .............................A....................   Ptroglodytes
  387863: .............................A....................   Ggorilla
  419084: .............................A....................   Pabelli
  340847: ......C.........G............A....................   Nleucogenys
  416495: ..C......G...................AC...................   Mmulatta
  367123: .........G...................AC...................   Panubis
  400944: .............................AC............G......   Cjacchus
 1047697: .............................AC............G......   Sboliviensis

                               DNA
          ------------------------------------------------->
              50102     50112     50122     50132     50142
   50093: CATGTAGGAGTTCTATATAATTTTGATGTCAAAATGAATCCTTCTACTGG   Hsapiens
  369832: ..................................................   Ptroglodytes
  387913: ..................................................   Ggorilla
  419134: ..................................................   Pabelli
  340897: ......A...........................................   Nleucogenys
  416545: .G...........................T....................   Mmulatta
  367173: TT................................................   Panubis
  400994: ...A......A................................T......   Cjacchus
 1047747: ...A.....C........................C...............   Sboliviensis

                 DNA
          ----------------->
              50151     50161     50171     50181     50191
   50143: -AAAAAACTAGAGACCATAGTCCCAGAGCATGTGGATTGGGCAATATTAG   Hsapiens
  369882: -.................................................   Ptroglodytes
  387963: -...........................................C.....   Ggorilla
  419184: A..............................................C..   Pabelli
  340947: -..............................................C..   Nleucogenys
  416595: -......................T.C...G.........A.A.GC..C..   Mmulatta
  367223: -........................C...G..C......A.A.GC..C..   Panubis
  401044: -........G...T........T............G..A.A..GC..C.A   Cjacchus
 1047797: -........G............T.G..........G..A.A..GC..C.A   Sboliviensis
```

```
              50201     50211               50229     50239
  50192: GCCATGTTTTCCCAGTACAT-AATTATAT-GCCAAGCATCAGGGTCCTTT   Hsapiens
 369931: ....................-........G....................   Ptroglodytes
 388012: ....................-........G....................   Ggorilla
 419234: A..................C-........G.....A..............   Pabelli
 340996: ..T.................-........G....G...............   Nleucogenys
 416644: ...............C....-........G....................   Mmulatta
 367272: ...............C....G........G....................   Panubis
 401093: ...............C.T..-.......CG........G..C.A......   Cjacchus
1047846: ...............CGT..-.......CG........G..T.A....C.   Sboliviensis

              50249     50259     50269     50279     50289
  50240: CTCAGTCCCCACTCTAGACAGAAAAGTCTAACACCACATAGACAAAACTG   Hsapiens
 369980: .............................................G....   Ptroglodytes
 388061: .............................................G....   Ggorilla
 419283: ........T..T.................................G....   Pabelli
 341045: ........-....................................G....   Nleucogenys
 416693: .........................A...................G....   Mmulatta
 367322: .........................A...................G....   Panubis
 401142: A.................G..........................G...C   Cjacchus
1047895: A.......T....................................G....   Sboliviensis

              50299     50309     50319     50329     50339
  50290: GCAAACTCAACACAGGGCACCGTCCATAAAGGAACAAAAAAGTTTGAGTA   Hsapiens
 370030: ........................................G.........   Ptroglodytes
 388111: .....................A............................   Ggorilla
 419333: ............T.......A.............................   Pabelli
 341094: ...G........T.......T.............................   Nleucogenys
 416743: ...G.......GT........C....................C.......   Mmulatta
 367372: ...G......TGT.C......C....................C.......   Panubis
 401192: ...G........TGA.A....A......T....C......T.........   Cjacchus
1047945: ...G........TGA.A...TA......T...........T.........   Sboliviensis

                                                Simple
                                               ---------->
              50349     50359     50369     50379     50389
  50340: AAGATGGCAATAAAGCAGGTAATAAAATCTAAGAAACTTTACTATTGTTA   Hsapiens
 370080: ..............T...................................   Ptroglodytes
 388161: ..............T...................................   Ggorilla
 419383: .......A......T..................................-   Pabelli
 341144: .G............T...................................   Nleucogenys
 416793: .G............T...................................   Mmulatta
 367422: .G............T...................................   Panubis
 401242: .G.....T.G...T.G........................T...C.C..   Cjacchus
1047995: .G............T............C.............T...A....   Sboliviensis

                     Simple
         ----------------------------->
                        50401     50410     50420     50430
  50390: TT---AT-----TATCATTATTA-TATTAAACTCTACAGGCTGTAGTTAT   Hsapiens
 370130: ..---..-----...........-..........................   Ptroglodytes
 388211: ..---..-----...........-..........................   Ggorilla
 419432: -----..-----...........-..........................   Pabelli
 341194: ..---..-----...........-................T.........   Nleucogenys
 416843: ..ATC..-----...........-..C.......................   Mmulatta
 367472: ..ATC..-----...........-..C.......................   Panubis
 401292: ..---G.TATCA...........-C.G.....................T.   Cjacchus
1048045: ..---G.-----.........C.T.....---C.C.............T.   Sboliviensis
```

```
              50440     50450     50460     50470     50480
                  :         :         :         :         :
  50431: CATCAGGAAGGTTAAAGGGGGACACATAGCTGGTTCCCTCATGGGCAAAA Hsapiens
 370171: .........................................C........ Ptroglodytes
 388252: .................................................. Ggorilla
 419471: .................................................. Pabelli
 341235: ................C................................. Nleucogenys
 416887: ............................................A..... Mmulatta
 367516: ..........................G.....T...........A..... Panubis
 401338: .........A................A...C....T...........G.. Cjacchus
1048084: .................A........A...CA...............G.. Sboliviensis

              50490     50500     50510     50520     50530
                  :         :         :         :         :
  50481: GAAGGAGCTTTTCTACTCAACACCCAGACTATTATTTCATCCTTCTCTTA Hsapiens
 370221: .........C........................................ Ptroglodytes
 388302: .........C........................................ Ggorilla
 419521: .........C........................................ Pabelli
 341285: .........C............T........................... Nleucogenys
 416937: .......T.C..........TG.T.....A.............C...... Mmulatta
 367566: .........C..........TG.T.....A.............C...... Panubis
 401388: .............C.........T.......................... Cjacchus
1048134: ......A..C.................G..G................... Sboliviensis

              50540     50550     50557     50567     50577
                  :         :         :         :         :
  50531: GGTTAAAGAGCATTTGACAACTCAG---TAATATTATCATACCTTAGGTG Hsapiens
 370271: .........................---C..C......G........... Ptroglodytes
 388352: .........................---C..C.................. Ggorilla
 419571: .........................---C..................... Pabelli
 341335: .........................---C..................... Nleucogenys
 416987: .........................---C.G................... Mmulatta
 367616: .........A...............---C..................A.. Panubis
 401438: ................G..G.....CAA...................... Cjacchus
1048184: ...................G.....CAA......C............... Sboliviensis

              50587     50597     50607     50617     50627
                  :         :         :         :         :
  50578: AGTCCTTGAACCCTGGTTTTAATGAGTCTACAGTATGACTAAAATGAAGC Hsapiens
 370318: .................................................. Ptroglodytes
 388399: .................................................. Ggorilla
 419618: ........................G......................... Pabelli
 341382: ...............................C.................. Nleucogenys
 417034: .......................................G.......... Mmulatta
 367663: ......................................TG.......... Panubis
 401488: ...........T..T.......................T........... Cjacchus
1048234: ...........T..T.......................T........... Sboliviensis

              50637               50656     50666     50676
                  :                   :         :         :
  50628: ACCTAGAAAAGAAAAAAAA-GGGATCTTTTGGCATCAGAAGCAATGTACA Hsapiens
 370368: ...................AA............................. Ptroglodytes
 388449: ...................--............................. Ggorilla
 419668: .........G........G---............................ Pabelli
 341432: ...................--..............--------.....T. Nleucogenys
 417084: ...................-A.....CA...................... Mmulatta
 367713: ...................--.....CA............AA........ Panubis
 401538: ......G............--A.....G......G......A.......G Cjacchus
1048284: ......G.........T..--A.....G.....G.......A......TG Sboliviensis
```

```
              50686     50696     50705     50715     50725
                  :         :         :         :         :
  50677: GTCTATAAAGGTTGACAGTTG-TAAGTATCTATTTTTCTATTTTAGATCA  Hsapiens
 370418: .....................-----...........T............  Ptroglodytes
 388497: .....................-----........................  Ggorilla
 419715: .....................T............................  Pabelli
 341473: .....................-............................  Nleucogenys
 417133: ......C..............-....G.......................  Mmulatta
 367761: ..G...C..............-....G.......................  Panubis
 401586: ....GC........-.....C--.......C........G..........  Cjacchus
1048332: ....GC........-.....T-............................  Sboliviensis


                                      MIR
                       ------------------------------------>
              50735     50745     50755     50762
                  :         :         :         :
  50726: AGATCAGTAGTGATAGCTAACATTTAATAATGT---GTTCCAAG------  Hsapiens
 370463: .....CA..........................---........------  Ptroglodytes
 388542: .....CA..........................---........------  Ggorilla
 419765: ......A..............G......G....TGT........------  Pabelli
 341522: ...C..A..........................TGT........------  Nleucogenys
 417182: ...C..A.............-............TGCT......A------  Mmulatta
 367810: ...C..A.............-............TGCT......A------  Panubis
 401633: ...C..A......G...................TGTC......ACTCAGC  Cjacchus
1048380: ...A..A..............T...........TATC......ACTCAGC  Sboliviensis


                                 MIR
         -------------------------------------------------->
              50776     50786     50796     50806     50816
                  :         :         :         :         :
  50767: TCCTGAACTAAGTCTTTACATATCTTATTTAACCTTCCCAACAACTTTAT  Hsapiens
 370504: ..................................................  Ptroglodytes
 388583: ..................................................  Ggorilla
 419809: ..................................................  Pabelli
 341566: ............................................T.....  Nleucogenys
 417225: .........................................A....G...  Mmulatta
 367853: .........................................A....G...  Panubis
 401683: ....A.............................................  Cjacchus
1048430: ....A....................................G........  Sboliviensis


                                 MIR
         -------------------------------------------------->
              50826     50836     50846     50856     50862
                  :         :         :         :         :
  50817: GAGTAGATACTATTACCCTCATTTTATGTTAGGGAAACTAT----AAGTT  Hsapiens
 370554: .........................................----.....  Ptroglodytes
 388633: .........................................----.....  Ggorilla
 419859: ...........G............--..............C----.....  Pabelli
 341616: ..............G..........................----.....  Nleucogenys
 417275: A......................C.................----.....  Mmulatta
 367903: A......................C.................----.....  Panubis
 401733: ...............-....................G....TAAT....C  Cjacchus
1048480: ....................................G....TAAT....C  Sboliviensis
```

```
                          MIR
               ------------------------------->
                 50872     50882     50892     50902     50912
                     :         :         :         :         :
   50863: TAAGAGAGATCAAGCAGCTTGCCTCAGACCATTGTCAGGTTCCAGGGACT   Hsapiens
  370600: ......................................T...........   Ptroglodytes
  388679: ......................................T...........   Ggorilla
  419903: ............CA.........................C..........   Pabelli
  341662: .............A.........................C..........   Nleucogenys
  417321: ...........G..........G........C.......C..........   Mmulatta
  367949: ...........G..........G........C.......C..........   Panubis
  401782: C...................................GA.C..........   Cjacchus
 1048530: C....................................A.C......T...   Sboliviensis

                 50922     50932     50942     50952     50962
                     :         :         :         :         :
   50913: AATCTCTTATCCCCTGGACACTTCTGTCCCTACTGTGCTTAGGCTGTTAA   Hsapiens
  370650: ..................................................   Ptroglodytes
  388729: ..................................................   Ggorilla
  419953: ...............A...C.............A................   Pabelli
  341712: ...............A...C..............................   Nleucogenys
  417371: ............G..A...C...............CT.........A...   Mmulatta
  367999: ............G..A...C...............CT.........A...   Panubis
  401832: ............A..A...C....C...........T.....T.......   Cjacchus
 1048580: ............A..A..AC.......T........T.....T.......   Sboliviensis

                 50972     50982     50992     51002     51012
                     :         :         :         :         :
   50963: CTAAGACAGTACCCATCTGGGGCTGTGTAAGACCTTACATGGTTTCCCAG   Hsapiens
  370700: .........................CA.......................   Ptroglodytes
  388779: .........................C........................   Ggorilla
  420003: ........................................T.........   Pabelli
  341762: ..................................................   Nleucogenys
  417421: ...........----------------------------------.....   Mmulatta
  368049: ...........----------------------------------.....   Panubis
  401882: ...G....................A.........................   Cjacchus
 1048630: ...G.G..................AC........................   Sboliviensis

                 51022     51032     51042     51052     51062
                     :         :         :         :         :
   51013: CACGAACAATAATAAGATTTGCAAGTATCCTAAGAGTTGTTAAGTGATAT   Hsapiens
  370750: ..................................................   Ptroglodytes
  388829: ...A..............................................   Ggorilla
  420053: ...A..............................................   Pabelli
  341812: ...A..............................................   Nleucogenys
  417437: ...A..............................................   Mmulatta
  368065: ...A..............................................   Panubis
  401932: ...A........G...................C..............C..   Cjacchus
 1048680: ...A..............................................   Sboliviensis

                                                        MIR
                                                       ---->
                 51072     51082     51092     51102     51112
                     :         :         :         :         :
   51063: TCTTCTAGACTTTCCACTGCAATTAAAATATACGTAATGGGAAAATCTCT   Hsapiens
  370800: .......................G........G.................   Ptroglodytes
  388879: .......................G........G.................   Ggorilla
  420103: .......................G........T......A..........   Pabelli
  341862: ...C...................G........T.................   Nleucogenys
  417487: .......................A........T.................   Mmulatta
  368115: .......................A........T.................   Panubis
  401982: ...C..CT.....T..T......A........GC...C..A.........   Cjacchus
 1048730: ...C..CTGA......T......A........TC......A........C   Sboliviensis
```

```
                               MIR
         ---------------------------------------------------->
              51122     51132     51142     51152     51162
  51113: AACGTTAGCCTATATAACCTGAAAAGTCACCTTTCCTTTCTGAGTCTCAG Hsapiens
 370850: ..T.................A............................. Ptroglodytes
 388929: ..T.................A............................. Ggorilla
 420153: ..T..............T................................ Pabelli
 341912: ..T.............................................G. Nleucogenys
 417537: ..T............................................... Mmulatta
 368165: ..T............................................... Panubis
 402032: ..T.....................C......................... Cjacchus
1048780: ..T......................................A........ Sboliviensis

                               MIR
         ---------------------------------------------------->
              51171     51181     51191     51201     51211
  51163: TTTT-CAATCCATAAAGTGTGGATAACAATTCCTAACTGTTATGAAGTTC Hsapiens
 370900: ....-.....TG...................................... Ptroglodytes
 388979: ....-.....TG...................................... Ggorilla
 420203: ....-.....TG...................................... Pabelli
 341962: ....-.....TG........................A.....C....... Nleucogenys
 417587: ....-.....TG..................................T..G Mmulatta
 368215: ....-.....TG..............T...................T..G Panubis
 402082: ....T.....TG...G..............C................... Cjacchus
1048830: ....T.....TG......A...........G.......T....A...... Sboliviensis

                    MIR
         ---------------------------->
              51221     51230     51239     51249     51259
  51212: AAACATAATCATGAAAA-TTTTTTAA-CTTGGTAATGTCTAAAATAGCAT Hsapiens
 370949: .................T........-....................... Ptroglodytes
 389028: .................T........-..........C............ Ggorilla
 420252: ...T.............T........-....................... Pabelli
 342011: .................TC.......-....................... Nleucogenys
 417636: .................T........-....................... Mmulatta
 368264: .................T........-....................... Panubis
 402132: ...TG............A........A..........C.......G.... Cjacchus
1048880: ...TG......CT....A.....A..C..........C.......G.T.. Sboliviensis

              51269     51279     51289     51299     51309
  51260: TATGTTTATTATAAATTTAACCTTCTAGAGGACAATAATAGTACCTTTAT Hsapiens
 370998: .............................A..................G. Ptroglodytes
 389077: .............................A..................G. Ggorilla
 420301: ..................................G.............G. Pabelli
 342060: ...........................................A....G. Nleucogenys
 417685: ..C.......G................C...G....C......A..C.G. Mmulatta
 368313: ..C.......G...........A....C........C......A..C.G. Panubis
 402182: ..C.........C.................A...............C.G. Cjacchus
1048930: ..CA.......................................A..C.G. Sboliviensis
```

```
              51319     51329     51339     51349     51359
  51310: CACATTTAAATCACTGAATGAAGGGACTCTAAAAGTAAACTGCAGAGATT  Hsapiens
 371048: ..................................................  Ptroglodytes
 389127: ..................................................  Ggorilla
 420351: ..................................................  Pabelli
 342110: ..A.G.............................................  Nleucogenys
 417735: ...G...................A...C......................  Mmulatta
 368363: .......................A...C......................  Panubis
 402232: ..................A...................G......G....  Cjacchus
1048980: ..................A.....A........------...........  Sboliviensis

              51369     51379     51389     51399     51409
  51360: TATTTGTGATACCACTGATAGTAAAAAAGAGACCACACGTTGCTTGTATT  Hsapiens
 371098: ..................................................  Ptroglodytes
 389177: ........................T.........................  Ggorilla
 420401: ....................................G..C..........  Pabelli
 342160: ............T.......................G.............  Nleucogenys
 417785: ....................................GT............  Mmulatta
 368413: ....................................GT............  Panubis
 402282: ........G..............GGG......GT.---------......  Cjacchus
1049024: ..................G.....GG......T..---------..C...  Sboliviensis

              51419     51429     51439     51449     51459
  51410: ACATTGTTTGCATTACAGCATACTCCATTAATCAAACCATTATCAATTTT  Hsapiens
 371148: ..................................................  Ptroglodytes
 389227: ..................................................  Ggorilla
 420451: ....................................T.............  Pabelli
 342210: .................A......T....T....................  Nleucogenys
 417835: ......C.....C.....................C...............  Mmulatta
 368463: ......C.....C.............G.......................  Panubis
 402323: .....A....T.CC..........AA........G...............  Cjacchus
1049065: .....A......C...........AA........................  Sboliviensis

              51469     51479     51486     51496     51506
  51460: TAAGAAATATGATTTATTCAATGTG---TTTTTTCTCTCTCAAGATGGCA  Hsapiens
 371198: .........C...............T--......................  Ptroglodytes
 389277: .........................---......................  Ggorilla
 420501: .........................---......................  Pabelli
 342260: ....................T....---C..........C..........  Nleucogenys
 417885: ................C........TTT........T.............  Mmulatta
 368513: ................C........GTT......................  Panubis
 402373: ...........C.C..C........T--....G......C.........G  Cjacchus
1049115: ...........C.C..C........G--...........C..........  Sboliviensis

              51516     51526     51536     51546     51556
  51507: ATTTTATTGGTATAAAGATAGAATTAATTTTGCTGACCCAGAGCGGGAGC  Hsapiens
 371246: ...................G.......................T...C..  Ptroglodytes
 389324: ...................G.......................T...C..  Ggorilla
 420548: .................................A.............CA.  Pabelli
 342307: ...................G....................C......C..  Nleucogenys
 417935: ..................G...........G................T..  Mmulatta
 368563: ..............................G................T..  Panubis
 402421: G....C..AA......C........C....G...AC...T----------  Cjacchus
1049163: G........A......T........C....G............G...TAT  Sboliviensis
```

```
              51566     51576     51586     51596     51606
                  :         :         :         :         :
  51557: ATGAAAGACTTCTGGGTTCTGAACAAACCTCTGAGGAATTCCATACCTTC  Hsapiens
 371296: .................C.........T.....G................  Ptroglodytes
 389374: .................C.........T.....G................  Ggorilla
 420598: .................C.........T..G..G................  Pabelli
 342357: ............C....C.........T.....G................  Nleucogenys
 417985: ................CC....GT...T.....T................  Mmulatta
 368613: ................CC...GG....T.....T................  Panubis
 402461: ---...A...C.....CC...GG....T....AT................  Cjacchus
1049213: ---...A.........CC..TGGT...T.....T................  Sboliviensis

              51616     51626     51636     51646     51656
                  :         :         :         :         :
  51607: TCCAATTGCTCTTCAGTCTCTCCCCTGGCACCAAGGAGGACCATGCTTAG  Hsapiens
 371346: ............G.....................................  Ptroglodytes
 389424: ............G.....................................  Ggorilla
 420648: ............G......T...G.............A...........A  Pabelli
 342407: ............G....................................C  Nleucogenys
 418035: ....G.......G..C..................................  Mmulatta
 368663: ..T.G.......G..C..................................  Panubis
 402508: ............G..C..................................  Cjacchus
1049260: ............G..C..................................  Sboliviensis

              51666     51676     51686     51696     51706
                  :         :         :         :         :
  51657: AGCATAAAGCAGACTCAGCGAAGAAAAGAAGATGTTATCTCCTATGTTGT  Hsapiens
 371396: ..................................................  Ptroglodytes
 389474: ..................................................  Ggorilla
 420698: ..................T.G.............................  Pabelli
 342457: ....................G......................G......  Nleucogenys
 418085: ...............T....G......................G......  Mmulatta
 368713: .........T.....T....G.............................  Panubis
 402558: .........T..........GG............................  Cjacchus
1049310: .........T..........GG............................  Sboliviensis

              51716     51726     51736     51746     51756
                  :         :         :         :         :
  51707: TACTGTTCAGCTCTTTGAACACATCAAGGCAAAATTCAACGTTAGCTGTG  Hsapiens
 371446: ..................................................  Ptroglodytes
 389524: ..................................................  Ggorilla
 420748: .G................................................  Pabelli
 342507: ..................................................  Nleucogenys
 418135: ........................................A.........  Mmulatta
 368763: ..................................................  Panubis
 402608: .......G................................A.........  Cjacchus
1049360: .......G................................A.........  Sboliviensis

              51766     51776     51783     51793     51803
                  :         :         :         :         :
  51757: CTGAGAGAACCCATTTTTATGCC---GACTGCCTGAGAACAGAAGGGAAA  Hsapiens
 371496: ..........G............---A.......................  Ptroglodytes
 389574: .......................---........................  Ggorilla
 420798: .......................---...........G............  Pabelli
 342557: .C....................T---...........G............  Nleucogenys
 418185: .....G.................AAT....................A...  Mmulatta
 368813: .......................AAT....................A...  Panubis
 402658: .......................AACA.......................  Cjacchus
1049410: .......................AACC............T..........  Sboliviensis
```

```
              51813     51823     51833     51843     51853
                  :         :         :         :         :
   51804: AAATTCTCAGAACATCACTTGAAGTTACAGATAGTATCTGGAGTTCAGTT  Hsapiens
  371543: ...A.......................A..............A.......  Ptroglodytes
  389621: .....................................T..........C.  Ggorilla
  420845: ..........................................A.......  Pabelli
  342604: ....................C..........................A..  Nleucogenys
  418235: ..................................................  Mmulatta
  368863: .....T....................................T.......  Panubis
  402708: ....GA.................A.....A....G...............  Cjacchus
 1049460: .....A...C.............A.G....C...G...............  Sboliviensis

              51863     51873     51883     51893     51903
                  :         :         :         :         :
   51854: ATTGTATTTCATGAGTTTAAGTATCAGGGCACACTGCTTCATGAAAAACT  Hsapiens
  371593: ...........................A....................T.  Ptroglodytes
  389671: ...T..............................................  Ggorilla
  420895: ...........C....................................T.  Pabelli
  342654: ................................................T.  Nleucogenys
  418285: ....C...........................................T.  Mmulatta
  368913: ....C.C.........................................T.  Panubis
  402758: ...AC.....G.......................----------------  Cjacchus
 1049510: ...AA...........................................T.  Sboliviensis

              51913     51923     51933     51943     51953
                  :         :         :         :         :
   51904: ACATACCCTTATCACAATTTCTGAGAAATGAGTAAATGCTAGACAGGGGA  Hsapiens
  371643: .............................T.............G......  Ptroglodytes
  389721: .............................T.............G......  Ggorilla
  420945: ......G.....................CT.............G......  Pabelli
  342704: ...A.G.......................T.............G......  Nleucogenys
  418335: .......T.........-...........TCA...........G......  Mmulatta
  368963: .......T.........-...........T.A...........G......  Panubis
  402792: ------.......................T.............GT.....  Cjacchus
 1049560: .T.C.......................G.T.............G....A.  Sboliviensis

              51963     51973     51983     51993     52003
                  :         :         :         :         :
   51954: ATTCACATTAGTTTTACATTCCAATGAACAGAAGATTTACTATTCCTTCA  Hsapiens
  371693: ..................................................  Ptroglodytes
  389771: ..................................................  Ggorilla
  420995: ..................................................  Pabelli
  342754: ......................................T...........  Nleucogenys
  418384: ............G.....................................  Mmulatta
  369012: ......G.............G..............G.C............  Panubis
  402836: .......G........G.G..T..C...T.....................  Cjacchus
 1049610: .......G........G....T......T.....................  Sboliviensis

              52013     52023     52033     52043     52053
                  :         :         :         :         :
   52004: GCCAATGGTTAAAATCTATCTTACTATCATTTGTGTATATTTTTAAATAA  Hsapiens
  371743: ..................................................  Ptroglodytes
  389821: ..................................................  Ggorilla
  421045: ....G......................A.........A............  Pabelli
  342804: ..................................................  Nleucogenys
  418434: .....................................C............  Mmulatta
  369062: ...........................T.........C............  Panubis
  402886: .....CA........................G................G.  Cjacchus
 1049660: ..A..CAC.......................G................G.  Sboliviensis
```

```
              52063     52073     52083     52093     52103
                  :         :         :         :         :
   52054: TCTATAATAGTTCTGGAGCAAAAGATTAAAAGGAATTGAATCTTTGTATT   Hsapiens
  371793: ........T.........................................   Ptroglodytes
  389871: ........T.........................................   Ggorilla
  421095: ........T.........................................   Pabelli
  342854: ....A...T.......................A.................   Nleucogenys
  418484: ........T..........G......G...........G.......C...   Mmulatta
  369112: ....G...T.........TG..................G.......C...   Panubis
  402936: ........T.................C...........G........G..   Cjacchus
 1049710: ....A...G.................C.........G.G........G..   Sboliviensis

              52113     52123     52133     52143     52153
                  :         :         :         :         :
   52104: CTGAATTTCCATGCTTTAAGACATTCCTCATCCTGGAATTTGTGCAGAAA   Hsapiens
  371843: ..................................................   Ptroglodytes
  389921: .-A....................................C..........   Ggorilla
  421145: .................G........T.........C.............   Pabelli
  342904: ..........................T.......................   Nleucogenys
  418534: T...........A.................C................T..   Mmulatta
  369162: T...........A.................C................T..   Panubis
  402986: ............A.......G........T.................-..   Cjacchus
 1049760: ........T.................T..T.................--.   Sboliviensis

              52163     52173     52183     52193     52203
                  :         :         :         :         :
   52154: AAAATCTTGAGCTACACACGATCAGCCAGTCGAGATATTTTCCCACTAGA   Hsapiens
  371893: ...G.................................C............   Ptroglodytes
  389970: ..................................................   Ggorilla
  421195: ..................AA..............................   Pabelli
  342954: .....A.............T..............................   Nleucogenys
  418584: ..................A.........A..A..................   Mmulatta
  369212: ..................A.........A..A...C..............   Panubis
  403035: .G...........G....A............A.....C............   Cjacchus
 1049808: ...G......A..G....A.........A..A.....C.......G....   Sboliviensis

              52213     52223     52230     52240     52250
                  :         :         :         :         :
   52204: CTACCCGCTTTTGCAGTGTCAC---AGGAGAACATCTCCATCTCAGTCAT   Hsapiens
  371943: ......A...............---.....G...................   Ptroglodytes
  390020: ......A...............---.....G...................   Ggorilla
  421245: ......A...............AGG.........................   Pabelli
  343004: .A....A...............AGG...............C.........   Nleucogenys
  418634: .C....A....G..........AGG.......................G.   Mmulatta
  369262: ......A...............AGG.......................G.   Panubis
  403085: ...T..AT..............AGG.................C.......   Cjacchus
 1049858: ..G...A...............AGG.................C.......   Sboliviensis

                                                        Alu
                                                        -->
              52260     52270     52280     52290     52300
                  :         :         :         :         :
   52251: TTTTGCACAAACAAGTTGACAGCACTCGCAAAATTTCCAGCACATTTTTT   Hsapiens
  371990: ...........................................G......   Ptroglodytes
  390067: ..................................................   Ggorilla
  421295: ...........................A......................   Pabelli
  343054: ...........................A........G.............   Nleucogenys
  418684: .....T....................T...G..........T.G.....    Mmulatta
  369312: .....T....................T...G...........TG.....    Panubis
  403135: ..........................T.....T..C..T..........    Cjacchus
 1049908: .....T....................T........C..T..........    Sboliviensis
```

```
                                Alu
        ------------------------------------------------->
             52310     52320     52330     52340     52349
 52301: ATTTTTATTTTACTTTTTATTTTATTTTATTTTATTTTATTTTA-TTTAT  Hsapiens
372040: ...........TA-.G............................T.....  Ptroglodytes
390117: ............T--------------------------------.....  Ggorilla
421345: ............T-------.......------------------.....  Pabelli
343104: ............T---..TA........................T.....  Nleucogenys

                                Alu
        ------------------------------------------------->
             52358     52368     52378     52388     52398
 52350: TT-TTGAGACAGAATCTCGCTCTGTCGCCCAGACTGGAGTGCAGTGGTGC  Hsapiens
372089: ..-...............................................  Ptroglodytes
390135: ..-.A.............................................  Ggorilla
421370: ..T..........G..................G.......A....A....  Pabelli
343151: ..---........G....A.............G.....A.....C...A.  Nleucogenys

                                Alu
        ------------------------------------------------->
             52408     52418     52428     52438     52448
 52399: GATCTCAGCTCACTGCAAGCTCCGCCTCCTGGGTTCATGCCATTCTCCTG  Hsapiens
372138: A....................................C............  Ptroglodytes
390184: ...............T.......A.............C............  Ggorilla
421420: A.............A..............A.......CA...........  Pabelli
343198: ................................A....CA...........  Nleucogenys

                                Alu
        ------------------------------------------------->
             52458     52468     52478     52488     52498
 52449: CCTCAGCCTCCCGAGTAGCTGGGACTACAGGCGCCTGCCACCACGCCCGG  Hsapiens
372188: ...................................C.......T...A..  Ptroglodytes
390234: ............C...................A..C..............  Ggorilla
421470: ...............................T...........T...G..  Pabelli
343248: ................................A..C.......T...A..  Nleucogenys

                                Alu
        ------------------------------------------------->
             52508     52518     52528     52538     52548
 52499: CTAACTTTTGTATTTTTTGGTAGAGACGGGGTTTCACCGTGTTAGCCAGG  Hsapiens
372238: ....T.............A...............................  Ptroglodytes
390284: ....T.............A...............................  Ggorilla
421520: ....T.............A......G........................  Pabelli
343298: ....T.............A.........A.....................  Nleucogenys

                                Alu
        ------------------------------------------------->
             52558     52568     52578     52588     52598
 52549: ATGGTCTCGACCTCCTGACCTCGTGATCCACCCACCTCGGCCTCCCAAAG  Hsapiens
372288: ..........T......................G................  Ptroglodytes
390334: ..........T.............A........G................  Ggorilla
421570: ..........T..........T...........G................  Pabelli
343348: ........A.T..........T........G..G...TT...........  Nleucogenys
```

```
                        Alu
         ------------------------------------------------->
              52608     52618     52628     52638     52648
  52599: TGCTGGGATTACAGGCGTGAGCCACCATGCCCGCACTTCCAGCACATTTT  Hsapiens
 372338: ..................................C...............  Ptroglodytes
 390384: ..........G.......................C...............  Ggorilla
 421620: ..........................GC.....GC...............  Pabelli
 343398: ..........................GCC...AGC...............  Nleucogenys
 418716:                                   .T.......T.G....  Mmulatta
 369344:                                   .T........TG....  Panubis
 403169:                                     .C..T.........  Cjacchus
1049940:                                   .T.C..T.........  Sboliviensis

              52658     52668     52678     52688     52697
  52649: TATCTCCTCAGTGGGACCTCATTCCCTGTATCCCTCCATGAAA-TAAGAC  Hsapiens
 372388: ...........................................G......  Ptroglodytes
 390434: ...........................................G......  Ggorilla
 421670: ...........................................G.T....  Pabelli
 343448: ...TG....................T.................GG.....  Nleucogenys
 418732: ...............G..........................TG.C....  Mmulatta
 369360: ..........................................TG......  Panubis
 403183: .............A...........G....-............GC.....  Cjacchus
1049956: ..............................-............GG....T  Sboliviensis

              52707     52717     52727     52737     52747
  52698: AATACTAACAAGTAGGACCTGCCATAAGAAAGTGGTCAGAGATCTCAGCC  Hsapiens
 372438: ..................................................  Ptroglodytes
 390484: ..................................................  Ggorilla
 421720: ................G.................................  Pabelli
 343498: ..................................................  Nleucogenys
 418782: ................T........................C........  Mmulatta
 369410: ................T........................C........  Panubis
 403232: ................TT......C................CC......T  Cjacchus
1050005: G...............TT..C...C................CC.......  Sboliviensis

              52757     52767     52777     52787     52797
  52748: TGGAGGCCACAGCCATCATGTTTATGAAAATAGAAATGGAAAAGGAAAAT  Hsapiens
 372488: ..........................................C.......  Ptroglodytes
 390534: ..................................................  Ggorilla
 421770: ..................................................  Pabelli
 343548: ..................................................  Nleucogenys
 418832: ............T.G.........C.....C...................  Mmulatta
 369460: .............TG.........C.........................  Panubis
 403282: CT...A............................................  Cjacchus
1050055: CT...A.....A..G.................A.................  Sboliviensis

              52807     52817     52827     52837     52847
  52798: CCAAGAGAAGAAAAGATGATGCTGGAATATGTGTTATCTTAGGACAAGAA  Hsapiens
 372538: ..................................................  Ptroglodytes
 390584: ..................................................  Ggorilla
 421820: ...................................T..............  Pabelli
 343598: ..................................................  Nleucogenys
 418882: .......................................C..........  Mmulatta
 369510: .......................................C..........  Panubis
 403332: .......---....A.C.G.A..............C..............  Cjacchus
1050105: ....C..G.A......C.G....-..G.....A.................  Sboliviensis
```

```
                                                  LINE2
                                   ----------------------------->
              52857     52867     52877     52887     52897
                  :         :         :         :         :
   52848: TATGTCAGGAAAGAAGAGTTCATTCTATTCAGTTCCACAAACATTGTTTG  Hsapiens
  372588: ..................................................  Ptroglodytes
  390634: ..................................................  Ggorilla
  421870: ..G......................A......G.................  Pabelli
  343648: ..................................................  Nleucogenys
  418932: .............................................AA...  Mmulatta
  369560: .............................................AA...  Panubis
  403379: .................A.....................G......A...  Cjacchus
 1050154: ........A........AC...........................A...  Sboliviensis

                                    LINE2
          ------------------------------------------------->
              52907     52917     52927     52937     52947
                  :         :         :         :         :
   52898: AGCACCGCTAACTGTAGAGGGCTATGGATATAAAAACAATCAAGAATAGT  Hsapiens
  372638: ......................................T...........  Ptroglodytes
  390684: .C....A..........................................G  Ggorilla
  421920: .....T............................................  Pabelli
  343698: ...........................C......................  Nleucogenys
  418982: ....T...C........................G..TG............  Mmulatta
  369610: ....T...C........................G..TG............  Panubis
  403429: ....G.A.A..G.................GC.T...TG........C...  Cjacchus
 1050204: ....G.A.............................TG............  Sboliviensis

                LINE2
          --------------->
              52957     52967     52977     52987     52997
                  :         :         :         :         :
   52948: TTCTGATCTCAGGGAGACGTCATTCTTCAGGAGCCTCATTGCCATCAAAC  Hsapiens
  372688: .....C............................................  Ptroglodytes
  390734: .....C............................................  Ggorilla
  421970: .....C...A........T...............................  Pabelli
  343748: .....C.......A....T...............................  Nleucogenys
  419032: .....C............T...............T............G..  Mmulatta
  369660: .....C............T.............A.T............G..  Panubis
  403479: .....C..........G.T..........A.C.................T  Cjacchus
 1050254: .....C..........G.T....................CA.........  Sboliviensis

                                                      LINE2
                                           --------------->
              53007     53017     53027     53037     53047
                  :         :         :         :         :
   52998: ACTTCCTTCCTTTAACTTCTGAGTCATCATTCTCTCTCAGTTCACGGCCT  Hsapiens
  372738: .................................G..........T.....  Ptroglodytes
  390784: ..................................................  Ggorilla
  422020: .....................................TG......A....  Pabelli
  343798: .........T...........T.........AG....TG......A....  Nleucogenys
  419082: .........T.........G.................TG..C...A....  Mmulatta
  369710: .........T.........G.T...............TG..C...A....  Panubis
  403529: ....T....T...........G........C...C..TG...........  Cjacchus
 1050304: ....TA...T...........G..T...T.G......TG......A....  Sboliviensis
```

```
                                LINE2
           ------------------------------------------------->
                 53057     53067     53077     53087     53097
                     :         :         :         :         :
   53048: GCCTCCTTGACTGCTCCTTCTCAGTCTCTTCTGCAGAATCGTTCTCAATT  Hsapiens
  372788: ..................................................  Ptroglodytes
  390834: ..................................................  Ggorilla
  422070: ..................C......T....T...................  Pabelli
  343848: ..............................T........T.....T....  Nleucogenys
  419132: ..............................T...................  Mmulatta
  369760: ..............................T........T..........  Panubis
  403579: ......................CA......A.........TC........  Cjacchus
 1050354: .G....................CC......T.........TC........  Sboliviensis

                                LINE2
           ------------------------------------------------->
                 53107     53117     53127     53137     53147
                     :         :         :         :         :
   53098: CTCAACCTCTAGACATCACAATAAAACAGAATTCAGATCTTGAGCCTCTT  Hsapiens
  372838: ..................................................  Ptroglodytes
  390884: ..................................................  Ggorilla
  422120: ..................................................  Pabelli
  343898: ...............C...................T..............  Nleucogenys
  419182: T.G......................G.................A......  Mmulatta
  369810: T.G......................G.................A......  Panubis
  403629: T.............G...A....C........G--------------..C  Cjacchus
 1050404: T......................C........G.TC..............  Sboliviensis

                                LINE2
           ------------------------------------------------->
                 53157     53167     53177     53187
                     :         :         :         :
   53148: CTCTATGAACAGTCATACCCTGGGTGATCTCATCCAACCTCATTCCTCC-  Hsapiens
  372888: .................T..G............................-  Ptroglodytes
  390934: .....................................A...........-  Ggorilla
  422170: ...C.................................T...........-  Pabelli
  343948: .................................................-  Nleucogenys
  419232: ...........C......T.CA...................---------  Mmulatta
  369860: ...........C.....TTACA...........................T  Panubis
  403665: A..........C........CA...........T.........G.....-  Cjacchus
 1050454: ...........C........CA...........T....T....G.....-  Sboliviensis

                                LINE2
           ------------------------------------------------->
                                     53201     53211     53221
                                         :         :         :
   53197: -------------------------AATGTCACCAATATGCTATTAGCTC  Hsapiens
  372937: -------------------------........T................  Ptroglodytes
  390983: -------------------------........T.............T..  Ggorilla
  422219: -------------------------........T................  Pabelli
  343997: -------------------------........T.G..............  Nleucogenys
  419273: -------------------------T.......T................  Mmulatta
  369910: AATATCTAATTAGGAGGAATGTCCT........T................  Panubis
  403714: -------------------------........T........G.......  Cjacchus
 1050503: -------------------------.G...A..T........G.......  Sboliviensis
```

LINE2

```
             53231     53241     53251     53261     53271
                 :         :         :         :         :
  53222: CCACATTTATGCTGACCTTGAATTCCAAACCCACATATCCAACTGCTTAC  Hsapiens
 372962: .TG...............................................  Ptroglodytes
 391008: .TG...............................................  Ggorilla
 422244: .TG......C....T...................................  Pabelli
 344022: .TG...........C..............T....................  Nleucogenys
 419298: .TGT.........TG..................T............C...  Mmulatta
 369960: .TG..........TG..................T............C...  Panubis
 403739: ..GT....C.....C.T...............--...A......A.C...  Cjacchus
1050528: .TG.....C.....T..................T..........A.C...  Sboliviensis
```

LINE2

```
             53281     53291     53297     53307     53317
                 :         :         :         :         :
  53272: TCAACATCCCCACTTGGATGTCTA----ATATATATCTCAAACTTAACAT  Hsapiens
 373012: ........................----......................  Ptroglodytes
 391058: ........................----......................  Ggorilla
 422294: ........................----......................  Pabelli
 344072: ........................----......................  Nleucogenys
 419348: ......A.........A.......----................G.....  Mmulatta
 370010: ........................----................G.....  Panubis
 403787: ...--...T...............ATAT...................TG.  Cjacchus
1050578: ...TT..--...............----....G...............G.  Sboliviensis
```

LINE2

```
             53327     53337     53347     53357     53367
                 :         :         :         :         :
  53318: GTCCAAAAGCAAATGCTTGATTTTCTAACCCCTCTCTCCAGCCTGTGTCC  Hsapiens
 373058: .............................T....................  Ptroglodytes
 391104: .............................T...A................  Ggorilla
 422340: ...................G.......C.T....................  Pabelli
 344118: ...T...............G.........T....................  Nleucogenys
 419394: ..........G...C....G........TT...............C....  Mmulatta
 370056: ..........G...C....G........TT...............C....  Panubis
 403835: .............C.....GC..A.....T....................  Cjacchus
1050622: ...................GC..A.....T..............T.....  Sboliviensis
```

LINE2

```
             53377     53387     53397     53407     53417
                 :         :         :         :         :
  53368: CTCACAGCCTTCTTTACCTCTGTAAATGGCAATTCCATTCTTCTATTCTT  Hsapiens
 373108: ...............G..................................  Ptroglodytes
 391154: ..................................................  Ggorilla
 422390: ...........................................C......  Pabelli
 344168: ...........................................C......  Nleucogenys
 419444: ............................A......T.......C.G.--.  Mmulatta
 370106: ............................A......T.......C.G.--.  Panubis
 403885: ............................A.....T........CGC.--.  Cjacchus
1050672: ................T..........................C...--.  Sboliviensis
```

```
                       LINE2
          ------------------------------------------------->
                53427     53437     53447     53457     53461
   53418: TCTCAAGCTAAAAACCTTGTGATCATCCTTAAGTACTTTCTC------GT  Hsapiens
  373158: ...................G......................------..  Ptroglodytes
  391204: ...................G......................------..  Ggorilla
  422440: ......A............C......................ATTCTTA.  Pabelli
  344218: ...................G......................ATTCTTA.  Nleucogenys
  419492: ...................G......................------A.  Mmulatta
  370154: ...................G......................------A.  Panubis
  403933: .............G.....G........A....C........------A.  Cjacchus
 1050720: ......A............G........AC.....T......------A.  Sboliviensis

                       LINE2
          ------------------------------------------------->
                53471     53481     53491     53501     53510
   53462: TCTTATTCTTATTCTCACTCTTAGTCAATCAATGAAAAAA-TCCTGTTTT  Hsapiens
  373202: ................GT.....C................-.......C.  Ptroglodytes
  391248: ................GT.....C...............G-.........  Ggorilla
  422490: .................T.....T.......T........-.........  Pabelli
  344268: .................T.....T................-........A  Nleucogenys
  419536: ........------------------.....G........A.........  Mmulatta
  370198: ........------------------.....G........A.........  Panubis
  403977: ........------------------.G............A.........  Cjacchus
 1050764: .....C..------------------..............A.T.......  Sboliviensis

                       LINE2
          ------------------------------------------------->
                53520     53530     53540     53550     53560
   53511: CCCCATCTTTGGAAATATTCCCCATCTGCACATTTCTCATGCTCCCCAGT  Hsapiens
  373251: ..................................................  Ptroglodytes
  391297: ..................................................  Ggorilla
  422539: ..................................................  Pabelli
  344317: ...........................................T...T..  Nleucogenys
  419568: .........................................T........  Mmulatta
  370230: .....................T............................  Panubis
  404009: ...................T..T...........................  Cjacchus
 1050796: .....................T.......................T....  Sboliviensis

                       LINE2
          ------------------------------------------------->
                53570     53580     53590     53600     53610
   53561: TTTAACAGCATGAACGGTGCTCCTGAATTACTGAAATAATCTCCTATCTT  Hsapiens
  373301: ..................................................  Ptroglodytes
  391347: ...........................................G......  Ggorilla
  422589: ..................................................  Pabelli
  344367: ..................................................  Nleucogenys
  419618: ...........A..-...............T.T.................  Mmulatta
  370280: ...........A..-.................T.................  Panubis
  404059: ...C.T.......GA...............................A..G  Cjacchus
 1050846: .....T.......G-..G......................A.....A..G  Sboliviensis
```

```
                              LINE2
          ------------------------------------------------->
               53620     53630     53640     53650     53660
   53611: CTTTCTACTCCAGTCTTTATCCTCCTCCATAATATCTTTGATAGACTAGT  Hsapiens
  373351: ..................................................  Ptroglodytes
  391397: ..................................................  Ggorilla
  422639: T...........................G................T....  Pabelli
  344417: .........G........................................  Nleucogenys
  419667: ..............................G...................  Mmulatta
  370329: ..............................G...................  Panubis
  404109: .......G....T......C..........G.............        Cjacchus
  404482:                                             ......  Cjacchus
 1050895: ...................C..........G....T..............  Sboliviensis

                              LINE2
          ------------------------------------------------->
               53668     53678     53688     53698     53708
   53661: ATTTTTT--AAATATCAGTTGGTTAGGTACTCCTCTGCTCAAAAACTCCA  Hsapiens
  373401: .......TA...........A.............................  Ptroglodytes
  391447: ......A--...........A.............................  Ggorilla
  422689: ......A--...........A.............................  Pabelli
  344467: ......A--...........A........T...C..T.............  Nleucogenys
  419717: .......--..G..A.....A.............................  Mmulatta
  370379: .......--..G........A.............................  Panubis
  404488: .......--...........A....T........................  Cjacchus
 1050945: G..G...                                             Sboliviensis

                              LINE2
          ------------------------------------------------->
               53718     53728     53738     53748     53758
   53709: CTGTCTTCCCTTTTACTCAGAGAAGAAGACAAAGTCCTTACTGTGACCAG  Hsapiens
  373451: .................................................A  Ptroglodytes
  391495: .......................................G.........A  Ggorilla
  422737: ............................C....................A  Pabelli
  344515: ............................C....................A  Nleucogenys
  419765: ....................G..G....C....................A  Mmulatta
  370427: ....................G..G....C....................A  Panubis
  404536: ...G..........TT............C......G.............C  Cjacchus

                              LINE2
          ------------------------------------------------->
               53768     53778     53788     53798     53808
   53759: CAAGACCCTCTGTGATCTGGCCCTGGCCACCTGTCAGACCTAATCTCCTA  Hsapiens
  373501: .........................................C........  Ptroglodytes
  391545: .........................................C........  Ggorilla
  422787: ....G..................................T.C........  Pabelli
  344565: ....G....................................C........  Nleucogenys
  419815: ....G....................................C........  Mmulatta
  370477: ....G....................................C........  Panubis
  404586: .G.....................................T.C........  Cjacchus
```

```
                                    LINE2
                 53818     53828     53838     53848     53858
 53809: CCACTCTCCCCCAAGCTCACTCCAATTTAGCCATGTAACCTCCTTGCATA Hsapiens
373551: ..........A....................................G.. Ptroglodytes
391595: ..........A....................................... Ggorilla
422837: ..........A....................................... Pabelli
344615: T.........A...........................A........... Nleucogenys
419865: .......T..A.C..................T....C............. Mmulatta
370527: .......T..A.C.........T...........A.C............. Panubis
404636: ..........A.C...........C...........C...C........C Cjacchus
```

```
                                    LINE2
                 53868     53878     53888     53898     53908
 53859: CCTTCAACATAACAAGTACATTCAACCTCAGGGCATTTGCACTTTCTATT Hsapiens
373601: .................................................. Ptroglodytes
391645: .................................................. Ggorilla
422887: ...................G.............................. Pabelli
344665: .........................TG............T.......... Nleucogenys
419915: T...............C................................. Mmulatta
370577: ................C................................. Panubis
404686: .T....G.........C............GA................... Cjacchus
```

```
                                    LINE2
                 53918     53928     53938     53948     53958
 53909: CCTCTTCCATGAAGTTCTTCCTGGAGATATTTGAATGGCTCATTCCAGCA Hsapiens
373651: .....................A.A.......................... Ptroglodytes
391695: .......................A.......................... Ggorilla
422937: .......................A.............T............ Pabelli
344715: .......................A.......................... Nleucogenys
419965: ............T........C.A..............T..C........ Mmulatta
370627: .......G....T........C.A..............T..C........ Panubis
404736: .........A..T..........A......C..................C Cjacchus
```

```
                                    LINE2
                 53968     53978     53988     53998     54008
 53959: GTTTAATCATCTCTGTTCCGATAGCATCTCATTACTAAGGCTCTCCCTGA Hsapiens
373701: .................................................. Ptroglodytes
391745: .................................................. Ggorilla
422987: A.................T.....................T......... Pabelli
344765: ..................TA..........G.G................. Nleucogenys
420015: .C........A.......A..C........G...........G....... Mmulatta
370677: ..........A.......A.......................G....... Panubis
404786: AC................A............................... Cjacchus
```

```
                                    LINE2
                 54018     54028     54038     54048     54058
 54009: CCATTAATTCTGTTTGAAATCTCAGCCCACATCCCTCCATTCCATTACCA Hsapiens
373751: ...............A.................................. Ptroglodytes
391795: .................................................. Ggorilla
423037: ...........................A....G................. Pabelli
344815: .................................................. Nleucogenys
420065: .....C.............................A.............G Mmulatta
370727: .....C......................T......A.............G Panubis
404836: T.----.....A...............T....................TG Cjacchus
```

```
                                  LINE2
        ------------------------------------------------->
                  54068     54078     54088     54098     54108
                      :         :         :         :         :
   54059: TAGCACATACCACCACCTGGCTTACTACATATGTATTTATTATCTGATTC  Hsapiens
  373801: ..................................................  Ptroglodytes
  391845: .....T............................................  Ggorilla
  423087: ................................A.................  Pabelli
  344865: ....................T......A......................  Nleucogenys
  420115: .............................G....................  Mmulatta
  370777: ..................................................  Panubis
  404882: .................G...........C.............A......  Cjacchus

                                  LINE2
        ------------------------------------------------->
                  54118     54128     54138     54148     54158
                      :         :         :         :         :
   54109: CCTTCCCTGTATAGTACATTACACCAAGGTCTTTGTTTTGTTTTTATGTT  Hsapiens
  373851: ........C.....C..............A....................  Ptroglodytes
  391895: ........C....................A....................  Ggorilla
  423137: ........C....................A....................  Pabelli
  344915: ........C....................A....................  Nleucogenys
  420165: ........C............G.G.....A...C......G.........  Mmulatta
  370827: ........C............G.G.....A...CA.....G.G...C...  Panubis
  404932: ...C....C............G.A.....A.------..........A..  Cjacchus
 1052163:                                  .................  Sboliviensis

                                  LINE2
        ------------------------------------------------->
                  54168     54178     54188     54198     54208
                      :         :         :         :         :
   54159: TTATTCCAGCATCTAGACAAGCTCTATCCATAGAGTAGTGACCCAGTACA  Hsapiens
  373901: ..........................C.....C.................  Ptroglodytes
  391945: ..................................................  Ggorilla
  423187: ................................C.................  Pabelli
  344965: ...C......................................T.......  Nleucogenys
  420215: .............C.......TC...........A...............  Mmulatta
  370877: .............C.......TC...........A...............  Panubis
  404976: ......................C...........AC..CC..T.TA....  Cjacchus
 1052180: ......................C.......C...A...CC..T.TA....  Sboliviensis

                 LINE2               LINE2
        -------------------->----------------------------->
                  54218     54228     54238     54248     54258
                      :         :         :         :         :
   54209: TGTTTGCAGAAGGAAGAAATGAAGAAGAGAATTTCAATCCAATAAGTACT  Hsapiens
  373951: ..................................................  Ptroglodytes
  391995: ..................................................  Ggorilla
  423237: ......TG..........................................  Pabelli
  345015: .......G..........................................  Nleucogenys
  420265: ...........A....................A.................  Mmulatta
  370927: .........................................G........  Panubis
  405026: .....A...........T.........G.............G....C.TC  Cjacchus
 1052230: .....A...........CG........G........G....G........  Sboliviensis
```

```
                                   LINE2
          ---------------------------------------------------------->
                   54268     54278                   54292     54302
   54259: GTGTGATATGCTGGTATGCACATAATC------AAGTAGAGAGGAGAGAC  Hsapiens
  374001: ...........................------.................  Ptroglodytes
  392045: ...........................------.................  Ggorilla
  423287: A...........A..C...........------................T  Pabelli
  345065: A..............C...........------.................  Nleucogenys
  420315: A..........................------................G  Mmulatta
  370977: A..........................------................G  Panubis
  405076: A..--..........G......G....CTGGTA...G.............  Cjacchus
 1052280: A..--..........G....T.G...ACTGTTA...G.............  Sboliviensis

                                   LINE2
          ---------------------------------------------------------->
                   54312     54322     54332     54342     54352
   54303: ACAGAACCAAACTGAGAATTATAGTTAGCCTTCCTGGAGGAGATAATATC  Hsapiens
  374045: ..................................................  Ptroglodytes
  392089: ...................................A..............  Ggorilla
  423331: ...A.C.....................................G......  Pabelli
  345109: ...AGC............................................  Nleucogenys
  420359: .................G................................  Mmulatta
  371021: .................G................................  Panubis
  405124: .........GGA.A...G...C.....A....A...A.------......  Cjacchus
 1052328: .........GGG.AG..G...C..........A...A.------......  Sboliviensis

                                   LINE2
          ---------------------------------------------------------->
                   54362     54372     54382     54392     54402
   54353: CCTGCTGCTTCTAGAAGAATGAAATAAGCATCACCTAGGTATATACATGT  Hsapiens
  374095: ..................................................  Ptroglodytes
  392139: ..................................................  Ggorilla
  423381: ..................................................  Pabelli
  345159: .............................G....................  Nleucogenys
  420409: ...................................C.T............  Mmulatta
  371071: ..........T........................C.T............  Panubis
  405168: .................................T.C.A......G.....  Cjacchus
 1052372: .................................TTC.A............  Sboliviensis

                                   LINE2
          ---------------------------------------------------------->
                   54412     54422     54432     54442     54452
   54403: AAGAAGAGCACTCCTATCAGAAGGAATAGCATGAGCAGAGACTCAAACAC  Hsapiens
  374145: ...............G..................................  Ptroglodytes
  392189: ...............G..................................  Ggorilla
  423431: ...............G..................................  Pabelli
  345209: ...............G.........................T........  Nleucogenys
  420459: ...............G................A.................  Mmulatta
  371121: ...............G................A.................  Panubis
  405218: .G...........T.G.T.............A.......AC.........  Cjacchus
 1052422: .G.............G.......................AC.........  Sboliviensis
```

LINE2 →

```
              54462     54472     54482     54492     54502
  54453: CTGAAAGATAAATTTTATATTACAAATGCATGAAATAAGAGTCCAAAAGT  Hsapiens
 374195: ..................................................  Ptroglodytes
 392239: ..................................................  Ggorilla
 423481: ......................T...........................  Pabelli
 345259: ......................T................T..........  Nleucogenys
 420509: ......................T..G........................  Mmulatta
 371171: ......................T..G..................C.....  Panubis
 405268: .C....................T...................T.......  Cjacchus
1052472: .CA.......C...........T..............G....G.......  Sboliviensis

              54512     54522     54532     54542     54552
  54503: GGTGGGCAGTGGGATTGAAGATGGGCAGGACTGTGGCCTTAGAAGCCTTG  Hsapiens
 374245: ..................................................  Ptroglodytes
 392289: .....A............................................  Ggorilla
 423531: .............G........A...........................  Pabelli
 345309: ........A....G.CA.....A...........................  Nleucogenys
 420559: .....A....A..GCCA..T..A.........A....T..........G.  Mmulatta
 371221: .........AA..GCCA..T..A.........A...............G.  Panubis
 405318: ......A...A.AGC....A.AA.TTG.........T...G.....T...  Cjacchus
1052522: ..........A.AGC....A..A.T...T...........G.....T...  Sboliviensis

              54562     54572     54582     54592     54602
  54553: CATGCCCCTTGATGAGAAATCAGAGATCACACCCCACATAGCTCATGATG  Hsapiens
 374295: ....T..........................................G..  Ptroglodytes
 392339: ..................................................  Ggorilla
 423581: ..................................................  Pabelli
 345359: ......T...........................................  Nleucogenys
 420609: ..................................................  Mmulatta
 371271: ..................................................  Panubis
 405368: ..A.T.......G..........C........T...G............A  Cjacchus
1052572: ............G.......................G............A  Sboliviensis

              54612     54622     54632     54642     54652
  54603: GAGTTGGGTCTTCATGTCACCTAGTCAGAAGTTCATATCATCTTTCTGAT  Hsapiens
 374345: ..................................................  Ptroglodytes
 392389: ..................................................  Ggorilla
 423631: ........................................C.........  Pabelli
 345409: ..................................................  Nleucogenys
 420659: .......T....T.......T.............................  Mmulatta
 371321: .......T....T.......T.............................  Panubis
 405418: ..A.............C....C..........-.................  Cjacchus
1052622: ..A.............C..........A....-........T.......G  Sboliviensis

              54662     54672     54682     54692     54702
  54653: AATCAATTTCTAAATCTCTCTCCTTCTCTTCTTCTCTCTCCCCAGACAGC  Hsapiens
 374395: ..............................................-...  Ptroglodytes
 392439: ..............................................-...  Ggorilla
 423681: ..................................................  Pabelli
 345459: ......C...............T...............-----.......  Nleucogenys
 420709: C........A................................TG......  Mmulatta
 371371: C........A................................TG......  Panubis
 405467: ...........C.............T.......---............A.  Cjacchus
1052671: .................................T.T............A.  Sboliviensis
```

```
                                    LTR
                  54715     54725     54735     54745
   54703: ATGTT-------AAAAAATGTTGGTTGCCCACCATTCAGCCCTTCCCTTT  Hsapiens
  374444: ..A..-------TT....A......A.....G..................  Ptroglodytes
  392488: ..A..-------TT....A..G...A.....G..................  Ggorilla
  423731: ..A..------A......A....A.......................G..  Pabelli
  345504: ..A..------A......A...............................  Nleucogenys
  420759: ..A..AAAAAAA....C.G.....C.........................  Mmulatta
  371421: ..A..-AAAAAA....C.G.....C.........................  Panubis
  405514: ..A..-AAAGAA......A.....C.......TTC...............  Cjacchus
 1052721: ..A..--AGAGA...............A.....C....A..........G  Sboliviensis

                          LTR
        54755     54765     54775     54785     54795
   54746: CACTCCCTTGTTTTGGGCTAAGAGAACTTGCTTTAATGCAGATTTCATGG  Hsapiens
  374487: .................................................C  Ptroglodytes
  392531: .................................................C  Ggorilla
  423775: ...............T..................G..............C  Pabelli
  345548: ..............TT..................G..............C  Nleucogenys
  420809: ...............T.............C....G..............C  Mmulatta
  371470: ...............T.........G...C....G..............C  Panubis
  405563: .....AG........T...........CC.....G..............C  Cjacchus
 1052769: ...G.GA........T.....A.....C....C.G..............C  Sboliviensis

                          LTR
        54805     54815     54825     54834     54844
   54796: AGTCATGTGCTTCAGAGGAGGCTTCACCCTTC-TCAGGCCCCAAGAGCAA  Hsapiens
  374537: ................................-.................  Ptroglodytes
  392581: ................................-.........G.......  Ggorilla
  423825: ..C.............................-..............G..  Pabelli
  345598: ..C.............................-.................  Nleucogenys
  420859: ..C.............................C.................  Mmulatta
  371520: ..C.............................C.................  Panubis
  405613: ..C....G........................-C............A...  Cjacchus
 1052819: ..C....G........................-C............A...  Sboliviensis

                          LTR
        54854     54864     54874     54884     54894
   54845: GCCTGGATTAGTCTAAGCCAATTGAGGAAATTCTATGTCCCAAGTTTCTA  Hsapiens
  374586: ..................................................  Ptroglodytes
  392630: ..................................................  Ggorilla
  423874: ..................................................  Pabelli
  345647: .................................................G  Nleucogenys
  420909: ....................C......G.......G..............  Mmulatta
  371570: ...........................G......................  Panubis
  405662: ....................G......G.........C............  Cjacchus
 1052868: ....A.............A.G......G......................  Sboliviensis
```

```
                                   LTR
          ------------------------------------------------------------>
                  54904     54914     54924     54934     54944
                      :         :         :         :         :
  54895: GTGTAGGAGTCAGAAAGTGTCATAATTCTTACGAGAAAGACAGGAGAGGT  Hsapiens
 374636: ...................C.G..........A.................  Ptroglodytes
 392680: ..T................C............................T.  Ggorilla
 423924: ...................C............A.................  Pabelli
 345697: ...................C............A..............T..  Nleucogenys
 420959: ...............G...C.......TG...A.................  Mmulatta
 371620: ...............G...C........G...A.................  Panubis
 405712: ...............G..AC.G...G......TG.........A...T..  Cjacchus
1052918: ...............G..AC....TG......TG........AA...C..  Sboliviensis

                                   LTR
          ------------------------------------------------------------>
                  54954     54964     54974     54984     54994
                      :         :         :         :         :
  54945: TGCTGGAAGGCTTCTAGAAAAGGCTTCTAATAAGAAGAGATCCACAAGAG  Hsapiens
 374686: .......G..............A...........................  Ptroglodytes
 392730: .......G................................C.........  Ggorilla
 423974: .......G...................CT...........C.........  Pabelli
 345747: .A.....G....................G.....................  Nleucogenys
 421009: .......G.......G...........C................A.....  Mmulatta
 371670: .......G.......G...........CG.....................  Panubis
 405762: .......G.....T.G............G...T..............A..  Cjacchus
1052968: .............T.G............G...T..............A..  Sboliviensis

                                   LTR
          ------------------------------------------------------------>
                  55004     55014     55024     55034     55044
                      :         :         :         :         :
  54995: GAAACCAGCCCTCTTCTTCCTCTAACACTTGGGTTATCTGCATGTGTCTA  Hsapiens
 374736: .......................C..........................  Ptroglodytes
 392780: .......................C..........................  Ggorilla
 424024: .......................C...G......................  Pabelli
 345797: .......................C...G......................  Nleucogenys
 421059: .......T...............C...G......................  Mmulatta
 371720: .......T...............C...G......................  Panubis
 405812: ..........-............C...AAA.........A......A...  Cjacchus
1053018: A.........-............C...G..................A...  Sboliviensis

                                   LTR
          ------------------------------------------------------------>
                  55054     55064     55074     55084     55094
                      :         :         :         :         :
  55045: CTACTGCAGCTGCTATTTTGTGTCCGTGAGGTAGCTAGTCTAAGAGGATA  Hsapiens
 374786: ..................................................  Ptroglodytes
 392830: .......................A.....................T....  Ggorilla
 424074: .........................A........................  Pabelli
 345847: ............G.....................................  Nleucogenys
 421109: ......................A..ACA...A..G....T..........  Mmulatta
 371770: ......................A..ACA.C.A..G....T..........  Panubis
 405861: .......G..A...........A..A.........C..............  Cjacchus
1053067: ..........A...........A..A.........C..............  Sboliviensis
```

```
                  LTR
          ------------------------------------------------->
           55104     55114     55124     55134     55143
               :         :         :         :         :
  55095: ACCCAGGATTTCAGAGAAGAAGGACAGGCAGAACTTGCGTCCTCGA-TAA  Hsapiens
 374836: .G...............G............................-...  Ptroglodytes
 392880: .G...............G..........................A.-...  Ggorilla
 424124: .G.............A.G.......G.A......G...........G...  Pabelli
 345897: .G...............G.........A..........A.......G...  Nleucogenys
 421159: .G...............G......TG.A..........A.T.....-.G.  Mmulatta
 371820: .G...............G......TG.A..........A.T...A.-.G.  Panubis
 405911: .................G.......G.A.........G.....T..-.GG  Cjacchus
1053117: .................C.......G.A.G.......G.....T..-.G.  Sboliviensis

                  LTR
          ------------------------------------------------->
           55153     55163     55173     55183     55193
               :         :         :         :         :
  55144: CACACCATTGAGTGAATTGATTACCCAATCCTGGATCTGCCCTAGCTTGA  Hsapiens
 374885: ..................................................  Ptroglodytes
 392929: ..................................................  Ggorilla
 424174: ..............T...................................  Pabelli
 345947: ..............T...................................  Nleucogenys
 421208: .................G................................  Mmulatta
 371869: .................G................................  Panubis
 405960: .................G.......T........................  Cjacchus
1053166: .............A...G.......T............A...........  Sboliviensis

                  LTR
          ------------------------------------------------->
           55203     55213     55222     55232     55242
               :         :         :         :         :
  55194: GATTTCTTTTCATGAGAAATAATAA-TTTTTGCTTATTGTTTAAGCCATT  Hsapiens
 374935: ..............T..........-.....T..................  Ptroglodytes
 392979: ............C.T..........-.....T..................  Ggorilla
 424224: ..............T.........T-.....T......A...........  Pabelli
 345997: ....C.........T..........-.....T..................  Nleucogenys
 421258: .......C......T....C.....-.....T..................  Mmulatta
 371919: .......C......T....C.....-.....T..................  Panubis
 406010: ..............T..........T.....T..................  Cjacchus
1053216: ..............T..........-C....T....C.............  Sboliviensis

               LTR
          ------------------------------------------->
           55252     55262     55272     55282     55292
               :         :         :         :         :
  55243: TTAGTTGAGTCTTCTGTTACTTGCAGCTAAAAGAATTCTAACTCACTTCA  Hsapiens
 374984: C.G...............................................  Ptroglodytes
 393028: ..................................................  Ggorilla
 424273: .............TC..........A..G.....................  Pabelli
 346046: ..................................................  Nleucogenys
 421307: ...............................T..................  Mmulatta
 371968: ...............................C..................  Panubis
 406060: G................................C................  Cjacchus
1053265: G.T...C..........................C................  Sboliviensis
```

```
              55302     55310     55320     55330     55340
                  :         :         :         :         :
   55293: AGGGTTTTTCTATA--CACCCTTGTTCACTAGGGCCAGTTAGCCCACTTA  Hsapiens
  375034: ..............--................A....A............  Ptroglodytes
  393078: ..............--.....................A............  Ggorilla
  424323: ..............--.....................A............  Pabelli
  346096: ..............--A....................A............  Nleucogenys
  421357: ......G.......--......A..............A......T.....  Mmulatta
  372018: ......G.......--......A..............A......T.....  Panubis
  406110: ..............TT.....................A............  Cjacchus
 1053315: .............T--........C............A............  Sboliviensis

                        55359     55369     55379     55389
                            :         :         :         :
   55341: CTCCACATT-CACCTTTCTGTAGCATAGTTACATTATTCCCTTGTTTAGT  Hsapiens
  375082: .........-........................................  Ptroglodytes
  393126: .........-........................................  Ggorilla
  424371: .........-........................................  Pabelli
  346144: .........-.............................T..........  Nleucogenys
  421405: .........-....G..G................................  Mmulatta
  372066: .........-....G............C......................  Panubis
  406160: ......T..C.C..C.............CC......C.............  Cjacchus
 1053363: ......G..-.C..........A.....CC...C...AA..-........  Sboliviensis

              55399     55409     55419     55429     55439
                  :         :         :         :         :
   55390: CCACCTGGAATACAGAAATATTTGATTCTTTTACCTCCTATCTTCTAGAT  Hsapiens
  375131: .....................G............................  Ptroglodytes
  393175: ..................................................  Ggorilla
  424420: ........G............................AC......C....  Pabelli
  346193: ........G.............................C...........  Nleucogenys
  421454: ........G...............G...........T.C..........C  Mmulatta
  372115: ........G...............G...........T.C..........C  Panubis
  406210: ........G......-.......TG.............C...........  Cjacchus
 1053411: ........G..C...........TG.............C...........  Sboliviensis

              55449     55459     55469     55479     55489
                  :         :         :         :         :
   55440: TACAGCTTTGCCCTCTTTTAGAATCCAAGTCTAAGCATATAGGCTATAGG  Hsapiens
  375181: .........................................T........  Ptroglodytes
  393225: ..................................A......T........  Ggorilla
  424470: .........................................T...G....  Pabelli
  346243: ..T......................................T........  Nleucogenys
  421504: .........................................T........  Mmulatta
  372165: .........................................T........  Panubis
  406259: .........................TG.........C....CA..C..C.  Cjacchus
 1053461: ..........................G.........C....T...C..T.  Sboliviensis

              55499     55509     55519     55529     55539
                  :         :         :         :         :
   55490: ACACTGTGACAAAGGCCAATACATACCCCTGCCAATACATATTTATCCTA  Hsapiens
  375231: .........................T........................  Ptroglodytes
  393275: .........................T........................  Ggorilla
  424520: .........................T........................  Pabelli
  346293: ....A..A.................T.......G................  Nleucogenys
  421554: .....A..................TT...G........G.........C.  Mmulatta
  372215: .....A..................TT...G........G.........C.  Panubis
  406309: .....A.......A...........T...CA................TG.  Cjacchus
 1053511: .....A...................T...C.......TG...........  Sboliviensis
```

```
              55549     55559     55569     55579     55589
   55540: GTTACTAATGTATCTTATTTCCAGTCAGTACGTATCTGAGCTGAGCTTTC  Hsapiens
  375281: .....C.........................A..................  Ptroglodytes
  393325: .........A.G........T..........A..................  Ggorilla
  424570: .....CG........................A..................  Pabelli
  346343: ...............................A..................  Nleucogenys
  421604: ...G......                                          Mmulatta
  372265: ...G......                                          Panubis
  406359: ........A..G.........G...T...G.A..................  Cjacchus
 1053561: .....G.....G.........T...T.............T..........  Sboliviensis

              55599     55609     55619     55629     55639
   55590: TGGAGATGTGTTCACTGGGATATGTCCTTGTTTGTCATCAATTTAGGCAT  Hsapiens
  375331: .............G....................................  Ptroglodytes
  393375: .............G....................................  Ggorilla
  424620: .............T....................................  Pabelli
  346393: ............TT.........................G..........  Nleucogenys
  406409: .............T.....G..C...........................  Cjacchus
 1053611: .......T.....T.....G.........A....................  Sboliviensis

              55649     55659     55669     55679     55689
   55640: TTATGTCATGCTTTTAATCTTTTATCAGGCAGAGACAGACCAAAAGGGAA  Hsapiens
  375381: ................G..C..............................  Ptroglodytes
  393425: ................G..C..............................  Ggorilla
  424670: ...................C..C............G..............  Pabelli
  346443: ...............C...C..............GG..............  Nleucogenys
  406459: .C........T....C...C..A............G......G.......  Cjacchus
 1053661: ...............C...C...............G......G.......  Sboliviensis

              55699     55709     55719     55729     55739
   55690: TATATGTCACTTGTGGACTTTCAAAATAATGACCCATGACATTTATATAG  Hsapiens
  375431: ..................................................  Ptroglodytes
  393475: ....C.............................................  Ggorilla
  424720: .....A.........................G.T................  Pabelli
  346493: ...............................G..................  Nleucogenys
  406509: .......G............AG.C..A....G...C...T..........  Cjacchus
 1053711: .......G............A..........G..................  Sboliviensis

              55749     55759     55769     55779     55789
   55740: GAAAATTCACAAATCCTGTAATCAAAACCAGTTGCCCTAAAATAATCATT  Hsapiens
  375481: ..................................................  Ptroglodytes
  393525: ..................................................  Ggorilla
  424770: ......G...........................................  Pabelli
  346543: ................................................C.  Nleucogenys
  406559: ....................................T.............  Cjacchus
 1053761: .................A........G.......................  Sboliviensis

              55799     55809     55819     55829     55839
   55790: AAGGAGTAGATGCTGTGCATTCCAGTTCTCAGAAAGCCCAGCACAGTGTA  Hsapiens
  375531: ..................G...............................  Ptroglodytes
  393575: ..................G...............................  Ggorilla
  424820: -....A...........................C................  Pabelli
  346593: ...TC.............G...............................  Nleucogenys
  406609: .................TT.............GC.............C..  Cjacchus
 1053811: ...T..G..........TG.............GC.............CC.  Sboliviensis
```

```
              55849     55859     55869     55879     55889
                  :         :         :         :         :
  55840: ATAGGACCCAGTAGTTATACAGCACCAAGCCTGGACCACAGTTTTCTTTG  Hsapiens
 375581: ...........C............................C.........  Ptroglodytes
 393625: ...........C............................C.........  Ggorilla
 424869: ...........C.....C..................T...C.........  Pabelli
 346643: ...........C............................C.........  Nleucogenys
 421609:            ..A.G....TA..........T.......C.........  Mmulatta
 372270:            ..A.G....TA..........A.......C.........  Panubis
 406659: .....G.....C........TAT....G...C........CC........  Cjacchus
1053861: ....AG..T..C........TAT....G............C.........  Sboliviensis

              55899               55918     55928     55938
                  :                   :         :         :
  55890: CTCTTGACAGTTTTTAAAA-AATATAACAGAAGGGTTTTACTCATCTTAT  Hsapiens
 375631: ...................-.............C................  Ptroglodytes
 393675: ...................-.........................T....  Ggorilla
 424919: ...................-..............................  Pabelli
 346693: ...................-..............................  Nleucogenys
 421648: ...............TT..-.....G......A.........A..A....  Mmulatta
 372309: ...............T...-.....G......A.........A..A.C..  Panubis
 406709: G.T...........A....-T.......G.............A......C  Cjacchus
1053911: T........A....A....C......................A.......  Sboliviensis

              55948
                  :
  55939: TTTGCTAAAA----------------------------------------  Hsapiens
 375680: ..........----------------------------------------  Ptroglodytes
 393724: ..........----------------------------------------  Ggorilla
 424968: ..........----------------------------------------  Pabelli
 346742: ..........TACAATTGCTAAAATACAATACCAAGCCTGGACCACACTT  Nleucogenys
 421697: ..........----------------------------------------  Mmulatta
 372358: ..........----------------------------------------  Panubis
 406758: ..........----------------------------------------  Cjacchus
1053961: ..........----------------------------------------  Sboliviensis


  55949: --------------------------------------------------  Hsapiens
 375690: --------------------------------------------------  Ptroglodytes
 393734: --------------------------------------------------  Ggorilla
 424978: --------------------------------------------------  Pabelli
 346792: TTCTTTGCTCTTGACAGTTTTAAAAAAATATAACAGAAGGGTTTTACTCA  Nleucogenys
 421707: --------------------------------------------------  Mmulatta
 372368: --------------------------------------------------  Panubis
 406768: --------------------------------------------------  Cjacchus
1053971: --------------------------------------------------  Sboliviensis

                        55952     55962     55972     55982
                            :         :         :         :
  55949: ----------------TACAATTCTGTACCTTTGACAGGATGTTTTCCTT  Hsapiens
 375690: ----------------.......A..........................  Ptroglodytes
 393734: ----------------.......A.A.T......................  Ggorilla
 424978: ----------------.......A....T.....................  Pabelli
 346842: TCTTATTTTGCTAAAA.......A..............T...........  Nleucogenys
 421707: ----------------.....C.A.......C..G...A...........  Mmulatta
 372368: ----------------.....C.A.......C..G...A...........  Panubis
 406768: ----------------.......G....G.......G.......G.....  Cjacchus
1053971: ----------------.......G.........A..........G.....  Sboliviensis
```

```
                55992     56002     56012     56022     56032
                    :         :         :         :         :
  55983: TAAGGTGATTTTAATACAGCTCTCTGTATTATGTGGTAATAACAGTGT  Hsapiens
 375724: C.............................................A...  Ptroglodytes
 393768: C.................................................  Ggorilla
 425012: C..............................C..A...........C..C  Pabelli
 346892: C..............................C............T.....  Nleucogenys
 421741: C.........................G....CA......C..G.......  Mmulatta
 372402: C.........................G....CA......C..G.......  Panubis
 406802: C......G...................CAC.CA...A......C.C....  Cjacchus
1054005: C...........................AC.CA...A......CTC....  Sboliviensis

                56042     56052     56062     56072     56082
                    :         :         :         :         :
  56033: CTTTGACAGCCCTTTGTTTAGTTACTTTAATAATGAAAGCACTTCCAATC  Hsapiens
 375774: .....................C..T.........................  Ptroglodytes
 393818: .................................................T  Ggorilla
 425062: ..................................................  Pabelli
 346942: ............A.....................................  Nleucogenys
 421791: ..............CA..................................  Mmulatta
 372452: ..............C...................................  Panubis
 406852: .CG...A...T........CA............................A  Cjacchus
1054055: ..G...A...T......................C...............A  Sboliviensis

                56092     56102     56112     56122     56132
                    :         :         :         :         :
  56083: GTGAAGTATATCCAAGTCAGTTGCTCTTCACACTATGTTCTGCATTTTAG  Hsapiens
 375824: ..............................TG..................  Ptroglodytes
 393868: ...............................G.................C  Ggorilla
 425112: ........C.....G..................--...C...........  Pabelli
 346992: ..................G...............................  Nleucogenys
 421841: .....A.............A..............................  Mmulatta
 372502: ...................A..............................  Panubis
 406902: ..A.................C.........T...G.AG.......G....  Cjacchus
1054105: ..A.................C.......T.T.....AG.......G....  Sboliviensis

                56142     56152     56162     56172     56182
                    :         :         :         :         :
  56133: TGAATTAAGTAAGCTAGTGGAGTTGCATTCTGGAAAGTAACGTGAATTTA  Hsapiens
 375874: .........A.......C.......................A.A......  Ptroglodytes
 393918: .............T.............................A.....G  Ggorilla
 425160: ...............G.....-...T.........T....T..A......  Pabelli
 347042: .........G...............T.........T....T..A..----  Nleucogenys
 421891: ....C....................T..............T..A...G..  Mmulatta
 372552: .........................T.......C......T..A...G..  Panubis
 406952: .....G...................T.........T.G..T..A......  Cjacchus
1054155: .....G...................T.C.......T.G..T..A......  Sboliviensis

                56192     56202     56212     56222     56232
                    :         :         :         :         :
  56183: TTTATCTTAAATTTATGTCCTGTTGTTGTTATAAAATCGCCTATTCACAT  Hsapiens
 375924: ...................A..............................  Ptroglodytes
 393968: ...................A..................A...........  Ggorilla
 425209: ...................A..............................  Pabelli
 347088: ...................A..............................  Nleucogenys
 421941: ..............G.A..A..............................  Mmulatta
 372602: ................A..A......---.....................  Panubis
 407002: ......------.......A......A.C........A....G...G...  Cjacchus
1054205: ......------...C...A......A.........CA....G.......  Sboliviensis
```

```
              56242     56252     56262     56272     56282
  56233: ATCATAACTATTGTAGCCCTGCAGTTACTAGAGCACCTAAGAAAAGAGTT  Hsapiens
 375974: ..............G...................................  Ptroglodytes
 394018: ..................................................  Ggorilla
 425259: .............................................T....  Pabelli
 347138: ...............................................C..  Nleucogenys
 421991: ......G...........................................  Mmulatta
 372649: ......G...........................................  Panubis
 407046: ------G.C.C...........................G...........  Cjacchus
1054249: ------..C..............................T..........  Sboliviensis

              56292     56302     56311     56321     56331
  56283: TGGAATACACTGATAGGCCACTT-ATTGTGTGCCAGACACTTTTATTAGT  Hsapiens
 376024: .......................-..........................  Ptroglodytes
 394068: .......................-..........................  Ggorilla
 425309: .......................-..........................  Pabelli
 347188: .......................A.C...T....................  Nleucogenys
 422041: .......TG..............-.C......GG...............G  Mmulatta
 372699: ........G..............-.C......G..............T..  Panubis
 407090: .............C.........-.A..........G....C........  Cjacchus
1054293: .............C.........-............G.............  Sboliviensis

              56341     56351     56361     56371     56381
  56332: CTGTTTATTTGATTGTTTCTTCTTTTATAACCACAAGTACACAAGGTTGA  Hsapiens
 376073: ...........T...........................T..........  Ptroglodytes
 394117: ...........T......................................  Ggorilla
 425358: ...........T...........................T..........  Pabelli
 347238: ...........T...........................T..........  Nleucogenys
 422090: ...........T......................................  Mmulatta
 372748: ...........T......................G...............  Panubis
 407139: ........C..T...............C.G.......C.T..........  Cjacchus
1054342: .......--..T...............C.G.......C.T..........  Sboliviensis

              56391     56401     56411     56421     56431
  56382: GGTCATCACATGCTCTGGTTCTTGGCATAAGCCATGACTGGATTAGAAGG  Hsapiens
 376123: ...................................C..............  Ptroglodytes
 394167: .......G...........................C..............  Ggorilla
 425408: ...................................C.........C....  Pabelli
 347288: ...................................C.........C....  Nleucogenys
 422140: ...........A...............A.......C.........C....  Mmulatta
 372798: ...........A...............A.......C.........C....  Panubis
 407189: ......TG......TCA.....CA...........TC........C....  Cjacchus
1054390: ......TG......TC.......A...........C.........C...A  Sboliviensis

                                                    LINE1
                                                    ---->
              56441     56451     56461     56471     56481
  56432: CCATTCTCTTCTTTACTTATGGCCTTTGACCCAGTTCATTTCCTTTCTTC  Hsapiens
 376173: ......................T...........................  Ptroglodytes
 394217: ...C........................................---...  Ggorilla
 425458: ....A..............C..............................  Pabelli
 347338: ..................................................  Nleucogenys
 422190: ..................................G...............  Mmulatta
 372848: ..................................................  Panubis
 407239: ..........A...G...................................  Cjacchus
1054440: TG............G...................................  Sboliviensis
```

```
                                  LINE1
       ------------------------------------------------->
                56491     56501     56511     56521     56531
56482:   TCCACCACCATAGACACCACTTATAATTTGTTTAAAGCATATTCTTTTAT Hsapiens
376223:  .................................................. Ptroglodytes
394264:  .................................................. Ggorilla
425508:  ..T......G........................................ Pabelli
347388:  ...................................G.............. Nleucogenys
422240:  ..........C....................................... Mmulatta
372898:  ..........C....................................... Panubis
407289:  ..................G............................... Cjacchus
1054490: .....A.........G.................................. Sboliviensis
```

```
                                  LINE1
       ------------------------------------------------->
                56541     56551     56561     56571     56581
56532:   GTATATGTGCTCCTGTAAAACATTGATTTCTTTATCTTACGCATTTTTAA Hsapiens
376273:  .......................................A.......... Ptroglodytes
394314:  ......A.......A........AA......................... Ggorilla
425558:  T......................................T.......... Pabelli
347438:  T......................................T.......... Nleucogenys
422290:  T........T.T..................C.C......T........T. Mmulatta
372948:  T....C.....T.A................C.C......T.......A.. Panubis
407339:  T.C....G.T..........T..................T.......... Cjacchus
1054540: T.C....G............T..................T.......... Sboliviensis
```

```
                             LINE1                    Alu
       ------------------------------------------->------->
                56591     56601     56611     56621     56631
56582:   ATTTTCATAAATGAAGTTATGTTAGATAATACTTAACATCAGCTTTGGGA Hsapiens
376323:  .................................................. Ptroglodytes
394364:  .G................................................ Ggorilla
425608:  ....................................-..A.......... Pabelli
347488:  .......................................A.......... Nleucogenys
422340:  .......................................A..T..C.A.. Mmulatta
372998:  ....................................T..A.....CA... Panubis
407389:  ....G.......................G..........A.......... Cjacchus
1054590: ....G...................A...G..T.......A.......... Sboliviensis
```

```
                                   Alu
       ------------------------------------------------->
                56641     56651     56661     56671     56681
56632:   GGCGGAGGCAGGTGGATCCCTTGAGCCCAGGAGTTTGAAGCCAGCCTGGG Hsapiens
376373:  .................................................. Ptroglodytes
394414:  .................................................. Ggorilla
425657:  ...T.......T................................T..... Pabelli
347538:  ...T.............................................. Nleucogenys
422390:  ...C..............................C...........C... Mmulatta
373048:  ...T.......C......................C...........C... Panubis
407439:  ...C..CA.....A...A................................ Cjacchus
1054640: ...C......A..A.................................... Sboliviensis
```

```
                                   Alu
         -------------------------------------------------------->
                   56691     56701     56711     56721     56731
    56682: CAACGTGGCGAAAACCCCGTCTCTACAAAAAATTAAAAACATAGCCAGT  Hsapiens
   376423: ...T..............A........................C......  Ptroglodytes
   394464: ..................................................  Ggorilla
   425707: ..................A...............C...C.A.........  Pabelli
   347588: ....A.A..........T.................T..............  Nleucogenys
   422440: ...T...........T..........-.............A.........  Mmulatta
   373098: ...T....T......T..........-........T....A.........  Panubis
   407489: ..T.A..A..........A.....C.......T..T....A...T.....  Cjacchus
  1054690: ..T.A.........T.A.A.....C..........TTTT.A...T.....  Sboliviensis

                                   Alu
         -------------------------------------------------------->
                   56741     56751     56761     56771     56781
    56732: CACAGTGGCACACACTTGCAGTCTCAGCTACTCAGGAGGCTAAGGTGGGA  Hsapiens
   376473: ..........................................T.......  Ptroglodytes
   394514: ..................................................  Ggorilla
   425757: .............G.C.......C..........................  Pabelli
   347638: ..TG...................C..........................  Nleucogenys
   422489: ..TG........TG.C.......C..........................  Mmulatta
   373147: ...G........TG.C.......C.......................T..  Panubis
   407539: ...G.A......TG.C..T....C................GT........  Cjacchus
  1054740: ...G........TG.C..T....A................AT........  Sboliviensis

                                   Alu
         -------------------------------------------------------->
                   56791     56801     56811     56821     56831
    56782: GGATTGCTTCAGCCCAGGAGGTCAAGGCTGCTGTGAGCAGTGACTGCACC  Hsapiens
   376523: ..................................................  Ptroglodytes
   394564: ...C....................................G.........  Ggorilla
   425807: ...........T.....A.........................T......  Pabelli
   347688: ..............................................T.T.  Nleucogenys
   422539: ................................A.......C..T...G..  Mmulatta
   373197: ................................A..........T......  Panubis
   407589: .........G..........T......................T....A.  Cjacchus
  1054790: .........G.................................T....A.  Sboliviensis

                                   Alu
         -------------------------------------------------------->
                   56841     56851     56858     56868     56878
    56832: ACTGTACTCCAGGCTGGGAGACAGAG---TAAGACCCTGTCTCAAAACAA  Hsapiens
   376573: ..C......T................---.....................  Ptroglodytes
   394614: .............G............---.....................  Ggorilla
   425857: ......G.....C......A......---.....................  Pabelli
   347738: ..........................CTC.....................  Nleucogenys
   422589: ............C.............---....T............C...  Mmulatta
   373247: ............C.............---.................C...  Panubis
   407639: ..-----.....C..T..CA......---.G...............--..  Cjacchus
  1054840: ....A.......C..T..CA......---.G................A..  Sboliviensis
```

```
            Alu                      LINE1
                 56888     56898     56908     56918     56928
  56879: AACAAAGAAAAACAAAAAAATACAAGTTTTCACATACTTTTCACCTAGTA  Hsapiens
 376620: .......C..........................................  Ptroglodytes
 394661: .......C...................A......................  Ggorilla
 425904: .......C....................................T...C.  Pabelli
 347788: ...G...C..........................................  Nleucogenys
 422636: .......C....................................T.....  Mmulatta
 373294: .......C...........................G........T.....  Panubis
 407679: ...------------.T.....T......CA.............T.T...  Cjacchus
1054887: ...------------.T.....T......CA.............T.....  Sboliviensis

                             LINE1
                 56938     56948     56958     56968     56978
  56929: CTATATTTTTAAGATTCATCCATGTTATTCTGGGAATATAGTCCGTACCT  Hsapiens
 376670: ..................................................  Ptroglodytes
 394711: ..................................................  Ggorilla
 425954: ...........................................T......  Pabelli
 347838: ...C..............................................  Nleucogenys
 422686: ..................................................  Mmulatta
 373344: .................T................................  Panubis
 407717: ...G...........C.T...........A........C.....A.G...  Cjacchus
1054925: ...G........................................A.....  Sboliviensis

                             LINE1
                 56988     56998     57008     57018     57028
  56979: TCTAACTGTTGCATTCTTCTGCTTCCACCATATTAATTTGCCCATTCATT  Hsapiens
 376720: ..................................................  Ptroglodytes
 394761: ...............................................G..  Ggorilla
 426004: .....T..C.................-..G....C......A........  Pabelli
 347888: ........C.........................................  Nleucogenys
 422736: ...G....C.C.......T............------------.......  Mmulatta
 373394: ........C.C.......T...............C...............  Panubis
 407767: ........C...............G.G...C------------.......  Cjacchus
1054975: ........C.A..........T..G.....C...C......T....T...  Sboliviensis

                             LINE1
                 57038     57048     57058     57068     57078
  57029: TGCCGATGGCAGATGCCCAAATGGTTTCCTTCCTCATGCCACCACAAACA  Hsapiens
 376770: ....A.............................................  Ptroglodytes
 394811: ....A.............................................  Ggorilla
 426053: ....A...A........G..............T.................  Pabelli
 347938: ....A...A.......T...............T........T........  Nleucogenys
 422774: ....A.C.A.....A........T........T............T....  Mmulatta
 373444: ....A...A.......................T..G.........T.G..  Panubis
 407805: ....AG........AT.....C..C...---......A............  Cjacchus
1055025: ....A.........A.........C...---.T....A............  Sboliviensis
```

```
                                    LINE1
         ---------------------------------------------------->
                     57088     57098     57108     57118     57128
                         :         :         :         :         :
   57079: GTGCCATGATGAATATCAGCACACCTACTCCCTTACACAGCTGTGTGAGA  Hsapiens
  376820: ..........................................C.......  Ptroglodytes
  394861: ..................................................  Ggorilla
  426103: .............C................................A...  Pabelli
  347988: .....C.A.....C....................................  Nleucogenys
  422824: .............C...................C................  Mmulatta
  373494: .............C...................C................  Panubis
  407852: .............C.............T...............C......  Cjacchus
 1055072: .............C...G.........T...............CA.....  Sboliviensis

            LINE1
         -------->
                     57138     57148     57158     57168
                         :         :         :         :
   57129: ATTTCTTTGTAAAGGAAATGGCCAACTTACAGGGTTCATGTATGTGCCA-  Hsapiens
  376870: ......................T..........A...............-  Ptroglodytes
  394911: ......................T..........................-  Ggorilla
  426153: ......................T..........................G  Pabelli
  348038: ......................T..........................-  Nleucogenys
  422874: ......................T..........................G  Mmulatta
  373544: ......................T...............C..........G  Panubis
  407902: .........C.G..........T..A......A.....C....A.T...G  Cjacchus
 1055122: ..............A.......T..A......A....G.....A.....G  Sboliviensis

                     57187     57197     57207     57217     57227
                         :         :         :         :         :
   57178: GTACTATATGTGTATTATTCAGAATCTTGAGTCTTGCAGGGTGAGTGATG  Hsapiens
  376919: .........A........................................  Ptroglodytes
  394960: A.................................................  Ggorilla
  426203: ........CA.......................C................  Pabelli
  348087: ................T.................................  Nleucogenys
  422924: ......C.............................T.............A  Mmulatta
  373594: ......CG.A.......C...C..........T..T..............  Panubis
  407952: .G.G.....A.........T....................A.........  Cjacchus
 1055172: .G.G...............T...C................A.T.......  Sboliviensis

                     57237     57247     57257     57267     57277
                         :         :         :         :         :
   57228: ATGTTCGCCTCACTTCACAGTTGGATCTTACAGCTCTTCAAGCATTGAAC  Hsapiens
  376969: ..................................................  Ptroglodytes
  395010: .....T............................................  Ggorilla
  426253: .........................................A......G.  Pabelli
  348137: ................................................G.  Nleucogenys
  422974: .................T....A.........................G.  Mmulatta
  373644: ......................A....................T....G.  Panubis
  408002: .....T..........--....A...A.....................GT  Cjacchus
 1055222: .....T......TG........A...A..C..............G...G.  Sboliviensis

                     57287     57297     57301     57311     57321
                         :         :         :         :         :
   57278: TCATAGTACCAAGAAAGAATA------CTTCAGGTTCAGGTTTTTCTCAA  Hsapiens
  377019: .....................------.......................  Ptroglodytes
  395060: ..G..................------.......................  Ggorilla
  426303: ....................T------........A.............G  Pabelli
  348187: .....................------.......................  Nleucogenys
  423024: ...............G.....------.................A.....  Mmulatta
  373694: ...............G...A.------.................A.....  Panubis
  408050: .T..G.......A......C.CTTTAG.......................  Cjacchus
 1055272: .T.C............T....CTTTAG.......................  Sboliviensis
```

```
                57331     57341     57351     57361     57371
                    :         :         :         :         :
  57322: AAATCATTGGCAATACATAATTATAGTTAATTCAAATGAACTTTGTATTA  Hsapiens
 377063: ..................................................  Ptroglodytes
 395104: ..................................................  Ggorilla
 426347: ......................G..A........................  Pabelli
 348231: ......................G..A.................C......  Nleucogenys
 423068: .........................A.G......................  Mmulatta
 373738: ........A................A........................  Panubis
 408100: ...............T.....A...A..............T....A....  Cjacchus
1055322: .....G.........T.G...A...A...........A..T....A.A..  Sboliviensis
```

```
                57381     57391     57401     57411     57418
                    :         :         :         :         :
  57372: ATGATAGTTTGAATTAGAATAATTCAAATTCTAATCAATAATA---TTAA  Hsapiens
 377113: ...........................................---....  Ptroglodytes
 395154: ...........................................---....  Ggorilla
 426397: ...........................................---....  Pabelli
 348281: .............................C.............---....  Nleucogenys
 423118: ..T........................................---....  Mmulatta
 373788: ..T........................................---....  Panubis
 408150: ..T.A.........................T....T.G...C.TAG....  Cjacchus
1055372: .AT...........................T....T.G..GC.TAGC...  Sboliviensis
```

```
                57428     57438     57448     57458     57468
                    :         :         :         :         :
  57419: AACTCTGTCTTGCCAATAGACATGCTAAATATCAAATGAACTTTGTTTCT  Hsapiens
 377160: ..................................................  Ptroglodytes
 395201: ..................................................  Ggorilla
 426444: ....................................C...........A.  Pabelli
 348328: ................................................A.  Nleucogenys
 423165: ....................................C...........A.  Mmulatta
 373835: ................C...............................A.  Panubis
 408200: ........A.......-...T........................C..A.  Cjacchus
1055422: ................-............................C..A.  Sboliviensis
```

```
                57478     57488     57498     57508     57518
                    :         :         :         :         :
  57469: TGGCAAGACAGAGTTTTAACTATAGTATTAATGTAGTGCCAAAGTGCACG  Hsapiens
 377210: .....................G..T.......................T.  Ptroglodytes
 395251: ........................T.......................T.  Ggorilla
 426494: ........................T.....................A.T.  Pabelli
 348378: ........................T.......................T.  Nleucogenys
 423215: ........................T.....................A.T.  Mmulatta
 373885: ........................T.....................A.T.  Panubis
 408249: ............A.....GT....T...........CA........A.T.  Cjacchus
1055471: .........GA.A......T...GC...G.......CA........A.T.  Sboliviensis
```

```
                57528     57538     57548     57554     57564
                    :         :         :         :         :
  57519: GGCTTTCCTCATTCATTTTCCAAAATTCTACCTTT----CTGTTTGCAGA  Hsapiens
 377260: ...................................----...........  Ptroglodytes
 395301: ..............G....................----...........  Ggorilla
 426544: ....................A..............CTAG...........  Pabelli
 348428: ...........C.......................----...........  Nleucogenys
 423265: ..................C................CTAG...........  Mmulatta
 373935: ...................................CTAG...........  Panubis
 408299: A.............C.............C......CTAG.CA........  Cjacchus
1055521: A...........................C....C.CTAG.CA........  Sboliviensis
```

```
                     57574     57584     57594     57604     57614
                         :         :         :         :         :
   57565: GATCTTTCACAATGGAGTTTCTATGTTAACCATATATTTAACTAGTTTTA  Hsapiens
  377306: ..........G...C...................................  Ptroglodytes
  395347: ............-..........G..........................  Ggorilla
  426594: .............................T....................  Pabelli
  348474: ..................A...............C...............  Nleucogenys
  423315: ................A............T...C.........G......  Mmulatta
  373985: ................A............T....................  Panubis
  408349: ..........................G.CTGT...........G......  Cjacchus
 1055571: ............................TT.T...G.......G......  Sboliviensis

                     57624     57634     57644     57654     57664
                         :         :         :         :         :
   57615: TTGACAATTCCCTCTACATCTGGGTGATTGTCTTTTCATGTGCAGAGGTG  Hsapiens
  377356: ...................................G..............  Ptroglodytes
  395396: ...................................G..............  Ggorilla
  426644: ........A..........................G..............  Pabelli
  348524: ...................................G..............  Nleucogenys
  423365: ...................................G..CC..........  Mmulatta
  374035: .............A.....................G..CA..........  Panubis
  408399: ..A.............T..A.T........C....G..............  Cjacchus
 1055621: ..A.............T....T........C...AG..............  Sboliviensis

                                                LINE2
                                           ---------------->
                     57674     57684     57694     57704     57714
                         :         :         :         :         :
   57665: TGTATGGATGGATTTATAGATTTTGGTTCATTTAATATGTTGGTATTCCT  Hsapiens
  377406: ..................................................  Ptroglodytes
  395446: ..................................................  Ggorilla
  426694: ................C...........................C.....  Pabelli
  348574: ............................................C.....  Nleucogenys
  423415: .A.....G......G.CG..........................C.....  Mmulatta
  374085: .A............G.C..............G............C.....  Panubis
  408449: ..........A...C.C.......C.......G.....AC....C..A..  Cjacchus
 1055671: .A..............C...............G.....AC....C.....  Sboliviensis

                        LINE2
          ------------------------------->
                     57724     57734     57744     57754     57764
                         :         :         :         :         :
   57715: CACTACATTGAACATTCTGTAAGGGCAGAGAAAATTCTTCTGTGAAATTG  Hsapiens
  377456: .......C..........T..................A............  Ptroglodytes
  395496: ..................T..................A..C.........  Ggorilla
  426744: ..................T..................A............  Pabelli
  348624: ..................T..................A............  Nleucogenys
  423465: ............T.....T..................A............  Mmulatta
  374135: ......G.....T.....T..................A.........C..  Panubis
  408499: .....A............T...............G..A.....A......  Cjacchus
 1055721: .....A............T.....A.........G..A.....A......  Sboliviensis

                     57774     57784     57794     57804     57814
                         :         :         :         :         :
   57765: TCAGTGTCTTCCACATTCACCCAGGAATCAATACTTGTTAGGGAAACCAA  Hsapiens
  377506: ....................................A.............  Ptroglodytes
  395546: ..................................................  Ggorilla
  426794: ..--..........................G...................  Pabelli
  348674: ..................................................  Nleucogenys
  423515: ......C.........................G.................  Mmulatta
  374185: .....................................C............  Panubis
  408549: ...--.......C........G..............AC........T...  Cjacchus
 1055771: ....................................AC........T...  Sboliviensis
```

```
             57824     57834     57841     57851     57861
   57815: TATTCTGGATATTTTACAGCT---AGTCTAATTAATGTTGCTTAGCCCAT  Hsapiens
  377556: .....................---.................C...T....  Ptroglodytes
  395596: .....................---.................C...T....  Ggorilla
  426842: ..................A..TCA.............C...C...T....  Pabelli
  348724: ..................A..---.................C...T....  Nleucogenys
  423565: ..................A..TCA.................C...T..G.  Mmulatta
  374235: ........G.........A..TCA.................C...T....  Panubis
  408597: ..........G.......A..TCA......G..........C...T....  Cjacchus
 1055821: ..........G.......A..TCA......G..........C...T....  Sboliviensis

             57871     57881     57891     57901     57911
   57862: TTCAGGGAAGCAGAACTTATGGAAGGCAGAAGTCCCAAGTCACCCAGAAT  Hsapiens
  377603: ...................C..............................  Ptroglodytes
  395643: ..................................................  Ggorilla
  426892: .................CT.........A.....................  Pabelli
  348771: .................C..-.............................  Nleucogenys
  423615: .................C.......................T........  Mmulatta
  374285: .............----------------............T....T...  Panubis
  408647: ....T.........C..C..A..................A...A......  Cjacchus
 1055871: ....T............C.......A.................A......  Sboliviensis

             57921     57931     57941     57951     57959
   57912: TTTATCAAAGATGCATATGGACCCCCAAAACAGCCCACTCAC--GTGGTA  Hsapiens
  377653: ..........................................--......  Ptroglodytes
  395693: ..........................................--......  Ggorilla
  426942: ............CT.......T...................T--......  Pabelli
  348820: .............T....A.......................--......  Nleucogenys
  423665: ............CT.............G..............--A..A..  Mmulatta
  374319: ............CT............................--...A..  Panubis
  408697: ...........A.--.....................C.A...AA....C.  Cjacchus
 1055921: .............--.....................C.A...AA......  Sboliviensis

             57969     57979     57989     57995     58005
   57960: AGGGAATGTTGGCTGTGTCTTGGAAAAAAAAAA----TCTTCAAGCAAAT  Hsapiens
  377701: ......CA.........................TCT-.............  Ptroglodytes
  395741: ......C..........................TCT-.............  Ggorilla
  426990: ......A......................G...TCT-.............  Pabelli
  348868: ......A....................C....TCT--.............  Nleucogenys
  423713: ......AA...............G..G.....ATCT..............  Mmulatta
  374367: ......A................G..G.....ATCT..............  Panubis
  408745: ...A.--....A.................TCT----........G....  Cjacchus
 1055969: ...A.--....A.................TCT----........G....  Sboliviensis

             58015     58025     58035     58045     58055
   58006: GAGTTGTGTCATCTGGAAGTTGTTATGACAGAAACGAAGAAAAGAAGGAT  Hsapiens
  377750: ...G..............................................  Ptroglodytes
  395790: ...G..............................................  Ggorilla
  427039: ...G..............................................  Pabelli
  348916: ...G....A.........................T...............  Nleucogenys
  423763: ...G....C.........................T.............G.  Mmulatta
  374417: ...G....C......A..................T............A..  Panubis
  408789: ...GC........................--------.............  Cjacchus
 1056013: ...A.........................--------.............  Sboliviensis
```

```
                  58065     58075     58085     58095     58105
   58056: ATCTGTCACTGTAGCCCTTTGAGGTTGAGAAGAGGGATTTCCAGAAGAGA  Hsapiens
  377800: ...................C..............................  Ptroglodytes
  395840: ...................C..............................  Ggorilla
  427089: .........A.........C......---................C....  Pabelli
  348966: ................AC.C..............................  Nleucogenys
  423813: ..........A........CA....................T.......-  Mmulatta
  374467: .......G...........CA............................-  Panubis
  408831: -...A.-G...........CT............................-  Cjacchus
 1056055: -...A-.G.A...A.....CT.............................  Sboliviensis

                  58115     58125     58135     58145     58155
   58106: CAACTCTATTTGGGATACGTGTGGAAGGGAACACATCTTATCTTATCTGA  Hsapiens
  377850: .................T................................  Ptroglodytes
  395890: .................T................................  Ggorilla
  427136: .................T.CT....................-----....  Pabelli
  349016: .................T................................  Nleucogenys
  423862: -................T.............T..................  Mmulatta
  374516: -............A...T................................  Panubis
  408878: --...............T......................G-------A.  Cjacchus
 1056103: ...TC............T......................G-------A.  Sboliviensis

                  58165     58175     58185     58195     58205
   58156: AGGGATACTGACACTGAAGGACTGCAACAGACGGAGGGTCTGGTTCTGTC  Hsapiens
  377900: ...............AG..........................C......  Ptroglodytes
  395940: ...............A...............T..................  Ggorilla
  427181: ...............A..................................  Pabelli
  349066: ...............A...............T.......T..........  Nleucogenys
  423911: .........A.....A......C.........A....A............  Mmulatta
  374565: .........A.....A................A.................  Panubis
  408919: TA.............A...........TG..TA..A.A.....GC.....  Cjacchus
 1056146: T..............A...........TG..TA..A...T...GC.....  Sboliviensis

                  58215     58225     58235     58245     58255
   58206: CTTTTGGGGGAAATACATCTCCAAGGGTGGCTAAGACACCAGTATCTTAC  Hsapiens
  377950: ......................................A...........  Ptroglodytes
  395990: ..................................................  Ggorilla
  427231: .....................................-....C.......  Pabelli
  349116: ..............................A...........C.......  Nleucogenys
  423961: ..C............-...................G......C.......  Mmulatta
  374615: ..C............-.........T.........G..T...C.......  Panubis
  408969: .C.....A..................................C.......  Cjacchus
 1056196: .......A........G................G........C.......  Sboliviensis

                  58265     58275     58285     58295     58305
   58256: AGTATGTACCATACCCTGTGTACATTCTTCCCCTTTCACAAGACTCTTCT  Hsapiens
  378000: .A................................................  Ptroglodytes
  396040: .A................................................  Ggorilla
  427280: .A.C...C.........A................................  Pabelli
  349166: .A.C........G.....................................  Nleucogenys
  424010: .A.C.............AG....G..........C..G............  Mmulatta
  374664: .A.C.............AG....G..........C...............  Panubis
  409019: .A.C.A.....C.....A....GG.....T.....C..TG..........  Cjacchus
 1056246: .A.C.A.....C.....A....GG...............G..........  Sboliviensis
```

```
              58315     58325     58335     58345     58355
                  :         :         :         :         :
  58306: CTCATGACCACTTATCTAGCACCTACCTCTCTTTCCCTAAATCAGAGCTA Hsapiens
 378050: .................................................. Ptroglodytes
 396090: .................................................. Ggorilla
 427330: ...G...........................C.................. Pabelli
 349216: ...G...T.......................................... Nleucogenys
 424060: ...............................C.................. Mmulatta
 374714: ...........................C...C.................. Panubis
 409069: A.TG....................G......C.................. Cjacchus
1056296: A.TG...................CG......C.................. Sboliviensis

              58365     58375     58385     58395     58405
                  :         :         :         :         :
  58356: TTCTTTTACTCTTACTCTCTGCCCTACAACTTTGTGAATTTCACATATGC Hsapiens
 378100: .................................................. Ptroglodytes
 396140: ..............................C................... Ggorilla
 427380: C...A.......C..................................C.. Pabelli
 349266: ............C............G...T.................... Nleucogenys
 424110: ........G...C......C.............................. Mmulatta
 374764: ........G...C..................................... Panubis
 409119: ..........T.C.--................A..............A.. Cjacchus
1056346: ............C.--................G.............CC.. Sboliviensis

              58415     58425     58435     58445     58455
                  :         :         :         :         :
  58406: TCATTCCTAAAAATAGTAGGAATTTCAGAATGTTCACATAAACATAAACC Hsapiens
 378150: .................................................. Ptroglodytes
 396190: .................................................. Ggorilla
 427430: ............G..............C...................... Pabelli
 349316: ...........................................G...... Nleucogenys
 424160: ...................A.............................. Mmulatta
 374814: ...................A.............................. Panubis
 409167: .T.......G..G............G........T.....T.T.A..... Cjacchus
1056394: ......T..G..G........................G....T....... Sboliviensis

              58465     58475     58485     58495     58505
                  :         :         :         :         :
  58456: ATTATATTTTACCTGCATGTACAGAATACACATATTACTTAGGAGACTAA Hsapiens
 378200: ...........G...................................... Ptroglodytes
 396240: .................................................. Ggorilla
 427480: ..GT.............................................. Pabelli
 349366: ......................................C........... Nleucogenys
 424210: ..G............................................... Mmulatta
 374864: ..G............................................... Panubis
 409217: ..GTC.............T............................... Cjacchus
1056444: ..GTC................T............................ Sboliviensis

              58515     58525     58535     58545     58555
                  :         :         :         :         :
  58506: TTTTAAAGCTATAAGAGGAAACTGAGAGGCAGAAAAAGAAAGAAATTGTC Hsapiens
 378250: ................C...............C................. Ptroglodytes
 396290: .......................C........G................. Ggorilla
 427530: ...........................A....G................. Pabelli
 349416: ................................G................. Nleucogenys
 424260: ................A...............G................. Mmulatta
 374914: ................................G.........G....C.. Panubis
 409267: ..........G..............A......G................. Cjacchus
1056494: ..........G..............A......G................T Sboliviensis
```

```
                 58565     58575     58585     58594     58604
                     :         :         :         :         :
  58556: TCCAACACATAGCAATTAATAAGGCTAGCATAGT-AAGTTAAGATCTTCT  Hsapiens
 378300: ..................................-...............  Ptroglodytes
 396340: ...C..............................-...............  Ggorilla
 427580: ............TG....................-...............  Pabelli
 349466: .....A......TG...................C-...............  Nleucogenys
 424310: ............TG....................-...............  Mmulatta
 374964: ............TG....................-.....           Panubis
 375304:                                         ..........  Panubis
 409317: ..........GATG....G..........G....G.....G.........  Cjacchus
1056544: ............TG....G..........G....G.....G.A.......  Sboliviensis

                 58614     58624     58634     58644     58654
                     :         :         :         :         :
  58605: AAGGAGCTAAGAAGTGTGTCAATACACAGAGGCCACAGCATGCATGACTA  Hsapiens
 378349: ..........................--......................  Ptroglodytes
 396389: ...........................G......................  Ggorilla
 427629: .................T.........C.................A....  Pabelli
 349515: .................T.........C.G...............A....  Nleucogenys
 426698:    ..............T..........A.....................  Mmulatta
 375314: .................T..........A.....................  Panubis
 409367: ...........GG....T........--T........A............  Cjacchus
1056594: .................T...................A............  Sboliviensis

                           58669     58679     58689     58699
                               :         :         :         :
  58655: GCTTGTGT-----TTTGAATGAAGATTAGTAAGTAGAAAATATCAACGGA  Hsapiens
 378397: ........-----.....................................  Ptroglodytes
 396439: ........-----..................................A.G  Ggorilla
 427679: ......A.-----.................................TA..  Pabelli
 349565: .T......-----.................................TA..  Nleucogenys
 426745: ........-----....................C.............A..  Mmulatta
 375364: ........-----..................................A..  Panubis
 409415: .......GCATGAC......A..........C.........G.....A..  Cjacchus
1056644: ........-----.......A..........................A..  Sboliviensis

                 58709                         58724     58734     58744
                     :                             :         :         :
  58700: GTTCCATTTTACTAAA-----CAAAGATCTGAAATCAAGTCAGCCTGCCT  Hsapiens
 378442: .....G..........-----.............................  Ptroglodytes
 396484: ................-----....A........................  Ggorilla
 427724: ................-----.............................  Pabelli
 349610: ................-----.G.....................A.CT..  Nleucogenys
 426790: ........C.......-----.........................A...  Mmulatta
 375409: ................-----.G...........................  Panubis
 409465: ..C.............CATTT....T........................  Cjacchus
1056689: .....G..........CTTTT.............................  Sboliviensis

                 58754     58764     58774     58784     58794
                     :         :         :         :         :
  58745: CAAAGATTTTTAAATGCACACTATCAATGGCATCCAAGTATGCTAATGTA  Hsapiens
 378487: ...........C.........C...G........................  Ptroglodytes
 396529: ...........C.........C............................  Ggorilla
 427769: ..........C.......................................  Pabelli
 349655: ..........................G..........A............  Nleucogenys
 426835: ...-.........................AT...A..A............  Mmulatta
 375454: ...-..............T..........A....A..A............  Panubis
 409515: ...............A.............AT-C....AC.........C.  Cjacchus
1056739: ........C....................AT.CT...AC.........C.  Sboliviensis
```

```
              58804     58814     58824     58834
  58795: TTTATCAAGGGGTCAGCAAAAGTCATACTCAATTAAATAATAAGTT----  Hsapiens
 378537: ..............................................----  Ptroglodytes
 396579: ..............................................----  Ggorilla
 427819: ...C.........................-................----  Pabelli
 349705: ...........................T...........G......----  Nleucogenys
 426884: ...........A..................................AGTT  Mmulatta
 375503: ...........A..................................AGTT  Panubis
 409564: ....A......T.............C...----...........C.----  Cjacchus
1056789: ...........T........T.A..C...-----..G.......C.----  Sboliviensis

              58850     58860     58870     58880     58890
  58841: GCCCCTTCATTTGGTTCATTATGCAACCTGTCCAAATGCCACAGTTTACT  Hsapiens
 378583: ..................................................  Ptroglodytes
 396625: ..................................................  Ggorilla
 427864: ..........................................GA......  Pabelli
 349751: ..T.......................................G......C  Nleucogenys
 426934: .........C.............................T.TG.......  Mmulatta
 375553: .........C.............................T.TGA......  Panubis
 409606: .............C.......CA.G................TG.......  Cjacchus
1056831: ..............C......CA..................TG.......  Sboliviensis

              58900     58910     58920     58930     58940
  58891: CTTATTTATTGTCCCCTTTGACAACAAGCACAGCTGAGATAATAACGTAC  Hsapiens
 378633: ....C.............................................  Ptroglodytes
 396675: ....C.............................................  Ggorilla
 427914: ...............A.............................A....  Pabelli
 349801: ..............TA.............................A....  Nleucogenys
 426984: .................................T...........A....  Mmulatta
 375603: ...............................G.T...........A....  Panubis
 409656: .......G....T.....C....................C..CG.A...A  Cjacchus
1056881: .......GC...T.....A............G.T.....C.....A...A  Sboliviensis

              58950     58960     58970     58980     58990
  58941: AGAAACATTTCTCTTACCTTTGTTGCTTTCCAGTAGTGAAGTTCACAGAG  Hsapiens
 378683: ..................................................  Ptroglodytes
 396725: ................................................G.  Ggorilla
 427964: ..................................G..........AG.T.  Pabelli
 349851: ..................................G..........AG.T.  Nleucogenys
 427034: ........................A.........G.............T.  Mmulatta
 375653: ........................A.........G.............T.  Panubis
 409706: ........G.A.......................G.............T.  Cjacchus
1056931: ......G...A...........C........G..G.............T.  Sboliviensis

              59000     59010     59020     59030     59040
  58991: TGGCAAGTTAGACTGGAGCTGCGTCCCTCAAAAGGAAATATAAAGCCTTC  Hsapiens
 378733: ..................................................  Ptroglodytes
 396775: ..................................................  Ggorilla
 428014: ..................................................  Pabelli
 349901: ..................................................  Nleucogenys
 427084: ..................................................  Mmulatta
 375703: .A................................................  Panubis
 409756: ......................A.-G..................A.....  Cjacchus
1056981: ...T..................A.-G..................A.....  Sboliviensis
```

```
                 59050     59060     59070     59080     59090
                     :         :         :         :         :
   59041: ATATTTTGCCCCTCCCTTTTCATTTGTGCCCTTAGTGATTCAGCTCTTCA   Hsapiens
  378783: ............................T.....................   Ptroglodytes
  396825: ............................T....................G   Ggorilla
  428064: ............................T.....................   Pabelli
  349951: .G..........................T.....................   Nleucogenys
  427134: ............................T.....................   Mmulatta
  375753: ............................T.....................   Panubis
  409805: ............................T....C................   Cjacchus
 1057030: ............................T.....................   Sboliviensis

                 59100     59110     59120     59130     59140
                     :         :         :         :         :
   59091: CCTGCTGGTTGGAAAGTACAGCCCTGAAAATGAAGTCCATCAAGAGCTTC   Hsapiens
  378833: ..................................................   Ptroglodytes
  396875: .......A..........................................   Ggorilla
  428114: ............C..................T.........G........   Pabelli
  350001: .......................T..........................   Nleucogenys
  427184: ..............................G...................   Mmulatta
  375803: ..............................G...................   Panubis
  409855: ...........A......T...........G.....T.............   Cjacchus
 1057080: ...........A..................G.....T.........T...   Sboliviensis

                                  Alu
                         ------------------------------------>
                 59150     59160     59170     59180     59190
                     :         :         :         :         :
   59141: TTTAAATCCCCAGAGCCCAGCATGGTGGCTCATTCCTGTAATCCCAGCAC   Hsapiens
  378883: ..................................................   Ptroglodytes
  396925: ..................................................   Ggorilla
  428164: .........G........................................   Pabelli
  350051: ..................................................   Nleucogenys
  427234: ..................................................   Mmulatta
  375853: ..................................................   Panubis
  409905: ........T...........TT..A.......C...G.C...........   Cjacchus
 1057130: ........T...........T...........C...G.C...........   Sboliviensis

                           Alu
          ------------------------------------------------->
                 59200     59210     59220     59226     59236
                     :         :         :         :         :
   59191: TTTGGAAGGCTGAGGTGGGAGGGTTACTTGAG----GCTAGGAGTTTGAG   Hsapiens
  378933: ..........C.....................----..............   Ptroglodytes
  396975: ..........C.....................----..............   Ggorilla
  428214: ..........CA....................----..............   Pabelli
  350101: ..........C....................A----..............   Nleucogenys
  427284: ..........C..............T......----..............   Mmulatta
  375903: .........................T......----..............   Panubis
  409955: C.CA.....AGC..........A..G......GCAA............G.   Cjacchus
 1057180: ..CA.G...AGA.T........A..GT.....GCGA............G.   Sboliviensis
```

```
                         Alu
           ------------------------------------------------->
                 59246     59256     59266     59276     59285
   59237: GCCAGCCTGGGCAACATAGTGAGACCCTGATCTCTATTTT-AAAAATTAA  Hsapiens
  378979: .......................................A-.........  Ptroglodytes
  397021: .......................................A-.........  Ggorilla
  428260: .G......................T.T............A-.........  Pabelli
  350147: .......................................A-.........  Nleucogenys
  427330: .......................................A-.........  Mmulatta
  375949: ...................A....................-.........  Panubis
  410005: A............................G..........A.......T.  Cjacchus
 1057230: A.....................A......G..........A.........  Sboliviensis

             Alu
           -------->
                 59295     59305     59315     59324     59334
   59286: AAAATAAAAATAAAATTTGTAATGAGATCCCC-AGAGACTCCCAAGATGA  Hsapiens
  379028: ..................T.............-.................  Ptroglodytes
  397070: ..................T.............-.................  Ggorilla
  428309: .....G............T.............-........T........  Pabelli
  350196: ..................T.C...........-................G  Nleucogenys
  427379: ..................T.............C................G  Mmulatta
  375998: ..................T.............C................G  Panubis
  410055: .................AC.........T.G.-..........G.....G  Cjacchus
 1057280: .................AC.........T...-................G  Sboliviensis

                                             Alu
                                      ------------------------>
                 59344     59354     59364     59374
   59335: CCAGTGCTTTTCAGAGAAGTGCTCAGTTCTTTTTCTATTTATTTA-----  Hsapiens
  379077: .............................................-----  Ptroglodytes
  397119: .........................C...................-----  Ggorilla
  428358: ...................C..C......................TTTAT  Pabelli
  350245: ......................C.T....................-----  Nleucogenys
  427429: ....C......T.......C..CTGTC..AA..............TTTAT  Mmulatta
  376048: ...........T....G..C..C......................-----  Panubis
  410104: ...................CA.C........                     Cjacchus
 1057329: ...................CA.C........                     Sboliviensis

                         Alu
           ------------------------------------------------->
                                     59386     59396     59406
   59380: -----------------------TTTTGAGATGGAGTCTCACAGGCTGGA  Hsapiens
  379122: -----------------------...........................  Ptroglodytes
  397164: -----------------------...............A...........  Ggorilla
  428408: TTATTTATTTATTTATTTATTTG...........................  Pabelli
  350290: -------------------TTTA...........................  Nleucogenys
  427479: TTAGTTAGTTAGTTAGTTAGTTA...........................  Mmulatta
  376093: -----------TTTATTTATTTA...........................  Panubis
```

```
                Alu
        ------------------------------------------------->
                 59416     59426     59436     59446     59456
   59407: GTGCAGTGGTGTGATCTCAGCTCACTGCAACCTCTGCCTCCTGGGTTTAA  Hsapiens
  379149: ....G.............................C...............  Ptroglodytes
  397191: ...........                                         Ggorilla
  428458: .................TG.....A......T..C...............  Pabelli
  350321: .........CA......TG........................A......  Nleucogenys
  427529: .........CACC.....G...............CA.......-------  Mmulatta
  376132: .........CACC.....G...............CA.......-------  Panubis
```

```
                Alu
        ------------------------------------------------->
                 59466     59476     59486     59496     59506
   59457: GCAGTTCTCCTGCCTCAGCCTCCTGAGTAGCTGGGATTATAGGTGTGCAC  Hsapiens
  379199: ...A..............................................  Ptroglodytes
  428508: T..A.......................................CACA...  Pabelli
  350371: ...A.......................................CAC....  Nleucogenys
  427572: -------------------------..................C.A....  Mmulatta
  376175: -------------------------..................C.A....  Panubis
```

```
                Alu
        ------------------------------------------------->
                 59516     59526     59536     59546     59556
   59507: CACCACACCCAGCTAATTTTTGTATTTTTAGTGGAGACAGGGTTTCATCA  Hsapiens
  379249: ..............G...................................  Ptroglodytes
  397340:                                              .....  Ggorilla
  428558: ..................................................  Pabelli
  350421: .........TG......................A................  Nleucogenys
  427597: ............A-...G...-----.A.G.................C..  Mmulatta
  376200: ............GC.......-----.A...................C..  Panubis
```

```
                Alu
        ------------------------------------------------->
                 59566     59576     59586     59596     59606
   59557: TGTTGGTCAGGCTGGTCTCAAACTCCTGACCTCGTGATCCACCTGCCTCA  Hsapiens
  379299: .................................A.........C......  Ptroglodytes
  397345: .........................................G.C......  Ggorilla
  428608: ...................G.......................CA.....  Pabelli
  350471: ...................G.......................CA.....  Nleucogenys
  427641: ...................G........C..........TG.........  Mmulatta
  376245: ...................G........C...T......TG.........  Panubis
```

```
                Alu
        ---------------------------------------------->
                 59616     59626     59636     59646     59656
   59607: GCCTCCTAAAGTGCTGGGATTACAGGCGTGAACGACCATGCCCGGCCCTG  Hsapiens
  379349: ......................T....................A......  Ptroglodytes
  397395: ..T........................................A..T...  Ggorilla
  428658: ......C..........................C..G.....A.......  Pabelli
  350521: ......C..........................C........A.......  Nleucogenys
  427691: A................................C........T.A.....  Mmulatta
  376295: A................................C........T.A.....  Panubis
  410122:                                                  .  Cjacchus
 1057347:                                                  .  Sboliviensis
```

```
                    59666     59676     59686     59696     59706
                        :         :         :         :         :
  59657: CACCCAGTTCTTAACCTCCTTCTGTTTTACTAAGGGAAAACATTAACAAG  Hsapiens
 379399: .........................................C.C......  Ptroglodytes
 397445: ..................................................  Ggorilla
 428708: ..................T.........................G.....  Pabelli
 350571: ...............T............................G.....  Nleucogenys
 427741: ......A.....................................G.....  Mmulatta
 376345: ......A.....................................G.....  Panubis
 410123: ........................C....G.....A........G.....  Cjacchus
1057348: ........................C....G....AA......C.G.....  Sboliviensis

                    59716     59726     59736     59746     59756
                        :         :         :         :         :
  59707: CCTGGCCTCTATTCAAGAAAAATACGCTGTCTGTCAAAGAGATTGCCATC  Hsapiens
 379449: ......................C...........................  Ptroglodytes
 397495: ......................C.......A...................  Ggorilla
 428758: ......................C.A.........................  Pabelli
 350621: ......................C.A....C.......G............  Nleucogenys
 427791: ...................G..C.A.........................  Mmulatta
 376395: ...................G..C.A.........................  Panubis
 410173: T..A..T...............C.A.........................  Cjacchus
1057398: ...A..T...............C.A.----A...................  Sboliviensis

                    59766     59776     59786     59796     59806
                        :         :         :         :         :
  59757: AACCCAACAAGTTAGCAGTGCCCTATGCAAAATTCCAGGGATCTCCAGTG  Hsapiens
 379499: .............G........A...........................  Ptroglodytes
 397545: .............G........A...........................  Ggorilla
 428808: .............G........A..C........................  Pabelli
 350671: .............G........A...........................  Nleucogenys
 427841: ..T..........G........A...........................  Mmulatta
 376445: ..T..........G........A...........................  Panubis
 410223: ...T.........G........A.G............A............  Cjacchus
1057444: .............G......-.A.......................C...  Sboliviensis

                    59816     59826     59836     59846     59856
                        :         :         :         :         :
  59807: TCAGTGCACATGGTGCAAAGCTACTCAGTAGGAAAGATCCCAGCTGTGGC  Hsapiens
 379549: ..G...............................................  Ptroglodytes
 397595: ..G...............................................  Ggorilla
 428858: ...........................A...................TT.  Pabelli
 350721: ..G........................A......................  Nleucogenys
 427891: ..C........................A........G.............  Mmulatta
 376495: ..C........A...............A......................  Panubis
 410273: ..G...........A...............A......C............  Cjacchus
1057493: ..G...........A......................C............  Sboliviensis

                    59865     59875     59885     59895     59905
                        :         :         :         :         :
  59857: AGAT-GCTTCCTGGCCTGAGGAACACTCATCCCAAGATCTTCTGGGCTCT  Hsapiens
 379599: ....-.............................................  Ptroglodytes
 397645: ....-.............................................  Ggorilla
 428908: TAT.G.............................................  Pabelli
 350771: ....-.............................................  Nleucogenys
 427941: ...C-..................G..........G...............  Mmulatta
 376545: ....-..................G..........................  Panubis
 410323: ....-........A.......T............................  Cjacchus
1057543: ....-........A..........................C.........  Sboliviensis
```

```
              59915     59925     59935     59945
                  :         :         :         :
  59906: CTTGCATGCAGCATATGTTTTTATGTGGAGTTTCAGCAAACTACT-----   Hsapiens
 379648: ..............................C........T.....-----   Ptroglodytes
 397694: ..............................C........T.....-----   Ggorilla
 428958: ......                                              Pabelli
 433648:       ..G.....................C........T.....-----   Pabelli
 350820: ........G.....................C........C.....-----   Nleucogenys
 427990: ........G.....................C........T.....-----   Mmulatta
 376594: ........G.....................C........T.....GAAGG   Panubis
 410372: T.C.....G.....................C....A...T.....-----   Cjacchus
1057592: T.......G..................T..C........T.....-----   Sboliviensis

              59960     59970               59986     59996
                  :         :                   :         :
  59951: AGCAGGTAAATCCTGTCCCACAAAAAA----ACACAAGAGTTATCGAGTT   Hsapiens
 379693: ................T..........----...................   Ptroglodytes
 397739: ...........................----.A..............A..   Ggorilla
 433687: ....................T......----...................   Pabelli
 350865: ....................T......----...................   Nleucogenys
 428035: ....................T......----.............TTT...   Mmulatta
 376644: GA..................T......----.............TTT...   Panubis
 410417: ....................T......----.............G.....   Cjacchus
1057637: ...........T.......GT......AAAT.............G.....   Sboliviensis

              60006     60016     60026     60036     60046
                  :         :         :         :         :
  59997: AGTAATAACTCAAGCAAGAGTTATTCTGTGGTTTCAGTTTAAAAGCTTGG   Hsapiens
 379739: ..................................................   Ptroglodytes
 397785: ..................................................   Ggorilla
 433733: ....G.............................................   Pabelli
 350911: ....................................C.............   Nleucogenys
 428081: .........................................G........   Mmulatta
 376690: .........................................G........   Panubis
 410463: .......GT...........C..G.....................T....   Cjacchus
1057687: ...............................C..................   Sboliviensis

              60056     60066     60076     60086     60096
                  :         :         :         :         :
  60047: TGGGAGCAAGAAGCCAGCCCTACCTAAAGTGAATTCAGAGGCTGAGTAAT   Hsapiens
 379789: .........C........................................   Ptroglodytes
 397835: .CT......C.....................................C..   Ggorilla
 433783: .........C...........G............G..........A.C..   Pabelli
 350961: .........C.....................................C..   Nleucogenys
 428131: .........C..............C.........G.......C....T..   Mmulatta
 376740: .........C..............C.................C....T..   Panubis
 410513: .........C..................A..G.A.............C..   Cjacchus
1057737: ....G....C...................C.................C..   Sboliviensis

              60106     60116     60126     60136     60146
                  :         :         :         :         :
  60097: GATTCAGGAGCCAGGAAGAAAAATTACAGATTAGAGGTAGGAATGAGAGT   Hsapiens
 379839: ............G.....................................   Ptroglodytes
 397885: ...........GG.....................................   Ggorilla
 433833: ..........TGG...............C.....................   Pabelli
 351011: ..........TGGA............................G.......   Nleucogenys
 428181: ..........TGG.....................C...............   Mmulatta
 376790: ..........TGG.....................C...............   Panubis
 410563: ..........TTG...........C........C................   Cjacchus
1057787: ..........TTG....................C................   Sboliviensis
```

```
                  60156     60166     60176     60186     60196
                      :         :         :         :         :
  60147: TAGAGACAGATCATGTTTGGGGTGTGCAGTAGGCAGAATCAGTATTACGA  Hsapiens
 379889: ..........................................C.......  Ptroglodytes
 397935: ..................................................  Ggorilla
 433883: ..........................T....................T..  Pabelli
 351061: ....................A.....T.......................  Nleucogenys
 428231: ..........................T...................CT..  Mmulatta
 376840: ..........................T...................CT..  Panubis
 410613: ...........AG........C....T...........G........T..  Cjacchus
1057837: ...........AG........C....T........C......C....A..  Sboliviensis

                  60206     60216     60226     60236     60246
                      :         :         :         :         :
  60197: GAAAAGCCCAGCTTCTCAAAGACCAGTCTTGACCATGCAATCCACAGTCC  Hsapiens
 379939: ...............C..................................  Ptroglodytes
 397985: ...............C..................................  Ggorilla
 433933: ............A..C...............................C..  Pabelli
 351111: ...............C............C..................C..  Nleucogenys
 428281: ...............C...............................C..  Mmulatta
 376890: ...............C...............................C..  Panubis
 410663: ...............C.....G...................GA....C..  Cjacchus
1057887: ...............C.....G...................G.....C..  Sboliviensis

                  60256     60266     60275     60285     60295
                      :         :         :         :         :
  60247: AGCCTCCCCAGGTTGTTACCCC-TGCCAACCAATGATGGCAGACTGTACC  Hsapiens
 379989: ......................-...........................  Ptroglodytes
 398035: ......................-.A.........................  Ggorilla
 433983: ...............A......-.......................C...  Pabelli
 351161: ...............A......-..........C............C...  Nleucogenys
 428331: ...............A......-......T........A.......C...  Mmulatta
 376940: ...............A......-......T........A.......C...  Panubis
 410713: ...............A......T.......T...A.---.......C...  Cjacchus
1057937: ...............A......T.......T......A........C...  Sboliviensis

                  60305     60313     60323     60332     60342
                      :         :         :         :         :
  60296: GCACTCAAGAC--AAGAAATTCCTTTATGGCCAGTGA-TTTTCTCTTCAT  Hsapiens
 380038: ...........AG........................-............  Ptroglodytes
 398084: .T.........AG........................-............  Ggorilla
 434032: .....T....TAG........................-............  Pabelli
 351210: C....T....TAG........................-......T.....  Nleucogenys
 428380: .....T.T..TAG........................-............  Mmulatta
 376989: .....T.T..TAG........................-............  Panubis
 410760: A....T....TAG..A...A.......A.........T............  Cjacchus
1057987: ATG..T....TAG..A...A.......A.....A...-......T.....  Sboliviensis

                  60352     60362     60372     60382     60392
                      :         :         :         :         :
  60343: TTTCAGTGCACAATATTTCTTCTCTTAGTTGAAACTAAACCGTTCTACAA  Hsapiens
 380087: ..............................C...................  Ptroglodytes
 398133: ..............................C...................  Ggorilla
 434081: ..............................C..........A.....T..  Pabelli
 351259: ..............................C..........A....GT..  Nleucogenys
 428429: ......C...G................T..C.G........A........  Mmulatta
 377038: ......C...G................T..C.G........A........  Panubis
 410810: ...........TG.......C........CC..........A........  Cjacchus
1058036: .....A.....TGC......C........CC.........TA........  Sboliviensis
```

```
              60402     60412     60422     60432     60442
                  :         :         :         :         :
   60393: GACAAGAGAACCAGAAAACATGGTTCTAAAATTATAGTCCTAATACCCAT  Hsapiens
  380137: ..................................................  Ptroglodytes
  398183: ......................................G...........  Ggorilla
   96280:                                                ...  Pabelli
  434131: ...............................................     Pabelli
  114749:                                               ....  Nleucogenys
  351309: ..............................................      Nleucogenys
  428479: ..................................................  Mmulatta
  377088: ..................................................  Panubis
  410860: ..............G........G......G.GT.CA........T....  Cjacchus
 1058086: .......................G......G..............T....  Sboliviensis

              60452     60462     60472     60482     60492
                  :         :         :         :         :
   60443: TTTGCCCACAAGGTTATCATAAAAATAACTGTACACAACCAATCACACAG  Hsapiens
  380187: ...............C..................................  Ptroglodytes
  398233: ...............C..................................  Ggorilla
   96283: C...T..............AGG....                          Pabelli
  434200:                           .................T.T....  Pabelli
  114753: C...T..............AGG....                          Nleucogenys
  351378:                           .................T......  Nleucogenys
  428529: ...............C.........C...AA..........G........  Mmulatta
  377138: ...............C.........................G........  Panubis
  410910: .....A..T......C...A..............................  Cjacchus
 1058136: .....A..T......C............T.....................  Sboliviensis

              60501     60511     60521     60531     60541
                  :         :         :         :         :
   60493: AACTAAAA-GGGATCCCAAATCTGTCCAGTCTTTTTCTGCTCAGAGCACA  Hsapiens
  380237: ........-.........................................  Ptroglodytes
  398283: ........-.........................................  Ggorilla
  434224: ........-......T...........C......................  Pabelli
  351402: ........-.........................................  Nleucogenys
  428579: ........G.........G............---................  Mmulatta
  377188: ........-.........................................  Panubis
  410960: ........-..........G.TG...........C...............  Cjacchus
 1058186: .G....G.-..........G...........--.C...............  Sboliviensis

              60551     60561     60571     60581     60591
                  :         :         :         :         :
   60542: TGTTTTCTGACATGAATCAATTCCAGCTGGGGGCTTTCAGAGCTTAAAAA  Hsapiens
  380286: ..................................................  Ptroglodytes
  398332: ..................................................  Ggorilla
  434273: ...............G........................G.........  Pabelli
  351451: ...............G........................G.........  Nleucogenys
  428626: ........................................G.........  Mmulatta
  377237: ........................................G.........  Panubis
  411009: ...............G........................G........G  Cjacchus
 1058233: C..............GAT...............A......GA.C.....G  Sboliviensis

              60601     60611     60621     60631     60641
                  :         :         :         :         :
   60592: CCTCATATCTACCATTAGGGCTGATCTTCTACCTTTCGACTTTGTTTTCA  Hsapiens
  380336: ..................................................  Ptroglodytes
  398382: ..................................................  Ggorilla
  434323: -...........T.....................................  Pabelli
  351501: ......C.....T..............................C......  Nleucogenys
  428676: ....G..........................A.....A............  Mmulatta
  377287: .....................................A............  Panubis
  411059: ...................A..............................  Cjacchus
 1058283: .................A................................  Sboliviensis
```

```
               60651               60669     60679     60689
   60642: TCTCTCTTTAGATTCCAT--AAAAAGGGGGTTGAATTTCTTAGCTTTTTT  Hsapiens
  380386: .....T............--.........A....................  Ptroglodytes
  398432: .G................--..............................  Ggorilla
  434372: ................G.--............................C.  Pabelli
  351551: ....A.............--............................C.  Nleucogenys
  428726: ...............TG.--........A.....................  Mmulatta
  377337: ................G.--........A.....................  Panubis
  411109: ........G.........-T................C...........--  Cjacchus
 1058333: ........G.........AA................C...........--  Sboliviensis

                         60701     60711     60721     60731
   60690: TT--------TTTCTGTCTAAGCATTGGGGTCATCAAAGAAGGTATTTTT  Hsapiens
  380434: ..T-------..................................C.....  Ptroglodytes
  398480: ..--------..................................C.....  Ggorilla
  434420: ..TT------..................................C.....  Pabelli
  351599: ..TT------..............................C...C.....  Nleucogenys
  428774: ..TTTTTC--.....................A..................  Mmulatta
  377385: ..TTTTTTTT.....................A..................  Panubis
  411156: ----------A..........C....A.................C....C  Cjacchus
 1058381: ----------...........C....A.................C....C  Sboliviensis

               60741     60751     60761     60771     60781
   60732: ATGCCTTTGATCTAGATTGTACTCCTGTTATTTTCCCTGTCGCAGAGGAA  Hsapiens
  380477: .....................T............................  Ptroglodytes
  398522: .....................T..................T.........  Ggorilla
  434464: .....................T....................G.......  Pabelli
  351643: ................C....T....................G.......  Nleucogenys
  428822: .....................G.....C............T.G.......  Mmulatta
  377435: .....................T.....C..............G.......  Panubis
  411196: ..A.............C....T................T..AA.A.....  Cjacchus
 1058421: .GA...G.........C....T...................AG.AG.A..  Sboliviensis

                               LTR
             ------------------------------------------------>
               60791     60801     60811     60821     60831
   60782: GTACTTATTTCTGATTTTTCCTTCCAGTTGGAAGGACATCCATTAGCATA  Hsapiens
  380527: ...T.......................................C......  Ptroglodytes
  398572: ..................................................  Ggorilla
  434514: A.......................................T.........  Pabelli
  351693: ........................................T.........  Nleucogenys
  428872: ...........................C.............G........  Mmulatta
  377485: ...........................C.............G........  Panubis
  411246: ......CG.............................T..TG........  Cjacchus
 1058471: .......G................................T....A....  Sboliviensis

                            LTR                        LTR
          ------------------------------------------> --->
               60841     60851     60861     60871     60880
   60832: ACACTCTTTGCCTCTCTTTACCCTCAAATTTACTCCTGCTCCCC-AATGT  Hsapiens
  380577: ............................................-.....  Ptroglodytes
  398622: ............................................-.....  Ggorilla
  434564: ....................T.......................-.....  Pabelli
  351743: ....................T....G..................-.....  Nleucogenys
  428922: .........................G..................-.....  Mmulatta
  377535: .........................G..................-.....  Panubis
  411296: ........C...........T....G....C.............T.G...  Cjacchus
 1058521: ........CA..........T....G....C.............T.G...  Sboliviensis
```

```
                                     LTR
          --------------------------------------------------->
                60890     60900     60910     60920     60930
                    :         :         :         :         :
   60881: CTTAGCCTGGATTCCCTGAAAGCAGAGCCCTAGATAAAGGCTCACATGCA  Hsapiens
  380626: ..................................................  Ptroglodytes
  398671: ..................................................  Ggorilla
  434613: ...........................T......................  Pabelli
  351792: ...........................T......................  Nleucogenys
  428971: ...............................G........T.....C...  Mmulatta
  377584: ...............................G........T.........  Panubis
  411346: .....T........T........G......C...................  Cjacchus
 1058571: ....AT........T........G......C......TA--CT.......  Sboliviensis

                                     LTR
          --------------------------------------------------->
                60940     60950     60960     60970     60980
                    :         :         :         :         :
   60931: TGTAGTTTATTTAGGAAGTGGTGTCAGGGAATAAAAGCAGAAGAGAAAGA  Hsapiens
  380676: ..................................................  Ptroglodytes
  398721: ...............................................G..  Ggorilla
  434663: ..................................................  Pabelli
  351842: ................................................A.  Nleucogenys
  429021: ...........C..........A........A..............C...  Mmulatta
  377634: ...........C..........A........A....A.........C...  Panubis
  411396: ............G...G.....C.............AT............  Cjacchus
 1058619: ............G.........C.............AT....A.......  Sboliviensis

                                     LTR
          --------------------------------------------------->
                60990               61003     61013     61023
                    :                   :         :         :
   60981: GGGGTGGAACAGAG-------ATGGAAAGAAAGTTAATACAATTCTTACA  Hsapiens
  380726: ..............-------...........C.................  Ptroglodytes
  398771: ..............-------.C...........................  Ggorilla
  434713: ..............-------.............................  Pabelli
  351892: ..............-------.............................  Nleucogenys
  429071: ..............-------............C...C..G.........  Mmulatta
  377684: ..............-------............C...C..G.........  Panubis
  411446: .....A........-------..C..........................  Cjacchus
 1058669: .....A........TTAAAAA.AAA...A.....................  Sboliviensis

                                     LTR
          --------------------------------------------------->
                61033     61043     61053     61063     61073
                    :         :         :         :         :
   61024: AAATTGGCCACTGGTATCTGAATCCATGGACTTTCGGAGGAATCTTACAA  Hsapiens
  380769: ..................................................  Ptroglodytes
  398814: ..................................T...............  Ggorilla
  434756: ................C........G........T...............  Pabelli
  351935: .............A..C........G........T...............  Nleucogenys
  429114: ................C.................T.............C.  Mmulatta
  377727: ................C.................T.............C.  Panubis
  411489: C.....A.........C.................T..........A..C.  Cjacchus
 1058719: C...G...........C.........C.......-........A......  Sboliviensis
```

```
                                   LTR
         ------------------------------------------------->
              61083     61093     61103     61113     61123
  61074: AATGTGCATCAGAGCTGTCCACCGAGAGGAAGAAGAGGGGAAGCATGTAT  Hsapiens
 380819: ..................................................  Ptroglodytes
 398864: ..................................................  Ggorilla
 434806: .......C........A......C.............A........A...  Pabelli
 351985: .......C...............C......................A..C  Nleucogenys
 429164: .......C...............C....................G.A...  Mmulatta
 377777: .......C...............C....................G.A...  Panubis
 411539: ..C.CATC...............C..................A...A...  Cjacchus
1058768: ......TC...............C.........G..A.....---.A...  Sboliviensis
```

```
                                   LTR
         ------------------------------------------------->
              61133     61143     61153     61163     61173
  61124: CTACAGGCTTGTATCCCCATAGGTCAAGGAAGGTCCCATGGGTGTTAACT  Hsapiens
 380869: ..................................................  Ptroglodytes
 398914: ..................................................  Ggorilla
 434856: ....................T.............................  Pabelli
 352035: ....................T..........................C..  Nleucogenys
 429214: ....................T.............................  Mmulatta
 377827: ......T.............T.............................  Panubis
 411589: ...TG.....TC.C.T....T......A..G...G.T...A...G.....  Cjacchus
1058815: ...TG.....TC...T....T.........G..CG.........G....C  Sboliviensis
```

```
                                   LTR
         ------------------------------------------------->
              61183     61189     61199     61208     61218
  61174: CGGATCACACT----TGAGTGTCAATTATGCACATT-CACACCAAAGCAT  Hsapiens
 380919: ...........----.....................-.............  Ptroglodytes
 398964: ...........----.....................-.............  Ggorilla
 434906: .A....G....----..........G..........-.............  Pabelli
 352085: ......G....----..........G..........-.............  Nleucogenys
 429264: ......G....----..........G..........-.............  Mmulatta
 377877: T.....G....----..........G..........-.............  Panubis
 411639: .A.........TGCA..........A.G........C.............  Cjacchus
1058865: T..........TGCC..........A.G........C.............  Sboliviensis
```

```
                                   LTR
         ------------------------------------------------->
              61228     61238     61248     61258     61268
  61219: CATCACAAAGCCCTGGAACTGGAAGCAAGGTGTGAGAGGAAGTGCTGCCA  Hsapiens
 380964: ..................................................  Ptroglodytes
 399009: .............C....................................  Ggorilla
 434951: .................G.......A........................  Pabelli
 352130: .C...............GG...............................  Nleucogenys
 429309: ....G............G................................  Mmulatta
 377922: ....G............G................................  Panubis
 411689: .................G...........................C....  Cjacchus
1058915: .................G..................A.............  Sboliviensis
```

```
                  LTR
           61278     61288     61298     61308     61315
   61269: GTTTGCAATGCACAAAGTTATTTAAAGCCTATGCAGAACTGG---TGGCC   Hsapiens
  381014: .A........................................---.....   Ptroglodytes
  399059: .A........................................---.....   Ggorilla
  435001: .A........................A...............---.....   Pabelli
  352180: .A........................................---.....   Nleucogenys
  429359: .AA..........C............................TGC.....   Mmulatta
  377972: .A...........C............................TGC.....   Panubis
  411739: .G.....................G..................---..A..   Cjacchus
 1058965: .G........................................---.....   Sboliviensis

                  LTR
           61325     61335     61345     61355     61365
   61316: TCAGCAGCAGCTAGATTAAGAGGTAGGGCAGCAGAATATGAAGTGACTAA   Hsapiens
  381061: ............................TG....................   Ptroglodytes
  399106: ............GA............C.TG....................   Ggorilla
  435048: ......A.....................TG....................   Pabelli
  352227: .............................G....................   Nleucogenys
  429409: ............G...............TG......C.............   Mmulatta
  378022: ............G...............TG......C.............   Panubis
  411786: .......A...................ATGA........A.........    Cjacchus
 1059012: .......A.............A......TGA..................    Sboliviensis

          LTR
           61375     61385     61395     61405     61415
   61366: CAAAAGGCGTCTAACACATCTAACCTATGTTGGTGGGTTTGGAGAGGAAA   Hsapiens
  381111: ..................................................   Ptroglodytes
  399156: ..................................................   Ggorilla
  435098: .-......A.......................A.................   Pabelli
  352277: ........A................A........................   Nleucogenys
  429459: ........A.................G..................A....   Mmulatta
  378072: ........A.................G..................A....   Panubis

                            Simple
           61425     61435               61440     61450
   61416: TGCCCTCAAGTTTTGTTTTGTT---------------TTGTTTTGTTTTG   Hsapiens
  381161: ......................---------------..T.........-   Ptroglodytes
  399206: .......C..........C...---------------............-   Ggorilla
  435147: ......................---------------....---------   Pabelli
  352327: ......................----------------------------   Nleucogenys
  429509: ......................TTGTCTTGTTTTGTC....C........   Mmulatta
  378122: ......................----------TTATC.........C...   Panubis

          Simple
           61460     61470     61480     61490     61500
   61451: TTTTGTTTTGTTTTGTTTTCACAGGTTGCCAAGCGTCCAAAGGAAGTATG   Hsapiens
  381195: ---------.........................A...............   Ptroglodytes
  399240: ---------.........................................   Ggorilla
  435174: ---------............T...GA.......................   Pabelli
  352349: ---------............T...GA.......................   Nleucogenys
  429559: ...................T.T...CA.....A.................   Mmulatta
  378162: .C.................T.T...CA.......A...............   Panubis
```

```
                    61510     61520     61530     61540     61550
                        :         :         :         :         :
  61501: GATTCTCATCTATTCCTTCTGCCCTAAGTTTTCCCAGGGCTAAGCCCAAT   Hsapiens
 381236: ........................................C.........   Ptroglodytes
 399281: ........................................C........C   Ggorilla
 435215: ..........................G.......T.....C.....TG..   Pabelli
 352390: ..................................T.....C.........   Nleucogenys
 429609: ........................................C.....TG..   Mmulatta
 378212: ........................................C.....TG..   Panubis

                                                  LTR
                                             --------->
                    61560     61570     61580     61590     61600
                        :         :         :         :         :
  61551: TTTGAAACTCCAGTAATCCCTGTGACTAGATTGTACACCAAAGAATTAGA   Hsapiens
 381286: ..............................C...................   Ptroglodytes
 399331: ..................................................   Ggorilla
 435265: .............................G.....AGT............   Pabelli
 352440: ............................AG.......T............   Nleucogenys
 429659: .............................G.......T............   Mmulatta
 378262: .........T...........A.......G.......T............   Panubis

                                  LTR
         ------------------------------------------------->
                    61610     61620     61630     61640     61650
                        :         :         :         :         :
  61601: AAACCTATTATTGGCTCATGCCTAATCCCCAGCCTCCAACCTATCATTAT   Hsapiens
 381336: ..................................................   Ptroglodytes
 399381: ..................................................   Ggorilla
 435315: ..................................................   Pabelli
 352490: .....------------------------------------.........   Nleucogenys
 429709: ................................A......TT.........   Mmulatta
 378312: ................................A......T.........C   Panubis

                                  LTR
         ------------------------------------------------->
                    61660     61670     61680     61690     61700
                        :         :         :         :         :
  61651: GTAATGTATATTTTTATTCTCTATCTGTCTGATTTATTTGACTTTATATT   Hsapiens
 381386: ..................................................   Ptroglodytes
 399431: ..G...............................................   Ggorilla
 435365: .C...A................G....C...................C..   Pabelli
 352504: .C...A.....................C...............   Nleucogenys
 429759: .....A.....................C...................C..   Mmulatta
 378362: .....A.------------------------.C..............C..   Panubis

                                LTR
         -------------------------------------------->
                    61710     61720     61730     61740     61750
                        :         :         :         :         :
  61701: CATTTTTTCAGGACTCAGGTGAGGTGAGGGGAGTTATTTATTAGGGACTT   Hsapiens
 381436: ...................A..............................   Ptroglodytes
 399481: ...................A....................C.........   Ggorilla
 435415: ...............T..................................   Pabelli
 429809: ..................................................   Mmulatta
 378388: ..................................................   Panubis
```

```
                 61760     61770     61780     61790     61800
                     :         :         :         :         :
 61751: CATAACCTAGGACAGTGATCATGGGCTGGAGAGCAGAGGATGGACTAAAT  Hsapiens
381486: ..................................................  Ptroglodytes
399531: .G......G....G....................................  Ggorilla
435465: ......................A.......................G.C.  Pabelli
429859: ................................................T.  Mmulatta
378438: ..............A.................................T.  Panubis

                 61810     61820     61830     61840     61850
                     :         :         :         :         :
 61801: ACTGCCCCCAGCCACCCGCCTTGTCTTGGGTAGGACACTTACATCACCTG  Hsapiens
381536: ..................................................  Ptroglodytes
399581: ..................................................  Ggorilla
435515: ................................................A.  Pabelli
429909: .................A................................  Mmulatta
378488: .................A................................  Panubis

                 61860     61870     61880     61890     61900
                     :         :         :         :         :
 61851: TAACTGATGGCCCTTGTCACAAGTTAGAGGAGGAGGAGGATTTCAAGGAA  Hsapiens
381586: ..................................................  Ptroglodytes
399631: ..................................................  Ggorilla
435565: ..................................................  Pabelli
429959: ..................................................  Mmulatta
378538: ................GT................................  Panubis

                 61910     61920     61930     61940     61950
                     :         :         :         :         :
 61901: GAACTTGGAGGCAATCTGGGGGAATAAGTAATCTTAGCAGAAGTTGCTTT  Hsapiens
381636: ....C.............................................  Ptroglodytes
399681: ....C.............................................  Ggorilla
435615: ....C.............................................  Pabelli
430009: ....C.............................................  Mmulatta
378588: ....C.............................................  Panubis

                 61960     61970     61980     61990     62000
                     :         :         :         :         :
 61951: AGTAGAGTACTGAGGGAAAAATCTAGGCTGCAGTGCATCATAGAACACGC  Hsapiens
381686: ..................................................  Ptroglodytes
399731: ..................................................  Ggorilla
435665: ................................................A.  Pabelli
430059: ...G.G..........................................A.  Mmulatta
378638: ...G.G..........................................A.  Panubis

                 62010     62020     62030               62045
                     :         :         :                   :
 62001: TAGAGAAGAACACACCCTTTCCCAGAGTTTGGCAGTA-----AAGTCGTG  Hsapiens
381736: ..............................A......-----........  Ptroglodytes
399781: .....................................-----........  Ggorilla
435715: .....................................AAGTT.....A..  Pabelli
430109: ..C............................T.-----------------  Mmulatta
378688: ..C.......T....................C.G...-----........  Panubis

                 62055     62065     62075     62085     62095
                     :         :         :         :         :
 62046: ACATAGGTGATGTTAGAGGAAGCCAAGTTGAAGAACCCTGTTAAGCATGA  Hsapiens
381781: ...................................T..............  Ptroglodytes
399826: ..................C................T..............  Ggorilla
435765: .T.................................T..............  Pabelli
430142: --....A............................T..............  Mmulatta
378733: ......A............................T..............  Panubis
```

```
             62105     62115     62125     62135     62145
                 :         :         :         :         :
 62096: CTTTACCCTACTCTGACAGGGAAAAGCAATGGACAATGTGTATTGACAAT  Hsapiens
381831: ..........................A.......................  Ptroglodytes
399876: ..................................................  Ggorilla
435815: ............................................C.....  Pabelli
430190: ...-----......T.............................C.....  Mmulatta
378783: ...-----......T.............................C.....  Panubis

             62155     62165     62175     62185     62195
                 :         :         :         :         :
 62146: GTGACTCCTTCCGCTGCCCTCTCAGAGGAAGGAGATCACAAGAGCTGAAG  Hsapiens
381881: ............C.....................................  Ptroglodytes
399926: ............C.....................................  Ggorilla
435865: ............C.............A.......................  Pabelli
430235: .....CA.....C...........................G...T.....  Mmulatta
378828: .....CA.....C.....................................  Panubis

                                          LTR
                                    --------------------->
             62205     62215     62225     62235     62245
                 :         :         :         :         :
 62196: ATCCAAGACCACCTCTGTGGTTTCGTTAGTGATATGGTTTGGGTCTGTGT  Hsapiens
381931: ........T.................................C.......  Ptroglodytes
399976: ..........................................C.......  Ggorilla
435915: ........................A.................C.......  Pabelli
430285: ........................A..........T......CG......  Mmulatta
378878: ........................A..........T......TG....T.  Panubis

                             LTR
        ------------------------------------------------->
             62255     62265     62275     62285     62295
                 :         :         :         :         :
 62246: CCCCACCAAAATCTCCTCTCGAATTGTAATCCCCATAATCCCCATGTGTT  Hsapiens
381981: ..................................................  Ptroglodytes
400026: ..................................................  Ggorilla
435965: .......C..........................................  Pabelli
430335: .......C............A..............C........GA....  Mmulatta
378928: .......C.....................................A....  Panubis

                             LTR
        ------------------------------------------------->
             62305     62315     62325     62335     62345
                 :         :         :         :         :
 62296: GAGGAAGAGACCAGGTGGGAGGTGATTGGATCATGGGGATGGTTTCCCCT  Hsapiens
382031: ..................................................  Ptroglodytes
400076: ..................................................  Ggorilla
436015: .......G..........................................  Pabelli
430385: .......G...........................A..C.......T..C  Mmulatta
378978: .......G......................................T..C  Panubis

                             LTR
        ------------------------------------------------->
             62355     62365     62375     62385     62395
                 :         :         :         :         :
 62346: GTGCTGTTCTCACGATAGTGAGTGAGTTCTCATGAGATCTGATGGTTTTA  Hsapiens
382081: .............A..............................AC....  Ptroglodytes
400126: .............A..............................A.....  Ggorilla
436065: .....................................C............  Pabelli
430435: ....G...A.........................................  Mmulatta
379028: ........A..G............................C.........  Panubis
```

```
         LTR -->                             Simple -->
                 62405     62415                   62423     62433
 62396: TAAGGGGCCCTTCCTGCTTCG------------CTCTCTCTCTCTCTCTC  Hsapiens
382131: .....................----------------------.......  Ptroglodytes
400176: .....................CTCTCTCTCTCT.................  Ggorilla
436115: ..............C....T.------------------------T....  Pabelli
430485: ........T...........A--------------------------...  Mmulatta
379078: ........T....TC.....A--------------------------...  Panubis

         Simple -->  LTR -->
                 62443     62453     62463     62473     62483
 62434: TCTCTCTCTCTCTAATCTGCCTCCATGTAAGATGTTCCTACTTCTCCTTC  Hsapiens
382159: .....G.........C.A.....................G..........  Ptroglodytes
400226: ...............C.A.....................G..........  Ggorilla
436141: ...............C.......................G....C.....  Pabelli
430509: .........T.............................G....C.T...  Mmulatta
379102: .........T.............................G....C.T...  Panubis

         LTR -->
                 62493     62503     62513     62523     62533
 62484: TGCCATGATTGTGTTTCCTGAGGTCTCCCCAACCATGTGGAACTATGACT  Hsapiens
382209: ..................................................  Ptroglodytes
400276: ..................................................  Ggorilla
436191: ...............................G............GA..G.  Pabelli
430559: ................G.....A........G..........A.G...G.  Mmulatta
379152: ...............................G..........A.G...G.  Panubis

         LTR -->
                 62543     62553     62563     62573     62583
 62534: CAACTAAATGTCTTTCCTTTATAAATTACCAAGTCTCAGGTAGTATTCTT  Hsapiens
382259: ........CC........................................  Ptroglodytes
400326: ........CC........................................  Ggorilla
436241: ...T....CC........................................  Pabelli
430609: ...T....CT..................................G.....  Mmulatta
379202: ...T....CT........................................  Panubis

         LTR -->
                 62593     62603     62613     62623     62633
 62584: TATGGCAATGTGAAAATGAACTAATACAATTAGGGGTGATGGGTAAAGGG  Hsapiens
382309: ..C...............................................  Ptroglodytes
400376: ..................................................  Ggorilla
436291: ..CA...G........C.................T...............  Pabelli
430659: .......G......................C...................  Mmulatta
379252: .......G......................C...................  Panubis

                 62643     62653     62663     62673     62683
 62634: AAGGTGTGGGTACAAAAGGGAAAAGAAAAAAAATTACTGTAGAATCAAGA  Hsapiens
382359: ..................................................  Ptroglodytes
400426: ..................................................  Ggorilla
436341: ..........-.....................T.......G.........  Pabelli
430709: ........C.......................T.................  Mmulatta
379302: ........C.......................T.................  Panubis
 78122:              ....AAA....A..............AG..CC.C.A.  Sboliviensis
```

```
             62693     62703     62713     62723     62733
 62684: TATTCTACCAGATTTTTTAAGAATAAAATTGGACAAAGAGGAATCTGTTT  Hsapiens
382409: .......G......G...................................  Ptroglodytes
400476: .......G......G..............C....................  Ggorilla
436390: .......G....C.C.......GC..........................  Pabelli
430759: .......G.........A.......G..............A.........  Mmulatta
379352: .......G.................G..............A.........  Panubis
 78159: -----G.G.T.T..C.....A..A...TG.A..                  Sboliviensis

             62743     62753     62763     62773     62783
 62734: AGACAGCATTAGCTGTTCTTGGGAGCTTGGGGTGGGAGTCGGGATAAAGA  Hsapiens
382459: .......................................GA.........  Ptroglodytes
400526: .......................................G..........  Ggorilla
436440: ........C..............................G..T.......  Pabelli
430809: .......................................G..........  Mmulatta
379402: .................T.....................G..........  Panubis

             62793     62803     62813     62823     62833
 62784: AGACTCTGGGAGCATCTGCCCTATGTGTGGGAGATAGAAAAGGGTAGAGA  Hsapiens
382509: ..................................................  Ptroglodytes
400576: .............C....................................  Ggorilla
436490: ...........A...............A.T....................  Pabelli
430859: C............C...........A.........T..............  Mmulatta
379452: C............C...........A.....G...T..............  Panubis

             62843     62853     62863     62873     62883
 62834: ATGATGACTTTCAGGAGTTTTTACGTGCATTATGGATGTCATCCTGCTTT  Hsapiens
382559: ........................T....................T....  Ptroglodytes
400626: ........................T....................T....  Ggorilla
436540: ........................T.................T..T.C..  Pabelli
430909: ........................TC...................T....  Mmulatta
379502: ........................TC...................T....  Panubis

                              DNA
         ------------------------------------------------>
             62893     62903     62913     62923     62933
 62884: GTAGGATTGCAAAATTTAGCAAATAAAAATACAGGATGACCAGTTAAATT  Hsapiens
382609: ..................................................  Ptroglodytes
400676: ...........................................C......  Ggorilla
436590: ..................................................  Pabelli
430959: T.................................................  Mmulatta
379552: T.................................................  Panubis

                              DNA
        ------------------------------------------------->
             62943     62953     62963     62973     62983
 62934: TGAATTTCAGACAAATGATGAATATTTTTATAAGTATATACCAAATATTG  Hsapiens
382659: ..........G.......................................  Ptroglodytes
400726: ..........G.......................................  Ggorilla
436640: ...........A....T......................C..........  Pabelli
431009: ....................G............A.....C..........  Mmulatta
379602: ....................G............A.....C..........  Panubis
```

```
                              DNA
        ---------------------------------------------------->
                 62993     63003     63013     63023     63032
                     :         :         :         :         :
 62984: TATGGGACAGATTTATACTACATATGGGGCATATTTACATG-AAAAAAGT  Hsapiens
382709: .........................................-........  Ptroglodytes
400776: .........................................-........  Ggorilla
436690: .....................................T...A........  Pabelli
431059: ....A..............GT...A............T...-..G.....  Mmulatta
379652: ....A..............GT...A............T...-..G.....  Panubis

                              DNA
        ---------------------------------------------------->
                 63042     63052     63062     63072     63082
                     :         :         :         :         :
 63033: ATTCATTGTTTATCTGAAATTCAAATTTAACTGGGTGGTCTGTATTTTAT  Hsapiens
382758: ..................................................  Ptroglodytes
400825: ..................................T...............  Ggorilla
436740: .....C......C........................T............  Pabelli
431108: ................................A....T............  Mmulatta
379701: ................................A....T............  Panubis

             DNA
        ------------>
                 63092     63102     63112     63122     63132
                     :         :         :         :         :
 63083: CTGACAACCTTACCTCTTTGGCATTTTCTTGAGAGATTGTGATAAATACT  Hsapiens
382808: .........A........C...............................  Ptroglodytes
400875: .........A.............................C..........  Ggorilla
436790: .........................................G........  Pabelli
431158: ................................C........G........  Mmulatta
379751: ................................C........G........  Panubis

                 63142     63152     63162     63172     63182
                     :         :         :         :         :
 63133: TACATTTGCTTACATTTTTTTTATCCAAACACAAGAATTAAGAAAAGTCT  Hsapiens
382858: ..T...............................................  Ptroglodytes
400925: ..T........C.T....................................  Ggorilla
436840: ..T..............C...G............................  Pabelli
431208: .................C...G...                           Mmulatta
379801: .................C...G...                           Panubis

                 63192     63202     63212     63222     63232
                     :         :         :         :         :
 63183: ATAAGGCAGCTCAACGTTAAGCTCTATGCTAAATTTGTATGAGGAAGGGA  Hsapiens
382908: ........T.........................................  Ptroglodytes
400975: ..................................................  Ggorilla
436890: ...............A.........................TTA......  Pabelli

                 63242     63252     63262     63272     63282
                     :         :         :         :         :
 63233: AGAAAATGGACCACAGGTTCCTGCTGTTATAGGTAAGAAATGGGACAATT  Hsapiens
382958: ..................G.............A...............C.  Ptroglodytes
401025: ..................G.............A.................  Ggorilla
436940: ................T.G.............A.................  Pabelli

                 63292     63302     63312     63322     63332
                     :         :         :         :         :
 63283: GATGGAGAAAAGCTTCCCAAAGAGACTAAGGGAGCAAGAACAATGCAGGT  Hsapiens
383008: ..................................A.......G.......  Ptroglodytes
401075: ..........................................G.......  Ggorilla
436990: ..............................A...................  Pabelli
101463:                          ....CA..AA.....A.G......A  Cjacchus
```

```
             63342     63352     63362     63372     63382
                 :         :         :         :         :
 63333: TGAAGAACCAACTTTCCCCAAAATGAAAGACAACTCTTTCCAAACATAAG  Hsapiens
383058: ............................................T.....  Ptroglodytes
401125: ..................................................  Ggorilla
437040: G.....GT.-........................................  Pabelli
352547:                                                  .  Nleucogenys
139222:                 ...T..T.C..T...TG....C.TT...----..  Mmulatta
101488: ......                                              Cjacchus

             63392     63402     63412     63422     63432
                 :         :         :         :         :
 63383: AGTAGAGAAGTTTGGATACATATTTAAATACATTTTTATATAGGAGATTT  Hsapiens
383108: ..................................................  Ptroglodytes
401175: ..................................................  Ggorilla
437089: ..............................T........G.G........  Pabelli
352548: ..............................T......G............  Nleucogenys
139252: .AG..G...A............CG.GG..                       Mmulatta

             63442     63452     63462     63472     63481
                 :         :         :         :         :
 63433: CAAAGTTTTGCCTGGATTGTCAATTTCTCATGGACAAGTATCGTGC-TTT  Hsapiens
383158: ...............G......G..............T........-...  Ptroglodytes
401225: ...............G......G.......................-...  Ggorilla
437139: .............A...........................T....-...  Pabelli
352598: .......................................G.T....T...  Nleucogenys

             63491     63501     63511     63521     63531
                 :         :         :         :         :
 63482: TTGCTAAGTTTCGGGCATTTAGTAAGCACTTAATTATCATTGGCTAAACT  Hsapiens
383207: ............T............................T........  Ptroglodytes
401274: ............T............................T........  Ggorilla
437188: ..........C.T.................C.....C....T........  Pabelli
352648: ............T.................C.....C....T...T....  Nleucogenys

             63541     63551     63561     63571     63581
                 :         :         :         :         :
 63532: GAACTGAGCATCTTTTTATGAGGTTAGGAGTTGTGTTTTAGGTTGGGATG  Hsapiens
383257: ......C.....................G.....................  Ptroglodytes
401324: ......C.....................G.....................  Ggorilla
437238: .....................T......G.....................  Pabelli
352698: ..........................A.G...........C.C.......  Nleucogenys

             63591     63601     63611     63621     63631
                 :         :         :         :         :
 63582: AGGCAAGAGATGAAGATTTAGAATGGCAATTGGGAAAATTCAAAAGGAAC  Hsapiens
383307: .......................C..........................  Ptroglodytes
401374: ..................................................  Ggorilla
437288: .............GT...C...G........T.................G  Pabelli
352748: ..............................GT.........G.......G  Nleucogenys

             63641     63651     63661     63671     63681
                 :         :         :         :         :
 63632: TGATCAAGAACAGACAAAAGGTTTCCTCACTGACTACCTGCAAAGGCTGG  Hsapiens
383357: ..................................................  Ptroglodytes
401424: ..................................................  Ggorilla
437338: .....................C............................  Pabelli
352798: .................................................C  Nleucogenys
```

```
                  63691     63701     63711     63721     63731
                      :         :         :         :         :
 63682: AGCCACACATTATCAGTAACGTAAAGTGAGAAGGATATGCTAGCAACTGC   Hsapiens
383407: ...................T.........A....................   Ptroglodytes
401474: ...................T..............................   Ggorilla
437388: ...................T...........................C..   Pabelli
352848: ...................T................G.............   Nleucogenys

                  63741     63751     63761     63771     63781
                      :         :         :         :         :
 63732: GTTTTGCTGTGATTTGCAACGTTCCCACCTACCAGGATTCTCAGATCCTT   Hsapiens
383457: ....................A...........A.................   Ptroglodytes
401524: ....................A...........A.................   Ggorilla
437438: A....A.............T..............................   Pabelli
352898: A................C..A.............................   Nleucogenys

                  63791     63801     63811     63821     63831
                      :         :         :         :         :
 63782: GGAATGTGATTGCTGCAACCAACTTCCATTTTTTGCAAAAGTTTAACAAT   Hsapiens
383507: ......................G.....................C.....   Ptroglodytes
401574: ............................................C.....   Ggorilla
437488: ..........CA..A.............................C.....   Pabelli
352948: ..........CA..A.............................C.....   Nleucogenys

                  63841     63851     63861     63871     63881
                      :         :         :         :         :
 63832: TTTCTGACAGGAAATTTTCAAAAACACATTATTTTTCAATAAGATGCCAT   Hsapiens
383557: ..........C.....................................G.   Ptroglodytes
401624: ..........C....................................TG.   Ggorilla
437538: ............C.........G.....G........G..........G.   Pabelli
352998: ............C.........G--...G...................G.   Nleucogenys

                  63891     63901     63911     63921     63931
                      :         :         :         :         :
 63882: AATTCTTACTTTCCCAGACATAAAGCAATTCTTCCAAAAGCAGTCGTGTC   Hsapiens
383607: ..................................................   Ptroglodytes
401674: ..................................................   Ggorilla
437588: .............................................A....   Pabelli
353046: .............................................A....   Nleucogenys

                  63941     63951     63961     63971     63981
                      :         :         :         :         :
 63932: CTGTGAAAACCAAGAATTTTTCAAACAATACCCAGAGCACTTTCCATTAG   Hsapiens
383657: ..................................................   Ptroglodytes
401724: ..................................................   Ggorilla
437638: ..........A..........A......C.....................   Pabelli
353096: ..A..................A......C.....................   Nleucogenys

                  63991     64001     64011     64021     64031
                      :         :         :         :         :
 63982: TCAGACTAGCTCTTGATAGAGACCCAGATATGCAATGAAATGGAAAACGT   Hsapiens
383707: ..................................................   Ptroglodytes
401774: ..................................................   Ggorilla
437688: ................................................A.   Pabelli
353146: .......................T.......................TA.   Nleucogenys

                  64041     64051     64061     64071     64081
                      :         :         :         :         :
 64032: GAAGCACAGACCCAAGAAAAACCATCAACAATGCTTCTCTTTTATTTTTC   Hsapiens
383757: ......................................T...........   Ptroglodytes
401824: .......G..............................T...........   Ggorilla
437738: ..................................................   Pabelli
353196: ..................................................   Nleucogenys
```

```
             64091     64101     64111     64121     64131
                 :         :         :         :         :
 64082: AGGAGGTAGAGAGAGAGCCTTTCAACTACAATGAACAATTCTAAGTACAG  Hsapiens
383807: ..................................................  Ptroglodytes
401874: ..................................................  Ggorilla
437788: ...........                                         Pabelli
438404:            .A.....................................  Pabelli
353246: .................G................................  Nleucogenys

             64141     64151     64161     64171     64181
                 :         :         :         :         :
 64132: GAAGCATGGATGCTCCAAGCCTTGTATAGACATCTTTACTTTAGTCTATT  Hsapiens
383857: .................G.T.....G........................  Ptroglodytes
401924: .................G................................  Ggorilla
438443: ....GG........T....A....C....G.C..G.......C.....CA  Pabelli
353296: .....G....G...T.........C......C------------.T....  Nleucogenys

             64191     64201     64211     64221     64231
                 :         :         :         :         :
 64182: CACTTGGAAAATTTTCCTCAGGCAAGTCAAGAGGAATCTGCTACTTGAAA  Hsapiens
383907: ......................T.......A...................  Ptroglodytes
401974: ......................T...........................  Ggorilla
438493: ..G.....G.............T.............G.............  Pabelli
353334: ......................T..........................G  Nleucogenys

             64241     64251               64266     64273
                 :         :                   :         :
 64232: CAAACTAAAAGAAAACGGGCTATTTATTT-----AAAAAAAAC---TATA  Hsapiens
383957: .............................-AAAA.........---....  Ptroglodytes
402024: ...............A............AAAAAA....C....---....  Ggorilla
438543: ...............A............A-----........TATA....  Pabelli
353384: .................A...........---TA.........---....  Nleucogenys
 78156:              ..A.A...G...C...-----........A---.G..  Sboliviensis

                                                LINE1
                                         ---------------->
             64283     64293     64303     64313     64323
                 :         :         :         :         :
 64274: AAACTCATCAAGAGATGCAGTTAAGAAACTAAGCAAGCCACAGGCTGTGA  Hsapiens
384003: ...............................................C..  Ptroglodytes
402071: ..............................................AA..  Ggorilla
438588: .................                                   Pabelli
438913:                  ........-.....................G..  Pabelli
353428: .....................................G.........G..  Nleucogenys
411846: .....TG.........AG................T.........TG.G.G  Cjacchus
 78185: G.                                                  Sboliviensis

                                LINE1
        ------------------------------------------------->
                       64337     64347     64357     64367
                           :         :         :         :
 64324: GAAA------ATATTCACAATGCAGGCCTAAAAAAAGTATTTGTATACAG  Hsapiens
384053: ....------........................................  Ptroglodytes
402121: ....TGGCAC..G.C......--.A....TCT.C...A...-------.T  Ggorilla
438945: ....------.C............A.........................  Pabelli
353478: ....------.C......................................  Nleucogenys
411896: A...------....................T...................  Cjacchus
```

```
                              LINE1
          ---------------------------------------------------->
                64377     64387     64397     64407     64417
                    :         :         :         :         :
   64368: AATACACAAAAAAGTACGACTCAAAAAGTTAAATACAAATAGGCAAAATA  Hsapiens
  384097: .............C...A.................T..............  Ptroglodytes
  402162: G...G...T..GGA..TCCA....G.G.AA...                   Ggorilla
  438989: ...G.........A...A...............C.T..............  Pabelli
  353522: ...G.............A.................T.............G  Nleucogenys
  411940: .................A.....G...AC......T..............  Cjacchus

                              LINE1
          ---------------------------------------------------->
                64427     64437     64447     64457     64467
                    :         :         :         :         :
   64418: TTTATACAGTCACTTCACTAAAGAATGCATATAAATGGTCAATAAGCACA  Hsapiens
  384147: ..................................................  Ptroglodytes
  439039: .........G......................G.................  Pabelli
  353572: .........G.......T..............G.................  Nleucogenys
  411990: ...G.....G.................T....G.........C.......  Cjacchus

                              LINE1
          ---------------------------------------------------->
                64477     64487     64497     64507     64514
                    :         :         :         :         :
   64468: TAACAATGTGTCCAACATCATTAGTCAGCAGAGATGTGCAAATT---AAA  Hsapiens
  384197: .......................................G....---...  Ptroglodytes
  439089: ..................T...............A....G....---...  Pabelli
  353622: ..................................A....G....AAA...  Nleucogenys
  412040: ..................................A...AT....---...  Cjacchus

                              LINE1
          ---------------------------------------------------->
                64524     64534     64544     64554     64564
                    :         :         :         :         :
   64515: AAAACACAGACATTCCACTTCACAACCATTAGAATGAGGAAAGAAGAAAG  Hsapiens
  384244: ..................................................  Ptroglodytes
  439136: ..................................................  Pabelli
  353672: ........................G.........................  Nleucogenys
  412087: ......T.......T.........C....C.A.G....TC..........  Cjacchus

                              LINE1
          ---------------------------------------------------->
                64574     64584     64594     64604     64614
                    :         :         :         :         :
   64565: ATTGACTACACCAAATGTAGACAAAGATATGGACCAACCAGAATGCTACT  Hsapiens
  384294: .................C............C...................  Ptroglodytes
  439186: ........T..................................CA.....  Pabelli
  353722: ...........................................CT.....  Nleucogenys
  412137: .C........T.............G........G.........G......  Cjacchus

                              LINE1
          ---------------------------------------------------->
                64624     64634     64644     64654     64664
                    :         :         :         :         :
   64615: ACAATGTTCAACCACTTTGGAAAACTGCCAGATTCTTAAAAAGTGAAATA  Hsapiens
  384344: ...G......G..............................G........  Ptroglodytes
  439236: ...G..............A...............................  Pabelli
  353772: ...T..............A.......C.......................  Nleucogenys
  412187: ...G.............................ATG.............G  Cjacchus
```

LINE1 →

```
              64674     64684     64694     64704     64714
  64665: TACATCTACCCTATGACCTAGCAATTCCTTCAAAGAGCCTTTCAACTACA   Hsapiens
 384394: ..................................................   Ptroglodytes
 401885:                                .G.................   Ggorilla
 439286: ..................................................   Pabelli
 353822: ..................................................   Nleucogenys
 412237: .G.......T.........................                  Cjacchus

              64724     64734     64744     64754     64764
  64715: ATGAACAATTCTAAGTACAGGAAGGGCGGATGCTTCAAGACTTGCATAGG   Hsapiens
 384444: ..................G...............................   Ptroglodytes
 401904: ........................CAT.......C..G.C....T....A   Ggorilla
 439336: ..........................T.......................   Pabelli
 353872: .C................................................   Nleucogenys

              64774               64777     64787     64797
  64765: CCTCGTTACT-----------------TTTGTCTACACAGTTGGAGAATT   Hsapiens
 384494: ..........AGAGTTGCATAGGCCTCG......................   Ptroglodytes
 401954: .A..T.....-----------------..A.....TT..C.....A....   Ggorilla
 439386: ..........-----------------..C....................   Pabelli
 353922: ..........-----------------.......................   Nleucogenys

              64807     64817     64827     64837     64847
  64798: TTCCTCAGGGAAGTCAAGAGGAATCTGCTACTTGAAACAAACTAAAAGAA   Hsapiens
 384544: ..................................................   Ptroglodytes
 401987: .........T........................................   Ggorilla
 439419: .........T.............G..........................   Pabelli
 353955: .........T...................G........G...........   Nleucogenys

              64857               64876     64885     64895
  64848: AAAGGGCTATTTATTAAAA-AAAAAAAAAAT-TATAAAACTCATCAAGAG   Hsapiens
 384594: ...............T----..........C-..................   Ptroglodytes
 402037: ...................-......C...C-..................   Ggorilla
 439469: .................---..........C-..................   Pabelli
 354005: ...................C..........C-...............C..   Nleucogenys
 112813:          .C..C.....-...........AC.A......            Panubis
1059093:                                           ........   Sboliviensis

              64905     64915     64925     64935     64945
  64896: ATGCAGTTAAGAAACTAAGCAAGCCACAGGCTAAGAGAAATGGCACATGT   Hsapiens
 384639: .............................................T....   Ptroglodytes
 402085: ..................................................   Ggorilla
 439515: ..................................................   Pabelli
 354054: ........................................C.........   Nleucogenys
 412277:                             .................T.A..   Cjacchus
1059101: ..A.................T.......                         Sboliviensis
1059514:                             .................T.A..   Sboliviensis
```

```
              64955     64965     64975     64985
  64946: CCACAATAAGCCTTCTACAAGAATTATGATAGACATAAGG----------   Hsapiens
 384689: ....G...................................GCCACACATT   Ptroglodytes
 402135: ........................................----------   Ggorilla
 439565: .........................G..............GCCACACGTT   Pabelli
 354104: ...............................C........GCCACACACT   Nleucogenys
 412299: ........................---..C...TG.....GCCACACGTT   Cjacchus
1059536: ........................---..C...TG.....GCCACACATT   Sboliviensis

                              LINE1
           ----------------------------------------------->
              64993     65003     65013     65023     65033
  64986: --ATATCCATCAAGAGGAAAAAGCATGAATAAACTCCGTTGCATCCGTAC   Hsapiens
 384739: GG....................------------------..........   Ptroglodytes
 402175: --................................................   Ggorilla
 439615: GG.............A..............................A...   Pabelli
 354154: GG............................................A...   Nleucogenys
 412346: AT...........................C......T..-......A...   Cjacchus
1059583: ---..........................C......T.........A.G.   Sboliviensis

                             LINE1
         ------------------------------------------------->
              65043     65053     65063     65073     65083
  65034: AATGGAACATACACAGCATTAAAAAGCCCCAAACTACTGATGCACACAAC   Hsapiens
 384771: .........C........................................   Ptroglodytes
 402223: ..................C...............................   Ggorilla
 439665: ..................................................   Pabelli
 354204: ..........................                           Nleucogenys
 412395: .......T.CCTGA......T.....-..........A......T.....   Cjacchus
1059630: .........CC.........T................AA.....T.....   Sboliviensis

                             LINE1
         ------------------------------------------------->
              65093     65103     65112     65122     65132
  65084: AACATAGACAAATATAAAAAGTGTCAT-GCTGAGTGAGAGAGGCCAGGAC   Hsapiens
 384821: ....C......G.........CA....-......................   Ptroglodytes
 402273: ......................A....-......................   Ggorilla
 439715: ......................A....-......................   Pabelli
 412444: ....C.A........G.....CA....T.........A...A........   Cjacchus
1059680: ........T......G.....CA....T.........A...AC.......   Sboliviensis

            LINE1                          LINE1
         ---------->           --------------------------->
              65142     65152     65162     65172     65182
  65133: ACAAAGAAGTAGACAGTTTCTAGCAAAAATAATACATGAATATGAAAACC   Hsapiens
 384870: C.................CT..............................   Ptroglodytes
 402322: ...................T..............................   Ggorilla
 439764: ...............C...T.......T....C.................   Pabelli
 412494: ...................T.......G......T............T-.   Cjacchus
1059730: ...................T..............T.............-.   Sboliviensis
```

```
                                  LINE1
         ------------------------------------------------->
              65192     65202     65212     65222     65232
                  :         :         :         :         :
   65183: AGATTGGTGGTCGTTGCTCTTCGGGGAGGGTTGACTGGAAATGGTTAGAA   Hsapiens
  384920: .......C....A.....................................   Ptroglodytes
  402372: .........................................C........   Ggorilla
  439814: ............A........T..A................C........   Pabelli
  412543: ..........CTT........G...A....A...........AA...A..   Cjacchus
 1059779: ...........TT........G...A................AA......   Sboliviensis

                                  LINE1
         ------------------------------------------------->
              65242     65252     65262     65272     65282
                  :         :         :         :         :
   65233: GCAGCTTTCTGTAGCAATAGAAATGTTTCATTTTCTGGTAGGGGAATGGG   Hsapiens
  384970: .A.........................................A......   Ptroglodytes
  402422: ..................................................   Ggorilla
  439864: .A................................................   Pabelli
  412593: .A.........G.......T.............C........A.......   Cjacchus
 1059829: .A.........G.......T.............C.......AA.......   Sboliviensis

                                  LINE1
         ------------------------------------------------->
              65292     65302     65312     65322     65332
                  :         :         :         :         :
   65283: TTACATGCATGCATGTGTTAGTGTCAAAATTGACTGAACTGATTGATTTG   Hsapiens
  385020: ................A.................................   Ptroglodytes
  402472: ................A.................................   Ggorilla
  439914: ................A............C............C.......   Pabelli
  380382:                                               ....   Panubis
  412643: ................A.----------------...............C   Cjacchus
 1059879: ................A................T................   Sboliviensis

            LINE1
         -------->
              65342     65352     65362     65372     65382
                  :         :         :         :         :
   65333: AGCATTTCAATCAATTATATGTCAGTATGGCGGGGTTTTTTTTTTCCTTT   Hsapiens
  385070: ...................................-..............   Ptroglodytes
  402522: ...................................-.........T.C..   Ggorilla
  439964: ...G....................A.....GC....A.......C.T...   Pabelli
  127368:                                      ........TT...   Nleucogenys
  142914:                                    .C........TT...   Mmulatta
  380386: T.TT.G.TG.C..CA.G......-----------T..........TT...   Panubis
  412677: ........................A.....T..----....C.....C..   Cjacchus
 1059929: ...........-............A.....T..TT..C...CCC.TT...   Sboliviensis

                                                   LINE1
                                               ---------->
              65392     65402     65412               65427
                  :         :         :                   :
   65383: TTTTTCCAGAGGATCTTATGGAGAAAGATGTTTTA-----TTTTAAATTA   Hsapiens
  385119: ...........................G.......-----..........   Ptroglodytes
  402571: ...........................G.......-----..........   Ggorilla
  440014: ..........................TG.......-----..........   Pabelli
  127381: .....TTT...A                                         Nleucogenys
  142929: .....T.T...                                          Mmulatta
  380425: .....TTT...A.GTG.C..TTC.T.--......GCTCAC....T...GG   Panubis
  412723: ..C................A.......TA......-----..........   Cjacchus
 1059978: ...................A..C....T.A.....-----..........   Sboliviensis
```

```
                             LINE1
         -------------------------------------------------->
              65437     65447     65457     65467     65477
                  :         :         :         :         :
   65428: AGCTTTTTATTTTGAGGTAAAATTGAAGATGCGCTCAGGTGCCATTGTGA  Hsapiens
  385164: ..............................A................C..  Ptroglodytes
  402616: ................................A..............C..  Ggorilla
  440059: ................................A.................  Pabelli
  380473: G.T.G...G...                                        Panubis
  412768: ...........................A....A.....A...........  Cjacchus
 1060023: ........G.......A.............A.A.....A....G......  Sboliviensis

                             LINE1
         -------------------------------------------------->
                        65495     65505     65515     65525
                            :         :         :         :
   65478: AAATTAATA--GAGATGCTGTGTACTGTTTAGTCAGTTTCTCACAATGGT  Hsapiens
  385214: .........CA.......................................  Ptroglodytes
  402666: .........CA.......................................  Ggorilla
  440109: .........CA.......................................  Pabelli
  412818: ....----.CC....-----..............................  Cjacchus
 1060073: ....----.CA.......................................  Sboliviensis

                             LINE1
         -------------------------------------------------->
              65535     65545     65555     65565     65575
                  :         :         :         :         :
   65526: AACATGTTGCAAAGCTGTAGTACATAGTCACAACCAGGATATCGACATTA  Hsapiens
  385264: ..................................................  Ptroglodytes
  402716: ..................................................  Ggorilla
  440159: .....A....................................T.......  Pabelli
  412859: .....A..C....AG.A.........................T.......  Cjacchus
 1060119: .....A..C....A..A................A........T.......  Sboliviensis

                LINE1
         ---------------->
              65585     65595     65605     65615     65625
                  :         :         :         :         :
   65576: ACAAAGTCAAGATACACAGTCAAGAAGCTCCTGGGGTAAAGCTTGCAAGT  Hsapiens
  385314: ....................................A.............  Ptroglodytes
  402766: ....................................A.............  Ggorilla
  440209: .............................A......AG............  Pabelli
  133035:                                       ..A...C..TA.  Nleucogenys
  412909: ..........T................-.......AA.....-.....A.  Cjacchus
 1060169: ....................................A........A..A.  Sboliviensis

              65635     65645     65655     65665     65675
                  :         :         :         :         :
   65626: CTTAGCTCTCTGAGCATGGGGAGGATTTGGAGGTTGGCCTTAGTCTCTTT  Hsapiens
  385364: ...............G.......................C......T...  Ptroglodytes
  402816: .......................................C......T...  Ggorilla
  440259: .........................C............AC......T...  Pabelli
  133047: ..C....T.TGA....GA..A..A.C..                        Nleucogenys
  412957: ...G........G.................G........C......T...  Cjacchus
 1060219: ....................A.........G.....A.TA......T...  Sboliviensis

              65685     65695     65705     65715     65725
                  :         :         :         :         :
   65676: AGATGTCATCCACAAAGCCTCCCTCTGCTCACACTAAAATTTACTTTAAA  Hsapiens
  385414: ....TC..............T.............................  Ptroglodytes
  402866: ..................................................  Ggorilla
  440309: ....T.............................................  Pabelli
  413007: ..G.TC.G...........C...................C........T.  Cjacchus
 1060269: ..G.T..G...............................C..........  Sboliviensis
```

```
              65735     65745     65753     65763     65773
  65726: TATTTCTTGTAGGACTGAAGTCAGC--ACCTCACAGTTAGACAATGAATG Hsapiens
 385464: .........................--....................... Ptroglodytes
 402916: .........................--....................... Ggorilla
 440359: .........................--...A................... Pabelli
 354947:                                              ..... Nleucogenys
 413057: ................TTGTCGCA.--...A..A......G......... Cjacchus
1060319: .......CA.......TT-......AT...A..A......G......... Sboliviensis

              65783     65793     65802     65812     65822
  65774: GAAGGAGGTTCTTGTGGTTCTTTAATA-GTTTTGTGCGTATAAATTACCT Hsapiens
 385512: ...........................-..C.....T............. Ptroglodytes
 402964: ...........................-........T............. Ggorilla
 440407: ...........................-........T............. Pabelli
 354952: ........-...........-.....C-........T............. Nleucogenys
 413105: A.....A...A..............G.-........T..G........T. Cjacchus
1060368: A...A.A...A..............G.G........T...........T. Sboliviensis

              65832     65842     65852     65862     65872
  65823: CTCCAATTTGGTGGTCTCCTCCATTTGTTGATCCCAATGAACCTCTGAAT Hsapiens
 385561: ............................G..................... Ptroglodytes
 403013: .................................................. Ggorilla
 440456: .................................................. Pabelli
 354999: .................................................. Nleucogenys
 413154: .......G............A...............G.-........... Cjacchus
1060418: .......G............AT................-........... Sboliviensis

              65882     65892               65905     65915
  65873: ATTTATACCTTGTGCAGTGTCCTTACCTG-------TTTTAAACATTAGA Hsapiens
 385611: ...........T.................-------.............. Ptroglodytes
 403063: .....C.......................-------.............. Ggorilla
 440506: .............................-------.............. Pabelli
 355049: .............................-------.............. Nleucogenys
 413203: ..............TC.............TGACTAT...G.......... Cjacchus
1060467: .--------.....T..............TGACTAT.............. Sboliviensis

                                        LINE1
                                ----------------------------->
              65925     65935     65945     65955     65965
  65916: ATGTGTCAAAAATAATGTTTTGTCAATATACAAAAGTCAATTGCTTTCCT Hsapiens
 385654: .................................................. Ptroglodytes
 403106: .................................................. Ggorilla
 440549: ........................G......................... Pabelli
 355092: ...................C.............................. Nleucogenys
 413253: ...................................CC..G.......... Cjacchus
1060509: .................................................. Sboliviensis

                              LINE1
         ------------------------------------------------->
              65975     65985     65995     66005     66015
  65966: GTATACTAGCAATGTACAACTGGAATTTGAAATTGAAAACATAGCACTGT Hsapiens
 385704: .................................................. Ptroglodytes
 403156: .................................................. Ggorilla
 440599: ........................................T......... Pabelli
 355142: ...................T.............................. Nleucogenys
 413303: A.G.....C...CA...........C..T.....A...G.--........ Cjacchus
1060559: A.......C...CA.G..GT.....C........A.....--........ Sboliviensis
```

```
                                LINE1
          ------------------------------------------------->
                66025     66035     66045     66053     66063
   66016: TTATATCAGCATCCCCAAAAAGAACTACTTAG--ATACAAATCTAACAAA  Hsapiens
  385754: ................................--................  Ptroglodytes
  403206: ................................--................  Ggorilla
  440649: ................................--................  Pabelli
  355192: .............T..................--................  Nleucogenys
  413351: ....G...C..A.T......TT..A.......--...G............  Cjacchus
 1060607: ........C..A.T......TA..A.......GT...T............  Sboliviensis

                                LINE1
          ------------------------------------------------->
                66073     66083     66093     66103     66113
   66064: GTATGTGTAAGACCTAGATGAAGAAAACTGCAAAATTCTGATCAAAGAAA  Hsapiens
  385802: ..................................................  Ptroglodytes
  403254: ..................................................  Ggorilla
  440697: ..................................................  Pabelli
  355240: ......A...........................................  Nleucogenys
  413399: ......AG......................T.......C...A.......  Cjacchus
 1060657: ......A.......................T...........A.......  Sboliviensis

                                LINE1
          ------------------------------------------------->
                66123     66133     66142     66152     66162
   66114: TCAAAGAAGATCTAAATAAAT-GAATGATATTTTATGTTCATGGATAAGA  Hsapiens
  385852: .....................G............................  Ptroglodytes
  403304: .....................G............................  Ggorilla
  440747: ...---...............G............................  Pabelli
  355290: .....................G............................  Nleucogenys
  413449: .....A...............G...G..C.....................  Cjacchus
 1060707: .................C...G...G....G...................  Sboliviensis

                                LINE1
          ------------------------------------------------->
                66172     66182     66192               66211
   66163: ACATTCAATCATGTTACGATGTCATTTCTTTCCAACTTG-TTTATAGATT  Hsapiens
  385902: .........................C.............-..........  Ptroglodytes
  403354: .......................................-..........  Ggorilla
  440794: ..........T........................T...-..........  Pabelli
  355340: .................A.....................-..........  Nleucogenys
  413499: .A....TG........AA...........G...T.....G..........  Cjacchus
 1060757: ......TG........AA...............T.....T..........  Sboliviensis

                                LINE1
          ------------------------------------------------->
                66221     66231     66241     66251     66261
   66212: CAACATATTCCCAATCAGGGTCCCAGCAAATTGCTTTGTGGATATCTGAG  Hsapiens
  385951: .................................T................  Ptroglodytes
  403403: ...................A..............................  Ggorilla
  440843: ...........T......................................  Pabelli
  355389: ..................................................  Nleucogenys
  413549: ..G............G.AA...T......G..A....C...G.......A  Cjacchus
 1060807: ..G..............AA..GT......G..AT.......G.......A  Sboliviensis
```

```
                          LINE1
         ------------------------------------------------->
                66271     66281     66291     66301     66311
                    :         :         :         :         :
    66262: AGCTAATTCTAAAATTTATATAGAAAGGCAAAAAACTCGGACTAACCAAC   Hsapiens
   386001: ..................................................   Ptroglodytes
   403453: ..................................................   Ggorilla
   440893: ..................................................   Pabelli
   355439: ..................C...................A...........   Nleucogenys
   413599: ..............C.......................A...........   Cjacchus
  1060857: ..................................................   Sboliviensis

                          LINE1
         ------------------------------------------------->
                66321     66331     66341     66351     66361
                    :         :         :         :         :
    66312: ATAATATTATAGAAAAAAGGAAAGTATTTTTTGGTAGAGGGCAGATACTA   Hsapiens
   386051: ..................................................   Ptroglodytes
   403503: ..................................................   Ggorilla
   440943: ............................................T.....   Pabelli
   355489: ..........................G.......................   Nleucogenys
   413649: ..G.C....A...G............A.........A...A........G   Cjacchus
  1060907: ..G.CT....-..GGG...TC...G...                         Sboliviensis

                          LINE1
         ------------------------------------------------->
                66371     66381     66391     66401     66411
                    :         :         :         :         :
    66362: CCTGACTTCAAAACATACTATAAAGCTATATGAATCAAGACATGTGTGTT   Hsapiens
   386101: ...............................T..................   Ptroglodytes
   403553: ...............................T..................   Ggorilla
   440993: ...............................T..................   Pabelli
   355539: ...................G..T........T.......--.........   Nleucogenys
   413699: .................T.......T....CT..........-A......   Cjacchus

                          LINE1
         ------------------------------------------------->
                66421     66431     66441     66449     66459
                    :         :         :         :         :
    66412: ATTGGTGAAGGAATGGACAAATGGATCAATAGAATAG--AAAAAAAATCC   Hsapiens
   386151: .....................................---..........   Ptroglodytes
   403603: .....................................--...........   Ggorilla
   441043: .....................................-A...........   Pabelli
   355587: ....................G.............G.A-A...........   Nleucogenys
   413748: ....A...............GCA..............GA.G......AAA   Cjacchus

               LINE1                   LTR
         ----------->------------------------------------->
                66469     66479     66489     66499     66509
                    :         :         :         :         :
    66460: CAGAAATAGACCTGATATGGTTTGGCTCTGTGTCCCCACCCAAATCTCAT   Hsapiens
   386198: ..........T.......................................   Ptroglodytes
   403651: .............................T....................   Ggorilla
   441092: ..................................................   Pabelli
   355636: .T................................................   Nleucogenys
   413798: ....T.......CAG.........A...--.......G............   Cjacchus
```

```
                          LTR
        ---------------------------------------------------->
                  66519                                66536
  66510: GTTGAACTGTAATCCCCC-----------------------GGAGGTGAT   Hsapiens
 386248: ..................ATTGTTAGAGGTGAAGTCTAGAG.........   Ptroglodytes
 403701: ..................ATTGTTAGAAGTGAAGTCTAGAT.........   Ggorilla
 441142: ..................ATTGTTAGAGGTGAAGTCTAGAG.........   Pabelli
 355686: ..C...............ATTGTTAGAGGTGAAGTCTAGAG.........   Nleucogenys
 413846: ......T.........T.ATTGTTAGAGGTGAAGCCTGGAG........C   Cjacchus

                          LTR
        ---------------------------------------------------->
                  66546     66556     66565     66575     66585
  66537: TGGATCGTGGGGGTGTTTTCTAATGG-TTAGCACTATTCCCCTAGTGCTA   Hsapiens
 386298: ..........................C.......C.....T.........   Ptroglodytes
 403751: ......T..C................-.......................   Ggorilla
 441192: ..........................C.......C...............   Pabelli
 355736: ............ACA.....                                 Nleucogenys
 413896: ............A..........CA.C..T.T..CT.....T........   Cjacchus

                LTR                        Simple
        -------------------------->------------------------->
                  66595     66605     66615     66625     66635
  66586: TCTCATGATAGAGTTCTCATGAAATCTGTTTTTTTGTTTGTTTGTTTGTT   Hsapiens
 386348: ................................----------------..   Ptroglodytes
 403800: ................................-----------.......   Ggorilla
 441242: ..........A.....................------------C.....   Pabelli
 413946: ..................T.............------------------   Cjacchus

             Simple                      LTR
        ----------------->----------------------------------->
                  66645     66655     66665     66675     66685
  66636: TGTTTGTTTGTTTGTTTTTAGTACGTATCACTTCTGTCTTGGCTCTGTCT   Hsapiens
 386382: .......................T..........................   Ptroglodytes
 403839: .......................T..........................   Ggorilla
 441280: .......................T..........................   Pabelli
 413978: ---------------C..A...GT...........C....T.....C...   Cjacchus

                          LTR
        ---------------------------------------------------->
                  66695     66705     66715     66725     66735
  66686: CTCCTGCTCTGCCATGATAAGACATGCTTACTTCACCTTCACCTTCCACC   Hsapiens
 386432: ..................................................   Ptroglodytes
 403889: ......T...........................................   Ggorilla
 441330: ..............C.......T...........................   Pabelli
 414013: -...C..........A........CA........C.......T..T....   Cjacchus

                          LTR
        ---------------------------------------------------->
                  66745     66755     66765     66775     66785
  66736: ATGATCGGAAGTTTCCTGAGGCCTCCCAGCCATGTTTCCTGTACAGTCCA   Hsapiens
 386482: ..C..T............................................   Ptroglodytes
 403939: .....T............................................   Ggorilla
 441380: ......A........A...C..............................   Pabelli
 414062: ......A.........................C...........T.....   Cjacchus
```

```
                         LTR
             66795     66805     66815     66825     66835
                 :         :         :         :         :
 66786: CAGGATTGTGAGCCACTTAAGCCTTTTTTTCTTCATAAATTACCTAGTCT  Hsapiens
386532: .....................G............................  Ptroglodytes
403989: ............................................C.....  Ggorilla
441430: .....................T......................C.....  Pabelli
414112: .....CCAC-..T..A....A......G..TG.T..........C.....  Cjacchus

                    LTR                       LINE1
             66845     66855     66865     66875     66885
                 :         :         :         :         :
 66836: CAGGTAGTTCTTTACAGCAGTGTGAGAACAGACTAATACGAGACCTAAAC  Hsapiens
386582: ..................................................  Ptroglodytes
404039: ..................................................  Ggorilla
441480: ..................CA...................A..........  Pabelli
414161: ....G....................A..TG..T.............T..A  Cjacchus

                        LINE1
             66895     66905               66919     66929
                 :         :                   :         :
 66886: AACTATAGTCAGCTGATCTTTGACAAAG------AGCAAACTCACTGGAG  Hsapiens
386632: ............................------................  Ptroglodytes
404089: ............................------................  Ggorilla
441530: ..G.........................------..A........C....  Pabelli
414211: .TGC........................GAGAAA..GC..T......A..  Cjacchus

                        LINE1
             66939     66949     66959     66969     66979
                 :         :         :         :         :
 66930: AAAGGACAGTCTTTTCAACAAGTAATTCTGGAACAACTGGACAACCACAT  Hsapiens
386676: ......T...........................................  Ptroglodytes
404133: ......T.................G..---------..............  Ggorilla
441574: ......T...........................................  Pabelli
414261: ......T.C..........CTA.G..................G.......  Cjacchus

                        LINE1
             66982     66992     67002     67012     67022
                 :         :         :         :         :
 66980: AC-------CAAAAAAAAAATCAATCTAGGCACTAACCTTATACTTTTTA  Hsapiens
386726: ..--------........................................  Ptroglodytes
404174: .T-------T...........................T......C.....  Ggorilla
441624: ..GAAAAAAA..................................C.....  Pabelli
414311: ..----------.........T..............T..--...C.....  Cjacchus

                        LINE1
             67032     67042     67052     67062     67072
                 :         :         :         :         :
 67023: CAAAAATTAACTCAAAAATGACCATGGACTTGAATGTAAAATCCAAAGCT  Hsapiens
386768: ................................................G.  Ptroglodytes
404217: ..................................................  Ggorilla
441674: ..................................A..G............  Pabelli
414349: -...........G............A.........A......G......A  Cjacchus
```

```
                               LINE1
        ------------------------------------------------->
             67082     67092     67101     67111     67121
 67073: ATAAAATTTCTAGAAAATGATCTGT-GAAAAAATCTAGGTGACTTTAGGT  Hsapiens
386818: .........................-........................  Ptroglodytes
404267: .........................-........................  Ggorilla
441724: .........................-........................  Pabelli
414398: ......C..T.....G....C..A.AA........C....A.........  Cjacchus

                               LINE1
        ------------------------------------------------->
             67131     67141     67151     67161     67171
 67122: TTCTTGATGAGTTTTTAGATACAATACCCAAAGAATGATCCAAGAAAGAA  Hsapiens
386867: ..................................................  Ptroglodytes
404316: ..................................................  Ggorilla
441773: ........................C.........................  Pabelli
414448: ....................................A.C...........  Cjacchus

                               LINE1
        ------------------------------------------------->
             67181     67191     67201     67211     67221
 67172: AAAGTTGATGTTAGACTTAATTAAAATTAGAAACTTCTGTGATACAAAAG  Hsapiens
386917: ..................................................  Ptroglodytes
404366: ...............-..................................  Ggorilla
441823: ..................................................  Pabelli
414498: ..........C..............................C.GT.....  Cjacchus

                               LINE1
        ------------------------------------------------->
             67231     67241     67251     67261     67271
 67222: ACACTGTTAAGAGAATGAAAAGACAATCCCACAGACTAGGAGAAAATATT  Hsapiens
386967: ...................................G..............  Ptroglodytes
404415: ..G...............................................  Ggorilla
441873: ..................................................  Pabelli
414548: ........G.A................T......................  Cjacchus

                               LINE1
        ------------------------------------------------->
             67281     67291     67301     67310     67320
 67272: TGCACAATACACATCACATAGAGGATTTGTATCC-AAAACAAGCAAGAAA  Hsapiens
387017: ..................................-...............  Ptroglodytes
404465: ..................................-...............  Ggorilla
441923: ..................................-...............  Pabelli
414598: ..T...............................A.......A.......  Cjacchus

                               LINE1
        ------------------------------------------------->
             67328     67338     67348     67357     67367
 67321: CTC--AAAATTCAACAATAAGAAAACAAACAACCCAAT-AAAAAAAAGTT  Hsapiens
387066: ...--........................T........-...........  Ptroglodytes
404514: ...--.................................A...........  Ggorilla
441972: ...--.................................---.........  Pabelli
414648: ...TT....C.......................T....A....C.....A  Cjacchus
```

```
                              LINE1
        ------------------------------------------------->
             67377     67387     67397     67407     67417
                 :         :         :         :         :
 67368: AGCAAAACATCTGAACAGACACCTCACCAAAAGAGACATATGAATGGAAA  Hsapiens
387113: ..................G....................C..........  Ptroglodytes
404562: ..................................................  Ggorilla
442017: ...................T.......A......................  Pabelli
414698: ...................TG.....TG....A.T....G..........  Cjacchus

                              LINE1
        ------------------------------------------------->
             67427     67437     67447     67457     67467
                 :         :         :         :         :
 67418: ATAAGCATATGAAAAGGTGTTAAACATTATATATCCTTAGGGAGTTTCAA  Hsapiens
387163: .........C........................................  Ptroglodytes
404612: ..............G.                                    Ggorilla
442067: ................A.................................  Pabelli
414748: ................AC...C.G...............A...A..C...  Cjacchus

                              LINE1
        ------------------------------------------------->
             67477     67487     67497     67507     67516
                 :         :         :         :         :
 67468: ATTAAGACAACACTGAGATACCATTATGTCTATTTGAATGGCTAAA-ATC  Hsapiens
387213: ..............................................-...  Ptroglodytes
442117: ......G.......................................-...  Pabelli
414798: ..C..................T........................C...  Cjacchus

                              LINE1
        ------------------------------------------------->
             67526     67536     67546     67556     67566
                 :         :         :         :         :
 67517: CAAAGCTCTGACAACACCAAATGCTGCTGAAGATACAGAGCAACATGAAC  Hsapiens
387262: ..................................................  Ptroglodytes
442166: .................A............G...................  Pabelli
414848: .........AT...................G....TG....G...G....  Cjacchus

                              LINE1
        ------------------------------------------------->
             67576     67586     67596     67606     67616
                 :         :         :         :         :
 67567: TCTCACTTATTATTGGGGTGTTATTGGGAGGGATGCAAAATAATACTGCC  Hsapiens
387312: ..................................................  Ptroglodytes
442216: ........G.........................................  Pabelli
414898: ........G...C......A...............T..............  Cjacchus

                   LINE1
        ---------------------------->
             67626     67636     67646     67655
                 :         :         :         :
 67617: ACTTTGAAAGACAGTTTGGCAATCTCTTGTTT-TATTGTTTTCTTG----  Hsapiens
387362: ............................T...-.............----  Ptroglodytes
442266: .........C..................T..--.............----  Pabelli
380411:                       .T.T..T...-.T..T....T...AGAA  Panubis
414948: ............................T...G.............----  Cjacchus
```

```
                       67669     67679     67689     67699
                          :         :         :         :
 67662: ------------ATGTTTTATTGGTTTTTTAATGGATTTTTATGTTTATT  Hsapiens
387407: ------------..........-...........................  Ptroglodytes
405770:             .......GCCCAC..........GG..G.T.....---  Ggorilla
442310: ------------......................................  Pabelli
380438: GTGTCTGTTCAT.......GC.CAC..........GG..G.T........  Panubis
414994: ------------.GA..............A--------------------  Cjacchus
 82636:                ....T..TT......-------....T.T...---  Sboliviensis


                                                LINE1
                                         ----------------->
             67709     67719     67729     67739
                 :         :         :         :
 67700: GGGTTTTTATGTTTTATTACATTTTTATGTAATTTTTTTTTATTTTTT--  Hsapiens
387444: ................................-......A........--  Ptroglodytes
405805: -----------....C...T.AA...GCT...G..CC.CA..GA..C.--  Ggorilla
442348: ....................C...........-......A........--  Pabelli
415012: --------..A......C.G..........TG-......A.TGA....--  Cjacchus
 82661: ---.....T.T....T...A......T.T.TT.........T......TT  Sboliviensis


                              LINE1
        ------------------------------------------------->
             67753     67763     67773     67783     67793
                 :         :         :         :         :
 67748: ----ATTGGCTTTTAGTTCTGAGTTATATGTGCAGGAAGTGCAGGTTTGT  Hsapiens
387491: -----......................................T......  Ptroglodytes
405842: -------..A.A....AC..TT..C.G...GA....                Ggorilla
442395: ----..............................A...............  Pabelli
415051: -TTA....AT.............G.G...-....................  Cjacchus
 82708: TTGAG..A.AA.C.T.C....                               Sboliviensis


                              LINE1
        ------------------------------------------------->
             67803     67813     67823     67833     67843
                 :         :         :         :         :
 67794: TACATAGGTAAACGTATGCCATGTGGTTTGCTAAACCTATCAACCTATCA  Hsapiens
387536: ..................................................  Ptroglodytes
442441: ....C........A....................................  Pabelli
415099: .......T.....AC...........C.....GC................  Cjacchus


                              LINE1
        ------------------------------------------------->
             67853     67863     67873     67883     67893
                 :         :         :         :         :
 67844: GCTAGGTATTAAGCCCAGCATGTATCAGCTTTTTTCCCTAATGTTCTCCC  Hsapiens
387586: ..................................................  Ptroglodytes
442491: ...........................................C......  Pabelli
415149: C.............T.......C......-..........G..C......  Cjacchus


                              LINE1
        ------------------------------------------------->
             67903     67913     67923     67933     67943
                 :         :         :         :         :
 67894: ACTCCTGCCCTCCCCCAACAGGCTCCAGTAAGTGTTGTTCCCCTCCTTGT  Hsapiens
387636: ..................................................  Ptroglodytes
442541: ..............................................C...  Pabelli
415198: ....G.........-------A....---------..CC.T..C..AACA  Cjacchus
```

```
                              LINE1
        ------------------------------------------------->
             67953     67963     67973     67983     67993
                 :         :         :         :         :
 67944: GTCCATGTGTTCTCACTGTTCAGCTCCCACTTATAAGTGAAAACATGAGG  Hsapiens
387686: ..................................................  Ptroglodytes
442591: ...................................G..............  Pabelli
415232: .G.............T....................A..........CA.  Cjacchus

                              LINE1
        ------------------------------------------------->
             68003     68013     68023     68033     68043
                 :         :         :         :         :
 67994: TCTTTGGTTTTCTGTTCCTGTGTTAATTTGCTGAGGATGACAGCTTCCAA  Hsapiens
387736: ......................................A..........G  Ptroglodytes
442641: ..............C.....C.................A..........G  Pabelli
415282: .A..............T...CA...G............A.T........G  Cjacchus

                              LINE1
        ------------------------------------------------->
             68053     68063     68073     68083     68093
                 :         :         :         :         :
 68044: CTTCAACCATGTCCCCGAAAAGGAAATGATCTCATTCCTTTTTATGGTTG  Hsapiens
387786: ..................................................  Ptroglodytes
442691: ...............T..................................  Pabelli
415332: T....T.........TTC......C.C...T.T.....C...........  Cjacchus

                              LINE1
        ------------------------------------------------->
             68103     68113     68123     68133     68143
                 :         :         :         :         :
 68094: CATAGTATTCCATGGTGTATATGCACCACATTTTCTTTATCCAGCCTATC  Hsapiens
387836: ..................................................  Ptroglodytes
442741: ..........TG....A...........................T.....  Pabelli
415382: .......................T....................T.....  Cjacchus

                              LINE1
        ------------------------------------------------->
             68153     68163     68173     68183     68193
                 :         :         :         :         :
 68144: ATTGATGGGTATTTGGGTTGATTCCATGTCTTTGCTATTGTGAATAGTGC  Hsapiens
387886: ..................................................  Ptroglodytes
442791: ..............................................T...  Pabelli
415432: .C.....A.CG...........C..T.C..G...................  Cjacchus

                              LINE1
        ------------------------------------------------->
             68203     68213     68223     68233     68243
                 :         :         :         :         :
 68194: TGCAGTGAACATATGTGTGCATGAATCTTCATAATAGAATGATTTATATT  Hsapiens
387936: ....A.............................................  Ptroglodytes
442841: ....A.............................................  Pabelli
415482: ....A........C....T.G..T........G..............G..  Cjacchus

                              LINE1
        ------------------------------------------------->
             68253     68263     68269     68279     68289
                 :         :         :         :         :
 68244: CCTTTGGGTATATATCCAGT----AATGGGATTGCTGGGTCAAATGGTAT  Hsapiens
387986: ....................----..........................  Ptroglodytes
442891: ....................----..........................  Pabelli
415532: ..............CT....AATG..............T....G......  Cjacchus
```

```
                               LINE1
        ------------------------------------------------->
             68299     68309     68319     68329     68339
 68290: TTATGGTTCTAAATCTTTGAGGAATCACCACCCTGTCTTCCACAATGGTT  Hsapiens
388032: ...........................................G......  Ptroglodytes
442937: ..................................................  Pabelli
415582: ..C.........C.G...A.........TG.A..A...............  Cjacchus

                               LINE1
        ------------------------------------------------->
             68349     68359     68369     68379     68389
 68340: GAACTAATTTACATTACCACCAACAGTGTAAAAGCGTTCCTATTTCTCCA  Hsapiens
388082: ..................................................  Ptroglodytes
442987: .................A.................A..............  Pabelli
415632: .............C.CT.....G.                            Cjacchus

                               LINE1
        ------------------------------------------------->
             68399     68409     68419     68429     68439
 68390: CAACCTTGCCAGCATCTGTTGTTTTCTGACTTTTTAATAATTGCTATTCT  Hsapiens
388132: ..................................................  Ptroglodytes
443037: ..................................................  Pabelli

                               LINE1
        ------------------------------------------------->
             68449     68459     68469     68479     68489
 68440: GACTGACGTGAGATGGTTTCTTATTGTGGTTTTTGATTTGCATTTCTCTA  Hsapiens
388182: .............................-....................  Ptroglodytes
405670:                                ...................  Ggorilla
443087: ...C.......................A.-....................  Pabelli

                               LINE1
        ------------------------------------------------->
             68499     68509     68519     68529     68539
 68490: ATGATCAGTGATGTTGAGCTATTTTTGTGTTTGTTGACCACATGTATGTC  Hsapiens
388231: ..................................................  Ptroglodytes
405689: ..................................................  Ggorilla
443136: .............A..................T.................  Pabelli

                               LINE1
        ------------------------------------------------->
             68549     68559     68569     68579     68589
 68540: TTTTTTTTTTTTTTTTTTTGAGAAGTGTCTGTTCATATGTTTTGCCCACT  Hsapiens
388281: ----.................T............................  Ptroglodytes
405739: -----....C........................................  Ggorilla
443186: ----..............................................  Pabelli

                               LINE1
        ------------------------------------------------->
             68599     68608     68618     68628     68638
 68590: TTTTAATGGGGTTGTTTG-TTTTTTTCTTATAAATTTGCTTAAGTTCCTC  Hsapiens
388327: ..................-...................A...........  Ptroglodytes
405784: ..................-...............................  Ggorilla
443232: ..................T.....................G.........  Pabelli
```

```
                              LINE1
             68648     68658     68668     68678     68688
 68639: ATAGATTCTGGATATTAGACCTTTGTCAGATGGACAGGTTGCAAAAATCT Hsapiens
388376: .................................................. Ptroglodytes
405833: .................................................. Ggorilla
443282: .................................................. Pabelli
```

```
                              LINE1
             68698     68708     68718     68728     68738
 68689: TCTCCCCTCTCGTAGGTTGTCTGTTCACTCTGCTGATAGTTTTGTTTGTT Hsapiens
388426: .................................................. Ptroglodytes
405883: .................................................. Ggorilla
443332: ............................C..................... Pabelli
```

```
                              LINE1
             68748     68758     68768     68778     68788
 68739: GTGCAGAAGCTCTTTAGTTTGATTTGATCCCATTCGTCTTGTTTTGCTTT Hsapiens
388476: .................................................. Ptroglodytes
405933: ...................................A.............. Ggorilla
443382: ..................................T...A........... Pabelli
```

```
                              LINE1
             68798     68808     68818     68828     68838
 68789: TGTTGCTATTGCTTTTGGTGATTTCATCATAAAGTCTTCACCCATGCCTA Hsapiens
388526: .....A............................................ Ptroglodytes
405983: .................................................. Ggorilla
443432: .................................................. Pabelli
```

```
                              LINE1
             68848     68858     68868     68878     68888
 68839: TGTCCTGAATAGTATTGCCTAGATTTTCTTCCAGGATTTTATAGTTTTGG Hsapiens
388576: ...................................G.............. Ptroglodytes
406033: .............G.....................G.............. Ggorilla
443482: ...............CA..................G.............. Pabelli
```

```
                              LINE1
             68898     68908     68918     68928     68938
 68889: GTTTTACATTTAAGTCTTTAATCTATCTTGAGTGAATTTTTGTATAAGGT Hsapiens
388626: ................C................T.............A.. Ptroglodytes
406083: .................................T................ Ggorilla
443532: A................................T................ Pabelli
```

```
                              LINE1
             68948     68958     68968     68978     68988
 68939: GTAAGAAAGGGATCCAGTTTCAATTTTCTGCATATAGCTAACCAGTTCTC Hsapiens
388676: A.........T.................CA.................... Ptroglodytes
406133: ..........................................T....... Ggorilla
443582: ............A..................................... Pabelli
```

```
                           LINE1
        -------------------------------------------------->
                 68998     69008     69018     69028     69038
                     :         :         :         :         :
 68989: CCAGTACCATTTATTAAATAGGGAATCTTTTCCACACTGCTTGTTTTTTT  Hsapiens
388726: ................................................G.  Ptroglodytes
406183: .................................C..............G.  Ggorilla
443632: ........................C.......................G.  Pabelli

                           LINE1
        -------------------------------------------------->
                 69048     69058     69068     69078     69088
                     :         :         :         :         :
 69039: CGGGTTTATTGAAGATCAGATGTTTGCAGATGTGCAGTTTTATTTCTGAG  Hsapiens
388776: ..................................................  Ptroglodytes
406233: .....................A.......................A....  Ggorilla
443682: .A....G...........................................  Pabelli

                           LINE1
        -------------------------------------------------->
                 69098     69108     69118     69128     69138
                     :         :         :         :         :
 69089: TTCTCGATTTTGTTCCATTGGTCTATGTGGCTGTTTTTGTACCAGTACCA  Hsapiens
388826: .....T............................................  Ptroglodytes
406283: .....T............................................  Ggorilla
443732: .....T............................................  Pabelli

                           LINE1
        -------------------------------------------------->
                 69148     69158     69168     69178     69188
                     :         :         :         :         :
 69139: TGCTGTTTGGGTTACTGTGGCCTTGTAGTACAGTTTGAAGTCAGGTACTA  Hsapiens
388876: ................................................C.  Ptroglodytes
406333: ................................................C.  Ggorilla
443782: ................................................C.  Pabelli

                           LINE1
        -------------------------------------------------->
                 69198     69208     69218     69228     69238
                     :         :         :         :         :
 69189: TGAGCTCCAGCTTTGTTCATTCTCTTAGGATTGTCTTGGCTATGCAAGGT  Hsapiens
388926: ..................................................  Ptroglodytes
406383: ..................................................  Ggorilla
443832: .........................C........................  Pabelli

                           LINE1
        -------------------------------------------------->
                 69248     69258     69268     69278     69288
                     :         :         :         :         :
 69239: CTTTTTTGGTTTCATATAAACTTTAAAATAGTTTTTTCTAATTCTGTGAA  Hsapiens
388976: ....................-.............................  Ptroglodytes
406433: ..................................................  Ggorilla
443882: ..................................................  Pabelli

                           LINE1
        -------------------------------------------------->
                 69298     69308     69318     69328     69338
                     :         :         :         :         :
 69289: GAATGTCAGTGGTAGTTTGATGGGAATAGCATTGAATCTATAAATTGCTT  Hsapiens
389025: ........A.........................................  Ptroglodytes
406483: ....A...A.........................................  Ggorilla
443932: ........A.........................................  Pabelli
```

```
                          LINE1
             69348     69358     69368     69378     69388
 69339: TGGGCAGTATGACTATTTTCACAATATTGATTCTTCCTATCCATGACCAT  Hsapiens
389075: ..................................................  Ptroglodytes
406533: ..................................................  Ggorilla
443982: ..................................................  Pabelli

                          LINE1
             69398     69408     69418     69428     69438
 69389: GGAATGTTTTTTAATCTCCTTGTGTCCTCTCTGACTTCCTTGAGCAGTGG  Hsapiens
389125: ..................................................  Ptroglodytes
406583: ..................................................  Ggorilla
444032: ..................................T...............  Pabelli

                          LINE1
             69448     69458     69468     69478     69488
 69439: TTTATATTTCTCCTTAAAGAGGTCCTTCACTTCCCTTGTTATCTGTATTC  Hsapiens
389175: ..................................................  Ptroglodytes
406633: ..................................................  Ggorilla
444082: ..................................................  Pabelli

                          LINE1
             69498     69508     69518     69528     69538
 69489: CTAGGTATTTTATTCTCTTTGTGGCAATTGTGAATGGGAGTTCATTCATG  Hsapiens
389225: ..................................................  Ptroglodytes
406683: ..................................................  Ggorilla
444132: ..................................................  Pabelli

                          LINE1
             69548     69558     69568     69578     69588
 69539: ATTTCGGTTTCTGCTTGTCTGTTGTTGGTGTATAGGAATGCTTGTGATTT  Hsapiens
389275: ..................................................  Ptroglodytes
406733: ..................................................  Ggorilla
444182: ..................................................  Pabelli

                          LINE1
             69598     69608     69618     69628     69638
 69589: CTGCACATTGATTTTGTATCCTGAGACTTTGCTAAAGTTGCTTATCAGCT  Hsapiens
389325: .........................C........................  Ptroglodytes
406783: .............................................G....  Ggorilla
444232: ..................................................  Pabelli

                          LINE1
             69648     69658     69668     69678     69688
 69639: TAAGAAGCTTTTGGGCTGAGATAATGGGGTTTTCTAGATATAGGATCATG  Hsapiens
389375: ...............G......G...........................  Ptroglodytes
406833: ..............A.......G...........................  Ggorilla
444282: ......................G...........................  Pabelli
```

```
                          LINE1
        ---------------------------------------------------->
              69698     69708     69718     69728     69738
 69689: TCATCTGCAAACAAAGACAATTTGACTTCCTCTCTTCCTATTTGAATAAC  Hsapiens
389425: ..................................................  Ptroglodytes
406883: ..................................................  Ggorilla
444332: ...............C...........................AC.....  Pabelli

                          LINE1
        ---------------------------------------------------->
              69748     69758     69768     69778     69788
 69739: CTTTATTTATTTCTCTTTCCTGATTGCCTTGGCCAGAACTTCTGACAATA  Hsapiens
389475: ........C..........................A..............  Ptroglodytes
406933: ........C.........................................  Ggorilla
444382: ........C.........................................  Pabelli

                          LINE1
        ---------------------------------------------------->
              69798     69808     69818     69828     69838
 69789: CGTTGACTAAAAGTGGTAAGAAGGCATCCTTGTCTTGTGCCAGTTTTCAA  Hsapiens
389525: T.................................................  Ptroglodytes
406983: ..................................................  Ggorilla
444432: .A...............G...G..............A.............  Pabelli

                          LINE1
        ---------------------------------------------------->
              69848     69858     69868     69878     69888
 69839: GGGGCTTGCCTCCAGCTTTTGCCCATTCAGTATGATATTGGCTCTGGGTT  Hsapiens
389575: ..................................................  Ptroglodytes
407033: .......................TG.........................  Ggorilla
444482: ...CTA...............T.....................G......  Pabelli

                          LINE1
        ---------------------------------------------------->
              69898     69908     69918     69928     69938
 69889: TGTCATAAAAGGCTCTTATTATTTTGAGGTATGTTCCTTCAATAGCTAGT  Hsapiens
389625: ..................................................  Ptroglodytes
407083: ..................................................  Ggorilla
444532: ..................................................  Pabelli

                          LINE1
        ---------------------------------------------------->
              69948     69958     69968     69978     69988
 69939: ATATTGAGAGTTTTTAACATGAAGGGATGTTGAATTTTATCAAAGGCTTT  Hsapiens
389675: ..................................................  Ptroglodytes
407133: .......................-..........................  Ggorilla
444582: T......................A.......................C..  Pabelli

                          LINE1
        ---------------------------------------------------->
              69998     70008     70018     70028     70038
 69989: TTCTGCATCTATTGATAATCATTTGATTTTTGTCTTTAGGTCTGCTTACG  Hsapiens
389725: ......................G.........................T.  Ptroglodytes
407182: ......................G..........................A  Ggorilla
444632: ......T.......G...C..CG.........C................A  Pabelli
```

```
                  LINE1
        ------------------------------------------------->
             70048     70058     70068     70078     70088
 70039: TGATGAATTACATTTGCATATGTTGAACAAGCCTTGCATCCCGGGGATGA Hsapiens
389775: ............................C..................... Ptroglodytes
407232: ............................C.............A....... Ggorilla
444682: ............................C.............A....... Pabelli
```

```
                  LINE1
        ------------------------------------------------->
             70098     70108     70118     70128     70138
 70089: AGCAAACTTGATCACGGTGGATAAGCTTTTTGATGTGCCGCTGGATTCAG Hsapiens
389825: ......................................T........... Ptroglodytes
407282: ...............A......................T........... Ggorilla
444732: ..............T......G............-...T........TG. Pabelli
```

```
                  LINE1
        ------------------------------------------------->
             70148     70158     70168     70178     70188
 70139: TTTGCCAGTATTTTATTGAGGATTTTTGCATTGATGTTCATCAGAGATAT Hsapiens
389875: ..................................C............... Ptroglodytes
407332: .................................................. Ggorilla
444781: ................................A................. Pabelli
```

```
                  LINE1
        ------------------------------------------------->
             70198     70208     70218     70228     70238
 70189: TGGCCTGAAGTTTTCTTTTTTATTGTTGTATCTCTGCCAGGTTTTGGTGT Hsapiens
389925: .....................G..........................A. Ptroglodytes
407382: ...................---..........................A. Ggorilla
444831: ...........................................C....A. Pabelli
```

```
                  LINE1
        ------------------------------------------------->
             70248     70258     70268     70278     70288
 70239: CAGGATGACGCTGGCCTCATAAAATGAGTTAGAGAGGAGTCCCTCCTTTT Hsapiens
389975: ........T......................................... Ptroglodytes
407429: .........A........................................ Ggorilla
444881: ..A.....T......................................... Pabelli
```

```
                  LINE1
        ------------------------------------------------->
             70298     70308     70318     70328     70338
 70289: CAATTGTTTGGAATAGTTTCAGAAGAGATGGTACCAGCTCCTCTTTGTAC Hsapiens
390025: ....................G.....A....................... Ptroglodytes
407479: ..........................A............-.......... Ggorilla
444931: ..........................A....................... Pabelli
```

```
                  LINE1
        ------------------------------------------------->
             70348     70358     70368     70378     70388
 70339: CTCTGGTAGAACACAGTGATAAATCCATCTGGACCTGAGCTTTTTTTGGT Hsapiens
390075: .................................................. Ptroglodytes
407528: .............G.................................... Ggorilla
444981: ...........................C..A................C.. Pabelli
```

```
                                    LINE1
         ---------------------------------------------------->
                     70398     70408     70418     70428     70438
  70389: TGGTAGGCTTCTTGACTTCTGCCTCAATTTCAGAACTTGTTACTGGTCTA  Hsapiens
 390125: ...............T..................................  Ptroglodytes
 407578: ...............T..................................  Ggorilla
 445031: .....A.........T..................................  Pabelli

                                    LINE1
         ---------------------------------------------------->
                     70448     70458     70468     70478     70488
  70439: TTCAGGGATTCAACTTCTTCCTGGTTCAGTCTTGGGAGGGTGTATTTGTC  Hsapiens
 390175: ..................................................  Ptroglodytes
 407628: ....................T.........................C...  Ggorilla
 445081: ...........................................G......  Pabelli

                                    LINE1
         ---------------------------------------------------->
                     70498     70508     70518     70528     70538
  70489: CAGGAATTTTTCCATTTCTTCTAGATTTTTCAGTTTATTTGCATAGATGT  Hsapiens
 390225: .................................C...............-  Ptroglodytes
 407678: ..................................................  Ggorilla
 445131: .............T....................................  Pabelli

                                    LINE1
         ---------------------------------------------------->
                     70548     70558     70568     70578     70588
  70539: GTTTATAGTATTCTCTGATGGTTTGTATTTCTGTGGGGTTAGTGGCAGTA  Hsapiens
 390274: -.................................................  Ptroglodytes
 407728: ..................................................  Ggorilla
 445181: A..........................C..........C......TG.C.  Pabelli

                                    LINE1
         ---------------------------------------------------->
                     70598     70608     70618     70628     70638
  70589: TCCCCCTTCTGGTTTCTGACTGTGTTTATTTGAATCCTCTCTCTTTTCTT  Hsapiens
 390323: ........A.........................................  Ptroglodytes
 407778: ...A....A........A................................  Ggorilla
 445231: ..T.....A..................-......................  Pabelli

                                    LINE1
         ---------------------------------------------------->
                     70648     70658     70668     70677     70687
  70639: CTTTATTAGTCTTGCTAAAGGTCTATTTTATTGA-TTTTTCAAAAAACCA  Hsapiens
 390373: ...............C..................-...............  Ptroglodytes
 407828: ..................................-...............  Ggorilla
 445280: ............CA....T.............A.T.............A.  Pabelli

                                    LINE1
         ---------------------------------------------------->
                     70697     70706     70716     70726     70736
  70688: GCTTCTGGATTCATTGA-TTTTTTGAAGGGTTACTCGTGTCTCTATCTCC  Hsapiens
 390422: .................-...........A....................  Ptroglodytes
 407877: .................-................................  Ggorilla
 445330: ...C.............T................................  Pabelli
```

```
                                 LINE1
        ------------------------------------------------->
               70746     70756     70766     70776     70786
 70737: TTCAGTTCCATTCTGACCTTGATTATTTCCTGTCTTCTGCTAGCTCTGGG  Hsapiens
390471: .....................G............................  Ptroglodytes
407926: .......T.............G.......G....................  Ggorilla
445380: .....................G............................  Pabelli

                                 LINE1
        ------------------------------------------------->
               70796     70806     70816     70826     70836
 70787: GTTTGTTTACTCTTGGTTCTCCAGTTCTTTTACTTGTGATGTTAATGTGT  Hsapiens
390521: .....................T............................  Ptroglodytes
407976: .....................T............................  Ggorilla
445430: ........G.........G..T.......................G.A..  Pabelli

                                 LINE1
        ------------------------------------------------->
               70846     70856     70866     70875     70885
 70837: CGATTTGAGATCTTTCTAGCTTTTTGATGTGGGTA-TTTAGTGCTATAAA  Hsapiens
390571: ...................................-..............  Ptroglodytes
408026: ........................C..........T..............  Ggorilla
445480: ...................................-..............  Pabelli

                                 LINE1
        ------------------------------------------------->
               70895     70905     70915     70925     70935
 70886: TTTCCCTCTTAGCACTGTGTTAGCTGCATCCCAGAGATTCTGATACATTA  Hsapiens
390620: ..................................................  Ptroglodytes
408076: ........--.......C................................  Ggorilla
445529: .................C............G...................  Pabelli

                                 LINE1
        ------------------------------------------------->
               70945     70955     70965     70975     70985
 70936: TCTTTTTGTTTTCATTGGTTTCAAAGAACTTCTTGATTTCTGCCTTAATT  Hsapiens
390670: ..................................................  Ptroglodytes
408124: ...G..............................................  Ggorilla
445579: ...C..............................................  Pabelli

                                 LINE1
        ------------------------------------------------->
               70995     71005     71015     71025     71035
 70986: TCACCATTTACCCAGGAGTCATTCAGGAGCAGGTTGTTCAATTTCCATGT  Hsapiens
390720: ...........................C......................  Ptroglodytes
408174: ..................................................  Ggorilla
445629: ....T.............................................  Pabelli

                                 LINE1
        ------------------------------------------------->
               71045     71055     71065     71075     71085
 71036: AGTTGTGTGGTTTTGAGTGGGCTTTTAAATCTTGAGTTCTAATTTGATTG  Hsapiens
390770: ..................................................  Ptroglodytes
408224: .......................................G..........  Ggorilla
445679: ..................................................  Pabelli
```

```
                               LINE1
        ------------------------------------------------->
             71095     71105     71115     71125     71135
 71086: CACTGTGGTCTGAGAGACTGTTAAGATTTCAGTTCTTTTGCATTTGCTGA   Hsapiens
390820: ..................................................   Ptroglodytes
408274: ..................................................   Ggorilla
445729: ..................AA--............T.....TG........   Pabelli

                               LINE1
        ------------------------------------------------->
             71145     71155     71165     71175     71185
 71136: GGAGTGTTTTACTTCCAATTATGTGATCAATTTTAGAGTAAGTGTCATGT   Hsapiens
390870: .AT...............................................   Ptroglodytes
408324: ..................................................   Ggorilla
445777: ..................................................   Pabelli

                               LINE1
        ------------------------------------------------->
             71194     71204     71214     71224     71234
 71186: GGCACC-AAAAAAATGTATATTCTATTGTTTTGGGGTGGAGAGTTCTGTA   Hsapiens
390920: ......A...........................................   Ptroglodytes
408374: ......-........................C.T................   Ggorilla
445827: ......-.........................T.................   Pabelli

                               LINE1
        ------------------------------------------------->
             71244     71254     71264     71274     71284
 71235: TGTATCTATCAGGTCCACTTGGTCTAGAACTAAGCTCAAGTCCTGAATAT   Hsapiens
390970: G.................................T..............C   Ptroglodytes
408423: G.................................T...............   Ggorilla
445876: G...........C.T..............G....T...............   Pabelli

                               LINE1
        ------------------------------------------------->
             71294     71304     71314     71324     71334
 71285: ATTTGCTAACTTTCTCTCTCAATGATTTGTCTAATACTGGCAATGGGGTA   Hsapiens
391020: .......................CG.........................   Ptroglodytes
408473: ..................................................   Ggorilla
445926: ..................................................   Pabelli

                               LINE1
        ------------------------------------------------->
             71344     71354     71364     71374     71384
 71335: TTAAAGTCTTCCACTATTATTGTGTGGGAGTCTAAGTCTCTTTGTAGGTC   Hsapiens
391070: ..................................................   Ptroglodytes
408523: ..............G...................................   Ggorilla
445976: ...........................A..............C.......   Pabelli

                               LINE1
        ------------------------------------------------->
             71394     71404     71414     71424     71434
 71385: TCTAAGAACTTGTTTTATGAATCTGGGTGTTCCTGTATTAGGTGCATATA   Hsapiens
391120: ..................................................   Ptroglodytes
408573: ..................................................   Ggorilla
446026: .....................................C............   Pabelli
```

```
                         LINE1
        ------------------------------------------------->
                 71444     71454     71464     71474     71484
                     :         :         :         :         :
 71435: TATTAAGAATAGTTAGCTTTTCTTGTTGAATTGAACCTTTTAACATTATG  Hsapiens
391170: ..................................................  Ptroglodytes
408623: .....................................A............  Ggorilla
446076: .T.....G..........................G..C............  Pabelli

                         LINE1
        ------------------------------------------------->
                 71494     71504     71514     71524     71534
                     :         :         :         :         :
 71485: TAAGACCCTTCTTTGTCTTTTTTGACCTTTGTTGGTTTAAAGTCTATTTT  Hsapiens
391220: .................................A........C.......  Ptroglodytes
408673: ..................................................  Ggorilla
446126: ...TG.............................................  Pabelli

                         LINE1
        ------------------------------------------------->
                 71544     71554     71564     71574     71584
                     :         :         :         :         :
 71535: GTCAGAAACTAGGATTGCAACCCCTGCTTTTTTCTGCTTTCCATTTGCTT  Hsapiens
391270: ..................................................  Ptroglodytes
408723: ..........G.........T.............................  Ggorilla
446176: A..................G..T...........................  Pabelli

                         LINE1
        ------------------------------------------------->
                 71594     71604     71614     71624     71634
                     :         :         :         :         :
 71585: GGTAAATTTGCCTCCAACCCCTTTATTTGAATCTATGTGCATCTTTGCACA  Hsapiens
391320: .............................G....................  Ptroglodytes
408773: .............................G....................  Ggorilla
446226: .............G...............G.................T..  Pabelli

                         LINE1
        ------------------------------------------------->
                 71644     71654     71664     71674     71684
                     :         :         :         :         :
 71635: TGAGATGTGTCTCTTGAATACAGCACACCAACGGGTCTTGTCTTTGTGTC  Hsapiens
391370: ...............................T..................  Ptroglodytes
408823: ..................................................  Ggorilla
446276: ...............................T...............A..  Pabelli

                         LINE1
        ------------------------------------------------->
                 71694     71704     71714     71724     71734
                     :         :         :         :         :
 71685: CAGCTTGCCATTCTGTGTCCTTTAATTGGGGCATTTAGTCCATTTACATT  Hsapiens
391420: ...................T..................C...........  Ptroglodytes
408873: ...................T...........T......C...........  Ggorilla
446326: ...................T.................AC...........  Pabelli

                         LINE1
        ------------------------------------------------->
                 71744     71754     71764     71774     71784
                     :         :         :         :         :
 71735: TAAAGTTCATATTGTTATGTGTGAATTTGATCCTGTCATCATGATGCTGG  Hsapiens
391470: ..................................................  Ptroglodytes
408923: ..................................................  Ggorilla
446376: ...................................C......C.......  Pabelli
```

```
                                     LINE1
        ------------------------------------------------------>
                 71794     71804     71814     71824     71834
  71785: CCAGTTAATTTTACAGACTTGTTAATGTAGTTGCTTCATAGTGTCATTGG  Hsapiens
 391520: ................G.................................  Ptroglodytes
 408973: ................G.............................C...  Ggorilla
 446426: ................GT...C....C.......................  Pabelli

                                     LINE1
        ------------------------------------------------------>
                 71844     71854     71864     71874     71884
  71835: TCTGTGTACTTCAGTGTGTTTTTGTAGTGGCTGGTAACAGTTTTTCCTAT  Hsapiens
 391570: ............................-.....................  Ptroglodytes
 409023: ..................................................  Ggorilla
 446476: ...A..............................................  Pabelli

                                     LINE1
        ------------------------------------------------------>
                 71894     71904     71914     71924     71934
  71885: CCATGTTTAGTGATTGCTTCAGGAGCTCTTGCAAGGCAGGCCTGGTGGTG  Hsapiens
 391619: ............C.....................................  Ptroglodytes
 409073: ............C.....................................  Ggorilla
 446526: ............C...........A.........................  Pabelli

                                     LINE1
        ------------------------------------------------------>
                 71944     71954     71964     71974     71984
  71935: ACAAAATCCCTCAGCATTTGCTTGTCTGAAAAATATTTTATTTCTCCTTC  Hsapiens
 391669: .............................................G....  Ptroglodytes
 409123: ...................................C..............  Ggorilla
 446576: ..................................................  Pabelli
 435661:                                     ..............  Mmulatta

                                     LINE1
        ------------------------------------------------------>
                 71994     72004     72014     72024     72034
  71985: ATTTATGAAGCTCAGTTTGGCCAGATACGAAATTCTGGGTTGGAATTTAT  Hsapiens
 391719: .............G.............T......................  Ptroglodytes
 409173: .............G.............T........A.............  Ggorilla
 446626: .....C.....................T......................  Pabelli
 435675: .C............A............T......................  Mmulatta

                                     LINE1
        ------------------------------------------------------>
                 72044     72054     72064     72074     72084
  72035: TTTATTTAAGAATGTTGAACATTAGTCCCCAATCTCTTCTGACTTGCAGA  Hsapiens
 391769: ................................................--  Ptroglodytes
 409223: ..................................................  Ggorilla
 446676: .............................G...........G........  Pabelli
 435725: .........................................G...A...G  Mmulatta
```

```
                                LINE1
          ------------------------------------------------->
                72094     72104     72114     72124     72134
   72085: GTTTCTACTGAGAGGTCCACTGTTAGTCTGATGGGCTTCCCTTTGTAGAT Hsapiens
  391817: .................................................. Ptroglodytes
  409273: .................................................. Ggorilla
  446726: ................................................G. Pabelli
  435775: .....AG.....------C...........................G.G. Mmulatta

                                LINE1
          ------------------------------------------------->
                72144     72154     72164     72174     72184
   72135: GACCTGGCCTTTCTCTCTTGCCCCCCTTTAACATTTTTTCCTTCATTTCA Hsapiens
  391867: ........T......................................... Ptroglodytes
  409323: ....................T............................. Ggorilla
  446776: ....................-.T........................... Pabelli
  435819: ....................-.T...C................-...... Mmulatta

                                LINE1
          ------------------------------------------------->
                72194     72204     72210     72220     72230
   72185: ACCTTGGAGAATCTGATGAT----TATGTGTCTTGGGGTAGATCTTTTCA Hsapiens
  391917: ....................TATG.......................... Ptroglodytes
  409373: ................C...----.......................... Ggorilla
  446825: .............C......----.......................... Pabelli
  435867: ....CA..........A...----.T...........A......C..... Mmulatta

                                LINE1
          ------------------------------------------------->
                72240     72250     72260     72270     72280
   72231: TGGAGTATCTTACTGGGGTTCTCTGGATTTTCTGAATTTGAATATTGGCC Hsapiens
  391967: A...........TG.................................... Ptroglodytes
  409419: .................................................. Ggorilla
  446871: ....................................A............. Pabelli
  435913: ............T....................A.............T.. Mmulatta

                                LINE1
          ------------------------------------------------->
                72290               72298     72308     72318
   72281: TGTCTTTCTAGGTT------------GGGGAAATTCTCCTGGATGATATC Hsapiens
  392017: ..............TCTAGAATTTCCA....................... Ptroglodytes
  409469: CA............------------........................ Ggorilla
  446921: ..............------------........................ Pabelli
  435963: ..............------------..................A..... Mmulatta

                                LINE1
          ------------------------------------------------->
                72328     72338     72348     72358     72368
   72319: CTGAAGTGTGTTTTCCAATTTGGTTCCATTCTCCCCATCTTTCAGGTACT Hsapiens
  392067: .................................................. Ptroglodytes
  409507: ..........................................T....... Ggorilla
  446959: .................................................. Pabelli
  436001: .................................................. Mmulatta
```

```
                             LINE1
        ------------------------------------------------------>
                 72378     72388     72398     72408     72418
                     :         :         :         :         :
  72369: CCAATCAGTCACAGCTTTGGTCTTTCTACATAGTCTCATAATTCTTGGAG  Hsapiens
 392117: ...G..........G...................................  Ptroglodytes
 409557: ...G..........G...................................  Ggorilla
 447009: ...G..........G...................................  Pabelli
 436051: ............CAG..CA...............................  Mmulatta
```

```
                             LINE1
        ------------------------------------------------------>
                 72428     72438     72447     72457     72467
                     :         :         :         :         :
  72419: GTTTTGTTCATTCTTTTTCATTC-TTTTTTCTCTAATCTTTTCTGCCTGC  Hsapiens
 392167: ....A..................-................G.........  Ptroglodytes
 409607: ....A..................-................G.........  Ggorilla
 447059: .......................-C...............G.........  Pabelli
 436101: A......................T................G.........  Mmulatta
```

```
                             LINE1
        ------------------------------------------------------>
                 72477     72487     72497     72507     72517
                     :         :         :         :         :
  72468: CTTATTTCAGCAAGAGAATTATCAGTGCTTATAGTCTTCAAGCACTGATA  Hsapiens
 392216: ..................-................T..............  Ptroglodytes
 409656: ..................-...........................T...  Ggorilla
 447108: ..................-.......................T.......  Pabelli
 436151: ..............----------------....................  Mmulatta
```

```
                             LINE1
        ------------------------------------------------------>
                 72527     72537     72547     72557     72567
                     :         :         :         :         :
  72518: TTCTCTCTCCTACTTGGTTGATTTGGCTGTTGATACTTCGTGTTTGCATC  Hsapiens
 392265: .......................................A..........  Ptroglodytes
 409705: ..................................................  Ggorilla
 447157: ........T.......T.C.........A.....................  Pabelli
 436185: ........T.......A..............A.......A.......C..  Mmulatta
```

```
                             LINE1
        ------------------------------------------------------>
                 72577     72587     72597     72607     72617
                     :         :         :         :         :
  72568: ATGAAGTTCTTATGCTGTGTTTTTCAGTTCCATGAGGTCGTTTATGTTCC  Hsapiens
 392315: ...........................C......................  Ptroglodytes
 409755: ...........................C......................  Ggorilla
 447207: ..........C...T...........CC...........A..........  Pabelli
 436235: ......C...C............................A..........  Mmulatta
```

```
                             LINE1
        ------------------------------------------------------>
                 72627     72637     72647     72657     72667
                     :         :         :         :         :
  72618: TCTCTAGGCTGGTTATTCTGGTTAACCAACTCCTGTAACCTTTTACCATG  Hsapiens
 392365: ......................................T...........  Ptroglodytes
 409805: ......................................T.........C.  Ggorilla
 447257: .......A...A................C.........T......-T...  Pabelli
 436285: .......A....................G........GT.......T...  Mmulatta
```

```
                                       LINE1
         ------------------------------------------------------------------>
                 72677     72687     72697     72707     72717
                     :         :         :         :         :
 72668: GTTCTTAGCTTTTTTGCATTGGGTTAGAACATAATCCTTTAGCTCAGTGA  Hsapiens
392415: ...........C......................................  Ptroglodytes
409855: ...........C......................................  Ggorilla
447306: ...........C......................................  Pabelli
436335: ...........C..G.....................T.............  Mmulatta

                                       LINE1
         ------------------------------------------------------------------>
                 72727     72737     72747     72757     72767
                     :         :         :         :         :
 72718: AATTCATTATTACCCAATTATTACACACTTTCTAAATCCTGCTTCTGTCA  Hsapiens
392465: ..................................................  Ptroglodytes
409905: ..................................................  Ggorilla
447356: ........................C...........C.............  Pabelli
436385: .................-----------........C.............  Mmulatta

                                       LINE1
         ------------------------------------------------------------------>
                 72777     72787     72797     72807     72817
                     :         :         :         :         :
 72768: GTTCCTCCATCTCAGCTTCAGGCCTGTTCTCTGTACCTGCTGGAGATGTG  Hsapiens
392515: ..............................G...C...............  Ptroglodytes
409955: ..................................C...............  Ggorilla
447406: A.....T.......................G...C......A........  Pabelli
436424: ...........-..................G.A.C...............  Mmulatta

                                       LINE1
         ------------------------------------------------------------------>
                 72827     72837     72847     72855     72865
                     :         :         :         :         :
 72818: TTGCAATCATTTGGAGGAGAAGAGGCATTCTGGTTT--GGGATTTTCATT  Hsapiens
392565: ..........................T.........--....C......C  Ptroglodytes
410005: ....C...............................--............  Ggorilla
447456: .....................C..............--.......G...C  Pabelli
436473: ...................G................TG...........C  Mmulatta

                                       LINE1
         ------------------------------------------------------------------>
                 72875     72885     72895     72905     72915
                     :         :         :         :         :
 72866: GTTTTTGTGCTGGTTTTTCCTCATCTCCATGGATTTATCTGCCTTTGATC  Hsapiens
392613: ..........................T.......................  Ptroglodytes
410053: ..........................T.....................C.  Ggorilla
447504: .........G................T.......................  Pabelli
436523: .........T................T.............A.........  Mmulatta

                                       LINE1
         ------------------------------------------------------------------>
                 72925     72935     72945     72955     72965
                     :         :         :         :         :
 72916: TTTGAGGCTGATGACCTTTGGATGGGGCTTTTGTGGGGTCTTTTTTGTTG  Hsapiens
392663: ..................................................  Ptroglodytes
410103: ..................................................  Ggorilla
447554: ..................................................  Pabelli
436573: .............G........CA.............A............  Mmulatta
```

```
                         LINE1
 ------------------------------------------------------------>
             72975     72985     72994     73004     73014
                 :         :         :         :         :
 72966: ATGTTGTTGTTGTTGCTTTCTGTTTG-TTTTTCTTCTAACAGTCAGACCC   Hsapiens
392713: ..........................-...........G...........   Ptroglodytes
410153: ...C......................-.......................   Ggorilla
447604: ..........................-........T..........G...   Pabelli
436623: ..A.......................T...................G...   Mmulatta

                         LINE1
 ------------------------------------------------------------>
             73024     73034     73044     73054     73064
                 :         :         :         :         :
 73015: GTCTTCTGCAGGTCTGCTGCAGTTTGCTGGGGGTCCACTCCAGACCCTGT   Hsapiens
392762: A...........................................T.....   Ptroglodytes
410202: ...............................................C.C   Ggorilla
447653: C..................T..............................   Pabelli
436673: C.....G....C........G.............................   Mmulatta

                         LINE1
 ------------------------------------------------------------>
             73074     73084     73094     73104     73114
                 :         :         :         :         :
 73065: TGGCCTGGCTATCACCAGCAGAGTTTTCAGAATGGCAAAGATTCCTGCCT   Hsapiens
392812: .......................G..........................   Ptroglodytes
410252: .......................G..........................   Ggorilla
447703: .......TA..............G..........................   Pabelli
436723: ........G..............C..........................   Mmulatta

                         LINE1
 ------------------------------------------------------------>
             73124     73134     73144     73154     73164
                 :         :         :         :         :
 73115: GCTCCTTCCTCTGGAAGCTTCATCCCAGACAGGCACCGACCCGAAGCCAG   Hsapiens
392862: .....................G...................T........   Ptroglodytes
410302: .....................G...................T........   Ggorilla
447753: .....................G.......G....-......T........   Pabelli
436773: .............C.......G..T....G......T....T........   Mmulatta

                         LINE1
 ------------------------------------------------------------>
             73174     73184     73194     73204     73214
                 :         :         :         :         :
 73165: CCAGAGCTATCTTGTATGAGGTGTCTGCCAACCCCTGTTGGGAGGTCTCA   Hsapiens
392912: ...............T..................................   Ptroglodytes
410352: ..................................................   Ggorilla
447802: ..................................................   Pabelli
436823: A.................................................   Mmulatta

                         LINE1
 ------------------------------------------------------------>
             73224     73234     73244     73254     73264
                 :         :         :         :         :
 73215: CCCAGACAGGAGGCATGGGGTCAGGGACCCTCTTGAGGAAGCAGTCTGTC   Hsapiens
392962: .......................C..........................   Ptroglodytes
410402: ..................................................   Ggorilla
447852: ...................T.............................A   Pabelli
436873: .........................................A.T..CA..   Mmulatta
```

LINE1

```
                 73274     73284     73294     73304     73314
 73265: CCTTAGCAGAGCTGGTGTTCTGTGCTAGAGGAGTCCCCCCTCGTCAGATT  Hsapiens
393012: ..................................................  Ptroglodytes
410452: ..............................A...................  Ggorilla
447902: T.........................G......--.......A.......  Pabelli
436923: ..........................G.......-GT.............  Mmulatta
```

LINE1

```
                 73324     73334     73344     73354     73364
 73315: CAGCCTATCTCTTCAGAGCCAGCAGGCAGGAAAGATTAAGTCTGCTGAAC  Hsapiens
393062: .....G............................................  Ptroglodytes
410502: ..................................................  Ggorilla
447950: .....G............................................  Pabelli
436972: .....A............T...............................  Mmulatta
```

LINE1

```
                 73374     73384     73394     73404     73414
 73365: CTGGGACCATGGCCACCCCTCCCCACAGGTGCTCTGTCCTAGGGAGATGA  Hsapiens
393112: ..................................................  Ptroglodytes
410552: ..................................................  Ggorilla
448000: ....A..................................C..........  Pabelli
437022: .............................C.........C.....C....  Mmulatta
385520:          ..............................C..........  Panubis
```

LINE1

```
                 73424     73434     73444     73454     73464
 73415: GAGTTCTGTCTATAAGCTCCTTATTGGAGCTGGTGGACTTCCTGCAGGGA  Hsapiens
393162: ..................................................  Ptroglodytes
410602: ..................................................  Ggorilla
448050: ..................................................  Pabelli
437072: ...........G......................................  Mmulatta
385561: ...........G......................................  Panubis
```

LINE1

```
                 73474     73484     73494     73504     73514
 73465: AGCCCTGCCTGGTGAGGAGGGATCTAGAGAAGCAGTCTGGCCACAGCCAC  Hsapiens
393212: T.................................................  Ptroglodytes
410652: ....................A.............................  Ggorilla
448100: T.......................................A.........  Pabelli
437122: G......................................T..........  Mmulatta
385611: G......................................T..........  Panubis
```

LINE1

```
                 73524     73534     73544     73554     73564
 73515: TTTGCTACACTGTGGGGAATTCCATCAAGTCCAAACCTCCCAGTCCCCTT  Hsapiens
393262: ........................C.........................  Ptroglodytes
410702: ........................C.........................  Ggorilla
448150: ........................C.........................  Pabelli
437172: .......T........A..G....C................G...T....  Mmulatta
385661: .......T........A..G....C....................T....  Panubis
```

```
                             LINE1
        ------------------------------------------------->
              73574     73584     73594     73604     73614
 73565: AGCAATGTCAGGGGAAAACTGCCTACTAAAGCCACAGTAATAGTGGTCAG  Hsapiens
393312: ................................................GC  Ptroglodytes
410752: .....C............................................  Ggorilla
448200: ......A......A.....C............................GC  Pabelli
437222: ...................C..T.........................GC  Mmulatta
385711: ...................CA.T.........................GC  Panubis

                             LINE1
        ------------------------------------------------->
              73624     73634     73644     73654     73664
 73615: CCCTCCCCCCTCCCCAACCCTCTGGGAACTCGATCATCCCAGGAAGATTC  Hsapiens
393362: ...............C.T..C.............................  Ptroglodytes
410802: ..................................................  Ggorilla
448250: ...............C....C.............................  Pabelli
437272: ....T..T.......T....C..........T...........C......  Mmulatta
385761: ....T..T.......T....C..........T...........C......  Panubis

                             LINE1
        ------------------------------------------------->
              73674     73684     73694     73704
 73665: CAGACTACTGTGCTGATCATGGGGATTTCAAGCCAGGGTGCTTAGCT---  Hsapiens
393412: ...............................................---  Ptroglodytes
410852: .....................................A.........---  Ggorilla
448300: ..................................G........G...---  Pabelli
437322: ......G....T...........A.......................TGC  Mmulatta
385811: ......G....T...........A................T......---  Panubis

                             LINE1
        ------------------------------------------------->
                        73718     73728     73738     73748
 73712: -------------TGCTGGGCTCCATGGGACTGGGATCCACTGAGCGAGA  Hsapiens
393459: -------------.................G...................  Ptroglodytes
410899: -------------.....................................  Ggorilla
448347: -------------.A....C..........G......A...C...T....  Pabelli
437372: TGGGCTCCATGGG.................G.........T.........  Mmulatta
385858: -------------.................G...............A...  Panubis

                             LINE1
        ------------------------------------------------->
              73758     73768     73778     73788     73798
 73749: CTGCTTGGCTCCCTGGCTTCAGCCCCCTTTCCATGCGAGTGGACTGTTCT  Hsapiens
393496: ...................................G..............  Ptroglodytes
410936: ..............T.........T..........G.........A....  Ggorilla
448384: ...............T...................G.C............  Pabelli
437422: ...................................G....A...A.....  Mmulatta
385895: ...................................G........A.....  Panubis
```

```
                              LINE1
         ---------------------------------------------------->
                 73808     73818     73828     73838     73848
                     :         :         :         :         :
  73799: CCTTTCTCACTGGAGTTCTAGGCACCACTAGAGTATGTAAAAACTCCTGC  Hsapiens
 393546: ...G.....................TG.......................  Ptroglodytes
 410986: ...G..............................................  Ggorilla
 448434: ..CG.....................TG.......................  Pabelli
 437472: .T.G........A.............G.......................  Mmulatta
 385945: ...G..............................................  Panubis
```

```
                              LINE1
         ---------------------------------------------------->
                 73858                 73874     73884     73894
                     :                     :         :         :
  73849: AGCTCAGTGCCTGCCCA----GCCACCCAGTTTTGTGCTTGAAACCCAGG  Hsapiens
 393596: .................----.............................  Ptroglodytes
 411036: .........T.......----.............................  Ggorilla
 448484: .................----.........................TG..  Pabelli
 437522: .................AACA.T.....T.....................  Mmulatta
 385995: .................AACA.T.....T.....................  Panubis
```

```
                              LINE1
         ---------------------------------------------------->
                 73904     73914     73924     73934     73944
                     :         :         :         :         :
  73895: GCCCTAGTGGTGTAGGCTCATGAGGGAATCTCCTGATTCATAGATTGCAA  Hsapiens
 393642: ..................................................  Ptroglodytes
 411082: ....................C.............................  Ggorilla
 448530: ..................T.C.............................  Pabelli
 437572: ................T...CA..........T.................  Mmulatta
 386045: ........A.......T...C...........T.................  Panubis
```

```
                              LINE1
         ---------------------------------------------------->
                 73954     73964     73974     73984     73994
                     :         :         :         :         :
  73945: AAATCCATGGGAAAAGCGTTGTACCCCTGATGGGTAGCACAGTCCCTCAC  Hsapiens
 393692: ......G......................G....................  Ptroglodytes
 411132: ........................-.........................  Ggorilla
 448580: .................A...........C....................  Pabelli
 437622: ...C.............T.CA...T....G............C.......  Mmulatta
 386095: .................T.CA...T....G............C.......  Panubis
```

```
                              LINE1
         ---------------------------------------------------->
                 74004     74014     74024     74034     74044
                     :         :         :         :         :
  73995: CGCTTCCCTTGGCTGGGGGAGCAAGGCCCCCAACTCCGTGCACTTCCCAG  Hsapiens
 393742: T.................................................  Ptroglodytes
 411181: .....................-............................  Ggorilla
 448630: ...............A........A.........................  Pabelli
 437672: .A....................G............T..............  Mmulatta
 386145: .A....................G............T..............  Panubis
```

```
                                LINE1
        --------------------------------------------------->
              74054     74064     74074     74084     74094
                  :         :         :         :         :
 74045: GTGAAGCGATGTTCCAACCTGCTTGGGCTTGCTCTCAGTGGATTGCACCC  Hsapiens
393792: ..................................................  Ptroglodytes
411230: ..................................................  Ggorilla
448680: ......T....-.G....T...............................  Pabelli
437722: ...........C....C........................G........  Mmulatta
386195: .......A...C....C................T.......G........  Panubis

                                LINE1
        --------------------------------------------------->
              74104     74114     74124     74134     74144
                  :         :         :         :         :
 74095: ACCGCCTAGCCAATCCTGATGAGATGAACTGTGTACCTCAGTTGGAAATG  Hsapiens
393842: ..T.............C.............T.........A.........  Ptroglodytes
411280: ................C.................................  Ggorilla
448729: .T..............C......................T..........  Pabelli
437772: ..T.......T.G...CA...............C................  Mmulatta
386245: ..........T.G...CAG..............C................  Panubis

                                LINE1
        --------------------------------------------------->
              74154     74164     74174     74184     74194
                  :         :         :         :         :
 74145: CAGAAGTCACCCACATTCTGCATTGGTCTTGCTGGGAGCTGAAGGCCAGA  Hsapiens
393892: .............................C....................  Ptroglodytes
411330: ..................................................  Ggorilla
448779: ........................A....C....................  Pabelli
437822: ...................A..C......C....................  Mmulatta
386295: ...................A..C......C....................  Panubis

                      LINE1                        LINE1
        ------------------------------->          ---->
              74204     74214     74224     74234     74244
                  :         :         :         :         :
 74195: GCTGTTTCTATTCAGCCAAATTGGCCCCAGCTTGCCAATCTCTTTAACAA  Hsapiens
393942: ...................C..............................  Ptroglodytes
411380: ................T.................................  Ggorilla
448829: ...................C..................A...........  Pabelli
437872: ..A................C............................G.  Mmulatta
386345: ..A................C............................G.  Panubis

                                LINE1
        --------------------------------------------------->
              74254     74264     74274     74284     74294
                  :         :         :         :         :
 74245: ACCCAACCATAGTGTTACCATGCAATCCAGCAATTGCATTCCTAGGTATT  Hsapiens
393992: ................................G.................  Ptroglodytes
411430: ..................................................  Ggorilla
448879: .....GT...........................................  Pabelli
437922: ..................TC..............................  Mmulatta
386395: ..................T...............T..............  Panubis
```

```
                                  LINE1
          ------------------------------------------------------>
                 74304     74314     74324     74334     74344
  74295: TACCTGAATAGGTTGAAATTTATATCCACCCAAATAAGCTTAAATTTATG  Hsapiens
 394042: ....................C.............................  Ptroglodytes
 411480: ....................C.............................  Ggorilla
 448929: ....................C.................T...........  Pabelli
 437972: .....T.C....C.......C..G..............T.......C...  Mmulatta
 386445: .......G....C.......C..G..............T...........  Panubis
```

```
                                  LINE1
          ------------------------------------------------------>
                 74354     74363     74373     74383     74393
  74345: CCACACAAAT-ACCTGCACACAAGTGTTTATGGAAGCTTTACTTATTGTC  Hsapiens
 394092: .T........C.......................................  Ptroglodytes
 411530: ..........-.......................................  Ggorilla
 448979: ..........-..............................T........  Pabelli
 438022: ..........-..............A...............T........  Mmulatta
 386495: ..........-..............................T........  Panubis
```

```
                                  LINE1
          ------------------------------------------------------>
                 74403     74413     74423     74433     74443
  74394: AAAAATTGGAACTAACAAAAATGTCCTTTAATAGTTGAATGGGTAAACAA  Hsapiens
 394142: .......................................C..........  Ptroglodytes
 411579: ..................................................  Ggorilla
 449028: .......................................CA.........  Pabelli
 438071: ..................T.......C............C.....C....  Mmulatta
 386544: ..................T.......C............C.....C....  Panubis
```

```
                                  LINE1
          ------------------------------------------------------>
                 74453     74463     74473     74483     74493
  74444: ACTGTGGTACATCCACACAATGGAGTATTATTCGGCACTTAAAAAAAGAT  Hsapiens
 394192: .......................................A..........  Ptroglodytes
 411629: .......................................A..........  Ggorilla
 449078: .....................T.................A......T...  Pabelli
 438121: ....................C..................AC.....T...  Mmulatta
 386594: ................................T......AC.....T...  Panubis
```

```
                                  LINE1
          ------------------------------------------------------>
                 74503     74513     74523     74533     74543
  74494: CCGTCAAACCATGAAAAGATATGGAGAAACTTTAAATGCATATTGTTAAG  Hsapiens
 394242: ..................................................  Ptroglodytes
 411679: ..................................................  Ggorilla
 449128: ..A....G.............................T......T.....  Pabelli
 438171: .......GG.....................C.....C.............  Mmulatta
 386644: .......GG.....................C.....C.............  Panubis
```

```
                             LINE1
        --------------------------------------------------->
                 74553     74563     74573     74583     74593
 74544: TGAAAGAAGACAACCTATGGCTACATACTGTGTGATTCAAACTATGTTAC  Hsapiens
394292: .........C........................................  Ptroglodytes
411729: ..................................................  Ggorilla
449178: .........C....G.............G.....................  Pabelli
438221: .........C..............G....................A.A..  Mmulatta
386694: .........C..............G....................A.A..  Panubis

                             LINE1
        --------------------------------------------------->
                 74603     74613     74623     74633     74643
 74594: ATTTTGGAAAAGACAAAACTATAGAGAGCATACAAAGATCAGTAGTTGCC  Hsapiens
394342: ..............................A...................  Ptroglodytes
411779: ..................................................  Ggorilla
449228: ....................................A.............  Pabelli
438271: ...........A................AG....................  Mmulatta
386744: ...........A................AG....................  Panubis

          LINE1
        ------------>
                 74653     74663     74673     74683     74693
 74644: AAGGGTTTAAGGTGTCAGCCTAAAAAAGAAACATGCACACACTAGAGAAA  Hsapiens
394392: .................................CA...............  Ptroglodytes
411829: .............A....................................  Ggorilla
449278: ...........................T.....CAT..............  Pabelli
438321: .....C...........................CA.....G.........  Mmulatta
386794: .................................CA.....G......G..  Panubis

                                              LTR
                           ------------------------------->
                 74703                                   74714
 74694: GTTATTCTTTAAATATGTT-----------------------------AT  Hsapiens
394442: ...................-----------------------------..  Ptroglodytes
411879: ...................-----------------------------..  Ggorilla
449328: AC.................-----------------------------.C  Pabelli
438371: A..................GGGTTTACTTGGGAAAAGAAAGAAAGATT..  Mmulatta
386844: A..................GGGTTTACTTGGGAAAAGAAAAAAAGATT..  Panubis

                              LTR
        --------------------------------------------------->
                 74724     74734     74744     74754     74764
 74715: AGCCTGGAATGCATGGAATGGCAAGCCACGAGTGCATTCAAAGAGGGAAG  Hsapiens
394463: ....C........................C....................  Ptroglodytes
411900: ..................................................  Ggorilla
449349: .............................C....................  Pabelli
438421: ..................C..........C.....C..............  Mmulatta
386894: ..................C..........C.....C..............  Panubis
```

```
                                LTR
         ------------------------------------------------------->
                 74774     74784     74794     74804     74814
                     :         :         :         :         :
 74765: GTTAAGGGAAGCTTTTTAGCAAAAAGAGATTTACATAAGCCACTTAGAGA  Hsapiens
394513: ..................................................  Ptroglodytes
411950: ..................................G...............  Ggorilla
449399: .....A..........................T...............A.  Pabelli
438471: ................................................A.  Mmulatta
386944: .....A..........................................A.  Panubis

                                LTR
         ------------------------------------------------------->
                 74824     74834     74844     74854     74864
                     :         :         :         :         :
 74815: TTGGGTTCATCTGTTCCAGAGGTTCAAAGCTGGAGTTGTTGTTAGTTCAT  Hsapiens
394563: ........................................---.......  Ptroglodytes
412000: ..................................................  Ggorilla
449449: ..........T.......................................  Pabelli
438521: .A........T...............................C.......  Mmulatta
386994: .A........T...........................C...C.......  Panubis

                                LTR
         ------------------------------------------------------->
                 74874     74884     74894     74904     74914
                     :         :         :         :         :
 74865: TGTTGGAGATGCTGGGAAATTGTTCCTCTAAGAACATCTTGTCTGAATTA  Hsapiens
394610: ...................G..............................  Ptroglodytes
412050: ...................G..............................  Ggorilla
449499: ...................G..............................  Pabelli
438571: ...................G..........G...T......C........  Mmulatta
387044: ...................G..........G...T......C........  Panubis

                                LTR
         ------------------------------------------------------->
                 74924     74934     74944     74954     74964
                     :         :         :         :         :
 74915: GTGCATCATAAAGAATTTCTGGTAATAAACCTTATCAAAGCAGGAGATGC  Hsapiens
394660: ..................................................  Ptroglodytes
412100: .................................................T  Ggorilla
449549: ..................................................  Pabelli
438621: ....G...............A..G.....T....................  Mmulatta
387094: ....................A..G.....T....................  Panubis

                                LTR
         ------------------------------------------------------->
                 74974     74984     74994     75004     75014
                     :         :         :         :         :
 74965: ATCAAGGATGAGAAAGAGGTTTTAGAAAGTCCTTTGAAACAGTTCTTCTC  Hsapiens
394710: ..G...............................................  Ptroglodytes
412150: ..................................................  Ggorilla
449599: ..G.........C.....................................  Pabelli
438671: ..G...............T...............G...............  Mmulatta
387144: ..G...............T...............G...............  Panubis
```

```
                                      LTR
         ------------------------------------------------------>
                 75024     75034     75044     75054     75064
                     :         :         :         :         :
 75015: TCAGACGTGTCAGCATGAGTCTCCTCTCCTTTGGGTCTTCCCAGACCTGA  Hsapiens
394760: ...............................C..................  Ptroglodytes
412200: ..................................................  Ggorilla
449649: .T....A...........................................  Pabelli
438721: .....T..........C...........T................T....  Mmulatta
387194: .....T..........C...........T................T....  Panubis

                                      LTR
         ------------------------------------------------------>
                 75074     75084     75094     75104     75114
                     :         :         :         :         :
 75065: TTTGTCTGGGTCTGACAAAAGTGATTTCTTCCTAGTATCTGCAACTTTCA  Hsapiens
394810: ..................................................  Ptroglodytes
412250: ..................................................  Ggorilla
449699: .....................................A............  Pabelli
438771: ................G.G...........T..G................  Mmulatta
387244: ......C.........G.............T..G................  Panubis

        LTR                        LINE1
        -->  -------------------------------------------------->
                 75124     75134     75144     75154     75164
                     :         :         :         :         :
 75115: CAAAGTCATGGAAATGGATTGGGAAGGGGTAAATAGGTGGAGCACAGGGA  Hsapiens
394860: ............................A.....................  Ptroglodytes
412300: ............................A.....................  Ggorilla
449749: ......A.....................A.....................  Pabelli
438821: .....GA.C...................A....C................  Mmulatta
387294: .....GA.CA..................A....C................  Panubis

                                     LINE1
         ------------------------------------------------------>
                 75174     75184     75194     75204     75214
                     :         :         :         :         :
 75165: ACATTTAGGGCAGTGAAACTTTTCTGTGTGACTCTATAATTAATGGTGGA  Hsapiens
394910: ..................G................G..............  Ptroglodytes
412350: ..................................................  Ggorilla
449799: ...........................A......--..............  Pabelli
438871: ................................A.................  Mmulatta
387344: ................................A.................  Panubis

                                     LINE1
         ------------------------------------------------------>
                 75224     75234     75244     75254     75262
                     :         :         :         :         :
 75215: TAGAAGTCATTATACACTTGTCAAAGCCCACAGGACTATGTGACAC--AG  Hsapiens
394960: ..............................T...............AA..  Ptroglodytes
412400: ..............................................--..  Ggorilla
449847: ..............................T...............AA..  Pabelli
438921: ...........T.G...G.A........T.T......G.A......AA.C  Mmulatta
387394: ...........T.G...G.A........T.T......G.A......AA.C  Panubis
```

```
                                LINE1
        ───────────────────────────────────────────────────►
                75272     75282     75292     75302     75312
                    :         :         :         :         :
 75263: AGTGAATTTAATGTAAACTATGGACTTTAGTTAATAATCAGATATCATAT  Hsapiens
395010: .....................T............................  Ptroglodytes
412448: ..................................................  Ggorilla
449897: G.................................................  Pabelli
438971: ............................C.....................  Mmulatta
387444: .....................................C............  Panubis

                                LINE1
        ───────────────────────────────────────────────────►
                75322     75332     75342     75352     75362
                    :         :         :         :         :
 75313: GGATTCATCAGATGTAACAAATGTACCACAATAATGCAAGATGTTAATAA  Hsapiens
395060: ..................................................  Ptroglodytes
412498: ....................................T.............  Ggorilla
449947: ..................................................  Pabelli
439021: ....---------................T....................  Mmulatta
387494: ....---------................T....................  Panubis

                                LINE1
        ───────────────────────────────────────────────────►
                75372     75382     75392     75402     75412
                    :         :         :         :         :
 75363: TAGAGAAAACTGTGACTGGGCAGGGTGGAGGCATTGTATAGGAAACCTCC  Hsapiens
395110: ............................................C.....  Ptroglodytes
412548: ..................................................  Ggorilla
449997: ..............G.......................C.....C...TT  Pabelli
439062: ..............G........T.......T..G.........TG...T  Mmulatta
387535: ..............G........T.......T.CG.........TG...T  Panubis

                                LINE1
        ───────────────────────────────────────────────────►
                75422     75432     75442     75452     75462
                    :         :         :         :         :
 75413: GTACTTTCTATTCCACTGTTCTATAAACTTAAACAGCTTAGTTTAAAAAT  Hsapiens
395160: ..................................................  Ptroglodytes
412598: ..................................................  Ggorilla
450047: ...............................................C..  Pabelli
439112: ..................................................  Mmulatta
387585: ..................................................  Panubis

      LINE1                                          Alu
        ──►                                     ────────────►
                75472     75482     75492     75502     75511
                    :         :         :         :         :
 75463: AATAATAATGATGTTGTGCTTTTTTCTGGGCTTAAGTTTTTT-TCTTGTT  Hsapiens
395210: .................A........................C.T..T..  Ptroglodytes
412648: ..........................................-.......  Ggorilla
450097: .G........................................--------  Pabelli
439162: ..........---C................T...........---..T..  Mmulatta
387635: ..........---C................T.....A.....--------  Panubis
```

```
                            Alu
        ------------------------------------------------->
             75521     75531     75540     75549     75558
 75512: TTTTTCTTTTTTTATTTTTGAAACAG-CGTCTTTC-TTTGTCG-CCCAGG  Hsapiens
395260: ..........................-........-.......-......  Ptroglodytes
412697: ..........................-........-......A-......  Ggorilla
450139: -----......C.............AC.A......T......AC......  Pabelli
439206: .GA.AT.....A.T............-........-..C....-.T....  Mmulatta
387674: ---------.A..T....GT......-........-..C....-.T....  Panubis

                            Alu
        ------------------------------------------------->
             75567     75577     75587     75597     75607
 75559: CT-GGAGTGCAGTGGCGGGATCTCAGCTCACTGCGGCTTACACCTCCCAG  Hsapiens
395307: ..-.............A..................A..............  Ptroglodytes
412744: ..-...............................................  Ggorilla
450184: ..G........A...                                     Pabelli
439253: ..-.............A...A..................C.........T  Mmulatta
387712: ..-.............A...A..................C......T...  Panubis

                            Alu
        ------------------------------------------------->
             75617     75627     75637     75647     75657
 75608: GTTCAAGCGATTCTTCTGCCTCAGCCTCTCAAGTAGCCGGGACTACAGGC  Hsapiens
395356: .......T.........................................G  Ptroglodytes
412793: ..................................................  Ggorilla
450552: ...........--C.T...G.....T..---..C....-.........CT  Pabelli
439302: ......................................A....G.G....  Mmulatta
387761: ......................................A...........  Panubis

                            Alu
        ------------------------------------------------->
             75667     75677     75687     75697     75707
 75658: ATGCACCACCACACCTGGCTAATCTTTGTATTTTCAGTAGAGATAGGGTT  Hsapiens
395406: ....G.............................................  Ptroglodytes
412843: ..................................................  Ggorilla
450596: GCA..----.....T.-....--TC.....-............C..A--.  Pabelli
439352: .......................T...................C......  Mmulatta
387811: .......................T...................C.....G  Panubis

                            Alu
        ------------------------------------------------->
             75717     75727     75737     75747     75757
 75708: TCACCATGTTGGTTAGATTGGTCTCAAACTCCTGACCTCAGGTGATCTGC  Hsapiens
395456: ................G.................................  Ptroglodytes
412893: ..................................................  Ggorilla
450636: ..............GTG........G........................  Pabelli
439402: ................G........G.........TG........C....  Mmulatta
387861: ................G........G.........TG........C....  Panubis
```

```
         Alu
        ------------------------------------------------->
             75767     75777     75787     75797     75807
 75758: CTGCCTCAGCCTCCCAAAGTGCTGGGATTACAGGTGTGAGCCACCACGTC  Hsapiens
395506: ................................................C.  Ptroglodytes
412943: ................................................C.  Ggorilla
450686: ................................................C.  Pabelli
439452: ...............................................AC.  Mmulatta
387911: ...............................................AC.  Panubis

        Alu
        ---->
             75817     75827     75837     75847     75857
 75808: TGGCCATCTGAGCCTAAGTCTTAAGAAGACCTGATCATTTCAATTCATGC  Hsapiens
395556: .A................................................  Ptroglodytes
412993: ..................................................  Ggorilla
450736: ....................................T...T..C......  Pabelli
439502: ..A..........................T............TC...G..  Mmulatta
387961: ..A......A...................A.............C...G..  Panubis

             75867     75877     75887     75897     75907
 75858: TCTTAGGAGTCTATAGTCACCATTTGAGTCTTGCTGGAGAAAGCATGTGA  Hsapiens
395606: ............G.....................................  Ptroglodytes
413043: ..................................................  Ggorilla
450786: ..........G.......................................  Pabelli
439552: ...............................C.....G.......C....  Mmulatta
388011: .......................C.......C.....G.G..........  Panubis

             75917     75927     75937     75947     75957
 75908: AAAAACCACAAGGAGGGAGCAAGTGGAAAGGAAGAGGTCCTGAAACAACA  Hsapiens
395656: ..................................................  Ptroglodytes
413093: ..................................................  Ggorilla
450836: ............A............................A........  Pabelli
439602: ..................................................  Mmulatta
388061: ..................................C...............  Panubis

             75967     75977     75987     75997     76007
 75958: GAGAAGAATCAAAGAAACAAGTCAATCGGGAGAAGCACAGCCTGCTCGTT  Hsapiens
395706: ...............................................A..  Ptroglodytes
413143: ..................................................  Ggorilla
450886: .......G..................A....................A..  Pabelli
355749:                                               .A..  Nleucogenys
439652: .......G..G............G..A..........G....C....A..  Mmulatta
388111: .......G...............G..A..........G....C....A..  Panubis

             76017     76027     76037     76047     76057
 76008: TTCCCAGTTGAGCCATTGCAGCCAGCCTCGAACTATTCCAGAAATTCTAG  Hsapiens
395756: ................................A.........C.......  Ptroglodytes
413193: ..........................................C.......  Ggorilla
450936: ...........................C.A...C........C.......  Pabelli
355753: .............................A........T...C.......  Nleucogenys
439702: ............T..............CTA............C.......  Mmulatta
388161: ............T..............CGA............C.......  Panubis
```

```
                                LTR
        ------------------------------------------------->
             76067     76077     76087     76097     76107
 76058: CAGAGGTACCAGGCATGTGAGTGAGTGAAGGAGCTTTCTTGGACCTTCTA  Hsapiens
395806: ..................................................  Ptroglodytes
413243: .................A................................  Ggorilla
450986: ..................................................  Pabelli
355803: ..................................................  Nleucogenys
439752: ......C.A.........................C...............  Mmulatta
388211: ......C.A.........A...............C...............  Panubis

                                LTR
        ------------------------------------------------->
             76117     76127     76137     76147     76157
 76108: GCCCTAGCAGACACAACGCAGAGCAGAAACAAGCCATCTCCACTGTTCTG  Hsapiens
395856: ..................................................  Ptroglodytes
413293: ..................................................  Ggorilla
451036: ..................................................  Pabelli
355853: ..................................................  Nleucogenys
439802: .......T.........A............G.................CA  Mmulatta
388261: .................A............G.................CA  Panubis

                                LTR
        ------------------------------------------------->
             76167     76177     76187     76197     76207
 76158: TTTGAATCCCTGACTCACAGAATTGCATACAAGTAAAATGGTCGTTATAT  Hsapiens
395906: ............G.C...................................  Ptroglodytes
413343: ...........................................A......  Ggorilla
451086: C.............C...................................  Pabelli
355903: ..............A............C...................C..  Nleucogenys
439852: ..C...........C...........C...............T.....T.  Mmulatta
388311: ..C...........C...G......----...........C.T.....T.  Panubis

                                LTR
        ------------------------------------------------->
             76217     76227     76237     76247     76257
 76208: TAAGCCACTTAGTTTTAAGATTGTTTATTATGCAGCAGCAGGTACGTGAA  Hsapiens
395956: ...................G........................T.....  Ptroglodytes
413393: ..................................................  Ggorilla
451136: ...................G..............................  Pabelli
355953: ....T..............G..............................  Nleucogenys
439902: .........C...G.....G........................T.....  Mmulatta
388357: ......G......G.....G........................T.....  Panubis

        LTR
        --->
             76267     76277     76287     76297     76307
 76258: ACACCAAGGAAATTTTTAACAATGTTCAAGAAAAGCTGATATTTCCTTTT  Hsapiens
396006: ................A..A..............................  Ptroglodytes
413443: ...................T..............................  Ggorilla
451186: ........T..........A..............................  Pabelli
356003: ........T..........A..............................  Nleucogenys
439952: ........T........T.A..C...........................  Mmulatta
388407: ........T........T.A..............................  Panubis
```

```
                  76317     76327     76337     76347     76356
                      :         :         :         :         :
  76308: GTTTCTCTTCCCTATAATGTTTGGCTCTATGCAAGTGGAAAAAAG-AGTG  Hsapiens
 396056: .............................A...............G....  Ptroglodytes
 413493: ..........................................C..-....  Ggorilla
 451236: ....T...........G.............A..............G....  Pabelli
 356053: .............................................G....  Nleucogenys
 440002: ......................................G......G....  Mmulatta
 388457: .............................................G....  Panubis

                  76366     76376     76386     76396     76406
                      :         :         :         :         :
  76357: TGCTTTCGTGTTCTGTCCATTCTACCAGAACTTTAGCTGAAGTCATTAGG  Hsapiens
 396106: ..................................................  Ptroglodytes
 413542: ..................................................  Ggorilla
 451286: ......T.....................................G.....  Pabelli
 356103: .......A..........................................  Nleucogenys
 440052: .....CT...........G...........T....A..............  Mmulatta
 388507: .....CT...........G...........T....A..............  Panubis

                  76416     76426     76436     76446     76456
                      :         :         :         :         :
  76407: AAAGGTGTACCTAGTTGAGATAACTACTCTTATAAAATCCTTTAATATGG  Hsapiens
 396156: ..................................................  Ptroglodytes
 413592: ..................................................  Ggorilla
 451336: .........T........................................  Pabelli
 356153: ...................C.........G....................  Nleucogenys
 440102: .......................................T..........  Mmulatta
 388557: .......................................T..........  Panubis

                                   LTR
            ---------------------------------------------->
                  76466     76476     76486     76496     76506
                      :         :         :         :         :
  76457: AACAGAGGGTGGCAGGAGGCAGCCAAATGCCTAGGCAGATAGGGACGGGT  Hsapiens
 396206: .............................................T....  Ptroglodytes
 413642: ..................................................  Ggorilla
 451386: .......A..........................................  Pabelli
 356203: ..................................................  Nleucogenys
 440152: ...............C......T............G..............  Mmulatta
 388607: ...............C......T.......................A..G  Panubis

                                 LTR
         ------------------------------------------------->
                  76516     76526     76536     76546     76556
                      :         :         :         :         :
  76507: CCCCGGTGAAACCCCACCTCCAAGCCAAAGACAGTTTAAAGCCTGAAAGC  Hsapiens
 396256: ..................................................  Ptroglodytes
 413692: ..................................................  Ggorilla
 451436: ...T..............................................  Pabelli
 356253: ....A.............................................  Nleucogenys
 440202: ...T...................C...AG.....................  Mmulatta
 388657: ...............................AG.................  Panubis
```

```
                         LTR
        ------------------------------------------------->
             76566     76576     76586     76596     76606
                 :         :         :         :         :
 76557: CAAGCTACAAGTTAAATCCTCAGACCAGATTGAGAACTTGTTTTCCTATT  Hsapiens
396306: .................................A................  Ptroglodytes
413742: ..................................................  Ggorilla
451486: ..................................................  Pabelli
356303: ..................................................  Nleucogenys
440252: .........................................C........  Mmulatta
388707: .........................................C........  Panubis
417851:                      ...A................C........  Cjacchus

                         LTR
        ------------------------------------------------->
             76616     76626     76636     76646     76656
                 :         :         :         :         :
 76607: TGGCATGCTTTCCTCTGATTGGTCCCCACCCTTAACCTATTTTACATATA  Hsapiens
396356: ..................................................  Ptroglodytes
413792: ..................................................  Ggorilla
451536: ..................................................  Pabelli
356353: ..................................................  Nleucogenys
440302: ..................................................  Mmulatta
388757: ..................................................  Panubis
417880: ...TG............G...A...........CC.............G.  Cjacchus

                         LTR
        ------------------------------------------------->
             76666     76676     76686     76696     76706
                 :         :         :         :         :
 76657: CCTACCCTTTCCTAATTGGTTTTCTACATTGTCATGCCCACCTTTGAGTG  Hsapiens
396406: ..................................................  Ptroglodytes
413842: ..................................................  Ggorilla
451586: .......................T.C..C.....................  Pabelli
356403: ....T.......................................CA....  Nleucogenys
440352: ..................................................  Mmulatta
388807: ............................................C.....  Panubis
417930: ......T.A...................C....---TA.........A..  Cjacchus

                         LTR
        ------------------------------------------------->
             76716     76726     76735     76745     76755
                 :         :         :         :         :
 76707: GTGTCTTCACTTTAAACTTTTTTGC-TACTCACAAACCAATCAGCATGCA  Hsapiens
396456: .........................-........................  Ptroglodytes
413892: .........................-........................  Ggorilla
451636: .........................A........................  Pabelli
356453: .........................A......................T.  Nleucogenys
440402: C................A.......A...............T........  Mmulatta
388857: .................A.......A........................  Panubis
417977: .........................A.............G.........T  Cjacchus

                         LTR
        ------------------------------------------------->
             76765     76775     76785     76795     76805
                 :         :         :         :         :
 76756: TTCCCCATTCTGAGTCCATGAAAGACCCTGGACCCAGCCACAGAGGGACT  Hsapiens
396505: ..........................T.C...............A.....  Ptroglodytes
413941: ..................................................  Ggorilla
451686: ............................C...............A.....  Pabelli
356503: ...............T............CA....................  Nleucogenys
440452: ........................G...C........T............  Mmulatta
388907: ........................G...C........T............  Panubis
418027: ...................A........CA............C.....T.  Cjacchus
```

```
                                  LTR
         ------------------------------------------------------>
                   76815     76825     76835     76845     76855
 76806: TTCACACCTTCAGGTAGGGGGACCACTCCCATGTTCCCTCTCCACTGAAA  Hsapiens
396555: ..........................C.......................  Ptroglodytes
413991: ..........................C.......................  Ggorilla
451736: ..........................C.......................  Pabelli
356553: .........................TC.......................  Nleucogenys
440502: ..........G...............C....C..................  Mmulatta
388957: ..........G...............C....C..................  Panubis
418077: ..T........T.T............C.......C....T..........  Cjacchus
```

```
                                  LTR
         ------------------------------------------------------>
                   76865     76875     76885     76895     76905
 76856: GCTGTTTCATCGCTCAATAAAATTCTTTTCCATCCTCCTCACTCTTCAGT  Hsapiens
396605: ..................................................  Ptroglodytes
414041: ........................A.........................  Ggorilla
451786: ...........A....................................A.  Pabelli
356603: ................................................A.  Nleucogenys
440552: ................................................A.  Mmulatta
389007: ....................................T...........A.  Panubis
418127: ...........A.......C.................A....CT.C..A.  Cjacchus
```

```
                                  LTR
         ------------------------------------------------------>
                   76915     76925     76935     76945     76955
 76906: GTCCTGCGTATCCTCTTTCTTCTTGAGTATGGTACAAGAGCTCAGGGATC  Hsapiens
396655: ....C.............................................  Ptroglodytes
414091: ..................................................  Ggorilla
451836: .......A.......A..............A...T..............T  Pabelli
356653: ............G..A-.................................  Nleucogenys
440602: .......A.......A..................T...............  Mmulatta
389057: .......A.......A......C...........................  Panubis
418177: ....CAT........G...........C..................A...  Cjacchus
```

```
                                  LTR
         ------------------------------------------------------>
                   76965     76975     76985     76995     77005
 76956: GCTGAATGTGGGTACAAGCTATAACACAGGCAAGTTGGGGCATGCCAGCA  Hsapiens
396705: ...................A..............................  Ptroglodytes
414141: ..................................................  Ggorilla
451886: ...A..............................C...............  Pabelli
356702: ..................................C...............  Nleucogenys
440652: A.................T...............C....A..........  Mmulatta
389107: A.................................C....A..........  Panubis
418227: A..........A......................C...C.......T...  Cjacchus
```

```
                                  LTR
         ------------------------------------------------------>
                   77015     77025     77035     77045     77055
 77006: TGGCTGAGTGGGGCCCAGGTGGGGCATTGCCAGCCAGAGATCTTCAGCTT  Hsapiens
396755: ....C.............................................  Ptroglodytes
414191: ....C.............................................  Ggorilla
451936: ....................................C.............  Pabelli
356752: ..................................................  Nleucogenys
440702: ....CT....AA..TGG.ACAA.A.G.CA.TG.T...G.GATCCTG....  Mmulatta
389157: ....C............T....A...........................  Panubis
418277: ..A.................A..............G......CC......  Cjacchus
```

```
                      LTR
            ----------------------------->
                  77065     77075     77085
                      :         :         :
  77056: GCAAAGCAAGCAAGAAGAAAAATCCTACATCAG-----------------  Hsapiens
 396805: .................................-----------------  Ptroglodytes
 414241: .................................-----------------  Ggorilla
 451986: .........C.......................-----------------  Pabelli
 356802: .........C.....................-.-----------------  Nleucogenys
 440752: ..G...TG.CTG..............G.....AAATCCCACTTGAACAAA  Mmulatta
 389207: .........C.......................-----------------  Panubis
 418327: .....AG..C...........C...........-----------------  Cjacchus
1060903:                            ..A..T-----------------  Sboliviensis

                                                      77097
                                                          :
  77089: -----------------------------------------AACTTTATG  Hsapiens
 396838: -----------------------------------------.........  Ptroglodytes
 414274: -----------------------------------------.........  Ggorilla
 452019: -----------------------------------------.........  Pabelli
 356834: -----------------------------------------.........  Nleucogenys
 440802: TACCAAAGATTAAATAGAATAAAAGTATTTCTTAAGTTGGT..T......  Mmulatta
 389240: -----------------------------------------.........  Panubis
 418360: -----------------------------------------T....CT..  Cjacchus
1060909: -----------------------------------------G........  Sboliviensis

                  77107     77117     77127     77137
                      :         :         :         :
  77098: AGGGAAATCAAGGATTAGCTGATCTCATTCTGTGGAATGA----------  Hsapiens
 396847: ........................................----------  Ptroglodytes
 414283: ........................................----------  Ggorilla
 452028: G.......................................----------  Pabelli
 356843: ........................................----------  Nleucogenys
 440852: .A......A...TG-...T..T...-G....CAAAT....TACTGAATAC  Mmulatta
 389249: .........-..............................----------  Panubis
 418369: ......G.................................----------  Cjacchus
1060918: ......G.................................----------  Sboliviensis

                  77145     77155     77165     77175     77185
                      :         :         :         :         :
  77138: --GCATGTTTAGTTGACAACATACCTGTGCACAACAAAGAAGGGATGATA  Hsapiens
 396887: --................................................  Ptroglodytes
 414323: --................................................  Ggorilla
 452068: --........................A..............-........  Pabelli
 356883: --.................T......A...................A...  Nleucogenys
 440900: ACA..CACAG...CAGTC...G.----..AT...TT..A.TT....-...  Mmulatta
 389288: --.............................T......C...........  Panubis
 418409: --....................G...A.....................C.  Cjacchus
1060958: --....................G...A.....................C.  Sboliviensis

                            77200     77210     77220     77230
                                :         :         :         :
  77186: TATAGTG-----GTTTTCAGCCTTCATTCCTAATTCTTTCTCACTGTAAA  Hsapiens
 396935: .......-----....C............................A....  Ptroglodytes
 414371: .......-----....................T............A....  Ggorilla
 452115: .......-----....C.G..........................A....  Pabelli
 356931: .......-----....C............................A....  Nleucogenys
 440945: .C...AAAAAAT.......AT----...ATA.T..TC.AT.TT..A.GC.  Mmulatta
 389336: C......-----...                                     Panubis
 418457: ..C....-----....C...T.................----G..A....  Cjacchus
1061006: ..C....-----....C........................AG..A....  Sboliviensis
```

```
                                                 LINE2
                                       ------------------->
                         77241     77251     77261     77271
                             :         :         :         :
   77231: TTCATCA---------AAAGCCTTAGTTGGGTATCCAACAAATATTAATT   Hsapiens
  396980: .......---------.......................C..........   Ptroglodytes
  414416: .......---------..................................   Ggorilla
  452160: G......---------.......................C.G........   Pabelli
  356976: .....G.---------....A..................C.....CC...   Nleucogenys
  440991: C.TC...GTGTAAGGG...ATA...-C.AT..TCT.TT.....CC....-   Mmulatta
  418498: ..T....---------.......................C.........C   Cjacchus
 1061051: ..T....---------.......................C..........   Sboliviensis
```

```
                    LINE2                        LTR
          -------------------------------->------------------>
               77281     77291     77301     77311     77321
                   :         :         :         :         :
   77272: GATCATCTATAACATGCAAGGTACTGTTCTAGTGTAACTGAGTTATCCCA   Hsapiens
  397021: ..................................................   Ptroglodytes
  414457: ..................................................   Ggorilla
  452201: ...........................................-......   Pabelli
  357017: .............G..................G.....-....-......   Nleucogenys
  441039: -----------------..AAG..CA....CA.TG.G.ATGA.-T.A.A.   Mmulatta
  418539: T.....G...G......................A.........-......   Cjacchus
 1061092: ......G...G.........T............A.........-......   Sboliviensis
```

```
                                   LTR
          ------------------------------------------------->
                         77332     77338               77348
                             :         :                   :
   77322: ATTT---------TTCTAAGAGATA----TAGAATGAGG----------T   Hsapiens
  397071: ....---------............----..........----------.   Ptroglodytes
  414507: ....---------............----..........----------.   Ggorilla
  452250: ....---------............----..........-------TTT.   Pabelli
  357065: ....---------......A.....----..........--------TT.   Nleucogenys
  441071: ..C.GGAAATGTA.G....AT..C.ATATC.A.......GACTTCTTTT.   Mmulatta
  389675:                                                  .   Panubis
  418588: ....---------.......C....----A.....A...----------.   Cjacchus
 1061141: ....---------.......C....----A.........-----------   Sboliviensis
```

```
                                   LTR
          ------------------------------------------------->
               77358     77368               77378     77388
                   :         :                   :         :
   77349: TTTTTTCTCTTCTTTTCTCTTT----------CCTCCCTTTCCCCAATTC   Hsapiens
  397098: ......................----------.............C....   Ptroglodytes
  414534: ......................----------..................   Ggorilla
  452280: ......................----------.............C....   Pabelli
  357094: .C....................----------.........T...CG...   Nleucogenys
  441121: ......T.T..T....T.T...GTGGGGGGGG.............CG...   Mmulatta
  389676: ......................----------.............G....   Panubis
  418615: ......................----------.....T.......-----   Cjacchus
 1061167: ...................A..----------.....T.......-----   Sboliviensis
```

```
                              LTR
         ------------------------------------------------------>
                  77398     77408     77418     77428     77438
  77389: CCCCACTTCTTACTTAGCTCTTTAGAAATGCAGTTAAAACCATTTACCTT Hsapiens
 397138: ................................A................. Ptroglodytes
 414574: ................................A................. Ggorilla
 452320: ................................A............C.... Pabelli
 357134: ................................A.....G........... Nleucogenys
 441171: ..GT............................A.........C...T... Mmulatta
 389716: .TG.............................A.........C...T... Panubis
 418650: ---.............................A.....G.......A... Cjacchus
1061202: ---T............................A........G....A... Sboliviensis

                              LTR
         ------------------------------------------------------>
                  77448     77458     77468     77478     77488
  77439: CCCTTCAACAGGCAAAATTATCTAACCATGTGCTTACTTAAAAGCTCCAG Hsapiens
 397188: ...........................G...................... Ptroglodytes
 414624: ...........................G...................... Ggorilla
 452370: ............A..........----G...................... Pabelli
 357184: ..........................TG...................... Nleucogenys
 441221: ...............C..........TG.....................- Mmulatta
 389766: ..........................TG...................... Panubis
 418697: ...A..T.......T.........G.TG...........T...A.....C Cjacchus
1061249: ...........A..T.........G.TG...............A....T. Sboliviensis

                              LTR
         ------------------------------------------------------>
                  77498     77508     77518     77528     77538
  77489: GGCAGAAATATCTCCCACCAGGAGATTGCCTTGAGAGACAATAGTCAATT Hsapiens
 397238: .................................................. Ptroglodytes
 414674: .................................................. Ggorilla
 452416: .........................G......C................. Pabelli
 357234: .....................A............................ Nleucogenys
 441270: A........C...............................C........ Mmulatta
 389816: .........C...............................C........ Panubis
 418747: A........C.................A.............C........ Cjacchus
1061299: A........C.................A.........G............ Sboliviensis

                              LTR
         ------------------------------------------------------>
                  77548     77558     77568     77578     77588
  77539: TATAACCAAAAGTATGCCTGCTACAAAATTCTCTCCCACCTGGAGAGAAT Hsapiens
 397288: ..............................G................... Ptroglodytes
 414724: .................................................. Ggorilla
 452466: .................................................. Pabelli
 357284: ..............................A.............. Nleucogenys
 441320: ..............................................T... Mmulatta
 389866: .................................................. Panubis
 418797: ..............................T................... Cjacchus
1061349: ..............................T........T.A........ Sboliviensis
```

LTR

```
               77598     77608     77618     77628     77638
  77589: CTGGAGACAAGACCACTCTACAATCTAGTTCTGACCATGATGGTGCCAGC  Hsapiens
 397338: .......................C..........................  Ptroglodytes
 414774: ....................................G.............  Ggorilla
 452516: .......................C..........................  Pabelli
 441370: ..........C............C...A......................  Mmulatta
 389916: ..........C............C...A.....................T  Panubis
 418847: ....................T..C....-...............A.....  Cjacchus
1061399: ....................T..G..........T...C.....A.....  Sboliviensis
```

LTR

```
               77648     77658     77668     77678     77688
  77639: TCAACCACCCAGTAGGTCAAATACCAAAACAAGTCACATGGACTCCCCAC  Hsapiens
 397388: ..................................................  Ptroglodytes
 414824: ..................................................  Ggorilla
 452566: .......T..........................................  Pabelli
 441420: ............................G.....................  Mmulatta
 389966: ............................G................T....  Panubis
 418896: ........T......A............G......C..G...........  Cjacchus
1061449: ...............A............G.........G.A.........  Sboliviensis
```

LTR

```
               77698     77707     77717     77727     77737
  77689: CAGCTCACTCCCTCCC-TGCGTGCCATTCATGCCACATTCCCTCTTAAAA  Hsapiens
 397438: ................-.................................  Ptroglodytes
 414874: ...G............-...A.............................  Ggorilla
 452616: ................C...A..G..........................  Pabelli
 441470: ................C...A......................T......  Mmulatta
 390016: ................C...A......................T......  Panubis
 418946: .............T..T...A.........CA..........GT......  Cjacchus
1061499: ....-..T.....T..C...A......................-......  Sboliviensis
```

LTR

```
               77747     77757     77767     77777     77787
  77738: TCCCCTGCTTTCTGCTCCAAAAGTGAAGCAGTACCCTTAAGGCAGGAAGC  Hsapiens
 397487: G.................................................  Ptroglodytes
 414923: G................G................................  Ggorilla
 452666: GG.........................................CA.....  Pabelli
 441520: G.................................................  Mmulatta
 390066: G.................................................  Panubis
 418996: G..............................C..........G.......  Cjacchus
1061547: A..............................C................A.  Sboliviensis
```

LTR

```
               77797     77807     77817     77827     77837
  77788: CTATACTTCTTCCCCTAAGCTAGCTTTGGAATAGAAAATCACGTTCTTTA  Hsapiens
 397537: ..............................G..................-  Ptroglodytes
 414973: ..................................................  Ggorilla
 452716: .CG..................................G............  Pabelli
 441570: ....G............................A...G...T........  Mmulatta
 390116: ....GT...........................A...G.A.T........  Panubis
 419046: .AG....C..........T......G.......A...G...T........  Cjacchus
1061597: .AG....C.................G.......A...G............  Sboliviensis
```

```
                                   LTR
          ------------------------------------------------->
               77847     77857     77867     77877     77887
                   :         :         :         :         :
  77838: TACCAGACCTCATTCTTGTGCGCTGCACTCTGCAAGCAGTGAGTGACTGC   Hsapiens
 397586: .........................G........................   Ptroglodytes
 415023: ..............................................T...   Ggorilla
 452766: ...........G.............G........................   Pabelli
 441620: ........T.TG.............G........................   Mmulatta
 390166: ..........TG.........A...G........................   Panubis
 419096: ..-TG............C..T....G.T...........G...C......   Cjacchus
1061647: ..............T..C..T....G.T...............C......   Sboliviensis

                                   77902     77912     77922
                                       :         :         :
  77888: TTTGCA---------------CTAGCATGATGCATACAGCAAGGAATACA   Hsapiens
 397636: ......---------------........G....................   Ptroglodytes
 415073: ......---------------.............................   Ggorilla
 452816: ......---------------........G....................   Pabelli
 441670: ......---------------........G.........T..........   Mmulatta
 390216: ......---------------........G....................   Panubis
 419145: ......GTGTATTCGGTTGCA........G....................   Cjacchus
1061697: ....T.GTGTATTTGGTTGCA........G..T...............T.   Sboliviensis

               77932     77942     77952     77962     77972
                   :         :         :         :         :
  77923: GCAAATTCCCCACCTTTGGGATCTTCCCTGCACGGATCATGTAGGAAGTG   Hsapiens
 397671: .................................A................   Ptroglodytes
 415108: ..................................................   Ggorilla
 452851: ..............................A...................   Pabelli
 441705: .....G........A...........................G.......   Mmulatta
 390251: .....G........A...........................G.......   Panubis
 419195: ..C.CG........A.G.A.............G.........G....A..   Cjacchus
1061747: ..C.TG..G.....A.G.A.............G.A.......G....A..   Sboliviensis

                          Simple
           ----------------------------->
               77982     77992     78002     78012     78022
                   :         :         :         :         :
  77973: GTGGGATGGGCTGGGCTGGGCTGGGCTGGATCAGATCGATTTTGTGACAT   Hsapiens
 397721: .............T....................................   Ptroglodytes
 415158: ................C.A...............................   Ggorilla
 452901: ..............................................G...   Pabelli
 441755: .C...-----........................................   Mmulatta
 390301: .C...-----........................................   Panubis
 419245: .....----------...A...A.......C....CT..C..........   Cjacchus
1061797: .....----------...AA................TA.C..........   Sboliviensis

               78032     78042     78052     78062     78072
                   :         :         :         :         :
  78023: GCAGGCTTTATAAACCCTCTTTTGCCACAGTTTCAGAGGGGTCCTAATGC   Hsapiens
 397771: .......................................A..........   Ptroglodytes
 415208: ................T.................................   Ggorilla
 452951: .......................................A..........   Pabelli
 441800: ..........................T.............A.........   Mmulatta
 390346: ..........................T.............A.........   Panubis
 419285: ....A....G....T.....G....T...................G....   Cjacchus
1061837: ....A....G..........G........................G....   Sboliviensis
```

```
              78082     78092     78102     78112     78122
                  :         :         :         :         :
  78073: TGCTGTTATAAAAATAACTAAATAATAAAAATTAAAATTGAGAAGTCCAT   Hsapiens
 397821: ..........................C.......................   Ptroglodytes
 415258: ..................................................   Ggorilla
 453001: ..................................................   Pabelli
 441850: ................................................G.   Mmulatta
 390396: ................................................G.   Panubis
 419335: .AT.AC.........C.G.....GG.........................   Cjacchus
1061887: ..T..C...........G..................CC.....G..-...   Sboliviensis

              78132     78142     78152     78162     78172
                  :         :         :         :         :
  78123: GTTACTCGTTTATTATAGAAAATTCTGAGTTTCCATCTGCAATTCAGATT   Hsapiens
 397871: .......A......................................T...   Ptroglodytes
 415308: .......A..........................................   Ggorilla
 453051: .......A..........G...............................   Pabelli
 441900: A......A.................................G......G.   Mmulatta
 390446: A......A.................................G........   Panubis
 419385: ....T..A..............G...A.........T............G   Cjacchus
1061936: .......A............................T..........G.G   Sboliviensis

              78182     78192     78202     78212     78222
                  :         :         :         :         :
  78173: TTTAATATAGTGTCTTCCTCAACTGTCTTGAAAGCTGAGTGATTTCTTCC   Hsapiens
 397921: .........................................C........   Ptroglodytes
 415358: .........A...............................C........   Ggorilla
 453101: .........................................C........   Pabelli
 441950: ...................A.........-------------------..   Mmulatta
 390496: ...................A.........-------------------..   Panubis
 419435: ...........C....T........................C........   Cjacchus
1061986: ...............C.........................C........   Sboliviensis

              78232     78242     78252     78262     78272
                  :         :         :         :         :
  78223: ACAGCTGTCCAATCTCTGCGGCTAGTTACCCTTTGTCCTAGGAAACCATT   Hsapiens
 397971: ................................................C.   Ptroglodytes
 415408: ..................T...............................   Ggorilla
 453151: ..................T...........A...............TG..   Pabelli
 441981: ...........G.....AT............................G..   Mmulatta
 390527: ...........G.....A.............................G..   Panubis
 419485: .................A.T..CG..........................   Cjacchus
1062036: .........T.........T...................G..........   Sboliviensis

              78282     78292     78302     78312     78322
                  :         :         :         :         :
  78273: CTCTTGTCACCTACAGACCCTGAAGCTTCTGTCCGGAAAACATAGTTTCT   Hsapiens
 398021: ..................................................   Ptroglodytes
 415458: ..................................................   Ggorilla
 453201: .................................T................   Pabelli
 442031: .................................T..G.....--......   Mmulatta
 390577: .........T.......................T..G.....--......   Panubis
 419535: ..........G......................TA.G...TG..A.....   Cjacchus
1062086: ................G................TA.G.......A.....   Sboliviensis
```

```
                 78332     78342     78352     78362     78372
                     :         :         :         :         :
  78323: CACTCAGCTGCCTTTTTGCCTATATATTGGCTAGAGCAGCAGGAGATTGA  Hsapiens
 398071: ..................................................  Ptroglodytes
 415508: ................G.-....G..........................  Ggorilla
 453251: ..................................................  Pabelli
 442079: .....CA...........................................  Mmulatta
 390625: .....CA...........................................  Panubis
 419585: ..T....................G...........A.....A........  Cjacchus
1062136: ..T...................C............A.....A.......C  Sboliviensis

                 78382     78392     78401     78411     78421
                     :         :         :         :         :
  78373: GTCTGTAATGAAATGGAAAG-CCCATGTGCCCAAATTAGGCATCTCTGAT  Hsapiens
 398121: ....................-.............................  Ptroglodytes
 415557: ....................-.............................  Ggorilla
 453301: ....................-.............................  Pabelli
 442129: .......G............-.............................  Mmulatta
 390675: ....................-.............................  Panubis
 419635: .C.A..........A....ATT.................ATT........  Cjacchus
1062186: .C..A........C.....ATT..................TT........  Sboliviensis

                 78431     78440     78450     78460     78470
                     :         :         :         :         :
  78422: AAATGGAACCC-CATTAGACTCCTCCTCTAATGGCCACAGATCCTGGGTT  Hsapiens
 398170: ..-........-......................................  Ptroglodytes
 415606: ...........-......................................  Ggorilla
 453350: ........A..-......................................  Pabelli
 442178: ...........-......................................  Mmulatta
 390724: ..G........-...............G......................  Panubis
 419685: ...........A.........T.......C............T.......  Cjacchus
1062236: ...........T.........T.......G............T.......  Sboliviensis

                 78480     78490     78500     78510     78520
                     :         :         :         :         :
  78471: TTAAAACAGTATTGCTGGTTTGACTTACTAAGGCTGATCAGCTTCCTGCA  Hsapiens
 398218: ..........C......................................G  Ptroglodytes
 415655: ..........C.......................................  Ggorilla
 453399: ..........C.................C.....C.............GG  Pabelli
 442227: ..........C.................C...................GG  Mmulatta
 390773: ..........C.................C................T..GG  Panubis
 419735: ..........C...........G.........C...........T...GG  Cjacchus
1062286: ..........C...........G.........C...............GG  Sboliviensis

                 78530     78540     78550     78560     78570
                     :         :         :         :         :
  78521: TTTCTCAATCTTACAAAAAATCACCACCCCTCCACCCTAGCCTGTCACCA  Hsapiens
 398268: ............G...............................G.....  Ptroglodytes
 415705: ............................................G.....  Ggorilla
 453449: ...........CG..............T................G.....  Pabelli
 442277: ............G...............................G.....  Mmulatta
 390823: ............G...............................G.....  Panubis
 419785: .........G.CG......C.T...............C......G.....  Cjacchus
1062336: .........G.CG.....C..................C......G.....  Sboliviensis
```

```
              78580     78590     78600     78610     78620
                  :         :         :         :         :
  78571: CCCTCTGTGTTCCCATGCTCCTTAAAGACTGCATTCCACAGTGCTTGCGT  Hsapiens
 398318: ..T........................................T......  Ptroglodytes
 415755: ...........................................T....A.  Ggorilla
 453499: ...........................................T......  Pabelli
 442327: ..-........................................T......  Mmulatta
 390873: ...........................................T......  Panubis
 419835: ...................T.......................T...T..  Cjacchus
1062386: .....C.............T.......................T....A.  Sboliviensis

              78630     78640     78650     78660     78670
                  :         :         :         :         :
  78621: TAATGGGGTGGAGAGGCAAGGTTCAGCTTCTCAAAGAAAGGCTTACTCAG  Hsapiens
 398368: ...........................C........G.............  Ptroglodytes
 415805: ..............C...................................  Ggorilla
 453549: ...........................C......................  Pabelli
 442376: ...........................C......................  Mmulatta
 390923: ...........................C......................  Panubis
 419885: ...C............T...A......C..................C...  Cjacchus
1062436: ...C...........A.........A.C..................C...  Sboliviensis

              78680     78690     78700     78710     78720
                  :         :         :         :         :
  78671: AGAATACACAGATCAACTGTTGGGAGTTCCTATCCATTTGCCTTGTTGTT  Hsapiens
 398418: ..................................................  Ptroglodytes
 415855: ..................................................  Ggorilla
 453599: ..................................................  Pabelli
 442426: ..................................................  Mmulatta
 390973: ..................................................  Panubis
 419935: ....C.............................................  Cjacchus
1062486: ....C..................A......................G...  Sboliviensis

                        78734     78744     78754     78762
                            :         :         :         :
  78721: TTTA------CAACCTGTGTACTGAATAATGCTGAGAACAGTAA--ATAG  Hsapiens
 398468: ....------..................................--....  Ptroglodytes
 415905: ....------..................................--....  Ggorilla
 453649: ....------T.................................--....  Pabelli
 442476: ....------.......C..........................AT....  Mmulatta
 391023: ....------.......CA.........................--....  Panubis
 419985: ....GATCGC...............G...........G......--....  Cjacchus
1062536: ....GATTGC......A........G...........G......--....  Sboliviensis

              78772     78782     78792
                  :         :         :
  78763: TGAACATGATATGTTGCTGTTATCCGAGGTT-------------------  Hsapiens
 398510: ........................T......-------------------  Ptroglodytes
 415947: ...............................-------------------  Ggorilla
 453691: ........................T......NNNNNNNNNNGTAGGGAGG  Pabelli
 442520: ..................A.....T......-------------------  Mmulatta
 391065: ..................A.....T......-------------------  Panubis
 420033: A.........G..C........C.T......-------------------  Cjacchus
1062584: .............C..........T......-------------------  Sboliviensis
```

```
                   78800     78810               78821
                           :         :                   :
  78794: ---TGTGTGTACAATAGAGTCTTATCT-G--------AGAATTTA-TT--  Hsapiens
 398541: ---...............A........G.--------........-..--  Ptroglodytes
 415978: ---........................-.--------........-..--  Ggorilla
 453741: TGG.A.CCAG.TC.C.....AAATG..G.GTCACACA.C......A..AG  Pabelli
 442551: ---..............--........G.--------.....G..A..--  Mmulatta
 391096: ---........................G.--------.....G..A..--  Panubis
 420064: ---..................C...G.G.--------.......T-..    Cjacchus
1062615: ---..................C...ACG.--------.......T-..--  Sboliviensis

                         78834     78844     78854     78863
                             :         :         :         :
  78829: --------------GAGAGAAAAAGAAAAGAGAGAGAAAG-AAGAAAGAA  Hsapiens
 398577: --------------..........................-.........  Ptroglodytes
 416013: --------------..........................-.........  Ggorilla
 120721:                        ...TC.A...T...TCA-.....T..T  Pabelli
 453791: TGTAGTCTTGTTTA......C..                             Pabelli
 442586: --------------.......................G..-..AG.....  Mmulatta
 391133: --------------.                                     Panubis
 391334:                ..........A.G..AG..A....GA-...G.....  Panubis
 103345:               ..A....G..AG...........G..A.........  Cjacchus
  84097:                    .....A....A.A.A.A...A-..A...     Sboliviensis
  88042:                                                 .G  Sboliviensis
1062651: --------------..A..                                 Sboliviensis

              78873               78892     78899
                  :                   :         :
  78864: AAAAGGGAAGAGAGAAGGA-AAAGAAAGAATGAAAG---AAAGAAAG---  Hsapiens
 398612: ......A............-.......-----------------------  Ptroglodytes
 416048: ...................-................--------------  Ggorilla
 120747: C........A.AG...                                    Pabelli
 453876:                 AAT-.T.....T.C.A...ATTT.T.T..TATAC  Pabelli
 442621: .....A..GA..GA..A..-..GA......A.....---........AAA  Mmulatta
 391368: .G..A.AG.A.....GA..G..........A....A---.G....G.AAG  Panubis
 103381: .G..A.A..A...A..AA.-..........A.....---.......----  Cjacchus
  88044: ....A.A..AGA.A..AA.-.......A..                      Sboliviensis

                                                      78912
                                                          :
  78907: --------------------------------------------GAAAGA  Hsapiens
 398638: --------------------------------------------......  Ptroglodytes
 416083: --------------------------------------------A..G..  Ggorilla
 453909: CAA-----------------------------------------AG...G  Pabelli
 144240:                                                ...  Nleucogenys
 442667: GAAAGGAAAGAAGAAAGAAAGAAAGAAAGAAAGAAAGAAAGAAA......  Mmulatta
 391415: GAAGGA--------------------------------------AGG..G  Panubis
 103423: --------------------------------------------......  Cjacchus
1062719:                                             A.G.A.  Sboliviensis
```

```
              78922     78929               78945     78955
  78913: AAGAGAGAGAGAAA---AGGAAGAAA----GAAAGAGAGAGAAAGAGAGA  Hsapiens
 398644: ....A.C.......---.A.......----..........--........  Ptroglodytes
 416089: ..............---.........----....................  Ggorilla
 127575:                                .G...AG....GG....A.  Pabelli
 453918: .....T..A..G.G-CT..A.GTC..TGTG.                     Pabelli
 144243: G.AG.....G..G.---....GAC..----.G..AT....TGGG.T.G..  Nleucogenys
 152035:                                                  .  Mmulatta
 442717: ....A...A.....GAA..A......----......A...A.G.A...A   Mmulatta
 391427: G.A.....A.....---G.A.G...G----...G..AG..AGG.AG..AG  Panubis
 103429: ....A...A.....                                      Cjacchus
 105315:                  .AA..A...----A......G..AG.G.G.G.   Cjacchus
 126216:                                                  .  Cjacchus
1062725: ....A...A.....---..A......----....A...A.......A...  Sboliviensis

              78965     78975     78985     78992
  78956: AAGAAAAAGAAAGAAGAAAGAAAGAAAAAGAA---GGAGGGAAGGAAGT-  Hsapiens
 398685: ...........GA...................---.............G-  Ptroglodytes
 416132: ................................---.............G-  Ggorilla
 127594: G...C..G....TGG..TG.GGT.GG.C...G---.TG.ATGG...T.G-  Pabelli
 144286: C...                                                Nleucogenys
 152036: ..A.....A...C..A.C.A...C.....C..                    Mmulatta
 442795:                                 GAAA..AA...A...AGA  Mmulatta
 391470: G.AGG..G...G....G...GG...G......---A..AA...A...AGG  Panubis
 126217: ..A.....A...A..A...A..G..                           Cjacchus
 131041:                                     A..A......GAG-  Cjacchus
1062768: .......GA..GA........G.A.G......---AA..AA..A...A    Sboliviensis

              79010     79019               79037     79047
  79002: -AGGCAGGGAGAGGAGGA-AGGAAGGGA--GAGAGGGAGGGAGGAAGGGA  Hsapiens
 398731: -.................-.........--....................  Ptroglodytes
 416178: -.................-.........--....................  Ggorilla
 127640: ------A.....T.....-..AC..A..TG....A...ACAGA..CA.A.  Pabelli
 442813: A..AA.A...........-.......A.--.........AA...G..A..  Mmulatta
 391517: A...A.............-.......A.--..........A...G..A..  Panubis
 131054: -GAAG.........                                      Cjacchus
 421251:               ...G-...G.....--.G..................  Cjacchus
 137579:         .....A....G......A.G--.........A..AACC.A..  Sboliviensis

              79057     79067     79077     79087
  79048: GGAAGGCAGGCAGGAAGGCAGGCAGGAAGGCAGGCAGGAAGAC-------  Hsapiens
 398777: .................A........C....-------------------  Ptroglodytes
 416224: .....................                               Ggorilla
 127683: ..G.A.AG..G..AG.                                    Pabelli
 442860: ..G...G...G...........A...C.......A---------------  Mmulatta
 391564: ..G.......G...G...A......................G.AGGCAGG  Panubis
 421284: A.G.AAAGAAA.......A...A.......A...A.....----------  Cjacchus
 137619: CAG..A..A.G.AATG..AT..------......GGAC.G----------  Sboliviensis

                        79097     79107     79117     79127
  79091: -------------AGGCAGGCAGGCAGGAAGGAAGGAAGGGAGGGAGGGA  Hsapiens
 398808: -----------------------------.....................  Ptroglodytes
 442895: -------------...........A...........G.....AA......  Mmulatta
 391614: AAGACAGGCAGGA..........AA...........G.............  Panubis
 421324: -------------.AAA..AA.TAA..A...A...AG.AA..AA..AA..  Cjacchus
 137653: -------------....GAATG..G.A.G.....ATG.......C..A..  Sboliviensis
```

```
              79137     79146     79156     79166     79176
                  :         :         :         :         :
  79128: GGGAGGGAAGGAG-GACTTTTTCAGTGCTGAGATCCAAAAGGGAGAATCT  Hsapiens
 398829: .............-....................................  Ptroglodytes
 442932: .A...........-.......C..A.............G...A.......  Mmulatta
 391664: .A...........-.......C..A.............G...A.......  Panubis
 421361: AA...A..G...AA.......C................G...........  Cjacchus
 137690: T.A..A....                                          Sboliviensis
1062891:           ..CA.......C..A..T..........G..T........  Sboliviensis

              79186     79196     79205     79215     79225
                  :         :         :         :         :
  79177: GGAACAGGGAGGGAAAAAAAAT-GTTGTTGGTTTTTGTTAGTTCTTATTT  Hsapiens
 398878: ............A........AT.................T.........  Ptroglodytes
 442981: .....................----....A..........T........C  Mmulatta
 391713: .............G.......----.......A.......T.........  Panubis
 421411: ....................C.---.........C.....C.........  Cjacchus
1062931: ............A.......C.---.........C.....C.........  Sboliviensis

              79235     79245     79255     79265     79275
                  :         :         :         :         :
  79226: TGTTGGGATAGGGAGGTGGTATCCAGATCACAGAGTAAATGCTGGGTCAC  Hsapiens
 398928: ..G....G.....................................A....  Ptroglodytes
 453733:         ..........................................  Pabelli
 443027: ..G....G...T..........-.................A......G..  Mmulatta
 391759: ..G....G...T...................................G..  Panubis
 421458: ..G....G.......T...C..T...........................  Cjacchus
1062978: ..G....GC.....A.......T...........................  Sboliviensis

              79285     79295     79305     79315     79325
                  :         :         :         :         :
  79276: ACAACAATTTAATTAGTGTAATCTTGTTTAGAGAGATAAATATAAAATAT  Hsapiens
 398978: ....................G.............................  Ptroglodytes
 453775: ....................G...............C.............  Pabelli
 443076: .....T...-----.....TC.............................  Mmulatta
 391809: .....T...-----.....TC...............G.............  Panubis
 421508: ..G-...........AC...T..G................C.C.......  Cjacchus
1063028: ...-...........AC...T..A..........................  Sboliviensis

              79335     79344     79354     79364     79374
                  :         :         :         :         :
  79326: TTAACAAATCCACTCAT-AAAAATAATTCAAAACAGAGTACTTGATTTAA  Hsapiens
 399028: .................-................................  Ptroglodytes
 453825: .................-................................  Pabelli
 443121: ..............T..-................................  Mmulatta
 391854: ..............T..-................................  Panubis
 421557: .................-................................  Cjacchus
1063077: .................A...........G....................  Sboliviensis

              79384     79394     79404     79414     79424
                  :         :         :         :         :
  79375: TAAACATAGAAATACTAAAAATGTATATAATATACCAAAGAAGGAAGAGT  Hsapiens
 399077: ......C...........................................  Ptroglodytes
 453874: ....T.................T...........................  Pabelli
 443170: ....T.............................................  Mmulatta
 391903: ....T.............................................  Panubis
 421606: ....T.................T.C.G..GA............A......  Cjacchus
1063127: .----.................T.C.G..G.............A......  Sboliviensis
```

```
              79434     79444     79454     79464     79474
                  :         :         :         :         :
   79425: GAAAGGAGCTAGAAGTCATTGTGGAGGGATCAAAATGGAGGCATAAAGAG  Hsapiens
  399127: ..................A.........................G.....  Ptroglodytes
  453924: ..................A.................A.............  Pabelli
  357743:                           ........................  Nleucogenys
  443220: ..............A...A...............................  Mmulatta
  391953: ..............A...A...............................  Panubis
  421656: .......A..........A....A..A.............-.........  Cjacchus
 1063173: .......A..........A....A..A.............-T........  Sboliviensis

                        79484     79494     79504     79514
                            :         :         :         :
   79475: ATGGGCA----------GTGAGGGAGGAGAAGGACGAAGGCAGAAGAAAG  Hsapiens
  399177: .......----------.................................  Ptroglodytes
  453974: ..A....----------..................A..............  Pabelli
  357767: .......----------.................................  Nleucogenys
  443270: .......----------.G.........................G.....  Mmulatta
  392003: .......----------.G.........................G.....  Panubis
  421705: .......TGGGAGTCTGAG...............T.........G.....  Cjacchus
 1063222: ......TTGGGAGT---C.....AG.........T......G..G.....  Sboliviensis

              79524     79534     79544     79554     79564
                  :         :         :         :         :
   79515: GAAGATGCCAGAGCTAACGGGAAAGAAAAAGTGTGTCAAACAACAGGCTC  Hsapiens
  399217: ..................................................  Ptroglodytes
  454014: ..................................................  Pabelli
  357807: .................T..................G.............  Nleucogenys
  443310: ........A.........................................  Mmulatta
  392043: ........A..................................G......  Panubis
  421755: ........A........T...........C...........C........  Cjacchus
 1063269: ........A....................C.C.........C........  Sboliviensis

              79574     79584     79594     79604     79614
                  :         :         :         :         :
   79565: CAGTAGGAGCAGGGTTGTGGGAGATCAAACTTGTCCTCCATGATTGATTT  Hsapiens
  399267: ..............................A...................  Ptroglodytes
  454064: ..A...........................A...................  Pabelli
  357857: ........A.....................A..G.....G..........  Nleucogenys
  443360: ...C..A....................GG.A.........C.........  Mmulatta
  392093: ...C..C.....................G.A...................  Panubis
  421805: ...C..........................G.A.T......C........  Cjacchus
 1063319: ...C....................G.....A.A.T...............  Sboliviensis

                                                  MIR
                                            -------------->
              79624     79631     79641     79651     79661
                  :         :         :         :         :
   79615: CATCGAGGCCAGACCA---CTAGTCCTGTTTTCCAGGCAATCTTGGGAAA  Hsapiens
  399317: ................---...............G...............  Ptroglodytes
  454114: ................---...............................  Pabelli
  357907: ....C.....G.....---.........G.....G...............  Nleucogenys
  443410: ....A...........---..............TG......T.C......  Mmulatta
  392143: .............TG.TGC.C............TG......T.G......  Panubis
  421855: ...T...A......T.---..............TG...............  Cjacchus
 1063369: ...T..........T.---.C............TG...............  Sboliviensis
```

```
                                MIR
         ------------------------------------------------->
              79670     79680     79690     79700     79710
                  :         :         :         :         :
   79662: ATCCT-CTGGCCTCATATTTTTCAGCTGCAAATCAAGGCAGTTGTATCTA  Hsapiens
  399364: .....C............................................  Ptroglodytes
  454161: .....C...........................T................  Pabelli
  357954: .....C.C......................G............C.....  Nleucogenys
  443457: .....C............................................  Mmulatta
  392193: .....C............................................  Panubis
  421902: ..A..G........G............A.....T..............AG  Cjacchus
 1063416: .C...C.C......C...............................C.AG  Sboliviensis

                    MIR
         --------------------------->
              79720     79730     79740     79750     79760
                  :         :         :         :         :
   79711: ATGCTTTGCTGGAGTCACTTCTCTGACCTGGTCTTGCCCCATCTCTGAGG  Hsapiens
  399414: ..................................................  Ptroglodytes
  454211: ...A..............................................  Pabelli
  358004: ...A..............................................  Nleucogenys
  443507: ...A........................................G.....  Mmulatta
  392243: ...A........................................G.....  Panubis
  421952: ..---.......G....T..T..C.T................T.......  Cjacchus
 1063466: ..---......AGA...T..T..CAT..........T.............  Sboliviensis

              79770     79780     79790     79800     79810
                  :         :         :         :         :
   79761: CCTAAAGGTGGCCCTGTGAGTGGCCAAAGTTCAGCAGACCCATGAAAGCA  Hsapiens
  399464: ..................................................  Ptroglodytes
  454261: ...........T......................................  Pabelli
  358054: ..................................................  Nleucogenys
  443557: ...............................T.............G....  Mmulatta
  392293: ...............................T.............G....  Panubis
  421999: ...G......................C.....................G.  Cjacchus
 1063513: T..G.....................GC..C..................GC  Sboliviensis

              79820     79830     79840     79850     79860
                  :         :         :         :         :
   79811: CTTATACTGCTGTTTGTTCTGCTAAATCATTAATTAGCTTGTATTTTTTT  Hsapiens
  399514: ......................G...........................  Ptroglodytes
  454311: ...........A......................................  Pabelli
  358104: ..................................................  Nleucogenys
  443607: ........................T..A............---.......  Mmulatta
  392343: ........................T..A............T--.......  Panubis
  422049: ..................................G.C......-......  Cjacchus
 1063563: ..................................G.C.............  Sboliviensis

              79870     79880               79895     79905
                  :         :                   :         :
   79861: CTCCCTCAAAGTATGTTTTGTAATTTA-----CGCTTGCTGCTTTTTCTT  Hsapiens
  399564: ...........................-----.A................  Ptroglodytes
  454361: ........C..................-----..................  Pabelli
  358154: ..............A............-----..................  Nleucogenys
  443654: ..........A...A.G..........-----..............AT..  Mmulatta
  392391: ..........A...A............-----..............AT..  Panubis
  422098: .A........C.G.A............ATTTA.A................  Cjacchus
 1063613: .A..........G.A............ATTTA.A............C...  Sboliviensis
```

```
               79915     79925     79935     79945     79955
   79906: CTGAGGTCCTTTTTGGGTCCAAGAGTCTATGATTCTGGAACCAACAGCTA  Hsapiens
  399609: ..................................................  Ptroglodytes
  454406: ...................A...............C..............  Pabelli
  358199: ...G........A..............A......................  Nleucogenys
  443699: ..........................T.......................  Mmulatta
  392436: .....................G....T.......................  Panubis
  422148: .......G.....G..T...............................A.  Cjacchus
 1063663: ................T.................................  Sboliviensis

               79965     79975     79985     79995     80005
   79956: TCTGATTACCTGATTCTCACAGCTTTTAATGTCAGCCCATAGACTCCAGG  Hsapiens
  399659: ............C.....................................  Ptroglodytes
  454456: ............C....T................................  Pabelli
  358249: ..C.........C.....................................  Nleucogenys
  443749: ......C.-...C.....G...............................  Mmulatta
  392486: ......C.....C............C........................  Panubis
  422198: .T........A.C..............G......AA..........T...  Cjacchus
 1063713: .T....G.....C...G..................G.......G..T...  Sboliviensis

               80015     80025     80035     80045     80055
   80006: AAATGAGATACTGTGCTATTAAGCAAGCAAGAGAGTCTATAAAGCATTGA  Hsapiens
  399709: ......................T.................G.........  Ptroglodytes
  454506: ..................................................  Pabelli
  358299: ..................................................  Nleucogenys
  443798: ........C...................--...................  Mmulatta
  392536: ........C...................--...................  Panubis
  422248: ..G...................C.CG..................C...  Cjacchus
 1063763: .....C................C.C.....................C...  Sboliviensis

               80065     80075     80085     80095     80105
   80056: ATCATGAGATTCTGTAACTGACCCAGGTACAAATGCCAGAAGACTGGTCT  Hsapiens
  399759: ...................................T..............  Ptroglodytes
  454556: ....................T..............T..............  Pabelli
  358349: .....A..............T.....A........T..............  Nleucogenys
  443846: ..............C.....T..............T..............  Mmulatta
  392584: ..............C.....T..............T..............  Panubis
  422298: ............CA......T..............T.......-------  Cjacchus
 1063813: ............CA......T..............TTG.....-------  Sboliviensis

               80115     80125     80135     80143     80153
   80106: TTTGCCTGTCTGTTTTACAGGCAAGAAACAAA-CAG-AATTTCTTCTTCC  Hsapiens
  399809: ................................-...-.............  Ptroglodytes
  454606: ...T...........C.....G..........-...-.............  Pabelli
  358399: ...............C.....G..........-...-.............  Nleucogenys
  443896: ......C........C.....G..........-...A.............  Mmulatta
  392634: ......C........C.....G..........-...A.............  Panubis
  422341: ---------......C.....G..........C...-.............  Cjacchus
 1063856: ---------......C.....G..........CT..-.............  Sboliviensis
```

```
                        80170     80180     80190     80200
                            :         :         :         :
  80154: AATTACAT---AACACAATGAATAGAGACAAGATGACACCATTTGTAGTT  Hsapiens
 399857: ........---.......................................  Ptroglodytes
 454654: ........---.G.........................G...........  Pabelli
 358447: ........AGC.G...................G.....G...........  Nleucogenys
 443945: ........---.G.........................G...........  Mmulatta
 392683: .G......---.G.....C...................G...........  Panubis
 422381: .G......---.G....G...G................G.....T.G...  Cjacchus
1063896: .G......---.GT........................G.C...TA....  Sboliviensis

              80210     80220     80230     80240     80250
                  :         :         :         :         :
  80201: CATATTTGCTCAACTTATTAAAGAACAAAAGCTCACGGAATCACTGGAAC  Hsapiens
 399904: ..................................................  Ptroglodytes
 454701: ....................................A...........GT  Pabelli
 358497: ..C.................................A.............  Nleucogenys
 443992: .............T......................A.............  Mmulatta
 392730: .............T......................A.............  Panubis
 422428: .............T........A...G........--..G..........  Cjacchus
1063943: T............T...........A..........A..G..........  Sboliviensis

              80260     80270     80280     80290     80300
                  :         :         :         :         :
  80251: TTGCCAATGAAGGCAAAATAGTGCAAAGACTGCCAAGGTTTTTAGATTTA  Hsapiens
 399954: .....C............................................  Ptroglodytes
 454751: .....C...G........................................  Pabelli
 358547: .....C...G.............TG.........G...............  Nleucogenys
 444042: .....C...G..........A.....................G.......  Mmulatta
 392780: .....C...G..........A.....................G.......  Panubis
 422476: ....TCGC.GG...C.......A...................G.......  Cjacchus
1063993: ....TC.C.GG...C..G....C..........TG.......G.......  Sboliviensis

              80310     80320     80330     80340     80350
                  :         :         :         :         :
  80301: TTGTGTTTAAATAATGGTCTCAGATTTCCTGGGCACCTTCACACAGAATC  Hsapiens
 400004: ..................................................  Ptroglodytes
 454801: ..................................................  Pabelli
 358597: ..................................................  Nleucogenys
 444092: ........C.........................................  Mmulatta
 392830: ........C.........................................  Panubis
 422526: A..........CG...A....................C..T.........  Cjacchus
1064043: A...........G...A..............A..................  Sboliviensis

              80360     80370     80380     80390     80400
                  :         :         :         :         :
  80351: AATGTTGATATATTTGCACATTGACTAAACACATTTGATGTCTTGGCCTC  Hsapiens
 400054: ..................................................  Ptroglodytes
 454851: .G......CG........................................  Pabelli
 358647: ........................T.........................  Nleucogenys
 444142: .......--......................T.C....C...........  Mmulatta
 392880: .......--......................T.C....C...........  Panubis
 422576: .......--......................T..................  Cjacchus
1064093: .T.....--..........G...........T..................  Sboliviensis
```

```
                 80410     80420     80430     80440     80450
  80401: TCAAGTTACTTATTTACTTATTTAATGTGTATTCATGGGCAGATATTACC  Hsapiens
 400104: ..................................................  Ptroglodytes
 454901: ....T...............................T.............  Pabelli
 358697: .................................-..T........C...A  Nleucogenys
 444190: .....--------....................T..T..G..........  Mmulatta
 392928: .....--------....................T.....G..........  Panubis
 422624: ....T.......C.C.............T....T..T.............  Cjacchus
 139519:      ...T.......T.........                          Sboliviensis
1064141: ..G.T                                               Sboliviensis
1064167:                           ..T....T..T.........G.T.  Sboliviensis

                 80460     80470     80480     80490     80500
  80451: AAATGAGTGGCTGAGCACCATGAGCATGGACTCTGGTCTCATTTTACTTT  Hsapiens
 400154: ..................................................  Ptroglodytes
 454951: ..............................A...................  Pabelli
 358746: ...........................A..A...................  Nleucogenys
 444232: ....A..........T..............A.....C.............  Mmulatta
 392970: ....A.........................A.....C.............  Panubis
 422674: ...C...........T..G......C....A...................  Cjacchus
1064191: G................TG...........A.............C.....  Sboliviensis

                 80510     80516     80526     80536     80546
  80501: TCATCTTTTACATAC----CCTAGGACTATACTGGTATAGAGTAGTGTAC  Hsapiens
 400204: ...............----...............................  Ptroglodytes
 455001: ...............----...............................  Pabelli
 358796: ...............----...............................  Nleucogenys
 444282: .T.............----.........G...........G.........  Mmulatta
 393020: .T.............----.........G...........G.........  Panubis
 422724: .T..T........G.CCTA................--------.C.A...  Cjacchus
1064241: .T...........G.CCTA.........G........A.....C......  Sboliviensis

                                        LINE1
                            ------------------------------>
                 80556     80566     80576     80586     80596
  80547: AAAACAGACTTGATTAATTAGTGATATTTTAATACATGTGTACAATGTGT  Hsapiens
 400250: ..................................................  Ptroglodytes
 455047: ..............C....T.........................C....  Pabelli
 358842: ..................................................  Nleucogenys
 444328: ..........................C....................C..  Mmulatta
 393066: ..........................C.......................  Panubis
 422766: ----------.......A..A.............G...............  Cjacchus
1064291: .................A..ACA...........G........C......  Sboliviensis

                               LINE1
         ------------------------------------------------->
                 80606     80616     80626     80636     80646
  80597: AGTGATCCATTCTGGCTAACATTCGTCACCTCAAGCCTTGGTCATTTCTG  Hsapiens
 400300: .A......................A.........................  Ptroglodytes
 455097: .A.....................................T.........T  Pabelli
 358892: .A...A.................................T.........T  Nleucogenys
 444378: .A...................G..........TGA....T.........T  Mmulatta
 393116: .A.....T................................CGA....T.........T  Panubis
 422806: .A....A.G..........------------G.G.....TA........T  Cjacchus
1064341: .A....A........T.G.------------........TA........T  Sboliviensis
```

```
                               LINE1
         ---------------------------------------------------->
               80656     80666     80676     80686     80696
  80647: TGTGTTAGGAACATTCAAAATCCTCTCCTCTAGCTTTTTAAAAATATGCA  Hsapiens
 400350: .......................................T..........  Ptroglodytes
 455147: --................................................  Pabelli
 358942: .........................................C........  Nleucogenys
 444428: ......GA..........................................  Mmulatta
 393166: ......GA..........................................  Panubis
 422844: ......G......C....................................  Cjacchus
1064379: ......G.................T......C..................  Sboliviensis

                               LINE1
         ---------------------------------------------------->
               80706     80716     80726     80736     80746
  80697: CTAAATTATTGGTGACCATATTCACCCTACAGTGCTATAGAACACTAGAC  Hsapiens
 400400: .............A..................C.................  Ptroglodytes
 455195: ..................................................  Pabelli
 358992: ...............A.................................G  Nleucogenys
 444478: ......G..................T..........C.............  Mmulatta
 393216: ......G..................T..........C.............  Panubis
 422894: ...................G....T........A................  Cjacchus
1064429: .................................A...............T  Sboliviensis

                               LINE1
         ---------------------------------------------------->
               80756     80766     80776     80786     80796
  80747: TTATTCTACCCATCTAGCTAATACTTTGTACCTGTTAACCAACCTCTCTC  Hsapiens
 400450: ................................................C.  Ptroglodytes
 455245: ...............G...G...T........................C.  Pabelli
 359042: ................A..G.-.T......................C.C.  Nleucogenys
 444528: ..C..........G.....G...T........................C.  Mmulatta
 393266: ..C.........C......G...T...................T....C.  Panubis
 422944: ..G....-...........G...T....C.--................C.  Cjacchus
1064479: ......C-...........G...T....C.T.................C.  Sboliviensis

                               LINE1
         ---------------------------------------------------->
               80806     80815     80825     80835     80845
  80797: TATCCTCCCCTACCTT-TATCCTTCTCAGCCTCTAATAACCACAATTCTA  Hsapiens
 400500: ................-.................................  Ptroglodytes
 455295: ............T...C..........CC.....................  Pabelli
 359091: ............T...C.................................  Nleucogenys
 444578: ......-.....T..CC...G.............................  Mmulatta
 393316: ............T..CC...........................G.....  Panubis
 422991: ...T........T..CC........C..A.T........T..........  Cjacchus
1064528: ...T.......CT..CC........C..A.TG..................  Sboliviensis

                               LINE1
         ---------------------------------------------------->
               80855     80865     80874     80884     80894
  80846: CTCTCTACTTCACTGACCTCCA-TTTTTTTTAGCTCTCACACACAAGTGA  Hsapiens
 400549: ......................-...........................  Ptroglodytes
 455345: ......................-...........................  Pabelli
 359141: ..........T...........T...........................  Nleucogenys
 444627: ......................--..........................  Mmulatta
 393366: ......................T...........................  Panubis
 423041: ..........T.....A.....--........T........T.AG.....  Cjacchus
1064578: ......................--........T........T.AG.....  Sboliviensis
```

```
                                    LINE1
          ------------------------------------------------->
                  80904     80914     80924     80934     80944
                      :         :         :         :         :
  80895: AAACATGCTGCATTTGTCTTTCTGTGCCTGACTTATTTCGCTTAACATAA  Hsapiens
 400598: ........G.........................................  Ptroglodytes
 455394: .......TG..............................A..........  Pabelli
 359191: .......TG.............................T...........  Nleucogenys
 444675: .......TG................A........T...............  Mmulatta
 393416: .......TG................A........T...............  Panubis
 423089: .......TG..............................A........G.  Cjacchus
1064626: .......TG..............................A..........  Sboliviensis

                                    LINE1
          ------------------------------------------------->
                  80954     80964     80974     80984     80994
                      :         :         :         :         :
  80945: CATCCTCCAGGCTCATCCATGTTGCTGTGAATAACAGGATTTTATTCTTT  Hsapiens
 400648: ...................C..............................  Ptroglodytes
 455444: .....C...........TG...............................  Pabelli
 359241: .G.....................T.....T................-...  Nleucogenys
 444725: ...........T......................................  Mmulatta
 393466: ...........T......................................  Panubis
 423139: TG.................CA...........G.TCA.....C....A..  Cjacchus
1064676: TG.................CA...........G.TCAT....C....A..  Sboliviensis

                                    LINE1
          ------------------------------------------------->
                  81004     81014     81024     81034     81044
                      :         :         :         :         :
  80995: TTTAAGGCTGAATAGTATTCCATTGTGCACAGTTACCACATTTTCTTTAT  Hsapiens
 400698: ..................................................  Ptroglodytes
 455494: .......T......C.....T......T...................C..  Pabelli
 359290: ...........................T......................  Nleucogenys
 444775: .........................A.T......................  Mmulatta
 393516: .........................A.TG.....................  Panubis
 423189: .......TG..G...............T.T..G............C....  Cjacchus
1064726: ...G...TG..................T.T..G.................  Sboliviensis

                                    LINE1
          ------------------------------------------------->
                  81054     81064     81074     81084     81094
                      :         :         :         :         :
  81045: CCATTCATCTGTTGATGGACGATTAGGGTGATTATACGTCTTAGCTATTG  Hsapiens
 400748: .....................................A............  Ptroglodytes
 455544: .....................................A........G...  Pabelli
 359340: .....................................A............  Nleucogenys
 444825: ..............G......................A............  Mmulatta
 393566: .....................................A............  Panubis
 423239: .............T.....G.......A...C..C..A..C.G....C..  Cjacchus
1064776: ...................G.......A...C.GC.T...C......C..  Sboliviensis

                                    LINE1
          ------------------------------------------------->
                  81104     81113     81123     81133     81143
                      :         :         :         :         :
  81095: TGAATAGTGCTGCAAT-AACATGGGAGTGTAGGTGTCTCTTTGATGCACT  Hsapiens
 400798: ................-.................................  Ptroglodytes
 455594: ........A.......-........G........A..........A....  Pabelli
 359390: ........A.......-........G........A...............  Nleucogenys
 444875: ................-....CT..T..A..-..A...............  Mmulatta
 393616: ............T..C-....CT..T........A...............  Panubis
 423289: ....C.....C.T...A....CA..G........A...T...........  Cjacchus
1064826: .......CA...T...A....CA..G........A...............  Sboliviensis
```

```
                LINE1
               81153     81163     81173     81183     81193
                   :         :         :         :         :
    81144: GATTTCCTTCTTTTGGACAAGTACTCATCAAAACAAAAATTTTAACTAAC  Hsapiens
   400847: ..................................................  Ptroglodytes
   455643: ..................................................  Pabelli
   359439: ..................................................  Nleucogenys
   444923: A.....................GT..........................  Mmulatta
   393665: A......................T..........................  Panubis
   423339: .........T..A...G......T.......C.TG..........T...A  Cjacchus
  1064876: .........T..A..A...T...T.........TG..........T...A  Sboliviensis

               81203     81213     81223     81233     81243
                   :         :         :         :         :
    81194: ACAAATGAAAAGAGTGTTACATAAGCCTTCCCTCCTGGCTTTTCTTGCAC  Hsapiens
   400897: ..................................................  Ptroglodytes
   455693: ...........A..........T...........................  Pabelli
   359489: ..................................................  Nleucogenys
   444973: .................................T................  Mmulatta
   393715: .............A...................T................  Panubis
   423389: ....G.....................G.......................  Cjacchus
  1064926: ....G......C..............G................T......  Sboliviensis

               81253     81263     81273     81283     81293
                   :         :         :         :         :
    81244: TCAAACCTGAATGCTTTCTTCATTTGGAAAACTTCAAAGGAGGTGACTCA  Hsapiens
   400947: ..................................................  Ptroglodytes
   455743: ......................................T...A.......  Pabelli
   359539: ..................................................  Nleucogenys
   445023: ...........................G.......T..............  Mmulatta
   393765: ...........................G.......T...........C..  Panubis
   423439: ............A....-....................A...........  Cjacchus
  1064976: .........C.......-................TGT.A...........  Sboliviensis

               81303     81313     81323     81333     81343
                   :         :         :         :         :
    81294: GAAGCTTCTTCTCATCCATCATTCAAACAATCAGGTATATTTCAGTATGT  Hsapiens
   400997: .................G................................  Ptroglodytes
   455793: ...A............TG................................  Pabelli
   359589: .................G.........G......................  Nleucogenys
   445073: .....C.T.........G----.....G.....A................  Mmulatta
   393815: .....C.T.........G.........G.G...A................  Panubis
   423488: .......T...C....TG.........G.................C.AA.  Cjacchus
  1065025: ...C...T...C..C..G.........G....................A.  Sboliviensis

               81353     81363     81373     81383     81393
                   :         :         :         :         :
    81344: TCTGATATTGCAAGCTGATCTTTTCTTAACTCAAGCTTTTGAATATTTTT  Hsapiens
   401047: ..................................................  Ptroglodytes
   455843: ..................................A...............  Pabelli
   359639: ..................................A...............  Nleucogenys
   445119: ........C...................GG....A..............G  Mmulatta
   393865: ............................GG....A..............G  Panubis
   423538: ....T........A.....A.........G....A......G.A.....G  Cjacchus
  1065075: ..................-----......G....A........A.....G  Sboliviensis
```

```
               81401     81411     81418     81428     81438
   81394: G-TTTAA-GACTGCTCTTCAG---AGAATATACTACCTCCACTTTCAATA  Hsapiens
  401097: .-.....-.............---..........................  Ptroglodytes
  455893: .-.....-.............---..........................  Pabelli
  359689: .-.....-.............---...........T..............  Nleucogenys
  445169: .-.....-.............---.........C................  Mmulatta
  393915: .-.....-.......T.....---.........C................  Panubis
  423588: .T.....-............TAGC..................C......C  Cjacchus
 1065120: .-.....C............TAGC................T.C......C  Sboliviensis

               81448     81458     81468     81478     81488
   81439: GCTGTATAAAGACAGGCTGACTCAGGATGGGAAGGACAAGTCCCAGGAAG  Hsapiens
  401142: ..................................................  Ptroglodytes
  455938: ............................C.....................  Pabelli
  359734: .................C..........C.............G.......  Nleucogenys
  445214: ...A......................................T.......  Mmulatta
  393960: ...A......................................T.......  Panubis
  423637: A..A.............A..........T....A..T........A....  Cjacchus
 1065169: ...A............TA......C..CT.......T.............  Sboliviensis

               81498     81508     81518
   81489: GATATTTTCTGTTGGATACAGATAAACTGTTGACTAAG------------  Hsapiens
  401192: ......................................TCTGGACTCATC  Ptroglodytes
  455988: ...............T......................TCTGGACTCATC  Pabelli
  359784: ..............................-.......TCTGGACTCATC  Nleucogenys
  445264: .T.........................G..G..T....TTTGGACTCATC  Mmulatta
  394010: .T.........................G..G..T....TTTGGACTCATC  Panubis
  423687: TT........A...A.......G...T......T....CCTGGACTCGTC  Cjacchus
 1065219: .T........AC..........G...T......T....TCTGGACTCATC  Sboliviensis

               81536     81546     81556     81566     81576
   81527: TCTGGACTAGACTGTCCATGCACTCTCAAGCACAGTTAAGACTCATGGGG  Hsapiens
  401242: ...............A..................................  Ptroglodytes
  456038: ..................................................  Pabelli
  359833: ...................T........................G.....  Nleucogenys
  445314: .........................C................AT......  Mmulatta
  394060: ................G........C................AT...T..  Panubis
  423737: .........A....G...........TG........G....TAT...A..  Cjacchus
 1065269: .........A....G...........TG.............TAT..A...  Sboliviensis

               81586     81596               81612     81622
   81577: CCATGTGCAGTGAGGCACCTTTGCCCTCT----CATCTGCTGCTGGTTCT  Hsapiens
  401292: .............................----.................  Ptroglodytes
  456088: .............................----..........C......  Pabelli
  359883: .............................----.................  Nleucogenys
  445364: .........A...................CATC..........C......  Mmulatta
  394110: .........A...................CATC..........CA.....  Panubis
  423787: .........A...A............A.-----..........CA.C...  Cjacchus
 1065319: .........A...A............AT.----..........CAC....  Sboliviensis
```

```
                  81632     81642     81652     81662     81672
                      :         :         :         :         :
  81623: CACACAGCTGAGGATTCGATAGGGTAAATTACCTACCGGCTCCCCCTCCT  Hsapiens
 401338: ..................................................  Ptroglodytes
 456134: .................A................................  Pabelli
 359929: .......AG........A................................  Nleucogenys
 445414: T......A......C..A..................TT...-........  Mmulatta
 394160: .......A......C..A..................TT...-G.......  Panubis
 423832: ..A....A.......G.AG.....G........CG.T.............  Cjacchus
1065365: .......A......G..AG..............CG.T........G...C  Sboliviensis

                  81682     81692     81702     81712     81722
                      :         :         :         :         :
  81673: GCTTGCCTTTCACCTTCAGTCTCCTCTTTGCTGGGCATCCATCACTGTGC  Hsapiens
 401388: ...C..............................................  Ptroglodytes
 456184: ...C..............................................  Pabelli
 359979: ...C..............................................  Nleucogenys
 445463: ...C........................CA....................  Mmulatta
 394209: ...C........................CA................A...  Panubis
 423882: ...CAG...........................A................  Cjacchus
1065415: ...CAG...........................A...........CC...  Sboliviensis

                  81732     81742     81752     81762     81772
                      :         :         :         :         :
  81723: TTCCTGCTACCCAGTCTTGGAGTTTCTTGTGGCTTTGATTCTACCTTGTA  Hsapiens
 401438: ...........................C......................  Ptroglodytes
 456234: .......A........................T..............T..  Pabelli
 360029: .....A..........................T..............T..  Nleucogenys
 445513: .....A..........................T..............T.C  Mmulatta
 394259: ................................T...C..........T.C  Panubis
 423932: .................C.......................C.....T..  Cjacchus
1065465: .............................C..T........C.....T..  Sboliviensis

                                          LINE1
                                   ------------------------->
                  81782     81792     81802     81812     81822
                      :         :         :         :         :
  81773: TATATTTAATTACTTAATCATTAATGTCTTTTATATGTTTAATCATTTTG  Hsapiens
 401488: ..................................................  Ptroglodytes
 456284: ...................................C..............  Pabelli
 360079: .G................................................  Nleucogenys
 445563: ..........C.....----------------------------------  Mmulatta
 394309: ..........C................A......................  Panubis
 423982: ........T.C....T...................G.C............  Cjacchus
1065515: ..........C.......................................  Sboliviensis

                               LINE1
         ------------------------------------------------->
                  81827     81837     81847     81857     81867
                      :         :         :         :         :
  81823: TT-----TAGCCCTATTAATTACATGAATGAAAATATCATATTCTCTTAT  Hsapiens
 401538: ..AACTC...........................................  Ptroglodytes
 456334: ..AACTC..............................T............  Pabelli
 360129: ..AACTC............................G.......A......  Nleucogenys
 445579: ------------------------------.......T.......T....  Mmulatta
 394359: ..AACTC...........C..........................T....  Panubis
 424032: G.AACTC...........................C..T..-.........  Cjacchus
1065565: ..AACTC...........................C..T..-.........  Sboliviensis
```

```
                  LINE1
         ------------------------------------------------->
              81877     81887     81897     81907     81917
  81868: CTGAAGCTGTTGGAAAGTTGAATCTTTCTGTTTGCTCTGTTCACTGACGC  Hsapiens
 401588: ................................................T.  Ptroglodytes
 456384: .....T......A...................................T.  Pabelli
 360179: ............A......T............................T.  Nleucogenys
 445599: .........................................T......T.  Mmulatta
 394409: .........................................T......T.  Panubis
 424081: ..............G.................A............-----  Cjacchus
1065614: .C............G.................A...........C-----  Sboliviensis

                  LINE1
         ------------------------------------------------->
              81926     81936                     81955     81965
  81918: -AAGTCTTGGGAGATCACCTCCTCCAGTG-TTTTGCAGTATTACATTTGG  Hsapiens
 401638: T...............G............-....................  Ptroglodytes
 456434: T..............TG............T....................  Pabelli
 360229: T..............TG............T....................  Nleucogenys
 445649: T.C..........G..G............-..............T.....  Mmulatta
 394459: T.C..........G..G............-..............T.....  Panubis
 424126: -.........A...CTG.T.T......G.-.............A......  Cjacchus
1065659: -.........A....TG.T.T......G.-.............A......  Sboliviensis

                  LINE1
         ------------------------------------------------->
              81975     81985     81995     82005     82015
  81966: AAGCTTATCTTCAGAGGAAGAATTCTGTGCAATCCAGGCTGAGGAAATGC  Hsapiens
 401687: ..................................................  Ptroglodytes
 456484: .........................C........................  Pabelli
 360279: ......C.........................................A.  Nleucogenys
 445698: ......G...........................................  Mmulatta
 394508: ............G.....................................  Panubis
 424174: .............A.....-.............G................  Cjacchus
1065707: .............AG..................G................  Sboliviensis

                  LINE1
         ------------------------------------------------->
              82025     82035     82045     82055     82064
  82016: TCTTCGGAGGAGTTTTGTCTTTGTTTTTGTCAGGAACTCGGGGGGTA-TA  Hsapiens
 401737: ......................................T.......-..  Ptroglodytes
 456534: ..............................G................-..  Pabelli
 360329: ...............................................-..  Nleucogenys
 445748: .................----...............A.T........-..  Mmulatta
 394558: .................----..........................-..  Panubis
 424223: ....TA.....CG.......................T....C..C..C.G  Cjacchus
1065757: ....TA.....C.............................CT.C..C.G  Sboliviensis

                  LINE1
         ------------------------------------------------->
              82074     82084     82094     82104     82114
  82065: TCATATTGTTTTTTATGTCAGTTTCTTAGCTTGACAGTTCTCAAATTAAC  Hsapiens
 401786: .....................C............................  Ptroglodytes
 456583: .....C............................................  Pabelli
 360378: .....C..........C.................................  Nleucogenys
 445793: C.C..C...........................G................  Mmulatta
 394603: ....GC........................C..G................  Panubis
 424273: .....C..C......---------------C..G..A.............  Cjacchus
1065807: .....C.........---------------C..G................  Sboliviensis
```

```
              LINE1
        ------------------->
                 82124     82134     82144     82154     82164
                     :         :         :         :         :
  82115: TCAGTAATATAAATTCAAAACTATGGTATTACAGTCCCATGGATATAATT   Hsapiens
 401836: .....................C...........................-   Ptroglodytes
 456633: .....................C.....G.....................-   Pabelli
 360428: ......C..............C.................C..C......-   Nleucogenys
 445843: .....................C............G..............-   Mmulatta
 394653: .....................C............G..............-   Panubis
 424308: C....................C.....T......G....G....C....-   Cjacchus
1065842: C....................C...A........GT........C....-   Sboliviensis

                        Simple
          ---------------------------->
                 82174     82184     82194     82204     82214
                     :         :         :         :         :
  82165: TTTTATTATTTTTATTATTATTATTATTATTAATTTAAAGGTCAGGCCAA   Hsapiens
 401885: -----------------................C................   Ptroglodytes
 456682: -----.....A....C.........G...............C........   Pabelli
 360477: -------T.....T...........................C........   Nleucogenys
 445892: --------------...........................C........   Mmulatta
 394702: --------------...........................C........   Panubis
 424357: -----------------..G.....................C........   Cjacchus
1065891: -----------------..........G............AC.T......   Sboliviensis

                 82224     82234     82244     82254     82264
                     :         :         :         :         :
  82215: GATAATAGTGTGCCCTTTAAATCTTTCTTCCATGTGGTTAAATTATTTTC   Hsapiens
 401918: .G.......................................G........   Ptroglodytes
 456727: ............T.....................................   Pabelli
 360520: ..................................................   Nleucogenys
 445928: ..................................................   Mmulatta
 394738: ............................C..........-----......   Panubis
 424390: ..C....A....T...........A....T.T.................T   Cjacchus
1065924: ...G...A....TT..........A..................G....G.   Sboliviensis

                 82274     82284     82294     82304     82314
                     :         :         :         :         :
  82265: CTAATCCAACATTTCACCAAGCATGTTGCTCATTAAAGGCTCCAGTATGA   Hsapiens
 401968: .......................C..........................   Ptroglodytes
 456777: .....................G..........................T.   Pabelli
 360570: .........T......................................T.   Nleucogenys
 445978: ......T................................G........C.   Mmulatta
 394783: .......................................G........C.   Panubis
 424440: ........G............T....C..............T......T.   Cjacchus
1065974: ................................................T.   Sboliviensis

                 82324     82334     82344     82354     82364
                     :         :         :         :         :
  82315: TGCCGTTTCTCTGTTCCAACAATTGGCTTCATAGACCACTTTTTGAATGT   Hsapiens
 402018: ...........................C......................   Ptroglodytes
 456827: ...A....................T..C......................   Pabelli
 360620: ...A....................CA.C....................T.   Nleucogenys
 446028: ...A....................C..C....................T.   Mmulatta
 394833: ...A....................C..C....................T.   Panubis
 424490: C.AA....................CA.C................A...T.   Cjacchus
1066024: ...A........C...........CA.C..............A.A...T.   Sboliviensis
```

```
                                                   Alu
              82374     82384     82394     82404     82414
  82365: TGGTGTAGTATAAAAAAGAAATATCTGTAAGGCACAGTGGCTTACACCTG  Hsapiens
 402068: ...........................C......................  Ptroglodytes
 456877: ...........................C......................  Pabelli
 360670: ...........................C..-...................  Nleucogenys
 446078: ......G..............A.....C......................  Mmulatta
 394883: ......G....................C......................  Panubis
 424540: .A.........................CC.....................  Cjacchus
1066074: ....A..C...................CC............AG.------  Sboliviensis
```

```
                                  Alu
              82424     82434     82444     82454     82464
  82415: TAATCCCAGCACTTTGGGAGGCTGAAGCAGGTGGATCACTTAAGCCCAGA  Hsapiens
 402118: ..................................................  Ptroglodytes
 456927: ...................A..............................  Pabelli
 360719: ..........C..............-........................  Nleucogenys
 446128: ....................A....................G...T.GT.  Mmulatta
 394933: ....................A....................G...T..T.  Panubis
 424590: .....T.G.....A.........A.G........................  Cjacchus
1066118: ..T.T...............C....G.....A....T.............  Sboliviensis
```

```
                                  Alu
              82474     82483     82493     82503     82513
  82465: AGTTAGAGACCAGCCT-GGTAACATAGTGAAACCCTGTCTCTACAAATAA  Hsapiens
 402168: ................G.....T...........................  Ptroglodytes
 456977: ................G..C....C...................C.....  Pabelli
 360768: ..........A.....G..C.............G........G.......  Nleucogenys
 446178: .....-..........G..C..............................  Mmulatta
 394983: .....-..........G..C..............................  Panubis
 424640: .A..............G..C....C..........C..............  Cjacchus
1066168: .A...-----......G..C....C.................G.....T.  Sboliviensis
```

```
                                  Alu
              82523     82533     82543     82553     82563
  82514: TAATAATACAAAAATTAGCTGGATGTGGTGGTGCACACCTGTAGTCCCAG  Hsapiens
 402218: ..................................................  Ptroglodytes
 457027: C..C....................................A.........  Pabelli
 360818: ..G.......................A....C....G.............  Nleucogenys
 446227: ......................GC.......CA.................  Mmulatta
 395032: ......................G........CA.................  Panubis
 424690: .......G..G...........G...A.....A.......A.........  Cjacchus
1066213: .......G.-TTG.C.....A.G.........A.................  Sboliviensis
```

```
                                  Alu
              82573     82583     82593     82603     82613
  82564: CTACTCAGGAGGCTGAGGTGGGCAGATCAAGGCTGAGGTGGGCGGATCAC  Hsapiens
 402268: ..................................................  Ptroglodytes
 457077: ........C.........................................  Pabelli
 360868: ......T..--------------------.....................  Nleucogenys
 446277: .....TG..--------------------.........CA..T.......  Mmulatta
 395082: .....TG..--------------------.........CA..T.......  Panubis
 424740: A....T...--------------------.........CA...A......  Cjacchus
1066262: A........--------------------..............AT.....  Sboliviensis
```

```
                                Alu
         -------------------------------------------------->
              82623     82633     82643     82653     82663
  82614: TTGAGCCCAGGAGGCAGAGATTGCAGTGAGCCCAGATCACACTACTGCAC  Hsapiens
 402318: .......................T..................C.......  Ptroglodytes
 457127: .......T..................................C.......  Pabelli
 360898: ................C.......T.................C.......  Nleucogenys
 446307: .............................A............C.......  Mmulatta
 395112: .............................A............C.......  Panubis
 424770: .........................A...............AC.......  Cjacchus
1066292: ......................A...C........G.....AC.......  Sboliviensis

                                Alu
         -------------------------------------------------->
              82673     82683     82693     82703     82713
  82664: TCCAGCCTGGGCACCAGAGTGAGACTCTGCGAAAAAAAAAAAAAAAAAAA  Hsapiens
 402368: ........................T.....C.........--------..  Ptroglodytes
 457177: ............G................TCC........----------  Pabelli
 360948: .....G......G.............--.TC.....G.....G...G...  Nleucogenys
 446357: ........C....................TC.T........G....G...  Mmulatta
 395162: ........C....................TC.T........TC...G...  Panubis
 424820: C........A................----TG....G....G.G...G.G  Cjacchus
1066342: C........A................----TG....G....G.G...G.G  Sboliviensis

           Alu                         DNA
         --------->       ------------------------------>
              82723     82731
  82714: AAAAAAAAAGAAA--GAAAGAAGGAA------------------------  Hsapiens
 402410: .....G.......--...........------------------------  Ptroglodytes
 457217: ---------------...........------------------------  Pabelli
 360996: G....G......GAA.......A...AAGAAAGAAAGAAAGAAAGAAAGA  Nleucogenys
 446407: ...G........G--...G.......                          Mmulatta
 395212: ...G........G--...G.......                          Panubis
 424866: G.G.GG.G..GG.--..G..GGA..                           Cjacchus
1066388: G.C.GG.G..G.G--AGG........                          Sboliviensis

                                DNA
         -------------------------------------------------->
                                                  82740     82749
  82738: -------------------------------------ATA-TCACGGTTA  Hsapiens
 402434: -------------------------------------...-....A....  Ptroglodytes
 457228: -------------------------------------...-....A....  Pabelli
 361046: AAGAAAGAAAGAAAGAAAGAAAGAAAGAAAGAAAGAA.G.-....A....  Nleucogenys
 446584:                                      .G.A....A....  Mmulatta
 395390:                                      .G.A....A....  Panubis
1066557:                                        .-.........  Sboliviensis

                                DNA
         -------------------------------------------------->
              82759                                   82769
  82750: ATTGAAAAGGCTTT------------------------------TATGAA  Hsapiens
 402446: ...........---------------------------------.---T.  Ptroglodytes
 457240: ...........---------------------------------......  Pabelli
 361095: .........T.---------------------------------......  Nleucogenys
 446597: T.....G.A...A.GAACATTCTCCTTTAGTTAATTGAAGAAGC......  Mmulatta
 395403: ......G.A..---------------------------------......  Panubis
1066567: .....---------------------------------------------  Sboliviensis
```

```
         DNA                 Alu
         ---------------->----------------------------------->
                   82779     82789     82799     82809     82819
                       :         :         :         :         :
   82770: AATTCTCCTTTAGGCCCGGCGCAGTGGCTCACACCTGTAATCACAGCATT   Hsapiens
  402460: ................T.................................   Ptroglodytes
  457257: .................A..A........T.T..........C.......   Pabelli
  361112: ..........................................C.......   Nleucogenys
  446647: C................A.........T........A.....C.......   Mmulatta
  395420: C................A.T................A.....C.......   Panubis
 1066572: ----------------------------...TG.........C....CC.   Sboliviensis
```

```
                             Alu
         ------------------------------------------------->
                   82829               82840     82850     82860
                       :                   :         :         :
   82820: TTGGGAGGCTGAGGCAG---------GCGGATCACAAGGACAAGAGATTG   Hsapiens
  402510: .................---------.T......................   Ptroglodytes
  457307: .................---------........................   Pabelli
  361162: .................---------........................   Nleucogenys
  446697: .................TAGACTAGCCT..T.A...T..T..G...T.CA   Mmulatta
  395470: .......A.........---------..A.....................   Panubis
 1066594: .........CA......---------CTA.....T....T.....A..C.   Sboliviensis
```

```
                             Alu
         ------------------------------------------------->
                   82870     82880     82890     82900     82910
                       :         :         :         :         :
   82861: AGACCATGCTGGCCAACATGGTGAAACCTCATCTCTACTAAAAATACAAA   Hsapiens
  402551: ...................................C..............   Ptroglodytes
  457348: ............................C.....................   Pabelli
  361203: .......T...........A........C........TG..G..-.....   Nleucogenys
  446747: ....TG......................C.....................   Mmulatta
  395511: .....G......................C.....................   Panubis
 1066635: .......C..............A.....C....G.............---   Sboliviensis
```

```
                             Alu
         ------------------------------------------------->
                   82920     82930                         82944
                       :         :                             :
   82911: AATTAGCTGGGCGTGGTGGCACGT----------------GCCTGTAGTC   Hsapiens
  402601: ........................----------------..........   Ptroglodytes
  457398: ........................----------------..........   Pabelli
  361252: ...........T..........C.----------------.........T   Nleucogenys
  446797: ...............T........TTTGACTCTGTTTTGT..A......A   Mmulatta
  395561: ...........T...T........TTTGACTCTGTTTTGT..A......A   Panubis
 1066682: .....T.....T..A....TGT..----------------..........   Sboliviensis
```

```
                             Alu
         ------------------------------------------------->
                   82954     82964     82974     82984     82994
                       :         :         :         :         :
   82945: CCAGCTATTCAGGAGGCTGAGGCAGGAGAATTGCTTGAACCTGGGAGGCG   Hsapiens
  402635: .......C...................C......................   Ptroglodytes
  457432: ......GC.........................................A   Pabelli
  361286: .......C..G.....................................T.   Nleucogenys
  446847: .......C..G.......................................   Mmulatta
  395611: .......C..G.......................................   Panubis
 1066716: .......C.TG.......------....C..CAT.......CA......A   Sboliviensis
```

```
                                Alu
         ------------------------------------------------->
              83004     83014     83024     83034     83044
  82995: GAGGTTGCAGTGAGCCAAGATCGCGCCACTGTGCCCCAGCCTGGCAACAG  Hsapiens
 402685: ........................A.........T...............  Ptroglodytes
 457482: ......................A...........T...............  Pabelli
 361336: ....C..................T.......CA.T...............  Nleucogenys
 446897: ...............................CA.T.....A.........  Mmulatta
 395661: .....................T.........CA.T.....A.........  Panubis
1066760: ...A.......A.....T.....T........A.T.........TG....  Sboliviensis

                             Alu                     DNA
         ------------------------------------------>------>
              83054     83064     83074     83084     83094
  83045: AGTGAGACTCTGTCTCAAAAAAAAAAAAAAAAAAAAAAAAAAACTCCTTT  Hsapiens
 402735: ................----......................T.......  Ptroglodytes
 420256:                 ..........................T.......  Ggorilla
 457532: ..C.............-----....................TT.......  Pabelli
 361386: ..CA.....TC.....-----------.....C........TT.......  Nleucogenys
 446947: ..CA.A..........-------..................---......  Mmulatta
 395711: ..CA.A..........---......................---......  Panubis
1066810: ..CA............---......G.....G....G....TT..G....  Sboliviensis

                                DNA
         ------------------------------------------------->
              83104     83114     83124     83134     83144
  83095: ATTTTCAAACCACATATCTCTGTGAAGCCATATATTCTTCAACCAAAACA  Hsapiens
 402781: ....C......................T......................  Ptroglodytes
 420290: ..................................................  Ggorilla
 457577: .....................A.......................G....  Pabelli
 361425: G............G.....A.A...G........................  Nleucogenys
 446987: .....G.............A..............................  Mmulatta
 395755: .....G.............A..............................  Panubis
1066857: ......C............A.A.............A......TT......  Sboliviensis

                                DNA
         ------------------------------------------------->
              83154     83164     83174     83184     83194
  83145: ACATATTGCAACAGATTGAAGGCACATGCAGATACATAAATCTAGCAAGA  Hsapiens
 402831: ....................T.............................  Ptroglodytes
 420340: ....................T.............................  Ggorilla
 457627: ....................T...A.........................  Pabelli
 361475: ...C................T.........................C...  Nleucogenys
 447037: ....................T.......................A.C...  Mmulatta
 395805: ....................T.......................A.C...  Panubis
1066907: ....................T......A..................C...  Sboliviensis

                                DNA
         ------------------------------------------------->
              83204     83214     83224     83234     83244
  83195: CATAATTACACTAAATCAAACATTAAAGAGATTTGTAAAAATGTAAAACA  Hsapiens
 402881: ............................................G.....  Ptroglodytes
 420390: .........G..........T.............................  Ggorilla
 457677: .................G...............C..........-.....  Pabelli
 361525: .................................CA...............  Nleucogenys
 447087: ........................G..A.....C................  Mmulatta
 395855: ........................G..A.....C................  Panubis
1066957: ..........................T..............GC.......  Sboliviensis
```

```
                              DNA
          ------------------------------------------------->
                  83254     83264     83274     83284     83292
  83245: ATGCCTTTTTTACCACTATTTTTTGTATACTGGAAAACATAGC--TTTTT  Hsapiens
 402931: .......................................C.T.--.....  Ptroglodytes
 420440: ...........................................--.....  Ggorilla
 457726: ...........................................--.....  Pabelli
 361575: ............................C..............--.....  Nleucogenys
 447137: ............T............................A.--.....  Mmulatta
 395905: ............T............................A.--.....  Panubis
 425934:   ..T.C...G........AC........T......G......TT.....  Cjacchus
1067007: ....T.C.............C........T......G.....T--.....  Sboliviensis
```

```
                              DNA
          ------------------------------------------------->
                  83302     83312     83322     83331     83341
  83293: AAATTAAAAATGTGTCATTTACATTAACATGCAA-TGGTTGTAATGTTTT  Hsapiens
 402979: ...A..............................-........T......  Ptroglodytes
 420488: .....................A............-...............  Ggorilla
 457774: ...A..............................-..A.....T......  Pabelli
 361623: ...A.........................A....-........T......  Nleucogenys
 447185: ...A.................T.....T......-C.......T.A....  Mmulatta
 395953: ...A............C.----............-C......GT.A....  Panubis
 425982: ....A.............................T........T.-..A.  Cjacchus
1067055: ...-.......A......................-........T.T..A.  Sboliviensis
```

```
                              DNA
          ------------------------------------------------->
                  83351     83361     83371     83381     83391
  83342: TTAATATTAAGATGTTATTTTATAAGTTTATATTTAAACTTTTTCTGCTT  Hsapiens
 403028: .........................................C........  Ptroglodytes
 420537: ............C.....................................  Ggorilla
 457823: .........................T...................C....  Pabelli
 361672: ......C.G...G............T.....................T..  Nleucogenys
 447234: .........................T.....G.............C.T..  Mmulatta
 395998: ............C............T.....G.............CAT..  Panubis
 426031: .....G...........C.......T..................A-.T..  Cjacchus
1067103: ....GG...................T..................A-.T..  Sboliviensis
```

```
                              DNA
          ------------------------------------------------->
                  83401     83411     83418     83428     83438
  83392: AAATTTCTGATGGAGTAAAT---ATCATTAGAAAAATGCACATAAACAAA  Hsapiens
 403078: ....................---...........................  Ptroglodytes
 420587: ....................---...........................  Ggorilla
 457873: ....................---...........................  Pabelli
 361722: ............T.....C.---...-............G..........  Nleucogenys
 447284: ............T.......---...........................  Mmulatta
 396048: ............T.......---................G..........  Panubis
 426080: ............C.......--T..................C.....C..  Cjacchus
1067152: ............C.......AGC........................C..  Sboliviensis
```

```
                                DNA
          -------------------------------------------------->
               83447     83457     83467     83477     83487
   83439: AG-TTCTTTGAGATTTCCTTAATAATTCTGAAGAGCTTAAAGGGGTCCTG  Hsapiens
  403125: ..-...................C...........................  Ptroglodytes
  420634: ..-...............................................  Ggorilla
  457920: ..-...............................AT.....C........  Pabelli
  361768: ..-..........A.....................T..............  Nleucogenys
  447331: ..-........................TG.....AT......T.......  Mmulatta
  396095: ..-.........................G.....AT......T.......  Panubis
  426128: ..-...........--...................-...........T..  Cjacchus
 1067202: ..T..T........--...................-.......A......  Sboliviensis

                      DNA                     LINE2
          -------------------------->   ------------------------>
               83497     83507     83517     83527     83537
   83488: AAACCAAAATGTCTGGGTACCTGCTGTACTGTACATTTCTGCTGCATTTA  Hsapiens
  403174: ..................................................  Ptroglodytes
  420683: ..................................................  Ggorilla
  457969: ..................................................  Pabelli
  361817: .....................A............................  Nleucogenys
  447380: ..................G.GCA......CA..T................  Mmulatta
  396144: ....................GC.......C..GT...........G....  Panubis
  426174: .............C...C.T.C..C..G..............C....G..  Cjacchus
 1067249: .............C...C.TT.............................  Sboliviensis

                               LINE2
          -------------------------------------------------->
               83547     83557     83567     83577     83587
   83538: GTATGCTGAACATTCTCTTCTAGCTTTCATTTCTCTTCTCTCATCTTCCT  Hsapiens
  403224: ..................................................  Ptroglodytes
  420733: ..................................................  Ggorilla
  458019: ..................................................  Pabelli
  361867: ................................................T.  Nleucogenys
  447430: ...C..............................................  Mmulatta
  396194: ...C..............................................  Panubis
  426224: .C..............T...................C.............  Cjacchus
 1067299: .C..............T.........................T.......  Sboliviensis

                               LINE2
          -------------------------------------------------->
               83597     83607     83617     83627     83637
   83588: CAGTGCACCATTCCAACTCCTTTTCCTCTTTCCAACATCTAGTTTTCCCC  Hsapiens
  403274: ........................................G.........  Ptroglodytes
  420783: ........................................G.........  Ggorilla
  458069: ........................................G.........  Pabelli
  361917: ..................................T.....G.........  Nleucogenys
  447480: ..................TA..............CTG...G.........  Mmulatta
  396244: ..................TG.............GCTG...G....C....  Panubis
  426274: ........T..G........-C............C.....G.........  Cjacchus
 1067349: ........T........C.......TG.......C.....G......A..  Sboliviensis
```

```
                                     LINE2
         ---------------------------------------------------------->
                83647     83657     83667     83677     83687
                    :         :         :         :         :
   83638: TAGTTTTCATCCTTTGCTTCCTTATTATTATATTATGTCTTCTCTCTCTC  Hsapiens
  403324: ...............A..............C...................  Ptroglodytes
  420833: ...................T................A..........T..  Ggorilla
  458119: ...................................C..............  Pabelli
  361967: ........G.........C...............................  Nleucogenys
  447530: ..................................................  Mmulatta
  396294: ..................................................  Panubis
  426323: ...................T..............................  Cjacchus
 1067399: ...................T.....................G.A......  Sboliviensis

                                     LINE2
         ---------------------------------------------------------->
                83693     83703     83713     83723     83733
                    :         :         :         :         :
   83688: T----GGGTGACCTCCACCACTCACATTATTTTAACAACGTCCCAAATAG  Hsapiens
  403374: .----...........................C.................  Ptroglodytes
  420883: .----.............................................  Ggorilla
  458169: .----..A...............................T..........  Pabelli
  362017: .----..A................................C.........  Nleucogenys
  447580: .CTCT..A.......A....................G.............  Mmulatta
  396344: .CTCT..A.......A...........C......................  Panubis
  426373: .----..A....T..A......A................A..........  Cjacchus
 1067449: .----..A...TT..A......A....G...........A..........  Sboliviensis

                                     LINE2
         ---------------------------------------------------------->
                83743     83753     83763     83773     83783
                    :         :         :         :         :
   83734: ATGACATCCAAATATGTATCTGTAATCTGATTATTTTACCATACTCTGTA  Hsapiens
  403420: ..................................................  Ptroglodytes
  420929: ..................................................  Ggorilla
  458215: G.................................................  Pabelli
  362063: .................................................G  Nleucogenys
  447630: .........................C.......................G  Mmulatta
  396394: .........................C.......................G  Panubis
  426419: C...........C..........T.C.............A.........G  Cjacchus
 1067495: .......................T.C.............A.........G  Sboliviensis

                                     LINE2
         ---------------------------------------------------------->
                83793     83803     83813     83823     83833
                    :         :         :         :         :
   83784: CTAGCAAAACCAGCTACCTACATCTTGATGTTTCTTAAAAAGCTAAACTC  Hsapiens
  403470: G........T......T.................................  Ptroglodytes
  420979: ....A.....................C.......................  Ggorilla
  458265: ................T...T.............................  Pabelli
  362113: ................T.................................  Nleucogenys
  447680: .C..............T.................................  Mmulatta
  396444: .C..............T.................................  Panubis
  426469: .C........A.....T......A.................A........  Cjacchus
 1067545: .C........A.....TG.....A..........................  Sboliviensis
```

```
                                     LINE2
         ------------------------------------------------------>
                  83843     83853     83863     83873     83883
                      :         :         :         :         :
  83834: AAATATTCCCAAACTGAGCATGTGAGTGAATGGTACCATCATTAAATCCT  Hsapiens
 403520: ..................................G...............  Ptroglodytes
 421029: ..................................................  Ggorilla
 458315: ..................................................  Pabelli
 362163: .............T....................................  Nleucogenys
 447730: ..................................................  Mmulatta
 396494: ..................................................  Panubis
 426519: G.G...............................................  Cjacchus
1067595: G.G................G.................G............  Sboliviensis

                                     LINE2
         ------------------------------------------------------>
                  83893     83903     83913     83923     83933
                      :         :         :         :         :
  83884: ACCACGTAAGTCAGAGATCTGTGAGTCATCCTAGATTCCTTTTTCCCCTA  Hsapiens
 403570: ........G.........................................  Ptroglodytes
 421079: .....A............................................  Ggorilla
 458365: G....A................................T...........  Pabelli
 362213: G.................................G...............  Nleucogenys
 447780: G..................C.......................G......  Mmulatta
 396544: G....A.............C.......................G......  Panubis
 426569: T.....................A....................GTTT.C.  Cjacchus
1067645: T....C.....................................GTTT.C.  Sboliviensis

                                     LINE2
         ------------------------------------------------------>
                  83943     83953     83963     83973     83983
                      :         :         :         :         :
  83934: CCTCCCTCATCCACTACCAAGCCCTGTAGTTTTTGCTTCTTAAATATTCC  Hsapiens
 403620: ..................................................  Ptroglodytes
 421129: ..................................................  Ggorilla
 458415: ..................................................  Pabelli
 362263: ..................................A...............  Nleucogenys
 447830: ..................................................  Mmulatta
 396594: .................................................T  Panubis
 426619: .T..----.......G..................................  Cjacchus
1067695: .T..----..........................................  Sboliviensis

                  83993     84003     84013               84031
                      :         :         :                   :
  83984: TCCTTCCGTGGATTGCGTTAGCTCAAAATCTCATGACC--TAGATACATT  Hsapiens
 403670: ......................................--..........  Ptroglodytes
 421179: ......................................--..........  Ggorilla
 458465: ...C...A........A.....................--..........  Pabelli
 362313: ...C..................................TA..........  Nleucogenys
 447880: ...C........G.........................--..........  Mmulatta
 396644: ...C........G.........................--..........  Panubis
 426665: ...C...A........A.........G...........--..........  Cjacchus
1067741: ...C...A........A.........G...........--..........  Sboliviensis
```

```
                  84041     84051     84061     84071     84081
                      :         :         :         :         :
  84032: ATTGCAAACCTTCCTAATGGGCTTCCTATTACTCCCTGTTACTACTGGTC  Hsapiens
 403718: .................................T................  Ptroglodytes
 421227: .................................T................  Ggorilla
 458513: .......G.............T...........T.........---....  Pabelli
 362363: .................................----......---....  Nleucogenys
 447928: .................................T.........---....  Mmulatta
 396692: .........T.......................T.........---....  Panubis
 426713: ...........................G.....-------------...T  Cjacchus
1067789: ................G..........G.....-------------....  Sboliviensis

                         Simple
                   ------------------------->
                  84091     84101     84111     84121     84131
                      :         :         :         :         :
  84082: CCTCGTTTCTCAAAAAAAAAAAAAAAAAAAAAAAGTCTGTAGTTATTATC  Hsapiens
 403768: ...........---------.................C.........G..  Ptroglodytes
 421277: ...........----------................C............  Ggorilla
 458560: .T.........--........................CA.........C.  Pabelli
 362406: .T.........------------..............CAG..........  Nleucogenys
 447975: .T..AC.....-----------.............C.CA...A..C.G..  Mmulatta
 396739: .T..AC.....------------............C.CA...A..C.G..  Panubis
 426750: .T..A.....A-----------............T..CA...........  Cjacchus
1067826: TT..A......---------..............A..CA...........  Sboliviensis

                  84141     84151     84161     84171     84181
                      :         :         :         :         :
  84132: CTGAAATACAGATCTATTCATACCACCCACATGCTTAAAACTCTTCAGTA  Hsapiens
 403809: ..........A.....G........................C........  Ptroglodytes
 421317: ..........A.....G........................C........  Ggorilla
 458608: ..........A.....G........................C.....A..  Pabelli
 362444: ..........A.....G........................C.....A..  Nleucogenys
 448014: ..........A..T..G........................C.....A..  Mmulatta
 396777: ..........A.....G........................C.....A..  Panubis
 426789: ..........A.....G............A......T....C.....A..  Cjacchus
1067867: ..........A.....G..C................T....C.....A..  Sboliviensis

                  84191     84201     84211     84221     84231
                      :         :         :         :         :
  84182: GCTCCCCAGGGCCTTCAGAAAAAAAACTCAGGTCTTTCATCATGACACAA  Hsapiens
 403859: .....................................G............  Ptroglodytes
 421367: ..........................T..........G............  Ggorilla
 458658: .T................................................  Pabelli
 362494: .T........................T.......................  Nleucogenys
 448064: .T.......................-........................  Mmulatta
 396827: .T.......................-........................  Panubis
 426839: .T................G......-........................  Cjacchus
1067917: .T................G......-.........C..............  Sboliviensis

                  84241     84251     84261     84271     84281
                      :         :         :         :         :
  84232: GAAGCCTTCATTATTAGGCAATTTTTCCTTCCCAGTCTCATCACCCTTGT  Hsapiens
 403909: .........G........................................  Ptroglodytes
 421417: .........G........................................  Ggorilla
 458708: ..G......---......................................  Pabelli
 362544: ..G......---......................................  Nleucogenys
 448113: ..G........C...........C..........................  Mmulatta
 396876: ..G........C...........C....................T.....  Panubis
 426888: ..G........C........T..........T....G.......T.A.C.  Cjacchus
1067966: ..G..T...T.C........T.......A.....A.........T.A.C.  Sboliviensis
```

```
              84291     84301     84311     84321     84331
                  :         :         :         :         :
  84282: GAGTGTCTCTTCCTGCATTTCTTGCTTTAGCAAAACAGAATTATTTGCTT  Hsapiens
 403959: ..................................................  Ptroglodytes
 421467: ..................................................  Ggorilla
 458755: ..................................................  Pabelli
 362591: ..................................................  Nleucogenys
 448163: .......................A.....A......G..G.......T..  Mmulatta
 396926: .......................A.....A......G..G.......T..  Panubis
 426938: ..............................T....TG.GG.......T.C  Cjacchus
1068016: ...C..........................T....TG.GG.G.....T.C  Sboliviensis
```

```
              84340     84350     84360     84370     84380
                  :         :         :         :         :
  84332: TTCCCC-ATCACAATTTGCGTGTCTCCTCCCTTTGCTGTAATGCATGCCA  Hsapiens
 404009: ......-.AA...........C............................  Ptroglodytes
 421517: ......-.A............C............................  Ggorilla
 458805: ......T.A............C.......................C....  Pabelli
 362641: ......T.A............C............................  Nleucogenys
 448213: ......T.A............C............................  Mmulatta
 396976: ......T.A........A...C...................A........  Panubis
 426988: ......T.AT.........A.C.......................CC...  Cjacchus
1068066: ....T.T.A..........A.C.......................CC...  Sboliviensis
```

```
              84390     84400     84410     84420     84430
                  :         :         :         :         :
  84381: TCTCAGACTTAACCACTTGTTGAGCTCCTATTCATCCTTTAAACCCTGAG  Hsapiens
 404058: ..................................................  Ptroglodytes
 421566: ..............................C...................  Ggorilla
 458855: .............A....................................  Pabelli
 362691: ........................................-.........  Nleucogenys
 448263: ...........G......................................  Mmulatta
 397026: ...........G......................................  Panubis
 427038: ...........T.......................T..C....T.T....  Cjacchus
1068116: ...........T....G..................T.AC......T....  Sboliviensis
```

```
                                             LINE1
                                      ------------------->
              84438     84448     84458     84468     84478
                  :         :         :         :         :
  84431: AAGTT--CTTCCCTTCCTCTGAAGAGTCTTTTGGATTGCATGAATGTCAG  Hsapiens
 404108: .....--.............................G.............  Ptroglodytes
 421616: .....--.............................GT............  Ggorilla
 458905: ...G.--....G............................C....A....  Pabelli
 362740: ...G.--....T............................C.........  Nleucogenys
 448313: ...G.--....T............................CC........  Mmulatta
 397076: ...G.--....T............................CC........  Panubis
 427088: C..G.GT....T..............C.............CC........  Cjacchus
1068166: C..G.GT....T..............C-............CC..C.....  Sboliviensis
```

```
                                 LINE1
         ------------------------------------------------->
              84488     84498     84508     84518     84528
                  :         :         :         :         :
  84479: TTCCTGGTTTTTCTATTTGTATTATAGTTGTGCAAAATATTGTTATTGGG  Hsapiens
 404156: ...............................................C..  Ptroglodytes
 421664: ..................................................  Ggorilla
 458953: ..................................................  Pabelli
 362788: ........................................C.........  Nleucogenys
 448361: ........G.....G......-............................  Mmulatta
 397124: ........G.....G......-............................  Panubis
 427138: .......A.......C..T....G........T....C.......-....  Cjacchus
1068215: .......A.......C..T....GC.......T....C....C......A  Sboliviensis
```

```
            LINE1
          -------->
                   84538     84548     84558     84568     84578
                       :         :         :         :         :
   84529: GGAAACTGGTAGAAAATTTCCTTATACATTTTTTGGCAATTTCCTGCTAA  Hsapiens
  404206: ..................................................  Ptroglodytes
  421714: ..................................................  Ggorilla
  459003: ..................................................  Pabelli
  362838: ................G.................................  Nleucogenys
  448410: .A.............................................A..  Mmulatta
  397173: .A.............................................A..  Panubis
  427187: ................C.CT...G.G..........GG........AG..  Cjacchus
 1068265: ................C..T...G.G..........AG.........G..  Sboliviensis

                                                 DNA
                                          --------------->
                   84588     84597     84607     84616     84626
                       :         :         :         :         :
   84579: CCTATAAGTATTTCCAAG-TTTTTTAAAGATG-TTTATAGATACTTCCCA  Hsapiens
  404256: ..................T.............-..........T......  Ptroglodytes
  421764: ..................-.............-..........T......  Ggorilla
  459053: .T................-.............-.................  Pabelli
  362888: .T................T.............-...............A.  Nleucogenys
  448460: .T.G..............T.............C.............A...  Mmulatta
  397223: .T.G..............T.............C..........----...  Panubis
  427237: .............T..---G............C..............T..  Cjacchus
 1068315: ..................--...C......G.CC................  Sboliviensis

                            DNA
          ------------------------------------------------>
                   84636     84646     84656     84666     84676
                       :         :         :         :         :
   84627: TTTTATCACACCAAATATTAGAAGATGTGTATTCAAGAGTTGAACTTTGA  Hsapiens
  404305: ............................................A...A.  Ptroglodytes
  421812: ............................................A...A.  Ggorilla
  459101: ............................................A...A.  Pabelli
  362937: ........................................----------  Nleucogenys
  448510: .....................................T......A...A.  Mmulatta
  397269: .....................................T......A...A.  Panubis
  427284: ...............................C............GG..A.  Cjacchus
 1068363: ..........................A....G........G...G...A.  Sboliviensis

                            DNA
          ------------------------------------------------>
                   84685     84695     84705     84715     84725
                       :         :         :         :         :
   84677: TAAA-ATCTACAATACTTATTGCTTCATCAAGAACATTCAGAAGTGAAAA  Hsapiens
  404355: ...C-.............................................  Ptroglodytes
  421862: ...C-.............................................  Ggorilla
  459151: ....-.............................................  Pabelli
  362977: -----..T--........................................  Nleucogenys
  448560: ....-...........................-.................  Mmulatta
  397319: ....-...........................-.................  Panubis
  427334: ....T.......G..T..........T..............C........  Cjacchus
 1068413: ....T.......G..T..........T...................G...  Sboliviensis
```

```
                                DNA
         ------------------------------------------------->
                84735     84745     84755     84765     84775
                    :         :         :         :         :
   84726: ATGACGTTTTCATATATGTGTGGCAGTGAGGAATCCATGGCTACCAGCCC  Hsapiens
  404404: .......................................A..........  Ptroglodytes
  421911: .......................................A..........  Ggorilla
  459200: .............G.........................A..........  Pabelli
  363020: .....A......C..........................A..........  Nleucogenys
  448608: .C...A.....C...........................A..........  Mmulatta
  397367: .C...A.....C...........................A.C........  Panubis
  427384: .---.A.......G................A........A.......T..  Cjacchus
 1068463: .---.A.......G................A..C.....A.......T..  Sboliviensis

                                DNA
         ------------------------------------------------->
                84785     84795     84805     84815     84825
                    :         :         :         :         :
   84776: AGTTTGGAGCCAACATGCCCTACCTCATGCTAAATTACAGTAGTCCTAGC  Hsapiens
  404454: G.................................................  Ptroglodytes
  421961: ..............................................C.T.  Ggorilla
  459250: .............A..............................T...T.  Pabelli
  363070: .............A.......G.T........................T.  Nleucogenys
  448658: ..C......---------..............................T.  Mmulatta
  397417: ..C......---------..............................T.  Panubis
  427431: .A...........A...T.....T..........C....A......C.T.  Cjacchus
 1068510: .A...........A...T.....T..........C....A........T.  Sboliviensis

                                DNA
         ------------------------------------------------->
                84835     84845     84855     84865     84875
                    :         :         :         :         :
   84826: CATCACTTTTGCACCATCAGCACAAATCACAACACAGTGGAAAAGGCAAA  Hsapiens
  404504: ..................................................  Ptroglodytes
  422011: ............................G.....................  Ggorilla
  459300: ............................G.....................  Pabelli
  363120: ............................G.....................  Nleucogenys
  448699: ..................................................  Mmulatta
  397458: ..................................................  Panubis
  427481: .........CCT......TA........G...T......A..........  Cjacchus
 1068560: ..........CT.......A.....T......T.................  Sboliviensis

                                DNA
         ------------------------------------------------->
                84885     84895     84905     84915     84925
                    :         :         :         :         :
   84876: AGTGTTTTAGTATTATTATAAAATTGGTTGTGTTCTTGTGGTCTCAGAAA  Hsapiens
  404554: ..................................................  Ptroglodytes
  422061: ..................................................  Ggorilla
  459350: ............................................A.....  Pabelli
  363170: ...T.....................A......A.................  Nleucogenys
  448749: ................................A.................  Mmulatta
  397508: ........................C.......A.................  Panubis
  427531: .A.......................A......A...CC......TG.G..  Cjacchus
 1068610: .A.......................A......A...CC.......G.G..  Sboliviensis
```

DNA →

```
                   84935     84945     84955     84965     84975
  84926: CTCTCAGGGATCCACAGATCACACACAGAGAATCTCTGATGGGAAAGACC  Hsapiens
 404604: ..................................................  Ptroglodytes
 422111: .....................................A............  Ggorilla
 459400: ........................................T.........  Pabelli
 363220: .........................T..............C.........  Nleucogenys
 448799: ....T...........C........TG.............CC........  Mmulatta
 397558: ....T...................GTG.............CC........  Panubis
 427581: T........................TG..........C..CA......T.  Cjacchus
1068660: T.................C......TG..........C..TA......T.  Sboliviensis

                   84985     84995     85005     85015     85025
  84976: AGGGTAAACTTAAGCAAAAACTATATCATTAAATAAGTCCACATTCTTTT  Hsapiens
 404654: ..................................................  Ptroglodytes
 422161: ..................................................  Ggorilla
 459450: ...............G..................................  Pabelli
 363270: .....G............................................  Nleucogenys
 448849: .....G..T.............G...........................  Mmulatta
 397608: .....G..T.............G.G.........................  Panubis
 427631: .....G.............G..G...T.......................  Cjacchus
1068710: .....G........T-......G...........................  Sboliviensis

                   85035     85045     85054     85064     85074
  85026: TCATAATCAGATGAAATTGCTTCCTT-CCCCATGCTATTGAGGAACGATG  Hsapiens
 404704: .................G........-.......................  Ptroglodytes
 422211: .................G........-...................A...  Ggorilla
 459500: ..........................-.T................A....  Pabelli
 363320: C........A........A.......-.......................  Nleucogenys
 448899: ..........................C......C................  Mmulatta
 397658: ..........................C......C................  Panubis
 427681: .........C............-----.T....C....C....G.T....  Cjacchus
1068759: G...........A.........-----......C....C....G.T....  Sboliviensis

                   85084     85094     85104     85114     85124
  85075: TTATTCTCTCTCCCATGATCTTCTCCCCCTTAATAACTTTAGGGATGAGG  Hsapiens
 404753: ...........................G......................  Ptroglodytes
 422260: ...........................G......................  Ggorilla
 459549: ..............G.......................G...A.......  Pabelli
 363369: ..............G.A.................................  Nleucogenys
 448949: .C.......G.......................C................  Mmulatta
 397708: .C.......G.......................C................  Panubis
 427726: .....................CTC...T.....................A  Cjacchus
1068804: ...........T....C......-...T......................  Sboliviensis

                   85134     85144     85154     85164     85174
  85125: AGAGGAATCTTCAGCCTTGCTGTTGCCTTAAAGTGAAAAAGCCAGAGCCC  Hsapiens
 404803: ..................................................  Ptroglodytes
 422310: ..................................................  Ggorilla
 459599: ...............................................T..  Pabelli
 363419: ..................................................  Nleucogenys
 448999: ...C.C......G............................-------T.  Mmulatta
 397758: .....C......G............................-------T.  Panubis
 427776: ..G..............G.................G..G.AA....--A.  Cjacchus
1068853: ..G..............A.................G..G.A.....A...  Sboliviensis
```

```
                  85184     85194     85204     85214     85224
                      :         :         :         :         :
   85175: TTTCTTGTCCCCAGGAAGTCTTGTGCTGTTGAAGTGCTATTGCAGCCACT  Hsapiens
  404853: ......................................G...........  Ptroglodytes
  422360: ......................................G...........  Ggorilla
  459649: ..................................................  Pabelli
  363469: .............................................T....  Nleucogenys
  449042: .............-....................................  Mmulatta
  397801: .............-....................................  Panubis
  427824: .........T...A..................C.................  Cjacchus
 1068903: .....G...T.........................T..............  Sboliviensis

                  85234     85244     85254     85264
                      :         :         :         :
   85225: GCCACACGGTGTCACAGGCGGTGTGGCCAGCAATGTGCTGCACCACAC--  Hsapiens
  404903: ...................A........................T...CA  Ptroglodytes
  422410: .......A........................................--  Ggorilla
  459699: ................................................CA  Pabelli
  363519: .......A........................................CA  Nleucogenys
  449091: ..................T........................A....CA  Mmulatta
  397850: ..................T........................A....CA  Panubis
  427874: ................A.T........................AG...CA  Cjacchus
 1068953: ......A.........A......................A...AG.C.CA  Sboliviensis

                  85281     85291     85301     85311     85321
                      :         :         :         :         :
   85273: -CAAGTTACAGGTGATCTCTGAAACCTTTGCCCAAGAACACGCTATAACT  Hsapiens
  404953: A........C.............G..........................  Ptroglodytes
  422458: -.................................................  Ggorilla
  459749: A....G..AC........................................  Pabelli
  363569: AT...G...CA..............................A........  Nleucogenys
  449141: A....G...C..............................T.........  Mmulatta
  397900: A....G.G.C..............................T.........  Panubis
  427924: A....G...T.....A...................C...C..........  Cjacchus
 1069003: A....G...T.....A..................................  Sboliviensis

                                  DNA
                        ---------------------------->
                  85331     85341     85351     85361     85371
                      :         :         :         :         :
   85322: TTTTTCTATTTTTATGAAAAGCATATATTCCTTGCACATTTCCAAGCTGA  Hsapiens
  405003: .....................G............................  Ptroglodytes
  422507: ..................................................  Ggorilla
  459799: .......G..........................A...............  Pabelli
  363619: ...G.......-......................................  Nleucogenys
  449191: .......T......-A..........................T.......  Mmulatta
  397950: .......T......-A..................................  Panubis
  427974: .....A.T.....T----...........T...............A....  Cjacchus
 1069053: .......T...C.T.TTT...........T...............A..A.  Sboliviensis

                           DNA
          ------------------------------------------------->
                  85381     85391     85401     85411     85421
                      :         :         :         :         :
   85372: TAGCTAAAATGTTGCCCATAATATAAATAATTGCAGATGATTTAATTCAG  Hsapiens
  405053: .......................C..........................  Ptroglodytes
  422557: ..................................................  Ggorilla
  459849: ......................C...........................  Pabelli
  363668: ......................C....-......................  Nleucogenys
  449240: ......................C...........................  Mmulatta
  397999: ......................C...........................  Panubis
  428020: ........G.T.A.........C.......C......G.GC.........  Cjacchus
 1069103: ........G.T....A...C..C..............G.GC....G....  Sboliviensis
```

```
                                  DNA
          ------------------------------------------------------>
                   85431     85441    85450     85460     85470
                       :         :         :         :         :
   85422: GCAAATTGTGAGTTCTGTTGT-TTTAAAATAAAACAGTTCCATCATTCTT   Hsapiens
  405103: .....................-.................G..........   Ptroglodytes
  422607: .....................G...................G........   Ggorilla
  459899: .............G.......-..................T.........   Pabelli
  363717: ....T........GT......-............................   Nleucogenys
  449290: ...........A.G.......-............................   Mmulatta
  398049: ...........A.G.......-............................   Panubis
  428070: ...........A.G.......-............................   Cjacchus
 1069153: ...........A.G.......-..-----...........T....C....   Sboliviensis

                                  DNA
          ------------------------------------------------------>
                   85480     85490     85500     85510     85520
                       :         :         :         :         :
   85471: TACATGCATGCAGTGAAATCTAACCATGTGAGATCCACCATCGTTAATAA   Hsapiens
  405152: ..T...............................................   Ptroglodytes
  422657: ..................................................   Ggorilla
  459948: ..........................................A.......   Pabelli
  363766: .........................T..........G.....A.......   Nleucogenys
  449339: ..........................................AC......   Mmulatta
  398098: ..........................................AC......   Panubis
  428119: C.......C.................................A.......   Cjacchus
 1069197: ........................G.................A.......   Sboliviensis

                                  DNA
          ------------------------------------------------------>
                                            85528     85538     85548
                                                :         :         :
   85521: ATACAC----------------------ATGAAAAACCTATGAAATACAC   Hsapiens
  405202: ....T.----------------------......................   Ptroglodytes
  422707: ......----------------------........T.............   Ggorilla
  459998: ......ATGAAAAACCTATGAAATACAT.....................T   Pabelli
  363816: ..G...----------------------............A........A   Nleucogenys
  449389: ......----------------------......................   Mmulatta
  398148: ......----------------------......................   Panubis
  428169: ....------------------------.........--.......T...   Cjacchus
 1069247: ..--------------------------C........--.......T...   Sboliviensis

                                  DNA
          ------------------------------------------------------>
                   85558     85568     85578     85588     85598
                       :         :         :         :         :
   85549: ATGAAAAACCTCTTGAACATTCAGATATTTTACATCATTCCTTTTACCTC   Hsapiens
  405230: ..................................................   Ptroglodytes
  422735: .............C....................................   Ggorilla
  460048: ........................G.........................   Pabelli
  363844: ................................................CT   Nleucogenys
  449417: ..................G....................A.........T   Mmulatta
  398176: ..................G..............................T   Panubis
  428193: .C........C...C....C...............T.....-.......T   Cjacchus
 1069270: ......C...C...C....C.............................T   Sboliviensis
```

DNA

```
                  85608     85618     85628     85638     85648
  85599: GGTGTTATATTTCCACTTCACTTCCTCCAAAGAATTTTCTTCTAATTTAA  Hsapiens
 405280: ..................................................  Ptroglodytes
 422785: ..................................................  Ggorilla
 460098: .......C..........................................  Pabelli
 363894: A.....G.........C...T.............................  Nleucogenys
 449467: ....................T...................C.........  Mmulatta
 398226: ....................T...................C.........  Panubis
 428242: ....................T.............................  Cjacchus
1069320: ....................T.............................  Sboliviensis
```

DNA

```
                  85658     85668     85678     85688     85698
  85649: TATACTTTTACATTTGAAAGTATTTTATTGATAACCTTGCCATATTTCTC  Hsapiens
 405330: ..................................................  Ptroglodytes
 422835: ..................................................  Ggorilla
 460148: .................................................T  Pabelli
 363944: .................G..................C.............  Nleucogenys
 449517: ....................................C.............  Mmulatta
 398276: ....................................C.............  Panubis
 428292: ..C..................C...C..........C.............  Cjacchus
1069370: ..C............A.....C..............C..........T..  Sboliviensis
```

DNA

```
                  85708     85718     85728     85738     85748
  85699: TGAAATGAAAGTACGTACATGCATTTAAAATACAAAATGTTCTTATGACC  Hsapiens
 405380: .............T....................................  Ptroglodytes
 422885: .............T....................................  Ggorilla
 460198: ......C...........................................  Pabelli
 363994: .............G.........C..-....................G..  Nleucogenys
 449567: ...............................................G..  Mmulatta
 398326: ...............................................G..  Panubis
 428342: .A...........T...T.............................G..  Cjacchus
1069420: .............T.................................G..  Sboliviensis
```

DNA

```
                  85757     85767     85777     85787     85797
  85749: CAT-GGTCATTTTTCTATATGTTTAAAAAGTTTCATCTGGATTCAGTTAT  Hsapiens
 405430: ...-.......G......................................  Ptroglodytes
 422935: ...-.......G......................................  Ggorilla
 460248: ...-..C..C.A.....A...........-....................  Pabelli
 364043: .G.-.......-..T..A................................  Nleucogenys
 449617: ...-.......G.....A................................  Mmulatta
 398376: ...-.......G.....A................................  Panubis
 428392: ...G.......G.....A............C...................  Cjacchus
1069470: ...-.......G.....A............C...................  Sboliviensis
```

```
                                  DNA
          --------------------------------------------------->
               85803     85813     85823     85833     85843
                   :         :         :         :         :
  85798: ----CACTATACTTGTTGTTAGTGTACAATTGAAAATATATATATTTAAA  Hsapiens
 405479: ----...................A..........................  Ptroglodytes
 422984: ----...................A..........................  Ggorilla
 460296: ----....G..............A......G...................  Pabelli
 364091: ----...................A..............C...........  Nleucogenys
 449666: TGTAT....-.............A................C.........  Mmulatta
 398425: TGTAT....-.............A................C.........  Panubis
 428442: ----...................A.....C......A.-..........G  Cjacchus
1069519: ----...................A.....A......A--.C........G  Sboliviensis
```

```
                                  DNA
          --------------------------------------------------->
               85853     85863     85873     85883     85893
                   :         :         :         :         :
  85844: TTTTGATAAATAATTATTAAACATAAAAGCTAATTTTTTCTGTAGTTGCA  Hsapiens
 405525: .................................C................  Ptroglodytes
 423030: ..................................................  Ggorilla
 460342: ...........................T..........A...........  Pabelli
 364137: ......................................A...........  Nleucogenys
 449715: .....G...G...........................GA...........  Mmulatta
 398474: .....G...G...........................GA...........  Panubis
 428487: ...............C.......C.............CA....G......  Cjacchus
1069563: .......................C.............CA....G......  Sboliviensis
```

```
                    DNA
          -------------------------->
               85903     85913                85932     85942
                   :         :                    :         :
  85894: TCTCTTAATGAAGTGAATGAGAACAAAAA-TTTTTCTGTGGTCTTTCATT  Hsapiens
 405575: .............................-....................  Ptroglodytes
 423080: .............................-.........T..........  Ggorilla
 460392: .......................T.....-....................  Pabelli
 364187: .......................G.....-....................  Nleucogenys
 449765: ........-----................-....C...............  Mmulatta
 398524: ........-----................-....................  Panubis
 428537: ......T..........CA..........-....................  Cjacchus
1069613: ......T..........CA..........T....................  Sboliviensis
```

```
                                             DNA
                                  --------------------------->
               85949     85959     85969     85979     85989
                   :         :         :         :         :
  85943: CAGG---ACAATTTGGTAGGCAAAATACTTTTTAAAATGTATTGATTCTG  Hsapiens
 405624: ....---...................C.......................  Ptroglodytes
 423129: ....---........................................G..  Ggorilla
 460441: ....ACA......................................--...  Pabelli
 364236: ....ACA......................................C....  Nleucogenys
 449809: ....ACA....................A..........A......C.    Mmulatta
 398568: ....ACA....................A.................C....  Panubis
 428586: ....ACA...G..............C...................C....  Cjacchus
1069663: .G..ACA.TG....C..............................--...  Sboliviensis
```

```
                                      DNA
         -------------------------------------------------->
                  85999     86009     86019     86029     86039
  85990: CATCTTAGATAGCATCTTAGATCTTAATAATAGGAGATTAAAACATTAAT  Hsapiens
 405671: ............G.....................................  Ptroglodytes
 423176: ...............................................-..  Ggorilla
 460489: ............A..............................T......  Pabelli
 364286: A.C.........A.....................................  Nleucogenys
 398618: .........C----................................G...  Panubis
 428636: .......C....AC........G......................---..  Cjacchus
1069711: .......C....A.........G.......C...................  Sboliviensis

                                      DNA
         -------------------------------------------------->
                  86047     86057     86067     86077     86087
  86040: CATTT--TATAAGATAAAATATAATAAAGATGGCCTGTGATATGAGTAAA  Hsapiens
 405721: .....--...........................................  Ptroglodytes
 423225: A....--..................................G........  Ggorilla
 460539: .....--..................G........................  Pabelli
 364336: .G...--..................G.............C..........  Nleucogenys
 398664: .....TA..............G...G................G.......  Panubis
 428683: .....--.............T....G.G...A..T...A...G.......  Cjacchus
1069761: .....--.............G....G.G...C..T...A...G.......  Sboliviensis

                                DNA                  LINE1
         ------------------------------------------>------->
                  86097               86110     86120     86130
  86088: CATTTCATGGTAAAAA-------CTGTCATCTTACTTGAGCATTAGGACA  Hsapiens
 405769: ................-------..---......................  Ptroglodytes
 423273: ................-------..---......T...............  Ggorilla
 460587: ................-------T.---.A....................  Pabelli
 364384: ................-------..---......................  Nleucogenys
 398714: ................-------..---.......C..............  Panubis
 428731: ..........C.....ACA----..---......T...............  Cjacchus
1069809: ..........C.....AAAAAAG..---......T...............  Sboliviensis

                                     LINE1
         -------------------------------------------------->
                  86140     86150     86160     86170     86180
  86131: AATACTTAGTGCATGCAGGTCTTAAAACCTAGATGACGGGTTGACAGGTG  Hsapiens
 405809: ........A.........................................  Ptroglodytes
 423313: ........A.........................................  Ggorilla
 460627: ........A.......G................A..........T.-...  Pabelli
 364424: ........A.......G............C......T.......T.....  Nleucogenys
 450006:             ..A..............C......T......CT.....  Mmulatta
 398754: .....CA.A..T.................C......T......CT.....  Panubis
 428774: ..C..C..A...G..AG..G.........-..............TG....  Cjacchus
1069856: ..C..C..A......AG..G....G....-.C............TG....  Sboliviensis
```

```
                                    LINE1
          ------------------------------------------------------>
                86190     86200     86210     86220     86230
   86181: CAGCAAACCACCATGGCACATGTATACCTGTGTAACAAACCTGCACGTTC  Hsapiens
  405859: .........................................C........  Ptroglodytes
  423363: .........................................C........  Ggorilla
  460676: ..................................................  Pabelli
  364474: ..................................................  Nleucogenys
  450044: .............................................T.C..  Mmulatta
  398804: .............................................T.C..  Panubis
  428823: ...................G.........A................T...  Cjacchus
 1069905: .......................G.....A....................  Sboliviensis

                                 LINE1                        DNA
          ------------------------------------------->  ------>
                86240     86250     86260     86270     86279
   86231: TGTACATGTAGCCCAGAACTTAAAGTGAAATAAAAATAATAAAA-ATAAA  Hsapiens
  405909: .........................CA..............T..-.....  Ptroglodytes
  423413: ....T.....................A...C.............-.....  Ggorilla
  460726: ....................CG.TCCA..CC...TGG..G.G..-.      Pabelli
  364524: ..........................A.G.........G.....-...C.  Nleucogenys
  450094: ..........................A.................A.....  Mmulatta
  398854: ..........................A.................A.....  Panubis
  428873: ..A.......................A........G.C......T.....  Cjacchus
 1069955: ..A.......................A....C...G........C.....  Sboliviensis

                                    DNA
          ------------------------------------------------------>
                86289     86299     86309     86319     86329
   86280: TAAATAAAAACTATCTTATTTGATAAAATAGCTCTTATATACTTATTAAA  Hsapiens
  405958: ...............................T..................  Ptroglodytes
  423462: ....C..........................T..................  Ggorilla
  364573: .............................G.T.........T........  Nleucogenys
  450144: ...............................T.........T........  Mmulatta
  398904: ...............................T.........T........  Panubis
  428923: ..................C......C.....T.-.......T.....T..  Cjacchus
 1070005: ...............................T.-.......T........  Sboliviensis

                                    DNA
          ------------------------------------------------------>
                86339     86349     86359     86369     86379
   86330: TAATACACAGCACTCTTGAAAGAAATTATAAAATGTAATGGGTTTTATTC  Hsapiens
  406008: ..................................................  Ptroglodytes
  423512: ..................................................  Ggorilla
  364623: .............A..............C.....................  Nleucogenys
  450194: .............A..............C...........A.........  Mmulatta
  398954: .............A..............C...........A.........  Panubis
  428972: ............AAT.......C....GC...G.................  Cjacchus
 1070054: ............AA.............GC.....AC..............  Sboliviensis
```

```
                                       DNA
                86389     86399     86409     86419     86429
   86380: AACGTAGCTTAAAAATATATACTCAGTAAAACATCCCAGTTAAATTTAAA  Hsapiens
  406058: ...T..............................................  Ptroglodytes
  423562: ..T...............................................  Ggorilla
  364673: .......................................A..........  Nleucogenys
  450244: .GT................................T...C..........  Mmulatta
  399004: .GT................................T..............  Panubis
  429022: ........A.-..........T............................  Cjacchus
 1070104: ..T.....A............T...........C....C...........  Sboliviensis

                                       DNA
                86439     86449     86459     86469     86479
   86430: ATAATCAGTCACATAGTATATACTGCTTTACCTGATTGGGTATTTTTCTT  Hsapiens
  406108: ..................................................  Ptroglodytes
  423612: ..................................................  Ggorilla
  364723: ...............AC.................................  Nleucogenys
  450294: ...............AC............G....................  Mmulatta
  399054: ...............AC.......................A.........  Panubis
  429071: T..-...........AC...............C.................  Cjacchus
 1070154: ...-...........AC............G.....C.........C....  Sboliviensis

                                       DNA
                86489     86499     86509     86519     86529
   86480: ATACTAGGGTTAAGTATTAAGAAGAGTAGAAAGGAATTTGTCACATTTAA  Hsapiens
  406158: ......................................CA..........  Ptroglodytes
  423662: ......................................C...........  Ggorilla
  364773: ........A.............................CA..........  Nleucogenys
  450344: .C....................................------------  Mmulatta
  399104: .C....................................------------  Panubis
  429120: ....G.............G...G...............C...C.......  Cjacchus
 1070203: CC............A...G...G...............C...........  Sboliviensis

                                       DNA
                86539                   86557     86567     86577
   86530: ATTCATTGGCAAAAAAAAA--AAAAAAAAAACACCAGGTAGAGTATTCAG  Hsapiens
  406208: ...................AC.............................  Ptroglodytes
  423712: .......T.G-----------C............................  Ggorilla
  364823: ...............------C..G...........T.............  Nleucogenys
  450382: ---......T.........A-.......TCC.....T.............  Mmulatta
  399142: ---......T........---.......TCC.....T.......G.....  Panubis
  429170: ....G.G..------------GGG....------..C........GC...  Cjacchus
 1070253: ......G..------------GGG....------.GTT....A.......  Sboliviensis

                                       DNA
                86587     86597     86607     86617     86626
   86578: TAGCTCTTGTAGATGTTCTTTTGCTTTGGTTTGCAGAGGGA-TGCAATTT  Hsapiens
  406258: ..................................G......-........  Ptroglodytes
  423751: .........................................-........  Ggorilla
  364867: .......................................A.-........  Nleucogenys
  450428: ..A......C...........C...........TG....A.-........  Mmulatta
  399186: ..A......C...........CA..........TG....A.-........  Panubis
  429203: C.C..TC........G.....C......-........A...AC.......  Cjacchus
 1070286: .....TC..C..................-.............AG.......  Sboliviensis
```

```
                          DNA
              86636     86646     86656     86666     86676
  86627: GACAAAAGTCAAGGATTGGAGGAGGCTGAGGTATGAATGGAAAATTGTCA Hsapiens
 406307: .................................................. Ptroglodytes
 423800: .................................................. Ggorilla
 364916: .................A..A............................. Nleucogenys
 450477: ........A...................................C..... Mmulatta
 399235: ........A...................................C..... Panubis
 429252: ...G....C......................................... Cjacchus
1070335: ..........................C...A................... Sboliviensis

                DNA
              86686     86696     86706     86716     86726
  86677: TTACTCCCACCACTGCCCTGTAGGAGAGGAGTCCCCTCTCACATTTTCCT Hsapiens
 406357: .............................................C.... Ptroglodytes
 423850: ............G..................................... Ggorilla
 364966: ..G.....................C......................... Nleucogenys
 450527: .....................C........T................... Mmulatta
 399285: .....................C........T................... Panubis
 429302: ..........T...T.......AA......A................... Cjacchus
1070385: ..........T...........AA...C...A.T.....T.......... Sboliviensis

              86736     86746     86756     86766     86776
  86727: ACCTACTTTTCTGCAGTTTTAGTTTACTATGGCAAGCTTCTAGTATTTTG Hsapiens
 406407: ....G............................................. Ptroglodytes
 423900: ....................G............................. Ggorilla
 365016: ..A..............................................C Nleucogenys
 450577: ..A............................................... Mmulatta
 399335: ..A............................................... Panubis
 429352: ..A...A......G..........G...........T...C......... Cjacchus
1070435: ..AC..A.................G.......T...T...C......... Sboliviensis

              86786     86795     86805     86815     86825
  86777: GTAATTTTTTTAAAG-TAGTAACTTTTAAAGAAGCAAAGCTAAGCATCCC Hsapiens
 406457: ..............--C................................. Ptroglodytes
 423950: ..............A-.................................. Ggorilla
 365066: .........AA...A-.................................. Nleucogenys
 450627: ..G...........A-.......G.......................... Mmulatta
 399385: ..G.......A...A-.......G.......................... Panubis
 429402: .........C....AA.................................. Cjacchus
1070485: .........C....AA..T............................... Sboliviensis

              86835     86845     86855     86865     86875
  86826: TGGAATAATATTATAAATCAAAACAAATATTGACCAAATATTCAATTTAA Hsapiens
 406505: .................................................. Ptroglodytes
 423999: ..........................G....................... Ggorilla
 365115: .............C...............................C.... Nleucogenys
 450676: ....G.....................................T.T..... Mmulatta
 399434: ....G.......................................T..... Panubis
 429452: C.A...G...........A....A...C......TG..C.G........G Cjacchus
1070535: ..................A........C....----....G........G Sboliviensis
```

```
                86885     86895     86905     86915     86925
   86876: TGCAGTGGTTTTCAGGAACAATTTTTTAACATAAACTATAATTAATGTAC  Hsapiens
  406555: .............................................C....  Ptroglodytes
  424049: A.................................................  Ggorilla
  365165: ..........................C.......................  Nleucogenys
  450726: .................G................................  Mmulatta
  399484: C.................................................  Panubis
  429502: ............T.......G.....-..T.......G........T...  Cjacchus
 1070581: ............T.....T.G.....-..TG...............T...  Sboliviensis

                                    86935     86945     86955
   86926: ATGATGT--------------------TAGAGATGACACCCCTGGGACAA  Hsapiens
  406605: .......--------------------.......................  Ptroglodytes
  424099: .......--------------------.......................  Ggorilla
  365215: .......--------------------.......................  Nleucogenys
  450776: .......--------------------...................G...  Mmulatta
  399534: .......--------------------......C............G...  Panubis
  429551: ..A..T.GATCATTGACCCTCGTATTT......C........-A......  Cjacchus
 1070630: .....T.GATCATTGACCCTCATATTT...............-.......  Sboliviensis

                86965     86975     86985     86995     87005
   86956: GGTCAAGGCCAGGAGTTTTCTCATGCAGCAACAGAGAAGTACTCATAAGT  Hsapiens
  406635: ..................................................  Ptroglodytes
  424129: ..................................................  Ggorilla
  365245: ........A..........                                 Nleucogenys
  450806: ........A...................................G.....  Mmulatta
  399564: ........A...................................G.....  Panubis
  429600: ......A.G.G.....G........T..TG..............C.....  Cjacchus
 1070679: ......A.G.G.....G........T..T..........G..........  Sboliviensis

                87015     87025     87035     87045     87052
   87006: ATAGGAGTAGGTTTCATCCAGCCTGGGCAACTGAGGGACTGAAGG---GG  Hsapiens
  406685: .............................................---..  Ptroglodytes
  424179: .............................................---..  Ggorilla
  450856: ................C..G..T.CA..................AAGT..  Mmulatta
  166397:                 ...........TG..A......              Panubis
  399614: ..---...........                                    Panubis
  399649:                                       ......AAGT..  Panubis
  429650: ..........A.......AG..T.....................AGGGAA  Cjacchus
 1070729: .C..A.....A.......AG..T.....................AAGGAA  Sboliviensis

                87062     87072     87082     87092     87102
   87053: GTAATGGTGGCTGTTCCTGGAAGTCCTGGAGAAAGTTATGTTAAAGGCAG  Hsapiens
  406732: ...................................G..............  Ptroglodytes
  424226: ...................................G..............  Ggorilla
  450906: .........A..TC.....................G..............  Mmulatta
  399661: .........A..TC....................CG..............  Panubis
  429700: ..G......A.CAC---------............A......G.......  Cjacchus
 1070779: ..G......A...C.T...................A..............  Sboliviensis

                87112     87122     87132     87142     87152
   87103: GACCATTTCCAGGAAAGCAGAAGCTGGTTCTAGAGGCGATGAGATGGGAA  Hsapiens
  406782: ..................................................  Ptroglodytes
  424276: .........................A........................  Ggorilla
  450956: .....................G...............A............  Mmulatta
  399711: .....................G..............AA............  Panubis
  429741: ..TT.......A...G.....G...............AG...........  Cjacchus
 1070829: ...........A...G.....G...............A............  Sboliviensis
```

```
              87162     87172     87182     87191     87201
                  :         :         :         :         :
  87153: GAGTGTGATGAGGTGACTGGCTCAGAAATTTGG-CTCAGAGGATGGCGCT   Hsapiens
 406832: .GC..........G........T..........-................   Ptroglodytes
 424326: .G...........G...................-................   Ggorilla
 451006: .G...........G..T................-............T...   Mmulatta
 399761: .G...........G..T................-............T...   Panubis
 429791: .G......C....G..T................G................   Cjacchus
1070879: .G......C....G..T................G................   Sboliviensis

              87211     87221     87226     87236     87246
                  :         :         :         :         :
  87202: TAACAAGTCACCTCTCACCTAAAA-----TAGACCTTGGTAAACCTCATT   Hsapiens
 406881: ........................-----..................T..   Ptroglodytes
 424375: ........................-----..................T..   Ggorilla
 451055: ........................-----.G................T..   Mmulatta
 399810: ........................-----.G................T..   Panubis
 429841: ...........T.......C....ATAAAGG............T...T..   Cjacchus
1070929: ..........AT.......C....ATAAAGG............T...T..   Sboliviensis

              87256     87266     87276     87286     87296
                  :         :         :         :         :
  87247: TGTCAGTAAGTCCCTACTTCAGGCAAGGCGCTAGAGACTTCTCACTTAGC   Hsapiens
 406926: .....A......................T.....................   Ptroglodytes
 424420: .....A......................T.....................   Ggorilla
 451100: .A...A......................T.........G.......C.A.   Mmulatta
 399855: .A...A......................T.................C...   Panubis
 429891: ..........................TCT....CG.......TG.G.G..   Cjacchus
1070979: .....A....................T.T....C.............G..   Sboliviensis

              87306     87316     87326     87336     87346
                  :         :         :         :         :
  87297: CTACCGTTTAGGCATAGACAATAACTAAGAATCCATTTTATAAAGAAAAG   Hsapiens
 406976: ..................................................   Ptroglodytes
 424470: ................................A.................   Ggorilla
 451150: .C...A...........................................C   Mmulatta
 399905: .C...A...........................................A   Panubis
 429941: .....A.........G................................G.   Cjacchus
1071029: .....A.........G................................G.   Sboliviensis

              87356     87366     87376     87386     87396
                  :         :         :         :         :
  87347: ATCACTGAAGAACCCTTAAAAATAGACAATCAAACATAGTATGTAACCAT   Hsapiens
 407026: ......................C...........................   Ptroglodytes
 424520: ..................................................   Ggorilla
 451200: ...........G......................................   Mmulatta
 399955: ...........G......................................   Panubis
 429991: ......A...GG.........--..........................A   Cjacchus
1071079: ..........GG-G.......--...TGC............C..G.....   Sboliviensis

              87406     87416               87429     87439
                  :         :                   :         :
  87397: CCCTGGATCCCATTTGGTCCTCAAGAAG-------CATTGATCAAAATGT   Hsapiens
 407076: ............................-------.G..C..........   Ptroglodytes
 424570: ............................-------.G..C..........   Ggorilla
 451250: .T..........................-------....C...G......   Mmulatta
 400005: .T..........................-------....C...G......   Panubis
 430039: .....AG.....................CAAATGT..A.C..........   Cjacchus
1071126: .....AG.....................-------....C..........   Sboliviensis
```

```
              87449     87459     87469     87479     87489
                  :         :         :         :         :
  87440: CAAGCAGCCAAAAATGATTCCTGGTTGCAGCCAGACTTGTTGCATAGAGG   Hsapiens
 407119: ..................................................   Ptroglodytes
 424613: ......A...........................................   Ggorilla
 451293: .............................T.TG......A..........   Mmulatta
 400048: .............................T.TG......A..........   Panubis
 430089: ...T.................G.................G..T.......   Cjacchus
1071169: .....................C.................G..AT......   Sboliviensis

              87499     87509     87519     87529     87539
                  :         :         :         :         :
  87490: ATAATGTAGCGCTGTGGTTATGTGAAGGCATTCCAAAGAACAGCACTTGT   Hsapiens
 407169: ...........................................A......   Ptroglodytes
 424663: ..................................................   Ggorilla
 451343: ..........T.........C........G....................   Mmulatta
 400098: ..........T.........C........G....................   Panubis
 430139: ........C.T..AC.....CA....-C......................   Cjacchus
1071219: ........C.T..A......A.............................   Sboliviensis

              87549     87559     87569     87578     87588
                  :         :         :         :         :
  87540: TCTACAGAAATATTCACAGAGCCATGTAACAGAAG-GAGAGGAAGCAGGG   Hsapiens
 407219: ...................................-..............   Ptroglodytes
 424713: ...................................-..............   Ggorilla
 451393: ........................C..........A.G.....G......   Mmulatta
 400148: ........................C..........A.G.....G......   Panubis
 430188: ..............A...........G........A.......G....A.   Cjacchus
1071269: ...........G..A............C.......A.......G....C.   Sboliviensis

              87598     87607     87617     87627     87637
                  :         :         :         :         :
  87589: TAATTGTTCTGATATGAT-AAAAAAGTTACAGAACACATTTTTCACTAAG   Hsapiens
 407268: ..................-...................C...........   Ptroglodytes
 424762: ..................A...................C......G....   Ggorilla
 451443: ........T.........--..............T..G............   Mmulatta
 400198: ..................--..............T...............   Panubis
 430238: .............GCC.C--..C...........T...............   Cjacchus
1071319: ..............CC..--..C...........................   Sboliviensis

              87647     87657     87667     87677     87687
                  :         :         :         :         :
  87638: GACTGATAAAAATAATGTTAAATACCAATCAATTTTAAAAATGTTAAATA   Hsapiens
 407317: ..................................................   Ptroglodytes
 424812: ....................................T.............   Ggorilla
 451491: ..................A.....T.......-.................   Mmulatta
 400246: ....A.............A.....T.......-.................   Panubis
 430286: .......T....CC....A.....T..........A.......C......   Cjacchus
1071367: .......T....CC....AC....T..................C......   Sboliviensis

              87697     87707     87717     87727     87737
                  :         :         :         :         :
  87688: TCGATCCAAACCAAATGGAAGAGAAAGTTTGAATTAAGGGAGTGGAGATG   Hsapiens
 407367: ..................................................   Ptroglodytes
 424862: ..................................................   Ggorilla
 460745: ...........................G......................   Pabelli
 451540: ..........G...........A.G..G.............A.....G..   Mmulatta
 400295: ..........A................G.............A.....G..   Panubis
 430336: CT......T.GA.........G.....G.................G....   Cjacchus
1071417: CT.....C..GA.........G.....G......................   Sboliviensis
```

```
              87747     87757     87767     87777     87787
                  :         :         :         :         :
   87738: GCAGATGTCAGAGAAGGGTTACCGCACAGAGCTGGGAAAGAAGGAAGGGA   Hsapiens
  407417: .......G..........................................   Ptroglodytes
  424912: ..................................................   Ggorilla
  460795: .G...............................C...G............   Pabelli
  451590: .G..................T.G..............G...CA.......   Mmulatta
  400345: .G.............C....TTG..............G...CA.......   Panubis
  430386: .G....T......G...AA.CGGA.............G......G.....   Cjacchus
 1071467: .G...............AA.CGGA.............G......G.....   Sboliviensis

              87797     87807     87817     87827     87837
                  :         :         :         :         :
   87788: AAAGCGCAAAGTTGAGAGAAACAGATGGCAGAACTTCCAGGTTGAATCCA   Hsapiens
  407467: ........................T..A......................   Ptroglodytes
  424962: .....A.....................A......................   Ggorilla
  460845: ....G......C.............C.A......................   Pabelli
  451640: .G..G......C...............A..................G...   Mmulatta
  400395: .G..G......C...............A..................G...   Panubis
  430436: .GT.G..C...C....G.....G....A.CT.G.............C...   Cjacchus
 1071517: .G..G......C....G........C.A.TT...............C...   Sboliviensis

              87847     87857     87867     87877     87887
                  :         :         :         :         :
   87838: TTAGATCACCCAAGGAGAATGAGAGAAGAACGTCTGGGATAGATGCCTGG   Hsapiens
  407517: ...........................................CA.....   Ptroglodytes
  425012: ...............................T...........C......   Ggorilla
  460895: ..............................T...C............C..   Pabelli
  451690: .......GT................GCA..TA..................   Mmulatta
  400445: .......GT................G.A..TA..................   Panubis
  430486: .................C.........A..TA.A................   Cjacchus
 1071567: ............T..............A..T..A................   Sboliviensis

              87897     87907     87917     87927     87937
                  :         :         :         :         :
   87888: GAAATGTCTGCATGTAAGGGGTGGCAATGGAGAAGGAGTTGAAGACCGGA   Hsapiens
  407567: ............C.............G.......................   Ptroglodytes
  425062: ............C.............G...................T...   Ggorilla
  460945: ............C.............G.......................   Pabelli
  451740: ............A......A......G..........A..........A.   Mmulatta
  400495: ............A......A......G..........A..........A.   Panubis
  430536: ............A..C....A....CG....................A..   Cjacchus
 1071617: ............A.......A.....G....................A..   Sboliviensis

              87947     87957     87967     87977     87987
                  :         :         :         :         :
   87938: AGTCAGAGATGTACCGGAGAACAAAATACAGAATTCCGAGGAAGAGGGGT   Hsapiens
  407617: ..............A...................................   Ptroglodytes
  425112: ..............A...................................   Ggorilla
  460995: ..............A.....................T..A...C......   Pabelli
  451790: ............G.A.....G................A............   Mmulatta
  400545: ............G.A.....G................A............   Panubis
  430586: ........G.....A.......G...G......C..AA.......T...G   Cjacchus
 1071667: T.............A.....G.....G.T....C..AA..........AG   Sboliviensis
```

```
              87997     88007     88017     88027     88037
   87988: CAACAGAGACAAATAGGTTCAAAATTTCAGTGAGAGACAATAAATTTTGA Hsapiens
  407667: ................................G................. Ptroglodytes
  425162: .................A................................ Ggorilla
  461045: ...............................A.AG.............T. Pabelli
  451840: .........A..............A.........G........C...... Mmulatta
  400595: ........................A.........G........C...... Panubis
  430636: ...................TG...A..----------------.C.CCAG Cjacchus
 1071717: ...................TG...A..----------------....A.G Sboliviensis

              88047     88057     88067     88077     88087
   88038: TGCACAGAAGTTATTATCAGGAAGCTATTAGTAAGTCACCAAAGAGATTG Hsapiens
  407717: .................................................. Ptroglodytes
  425212: .................................................. Ggorilla
  461095: .................................................. Pabelli
  451890: ......................G........................... Mmulatta
  400645: .................T....G........................... Panubis
  430671: .T..G.....G......T....G........................... Cjacchus
 1071752: .A..G.....G......T....G...G....................... Sboliviensis

                            LINE1
              88097     88106     88116     88126     88136
   88088: TTTTTCATGAG-TTTTTAAAATTTTGGTAAAATATGCAGAACATAAAATT Hsapiens
  407767: ........T..-...................................... Ptroglodytes
  425262: ........T..-...................................... Ggorilla
  461145: ...........-...................................... Pabelli
  451940: .C......A..-...................................... Mmulatta
  400695: .C......A..-...................................... Panubis
  430721: ...........-.....T.....-----------.A.....T.....CA. Cjacchus
 1071802: ...........T.....T.................A.....T......A. Sboliviensis

                          LINE1
              88146     88156     88166     88176     88186
   88137: CACCATTTTAACCATTTTTAAGTGTACTTTCAGTGTGGTCTTAAGTACAT Hsapiens
  407816: .................................................. Ptroglodytes
  425311: .................................................. Ggorilla
  461194: ...........................A.C.................... Pabelli
  451989: ....-......................A...................... Mmulatta
  400744: ....-......................A...................... Panubis
  430759: ...T........A.........-..C.A....--..A..A.......T.. Cjacchus
 1071852: ...TGC......A..G.......C...A....--..A..A.......T.. Sboliviensis

                          LINE1
              88196     88206     88216     88226     88236
   88187: CCACATGTTGTGTAACCATCACCATCATCCATCTCCTGAACTTCTTTATT Hsapiens
  407866: ...........................A...................... Ptroglodytes
  425361: ...........................A...................... Ggorilla
  461244: ........................C......................... Pabelli
  452038: ........................C......................... Mmulatta
  400793: ........................C......................... Panubis
  430806: ............A..................................... Cjacchus
 1071900: .................................G.....G.......... Sboliviensis
```

```
                          LINE1                  Alu
                  88246     88254     88264     88274     88284
  88237: GTCTCAAACTGAAACTC--TATCATCTGGGTGTGGTGGCTCACACCTGTA  Hsapiens
 407916: .................TG...............................  Ptroglodytes
 425411: .................TG...............................  Ggorilla
 461294: A................TG............................A..  Pabelli
 452088: ..............T..TG..........A.........C...T......  Mmulatta
 400843: ..............T..TG..........A.........C...T......  Panubis
 430856: .............G...TG.......CA...........C..........  Cjacchus
1071950: .................TG.......CA...........G..........  Sboliviensis

                                   Alu
                  88294     88304     88314     88324     88334
  88285: ATCTCAGCACTTTGGAAGGCCGAGCCAGGAGGATCACCTGAGGCCACAAG  Hsapiens
 407966: ..................A...............................  Ptroglodytes
 425461: ..................A..A............................  Ggorilla
 461344: ...C...........G..................................  Pabelli
 452138: ..TC..............T.......................T.......  Mmulatta
 400893: ..TC..............T..............C................  Panubis
 430906: ...C...........G.....A..G....TTC..................  Cjacchus
1072000: ...C...........G.....A..G....TT...................  Sboliviensis

                                   Alu
                  88344     88354     88364     88374     88384
  88335: TTTGAGACCAGCCTGGGCATTGTAGTGAAACCCTGTCTCTATGTAAAAAT  Hsapiens
 408016: ..C............A............G.....................  Ptroglodytes
 425511: ..C.........................G.....................  Ggorilla
 461394: ..CA.......A................G...............G.....  Pabelli
 452188: ..C.................C.......G.....A.....T....TT...  Mmulatta
 400943: ..C.................C.......G.....A.....T....TT...  Panubis
 430956: ...................A........G.T...........A..GT...  Cjacchus
1072050: ...................AC.......G.T............C..T...  Sboliviensis

        Alu                                           LINE1
                                    88391     88401     88411
  88385: AAT-----------------------AATAATAATAATAAAAGAATCTCT  Hsapiens
 408066: ...--------------------------.....................  Ptroglodytes
 425561: ...--------------------------.....................  Ggorilla
 461444: ...--------------------------.......--....A.......  Pabelli
 452238: ...------------------------------.......T.........  Mmulatta
 400993: ...------------------------------.......T.........  Panubis
 431006: G..GAGATGATGATGATGATGATGATG..G..G..G.........A....  Cjacchus
1072100: G..-----------------GAGAATG..G.GG..G....G....A....  Sboliviensis

                                  LINE1
                  88421     88431     88441     88451     88461
  88412: GTATCCATTACACAGCATCTTCCTATTCTCCCATTCCCCCAGCCCATGAT  Hsapiens
 408090: .............................................G....  Ptroglodytes
 425585: ..................................................  Ggorilla
 461466: ..............................T........T..........  Pabelli
 452258: ..........................................A.......  Mmulatta
 401013: ..........................................A.......  Panubis
 431056: T........................................T........  Cjacchus
1072133: ...............................T.............G....  Sboliviensis
```

```
                                        LINE1
                 ------------------------------------------------->
                      88471     88481     88491     88501     88511
     88462: AACCACTATTTTACTTTATGTCTCTGTGAATTTTTCTATTCTAGGTCCCT  Hsapiens
    408140: .................G................................  Ptroglodytes
    425635: .................G................................  Ggorilla
    461516: .......G.................A........................  Pabelli
    452308: ........................C.........................  Mmulatta
    401063: ..T.....................CA........................  Panubis
    431106: ...T...C....G........................C......A.....  Cjacchus
   1072183: ...T...C....G...C....................G......A.....  Sboliviensis
```

```
                                        LINE1
                 ------------------------------------------------->
                      88521     88531     88541     88551     88561
     88512: CACATAAGTGGAATCATGTGTCCTTTTGTTTCCGGCAATATGTGTCCTTC  Hsapiens
    408190: ..................................................  Ptroglodytes
    425685: ..................................................  Ggorilla
    461566: ...................C............T.................  Pabelli
    452358: ................................T.................  Mmulatta
    401113: ..................................................  Panubis
    431156: ......GA.......----------------------.............  Cjacchus
   1072233: ..............T----------------------.C...........  Sboliviensis
```

```
                                        LINE1
                 ------------------------------------------------->
                      88571     88581     88591     88601     88611
     88562: TGTTTCCGGCTTATTTCACTCAGCATAACGTTTTCAGGGTTGGTCTCTGT  Hsapiens
    408240: ......T..........................................-  Ptroglodytes
    425735: .......A..........................................  Ggorilla
    461616: ......T......................A...............C....  Pabelli
    452408: ......T.T.........................G..........C....  Mmulatta
    401163: ......T.T.........................G..........C....  Panubis
    431184: ......T.....................T.........A...A..C....  Cjacchus
   1072261: C.....T................A.....A............A..C....  Sboliviensis
```

```
                                        LINE1
                 ------------------------------------------------->
                      88621     88631     88641     88651     88661
     88612: TGTAGCGTGTATCAGGATGTTATTTCTTTTTAAGGCTGAATAGTATTCCA  Hsapiens
    408289: ......A...............C...........................  Ptroglodytes
    425785: ......A...............C...........................  Ggorilla
    461666: ......A...........................................  Pabelli
    452458: ......A..C........................AG......A.....T.  Mmulatta
    401213: ..C...A...........................A.......A.....T.  Panubis
    431234: ......A..--.......A..G...................C........  Cjacchus
   1072311: ..C...A...........A..G...................C........  Sboliviensis
```

```
                                        LINE1
                 ------------------------------------------------->
                      88671     88681     88691     88701     88711
     88662: TTGTCAGTATATACCATAATATATACCATGGACATTTGGGTTGTTTACAC  Hsapiens
    408339: ..................G...............................  Ptroglodytes
    425835: ..................G...............................  Ggorilla
    461716: ..................G.........C.........A...........  Pabelli
    452508: ..................G...............................  Mmulatta
    401263: ..................G...............................  Panubis
    431282: C.................------------........A...........  Cjacchus
   1072361: C.................------------........A...........  Sboliviensis
```

LINE1

```
88721 88731 88741 88751
   88712: CTTTTGTTTATTTTGAATAGCGCCACAATGAACATTGGTGTACAA-----  Hsapiens
  408389: .............................................-----  Ptroglodytes
  425885: .........G................................A..-----  Ggorilla
  461766: ..................................C..........-----  Pabelli
  452558: ....................T.T......................-----  Mmulatta
  401313: ............C.......T.T......................-----  Panubis
  431320: ......G.............TA-.G....A...............-----  Cjacchus
 1072399: ......G.............T.A......A..T............ATATA  Sboliviensis
```

LINE1

```
88765 88775 88785
   88757: ---------------------ATATATATTCGAGTCCCCGTTTTCAATTA  Hsapiens
  408434: ---------------------.................T...........  Ptroglodytes
  425930: ---------------------.................T...........  Ggorilla
  461811: ---------------------..................A..........  Pabelli
  452603: ---------------------..............G..T..........C  Mmulatta
  401358: ---------------------..............G..T..........C  Panubis
  431364: -----ATATATATATATTTAC.........TA......T..........C  Cjacchus
 1072449: TATATTTACATATATATTTAT..T..C...TA...T..T......G.A.C  Sboliviensis
```

LINE1

```
88795 88805 88815 88825 88835
   88786: TTTTGTATATACGCCTGGAAGTAGGATTGCTGGATTATACGTTAATCCTG  Hsapiens
  408463: ..............................................T..A  Ptroglodytes
  425959: ......G.......................................T..A  Ggorilla
  461840: ......G.....A..........................T......T..A  Pabelli
  452632: ......G.....A...........................A.....T..A  Mmulatta
  401387: ......G.....A...........................A.....T..A  Panubis
  431409: ......G....TA..........A.G.....A....G..T......T..A  Cjacchus
 1072499: ......G....TA..........A.G.....A.......T......T..A  Sboliviensis
```

LINE1

```
88845 88855 88865 88882
   88836: TATTTAATTATTTGAGGAACTGCTCTATTGATGTCCAC---AGCTACTGC  Hsapiens
  408513: ......................................---.........  Ptroglodytes
  426009: ......................................---.........  Ggorilla
  461890: .....................A..G.............---.........  Pabelli
  452682: ......G.................G.............---.......T.  Mmulatta
  401437: ......G.................G.............---.......T.  Panubis
  431459: ........................A.............AAA...C.....  Cjacchus
 1072549: CT.....C................G.............---..GC.....  Sboliviensis
```

LINE1

```
88892 88902 88912 88922 88932
   88883: ACCAATTTACATTTCCACCAGCAATGCGATTGCTAGAGTTCCAATTTCTC  Hsapiens
  408560: ..................................................  Ptroglodytes
  426056: ................................T.G...............  Ggorilla
  461937: ...........................A......................  Pabelli
  452729: .............C.............A...T...........G......  Mmulatta
  401484: .............C.............A...T....G......G......  Panubis
  431509: .............C.............AG.....................  Cjacchus
 1072596: .............C.............A......................  Sboliviensis
```

```
                      LINE1                      LINE1
          --------------------------->          ---->
                 88942     88952     88962     88972     88982
     88933: TACATCATCACCACCACTTGCTATTTTCTTATTCTCAATGTGGTTCTGAA  Hsapiens
    408610: ...............G.............G.C..................  Ptroglodytes
    426106: ...G...........G.............G....................  Ggorilla
    461987: ...............G.............G..........C.........  Pabelli
    452779: ........T....T........G......G...............T....  Mmulatta
    401534: ........T....T........G......G...............T....  Panubis
    431559: ....                                                Cjacchus
   1072646: ....                                                Sboliviensis

                                 LINE1
          ------------------------------------------------->
                 88992     89002     89012     89022     89032
     88983: GTTCAGCATCTTTTCATGTAATCATTGGCCACCTGTATCTTTGGAAAATT  Hsapiens
    408660: ................................................A.  Ptroglodytes
    426156: ................................................A.  Ggorilla
    462037: ................................................A.  Pabelli
    452829: ........................C........A..........G...A.  Mmulatta
    401584: ........................C........A..........G...A.  Panubis

                                 LINE1
          ------------------------------------------------->
                 89042     89052     89062               89078
     89033: GTCTGTTAAAATCCCTTTTCTCACTATTAAATTGGG----TTGTTTGTTG  Hsapiens
    408710: ....A.....G.........................----..........  Ptroglodytes
    426206: ....A.....G.........................----..........  Ggorilla
    462087: ....A.....G.........................TTGT..........  Pabelli
    452879: .C..A.....G...T.......T.............----..........  Mmulatta
    401634: .C..A.....G...T.......T.............----..........  Panubis

                                 LINE1
          ------------------------------------------------->
                 89088     89098     89108     89118     89128
     89079: TTGTTGAGTAGAAAGAGTGCTTTATATATTTTGGATATGAATTATATATC  Hsapiens
    408756: ..................................................  Ptroglodytes
    426252: ..................................................  Ggorilla
    462137: ..................................................  Pabelli
    452925: ....A....----T......................G.............  Mmulatta
    401680: ....A....----T......................G.............  Panubis

                                 LINE1
          ------------------------------------------------->
                 89138     89148     89158     89168     89178
     89129: AAATTATTATTTGTAAGTATTTTCTCTCATTCTGTACATTACTTTTTAAC  Hsapiens
    408806: ..................................................  Ptroglodytes
    426302: ..................................................  Ggorilla
    462187: ..................................C...............  Pabelli
    452971: ................A...........G........G............  Mmulatta
    401726: ................A...........G........G............  Panubis
```

```
                              LINE1
        ------------------------------------------------------>
                 89188     89198     89208     89218     89228
                     :         :         :         :         :
  89179: TATGTCTTGATAGTGTCCTTTGAGGCACAAAAGTTATTAATCTTAATGAA  Hsapiens
 408856: ........A.........................................  Ptroglodytes
 426352: ..................................................  Ggorilla
 462237: ........A.........................................  Pabelli
 453021: .......................T..........................  Mmulatta
 401776: .......................T..........................  Panubis

                              LINE1
        ------------------------------------------------------>
                 89238     89248     89258     89268     89278
                     :         :         :         :         :
  89229: GCCAAATTAATCTATTTTCTCTTTTGGTTATGGTGCTTTTGGTGTCATAT  Hsapiens
 408906: .......................................CA.........  Ptroglodytes
 426402: ..................................................  Ggorilla
 462287: ................A......................CT.........  Pabelli
 453071: .T..........................A.....................  Mmulatta
 401826: .T..........................A.....................  Panubis

                              LINE1
        ------------------------------------------------------>
                 89288     89298     89308     89318     89328
                     :         :         :         :         :
  89279: CCAAGAAGTCATTGTTAAGTCCAATGTTATGAAGCCTCTTGCCTATCTTT  Hsapiens
 408956: T.................................................  Ptroglodytes
 426452: T.................................................  Ggorilla
 462337: T.................................................  Pabelli
 453121: T.......C...........A....A.....................-..  Mmulatta
 401876: T........................A.....................-..  Panubis

                              LINE1
        ------------------------------------------------------>
                 89338     89348     89358     89368     89378
                     :         :         :         :         :
  89329: TTTTTCTAAAGTTATCATTTTGACTCTAACACTTAGGTCTTTAATACTTT  Hsapiens
 409006: .............................................C....  Ptroglodytes
 426502: .............................................C....  Ggorilla
 462387: .............................................C....  Pabelli
 453170: .A.....................T.......A.............C....  Mmulatta
 401925: .A.....................T.......A.............C....  Panubis

                              LINE1
        ------------------------------------------------------>
                 89388     89398     89408     89418     89428
                     :         :         :         :         :
  89379: TGAGTTAGCCTTTATATATGCTTTAAGGTAAGGGTCGAACTTCATTCACT  Hsapiens
 409056: .........T........................................  Ptroglodytes
 426552: .........T........................................  Ggorilla
 462437: .........T........................................  Pabelli
 453220: .......A.T.C...........C............C..A..........  Mmulatta
 401975: .......A.T.C...........C............C..A..........  Panubis
```

```
                                   LINE1
         ------------------------------------------------------>
                   89438     89448     89458     89468     89478
                       :         :         :         :         :
 89429: GGCATGTAGATATCCAGGTTTTCCAGCATCATTTGTTAAAAAGACTGTAA  Hsapiens
409106: ..................................................  Ptroglodytes
426602: ..................................................  Ggorilla
462487: .........................T..C.....................  Pabelli
453270: ...........T..T..A..........C........G............  Mmulatta
402025: ...........T..T..A..........C........G............  Panubis

                                   LINE1
         ------------------------------------------------------>
                   89488     89498     89508     89518     89528
                       :         :         :         :         :
 89479: TTTTCCCCATTGAATGGTCTTGAAACACTTGTCAAAATCTTTTGACCACT  Hsapiens
409156: ..................................................  Ptroglodytes
426652: ...........................T............C.........  Ggorilla
462537: ...............T................................T.  Pabelli
453320: ...C.....C...................A..................T.  Mmulatta
402075: ...C.....C...................A..................T.  Panubis

                                   LINE1
         ------------------------------------------------------>
                   89538     89548     89558     89568     89578
                       :         :         :         :         :
 89529: TACAGAAGGGTTTATTTATGGGGTCTCTATTCTATTCCGTTGGTCTATAT  Hsapiens
409206: ..................................................  Ptroglodytes
426702: ..................................................  Ggorilla
462587: ..............................................C...  Pabelli
453370: ......................................A...........  Mmulatta
402125: ......................................A...........  Panubis

                                   LINE1
         ------------------------------------------------------>
                   89588     89598     89608     89618     89628
                       :         :         :         :         :
 89579: ATCTGTCTTTAGCCAATACCACATCATTTGGATTACTGTAACTTTCTAAT  Hsapiens
409256: ..........T..................................G....  Ptroglodytes
426752: ..........T..................................G....  Ggorilla
462637: ..........T..................................G....  Pabelli
453420: ..........T..................................G....  Mmulatta
402175: ..........T..................................G....  Panubis

                                   LINE1
         ------------------------------------------------------>
                   89638     89648     89658     89668     89678
                       :         :         :         :         :
 89629: AGGTTTTGAAAACAGGAAATGTGAAACCTCCAAATTTTGTTCTTTTTTCA  Hsapiens
409306: ..................................................  Ptroglodytes
426802: ..................................................  Ggorilla
462687: ..................................................  Pabelli
453470: .......................................A..-.......  Mmulatta
402225: .............C..................                    Panubis
402598:                                 ..........-.......  Panubis
```

```
                                     LINE1
        ---------------------------------------------------->
                     89688     89698     89708     89718     89728
                         :         :         :         :         :
  89679: AGACTGTTTTGGCAATTCAAGGTGTGTTGAAATTCCATATTAATTTTAGG  Hsapiens
 409356: ...T..............................................  Ptroglodytes
 426852: ...T................................-.............  Ggorilla
 462737: .............T....................................  Pabelli
 453519: ....G........T...T................................  Mmulatta
 402615: ....G........T...T................................  Panubis

                    LINE1                      LINE1
        --------------------------->  ------------------------>
                     89738     89748     89758     89768     89778
                         :         :         :         :         :
  89729: ATGAGTTTTTCTATCTTTGCAAAAAAAAATCTGTAGGTAGCTTTGGGTAA  Hsapiens
 409406: ....................-.........A...................  Ptroglodytes
 426901: ....................--.......AA........C..........  Ggorilla
 462787: ....................-.............................  Pabelli
 453569: ..................A.--......G...................G.  Mmulatta
 402665: ..................A.--......G...................G.  Panubis

                                LINE1                     LINE1
        ------------------------------------------------->---->
                     89788     89798     89806     89816     89826
                         :         :         :         :         :
  89779: TATTGTCATCTTAACAATATT--GTCTTTTAGTTCACAAACATAGCTTAA  Hsapiens
 409455: .....................--...........................  Ptroglodytes
 426949: .....................--...........................  Ggorilla
 462836: .....................AA.................G........  Pabelli
 453617: .....................AA........A.............T....  Mmulatta
 402713: .....................AA........A.............T....  Panubis

                                     LINE1
        ---------------------------------------------------->
                     89836     89846     89856     89866     89876
                         :         :         :         :         :
  89827: TTCTGCTAAGCATTTAAAGAAGAAGTAATACCAATTCTTCTCAAACTCTT  Hsapiens
 409503: ..................................................  Ptroglodytes
 426997: ........................A.........................  Ggorilla
 462886: .................................G................  Pabelli
 453667: .........A........................................  Mmulatta
 402763: .........A........................................  Panubis

                                     LINE1
        ---------------------------------------------------->
                     89886     89896     89906     89916     89926
                         :         :         :         :         :
  89877: CCAAATAAACAAAGAGTGAGGAATCCTTCCAAACTTATTTTAGGAAGCGA  Hsapiens
 409553: ..............-...................................  Ptroglodytes
 427047: ..................................................  Ggorilla
 462936: ...............................................T..  Pabelli
 453717: ................................................A.  Mmulatta
 402813: ................................................A.  Panubis
```

```
                           LINE1
             89936     89946     89956     89966     89976
 89927: GACTTACCATGATACCAAAACCACACAAGGACACTACAAGAAAAGAAAAT  Hsapiens
409602: ..................................................  Ptroglodytes
427097: ..............................G...................  Ggorilla
462986: ..A...............................................  Pabelli
453767: ..A.................T...............T.............  Mmulatta
402863: ..A.................T...............T.............  Panubis
```

```
                           LINE1
             89986     89996     90006     90016     90026
 89977: TACAGGCCAATATCCCTGATGAACATAGATGCAAAAATCTTCAAACAAGT  Hsapiens
409652: ..................................................  Ptroglodytes
427147: ..................................................  Ggorilla
463036: ....A........................................A....  Pabelli
453817: ....T.........G.........G....................A....  Mmulatta
402913: ....T.........G........TG....................A....  Panubis
```

```
                           LINE1
             90036     90046     90056     90066     90073
 90027: ACTAGCAAACCGAATTCAACAGCATGTCAAAAGAATCATTCAA---CATG  Hsapiens
409702: ...........A...............................---....  Ptroglodytes
427197: ...........A...............................---....  Ggorilla
463086: G..........A.............A.................---....  Pabelli
453867: ...........A.............A.......G.........CAT....  Mmulatta
402963: ...........A.............A.......G.........CAT....  Panubis
```

```
                           LINE1
             90083     90093     90103     90113     90123
 90074: AAGTGGAATTTATCCCAGGGATGCAAGGATGGTTCAATATATGCAAATCA  Hsapiens
409749: ..................................................  Ptroglodytes
427244: ..................................................  Ggorilla
463133: ...........G.....A..........................C.....  Pabelli
453917: ......................A...........................  Mmulatta
403013: ..................................................  Panubis
```

```
                           LINE1
             90133     90143     90153     90163     90173
 90124: ATAAATTTGATACACCACATTAACAGAATGAAGGACAAAAACCATATAAT  Hsapiens
409799: ..................................................  Ptroglodytes
427294: ..................................................  Ggorilla
463183: ..................................................  Pabelli
453967: ............T............................T...G...C  Mmulatta
403063: ............T............................T...G...C  Panubis
```

```
                                  LINE1
         ------------------------------------------------->
               90183     90193     90203     90213     90223
                   :         :         :         :         :
  90174: CATCTCAATAGATACAGAAACAGCATCTGAAAACATTTAACATTTTTTCA  Hsapiens
 409849: ....C.........T...................................  Ptroglodytes
 427344: ..............T...................................  Ggorilla
 463233: ..............T...................................  Pabelli
 454017: .............G............T.............A.........  Mmulatta
 403113: .............G............T.............A.........  Panubis

                                  LINE1
         ------------------------------------------------->
               90233     90243     90253     90263     90273
                   :         :         :         :         :
  90224: TGATAAAAATTTGCAAGAAATTAGGCACAAAAAATACATACCTCAACAAA  Hsapiens
 409899: ..................................................  Ptroglodytes
 427394: ..................................................  Ggorilla
 463283: ..................................................  Pabelli
 454067: ..................T.................T.............  Mmulatta
 403163: ..................T.................T.............  Panubis

                                  LINE1
         ------------------------------------------------->
               90283     90293     90303     90313     90323
                   :         :         :         :         :
  90274: ATAAAGCCCCATGCACAGCTAAAATTATATTCAATGGTGAGAATTTGAAA  Hsapiens
 409949: ....................................A.............  Ptroglodytes
 427444: ....................................A.............  Ggorilla
 463333: ...........................C........A.............  Pabelli
 454117: .........AT.........................A.....T.......  Mmulatta
 403213: .........AT.........................A.....T.......  Panubis

                                  LINE1
         ------------------------------------------------->
               90333     90343     90353     90363     90373
                   :         :         :         :         :
  90324: CTTTTTCCTCTAAGATCAGGAACAAGACAAGGATGCTTACTCTCATCACT  Hsapiens
 409999: ..................................................  Ptroglodytes
 427494: ..................................................  Ggorilla
 463383: ...........................................T......  Pabelli
 454167: .............A....................................  Mmulatta
 403263: .............A....................................  Panubis

                                  LINE1
         ------------------------------------------------->
               90383     90393     90403     90413     90423
                   :         :         :         :         :
  90374: TCTGTTCAACATAGCACTGGAAGATCTAGCCAGAGCAATTAGACAATAGA  Hsapiens
 410049: .......................T......................G...  Ptroglodytes
 427544: .................A.....T......................G...  Ggorilla
 463433: .......................T..............C.......G...  Pabelli
 454217: .......................T..................G...A...  Mmulatta
 403313: .......................T..................G...A...  Panubis
```

```
                         LINE1
        ------------------------------------------------->
              90433     90443     90453     90463     90473
                  :         :         :         :         :
 90424: AAGAAATAAAAGCTATTCAAATCAGAAAGGAAGAATTAAACAATCTCTGT  Hsapiens
410099: ..................................................  Ptroglodytes
427594: ..................................................  Ggorilla
463483: ............A.....................G...............  Pabelli
454267: ..A.G.......A...................C.G...........-G..  Mmulatta
403363: ..A.G.......A...................C.G...........-G..  Panubis

                         LINE1
        ------------------------------------------------->
              90483     90493     90503     90513     90523
                  :         :         :         :         :
 90474: TTGGAGATGACATGATCTTATGTATAGAAAACCCTAAAGCAGCTACCAAA  Hsapiens
410149: ...........................................A......  Ptroglodytes
427644: .......A..........................................  Ggorilla
463533: .....................T............................  Pabelli
454316: .--...............................................  Mmulatta
403412: .--...............................................  Panubis

                         LINE1
        ------------------------------------------------->
              90533     90543     90553     90563     90573
                  :         :         :         :         :
 90524: AATATGTTGAAACTAATAAATGAATTCTACAAAGCTGCAGTTTACAAAAC  Hsapiens
410199: ..................................................  Ptroglodytes
427694: ..................................................  Ggorilla
463583: ........................................GA........  Pabelli
454364: ........AG.............G..........T.....GA........  Mmulatta
403460: ........AG.............G..........T.....GA........  Panubis

                         LINE1
        ------------------------------------------------->
              90583     90593     90603     90613     90623
                  :         :         :         :         :
 90574: CAACATACAAAAATTATTACTGTTTTTATGTACTAACAATGAACTATACA  Hsapiens
410249: ................................................T.  Ptroglodytes
427744: ................................................T.  Ggorilla
463633: ................C...A......................A..C.T.  Pabelli
454414: ......G.........C..G...........G........T.......T.  Mmulatta
403510: ......G.........C..G....................T.......T.  Panubis

                         LINE1
        ------------------------------------------------->
              90632     90642     90652     90662     90672
                  :         :         :         :         :
 90624: AAAAAG-AAATCAAGAAAACAATCCCATTTGCAATGGCCACTAAAAAGTG  Hsapiens
410299: ......-...........................G...............  Ptroglodytes
427794: ......-...........................G...............  Ggorilla
463683: ......-...............................T...........  Pabelli
454464: ......-..............C................T...........  Mmulatta
403560: ......A..............C................T...........  Panubis
```

```
                             LINE1
        ------------------------------------------------->
             90682     90692     90702     90712     90722
 90673: AAATACCTGGAAATAAATTTAACAAATGAAGTGAAATATCTGTACACTGA  Hsapiens
410348: ..................................................  Ptroglodytes
427843: ..................................................  Ggorilla
463732: .......................C..........................  Pabelli
454513: ......T................C............G....A........  Mmulatta
403610: ......T................C............G....A........  Panubis
```

```
                             LINE1
        ------------------------------------------------->
             90732     90742     90752     90762     90772
 90723: AAACCAAAACATTGATGAAAGAAACTGAAGAAAACACAGATAAATAAAAG  Hsapiens
410398: ..................................................  Ptroglodytes
427893: ..................................................  Ggorilla
463782: .................................................T  Pabelli
454563: ..................................................  Mmulatta
403660: ..................................................  Panubis
```

```
                             LINE1
        ------------------------------------------------->
             90782     90792     90802     90812     90822
 90773: ATATCTCATGTTCATGGTTTGGAGGAATTAATATTGTTAACATGCCCACA  Hsapiens
410448: ..............C........................C..........  Ptroglodytes
427943: .......................................C..........  Ggorilla
463832: ..................................................  Pabelli
454613: ..C...TG....................................T.....  Mmulatta
403710: ......TG....................................T.....  Panubis
```

```
                             LINE1
        ------------------------------------------------->
             90832     90842     90852     90862     90872
 90823: CCACCAAAAGGGATCCACAGATTCATTTCAATCCAATTCCTATCAAAATT  Hsapiens
410498: ..................................................  Ptroglodytes
427993: ..................................................  Ggorilla
463882: ..................................................  Pabelli
454663: .......................................T...-------  Mmulatta
403760: .......................................T...-------  Panubis
```

```
                             LINE1
        ------------------------------------------------->
             90882     90892     90901     90911     90921
 90873: CCAATGACAATTTTTACAGAAATATAAG-TAAAAATTCTAAAATTTGCAT  Hsapiens
410548: ............................-A....................  Ptroglodytes
428043: .......................G....-A....................  Ggorilla
463932: ............................AA......C.............  Pabelli
454706: -------------------.........-A......C.............  Mmulatta
403803: -------------------.........-A......C.............  Panubis
```

```
                                 LINE1
         ------------------------------------------------------>
                  90931     90941     90951     90961     90971
                      :         :         :         :         :
  90922: GGAATGAGAAAAGACCCCCAATGGCCAAAGCAACCTTGAAAAATAACAAT   Hsapiens
 410597: ..................................................   Ptroglodytes
 428092: ..................................................   Ggorilla
 463982: ..................................................   Pabelli
 454736: .......A....C.......G........CT...........C....G..   Mmulatta
 403833: .......A............G........CT................G..   Panubis

                                 LINE1
         ------------------------------------------------------>
                  90981     90991     91001     91011     91021
                      :         :         :         :         :
  90972: GTGGGAGACATCACCCTACCTGACTTTAAAATATCCTACAAAGTTGTAGG   Hsapiens
 410647: ..................................................   Ptroglodytes
 428142: .C................................................   Ggorilla
 464032: .C.....G......A.....C.............................   Pabelli
 454786: .C.A...G..................C.......T........C......   Mmulatta
 403883: .C.....G..................C.......T........C......   Panubis

                                 LINE1
         ------------------------------------------------------>
                  91031     91041     91051     91061     91071
                      :         :         :         :         :
  91022: AATCAAATAGCATGGTACTGGCTAAAAGCAGACATATAGACCAATGAAAC   Hsapiens
 410697: ...........................A......................   Ptroglodytes
 428192: ...........................A...G..................   Ggorilla
 464082: ...........................A......................   Pabelli
 454836: ......GC...................A................C.....   Mmulatta
 403933: ......GC...................A......................   Panubis

                                 LINE1
         ------------------------------------------------------>
                  91081     91091     91101     91111     91121
                      :         :         :         :         :
  91072: AGAATAGAAGACCCAGAAATAAATCCATGCTTATACAGTGAATTGGTTTT   Hsapiens
 410747: .......................................C..........   Ptroglodytes
 428242: .......................................C..........   Ggorilla
 464132: ..................................................   Pabelli
 454886: ............T.................A..............A....   Mmulatta
 403983: ............T.................A....T.........A....   Panubis

                                 LINE1
         ------------------------------------------------------>
                  91130     91140               91149     91159
                      :         :                   :         :
  91122: TAAC-AAAGATGCCAAGGAC-----------AAAGGAAAGTCTCTTCAAT   Hsapiens
 410797: ....-...............-----------...................   Ptroglodytes
 428292: ....A...............-----------...................   Ggorilla
 464182: .G..-....T..........-----------.C.................   Pabelli
 454936: C...-...............ACACAATCAGG......C.....T......   Mmulatta
 404033: C...-...............ACACAATCAGG......C.....T......   Panubis
```

```
                                        LINE1
         -------------------------------------------------------->
                   91169     91177     91187     91197     91207
                       :         :         :         :         :
 91160: AAAGGGTGTTGAAA--AAATTGGATATCCATATGGAGAAAAAGGAAATTA  Hsapiens
410835: ..............--..................................  Ptroglodytes
428331: ..............--..................................  Ggorilla
464220: ..............--..................................  Pabelli
454985: ..............AC..................................  Mmulatta
404082: ..............AC..................................  Panubis

                                        LINE1
         -------------------------------------------------------->
                   91217     91227     91237     91247     91257
                       :         :         :         :         :
 91208: GATCCTCATCTCACACCATATACAAAAATCGACTGAAAATGGATTGAAGA  Hsapiens
410883: ..............................A...................  Ptroglodytes
428379: ..............................A...................  Ggorilla
464268: ......................T.......A...........T.......  Pabelli
455035: ..........................TG..A........CA........G  Mmulatta
404132: ...........................G..A........CA........G  Panubis

                                        LINE1
         -------------------------------------------------------->
                   91267     91277     91287     91297     91307
                       :         :         :         :         :
 91258: CTTAAACGTAAGGACTGAAAATGTAGAACTACTAAAAGAAAACATAGAGG  Hsapiens
410933: ..................................................  Ptroglodytes
428429: ..................................................  Ggorilla
464318: ......T..........C................................  Pabelli
455085: ....T.T.....A.....................................  Mmulatta
404182: ....T.T....TA.....................................  Panubis

                                        LINE1
         -------------------------------------------------------->
                   91317     91327     91337     91347     91357
                       :         :         :         :         :
 91308: AAAAGCTTCATGACATTAGCCTAGGCAATGATTTTTTAGATATGATTCCA  Hsapiens
410983: ...................T..G...........................  Ptroglodytes
428479: ...................T..G...........................  Ggorilla
464368: ........T....A...G.T..G......T....................  Pabelli
455135: .................G.T..G.A................G........  Mmulatta
404232: .................G.T..G.A.........................  Panubis

                                        LINE1
         -------------------------------------------------------->
                   91367     91377     91387     91397     91407
                       :         :         :         :         :
 91358: AAAGCACAGGCCGCAAAAGTGAAAATAGACAAATGTGATTACATAAAACT  Hsapiens
411033: ..................................................  Ptroglodytes
428529: ..................................................  Ggorilla
464418: ....................A............C................  Pabelli
455185: ............A.......A............................-  Mmulatta
404282: ............A.......A............................-  Panubis
```

```
                                        LINE1
                 91417     91427     91437     91447     91457
  91408: GAAAAGCTTCTGCACAGCCAAGGAGTGAAGAATTCACAATCAACAGAGAG  Hsapiens
 411083: ..................................................  Ptroglodytes
 428579: ..................................................  Ggorilla
 464468: ..........................A.......G.T.............  Pabelli
 455234: -.G...............................................  Mmulatta
 404331: -.G...............................................  Panubis

                     LINE1                             Simple
                 91467     91477     91487     91497     91505
  91458: ATGAGATGACATTCCAAATGGGAAAAAACGATATGGAACAGC--AAATAT  Hsapiens
 411133: ............................A.............AA......  Ptroglodytes
 428629: ............................A.............-A...A.A  Ggorilla
 464518: ....C.....C.A...............T.............-A...---  Pabelli
 455283: ..........C.A...............T...C.........-A......  Mmulatta
 404380: ..........C.A...............T...C.........-A......  Panubis

                       Simple                    LINE1
                           91513     91523     91533     91543
  91506: ATATA------------TATATACATATATATTTAAAATGGTCAAAGGAC  Hsapiens
 411183: .....------------....C................C...........  Ptroglodytes
 428678: .A.A.AANNNNNNNNNN.................................  Ggorilla
 464564: .....------------.G..........C........A..........T  Pabelli
 455332: ...C.------------....C.T...C.............G.......G  Mmulatta
 404429: ...C.------------....C.T...C.............G.......G  Panubis

                                        LINE1
                 91553     91563     91573     91583     91593
  91544: CTGAATAGACATTTTTCAAAAGAAGATATACAAATGGCCAACAATTCCAT  Hsapiens
 411221: ..................................................  Ptroglodytes
 428728: ...........C.............................G........  Ggorilla
 464602: ...............................G..................  Pabelli
 455370: ...........-..............A................G..A...  Mmulatta
 404467: ...........-...............................G..A..C  Panubis

                                        LINE1
                 91603     91613     91623     91633     91643
  91594: AGAAAGCTGCTTAACATCACTAATCCTCAGGGAAATGCAAATCGGAACCA  Hsapiens
 411271: ..................................................  Ptroglodytes
 428778: ..................................................  Ggorilla
 464652: .................A................................  Pabelli
 455419: .....AT........T...T.......................A...T..  Mmulatta
 404516: .....AT........T..GT.......................A...T..  Panubis
```

```
                                LINE1
         ------------------------------------------------->
               91653     91663     91673     91683     91693
                   :         :         :         :         :
  91644: CAGTGAGATACCATCTTACACCCGATAGGATGGCTATTATCAAAGAGTCA  Hsapiens
 411321: ...............................A..................  Ptroglodytes
 428828: ..................................................  Ggorilla
 464702: ..A....................A..........................  Pabelli
 455469: ..A....................A..........................  Mmulatta
 404566: ..A....................A..........................  Panubis

                                LINE1
         ------------------------------------------------->
               91703     91713     91723     91733     91743
                   :         :         :         :         :
  91694: AAAGATAACAAGTGTTGGTGAGGATGTGGAGAAAAGGGAACCCTTGTACA  Hsapiens
 411371: ..................................................  Ptroglodytes
 428878: ..................................................  Ggorilla
 464752: ..................................................  Pabelli
 455519: ..G.............T...........................G.....  Mmulatta
 404616: ..G.............T...........................G.....  Panubis

                                LINE1
         ------------------------------------------------->
               91753     91763     91773     91783     91793
                   :         :         :         :         :
  91744: CTGTTGGTGGGAATAAAATTTGTACAGCCATTTAGAAAACCTTATGGAGA  Hsapiens
 411421: ..................................................  Ptroglodytes
 428928: ..................................................  Ggorilla
 464802: ..................................................  Pabelli
 455569: T............................G..........A.........  Mmulatta
 404666: T............................G..........A.........  Panubis

                                LINE1
         ------------------------------------------------->
               91803     91813     91823     91833     91843
                   :         :         :         :         :
  91794: TTCCTCAGAATTTTAAGAACAGATCTAACATATGATTCAGCAATCCCACT  Hsapiens
 411471: ..................................................  Ptroglodytes
 428978: ..................................................  Ggorilla
 464852: .............................................T....  Pabelli
 455619: ......C...........................................  Mmulatta
 404716: ......C.......................................T...  Panubis

                                LINE1
         ------------------------------------------------->
               91853     91863     91873     91883     91893
                   :         :         :         :         :
  91844: TCTTGGAATATACACAAAGGGTATGAAATATATATGTCAAAGAATTATTT  Hsapiens
 411521: ..................................................  Ptroglodytes
 429028: ..................................................  Ggorilla
 464902: ....................A.............................  Pabelli
 455669: ............TC...............C.GC.................  Mmulatta
 404766: ............TC...............C.GC.................  Panubis
```

```
                               LINE1
          ------------------------------------------------->
               91903     91913     91923     91933     91943
                   :         :         :         :         :
   91894: GTATCCCATGTTCATTACAGCATTATTCACAATAGCCAAGACATGAAATC Hsapiens
  411571: .........................C........................ Ptroglodytes
  429078: .....................G............................ Ggorilla
  464952: .....T............................................ Pabelli
  455719: .....T..........G..A..............A............... Mmulatta
  404816: .....T..........G................................. Panubis

                               LINE1
          ------------------------------------------------->
               91953     91963     91973     91983     91991
                   :         :         :         :         :
   91944: ACCCTGTGTCCACCAACGAATGAACAAATAAAGAAAATATG--ATATATA Hsapiens
  411621: .G.....A................T................----..... Ptroglodytes
  429128: .G......................T................----..... Ggorilla
  465002: .G......................T................AT....... Pabelli
  455769: .G...............A......T................----..... Mmulatta
  404866: .G...............A......T................----..... Panubis

                               LINE1
          ------------------------------------------------->
               92001     92011               92029     92039
                   :         :                   :         :
   91992: TATACACAATGAAATACTCTTCCACCAT--AAAAAAGACAGTTCTGTCAT Hsapiens
  411667: .........C..................--.................... Ptroglodytes
  429174: ............................--.................... Ggorilla
  465052: ..................A.........--.................... Pabelli
  455815: ..................A.........AA.................... Mmulatta
  404912: ..................A.........AA.................... Panubis

                               LINE1
          ------------------------------------------------->
               92049     92059     92069     92079     92089
                   :         :         :         :         :
   92040: TTGCAACAACATGGATGAACCTGGAGGATATTATGTTAAGTGAAGTAAGC Hsapiens
  411715: ...................A.............................. Ptroglodytes
  429222: ...................A..........-----............... Ggorilla
  465100: ................A................................. Pabelli
  455865: .C................................................ Mmulatta
  404962: .C................................................ Panubis

                               LINE1
          ------------------------------------------------->
               92099     92109     92119     92129     92139
                   :         :         :         :         :
   92090: TAGACACAGAAAGACAAATACTACATAACTTCAATTATATGTGGAATCTA Hsapiens
  411765: C................................................. Ptroglodytes
  429267: ...........................................C...... Ggorilla
  465150: .............................C.................... Pabelli
  455915: A..G.........................C.................... Mmulatta
  405012: A..G.........................C.................... Panubis
 1072668:                                        ........... Sboliviensis
```

```
                              LINE1
         -------------------------------------------------->
              92149     92159     92169     92179     92189
                  :         :         :         :         :
  92140: AAAACGTTGAACTCATAGAAACAGAGAGTAGAATGGTGGCTACATGAGGG  Hsapiens
 411815: .....A............................................  Ptroglodytes
 429317: .....A...C..................................C.....  Ggorilla
 465200: ..................................................  Pabelli
 455965: .....................................CAT...C......  Mmulatta
 405062: .....................................CAT...C......  Panubis
1072679: C...A..C............G....A.A.................AA...C  Sboliviensis

                              LINE1
         -------------------------------------------------->
              92199     92209     92219     92229     92239
                  :         :         :         :         :
  92190: TGGTGGGGTGGAGGGGTTGGGAAGATGTTGTTCAAGGATATGAAATTTCA  Hsapiens
 411865: ..................................................  Ptroglodytes
 429367: ..................................................  Ggorilla
 465250: ............AACA..............G.A.................  Pabelli
 456015: ..............................G.A.................  Mmulatta
 405112: .............................AG.A.................  Panubis
1072729: .....AA......................AG.A.............G...  Sboliviensis

                              LINE1
         -------------------------------------------------->
              92249     92259     92269     92279     92289
                  :         :         :         :         :
  92240: ATTAAACATGAGGAATAAGTTCAAGGCATCTATTTTACATCATCATGACT  Hsapiens
 411915: ........................A.........................  Ptroglodytes
 429417: ..................................................  Ggorilla
 465300: ............T.....................................  Pabelli
 456065: G.......A.........................................  Mmulatta
 405162: G.......A.........................................  Panubis
1072779: G....G..A.......................................A.  Sboliviensis

                              LINE1
         -------------------------------------------------->
              92299     92309     92319     92329     92339
                  :         :         :         :         :
  92290: ATGTTTAATACCATTATATTGTATACTTGAAAATTGCTGAGGGAATAGAT  Hsapiens
 411965: ..................................................  Ptroglodytes
 429467: ..................................................  Ggorilla
 465350: ..A........T......C...............................  Pabelli
 456115: ..A........TG...............................G.....  Mmulatta
 405212: ..A........TG...............................G.....  Panubis
1072829: ..A.....C....A.....................C.....A..G.....  Sboliviensis

                              LINE1
         -------------------------------------------------->
              92349     92359     92367     92377     92387
                  :         :         :         :         :
  92340: TTTAAGTCTTCTAGCTACAA--AAATGATAATTATATGAGGTAATGGATA  Hsapiens
 412015: ....................--............................  Ptroglodytes
 429517: ....................--.........A..................  Ggorilla
 465400: ..C........C........--............................  Pabelli
 456165: .......G............--..                            Mmulatta
 460942:                            ..G....................  Mmulatta
 405262: .......G............--............................  Panubis
1072879: G......G...C........AG...................A......C.  Sboliviensis
```

```
                                   LINE1
          --------------------------------------------------->
                92397     92407     92417     92427     92435
   92388: TGCAAATTAGCTTAATTTAGCCATTCCACAATGTGTGCAT--GTCAAAAC  Hsapiens
  412063: ........................................--........  Ptroglodytes
  429565: ........................................--........  Ggorilla
  465448: .C....C.....C.............                          Pabelli
  460965: ...T...............................G....AT........  Mmulatta
  405310: ...T...............................G....AT........  Panubis
 1072929: ...T.......C...G..............G.....A...ATA...G...  Sboliviensis
```

```
                                   LINE1
          --------------------------------------------------->
                92445     92455     92465     92475
   92436: TTCATGTTGTATACCATTAATATATACAATTTTTATTTACCAATTTTTT-  Hsapiens
  412111: .................................................-  Ptroglodytes
  429613: .................................................T  Ggorilla
  465633:                               ..........A........T  Pabelli
  461015: ..........................................T......-  Mmulatta
  405360: ..........................................T......-  Panubis
  433081:                      ..C...............T..T...G..-  Cjacchus
 1072979: .......................CA..............T......G..-  Sboliviensis
```

```
            LINE1
          ------->
                92494     92504     92513     92523     92533
   92485: AAAAAAATTAACTGTTAAAAAAAGG-TTCTGAAAACAAATTAAGAACTTC  Hsapiens
  412160: T................G.......-........................  Ptroglodytes
  429663: .........................-........................  Ggorilla
  465653: T..............A........T-C.---G..................  Pabelli
  461064: ................T........-..T.........T...........  Mmulatta
  405409: ................T........-..T.........T...........  Panubis
  433109: T..T...................A.C...........GT..........G  Cjacchus
 1073028: T..T........--..T.C......-...........GTC.........T  Sboliviensis
```

```
                92541     92551     92561     92571     92581
   92534: ATATGCC--TACTACCTAAATTTGACATAAGTTAATATATTGTCATTCCT  Hsapiens
  412209: .......--.........................................  Ptroglodytes
  429712: .......--.........................................  Ggorilla
  465699: .......--................TG.......................  Pabelli
  461113: .......CC...C..............C..........G...........  Mmulatta
  405458: .......--..................C..........G...........  Panubis
  433159: .......--...C..............C......................  Cjacchus
 1073075: .......--..................C......................  Sboliviensis
```

```
                                                 LINE1
                                  ------------------------->
                92591     92601     92611     92621     92631
   92582: ATGTTAAAATATAATACATTACAGATATAATATTATAGGTGCTTTTAAAA  Hsapiens
  412257: ...............................G..................  Ptroglodytes
  429760: ...............................G..................  Ggorilla
  465747: ..........---..................G..........C.......  Pabelli
  461163: .........C.C................-..G.............A....  Mmulatta
  405506: .........C.C................-..G....C.............  Panubis
  433207: .CA......................C.....G.......G..........  Cjacchus
 1073123: G.C......................C.....G..................  Sboliviensis
```

```
                                 LINE1
        ------------------------------------------------------------------>
                                 92649     92659     92669     92679
  92632: TGTTTCATA--AGTGATATTTACACACTCTTTTCCATTTGATTTCCAGTT  Hsapiens
 412307: .........--...............T.......................  Ptroglodytes
 429810: .........--...............T.......................  Ggorilla
 465794: .........--...............T.........C.............  Pabelli
 461212: .........--...A...........T.......................  Mmulatta
 405555: .........--...............T.......................  Panubis
 433257: .....T...TA...............T.......----..T...T.....  Cjacchus
1073173: .....T...T-.AG............T.......----..T...T.....  Sboliviensis

                                 LINE1
        ------------------------------------------------------------------>
                       92689     92699     92709     92719     92729
  92680: CCTTGCTATTATGTCTAGAGATTTATTCTTTTTGACACATGATTATCCAC  Hsapiens
 412355: ..........................C.......................  Ptroglodytes
 429858: .............C....................................  Ggorilla
 465842: ..................................................  Pabelli
 461260: .............................C....................  Mmulatta
 405603: .............................C....................  Panubis
 433303: ........---------..T......CG......G..............G  Cjacchus
1073218: ........---------..T...................G.........G  Sboliviensis

                                 LINE1
        ------------------------------------------------------------------>
                       92739     92749     92759     92769     92779
  92730: TTCATTTATATTCACTGCTGTAGAATAGTCCACTGTATAAACCTAGCTTA  Hsapiens
 412405: .............T....................................  Ptroglodytes
 429908: ..................................................  Ggorilla
 465892: ..................................................  Pabelli
 461310: ...................A..............................  Mmulatta
 405653: ...................A..............................  Panubis
 433344: ...........................C...........G.T........  Cjacchus
1073259: ......--..G..............A.C........G..G..........  Sboliviensis

                                 LINE1
        ------------------------------------------------------------------>
                       92788     92798     92807     92817     92827
  92780: AT-TTTTCCTTATTCTTCTTTTGATATG-CATTTAAGCTGTTTCTAATTC  Hsapiens
 412455: ..-.........................-.....................  Ptroglodytes
 429958: ..-.........................-.....................  Ggorilla
 465942: ..-.........G..............A-.....................  Pabelli
 461360: ..-........................A-........T............  Mmulatta
 405703: ..-........................A-........T............  Panubis
 433394: ..C.....T..G...............AA........T.......C....  Cjacchus
1073307: ..T.....T..G...............AA.......AT............  Sboliviensis

                                 LINE1
        ------------------------------------------------------------------>
                       92837     92847     92857     92867     92877
  92828: TTCGCCATTGGCAAGAATGCTGCAATTCATGCCTTTGTATGTATCTTGTG  Hsapiens
 412503: ........................................T.G.......  Ptroglodytes
 430006: ..................................................  Ggorilla
 465990: ..................................................  Pabelli
 461408: ..........T.....T..T................A..C..G.......  Mmulatta
 405751: ..........T.....T..T................A..C..G.......  Panubis
 433444: ..............................T...........G.......  Cjacchus
1073357: ..T.........G.................T...........G.......  Sboliviensis
```

```
                                    LINE1
              ------------------------------------------------->
                    92887     92897     92907     92917     92927
                        :         :         :         :         :
   92878: CAACATAGTACATGGGTTCATCTAGGCTATACATAGAAAGAAAGAGACAG  Hsapiens
  412553: ..................................................  Ptroglodytes
  430056: .......A..........................................  Ggorilla
  466040: ..................................................  Pabelli
  461458: ...............................T..............--..  Mmulatta
  405801: ...............................T..............--..  Panubis
  433494: ..........................G..........G.....T.A.G..  Cjacchus
 1073407: ............G.............G..........G.....T.A.G..  Sboliviensis
```

```
       LINE1                         Alu
              >------------------------------------------------>
                                      92935     92945     92955
                                          :         :         :
   92928: GTTTTT----------------------TTCTTTGAGACAGAGTCTCACT  Hsapiens
  412603: A.....----------------------...............C......  Ptroglodytes
  430106: A.....----------------------...............C......  Ggorilla
  466090: ......----------------------......................  Pabelli
  461506: ......----------------------................----..  Mmulatta
  405849: ......----------------------................----..  Panubis
  433544: T.....GGTTTTTTGTTTTTGT------..T...------..........  Cjacchus
 1073457: T.....GGGTTTTTGTCTCTGTTTTTGT..T...................  Sboliviensis
```

```
                                     Alu
              ------------------------------------------------->
                    92965     92975     92985     92995     93004
                        :         :         :         :         :
   92956: CAGTCAGCCAGGCTGGAGAGCAGTGGCACAATCTCAGCCCACTG-CAACC  Hsapiens
  412631: ............................................-.....  Ptroglodytes
  430134: ............................................-.....  Ggorilla
  466118: ..............A...T.........................-.....  Pabelli
  461530: ..................G.........................-.....  Mmulatta
  405873: .............A....G.........................-.....  Panubis
  433582: .T....C........A..T..................TT.....-A....  Cjacchus
 1073507: .T....C........A..T...................T.....C.....  Sboliviensis
```

```
                                     Alu
              ------------------------------------------------->
                    93014     93024     93034     93044     93054
                        :         :         :         :         :
   93005: TCTACCTCCCAGGTTCAAGCGATCCTCCCACCTCAGCCTCCCAAGTAGCT  Hsapiens
  412680: .......T..........................................  Ptroglodytes
  430183: .......T...........T..............................  Ggorilla
  466167: ..........................................G.......  Pabelli
  461579: ...................G.....C...............TG.......  Mmulatta
  405922: ...................G.....C...............TG.......  Panubis
  433631: .---------G.........A.......T.....................  Cjacchus
 1073557: ..........G.A.......A.............................  Sboliviensis
```

```
                                     Alu
              ------------------------------------------------->
                    93064     93074     93084     93094     93104
                        :         :         :         :         :
   93055: AGGATTACAGGCATGCACCGCCATGCCTGGCTAATTTATTTTTATTTTTA  Hsapiens
  412730: ..................................................  Ptroglodytes
  430233: ............................C.....................  Ggorilla
  466217: ..................................................  Pabelli
  461629: ..................................................  Mmulatta
  405972: ..................................................  Panubis
  433672: .............CA...-A.................----.........  Cjacchus
 1073607: .......T.....CA...-A............G....----.....C---  Sboliviensis
```

```
                         Alu
          93113     93123     93133     93143     93153
  93105: TT-TTTTGTAATTTTAGTAGAGACAGGGTTTCACCATGTTGGCCAGGCTG  Hsapiens
 412780: ..-....................TG.........................  Ptroglodytes
 430283: ..-...............................................  Ggorilla
 466267: ..-....................TG.........................  Pabelli
 461679: ..-.............................G.................  Mmulatta
 406022: ..-...............................................  Panubis
 433717: ..C..................C.....-..............T.......  Cjacchus
1073649: ---..............C...C...A.-......................  Sboliviensis
```

```
                         Alu
          93163     93173     93183     93190     93200
  93154: GTCTCAAATTCCTGACCTCAAGTGATCTGCCCCT---CTCAGCCTCCCAA  Hsapiens
 412829: ..................................---.............  Ptroglodytes
 430332: ..................................---.............  Ggorilla
 466316: ........C......T..................---.............  Pabelli
 461728: ........C.........................----...A....A...  Mmulatta
 406071: ........C.........................----...A....A...  Panubis
 433766: ........C..T.....................CTCA.C......T....  Cjacchus
1073695: .....G..C..T............G........CTCA.A......T....  Sboliviensis
```

```
                   Alu                      LINE1
          93210     93220     93230     93240     93250
  93201: ATTGCTGGGATTACAGGTCAGAGCCACCACACCTGGCTTAGGGTATATAT  Hsapiens
 412876: .....................................C............  Ptroglodytes
 430379: .....................................C............  Ggorilla
 466363: ..................G..................C............  Pabelli
 461775: ....T.............G.C......TG...G....C............  Mmulatta
 406118: ....T.............G.C......TG...G....C............  Panubis
 433816: ......T......T..ACAT......TGG.G....C.C............  Cjacchus
1073745: .............T...CAT......TG..G....C.C............  Sboliviensis
```

```
                        LINE1
          93260     93270     93280     93290     93300
  93251: TTAAACATAGAATTACCAAAATAACCAGATATTGCCAAGTTTTTCCAAAA  Hsapiens
 412926: ..................................................  Ptroglodytes
 430429: ..................................................  Ggorilla
 466413: ...................................T..............  Pabelli
 461825: ................................G.................  Mmulatta
 406168: ..................................................  Panubis
 433866: ................T.................................  Cjacchus
1073795: ......G.........T.......T....A....................  Sboliviensis
```

```
                        LINE1
          93310     93319     93329     93339     93349
  93301: TAGTTGTACCAAT-AAACATTTTCAACAACATATTTATCAATTTTACTTG  Hsapiens
 412976: .............-....................................  Ptroglodytes
 430479: .............-....................................  Ggorilla
 466463: .............-....................................  Pabelli
 461875: ........A....A...........G....................G...  Mmulatta
 406218: .....A..A....-...........G....................G...  Panubis
 433916: .......G.....-..............G...T.............T.C.  Cjacchus
1073845: C......G.....-..............G...T.............T...  Sboliviensis
```

```
  LINE1                                          LINE1
 -------->                                  ------------->
                 93359     93369     93379     93389     93399
    93350: TCCACATCACTCCATCCCTAAATATCTAAGAATAGTTAGTATTATTTACA  Hsapiens
   413025: ..............C..........T........................  Ptroglodytes
   430528: .........................T........................  Ggorilla
   466512: ..........A..............T........................  Pabelli
   461925: .T........A...G.......A..T........................  Mmulatta
   406267: .T........A...G.......A..T........................  Panubis
   433965: ..........A...A...C..G...T........................  Cjacchus
  1073894: ..........A...A...C..G...T........................  Sboliviensis

                          LINE1
 -------------------------------------------------------->
                 93409     93419     93429     93439     93449
    93400: ATAAAAAAGACATGGAACCAACCCAAATGCCCATCAATGACAGACTGGAT  Hsapiens
   413075: ....C.............................................  Ptroglodytes
   430578: ....C.............................................  Ggorilla
   466562: ....C...........................G.................  Pabelli
   461975: ....C.......CA........A...........................  Mmulatta
   406317: ....C........A........A...........................  Panubis
   434015: ....C........A..............T.................A...  Cjacchus
  1073944: ....C.......--..............T.............A...A...  Sboliviensis

                          LINE1
 -------------------------------------------------------->
                 93459     93469     93479     93489     93499
    93450: AAAGAAAATGTGGTACATATACACCATGGAATACTATGCAGCCATAAAAA  Hsapiens
   413125: ..................................................  Ptroglodytes
   430628: ..................................................  Ggorilla
   466612: .......................A..........................  Pabelli
   462025: ..................G..T.....A.................G....  Mmulatta
   406367: .....................T.....A.................G....  Panubis
   434065: ....................G.............................  Cjacchus
  1073992: ....................GG.....A......................  Sboliviensis

                          LINE1
 -------------------------------------------------------->
                 93509     93519     93529     93539     93549
    93500: GGAATGAGATCATGTCCTTTGCAGGGACATGGATGAAGCTGGAAGCCATC  Hsapiens
   413175: ..................................................  Ptroglodytes
   430678: ..................................................  Ggorilla
   466662: ..................................................  Pabelli
   462075: ..................................................  Mmulatta
   406417: ........................................A.........  Panubis
   434115: ......C...........................................  Cjacchus
  1074042: ................T.................................  Sboliviensis

                          LINE1
 -------------------------------------------------------->
                 93559     93569     93578     93588     93598
    93550: AACCTCAGCAAACTAACACATTAACAG-AAAACCAAACATCACATGATCT  Hsapiens
   413225: ...........................-...........C..........  Ptroglodytes
   430728: ...........................-...........C..........  Ggorilla
   466712: ...........................-...........C......T...  Pabelli
   462125: .T.........................-...T.......CTG.T..T...  Mmulatta
   406467: .T.........................-...T.......CTG.T..T...  Panubis
   434165: .T.........................-.....TG....C.G..A.T...  Cjacchus
  1074092: .T.........................A.....TG..T.C....A.T...  Sboliviensis
```

```
                    LINE1
         ---------------------------------------------------->
                   93608     93618     93628     93638     93648
   93599: CACTCATAAGTGGGAATTGAACAATGAGAACATGGATACAGGGAGGGGAA  Hsapiens
  413274: ............................................A.....  Ptroglodytes
  430777: ................C...........................A.....  Ggorilla
  466761: ...............................................A..  Pabelli
  462174: TG.AT....T.....G.........C...............A........  Mmulatta
  406516: TG.AT....T.....G.........C...............A........  Panubis
  434214: ...............G.....T...A........................  Cjacchus
 1074142: ............T..G.....T............................  Sboliviensis

                    LINE1
         ---------------------------------------------------->
                   93658     93668     93677     93687     93697
   93649: CAACACACACAGGGACCAGTAGGGGGAT-GGGGGAAAAGGGAAGGGAGAG  Hsapiens
  413324: ............................-.....G...............  Ptroglodytes
  430827: .....T......................-.....G...............  Ggorilla
  466811: ............................-.....G.........A.....  Pabelli
  462224: ....G.....T...G.....T.......-.....GC....A.T.......  Mmulatta
  406566: ....G.....T...G.....T.......-.....GC..............  Panubis
  434264: ..T.......T...G.....CAA.....T..A..GC.....G..AA....  Cjacchus
 1074192: ..........T...G.....CAA.....T.A....C...A.G..AA....  Sboliviensis

                    LINE1
         ---------------------------------------------------->
                   93707     93717     93727     93737     93747
   93698: CATTGGGACAAATAGCTAATGCATGCAGGGCATAAAACCTAGGTGATGGG  Hsapiens
  413373: ..........................................A.......  Ptroglodytes
  430876: ...............T..........................A.......  Ggorilla
  466860: ....A.....................................A.......  Pabelli
  462273: ....A.....................................A.......  Mmulatta
  406615: ....A.....................................A.......  Panubis
  434314: ....A..............C...........G....G....AA...C...  Cjacchus
 1074242: ....A...............AT.....A...G....G.....A.....C.  Sboliviensis

                    LINE1
         ---------------------------------------------------->
                   93757     93767     93777               93786
   93748: TTGATCGGGGCAGCGAACCACCATGGCATATG-----------TATACCT  Hsapiens
  413423: ................................-----------.......  Ptroglodytes
  430926: .....T..........................-----------.......  Ggorilla
  466910: ........T.....A.................-----------....T..  Pabelli
  462323: ........T.....A.................CAGTAAATCACC.C...C  Mmulatta
  406665: ........T.....A.................CAGTAAATCACC.C...C  Panubis
  434364: ..CG.G...A....C.............C...-TATACACATGC.....C  Cjacchus
 1074292: ..C..G........A.............C.C.-TATGCACCTG......C  Sboliviensis

                    LINE1
         ---------------------------------------------------->
                   93796     93806     93816     93825     93835
   93787: GTGTAACAAACCTACACGTTCTGCACTTGTAT-CCCAGAACTTAAAGTAA  Hsapiens
  413462: ................................-.................  Ptroglodytes
  430965: .................A......T.......-.................  Ggorilla
  466949: ................................-...............-.  Pabelli
  462373: A................T.......T......C.................  Mmulatta
  406715: A................T.......T......C.................  Panubis
  434413: A....G.......G..T.......GT..-...-....A............  Cjacchus
 1074341: A............GTGT...............-.................  Sboliviensis
```

```
                 LINE1
              93845     93855     93865     93875     93885
                  :         :         :         :         :
  93836: AATTAAAAATAAAATTTAAAAAAGAATAGTTAGTATTACTGGTGTGAACC   Hsapiens
 413511: ..................................................   Ptroglodytes
 431014: ..................................................   Ggorilla
 466997: ..............................G...................   Pabelli
 462423: ...                                                  Mmulatta
 461945:           .........----...............T.             Mmulatta
 406765: ...                                                  Panubis
 406287:           .........----...............T.             Panubis
 434461: ...A........C....TT....T..............T..A......T.   Cjacchus
1074390: ...A.............T....GT...----.......T..A......T.   Sboliviensis

              93895     93905     93915     93925     93935
                  :         :         :         :         :
  93886: TCTCTGTTGTTTTAAATGCATTTTCCCCACCTAAAAAGAGTAAGAATCTT   Hsapiens
 413561: ...........................G......................   Ptroglodytes
 431064: ..........................AG......................   Ggorilla
 467047: ...........................G......................   Pabelli
 434511: C.........................TG..............G.......   Cjacchus
1074436: .........C.......A........TG......................   Sboliviensis

              93945     93955     93965     93975     93985
                  :         :         :         :         :
  93936: TCCACTGTAAACTTCTACATTGCTTCAGGATTTACTTGTCAAGGTCTATG   Hsapiens
 413611: ...............C..................................   Ptroglodytes
 431114: ...............C.............................T....   Ggorilla
 467097: ...............C..................................   Pabelli
 434561: ...............C.T....................C..C........   Cjacchus
1074486: ......T........C.T................................   Sboliviensis

              93994     94004     94014     94024     94034
                  :         :         :         :         :
  93986: -AAAAATGCCTTGGGGATTTTGAATAGGAGTGAATTTCTCAAGACATGAA   Hsapiens
 413661: -......................G...A......................   Ptroglodytes
 431164: -..........................A......................   Ggorilla
 467147: A..........................A......................   Pabelli
 434611: -......T.....T.......T..CG.A.................T....   Cjacchus
1074536: -.........A..............G.A.................G....   Sboliviensis

              94043     94053     94063     94073     94083
                  :         :         :         :         :
  94035: A-GGAAGCAGCTGAGCCCATAATCTTTGTAATCACTTTTGTCACTATAGC   Hsapiens
 413710: .-...........................................G....   Ptroglodytes
 431213: .-................................................   Ggorilla
 467197: .-................................................   Pabelli
 434660: .A......C.............C....A......................   Cjacchus
1074585: .A......CA............C....A......................   Sboliviensis

              94093     94103     94113     94123     94133
                  :         :         :         :         :
  94084: TGCTAAACACCACAATCTCAACACCTTGACTTAGACTCGCGCTTCATGTT   Hsapiens
 413759: ..................................................   Ptroglodytes
 431262: ..................................................   Ggorilla
 467246: ..................T..................T..C.........   Pabelli
 434710: AA.G..............T.............CT...T..C.........   Cjacchus
1074635: AA................T.............CTG..T..C....G....   Sboliviensis
```

```
              94143     94153     94163     94173     94183
  94134: TTGTCTCCGTCAGTGATATTTTGATGCTACCATATGCCATATAGCAAAGT  Hsapiens
 413809: ......................................G.......G...  Ptroglodytes
 431312: ..............C.......................G.......G...  Ggorilla
 467296: ........A................................G....C...  Pabelli
 434760: ..............A..............T...G....G.T..C..GT..  Cjacchus
1074685: .........................C...T.G...........C..GT..  Sboliviensis

              94192     94202     94212     94222     94231
  94184: CCC-TTTCCTTCCACCGTGTGTTTCTCAGCATGTGAACAAC-TCAGTCTC  Hsapiens
 413859: ...-.....................................-........  Ptroglodytes
 431362: ...-.....................................-........  Ggorilla
 467346: ...-..........T.A..............C.........-........  Pabelli
 434810: ...A...T......G...C......................T........  Cjacchus
1074735: ..TG..........GT..C......................-..G.....  Sboliviensis

              94241     94251     94261     94271     94281
  94232: AGAATTCCATTTGGAGGTCTGCTTTCTAGAGCTATTCCTGATACCAAGTA  Hsapiens
 413907: ..................................................  Ptroglodytes
 431410: ..................................................  Ggorilla
 467394: .....................T............................  Pabelli
 434860: .........C...........T.....GC.....G...............  Cjacchus
1074784: .........CGG.........T.....GC.....G...............  Sboliviensis

                                  LTR
              ---------------------------------------------->
              94291     94301     94311     94321     94330
  94282: CTTGCTATTAATCAAGGTCGCTACAGAAAACAAAGGGCACC-ATCAAATC  Hsapiens
 413957: ........A....G...........................-........  Ptroglodytes
 431460: ........A................................-........  Ggorilla
 467444: T.............G...T......................-........  Pabelli
 434910: ...A.......CAGG....................A.....A.......G  Cjacchus
1074834: ...A.......CAGG..G.A..G............A.....-.......G  Sboliviensis

                              LTR
         ------------------------------------------------->
              94340     94350     94360     94370     94380
  94331: TGTAATCAAATAGAGGGACTATTTACAACAGTATGATTCGGACTAAGAGG  Hsapiens
 414006: .........G...................G....................  Ptroglodytes
 431509: .........G........................................  Ggorilla
 467493: .........G........................................  Pabelli
 434960: .........GA.....-.................A...A.....C..G.A  Cjacchus
1074883: ......T..GA.....-......G..........G...A........G.A  Sboliviensis

                              LTR
         ------------------------------------------------->
              94390     94400     94410     94420     94430
  94381: AAGCAACAAAGAATGGTGAAACATCCAAGATCTAGCAATAGCGAAGAGAC  Hsapiens
 414056: ...........G......................................  Ptroglodytes
 431559: ...........G......................................  Ggorilla
 467543: ...........G..............................A.......  Pabelli
 435009: .....G.....G.CA........C.......G......C..T........  Cjacchus
1074932: .......C...G.CA.........TT............C..T.....CC.  Sboliviensis
```

```
                                     LTR
         ------------------------------------------------------------>
                     94440     94450     94460     94470     94480
                         :         :         :         :         :
  94431: TATTCTAACCCTAGTCCAGAAGGGCAAATGGTGAAGTCATTACCAAACCC  Hsapiens
 414106: ......................A...........................  Ptroglodytes
 431609: ......................A...........................  Ggorilla
 467593: ..........G.....T.................................  Pabelli
 435059: .......................A..........T.....C.........  Cjacchus
1074982: .......................A....G....TT.....C.........  Sboliviensis

                                     LTR
         ------------------------------------------------------------>
                     94490     94500     94510     94520     94530
                         :         :         :         :         :
  94481: CAGAGAAAGTAGATGTAGCTTTGGAAAACATTGCCCAATAAGCTCTGTAG  Hsapiens
 414156: ......C....T.....................T................  Ptroglodytes
 431659: ...........T.....................T................  Ggorilla
 467643: .............................G....................  Pabelli
 435109: ...........A......T...A.G...GG...................A  Cjacchus
1075032: ...........A......T...A.....GG...................A  Sboliviensis

                                     LTR
         ------------------------------------------------------------>
                     94540     94550     94560     94570     94577
                         :         :         :         :         :
  94531: CCTTTGGTAAAGTAACGCAAACATCTCTTTTGACCCTCAAAGTGAG---G  Hsapiens
 414206: ..............................................---.  Ptroglodytes
 431709: ..............................................---.  Ggorilla
 467693: ...............T..............................---.  Pabelli
 435159: ...........A...T.........C...G.........C..AA..---.  Cjacchus
1075082: ...........A...T.........C...G.........C..A...GAC.  Sboliviensis

                                     LTR
         ------------------------------------------------------------>
                     94587     94597     94607     94617     94627
                         :         :         :         :         :
  94578: GATCCAGTTGGCTAAGCACTCTGTCTTCACCTAGTTGCTGACCTACTCTT  Hsapiens
 414253: ..G...T...........................A...............  Ptroglodytes
 431756: ..G...T...........................A...............  Ggorilla
 467740: ..G...............................A....A..........  Pabelli
 435206: A.GA.C.....T.C......--..T......G-AA..........G....  Cjacchus
1075132: A.GA.......T........--..........-.A...............  Sboliviensis

                                     LTR
         ------------------------------------------------------------>
                     94637     94647     94657     94667     94677
                         :         :         :         :         :
  94628: GGTGCCTGCCATTGACCAAATCTAACTGGATGCCAGAGGGCACAGGAGCC  Hsapiens
 414303: ..................................................  Ptroglodytes
 431806: ..................................................  Ggorilla
 467790: ................................................T.  Pabelli
 435253: ..............G.................T.....A...........  Cjacchus
1075179: ........T..........G............T.....A...........  Sboliviensis
```

```
                           LTR
         ------------------------------------------------->
                 94687     94697     94707     94717     94727
                     :         :         :         :         :
   94678: TTATTATGTCTCTGTACAACTCAGCTTCTTGGAACCCATACGAGGTAGAG   Hsapiens
  414353: .....................................G...A........   Ptroglodytes
  431856: .....................................G...A........   Ggorilla
  467840: ..................................................   Pabelli
  435303: ..............................A.........G.........   Cjacchus
 1075229: ..............................A.........G.........   Sboliviensis

                          LTR
         ---------------------------------------------->
                 94737     94747     94757     94767     94777
                     :         :         :         :         :
   94728: AAGAATGAAAGGTGTAACTGAAGAAGCAAAATAAGATACCCAGCACAGCC   Hsapiens
  414403: ..................................................   Ptroglodytes
  431906: ..................................................   Ggorilla
  467890: ..................................................   Pabelli
  435353: .G..............G.................................   Cjacchus
 1075279: ................G.................................   Sboliviensis

                 94786     94796     94806     94816     94826
                     :         :         :         :         :
   94778: ACAT-TCCCATGAGGGCAAATTTCTTTATGTCTCTAGAACTTCACATCTC   Hsapiens
  414453: ....-.............................................   Ptroglodytes
  431956: ....-.............................................   Ggorilla
  467940: ....CC............................................   Pabelli
  435403: ....-C....C..............................C........   Cjacchus
 1075329: ....-C....C...........................C..C........   Sboliviensis

                 94836     94846     94856     94865     94875
                     :         :         :         :         :
   94827: CATTGGTACTCCAGAGACAAGCATCAACAATAGTT-ACATTTTTTATGTA   Hsapiens
  414502: ...................................-..............   Ptroglodytes
  432005: ..C................................-..............   Ggorilla
  467990: ...........................G.......-..............   Pabelli
  435452: .....A.......A..................A..G.G...G...T.C..   Cjacchus
 1075378: .....A..........G...............A..G.T...G...T.A..   Sboliviensis

                 94884     94894     94901     94911     94921
                     :         :         :         :         :
   94876: -AAAAACCATATTCAGGTACATGA---AACACAAGGATTCCAATGAACAA   Hsapiens
  414551: -.....G.................---.....G.................   Ptroglodytes
  432054: -.....G.................---....TG.................   Ggorilla
  468039: -.....G........A........---.....G.................   Pabelli
  435502: T.....T........A.....CA.GAA.....CG................   Cjacchus
 1075428: C.....T........A.....CA.GAA.....G..........A...TG.   Sboliviensis

                 94931     94941     94951     94961     94971
                     :         :         :         :         :
   94922: AAGGGTAAAAGAAAGACAATTCACGAAAGAGGAAATTAAACTAGAAAAGA   Hsapiens
  414597: ..................................................   Ptroglodytes
  432100: ..................................................   Ggorilla
  468085: ......G...A.......................................   Pabelli
  175596:           .......C.....T.......                       Panubis
  435552: ......----A.............--...........C.........---   Cjacchus
 1075478: ........................--...........C.........---   Sboliviensis
```

```
              94981     94991     95001     95011     95021
                  :         :         :         :         :
  94972: GATGTAAAATAACATTGGCTGTCTGTTGCCAATCAAATGAAAGCTTTGAA  Hsapiens
 414647: ..................................................  Ptroglodytes
 432150: ..................................................  Ggorilla
 468135: ...........................................A......  Pabelli
 435593: --.......C..A.........T..G........G......T........  Cjacchus
1075523: --..........A.......A.T...........................  Sboliviensis

              95031     95041     95051     95061     95071
                  :         :         :         :         :
  95022: GTCACTCAGAATAATTGAAGCAGTTTGAATAAAGAGCGGAGATGTGATAG  Hsapiens
 414697: ..................................................  Ptroglodytes
 432200: ..................................................  Ggorilla
 468185: A...................................T.....G.......  Pabelli
 435641: ...........A........................T.....GTCA.C..  Cjacchus
1075571: ...........A........................T.....G.CA.C..  Sboliviensis

              95081     95091     95101     95111     95121
                  :         :         :         :         :
  95072: ATTACTTAGTGAATATGAAGGAGCAGCATAGAAGATGGTAGGAGGTACAT  Hsapiens
 414747: ..................................................  Ptroglodytes
 432250: ..................................................  Ggorilla
 468235: ..................................................  Pabelli
 435691: T.............-.T...............................C.  Cjacchus
1075621: ...............CT.............................C..G  Sboliviensis

              95131     95141     95151     95161     95171
                  :         :         :         :         :
  95122: GGTATACCTGCATGGCCAACAAAATTAAAATAGGACATTGAAGACAGAGA  Hsapiens
 414797: ......A............A..............................  Ptroglodytes
 432300: ......A............A............A.................  Ggorilla
 468285: ......A............-..............................  Pabelli
 435740: .A.............T...A............A.....G...........  Cjacchus
1075671: .A............AT...A..................C...........  Sboliviensis

              95181     95190     95200     95210     95220
                  :         :         :         :         :
  95172: AAAACGATGGCTAGAAAA-GCAGTAGGAATAAAAGAGAAGATCATGCTTC  Hsapiens
 414847: ..................-...............................  Ptroglodytes
 432350: ..................-...............................  Ggorilla
 468334: ..................-...............................  Pabelli
 435790: .G.C.A............G.TG..G........G...-.....T......  Cjacchus
1075721: ...T.C............G.....G......T.G.........T.A....  Sboliviensis

              95230     95240     95250     95260     95270
                  :         :         :         :         :
  95221: CTGGCTTCATGGTATGAGAATGGAGGAGTGAGTAGATACCCATGAGAAAA  Hsapiens
 414896: ........................................T.........  Ptroglodytes
 432399: .....................A..................T.........  Ggorilla
 468383: ...A...................G..........................  Pabelli
 435839: ....G.....A....C.....A.G............CG............  Cjacchus
1075771: ....T...C.A....C....GACGA...........CT............  Sboliviensis

              95280     95290     95300     95310     95320
                  :         :         :         :         :
  95271: GATCAAGGGGCATGCAGCCATCAGGGAAAATCCATAGTGCAGATAAAGCA  Hsapiens
 414946: ..................................................  Ptroglodytes
 432449: ..................................................  Ggorilla
 468433: ........T...C.....................................  Pabelli
 435889: .........A.......T...TG.......C.T.C...............  Cjacchus
1075821: .........A............G...........C..G..T.........  Sboliviensis
```

```
              95330     95340     95350     95360     95370
  95321: AGGAGGAAGAAGGACAGTTCTAGTAAAAGGGAGAACATCAGTATAGGATG  Hsapiens
 414996: ........................................A.........  Ptroglodytes
 432499: ........................................A.........  Ggorilla
 468483: ........................................A.........  Pabelli
 435939: ...........A............................A....T....  Cjacchus
1075871: ...........A............-......G........A....T....  Sboliviensis

              95380     95389     95399     95409     95419
  95371: TTTCTTCGCAATAG-AAAAAGAAGGCCAAGAGGAATCAGGGAGAGAGTTA  Hsapiens
 415046: ..............-...................................  Ptroglodytes
 432549: ......A.......-...................................  Ggorilla
 468533: ......AA......-...................................  Pabelli
 435989: ......A.G.....-.........................A.....A...  Cjacchus
1075920: ......A.G.....A...........................A...A...  Sboliviensis

              95429     95439     95449     95459     95469
  95420: TAAGAGAAGACTAAGGGGACCAGGTTAGATTTGGTTTGGTTTGAAAGCAT  Hsapiens
 415095: ..............C.....TG.A.............A............  Ptroglodytes
 432598: ..............C.....TG.A..........................  Ggorilla
 468582: .......C..G...A.....AG...A........................  Pabelli
 436038: .......TC.GC........AGAA.................           Cjacchus
 436347:                                            ...AA..  Cjacchus
1075970: ......GTC.GC........AG...............A...C....A...  Sboliviensis

              95479     95489     95499     95509     95519
  95470: ACAGTAAATATGATGTCTGTCCCTGGTGGTGTTGGTTGAGTAGGAAGGAG  Hsapiens
 415145: ............................T.....................  Ptroglodytes
 432648: ....C.......................T.....................  Ggorilla
 468632: ..........................C.........G.............  Pabelli
 436354: ..............A..........T.......A..G.....A.......  Cjacchus
1076020: ....................................G.....A.......  Sboliviensis

              95529     95539     95549               95565
  95520: GAAGGGAGGCAAGAGATAATATCATTTTGTCTGTGCT----ACTGTAATT  Hsapiens
 415195: ...................C.................----......C..  Ptroglodytes
 432698: ...................C.................----......C..  Ggorilla
 468682: --------.............................----......C..  Pabelli
 436404: .G..A..A.G..........................GCTAAG.....TA.  Cjacchus
1076070: .G..A..A.G...........................CTAA.....GC..  Sboliviensis

                                                      LTR
                                                    ------>
              95575     95585     95595     95605     95615
  95566: ACATATATCAGGGGAAATTCAGCCAGATATCAGGTAAAATTCACCCCTGA  Hsapiens
 415241: ..................................................  Ptroglodytes
 432744: ..................................................  Ggorilla
 468720: ..................................................  Pabelli
 436454: ......                                              Cjacchus
1076120: ......                                              Sboliviensis
```

```
                          LTR
                 95625     95635     95645     95655     95665
   95616: TATTTCACGTAGGTTCTTTTCTATTTTCCCTAAGTGTCGGCTGGTCTGAG  Hsapiens
  415291: ..................................................  Ptroglodytes
  432794: ..................................................  Ggorilla
  468770: .......T.............................T..T.........  Pabelli

                          LTR
                 95675     95685     95695     95705     95715
   95666: AAATAAAGGGATAGAGTACAAAAGAGAGAAATTTTAAAGCTGGGTGTCTG  Hsapiens
  415341: .......A..........................................  Ptroglodytes
  432844: .......A..........................................  Ggorilla
  468820: ..................................................  Pabelli

                          LTR
                 95725     95735     95745     95755     95765
   95716: GGGAAGACATCACAGTTTGGCAGGTTCCATGATACCCCCTGAGCCACAAA  Hsapiens
  415391: ...G...........G.C................................  Ptroglodytes
  432894: ...G...........G.C................................  Ggorilla
  468870: ...G..........TG.................G................  Pabelli

                          LTR
                 95775     95785     95795
   95766: ACCAGCAAGTTTTTATTAGTGATTTCAAAAGGAG----------------  Hsapiens
  415441: ................................G.----------------  Ptroglodytes
  432944: ................................G.----------------  Ggorilla
  468920: ................................G.AGGGAGTGTAGGAATA  Pabelli

                          LTR
                                                     95805
   95800: --------------------------------------------AGGGAG  Hsapiens
  415475: --------------------------------------------......  Ptroglodytes
  432978: --------------------------------------------......  Ggorilla
  468970: GGGGAGGGAGTGTAGGAATAGGGGAGGGAGTGTAGGAAAAGGGG......  Pabelli

                          LTR
                 95815     95825     95835     95845     95855
   95806: TGTACGAATAGAGTGTGGGTCACAGAGATCACGTGCTTCACAAGGTGATA  Hsapiens
  415481: ...........G......................................  Ptroglodytes
  432984: ...........G......................................  Ggorilla
  469020: .....A.....G.....CA............................T..  Pabelli

                          LTR
                 95865     95875     95885     95895     95905
   95856: GAATATCACAAGGCAAATGGAGGCAGGGTGAGATCACAGGACCACAGGAC  Hsapiens
  415531: .................C..........C.......A.............  Ptroglodytes
  433034: ....................................A.............  Ggorilla
  469070: .......................G....C.....................  Pabelli
```

```
                                LTR
         ------------------------------------------------->
                  95915     95925     95935     95945     95955
 95906: CTGGGTGAAATTAAAATTGCTAATGAAGTTTCGGGCACGCATTGTCATTG  Hsapiens
415581: ..................................................  Ptroglodytes
433084: ...............................T..................  Ggorilla
469120: ..................................................  Pabelli
```

```
                                LTR
         ------------------------------------------------->
                  95965     95975     95985     95995     96005
 95956: ATAACATCTTATCAGGAGACAGGGTTTGAGAGCAGACAACCGGTCTGACC  Hsapiens
415631: .........................................A........  Ptroglodytes
433134: ....................T.....C..............A........  Ggorilla
469170: ................T.................................  Pabelli
```

```
                                LTR
         ------------------------------------------------->
                  96015     96025     96035     96045     96055
 96006: AAAAATTTATTAGATGGGACTTTCCTCATCCTAATAAGCCTAGGAGCGCT  Hsapiens
415681: ...................A..........................T...  Ptroglodytes
433184: ...................A...T..........................  Ggorilla
469220: .............G.....A.....................GA...T...  Pabelli
```

```
                                LTR
         ------------------------------------------------->
                  96065     96075     96085     96095     96105
 96056: ACGGGAGGTTGGGGCTTATTTCATCCCTACAGCTTCAACCATAAAAGACG  Hsapiens
415731: ..A.A...........C...............................T.  Ptroglodytes
433234: ....A...........................................T.  Ggorilla
469270: ..........A.......................................  Pabelli
```

```
                                LTR
         ------------------------------------------------->
                  96114     96124     96134     96144     96154
 96106: GCTG-CCCCCCAAAGCGGCCATTTTAAAGGCCTACCCTCAGGGGCATATT  Hsapiens
415781: ....-.....T.......................................  Ptroglodytes
433284: ....-.............................................  Ggorilla
469320: ....C...................................A...TG....  Pabelli
```

```
                                LTR
         ------------------------------------------------->
                  96164     96174     96184     96194     96204
 96155: CTCTTTCTCAGGGATGTTCCTTGATGAGAAAAAGAATTCAGCGATATTTC  Hsapiens
415830: .......................C..........................  Ptroglodytes
433333: .......................C..........................  Ggorilla
469370: .......................C.....G....................  Pabelli
```

```
                                LTR
         ------------------------------------------------->
                  96214     96224     96234     96244     96254
 96205: TCCCATTTGCTTTTGAAAGAAGAGAAATATGGCTCTGTTCCACCCAGCTC  Hsapiens
415880: ........................................TG........  Ptroglodytes
433383: ..................................................  Ggorilla
469420: .........................................G........  Pabelli
```

```
                         LTR
        ------------------------------------------------->
             96264     96274     96284     96294     96304
 96255: ACCGGCGGTCAGAGTTTAAGGTTATCACTCATGTTCCCTGAACATTGCTG  Hsapiens
415930: ..........................T.......................  Ptroglodytes
433433: ...A..A...................T.......................  Ggorilla
469470: ...T.T....................T.......................  Pabelli
```

```
                         LTR
        ------------------------------------------------->
             96314     96324     96334     96344     96354
 96305: TTATCCTGTTCTTTTTTCAAGGTGCCCAGATTTCATATTGTTCAAACACA  Hsapiens
415980: ..................................................  Ptroglodytes
433483: ..................................................  Ggorilla
469520: ..................................................  Pabelli
```

```
                         LTR
        ------------------------------------------------->
             96364     96374     96384     96394     96404
 96355: CATGCTCTACAAATAATTTATGCAGTTAATGCAATCATCACAGGGTCCTG  Hsapiens
416030: ........................T.........................  Ptroglodytes
433533: ........................T.........................  Ggorilla
469570: .............C....................C.T............T  Pabelli
```

```
                         LTR
        ------------------------------------------------->
             96414     96424     96434     96444     96454
 96405: GGACCACATACATTCTCCTCAGCTTGCAAAGATGATGGGATTAAGAGATT  Hsapiens
416080: ....G....................A........................  Ptroglodytes
433583: ....G....................A........................  Ggorilla
469620: ....G........C...........A.......A................  Pabelli
```

```
                         LTR
        ------------------------------------------------->
             96464     96474     96484     96494     96504
 96455: AAAGTAAAGACAGGCATAGGAAATCACAAGGGTATTGATTGAGGAAGTGA  Hsapiens
416130: ..................................................  Ptroglodytes
433633: ..................................................  Ggorilla
469670: ..................................................  Pabelli
```

```
                         LTR
        ------------------------------------------------->
             96514     96524     96534     96544     96554
 96505: TAAGTGTTCATGAAATCTTCACAATTTATGTTCAGAGATTGCAGTAAAGA  Hsapiens
416180: ..................................................  Ptroglodytes
433683: ......................C...........................  Ggorilla
469720: .......C..........................................  Pabelli
```

```
                         LTR
        ------------------------------------------------->
             96564     96574     96584     96594     96604
 96555: CAGGCATAAGAAATTATAAAAGTATTAATTTGGGGAACTAATAAATGTCC  Hsapiens
416230: ..........................G.......................  Ptroglodytes
433733: ..................................................  Ggorilla
469770: ..T...................C...............A...........  Pabelli
```

```
                          LTR
          ------------------------------------------------->
                 96614     96624     96634     96644     96654
   96605: ATGAAAACTTCATAATCTATGTTCTTCTGCCATGGCTTCAGCCAGTCCCT  Hsapiens
  416280: .....................................C............  Ptroglodytes
  433783: .....................................C............  Ggorilla
  469820: .................................A................  Pabelli

                 LTR
          ----------------------------->
                 96664     96674     96684     96694     96704
   96655: CGGTTCAGGGTCCCTGACTTCCTGCAACATACATGTGAGACTATTTCCCG  Hsapiens
  416330: .C................................A...............  Ptroglodytes
  433833: .C................................A...............  Ggorilla
  469870: .C......T.............C...........A..............A  Pabelli
  436450:                           .T......A.......G......-  Cjacchus
 1076119:                              .....A......G.......T  Sboliviensis

                 96714     96724                         96746
   96705: CTCTGCTTTTCAAACCTTACTGGAGTTG-----TTTTCC---CTCATGAA  Hsapiens
  416380: ............................-----......---........  Ptroglodytes
  433883: ............................-----......---....G...  Ggorilla
  469920: ......................C.....-----......---........  Pabelli
  170210:      ....CT..........CA.T...CAAGA..C..AGTG.....T..  Mmulatta
  436473: ----T..C......T.............-----......---........  Cjacchus
 1076140: ....T.......................-----......---........  Sboliviensis

                 96756     96766     96776     96786     96796
   96747: AACTAAGAAAGGAAAGCTAGTTAATCTTATTCTGAGGTTGTTCAATATAT  Hsapiens
  416422: ......C...........................................  Ptroglodytes
  433925: ..................................................  Ggorilla
  469962: ..................................................  Pabelli
  170255: ..-------..C...T.A.T.A...G.GCC.T....T..AC.T..AGAG.  Mmulatta
  436511: ......A.C.......T..A........G....................C  Cjacchus
 1076182: .....GA.C.......T..A........G..T.................C  Sboliviensis

                 96806     96813     96823     96833     96843
   96797: ACATATTCACAT---CTGTAGAGAGATCCTTGGGAATACAGTAATTGGCA  Hsapiens
  416472: ............---...................................  Ptroglodytes
  433975: ............---...................................  Ggorilla
  470012: ......CT....---...................................  Pabelli
  170298: .GCA...A.A..ATTT..A.A.A..TAAA...T..G..TG.AG.....A.  Mmulatta
  436561: .....CCT.T..---.......................AG..........  Cjacchus
 1076232: .....CCT.T..---.......................AG..........  Sboliviensis

                 96853     96862     96872     96882     96892
   96844: TATATTCTGTTATTTG-ATGCTTGAAAAATCTCTTCCACTAACCAGTTTC  Hsapiens
  416519: ................-...T.............................  Ptroglodytes
  434022: ................-.................................  Ggorilla
  470059: ..............G.-.................................  Pabelli
  170348: C.C.C---..G.CCC.T....CCTTCG.GC..G.GTAT....GAG..G.T  Mmulatta
  436608: ........A......A-................C................  Cjacchus
 1076279: ........A......A-................C................  Sboliviensis
```

```
              96902     96912     96922     96932     96941
   96893: CCTATAGATAGGCACAAGCACATAGGTAAGAAACAATAAATAA-ATGTTC  Hsapiens
  416568: ...........................................-......  Ptroglodytes
  434071: ...........................................-......  Ggorilla
  470108: ...........................................-......  Pabelli
  170395: .T.C...GC..AAGG...                                  Mmulatta
  466917:                    ........................-......  Mmulatta
  416930:                    ........................-......  Panubis
  436657: ........C........A.........................G....C.  Cjacchus
 1076328: ........C...G.......T......................A....CA  Sboliviensis

              96951     96961     96971     96981     96991
   96942: TCTTTAATTTGTAACTTCACAATGCTGAGAAAACTTTACAGCCTTCATAA  Hsapiens
  416617: ..................................................  Ptroglodytes
  434120: ..................................................  Ggorilla
  470157: .......-..........................................  Pabelli
  466947: .--..C........T..A.G....................A......G..  Mmulatta
  416960: .--..C........G..A.G..C.................A......G..  Panubis
  436707: .................A................................  Cjacchus
 1076378: .T...............A................................  Sboliviensis

              97001     97011     97021     97031     97041
   96992: GGAAGTGAGGTCCAGGAAAATCTAGGAGATATTTGTTAACCAACCTATAA  Hsapiens
  416667: ..................................................  Ptroglodytes
  434170: .......T..........................................  Ggorilla
  470206: ........................A...G.....C...............  Pabelli
  466995: ...............A...G..............C.C......T......  Mmulatta
  417008: ...............A...G..............C.C......T......  Panubis
  436757: .........T............C-.A....G...C........T......  Cjacchus
 1076428: .........T....................G...C........T......  Sboliviensis

              97051     97061     97071     97081     97091
   97042: AGACATTAGTAATGACAGGATATTTCCTGAAAGTGTAATTTCCCATTGAG  Hsapiens
  416717: ..................................................  Ptroglodytes
  434220: ..................................................  Ggorilla
  470256: ..................A...............................  Pabelli
  467045: ..G...............................................  Mmulatta
  417058: ..G...............................................  Panubis
  436806: ..G..................G................A...........  Cjacchus
 1076478: ..G.......T..........G...........C....A...........  Sboliviensis

              97101     97111     97121     97131     97141
   97092: GATTTGTTTTTAATTTCTGGATTCCTGGATCCAATGAAGTTGGCATAGGT  Hsapiens
  416767: .............................G....................  Ptroglodytes
  434270: .............................G....................  Ggorilla
  470306: .......C.....................G..........C......T..  Pabelli
  467095: .............................G.................T..  Mmulatta
  417108: .............................G.................T..  Panubis
  436856: .............................G........T..A.TG..T..  Cjacchus
 1076528: .............................G...........A.TG.....  Sboliviensis
```

```
               97151     97161     97171     97181     97191
                   :         :         :         :         :
   97142: TTATGAAATGCCAAGATACATAAGTTGGCAAGTGTTCACATGCAAAAAAC  Hsapiens
  416817: ................G.................................  Ptroglodytes
  434320: ................G.................................  Ggorilla
  470356: ................G.................................  Pabelli
  467145: .........AGTG...G......C..................-...C.C.  Mmulatta
  417158: .........AGTG...G......C..................-...C...  Panubis
  436906: .........A......G..............A..................  Cjacchus
 1076578: .........A......G.................................  Sboliviensis

               97201     97211     97219     97229     97239
                   :         :         :         :         :
   97192: TTCTTGGAATTCCTGAGCTCTCTGT--GGCAATATATGACATCAGGATAT  Hsapiens
  416867: .........................--.......................  Ptroglodytes
  434370: .........................--......................C  Ggorilla
  470406: ....C....................--.......................  Pabelli
  467194: .................T.....A.AT.........C.............  Mmulatta
  417207: .................T.....A.AT.........C.............  Panubis
  436956: ...........G.G.CAT.......--.A...C.C.............--  Cjacchus
 1076628: ...C.......GTG.CAT.......--.A...C............A..--  Sboliviensis

               97249     97259     97269     97279     97289
                   :         :         :         :         :
   97240: GTCCCATCTCGCACATCAGGATATGTCCTGTCAAGAATGTCTATCACATG  Hsapiens
  416915: ....................................C.............  Ptroglodytes
  434418: ....................................C.............  Ggorilla
  470454: ..........A..............G...C..T...C.............  Pabelli
  467244: ..........T.....................T..CC..C.C........  Mmulatta
  417257: ..........T.....................T..CC..C.C.......A  Panubis
  437002: ----------------------..........T.TGC.............  Cjacchus
 1076674: ----------------------..........T.TCC.A....C..AG..  Sboliviensis

               97299     97309     97319     97321     97331
                   :         :         :         :         :
   97290: CCAGGAGTACTTTTTAGGAACAGAAAAAAA--------TGTCTGAAATGG  Hsapiens
  416965: ..............................--------............  Ptroglodytes
  434468: ..............................--------............  Ggorilla
  470504: .......A.A....................----------C........A  Pabelli
  467294: .........................G....ATGTCTGA..C....C...A  Mmulatta
  417307: .........................G....ATGTCTGA..C....CG..A  Panubis
  437030: .                                                   Cjacchus
  437183:  ......C......A...............A-------..CTG......A  Cjacchus
 1076702: .                                                   Sboliviensis
 1076855:  ......C......................AAA-----..CTG......A  Sboliviensis

               97341     97351     97361     97371     97381
                   :         :         :         :         :
   97332: TTTCTCATTTGAACTCATCCAAGCTTTCTCTAAATTTAAGCAAACTCCTG  Hsapiens
  417007: ..................................................  Ptroglodytes
  434510: ...............................................A..  Ggorilla
  470546: ..................................................  Pabelli
  467344: .............T....................................  Mmulatta
  417357: .............T....................................  Panubis
  437225: C..T...........A...................A..............  Cjacchus
 1076899: ...T...........A.....C........------..........T...  Sboliviensis
```

```
                 97391      97401      97411      97419      97429
  97382: GTCATTTTCAGTTAGTACCTTTCCTCAAGTTCAACCT--TCATGACAAAC  Hsapiens
 417057: .....................................--...........  Ptroglodytes
 434560: .....................................--...........  Ggorilla
 470596: ..........A.......T..................--...........  Pabelli
 467394: ............................C...C....--...........  Mmulatta
 417407: .....................................--...........  Panubis
 437275: .............G.......................CT...........  Cjacchus
1076943: ..........A.A....T.....T..........T..CT....A......  Sboliviensis

                 97439      97449      97459      97469      97479
  97430: CTCAGCATCTCAGAAGATTTAGCCATAGTCTGAAATTCTCTTCCATAGAC  Hsapiens
 417105: .................................................T  Ptroglodytes
 434608: .................................................T  Ggorilla
 470644: ...........................A.....................T  Pabelli
 467442: .............................A...................T  Mmulatta
 417455: .............................A...................T  Panubis
 437325: .....T.......G.T.................................T  Cjacchus
1076993: .............G.....C.........................C...T  Sboliviensis

                 97489      97499      97509      97519      97529
  97480: TGGTCCCCTGTAATCCCAGTTTGCCTCAGCTTGTTATCCTGCTTTTTATT  Hsapiens
 417155: .................G................................  Ptroglodytes
 434658: .................G................................  Ggorilla
 470694: .............C...G.....T.......................T..  Pabelli
 467492: ATA..........C.........T.................T.....--.  Mmulatta
 417505: AT...........C.........T.................T.....--.  Panubis
 437375: ....T....A...C.........T.....T...C.......T.....---  Cjacchus
1077043: ....A....A...G.........T.....T...C.T.....T.....--.  Sboliviensis

                 97539      97548      97558      97568      97578
  97530: CCCCTCTATTCCCAGG-CTGAGCTTCTTGCTTCTGTCCTATGAGACGTTA  Hsapiens
 417205: ................-.................................  Ptroglodytes
 434708: ................-.................................  Ggorilla
 470744: ................-.....T...........................  Pabelli
 467540: ......C.........GT..........A...........G....T....  Mmulatta
 417553: ......C.........GT......................G....T....  Panubis
 437422: -.....C...T.....-...................T....-..G.A...  Cjacchus
1077091: T.....C.........-...................T....-....A...  Sboliviensis

                 97588      97598      97608      97618      97628
  97579: GATTCCTTCGCTTTGGTACCCAAGTAAACCCATCCTTCTCCATATACAGG  Hsapiens
 417254: ...................................A..............  Ptroglodytes
 434757: ..................................AG..............  Ggorilla
 470793: ..G...............................T.G.....C.......  Pabelli
 467590: .........T............G.............A....G.G..G...  Mmulatta
 417603: .........T............G.............A....G.G..G...  Panubis
 437469: T........T......................GA..A.......C.G...  Cjacchus
1077139: T........T......................GA..A.......C.G...  Sboliviensis
```

```
                   97638      97646      97656      97666      97676
                       :          :          :          :          :
   97629: AAGGTCCATTTTTCT--CTTACAGCCCTGGATGCAGACTCAGCTAAGAAG  Hsapiens
  417304: ...A.........G.--....T............................  Ptroglodytes
  434807: .............G.--....T............................  Ggorilla
  470843: ......T......GC--....T......................G.....  Pabelli
  467640: ............CA.--..C.TG.....................G.....  Mmulatta
  417653: ............CA.--..C.TG.....................G.....  Panubis
  437519: ...A.........G.--....T..........A..........AG.....  Cjacchus
 1077189: ..A..........T.GG....T..........A..........AG.....  Sboliviensis

                   97686      97696      97706      97716      97726
                       :          :          :          :          :
   97677: ACCATTATTCATTTTTGGAATTCTTCATCTAGGATATTTCCTCTTGTTTC  Hsapiens
  417352: ................T.................................  Ptroglodytes
  434855: ..................................................  Ggorilla
  470891: ..............................C.AT................  Pabelli
  467688: ..............................CAT.................  Mmulatta
  417701: ..............................C.T......T..........  Panubis
  437567: ......C...G.............G.....CA.............A...-  Cjacchus
 1077239: C.....C.................G.....CA.............A...-  Sboliviensis

                   97736                            97740      97742
                       :                                :          :
   97727: TTTCTCTCCT--------------------------ATCT--------TT  Hsapiens
  417402: ..........--------------------------....--------..  Ptroglodytes
  434905: ..........--------------------------....--------..  Ggorilla
  470941: ....C.....--------------------------....--------..  Pabelli
  467738: ...T...T..--------------------------....--------..  Mmulatta
  417751: ...T...T..--------------------------....--------..  Panubis
  437616: ...T...T..--------------------------....--------..  Cjacchus
 1077288: ...T...T..TCCATGAGATCCGATAAACCCATTGG....CATGAGAC..  Sboliviensis

                   97752      97758      97768      97778      97788
                       :          :          :          :          :
   97743: GAGCTTTTTAGATC----ATCAACACCCCATTAGTCTATTACCCAACTTA  Hsapiens
  417418: .............A----................................  Ptroglodytes
  434921: ..............----................................  Ggorilla
  470957: ...........T..----......................C..T......  Pabelli
  467754: .GA........T..----......................C.........  Mmulatta
  417767: .GA........T..----......................C.........  Panubis
  437632: .GA........T..----..........T.A.........C..T......  Cjacchus
 1077338: AGA........T..GTAAG..T.TT......A........C.........  Sboliviensis

                   97798      97808      97818      97828      97838
                       :          :          :          :          :
   97789: AATCAGGGAACTTATACCTCCCAAACTCATTCAGAGACTCCAAACATATA  Hsapiens
  417464: ........................T.........................  Ptroglodytes
  434967: .......................GT.........................  Ggorilla
  471003: ....-...................T...............A.........  Pabelli
  467800: ...............G........T...............G........G  Mmulatta
  417813: ...............G........T...............G........G  Panubis
  437678: .................T......T..........C.T.T-----.....  Cjacchus
 1077388: ..........T......T......T............T.TG........G  Sboliviensis
```

```
               97848     97858     97868     97878     97888
   97839: TATTGATACAGGAGACCTAAGAAGAGCATGTCTTGGGGGTTGAGGAAACA   Hsapiens
  417514: ..................................................   Ptroglodytes
  435017: ..................................................   Ggorilla
  471052: ..........................................G.......   Pabelli
  467850: ..........................T.........A.....G.......   Mmulatta
  417863: ...................G......T.........A.....G.......   Panubis
  437723: ...C...................C..................T.A.....   Cjacchus
 1077438: ...CA..........T.......C....C.............G.......   Sboliviensis

               97898     97908     97918     97928     97938
   97889: GGCAGGTGAGAAACTTCCAGATTGGAAACACAGCTTCCTTTCTCCCGTCC   Hsapiens
  417564: ................T................T................   Ptroglodytes
  435067: ................T....................G....           Ggorilla
  471102: ................T.............T..G.......T...TC...   Pabelli
  467900: ..............G.T.......T.........................   Mmulatta
  417913: ..............G.T.................................   Panubis
  437773: ..A.....G.......T.C..C.............C.......G..T...   Cjacchus
 1077488: ..A.............T.T..C.............C..........T...   Sboliviensis

               97948     97957     97967     97977     97987
   97939: AGCCCCTACTT-CATCCTATCTGTTTCCGGAACCTTGTTGTAGATGAATC   Hsapiens
  417614: ...........-..A...........................C.......   Ptroglodytes
  471152: ...........-........G.....AT..........C...........   Pabelli
  467950: ...........C........G.A....T......................   Mmulatta
  417963: ...........C........G.A....T...................T..   Panubis
  437823: .C......A..C........G......T....A...---...........   Cjacchus
 1077538: .T...T...G.C......C.G......T....A.................   Sboliviensis

               97996     98006     98016     98026     98036
   97988: TCCCT-TGACTTCATGATGTGCTGAGAAAACAAACTCATGGCTGGTGTTA   Hsapiens
  417663: .....-......TG....................................   Ptroglodytes
  471201: .....-GA....TG........................CA..........   Pabelli
  468000: .....-...T..TG........................CT.........G   Mmulatta
  418013: .....-...T..TG........................CT..........   Panubis
  437870: .G...A......TG.....................C.......CC.....   Cjacchus
 1077588: .G...A......TG........................C....CC.....   Sboliviensis

                                       LINE1
                       -------------------------------------->
               98046     98056     98066     98076     98086
   98037: AAAAGGGCCCATGACAATACCAAGTGTTGGGGAGAATGTGGAGAAATCAG   Hsapiens
  417712: ..................................................   Ptroglodytes
  471250: ..................................G...CA....C.....   Pabelli
  468049: ..................................G...............   Mmulatta
  418062: ..................................G...............   Panubis
  437920: .....T..A....                                        Cjacchus
  438213:              ......A...........A..G.C.....AT......   Cjacchus
 1077638: .....T..A..........A.........A.A..G.......AT......   Sboliviensis
```

```
                                     LINE1
         ------------------------------------------------------->
               98096     98106     98116     98126     98136
                   :         :         :         :         :
  98087: AACTCTATTCACAGTCGGTTGGAATGCACACTTGTGCAGAATTCTATGGA  Hsapiens
 417762: ............G.....................................  Ptroglodytes
 446903:                                               ....  Ggorilla
 471300: ............G.....................................  Pabelli
 468099: ...............A..................................  Mmulatta
 418112: ...............A....................T.............  Panubis
 438250: .G..........G..TC..G..........A........C..-.......  Cjacchus
1077688: ...........TG...A..........GT.A........CG.-.......  Sboliviensis

                                     LINE1
         ------------------------------------------------------->
               98146     98156     98166     98176     98186
                   :         :         :         :         :
  98137: GAAGAGTCTGGCATTTCCTCAAAATGTTAACCTGGATTTACCATATGACC  Hsapiens
 417812: .................................A................  Ptroglodytes
 446907: ......................C.............G.............  Ggorilla
 471350: ....................................A.............  Pabelli
 468149: .................................A..G.............  Mmulatta
 418162: .................................A..G.............  Panubis
 438299: A...................G...CA.......A..G.............  Cjacchus
1077737: A...................G....A.......AA.G......C......  Sboliviensis

                                     LINE1
         ------------------------------------------------------->
               98196     98206     98216     98226     98236
                   :         :         :         :         :
  98187: CAGCGATTTCATTCATAGGTTTATACTCAAAAGAAATGAAGAAATATGCC  Hsapiens
 417862: ..........................................C.......  Ptroglodytes
 446957: ...T..............................................  Ggorilla
 471400: ..........................................C.......  Pabelli
 468199: .....G..............................G.....C.......  Mmulatta
 418212: .....G..............................G.....C.......  Panubis
 438349: G..T................A.....CG..............C.......  Cjacchus
1077787: G..T...........C....A.....C...............C.......  Sboliviensis

                                     LINE1
         ------------------------------------------------------->
               98245     98255     98265     98275     98285
                   :         :         :         :         :
  98237: ATGCAAA-AAAATGTACATGAAAAGTCAAAACATCATTATTCATAATAGT  Hsapiens
 417912: .......-.....A................G...................  Ptroglodytes
 447007: .......-..........................................  Ggorilla
 471450: ......G-...............G....C.........G.....G.....  Pabelli
 468249: .....G.C...............G....G.G..C................  Mmulatta
 418262: .....G.C...............G....G.G..C................  Panubis
 438399: ...G...AT.T.C..........G....G.G.......-..........C  Cjacchus
1077837: ...GGG.A.....A.....A...G....G.G.......-.C..C.....C  Sboliviensis

                                     LINE1
         ------------------------------------------------------->
               98295     98305     98315     98325     98335
                   :         :         :         :         :
  98286: AAATGGATGGAAACAACACAAATGTCCATCAACTTATGAATAAAGAAAAT  Hsapiens
 417961: .......................A..........................  Ptroglodytes
 447056: .......................A..........................  Ggorilla
 471499: ...A..............................................  Pabelli
 468299: ...A..G..A........................GCCTG...C.C.....  Mmulatta
 418312: ...A..................................C..G........  Panubis
 438448: ...AA...A...........................C.............  Cjacchus
1077886: ...AA...A...................G.....................  Sboliviensis
```

LINE1

```
              98345     98355     98365     98375     98385
   98336: GTGGTCTATTCATAGAATGGAATATTATTCGACCACAAAAAGGAATGATG  Hsapiens
  418011: ..................................................  Ptroglodytes
  447106: ..................................................  Ggorilla
  471549: ...A..............................................  Pabelli
  468349: ..--.AA..C....C.....G.............................  Mmulatta
  418362: .....G........C...................................  Panubis
  438498: ...T.........TC.........G..----G..CA..............  Cjacchus
 1077936: ...C.........TC............----G.............C....  Sboliviensis
```

LINE1

```
              98395     98405     98415     98425     98435
   98386: TACTGATCCATGCAATGACGTGGACAAACCTTGAAGATAATACTAGATGA  Hsapiens
  418061: ...............CA.T...............................  Ptroglodytes
  447156: ...............CA.T...............................  Ggorilla
  471599: ..................T..........G..................A.  Pabelli
  365370:                                  ..A..............  Nleucogenys
  468397: ..........C....CA..A...............A.C..........C.  Mmulatta
  418412: ..........C....CA..A...............A.C..........C.  Panubis
  438544: C......TTG..TG.CA..A.A..TG...T.....A.....T..G.-...  Cjacchus
 1077982: .......T....TG.CA..A....TG...A.....A........G.-...  Sboliviensis
```

LINE1

```
                        98448               98466     98476
   98436: AAGAAG-------CCAGTCACAAAAGGAC--TTACTGTATGATTCGATTT  Hsapiens
  418111: ......-------..............G.--A.............C....  Ptroglodytes
  447206: ......-------..............G.--G....A........C....  Ggorilla
  471649: .....A-------................--G.............C....  Pabelli
  365387: ......-------T...............--..............C....  Nleucogenys
  468447: ......-------................--G.............C....  Mmulatta
  418462: ......-------................--A.............C....  Panubis
  438593: ......AGCC---..CA.TG.........AAG.............C....  Cjacchus
 1078031: ......AGCCCCC..CA.A..........AA.............TC...C  Sboliviensis
```

LINE1

```
              98486     98496     98506     98516     98526
   98477: ACGTGAAATGTTTAGAATAGGCAAATCCATAGAAACAGGAGGTAGATTAC  Hsapiens
  418152: .T..............................................C.  Ptroglodytes
  447247: .T..............................................C.  Ggorilla
  471690: ..A...........A................A................G.  Pabelli
  365428: ..AA.................T..........................C.  Nleucogenys
  468488: .TA........................................G....C.  Mmulatta
  418503: .TA.......................................CG....C.  Panubis
  438640: .TA...........C..G..A...........................CA  Cjacchus
 1078081: .TA.......C...C..G..A...........................CA  Sboliviensis
```

```
                              LINE1
           ------------------------------------------------------>
                    98536     98546     98556     98566     98576
                        :         :         :         :         :
   98527: TGGTTTCCAGGGTCTCGAGTAAGGGAAGAACGAGATACAAGTTTTCTTTT  Hsapiens
  418202: ...........A....C..A....................A.........  Ptroglodytes
  447297: ..C........A....C..A....................A.........  Ggorilla
  471740: ...........C...T..............T..AG...............  Pabelli
  365478: ..............................T..AG..TG..G....C...  Nleucogenys
  468538: ..A.............A..A..........T..AG......           Mmulatta
  418553: ..A.............A..A..........T..AG......           Panubis
  438690: ...............T...AG......A..G..AGG.....G........  Cjacchus
 1078131: ...............T...AG....G.A..G..AG......A........  Sboliviensis

                              LINE1
           ------------------------------------------------------>
                    98586     98596     98606     98616     98626
                        :         :         :         :         :
   98577: GGAGGTAGTGAAATTGTTGTGGAACGAGATCATGATGGTGATAGCACAAC  Hsapiens
  418252: ........................T.........................  Ptroglodytes
  447347: ........................T...............G.........  Ggorilla
  471790: ......................-.T.........................  Pabelli
  365528: ........................T....................C....  Nleucogenys
  438740: .........A.....A........T................C........  Cjacchus
 1078181: .....C...A....AA......C.T..........CA.C..C........  Sboliviensis

                              LINE1
           ------------------------------------------------------>
                    98636     98645     98655     98665
                        :         :         :         :
   98627: TTTGTGAATATAATAAAA-TCATTGAATTGTACAGTTGAA----------  Hsapiens
  418302: ..................-.....................----------  Ptroglodytes
  447397: ..................-.....................----------  Ggorilla
  471839: .............C....-.T...A.......T.......----------  Pabelli
  365578: ..................-.T...........T.......----------  Nleucogenys
  438790: .........G..C.....A...C....G............AATAGTGAAC  Cjacchus
 1078231: .........G..C.....A...CC...G..G...AC....AATGGTAAAC  Sboliviensis

                     LINE1
           ----------------------------------->
                    98675     98685     98693     98703     98713
                        :         :         :         :         :
   98666: TTTGTGGCATATAAATTATATGTTAA--AAAAGGGGGTCCACAAAACAAA  Hsapiens
  418341: .......T...........G......-A......................  Ptroglodytes
  447436: .......T...........G......--......................  Ggorilla
  471878: .......T..................TA......................  Pabelli
  365617: ...A...T..................TA...T..................  Nleucogenys
  438840: C..AG..T.............C.C..TA..........T.........--  Cjacchus
 1078281: C..A...T...........C.C.C..TA....................--  Sboliviensis

                    98723     98733     98743     98753     98763
                        :         :         :         :         :
   98714: CAGCCCCCCACTCTGGTTGTCAGGGAGATATTGGATTAAATGGCCTTGGA  Hsapiens
  418390: ..................................................  Ptroglodytes
  447484: ...-..............................................  Ggorilla
  471928: ...................................C..............  Pabelli
  365667: ........................T.........................  Nleucogenys
  438888: --.TT...T..A.G...A..G.CA..........................  Cjacchus
 1078329: --.TT...T.TG.....A..G.T..................CT.......  Sboliviensis
```

```
              98772     98782     98792     98802     98812
                  :         :         :         :         :
   98764: CAACAA-CCCTCTCCCTGGCCACAGACATTCTTCAGATTACAAGATATTC  Hsapiens
  418440: ......C...........................................  Ptroglodytes
  447533: ......C...........................................  Ggorilla
  471978: ......-.................................TG........  Pabelli
  365717: ......-...........A...............................  Nleucogenys
  438936: ..G...-T..............T........C..................  Cjacchus
 1078377: ...T..-T..............T......GAC.............G....  Sboliviensis

              98822     98832     98842     98852     98862
                  :         :         :         :         :
   98813: CAGAGGAAACACTGGAATGAGTCTGAAGCCAGGTGCTAAATGGAAGGACC  Hsapiens
  418490: ...G.........................A....................  Ptroglodytes
  447583: ...G..............................................  Ggorilla
  472027: ...G..........T.........................C.........  Pabelli
  365766: ...G..............................................  Nleucogenys
  438985: ....A.............A.........G..............T......  Cjacchus
 1078426: ....A..................G....G....CT........T......  Sboliviensis

              98872     98882     98892     98902     98912
                  :         :         :         :         :
   98863: ACCAAGAAACGTTGTGATCCTGACAGGTCAAGCAACTTCTTTTTCTGCTT  Hsapiens
  418540: ...G................C.............................  Ptroglodytes
  447633: ...G..............................................  Ggorilla
  472077: ...G.................C................A...........  Pabelli
  365816: ...G.....T............................A...........  Nleucogenys
  439035: .........T...........A....C....A......A...........  Cjacchus
 1078476: .-.............TT....A....C....A......A...........  Sboliviensis

              98922     98932     98942     98952     98960
                  :         :         :         :         :
   98913: AATTTTTAAATGAAAAATTAGAAAGCTGACATTCAAAATGGCCC--GTCT  Hsapiens
  418590: C...........................C............T..CT....  Ptroglodytes
  447683: C...........................C............T..CT....  Ggorilla
  472127: C...........................C..............----...  Pabelli
  365866: C...........................C........G......CT....  Nleucogenys
  439085: C..........A................C.............T.CT..T.  Cjacchus
 1078525: C.....A....-.............TCAC.T...........T.CT..T.  Sboliviensis

                                                     LTR
                                                ----------->
              98970     98980     98990     99000     99010
                  :         :         :         :         :
   98961: GTTTCAATTGCTCTTCTCAGTGTCAGCCTGTTAACTCAATGTGTTAGTCT  Hsapiens
  418640: .............C....................................  Ptroglodytes
  447733: .............C....................................  Ggorilla
  472173: ......................................G...........  Pabelli
  365916: ..........T..............A............GA..........  Nleucogenys
  439135: .........TT.......C....TG.............G.A.........  Cjacchus
 1078574: .....G...TT.......C...................G.A.A.......  Sboliviensis

                                  LTR
          -------------------------------------------------->
              99020     99030     99040     99050     99060
                  :         :         :         :         :
   99011: GTTTTCATGCTGCTGATAAAGACATACCTGAGATTAGGAAGTAAAAGAGG  Hsapiens
  418690: ..........................T.C......G..............  Ptroglodytes
  447783: ............................C......G..............  Ggorilla
  472223: ...........................G....-C.G.C...A........  Pabelli
  365966: ............................CA...C.G.C...A........  Nleucogenys
  439185: ................C.......CG.......C.G.....A........  Cjacchus
 1078624: ................C........G.......C.G.....A........  Sboliviensis
```

```
                                   LTR
          ------------------------------------------------------>
                   99070     99080     99090     99100     99110
   99061: TTTAATTGGACTTAGAGTTCCACGTGATTGGGGAGGCCTCAGAATCACGG  Hsapiens
  418740: .........................T.....................T..  Ptroglodytes
  447833: .........................T.....................T..  Ggorilla
  472272: ...............................................T..  Pabelli
  366016: ...T...............T..............................  Nleucogenys
  439235: ..............C.T......A...C.T.AA..............T..  Cjacchus
 1078674: ..............C.T..........C.TAA...............T..  Sboliviensis

                                   LTR
          ------------------------------------------------------>
                   99120     99129     99139     99149     99159
   99111: TAGGAGGCAAAAGTTA-TTCTTACATGGTGGCTGCAAGAGAAAATGAGGA  Hsapiens
  418790: .G..............-.................................  Ptroglodytes
  447883: ................-.................................  Ggorilla
  472322: .G..............-.................................  Pabelli
  366066: .G..............-.................................  Nleucogenys
  439285: C......T.....A..C........C.T..T.A.................  Cjacchus
 1078724: C......T.....A..C........CAA....A......T..........  Sboliviensis

                                   LTR
          ------------------------------------------------------>
                   99169     99179     99189     99199     99209
   99160: AGAAGCAAAAGAAGAAACCCCTAATAAACCCATTGGATCTCCTGAGACTT  Hsapiens
  418839: ......................G...................A.......  Ptroglodytes
  447932: ......................G..........C................  Ggorilla
  472371: ....................T.C..........C................  Pabelli
  366115: ....................A.G..........CA...............  Nleucogenys
  439335: ...........C.....T.T..G.........GCA......A........  Cjacchus
 1078774: ...........C.....T.T..G..........C.......A........  Sboliviensis

                                   LTR
          ------------------------------------------------------>
                   99219     99229     99239     99249     99259
   99210: ATTAACTATCATGAGAATAGCACAAGAAAGACCGGCCCCCATGATTCAAT  Hsapiens
  418889: ..................................................  Ptroglodytes
  447982: ..................................................  Ggorilla
  472421: ...........C.....G...G...............TT...........  Pabelli
  366165: .................................A................  Nleucogenys
  468590:                ...G.....G.......A..TG...G.........  Mmulatta
  418605:                ...G.....G.......A..TG...G.........  Panubis
  439385: T..........C..........TGG........A................  Cjacchus
 1078824: T..........C..........TGG........A................  Sboliviensis

                                   LTR
          ------------------------------------------------------>
                   99269                 99280     99290     99300
   99260: TACCTCTACCTGGG---------TCCCTCCAACAACATGTGGAAATTCTG  Hsapiens
  418939: ..............---------.........................C.  Ptroglodytes
  448032: ..............---------.........................C.  Ggorilla
  472471: G............A---------...........................  Pabelli
  366215: .............TAACAGCCTC....A......................  Nleucogenys
  468625: ......C.......---------...........G...............  Mmulatta
  418640: ......C.......---------...........G...............  Panubis
  439435: ......CT......---------......TC..C...A....G.......  Cjacchus
 1078874: ......CT......---------......T.G.C...A....G.......  Sboliviensis
```

```
                                       LTR
          ---------------------------------------------------->
                    99310     99320     99329     99339     99349
    99301: GTAGATACAATTCAAGTTGAGATTT-GGGTGGGAACACAGCCAAACCATA  Hsapiens
   418980: .........................-........................  Ptroglodytes
   448073: .........................-........................  Ggorilla
   472512: .............C...........-........................  Pabelli
   366265: ....................C....-........................  Nleucogenys
   468666: .........................-.......G................  Mmulatta
   418681: .........................G...G....................  Panubis
   439476: .G...............CA......-.......G............T...  Cjacchus
  1078915: .G...............CA......-.......G............T...  Sboliviensis

           LTR
           --->
                    99359     99369     99379     99389     99399
    99350: TCACTCAGCAAGGCAGATAACTTTCTCACTGAGCCTATGCAACAGAAAAC  Hsapiens
   419029: .......A....AT..................C.................  Ptroglodytes
   448122: ................................C.................  Ggorilla
   472561: .......A.....T..........G.......C...........C.....  Pabelli
   366314: ............AT..................C.................  Nleucogenys
   468715: .............T.A................CT.........C......  Mmulatta
   418731: .............T.A................CT.........C......  Panubis
   439525: .G..G.G......TCA.....A..........C.................  Cjacchus
  1078964: .G..G........TCA..G.............C....A............  Sboliviensis

                    99409     99419     99429     99439     99449
    99400: CATCTGGGATGGTTGTAAGGGGCACAGGAAGTGACTGGTAGGATCACTGC  Hsapiens
   419079: ..................................................  Ptroglodytes
   448172: ..................................................  Ggorilla
   472611: ...............C.........G...G....................  Pabelli
   366364: -..............C.........G...G.....C..............  Nleucogenys
   468765: -........................G...GT......-............  Mmulatta
   418781: -........................G...GT......-............  Panubis
   439575: -.....A...A....CG........----------...............  Cjacchus
  1079014: -..............C.........----------...............  Sboliviensis

                    99459     99469     99479     99484     99494
    99450: CAAAGCTGAGCATTCAGGAGAAGGCAATAG-----AATCCTATTCTCCAT  Hsapiens
   419129: ...........G..................-----......G........  Ptroglodytes
   448222: ..............................-----......G........  Ggorilla
   472661: ...GA.....................G...AATCA...............  Pabelli
   366413: ...G..........................-----......G........  Nleucogenys
   468813: ...G..A.......................-----......G....T...  Mmulatta
   418829: ...G..A.......................-----......G....T...  Panubis
   439614: ......A......G.G............G.-----......G........  Cjacchus
  1079053: ......A......G............G.G.-----......G.......C  Sboliviensis

                    99504     99514     99524     99534     99544
    99495: AGTATGCTATAAGATACTGAAGTACACTTCTTCACTATCTCTTTGGACTT  Hsapiens
   419174: .....A....G.......................G...............  Ptroglodytes
   448267: .....A....G.......................G...............  Ggorilla
   472711: ..........G.........G.............G...............  Pabelli
   366458: ..........G.........G.............G...............  Nleucogenys
   468858: ..........G.........G.............G...............  Mmulatta
   418874: ..........G.........G...T.........G...............  Panubis
   439659: ..........G.......C...C...........G...............  Cjacchus
  1079098: ..........G....C..C...C.........T.G...............  Sboliviensis
```

```
                 99554     99564     99574     99584     99594
                     :         :         :         :         :
   99545: AGAATTAGCACTATATTCCTTGTTATACAGAAAAATTACTAAGGAAATTC  Hsapiens
  419224: ..........................................T.......  Ptroglodytes
  448317: ..........................................T.......  Ggorilla
  472761: ........G............C...........................T  Pabelli
  366508: ..........................................T.......  Nleucogenys
  468908: ...............................T..-........-......  Mmulatta
  418924: ...............................T..-........-......  Panubis
  439709: ........A...........CA..C..........C.G............  Cjacchus
 1079148: ........A...........G...C............G............  Sboliviensis

                 99604     99614     99624     99634     99644
                     :         :         :         :         :
   99595: ATAGGATGACAAAAACTTTCAGAACTGAAAAACAGGAAATGTAAGCTTTT  Hsapiens
  419274: ..................................................  Ptroglodytes
  448367: G.................................................  Ggorilla
  472811: G.......................................A.........  Pabelli
  366558: G.................................................  Nleucogenys
  468956: ..G....A..........................A...............  Mmulatta
  418972: ..G....A.........................................G  Panubis
  439759: ..................................................  Cjacchus
 1079198: ..................................................  Sboliviensis

                 99654     99664     99674     99684     99694
                     :         :         :         :         :
   99645: TAGTTCTTTGGTATTCGAAGTATGCCTAAAAGACAATGCAAAATCCAAGA  Hsapiens
  419324: ................A.................................  Ptroglodytes
  448417: ................A.................................  Ggorilla
  472861: .....G..........A.................................  Pabelli
  366608: .....G...A......A.................................  Nleucogenys
  469006: .....G..........A........................C........  Mmulatta
  419022: .....G..........A........................C........  Panubis
  439809: .....G...T..G...A.C........T.........T...C........  Cjacchus
 1079248: .....T.A....G...A.T........T.........T...CC.......  Sboliviensis

                                       Simple
                           ------------------------------->
                 99704
                     :
   99695: AAAGAATGGTGGGGTTTT----T---------------------------  Hsapiens
  419374: ..................----.---------------------------  Ptroglodytes
  448467: ..................----.---------------------------  Ggorilla
  472911: ..................CGTT.---------------------------  Pabelli
  366658: ...T..A.......C...TGTT.---------------------------  Nleucogenys
  469056: .......A..........----.TTGTTCTTTTTTTTTTTTTTTTTTTTT  Mmulatta
  419072: .......A..........----.TTGTTCTTTTTTTTTTTTTTTTG----  Panubis
  439859: ........T.----C...----C---------------------------  Cjacchus
 1079298: ........T.--CT....----.---------------------------  Sboliviensis

                        Simple
          ---------------------------->
                 99721     99731     99741     99751     99761
                     :         :         :         :         :
   99714: --GTTTGTTTGTTTTTGTTTTTGTTTTACAGCTGGAGTAGAATACAAAGG  Hsapiens
  419393: --.........G.....G.....G..........................  Ptroglodytes
  448486: --.........G.....G.....G..................C.......  Ggorilla
  472934: --.................................G..............  Pabelli
  366681: --................................................  Nleucogenys
  469102: TG....T.......G.T.G...............................  Mmulatta
  419114: --....T.......G.T.G...............................  Panubis
  439874: --....T.........----------.........C....T........A  Cjacchus
 1079315: --..............----------......C.......T.........  Sboliviensis
```

```
                 99771     99781     99791     99801     99811
                     :         :         :         :         :
    99762: GATGGAGTTGAAACAAATGAGAGGAAATTGGAATTCTAAACTTATTCTCA  Hsapiens
   419441: ............................................C.....  Ptroglodytes
   448534: ............................................C.....  Ggorilla
   472982: ..................................................  Pabelli
   366729: ..........................................A.......  Nleucogenys
   469152: .....................G........A........C..........  Mmulatta
   419162: .....................G........A........C..........  Panubis
   439912: .............TG..C...G..C..............C..........  Cjacchus
  1079353: .............TG...............A........CG.........  Sboliviensis

                 99821     99831     99841     99851     99861
                     :         :         :         :         :
    99812: TTGGCATTAGAAAGGCACCTACATGTATTTCACATGAGCCGGTGACTGCT  Hsapiens
   419491: .........................C.............T..........  Ptroglodytes
   448584: ..................................................  Ggorilla
   473032: ....T............T.............G........A....T....  Pabelli
   366779: ..T..........................................T....  Nleucogenys
   469202: ..TAT..................G........A.......A....T....  Mmulatta
   419212: ..TAT..................G........A.......A....T....  Panubis
   439962: C...T............T.......C.....GA.......A...GT....  Cjacchus
  1079403: C.A.T....................C.....GA.......A...GT....  Sboliviensis

                 99871     99881     99891     99901     99911
                     :         :         :         :         :
    99862: GACTTGCATTCTTATTTTTTCCCTATAGATTAAAAAGGAGGTACAATGGT  Hsapiens
   419541: ..T...............................................  Ptroglodytes
   448634: ..T...............................................  Ggorilla
   473082: ..T.......T..............C...........T............  Pabelli
   366829: ..T.............-.................................  Nleucogenys
   469252: ..T..A....T.C..........................A........T.  Mmulatta
   419262: ..T.......T............................A........T.  Panubis
   440012: ..T.......-------......C................T....G....  Cjacchus
  1079453: .CT.......-------......C.....C.......A.TA....G....  Sboliviensis

                 99921     99931     99941     99951     99961
                     :         :         :         :         :
    99912: AGAACTGTAATCCTGTCCTTTGTCATAAATTTTCGTATTCATAAAGGTGA  Hsapiens
   419591: ...G..............................C...............  Ptroglodytes
   448684: ..................................C...............  Ggorilla
   473132: ..................................A...............  Pabelli
   366878: .....A......T.....................A...............  Nleucogenys
   469302: .....A......A..............C......A..C.T..........  Mmulatta
   419312: .....A......A.....................A..C.T.C........  Panubis
   440055: .....A.............G........G.....A..C...-G.......  Cjacchus
  1079496: .....A.................G..........T..C.......A....  Sboliviensis

                 99971     99981     99991    100001    100011
                     :         :         :         :         :
    99962: GTGTTAGCCCGCTTGTGAAATCTGAAGTTGAGTAACTTCAAATACTAACC  Hsapiens
   419641: .........T.............A..........................  Ptroglodytes
   448734: .........T.............A..........................  Ggorilla
   473182: .........T.............A..........................  Pabelli
   366928: .........T.............A..........................  Nleucogenys
   469352: .........T.............AG..................G......  Mmulatta
   419362: .........T.............A...................G......  Panubis
   440104: A........T.....C..........AC........--A......C....  Cjacchus
  1079546: A........TC....C...........C........--G......C....  Sboliviensis
```

```
              100018    100028    100038    100048    100058
                   :         :         :         :         :
  100012: ACAGA---GGGAGAGGCAGCAAGAGGAGAGGCATAAATTCAGGATCTCAC  Hsapiens
  419691: .....---..........................................  Ptroglodytes
  448784: .....---..........................................  Ggorilla
  473232: .....---..........................................  Pabelli
  366978: .....---.....................A....................  Nleucogenys
  469402: .....---..........................................  Mmulatta
  419412: .....---..........................................  Panubis
  440152: ....G-----------............................G.....  Cjacchus
 1079594: G....GCT.A.......G.T...........T..................  Sboliviensis

              100068    100078    100088    100098    100108
                   :         :         :         :         :
  100059: CCTTCATTCCACAGACACACATAGCCTCTCTGCCCACCTCTGCTTCCTCT  Hsapiens
  419738: .....................C............................  Ptroglodytes
  448831: .....................C............................  Ggorilla
  473279: .................--..C............................  Pabelli
  367025: ...............G.....C............................  Nleucogenys
  469449: .....................C..G............G............  Mmulatta
  419459: .............A.......C..G............G...C........  Panubis
  440191: .G................G..C..G...C...T.........T.......  Cjacchus
 1079644: .G................G..C..G...C....T......G.T.......  Sboliviensis

              100118    100128    100138    100148    100158
                   :         :         :         :         :
  100109: AGGAACACAGGTAAGAGCTTCAAGCCTCTCCAGCTTAATAACATGAATTA  Hsapiens
  419788: ...............T......................C...........  Ptroglodytes
  448881: ...............T..................................  Ggorilla
  473327: ...............T..................................  Pabelli
  367075: ...............T.....G................C...........  Nleucogenys
  469499: ...............T..........A.......................  Mmulatta
  419509: ...............T..........A.......................  Panubis
  440241: ......G........T..........A..............T..A.....  Cjacchus
 1079694: ...............T..........A.................A.....  Sboliviensis

              100168    100178    100188    100198    100208
                   :         :         :         :         :
  100159: TTTTTGAGAATAATAATGATACTGTGTTCTATATCATGCATCTCCTGCAT  Hsapiens
  419838: .........................................T........  Ptroglodytes
  448931: .........................................T........  Ggorilla
  473377: ......................................T...........  Pabelli
  367125: .........................A........................  Nleucogenys
  469549: ..................................................  Mmulatta
  419559: ...................C..............T...............  Panubis
  440291: C....T..C..............A.................T....T.GA  Cjacchus
 1079744: .....T..C..............A.................T....T...  Sboliviensis

              100218    100228    100238    100248    100258
                   :         :         :         :         :
  100209: TCTGTCTGATTATATTTTACTTATTCTGCCAGAGCAAAATTAAAATACCT  Hsapiens
  419888: ..................................................  Ptroglodytes
  448981: ..................................................  Ggorilla
  473427: ..................................................  Pabelli
  367175: ..................................................  Nleucogenys
  469599: ...C.........G..................G...T.............  Mmulatta
  419609: ....C...........................G..............T..  Panubis
  440341: ..........................C.T.C.G.............G...  Cjacchus
 1079794: .............................TG.G.................  Sboliviensis
```

```
                 100268     100278     100288     100298     100308
  100259: ATTTCATCTGATTTGTCCTTTATCTAAATTGCTTAGTTCCAAGTAAACCA  Hsapiens
  419938: ..............A................................T..  Ptroglodytes
  449031: ..............A................................T..  Ggorilla
  473477: ..........G..A.....................T..............  Pabelli
  367225: ..................................................  Nleucogenys
  469649: ...............................T..CA.......G......  Mmulatta
  419659: G..............................T..CA..............  Panubis
  440391: ......G......C.....................T.....T....G.A.  Cjacchus
 1079844: ....T.........T....................T.....T....G.A.  Sboliviensis

                 100318     100328     100338     100348     100358
  100309: AGGCACTTTTAGGAACACAGAGGGAGAGTGCCTTGCAGCCAGAGAGTCTT  Hsapiens
  419988: .........C........................................  Ptroglodytes
  449081: .........C........................................  Ggorilla
  473527: ..................................................  Pabelli
  367275: .........................A........................  Nleucogenys
  469699: GT.......................................T....C...  Mmulatta
  419709: GT.......................................T....C...  Panubis
  440441: .AA...CC.............A...A.......CA......C.A..C...  Cjacchus
 1079894: .AA.G.CC....C........A...........CC......C...AC...  Sboliviensis

                 100368     100378     100388     100398     100408
  100359: GAAGGAGATGTCAGGGACGCATCTTAACAGCTGGTTGGATGTGATCCACA  Hsapiens
  420038: ........C........T.........A......................  Ptroglodytes
  449131: ........                                            Ggorilla
  473577: ........C.......G.A..C.....G..........T........G..  Pabelli
  367325: ........C.....T............G......................  Nleucogenys
  469749: ..G.....C......A...T...G...G......................  Mmulatta
  419759: ........C......A..A........G......................  Panubis
  440491: ..T...A.CA.......T.TC......G.....A......AC........  Cjacchus
 1079944: ......AGCA.......A.TC......G.............G........  Sboliviensis

                 100418     100428     100438     100448     100458
  100409: GAGGTCTCCTGTTAGCATTCATTGTAAAGCCTTCCTACCTAGCCCTAGTG  Hsapiens
  420088: ...........................................T......  Ptroglodytes
  473627: ..AA...................A..........................  Pabelli
  367375: .......T..........................................  Nleucogenys
  469799: .......................A.....................C....  Mmulatta
  419809: .......................A................G....C....  Panubis
  440541: .....T....C......G.....A.....A....................  Cjacchus
 1079994: ..........C......G.....A.....A....................  Sboliviensis

                 100468                100482     100492     100502
  100459: TAGCCAGCAATGAAGG------AAAGAGGGTCTATTACTTATTTACAGTA  Hsapiens
  420138: ..A...........A.AAAGAT..........G..............A..  Ptroglodytes
  473677: ................------............................  Pabelli
  367425: ..............A.------.............C.........A.A..  Nleucogenys
  469849: ..A.....T.......AAAGAT.................G.....T.A..  Mmulatta
  419859: ..A.............AAAGAT......................G..A..  Panubis
  440591: ...A............AAAGAT.....T...................A..  Cjacchus
 1080044: ................AAAGAT.....T...................A..  Sboliviensis
```

```
             100512    100522    100532    100542    100552
 100503: GTCTTTAAAAACACTAATTTTGTGAGGCTTCTAATTAAGACATTAATATA  Hsapiens
 420188: ............-G..G......A..C..........G.......---..  Ptroglodytes
 473721: .............G..........C.C..........G............  Pabelli
 367469: .............G...C........C..........G............  Nleucogenys
 469899: .-...........G............C..CT......G...........G  Mmulatta
 419909: .............G............C..CT......G...........G  Panubis
 440641: ......GC.....A...CC.......T..........T..G....T.G..  Cjacchus
1080094: .......C.....G....C.......T..........T..G......G..  Sboliviensis

             100562    100572    100582    100592    100602
 100553: TTTAATATATGCACATTGTAGAAAGATTGAAACGTTAAAAATAAGATGAG  Hsapiens
 420234: ................................T............---..  Ptroglodytes
 473771: ..............................................A...  Pabelli
 367519: ...........................--------..........--...  Nleucogenys
 469948: ..C......A......................T.C...........AA..  Mmulatta
 419959: ..C......A......................T.C...........AA..  Panubis
 440691: .........................C.......A.......GT.AGAA..  Cjacchus
1080144: .........................T.......A.......GT.AGAA..  Sboliviensis

             100612    100622    100632    100642    100651
 100603: GAAAACTTTAAATGTCAAAATCTCACAACACAGATATATAA-TTTCTTTA  Hsapiens
 420281: A.......C....T.........................C.-........  Ptroglodytes
  91403:                                        ..-........  Ggorilla
 473821: A.................C..................G.C.-........  Pabelli
 367559: A....................................G.C.-........  Nleucogenys
 469998: A....................                               Mmulatta
 420009: A............................C.......G.C.-........  Panubis
 440741: A.......----.......C..C......C.......G.C.T........  Cjacchus
1080194: A.......----.................C.......G.C.T........  Sboliviensis

             100661    100671    100681    100691    100701
 100652: AGAAAATTGTACTACAAAATACCATTCCATTTATTAAAGTCATTCTGACA  Hsapiens
 420330: ..T...............................................  Ptroglodytes
  91413: G..GG.A.A..T.TT..G.C.AT..C.TC....                   Ggorilla
 473870: ........A.......................G......C....T.....  Pabelli
 367608: ................-------..........C................  Nleucogenys
 420058: .......CA...............C.G.......................  Panubis
 440787: ..............T.....GA..C.......G.G.............A.  Cjacchus
1080240: ..............T.....GA..C.........G.............A.  Sboliviensis

                                                       B3:7
                                                         <-
             100711    100721    100731    100741    100749
 100702: GGAATCTGATGCTTTTCCAGGAGTTCCAGATCACATCGAGT--TCACCAT  Hsapiens
 420380: .........................................--.......  Ptroglodytes
 153403:            .....TTTTTG...TT.....GTTATA...T-.T..A..  Ggorilla
 473920: .........................................--.......  Pabelli
 367651: .........................................--.......  Nleucogenys
 172477:          ..T.G...TT.CT....T....AT.GCTA.A.CA...T...  Mmulatta
 420108: .......................-.................--.......  Panubis
 440837: .....T...............................AG..--.......  Cjacchus
1080290: .....T..........T....................AG..--.......  Sboliviensis
```

```
                                 B3:7
         <--------------------------------------------------
             100759    100769    100779    100789    100799
                 :         :         :         :         :
 100750: GAATTCACTCAGTGAAGCCAACACCAAGTTCATGTTCGACCTGTTCCAAC Hsapiens
 420428: .......................................T.......G.. Ptroglodytes
 153441: .G.C..T..TGT.AC...A........A..TTGC..T..T..T..T...G Ggorilla
 473968: ...........................A...........T.......... Pabelli
 367699: ..........C............................T...-...... Nleucogenys
 172518: .G.......GG.C.CCAT..G...TCGA..TGG...T..T..T...A..G Mmulatta
 420155: .......................................T.......... Panubis
 440885: ......C........G.........C.....T..........A....... Cjacchus
1080338: ......C........G.........C.....T..G............... Sboliviensis
```

```
                                 B3:7
         <--------------------------------------------------
             100809    100811    100821    100831    100841
                 :         :         :         :         :
 100800: AGTTCAGAAAA--------TCAAAAGAGAACAACATCTTCTATTCCCCTA Hsapiens
 420478: ...........--------......A........................ Ptroglodytes
 153491: ..A.A..C...GATGATCG...T..A.-----....A..T.TC..T..CC Ggorilla
 474018: ...........--------..........................A.... Pabelli
 165531:   A.A..C...GATGATCG...T..A.-----....A..T.TC..T..CC Nleucogenys
 367748: ..                                                 Nleucogenys
 172568: ..C.G.AG...--------A....T..TGG...........T.......G Mmulatta
 420205: ...........--------...C..A.........C.............. Panubis
 440935: ...........--------......A..........T............. Cjacchus
1080388: ...........--------...G..A.---.................... Sboliviensis
```

```
                                 B3:7
         <--------------------------------------------------
             100851    100861    100871    100881    100891
                 :         :         :         :         :
 100842: TCAGCATCACATCAGCATTAGGGATGGTCCTCTTAGGAGCCAAAGACAAC Hsapiens
 420520: .................................................. Ptroglodytes
 153536: .G...C..T..G.T..CC.T..T.....A.G...G..C..T.G.AGTG.. Ggorilla
 474060: ........G............................G......A..... Pabelli
 165574: .G...C..T..G.T..CC.T..T.....T.G...G..C..T.G.A.TG.. Nleucogenys
 172610: .GG..C..TTGA.T..CA.T..C...C.....C.G..GA..CG..GAGC. Mmulatta
 420247: ...........A.......G.................G.......G.... Panubis
 440977: ...........A.........................G........A... Cjacchus
1080427: ...........A.........................G.......GA... Sboliviensis
```

```
                   B3:7
         <---------------------
             100901    100911    100921    100931    100941
                 :         :         :         :         :
 100892: ACTGCACAACAGATTAAGAAGGTAGCTATCAGCATCATTATGTTGTCCTG Hsapiens
 420570: ...........A.....C......................CA........ Ptroglodytes
 153586: .G......T......G.TG.....CG.G..                     Ggorilla
 474110: ...........A.....C......................C......... Pabelli
 165624: .G.....GT......G.TG.....CG.G..                     Nleucogenys
 172660: ..C..TTCC...T.GG..G....                            Mmulatta
 420297: ...........A.....C.......G.......T..-...C.C....... Panubis
 441027: ...........A.....C.......G.......T......C....A.... Cjacchus
1080477: ...........A.....C.......G....T..T......C...CA.... Sboliviensis
```

```
            100951    100959    100969    100979    100989
  100942: TTGCAGTTTTTCTCTG--GTTCCGTCGGCTAGCACGCAGATGGTAATAGA  Hsapiens
  420620: ................--..................T.............  Ptroglodytes
  474160: ...........T....TG....T...........A...............  Pabelli
  420346: ......C..C......TG......G.....T...T...............  Panubis
  441077: ..A.............TA....T...A...................C...  Cjacchus
 1080527: .C........C.....TA....T...........A...........C...  Sboliviensis
```

```
            100999    101009    101019    101029    101039
  100990: TGTGGTGGTCTGATGGGTAGCACAGGGGGCTGAGCAGGAATTCCCGTAAC  Hsapiens
  420668: ........C...................A................A....  Ptroglodytes
  474210: .....CA.C.................................T..A....  Pabelli
  367922:                                          ....A.-..  Nleucogenys
  420396: ........C.................................T.......  Panubis
  441127: .C......C....C..............C...............TA.T..  Cjacchus
 1080577: .C......C....................................A.TC.  Sboliviensis
```

```
            101048    101058    101068    101078    101088
  101040: TGT-GAGACCACTGACTTAAACAGATCTTTTGAGTAAAGTTTTCTTGTCC  Hsapiens
  420718: ...-.........-.....T..............................  Ptroglodytes
  474260: ...-.....A.......C........T.......................  Pabelli
  367930: ...G..............C............A...........T......  Nleucogenys
  173465:                            ..C..T..G.G.......CT..T  Mmulatta
  420446: ...-..AC......................C...................  Panubis
  441177: ...-...G..........................C......C........  Cjacchus
 1080627: ...-.........A...........................C........  Sboliviensis
```

```
                                           B3:6
                                   <-----------------
                      101092    101102    101112    101122
  101089: C----------------GCTTCATGTCTCTTCCAGGTTCTTCACTTTGAT  Hsapiens
  420766: .----------------.................................  Ptroglodytes
  474309: .----------------......C..G.......................  Pabelli
  367980: T----------------.T.C.............................  Nleucogenys
  173488: .TGAGCACCACAGTAATAT..TC.A............AT......C...A  Mmulatta
  420495: .----------------.............................C...  Panubis
  441226: T----------------.....C...........................  Cjacchus
 1080676: T----------------.....C...........................  Sboliviensis
```

```
                            B3:6
          <-----------------------------------------
            101132    101142    101152    101162    101172
  101123: CAAGTCACAGAGAACACCACAGGAAAAGCTGCAACATATCATGTGAGTCA  Hsapiens
  420800: ......................A..................C........  Ptroglodytes
  474343: ................T.....A..................C........  Pabelli
  368014: ..C...................A...G........G.....C........  Nleucogenys
  173538: A...G...GA...G.T.A.A.AC...G....                     Mmulatta
  420529: ......C.........                                    Panubis
  441260: .............------...AG........C.....C..G.....C..  Cjacchus
 1080710: ...A..................A.........C........G.....C..  Sboliviensis
```

```
            101182    101192    101202    101212    101222
  101173: CAGAGCACTATGGTTCAGCTTTAGATCCCCGAACAGGTCATAGTTTGAAC  Hsapiens
  420850: .........T...................T................A...  Ptroglodytes
  474393: .............................T................A...  Pabelli
  368064: ..........................G..T..G......G......A...  Nleucogenys
  441304: .....T...T...........C....A..T..G.............A...  Cjacchus
 1080760: .....TC..T...........C....A..T..G.............A...  Sboliviensis
```

```
                101232    101242    101252    101261    101271
   101223: CTGGAACTTCACAGAAACTAAGAAAAGGCCA-TTTTTAGGGAAAATCTTG  Hsapiens
   420900: ...............................-........T.........  Ptroglodytes
   474443: ........C......................-C.................  Pabelli
   368114: .C.A....................G......-..................  Nleucogenys
   441354: ..........................ACTT.G.........G.....C..  Cjacchus
  1080810: .........................T.....-......A..G.....C..  Sboliviensis

                101281    101291    101301    101311    101321
   101272: GACACAAAGATTGAGACATACAGAGTGGGTTGGCATTTCATGGCACATAA  Hsapiens
   420949: ..................................................  Ptroglodytes
   474492: ....A.............................................  Pabelli
   368163: ....A.........................................G...  Nleucogenys
   441404: ....A......................AT......A..............  Cjacchus
  1080859: ....A..............G.......AT......A..............  Sboliviensis

                                                 MIR
                                        ------------------->
                101331    101341    101351    101361    101371
   101322: TTATTATTCCTCCTTTCTTCGTTACTGAAAGACAGTCAGCACTGTACCTC  Hsapiens
   420999: ............A.....G.A.....A....................TC.  Ptroglodytes
   474542: ..................G.A.....................C.......  Pabelli
   368213: ..................G.............T.................  Nleucogenys
   441454: .....G....AAA.C.T.G.............----...........T..  Cjacchus
  1080909: ..........A.G.....G.A...........----...........T..  Sboliviensis

                                  MIR
           ------------------------------------------------->
                101381    101391    101401    101411    101421
   101372: AGAGCATAGGTCTGGATCAGGATAGGCTAGGTTCAGACTCCAGCTTTGCT  Hsapiens
   421049: ..............C.............G.....................  Ptroglodytes
   474592: ...................................A..............  Pabelli
   368263: ............................G......A..............  Nleucogenys
   441500: .........A.T....C...T.......G......A.T............  Cjacchus
  1080955: ...........T....C...T.......G..C.G.A.T....A.......  Sboliviensis

                                  MIR
           ------------------------------------------------->
                101431    101441    101448    101458    101468
   101422: CTTCACAAATGGTGAACATGAGC---AGGACACAACTGCTCAGAGTCCCA  Hsapiens
   421099: ...........A....T.A....AGG...............G........  Ptroglodytes
   474642: ..................C....---........................  Pabelli
   368313: ...........A......C.-..---...............G..T.....  Nleucogenys
   441550: ......G...........CAG..---........G-....TG....T...  Cjacchus
  1081005: ...........A......CAG.G---.........-....TG........  Sboliviensis

                                  MIR
           ------------------------------------------------->
                101478    101488    101498    101508    101518
   101469: GTGACCTCATCCCAGAAAACTAAGGGTAATAAGAAATCTGACTCAATACA  Hsapiens
   421149: .............................G..A......T..........  Ptroglodytes
   474689: ................................A.................  Pabelli
   368359: ..A.............................A.................  Nleucogenys
   441596: .....A..............A...........A.................  Cjacchus
  1081051: .....A.........G....A...........A.................  Sboliviensis
```

```
                                    MIR
         ------------------------------------------------->
             101528    101538    101547    101557    101567
  101519: TGCAAATACATGCAAATGTTTACAAC-ATTGCCTTGCCCATAAAATGTCA  Hsapiens
  421199: ..........................-.G.....................  Ptroglodytes
  474739: ...........A....CAA.......-..................A....  Pabelli
  368409: ................CA........-.............C....A....  Nleucogenys
  441646: ............G...CA.....TG.A.A.............G..A....  Cjacchus
 1081101: ............G...CA........A.A................A....  Sboliviensis

                      MIR
         ------------------------->
             101577    101585    101595    101605    101615
  101568: TAATAAATGTTATTA--TTATTATAAAATAGCTATAATTATACTAATCAC  Hsapiens
  421248: ...............--..........G.....................T  Ptroglodytes
  474788: ...........G...--....................C............  Pabelli
  368458: ...........G..G--G................................  Nleucogenys
  173696:      ...A.....TCT.......T..CC.ATA.....C.CGG..GA..-  Mmulatta
  187636:      ...A.....TCT.......T..CC.ATA.....C.CGG..GA..-  Panubis
  441696: ....G..---.....--.....G............C.-............  Cjacchus
 1081151: .......---.....--................G...-............  Sboliviensis

             101625    101633    101642    101652    101662
  101616: AATAATGTTGC--AAAATATTTA-ATTTTCATTGAGTCATTAATGAGATA  Hsapiens
  421296: ........GA---...TA.....-.....A.........C.........T  Ptroglodytes
  474836: ...........--..........-.....A..........A........T  Pabelli
  368506: ...........--....A.....-.....A...............C...T  Nleucogenys
  173740: ..........T--..GGAT.A..C.G.AGT.A.A.CC.C...G.ATTGAG  Mmulatta
  187680: ..........T--..GGAT.A..C.G.AGT.A.A.CC.C...G.ATTGAG  Panubis
  441740: ...........AA...TA.....-.....A.........C.........T  Cjacchus
 1081195: ..........----..TA.....-.....A.........C.........T  Sboliviensis

             101672    101682    101692    101702    101712
  101663: AAGAGGAATAAGCACAAGTCCAACTATATTTTGGAAAATGATTGCTATGG  Hsapiens
  421342: C..................................C..............  Ptroglodytes
  474883: ...................G...G....................G.....  Pabelli
  368553: .......................G..........................  Nleucogenys
  173788: .....ACC.T..T-------------.G.AA....C..C-.C.......A  Mmulatta
  187728: .....ACC.T..G-------------.G.AA....C..C-.CC......A  Panubis
  441789: ......................G-......C...................  Cjacchus
 1081240: ......................G-.............C............  Sboliviensis

             101722    101732    101742    101752    101761
  101713: AATATATTGGTTTAGAGCCTTAATAGTGCAAAATGCTTTGCTGG-AAAGT  Hsapiens
  421392: ............................................-..G..  Ptroglodytes
  474933: ....C.........C............A............T...-.....  Pabelli
  368603: ......................C.....................-.....  Nleucogenys
  173824: T.G.-.C....--.AG..--......ACTG...A.T.CCCA.A.C.G...  Mmulatta
  187764: T.G.-.C....--.AG..--......ACTG...A.T.CCCA.A.C.G...  Panubis
  441838: ....................................C.......-.....  Cjacchus
 1081289: ....C....................................A..-.....  Sboliviensis
```

```
              101770    101780    101790    101800    101810
                   :         :         :         :         :
  101762: AGA-AGTTTCTAGATTTAAACAGGCTTAGGTTCAAAACGTGGCACTTCTA  Hsapiens
  421441: ...-.AG...............................T...........  Ptroglodytes
  474982: ...--............C..............T.....T...........  Pabelli
  368652: ...-..G...............................T...........  Nleucogenys
  173869: G..TG.G..T.G..G........A..G........GT.C....         Mmulatta
  187809: G..TG.G..T.G..G........A..G........GT.C....         Panubis
  441887: ...-..A...............................T...........  Cjacchus
 1081338: ...-..G-...............A..............T...........  Sboliviensis

              101820    101830    101831              101845
                   :         :         :                   :
  101811: ATTTATGTCTCTATGAAGAG---------GTTTACTTTC------CATTC  Hsapiens
  421490: ..............A..C..---------.G..TT....CC----.....  Ptroglodytes
  475030: .........C....A.....---------..........------A....  Pabelli
  368701: ..............A.....---------..........------.....  Nleucogenys
  441936: ..G...........A.....GTTTGGTGT....TT...T------A....  Cjacchus
 1081386: ..G...........A.....---------.GG.TT...TTAAAAAA....  Sboliviensis

              101855    101865    101875    101885    101894
                   :         :         :         :         :
  101846: TCTGAGCTTTCTTGTTTTCACCTGAATTGAACTAAAGACTTAA-AGTTAC  Hsapiens
  421527: ...............G....T.....................G-......  Ptroglodytes
  475065: .......C..A...............................G-......  Pabelli
  368736: ..........................................G-......  Nleucogenys
  441980: ..........T.........TT......T..............G.A..G.  Cjacchus
 1081427: ....................TT.....................G......  Sboliviensis

                          MIR
            ------------------------------------>
              101904    101914    101924    101934    101944
                   :         :         :         :         :
  101895: CCATGTAAAGTCCTTAGCCATGTACCTGGCACACACTCTTCTTACGTGCA  Hsapiens
  421576: ..................................................  Ptroglodytes
  475114: .....................................T..........A.  Pabelli
  368785: ..................................G..G............  Nleucogenys
  442030: ...CA....AC.........A..............G.G..A...T.....  Cjacchus
 1081477: .........AC.........A................G..A...T.....  Sboliviensis

              101954    101964    101974    101984    101994
                   :         :         :         :         :
  101945: GAGAATGACCATCATGAGGAAACAGTCACAGATCAGTCAATGTGTCCTAC  Hsapiens
  421626: ......................G..C....................T...  Ptroglodytes
  475164: ......................G..C..............C.....T...  Pabelli
  368835: ......................G..C..T.................T...  Nleucogenys
  442080: ......................GC.C..T...----..........T...  Cjacchus
 1081527: ......................GCAC..T.................T...  Sboliviensis

              102004    102014    102024    102034    102044
                   :         :         :         :         :
  101995: AAGATAACAGCACCAACAGGTATAACAGGGCTTCCTGGCATAATCTATTT  Hsapiens
  421676: .....G.T.C........................................  Ptroglodytes
  475214: .......T.C........................................  Pabelli
  368885: .....G.T.C........................................  Nleucogenys
  442126: ...G.G.T.C......G.............A.-------...........  Cjacchus
 1081577: ...G.G.T.C......G....G........G.........G........  Sboliviensis
```

```
             102054    102064    102074    102084    102094
 102045: AAAATATCCAACCTTCAACATACTCATATCCTTGATGACTGTTAGAAGTG Hsapiens
 421726: .........................G...T....C............... Ptroglodytes
 475264: .....................................G............ Pabelli
 368935: .....................................G............ Nleucogenys
 442169: ...........A..............C..........GT........... Cjacchus
1081627: ...........A..............C..........GT.........C. Sboliviensis
```

LINE2

```
             102104    102114    102124    102134    102144
 102095: AAATATGGTCCTTGCCTTTAAGGAGCTGAGAGTTTAACTGGGAAGCTAAA Hsapiens
 421776: ................CA................................ Ptroglodytes
 475314: ................C................................. Pabelli
 368985: ................C..C.............................. Nleucogenys
 442219: ...............TC....A.......A.................... Cjacchus
1081677: ...............TC............A.................... Sboliviensis
```

LINE2

```
             102154    102164    102174    102184    102194
 102145: CCTAACCCTTTAAACCAACAAGGACAAAATCCAGTGGTAGACAGTGCTGC Hsapiens
 421826: ........................G......T............C..... Ptroglodytes
 475364: ..............T.........A......................... Pabelli
 369035: ...............A........A......................... Nleucogenys
 442269: .T.....A......T........GG...............G......... Cjacchus
1081727: .......A.........C.....GG......................... Sboliviensis
```

LINE2

```
             102204    102214    102224    102234    102244
 102195: ATCTATAGTTCAGAAGAGAAAAGATTGCAGTACATTAGAGCAAGAAGAAT Hsapiens
 421876: ....T...........................TG................ Ptroglodytes
 475414: ......................C........................... Pabelli
 369085: .....C...........................G................ Nleucogenys
 442319: .G..T......................G.....T........G....... Cjacchus
1081777: .G..T......................G..............G....... Sboliviensis
```

LINE2

```
             102254    102264    102274    102284    102294
 102245: TTCCTGGAAGAAGTGAAACATAAGGTGGATTTTGAAGGGTATTTGAGGTG Hsapiens
 421926: ..T...........C...T............................... Ptroglodytes
 475464: ..T...........C................................... Pabelli
 369135: ..T...........C.......................T........... Nleucogenys
 176059:                                                ..A Mmulatta
 189959:                                                ..A Panubis
 442369: ..T...........C........T...T............G........A Cjacchus
1081827: ..T...........C........T...T.........A..G......... Sboliviensis
```

```
                102304    102314    102318    102328    102338
  102295: AAATACACCAAGTATCAGGG------AATAAGCTCAAAGGTCCTCATTGA  Hsapiens
  421976: ....................------.......A................  Ptroglodytes
  475514: ....................------.......A................  Pabelli
  369185: ....................------.......A................  Nleucogenys
  176062: ..GGC....TG.....CA..GGCCAC.TC..A.A..G..A..AC.C....  Mmulatta
  189962: ..GGC....TG.....CA..GGCCAC.TC..A.A..G..A..AC.C....  Panubis
  442419: .....T.............A------.......A........A.......  Cjacchus
 1081877: ....................------.......A........A.......  Sboliviensis

                102348    102358    102367    102377
  102339: GACTACCAGCCATTAGGGACTGAT-CTAACAGACTTAGCAT-----GGG-  Hsapiens
  422020: ........................-................-----...-  Ptroglodytes
  475558: ...........T............-....TG..........-----...-  Pabelli
  369229: ...........T............-................-----...-  Nleucogenys
  176112: .G..GA..AA.-.GG....GA..C-.C.G...C.GC.T.C.CTTAG...A  Mmulatta
  190012: .G..GA..AA.-.GG....GA..C-.C.G...C.GC.T.C.CTTAG...A  Panubis
  442463: ......T....T.......T....-......C.........-----...-  Cjacchus
 1081921: ......T....T.A..T..T....C......C.........-----..T-  Sboliviensis

                102388    102398    102408    102418    102428
  102382: ---TTTAGTATTTACATTGATACAGCAATTGAATGATCTCCTTTTTTGAT  Hsapiens
  422063: ---..............................................C  Ptroglodytes
  475601: ---.........C.....................................  Pabelli
  369272: ---...T...........................................  Nleucogenys
  176160: TAA.C...GC..GCTT...CC.AGAT..CATGT...GA.TT.G....A..  Mmulatta
  190060: GAA.C...GC..GCTT...CC.AGAT..CCTGT...GA.TT.G....A..  Panubis
  442506: ---......................T....C.....G........C...C  Cjacchus
 1081965: ---......G.............G.T....C.....G........C...C  Sboliviensis

                              B3:5
                102438    102448    102458    102468    102478
  102429: GTTTGAAGGTTGATAGGTCAGGAAATGTTCATCACCAGTTTCAAAAGCTT  Hsapiens
  422110: ..................................................  Ptroglodytes
  158656:    ...GA.CG..CT......----------CTG.T.C...GGGC.....  Ggorilla
  475648: ..................................................  Pabelli
  369319: ............C........................A............  Nleucogenys
  176210: -...C...A....G.A.A...A.GCAA.A.....A..A..C......T..  Mmulatta
  190110: -...C...A....G.A.A...A.GCAA.A.....A..A..C......T..  Panubis
  442553: A..............A...............C..G...............  Cjacchus
 1082012: A..............A..................G...............  Sboliviensis

                            B3:5
                102488    102498    102508    102518    102528
  102479: CTGACTGAATTCAACAAATCCACTGATGCATATGAGCTGAAGATCGCCAA  Hsapiens
  422160: ..................................................  Ptroglodytes
  158693: ..CT.CA....AG...GGAT..AGAC..AT..CACA....GT..T.....  Ggorilla
  475698: ...........A.G...............................A....  Pabelli
  369369: ...........A......................................  Nleucogenys
  176259: T........A.A.G....CT....A...AT.....A.....C..A..T..  Mmulatta
  190159: T........A.A.G....CT....A...AT.....A.....C..A.....  Panubis
  442603: ...........A......................................  Cjacchus
 1082062: ...........A.......................A..............  Sboliviensis
```

```
                                  B3:5
          <-----------------------------------------
               102538    102548    102558    102568    102577
  102529: CAAGCTCTTCGGAGAAAAAACGTATCTATTTTTACAGGTAA-TTTCACCT Hsapiens
  422210: ..................G.......A..............-........ Ptroglodytes
  158743: ..G...T.AT.....GC.GGAA.TC.C.A.C.GC.....G.-G..      Ggorilla
  475748: ..........................A....C.........A........ Pabelli
  369419: .........T........G..A....A..............A........ Nleucogenys
  176309: ..G...G..T...........A..C..C..CC.T..A....-G..TG.TG Mmulatta
  190209: ..G...G..T...........A..C..C..CC.T..A....-G..TG.TG Panubis
  442653: ..................G.T.....A.......A......A.....A.. Cjacchus
 1082112: ..................G.......A.......A......A........ Sboliviensis

               102587    102597    102607    102617    102627
  102578: GGCCTACCCACATTTCATTTGCATCCTGATGTCTGTGTCTCTGAGTGGCC Hsapiens
  422259: ..................C....G.......................... Ptroglodytes
  475798: ...........C......C....G..................C....... Pabelli
  369469: ...........C......C....G.......................... Nleucogenys
  176358: T..T....                                           Mmulatta
  190258: T..T....                                           Panubis
  442703: .......GA.TC......C....G..C.G....C..........C..... Cjacchus
 1082162: .......AA..C......C....G....G........A......C..... Sboliviensis

               102637    102646    102656    102666    102676
  102628: AAATGGAAGAAAGC-AAGGCAGATGAGCCTGACCGATCCAGGTGGAGAGC Hsapiens
  422309: ..............-................G..A.C............. Ptroglodytes
  475848: ..............-........C.......G...GC............. Pabelli
  369519: .....A......AT-................G....C....A........ Nleucogenys
  442753: .TG.......G...T......C.....T.....T..CG.......G.... Cjacchus
 1082212: .C........G...T......C.....T.....T..CG............ Sboliviensis

               102686    102696    102706    102716    102726
  102677: ATTTACTCAGAGTGCAATAGCTCCGTTTCCACAACTCTCCCCCACTGGAG Hsapiens
  422358: ................T................................. Ptroglodytes
  475897: ................T.......A......................... Pabelli
  369568: ........G.......T.....................T........... Nleucogenys
  442803: ................T.......A.........G....T.......... Cjacchus
 1082262: ................T.......A.........G............... Sboliviensis

               102736    102746    102756    102766    102776
  102727: TGTCCCAGACCCTAACAATACATCACTGAAGTGTGGATTTAGGGATAATC Hsapiens
  422408: ............C..................................... Ptroglodytes
  475947: ............C.....C............................... Pabelli
  369618: ............C...G........G..................G..... Nleucogenys
  442853: ............CG.....CT....GG....------------------- Cjacchus
 1082312: ............CG......T....GG......................T Sboliviensis

                                                  Simple
                                         ------------------------->
               102786              102803    102813    102823
  102777: TTGTAATAAAAGAGGAGG---TTGGGTAATAGAGTGAGTAAGAGTAATAA Hsapiens
  422458: ..................---..................G.......... Ptroglodytes
  475997: ..................---...........G................. Pabelli
  369668: ..................---............................G Nleucogenys
  442884: -..C...T..........AGG......----------------------- Cjacchus
 1082362: ...C...T..........AGA......---------------..C.GC.. Sboliviensis
```

```
           Simple
           ------->
                  102833    102843    102853    102863    102873
                       :         :         :         :         :
  102824: GTAATAAGATACCATCGATAAACTGGCACTGACTCAGTCACATACGATAC  Hsapiens
  422505: ............................................GC....  Ptroglodytes
  476044: ............................................AC...T  Pabelli
  369715: ............T...............................GC....  Nleucogenys
  442910: --.....A.C.....G...G........................GT....  Cjacchus
 1082397: .......A.C......A..G.G......................GT....  Sboliviensis

                  102883    102889    102899    102909    102919
                       :         :         :         :         :
  102874: ATCTTGGTGGGAA----ATGTATGACTAATGGGATATTATTGGAATGGGC  Hsapiens
  422555: .............----.................................  Ptroglodytes
  476094: .............----.................................  Pabelli
  369765: .............----.................................  Nleucogenys
  442958: .............----.......G..............C..........  Cjacchus
 1082447: .............ATGT.......G.........................  Sboliviensis

                  102929    102939    102949    102959    102969
                       :         :         :         :         :
  102920: AGGCTTGGGTGAGTTCCTGAGAATAGTTGAGGAAGTACCAGGAAATACTG  Hsapiens
  422601: ...................G............................G.  Ptroglodytes
  476140: ...................G..............................  Pabelli
  369811: ..................................................  Nleucogenys
  443004: ............A......G.G.......----------...........  Cjacchus
 1082497: ............A......G.G............................  Sboliviensis

                  102979    102989    102999    103009    103019
                       :         :         :         :         :
  102970: AATGCACAGGATGAAAGATAAAAACAAAGATCAGAAACATCATGGTTGAA  Hsapiens
  422651: ..................................................  Ptroglodytes
  476190: ................T.................................  Pabelli
  369861: ..C...............C..........G....................  Nleucogenys
  443044: ..A.T....C........G......T...G........G...........  Cjacchus
 1082547: .........C..C............T...G....G...............  Sboliviensis

                  103029    103039    103049    103059    103069
                       :         :         :         :         :
  103020: ATTACTGGAAAGAGGTCTGAGAAGCAATGAATCTCCTTCAGGGAAGCCTG  Hsapiens
  422701: .........................................A........  Ptroglodytes
  476240: .........GC.......................................  Pabelli
  369911: .........G...A....................................  Nleucogenys
  443094: ........TG........C....................G..A..A...T  Cjacchus
 1082597: ........TG...A....C.....................C....A...C  Sboliviensis

                  103079    103089    103099    103109    103119
                       :         :         :         :         :
  103070: CTCTGCAGTTTGCAAACCACAGCCTCTTCTGCTTCTGCCTTTTGCCAGAT  Hsapiens
  422751: .............................................A....  Ptroglodytes
  476290: .................T................................  Pabelli
  369961: .......................G..........................  Nleucogenys
  443144: ...............................................---  Cjacchus
 1082647: ...C............................................G.  Sboliviensis
```

```
                103127      103137      103146                  103163
                     :           :           :                       :
  103120: GATATT--GACCTTCGGTGA-CCTCTTTCTTGCGCCAGC---CCGCACTC  Hsapiens
  422801: ......--.......A....-..................---..A.....  Ptroglodytes
  476340: ......--...T...A....-..................---..A.....  Pabelli
  370011: ......--.......A....-...........G......---..A.....  Nleucogenys
  190382: ......CA..A..G.A.G..G.T.....A.A.TC.TG..AAT.T.AG.CT  Panubis
  443191: ......--.G.....A....-..C........T....A.---..A.....  Cjacchus
 1082697: .G....--.G.....A....-..C...........TG..---..A.....  Sboliviensis

                103173      103183      103193      103203      103213
                     :           :           :           :           :
  103164: CGCTTTTGCATTGCCTACATGGCACCAGTATAAAAATATGCATGGACAGG  Hsapiens
  422845: .C................G.......G.......................  Ptroglodytes
  476384: .C...................T....T.......................  Pabelli
  370055: .C........................TA......................  Nleucogenys
  176535:    ......C.CAT.CC..GATA.G.T.C........G......C.----  Mmulatta
  190432: GAT......C.CAT.CC..GATA.G.T.C........G......C.----  Panubis
  443235: TTA.....T...ATT.G.........T.......................  Cjacchus
 1082741: TTA.....T...ATT.G.........T.......................  Sboliviensis

                103223      103228      103238      103246      103256
                     :           :           :           :           :
  103214: AGATACTGCAT-----CTATTCAGGGTCTGG--ATTCAGCTTACTATAAT  Hsapiens
  422895: ...........-----...............--.................  Ptroglodytes
  476434: ...........-----..G............--........C........  Pabelli
  370105: ...........-----..G............--........G.....TG.  Nleucogenys
  176578: -.G....CA..GTGAGA.GCC....AC..TACA...TCT......G..T.  Mmulatta
  190478: -.G....CA..GTGAGA.GCC....AC..TACA...TCT......G..T.  Panubis
  443285: ...........-----..C............--......G...T......  Cjacchus
 1082791: ...........-----..C............--..........T......  Sboliviensis

                            103263      103273                  103291
                                 :           :                       :
  103257: -------------TATAAATAACTAAATTTGGTAATAT--ATAGTTACAT  Hsapiens
  422938: -------------.........................--..........  Ptroglodytes
  476477: -------------.G.......................--G.........  Pabelli
  370148: -------------..C......G...G...........--..........  Nleucogenys
  176627: AGTCATCCCAATG....C....A.TT....A.AG..G.TGGC.A..G-..  Mmulatta
  190527: ACTCATCCCAATG....C....A.TT....A.AGG.G.TGGC.A..G-..  Panubis
  443328: -------------.......C.................--..........  Cjacchus
 1082834: -------------......--------.......G...--..........  Sboliviensis

                103300      103310      103320      103325
                     :           :           :           :
  103292: AAATT-ACTCTTACTTCCTACTTTCTTCCTTCAT-----ATCTCAA----  Hsapiens
  422973: .....-............................-----.......----  Ptroglodytes
  160129:                      ..AT....C.TT.-----..TC...TGGA  Ggorilla
  476512: .....-............................-----.......----  Pabelli
  370183: .....-............................-----.......----  Nleucogenys
  176676: .....G...T..TAAC.TCTT...A.......C.TGTTTC..CT..----  Mmulatta
  190576: .....G...T..TAAC.TCTT...A.......C.TGTTTC..CT..----  Panubis
  443363: .....-.....................T....T.-----...C...----  Cjacchus
 1082861: .....-..........................T.-----...C...----  Sboliviensis
```

```
                              B3:4
             103339    103349    103359    103369    103379
  103332: --AGGAATATTTAGATGCCATCAAGAAATTTTACCAGACCAGTGTGGAAT Hsapiens
  423013: --................................................ Ptroglodytes
  160153: AC.......C.......GTG.G.TTC..........C..G.CGA.T...A Ggorilla
  476552: --................................................ Pabelli
  370223: --.............C.................................. Nleucogenys
  176722: --..A....C......TATG.TG.A....A...T..TG.ATC.C.....C Mmulatta
  190622: --..A....C......TATG.TG.A....A...T..TG.ATC.C.....C Panubis
  443403: --...........................A.................... Cjacchus
 1082901: --...................G.......A.................... Sboliviensis

                              B3:4
             103389    103399    103409    103419    103429
  103380: CTGTTGATTTTGCAAATGCTCCAGAAGAAAGTCGAAAGAAGATTAACTCC Hsapiens
  423061: .................................................. Ptroglodytes
  160203: G.........CCA...AAAC..T...A..TCCA..C.AG.........T. Ggorilla
  476600: ..............C................................... Pabelli
  370271: ..............C................C.................. Nleucogenys
  176770: ............T...C..AG....T....................T... Mmulatta
  190670: ............T...C..AG....T....................T... Panubis
  443451: ............T.G.A.A...........................T... Cjacchus
 1082949: ................A................................. Sboliviensis

                   B3:4
             103439    103449              103462
  103430: TGGGTGGAAAGTCAAACGAATGGTA-------GGAGAGCCACCCATTAT- Hsapiens
  423111: .........................-------.................- Ptroglodytes
  160253: .....T...T.....T.CC.A....-------A..A.... Ggorilla
  476650: .........................-------.................- Pabelli
  370321: .........................-------.................- Nleucogenys
  176820: .....T.....CA....A.......GAGTATG..T.G.T..TT....GCA Mmulatta
  190720: .....T.....CA....A.......GAGTATG..T.G.T..TT....GC- Panubis
  443501: .........................-------.........T..G....- Cjacchus
 1082999: .........................-------.........T.......- Sboliviensis

                                       103479    103489
  103472: --------------------------------AGAAACACCTTTGAGAAA Hsapiens
  423153: --------------------------------.................. Ptroglodytes
  476692: --------------------------------.................. Pabelli
  370363: --------------------------------.................. Nleucogenys
  176870: AAAAAAACCAGAAACAAACAAACAGACAGACA.A......T.C.TGAC.. Mmulatta
  190769: AAAAAAACCAGAAACAAACAAACAAACAGACA.A......T.C.TGAC.. Panubis
  443543: --------------------------------.................. Cjacchus
 1083041: --------------------------------.................. Sboliviensis

             103498    103507    103517    103527
  103490: TCTA-TGCGAGTG-AGCCCTATGCCTGAAACTGCATGGGGGACCAT---- Hsapiens
  423171: ....-........-................................---- Ptroglodytes
  476710: C.C.-........-......G.......C...........-....G---- Pabelli
  370381: C...-........-......G.A.....C...........-....G---- Nleucogenys
  176920: ...GC..T.....A......GGA.T..TC.....G..A...G....ATAA Mmulatta
  190819: ...GC..T.....A......GGA.T..TC.....G..A..TG....ATAA Panubis
  443561: C...-...T....-......G.C.....C........A.......G---- Cjacchus
 1083059: C...-........-......G.C.....TG.......A.......G---- Sboliviensis
```

```
                       103540              103559
 103534: -------------GGGTGGGAATTGAGAT-GGCTTTGCAGGGAGGGAAG-  Hsapiens
 423215: -------------................-..........A.....C..-  Ptroglodytes
 476753: -------------.......G........-..G....G........CT.-  Pabelli
 370424: -------------....A...........-................CT.-  Nleucogenys
 176970: TGCACAAGCTTTG..AA....C......CA..T.........TT.....G  Mmulatta
 190869: TGCACAAGCTTTG..AA....CG.....CA..T.........TT.....G  Panubis
 443605: -------------..A.A..G.C......-..G...........A.C..-  Cjacchus
 157567:                                     .............-  Sboliviensis
1083103: -------------..A.A..G........-..G...                Sboliviensis

                                 103576    103586    103596
 103569: ----------------------AAGAGGGCACTCCAGATGAAGAGTTTTT  Hsapiens
 423250: ----------------------.....................T......  Ptroglodytes
 476788: ----------------------.....................GA...G.  Pabelli
 370459: ----------------------.....................GA...G.  Nleucogenys
 177020: GCAAGCCTGGGCCTAGGAGGTAGG.........G......GT.GAGCAA.  Mmulatta
 190919: GCAAGCCTGGGCCTAGGAGGTAGG.........G......GT.GAACAA.  Panubis
 443640: ----------------------.G.......A.G...G.....GAC..G.  Cjacchus
 157580: ----------------------G.....                        Sboliviensis
1083144:                             ...A.G..AG.....GAC..G.  Sboliviensis

             103606    103616    103626    103636    103646
 103597: CCAAATGATTATGAAGAGAGCCTAGGGGAGCCAAGGAGGAAATCACAGGA  Hsapiens
 423278: ..................................................  Ptroglodytes
 476816: ........A.........................................  Pabelli
 370487: ........G.....C....................A..............  Nleucogenys
 177070: TTCTGCA.A.G..G....-...A.........-..C...GG.CTG..T..  Mmulatta
 190969: TTC.GCA.A.G..G....-...A.........-..CG..GG.CTG..T..  Panubis
 443668: .T.CG...A.......C..........A.AT...A...............  Cjacchus
1083166: .T.C....A........C.........A.A....A...............  Sboliviensis

             103656              103675    103685    103695
 103647: AGCCAATTAGAAGAAAACA-TAGCCGGAGGATTCTTTGCTCATGTTCCTG  Hsapiens
 423328: ...........T.......-..............................  Ptroglodytes
 476866: ...........T.G.....-C.T.T....A...A.....CT...GC....  Pabelli
 370537: ...........T.G.....-C.T.T....A...A......T...GC....  Nleucogenys
 177118: ......A.GC.T.G....GC.T..T....A.C.G...A.A...AGC.A..  Mmulatta
 191017: ......A.GC.T.G....GC.T..T....A.C.G...A.A...AGC.A..  Panubis
 443718: .....G.....T.G.....-C...T....A...A.....A.....C....  Cjacchus
1083216: ...........T.G.....-C...T....A...A.....A.....C....  Sboliviensis

             103705    103715    103724              103736
 103696: GATGACAATAGCTTTGTGGATC-CCTTGTCTCCACT--------CAGGTC  Hsapiens
 423377: ......................-..C.......G..--------....C.  Ptroglodytes
 476915: C.....................-..C......TG..--------....C.  Pabelli
 370586: C....................G-..C.......G..--------....C.  Nleucogenys
 177168: ...C......T.-...G..G..-..GAAAA.GGGA.GATGCCTGT.T.C.  Mmulatta
 191067: ...C......T.-...G..G..-..GAAAA.GGGA.--------G.T.C.  Panubis
 443767: .....T..........C.....-..C......TG..--------....C.  Cjacchus
1083265: .....T................A..C......TG..--------....C.  Sboliviensis
```

```
             103746    103756    103766    103776    103786
 103737: CAATTTGAGATCATGTCCTTTACTTTAAATCAGAACCAATATTTTATGAT  Hsapiens
 423418: ..T...............................................  Ptroglodytes
 476956: T.T...........................TG..G.....T.........  Pabelli
 370627: T.T...........A................G..G.....T.........  Nleucogenys
 177216: TGTC...G.T.T.CA.T....C.....T......G..----...CT.CC.  Mmulatta
 191107: TGTC...G.T.T.CA.T....C.....T...G..G..----...CT.CC.  Panubis
 443808: ..T...........A.T....T............G..-..T.........  Cjacchus
1083307: ..T...........A.T....T............G.....T.........  Sboliviensis

             103796    103806    103816    103826    103836
 103787: GAATATTTAAAAGGAAACATTAGAAAGCATCTCCCTTCCCCTTTACTAAT  Hsapiens
 423468: .............A....................................  Ptroglodytes
 477006: .............A...................T.A..T...........  Pabelli
 370677: ...........G.A..............G....T.G..T...........  Nleucogenys
 177262: ..G.T...--------------------------..CTGT..G.GT...G  Mmulatta
 191153: ..G.T...--------------------------..CTGT..G.GT...G  Panubis
 443857: .............A......G......TG.......C.T.......C.G.  Cjacchus
1083357: .............A.............TG.........T.........G.  Sboliviensis

             103846    103856    103866    103876    103885
 103837: TGGGAAACAAGCAGCTCTCTGGTAAATCACCCTTTTGCCATCTG-AACTA  Hsapiens
 423518: .......................................G....-.....  Ptroglodytes
 477056: ...................C...................-..C.-.G..G  Pabelli
 370727: ...................C...................-....-.G..G  Nleucogenys
 177286: ..A......C.........CCA.C..--GA.T.G..C.....CAC.G..G  Mmulatta
 191177: ..A......C.........CCA.C..--GA.T.G..C.....CAC.G..G  Panubis
 443907: ....G..............CA.......................-.G..G  Cjacchus
1083407: ................T..CA........T..............-.G..G  Sboliviensis

             103895    103905    103915    103925    103935
 103886: GAGCTGCCTGGATCACATCTCTAGCCACAAGTTCTGCAGGGATTATCACA  Hsapiens
 423567: ............C.....................................  Ptroglodytes
 477104: ............C.......G........C....................  Pabelli
 370775: ............C.......G........C....................  Nleucogenys
 177334: .G..CA.....TCTT.....TC.CTT.AGCA..A.........A..G...  Mmulatta
 191225: .G..CA.....TCTT.G...TC.CTT.AGCA..G.........A..G...  Panubis
 443956: .......T....CG........GTT....C....................  Cjacchus
1083456: ............C..TG....--------C..C..A..............  Sboliviensis

                       103944    103954    103963    103973
 103936: G-----------CTCTCTTCCCCATCAAGGGC-AAGAGCTTAACAAATTC  Hsapiens
 423617: .-----------....................-..............G..  Ptroglodytes
 477154: .-----------..T...C.............A........G.....G..  Pabelli
 370825: .-----------....................A........G.....G..  Nleucogenys
 177384: AACGACAGCCCC.C.G.CC.G..CC.C.CA..AG..GAT..G....G..T  Mmulatta
 191275: AA----------.GACAGC....CC.C.CA..AG..GAT..G....G..T  Panubis
 444006: .-----------..G..C.........C.A..A.......G......G..  Cjacchus
1083498: .-----------..G............C....A.......G...G..G..  Sboliviensis
```

```
            103983      103993      104003      104013      104023
                 :           :           :           :           :
  103974: TCCATTCTATAGACATCTATCCTACCTCCCACCTCTCATTACAGGCCAAA   Hsapiens
  423655: .G................................................   Ptroglodytes
  477193: ..................T..T............................   Pabelli
  370864: ..................................................   Nleucogenys
  177434: CTT..----------C.C..T..T...TT.T.A..C.C.AGT.T.AG...   Mmulatta
  191315: CTT..----------C.C..T..T...TT.T.A..C.C.AGTGT.AG...   Panubis
  444045: .AG..A..............T....................A..A...C.   Cjacchus
 1083537: .AG..A..G...........T......                          Sboliviensis
```

```
            104033      104043      104053      104061      104071
                 :           :           :           :           :
  104024: CTTACAGCAACTCAACATGAGAGTGAATAGGAAGAT--ACCCCCGGAAGT   Hsapiens
  423705: ....................................--............   Ptroglodytes
  477243: ..........A................C........--.T..........   Pabelli
  370914: ....................................--............   Nleucogenys
  177474: TCC...----------.GA.AT..T...GT.G.A..TC...TTG..G..C   Mmulatta
  191355: TCC...----------.GA.AT..T...GT.G.A..TC...TTG..G..C   Panubis
  444095: .....T....G...........C.............--.....A......   Cjacchus
```

```
            104081      104091      104101      104111      104121
                 :           :           :           :           :
  104072: AGTGTCTGACAGCACAGGACATGCGTTTCATATTACAGAGCTCATGTCAC   Hsapiens
  423753: .......C.T........................................   Ptroglodytes
  477291: ...........A................................A.....   Pabelli
  370962: ........................A...................A.....   Nleucogenys
  177515: .A.AGT...T.CTTT..TGTTA.A......G...C..T-TT.T..AC..A   Mmulatta
  191396: .A.AG....T.CTTT..TGTTA.A......G...C..                Panubis
  444143: .A................G....T.........C...........AA...   Cjacchus
```

```
            104131                              104151      104161
                 :                                   :           :
  104122: TTGTCCTAAAAAGC------AGTCAGG----GCCTACTTCCTCTGAATGG   Hsapiens
  423803: ..............------.A.....----...................   Ptroglodytes
  477341: .............T------.A.....----.............T.....   Pabelli
  371012: ..........G...------.A.....----.........T.......CA   Nleucogenys
  177564: ..A..AG.G.C.C.TTTGTT.C.T..AATGT.TT.TT....C.CTC..--   Mmulatta
  444193: ..............------.......----.................T.   Cjacchus
```

```
                                      Simple
                              ------------------>
            104171                  104183      104193      104203
                 :                       :           :           :
  104162: GGACCCCGTAGTT--------AAAAAAAAAAAAAAAAAAGTAGGAAGAGG   Hsapiens
  423843: .............AAAAAAAA.............................   Ptroglodytes
  477381: .......A.....--------------.......................   Pabelli
  371052: .............---------------G.....................   Nleucogenys
  177612: ------..T.G.CC-------------TG..G...GG..A..A...A..C   Mmulatta
  444233: ....TA.A.....--------------...........G.C.........   Cjacchus
```

```
            104213      104223      104233      104243
                 :           :           :           :
  104204: AGGGAGGGAGAAAGGAAAGACACATGTTGAAAGAGTAGACAAAATCAG--   Hsapiens
  423893: ..................C.............................--   Ptroglodytes
  477417: .A..............................................--   Pabelli
  371087: .......................................TT.......--   Nleucogenys
  177643: ..AA.----------...T.GGATCT..C...A...T.GTTG..-...TA   Mmulatta
  444269: ..A.................G.T..T.............T........--   Cjacchus
```

```
            104261    104271    104281    104291    104301
104252: TTTATCAGTATTCCAAATCAGATGAATGGACTCATTCATACACAGAGAAC   Hsapiens
423941: ..............................G...................   Ptroglodytes
477465: ........G.....................G...................   Pabelli
371135: .........................T....G.......A...........   Nleucogenys
177682: ...T.T......TATTG.GG....GGCT..---.C...G...---.A..T   Mmulatta
444317: ...G...........TG........C....G...C...G.G.........   Cjacchus

            104311    104321    104331              104348
104302: GTGAACTCCTTCTCTATCACAAGAAGTGATGTCTCCATT---AAGGGCAA   Hsapiens
423991: C.....................................C---........   Ptroglodytes
477515: C.....................................C---.T...T..   Pabelli
371185: C....---.............................CC---.....T..   Nleucogenys
177726: A.C...........C.AT.T..T....A..CC...A..CTCAGGC..T..   Mmulatta
444367: C....................................CC---....TT..   Cjacchus

            104358    104368    104378    104388    104398
104349: CTTTATACGACTGGAGCCTTGAAGAAAGCTGCATCTGGTGAACCACTGGT   Hsapiens
424038: ..G..........................................T....   Ptroglodytes
477562: ......G......................C...............T....   Pabelli
371229: ......CT.....................................T....   Nleucogenys
177776: ..A...                                               Mmulatta
444414: ......C.AG...................................T....   Cjacchus

104399: CAG-----------------------------------------------   Hsapiens
424088: ...-----------------------------------------------   Ptroglodytes
477612: ...-----------------------------------------------   Pabelli
371279: ...GGAATAGAAATTTAGGAAATTAGGGAGTTACAATTTAATAATTGCAA   Nleucogenys
444464: ...-----------------------------------------------   Cjacchus

                                          104408    104418
104402: ---------------------------------TGAGTCTAACAATTCAA   Hsapiens
424091: ---------------------------------.................   Ptroglodytes
477615: ---------------------------------.........T.......   Pabelli
371329: TGTGTACTAAATAATGTATACAGAAAAATAAGA....C...TT.......   Nleucogenys
444467: ---------------------------------.........TGG..T..   Cjacchus

            104428    104436    104446    104456    104466
104419: AGATCAAAGTCAGTGAG--TCTCAAGTAGGGATTTGGGTCAATAATTAAC   Hsapiens
424108: .................--..............................T   Ptroglodytes
477632: .................--....T...............G.........T   Pabelli
371379: T................--....................G.........T   Nleucogenys
178111:         .....CA..GCC....G..GCT..CC...C.-GG.TT....T   Mmulatta
191851:         .....CA..GCC....G..GCT..CC...C.-GG.TT....T   Panubis
444484: ..........T......--...A................G.........T   Cjacchus

            104476    104486    104496    104506    104516
104467: GATCAGTCACGAACATTTGCAAAGCATCTTCCAGACAAGCCATTTGTAGC   Hsapiens
424156: ................................................C.   Ptroglodytes
477680: ................................................C.   Pabelli
371427: ................................................C.   Nleucogenys
178152: .........A.TC...C....TCAT..G.GT..TG..TATT.C....CAT   Mmulatta
191892: .........A.TC...C....TCAT..G.GT..TG..TATT.C....CAT   Panubis
444532: .....A............T.....T.............A.........C.   Cjacchus
```

```
                                                                B3:3
                                                             <--------
             104525              104539    104549    104559
104517: TTGTGT-AAAAGAC------TCTTTTATTCTTTCCCTTGCAGAAAAAATT  Hsapiens
424206: ......-.......------..............................  Ptroglodytes
477730: ......-G......------..............................  Pabelli
371477: ......-.......------..............................  Nleucogenys
178202: ...GAA-G.TG.G.CAACCT.T..C.G..TC...AT..A......C...C  Mmulatta
191942: ...GAA-G.TG.G.CAACCT.T..C.G..TC...AT..A......C...C  Panubis
444582: ......A......T------.......                         Cjacchus
```

```
                                  B3:3
        <--------------------------------------------------
             104569    104579    104589    104599    104609
104560: AAAAACCTAATTCCTGAAGGTAATATTGGCAGCAATACCACATTGGTTCT  Hsapiens
424249: ........C.............C...........................  Ptroglodytes
477773: .................T..GGC........A.G....G...C.......  Pabelli
371520: .................T..G.T.....A..A..T...............  Nleucogenys
178251: ..GG..T.GT.C..A..T..CTC....A.T..TC......AGC....G..  Mmulatta
191991: ..GG..                                              Panubis
420645:       ...C......GT..A.G........A.G........C.......  Panubis
```

```
                                  B3:3
        <--------------------------------------------------
             104619    104629    104639    104649    104659
104610: TGTGAACGCAATCTATTTCAAAGGGCAGTGGGAGAAGAAATTTAATAAAG  Hsapiens
424299: ..................................................  Ptroglodytes
477823: .........C...................................C....  Pabelli
371570: ...............................A....TG.......A....  Nleucogenys
178301: G......A..G.T.....T........A.....C.G.G.G.....G....  Mmulatta
420689: .....................G..T..A...C.A..T.............  Panubis
```

```
                          B3:3
        <-----------------------------------
             104669    104679    104689    104699    104709
104660: AAGATACTAAAGAGGAAAAATTTTGGCCAAACAAGGTATTGTCTATATTT  Hsapiens
424349: ..................................................  Ptroglodytes
477873: ..C....................C..........................  Pabelli
371620: ..A..........................G....................  Nleucogenys
178351: ..A.......G..A..G.........ATG..T.......GC..CT..A..  Mmulatta
420739: ..A...............................................  Panubis
```

```
             104719    104729              104743    104753
104710: TATTTATATAATGTAATATGTTAATAAAT------TTAAACATTTCTGAT  Hsapiens
424399: ..................C..........------...............  Ptroglodytes
477923: .............................------...............  Pabelli
371670: ..........G...............C..GGAATG...............  Nleucogenys
178401: .T...------...G..G..C.T.C.C..                       Mmulatta
420789: ............A................------.........G.....  Panubis
```

```
             104763    104773    104782    104792    104802
104754: GGAATGTACCATGACAAGTAAAAAA-TAAAATTGTTCATGTCTGTTTTTT  Hsapiens
424443: .........................-........................  Ptroglodytes
477967: ..............T..-.......-........................  Pabelli
371720: .........................T..C....T............A...  Nleucogenys
420833: .........------..........-.......TA............A...  Panubis
```

```
         104812    104822    104832    104842    104852
104803: TGTTGTTTTAGTCTTATAACTTTATTTAGTTAGGAATTCCTGAAGAAGCT  Hsapiens
424492: ..........C..........................A............  Ptroglodytes
478015: ..........A..........................A............  Pabelli
371770: .....................................A.........A..  Nleucogenys
420876: ..............................A..A...A.........A..  Panubis
```

```
         104862    104872    104882              104899
104853: ATTGTCTCTAACTCATTACATTCTTGGATTATTTCTT---AAAAGAAGGA  Hsapiens
424542: .....................................---.T........  Ptroglodytes
478065: ..................A..................---........A.  Pabelli
371820: .....T..........GGA....C..AG.........AGA.G........  Nleucogenys
420926: T.................A........G....G....---.G........  Panubis
```

```
         104909    104919    104929    104938    104948
104900: TGTGTTGATATCTCAATAATATTATCTTTTT-GCCTTGTGTCTCACTTGG  Hsapiens
424589: ...............................-..................  Ptroglodytes
478112: ...............................-..................  Pabelli
371870: .......C.G........C............C.TA......T...TAC.T  Nleucogenys
178866:                          .C...G-.T...A....C--T...A  Mmulatta
420973: .....................C.........T.T................  Panubis
```

```
                                             B3:2
                                     <--------------------
         104958    104968    104978    104988    104998
104949: TATTTGTTGGACACATTGATTTATTGTAGAATACATACAAGTCCATACAG  Hsapiens
424638: ..................................................  Ptroglodytes
164509:                      ..C.AC.....GA.A.....AGTG.GA..  Ggorilla
478161: ..................................................  Pabelli
371920: ...........T..............C.............A...G.....  Nleucogenys
178888: ..-....GT.CTCTG..A....G...C...GC...AGT..A..TG.G...  Mmulatta
421023: ...........TG.............C........C.......TG.....  Panubis
155530:                    .C..C..C.....GA.A.G...AATG.GA..  Cjacchus
```

```
                           B3:2
        <--------------------------------------------------
         105008    105017    105027    105037    105047
104999: ATGATGAGGCAATACA-CATCTTTTCATTTTGCCTCGCTGGAGGATGTAC  Hsapiens
424688: ................-....C............................  Ptroglodytes
164538: .......C....A.AGG.C...ACAG.A..G..T..A-.A.....G..GA  Ggorilla
478211: ............C...-.G..C............................  Pabelli
371970: ......G.........-AT..C...A........................  Nleucogenys
178937: .......CA..GAG.C-AT..C...AGC..CA.T.TC........CT.G.  Mmulatta
421073: ............A.TG-AG..C...A.........T..............  Panubis
155561: .......T....A.AGG.C...A.AG.A..G..T..A-.A.....GC.GA  Cjacchus
```

```
                           B3:2
        <--------------------------------------------------
         105057    105067    105075    105085    105095
105048: AGGCCAAGGTCCTGGAAATACCATACA--AAGGCAAAGATCTAAGCATGA  Hsapiens
424737: ......G....................--.....................  Ptroglodytes
164587: ....AC..A..........GAGG....CC....GG...--..C......T  Ggorilla
478260: ...........................--.....................  Pabelli
372019: ...........................--.....................  Nleucogenys
178986: .......AA.T..A.GG..T.....T.--..AA...T..C.........T  Mmulatta
421122: ..........A................--.....................  Panubis
155610: ....AC..A..........GAGG..T.CC....GG...--..C......T  Cjacchus
```

B3:2

```
                   105105                  105113    105123    105133
105096: TTGTGTTGCTGCC------------AAATGAAATCGATGGTCTCCAGAAG  Hsapiens
424785: .....C.......------------.........................  Ptroglodytes
164635: .C...C.......ATCTCACTCTAA.G..A.CC.GA.G.....GG.AG..  Ggorilla
478308: .....C.......------------..................G......  Pabelli
372067: .....C.......------------..........A.......G......  Nleucogenys
179034: .....C.T.....------------C..C..C...........GG.....  Mmulatta
421170: .....C.......------------G.........A.......G......  Panubis
155658: .C...C.......GTCCCACTCCGA.G..A.CT.GA.G.....GG.AG..  Cjacchus

                   105143    105152    105162    105172
105134: GTAAGAACTTGCATCTAC-AACTCTTCCTTCTACTGCCTGACATTT----  Hsapiens
424823: ..................-...........................----  Ptroglodytes
164685: ....ATCT.CATT..C..-.TG...                           Ggorilla
478346: ..................-...........................----  Pabelli
372105: ..........C.......-..............G.......T....----  Nleucogenys
179072: ....ACG...A.....C.TT.T....G...TC.T.T.....GG...TCTG  Mmulatta
421208: ....A.............-......----.............T...----  Panubis
155708: ....TTCT.CATT.....-.T....                           Cjacchus

                   105188    105198    105208    105218    105228
105179: TTCCAAAGATACCAAGTTTAAACAAGGTAAAAGCTGGTGACCGAGTTGCC  Hsapiens
424868: .........................................T........  Ptroglodytes
478391: ...................................TA....T..C.....  Pabelli
372150: ...................................T..............  Nleucogenys
179122: .CT..GG.C.T.TG.-----------...GG........G..-..CA.TG  Mmulatta
421249: ..T..................GT.........A..T......A.A..T..  Panubis

                   105238    105248              105264    105274
105229: TCAAAATAATGAAAAATTCTAAATGAGG--AATGA--TGACTCACCTTCA  Hsapiens
424918: ...............C...........A--.....--.............  Ptroglodytes
478441: .......G...........A........--.....--.............  Pabelli
372200: .G.TTG.GC..----C.G.C......AATC....GTC..T..........  Nleucogenys
179160: .....                                               Mmulatta
421299: ...................A..GG....--.....--.T.........T.  Panubis

                   105284    105294    105304    105314    105324
105275: TATTACAAAGATTTAAGCATAGGGCCTGACACAAACTGAAGGCATAGTTA  Hsapiens
424964: ......................A....................G......  Ptroglodytes
478487: ....G....T..............T..................T......  Pabelli
372246: .........T.................................T......  Nleucogenys
421345: .........T........................G........T..A...  Panubis

                   105334    105344    105354    105360    105370
105325: ATGATTAATTTATTAGAGTTACATGCATTGGA----TATCAATTTAATGA  Hsapiens
425014: ................................----..............  Ptroglodytes
478537: ..T.............................----.........G....  Pabelli
372296: ......................T..TGACC..GTTGCC....AA......  Nleucogenys
180339:                                       ....C..C..A.  Mmulatta
421395: ..T.......G.................AAT.----.........G....  Panubis
```

```
                                          105381    105391
105371: TA-----------------------------TTATTATGCAGCAATTTAA  Hsapiens
425060: ..-----------------------------...................  Ptroglodytes
478583: ..-----------------------------...................  Pabelli
372346: A.AATTCAAAATGAGGAATGATGTCTCACCT.C..AT.A..AAT......  Nleucogenys
180351: G.-----------------------------A......CT.A.TG...G.  Mmulatta
421441: ..-----------------------------..T................  Panubis

            105398
105392: ACTT---GACTGGGAGAAA-------------------------------  Hsapiens
425081: ....---............-------------------------------  Ptroglodytes
478604: ....---............-------------------------------  Pabelli
372396: G.A.AGG.C...AC.C...CTGAAGGCTTAGTTAATGATTAATTTATTAG  Nleucogenys
180372: .GA.-------......G.-------------------------------  Mmulatta
421462: ....---............-------------------------------  Panubis

                      105415    105425    105435    105445
105408: ------------TATATAGCAATGTGAGGAAAGTTTACGAATACACCAAG  Hsapiens
425097: ------------......................................  Ptroglodytes
478620: ------------......................................  Pabelli
372446: AGTTACATGCAT.GG...T....T...T..T.T.A.---T..G..G...T  Nleucogenys
180384: ------------...G..AT.T.....-A.......C.-TT.TT.T...A  Mmulatta
421478: ------------..C.......................A...........  Panubis

            105451    105461    105471
105446: TAAAA----AAGGGAATAGAAATTTAGGAATTTGGGGAA-----------  Hsapiens
425135: .....----.G............................-----------  Ptroglodytes
478658: .....----..............................-----------  Pabelli
372493: .T...CTTG.CT..G.G.A.T..A...C...G..A....AGTTTACAAAT  Nleucogenys
180420: .....-----------.G..C...C.C-----------------------  Mmulatta
421516: ..T..----...........G......--------..T.-----------  Panubis

                                                    105487
105481: -------------------------------------------TTGCAAT  Hsapiens
425170: -------------------------------------------.......  Ptroglodytes
478693: -------------------------------------------.......  Pabelli
372543: ACACCAAGTAAAAAAGGGAATAGAAATTTAGGAAATTAGGGAA..A....  Nleucogenys
180436: -------------------------------------------.------  Mmulatta
421543: -------------------------------------------.----..  Panubis

            105497    105506    105515    105525    105535
105488: TTAATAATTGCAA-TGTGTACTAAATA-ATGTATACAGAAAAATAAGATG  Hsapiens
425177: .............-.............-......................  Ptroglodytes
478700: .............-.............-..A...................  Pabelli
372593: .............-.............-......................  Nleucogenys
180437: ---G.G.......C.A..---......T..A.GC.TTC...G.CT..GG.  Mmulatta
421546: ACG..........-...A.........-...C..............CC..  Panubis

            105545    105555    105565    105575    105585
105536: AGCCTATTAAAAATTGACACATGTAGTAGGCTGTTGGCACAAGAAATAGT  Hsapiens
425225: .....................--C..........................  Ptroglodytes
478748: ..G......................A.........A.......G......  Pabelli
372641: .................................................A  Nleucogenys
180481: ..GTAG......--...A.T..A..C....T..A.AAA.G.......---  Mmulatta
421594: ............................A.....................  Panubis
```

```
            105595    105605    105615    105625    105635
105586: GATATATATAGTTCATTGTGTACAAAATAATGTAATCATATTTTTACATG  Hsapiens
425273: ..............................A...................  Ptroglodytes
478798: ......................TG..........................  Pabelli
372691: ...............C......TG...............G....C.....  Nleucogenys
180526: ---------GAC.T...T.--------------....-----....A.AA  Mmulatta
421644: ........C..........A.........T.A.........-.......A  Panubis

            105643    105653    105663    105673    105683
105636: TGTATC--ATACAGTTGTATACATACATATGTACACATTACATATACATA  Hsapiens
425323: A.....--.......................................G..  Ptroglodytes
478848: .....T--..........................................  Pabelli
372741: .....T--..........................................  Nleucogenys
180549: C....TCA..G.TACG....TTCC.....CAA...G..A.TT..C.T.A.  Mmulatta
421693: .....T--.....T.................C..................  Panubis

            105693                        105709    105719
105684: AAAACTTGATTCTGTT--------------TATACACATACACATATAAC  Hsapiens
425371: ....TG..........TTTACACACATATA....................  Ptroglodytes
478896: .....A.T........TTTACATACATA--......T.............  Pabelli
372789: .....A..........TTTACATACATGTA......T.............  Nleucogenys
180599: G...--------------------------....---------.G.CG..  Mmulatta
421741: .....A......C...TTTAGATACATATA.T..  Panubis

            105729    105739    105749    105759    105769
105720: CCAATGTATTTATATATCCAGGACTCATATTTTGCCTATTAGAATAATAA  Hsapiens
425421: .................T................................  Ptroglodytes
478944: .................T................................  Pabelli
372839: ..........G......T...............A...T............  Nleucogenys
180614: .GCC..C.---------------------------G...A.AG.C.....  Mmulatta
193599:                     A...GACC.CC.GCAG...A.AG.C.....  Panubis

            105779    105789    105799    105809    105818
105770: TATCTAATAAAGTGAACCTTCTGTATTTCACATTTGTTGCCAAAAT-AAG  Hsapiens
425471: ..............................................-...  Ptroglodytes
478994: ...................................A..........-...  Pabelli
372889: ...................................A..........-G..  Nleucogenys
180637: .--...G.G..A.A..AT...CACT...A.T..G----A.T.....T..T  Mmulatta
193629: .--...G.G..A.A..AT...CACT...A.T...----A.T.....T..T  Panubis

            105828    105838    105848    105858    105868
105819: GATTCTCCACATAGTCAATTCATTGTTAAGGTGTATTAGAAATCAACAGT  Hsapiens
425520: ........................................G.........  Ptroglodytes
479043: C...............................T.......G..TG.T...  Pabelli
372938: ........................................G...G.T...  Nleucogenys
180681: A.C.TCTG..TG.CA...A...G.....CAC...T.....TT.TT.TTCA  Mmulatta
193673: A.C.TCTG..TG.CA...A...G.....CAC...T.....TT.TT.TT.A  Panubis
```

```
                                                         B3:1
                                                   <-------------
            105878     105887     105897     105905    105915
105869: TAGTCATATCAGTTT-CTTTTTTCCATTTGTA--TAGCTTGAAGAGAAAC  Hsapiens
425570: ...............-...............G--................  Ptroglodytes
165659:             ..A-T..CC....TC.....----.......AG....A  Ggorilla
479093: ...............-.........G......--................  Pabelli
372988: ...............-................--................  Nleucogenys
180731: ..A.....C..T.C.A..C..C.TTT....A.AA...A.AAT...T...A  Mmulatta
193723: ..A.....C..T.C.A..C..C.TTT....A.AA...A.AAT...T...A  Panubis
159155:             ..A-T..C.....TC.....----.......AGAC..A  Cjacchus
161795:                  ..C.....TC.....----.......AGAC..A  Sboliviensis

                                 B3:1
        <-------------------------------------------------
            105925     105935     105945     105955    105965
105916: TCACTGCTGAGAAATTGATGGAATGGACAAGTTTGCAGAATATGAGAGAG  Hsapiens
425617: ...............................................C..  Ptroglodytes
165692: ....CTG...A...A..G...CC....GC..C.CAG.A..C...TC...A  Ggorilla
479140: ....G............T...............CA...............  Pabelli
373035: .................................C................  Nleucogenys
180781: .A.G.C...........G.A..G.....T...CCAGG.C.....GA...A  Mmulatta
193773: .A.G.C...........G.A..G.....T...CCAGG.C.....GA...A  Panubis
159188: .T..CTA...A...A.AGC..CC....GT..C.CAG.A..C...TC...A  Cjacchus
161824: .T..CTA...A...A.AGC..CC....GT..C.CAG.A..C...TCG..A  Sboliviensis

                                 B3:1
        <-------------------------------------------------
            105975     105985     105995     106005    106015
105966: ACACGTGTCGATTTACACTTACCTCGGTTCAAAGTGGAAGAGAGCTATGA  Hsapiens
425667: ....C.............................................  Ptroglodytes
165742: GA.TCG..G.TCC.GTC...C..C.......CCC.......C........  Ggorilla
479190: ....A...G.........................................  Pabelli
373085: G.......G.........................................  Nleucogenys
180831: .G.A.G..GA..C.G.....G..C.....TG.G.....G..C..T..C..  Mmulatta
193823: .G.A.G..GA..C.G.....G..C.....TG.G.....G..C..T..C..  Panubis
159238: GA.TCG..G.TCC..TC...C..C.A.....CCC.......C........  Cjacchus
161874: GA.TCG..A.TCC..TC...C..C.A.....CCC.......C........  Sboliviensis

                                 B3:1
        <-------------------------------------------------
            106025     106035     106045     106055
106016: CCTCAAGGACACGTTGAGAACCATGGGAATGGTGGATATCTTCAATGG--  Hsapiens
425717: ................................................--  Ptroglodytes
165792: T.....TTC..TT..ACA.GA......C..TAC.........TG...AAA  Ggorilla
479240: .....................T..........................--  Pabelli
373135: ..............................................A.--  Nleucogenys
180881: ...TG...CGGTCC..GCTG.......G..A.GC...GC.....G..A--  Mmulatta
193873: ...TG...CGGTCC..GCTG.......G..A.GC...GC.....G..A--  Panubis
159288: T.....TTC..TT..ACA.GA......C..TAC.........TG...AAA  Cjacchus
161924: T.....TTC..TT..ACA.GA......C..TACA........TG...AAA  Sboliviensis
```

```
                                  B3:1
         <-----------------------------------------------------
                 106069    106079    106089    106099    106109
                      :         :         :         :         :
106064: -G---GATGCAGACCTCTCAGGCATGACCGGGAGCCGCGGTCTCGTGCTA  Hsapiens
425765: -.---...............................A............T  Ptroglodytes
165842: C.---AGG..T..T..TA.T..A..CT.TCCA..T.C.AA.T.GTACT.G  Ggorilla
479288: -.---.......................T...............A..G..  Pabelli
176662:                                      .AA.T.GTACT.G  Nleucogenys
373183: -.---.......................T........              Nleucogenys
180929: -.CACA.A..T...TA...G..A...T..-TC.CT.T.T.GG.T.CA.GC  Mmulatta
193921: -.CACA.A..T...TA...G..A...T..-TC.CT.T.T.AG.T.CA.GC  Panubis
159338: C.---AGG..T..TT.TA.T..A..CT.TCCA..T.C.AA.T.GTACT.G  Cjacchus
161974: C.---AGG..T..T..TA.T..A..CT.TCCA..T.C.AA.T.GTACT.G  Sboliviensis

                                  B3:1
         <-----------------------------------------------------
                 106118    106128    106138    106148    106158
                      :         :         :         :         :
106110: TCTGGAG-TCCTACACAAGGCCTTTGTGGAGGTTACAGAGGAGGGAGCAG  Hsapiens
425811: .......-..........................................  Ptroglodytes
165889: ..AAA.A-.TA.C.....AA.............GGAT..AA.C..CA.CC  Ggorilla
479334: ...AA..-............................T.............  Pabelli
176675: ..AAA.A-.TA.C.....AA.............GGAT..AA.T..CA.CC  Nleucogenys
180977: C.A.A..T....G....GTT.........C...A..T..A..A..CA.C.  Mmulatta
193969: C.A.A..T....G....GTT.........C...A..T..A..A..CA.C.  Panubis
159385: ..AAA.A-.TA.G.....AA.T...........GGAT..AA.C..CA.CC  Cjacchus
162021: ..AAA.A-.TA.G..T..AA.T...........GGAT..AA.T..CA.CC  Sboliviensis

                                  B3:1
         <-----------------------------------------------------
                 106168    106178    106188    106198    106208
                      :         :         :         :         :
106159: AAGCTGCAGCTGCCACCGCTGTAGTAGGATTCGGATCATCACCTACTTCA  Hsapiens
425860: .......C.........A........CC.C....................  Ptroglodytes
165938: .G---........AG..A...GG.CT.TTG..TCGGA.AGGT.ACTACG.  Ggorilla
479383: .G...T.....................T.AAA.T.A.....T.ACG.C..  Pabelli
176724: .G---........AG..A...GG.CT.TTG..TCGGA.AGGT.ACTAC..  Nleucogenys
181027: .G................GCA...--.CT..ACTG...-..T.CG.CC..  Mmulatta
194019: .G................GCA...--.CT..ACTG...-..T.CG.CC..  Panubis
159434: .G---........AG..A.A.GG.CT.CT....AGGG.AGGT.CTTA...  Cjacchus
162070: .G---........AG..A...GG.CT.CTG...CGGG.AGGT.CTTA...  Sboliviensis

                                  B3:1
         <-----------------------------------------------------
                 106218    106228    106238    106248    106258
                      :         :         :         :         :
106209: ACTAATGAAGAGTTCCATTGTAATCACCCTTTCCTATTCTTCATAAGGCA  Hsapiens
425910: ..................................................  Ptroglodytes
165985: T..TGG.TG.....TA..GCC..C........T..C..T.....T..A..  Ggorilla
479433: ............................................C.....  Pabelli
176771: T..TGG.TG.....TA..GCC..C........T..C..T.....T..A..  Nleucogenys
181074: GG.C.....A.TG.T..C..C.....T..C.....G........C.....  Mmulatta
194066: GG.C.....A.TG.T..C..C.....T..C.....G........C.....  Panubis
159481: T..TGG.TG.....TA..GCC..C........T..G..T.....T..A..  Cjacchus
162117: T..TGG.TG.....TA..GCC..C........T..G..T.....T..A..  Sboliviensis
```

```
                                        B3:1
         <-------------------------------------------------------
                   106268    106278    106288    106298    106307
                        :         :         :         :         :
106259:  AAATAAGACCAACAGCATCCTCTTCTATGGCAGATTCTCATCCCCGT-AG  Hsapiens
425960:  ...C...........................................-..  Ptroglodytes
166035:  C..C..A...C.A.C...T.....T........GG...GC..T..T.-.A  Ggorilla
479483:  .............................................A.-..  Pabelli
176821:  C..C..A...C.A.C...T.....T.........G...GC..T..T.-.A  Nleucogenys
181124:  C...G.AT..................T.........T..T..T..T.A..  Mmulatta
194116:  C...G.AT..................TC........T..T..T..T.A..  Panubis
159531:  C..C..A...C.A.C...T.....T........GG...GC..T..C.-.A  Cjacchus
162167:  C..C..A...C.A.C...T..............GG...GT..T..T.-.A  Sboliviensis

                   106317    106323    106333    106343    106353
                        :         :         :         :         :
106308:  ATGCAATTAGTC----TGTCACTCCATTTGGAAAATGTTCACCTGCAGAT  Hsapiens
426009:  ............----.............A..............AG....  Ptroglodytes
166084:  .                                                   Ggorilla
479532:  ............----..........C..A..............AG..G.  Pabelli
176870:  .                                                   Nleucogenys
181174:  ...ATCG.T.C.ATGGCA.TG..G.T...A.C...AAACA...AC...TG  Mmulatta
194166:  ...ATCG.T.C.ATGGCA.TG..G.T...A.C...AAACA...AC...TG  Panubis
159580:  .                                                   Cjacchus
162216:  .                                                   Sboliviensis

                   106363    106373    106382    106392    106402
                        :         :         :         :         :
106354:  GTTCTGGTAAACTGATTGCTGGC-AACAACAGATTCTCTTGGCTCATATT  Hsapiens
426055:  ...G...................-..........................  Ptroglodytes
479578:  .......................A.....................T....  Pabelli
181224:  T.AT.CA..G---....A-..AA-.GTCGTCC.....T..AAA.GT.G.C  Mmulatta
194216:  T.AT.CA..T---....A-..AA-..TCGTCC.....T..AAA.GT.G.C  Panubis

                   106410    106420    106430    106438    106448
                        :         :         :         :         :
106403:  T--CTTTTCTTTCTCATCTTGATGATGATCGTCATC--ATCAAGAATTTA  Hsapiens
426104:  .--..........................A......--............  Ptroglodytes
479628:  .--.......A....C.............A....C.-C............  Pabelli
181269:  .CA...GCA....CAG....----------.C..C.AA.....TG.....  Mmulatta
194261:  .CA...GCA....CAG....----------.C..C.AA.....TG.....  Panubis

                   106458    106468    106478    106488    106498
                        :         :         :         :         :
106449:  ATGATTAAAATAGCATGCCTTTCTCTCTTTCTCTTAATAAGCCCACATAT  Hsapiens
426150:  ..................................................  Ptroglodytes
479675:  ..............................................CC..  Pabelli
181310:  ....CCCC.....TG.A.G.G.T.G.AACCA..C.TGA...T-------G  Mmulatta
194302:  ....CCCC.....TG.A.G.G.T.G.AACCA..C.TGA...T-------G  Panubis

                   106508    106518    106528    106538    106548
                        :         :         :         :         :
106499:  AAATGTACTTTTTCTTCCAGAAAAATTCTCCTTGAGGAAAAATGTCCAAA  Hsapiens
426200:  ..........C.......................................G  Ptroglodytes
479725:  ...C.......................................A.....G  Pabelli
181353:  ......C.....CT..-------------------------G..C.ATGC  Mmulatta
194345:  ......C.....CT..-------------------------G..C.ATGC  Panubis
```

```
                 106558    106568    106578    106588    106598
106549: ATAAGATGAATCACTTAATACCGTATCTTCTAAATTTGAAATATAATTCT  Hsapiens
426250: ........................C.........................  Ptroglodytes
479775: ......C......T.......T............................  Pabelli
181378: .....G...G...A------..AA.C..CA.T...----------.A...  Mmulatta
194370: .....G...G...A------..AA.C..CA.TG..----------.A...  Panubis

                 106608    106618    106628              106644
106599: GTTTGTGACCTGTTTTAAATGAACCAAACCAAATCATAC----TTTTTCT  Hsapiens
426300: ....C..................................----.......  Ptroglodytes
479825: ..........................C...........A----...C...  Pabelli
181413: CCCCT..GTT.C....G..A.G.--..T.GGT...T.G.AGTT..G.G.A  Mmulatta
194405: CCCCT..GTT.C....G..A.G.--..T.GGT...T.GTAGTT..G.GAA  Panubis

                 106654    106658    106668    106678    106687
106645: TTGAATTTAGCAA------CCTAGAAACACACATTTCTTTG-AATTTAGG  Hsapiens
426346: .............------......................-........  Ptroglodytes
479871: .C...........------......................-........  Pabelli
181461: C....GGAGA...AGTCTCT.C..T...GAGT..GAAC.A.T.....T..  Mmulatta
194453: C.A..GGAGA...AGTCTCT.C..T...GAGT..GAAC.A.T.....T..  Panubis

                 106697    106707    106717    106727    106737
106688: TGATACCTAAATCCTTCTTATGTTTCTAAATTTTGTGATTCTATAAAACA  Hsapiens
426389: ...................C..............................  Ptroglodytes
479914: ....................A.............................  Pabelli
181511: G..GGT.--------....TAA..CTAGT......AC..GT.....T...  Mmulatta
194503: G..GGT.--------...CTAA..CTAGT......AC..GT.....T...  Panubis

                 106747    106757    106767    106777    106785
106738: CATCATCAATAAAATAGTGACATAAAATCATTTTTGCTTTACA--TTTTC  Hsapiens
426439: ..................A................A......CTT.....  Ptroglodytes
479964: ................A.........GA..............CTT.....  Pabelli
181553: ..------.G......---.A.C..T.GTT.AC.C.GC.C.TG--..A.T  Mmulatta
194545: ..------.G......---.A.C..T.GTT.AC.CAGC.C.TG--..A.T  Panubis

                           106804    106814    106824    106834
106786: TCTCTGGAA-AGGGAAAGTGTCCAGTTACACACAGGAAAGACAATTTAGA  Hsapiens
426489: .........-....G...................................  Ptroglodytes
480014: .........-....G.................T.................  Pabelli
181593: AT...CC..C..AT.TT...C.A.A..G....T........A.......G  Mmulatta
194585: AT...CC..C..AT.TT...C.A.A..G....T........A.......G  Panubis

                 106844    106854    106864    106874    106884
106835: GATATATTAATCACAAACAAAGGAAAATTAAAAACAGAGTAGTTCATGAT  Hsapiens
426538: ...............T..................................  Ptroglodytes
480063: .............T.T.T................................  Pabelli
181643: A...C.G..GCA.A.C.T...TT....C.C...                   Mmulatta
194635: A...C.G..GCA.A.C.T...TT....C.C...                   Panubis

                                         LINE2
                                   ------------------------>
                 106894    106904    106914    106924    106934
106885: GAGCCTGGAGTAGAAGGCATATCCCAGAACAGGAGGAGCCTTGTAAACCA  Hsapiens
426588: A.................................................  Ptroglodytes
480113: ........G.........TCC.............................  Pabelli
```

```
                                 LINE2
         ---------------------------------------------------->
                  106944    106954    106964    106974    106984
106935: CATTAGGAACTTCCTATTTTATGCTAAAGGGATAAGAAACTCATTACAGG  Hsapiens
426638: ..................................................  Ptroglodytes
480163: .....................C.........................T..  Pabelli

                                 LINE2
         ---------------------------------------------------->
                  106994    107004    107014    107024    107034
106985: CTTTGATGGTTGTTTGTCAAAGAGGGGCATAAAATTATCATATCTTCATC  Hsapiens
426688: ..................................................  Ptroglodytes
480213: ...........T...........A......T.C............G....  Pabelli

                                 LINE2
         ---------------------------------------------------->
                  107044    107054    107064    107074    107084
107035: TTGAAAATACATCTCTGGTTACTCTGATATCAATGGATGAGAGGAAAGAA  Hsapiens
426738: ................................G.................  Ptroglodytes
480263: .......C..........C....................C..........  Pabelli

                                 LINE2
         ---------------------------------------------------->
                  107094    107104    107114    107124    107131
107085: CAGTGTGGTTACCATATATAAATCAGGAAATCATTAGAGTAT---TGGGG  Hsapiens
426788: ..........................................---.....  Ptroglodytes
480313: .......................T.............CA...---....A  Pabelli
188577:         .C..T..G..C.TG.ATATC..A...C..GT.G.ATACA.A.  Mmulatta

          LINE2
         ---------->
                  107141    107151    107161    107171    107181
107132: GTGGAAATGAAGAGAAAGAAACAGCCTGGCAGAATTATTTAGGAAGTAAT  Hsapiens
426835: .............................G....................  Ptroglodytes
480360: .A.......G...................GG...................  Pabelli
188619: A.AT...AT..A.A.T.A...A..AT..-------.G.G.TT...A.G..  Mmulatta

                  107191    107201    107211    107221    107231
107182: AGTTAAAAAAAGACATCTAAGTTTCTGAGCTATCTGACTGGAAGGATGGA  Hsapiens
426885: .......................G....C.....................  Ptroglodytes
480410: C......G....G...............C...G.........T.......  Pabelli
188662: .T.C....G.G.....T...                                Mmulatta

                                        LINE2
                      ------------------------------------->
                  107241    107251    107261    107271    107281
107232: AGAAAATGTTGTTTTTGAGAGAAAAAAAAGACCATGGGTTTAAATTTGTA  Hsapiens
426935: ..................................................  Ptroglodytes
480460: ................A....-............................  Pabelli

                                 LINE2
         ---------------------------------------------------->
                            107300    107310    107320    107330
107282: CTTGATGAA-TTGAGGTACTTTTAATATTCAAATGAATTTGCCTGGCAGG  Hsapiens
426985: .C.......-.C......................AG..............  Ptroglodytes
480509: ....T....T...............G.........G..............  Pabelli
```

```
                                  LINE2
        ---------------------------------------------------->
            107340    107350    107360    107370    107380
                 :         :         :         :         :
107331: TACTTGAAGATATTGATCTAAATCTCAGAAACAGAATATGATCTGAAGCT  Hsapiens
427034: ..................................................  Ptroglodytes
480559: C...........C..G.................T................  Pabelli

                                  LINE2
        ---------------------------------------------------->
            107390    107400    107410    107420    107430
                 :         :         :         :         :
107381: CTAAATTTGTGATATTCAACATAAATACTTTAGAGTTATTGGGATAAACA  Hsapiens
427084: ................................................T.  Ptroglodytes
480609: ...................T......C.....................T.  Pabelli

                                  LINE2
        ---------------------------------------------------->
            107440    107450    107460    107470    107480
                 :         :         :         :         :
107431: TGGTAATTGTAGCTAGAAGCAAAGATAAGATCCTAGGGAGGAAGGATAAA  Hsapiens
427134: ........................................A.........  Ptroglodytes
480659: ........-......A........................A.....G...  Pabelli

                                  LINE2
        ---------------------------------------------------->
            107490    107500    107510    107520    107530
                 :         :         :         :         :
107481: GTTAGAAGAAAGAAGAATCTGGAATTGACCTTGAAGTATATCAATATGTG  Hsapiens
427184: ....................A......................G......  Ptroglodytes
480708: ...................CA................T....T.......  Pabelli

             LINE2
        ------------->
            107540    107550    107560    107570    107580
                 :         :         :         :         :
107531: TAAAGATCATGAACTGATCATTTTTATTTTCCAGAAAGTAGCTTTTCTTA  Hsapiens
427234: .........G.......................A...A............  Ptroglodytes
480758: .........G...T....................................  Pabelli

            107590    107600    107610    107620    107630
                 :         :         :         :         :
107581: GGGTTCCATATTTACTCCCATAGATTCTTCCCAGTCAGTACTGAATCCCA  Hsapiens
427284: ..................................................  Ptroglodytes
480808: .................................T................  Pabelli

            107640    107650    107660    107670    107680
                 :         :         :         :         :
107631: CCTGGTTTCATAAAACATAAGTTTTAGAGAATTTGAACAAGGTTGAAGTT  Hsapiens
427334: ........................G.........................  Ptroglodytes
480858: ...................C.................A............  Pabelli

            107690    107700    107710    107720    107730
                 :         :         :         :         :
107681: TTCTTCAGCAAGATTCAGCAGTCCAGGATGAAGGCAGAGGAAACAAATGT  Hsapiens
427384: .........................A........................  Ptroglodytes
480908: ..................A......AA..T....................  Pabelli

            107740    107750    107760    107770    107780
                 :         :         :         :         :
107731: TTCACCACTTAACTGTGAGACTTGGTCAAGTTGTCGAGGGAATTGACAGA  Hsapiens
427434: ..............A...................................  Ptroglodytes
480958: ..............A...................................  Pabelli
```

```
                 107790    107800    107810    107820    107830
                      :         :         :         :         :
107781: CTAGCCAGAGAGTGTATAACATCAACAATCTGTTCAGTGAATACAGCTGG  Hsapiens
427484: ....................C.G..................GG......  Ptroglodytes
481008: ............G...G.....G.................G.GG......  Pabelli

                 107840    107850    107860    107870    107880
                      :         :         :         :         :
107831: GGAAAGAAAGCAAAGAAGGATGTAATAGTCTTGAAGAACACATGTGCATT  Hsapiens
427534: ..........................................G.......  Ptroglodytes
481058: ..................................................  Pabelli
167237:          .....A..A.T...T.....G.AT..A.T..G...GA....  Sboliviensis

                 107890    107900    107910    107920    107930
                      :         :         :         :         :
107881: ACAATACTTACTGAGGTCAAATAAGGATGGTATTGAGTTTATGAGAAAGA  Hsapiens
427584: ..........................------.......A..........  Ptroglodytes
481108: ..................T...........................T.  Pabelli
167278: .                                                   Sboliviensis

                 107940    107950    107960    107970    107980
                      :         :         :         :         :
107931: TGAAGAGGTCAGAAGTTTGGGCCAGGACCTATGATTTTGAAATATGCTTT  Hsapiens
427628: ........................................G...A...-  Ptroglodytes
481158: ........................................G...A....  Pabelli
222351:                           .TT...C.....A..G..CA...C  Mmulatta
235332:                           .TT...C.....A..G..CA...C  Panubis

                 107990    108000    108010    108020    108030
                      :         :         :         :         :
107981: AAAATGATAAATTGTAAGGAATTATAAAGCCCAAATTCTGTCCAATAGGT  Hsapiens
427677: ..............................T.........C...G.....  Ptroglodytes
481208: ..............................T...................  Pabelli
222375: .....C...G.                                         Mmulatta
235356: .....C...G..                                        Panubis

                 108040    108050    108060    108070    108080
                      :         :         :         :         :
108031: GAGGATTAATTTCTAATGATTCAATGAAGCTGAAAATGGTAGAGTGGATA  Hsapiens
427727: A.....C........G..................T..............  Ptroglodytes
481258: ....................G.............................  Pabelli

                 108090    108100    108110    108120    108130
                      :         :         :         :         :
108081: GTTTTGAAGAACTGATTGTTAGAATCACTTTAGACCATATCCTTCAACTA  Hsapiens
427777: ..................................................  Ptroglodytes
481308: ...........T.....A................................  Pabelli

                 108140    108150    108160    108170    108180
                      :         :         :         :         :
108131: TTATTAATTTTTCTGGGTTTAAAAGAAGGTACTTTTTAATAATGAAAATG  Hsapiens
427827: ..................................................  Ptroglodytes
481358: ............................T......C.............  Pabelli

                 108190    108200    108210    108220    108230
                      :         :         :         :         :
108181: TTAAGACAAAGCTCATAAGCATTAGATTCTTATAGAGGAGAGGGCATAGT  Hsapiens
427877: ..................................................  Ptroglodytes
481408: ..........................G.........C...........  Pabelli
```

```
          108240    108250    108260    108270    108280
108231: TGATATTTTGTTAGGAGAATTGAAATATTTACATACATTCTAGGCCCACC  Hsapiens
427927: ..........................................A.......  Ptroglodytes
481458: ..............................GT..................  Pabelli

          108290    108300    108310    108320    108330
108281: AGAAAACCAGTTGCCCCTGCATTGTAGCCATCTGCAAGAGACACTGAAGG  Hsapiens
427977: ......................................G...........  Ptroglodytes
481508: ............A.....................................  Pabelli

                                            LINE1
                                    ------------------>
          108340    108350    108360    108370    108380
108331: GAAAGCCAAAGTTTTTGTCTTGATTTTTTAAATAATGAATTAAAATCTAT  Hsapiens
428027: .....T....T.......T...............................  Ptroglodytes
481558: ..........T....G..T.........................C.....  Pabelli

                              LINE1
        -------------------------------------------------->
          108390    108400    108410    108420    108430
108381: TGAGTAAAACAGAATATGAGTCCCTGCTCATATAAAATAAATGACTAAAT  Hsapiens
428077: ...A............C...........G...C.................  Ptroglodytes
481608: ...A............C......T....G.....................  Pabelli

          108440    108450    108460    108470    108480
108431: TAAAAGTTTCATAAGAAATGCAAAATTTAAGTGCTTTCAAAACTCCTCCT  Hsapiens
428127: ..................................................  Ptroglodytes
481658: .................................................C  Pabelli

                              LINE1
         ------------------------------------------------->
          108490    108500    108510    108520    108530
108481: CATGGAATACCACTTTGTACCAATTATGATGGCCAAAATTAAAACTTATA  Hsapiens
428177: ................................T.................  Ptroglodytes
481708: ................................................A.  Pabelli

                              LINE1
        -------------------------------------------------->
          108540    108550    108560    108570    108580
108531: ATACTGGGCCATTGCAAGTTTTGAAAAGAATGTGAAGCAACTGGAACTCT  Hsapiens
428227: ...G..A...........................................  Ptroglodytes
481758: .....A............................................  Pabelli

                              LINE1
        -------------------------------------------------->
          108590    108600    108610    108620    108630
108581: CACAAACTGCTGATGGAAATACAAAAGGTGCAACCATGTTGGAAAACAGT  Hsapiens
428277: ..................................................  Ptroglodytes
481808: ....................................CA............  Pabelli
```

```
      LINE1
      -->
               108640    108650    108660    108670    108680
108631: CTTAAATCACTGAACTACACACTTACATTTTATTTTAATTAATTATGCCT Hsapiens
428327: .................................................. Ptroglodytes
481858: .........................T.....................T.. Pabelli

               108690    108700    108710    108720    108730
108681: CTATAAAGTTGACTCAAAAGAGGAAAAAGAGAATTGTCCAAAACACACAT Hsapiens
428377: .................................................. Ptroglodytes
481908: ...............G..G........................T...... Pabelli

               108740    108750    108760    108770    108779
108731: CTTTAGGTGAAAAAATCTTCTTCATTAAATGTTGTTTTTAAATGTA-TTT Hsapiens
428427: ...C..........................................T... Ptroglodytes
481958: T.............................................T... Pabelli

                           LINE1
             ------------------------------------------------>
               108789    108799    108809    108819    108829
108780: TAATTAATAAAAACTGTATACATTTATGTTGTAAAACATGTTGTTTTGAA Hsapiens
428477: .................................................. Ptroglodytes
482008: .................................................. Pabelli

                         LINE1
        -------------------------------------------------->
               108839    108849    108859    108869    108879
108830: ATATGTATACATAGTGGAATGAGTCAGTGGAGCTAATTTATATATACATT Hsapiens
428527: ............................A..................... Ptroglodytes
482058: ............................A..................... Pabelli

                         LINE1
        -------------------------------------------------->
               108889    108899    108909    108919    108929
108880: ACCTCACATAATTTTTTGCGGTAAGAACACTGCAAGTCTGTTCTCTTAGC Hsapiens
428577: ..................T............................... Ptroglodytes
482108: ..................T.A..T........A.......-......... Pabelli

                         LINE1
        -------------------------------------------------->
               108939    108949    108959    108969    108979
108930: AATTTTCAAAAATAAAATGTATTAACCATAGTCACCATGTTGTAAAACAG Hsapiens
428627: .....................G............................ Ptroglodytes
482157: G..................C......T....................T.. Pabelli

                         LINE1
        -------------------------------------------------->
               108989    108999    109009    109019    109029
108980: ACCTTCTGAACTCATGTCTCCTGTTTAACTGAAATTTGTATCCTTTGACC Hsapiens
428677: .................................................. Ptroglodytes
482207: ...............T.............................CA... Pabelli
```

```
                               LINE1
         ------------------------------------------------------>
              109039    109049    109059    109069    109079
109030: AACATCTCCTCAACTCCCTCTCACATCCCGGCTCCTGGTAACCACCATTT  Hsapiens
428727: .........................C........................  Ptroglodytes
482257: .........................C........................  Pabelli

                               LINE1
         ------------------------------------------------------>
              109089    109099    109109    109119    109129
109080: TACTCTTTATTTATATGAGTTCAACTTTTCTAGATTCCACATATAAGAGA  Hsapiens
428777: ..................................................  Ptroglodytes
482307: .........C..G............................AG.......  Pabelli

                               LINE1
         ------------------------------------------------------>
              109139    109149    109159    109169    109179
109130: GACCATGCAATATTTGTCTTTCTCTGCATGACTTATTTCACTCAATATAA  Hsapiens
428827: ..T..........................................C....  Ptroglodytes
482357: ..T...........................G..............C....  Pabelli

                               LINE1
         ------------------------------------------------------>
              109189    109199    109209    109219    109229
109180: TGTTCTCCAGGTTTACCCTGTTGTCATAAATGACAGGATTTCCTTCCTTT  Hsapiens
428877: ...........................G..............T.......  Ptroglodytes
482407: C................A.......G..........AG............  Pabelli

                               LINE1
         ------------------------------------------------------>
              109239    109249    109259    109269    109279
109230: TAAAGGCTGAATAGTAAGTATTCTACTGTGCATGTATATAACACATTTTC  Hsapiens
428927: ...................T..............................  Ptroglodytes
482457: ..............................A......G............  Pabelli

                               LINE1
         ------------------------------------------------------>
              109289    109299    109309    109319    109329
109280: CTCATCCAGTCACTCATTATTACATAGATGGGTTGATTCCGTATTTTGAT  Hsapiens
428977: ............T.......................C.T.A.........  Ptroglodytes
482507: ..TG......................C............T..........  Pabelli

                               LINE1
         ------------------------------------------------------>
              109339    109349    109359    109369    109379
109330: TATTGTGAATAATGCTGCAATGAACATGTGAATAAAGATATCTGTTCAAC  Hsapiens
429027: .........................G..G.....................  Ptroglodytes
482557: ............................G..............-......  Pabelli

                               LINE1
         ------------------------------------------------------>
              109389    109399    109409    109419    109429
109380: GTAACTGCTTTTATTTCCTTTGGAAATATACCCAAAAGTGGAATTATTAA  Hsapiens
429077: ..................................................  Ptroglodytes
482606: A.........................C.......................  Pabelli
```

```
                               LINE1
         ---------------------------------------------------->
                 109439    109449    109459    109469    109479
109430: ATCATATCATCGTTCTGTATTTACTTTTCTGAAGAATCTTTATGATGTTT  Hsapiens
429127: ........G.A.......................................  Ptroglodytes
482656: ........G.A............A..........................  Pabelli

                               LINE1
         ---------------------------------------------------->
                 109489    109499    109509    109519    109529
109480: TCCATAATGTCTATGCTAATTTATAATCCAACCAACAGAGTAAAATAAGT  Hsapiens
429177: ..................................................  Ptroglodytes
482706: ............G..............T.................C....  Pabelli

                               LINE1
         ---------------------------------------------------->
                 109539    109549    109559    109569    109579
109530: CTGCTTCTGCAATGTTCTTGTCAACACTTGTTATCATTTATGTTTTTGAC  Hsapiens
429227: ...A...................................G..........  Ptroglodytes
482756: ..A.....A...C.C..........T.............G..........  Pabelli

                               LINE1
         ---------------------------------------------------->
                 109588    109598    109608    109618    109628
109580: -AAAAGCCATCCTAACAGGAGTGAGGTGATATTTAATTATGGTTTAGTTG  Hsapiens
429277: -....................................C............  Ptroglodytes
482806: A.................................C....A..........  Pabelli

                               LINE1
         ---------------------------------------------------->
                 109638    109648    109658    109668    109678
109629: GCATTTCTCAAATGATTATTGATATTGCATGTTTTCTATTGACAAATGAT  Hsapiens
429326: ...........G......................................  Ptroglodytes
482856: ..G.............................................TC  Pabelli

                               LINE1
         ---------------------------------------------------->
                 109688    109698    109708    109718    109728
109679: TATTCAGATCATTGGCCTATTTTTAATTGGGTTATTTGTTTTATTGTTAT  Hsapiens
429376: ..................................................  Ptroglodytes
482906: ...........A...................C..................  Pabelli

                               LINE1
         ---------------------------------------------------->
                 109738    109748    109758    109768    109778
109729: TCAGTTGTTTCAGTTCCTTATATATTTTGGATATTAATCCCTTTTGAGAT  Hsapiens
429426: ..................................................  Ptroglodytes
482956: ..................................................  Pabelli

                               LINE1
         ---------------------------------------------------->
                 109788    109798    109808    109818    109828
109779: GCATGCTTTACAGATATTTTTTCTCCCATTTATCTCTTCACTGTGCTGAT  Hsapiens
429476: ..........................................C.......  Ptroglodytes
483006: ..........................................C.......  Pabelli
```

```
                          LINE1
         ------------------------------------------------>
                109838    109848    109858    109868    109878
109829: TCTCATTTGTGCAGAAGCTTTTTCATGGTCTCATTTGTCTAATTTTGCTT  Hsapiens
429526: ..............................................T.-.  Ptroglodytes
483056: ..................................................  Pabelli

                          LINE1
         ------------------------------------------------>
                109888    109898    109908    109918    109928
109879: TTTTTGCCTTTGATTTCATATCCAAAAAATTATTGCCCAGACCAATATCA  Hsapiens
429575: ..................................................  Ptroglodytes
483106: ..................................................  Pabelli

                          LINE1
         ------------------------------------------------>
                109938    109948    109958    109968    109978
109929: TGAAGCTTTTCTCATATGTTTTCTACTAGTTGTTTTATACTTTTAAGTCT  Hsapiens
429625: .............G...A............................A...  Ptroglodytes
483156: ...G....................C.........................  Pabelli

                          LINE1
         ------------------------------------------------>
                109988    109998    110008    110018    110028
109979: TACCTGTAATCTATTTGGAATTGATTTTTTATATGGTGTAAGATATGGTT  Hsapiens
429675: ..................................................  Ptroglodytes
483206: ....G.............................................  Pabelli

                          LINE1
         ------------------------------------------------>
                110038    110048    110058    110068    110078
110029: CTAATTTCTTTCTTCTCTATGTGAATACGGTTTTTTAAAACCATTTATTA  Hsapiens
429725: .......................................G..........  Ptroglodytes
483256: ...........................T.....................G  Pabelli

                          LINE1
         ------------------------------------------------>
                110088    110098    110108    110118    110128
110079: AAGAGACTGTCTTTTCCCCATTATATGTTCTTGGCGCATTTTAAAAAACT  Hsapiens
429775: ...........C........................A.............  Ptroglodytes
483306: .......C...C..............A........C..............  Pabelli

                          LINE1
         ------------------------------------------------>
                110138    110148    110158              110173
110129: AATTGGCCATAAATATCTGCATCTGTTTCTGGACTGTA-----TTATACT  Hsapiens
429825: ......................................TTCTC.......  Ptroglodytes
483356: .........AT...........................TTCTC....T..  Pabelli

                          LINE1
         ------------------------------------------------>
                110183    110193    110203    110213    110223
110174: TGTTTATGTGCCTGTTTTTATGCCAGTACGATGCCATTTTGATTAATATT  Hsapiens
429875: .............................A....................  Ptroglodytes
483406: .............................A...T................  Pabelli
```

```
LINE1
         110233    110243    110253    110263    110273
110224: GATTCATAAGTTATTTTGAAGTTAAGTAGTGTGATGCCTCCTGTTTTCTT  Hsapiens
429925: ..................................................  Ptroglodytes
483456: ....................................G.............  Pabelli

LINE1
         110283    110293    110303    110313    110323
110274: CTCTTTGTTCAAAATTGCTTGACTATTCTAGGATTTTTGTGGTTCTATAC  Hsapiens
429975: ..................................................  Ptroglodytes
483506: ..................................................  Pabelli

LINE1                                Simple
         110333    110343                  110357    110367
110324: ATATTTTAGGATTTTTTTTTTTTT------TTGGAGTTGGAGTTGGAGTT  Hsapiens
430025: ........................TTGGAG........A...........  Ptroglodytes
483556: ........................--------------............  Pabelli

Simple        Alu
         110377    110387    110397    110407    110417
110368: GGAGTTGGAGTCTCGCTCTGTCACCCAGCTGGAGTGCAGTGGCTCAATCT  Hsapiens
430075: ...........T.......C.......................G......  Ptroglodytes
483592: ........C.....A...G........................A.G....  Pabelli

Alu                                      LINE1
         110427    110437    110447    110457    110467
110418: CTGCTCACTGCAAGCTCCGCCTCCCAGGCGTGTATCTCATTTTCATTACA  Hsapiens
430125: ..................................................  Ptroglodytes
483642: .............................A..........G.........  Pabelli

LINE1
         110477    110487    110497    110507    110517
110468: GCATTATTCACAATAGCCAAGACATGAAATCAGCCTGTGTCCAACAACAA  Hsapiens
430175: ...........................................C......  Ptroglodytes
483692: ...........................................T......  Pabelli

LINE1
         110527    110537    110547    110557    110567
110518: ATGAATAAATAAAGAAAATATGATATATATACACAATGAAATACTATTCC  Hsapiens
430225: ..................................................  Ptroglodytes
483742: ..................................................  Pabelli

LINE1
         110577    110587    110597    110607    110617
110568: ACCATAAAAAAAAAGACAGTTCTGTCATTTGCAACAACATGGATGAACCT  Hsapiens
430275: .....--...........................................  Ptroglodytes
483792: .....--...........................................  Pabelli
```

```
                                  LINE1
        ------------------------------------------------->
                110627    110637    110647    110657    110667
110618: GGAGGACATTATGTTAAGTGAAGTAAGCTAGATACAGAAAGACTAATACT Hsapiens
430323: ................................C................. Ptroglodytes
483840: ......T...............A.........C................. Pabelli

                            LINE1                   LINE1
        -------------------------------------->----------->
                110677    110687    110697    110707    110717
110668: ACATAACCTCAATTATATGTGGAATCTAAAAATGTTGATTTTTGACAAAG Hsapiens
430373: .................................................. Ptroglodytes
483890: ................................CA................ Pabelli

                                  LINE1
        ------------------------------------------------->
                110727    110737    110747    110757    110767
110718: ATGCCAAGGACACACAATCAGGAAAGGACAGTCTCTTCAATAAGGAGTGT Hsapiens
430423: .................................................. Ptroglodytes
483940: .................................................. Pabelli

                                  LINE1
        ------------------------------------------------->
                110777    110787    110797    110807    110817
110768: TGAAAAAAATTGGATATCCATATGGAGAAAAAGGAAATTAGATCCTCATC Hsapiens
430473: ..-............................................... Ptroglodytes
483990: ..-...................C........................... Pabelli

                                  LINE1
        ------------------------------------------------->
                110827    110837    110847    110857    110867
110818: CCACACCATATACAAAAATCAACTGAAAACCGATTGAAGGCTTAAATGTA Hsapiens
430522: T....T........................T................... Ptroglodytes
484039: T........G....................T................... Pabelli

                                  LINE1
        ------------------------------------------------->
                110877    110887    110897    110907    110917
110868: ACGACTGAAAATGTAAAACTATCAGAAGAAAATATAGAGGAAAAGCTTCA Hsapiens
430572: .......................T.......................... Ptroglodytes
484089: .G...................C............................ Pabelli

                                  LINE1
        ------------------------------------------------->
                110927              110939    110949    110959
110918: TGACATTGGTCTGGCC--------TTTAGATATGATTCCAAAAGCACAGG Hsapiens
430622: ..............G.--------.......................... Ptroglodytes
484139: ..........T...G.AATAATTT........C................. Pabelli

                                  LINE1
        ------------------------------------------------->
                110969    110979    110989    110999    111009
110960: CCACAAAAGTAAAAATAGACAAATGTGATTACATAAAACTGAAACTTCTT Hsapiens
430664: ...........................G.....................G Ptroglodytes
484189: ..................G............T.................G Pabelli
```

```
                                 LINE1
        ------------------------------------------------->
                 111019    111029    111039    111049    111059
111010: CACAGCAAAGGAGTGAAGAATTCACAATCAACAGAGAGATAAGATGATCT  Hsapiens
430714: ..................................................  Ptroglodytes
484239: ...............................................C..  Pabelli

                                 LINE1
        ------------------------------------------------->
                 111069    111079    111089    111099    111109
111060: ACCAAATGAGAAAAAGTTTTTGTAAACTATATCGCTGATAAAGTATTCTT  Hsapiens
430764: ....G............A....A...........................  Ptroglodytes
484289: ........G.............A..........T....G...........  Pabelli

                                 LINE1
        ------------------------------------------------->
                 111119    111129    111139    111149    111159
111110: CTTCAAAATGTAAAGGGATCTGATAAAACTCAACAGCAAAAAAAAAAAAA  Hsapiens
430814: .....................................-------......  Ptroglodytes
484339: .........A.............A..........................  Pabelli

                                 LINE1
        ------------------------------------------------->
                 111169    111179    111189    111199    111209
111160: AAAAAAAACTTTTAAAATGTACAAAGAACCTGAATAGACATTTTTCAAAA  Hsapiens
430857: ....................G.............................  Ptroglodytes
484389: ...................GG.............................  Pabelli

                                 LINE1
        ------------------------------------------------->
                 111219    111229    111239    111249    111259
111210: GAAGACATACGAGTGGCCAGCAGTTACATAGAAAAATACTTAACATCACT  Hsapiens
430907: ..................................................  Ptroglodytes
484439: ............A.......T....................---......  Pabelli

                                 LINE1
        ------------------------------------------------->
                 111269    111279    111289    111299    111309
111260: AATCCTCAGGGAAATGCAAATCAGAAACACAATGAGATATCGTCTTACAC  Hsapiens
430957: ................................G.....G..A........  Ptroglodytes
484486: ..............C.....................G....A........  Pabelli

                                 LINE1
        ------------------------------------------------->
                 111319    111329    111339    111349    111359
111310: CCGATAGGATGGCTATTATCAAAGAGTCAAAAGATAACAAGTGTTGTTGA  Hsapiens
431007: ............T.................................G...  Ptroglodytes
484536: ..A.................C.........................G...  Pabelli

                                 LINE1
        ------------------------------------------------->
                 111369    111379    111389    111399    111409
111360: GGAGCTGGAGAAAAGGGAACCCTGGTACACTGCTGGTGGGAATAAAATTT  Hsapiens
431057: ................................T.................  Ptroglodytes
484586: ...T...........................AT..T..............  Pabelli
```

```
                                 LINE1
         ---------------------------------------------------->
                111419    111429    111439    111449    111459
111410: GTACAGCCATTTAGAAAACCTTATGGAGATGCCTCAGAAAATTAACAACA  Hsapiens
431107: ..................................................  Ptroglodytes
484636: ..........G..................................G.C..  Pabelli

                                 LINE1
         ---------------------------------------------------->
                111469    111479    111489    111499    111509
111460: GAACTAACATATGATTCAGCAATCCCACTTCTTGGAATACATCCAAAGGG  Hsapiens
431157: ..................................................  Ptroglodytes
484686: .........................A.............T..........  Pabelli

                                 LINE1
         ---------------------------------------------------->
                111519    111529    111539    111549    111559
111510: TATGAAATACGCATGTCAAAGAATTATTTGTATCTCATGTTCATTACAGC  Hsapiens
431207: .........T........................................  Ptroglodytes
484736: ..........AT......................................  Pabelli

                                 LINE1
         ---------------------------------------------------->
                111569    111579    111589    111599    111609
111560: ATTATTCACAATAGCCAAGACATGAAATCAGCCTGTGTCCACCAACAAAT  Hsapiens
431257: ..............................................G...  Ptroglodytes
484786: ..............................................G...  Pabelli

                                 LINE1
         ---------------------------------------------------->
                111619    111629    111639    111649    111659
111610: GAATAAATAAAGAAAATATGATATATATACACAATGAAATACTATTCCAC  Hsapiens
431307: ..................................A...............  Ptroglodytes
484836: ..................................................  Pabelli

                                 LINE1
         ---------------------------------------------------->
                111669    111679    111689    111699    111709
111660: CATAAAAAAGACAGTTCTGTCATTTGCAACAACATGGATGAACCTGGAGG  Hsapiens
431357: ..................................................  Ptroglodytes
484886: ..................................................  Pabelli

                                 LINE1
         ---------------------------------------------------->
                111719    111729    111739    111749    111759
111710: ATATTATGTTAAGTGAAGTAAGCTAGACACAGAAAGACTAATACTACACA  Hsapiens
431407: ..................................................  Ptroglodytes
484936: .....................................T............  Pabelli

                                 LINE1
         ---------------------------------------------------->
                111769    111779    111789    111799    111809
111760: ACCTCAATTATATGTGCAATCTAAAAATGTTGAACTCATAGAAACAGATA  Hsapiens
431457: ..................................................  Ptroglodytes
484986: ..........C.....G.................T...............  Pabelli
```

```
                                LINE1
        ------------------------------------------------->
            111819    111829    111839    111849    111859
111810: GTAGAATGCTGGCTACAAGAGGCTGGTAGGGTGGAAGAGTTGGGAAGATG  Hsapiens
431507: .....................................G.........G..  Ptroglodytes
485036: ........G..........................G.G............  Pabelli

                                LINE1
        ------------------------------------------------->
            111869    111879    111889    111899    111909
111860: TTGGTAAAGGATATGAAATTTCAGTTAAACAAGAGGAATAAGTTTAAGGT  Hsapiens
431557: .................................................C  Ptroglodytes
485086: ...C........................................C....C  Pabelli

                                LINE1
        ------------------------------------------------->
            111919    111929    111939    111949    111959
111910: ATCTTATCATGACTATATTTAATACCAATATTGTATACTTGAAAATTGCT  Hsapiens
431607: ..................................................  Ptroglodytes
485136: ..T.................C............C................  Pabelli

                                LINE1
        ------------------------------------------------->
            111969    111978    111988    111998    112008
111960: GAGAGAGGAAATTTTAA-TGTTCTGGTTCTAAAAAAGAATAATTATATGA  Hsapiens
431657: .........G.......G................................  Ptroglodytes
485186: .........G.......G..........A.....................  Pabelli

                                LINE1
        ------------------------------------------------->
            112018    112028    112038    112048    112058
112009: GGTAATGGATATGCTAATTAGCTTAATTTAGCCATTCAACAACGTGTACA  Hsapiens
431707: ..................................................  Ptroglodytes
485236: ..........................G.......................  Pabelli

                                LINE1
        ------------------------------------------------->
            112068    112078    112088    112098    112108
112059: TATATCAAAACATTATGTTACATACCATTAATAAATACAATTTTTACTTA  Hsapiens
431757: ............................C.....................  Ptroglodytes
485286: ...................CT...................C.........  Pabelli

                 LINE1
        ---------------->
            112118    112128    112138    112148    112158
112109: CCATTTTTTAAAAAAATTAACTGTAAAACAGTTTCTGAAAACAATTTAAG  Hsapiens
431807: A........T.....G..................................  Ptroglodytes
449149:    ......T........................................  Ggorilla
485336: .........T...................................C....  Pabelli
373316:                                       ............  Nleucogenys
```

```
                   112168    112178    112188
                        :         :         :
112159: AACTTCATATGCCTACTACCTCAATTTGACAA------------------ Hsapiens
431857: ...............................C------------------ Ptroglodytes
449196: ...............................C------------------ Ggorilla
485386: ...............................C------------------ Pabelli
373328: ...............................CAACAGCATTTTGTCAATT Nleucogenys


112191: -------------------------------------------------- Hsapiens
431889: -------------------------------------------------- Ptroglodytes
449228: -------------------------------------------------- Ggorilla
485418: -------------------------------------------------- Pabelli
373378: TTATTTGTCCACATCACACTAATCCTATGTATTTAAGAATAGTTAGTATC Nleucogenys


                                             112198    112208
                                                  :         :
112191: --------------------------------AAGTTAATATATTGTCAT Hsapiens
431889: --------------------------------.................. Ptroglodytes
449228: --------------------------------.................. Ggorilla
485418: --------------------------------......C.G...A..... Pabelli
373428: ATTTACAATAACAAAGACATGGAACCAACACA.................. Nleucogenys
230554:                                      ..C.C.G.A.... Mmulatta
240416:                                      ..C.C.G...... Panubis


                                                 LINE1
                                         ---------------->
                   112218    112228    112238    112248    112258
                        :         :         :         :         :
112209: TCCTATGTTAAAATATAATTCATGACAGATATAATGTTATAGGTGCTTTT Hsapiens
431907: ...................A.............................. Ptroglodytes
449246: ...................A.............................. Ggorilla
485436: ...................A.............................. Pabelli
373478: ...................A.............................. Nleucogenys
230567: .AT...TC.C...A..........                           Mmulatta
240429: .AT...TC.C...A..........                           Panubis


                                 LINE1
        ------------------------------------------------->
                   112268    112278    112288    112297    112307
                        :         :         :         :         :
112259: AAAATGTTTTACCTAAGTCATATTTATACATTC-TTTTTCGTTTGTTTTC Hsapiens
431957: .................................-................ Ptroglodytes
449296: .................................-......A......... Ggorilla
485486: ............AC....G.......C......T......A..C...... Pabelli
373528: ............A.....G.......C......-......A......... Nleucogenys


                                 LINE1
        ------------------------------------------------->
                   112317    112327    112337    112347    112357
                        :         :         :         :         :
112308: CTGTTCCTTGCGATTATGTCTAGAGATTTATTCTTCTTAACAAATGATTA Hsapiens
432006: ...........A.......................T.............. Ptroglodytes
449345: .................................................. Ggorilla
485536: .........C.T....C..................T.C............ Pabelli
373577: ...........TG....A....C............T............G. Nleucogenys
```

```
                                       LINE1
          ------------------------------------------------------------------>
                    112367    112377    112387    112397    112407
                         :         :         :         :         :
112358: TCCAGTTCATTTACATTCACTGCTGTAGAATACTCAGCTGTATGAAACTA  Hsapiens
432056: ........G.....................................C...  Ptroglodytes
449395: ..............................................C...  Ggorilla
485586: ..............................................C...  Pabelli
373627: .............T................................C...  Nleucogenys

                                       LINE1
          ------------------------------------------------------------------>
                    112417    112427    112437    112447    112457
                         :         :         :         :         :
112408: GCTTAATTTTTTCCTTATTCTTCTTTTGATATATATTTAAGTTGTTTCTA  Hsapiens
432106: ..................................................  Ptroglodytes
449445: ..................................................  Ggorilla
485636: ..................................................  Pabelli
373677: .............T...---..T........C..................  Nleucogenys

                                       LINE1
          ------------------------------------------------------------------>
                    112467    112477    112487    112497    112507
                         :         :         :         :         :
112458: ATTCTTCACCATTAACAAGAATGCTGCAATTCATGCCTTTGTATGTGTCT  Hsapiens
432156: .........T....G...................................  Ptroglodytes
449495: ..............G...................................  Ggorilla
485686: .............GG...................................  Pabelli
373724: .............GG...................................  Nleucogenys

                                       LINE1
          ------------------------------------------------------------------>
                    112516    112526    112536    112546    112556
                         :         :         :         :         :
112508: TGTGCAAC-ATAGTACATGGGTTCATCTAGGCTATATATAGAGAGAAGGA  Hsapiens
432206: ....A...-..................C..........A........T..  Ptroglodytes
449545: ....A...-......................................A..  Ggorilla
485736: ....A...A...............GC............G........A..  Pabelli
373774: ....A...-...........................---.T......A..  Nleucogenys

          LINE1                                Alu
          ------>               ---------------------------------------->
                              112567    112577    112587    112597
                                   :         :         :         :
112557: GAGAGG---------TTTTTTTGTTTTTTTTTTGACAGAGTCTCACTCAG  Hsapiens
432255: ......-------TT.......T...........................  Ptroglodytes
449594: .....TTTCTCTTTT.......T...........................  Ggorilla
485786: .....C-------CT..------...........................  Pabelli
373820: .....T-------TT....G..............................  Nleucogenys

                                        Alu
          ------------------------------------------------------------------>
                    112607    112617    112627    112637    112647
                         :         :         :         :         :
112598: TCACTCAGGCTGGAGTGCAGTGGTAAAATTTCAGCTCACTGCAACCTCTA  Hsapiens
432298: ....C........................C....................  Ptroglodytes
449644: ....C........................C....................  Ggorilla
485823: ....C........................C....................  Pabelli
373863: ....C........................C.............T......  Nleucogenys
```

```
                                   Alu
         ------------------------------------------------------->
                112657    112667    112677    112687    112697
112648: CCTCCTGGCTTCAAGAAATCCCCCCACCTCAGCCTCCCAAGTAGCTAGGA  Hsapiens
432348: ......................T...........................  Ptroglodytes
449694: ..................................................  Ggorilla
485873: .....C.....TG........T............................  Pabelli
373913: .....C........AC...G.-.................G..........  Nleucogenys

                            Alu                        Alu
         ------------------------------------> --------->
                112707    112717              112733    112743
112698: TTACATGAACCACCACACCAGGCTAATT----TTTATTTATTTATTTTGT  Hsapiens
432398: ............................TTTA..................  Ptroglodytes
449744: ............................TTTT..................  Ggorilla
485923: ........G...................TTTA..................  Pabelli
373962: .......T....................TTTA..................  Nleucogenys

                                   Alu
         ------------------------------------------------------->
                112752    112762    112772    112782    112792
112744: TT-ATTTTATTTTATTTGAAATGGAGTCTCGCTCCGTCCCCAGGCTGGAG  Hsapiens
432448: ..T...............................T...............  Ptroglodytes
449794: ..T...............................T...............  Ggorilla
485973: ..-----.C...........G.........T...G...............  Pabelli
374012: ..T...........................T...T...............  Nleucogenys

                                   Alu
         ------------------------------------------------------->
                112802    112812    112822    112832    112842
112793: TGCAGTGGCTCAATCTCAGCTCACTGCAACCTCTGCCTCCCTGGTTAGAG  Hsapiens
432498: ..................................................  Ptroglodytes
449844: ..................................................  Ggorilla
486018: .........................................CA.......  Pabelli
374062: ................................T.................  Nleucogenys

                                   Alu
         ------------------------------------------------------->
                112852    112862    112872    112882    112892
112843: CGATTCTTCTGCCCCTCAGCCTCCCGAGTAGCTGGGATTATAATTGCCTG  Hsapiens
432548: ........................T....................T....  Ptroglodytes
449894: .........................A........................  Ggorilla
486068: ..................................................  Pabelli
374112: .A.......................................T...-----  Nleucogenys

                                   Alu
         ------------------------------------------------------->
                112902    112912    112922    112932    112942
112893: CCACCACGCCCGGCTGATTTTTGTATATTTAGTAGACACAGAGTTACACC  Hsapiens
432598: ..........T....A.......................G..........  Ptroglodytes
449944: ..........T....A.......................G..........  Ggorilla
486118: ..........TA...A......................AG.G........  Pabelli
374157: --.GT.TTATTAAT.TT.......T.T.T...TT.T..G..G....CT.G.T  Nleucogenys
```

```
                                  Alu
        ------------------------------------------------->
            112952              112957    112967
112943: ATATTGGCCAGGCT---------------TATCTTGAACTCCTG------ Hsapiens
432648: ..............---------------...............------ Ptroglodytes
449994: ..............---------------..........C....------ Ggorilla
486168: .......T......---------------...............------ Pabelli
374205: C.G.CAC.......GGAGCGCAGTGGCACG......GCTCA...CAAGCT Nleucogenys

                                  Alu
        ------------------------------------------------->
            112979              112989    112999    113008
112972: --ACCTTCTG----------ATCCACCTGCCTCGGCATCCCAAAGT-GCT Hsapiens
432677: --........----------..........................-... Ptroglodytes
450023: --........----------..........................-... Ggorilla
486197: --........----------.............A............-... Pabelli
374255: CCG...C.C.GGTTCACGCC..T.T........A..C..T.CG...A... Nleucogenys

                         Alu                       Alu
        -------------------------------->----------------->
            113018    113028    113038    113045    113055
113009: GGCATTACAGGCGTGAGCCACCATGCCTGGCCCTA---TTTTTATTTTTT Hsapiens
432714: ...................................TTT.....T...... Ptroglodytes
450060: ....C...................A..........---............ Ggorilla
486234: ..G..................T.............---............ Pabelli
374305: ..G.C........CCC.......C...C...-----------..A..... Nleucogenys

                                  Alu
        ------------------------------------------------->
            113065    113075    113085    113095    113105
113056: TGTAATCTTAGTAGAGACAGGGTTTCACCATTTTACTCAGGCAGGTCTTG Hsapiens
432764: ......T........................................... Ptroglodytes
450107: ......T........................................... Ggorilla
486281: G.....T.C.........G............G..........T.....C. Pabelli
374344: ....T.T...........G............------------.....C. Nleucogenys

                                  Alu
        ------------------------------------------------->
            113115    113125    113135    113145    113155
113106: AACTCCTCACCTCAAATGATCTGCTCCCCCTCAATCTTCTAAATTGCTGG Hsapiens
432814: .................................................. Ptroglodytes
450157: .................................................. Ggorilla
486331: .......G................-.........C............... Pabelli
374382: .TT....G.....--G.....C..-..G...TGGC..C.C...G...... Nleucogenys

                        Alu                     LINE1
        ----------------------------->------------------->
            113165    113175    113185    113195    113205
113156: GATTACAGGCATGAGCCACCGCACCTAGCCTAGGGTATATATTTAAACAT Hsapiens
432864: ....................A.....G....................... Ptroglodytes
450207: ....................C.....G....................... Ggorilla
486380: ..........................G....................... Pabelli
374429: ..........G...........G..CG...GA.                  Nleucogenys
374781:                                   ...C...........C Nleucogenys
```

```
                              LINE1
        -------------------------------------------------->
            113215    113225    113235    113245    113255
113206: AGAATTGCTAAAATAACTAGATATGGCCAAGTTTTTCCAAAATAGTTGTA  Hsapiens
432914: .........................A.......................G  Ptroglodytes
450257: ............................-....................G  Ggorilla
486430: ........................T........................G  Pabelli
374797: ........................T......................T.G  Nleucogenys
```

```
                              LINE1
        -------------------------------------------------->
            113265    113275    113285    113295    113305
113256: CCAGTAAACATTTGCAACAGCATTTTTGTCATTTTTATTTGTCCACATCT  Hsapiens
432964: ............................................G.....  Ptroglodytes
450306: ..................................................  Ggorilla
486480: .............T................CA.................A  Pabelli
374847: ........G....T.................A.................A  Nleucogenys
```

```
              LINE1                         LINE1
        ----------------->            ------------------->
            113315    113325    113335    113345    113355
113306: CACTAATCCTATGTATTTAAGAATAGTTAGTATCATTTACAATAACAAAG  Hsapiens
433014: ..................................................  Ptroglodytes
450356: .......................................G..........  Ggorilla
486530: .................................A................  Pabelli
374897: ..................................................  Nleucogenys
```

```
                              LINE1
        -------------------------------------------------->
            113365    113375    113385    113395    113405
113356: ACATGGAACCAACACAAATGCCCATCAATGACAGACTAGATAAAGAAAAT  Hsapiens
433064: ....................T.............................  Ptroglodytes
450406: .......................................G..........  Ggorilla
486580: ..................C....G..........................  Pabelli
374947: ................................................G.  Nleucogenys
```

```
       LINE1                       LTR
        -->------------------------------------------------>
            113415    113425    113435    113445    113455
113406: GTGGAGCTCTCAGGCTCCGCCTAATTTATTGGTTTATAAGCTCTTTGTTC  Hsapiens
433114: ..................................................  Ptroglodytes
450456: ..................................................  Ggorilla
486630: ....................................C.............  Pabelli
374997: ................T.T.................C...T.........  Nleucogenys
```

```
                              LTR
        -------------------------------------------------->
            113465    113475    113485    113495    113505
113456: CTAAGGCAGATGAGAGCAGGAGGAAGGGATGAGGAAAAGGATTAATCAGT  Hsapiens
433164: ..................................................  Ptroglodytes
450506: ..................................................  Ggorilla
486680: ..................A..........C....................  Pabelli
375047: ................G.................................  Nleucogenys
```

```
                                 LTR
        --------------------------------------------------->
                 113515    113525    113535    113545    113555
                          :         :         :         :         :
113506: GAAAGAGAACTCATGAGTCATTCAATAAGATGTAGAGCAGTAGCGGTTTC Hsapiens
433214: .........................................G........ Ptroglodytes
450556: ................A........................G......C. Ggorilla
486730: ........G................................G........ Pabelli
375097: ..................................T......G.TA..... Nleucogenys

                                 LTR
        --------------------------------------------------->
                 113565    113575    113585    113595    113605
                          :         :         :         :         :
113556: TGGGAATTTCTTTGAGCAAAGGCATGTGTCTAAACTACGTAAGATCTTTA Hsapiens
433264: ..T.G.................................T........... Ptroglodytes
450606: ..T.G.................................T........... Ggorilla
486780: ..T...................................T........... Pabelli
375147: .TT...................................T........... Nleucogenys

                                 LTR
        --------------------------------------------------->
                 113615    113625    113635    113645    113655
                          :         :         :         :         :
113606: ACTTATAGGGACTGAAATGGGCGGGAGCGGGTTTCAGGAGGAGCCAAGAC Hsapiens
433314: .....................T...........................T Ptroglodytes
450656: .....................T...........................T Ggorilla
486830: .................C...T...........................T Pabelli
375197: ........A............T.....T.....................T Nleucogenys

                                 LTR
        --------------------------------------------------->
                 113665    113675    113685    113695    113705
                          :         :         :         :         :
113656: GTTTGATTATACTCCCCTGCTTCAAGGGACTGTTATCTCCCTGAGCAACC Hsapiens
433364: .................................................. Ptroglodytes
450706: .................................................. Ggorilla
486880: ........G.......A......G.T...G.................... Pabelli
375247: .........................-...G.................... Nleucogenys

                                 LTR
        --------------------------------------------------->
                 113715    113725    113735    113745    113755
                          :         :         :         :         :
113706: TGTGGAATACCGCTGAGCTGTTATGCTCTCGGGGCATAAAGACATGAAGG Hsapiens
433414: .................................................. Ptroglodytes
450756: .........................T........................ Ggorilla
486930: ...........A..................A.............C..... Pabelli
375296: ..............................A................... Nleucogenys

                                 LTR
        --------------------------------------------------->
                 113764    113774    113784    113794    113804
                          :         :         :         :         :
113756: C-ATAAGGAGACTTTTCTCCTCAGAGGCTGCCCATGGCTACGCATGGGTG Hsapiens
433464: .A................................................ Ptroglodytes
450806: .A................................................ Ggorilla
486980: .A.......................................C........ Pabelli
375346: .A.........................G.............C........ Nleucogenys
```

```
                         LTR
        -------------------------------------------------->
            113814    113824    113834    113844    113854
113805: TCTCACACAGGGGAGACCAACTCAACTGGCACCCCAGAAACTCTCTTTCC  Hsapiens
433514: ...T..............................................  Ptroglodytes
450856: ..................................................  Ggorilla
487030: ..................T...............................  Pabelli
375396: .......A..........................................  Nleucogenys

         LTR
        --->
            113864    113874    113884    113894    113904
113855: CACAAGAAGTACATGGTATACTTGCATAGCCAAAGAAATTAAAATAGGCC  Hsapiens
433564: ..................................................  Ptroglodytes
450906: ..................................................  Ggorilla
487080: ..........G.......................A..........-....  Pabelli
375446: ..................................A...............  Nleucogenys

            113914    113924    113934    113944    113954
113905: ATTGAAGACAGAGAAAAACAATGGCTAGAAAAGGCAGTAGGAATAAAAGA  Hsapiens
433614: ..................................................  Ptroglodytes
450956: ............................................G.....  Ggorilla
487129: ..................................................  Pabelli
375496: .............-....................................  Nleucogenys

            113964    113974    113984    113994    114004
113955: GAAGATCATGCTTCCTGGCTTCATGGCATGAGAATGGGGGAGTGAGTAGA  Hsapiens
433664: ..................................................  Ptroglodytes
451006: ..................................................  Ggorilla
487179: ..........................T.......C...A...........  Pabelli
375545: .....C....................T.......................  Nleucogenys

            114014    114024    114034    114044    114054
114005: CATCCATGAGAAAAGATCAAGGAGCATGCAGCCATCGGGGAAAATCCATA  Hsapiens
433714: ..C...................G...........................  Ptroglodytes
451056: ..C...................G...........................  Ggorilla
487229: T.C...CC..............G...........................  Pabelli
375595: T.C...C...............G............A..............  Nleucogenys

            114064    114074    114084    114094    114104
114055: GTGCAGATAAAGCAAGGAGGAAGAAGAAGGACAGTTCTAGTAAAAGGGAG  Hsapiens
433764: ...................---............................  Ptroglodytes
451106: ...................---............................  Ggorilla
487279: ...................---............................  Pabelli
375645: ...................---.......................-.A.T  Nleucogenys

            114114    114124    114134    114144    114154
114105: AACATCAATATAGGATGTTTCTTAGCAATAGAAAAAGAAGGCCAAGAGGA  Hsapiens
433811: ...........G......................................  Ptroglodytes
451153: ...............C..................................  Ggorilla
487326: ......................C.....C.....................  Pabelli
375691: TC..........................C...................A.  Nleucogenys
```

```
            114164    114174    114184    114194    114204
114155: ATTAGGGAGAGAGTTATAAGAGATCAGCAAGGGGACAGGGTTAGATTTGG  Hsapiens
433861: ..................................................  Ptroglodytes
451203: ..C...............................................  Ggorilla
487376: ..G..A....................................C.......  Pabelli
375741: ..C..............................................A  Nleucogenys

            114214    114224    114234    114244    114254
114205: TTTGGTTTGAAAGCATACAGTAAATATGATGTCTGTCCCTGGCAGTGTTG  Hsapiens
433911: ............A....T..........................G......  Ptroglodytes
451253: ...........................................G......  Ggorilla
487426: ..................G........................G......  Pabelli
375791: .................T...........C.............G......  Nleucogenys

            114264    114274    114284    114294    114304
114255: GCAGAGTAGGAAGGAGGAAGGGAGGCAAGAGATAATATCATTTTCTCTGT  Hsapiens
433961: ..G...............................................  Ptroglodytes
451303: .AG.................A.............................  Ggorilla
487476: ................................C.................  Pabelli
375841: ..T...............................................  Nleucogenys
233202:                                         .C..AC..A.  Mmulatta
243061:                                         .C..AC..A.  Panubis

            114314    114324    114334    114344    114354
114305: GCTCCAACTGTACTTACATATGAGACTATTTCCCTCTCTGCTTTTCAAAC  Hsapiens
434011: ............................................C.....  Ptroglodytes
451353: ....................G.............................  Ggorilla
487526: ..................................................  Pabelli
375891: ..........................G......G................  Nleucogenys
233212: .TAAG.......G..........................T..........  Mmulatta
243071: .TAAG.......G..........................T..........  Panubis

            114364    114374    114383    114393    114403
114355: CTTACTGGAGTTGTTTTCCC-TCATGAAAACCAAGAAAGGAAAGCTAGTT  Hsapiens
434061: ....................-.............................  Ptroglodytes
451403: ....................-.............................  Ggorilla
487576: ....................-..........T.................C  Pabelli
375941: ...T................-.....T....T..............T...  Nleucogenys
233262: ....................C..........T..AC..............  Mmulatta
243121: ....................C..........T..AC..............  Panubis

            114413    114423    114433    114443    114453
114404: AGTCTTGTTCTGAGGTTGTTCAATGTATACATATCTATATCTGTAGACAG  Hsapiens
434110: ..................................................  Ptroglodytes
451452: .......................C..........................  Ggorilla
487625: .A......................A....GG...G...............  Pabelli
375990: .......C..............T.A.G.......................  Nleucogenys
233312: .A.........C...........AA......................G..  Mmulatta
243171: .A.........C...........AA......................G..  Panubis
```

```
                  114463     114473     114483     114493     114503
                       :          :          :          :          :
114454: AATCCTTGGGAATACAGTAATTGACATATATTCTGTTATTTGATGCTTGA  Hsapiens
434160: .-................................................  Ptroglodytes
451502: .-......C.........................................  Ggorilla
487675: .-................................................  Pabelli
376040: .-................................................  Nleucogenys
233362: .-.................G...G..........................  Mmulatta
243221: .-.................G...G..........................  Panubis

                  114513     114523     114533     114543     114553
                       :          :          :          :          :
114504: AAAATCTCCTCCACTAACCAGTTTCCCTATAGATTGCCACAAGCACATAA  Hsapiens
434209: ..........................T.......................  Ptroglodytes
451551: ..................................................  Ggorilla
487724: G.......A.........A..........C.C....G............G  Pabelli
376089: .....T..A.........A...............A.G......A.....G  Nleucogenys
233411: ...............GG.................A.G............G  Mmulatta
243270: ...............G..................AAG............G  Panubis

                  114562     114572     114581     114591     114601
                       :          :          :          :          :
114554: T-AAGAAACAATAAATAAAATGTTC-TCTTGACTTTGTTACTTAACAATG  Hsapiens
434259: .-.......................-....T...................  Ptroglodytes
451601: .-.......................-....T...................  Ggorilla
487774: .-.......................-....T.......A...........  Pabelli
376139: .-................-......-....T.A.....A......T....  Nleucogenys
233461: .T.......................-...AT.A.....A........CA.  Mmulatta
243320: .T.......................-...AT.A.....A........CA.  Panubis
177048:    ....GG..G.GC...G...CC.T...A.GAGC..C.G.CC.G...C.  Sboliviensis

                  114611     114621     114631     114641     114651
                       :          :          :          :          :
114602: CTGAGAAAACTTTACAGCCTTCATAAGGAAGTGAGGTCCAGGAAAATCTA  Hsapiens
434307: ..................................................  Ptroglodytes
451649: ..............................A...................  Ggorilla
487822: ..................................................  Pabelli
376186: ..................................................  Nleucogenys
233510: ..................................................  Mmulatta
243369: ..................................................  Panubis
177095: A..GC...T..CC..GC....T.CTGAAG.T.A.A.CTG..ACT....C.  Sboliviensis

                             114669     114679     114689
                                  :          :          :
114652: GGAGATATT--TCTTAACCAATCTATAAAGGCATTAGTAA----------  Hsapiens
434357: .........--.............................----------  Ptroglodytes
451699: .........--.............................----------  Ggorilla
487872: .........--......T......................----------  Pabelli
376236: .........--.............................----------  Nleucogenys
233560: ......G..--.............................----------  Mmulatta
243419: ......G..--.........................G..----------  Panubis
177145: A..ACAG..TG.T....TG..CTGG..GG.C..G.G.A..TGCTCCAAGG  Sboliviensis

                  114698     114703     114713     114723     114733
                       :          :          :          :          :
114690: -TGACAGGATATTT-----CCTGAAAGTGTAATTTCCCATTGAGGATTTG  Hsapiens
434395: -.............-----...............................  Ptroglodytes
451737: -.............-----...............................  Ggorilla
487910: -.A...........-----..............................A  Pabelli
376274: -....G........-----...............................  Nleucogenys
233598: -........G....-----...A.....................A.....  Mmulatta
243457: -........G....-----........                         Panubis
177195: T..G.....A.AACTGGAAT...GGG...........T......---..T  Sboliviensis
```

```
                        114751    114761    114771    114781
                             :         :         :         :
  114734: TTTTTAAT--TTCTGGATTCCTGGAGCCAATGAAGTTGGTGTATGTTTAT  Hsapiens
  434439: ........--........................................  Ptroglodytes
  451781: ........--........................................  Ggorilla
  487954: ........--.........G............G.................  Pabelli
  376318: ........--........................................  Nleucogenys
  233642: ........--.........-..................AC..........  Mmulatta
  177242: ....GG..AG.GT..TT......ACAT..G...G.G.AT..GG....C..  Sboliviensis

              114791    114801    114811    114821    114831
                   :         :         :         :         :
  114782: GAAATATCAAGAGACATAAGTTGGCAAGTGTTCATATGCAAAAACTTCTT  Hsapiens
  434487: ......C.................T.........................  Ptroglodytes
  451829: ......C.................T.........................  Ggorilla
  488002: ......C...........................C.........T.....  Pabelli
  376366: ......C...........................C...............  Nleucogenys
  233689: .....GC...........................................  Mmulatta
  243528:                               ....................  Panubis
  177292: ...T...                                             Sboliviensis

              114841    114851    114861    114871    114881
                   :         :         :         :         :
  114832: GGAATTTCTGAGTTCTCTGTGGCAATATATGACATCAGGATATGTCCAGT  Hsapiens
  434537: ......C...................................C.......  Ptroglodytes
  451879: ......C...................................C.......  Ggorilla
  488052: ......C...........................................  Pabelli
  376416: ......C........................................C..  Nleucogenys
  233739: ......C.............CA.........................C..  Mmulatta
  243548: ......C.............CA.........................CA.  Panubis

              114891    114901    114911    114921    114931
                   :         :         :         :         :
  114882: CTCACACACCAGGATATGTCCTTTCTAGCCTGTCTATCACATGCTAGGAG  Hsapiens
  434587: ........T...................................C.....  Ptroglodytes
  451929: ........T.........................................  Ggorilla
  488102: ........T........T.....................T....C.....  Pabelli
  376466: ........T............AG.....................C.....  Nleucogenys
  233789: G.......T.............A......T.CC........CA.C.....  Mmulatta
  243598: G.......T.............A......T.CC........CA.C.....  Panubis
 1076856:                                              .....  Sboliviensis

              114941    114948    114958    114968    114978
                   :         :         :         :         :
  114932: AACTATTTAGGAACAG---AAAAAAATGCCTGAAATGATTTCTCATTTGA  Hsapiens
  434637: T...T...........---..........................A....  Ptroglodytes
  451979: ....T...........---..........................A....  Ggorilla
  488152: ....T...........--A...............................  Pabelli
  376516: ...AT...........---...................C...........  Nleucogenys
  233839: C.G.T...........--A...............................  Mmulatta
  243648: C.G.T...........---...............................  Panubis
 1076861: C...T...........AAA..........TG..........T........  Sboliviensis

              114988    114998    115008    115018    115028
                   :         :         :         :         :
  114979: ACTCATCCAAGCTTTCTCTAAATTTAAGCAAACTCCTGGTCATTTTCAGT  Hsapiens
  434684: ..................................................  Ptroglodytes
  452026: ..................................................  Ggorilla
  488200: ............................................C.....  Pabelli
  376563: ....................C...........................A.  Nleucogenys
  233887: .T................................................  Mmulatta
  243695: .T...........................C....................  Panubis
 1076911: ...A.....C........------..........T.............A.  Sboliviensis
```

```
                 115038    115048    115056    115066    115076
                      :         :         :         :         :
  115029: TAGTACCTTTCCTTAAGTTCAA--CCTTCAGGGCAAACCTCCGTGCCTCA  Hsapiens
  434734: ....T.................--......A...................  Ptroglodytes
  452076: ....T.................--......A...................  Ggorilla
  488250: ......T...............--......C..........A.C......  Pabelli
  376613: ......................--......T..........A.C......  Nleucogenys
  233937: .............C..C...C.--......T.A........A.CAT....  Mmulatta
  243745: .............C......C.--......T.A........A.CAT....  Panubis
 1076955: A....T.....T.C........TCT.....TAA........A.CAT....  Sboliviensis

                 115086    115096    115106    115116    115126
                      :         :         :         :         :
  115077: GGACGTTTAGCCATAGTCTGAAATTCTCTTCCATAGATTGGTCCCCTGTA  Hsapiens
  434782: ....T.............................................  Ptroglodytes
  452124: ....T.............................................  Ggorilla
  488298: ....A.................................C.A.........  Pabelli
  376661: ....A.............................................  Nleucogenys
  233985: .A.GA....A..........................G.A...........  Mmulatta
  243793: .A.GA....A..........................G.A...........  Panubis
 1077005: ...GA..C.........................C........A....A..  Sboliviensis

                 115136    115146    115156    115166    115176
                      :         :         :         :         :
  115127: ACCCCGGTTTGTCTCAGCTTGTTATCCTGTTTTTTTCTTCCCTCCATTCC  Hsapiens
  434832: ..................................................  Ptroglodytes
  452174: ..................................................  Ggorilla
  488348: ..................................................  Pabelli
  376711: ...T.A.A..........................................  Nleucogenys
  234035: G....A....A..................C.....--.............  Mmulatta
  243843: G....A....A..................C.....--.............  Panubis
 1077055: .G...A...........T...C.T...........--.............  Sboliviensis

                 115186    115196    115206    115216    115226
                      :         :         :         :         :
  115177: CAGGATGAGCTTGTTGCTTCTGTCCTATGAGACATTAGATTCCTTTTCTT  Hsapiens
  434882: ..................................................  Ptroglodytes
  452224: ..................................................  Ggorilla
  488398: ..................................................  Pabelli
  376761: .................................G................  Nleucogenys
  234083: ....C.......C...........T.......TG...........C....  Mmulatta
  243891: ....C.......C...........T.......TG...........C....  Panubis
 1077103: ....C.......C..........T....-........T.......C....  Sboliviensis

                 115236    115246              115265  115273
                      :         :                   :       :
  115227: TGGTACCCGAGTAAATCCATCCTACTCCA-ATAGAGGAAGGTCCA--TTT  Hsapiens
  434932: .............................-...............--...  Ptroglodytes
  452274: .............................-...............--...  Ggorilla
  488448: ...........................T.-...............--...  Pabelli
  376811: ...........................A.-...............--...  Nleucogenys
  234133: ........AG.....C.....T..A....T...............--.G.  Mmulatta
  243941: ........AG.....C.....T..A....T...............--.G.  Panubis
 1077152: ........A......C...GA........T.C.......A.....TT...  Sboliviensis
```

```
             115283    115293    115303    115313    115323
                  :         :         :         :         :
 115274: TTGTCTTATAGCGCTGGATGCAGACTCAGCTGAGAAGACCATTATTCATT  Hsapiens
 434979: ..........................................G.......  Ptroglodytes
 452321: ..................................................  Ggorilla
 488495: ............C....C...........................A....  Pabelli
 376858: ............C.....................................  Nleucogenys
 234181: ........C...C...........T.........................  Mmulatta
 243989: ............C...........T.T.......................  Panubis
1077202: ...G........C......A..........A......C.....C......  Sboliviensis

             115333    115343    115353    115363
                  :         :         :         :
 115324: TTTGGAATTCTTTATCTCAGATATTTCCTCTTCTTTCTTTTTCTTCT---  Hsapiens
 435029: ...............................................---  Ptroglodytes
 452371: ............................G..................---  Ggorilla
 488545: .................GC.............G..............---  Pabelli
 376908: ................................G..............---  Nleucogenys
 234231: ............C......T............G..............---  Mmulatta
 244039: ............C......T............G..............---  Panubis
1077252: ...........GC...................A...-..........TCC  Sboliviensis

                                           115379    115388
                                                :         :
 115371: -----------------------ATCT--------TTGGA-TTTTTAGTC  Hsapiens
 435076: -----------------------....--------.....C.........  Ptroglodytes
 452418: -----------------------....--------.....C.........  Ggorilla
 488592: -----------------------....--------.....C......A..  Pabelli
 376955: -----------------------....--------.....C.........  Nleucogenys
 234278: -----------------------....--------.....C........T  Mmulatta
 244086: -----------------------....--------.....CC........  Panubis
1077301: ATGAGATCCGATAAACCCATTGG....CATGAGAC..A..C........T  Sboliviensis

             115394    115404    115414    115424    115434
                  :         :         :         :         :
 115389: C----ATCAACGCCCCATTAGTCTATTCCCCGACTTCAATCAGGGAACTT  Hsapiens
 435095: .----.................................G.....A.....  Ptroglodytes
 452437: .----.....T...........................G...........  Ggorilla
 488611: .----......A..T.G..............A....A...........G.  Pabelli
 376974: .----......A.........A.........A....A..........T..  Nleucogenys
 234297: .----......A...................A..C.A.........C...  Mmulatta
 244105: -----......A..T................A..C.A.........C...  Panubis
1077351: .GTAAG..T.TT......A............A....A..........T..  Sboliviensis

             115444    115454    115464    115474    115484
                  :         :         :         :         :
 115435: ATACCTCTTAAACTCATTCAGAGACTCAAAACATATATATTGATACAGGA  Hsapiens
 435141: .......C.........................................C  Ptroglodytes
 452483: .......C.........................................C  Ggorilla
 488657: .......CC..T..............T.......................  Pabelli
 377020: ....T..CC..T...................T..................  Nleucogenys
 234343: .C.....CC..T............T...........G.............  Mmulatta
 244150: .......CC..T............T...........G.............  Panubis
1077401: ....T..CC..T............T.TG........G...CA........  Sboliviensis
```

```
             115494    115504    115514    115524    115534
 115485: GACCTAAGAAGAGCATGTCTTGGGGGTTGAGGAAACAGGCAGGTGAGAAA   Hsapiens
 435191: .........................C.CAC....................   Ptroglodytes
 452533: .........................C.CAC....................   Ggorilla
 488707: .............................G....................   Pabelli
 377070: ..........C.A...............TG....................   Nleucogenys
 234393: ..........C............A.....G....-...............   Mmulatta
 244200: ..........C............A.....G....-...............   Panubis
1077451: ..T.......C....C.............G.........A..........   Sboliviensis

             115544    115554    115564    115574    115584
 115535: TTTCCAGATTGGAAACACAGCTTCCTTTCTCCCATCCAGCCCCTACTTTC   Hsapiens
 435241: C..T.......A.....T..A.............................   Ptroglodytes
 452583: C..T.......A.....T.............T...A..............   Ggorilla
 488757: C..T.............................G................   Pabelli
 377120: C..T..............................................   Nleucogenys
 234442: CG.T......A......................G..............C.   Mmulatta
 244249: CG.T......A......................G..............C.   Panubis
1077501: C..T.T..C.............C..........T....T...T...G.C.   Sboliviensis

             115594    115604    115614    115624    115633
 115585: AGCCTATGTGTTTCTGGCACCTTGTTGTAGATAAATCTCCCT-TGACTTT   Hsapiens
 435291: ..........................................-.......   Ptroglodytes
 452633: ..........................................-.......   Ggorilla
 488807: ..........C......A..............G.........-.......   Pabelli
 377170: ................................G.........-...T...   Nleucogenys
 234492: .T.......A.......A..............G.........-...T...   Mmulatta
 244299: .T.......A.......A..............G.........-...T...   Panubis
1077551: .T...C...........A.A............G.....G...A.......   Sboliviensis

                                                        LINE1
                                                           >
             115643    115653    115663    115673    115683
 115634: GTGATGTGCTGAGAAAACAAACTCACGGCTGGTGTTAAAAAGGGCCCATG   Hsapiens
 435340: ..........................................A.......   Ptroglodytes
 452682: .........................T........................   Ggorilla
 488856: ..........................A.......................   Pabelli
 377219: ..........A.........................T.............   Nleucogenys
 234541: ..........................T.........G.............   Mmulatta
 244348: ..........................T...............A.......   Panubis
 438199:                                     .....AT..A....   Cjacchus
1077601: ..............................CC..........T..A....   Sboliviensis

                                 LINE1
         ------------------------------------------------->
             115693    115703    115713    115723    115733
 115684: ACAATACCAAGTGTTGGGGAGAATGTGGAGAAATCAGAACTCTATTCACG   Hsapiens
 435390: ..................................................   Ptroglodytes
 452732: .................................................A   Ggorilla
 488906: ......T..............G............................   Pabelli
 377269: .....................G...........................-   Nleucogenys
 234591: .....................G...........................A   Mmulatta
 244398: .....................G..........................T.   Panubis
 438213: ......A...........A..G.C.....AT.......G...........   Cjacchus
1077651: ......A.........A.A..G.......AT.................T.   Sboliviensis
```

```
                                LINE1
         ------------------------------------------------->
              115743    115753    115763    115773    115783
 115734: GTCGGTTGGAATGCACACTTGTGCAGAATTCTATGGAGAAGAGTCTGGCA  Hsapiens
 435440: ..................................................  Ptroglodytes
 452782: ..................................................  Ggorilla
 488956: ................................G.................  Pabelli
 377318: ....A.............................................  Nleucogenys
 234641: ..A............T..................................  Mmulatta
 244448: ..A....................T..........................  Panubis
 438263: ..TC..G..........A........C..-.......A............  Cjacchus
1077701: ...A..........GT.A........CG.-.......A............  Sboliviensis

                                LINE1
         ------------------------------------------------->
              115793    115803    115813    115823    115833
 115784: TTTCCTCAAAATGTTAACCTGGATTTACCATATGACCCAGCGATTTCATT  Hsapiens
 435490: ....................A.............................  Ptroglodytes
 452832: ....................A.............................  Ggorilla
 489006: .......................G.........A......T.........  Pabelli
 377368: ....T..................G.......G.........C........  Nleucogenys
 234691: ....................A..G..................G.......  Mmulatta
 244498: ...T................A..G..................G.......  Panubis
 438312: .......G...CA.......A..G.............G..T.........  Cjacchus
1077750: .......G....A.......AA.G......C......G..T.........  Sboliviensis

                                LINE1
         ------------------------------------------------->
              115843    115853    115863    115873
 115834: CATAGGTTTATACTCAAAAGAAATGAAGAAATATGCCATGCAAAAAAA--  Hsapiens
 435540: ...........................A.C..................--  Ptroglodytes
 452882: ...........................A.C..................--  Ggorilla
 489056: .............................C..................--  Pabelli
 377418: .............................C..G...T......G....--  Nleucogenys
 234741: .............................C..................AA  Mmulatta
 244548: T............................C..................AA  Panubis
 438362: .......A.....CG..............C..........G....T.T--  Cjacchus
1077800: ..C....A.....C...............C..........GGG.....--  Sboliviensis

                                LINE1
         ------------------------------------------------->
              115889    115899    115909    115919    115929
 115882: --TGTACATGAAAGGTCACAACATCATTATTCATAATAGTAAAAGGATGG  Hsapiens
 435588: --........C......................................T  Ptroglodytes
 452930: --................................................  Ggorilla
 489104: --................................................  Pabelli
 377466: --................G.G.......................A.....  Nleucogenys
 234791: AA...T............G.G......C................A....A  Mmulatta
 244598: AA...T..C.........G.G......C................A.....  Panubis
 438410: -AC...............G.G.......-..........C....A...A.  Cjacchus
1077848: -A.A.....A........G.G.......-.C..C.....C....A...A.  Sboliviensis
```

LINE1

```
                115939    115949    115959    115969    115979
 115930: AAACAACACAAATGTCCATCAACTTATGATTAAAGAAAATCTGGTCTATT  Hsapiens
 435636: ..................................................  Ptroglodytes
 452978: ..................................................  Ggorilla
 489152: ......................G......A..........G.........  Pabelli
 377514: ......................G...C..A....C.....A...C.....  Nleucogenys
 234841: .....G......................CA.G........G.........  Mmulatta
 244648: .....G..............T.G......AC.........G.........  Panubis
 438458: ..........................C..A..........G..T......  Cjacchus
1077896: ..................G..........A..........G..C......  Sboliviensis
```

LINE1

```
                115989    115999    116009    116019    116029
 115980: CATAGAATGGAATATTATTCGACCACAAAAAGGAATGATGTACTGATCCA  Hsapiens
 435686: .................................................G  Ptroglodytes
 453028: .................................................G  Ggorilla
 489202: ...GC...............AG...................G........  Pabelli
 377564: ....C...............AG...................G........  Nleucogenys
 234891: ....CC...............G...................G........  Mmulatta
 244698: ....CC...............G................C..G........  Panubis
 438508: ...TC.........G..----G..CA..............C......TTG  Cjacchus
1077946: ...TC............----G.............C...........T..  Sboliviensis
```

LINE1

```
                116039    116049    116059    116069
 116030: TGCAATGATGTGGACAAACCATGAAAATAACACTAGATTAAAGAAG----  Hsapiens
 435736: .....C............T.T........................----  Ptroglodytes
 453078: .....C..............T.........................----  Ggorilla
 489252: .....CA.CA..........T.........T.......G.......----  Pabelli
 377614: ........CA....G.....T.........T.......G.......----  Nleucogenys
 234941: .....G..C...........T...C.....T.......G.......----  Mmulatta
 244748: .....G..CA..........T...C.....T.......G.......----  Panubis
 438554: ..TG.CA.CA.A..TG...TT.........TT..G.-.G.......AGCC  Cjacchus
1077992: ..TG.CA.CA....TG...AT.........T...G.-.G.......AGCC  Sboliviensis
```

LINE1

```
                116082              116100    116110    116120
 116076: ---CCAGTCACAAAAGGAC--TTACTGTATGATTCCATTTACCTGAAATG  Hsapiens
 435782: ---................--G....................A.......  Ptroglodytes
 453124: ---................--.....................A.......  Ggorilla
 489298: ---.........G......AAA.............T.....TA.......  Pabelli
 377660: ---...A.....G......AAA.............TG....TA.......  Nleucogenys
 234987: ---.........G......AAA.............T.....TA.......  Mmulatta
 244794: ---.........G......AAA.............T.....TA.......  Panubis
 438603: ---..CA.TG.........AAG...................TA.......  Cjacchus
1078041: CCC..CA.A..........AA.............T....C.TA.......  Sboliviensis
```

```
                                 LINE1
          ---------------------------------------------------->
                116130    116140    116150    116160    116170
                     :         :         :         :         :
  116121: TTTGGAATAGGCAAATCCATAGAAACAGGAGGTAGATTCCTGGTTTCCAG  Hsapiens
  435827: ...A....T..............................T..........  Ptroglodytes
  453169: ...A....T.........................................  Ggorilla
  489345: ...A..G..T.............................A..........  Pabelli
  377707: ...A.....C.................A......C....G..........  Nleucogenys
  235034: ...A....T..................A..........TG..........  Mmulatta
  244841: ...A....T..................A..........TG..........  Panubis
  438650: ...AC..G..A............................A..........  Cjacchus
 1078091: C..AC..G..A............................A..........  Sboliviensis

                                 LINE1
          ---------------------------------------------------->
                116180    116190    116200    116210    116220
                     :         :         :         :         :
  116171: GGTCTCCAGGAAGGGAAGAATGAAGTACAAGATTTCTTTTGGAGGTAGTG  Hsapiens
  435877: .........A........................................  Ptroglodytes
  453219: .........A........................................  Ggorilla
  489395: ......G..AGT...............T...T...A..............  Pabelli
  377757: ......G..AGT............AG.....T...A..............  Nleucogenys
  235084: ......G..AG....................G...........A......  Mmulatta
  244891: ......G..AG....................G...........A......  Panubis
  438700: .....TG..AG......A..G....G.....G.................A  Cjacchus
 1078141: .....TG..AG....G.A..G........................C...A  Sboliviensis

                                 LINE1
          ---------------------------------------------------->
                116230    116240    116250    116260    116270
                     :         :         :         :         :
  116221: AAATTGTTGTGGAATGAGATCATGATGATGATAGCACAACTTTGTGAATA  Hsapiens
  435927: ...........................G......................  Ptroglodytes
  453269: ...........................G......................  Ggorilla
  489445: .....A..............G......G..-.....T............G  Pabelli
  377807: .....A..............G......G..-..................G  Nleucogenys
  235134: .....A..............G......G.....................G  Mmulatta
  244941: .....A..............G......G.....................G  Panubis
  438750: .....A.....................G...C.................G  Cjacchus
 1078191: ....AA......C............CAGC..C.................G  Sboliviensis

                                 LINE1
          ---------------------------------------------------->
                116279    116289    116299              116309
                     :         :         :                   :
  116271: TAAT-AAAATCATTGAATTGTACAGTTGAA----------TTTATGGTAT  Hsapiens
  435977: ...G-.........................----------...G......  Ptroglodytes
  453319: ....-.........................----------...G......  Ggorilla
  489494: ..C.A.........................AATGGTGAACC.........  Pabelli
  377856: ..C.A.........................AATGGTGAACC..G......  Nleucogenys
  235184: ..C.A.........................AATGGTGAACC.G.......  Mmulatta
  244991: ..C.A.........................AATGGTGAACC.G.......  Panubis
  438800: ..C.A.......C....G............AATAGTGAACC...G.....  Cjacchus
 1078241: ..C.A.......CC...G..G...AC....AATGGTAAACC.........  Sboliviensis
```

```
                       LINE1
          ------------------------------>
                 116319    116329    116338    116348    116356
                      :         :         :         :         :
  116310: ATAAATTATATGTTAATAAAAAGGGG-GTCCACAAAACAAA--CAGCCCC  Hsapiens
  436016: ..........................-..............--.......  Ptroglodytes
  453358: ..........................-..............--.......  Ggorilla
  489544: .C......C..C..............G..........G...AC.T....T  Pabelli
  377906: .C...G..C..C..............G.........TG.G.AC.C....T  Nleucogenys
  235234: ........C.................A..T.......G...AC......T  Mmulatta
  245041: ........C.................A..T.......G...AC......T  Panubis
  438850: ...........C.C............-..T.......------...TT..  Cjacchus
 1078291: .........C.C.C............-..........------...TT..  Sboliviensis

                 116366    116376    116386    116396    116406
                      :         :         :         :         :
  116357: CCACTCTGGTTGTCAGGGAGATATTGGATTAAATGGCCTTGGACAACAAC  Hsapiens
  436063: ..................................................  Ptroglodytes
  453405: ..................................................  Ggorilla
  489594: .T.T......G.....................T................-  Pabelli
  377956: .T.T......GT...............................TG....-  Nleucogenys
  235284: .T.T......G...................................G..-  Mmulatta
  245091: .T.T......G......................................-  Panubis
  438893: .T..A.G...A..G.CA............................G...-  Cjacchus
 1078334: .T.TG.....A..G.T..................CT..........T..-  Sboliviensis

                 116416    116426    116436    116446    116456
                      :         :         :         :         :
  116407: CCCTCTCCCTGGCCACAGACATTCTTCAGATTACAAGATATTCCAGGGGA  Hsapiens
  436113: ..................................................  Ptroglodytes
  453455: ..................................................  Ggorilla
  489643: ..A..........................................CA.AG  Pabelli
  378005: ........T.......................................AG  Nleucogenys
  235333: .......T............................A...........A.  Mmulatta
  245140: .......T........................................A.  Panubis
  438942: T..............T........C.....................AA..  Cjacchus
 1078383: T..............T......GAC.............G.......AA..  Sboliviensis

                 116466    116476    116486    116496    116506
                      :         :         :         :         :
  116457: AACACTGGAATGAGTCTGAAGCCAGGTGCTAAACAGAAGGACCATTGAGA  Hsapiens
  436163: .................................TG...............  Ptroglodytes
  453505: ..................................G...............  Ggorilla
  489693: .................................TG.T..A.G..CC....  Pabelli
  378055: .................................TG.T..A....C.....  Nleucogenys
  235383: .................................T..T.......C....C  Mmulatta
  245190: .................................T..T.......C....C  Panubis
  438992: ...........A.........G...........TG.T.......CCA...  Cjacchus
 1078433: ................G....G....CT.....TG.T.......C-A...  Sboliviensis

                                       LTR
                 --------------------------------------------->
                 116516    116526    116536    116546    116556
                      :         :         :         :         :
  116507: AATGTTGTGATCCTGACAGGTCAAGCAATTTATTTTTCGGCTTCATTTTT  Hsapiens
  436213: ............................................G.....  Ptroglodytes
  453555: ......................................T...........  Ggorilla
  489743: ...........T............A...C.........A...........  Pabelli
  378105: ........................A...C.........A...........  Nleucogenys
  235433: .............C.....T....A...C.........T...........  Mmulatta
  245240: ...................T....A...C..C......T...........  Panubis
  439042: ..............A....C....A...C.........T...........  Cjacchus
 1078482: ..C.....TT....A....C....A...C.........T..........A  Sboliviensis
```

```
                              LTR
                ----------------------------------->
                 116566    116576    116586    116594    116604
  116557: AAATGTAAAATTAGAAAGCTGCCATTTAAAATGG--CCCGTCTGTTTCAA  Hsapiens
  436263: .....A............................A.TC..T...........  Ptroglodytes
  453605: .....A....................C.......TC..T...........  Ggorilla
  489793: ...C.A..........C...T.....C......TCT..T..T........  Pabelli
  378155: .....A..............T.....C......TCT..T.CT..C.....  Nleucogenys
  235483: .....A......TA..A.........C.......--...A.T........  Mmulatta
  245290: .....A......TA..A.........C.......--.....T........  Panubis
  439092: ....AA....................C.......CT..T..T........  Cjacchus
 1078532: ....-A............TCA..T..C.......CT..T..T......G.  Sboliviensis

                                                   LTR
                                          ------------------->
                 116614    116624    116634    116644    116654
  116605: TTGCTCTTCTCAGTGTCAGCCTGTTAACTCAATGTGTTAGTCTGTTTTCA  Hsapiens
  436313: ....................A.............................  Ptroglodytes
  453655: ..................................................  Ggorilla
  489843: ...T..............A............G..CT..-..T...--...  Pabelli
  378205: ...T..................A........G...A.....T....C..G  Nleucogenys
  235531: ...T............T..............G...A..............  Mmulatta
  245338: ...T............T..............G...A..............  Panubis
  439142: ..TT.......C....TG.............G.A................  Cjacchus
 1078581: ..TT.......C...................G.A.A..............  Sboliviensis

                              LTR
                ----------------------------------------------->
                 116664    116674    116684    116694    116704
  116655: TGCTGCTGATAAAAACATACCTGAGACTGGCAAGAAAAAGAGGTTTAATT  Hsapiens
  436363: .............G....................................  Ptroglodytes
  453705: ..................................................  Ggorilla
  489890: .............G.......C....................T.G.....  Pabelli
  378255: .............G.......CA.....................A.....  Nleucogenys
  235581: .............G.......CA............G.......C......  Mmulatta
  245388: .............G.......CA............G.......C......  Panubis
  439192: .........C...G...CG...........G...................  Cjacchus
 1078631: .........C...G....G...........G...................  Sboliviensis

                              LTR
                ----------------------------------------------->
                 116714    116724    116734    116744    116754
  116705: GGGCTTAGAGTTCCACGTGATTGGGGAGGCCTCAGAATCACAGTAGGAGG  Hsapiens
  436413: ........................................TG..G.....  Ptroglodytes
  453755: ........................................TG........  Ggorilla
  489940: ..A....C.C......A...............T.......TT..G.....  Pabelli
  378305: ..A....C.C......A.......................TT.C......  Nleucogenys
  235631: ..A....C.C.....T........A........C......TG..G.....  Mmulatta
  245438: ..A....C.C.....T........A........C......TG..G.....  Panubis
  439242: ..A....C.T......A...C.T.AA..............TG.C......  Cjacchus
 1078681: ..A....C.T..........C.TAA...............TG.C......  Sboliviensis
```

```
                                     LTR
         ------------------------------------------------------------------>
                         116773    116783    116793    116803
 116755: CAAAAGTTA-TTCTTACATGGTGGCTGCAAGAGAAGATGAGGAAGAAGCA   Hsapiens
 436463: .........-.........................A..............   Ptroglodytes
 453805: .........-.........................A..............   Ggorilla
 489990: .........-...............A.........A..............   Pabelli
 378355: .........-...............A.........A......T.......   Nleucogenys
 235681: .........-...............A.........A..............   Mmulatta
 245488: .........-...............A.........A..............   Panubis
 439292: T.....A..C........C.T..T.A.........A..............   Cjacchus
1078731: T.....A..C........CAA....A......T..A..............   Sboliviensis

                                     LTR
         ------------------------------------------------------------------>
               116813    116822    116832    116842    116852
 116804: AAAGAAGAAA-CCCCTGATAAACCCATCGGATCTCCTGAGGCTTATTAAC   Hsapiens
 436512: ..........-.............................A.........   Ptroglodytes
 453854: ..........-.............................A.........   Ggorilla
 490039: ....C.....-.............................A.........   Pabelli
 378404: ....C.....-...T.C.......................A.........   Nleucogenys
 235730: ......A...A.................A...........A.........   Mmulatta
 245537: ......A...A.................A...........A.........   Panubis
 439342: ....C.....-T.T............G.A......A....A...T.....   Cjacchus
1078781: ....C.....-T.T.....................A....A...T.....   Sboliviensis

                                     LTR
         ------------------------------------------------------------------>
               116862    116871    116881    116891    116901
 116853: TATCATGAGAATAGCACA-AGAAAGACCGGCCCCCATGATTCAATTACCT   Hsapiens
 436561: ..............TG..-...............................   Ptroglodytes
 453903: ..................-...............................   Ggorilla
 490088: .....C..C...G.....-G........A.TG...G..............   Pabelli
 378453: .....C..C...G.....-G........A..A...G..............   Nleucogenys
 235780: .....C............G.A.........T............G......   Mmulatta
 245587: .....C............G.A.........T............G......   Panubis
 439391: .....C..........TG-G........A.....................   Cjacchus
1078830: .....C..........TG-G........A.....................   Sboliviensis

                                     LTR
         ------------------------------------------------------------------>
               116911    116921    116931    116941    116951
 116902: CTACCTGGGTCCCTCCAATAACATGTGGAAATTCTGGTAGATACAATTCA   Hsapiens
 436610: ....A.................C...........................   Ptroglodytes
 453952: ......................C...........................   Ggorilla
 490137: .C-.......G.......C...C.....G.....................   Pabelli
 378502: .CC.......A.......C...C.....G.....................   Nleucogenys
 235830: .C..A.............C...C.....G.....................   Mmulatta
 245637: .C..A.............C...C.....G.....................   Panubis
 439440: .CT............TC.CC...A....G........G............   Cjacchus
1078879: .CT............T.GCC...A....G........G............   Sboliviensis
```

```
                              LTR
              ------------------------------------>
             116961    116971    116981    116991    117001
                  :         :         :         :         :
 116952: AGTTGAGATTTGGGTGGGAACACAGCCAAACCATATCACTCAGCAAGGCA  Hsapiens
 436660: ..................................................  Ptroglodytes
 454002: ...........................T.....C................  Ggorilla
 490186: ...........T....A.G.............G...............A.  Pabelli
 378552: ...........T......G.............................A.  Nleucogenys
 235880: ..................G.............G...............T.  Mmulatta
 245687: ..................G.............G...............T.  Panubis
 439490: ...CA.............G............T....G..G.G......TC  Cjacchus
1078929: ...CA.............G............T....G..G........TC  Sboliviensis

             117011    117021    117031    117041    117051
                  :         :         :         :         :
 117002: GATAACTTTCTCACTGAGCCTATGCAACAGAAAACCATCTGGGATGGTTG  Hsapiens
 436710: ......A..........C................................  Ptroglodytes
 454052: .................C................................  Ggorilla
 490236: .................C................-...A...........  Pabelli
 378602: .................C................-...AC..........  Nleucogenys
 235930: .................CT...............-...............  Mmulatta
 245737: .................CT....C..........-...............  Panubis
 439540: A.....A..........C................-......A...A....  Cjacchus
1078979: A..G.............C....A...........-...............  Sboliviensis

             117061    117071    117081    117091    117101
                  :         :         :         :         :
 117052: TAAGGGGCACAGGAAGTGACTGGTAGGATCACTGCCAAAGCTGAGCACTC  Hsapiens
 436760: .........TG...........................G...........  Ptroglodytes
 454102: ..........G...........................G...........  Ggorilla
 490285: C.........G...G........C..............G........T..  Pabelli
 378651: C.........G...G........C..............G.......GT..  Nleucogenys
 235979: A.........G...GA.......C..............G.TAC....T..  Mmulatta
 245786: A.........G...GA.......C..............G.TA.....T..  Panubis
 439589: CG........----------.....................A.....TG.  Cjacchus
1079028: C.........----------.....................A.....TG.  Sboliviensis

             117111    117121    117131    117141    117151
                  :         :         :         :         :
 117102: AGGAGAAGGCAATAGAATCCTATTCTCCATAGTATGCTATAAGATACTGA  Hsapiens
 436810: .....................G..................G.........  Ptroglodytes
 454152: .....................G.............A....G.........  Ggorilla
 490335: .....................G..................G.........  Pabelli
 378701: .....................G..................G.........  Nleucogenys
 236029: .A.....A.............G..................G.........  Mmulatta
 245836: .A.....A.............G..................G.........  Panubis
 439629: G............G.......G..................G.......C.  Cjacchus
1079068: ...........G.G.......G.......C..........G....C..C.  Sboliviensis

             117161    117171    117181    117191    117201
                  :         :         :         :         :
 117152: AGTACACTTCTTCACTATCTCTTTGGACTTAGAATTAGCACTACATTCCT  Hsapiens
 436860: ..............G............................T......  Ptroglodytes
 454202: ..............G............................T......  Ggorilla
 490385: ......T.....A.G..CA............A......ATA..T......  Pabelli
 378751: ............A.G...A............A......A.A..T......  Nleucogenys
 236079: .............GG................A......A.T..T......  Mmulatta
 245886: .............GG................A......A.T..T......  Panubis
 439679: ..C...........G.......................A....T......  Cjacchus
1079118: ..C.........T.G.......................A....T......  Sboliviensis
```

```
                 117211     117221     117231     117241     117251
                      :          :          :          :          :
 117202: TGTTATACAGAAAAATTACTAAGGAAATTCATAGGATGACAAAAACTTTC  Hsapiens
 436910: ......................T.......G........T.......A..  Ptroglodytes
 454252: ......................T.......G...................  Ggorilla
 490435: ....T.................T.........................G..  Pabelli
 378801: ....TA................T...........................  Nleucogenys
 236129: ................A.....T.....G.....................  Mmulatta
 245936: ................A.....T.....G.....................  Panubis
 439729: CA..C..........C.G................................  Cjacchus
1079168: G...C............G................................  Sboliviensis

                 117261     117271     117281     117291     117301
                      :          :          :          :          :
 117252: AGAACTGAAAAACAGGAAATGTAAGCTTTTTAGTTCTTTGGTATTCGAAG  Hsapiens
 436960: ................................A.............A...  Ptroglodytes
 454302: ..............................................A...  Ggorilla
 490485: ........................A..........G....T.G...A...  Pabelli
 378851: ...................................G....T.G...A...  Nleucogenys
 236179: .....C.............................G......G...A...  Mmulatta
 245986: ...................................G......G...A...  Panubis
 439779: ...................................G...T..G...A.C.  Cjacchus
1079218: ...................................T.A....G...A.T.  Sboliviensis

                 117311     117321     117331                117342
                      :          :          :                     :
 117302: TATGCCTAAAAGACAATGCAAAATCCAAGAAAAGAATGG---------TG  Hsapiens
 437010: .......................................---------..  Ptroglodytes
 454352: .......................................---------..  Ggorilla
 490535: C......T.............C...........T.....CACTTTT--.T  Pabelli
 378901: .......T.............C...........T.....CACTTT---.T  Nleucogenys
 236229: C......T.............C.................CCCTTTTTC..  Mmulatta
 246036: C..T...T.............C.................CCCTTTTTC..  Panubis
 439829: .......T.........T...C................T---------.C  Cjacchus
1079268: .......T.........T...CC...............T---------.C  Sboliviensis

                                Simple
            ------------------------------------------->
                 117350     117360     117370                117377
                      :          :          :                     :
 117343: GGGT--TTTTGTTTGTTTGTTTTTGTTTTTG-----------TTTT--AC  Hsapiens
 437051: ....--.........................-----------....--..  Ptroglodytes
 454393: ....--.............G.....G.....-----------G...--..  Ggorilla
 490583: TT..--................G.T..C...---CTTTGTTT....--..  Pabelli
 378948: TT..--...............CG.T..C...---CTTTGTTT....--..  Nleucogenys
 236279: T.T.TG.................CT...C.TTTCTTTTTTTT....AA..  Mmulatta
 246086: T.T.TG.................CT...C.TTTCTTTTTTTT...A--..  Panubis
 439870: ------...C....T......---------------------....--..  Cjacchus
1079309: TT----...............---------------------....--..  Sboliviensis

                 117387     117397     117407     117417     117427
                      :          :          :          :          :
 117378: AGCTGGAGTAGAATACAAAGGGATGGAGTTGAAACAAATGAGAGGAAATT  Hsapiens
 437086: ..................................................  Ptroglodytes
 454428: .............C....................................  Ggorilla
 490626: .T...T.....T.........A.AC..........T....G.........  Pabelli
 378991: .T.........T.........A.A...........T....G.........  Nleucogenys
 236329: .T.......T.T.......C.A.A....C.....TT..............  Mmulatta
 246134: .T.......T.T.........A.A....C.....TT..............  Panubis
 439891: ......C....T........A.............TG..C...G..C....  Cjacchus
1079332: ...C.......T......................TG..............  Sboliviensis
```

```
                   117437    117447    117457    117467    117477
                        :         :         :         :         :
  117428: GGAATTCTAAACTTATTCTCATTGGCATTAGAAAGGCACCTACATGTATT  Hsapiens
  437136: ..........C.......................................  Ptroglodytes
  454478: ...............C..................................  Ggorilla
  490676: ..........C.......C......T.A.......T.........-....  Pabelli
  379041: ..........C..............T.A.......T.........-....  Nleucogenys
  236379: ..........C................A.......T....G....-....  Mmulatta
  246184: ..........C................A.......T.........-....  Panubis
  439941: ..........C..........C...T............T.......C...  Cjacchus
 1079382: .A........CG.........C.A.T....................C...  Sboliviensis

                   117487    117497    117507    117517    117527
                        :         :         :         :         :
  117478: TCACATGAGCCGGTGACTGCTGACTTGCATTCTTATTTTTTCCCTATAGA  Hsapiens
  437186: .......................T..........................  Ptroglodytes
  454528: .......................T..........................  Ggorilla
  490725: ...A..A....A....T......T.CA....T.C................  Pabelli
  379090: ...A..A....A....T.A....T.CA....T.C................  Nleucogenys
  236428: ...A.......A....T......T..A....T.C................  Mmulatta
  246233: ...A.......A....T......T..A....T.C................  Panubis
  439991: ..GA.......A...GT......T.......-------......C.....  Cjacchus
 1079432: ..GA.......A...GT.....CT.......-------......C.....  Sboliviensis

                   117537    117547    117557    117567    117577
                        :         :         :         :         :
  117528: TTAAAAAGGAGGTACAATGGTAGAACTGTAATCCTGTCCTTTGTCATAAA  Hsapiens
  437236: ..............T..................T................  Ptroglodytes
  454578: ..................................................  Ggorilla
  490775: ..........................A.......................  Pabelli
  379140: ..........................A..........T.........G..  Nleucogenys
  236478: ..........A...............A......G.A.........G....  Mmulatta
  246283: ..........A.......A.......A......G.A..............  Panubis
  440034: ...........T....G.........A.............G........G  Cjacchus
 1079475: C.......A.TA....G.........A.................G.....  Sboliviensis

                   117587    117597    117607    117617    117627
                        :         :         :         :         :
  117578: TTTTCATATTCATAAAGGTGAGTGTTAGCCCGCTTGTGAAATCTGAAGTT  Hsapiens
  437286: .....C........................T.............A.....  Ptroglodytes
  454628: .....C........................T.............A.....  Ggorilla
  490825: ........C....C...T............T....A.......-------  Pabelli
  379190: .G......C....C................T.....C......-------  Nleucogenys
  236528: .G...TC.C....C................T............-------  Mmulatta
  246333: .G...TC.C....C................T............-------  Panubis
  440084: ........C...-G.......A........T.....C..........AC.  Cjacchus
 1079525: .....T..C.......A....A........TC....C...........C.  Sboliviensis

                   117637    117647    117654    117664    117674
                        :         :         :         :         :
  117628: GAGTAACTTCAAATACTAACCACAGA---GGGAAAGGCAGCAAGAGGAGA  Hsapiens
  437336: ..........................---....G................  Ptroglodytes
  454678: ..........................---....G................  Ggorilla
  490868: -------------.C..CT.......---....G...T............  Pabelli
  379233: -------------.C..CT....T..---....G..........        Nleucogenys
  379436:                                              .....  Nleucogenys
  236571: -------------.C..CT.......---....G...........AAG.   Mmulatta
  236732:                                                  .  Mmulatta
  246376: -------------.C..CT.......---....G..........        Panubis
  246533:                                              .....  Panubis
  440133: .......--A......C........G-----------.............  Cjacchus
 1079575: .......--G......C....G....GCT.A..G....G.T.........  Sboliviensis
```

```
             117684     117694     117704     117714     117724
                  :          :          :          :          :
  117675: GGCATAAATTTAGGATCTCACCCTTCATTCCACAGACACACACAGCCTCT  Hsapiens
  437383: ..........C............................G..........  Ptroglodytes
  454725: ..........C.......................................  Ggorilla
  490902: ..T.......C............................G.....G....  Pabelli
  379441: ..T.......C..................................G....  Nleucogenys
  236733: ..........C..................................G....  Mmulatta
  246538: ..........C...................G..............G....  Panubis
  440170: ..........C....G......G................G.....G...C  Cjacchus
 1079623: ..T.......C...........G................G.....G...C  Sboliviensis

             117734     117744     117754     117764     117774
                  :          :          :          :          :
  117725: CTGCCCACCTCTGCTTCCTCTAGGAACACAGGTAAGAGCTTCAAGCCTCT  Hsapiens
  437433: ....................................T.............  Ptroglodytes
  454775: ....................................T.............  Ggorilla
  490952: ...................T......T.........T..........A..  Pabelli
  379491: ....................................T..........A..  Nleucogenys
  236783: ........G...........................T..........A..  Mmulatta
  246588: ........G...C.............T.........T..........A..  Panubis
  440220: ...T.........T.............G........T..........A..  Cjacchus
 1079673: ....T......G.T......................T..........A..  Sboliviensis

             117784     117794     117804     117814     117824
                  :          :          :          :          :
  117775: CCAGCTTAATAACATGAATTATTTTTGAGAATAATAATGATACTGTGTTC  Hsapiens
  437483: ..................................................  Ptroglodytes
  454825: ..................................................  Ggorilla
  491002: .........A........................................  Pabelli
  379541: .........A........................................  Nleucogenys
  236833: ..................................................  Mmulatta
  246638: ..................................................  Panubis
  440270: ............T..A.....C....T..C..............A.....  Cjacchus
 1079723: ...............A..........T..C..............A.....  Sboliviensis

             117834     117844     117854     117864     117874
                  :          :          :          :          :
  117825: TATATCATGCATCTCCTGCATTCTGTCTGATTATATTTTACTTATTCTGC  Hsapiens
  437533: ............T.....................................  Ptroglodytes
  454875: ............T.....................................  Ggorilla
  491052: ..C...............................................  Pabelli
  379591: ................................................T.  Nleucogenys
  236883: ........................C.........G...............  Mmulatta
  246688: ........................C.........G...............  Panubis
  440320: ............T....T.GA..........................C.T  Cjacchus
 1079773: ............T....T................................  Sboliviensis

             117884     117894     117904     117914     117924
                  :          :          :          :          :
  117875: CAGAGCAAAATTAAAATACCTATTTCATCTGATTTGTCCTTTATCTAAAT  Hsapiens
  437583: ...................................A..............  Ptroglodytes
  454925: ...................................A..............  Ggorilla
  491102: ...G.....G........................................  Pabelli
  379641: ...G.....G........A...............................  Nleucogenys
  236933: ...G...T..........T..G......................G.....  Mmulatta
  246738: ...G...T..........--.G......................G.....  Panubis
  440370: .C.G.............G.........G......C...............  Cjacchus
 1079823: TG.G.....................T.........T..............  Sboliviensis
```

```
             117934    117944    117954    117964    117974
             :         :         :         :         :
  117925: TGCTTAGTTCCAAGTAAACCAAGGCACTTTTAGGAACACAGAGGGAGAGT  Hsapiens
  437633: ..................T...........C...................  Ptroglodytes
  454975: ..................T...........C...................  Ggorilla
  491152: .......................A...CC.....................  Pabelli
  379691: ...........G............A..CC.............C..T....  Nleucogenys
  236983: .A.................T.......CC.G...................  Mmulatta
  246786: .A.................T.......CC.G...................  Panubis
  440420: ......T.....T....G.A..AA...CC.............A...A...  Cjacchus
 1079873: ......T.....T....G.A..AA.G.CC....C........A.......  Sboliviensis

             117984    117994    118004    118014    118024
             :         :         :         :         :
  117975: GCCTTGCAGCCAGAGAGTCTTGAAGGAGATGTCAGGGACGCATCTTAACA  Hsapiens
  437683: .............................C.........C........A.  Ptroglodytes
  455025: ................................................G.  Ggorilla
  491202: ............C....C.........A.C........TT.C......G.  Pabelli
  379741: ............C....C.........A.C........TT.C......G.  Nleucogenys
  237033: ............C.A..CT........A.C........TT.C......G.  Mmulatta
  246836: ............C.A..C.........A..........TT.C......G.  Panubis
  440470: ...CA.......C.A..C.....T...A.CA.......T.TC......G.  Cjacchus
 1079923: ....CC......C...AC.........AGCA.......A.TC......G.  Sboliviensis

             118034    118044    118054    118064    118074
             :         :         :         :         :
  118025: GCTGGTTGGATGTGATCCACAGAGGTCTCCTGTTAGCATTCATTGTAAAG  Hsapiens
  437733: ..................G.............C.................  Ptroglodytes
  455075: ......CTT.------------...CT.TA.------.....A.A.....  Ggorilla
  491252: .......................................A....A.....  Pabelli
  379791: ...............................C.......A....A.....  Nleucogenys
  237083: .....................A......................A.....  Mmulatta
  246886: .....................A......................A.....  Panubis
  440520: ....A......AC.............T....C......G.....A.....  Cjacchus
 1079973: ............G..................C......G.....A.....  Sboliviensis

             118084    118094    118104    118114    118124
             :         :         :         :         :
  118075: CCATCCTACCTAGCTCTAGTGTAACCAGCAATGAAAGAAAGATAAAGAGG  Hsapiens
  437783: ..T...............................................  Ptroglodytes
  455107: ..T...........C........G...........G------........  Ggorilla
  491302: ..T....G......C........G...A...C...G............TC  Pabelli
  379841: ..T...........C........G...........G............T.  Nleucogenys
  237133: ..T...........C.C............T.....G..............  Mmulatta
  246936: ..T........G..C.C..................G..............  Panubis
  440570: A.T...........C........GA..........G............T.  Cjacchus
 1080023: A.T...........C........G...........G............T.  Sboliviensis

             118134    118144    118154    118163    118173
             :         :         :         :         :
  118125: GTCGATTACTTATTTACAATAGTCTTTAAAAAC-GTAGTTTTGTAAGCCT  Hsapiens
  437833: .................................-................  Ptroglodytes
  455151: ...T..............G..............AC..A......G.....  Ggorilla
  491352: ...T..........C..................A...A............  Pabelli
  379891: ...T.............................A...A............  Nleucogenys
  237183: ...T......G.....T.....-..........A...A......G.....  Mmulatta
  246986: ...T...........G.................A...A......G.....  Panubis
  440620: ...T.......................GC....AA..ACC....G..T..  Cjacchus
 1080073: ...T........................C....A...A.C....G..T..  Sboliviensis
```

```
                118183    118193    118203    118213    118223
                     :         :         :         :         :
 118174: TCTAATTAGGACATTAATATATTTAATATATGCACATTGTAGAAAGATTG  Hsapiens
 437882: ..................................................  Ptroglodytes
 455201: ..................................................  Ggorilla
 491402: ...........G.............................A....C...  Pabelli
 379941: ........A..G.........C...................A....C...  Nleucogenys
 237232: CT................................................  Mmulatta
 247036: CT..................G..C......A...................  Panubis
 440670: ........T..G....T.G...........................C...  Cjacchus
1080123: ........T..G......G...........................T...  Sboliviensis

                118233    118237    118244    118254    118264
                     :         :         :         :         :
 118224: AAGCGTTAAAAA------TAAGA---GAAAAACTTTAAATGTCAAAATCT  Hsapiens
 437932: ..A.........------.....---.........C....T.........  Ptroglodytes
 455251: ..A.........------.....TGA.G......................  Ggorilla
 491452: .GA.........-------..A.---A.G........C...........C  Pabelli
 379991: .GAT........-------..A.---A.G....................C  Nleucogenys
 237282: ..A.........CGGTAGC..                               Mmulatta
 470282:                               ....................  Mmulatta
 247086: ..AT.C......------.....AAA........................  Panubis
 440720: ..A.A.......-----G.....-AA..........----......C..C  Cjacchus
1080173: ..A.A.......-----G.....-AA..........----..........  Sboliviensis

                118274    118283    118293    118303    118313
                     :         :         :         :         :
 118265: CACAACCCAGATATATCA-TTTCTTTAAGAAAATTGTACTACAAAATACC  Hsapiens
 437973: ......A........C..-..........T....................  Ptroglodytes
 455295: ......A........C..-...............................  Ggorilla
 491492: .......T.....CG.A.T........................G...GA.  Pabelli
 380031: .......T......G.T.T...............C........G...GA.  Nleucogenys
 470302: ..............G...-...G...........CA..............  Mmulatta
 247130: ..............G...-...............CA..............  Panubis
 440760: ..............G...T......................T.....GA.  Cjacchus
1080213: ..............G...T......................T.....GA.  Sboliviensis

                118323    118333    118343    118353    118363
                     :         :         :         :         :
 118314: ATTCCATTTATTAAAGTCATTCTGACAGGAATCTGATGCTTTTCCAGGAG  Hsapiens
 438022: ..................................................  Ptroglodytes
 455344: ..................................................  Ggorilla
 491542: .A................................................  Pabelli
 380081: .C.....A..........................................  Nleucogenys
 470351: .C.G..............................................  Mmulatta
 247179: .C.G..............................................  Panubis
 440810: .C.......G.G.............A......T.................  Cjacchus
1080263: .C.........G.............A......T..........T......  Sboliviensis

                                                B4:7
                                 <---------------------------
                118373    118383    118393    118403    118413
                     :         :         :         :         :
 118364: TTCCAGATCACATCGAGTTCACCATGAATTCACTCAGTGAAGCCAACACC  Hsapiens
 438072: ..................................................  Ptroglodytes
 455394: ..................................................  Ggorilla
 491592: .............TAC..................................  Pabelli
 380131: -.............ACA.................................  Nleucogenys
 470401: -.................................................  Mmulatta
 247229: --T...............................................  Panubis
 440860: ..............AG...............C........G.........  Cjacchus
1080313: ..............AG...............C........G.........  Sboliviensis
```

```
                                     B4:7
          <--------------------------------------------------
                118423    118433    118443    118453    118463
 118414: AAGTTCATGTTCGATCTGTTCCAACAGTTCAGAAAATCAAAAGAGAACAA  Hsapiens
 438122: ......................G...................A.......  Ptroglodytes
 455444: ..............G.......G...................A.......  Ggorilla
 491642: ....................T.............................  Pabelli
 380180: ..............C..............T..C...........C.....  Nleucogenys
 470450: .......................................C..A.......  Mmulatta
 247277: .......................................C..A.......  Panubis
 440910: C.....T.......C..A........................A.......  Cjacchus
1080363: C.....T..G....C........................G..---.....  Sboliviensis

                                     B4:7
          <--------------------------------------------------
                118473    118483    118493    118503    118513
 118464: CATCTTCTATTCCCCTATCAGCATCACATCAGCATTAGGGATGGTCCTCT  Hsapiens
 438172: ..................................................  Ptroglodytes
 455494: ..................................................  Ggorilla
 491692: ............................................C.....  Pabelli
 380230: ...............C..................................  Nleucogenys
 470500: ..C.........................A.....................  Mmulatta
 247327: ..C...............................................  Panubis
 440960: ...T........................A.....................  Cjacchus
1080410: ............................A.....................  Sboliviensis

                               B4:7
          <--------------------------------------
                118523    118533    118543    118553    118563
 118514: TAGGAGCCAAAGACAACACTGCACAACAAATTAGCAAGGTAGCTATCAGC  Hsapiens
 438222: .......AG...................G....AG...............  Ptroglodytes
 455544: .................................A................  Ggorilla
 491742: ....G............................A........G.......  Pabelli
 380280: ....G.........T....C...A.........A........G.......  Nleucogenys
 470550: ....G............................A........G.......  Mmulatta
 247377: ....G............................A........G.......  Panubis
 441010: ....G........A...................A........G.......  Cjacchus
1080460: ....G.......GA...................A........G....T..  Sboliviensis

                118573    118583    118593    118601    118611
 118564: ATCATTACGTTGTCCTGTTGCAGTTTTTCTCTG--GTTCCGTCGGCTAGC  Hsapiens
 438272: .................................--...............  Ptroglodytes
 455594: ............................G....--...............  Ggorilla
 491792: T......T............T............TG.............T.  Pabelli
 380330: T.......A..............A.........CG........A......  Nleucogenys
 470600: T........C.............C..C......TG......G.....C..  Mmulatta
 247427: T.......TC.............C..C......TG......G.....T..  Panubis
 441060: T...........A......A.............TA....T...A......  Cjacchus
1080510: T..........CA.....C........C.....TA....T..........  Sboliviensis
```

```
           118621    118631    118641    118651    118661
118612: ACGCAGATGGTAATAGATGTGGTGGTCTGATGGGTAGCACAGGGGGCTGT Hsapiens
438320: .........................C...................A...A Ptroglodytes
455642: .........................C.......................A Ggorilla
491842: ......T......C...........C.......................A Pabelli
380380: ............GC...........C.......................A Nleucogenys
470650: .T..................A....C....C.......G..........A Mmulatta
247477: .T.......................C.......................A Panubis
441110: .............C....C......C....C..............C...A Cjacchus
1080560: .A...........C....C......C.......................A Sboliviensis

           118671    118681    118691    118701    118711
118662: GCAGGAATTCCCATAACTGTGAGACCACTGACTTAAACAGATCTTTTGAG Hsapiens
438370: .............................-.................... Ptroglodytes
455692: .............................-C................... Ggorilla
491892: .................................................. Pabelli
380430: ..................................C............A.. Nleucogenys
470700: .........T..G..........C.......................... Mmulatta
247527: .........T..G.........AC......................C... Panubis
441160: ...........T..T........G.......................... Cjacchus
1080610: ..............TC.............A.................... Sboliviensis

                                              B4:6
                                         <--------------
           118721    118731    118741              118760
118712: TAAAGTTTTCTTGTCCCGCTTCATGTCTCTTCCAGGTTC-TTCACTTTGA Hsapiens
438419: .......................................-.......... Ptroglodytes
455741: .......................................-.......... Ggorilla
491942: ...C................C..................T.......... Pabelli
380480: ................T.T.C.......T..........-.......... Nleucogenys
470750: .......................................-.......C.. Mmulatta
247577: .......................................-.......C.. Panubis
441210: C......C........T.....C................-.......... Cjacchus
1080660: .......C........T.....C................-.......... Sboliviensis

                            B4:6
        <-------------------------------------------
           118770    118780    118790    118800    118810
118761: TCAAGTCACAGAGAACACCACAGAAAAAGCTGCAACATATCATGTGAGTC Hsapiens
438468: ..........................................C....... Ptroglodytes
455790: ..........................................C....... Ggorilla
491992: ..........................................C....... Pabelli
380529: ...........................G........G.....C....... Nleucogenys
470799: .......C.............................C....C....... Mmulatta
247626: .......C.............................C....C....... Panubis
441259: .............------.....G........C.....C..G.....C. Cjacchus
1080709: ....A............................C........G.....C. Sboliviensis

           118820    118830    118840    118850    118860
118811: ACAGAGCACTATGGTTCAGCTTTAGATCCCTGAACAGGTCATAGTTTAAA Hsapiens
438518: .................................................. Ptroglodytes
455840: ...........C...................................... Ggorilla
492042: ...................A.......G.....GA............... Pabelli
380579: ...........C...............G.....G......G......T.. Nleucogenys
470849: ..........T......................G................ Mmulatta
247676: ..........T......................G................ Panubis
441303: ......T...T...........C....A.....G................ Cjacchus
1080759: ......TC..T...........C....A.....G................ Sboliviensis
```

```
                 118870    118880    118890    118899    118909
                      :         :         :         :         :
  118861: CCTGGAACTTCACAAAAACTAAGAAAAGGCCA-GTTTTAGGGAAAATCTT  Hsapiens
  438568: ..............G.................-T.......T........  Ptroglodytes
  455890: ..............G.................-T................  Ggorilla
  492092: .........C....G.................-T........G...G.C.  Pabelli
  380629: ....A.........G.................-T........G...G.C.  Nleucogenys
  470899: ..............G........T........-T.C......G...G.C.  Mmulatta
  247726: ..............G........T........-T.C......G...G.C.  Panubis
  441353: ..............G............ACTT.GT........G.....C.  Cjacchus
 1080809: ..............G...........T.....-T.....A..G.....C.  Sboliviensis

                 118919    118929    118939    118949    118959
                      :         :         :         :         :
  118910: GGACACAAAGATTGAGACATACAGAGTGGGTTGGCATTTCATGGCACATA  Hsapiens
  438617: ..................................................  Ptroglodytes
  455939: .....A............................................  Ggorilla
  492141: ...TGA............C...............................  Pabelli
  380678: ...TGA............................................  Nleucogenys
  470948: ...TGA............G..........A....................  Mmulatta
  247775: ...TGA............................................  Panubis
  441403: .....A......................AT......A.............  Cjacchus
 1080858: .....A..............G.......AT......A.............  Sboliviensis

                                              MIR
                                     ------------------>
                 118969    118979    118989    118999    119009
                      :         :         :         :         :
  118960: ATTATTATTCCTCATTTCTGCGTTACTAAAAGACAGTCAGCACTGTACCT  Hsapiens
  438667: .....................A.....G....................TC  Ptroglodytes
  455989: ...................T.......G......................  Ggorilla
  492191: ...........A.....-G..T...G.G....................T.  Pabelli
  380728: ...........A.....-G..T...G.G....................T.  Nleucogenys
  470998: .............G...T...A.....G.............C......T.  Mmulatta
  247825: .............G.............G.............C......T.  Panubis
  441453: ......G....AA..C.T.........G........----........T.  Cjacchus
 1080908: ...........A.G.......A.....G........----........T.  Sboliviensis

                                MIR
          ------------------------------------------------->
                 119019    119029    119039    119049    119059
                      :         :         :         :         :
  119010: CAGAGCATAGGTCTGGATCAGGATAGGCTGGGTTCAGACTCCAGCTTTGC  Hsapiens
  438717: ...............C..................................  Ptroglodytes
  456039: .............................A....................  Ggorilla
  492240: ....................................A.T..A........  Pabelli
  380777: ....................................A.............  Nleucogenys
  471048: .............................A......A.............  Mmulatta
  247875: ...........................G........A.T.G.........  Panubis
  441499: ..........A.T....C...T..............A.T...........  Cjacchus
 1080954: ............T....C...T..........C.G.A.T....A......  Sboliviensis
```

```
                                   MIR
         ------------------------------------------------->
                  119069    119079    119086    119096    119106
  119060: TCTTCACAAATGATGAATAAGAGC---AGGACACAACTGCTCGGAGTCCC  Hsapiens
  438767: ........................AGG.......................  Ptroglodytes
  456089: ............G....C.T....---...............A.......  Ggorilla
  492290: .................C.C.-..---....T......T...A....A..  Pabelli
  380827: .................C.C....---G.................T....  Nleucogenys
  471098: ............G....C.G....---.T.............A.......  Mmulatta
  247925: .................C.T.-..---.........T.....A..A...T  Panubis
  441549: .......G....G....C.CAG..---........G-....T.....T..  Cjacchus
 1081004: .................C.CAG.G---.........-....T........  Sboliviensis

                                   MIR
         ------------------------------------------------->
                  119116    119126    119136    119146    119156
  119107: AGTGACCTCATCCCAGAAAACTAAGGGTAAGAAAAAATCTGACTCAATAC  Hsapiens
  438817: ........................................T.........  Ptroglodytes
  456136: .....................A........T..G................  Ggorilla
  492336: ..............................T...................  Pabelli
  380874: .......C......................T..-................  Nleucogenys
  471145: ....................G..G......T...................  Mmulatta
  247971: ........T....T................T...................  Panubis
  441595: ......A..............A........T...................  Cjacchus
 1081050: ......A.........G....A........T...................  Sboliviensis

                                   MIR
         ------------------------------------------------->
                  119166    119176              119195    119204
  119157: ATGCAAATACATGCAAATGTTTACAACAG-TGCCTTGCCCATAAAA-GTC  Hsapiens
  438867: .............................-................T...  Ptroglodytes
  456186: .........T.......CA.........T-..T.............T...  Ggorilla
  492386: .................CA......G...-.......T........A...  Pabelli
  380923: .................CA......G...-................A...  Nleucogenys
  471195: ..................A....T.....-....C...........A...  Mmulatta
  248021: .................CA...C......-..T............--...  Panubis
  441645: .............G...CA.....TG..AA.............G..A...  Cjacchus
 1081100: .............G...CA.........AA................A...  Sboliviensis

                     MIR                       Simple
         ----------------------->         ----------------->
                  119214    119224    119234    119244    119254
  119205: ATAATAAATGTTATTATTATTATAAAGTAGCTATAATTATACTAATCATA  Hsapiens
  438916: ..................................................  Ptroglodytes
  456235: ..........................A.....................C.  Ggorilla
  492435: ...---...---G.............A.....................C.  Pabelli
  380972: ................................................C.  Nleucogenys
  471244: ...............G..........A.....................C.  Mmulatta
  248068: .........---..............A..........C..........C.  Panubis
  441695: .....G...---.........G....A.......C..-..........C.  Cjacchus
 1081150: .........---..............A.....G....-..........C.  Sboliviensis
```

```
                   Simple
         ---------------------->
                           119271    119281    119291    119301
                                :         :         :         :
  119255: ATAATGT---GAAAATAATTTAATTTTCATTGAGTCATTAATGAGATTCA  Hsapiens
  438966: .......---.................A.........C............  Ptroglodytes
  456285: .......TGCA...--T...................G..........AA.  Ggorilla
  492479: .....A.TGCA......C.........A....................A.  Pabelli
  381022: .......TGCA................A....................A.  Nleucogenys
  471294: .A.....TACA......C.........A....................A.  Mmulatta
  248115: G....A.TGCA................A....................A.  Panubis
  441741: .......TGCA................A.........C..........A.  Cjacchus
 1081196: .......TG----..............A.........C..........A.  Sboliviensis

                 119311    119321    119331    119341    119351
                      :         :         :         :         :
  119302: GAGGAATAAGCACAAGTCCAAGTATATTTTGGAAAATGATTGCTATGGAA  Hsapiens
  439013: .....................C...........C................  Ptroglodytes
  456333: .....................C............................  Ggorilla
  492529: ..................................................  Pabelli
  381072: ..................................................  Nleucogenys
  471344: .........................................T........  Mmulatta
  248165: ..................------..........................  Panubis
  441791: ....................-.......C.....................  Cjacchus
 1081242: ....................-..............C..............  Sboliviensis

                 119361    119371    119381    119391    119401
                      :         :         :         :         :
  119352: TATATTGGTTTAGAGCCTTAATAGTGCAAAATGCTTTGCTGGAAGGTAGA  Hsapiens
  439063: ..................................................  Ptroglodytes
  456383: ............................................A...C.  Ggorilla
  492579: ....................T.T.....................A.....  Pabelli
  381122: ....................C.......................A.....  Nleucogenys
  471394: ...............A....................C-......A.....  Mmulatta
  248209: .............G..........A.AG................A....G  Panubis
  441840: ..................................C.........A.....  Cjacchus
 1081291: ..C....................................A....A.....  Sboliviensis

                 119411    119421    119431    119441    119451
                      :         :         :         :         :
  119402: AAGTTCTAGATTTAAACAGGCTTAGGTTCAAAACTTGGCACTTCTAATTT  Hsapiens
  439113: ..................................................  Ptroglodytes
  456433: .GT....G..........................G...............  Ggorilla
  492629: .G...........C....................................  Pabelli
  381172: .G................................................  Nleucogenys
  471443: .GT............-..................G.....T.........  Mmulatta
  248259: .G.............-..................................  Panubis
  441890: .GA.............................................G.  Cjacchus
 1081341: .G.-...............A............................G.  Sboliviensis

                 119461    119470    119473    119479    119489
                      :         :         :         :         :
  119452: ATGTCTCTATAAACA-GGGT-------TTTTTTCC----CCATTCTCTGA  Hsapiens
  439163: ...............-....-------........----...........  Ptroglodytes
  456483: ..........G..G.-..T.-------.AC...------...........  Ggorilla
  492679: ...............G....-------......T.----...........  Pabelli
  381222: ...............G....-------......T.----...........  Nleucogenys
  471492: .C...........G.-..T.-------.A....------...........  Mmulatta
  248308: .............T.-.A..-------......T-----.T.........  Panubis
  441940: .............G.-..T.TGGTGTG......TT----TA.........  Cjacchus
 1081390: .............G.-...G-------......TTAAAAAA.........  Sboliviensis
```

```
              119499    119509    119519    119528    119538
  119490: GCTTTCTTGTGTTCATCTGAATTGAACTAAAGACTTA-GAGTTACCCATG  Hsapiens
  439201: .....................................-............  Ptroglodytes
  456519: ..........T....C.....................-............  Ggorilla
  492718: ........A....G.......................A............  Pabelli
  381261: .....................................-..........A.  Nleucogenys
  471528: ...G......T....C.C...................-............  Mmulatta
  248345: ..........T.A........................-............  Panubis
  441985: .....T....T.....T......T.............A..A..G....CA  Cjacchus
 1081432: ..........T.....T....................A............  Sboliviensis

              119548    119558    119568    119578    119588
  119539: TAAAGTCCTTAGCCATGGACCTGGCATACACTCTTCTTACGTGCAGAGAA  Hsapiens
  439250: .................T........C.......................  Ptroglodytes
  456568: .................T........C.......................  Ggorilla
  492768: .................T........C.....G.................  Pabelli
  381310: .................T........C.....G.......A.........  Nleucogenys
  471577: ............T...CT.....A..C.....G..G....T.........  Mmulatta
  248394: .....CT....C.....T........C.....G....C..A.........  Panubis
  442035: ....AC.........A.T........C...G.G..A...T..........  Cjacchus
 1081482: ....AC.........A.T........C.....G..A...T..........  Sboliviensis

              119598    119608    119618    119628    119638
  119589: TGACCATCATGAGGAAAGAGCCACAGATCAGTCAATGTGTCCTACAAGAT  Hsapiens
  439300: .........................................T........  Ptroglodytes
  456618: ..................................................  Ggorilla
  492818: ........G........A.....T....T............T.G......  Pabelli
  381360: .......................T.................T........  Nleucogenys
  471627: ....A..............A...T..........T......T........  Mmulatta
  248444: ..........A............T..........G......T........  Panubis
  442085: ..................C....T..----...........T......G.  Cjacchus
 1081532: ..................CA...T.................T......G.  Sboliviensis

              119648    119658    119668    119678    119688
  119639: AATAGCACCAACAGGTATAACAGGGCTTCCTGGCATAATCTATTTAAAAT  Hsapiens
  439350: G...C.G...........................................  Ptroglodytes
  456668: ..................................................  Ggorilla
  492868: G...C.............................G...C..C........  Pabelli
  381410: G...C............C.............A..G...C..C........  Nleucogenys
  471677: G...C..........................................G..  Mmulatta
  248494: G...CT............................................  Panubis
  442131: G...C......G.............A.-------................  Cjacchus
 1081582: G...C......G....G........G.........G..............  Sboliviensis

                                                   LINE2 →
              119698    119708    119718    119728    119738
  119689: ATCCAACCTTCAACATACTCGTATCCTTGATGACTGTTAGAAGTGAAATA  Hsapiens
  439400: ........................T....C....................  Ptroglodytes
  456718: ....................A.............................  Ggorilla
  492918: ......A...T..T......A...T.......T...........T.....  Pabelli
  381460: ......A...T..T......A...T.......T..........AT.....  Nleucogenys
  471727: ......A.............A...........G.................  Mmulatta
  248544: ......A...A..T......A...T.......T...........T....G  Panubis
  442174: ......A.............AC..........GT................  Cjacchus
 1081632: ......A.............AC..........GT........C.......  Sboliviensis
```

LINE2 →

```
                        119748     119758     119768     119778     119788
  119739: TGGTCCTTGCCCATAAGGAGCTGAGAGTTTAACTGGGAAGCTAAACCTAA  Hsapiens
  439450: ..................................................  Ptroglodytes
  456768: ...........TT.....................................  Ggorilla
  492968: A.......T...TC....................................  Pabelli
  381510: A...........TC....................................  Nleucogenys
  471777: ............T.....................................  Mmulatta
  248594: .........T..T......A....C................C........  Panubis
  442224: ..........T.T...A.......A.....................T...  Cjacchus
 1081682: ..........T.T...........A.........................  Sboliviensis

                        119798     119808     119818     119828     119838
  119789: CCCTTTAAACCAACAAGGAGAAAATCTACTGGTAGACAGCGCTGCATCTT  Hsapiens
  439500: ............................G.....................  Ptroglodytes
  456818: ...................C......C.G..........T.........A  Ggorilla
  493018: ..................G.....C.C.A..........T..........  Pabelli
  381560: ..................G.....C.C.A.........ATAA........  Nleucogenys
  471827: ........C.................C.G..........T..........  Mmulatta
  248644: ........G.........G.....C.C.A..........T..........  Panubis
  442274: ..A......T........G.......C.G......G...T......G...  Cjacchus
 1081732: ..A.........C.....G.......C.G..........T......G...  Sboliviensis

                        119848     119858     119868     119878     119888
  119839: TAGTTCAGAAGAGAAAAGATTGCAGTACGTTAGAGCAAGAAGAATTTTCT  Hsapiens
  439550: ............................A.....................  Ptroglodytes
  456868: ...........T...............T...................C..  Ggorilla
  493068: ......................G.A...A....T...G............  Pabelli
  381610: ......................G.....A....T...G............  Nleucogenys
  471877: ..............G.......G.....A....T...G............  Mmulatta
  248694: ..............G.......G.....A....T...G............  Panubis
  442324: ......................G.....T........G............  Cjacchus
 1081782: ......................G.....A........G............  Sboliviensis

                        119898     119908     119918     119928     119938
  119889: GGAAGAAGTCAAATATAAGGTGGATTTTGAAGGGTATTTGAGGTGAAATA  Hsapiens
  439600: ..................................................  Ptroglodytes
  456918: .........G...C.................................G..  Ggorilla
  493118: ....A........C....................................  Pabelli
  381660: ..................................................  Nleucogenys
  471927: .............C.......A..........A.................  Mmulatta
  248744: .............CG......A..........A.................  Panubis
  442374: .............C....T...T............G........A.....  Cjacchus
 1081832: .............C....T...T.........A..G..............  Sboliviensis

                        119948     119958     119968     119978     119988
  119939: CACCAATTATCAGGGAATAACATCAAAGGTCCTCAATGAGACTACCAGCA  Hsapiens
  439650: ..................G.G..............T.............C  Ptroglodytes
  456968: ......G.............GCA............T.............C  Ggorilla
  493168: ......G.............GCA........A...T.............C  Pabelli
  381710: ......G.............GCA............T.............C  Nleucogenys
  471977: ......G.............GCA........A...T....GA.......C  Mmulatta
  248794: ......G.......A.....GCAT.......A...T....GA.......C  Panubis
  442424: T.....G.......A.....GCA........A...T.........T...C  Cjacchus
 1081882: ......G.............GCA........A...T.........T...C  Sboliviensis
```

```
                   119998      120007      120017      120027      120037
  119989: TTTAGGGACTGAT-CTAACAGACTTAGCATGGGTTTAGTATTTACATTGA Hsapiens
  439700: .............-.................................... Ptroglodytes
  457018: A............-.................................... Ggorilla
  493218: ......AG.....-.................................... Pabelli
  381760: ......A......-.................T.................. Nleucogenys
  472027: ......A......-...............C........A........... Mmulatta
  248844: .....-A......-...............C........A.......CC.. Panubis
  442474: ........T....-......C............................. Cjacchus
 1081932: ..A..T..T....C......C...........T......G.......... Sboliviensis
```

```
                                                        B4:5
                                                  <-----------
                   120047      120057      120067      120077      120087
  120038: TACAGCAATTGAATGATCTCCCTTTTTGATGTTTGAAGGTTGATAGGTCA Hsapiens
  439749: .....................T.......C.................... Ptroglodytes
  457067: .....................T.......C.................... Ggorilla
  493267: .....................T.......CA..........A........ Pabelli
  381809: .....................T.......CA..........A........ Nleucogenys
  472076: .G...T....C.G........T.C.......................... Mmulatta
  248892: .G...T....C.G......G.T.......C.................... Panubis
  442523: .....T....C.....G....T...C...CA..............A.... Cjacchus
 1081982: ...G.T....C.....G....T...C...CA..............A.... Sboliviensis
```

```
                                  B4:5
          <-------------------------------------------------
                   120097      120107      120117      120127      120137
  120088: GGAAATGTTCATCACCAGTTTCAAAAGCTTCTGACTGAATTCAACAAATC Hsapiens
  439799: .................................................. Ptroglodytes
  457117: .........................................A........ Ggorilla
  493317: .........................................A........ Pabelli
  381859: ............T............................A........ Nleucogenys
  472126: .......G.................................A........ Mmulatta
  248942: .........................................A........ Panubis
  442573: ...........C..G..........................A........ Cjacchus
 1082032: ..............G..........................A........ Sboliviensis
```

```
                                  B4:5
          <-------------------------------------------------
                   120147      120157      120167      120177      120187
  120138: CACTGATGCATATGAGCTGAAGATCGCCAACAAGCTCTTCGGAGAAAAGA Hsapiens
  439849: .................................................. Ptroglodytes
  457167: .......................................T.......... Ggorilla
  493367: .........C...............A.............T.......... Pabelli
  381909: .......................................T.......... Nleucogenys
  472176: ..............................T................... Mmulatta
  248992: .................................................. Panubis
  442623: .................................................. Cjacchus
 1082082: ...............A.................................. Sboliviensis
```

```
                B4:5
                    120197                                      120216    120226    120236
 120188: CGTATCAATTTTTACAGGT-AATTTCACCTGGCCTACCCACATTTCATTT  Hsapiens
 439899: .......G...........-............................C.  Ptroglodytes
 457217: .A.................A............................C.  Ggorilla
 493417: T..................A.....-------------...C......C.  Pabelli
 381959: .A.................A.....-------------...C......C.  Nleucogenys
 472226: ...T...............A...........A.........C....G.C.  Mmulatta
 249042: ...T...............A...........A.........C......C.  Panubis
 442673: T.............A....A.......A.........GA.TC......C.  Cjacchus
1082132: ..............A....A.................AA..C......C.  Sboliviensis

                    120246    120256    120266    120276    120285
 120237: GCATCCTGATGTCTGTGTCTCTGAGTGGCCAAATGGAAGAAAGC-AAGGC  Hsapiens
 439948: ...G.......................................T-.....  Ptroglodytes
 457267: ...G........................................-.....  Ggorilla
 493454: ...G....G................CA.................-.....  Pabelli
 381996: ...G....C.A..............CA..............G..-.....  Nleucogenys
 472276: ...G....G.....................T.............-.....  Mmulatta
 249092: ...G....G..........C..........T.............-.....  Panubis
 442723: ...G..C.G....C..........C......TG.......G...T.....  Cjacchus
1082182: ...G....G........A......C......C........G...T.....  Sboliviensis

                    120295    120305    120315    120325    120335
 120286: AGATGAGCCTGGCCGACTCAGGTGGAGAGCATTTACTCAGAGTGCATTAG  Hsapiens
 439997: .....T...........C................................  Ptroglodytes
 457316: ..............A..C................................  Ggorilla
 493503: .CC....T...T.T...C.......................A........  Pabelli
 382045: .C.....T...T.T...C........C.................T.....  Nleucogenys
 472325: .................C..A....G........................  Mmulatta
 249141: .................C..A....G...................T....  Panubis
 442773: .C.....T...A.T...G.......G........................  Cjacchus
1082232: .C.....T...A.T...G................................  Sboliviensis

                    120345    120355    120365    120375    120385
 120336: CTCCATTTCCACAACTCTCCCCCACTGGAGTGTCCCAGACCCCAACGATA  Hsapiens
 440047: ....G.........................................A...  Ptroglodytes
 457366: ....G.........................................A...  Ggorilla
 493553: ....G................A.-------CA......G....G..A...  Pabelli
 382095: .....................A.-------C.......G...TG.TA.C.  Nleucogenys
 472375: .................................T........TG..A...  Mmulatta
 249191: ....G............................T........TG..A.G.  Panubis
 442823: ..............G....T.......................G..A..C  Cjacchus
1082282: ..............G............................G..A...  Sboliviensis

                    120395    120405    120415    120425
 120386: CATCACTGAAGTGTGGATTTAGGGATAATCTTGTGATAAAAGAGGAGG--  Hsapiens
 440097: ....................................G...........--  Ptroglodytes
 457416: ..................................A.............--  Ggorilla
 493596: .....G............................A.............--  Pabelli
 382138: .....G............................A.............--  Nleucogenys
 472425: .....G.........C..................A.G...........--  Mmulatta
 249241: .....G.........C..................A.............--  Panubis
 442873: T....GG....--------------------..CA..T..........--  Cjacchus
1082332: T....GG......................T...CA..T..........AG  Sboliviensis
```

```
            120442    120452    120462    120472    120482
                 :         :         :         :         :
 120434: -TTGTGTAATAGAGTGAGTAAGAGTAATAAGTAATAAGATACCATCGATA  Hsapiens
 440145: -C..G..................................C..........  Ptroglodytes
 457464: -...G.............................................  Ggorilla
 493644: -...G...G.......-------------.................A...  Pabelli
 382186: -...G...G.......-------------.................A...  Nleucogenys
 472473: -...G...G.......-------------.................AG..  Mmulatta
 249289: -...G...G.......-------------.................AG..  Panubis
 442901: ----------..GT..-------------G.......A.C.....G...G  Cjacchus
1082382: A...G...GC..CA---------------........A.C......A..G  Sboliviensis

            120492    120500    120510    120520    120526
                 :         :         :         :         :
 120483: AACTGGCACTGAC--TCAGTCACATACGATACATCTTGGTGGGAA----A  Hsapiens
 440194: .............--..............................----.  Ptroglodytes
 457513: ......T......--...........GC.................----.  Ggorilla
 493680: .............CT.....G..                             Pabelli
 382222: .............CT.....G..                             Nleucogenys
 472509: ...C.........CT.....G..C..                          Mmulatta
 249325: ...C.........CT.....G..                             Panubis
 442928: .............--...........GT.................----.  Cjacchus
1082417: .G...........--...........GT.................ATGT.  Sboliviensis

            120536    120546    120556    120566    120576
                 :         :         :         :         :
 120527: TGTATGACTAATGGGATATTATTGGAATGGGCAGGCTTGGGTGAGTTCCT  Hsapiens
 440238: ..................................................  Ptroglodytes
 457557: ..................................................  Ggorilla
 442972: ......G..............C......................A.....  Cjacchus
1082465: ......G.....................................A.....  Sboliviensis

            120586    120596    120606    120616    120626
                 :         :         :         :         :
 120577: GAGAATAGTTGAGGAAGTACCAGGAAATATTGAATGCACAGGATGAAAGA  Hsapiens
 440288: ....G.............................................  Ptroglodytes
 457607: .G...........................CG...................  Ggorilla
 443022: .G.G.......----------........C....A.T....C........  Cjacchus
1082515: .G.G.........................C...........C..C.....  Sboliviensis

            120636    120646    120656    120666    120676
                 :         :         :         :         :
 120627: CAAAAACAAAGATCAGAAACATCATGGTTAAAATTACTGGAGAGAAGTCT  Hsapiens
 440338: ...........G......................................  Ptroglodytes
 457657: T............................G...........A...G....  Ggorilla
 443062: G......T...G........G........G..........T....G....  Cjacchus
1082565: T......T...G....G............G..........T.........  Sboliviensis

            120686    120696    120706    120716    120726
                 :         :         :         :         :
 120677: GAGAAGCAATGAATCTCCTTCAGGGAAGCCTGCTCTGCAGTTTGCAAACC  Hsapiens
 440388: .......................A..........................  Ptroglodytes
 457707: ..................................................  Ggorilla
 443112: C....................G..A..A...T..................  Cjacchus
1082615: C.....................C....A...C...C..............  Sboliviensis
```

```
                 120736    120746    120756    120766    120776
                      :         :         :         :         :
  120727: ACAGCCTCTTCTGCTTCTGCCTTTTGCCAAGATGATATTGACCTTCAGTG  Hsapiens
  440438: ..........................-.......................  Ptroglodytes
  457757: ..........................-.C.....................  Ggorilla
  493679:                             ..AC..GC.C............  Pabelli
  382221:                             ..AC..GC.C............  Nleucogenys
  472508:                             ..ACC.GC.C............  Mmulatta
  249324:                             ..ACC.GC.C............  Panubis
  443162: ..........................-.C.---.......G.........  Cjacchus
 1082665: ..........................-.C..G..G.....G.........  Sboliviensis

                 120786    120796    120806    120816    120826
                      :         :         :         :         :
  120777: ACCTCTTTCTTGTGCCAGCCCACATTCCCCTTTTGCATTGCCTACATGAC  Hsapiens
  440487: ..................................................  Ptroglodytes
  457806: ............C...........C....................G..G.  Ggorilla
  493701: ....G...T...............C...............T....G..G.  Pabelli
  382243: ....G...T...............C....................G..G.  Nleucogenys
  472530: ....A...-...G...........C.A..................G..G.  Mmulatta
  249346: ....G...-...G...........C.A..................G..G.  Panubis
  443208: ...C.............A......C..TTA.....T...ATT.G....G.  Cjacchus
 1082714: ...C........C..TG.......C..TTA.....T...ATT.G....G.  Sboliviensis

                 120836    120846    120856    120866    120876
                      :         :         :         :         :
  120827: ACCTGTATAAAAATATCCATGGACAGGAGATACTGCATCTATTCAGGGTC  Hsapiens
  440537: ..................G...................T...........  Ptroglodytes
  457856: ...G............G.................................  Ggorilla
  493751: ................G...A...................G.........  Pabelli
  382293: ................G...A...................G.........  Nleucogenys
  472579: .T..............G.................A.....G......T..  Mmulatta
  249395: .T..............G.......................G......T..  Panubis
  443258: ................G.......................C.........  Cjacchus
 1082764: ................G.......................C.........  Sboliviensis

                 120886    120896    120906    120916    120926
                      :         :         :         :         :
  120877: TGGATTCAGCTTACTGTTGTTACAAATAAGTAAGTTTGGTAATATATAGT  Hsapiens
  440587: ..................................................  Ptroglodytes
  457906: ...............A.AA...T......C...A...........G....  Ggorilla
  493801: ..............CA.AA...T......C...A................  Pabelli
  382343: ..T............C.AA...T......C...A................  Nleucogenys
  472629: ............CT.A.AA...T......C...A...A............  Mmulatta
  249445: .......G.......AGAA...T......C...A...A........G...  Panubis
  443308: .........G...T.A.AA...T....C.C...A................  Cjacchus
 1082814: .............T.A.AA...--------...A.......G........  Sboliviensis

                                                      B4:4
                                                     <----
                 120936    120946    120955    120965    120975
                      :         :         :         :         :
  120927: TACATAAATTACTCCTAATTCCTACTT-CTTCCTTCATATCTCAAAGGAA  Hsapiens
  440637: ...........................-......................  Ptroglodytes
  457956: ..............T..C.........T......................  Ggorilla
  493851: ..T...........T............T......................  Pabelli
  382393: ..G...........T....AT......T......................  Nleucogenys
  472679: ..............T............T.C....C...G...........  Mmulatta
  249495: ..............T............T.C........G...........  Panubis
  443358: ..............T..C.........T...T....T....C........  Cjacchus
 1082856: ..............T..C.........T........T....C........  Sboliviensis
```

```
                                   B4:4
         <--------------------------------------------------
                120985    120995    121005    121015    121025
                     :         :         :         :         :
  120976: TATTTAGATGCCATCAAGAAATTTTACCAGACCAGTGTGGAATCTACTGA  Hsapiens
  440686: .............................................GT...  Ptroglodytes
  458006: .............................................GT...  Ggorilla
  493901: ........A...........C........................GT...  Pabelli
  382443: ........A...........C.....TA.................GT...  Nleucogenys
  472729: .............................................GT...  Mmulatta
  249545: .......................A..............A......GT...  Panubis
  443408: ......................A......................GT...  Cjacchus
 1082906: ..............G.......A......................GT...  Sboliviensis
```

```
                                   B4:4
         <--------------------------------------------------
                121035    121045    121055    121065    121075
                     :         :         :         :         :
  121026: TTTTGCAAATGCTCCAGAAGAAAGTCGAAAGAAGATTAACTCCTGGGTGG  Hsapiens
  440736: ..................................................  Ptroglodytes
  458056: ..................................................  Ggorilla
  493951: .......C..........................................  Pabelli
  382493: .......C................C.........................  Nleucogenys
  472779: .........G........................................  Mmulatta
  249595: .........G........................................  Panubis
  443458: .....T.G.A.A...........................T..........  Cjacchus
 1082956: .........A........................................  Sboliviensis
```

```
                 B4:4
         <---------------
                121085    121095    121105    121115    121125
                     :         :         :         :         :
  121076: AAAGTCAAACGAATGGTAGGAGAGCCACCCATTATAGAAACACCTTTGAG  Hsapiens
  440786: ..................................................  Ptroglodytes
  458106: ..................................................  Ggorilla
  494001: ........................................A.........  Pabelli
  382543: .......................A..........................  Nleucogenys
  472829: ..........................G......G....C...........  Mmulatta
  249645: ..........................G.....GG....C...........  Panubis
  443508: ...........................T..G...................  Cjacchus
 1083006: ...........................T......................  Sboliviensis
```

```
                121135    121145    121155    121165    121175
                     :         :         :         :         :
  121126: AAACCTATGCCAGTGAGCCTTGTGCTTGACACTGCATGGGGGAACAGGTG  Hsapiens
  440836: ..........G................................C......  Ptroglodytes
  458156: ...T......G.C......C.A...C...A.............C..T.G.  Ggorilla
  494051: ..........G........C.....C.................C....G.  Pabelli
  382593: ..........G........C.....C...............A.C....GA  Nleucogenys
  472879: ..........G........C.....C...............-.C...CG.  Mmulatta
  249695: ..........G........C.....C...............-.C...CG.  Panubis
  443558: ..........T........C...C.C............A....C....GA  Cjacchus
 1083056: ..........G........C...C.C...TG.......A....C....GA  Sboliviensis
```

```
                  121185      121195      121205      121215
                       :           :           :           :
  121176: TGGGGATTGAGATGGGTTTGCAGGGAGGGCTGAAGAGGGCACTCCAG---  Hsapiens
  440886: ...............................................---  Ptroglodytes
  458206: ....A..........C............A.A................---  Ggorilla
  494101: ....A..........C.C..G.......A.A.......A........---  Pabelli
  382643: .A..A..........C............A.A................---  Nleucogenys
  472928: ...............C..........A.A.A.....A..........ATA  Mmulatta
  249744: ...............C..........A.A.A.....A..........---  Panubis
  443608: .A....C....................A..A..G.......A.G...---  Cjacchus
 1083106: .A.........................A..A..G.......A.G..A---  Sboliviensis


  121223: --------------------------------------------------  Hsapiens
  440933: --------------------------------------------------  Ptroglodytes
  458253: --------------------------------------------------  Ggorilla
  494148: --------------------------------------------------  Pabelli
  382690: --------------------------------------------------  Nleucogenys
  472978: AAGAGATTTTCCAAATAATAATATAAAGAGAGCCTCGGGGAGCCAAGACC  Mmulatta
  249791: --------------------------------------------------  Panubis
  443655: --------------------------------------------------  Cjacchus
 1083153: --------------------------------------------------  Sboliviensis


                  121230      121240      121248      121258      121268
                       :           :           :           :           :
  121223: --ATGAAGGATTTGTCCAAATG--AATATGAAGAGAGCCTAGGGGAGCCA  Hsapiens
  440933: --....................--..........................  Ptroglodytes
  458253: --......TG...T...T.T..--.T........................  Ggorilla
  494148: --......A....T........--.T........................  Pabelli
  382690: --.C....AG...T........--.T...................G....  Nleucogenys
  473028: AC..A...AG...T........--.....A..........C.........  Mmulatta
  249791: --..A...AG...T.......AAT.....A..........C.........  Panubis
  443655: --G.......C.....T.CG..--.........C..........A.AT..  Cjacchus
 1083153: --G.......C.....T.C...--..........C.........A.A...  Sboliviensis


                  121278      121288      121298      121308      121318
                       :           :           :           :           :
  121269: AGGAGGAAATCACAGGAAGCCAATTAGATGGAAACACATCTGGAGAATTA  Hsapiens
  440979: ..................................................  Ptroglodytes
  458299: ..............................A..G..T.G.C....G...C  Ggorilla
  494194: ..............................A.....T.G.C....G...C  Pabelli
  382736: G.............................A.....T.G.C....G...C  Nleucogenys
  473076: ..............................A.......G.C.........  Mmulatta
  249839: ..............................A.......G.C.........  Panubis
  443701: .A....................G...............G...........  Cjacchus
 1083199: .A....................................G...........  Sboliviensis


                  121328      121338      121348      121357      121367
                       :           :           :           :           :
  121319: TTTGCTTATGGCCCTGCATGACAATAGCTTTGTGGATC-CCCTGTCTCCG  Hsapiens
  441029: ....................C...........C.....-...........  Ptroglodytes
  458349: ......C...TT....G.....................-...........  Ggorilla
  494244: ......C...TT....G..........A..........-..T.......A  Pabelli
  382786: ......C...TT.T..G.....................-..T.......A  Nleucogenys
  473126: ......C...TT....G......G..............-..........A  Mmulatta
  249889: ......C...TT....G.....................-..........A  Panubis
  443751: .....AC...T.....G....T..........C.....-.........T.  Cjacchus
 1083249: .....AC...T.....G....T................A.........T.  Sboliviensis
```

```
                  121377    121387    121397    121407    121417
                       :         :         :         :         :
 121368: CTCAGGCCTATTTTGAGATCATATCCTTTACTTTAAATCAGACTCAAATT  Hsapiens
 441078: .G.......G........................................  Ptroglodytes
 458398: ........C.............G...................AC...GA.  Ggorilla
 494293: .....A..C.............G...................AC...T..  Pabelli
 382835: ........C.............G...................AC...T..  Nleucogenys
 473175: ........C.............G...........G.......AC...T..  Mmulatta
 249938: ........C.............G...........G.......AC...T..  Panubis
 443800: ........C...............T....T............GC.-.T..  Cjacchus
1083299: ........C...............T....T............GC...T..  Sboliviensis

                  121425    121435    121445    121455    121465
                       :         :         :         :         :
 121418: TTT--ATGATGAATATTTAATAGAAAACATTAGAAAGCGTCTCTCGTCTC  Hsapiens
 441128: ...--.....A..........................T............  Ptroglodytes
 458448: ...--...............A.................A....C.T..C.  Ggorilla
 494343: ..A--...............A.................A....C.T..C.  Pabelli
 382885: ...--...............A.................A....C.T..C.  Nleucogenys
 473225: ...TT...............A.................A....C.T....  Mmulatta
 249988: ...-T...............A.................A....C.T....  Panubis
 443849: ...--...............A.........G......T.....C.TC...  Cjacchus
1083349: ...--...............A................T.....C.T....  Sboliviensis

                  121475    121485    121495    121505
                       :         :         :         :
 121466: CTTTACTAATTGGGAAACAAGCAGCTCTCTGGTAAATCACCCTTTTGTC-  Hsapiens
 441176: .................................................-  Ptroglodytes
 458496: ...............................................C.A  Ggorilla
 494391: .............................C-................C.A  Pabelli
 382933: .............................C.............C...C.A  Nleucogenys
 473275: .............................C.........T.......C.A  Mmulatta
 250037: .............................C.....G...T.......C.A  Panubis
 443897: ......C.G.....G..............CA................C.A  Cjacchus
1083397: ........G.................T..CA........T.......C.A  Sboliviensis

                  121524    121534    121544    121554    121564
                       :         :         :         :         :
 121515: TCTGAGCTGGAGCTGCCTGGATCACATCTGTAGCCAATGTGTTCTGCAGG  Hsapiens
 441225: .......................C...........-.C............  Ptroglodytes
 458546: ...C.A..A............C.......C.....-.CAA..........  Ggorilla
 494440: .....A..A............C.......C.....-.CCA..........  Pabelli
 382983: .....A..A............C.......C.....-.CAA..........  Nleucogenys
 473325: .....A...............C.......C..A..-.CAC..........  Mmulatta
 250087: .....A...............C.......C..A..-GCAC..........  Panubis
 443947: ................T....CG......C.GTT.-.CAC..........  Cjacchus
1083447: .....................C..TG...CCGT..---------.A....  Sboliviensis

                  121574    121584    121594    121604    121614
                       :         :         :         :         :
 121565: GATTATCACAGCTCTCTTCCCCATCAAGGGCAAAGAGCTTGACAAAGTCT  Hsapiens
 441274: ..................................................  Ptroglodytes
 458595: ...............................-........A.........  Ggorilla
 494489: .............G.....T......G.............A.........  Pabelli
 383032: ..-..........G.................G........A.........  Nleucogenys
 473374: .............G.....T....................A......C..  Mmulatta
 250136: .............G.....T....................A......C..  Panubis
 443996: .............G..C.........C.A..........GA.........  Cjacchus
1083488: .............G............C............GA..G......  Sboliviensis
```

```
             121624    121634    121644    121654    121664
                  :         :         :         :         :
 121615: CCATTCTACAGACATCTTTCTTACCTCCCACCTCTCATTACAGGCCAAAC Hsapiens
 441324: A................................................. Ptroglodytes
 458644: ........T........A..C............................. Ggorilla
 494539: ........T........A..C............................. Pabelli
 383081: ........T........A..C............................. Nleucogenys
 473424: ......C.T...........C............................. Mmulatta
 250186: ......C.T...........C............................. Panubis
 444046: AG..A...T........A.TC...................A..A...C.. Cjacchus
1083538: AG..A..GT........A.TC.....                         Sboliviensis

             121674    121684    121694    121704    121714
                  :         :         :         :         :
 121665: TTACAGCAACTCAACATGAGAGTGAATAGGAAGATACCCCCGGAAGTAGT Hsapiens
 441374: ........................................-......... Ptroglodytes
 458694: .................................................. Ggorilla
 494589: ........................................A......... Pabelli
 383131: ..................................C.....A......... Nleucogenys
 473474: ........................................A......... Mmulatta
 250236: ........................................A......... Panubis
 444096: ....T....G...........C..................A.......A. Cjacchus

             121724    121734    121744    121754    121764
                  :         :         :         :         :
 121715: GTCTGACAGCACAGGACATGCGTTTCATATTACAGAGCTCAAGTCACTCA Hsapiens
 441423: .................................................T Ptroglodytes
 458744: ....C................A...................T......TG Ggorilla
 494639: ......G.............T....................T......TG Pabelli
 383181: .G....G.............T....................T......TG Nleucogenys
 473524: ....................T....................TA.....TG Mmulatta
 250286: ....................T....................TA.....TG Panubis
 444146: ...............G....T.........C..........TAA....TG Cjacchus

             121774    121784    121794    121804    121814
                  :         :         :         :         :
 121765: TCCTAAAATGCAATCAGGGCCTCCTTCCTCTGAATGGGGACCCCGTAGTT Hsapiens
 441473: ......................A..................T........ Ptroglodytes
 458794: ........A.............A...T....................... Ggorilla
 494689: ........A.............A..........T.T........A..... Pabelli
 383231: ....G...A.............A..........T.T........A..... Nleucogenys
 473574: ........A...G.T......CA.A........T.CA.......A..... Mmulatta
 250336: ........A...G.T.......A.A........T.CA.......A..... Panubis
 444196: ........A...G.........A............T.....TA.A..... Cjacchus
1083575:                                                 .. Sboliviensis

                       121825    121835    121845    121855
                            :         :         :         :
 121815: AAAAAAAAA---------TAAAAGTAGGAAGAGGAGGGAGGGAGAAAGGA Hsapiens
 441523: .........-------------........A................... Ptroglodytes
 458844: .........---------A............................... Ggorilla
 494739: T........---------A............................... Pabelli
 383281: T........---------A............................... Nleucogenys
 473624: ....G....-----------.T.A.C.............CT......... Mmulatta
 250386: ....G....-----------.T.A.C........................ Panubis
 444246: .........-----------..G.C...........A............. Cjacchus
1083577: .....T...TAAATAAATA.....C...........A..A.......... Sboliviensis
```

```
                 121864    121874    121884    121894    121904
                      :         :         :         :         :
 121856: AAGACACA-TGTTGGAAGAGTAGACAAAATCAGTTTATCAGTATTCCAAA  Hsapiens
 441560: ........-.........................................  Ptroglodytes
 458885: ........T.....A...................................  Ggorilla
 494780: ......T.-.....A.........T.......................T.  Pabelli
 383322: ......TG-.....A....T....T.......................T.  Nleucogenys
 473663: ....TG---.....A.........T.........G...............  Mmulatta
 250425: ....TG---.....A.........T.........G...............  Panubis
 444285: ....G.T.-.T...A.........T...........G...........TG  Cjacchus
1083627: ....G.TC-.....A.........T...........G...........TG  Sboliviensis

                 121913    121923    121933    121943    121953
                      :         :         :         :         :
 121905: TCAGATG-ATTGGAGACATTCATACACAGAGAACGTGAACTCCTTCTCTA  Hsapiens
 441609: .......-.......T..................A...............  Ptroglodytes
 458935: .......-.A.....T..................C...............  Ggorilla
 494829: .......-.C..A..T..C....----.......C...............  Pabelli
 383371: .......-.C..A..T..G....----.......A....T..........  Nleucogenys
 473710: .......A.C.A...T..C...............C.A.............  Mmulatta
 250472: .......-.C.A...T.TC...............C.A.............  Panubis
 444334: .......-.C.....T..C...G.G.........C...............  Cjacchus
1083676: .......-.C.....T..C...G.G.........AC.....A........  Sboliviensis

                 121963    121973    121983    121993    122003
                      :         :         :         :         :
 121954: TCACAAGAAGTGATGTCTCCATCAAGGGTAACTTTATACGACTGGAGCCT  Hsapiens
 441658: ..................................................  Ptroglodytes
 458984: ............................C....G................  Ggorilla
 494874: .....................................C............  Pabelli
 383416: .....................................CT...........  Nleucogenys
 473760: ....................G......A.........C............  Mmulatta
 250521: ....................G......-.........C.....-......  Panubis
 444383: .....................C.....T.........C.AG.........  Cjacchus
1083725: C......G.........A...C...............C.AG.........  Sboliviensis

                 122013    122023    122033    122043    122053
                      :         :         :         :         :
 122004: TGAAGAAAGCTGCATCTGGTGAACCACTGGTCAGTGAGTCTAACAATTCA  Hsapiens
 441708: ..........................T................T......  Ptroglodytes
 459034: ..........................T.......................  Ggorilla
 494924: .A.............T....A.....T................T......  Pabelli
 383466: .A............CT.......TT-T............G...T......  Nleucogenys
 473810: GA.............A..........T................T......  Mmulatta
 250569: GA.............A..........T................T......  Panubis
 444433: ..........................T................TGG..T.  Cjacchus
1083775: ..........................T........A.......TGG..T.  Sboliviensis

                 122063    122073    122083    122093    122103
                      :         :         :         :         :
 122054: AAGATCAAAGTCAGTGAGTCTCAAGCAGGGATTTGGGTCAATAATTAACG  Hsapiens
 441758: .........................T............G.........T.  Ptroglodytes
 459084: .........................T............G.........T.  Ggorilla
 494974: .........................T.A..........G.........T.  Pabelli
 383515: .....................A...T.A......A.A.G.........TT  Nleucogenys
 473860: ......G..................T.A..........G.........T.  Mmulatta
 250619: .........................T.A..........G.........T.  Panubis
 444483: ...........T.........A...T............G.........T.  Cjacchus
1083825: .................A...A...T............G.........TA  Sboliviensis
```

```
                                                                  B4:3
              122113    122123    122133    122143    122153
 122104: ATCAGTCACGAACATTTGCAAAGCATCTTCCAGACAAGCCATTTGTAGCT  Hsapiens
 441808: ...............................................C.A  Ptroglodytes
 459134: ...............................................C..  Ggorilla
 495024: .....................................A.G.......A..  Pabelli
 383565: ........T...A........................A.........A..  Nleucogenys
 473910: ..............C......................A.........C..  Mmulatta
 250669: .....................................A.........C..  Panubis
 444533: ....A............T.....T.............A.........C..  Cjacchus
1083875: .....................................A.........C..  Sboliviensis
```

```
                                                  B4:3
                                             <-------------
              122162    122172    122182    122192    122202
 122154: TGTGT-AAAAGACTCTTTTATTCTTTCCCTTGCAGAAAAAATTAAAAACC  Hsapiens
 441858: .....-............................................  Ptroglodytes
 459184: .....-............................................  Ggorilla
 495074: ...A.-............................................  Pabelli
 383615: ..CA.-.........--.................................  Nleucogenys
 473960: ...A.-............................................  Mmulatta
 250719: ...A.-.....................T......................  Panubis
 444583: .....A......T.......                                Cjacchus
 444911:                     ..T...........................  Cjacchus
1083925: .....A......T.......                                Sboliviensis
1084245:                     ..............................  Sboliviensis
```

```
                                  B4:3
         <-------------------------------------------------
              122212    122222    122232    122242    122252
 122203: TATTTCCTGATGGGACTATTGGCAATGATACGACACTGGTTCTTGTGAAC  Hsapiens
 441907: .........................C.......................T  Ptroglodytes
 459233: .CC.......A..T..........GCA....C...T..............  Ggorilla
 495123: ..A...........G..........A.....C...T.............T  Pabelli
 383662: ..A...........G............C...C..................  Nleucogenys
 474009: ...........A.A.G.........C.....C..................  Mmulatta
 250768: ..C......G...A.G.........C.....C..................  Panubis
 444941: ...........CA..G....AA..GAATG..C.A.T.............T  Cjacchus
1084275: ........A..AA........AT.GA..C..C.A.T..............  Sboliviensis
```

```
                                  B4:3
         <-------------------------------------------------
              122262    122272    122282    122292    122302
 122253: GCAATCTATTTCAAAGGGCAGTGGGAGAATAAATTTAAAAAAGAAAACAC  Hsapiens
 441957: ..................................................  Ptroglodytes
 459283: .............................G........T......G.T..  Ggorilla
 495173: .............................G........T........T..  Pabelli
 383712: ..............G................................T..  Nleucogenys
 474059: .....T........G..T..A...C.A...........T........T..  Mmulatta
 250818: ..............G..T..A...C.A...........T........T..  Panubis
 444991: ............................CG........T......G....  Cjacchus
1084325: ............................CG........T......G.T..  Sboliviensis
```

```
                                  B4:3
          <---------------------------------------
              122312    122322    122332    122342    122352
  122303: TAAAGAGGAAAAATTTTGGCCAAACAAGGTATTGTCTATATTTTATTTAT  Hsapiens
  442007: ..................................................  Ptroglodytes
  459333: ..................................................  Ggorilla
  495223: ..................................................  Pabelli
  383762: ............G..........G..........................  Nleucogenys
  474109: .............................................C....  Mmulatta
  250868: ..................................................  Panubis
  445041: .................................C................  Cjacchus
 1084375: ...................T.............C................  Sboliviensis

              122362    122372                    122386    122396
  122353: ATAGTGTAATATGTTAATACATGGAATG------TTAAACATTTCTGATG  Hsapiens
  442057: ............................------................  Ptroglodytes
  459383: ...A...............A..------------................  Ggorilla
  495273: ...A...............A..------------................  Pabelli
  383812: ...A...............A..------------................  Nleucogenys
  474159: ...A.A.............A..------------.........G......  Mmulatta
  250918: ...A.A.............A..------------.........G......  Panubis
  445091: ...AC.......A......T........TTAAAT................  Cjacchus
 1084425: ...A........A......T........TTAAAT................  Sboliviensis

              122403    122413
  122397: GAATGT---AACATGATAAGTAAAAAATA---------------------  Hsapiens
  442101: ......---....................---------------------  Ptroglodytes
  459421: ......---.C.....C...........----------------------  Ggorilla
  495311: ......---.C.....C..T........----------------------  Pabelli
  383850: ......ACT.C.....C.........G.----------------------  Nleucogenys
  474197: ....A.---.CT....C............AAATTTATCATGTCTCTGATG  Mmulatta
  250956: ......---------.C...........----------------------  Panubis
  445141: ...A.C---.C.....C...........----------------------  Cjacchus
 1084475: ...A..---.C.....C.....T.....----------------------  Sboliviensis

                        122423    122433    122443    122453
  122423: -------------------AAAATTGTTCATGTCTGTTATTTTGTTGTTT  Hsapiens
  442127: -------------------...............................  Ptroglodytes
  459446: -------------------...................T...........  Ggorilla
  495336: -------------------.....C.........................  Pabelli
  383878: -------------------.....C................C...C....  Nleucogenys
  474244: TAATGTACAAGTAAAAAAT......TA.......................  Mmulatta
  250975: -------------------......TA.......................  Panubis
  445166: -------------------....G.T....................---.  Cjacchus
 1084500: -------------------.G..G.T....................---.  Sboliviensis

              122463    122473    122483    122492    122502
  122454: TACTCTTATAACTTTATTTAGTTAGGAATACCTGAA-AAACTATTGTTTC  Hsapiens
  442158: ...........T........................-........T....  Ptroglodytes
  459477: ..G.................................G..G.......CC.  Ggorilla
  495367: ..G.................................G..G.......C..  Pabelli
  383909: ..G.............-...A...............G..G.......C..  Nleucogenys
  474294: ..G...................A..A..........G.....T....C..  Mmulatta
  251006: ..G...................A..A..........G.....T....C..  Panubis
  445194: ..G...........G.................A...G..........C..  Cjacchus
 1084528: G.G.............................A...G.......CA.C..  Sboliviensis
```

```
                  122512    122522    122532    122542    122552
   122503: TAACTCATGGAATTCCTGGGTTATTTCTTAGAAGAAGAAGGATGTGTTGC  Hsapiens
   442207: ...............T..................................  Ptroglodytes
   459527: ........TAC....T...A.........---.A...............A  Ggorilla
   495417: ........TA.....T.............------..........A...A  Pabelli
   383958: ....C...TA.....T.............---.A.........T.....A  Nleucogenys
   474344: ........TA.....T......G--....---.................A  Mmulatta
   251056: ........TA.....T........G....---.................A  Panubis
   445244: ........TA.........A............C................A  Cjacchus
  1084578: ........TA................A.....T...............AG  Sboliviensis
```

```
                  122562    122572    122582    122592    122602
   122553: TATCTCAATAATATTATCTTTTTTGTCTTGTGTTTCACGTGTTATTTGTT  Hsapiens
   442257: ..................................................  Ptroglodytes
   459574: ..................-......C.......C....T..G........  Ggorilla
   495461: .................................C....T..G........  Pabelli
   384005: ..................-..............C....T..G......C.  Nleucogenys
   474389: .................................C....T..G........  Mmulatta
   251103: ......................C..........C....T..G........  Panubis
   445294: ...............T.................C...TT..G..C.....  Cjacchus
  1084628: ...............T.................C...TT..G........  Sboliviensis
```

```
                                                 B4:2
                                       <---------------------
                  122612    122622    122632    122642    122652
   122603: GGACACATTGATTTATTGCAGAATACATACAAATCTGTACAGATGATGAG  Hsapiens
   442307: ..................................................  Ptroglodytes
   459623: ..................T.............G..CA.............  Ggorilla
   495511: ..................T.............G..C..............  Pabelli
   384054: ..................T.............GC.CA.............  Nleucogenys
   474439: ...TG......................C....G.................  Mmulatta
   251153: ................................G.................  Panubis
   445344: ..............T.............C....C.......C........  Cjacchus
  1084678: .................................C.C..............  Sboliviensis
```

```
                                B4:2
           <------------------------------------------------
                  122662    122672    122682    122692    122702
   122653: GCAATACAATTCCTTTAATTTTGCCTTGCTGGAGGATGTACAGGCCAAGG  Hsapiens
   442357: ..................................................  Ptroglodytes
   459673: ........CA......C.........C....................G..  Ggorilla
   495561: ........CG......C.........C.............G.........  Pabelli
   384104: ........CG......C.........C......C......G.........  Nleucogenys
   474489: ....A....G........................................  Mmulatta
   251203: ....A....G.....C..................................  Panubis
   445394: ........CG.T....C............................A....  Cjacchus
  1084728: ........CGCT.................................A....  Sboliviensis
```

```
                                 B4:2
         <------------------------------------------------
             122712    122722    122732    122742    122752
                  :         :         :         :         :
 122703: TCCTGGAAATACCATACAAAGGCAAAGATCTAAGCATGATTGTGCTGCTG   Hsapiens
 442407: ..................................................   Ptroglodytes
 459723: ..................................................   Ggorilla
 495611: ..T..........G....................................   Pabelli
 384154: ....C.............................................   Nleucogenys
 474539: .A................................................   Mmulatta
 251253: .A................................................   Panubis
 445444: .............G.......C...C........................   Cjacchus
1084778: ..........G..........C............................   Sboliviensis

                      B4:2
         <-----------------------------
             122762    122772    122782    122792    122802
                  :         :         :         :         :
 122753: CCAAATGAAATCGATGGTCTGCAGAAGGTAAGAACTTGCATCTACAACTC   Hsapiens
 442457: ..................................................   Ptroglodytes
 459773: ....................C.........................T...   Ggorilla
 495661: ..................T.A..........A.................-   Pabelli
 384204: ...............................A.................-   Nleucogenys
 474589: ..G.........A..................A.................-   Mmulatta
 251303: ..G.........A..................A.................-   Panubis
 445494: ..........CG..C................A.G................   Cjacchus
1084828: .........CCT...................A...........G......   Sboliviensis

             122812    122822    122832    122842    122852
                  :         :         :         :         :
 122803: TTCCTTCTACTGCCGGACATTTTTCCAAAGATACCAAGTTTAAACAAGGT   Hsapiens
 442507: ..................................................   Ptroglodytes
 459823: ..............A...................................   Ggorilla
 495710: ---...........T...T...............T........G......   Pabelli
 384253: ---...........T...T........................G......   Nleucogenys
 474638: ---...........T...T........................G......   Mmulatta
 251352: ---...........T...T........................G......   Panubis
 445544: ...........-..T....-......T..........A............   Cjacchus
1084878: ...........C..T...T.......TC.........A.....G....A.   Sboliviensis

             122862    122872    122882    122892    122902
                  :         :         :         :         :
 122853: AAAAGCTTATGACCGAGTTGCCTCAAAATGATGAAAAATTCTAAATGAGG   Hsapiens
 442557: ....-.............................................   Ptroglodytes
 459873: .......GG....................A.......C............   Ggorilla
 495757: ........T.....A.......C......A........C..A..G....A   Pabelli
 384300: ........G.....A...C...C.....CA...........A..G.....   Nleucogenys
 474685: G...A...G.....A.A..T.........A...........A..GG....   Mmulatta
 251399: ....A...G.....A.A..T.........A...........A..G.....   Panubis
 445592: ........G.....A....A.G.......A...........A........   Cjacchus
1084928: ........GA....A....A.A.......A...........A........   Sboliviensis
```

```
                                                      LINE2
                                                  ------------------>
               122912     122922     122932     122942     122952
  122903: AATGATGACTCACCTTCATATTACAAATATTTGAGCATAGGGCCTGACAC  Hsapiens
  442606: ..................................................  Ptroglodytes
  459923: ...........................G....A.................  Ggorilla
  495807: ...A..T.T..G.T..................A.................  Pabelli
  384350: ...A.AT......T..................A.................  Nleucogenys
  474735: ......T.........T...............A.................  Mmulatta
  251449: ......T.........................A.................  Panubis
  445642: ...........TG...................T.................  Cjacchus
 1084978: ............G...................T................T  Sboliviensis
```

```
                     LINE2
          ---------------------------->
               122962     122972     122982
  122953: AAACTGAAAGCTTAGTTTTTGTTTGTTTGTTT---GTT------------  Hsapiens
  442656: ........G.......................----..------------  Ptroglodytes
  459973: ........G..G.....AA..A..AA..TA..AGA...ACATGCATTGGA  Ggorilla
  495857: ........G.....A..AA.TA..AA..TA..                    Pabelli
  384400: ...T....G........AA..A..AA..TA..AGA...ACATGCATAGGA  Nleucogenys
  474785: ........G.....A..AA.TA..AA..TA..                    Mmulatta
  251499: ........G.....A..AA.TA..AA...A..AGA...ACATGCATAATA  Panubis
  445692: G...C...G.....A..A..AA..TA..A.                      Cjacchus
 1085028: G.......G.....A..A..AA..TA..A.                      Sboliviensis
```

```
                                         LINE1
                       ---------------------------------->
               122991     123001     123009                123026
  122988: ------TTTATTATTATTATTAT--AATACTTTAAGCTT---TAGGGTAC  Hsapiens
  442691: ----TG.................--..............---........  Ptroglodytes
  460023: TATCAA....A.GA.........GC.GC.A.....A...GAC.G..AG.A  Ggorilla
  384450: TATCAA...GA.GA.........GC.GC.A.....T...GAC.G..AG.A  Nleucogenys
  251549: TATCAA...GA.GA.........GC.GC.A.....A...GAC.G..AG.A  Panubis
```

```
                                 LINE1
          ------------------------------------------------->
               123035     123045     123055     123065     123075
  123027: ATGTGCA-CAATGTGCAGGTTAGTTACATATGTATACATGTGCCATGCTG  Hsapiens
  442732: .......-..........................................  Ptroglodytes
  460073: ..A.AT.G.......AG.AAAGT....GA..AC.                  Ggorilla
  384500: ..A.AT.G.......AG.AAAGT.....A..AC.                  Nleucogenys
  251599: ..ACAT.G.......AG.AAAGT.....A..AC.                  Panubis
```

```
                                 LINE1
          ------------------------------------------------->
               123085     123095     123105     123115     123125
  123076: GTGTGCTGCACCCATTAACTCGTCATTTAGCGTTAGGTATATCTCCTAAT  Hsapiens
  442781: .....................A.........A..................  Ptroglodytes
```

```
                                 LINE1
          ------------------------------------------------->
               123135     123145     123155     123165     123175
  123126: GCTATCCCTCCCCCCTCCCCCCACCCCACAACAGTCCTCAGAGTGTGATG  Hsapiens
  442831: ..................................................  Ptroglodytes
```

```
                                        LINE1
          ---------------------------------------------------------------->
                        123184    123194    123204    123214    123224
                             :         :         :         :         :
 123176: TTA-CCTTCCTGTGTCCAAGTGTTCTCATTGTTCAATTCCCATCTATGAT  Hsapiens
 442881: ...C..............................................  Ptroglodytes

                        123234    123244    123254    123264    123274
                             :         :         :         :         :
 123225: TTAATTCCATCTATGGCTTAGTTAATGATTAATTTATTAGAGTTACATGC  Hsapiens
 442931: ..................................................  Ptroglodytes
 459981:               ...G................................  Ggorilla
 495865:               ......A.....T.......................  Pabelli
 384402:         ..TG.A....................................  Nleucogenys
 474793:               ......A.....T.......................  Mmulatta
 251507:               ......A.....T.......G...............  Panubis
 445703:                      .....T............T..........  Cjacchus
1085039:                      .....T............T..........  Sboliviensis

                        123284    123294    123304    123314    123324
                             :         :         :         :         :
 123275: ATTGGATATCAATTTGATGATATTATTATGCAGCAATTTAAACTTGACTG  Hsapiens
 442981: ..................................................  Ptroglodytes
 460017: ...............A..................................  Ggorilla
 495901: ..A......................................T........  Pabelli
 384444: ..A......................................T........  Nleucogenys
 474829: ..AAT.............................................  Mmulatta
 251543: ..AAT.............................................  Panubis
 445732: .........................................C.....A..  Cjacchus
1085068: .........................................C.......-  Sboliviensis

                        123334    123344    123354    123364    123374
                             :         :         :         :         :
 123325: GGAGAAATATATACCAATGTGAGGAAAGTTTACAAATAGGCCGAGTAGAA  Hsapiens
 443031: ......................................C...........  Ptroglodytes
 460067: .............G...................G....CA..A....A..  Ggorilla
 495951: .......C.....G........................CA..A....A..  Pabelli
 384494: .............G........................CA..A..C.A..  Nleucogenys
 474879: .........C...G........................CA..A....A..  Mmulatta
 251593: .........C...G........................CA..A....A..  Panubis
 445782: .......C.C.C.G...C............G.......CA..A..C.A..  Cjacchus
1085117: .......C.....G......................C.CA..A..C.A..  Sboliviensis

                        123384    123394    123404    123414    123423
                             :         :         :         :         :
 123375: AAGGGAATACAAATTTAGGAATTTAGGGAATTACAATTTAATAATTGC-A  Hsapiens
 443081: .........G...............C......................-.  Ptroglodytes
 460117: .G.......G..............GA......G...............-.  Ggorilla
 496001: .........--------.A.........T.-----..AC.........-.  Pabelli
 384544: .........--------.A.........T.-----G.AG.........-.  Nleucogenys
 474929: .......G.--------.AG........T.-----..ACG........-.  Mmulatta
 251643: .........--------.AG  Panubis
 421838:                     ........T.-----..ACG........-.  Panubis
 445832: .........--------.A.........T------..A..C....C..-.  Cjacchus
1085167: .........--------.A.........T------..AC.C....C..G.  Sboliviensis
```

```
             123433    123443    123453    123463    123473
 123424: ATGTGTACTAAATAATGTATACAGAAAAATATGATGAGCCTATTAAAAAT  Hsapiens
 443130: ...............................A..................  Ptroglodytes
 460166: ............................T..A..................  Ggorilla
 496038: ...............................A..................  Pabelli
 384581: ...............C.....T.........A..............T...  Nleucogenys
 474966: ....A............C.............A..................  Mmulatta
 421863: ....A............C.............A-C....-...........  Panubis
 445867: ..AGA...................G......A---..............C  Cjacchus
1085203: ..A.....................G......A..A..............C  Sboliviensis

             123483    123493    123503    123513    123523
 123474: TGACACATGTAGTAGGCTGTTGGCACAAGAAATAGTGATACATACAGTTC  Hsapiens
 443180: ..................................................  Ptroglodytes
 460216: ..................................................  Ggorilla
 496088: ..................T...............A.....T...--....  Pabelli
 384631: ..................T.....................T...--....  Nleucogenys
 475016: ..............A.........................T.........  Mmulatta
 421911: ..............A.........................T.........  Panubis
 445914: ...T.T............................A.....T...--A...  Cjacchus
1085253: ...T..................A...........A.........--....  Sboliviensis

                                 Simple
                                 ------------------------->
             123533    123543    123553    123562    123572
 123524: ATTGTGTACAAAATAATGTAATCATATTTT-ACATGTGTATCATACAGTT  Hsapiens
 443230: ..............................-...................  Ptroglodytes
 460266: ..............................T.....A.............  Ggorilla
 496136: ................C....C.C......-....A.....T.....T..  Pabelli
 384679: ......C............T..........-....A.....T.....T..  Nleucogenys
 475066: .....A.........T.A...A........-....A.....T.....T..  Mmulatta
 421961: .....A.........T.A............-....A.....T.....T..  Panubis
 445962: ........G.....................T....A...G.T.....T..  Cjacchus
1085301: ..............................T....A.....T.....T..  Sboliviensis

                       Simple
         ------------------------------------------------->
             123582    123592    123602    123612    123622
 123573: GTATACATACATATGTACACATATACATATACGTAAAAACATGATTCTGT  Hsapiens
 443279: ................................A.................  Ptroglodytes
 460316: ......................-.........A.................  Ggorilla
 496185: .....-................-.........A......TG.........  Pabelli
 384728: ......................-........TA.................  Nleucogenys
 475115: ...............C......-.........A.................  Mmulatta
 422010: ...............C......-.........A..............C..  Panubis
 446012: .AT....G......--......-........T..................  Cjacchus
1085351: .......G......A.......-...GA...T........G.........  Sboliviensis
```

```
                                  Simple
         ----------------------------------------------------------->
                123632                  123644    123654    123664
                     :                       :         :         :
  123623: TTTTACATACATGTATA--------TACATATACACATATAACCCAATGT  Hsapiens
  443329: .................--------.........................  Ptroglodytes
  460365: .................--------.........................  Ggorilla
  496233: .....G......A...T--------.........T.......TT..G...  Pabelli
  384777: .....G......A...T--------................GTT..G...  Nleucogenys
  475164: .....G......A...T--------..........T......TT..G...  Mmulatta
  422059: .....G......A...T--------..........T......TT..G...  Panubis
  446059: ............A....CATACATA..T..............T.A.GCA.  Cjacchus
 1085400: G...........A.G..CCTACATAC.T.C............T.A..CA.  Sboliviensis

           Simple
         ---------->
                123674    123684    123694    123704    123714
                     :         :         :         :         :
  123665: ATTTATATATTCAGGACTCATATTTTACCTATTAGAATAATAATGTCTAT  Hsapiens
  443371: ...................G................G.....T.A.....  Ptroglodytes
  460407: ............................................A.....  Ggorilla
  496275: ...G......................G............G..---....A  Pabelli
  384819: ...G............T.........G...............---....A  Nleucogenys
  475206: ...G......................T......C........---....A  Mmulatta
  422101: ...G......................T......C........---....A  Panubis
  446109: ...........A.........--...G..C.....G......---....A  Cjacchus
 1085450: ...........A.........--...T..C............---....A  Sboliviensis

                123724    123734    123744    123754    123764
                     :         :         :         :         :
  123715: TAAAGTGAACCTTCTGTATTTCACATTTATTGCCAAAATAACGAATCTCC  Hsapiens
  443421: ..................................................  Ptroglodytes
  460457: ..................................................  Ggorilla
  496322: ..--..A.G................................A..TA....  Pabelli
  384866: ..--.....................................A..TA....  Nleucogenys
  475253: ..--A......................-.............A..TA....  Mmulatta
  422148: ..--A......................-.............A..TA....  Panubis
  446154: .........T...........T...................A..T.....  Cjacchus
 1085495: .G...................T...................A..T.....  Sboliviensis

                123774    123784    123794    123804    123814
                     :         :         :         :         :
  123765: ACATAGTCAATTCATTGTTAAGGTGTATTAGAGATCGACAGTTAGTCATA  Hsapiens
  443471: ..................................................  Ptroglodytes
  460507: ....................................A.............  Ggorilla
  496370: ..........C........................T..T...........  Pabelli
  384914: ......................................T.----......  Nleucogenys
  475300: ..........C...........................T...........  Mmulatta
  422195: ..........C...........................T...........  Panubis
  446204: .T..T.......G..................-....TTT....-......  Cjacchus
 1085545: .T..........G.......................TTT...........  Sboliviensis
```

```
                                                                B4:1
                                              <-----------------------
              123824     123834     123844     123854     123864
   123815: TCAGTTTCTTTTTTCCATTTGTATAGCTTGAAGAGAAACTCACTGCTGAG  Hsapiens
   443521: ..................................................  Ptroglodytes
   460557: ..................................................  Ggorilla
   496420: .....................C............................  Pabelli
   384960: .................................................C  Nleucogenys
   475350: ..................................................  Mmulatta
   422245: ..................................................  Panubis
   446252: ...........C......................................A  Cjacchus
  1085595: .................................................A  Sboliviensis
```

```
                                 B4:1
          <-----------------------------------------------------
              123874     123884     123894     123904     123914
   123865: AAATTGATGGAATGGACAAGTTTGCAGAATATGAGAGAGACATGTGTCGA  Hsapiens
   443571: ....................................C.....CA......  Ptroglodytes
   460607: ..........................................CC......  Ggorilla
   496470: ......T...............C...................CA...G..  Pabelli
   385010: ...C................................A.....C....G..  Nleucogenys
   475400: ......T................................G..CA...G..  Mmulatta
   422295: ......T................................G..CA...G..  Panubis
   446302: .........A..........................A..GTGGA...GA.  Cjacchus
  1085645: .........A...................C.........GTGC....GA.  Sboliviensis
```

```
                                 B4:1
          <-----------------------------------------------------
              123924     123934     123944     123954     123964
   123915: TTTACACTTACCTCGGTTCAAAATGGAAGAGAGCTATGACCTCAAGGACA  Hsapiens
   443621: ..................................................  Ptroglodytes
   460657: ......................G...........................  Ggorilla
   496520: ......................G...........................  Pabelli
   385060: ......................G...........................  Nleucogenys
   475450: .........G............G...........................  Mmulatta
   422345: .........G............G...........................  Panubis
   446352: .....G................G........GC......T..........  Cjacchus
  1085695: ......................G........GC......T..........  Sboliviensis
```

```
                                 B4:1
          <-----------------------------------------------------
              123974     123984     123994     124004     124014
   123965: CGTTGAGAACCATGGGAATGGTGAATATCTTCAATGGGGATGCAGACCTC  Hsapiens
   443671: ..................................................  Ptroglodytes
   460707: .......................G...C......................  Ggorilla
   496570: ....T.....T............G...........A..............  Pabelli
   385110: .......................G..........................  Nleucogenys
   475500: .........A.............G..C.......................  Mmulatta
   422395: .........A.............G..C.......................  Panubis
   446402: .A.....................G..G......G..---...........  Cjacchus
  1085745: .A.....C...............G..G.....GG..---...........  Sboliviensis
```

```
                                  B4:1
          <--------------------------------------------------
                    124024    124034    124044    124054    124064
                         :         :         :         :         :
  124015: TCAGGCATGACCTGGAGCCACGGTCTCTCAGTATCTAAAGTCCTACACAA  Hsapiens
  443721: ...........TG......G.........G....................  Ptroglodytes
  460757: ...........TG......G.........G....................  Ggorilla
  496620: ...........TG......G.......ATG....................  Pabelli
  385160: ...........TG......G.......GTG....................  Nleucogenys
  475550: ............G......G.......A.G...................G  Mmulatta
  422445: ...........TG..............A.G....................  Panubis
  446449: .........G.TG......GGAA....GTGA..........T........  Cjacchus
 1085792: .........G.TG......GG.A...TGTG......C.C..T........  Sboliviensis

                                  B4:1
          <--------------------------------------------------
                    124074    124084    124094    124104    124114
                         :         :         :         :         :
  124065: GGCCTTTGTGGAGGTCACTGAGGAGGGAGTGGAAGCTGCAGCTGCCACCG  Hsapiens
  443771: .............................C....................  Ptroglodytes
  460807: ...............T..A..........CA...................  Ggorilla
  496670: ...............T.............CA..G................  Pabelli
  385210: .............................CA..G................  Nleucogenys
  475600: .............................C...G.........A......  Mmulatta
  422495: .............................C...G................  Panubis
  446499: .............................C...G...A............  Cjacchus
 1085842: .............................CAA.G..CA......T...G.  Sboliviensis

                                  B4:1
          <--------------------------------------------------
                    124124    124134    124144    124154    124164
                         :         :         :         :         :
  124115: CTGTAGTAGTAGTCGAATTATCATCTCCTTCAACTAATGAAGAGTTCTGT  Hsapiens
  443821: ...............................................CA.  Ptroglodytes
  460857: .........G.A...G..C............................CA.  Ggorilla
  496720: ......G.....AATTCAC....A.ATG...................CA.  Pabelli
  385260: ............AATT.AC....A.AGT...................CA.  Nleucogenys
  475650: ..C.....AC..AGCTT.C....C...T..T................CA.  Mmulatta
  422545: ........AC..GGTTTAC....C...T..T................CA.  Panubis
  446549: GG..GAC.AC..GAATCAC....C.....C.................CA.  Cjacchus
 1085892: TGA..AC.AC..G..TCAC....C...------..............CA.  Sboliviensis

                                  B4:1
          <--------------------------------------------------
                    124174    124184    124194    124204    124214
                         :         :         :         :         :
  124165: TGTAATCACCCTTTCCTATTCTTCATAAGGCAAAATAAGACCAACAGCAT  Hsapiens
  443871: ..................................................  Ptroglodytes
  460907: ..................................................  Ggorilla
  496770: ......T...................C....-..................  Pabelli
  385310: ..........................C.......................  Nleucogenys
  475700: ..........................C.......................  Mmulatta
  422595: ..........................C.......................  Panubis
  446599: ..........................C.......................  Cjacchus
 1085936: ..........................C.................T.....  Sboliviensis
```

```
                          B4:1
                  <-------------------------------
                124224    124234    124244    124254    124264
                     :         :         :         :         :
  124215: CCTCTTCTATGGCAGATTCTCATCCCCATAGATGCAATTAGTCTGTCACT  Hsapiens
  443921: ..........................TG......................  Ptroglodytes
  460957: ........................A..G......................  Ggorilla
  496819: ...........................G......................  Pabelli
  385360: ........T..................T......................  Nleucogenys
  475750: ........T..................T......................  Mmulatta
  422645: ........T..................T......................  Panubis
  446649: ........T..................T......................  Cjacchus
 1085986: ........T..................T....C.................  Sboliviensis

                124273    124283    124293    124303    124313
                     :         :         :         :         :
  124265: CCATTTAG-AAAATGTTCACCTAGAGGTGTTCTGGTAAACTGATTGCTGG  Hsapiens
  443971: ........-.................A.......................  Ptroglodytes
  461007: ........-.........................................  Ggorilla
  496869: ...C....-................A........................  Pabelli
  385410: ........-.........................................  Nleucogenys
  475800: ......G.-................................C.C......  Mmulatta
  422695: ......G.-................................C........  Panubis
  446699: ..T...G.C..............C..TC......................  Cjacchus
 1086036: ..T...G.C........G.....C..TC......................  Sboliviensis

                124323    124333    124343    124353    124363
                     :         :         :         :         :
  124314: CAACAACAGATTCTCTTGGCTCATATTTCTTTTCTATCTCATCTTGATGA  Hsapiens
  444020: ..................................................  Ptroglodytes
  461056: ...................................T..............  Ggorilla
  496918: ......................T.................C.........  Pabelli
  385459: ..........C..A....A...............A.....----------  Nleucogenys
  475849: .................CA...............A.....----------  Mmulatta
  422744: .................CA...............A.....----------  Panubis
  446749: ..................A........--.....A.....----------  Cjacchus
 1086086: ..................A..............TA.....----------  Sboliviensis

                          124371    124381    124391    124401
                               :         :         :         :
  124364: TGATAGT------------CATCATCAAGAATTTAATGATTAAAATAGCA  Hsapiens
  444070: .......------------...............................  Ptroglodytes
  461106: .......------------...............................  Ggorilla
  496968: .......------------..C............................  Pabelli
  385499: --.....------------......................G........  Nleucogenys
  475889: --.....------------......................G......T.  Mmulatta
  422784: --.....------------......................G......T.  Panubis
  446787: --.....GTCATTCTTGTCA...........C.........G.....A.G  Cjacchus
 1086126: --.....------------......................G.....A..  Sboliviensis

                124411    124421    124431    124441    124451
                     :         :         :         :         :
  124402: TGCCTTTCTCTCTTTCTCTTAATAAGCCCACATATAAATGTACTTTTCCT  Hsapiens
  444108: ...............................................T..  Ptroglodytes
  461144: ...............................................T..  Ggorilla
  497006: ........--.....................C......C........T..  Pabelli
  385535: .A.A.....T..........T.....AAG.........CC.......T..  Nleucogenys
  475925: .........T..........T.....AA..........CC...A...T..  Mmulatta
  422820: .........T..........T.....AA..........CC.T.A...T..  Panubis
  446835: ..................C.T....A.A.......--.CC.......T..  Cjacchus
 1086162: ....................T....A.A.......--.CC.......T..  Sboliviensis
```

```
                 124461      124471      124481      124491      124501
                      :           :           :           :           :
  124452: TCCAGAAAAATTTCCCTTGAGGAAAAATGTCCAAGATAAGATGAATCATT   Hsapiens
  444158: C........................................C........   Ptroglodytes
  461194: ............CT..................................C.   Ggorilla
  497054: .......G....CT..............A............C........   Pabelli
  385585: ............CT............G.--................AT..   Nleucogenys
  475975: .......G....CT..G.....................C.......A...   Mmulatta
  422870: .......G....CTG.G.....................C.......A...   Panubis
  446883: ....T.......CT..............--------C.........AT..   Cjacchus
 1086210: .G..........CT..............--------C.........AT..   Sboliviensis

                 124511      124521      124531      124541      124551
                      :           :           :           :           :
  124502: TAATACCGTGTCTTCTAAATTTGAAATATAATTCTGTTTCTGACCTGTTT   Hsapiens
  444208: ......T..A.............................G..........   Ptroglodytes
  461244: .........A...................G....................   Ggorilla
  497104: ......T..A.............................G..........   Pabelli
  385633: ......TC.AC.A...................--.....G..........   Nleucogenys
  476025: ......T..A.................G...........G..........   Mmulatta
  422920: ......T..A.................G...........G..........   Panubis
  446925: ......TC.A..............T.C............G..........   Cjacchus
 1086252: ......TC.A..............T.C............G.....C....   Sboliviensis

                 124561      124571      124581      124591      124601
                      :           :           :           :           :
  124552: TAAATGAACCAAACCAAATCATACTTTCTCTTCAAATTTAGCAACCTAGA   Hsapiens
  444258: ....................G...G..T....TG................   Ptroglodytes
  461294: ...........................T....TG................   Ggorilla
  497154: ......................G..........G................   Pabelli
  385681: ......G....CG.......................C.............   Nleucogenys
  476075: ......G...........................G.CG............   Mmulatta
  422970: ......G...........................C.C.............   Panubis
  446975: ..G......................................T........   Cjacchus
 1086302: ..T...................................G.........T.   Sboliviensis

                 124611      124621      124631      124640      124650
                      :           :           :           :           :
  124602: AACACACATTTCTTTGAATTTAGGTGATACCT-AAATCCTTCTTATGTTT   Hsapiens
  444308: ................................-...........C.....   Ptroglodytes
  461344: ................................-.................   Ggorilla
  497204: ...C............................G.................   Pabelli
  385731: C..C............................G.................   Nleucogenys
  476125: ...C...................T........G............C....   Mmulatta
  423020: ...C...............G...T........G............C....   Panubis
  447025: ...C..T.........................G.................   Cjacchus
 1086352: ...C..T.........................G....T..A.........   Sboliviensis

                 124660                              124674      124684
                      :                                   :           :
  124651: CTAAATTTTGTGATTCT----------------ATAAAACACATCATCAA   Hsapiens
  444357: .................----------------.................   Ptroglodytes
  461393: .................----------------.................   Ggorilla
  497254: .................ATAAAATACATCATCA......T..........   Pabelli
  385781: .................ATAAAATACATCATCA......T..........   Nleucogenys
  476175: .................----------------......T..........   Mmulatta
  423070: .................----------------......T..........   Panubis
  447075: .................----------------......T....T.....   Cjacchus
 1086402: .................----------------......T....T.....   Sboliviensis
```

```
             124694    124701    124711    124721    124731
 124685: TAAAATAATGACATA---AAATCATTTTTGCTTTACCTGTTTTCTCTCTG  Hsapiens
 444391: .......G.......---....................T......T....  Ptroglodytes
 461427: .......G.......---....................T...........  Ggorilla
 497304: ............T..CTT....................T.........A.  Pabelli
 385831: ............T..CTT....................T.........A.  Nleucogenys
 476209: ............T..CTT..................T.T.........A.  Mmulatta
 423104: ............T..CTT..................T.T.........A.  Panubis
 447109: .....C......T..CTT....A...............T.........A.  Cjacchus
1086436: ............T.----....A...............T.........A.  Sboliviensis

             124741    124751    124761    124771    124781
 124732: GAAAGGGCAAGTGTCCAGTTACACATAGGAAAGATAATTTAGAGATATAT  Hsapiens
 444438: .......G..........................................  Ptroglodytes
 461474: .......G.................C........C..............C  Ggorilla
 497354: ......AG.........C..........C..............A......  Pabelli
 385881: ......AG..T......C.......C.................A......  Nleucogenys
 476259: .......G.........C......G..................A......  Mmulatta
 423154: .......G.........C...T..G..................A......  Panubis
 447159: T.T....G...............T....A...A..........A......  Cjacchus
1086482: T.C....G...............T........A..........AG..G..  Sboliviensis

             124791    124801    124810    124820    124830
 124782: TAATCATATATAAAGGAAAATTAAAAAC-AGAGTAGTTCATGATGAGCCT  Hsapiens
 444488: ............................-.....................  Ptroglodytes
 461524: ......C...C.................-.....................  Ggorilla
 497404: ..........C...........C.....A..............C..A...  Pabelli
 385931: ..........C...........C.....A...........C.....A...  Nleucogenys
 476309: ..........C...........C.....A..............CC.....  Mmulatta
 423204: ..........C...........C.....C..............CC.....  Panubis
 447209: .......G..G...........C.....A.....G......A.G......  Cjacchus
1086532: ....T..G..G........T..C.....A..G..G......A.A......  Sboliviensis

                                           LINE2
                          ----------------------------------->
             124840    124850    124860    124870    124879
 124831: GGAGTAGAAGGCATATCCCAGAACAGGAGGAGCCTTGTAAACCACA-TAG  Hsapiens
 444537: ..............................................T...  Ptroglodytes
 461573: ..............................................T...  Ggorilla
 497454: .............G.....G.....T..A..-..........T...T...  Pabelli
 385981: .............G...T.......TA...................T...  Nleucogenys
 476359: .............G...T......TT....................T...  Mmulatta
 423254: .............G...T......TT....................T...  Panubis
 447259: .C...........G......-....T....................T.G.  Cjacchus
1086582: .............G......-....T.....A...........G..T..A  Sboliviensis

                                LINE2
         ------------------------------------------------->
             124889              124908    124918    124928
 124880: GAACTTCCTATTTTATGCT-AAAGGGATAAGAAACTCATTACAGGCTTTG  Hsapiens
 444587: ...................-..............................  Ptroglodytes
 461623: ...................-..............................  Ggorilla
 497503: A.....T............A..........T..G.......G........  Pabelli
 386031: A.....T............A.T........T..G.......G........  Nleucogenys
 476409: A.....T............A.............G.....C.G........  Mmulatta
 423304: A.....T............A.............G.......G........  Panubis
 447308: ......TT...........A.............G...........T..--  Cjacchus
1086631: ......T..........G.A.....C.......G...........T..--  Sboliviensis
```

```
                    124939    124949    124959    124969
                         :         :         :         :
  124929: A---------TGGTTGTTTGTCAAAGAGGGGCATAAAATTATCATATCCA  Hsapiens
  444636: .---------........................................  Ptroglodytes
  461672: .---------........................G.............TT  Ggorilla
  497553: .TTGTT----..A...................................TG  Pabelli
  386081: .TTGTT----..A...................................TG  Nleucogenys
  476459: .TTATTTGAT..T..A...............G........---.....TG  Mmulatta
  423354: .TTGTTTGAC..T..A...............G........---.....TG  Panubis
  447356: ----------------.....T.................G........TG  Cjacchus
 1086679: ---------------......T..........................TG  Sboliviensis

          124979    124989    124999    125009    125019
               :         :         :         :         :
  124970: CATCTAGAAAATACATCTCTGGCTACGCTGATATCAATGGATGCGAGGAA  Hsapiens
  444677: ............................................A.....  Ptroglodytes
  461713: .....T................T...T................A......  Ggorilla
  497599: .....T....................T..A......G......A......  Pabelli
  386127: .....T....................TG.A.C....G......A......  Nleucogenys
  476506: .....T.G.......------------..A............AA..A...  Mmulatta
  423401: .....T.........------------..A............AA..A...  Panubis
  447390: ...T.T....................T.........G.A....A......  Cjacchus
 1086714: .....TA...................T...........A....A......  Sboliviensis

          125029    125039    125049    125059    125069
               :         :         :         :         :
  125020: AGAACAGTGTGGTTACCATATATAAATTAGGAAATCATTAGAGTATTGGG  Hsapiens
  444727: .C...............................G................  Ptroglodytes
  461763: ..................................................  Ggorilla
  497649: ...G..C....A..G...C...............C...............  Pabelli
  386177: ......C...........C...............C...............  Nleucogenys
  476544: .................G................C...............  Mmulatta
  423439: .................G................C...............  Panubis
  447440: TA............G.T............C....C....T..........  Cjacchus
 1086764: TA..............T.................A....T..........  Sboliviensis

          125079    125089    125099    125109    125119
               :         :         :         :         :
  125070: AGTGGAAATGGAGAGAAAGAAAGAGCCTGGGGGAATTATTTAGGAAATAA  Hsapiens
  444777: ...A..................C...........................  Ptroglodytes
  461813: G.........A...........C........A..............G...  Ggorilla
  497699: ......................C.........A..........A..G...  Pabelli
  386227: ......................C.........A..........A..G...  Nleucogenys
  476594: ......................TG........A..........A..G...  Mmulatta
  423489: ......................TG......-.A..........A..G...  Panubis
  447490: ......................C....CA.A......G........G..G  Cjacchus
 1086814: ......................C.......A...............G...  Sboliviensis

          125129    125139    125149    125159    125169
               :         :         :         :         :
  125120: TAGTTACAGAAAGACATCTAAGTTGCTGACCTATCTGACTGGATGGATGG  Hsapiens
  444827: ................................................A.  Ptroglodytes
  461863: C.....A.A...............T....G.............A......  Ggorilla
  497749: C....G..................T........G.CA.T.A.T..C....  Pabelli
  386277: .....G..................T.......GG.CA.T..CT..C....  Nleucogenys
  476644: .....G................A.T........G.CA.....T..C....  Mmulatta
  423538: .....G................A.T........G.CA.....T..C....  Panubis
  447540: ...C.G............C.G...T........G.C-.T...T.......  Cjacchus
 1086864: ...C.G...T........C.G...T........G.CA.T...T.......  Sboliviensis
```

```
                                   LINE2
             125179    125189    125199    125203    125211
 125170: AAGAATATCTTGTTTCTGAGAGAAAAAAAGA------CTTTGGG--TTTA  Hsapiens
 444877: ...............................------.......--....  Ptroglodytes
 461913: .....A..G......T........G....AG-----A.CA...A--....  Ggorilla
 497799: .....A..G                                           Pabelli
 498140:          ......T...C.........A.----GAACA...A--....  Pabelli
 386327: ....GA..G                                           Nleucogenys
 386668:          ......T...C.........A.AAAAGATCA...A--....  Nleucogenys
 476694: .....A..G.....G...                                  Mmulatta
 477032:                   .C.........A.-AAACATCA....--....  Mmulatta
 423588: .....A..G                                           Panubis
 423917:          ......T...C.........A.----CATCA....--....  Panubis
 447589: .....A..G.....                                      Cjacchus
 447936:               .T...............------TCAC...--...C  Cjacchus
1086914: .....A..G.....GT..                                  Sboliviensis
1087260:                   .............------TCA....TA.A..  Sboliviensis

                            LINE2
             125221              125240    125250    125260
 125212: AATTTGTACTTGATGAATT-AAGGTACTTTTAATATTCAAATGGATTTGC  Hsapiens
 444919: ...................-G.............................  Ptroglodytes
 461956: ...................-G.............................  Ggorilla
 498175: ............T......TG............G................  Pabelli
 386707: ............T......TG............G................  Nleucogenys
 477061: .G.........AT......TG............G...............T  Mmulatta
 423952: .G.........AC......TG............G...............T  Panubis
 447964: T..A........T......TG............G................  Cjacchus
1087286: T..A.A......T......TG............G................  Sboliviensis

                            LINE2
             125270    125280    125290    125300    125310
 125261: CTGGCAGGCACTTGAAGATATTAGTCTAAATCTCAGAAACAGAATATGAT  Hsapiens
 444968: ...........................................G......  Ptroglodytes
 462005: ........T....                                       Ggorilla
 462418:                                                ...  Ggorilla
 498225: .C................................................  Pabelli
 386757: ........................C..-.......A..............  Nleucogenys
 477111: ...A....T...........C.............................  Mmulatta
 424002: ...A................C.............................  Panubis
 448014: ...............................................T..  Cjacchus
1087336: ..................................................  Sboliviensis

                           LINE2          LINE2
             125320    125330    125340    125350    125360
 125311: CTGAAGCTCTAAATTTGTGATATTCAATATAAATACTTTAGAGTCATTGG  Hsapiens
 445018: ..................................................  Ptroglodytes
 462421: .......................G...C................T..C..  Ggorilla
 498275: ..................................C.........T.....  Pabelli
 386806: ............................................T.C...  Nleucogenys
 477161: .....A......................................T....A  Mmulatta
 424052: .....A......................................T.....  Panubis
 448064: ...................T.................C......T.C.A.  Cjacchus
1087386: ...................-.................C......T.C...  Sboliviensis
```

```
                                  LINE2
          ------------------------------------------------------->
                  125370    125380    125390    125400    125410
                       :         :         :         :         :
  125361: GATAAATATGGTAGTTGTAGCTAAAAGCAAAAATAAGATACTAGGGAGAA  Hsapiens
  445068: ..................................................  Ptroglodytes
  462471: .............A.........G.......G....T..C..........  Ggorilla
  498325: .....G...A...A............A....G.......C..........  Pabelli
  386856: .....G...A...AC................G.......C..........  Nleucogenys
  477211: .....G...A...A.................G.......C..........  Mmulatta
  424102: .....G..CA...A.................G.......C..........  Panubis
  448114: .............AC................G.......C..........  Cjacchus
 1087435: .............AC................G.......C.....A....  Sboliviensis

                                  LINE2
          ------------------------------------------------------->
                  125420    125430    125440    125450    125460
                       :         :         :         :         :
  125411: AGGATAAAGTTAGAAGAAAGAAGAATCTAGAATTGACCTTGAAGTATATC  Hsapiens
  445118: ..................................................  Ptroglodytes
  462521: ..................................................  Ggorilla
  498375: ....G.........G..........C.......................G  Pabelli
  386906: ......C.......................................G...  Nleucogenys
  477261: ............................................G.....  Mmulatta
  424152: ............................................G.....  Panubis
  448164: ......T--...................GT...CA..T..........C.  Cjacchus
 1087485: ........................C...GTC.AC..............C.  Sboliviensis

                  LINE2
          --------------------->
                  125470    125480    125490    125500    125510
                       :         :         :         :         :
  125461: AGCATGTGTAAAGATCAGGAATTGATCATTTTTATTTTCCAGAAAGTAGC  Hsapiens
  445168: ..................................................  Ptroglodytes
  462571: ..................................................  Ggorilla
  498425: .AT...............................................  Pabelli
  386956: .A................................................  Nleucogenys
  477311: ..T............................C..................  Mmulatta
  424202: ..T...............................................  Panubis
  448212: .AT.........-.C........................T..........  Cjacchus
 1087535: .AT.........-.C....T.C......C..........T..........  Sboliviensis

                  125520    125530    125540    125550    125560
                       :         :         :         :         :
  125511: TTTTCTTAGGGTTCCATATTTACTCCCATAGATTCTTCCCAGTCAGTACT  Hsapiens
  445218: .......G..........................................  Ptroglodytes
  462621: .......G..........................................  Ggorilla
  498475: .........A........................................  Pabelli
  387006: ..................................................  Nleucogenys
  477361: ........A....................................T....  Mmulatta
  424252: ............................C.....................  Panubis
  448261: ..................G.......T.............T.........  Cjacchus
 1087584: ..................G...................A...........  Sboliviensis
```

```
             125570    125580    125590    125600    125610
                  :         :         :         :         :
 125561: GAATCCCACCTGGTTTCATAAAACATAAGTTTTAGAGAATTTGAACAAGG  Hsapiens
 445268: ................................G.................  Ptroglodytes
 462671: ..................................................  Ggorilla
 498525: ..............................................G...  Pabelli
 387056: .......T....C.....G............G..................  Nleucogenys
 477411: ......T................-..........................  Mmulatta
 424302: ......T................-..........................  Panubis
 448311: ..G.......C........G......G......T...G......G.....  Cjacchus
1087634: ..G...T...................GG.....T...G......G.....  Sboliviensis

             125620    125630    125640    125650    125660
                  :         :         :         :         :
 125611: TTGAAGTTTTCTTCAGCAAGATTCAGCAGTCCAAGATGAAGGCAGAGGAA  Hsapiens
 445318: ..................................................  Ptroglodytes
 462721: ..................................................  Ggorilla
 498575: .................................GA..T...AG.-.....  Pabelli
 387106: .....................................T.....-......  Nleucogenys
 477460: .....................G...........G...T............  Mmulatta
 424351: .....................G...........G...T............  Panubis
 448361: .....A.............................G.C.........A..  Cjacchus
1087684: .....A....T......................G...C.........A..  Sboliviensis

             125670    125680    125690    125700    125710
                  :         :         :         :         :
 125661: ACAAATGTTTCACCACTTAACTATGAGACTTGGTCAAGTTGTCGAGGGAA  Hsapiens
 445368: .................C........................T.......  Ptroglodytes
 462771: ..................................AG..............  Ggorilla
 498624: .................................C................  Pabelli
 387155: ................A..........................AG.....  Nleucogenys
 477510: .......................C..........................  Mmulatta
 424401: .........................................CG.......  Panubis
 448411: ...........C..............................T.......  Cjacchus
1087734: ...........C..............................T.......  Sboliviensis

             125720    125730    125740    125750    125760
                  :         :         :         :         :
 125711: TTGACAGACTAGCCAGAGAGTGTATAACATCAACAATCTGTTCAGTGAAT  Hsapiens
 445418: ............................C.G...................  Ptroglodytes
 462821: ............................C.G...................  Ggorilla
 498674: ........................G.....G...................  Pabelli
 387205: ..................G...........G..................C  Nleucogenys
 477560: ..............................G...................  Mmulatta
 424451: ..............................G...................  Panubis
 448461: ......................GG......G...C..............C  Cjacchus
1087784: ......................G.......G...................  Sboliviensis

             125770    125780    125790    125800    125810
                  :         :         :         :         :
 125761: ACAGCTGGGGAAAGAAAGCAAAGAAGGATGTAATAGTCTTGAAGAACACA  Hsapiens
 445468: GG................................................  Ptroglodytes
 462871: GG................................................  Ggorilla
 498724: GG.....A.......................GG...............G.  Pabelli
 387255: GG...C.A...................C...GG...............G.  Nleucogenys
 477610: GG..........G..................GG...............G.  Mmulatta
 424501: GG..........G..................GG.............T.G.  Panubis
 448511: G........................A..A..GG...............G.  Cjacchus
1087834: G......A..................C....GG.............T.G.  Sboliviensis
```

```
             125820    125830    125840    125850    125860
                  :         :         :         :         :
 125811: TGTGCATTACAATACTTACTGAGGTCAAATAAGGATGGTATTGAGTTTAT  Hsapiens
 445518: ..................................................  Ptroglodytes
 462921: ..................................................  Ggorilla
 498774: ......................................C...........  Pabelli
 387305: ..................................................  Nleucogenys
 477660: ..................................................  Mmulatta
 424551: ...A...............................C..............  Panubis
 448561: ....T.......C.....A.........G................AC.G.  Cjacchus
1087884: ....T.C.....C.....A...............................  Sboliviensis

             125870    125880    125890    125900    125910
                  :         :         :         :         :
 125861: GAGAAAGATGAAGAGGTCAGAAGTTTGGGCCAGGACCTATGATTTTGAAG  Hsapiens
 445568: ...G..............................................  Ptroglodytes
 462971: ..................................................  Ggorilla
 498824: ......T.............................A.......C.....  Pabelli
 387355: ......T.A.............................G...........  Nleucogenys
 477710: ......T...........................TT..............  Mmulatta
 424601: ......T............................T..............  Panubis
 448611: ......T........A..........T...T...................  Cjacchus
1087934: ....------------..........T...T...................  Sboliviensis

             125920    125930    125940    125950    125960
                  :         :         :         :         :
 125911: TATGCTTTAAAATGATAAATTGTAAGGAATTATAAAGCTCAAATTCTGTC  Hsapiens
 445618: ...A..............................................  Ptroglodytes
 463021: ..CA.............C................................  Ggorilla
 498874: .G.A...........................................T..  Pabelli
 387405: .G.A..............................................  Nleucogenys
 477760: .G.A...........................G.G.............T..  Mmulatta
 424651: .G.A..........................CG.G.............T..  Panubis
 448661: ...A....................................T.........  Cjacchus
1087972: ..CA....TC......................A.......T.........  Sboliviensis

             125970    125980    125990    126000    126010
                  :         :         :         :         :
 125961: CAATAGGTGAGGATTAATTTCTAATGATTCAATGAAGCTGAAAATGGTAG  Hsapiens
 445668: ...........................................T......  Ptroglodytes
 463071: ........A.....C........G..........................  Ggorilla
 498924: ..G....................C....G.....................  Pabelli
 387455: ...C..............................................  Nleucogenys
 477810: ...........A...................G.................C  Mmulatta
 424701: ...A.......A...................G.................C  Panubis
 448711: A.....A....A.G.........................T..........  Cjacchus
1088022: AC....A....A......................................  Sboliviensis

             126020    126030    126040    126050    126060
                  :         :         :         :         :
 126011: AGTGGATAGTTTTGAAGAACTGATTGTTAGAATCACTTTAGACCATATCC  Hsapiens
 445718: .................................................T  Ptroglodytes
 463121: .........................A........................  Ggorilla
 498974: .........................T.......................T  Pabelli
 387505: ....................A............................T  Nleucogenys
 477860: ....................................--.G....G...TT  Mmulatta
 424751: ....................................--.G...TG...TT  Panubis
 448761: ......A.....C...................AG...............T  Cjacchus
1088072: ...A..A..........................G...............T  Sboliviensis
```

```
             126070    126080    126090    126100    126110
                  :         :         :         :         :
  126061: TTCAACTATTATTAATTTTTCTGGGTTTAAAAGAAGGTACTTTTTAATAA  Hsapiens
  445768: ..................................................  Ptroglodytes
  463171: ..................................................  Ggorilla
  499024: .....................................G.....A......  Pabelli
  387555: .....................CA..............G.....AC.....  Nleucogenys
  477908: ......A................A.............G.....A......  Mmulatta
  424799: ......A................A.............G.....A......  Panubis
  448811: ......---....................G.......GG...CAG.....  Cjacchus
 1088122: ....G.---....................G.......G....CCG.....  Sboliviensis

             126120    126129    126139    126149    126159
                  :         :         :         :         :
  126111: TGAAAATGTTAAGACAAA-GCTCATAAGCATTAGATTCTTATAGAGGAGA  Hsapiens
  445818: ..................-...............................  Ptroglodytes
  463221: ..................-...............................  Ggorilla
  499074: ..................G.....G.T........G.....C-...T...  Pabelli
  387605: ....C.............G.....G.T........G......-...T...  Nleucogenys
  477958: ..................G................G..............  Mmulatta
  424849: ..................G.T..............G..............  Panubis
  448858: ..................G.............G..G.T........T.T.  Cjacchus
 1088169: ..............T...G........T.......G.T........T.T.  Sboliviensis

             126169    126179    126189    126199    126209
                  :         :         :         :         :
  126160: GGGCATAGTTGATATTTTGTTAGGAGAATTGAAATATTTACATACATTCT  Hsapiens
  445867: ..................................................  Ptroglodytes
  463270: .........................................G........  Ggorilla
  499123: .............G...........A...............G..T.....  Pabelli
  387654: .............G.........A.................G..T.....  Nleucogenys
  478008: .......C...........................G..............  Mmulatta
  424899: .......C..A........................G..............  Panubis
  448908: ......................T..T...............T..T....C  Cjacchus
 1088219: ....................C.T..................T..T....C  Sboliviensis

             126219    126229    126239    126249    126259
                  :         :         :         :         :
  126210: AGGCCCACCAGAAAACCAGTTGCCCCTGCATTGTAGCCATCTGCAAGAGA  Hsapiens
  445917: .............C....................................  Ptroglodytes
  463320: ...............................................G..  Ggorilla
  499173: .....A...............A............................  Pabelli
  387704: .....................A.A..........................  Nleucogenys
  478058: .....................A.A...........A..............  Mmulatta
  424949: ........G..............A...........A..............  Panubis
  448958: ..-----...........A..A.A..........G...........A...  Cjacchus
 1088269: ..-----...........A..A.A..........G...........A...  Sboliviensis

             126269    126279    126289    126299    126309
                  :         :         :         :         :
  126260: CACTGAAGGGAAAGCCAAATTTTTTGTCTTGATTTTTTAAATAATGAATT  Hsapiens
  445967: ...C..............................................  Ptroglodytes
  463370: ......CA......T............T......................  Ggorilla
  499223: ..GG....................G..T......................  Pabelli
  387754: ...........................T......................  Nleucogenys
  478108: ...........................T........C.............  Mmulatta
  424999: ....................G......T........C.............  Panubis
  449003: A..CA............T.........T.........A............  Cjacchus
 1088314: A..A...A.........C.........T.........A.....C..T...  Sboliviensis
```

```
             126319    126329    126339    126349    126359
                  :         :         :         :         :
 126310: AAAATCTATTGAGTAAAACAGAATACGAGTCCCTGCTCATATAAAATAAA  Hsapiens
 446017: ......C.....A........................G............  Ptroglodytes
 463420: ............A........................G..........C.  Ggorilla
 499273: ............A...............A........G..........--  Pabelli
 387804: ............A............T...........G............  Nleucogenys
 478158: ...G........A.............A.A...-....G..........C.  Mmulatta
 425049: ...G........A........C........A......G..........C.  Panubis
 449053: .......GC...AC.......C..GT......A....G.....C......  Cjacchus
1088364: .......GC.T.AC.......C...TA.....AC...G.....C......  Sboliviensis

             126369    126379    126389    126399    126409
                  :         :         :         :         :
 126360: TGACTAAATTAAAAGTTTCATAAGAAATGCAAAATTTAAGTGCTTTCAAA  Hsapiens
 446067: .................................T................  Ptroglodytes
 463470: .....C............................................  Ggorilla
 499321: --.......C........G....T..........................  Pabelli
 387854: ..........................................T.......  Nleucogenys
 478207: .A............T...G............C.T.....A..........  Mmulatta
 425099: .A............T...G............C.T.....A..........  Panubis
 449103: ..................A........G......................  Cjacchus
1088414: ..................A........G......................  Sboliviensis

                                 LINE1
                  ---------------------------------------->
             126419    126429    126439    126449    126459
                  :         :         :         :         :
 126410: ACTCCTCCTCATGGAATACCACTTTGTACCAATTATGATGGCCAAAATTA  Hsapiens
 446117: .........................................T........  Ptroglodytes
 463520: .........................................           Ggorilla
 499369: ........C...........................C.............  Pabelli
 387904: T.......C.......................C.................  Nleucogenys
 478257: ......G.C........G................................  Mmulatta
 425149: ......G.C........G................................  Panubis
 449153: ...T....C..C............C...TGC......G.........A..  Cjacchus
1088464: ...T....C.................C.TGC......G......G..A..  Sboliviensis

                            LINE1
         ------------------------------------------------->
             126469                        126482    126492
                  :                             :         :
 126460: AAACTTATAATACTGGGC-----------------CATTGCAAGTTTTGA  Hsapiens
 446167: ............G..A..-----------------...............  Ptroglodytes
 499419: .......A.......A..-----------------...............  Pabelli
 387954: .......A.......A..-----------------...............  Nleucogenys
 478307: .......A......A...-----------------...............  Mmulatta
 425199: .....G.A......A...-----------------...............  Panubis
 449203: ...T...A........A.TTTTAAAAACTTCAAAAT.........G.C..  Cjacchus
1088514: ...T...A...T....A.-----------------T..............  Sboliviensis

                            LINE1
         ------------------------------------------------->
             126502    126512    126522    126532    126542
                  :         :         :         :         :
 126493: AAAGAATGTGAAGCAACTGGAACTCTCACAAACTGCTGATGGAAATACAA  Hsapiens
 446200: ..................................................  Ptroglodytes
 499452: ...A..........................G...................  Pabelli
 387987: ...AC..T......................C...................  Nleucogenys
 478340: ...T..C......A.............G..C...................  Mmulatta
 425232: ...T..C......A.............G..C...................  Panubis
 449253: .......A.....T..T.....TG......C..........AG.......  Cjacchus
1088547: .......A........T.....TG......C..........AG...T..  Sboliviensis
```

```
                                   LINE1
                 ------------------------------->
                       126561     126571     126581     126591
                            :          :          :          :
  126543: AAGGTGCAA-CCATGTTGGAAAACAGTCTTAAATCACTGAACTACACACT  Hsapiens
  446250: .........-........................................  Ptroglodytes
  499502: ..A......-...C......................T...T.........  Pabelli
  388037: .........C...C......................T...T.........  Nleucogenys
  478390: .........-...C...........C..........T.A.T...T.....  Mmulatta
  425282: T........-...C...........C..........T.A.T...T..G..  Panubis
  449303: ..T......-...CA.................G..G..............  Cjacchus
 1088597: .........-...C..................G...T.............  Sboliviensis

             126601     126611     126621     126631     126641
                  :          :          :          :          :
  126592: TACATTTTATTTTAATTAATTATGCCTCTATAAAGTTGACTCAAAAGAGG  Hsapiens
  446299: ..................................................  Ptroglodytes
  499551: ..A.........C.T...C...............A...........A.T.  Pabelli
  388087: ..A.........................C.................A.T.  Nleucogenys
  478439: ..A...............................A...........A...  Mmulatta
  425331: ..A.....T...A...G............G....A......T....A...  Panubis
  449352: ..-------...............................C.....A...  Cjacchus
 1088646: ..A.....................................C.....A.A.  Sboliviensis

             126651     126661     126671     126679     126689
                  :          :          :          :          :
  126642: AAAAAGAGAATTGTCCAAAACACACATCTTTAGGTG--AAAAAATCTTCT  Hsapiens
  446349: ..............................C.....--............  Ptroglodytes
  499601: .....A.....................T........--............  Pabelli
  388137: ........................T...........--............  Nleucogenys
  478489: T...........C.......................AA............  Mmulatta
  425381: T...........C.......................AA............  Panubis
  449395: ....................T......A........--.......A....  Cjacchus
 1088696: ......G....C...............A........--.......A....  Sboliviensis

                                                  LINE1
                                     -------------------------->
             126699     126709     126719     126729     126739
                  :          :          :          :          :
  126690: TCATTAAATGTTGTTTTTAAATGTATTTTTAATTAATAAAAACTGTATAC  Hsapiens
  446397: ...........G......................................  Ptroglodytes
  499649: .......C....A.............................T......T  Pabelli
  388185: ..................................................  Nleucogenys
  478539: ..................-....................C.......C..  Mmulatta
  425431: ..................-....................C.......C..  Panubis
  449443: .........AC..G..........G.........G...........G...  Cjacchus
 1088744: .........AC..G....................GG...-..........  Sboliviensis

                                        LINE1
                 -------------------------------------------------->
             126749     126759     126769     126779     126789
                  :          :          :          :          :
  126740: ATTTATGTTGTAAAACATGTTGTTTTGAAATATGTATACATAGTGGAATG  Hsapiens
  446447: ..................................................  Ptroglodytes
  499699: .................................A..............C.  Pabelli
  388235: ..................................................  Nleucogenys
  478588: ............................................A.....  Mmulatta
  425480: ......................................T.....A.....  Panubis
  449493: ......C..A..C.................C.....G.............  Cjacchus
 1088793: ......C..A..C.................C.....G.T.C.........  Sboliviensis
```

```
                                   LINE1
         ──────────────────────────────────────────────────►
              126799    126809    126819    126829    126838
 126790: AGTCAGTAGAGCTAATTTATATATACATTACCTCACATAA-TTTTTTGTG  Hsapiens
 446497: ........................................-.........  Ptroglodytes
 499749: .................................T......-.........  Pabelli
 388285: ........................................-.........  Nleucogenys
 478638: ........................................-.........  Mmulatta
 425530: ........................................-.........  Panubis
 449543: ........A........A......G...............T.........  Cjacchus
1088843: .................A......G...............T.........  Sboliviensis
```

```
                                   LINE1
         ──────────────────────────────────────────────────►
              126847              126855    126865    126875
 126839: G-TAAGAACACTGCAA------------AGCAATTTTCAAAAATAAAATG  Hsapiens
 446546: .-..............------------......................  Ptroglodytes
 499798: .-.G............GTCTATTCTCTT............C.........  Pabelli
 388334: .-..............GTCTATTCTCTT............C.........  Nleucogenys
 478687: .-..............GTCTACTCTATTG.....................  Mmulatta
 425579: .-..............GTCTACTCTATTG.....................  Panubis
 449593: .T...A.......A..GTCTGGTCTCTT......................  Cjacchus
1088893: .T...A.......A..GTCTAGTCTCTT......................  Sboliviensis
```

```
                                   LINE1
         ──────────────────────────────────────────────────►
              126885    126895    126905    126915    126925
 126876: CATTAACTATAGTCACCATGTTGTAAAATAGACCTTCTGAACTCATGTCT  Hsapiens
 446583: ................................T.................  Ptroglodytes
 499847: ...........................................T..T...  Pabelli
 388383: .................G.........................T..T...  Nleucogenys
 478736: .........................C.........C.......T..T...  Mmulatta
 425628: ......T..................C.........C.......T..T...  Panubis
 449643: A........................C.........C.......T.CT...  Cjacchus
1088943: A........................C.........C.......T.CT...  Sboliviensis
```

```
                                   LINE1
         ──────────────────────────────────────────────────►
              126935    126945    126955    126965    126975
 126926: CCTGTTTAACTGAAATTTGTATCCTTTGACCAACATCTCCTCAACTCCCT  Hsapiens
 446633: ............................T.....................  Ptroglodytes
 499897: ............................................T.....  Pabelli
 388433: .T........................C....G............T....-  Nleucogenys
 478786: ......................T...CA................T....C  Mmulatta
 425678: ..........................CA................T.....  Panubis
 449693: ...A................................---.....T.....  Cjacchus
1088993: ...A.............................T..---.....T.....  Sboliviensis
```

```
                                   LINE1
         ──────────────────────────────────────────────────►
              126985    126995    127005    127015    127025
 126976: CTCACATCCCGGCTCCTGGTAACCACCATTTTACTCTTTATTTATATGAG  Hsapiens
 446683: ......C...........................................  Ptroglodytes
 499947: ......C..TA....A.A........A.......................  Pabelli
 388482: ......C...A....A..........A.............A.........  Nleucogenys
 478836: ......C...A....A.....G....A.......................  Mmulatta
 425728: ......C...A....A.....G....A.......................  Panubis
 449740: ....TTC..T...........G.........C.T.....G.....--..C  Cjacchus
1089040: .....TC...........A..G.........C.T............C...  Sboliviensis
```

```
                          LINE1
         ------------------------------------------------->
              127035    127045    127055    127065    127075
                   :         :         :         :         :
 127026: TTCAACTTTTCTAGATTCCACATATAAGAGAGATCATGCAATATTTGTCT  Hsapiens
 446733: ..................................................  Ptroglodytes
 499997: ..................................................  Pabelli
 388532: ..................................................  Nleucogenys
 478886: ...................G..............................  Mmulatta
 425778: ...G...............G...............G..............  Panubis
 449788: ......C...............................A...........  Cjacchus
1089090: ....G.C...............................A....C......  Sboliviensis
```

```
                          LINE1
         ------------------------------------------------->
              127085    127095    127105    127115    127125
                   :         :         :         :         :
 127076: TTCTCTGCATGGCTTATTTCACTCAACATAACGTTCTCCAGGTTTACCAT  Hsapiens
 446783: ...............................T..................  Ptroglodytes
 500047: ...............................T..................  Pabelli
 388582: .........G.....................T..................  Nleucogenys
 478936: ........G..............T.......T.............C....  Mmulatta
 425828: ........G......................T.............C....  Panubis
 449838: ..G.....C....................G.T..................  Cjacchus
1089140: ........C......................T..................  Sboliviensis
```

```
                          LINE1
         ------------------------------------------------->
              127135    127145    127155    127165    127175
                   :         :         :         :         :
 127126: GCTGTCATAAATGACAGAGTTTCCTTCCTTTTAAAGGCTGAATAGTAAGT  Hsapiens
 446833: ......................T...........................  Ptroglodytes
 500097: .T....G..........G................................  Pabelli
 388632: .G...............G................................  Nleucogenys
 478986: TT........T......GA..............G....CA..........  Mmulatta
 425878: ..........T......GA..............G....CA..........  Panubis
 449888: .T.......C.......G........-T...........A..GG......  Cjacchus
1089190: .T.......C......AG........--...........A..AG......  Sboliviensis
```

```
                          LINE1
         ------------------------------------------------->
              127185    127195    127205    127215    127225
                   :         :         :         :         :
 127176: TTTCTACTGTGCATGTATATAGCACGTTTTCCTCATCCAGTCATTCATTA  Hsapiens
 446883: ..................................................  Ptroglodytes
 500147: A..........A......G..A...A.......TG........C......  Pabelli
 388682: A....................A...A........................  Nleucogenys
 479036: G......CA...G........A...A........................  Mmulatta
 425928: G....................A...A........................  Panubis
 449937: A.........C..........A...AA............T.....AG...  Cjacchus
1089238: A.........C....C.....A...AA............T.....AG...  Sboliviensis
```

```
                          LINE1
         ------------------------------------------------->
              127235    127245    127255    127265    127275
                   :         :         :         :         :
 127226: TTACATAGATGGGTTGATTCCGTATTTTGATTATTGTGAATAATGCTGCA  Hsapiens
 446933: ..................................................  Ptroglodytes
 500197: .......C................................C.........  Pabelli
 388732: .....................A............................  Nleucogenys
 479086: .......T...A.........A.........................T..  Mmulatta
 425978: ....T..C...A.........A.........................T..  Panubis
 449987: ...G...C....T.......AA......................A.....  Cjacchus
1089288: ...G...C....T..A.....A......................A.....  Sboliviensis
```

LINE1

```
              127285    127295    127305    127315    127325
127276: ATGAACGTGGGAATAAAGATATCTTTCCAACATAACTGCTTTTATTTCCT   Hsapiens
446983: ........................G.T....G..................   Ptroglodytes
464593:                                 ..................   Ggorilla
500247: ......A..CA....................G...T..............   Pabelli
388782: ......A......C......G.....................C.......   Nleucogenys
479136: ......A..A.......C................................   Mmulatta
426028: ......A..A.......C................................   Panubis
450037: .....AA..........C....G.......................G...   Cjacchus
1089338: .....TA..........C....G............T..............   Sboliviensis
```

LINE1

```
              127335    127345    127355    127365    127375
127326: TTGGAAATATACTCAAAACTGGAATTACTAAATCATATGGCAGTACTGTA   Hsapiens
447033: ..........................G.......................   Ptroglodytes
464611: ..........................G.......................   Ggorilla
500297: .......C................................T.T.......   Pabelli
388832: .......C................................T......A..   Nleucogenys
479186: .......C.C..............................T...T.....   Mmulatta
426078: ......GC.C..............................T...T.....   Panubis
450087: .......C..........G..................C..T...T....T   Cjacchus
1089388: .......C..........G.....C...............T...T....T   Sboliviensis
```

LINE1

```
              127385    127395                        127408
127376: TTTAATTTTCTGAAGAATCTTTATG-----------------ATGTTTTC   Hsapiens
447083: .....................A...-----------------........   Ptroglodytes
464661: .........................-----------------........   Ggorilla
500347: .......................A.-----------------........   Pabelli
388882: ...........A.............-----------------........   Nleucogenys
479236: GC.......................-----------------........   Mmulatta
426128: GC.......................-----------------........   Panubis
450137: .....C...T.....G.C.......ATTGAAGAAACTTTATT......C.   Cjacchus
1089438: .....C...T.....G.C.......GTTGAAGAAACTTTATT.......T   Sboliviensis
```

LINE1

```
              127418                  127430    127440    127450
127409: CATAATGTCTATGCTAA--------TTTATAATCCAACCAACAGAGTAAA   Hsapiens
447116: ..........G.A....--------.........................   Ptroglodytes
464694: ..........G.A....--------....................C....   Ggorilla
500380: ......T...G.A...---------...T...G.................   Pabelli
388915: ..........G.A....--------...........G.............   Nleucogenys
479269: ..........GCA....--------.........................   Mmulatta
426161: ..........G.A....--------.........................   Panubis
450187: ......A...GCC.A..CAGCTATT.G...G..T.C..TG--..GAC.GG   Cjacchus
1089488: .C....A...                                           Sboliviensis
```

```
                                 LINE1
         ------------------------------------------------------>
                  127460     127467     127477     127487     127497
127451: ATAAGCCTTT---TTCTGCAACGTTCTTGTCAACACTTGTTATCATTTGC  Hsapiens
447158: ..........---.....................................  Ptroglodytes
464736: ..........---.....................................  Ggorilla
500422: .C...T....---..........C..A......................T  Pabelli
388957: .C...T....---........T.............G.............T  Nleucogenys
479311: .C...T....---.........A..........................T  Mmulatta
426203: .C...T....---.........A..........................T  Panubis
450235: GCC.......GCC.....AG.G...AAA...TTA.GG.TG.G.TTC..CT  Cjacchus

                                 LINE1
         ------------------------------------------------------>
                  127507     127517     127527     127537     127547
127498: GTTTTTGACAAAAAGCCATTCTAACAGGAGTGAGGTGATAGCTCATTGTG  Hsapiens
447205: ..................................................  Ptroglodytes
464783: .........G........................................  Ggorilla
500469: ...........................T......................  Pabelli
389004: ........................................T.........  Nleucogenys
479358: ....................T......A......A.....T.........  Mmulatta
426250: ....................T.............---...T.........  Panubis
450285: ...AC..TT..T..TTTTCC....TG....GA..                  Cjacchus

                                 LINE1
         ------------------------------------------------------>
                  127557     127567     127577     127587     127597
127548: GTTTTAGTTGGCATTTCTCAAATGATTATTGATATTGCATGTTTTCTATT  Hsapiens
447255: ....................................T.............  Ptroglodytes
464833: ..................................................  Ggorilla
500519: ..................................................  Pabelli
389054: .....-....T.......................................  Nleucogenys
479408: .C...TC...A-...A..T.............C.....T...........  Mmulatta
426297: .C...TT...A-....T.T.............C.....T...........  Panubis

                                 LINE1
         ------------------------------------------------------>
                  127607     127617     127627     127637     127647
127598: GACAAATGTCTATTCAGATCATTGGCCTATTTTTAATTGGGTTATTTGTT  Hsapiens
447305: ....................G.............................  Ptroglodytes
464883: ..................................................  Ggorilla
500569: ..................................................  Pabelli
389103: .....G.............T.....................G........  Nleucogenys
479457: .......A..........................................  Mmulatta
426346: C......A...G......................................  Panubis

                                 LINE1
         ------------------------------------------------------>
                  127657     127666     127676     127686     127696
127648: TTATTGTTATTCAGTT-TTTCAGTTCCTTATATATTTTGGATATTAATCC  Hsapiens
447355: ................-................................T  Ptroglodytes
464933: ................-.................................  Ggorilla
500619: ................-......................A..........  Pabelli
389153: ................G..............C.......T..........  Nleucogenys
479507: ...........G....G..C...........G.G......G........T  Mmulatta
426396: ...........G....G..C...........G.G......G........T  Panubis
```

```
                           LINE1
        ------------------------------------------------->
            127706    127716    127726    127736    127746
127697: GTTTTCAGATGTATGCTTTACAGATATTTTCTCCCATTTGTCTCTTCACT Hsapiens
447404: .................................................. Ptroglodytes
464982: .................................................. Ggorilla
500668: .....G.....C...................................... Pabelli
389203: A....G......C.....C................--------------- Nleucogenys
479557: C....G..................C......................... Mmulatta
426446: C....G....A.............C......................... Panubis

                           LINE1
        ------------------------------------------------->
            127756    127766    127775    127785    127795
127747: CTGGTGATTCTCATTTGTGC-GAAGCTTTTTAATTCCATATGGTCTCATT Hsapiens
447454: ....................A...............G............. Ptroglodytes
465032: ....................A...............G............. Ggorilla
500718: ...C................A...............G............. Pabelli
389238: ------------.......AA............A..G............. Nleucogenys
479607: ...C...C...T........A..............TG.C..A........ Mmulatta
426496: ...C...C...T......A.A.....C........TG.C..A........ Panubis

                           LINE1
        ------------------------------------------------->
            127805    127815    127825    127835    127845
127796: TGTCTAATTTAGCTTTTGTTGCCTTTGATTTCATAACCAAAAAATTATTG Hsapiens
447504: .................................................. Ptroglodytes
465082: .................................................. Ggorilla
500768: ..........T.T..................................... Pabelli
389276: ..........T...............A.....G................. Nleucogenys
479657: ..........T....................................... Mmulatta
426546: ..........T....................................... Panubis

                           LINE1
        ------------------------------------------------->
            127855    127865    127875    127885    127895
127846: CCCAAACCAATATCATGAAACTTTTCCCCTCTGTTTTCTACTAGTTGTTT Hsapiens
447554: ..........................A...A................... Ptroglodytes
465132: ..............................A................... Ggorilla
500818: ......T.......................A................... Pabelli
389326: ..............................A................... Nleucogenys
479707: ..................TG..........A.A................. Mmulatta
426596: ..................CG..........A.A.............A... Panubis

                           LINE1
        ------------------------------------------------->
            127905    127915    127925    127935    127945
127896: TACAGTTTTAAGTCTTACCTGTAATCTATTTGGAATTGATTTTTCATATG Hsapiens
447604: ............................................T..... Ptroglodytes
465182: ............................................T..... Ggorilla
500868: ............................................T...C. Pabelli
389376: .................T....T.....................T..... Nleucogenys
479757: ............A...............................T..... Mmulatta
426646: ............A...............................T..... Panubis
```

```
                              LINE1
        ------------------------------------------------->
            127955    127965    127975    127985    127995
127946: GTGTAAGATATGGTTCTAATTTCATTTTTCTGCATGTGTATGTGGTTTCT Hsapiens
447654: .................................................. Ptroglodytes
465232: .................................................. Ggorilla
500918: ..........................C....................... Pabelli
389426: ..........................C....................... Nleucogenys
479807: ..........CA..............C..............A..C..... Mmulatta
426696: ..........C...............C.......C......A..C..... Panubis

                              LINE1
        ------------------------------------------------->
            128005    128015    128025    128035    128045
127996: TAAAACAATTTATTAAAGAGACTGTCCTTTTCCCATTAAATGTTCTTGGC Hsapiens
447704: ..............................-................... Ptroglodytes
465282: .................................................. Ggorilla
500968: ..............................C................... Pabelli
389476: ......C...............CA.......................... Nleucogenys
479857: ......CT......................CT.................. Mmulatta
426746: ......CT......................CT.................. Panubis

                              LINE1
        ------------------------------------------------->
            128055    128065    128075    128085    128095
128046: CCTTTGTTAAAAGCTATTTGGCCATAAATATCTGGATCTATTTCTGGACT Hsapiens
447753: ............A..........................G.......... Ptroglodytes
465332: ............A..........................G.......... Ggorilla
501018: .....T......A..........................G.......... Pabelli
389526: ............A......................G...G.......... Nleucogenys
479907: ....C.......A..........................G.......... Mmulatta
426796: ....C.......A.C.....A..................G.......... Panubis

                              LINE1
        ------------------------------------------------->
            128105    128115    128125    128135    128145
128096: GTATTCTCTTCTACTTCTTTATGTGCCTGTTTTTATGCCAGTACCATGCC Hsapiens
447803: .................................................T Ptroglodytes
465382: .................................................. Ggorilla
501068: ....C...........G................................. Pabelli
389576: ...C............G................................. Nleucogenys
479957: A.....C...A..T..G................................. Mmulatta
426846: ......C...A..T..G............C.................... Panubis

                              LINE1
        ------------------------------------------------->
            128155    128165    128175    128185    128195
128146: ATTTTGATTAATATTGATTCTTAAGATATTTTGAAGTTAAGTAGTGTGAT Hsapiens
447853: ............................................G..... Ptroglodytes
465432: .................................................. Ggorilla
501118: .................................................. Pabelli
389626: .................................................. Nleucogenys
480007: .....................................A............ Mmulatta
426896: .............................................A.... Panubis
```

```
                                 LINE1
        ------------------------------------------------------------->
                     128205    128215    128225    128235    128245
128196: GCCTCCTGCTTTGTTCTCTTTGTTCAAAATTGCTTGACTATTCTAGGTTT  Hsapiens
447903: .....A............................................  Ptroglodytes
465482: ..................................................  Ggorilla
501168: ..................................................  Pabelli
389676: ..................................................  Nleucogenys
480057: ............C.................................T...  Mmulatta
426946: ............C.................................T...  Panubis

                                 LINE1
        ------------------------------------------------------------->
                     128255    128265    128273    128283    128293
128246: TTTGTGGTTCTATACATATTTTAGGA--TTTTTAAAGAAATTTTGGTAGG  Hsapiens
447953: ..........................--......................  Ptroglodytes
465532: ..........................--.....T................  Ggorilla
501218: ..G.......................--.....T................  Pabelli
389726: ...............G..........AT.............C...A....  Nleucogenys
480107: ................C.........--........T........A....  Mmulatta
426996: .....T..........C.........--........T........A....  Panubis

                                 LINE1
        ------------------------------------------------------------->
                     128303    128313    128323    128333    128343
128294: TATTGAATTGAAACCATAGATAAATTTGGGAAGTATGAATATTTTAACAA  Hsapiens
448001: .G................................................  Ptroglodytes
465580: ......................................C...........  Ggorilla
501266: G..........................CA.....................  Pabelli
389776: G...........................................A.....  Nleucogenys
480155: G....C.........................................TG.  Mmulatta
427044: G....C..........................................G.  Panubis

                                 LINE1
        ------------------------------------------------------------->
                     128353    128363    128373    128383    128393
128344: TATTAATTCTCCCAATCCATGAAAATGGTACATCCTTCCATTGCTTTCTG  Hsapiens
448051: ..........T................A......................  Ptroglodytes
465630: ..........T................A......................  Ggorilla
501316: ..........T.......................................  Pabelli
389826: ..........T.....................A.......A.........  Nleucogenys
480205: ..C.......T............C..........................  Mmulatta
427094: ..C.......T............C..........................  Panubis

                                 LINE1
        ------------------------------------------------------------->
                     128403    128413                          128420
128394: TCTACTTCAATTTTTTTCAATGT-----------------------CTTT  Hsapiens
448101: ...............A.......CTTATAATTTTCGGTGTACAGGC....  Ptroglodytes
465680: ...............A.......CTTATAATTTTCAGTGTACAGGC....  Ggorilla
501366: ...............A.......CTTATAATTTTTAGTGTACAGGC....  Pabelli
389876: ...............A.......CTTATAATTTTCAGCATACAGGC....  Nleucogenys
480255: ...............A....C..TTTATAATTTTCAGTGTACAGGC....  Mmulatta
427144: ...............A.......TTTATAATTTTCAGTGTACAGGC....  Panubis
```

```
                                LINE1
         ---------------------------------------------------->
              128430    128440    128450    128460    128470
128421: CACATCCTTAGTTAAATTCATTCCTAAGTATATTTTGAACTATTGTAAAT  Hsapiens
448151: ..................................................  Ptroglodytes
465730: ..................................................  Ggorilla
501416: .........G........................................  Pabelli
389926: ...T.....G...................................C....  Nleucogenys
480305: .........G..........---...G.......A...G...........  Mmulatta
427194: .........G..........C.............A...G...........  Panubis

                                LINE1
         ---------------------------------------------------->
              128480    128490    128500    128510    128520
128471: GAAATTGTTTCCTCGATTTCTTTTTCAGGTGGTTGCTTGTAAGTACATAG  Hsapiens
448201: .....C............................................  Ptroglodytes
465780: ..................................................  Ggorilla
501466: .....C.....................A......................  Pabelli
389976: ..................................................  Nleucogenys
480352: ..............A...............A.............G.....  Mmulatta
427244: .......C......................A.............G.G...  Panubis

                                LINE1
         ---------------------------------------------------->
              128530    128540    128550    128560    128570
128521: AAGCACTACTAATACTTTGTATGTTGATTTTGTATTCTGCAACTATGCTG  Hsapiens
448251: ..............................................A...  Ptroglodytes
465830: ..............................................A...  Ggorilla
501516: ..............T...............................A...  Pabelli
390026: ..............T..................G............A...  Nleucogenys
480402: ..A......G....T..............................CA...  Mmulatta
427294: ..A...........T..............................CA...  Panubis

                                LINE1
         ---------------------------------------------------->
              128580    128590    128600    128610    128620
128571: AAGTCATTAGTTCTAACAGATTTTTTTGTGGAATCTTTAGGGTTTTCTAT  Hsapiens
448301: ..............G.....-.............................  Ptroglodytes
465880: ....................-........A....................  Ggorilla
501566: ....................-.............................  Pabelli
390076: ....................-..............C.A............  Nleucogenys
480452: ....................-...........................T.  Mmulatta
427344: ....................-...........................T.  Panubis

                                LINE1
         ---------------------------------------------------->
              128630    128640    128649    128659    128669
128621: ATAAAATATCATGTCACTTGCAAAC-GAGACTATTTAAGTTGTTTCTTTC  Hsapiens
448350: ......G.................T-........................  Ptroglodytes
465929: ......G.................T-........................  Ggorilla
501615: ......G..................G........................  Pabelli
390125: ...T..G..................G........................  Nleucogenys
480501: ......G.................TG......G.....A...........  Mmulatta
427393: ......G.................TG......G.....A...........  Panubis
```

```
                                 LINE1
         ------------------------------------------------------->
                  128679    128689    128699    128709    128719
128670: TGATTTTGAAGGCTTTTACATTTTTCTATTGCATAATTGCTCTGACGAGG  Hsapiens
448399: ...................G..........................T...  Ptroglodytes
465978: ..............................................T...  Ggorilla
501665: ..........................................C...T...  Pabelli
390175: ..............................................T.A.  Nleucogenys
480551: ..............................................T..A  Mmulatta
427443: ..............................................T...  Panubis

                                 LINE1
         ------------------------------------------------------->
                  128729    128739    128749    128759    128769
128720: AATTCTAGTACCAAACTGAATAAAAGAGTGGGCATTTTTGTCTTATTCTT  Hsapiens
448449: ..................................................  Ptroglodytes
466028: ..................................................  Ggorilla
501715: ....................C..............G..............  Pabelli
390225: ..................................................  Nleucogenys
480601: ....G..T..TG...T..................................  Mmulatta
427493: ....G.....TG...T..................................  Panubis

                                 LINE1
         ------------------------------------------------------->
                  128779    128789    128799    128809    128819
128770: AGAGGAAAAGTTTTAAATTTTCATAATTGCATATGATTTTAGCTGTGGGT  Hsapiens
448499: .............................A....................  Ptroglodytes
466078: .............................A....................  Ggorilla
501765: .............................A....................  Pabelli
390275: .A................A..........A....................  Nleucogenys
480651: ..T.....C.......C............A.................A..  Mmulatta
427543: ..C.....C.......C...........TA.................A..  Panubis

                                 LINE1
         ------------------------------------------------------->
                  128829    128839    128849                128866
128820: TGGTCATAGATGGCTTTTATTATGTTAAAATACAGTC---CTGTACCTAA  Hsapiens
448549: .....................................---..........  Ptroglodytes
466128: .....................................---..........  Ggorilla
501815: ............................G........CTT..........  Pabelli
390325: ............................G........CTT..........  Nleucogenys
480701: ..............C.............G........TTT..........  Mmulatta
427593: ............................G........TTT..........  Panubis

                                 LINE1
         ------------------------------------------------------->
                  128876    128886    128896    128906    128916
128867: TTTATTGAGAAATTTTATCATGAAATCAGGTTGAATTTATTCAAACGCTT  Hsapiens
448596: .........................G........................  Ptroglodytes
466175: .........................G...................T....  Ggorilla
501865: .........................G...................T....  Pabelli
390375: .........................G..C.....................  Nleucogenys
480751: .---.A.........C..-......G..T................A....  Mmulatta
427643: ...............C.........G..T................A....  Panubis
```

LINE1

```
                 128926    128936    128946    128956    128966
128917: TTTCTGCATCTCTTGAGATAATCATATCATTGTCCTTTATTCTGATAATA  Hsapiens
448646: ..................................................  Ptroglodytes
466225: ..........................--......................  Ggorilla
501915: ..................................................  Pabelli
390425: .............CA...................................  Nleucogenys
480797: .....TT...........................................  Mmulatta
427693: ..................................................  Panubis
```

LINE1

```
                 128976    128986    128996    129006    129016
128967: AAATTTATCATATTTATGGATTTTCATATATTAAACCATCCTTGCATCCC  Hsapiens
448696: ..................................................  Ptroglodytes
466273: ..................................................  Ggorilla
501965: ..................T...............................  Pabelli
390475: ....G...G.......C..........G......................  Nleucogenys
480847: T.................................................  Mmulatta
427743: T.................................................  Panubis
```

LINE1

```
                 129026    129036    129046    129056    129066
129017: TGTGATAAATCCTATTTCATTATGTCAATTGATCAACTACATGTGCTATT  Hsapiens
448746: ..................................................  Ptroglodytes
466323: ...A..............................................  Ggorilla
502015: .................A................................  Pabelli
390525: ..................................................  Nleucogenys
480897: ...................A.G....G............A..........  Mmulatta
427793: ..........................G....T.......A..........  Panubis
```

LINE1

```
                 129076    129086    129096    129106    129116
129067: GAATTCAGTCTGCTAGTAATTTTTGAGGATTTTTGCATCTATGCTCATCA  Hsapiens
448796: ..................................................  Ptroglodytes
466373: ..................................................  Ggorilla
502065: ..................................................  Pabelli
390575: ..................................................  Nleucogenys
480947: ..................................................  Mmulatta
427843: ..................................................  Panubis
```

LINE1

```
                 129126    129135    129145    129155    129165
129117: AGGATATTCATCTATAA-TTTTTTTTCTTGTAGTGGTCTTGTGTGGCTTT  Hsapiens
448846: .................-.G..............................  Ptroglodytes
466423: .................-.C..............................  Ggorilla
502115: .................T................................  Pabelli
390625: .................T...............................C  Nleucogenys
480997: .....G...........--...............................  Mmulatta
427893: .....G...........--...............................  Panubis
```

LINE1

```
              129175    129185    129195    129205    129215
129166: AGTATTTCCATATCCTGGCCTCCTAAAATGAGTTTGAAAGTATTTCCTCC  Hsapiens
448895: ..................................................  Ptroglodytes
466472: ........T........A................................  Ggorilla
502165: ...............C..................................  Pabelli
390675: ..................................................  Nleucogenys
481045: ........................-......A.C..........AT....  Mmulatta
427941: ...............................A............A.....  Panubis
```

LINE1

```
              129225    129235    129245    129255    129265
129216: ACTTCAGTTTTTTTGGAAGTATGAGAAGAATTGGTAGTAGTCCTTCCTTA  Hsapiens
448945: ..................................................  Ptroglodytes
466522: .....................................C............  Ggorilla
502215: ..............CA....................T..T..........  Pabelli
390725: ..........C.........................T.............  Nleucogenys
481094: T...................................T....T....T...  Mmulatta
427991: T...................................T....T....T...  Panubis
```

LINE1

```
              129275    129285    129295    129305    129315
129266: TATATTTGGTAGAATTCAGCTGTAGAGCCACTGGGTTCTGGGCTTTTCTT  Hsapiens
448995: ..................................................  Ptroglodytes
466572: ..................................................  Ggorilla
502265: ..................................................  Pabelli
390775: ...G....                                            Nleucogenys
481144: .........................CA...........G.....C.....  Mmulatta
428041: .........................CA...........G.....C.....  Panubis
```

LINE1

```
              129325    129335    129345    129355    129365
129316: TGATAAAAGAATTGTTATTACTAATTCAATCTCCTAACTCACCATTGGTC  Hsapiens
449045: ..................................................  Ptroglodytes
466622: ..................................................  Ggorilla
502315: ..................................................  Pabelli
481194: ...C..............................A..A............  Mmulatta
428091: ...C..............................A..A............  Panubis
```

LINE1

```
              129375    129385    129395    129405
129366: TGTTCAGATTTTCTGTTTCTTCATCGTAGATTTATCCTGG----------  Hsapiens
449095: ........................T...............----------  Ptroglodytes
466672: ........................T...............----------  Ggorilla
502365: ........................T...............----------  Pabelli
481244: .T...............C......T.........C.....TAGATTTATC  Mmulatta
428141: ..........G.............T.........C.....TAGATTTATC  Panubis
```

```
                           LINE1
        ------------------------------------------------->
              129411    129421    129431    129440    129450
129406: ----TAGATTATACGTATCTAGAAATTTATCAG-TTTTTTTCTAGGTTAT  Hsapiens
449135: ----.........T..................A-..........C.....  Ptroglodytes
466712: ----.........T..................A-................  Ggorilla
502405: ----.........T...................-................  Pabelli
481294: CTGA.........T.C........T........T................  Mmulatta
428191: CTGG.........T.C........T........-........C.......  Panubis
```

```
                           LINE1
        ------------------------------------------------->
              129460    129470    129480    129489    129499
129451: CCAATTTGTTGGTCTATAATTGGTCTTAGCAGTATCT-TATGATGCATTG  Hsapiens
449180: ...................................T.-............  Ptroglodytes
466757: ...................................T.-............  Ggorilla
502450: ...............G.....................-........T...  Pabelli
481344: .......T.............C..............CA............  Mmulatta
428240: .......T.............C..............CA............  Panubis
```

```
                           LINE1
        ------------------------------------------------->
              129509    129519    129529    129539    129545
129500: TATTTCTGTGGTATCAGTTGTAAAATCTCTTTCATTTCTGAT----TTTA  Hsapiens
449229: ..........A.....A.........................----....  Ptroglodytes
466806: ..........A.....A.........................----....  Ggorilla
502499: ................A..........----...........----....  Pabelli
481394: ..........A...............................TTTG....  Mmulatta
428290: ..........................................----....  Panubis
```

```
                           LINE1
        ------------------------------------------------->
              129555    129564    129574    129584    129594
129546: TTTGCATCTTTCCA-CTTTTTTCTAAGCCTTTAAAGGTTTATCATTTGTG  Hsapiens
449275: ..............-...................................  Ptroglodytes
466852: ..............-...................................  Ggorilla
502541: ..............-.....................A.............  Pabelli
481444: ..............C.........TG........................  Mmulatta
428336: ..............C.........T..................G......  Panubis
```

```
                           LINE1
        ------------------------------------------------->
              129604    129614    129624    129634    129644
129595: TATAATTTTTTAAAAGCAATTCTAACTTTTGTTGATCTTTTCTATTGTTT  Hsapiens
449324: .......................T..........................  Ptroglodytes
466901: .......................T..........................  Ggorilla
502590: .......................T..........................  Pabelli
481494: .......................T.........T................  Mmulatta
428386: ................A......T.T.......T............A...  Panubis
```

```
                                 LINE1
         ---------------------------------------------------->
                129654    129664    129674    129684    129694
129645: TCTAATCTCTATTTCATTTATTTCTGCTCTGATTATTATTATTTCCTTCC   Hsapiens
449374: .........-........................................   Ptroglodytes
466951: .........-........................................   Ggorilla
502640: ................................................G.   Pabelli
481544: .........C.........G..............................   Mmulatta
428436: .........C.........G..............................   Panubis
```

```
                                 LINE1
         ---------------------------------------------------->
                129704    129714    129724    129734    129744
129695: TTCTATTAATTTTGGGCTTAGCTTTGTTCTTGTCTTTTTAGTTCTTTGCG   Hsapiens
449423: ..................................................   Ptroglodytes
467000: ..........G......................................T   Ggorilla
502690: .....C.........................T..................   Pabelli
481594: ..G..C...C......A.......................A.......TA   Mmulatta
428486: .....C...C......A.........A..C..........A.......TA   Panubis
```

```
                                 LINE1
         ---------------------------------------------------->
                129754    129764    129774    129784    129794
129745: ATATTACATTAGGGTGTTTATTTGAGAGCTTTCTTCTTTTTTGATGTAGG   Hsapiens
449473: ......................................C...........   Ptroglodytes
467050: ..................................................   Ggorilla
502740: ..G...............................................   Pabelli
481644: .C................................................   Mmulatta
428536: .C...................C...........-................   Panubis
```

```
                    LINE1                     Alu
         ------------------------->-------------------------->
                129804    129814    129824    129834    129844
129795: TGTTTATTGCTATAAACTTCACTCTTGGCCAAGTGCAGTGGCTTATGCCT   Hsapiens
449523: ................A..T.................A............   Ptroglodytes
467100: ..................................................   Ggorilla
502790: ....................G.....T....G..................   Pabelli
481694: ..........-........TG..........G..................   Mmulatta
428585: ..........-........TG..........G..................   Panubis
```

```
                                  Alu
         ---------------------------------------------------->
                129854    129864    129874    129884    129894
129845: GTAATCCCAGCACTTTGAGAGGCCAAAATGAGAGGATTGCTTGTACCCAG   Hsapiens
449573: ..................................................   Ptroglodytes
467150: ..................................................   Ggorilla
502840: ..............................G...................   Pabelli
481743: ...........G........A.........G...........A.......   Mmulatta
428634: ....................A.........G...................   Panubis
```

```
                                Alu
         ------------------------------------------------->
                  129904     129914     129924     129934     129944
                       :          :          :          :          :
129895: GAGTTCAAGACCAGTCCAGGCAACATAGGGAAACCCCATCTCTACACAGA  Hsapiens
449623: ..................................................  Ptroglodytes
467200: ..................................................  Ggorilla
502890: ..............C..........C...C....................  Pabelli
481793: ..........T......G.................A..........A...  Mmulatta
428684: ..........T.....TG.................A..........A...  Panubis
```

```
                                Alu
         ------------------------------------------------->
                  129953     129963     129973     129983     129993
                       :          :          :          :          :
129945: AAAAATG-AAAAAGTAGCTAGGCTTGGTGGTGTACACCTGTGGTTCCAGC  Hsapiens
449673: .......-..........................................  Ptroglodytes
467250: .......-..........................................  Ggorilla
502940: .......-..........C......T........................  Pabelli
481843: .......A..........C...............T..T.C........A.  Mmulatta
428734: .......A..........C..T......-.....T..T.T........A.  Panubis
```

```
                                Alu
         ------------------------------------------------->
                  130003     130013     130023     130033     130043
                       :          :          :          :          :
129994: TACTTAGGAGGCTGAGGTGAGAAAATTCCTTAATCCCTGGAAGTTGAGGC  Hsapiens
449722: ..................................................  Ptroglodytes
467299: ..................................................  Ggorilla
502989: ...C..............................................  Pabelli
481893: ..TG.......T...A......T...C....G.......A..........  Mmulatta
428783: ..TG.......T...A.....CT...C....G..................  Panubis
```

```
                                Alu
         ------------------------------------------------->
                  130053     130063     130073     130083     130093
                       :          :          :          :          :
130044: TGCAGTGAGCCATAATTGTGCTACTACACTTCAGCCTGGGTGACAGAGTG  Hsapiens
449772: .............................................A....  Ptroglodytes
467349: .............................................A....  Ggorilla
503039: .....................C............................  Pabelli
481943: .....................C............................  Mmulatta
428833: .....................C............................  Panubis
```

```
                   Alu                        LINE1
         ----------------------->------------------------->
                  130103     130113     130123     130133     130143
                       :          :          :          :          :
130094: AGACCCTGTCTCAAAAAGAAAAATTACTCTTAGAAGTGCTTTTTATATGC  Hsapiens
449822: ..................................................  Ptroglodytes
467399: ..................................................  Ggorilla
503089: ...............................G..................  Pabelli
481993: ..................................................  Mmulatta
428883: ..................................................  Panubis
```

```
                       LINE1
         ------------------------------------------------->
             130153    130163    130173    130183    130193
130144: ATCCCATACATTAGTTTTGTTATGTTTTTTTTTCTATTTTTGTTTGCCTC  Hsapiens
449872: ......................--..........................  Ptroglodytes
467449: ......................--..........................  Ggorilla
503139: ...................GGG---.........................  Pabelli
482043: ..T.....GG..........A.----.......A................  Mmulatta
428933: ..T.....GG..........AT---........AA...............  Panubis
```

```
                       LINE1
         ------------------------------------------------->
             130203    130212    130222    130232    130242
130194: AACATACTTCTTAATAAA-TTTTTTTCATTGATCTCTTGAGTGTTTAGGA  Hsapiens
449920: ..................-...............................  Ptroglodytes
467497: ..................-...............................  Ggorilla
503186: ..................T........................A......  Pabelli
482089: ..G...A.C.........-.G.........A..............C....  Mmulatta
428980: ..G...A.C.........-.G.........A...................  Panubis
```

```
                       LINE1
         ------------------------------------------------->
             130252    130262    130272    130282    130292
130243: GTATGTCGGTTAATTTTAAAATATTTGGGCATTTTCCAAAATTCCTCCTG  Hsapiens
449969: ...........................A......................  Ptroglodytes
467546: ..................................................  Ggorilla
503236: .......A.........................................A  Pabelli
482138: ...C.GT.T.........................................  Mmulatta
429029: .....CT.T.........................................  Panubis
```

```
                       LINE1
         ------------------------------------------------->
             130302    130312    130322    130332    130342
130293: TTACTGAATTACAGTTTCAAGTATTGTGGTTGGTAAAGATACTTACTATG  Hsapiens
450019: ..................................................  Ptroglodytes
467596: ..................................................  Ggorilla
503286: ...........T......................................  Pabelli
482188: ...........T...............T......................  Mmulatta
429079: ...........T...............T......................  Panubis
```

```
                       LINE1
         ------------------------------------------------->
             130352    130362    130372    130382    130392
130343: ATTTCAATGTTCTCAAATTTCTATGCCTGAGAAAAATATGTATTCTGTAA  Hsapiens
450069: ..................................................  Ptroglodytes
467646: ..................................................  Ggorilla
503336: .......C..........................................  Pabelli
482238: ..................................G.C.............  Mmulatta
429129: ............................C.....G.C.............  Panubis
```

```
                          LINE1
        ------------------------------------------------->
            130402    130412    130422    130432    130442
130393: CTGGAGGATGGGATGTTCTGAATGGTCCATTTGATCTAAGATTTAGTTTA  Hsapiens
450119: ..................................................  Ptroglodytes
467696: ..................................................  Ggorilla
503386: ..................................................  Pabelli
482288: ....T.....................................G.......  Mmulatta
429179: ....T.......................G............GG.......  Panubis

                          LINE1
        ------------------------------------------------->
            130450    130460    130470    130480    130490
130443: ATTC--TGTTTCCTTATTGATTTTCTGTATGGAGGAAAGTGGGGTACTGA  Hsapiens
450169: ....--............................................  Ptroglodytes
467746: ....--..........C.................................  Ggorilla
503436: ....--..C.......C.................................  Pabelli
482338: .G..GT.C......C...................................  Mmulatta
429229: .G..AT.C............................T.............  Panubis

                          LINE1
        ------------------------------------------------->
            130500    130510    130520    130530    130540
130491: AGTCTTCTAATATTATTGTATTACTGTCTATCTCTCCCTTCAGATTTGTT  Hsapiens
450217: ..................................................  Ptroglodytes
467794: ..........---.....................................  Ggorilla
503484: ...........................G........T.............  Pabelli
482388: .........C........G.....................G.......A.  Mmulatta
429279: .........C......................................A.  Panubis

                          LINE1
        ------------------------------------------------->
            130550    130560    130570    130578    130588
130541: CATATTTATATATTTAGGTGCTCTGACATTGGATGC--ATATGTTTATAA  Hsapiens
450267: ....................................--............  Ptroglodytes
467841: ....................................--............  Ggorilla
503534: ....................................--............  Pabelli
482438: A............G............T.C.C.G...AT....A......C  Mmulatta
429329: A.........................T.C...G...AT....A......C  Panubis

                          LINE1
        ------------------------------------------------->
            130598    130608    130618    130628    130638
130589: TTGTTATATCTTCTTCATAAATTGATCCTTTTATCATTATAGAATAACCT  Hsapiens
450315: ................................................G.  Ptroglodytes
467889: ..................................................  Ggorilla
503582: ...............G...........G.............T........  Pabelli
482488: ...............G.C.....................C.T.....T..  Mmulatta
429379: ...............G.C.......................T.....T..  Panubis
```

```
                                      LINE1
         ---------------------------------------------------------->
                  130648    130658    130668    130678    130688
130639: TCCTCATCTCTTTTTACAGCTTTTGACTCAAAGCCTATTTTATGTGATAT  Hsapiens
450365: ............C......T........................G.....  Ptroglodytes
467939: ............C......T..............................  Ggorilla
503632: .....G.....C.......T......................CA......  Pabelli
482538: ...................T............................G.  Mmulatta
429429: ...................T.........G...........C......G.  Panubis
```

```
                                      LINE1
         ---------------------------------------------------------->
                  130698    130708    130718    130728    130738
130689: AAGTATAGCTATTCATGCTCTCTTTTGGTTTCCATTTGCATGCAATAACT  Hsapiens
450415: ..........C.......................................  Ptroglodytes
467989: ..................................................  Ggorilla
503682: ..A....T.................G........................  Pabelli
482588: ........G..C........A.............................  Mmulatta
429479: ...........C...................T..................  Panubis
```

```
                                      LINE1
         ---------------------------------------------------------->
                  130748    130758    130768    130778    130787
130739: TTCCCCATCCCTTCACTTTCAGTCTATGTCCTTAAAGGTGAAGTG-AGTC  Hsapiens
450465: .........................................G...-....  Ptroglodytes
468039: .............................................-....  Ggorilla
503732: ..................................T..........-....  Pabelli
482638: .............................T....C..........A....  Mmulatta
429529: ..A.....................G....T....C..........-....  Panubis
```

```
                                      LINE1
         ---------------------------------------------------------->
                  130797    130807    130817    130826    130836
130788: TCTTGTAGACAGCATATAGTTAGATCTTGTTTATTTTT-ATCCATTCAGT  Hsapiens
450514: ......................................-...........  Ptroglodytes
468088: ......................................-...........  Ggorilla
503781: ........G.......................T.....A...........  Pabelli
482688: .......AG...............C.............-.......TGA.  Mmulatta
429578: .......AG.............T.C.............-.......TGA.  Panubis
```

```
                                      LINE1
         ---------------------------------------------------------->
                  130846    130856    130866    130876    130886
130837: CAATCTATGTCTATGATTAGAAAATGTAATCCATTTATATTCAAGGTAAC  Hsapiens
450563: ..................................................  Ptroglodytes
468137: ..................................................  Ggorilla
503831: ............................................A.....  Pabelli
482737: ..C...G......C....................................  Mmulatta
429627: ..C...G......C...............C....................  Panubis
```

```
                                LINE1
         ---------------------------------------------------------->
                130896    130906    130916    130926    130936
                     :         :         :         :         :
130887: TATTAATCAATAAAGACTTATTACCACTATTTTGTTAATTGGTTTTCAGT  Hsapiens
450613: ..................................................  Ptroglodytes
468187: ..................................................  Ggorilla
503881: .......T................TG........................  Pabelli
482787: .......A................................T.........  Mmulatta
429677: .........G.............A................T.....TG..  Panubis

                                LINE1
         ---------------------------------------------------------->
                130946    130956    130966    130976    130986
                     :         :         :         :         :
130937: TGTTTGTAGATCTTTCATTCCTTTCTTCCTCTTTTGCTGTCTTCCTTTGT  Hsapiens
450663: ..................................................  Ptroglodytes
468237: ..................................................  Ggorilla
503931: ...............TG.................................  Pabelli
482837: ..C............TG.................................  Mmulatta
429727: ..C............TG............C....................  Panubis

                                LINE1
         ---------------------------------------------------------->
                130996    131006    131016    131026    131036
                     :         :         :         :         :
130987: GAATGATTTTCTGTAGTCACATGCTTTGATTCTTTACTTTTTTTCCTTTG  Hsapiens
450713: ........................A.........................  Ptroglodytes
468287: ........................A..................C.T-...  Ggorilla
503981: ......................T....................A.T-...  Pabelli
482887: .......................A............G.....A...-...  Mmulatta
429777: .......................A..................A...-...  Panubis

                                LINE1
         ---------------------------------------------------------->
                131046    131056    131066    131076    131086
                     :         :         :         :         :
131037: TGTATCTACTCTAAGTTTTTGCTTTGTATTTACAATGAGGATTATAGAAA  Hsapiens
450763: ..................................................  Ptroglodytes
468336: ..................................................  Ggorilla
504030: ...............................G..................  Pabelli
482936: .............G...........T........................  Mmulatta
429826: .............G...........T........................  Panubis

                                LINE1
         ---------------------------------------------------------->
                131096    131106    131116    131126    131136
                     :         :         :         :         :
131087: ATATTTTATATTTACATTAGGCTATTTTAAGCTGATAACAGTTTAACTCT  Hsapiens
450813: ..G............G................................T.  Ptroglodytes
468386: ................................................T.  Ggorilla
504080: ........................................A....G..T.  Pabelli
482986: ......................................T.A.......T.  Mmulatta
429876: ......................................T.A.......T.  Panubis
```

```
                                   LINE1
        ------------------------------------------------------>
                 131146    131156    131166                131183
131137: GTCTGCATTGAAAACCTCTGCACTTTTAATCTATGTCC---CCACATTTT  Hsapiens
450863: .A....................................CCA.........  Ptroglodytes
468436: .A....................................CCA...G.....  Ggorilla
504130: .A........G......................C....CCA.........  Pabelli
483036: .A...........G.......................TCCA.........  Mmulatta
429926: .A...................................TCCA.........  Panubis

                                   LINE1
        ------------------------------------------------------>
                 131193    131203    131213    131223    131233
131184: AAATATTTGATGTCATAATTTACATCTTTTTATGTTGTATATTCCTTAAC  Hsapiens
450913: ..................................................  Ptroglodytes
468486: .....................................C............  Ggorilla
504180: .......................T.............C............  Pabelli
483086: ............................G.....................  Mmulatta
429976: ............................G.....................  Panubis

                                   LINE1
        ------------------------------------------------------>
                 131243    131253    131263    131273    131283
131234: AAATTATTGTAGTTATTATTTTTTACTAGTGAGTTTTATACTTTCATATA  Hsapiens
450963: ..................................................  Ptroglodytes
468536: ..................................................  Ggorilla
504230: ..................................................  Pabelli
483136: ......C...T........G................A.............  Mmulatta
430026: ......C...T........G................A.............  Panubis

                                   LINE1
        ------------------------------------------------------>
                 131293    131303    131313    131323    131333
131284: TTTTGTGTTGCCCATTAGCATCTTTTTCTCTCACATTGAATAATTAATTC  Hsapiens
451013: ..................................................  Ptroglodytes
468586: ..................................................  Ggorilla
504280: .........A...................T..........G..C......  Pabelli
483186: .........A....                                      Mmulatta
430076: .........A....-A.............T....C.....G..C.....G  Panubis

                                   LINE1
        ------------------------------------------------------>
                 131343    131353    131363    131373    131383
131334: CCTTTAGCAATTCCTATAAGATATGTCTGCTCATAATGAACTCCCTCAGT  Hsapiens
451063: ..................................................  Ptroglodytes
468636: ..................................................  Ggorilla
504330: ..................................................  Pabelli
430125: T.......................A.........................  Panubis

                                   LINE1
        ------------------------------------------------------>
                 131390    131400    131409    131419    131429
131384: TTT---TCTTTGTCTGGAAAA-TTTTTTATCCCTCCATTTTTCAAGGATA  Hsapiens
451113: ...---...............-............................  Ptroglodytes
468686: ...---...............-............................  Ggorilla
504380: ...---.G.............T....................G.......  Pabelli
430175: ...TGG.T...A..CCTCT.C-...............C....G.....GG  Panubis
```

```
                                  LINE1
         ------------------------------------------------------------>
                 131439    131449    131459    131469    131479
131430: GCATTGTCATATTTGGTATTCTTATTTGGCAGTTTTTAATCTTTCAGTAC  Hsapiens
451159: ..................................................  Ptroglodytes
468732: ..................................................  Ggorilla
504427: ...............................C..................  Pabelli
430224: ...........CC...........G......AGC...-T...........  Panubis
```

```
                                  LINE1
         ------------------------------------------------------------>
                 131489    131499    131509    131519    131528
131480: TTTGAATATATTATCTCACTCTCTCTGGCCTCTAAGATCTTGGCTATG-A  Hsapiens
451209: ................................................-.  Ptroglodytes
468782: ......................----......................-.  Ggorilla
504477: ...............................G................-.  Pabelli
430273: ...A.......C.G...........CA....A....G...........A.  Panubis
```

```
                                  LINE1
         ------------------------------------------------------------>
                           131547    131557    131567    131577
131529: ACACTCTGA-GGACTTACTAGAACTCTCTTTTTGTTAATCCCTTATTTTT  Hsapiens
451258: ..C......-.......T................................  Ptroglodytes
468827: ..C......-.......T................................  Ggorilla
504526: ..C......-.......T................................  Pabelli
221247:                                                ...  Nleucogenys
430323: .TC.A....TA......T........C......................G  Panubis
```

```
                                  LINE1
         ------------------------------------------------------------>
                 131587    131597    131601    131607    131617
131578: TCCAGCTACTTTCACTATTCTCT------CTT----TTTATTTTTGACAG  Hsapiens
451307: .......................------...----..............  Ptroglodytes
468876: .......................------...----..............  Ggorilla
504575: .T.....................------...----..............  Pabelli
221250: .AA.A.......GCTG...T..CTATTGA...----G.A......-----  Nleucogenys
430373: .......G...............------...TGTC..C...........  Panubis
```

```
                                  LINE1
         ------------------------------------------------------------>
                 131627                131639    131649    131659
131618: TTTGATTATATTAT--------GGCCTTTTGTAGATTTGTTTGGACTAAA  Hsapiens
451347: ..............--------.T..........................  Ptroglodytes
468916: ..............--------.T..........................  Ggorilla
504615: .......G......--------.T..........................  Pabelli
221291: ...T.A.G....C.AAAATACAA.....G.GTG....CCA--.A..T.GC.  Nleucogenys
388796:                           .C...G..G....-----.T.C.T  Mmulatta
430417: ..............--------.T..........................  Panubis
203155:                                               ....  Cjacchus
```

```
         LINE1
         ──►
                131669     131679     131689     131699     131705
131660: TTTTTTTTTTATTTCTTTTTTTCCTTTATTTGTAATTATT----GTTTTC  Hsapiens
451389: .C..-........A...........................----......  Ptroglodytes
468958: .C.......................................----......  Ggorilla
504657: .C.GAC.GGAGAA.T..GACC.T...ATA.CTGG..AC..----A.A..T  Pabelli
221339: .C........T...T.......TT...T...T.G                 Nleucogenys
388815: ..........T...T.......TT...T.G                     Mmulatta
377629:  .........T...T.......TT.G.....T..G.               Panubis
430459: .                                                  Panubis
430495:                                     AC..----A.A..T  Panubis
203159: ....A.A.G...C.T..C..C.G.A..T.GCC...G.CA.TTGAA.C..T  Cjacchus

                131715     131725                      131743
131706: TTCTCAACTATCATTAGAATTTTTGAAT------------AATTAGGGTT  Hsapiens
188100:                                                ..  Ptroglodytes
451434: ............................------------........    Ptroglodytes
469004: ............................------------..........  Ggorilla
504703: .....C.AAT.TGGA..                                  Pabelli
503155:                                         ..G.....  Pabelli
405252:                                                 ..  Mmulatta
410127:                                                ...  Panubis
430505: .......A.T.GGAA..--.....T...ATTATATCACTA...AT.     Panubis
203209: A.T.T.GA.T.T.CC.C-...GACA...---------GGA....TA.A..  Cjacchus

                                        LINE1
                                   ──────────────────────►
                131753     131763     131773     131783     131793
131744: TTTTTTCTTTTTTTTTCTTTTTTTTAATTATTATTATACTTTAAGTTTTA  Hsapiens
188102: ..C.............T.......                           Ptroglodytes
451486:                         .........................  Ptroglodytes
469042: ......T.C.......AA..---------....................  Ggorilla
503163: ........A.....G.T.                                 Pabelli
504771:                    C..CAG..--.C.CC.....A..G.A....   Pabelli
405254: .C...............A....                             Mmulatta
410130: ......T............                                Panubis
430572:                    C...GG..--.C.CC.....A..G.A....   Panubis
203249: ......T.........T..                                Cjacchus

                               LINE1
        ──────────────────────────────────────────────────►
                131803     131813     131823     131833     131843
131794: GGGTACGTGTGCACAATGTGCAGGTTAGTTACATATGTATACATGTGCCA  Hsapiens
451512: ......A..C........................................  Ptroglodytes
469083: ......A...........................................  Ggorilla

                               LINE1
        ──────────────────────────────────────────────────►
                131853     131863     131873     131883     131893
131844: TGTTGGTGTGCTGCACCCATTAACTCGTCATTTAGCATTAGGTATATCTC  Hsapiens
451562: ..C...............................................A  Ptroglodytes
469133: ..C...............................................  Ggorilla
```

```
                                  LINE1
        --------------------------------------------------->
                 131903    131913    131923    131933    131943
131894: CTAATGCTATCCCTCCCTGCCTCCCCCCACCCGACAACAGTCCCCAGAGT Hsapiens
451612: ................TCC.............C................. Ptroglodytes
469183: .................C-........T....C................. Ggorilla

                                  LINE1
        --------------------------------------------------->
                 131953    131963    131973    131983    131993
131944: GTGATGTTCCCCTTCCTGTGTTCATGTGTTCTCATTGTTCAATTCCCACC Hsapiens
451662: ..............................................G... Ptroglodytes
469232: .................................................. Ggorilla

                                  LINE1
        --------------------------------------------------->
                 132003    132013    132023    132033    132043
131994: TATGAGTGAGAACATGCGGTGTTTGGTTTTCTGTCCTAGCGATAGTTTAC Hsapiens
451712: .................................................. Ptroglodytes
469282: .................T................................ Ggorilla

                                  LINE1
        --------------------------------------------------->
                 132053    132063    132073    132083    132093
132044: TGAGAATGATGATTTCCAATTTCATCCATGTCCCTACAAAGGACATGAAC Hsapiens
451762: ........-......................................... Ptroglodytes
469332: .................................................. Ggorilla

                                  LINE1
        --------------------------------------------------->
                 132103    132113    132123    132133    132142
132094: TCATCATTTTTTATGGCTGCATAGTATTCCATGGTGTATA-CTTGTCACA Hsapiens
451811: ........................................-TG....... Ptroglodytes
469382: ........................................NNNNN..... Ggorilla

                                  LINE1
        --------------------------------------------------->
                 132152    132162    132172    132182    132192
132143: TTTTCTTAATCCAGTCTATCATTGTTGGACATTTGGATTGGTTCCAAGTC Hsapiens
451860: .................................................. Ptroglodytes
469432: .................................................. Ggorilla

                                  LINE1
        --------------------------------------------------->
                 132202    132212    132222    132232    132242
132193: TTTGCTATTGTGAATAATGCCGCAATAAACATATGTGTGCATGTGTCTTT Hsapiens
451910: ........................G........C................ Ptroglodytes
469482: .................................................. Ggorilla

                                  LINE1
        --------------------------------------------------->
                 132252    132262    132272    132282    132292
132243: ATAGCAGCATGATTTATAGCACTTTGGGTATATACCCAGTAATGGGATGG Hsapiens
451960: ..................TTC............................. Ptroglodytes
469532: .................................................. Ggorilla
```

```
                                LINE1
         ------------------------------------------------------>
                  132302    132312    132322    132332    132342
                       :         :         :         :         :
132293:  CTGGGTCAAATGGTATTTCTAGTTCTAGATCCCTGAGGAATCACCACACT  Hsapiens
452010:  .........................................TG.......  Ptroglodytes
469582:  ...................C...........G..........G.......  Ggorilla

                                LINE1
         ------------------------------------------------------>
                  132352    132362    132372    132382    132392
                       :         :         :         :         :
132343:  GACTTCCACAATGGTTGAACTAGTTTACAGTCCCACCAACAGTGTAAAAG  Hsapiens
452060:  ..................................................  Ptroglodytes
469632:  ..................................................  Ggorilla

                                LINE1
         ------------------------------------------------------>
                  132402    132412    132422    132432    132442
                       :         :         :         :         :
132393:  TGTTCCTATTTCTCCACATCCTCTCCAGCACCTGTTGTTTCCTGACTTTT  Hsapiens
452110:  ..................................................  Ptroglodytes
469682:  ..................................................  Ggorilla

                                LINE1
         ------------------------------------------------------>
                  132452    132462    132472    132482    132492
                       :         :         :         :         :
132443:  TAATGATCGTCATTCTAATTGGTGTGAGATGGTATCTTATTGTGGTTTTG  Hsapiens
452160:  .......T.C...........................C............  Ptroglodytes
469732:  .........C...........................C............  Ggorilla

                                LINE1
         ------------------------------------------------------>
                  132502    132512    132522    132532    132542
                       :         :         :         :         :
132493:  ATTTGCATTTCTCTGATGGCCAGTGATGGTGAGCATTTTTTCATGTGTTG  Hsapiens
452210:  ............................A....................T  Ptroglodytes
469782:  ............................A....................T  Ggorilla

                                LINE1
         ------------------------------------------------------>
                  132552    132562    132572    132582    132592
                       :         :         :         :         :
132543:  TTTGGCTGCATAAATGTCTTCTTTTGAGAAGTGTCTGTTCATGTCCTTTG  Hsapiens
452260:  ................................................C.  Ptroglodytes
469832:  ................................................C.  Ggorilla

                                LINE1
         ------------------------------------------------------>
                  132602    132612    132622    132632    132642
                       :         :         :         :         :
132593:  CCCACTTGTTGATGGGGTTGTTCATTTTTTTCTTGTAAATTTGTTTGAGT  Hsapiens
452310:  .......T..........................................  Ptroglodytes
469882:  .......T..........................................  Ggorilla

                                LINE1
         ------------------------------------------------------>
                  132652    132662    132672    132682    132692
                       :         :         :         :         :
132643:  TCATTGTAGATTCTGGATATTAGCCCTTTGTCAGATGAGTAGGTTGCGAA  Hsapiens
452360:  ................T.............................T...  Ptroglodytes
469932:  ..............................................T...  Ggorilla
```

```
                              LINE1
          ------------------------------------------------->
                   132702    132712    132722    132732    132742
132693: AATTTTCTCCCATTTTGTAGGTTGCCTGTTCACTCTGATGGTAGTTTCTT  Hsapiens
452410: ................................................C.  Ptroglodytes
469982: ..................................................  Ggorilla

                              LINE1
          ------------------------------------------------->
                   132752    132762    132772    132782    132792
132743: TTGCTGTGCAGAAGCTCTTTAGTTTAATTAGATCCCATTTGTCAATTTTG  Hsapiens
452460: ............CA....................................  Ptroglodytes
470032: ............CA....................................  Ggorilla

                              LINE1
          ------------------------------------------------->
                   132802    132812    132822    132832    132842
132793: GCTTTTGTTGCCATTGCTTTTGGTGTTTTAGACACGAAGTCCTTGCCCAT  Hsapiens
452510: ..................................................  Ptroglodytes
470082: ..............................A...G...............  Ggorilla

                              LINE1
          ------------------------------------------------->
                   132852    132862    132872    132882    132892
132843: GCCTATGCCCTGAATGGTAATGCCTAGGTTTTCTTCTAGGGTTTTTATGG  Hsapiens
452560: ...........C......................................  Ptroglodytes
470132: ..................................................  Ggorilla

                              LINE1
          ------------------------------------------------->
                   132902    132912    132922    132932    132942
132893: TTTTAGGTCTAACATTTAAGTCTTTAATCTATCTTGAATTAATTTTTGTA  Hsapiens
452610: .............G...............C....................  Ptroglodytes
470182: .............G...............C...................T  Ggorilla

                              LINE1
          ------------------------------------------------->
                   132952    132962    132972    132982    132992
132943: TAAGGTGTAAGGAAGGGATCCAGTTTCAGCTTTCTACATATGGCTAGCCA  Hsapiens
452660: ..................................................  Ptroglodytes
470232: ..................................................  Ggorilla

                              LINE1
          ------------------------------------------------->
                   133002    133012    133022    133032    133042
132993: GTTTTCCCAGCACCATTTATTAAATAGGGAATCCTTTCCCCATTGCTTGT  Hsapiens
452710: ..................................................  Ptroglodytes
470282: ..................................................  Ggorilla

                              LINE1
          ------------------------------------------------->
                   133052    133062    133069    133079    133089
133043: TTTTCTCAGGTTTGTCAAAG---ATCAGATAGTTGTAGATATGCGGCGTT  Hsapiens
452760: ....................ATC...........................  Ptroglodytes
470332: .......G............ATC..................G.T..T...  Ggorilla
```

```
                                LINE1
         ------------------------------------------------->
                133099    133109    133119    133129    133139
                     :         :         :         :         :
133090: ATTTCTGAGGCCTCTGTTCTGTTCCATTGATCCGTATCTCTGTTTTGGTA  Hsapiens
452810: .................................A................  Ptroglodytes
470382: .................................AC...............  Ggorilla
```

```
                                LINE1
         ------------------------------------------------->
                133149    133159    133169    133179    133189
                     :         :         :         :         :
133140: CCAGTACCATGCTGTATTGGTTACTGTAGCCTTGTAGTATAGTTTGAAGT  Hsapiens
452860: ...............G..................................  Ptroglodytes
470432: T.................................................  Ggorilla
```

```
                                LINE1
         ------------------------------------------------->
                133199    133209    133219    133229    133239
                     :         :         :         :         :
133190: CGGGTAGCGTGACACCTCCAGCTTTGTTCTTTTGGCTTAGGATTGACTTG  Hsapiens
452910: .A......A.........................................  Ptroglodytes
470482: .A................................................  Ggorilla
```

```
                                LINE1
         ------------------------------------------------->
                133249    133259    133269    133279    133289
                     :         :         :         :         :
133240: GCGATGCGGGCTCTTTTTTGGTTCCATATGATCTTTAAACTAGTTTTTTC  Hsapiens
452960: ...............................A.......G..........  Ptroglodytes
470532: ...............................A.......G..........  Ggorilla
```

```
                                LINE1
         ------------------------------------------------->
                133299    133309    133319    133329    133339
                     :         :         :         :         :
133290: CAATTCTGTGAAGAAAGTCATTGGTAGCTTGATGGGGATGGCATTGAATC  Hsapiens
453010: ..................................................  Ptroglodytes
470582: ..................................................  Ggorilla
```

```
                                LINE1
         ------------------------------------------------->
                133349    133359    133369    133379    133389
                     :         :         :         :         :
133340: TATAAATTACCTTGGGCAGTATGGCCATTTTCACGATATTGATTCTTCCT  Hsapiens
453060: .................................T................  Ptroglodytes
470632: ..................................A...............  Ggorilla
```

```
                                LINE1
         ------------------------------------------------->
                133399    133409    133419    133429    133439
                     :         :         :         :         :
133390: ACCCATGAGCATGGAATGTTCTTCCATTTCTTTGTATCCTCTTTTATTTC  Hsapiens
453110: ..................................................  Ptroglodytes
470682: ..................................................  Ggorilla
```

```
                           LINE1                   LINE1
         ----------------------------------------->------->
                133449    133459    133469    133479    133489
                     :         :         :         :         :
133440: ACTGAGCAGTGGTTTGTAGTTCTCCTTGAAGAGGTCCTTCATTTATTCAT  Hsapiens
453160: .T................................................  Ptroglodytes
470732: .T................................................  Ggorilla
```

```
                                 LINE1
        ---------------------------------------------------->
            133499    133509    133519    133529    133539
133490: TTCTTCTAGATTTTCTAGTTTATTTGCGTAGAGGTGTTTGTAGTATTCTC  Hsapiens
453210: ...............................................A..  Ptroglodytes
470782: ...........................A......................  Ggorilla

                                 LINE1
        ---------------------------------------------------->
            133549    133559    133569    133579    133587
133540: TGATAGTAGTTTGTATTTCTGTGGGATCAGTGGTGATATCCCCTTTA--T  Hsapiens
453260: ....G..........................................--.  Ptroglodytes
470832: ....G..........................................--.  Ggorilla
483462:                                       ....T...TGT.  Mmulatta
430347:                                       ....T...TGT.  Panubis

                                 LINE1
        ---------------------------------------------------->
            133597    133607    133614    133624    133634
133588: CATTTTTTATTGCGTCTATTTGATT---CTTCTCTCTTTTTTTCTTTATT  Hsapiens
453308: .........................---......................  Ptroglodytes
470880: .........................---......................  Ggorilla
504564:    CCC.....TTT.TC.GC.AC..TCA..AT.....C..----......  Pabelli
483474: A..CCC.....TT...C.G...C..TCA..AT.....C...G....C...  Mmulatta
430359: A..CCC.....TT...C.GC..C..TCA..AT.....C...G....C...  Panubis

                           LINE1                 LINE1
        ----------------------------------------->---------->
            133639    133649                    133662
133635: AGT-----CTTGCTAGCAGTCTATC-------AATTTTGTT---------  Hsapiens
453355: ...-----...A.............-------.........---------  Ptroglodytes
470927: ...-----.................-------.........---------  Ggorilla
504607: TT.GACAGT...A.T.T.T.A.G..CTTTTGT.GA......---------  Pabelli
483524: TT.GACAGT...A.TAT.T.A....CTTTTGT.GA......NNNNNNNNN  Mmulatta
430409: TT.GACAGT...A.TAT.T.A.G..CTTTTGT.GA......---------  Panubis

                                 LINE1
        ---------------------------------------------------->
            133670    133680    133690    133700    133710
133664: ---TGGACTAAATCTGACTGGAGAATTTTGACCTTCTTATATCTGGATAC  Hsapiens
453384: ---.........................................A.....  Ptroglodytes
470956: ---.................A.............................  Ggorilla
504648: ---...............................................  Pabelli
483574: NNNNNNN..G..........C..........T..................  Mmulatta
430450: ---...............................................  Panubis

                                 LINE1
        ---------------------------------------------------->
            133720    133730    133738    133748    133758
133711: TTATATTTTTCTCCAAATTTGGAAGG--TTTTTTGATATTATATCTTAAA  Hsapiens
453431: ...C......................--......................  Ptroglodytes
471003: ..........................--......................  Ggorilla
504695: ..........................--.........G............  Pabelli
483624: .............-..........A.TT......A............T..  Mmulatta
430497: .............-..........A.--......A..........ACT..  Panubis
```

```
                              LINE1
         ------------------------------------------------->
                 133768    133778    133788    133798    133808
133759: ATATGCTTTTTGTTCCTTTTTCTTTCTTCTTCAGAAACTTCTATACATTG  Hsapiens
453479: .......................---........................  Ptroglodytes
471051: ..................................................  Ggorilla
504743: ..C..............C.....................C..........  Pabelli
483673: ...........A....G......G.......TG......C..........  Mmulatta
430544: ...........A....A......G.......TG......C..........  Panubis

                              LINE1
         ------------------------------------------------->
                 133817    133827    133837    133847    133857
133809: AATTTTT-ATACTTTTCCATAAACCCTGTAACCATTGTTTCTTCCTTTTA  Hsapiens
453526: .......-..........................................  Ptroglodytes
471101: .......-..........................................  Ggorilla
504793: .......-......................G................---  Pabelli
483723: .......T.......................G............T.....  Mmulatta
430594: .......T.......................G.G..........T.....  Panubis

                              LINE1
         ------------------------------------------------->
                 133867    133877    133887    133897    133904
133858: TTTTTTTCTTCTTCTCTGACTGTATATTTTCAAATAATCTGTC---AAGT  Hsapiens
453575: ...........................................---....  Ptroglodytes
471150: ...........................................---....  Ggorilla
504839: ....................A......................---....  Pabelli
483773: ....................CA.....................TTC....  Mmulatta
430644: ...........................................TTC....  Panubis

                              LINE1
         ------------------------------------------------->
                 133914    133924    133934    133944    133954
133905: TTACAGATTCTTTCTTCTGTTTGATCAATTCTGCTGTTGATGCTTTCTAC  Hsapiens
453622: ..................................................  Ptroglodytes
471197: ..................................................  Ggorilla
504886: ......................A...........................  Pabelli
483823: .C.......G..........................C.............  Mmulatta
430694: .C.......G..........................C.............  Panubis

                              LINE1
         ------------------------------------------------->
                           133972    133982    133992    134002
133955: TGCATTTTT--ATTTTATCCATTGTATTTTTCAGCTCTAGAATTTGTTTG  Hsapiens
453672: .........--......C............................----  Ptroglodytes
471247: .........--......C............................----  Ggorilla
504936: .........--...................................----  Pabelli
483873: ..A......TT................................CCAC..-  Mmulatta
430744: ..A......-T................................CCAC..-  Panubis
```

```
                                LINE1
        ------------------------------------------------->
            134012    134022              134040    134050
                 :         :                   :         :
134003: ATTTTTTTTTTTTAATAATTTCAGTCTCT--GTTAAATTTCCAATTTTCAT  Hsapiens
453716: --A.......AA........A.......--....................  Ptroglodytes
471291: --A........A................--....................  Ggorilla
504980: --A........A................CT....................  Pabelli
483922: --GC....AAAA................CT..........T.........  Mmulatta
430792: --GC......AA................CT..........T.........  Panubis
```

```
                                LINE1
        ------------------------------------------------->
            134060    134070    134080    134090    134100
                 :         :         :         :         :
134051: CATTTATTGTTTTTCTGTTTTCATTGATTGTTTTTCTGTGCTTTCTTTAG  Hsapiens
453762: ..................................................  Ptroglodytes
471337: ..................................................  Ggorilla
505028: ........................................G........A  Pabelli
483970: ....G...................CA........................  Mmulatta
430840: ....G.................T.C.........................  Panubis
```

```
                                LINE1
        ------------------------------------------------->
                      134119    134129    134139    134149
                           :         :         :         :
134101: GTTCCTTGA-TTTTTTTCAAACTTTTATTTTGAATTATTTGTCAGCCAGT  Hsapiens
453812: .........T........................................  Ptroglodytes
471387: .........T........................................  Ggorilla
505078: ......C..T..C......G..............................  Pabelli
484020: .........-.......A...T............................  Mmulatta
430890: .........-.......TT..T............................  Panubis
```

```
                                LINE1
        ------------------------------------------------->
            134159    134169    134179    134189    134199
                 :         :         :         :         :
134150: TCTTCCATCTTCATTTCTTTGGAGTCAGCTACTGGGAAGTTATTTTGTTC  Hsapiens
453862: .....T............................................  Ptroglodytes
471437: .....T............................................  Ggorilla
505128: .....T.C..........................................  Pabelli
484069: ....AT.C..............................A...........  Mmulatta
430939: ....AT................................A...........  Panubis
```

```
                                LINE1
        ------------------------------------------------->
            134209    134219    134229    134239    134249
                 :         :         :         :         :
134200: TTTTGGTAGTGTTATGTCTCCTTGGTTTTTTATGTTTGTTGTTGTCTTAC  Hsapiens
453912: ...........C......................................  Ptroglodytes
471487: ..................................................  Ggorilla
505178: ..............................--..................  Pabelli
484119: ...A...G.....................GC......C....C.C.....  Mmulatta
430989: ...A...G.....................GC......CC.....C.....  Panubis
```

```
                                     LINE1
         ------------------------------------------------------------>
                  134259    134269    134279    134288    134298
134250: CTGGATGTCTGCATGTTTGAAGAAGCAGATAATT-TTCCAGTCTTTGCAG  Hsapiens
453962: ..................................-...............  Ptroglodytes
471537: ..................................-...............  Ggorilla
505226: ..................................-.....A.....T...  Pabelli
484169: A........................T..GG....A...............  Mmulatta
431039: AC.......................T..GG....A...............  Panubis
```

```
                                     LINE1
         ------------------------------------------------------------>
                  134308    134318    134328    134338    134348
134299: ACTGGCTTTCTCTGGGAAAGTCCTTCACCAGTTATTCCATTCAGAGATTC  Hsapiens
454011: ..................................................  Ptroglodytes
471586: .....................................A...T........  Ggorilla
505275: ..................................................  Pabelli
484219: .....T...-..........C...............T.............  Mmulatta
431089: .....T...-..........C.......T.......T.............  Panubis
```

```
                                     LINE1
         ------------------------------------------------------------>
                  134358    134368    134378    134388    134398
134349: TGGGCAGGCCATTGGGCAGGTTTGCTGCTGGAGTCCTCAGTAAGGCTGGC  Hsapiens
454061: .......C..........................................  Ptroglodytes
471636: .......C................................C.........  Ggorilla
505325: ..................................................  Pabelli
484268: ...-..A......-...............A..........G...T..AT.  Mmulatta
431138: ...-.................A..........A.......A......AT.  Panubis
```

```
                                     LINE1
         ------------------------------------------------------------>
                  134408    134418    134428    134438    134448
134399: CTGGTGCTGAGTCAATGAGTGGTCAGGTCTGTTGCTTGAGTCCTGAGAGT  Hsapiens
454111: ........................T............C............  Ptroglodytes
471686: ..................................................  Ggorilla
505375: ..............G....................C..............  Pabelli
484316: ..............G....................C......T.......  Mmulatta
431187: ..............G....................C......T.......  Panubis
```

```
                                     LINE1
         ------------------------------------------------------------>
                  134458    134468    134478    134488    134498
134449: TGGCCCTTAAGTATAGATTCAATGGGGTTGACCTGTTGATTGAGTCCCCA  Hsapiens
454161: ............T.....................................  Ptroglodytes
471736: ............T.....................................  Ggorilla
505425: ...........CT........C............................  Pabelli
484366: ...........CT........C.................C...A......  Mmulatta
431237: ...........CT........C.................C...A......  Panubis
```

```
                                   LINE1
        ------------------------------------------------------------->
             134508    134518    134528    134538    134548
                  :         :         :         :         :
134499: AAGATCTAACTGGAGCCCAGGTCCATGAGGACAAGCCTGAAGCCTGCAGC  Hsapiens
454211: ........C.........................................  Ptroglodytes
471786: ........C.........................................  Ggorilla
505475: ........C.......................................T.  Pabelli
484416: ........-..........A...........A.........A......T.  Mmulatta
431287: ........C..A........A...........T.........G.....T.  Panubis
```

```
                                   LINE1
        ------------------------------------------------------------->
             134557    134566    134576    134586    134596
                  :         :         :         :         :
134549: C-ACAGGGCCAGCCT-AAGCCTGGGTCCACCAGGGCCAGCCAACCTGGCA  Hsapiens
454261: .-.............-..................................  Ptroglodytes
471836: .-.............-..................................  Ggorilla
505525: .T.............-.......................TG.........  Pabelli
484465: G-.T...........G......................----.....A..  Mmulatta
431337: .-..........A..G...........T........G.----.....A..  Panubis
```

```
                                   LINE1
        ------------------------------------------------------------->
             134606    134616    134626    134636    134646
                  :         :         :         :         :
134597: CTGGGATAGTCTGTGAGCTTGAGTCTTCAAAGAATGGCCAAACACTGAGA  Hsapiens
454309: ..................................................  Ptroglodytes
471884: ..................................................  Ggorilla
505574: ...................................A..............  Pabelli
484510: ......C..........T.....C.........CCA..............  Mmulatta
431382: ......C....--....T.....C.........C........T.......  Panubis
```

```
                                   LINE1
        ------------------------------------------------------------->
             134656    134666    134676    134686    134696
                  :         :         :         :         :
134647: TGGGACTGACACCTTGGTCCACTGGTTTGGGCTTGGAGCCTCAATCTGTG  Hsapiens
454359: ..................................................  Ptroglodytes
471934: ..................................................  Ggorilla
505624: ........G...A............................G........  Pabelli
484560: ...........T......................................  Mmulatta
431430: ................T.........C.......................  Panubis
```

```
                                   LINE1
        ------------------------------------------------------------->
             134706    134716    134726    134736    134746
                  :         :         :         :         :
134697: TGGGTACATCTGAGCCTTTAGTTCCCATAGTTTGGCCTGGATCCTGAGTC  Hsapiens
454409: ..................G...............................  Ptroglodytes
471984: ..................G...............................  Ggorilla
505674: ..................G.....A.....A...................  Pabelli
484610: ................C.G.....AAG.........T.............  Mmulatta
431480: .............A....G.....AAG.......................  Panubis
```

```
                              LINE1
          ------------------------------------------------------->
                   134756    134766    134776    134786    134796
                        :         :         :         :         :
134747: CACTGGGGAAGGTCTTGGTCCTGGGTCCATGGGAGCTGGCCTGAAGCCTG   Hsapiens
454459: .................T................................   Ptroglodytes
472034: ..........................                           Ggorilla
505724: TG.............GCA.........T..........A...........   Pabelli
484660: ...............GT...T........C....................   Mmulatta
431530: ..T............GT...T..................T..........   Panubis

                              LINE1
          ------------------------------------------------------->
                   134806    134816    134826    134836    134846
                        :         :         :         :         :
134797: GGTCTGCAGGAGTGGTCTTGGAGCCTCAGTCCACAAAGTATTGCACACAC   Hsapiens
454509: ..................................................   Ptroglodytes
505774: ........................................C.........   Pabelli
484710: .....A........A.........A...................TG....   Mmulatta
431580: .....AT.......A.........A...................TG....   Panubis

                              LINE1
          ------------------------------------------------------->
                   134856    134866    134876    134886    134896
                        :         :         :         :         :
134847: TGGAATCTACTGAGGTGGGCCTGGACCCTTGATATCCTGGAGTAAGCCTA   Hsapiens
454559: ..................................................   Ptroglodytes
505824: ..................................................   Pabelli
484760: .A.................T.......A..........A........T..   Mmulatta
431630: .A.................T.......A..........A........T..   Panubis

                              LINE1
          ------------------------------------------------------->
                   134906    134916    134926    134936    134946
                        :         :         :         :         :
134897: GACCCTGGGTCTTCTGGAGCCTGGGACAGAAGAAACAGCCTGGATGCTGG   Hsapiens
454609: ..............................G..............A....   Ptroglodytes
505874: ..............................G..............A....   Pabelli
484810: ..............................G.G............A..T.   Mmulatta
431680: ..............................G.G............A....   Panubis

                              LINE1
          ------------------------------------------------------->
                   134956    134966    134976    134986    134996
                        :         :         :         :         :
134947: TGCTGTGGTCAGCATGGAGCCTGGGCTCACTAAGGCTAGTCTGAAGTCCA   Hsapiens
454659: ..................................................   Ptroglodytes
505924: ...................................T............T.   Pabelli
484860: ....C...C..C......A...........G.......AC......C.T.   Mmulatta
431730: ....C...C..C......A....................C......C.T.   Panubis

                              LINE1
          ------------------------------------------------------->
                   135006    135016    135026    135036    135046
                        :         :         :         :         :
134997: GAATAATGAGCACTTGTTTGTACCTAGAGCCACTGTGGCTGGCCTGGACC   Hsapiens
454709: ..................................................   Ptroglodytes
505974: ........G.......C.................................   Pabelli
484910: ........GCT.....C...........A......A..............   Mmulatta
431780: ..G.....G.T.....C...........A......A..............   Panubis
```

```
                                   LINE1
        ------------------------------------------------------------>
              135056    135066    135076    135086    135096
                   :         :         :         :         :
135047: CTTGGGTGACAGAAACTAGCATGGGCCTTGTGTCCTCTGGTGCTTTCCTG   Hsapiens
454759: .................................................A   Ptroglodytes
506024: ....A.............................................   Pabelli
484960: .....T....................T..-....................   Mmulatta
431830: .....T....................T..-....................   Panubis

                                   LINE1
        ------------------------------------------------------------>
              135106    135116    135126    135136    135146
                   :         :         :         :         :
135097: GAGGCTGAGTGTGCTAGAGGATAATTCTTCACAGGATAACCTGGGTCCTG   Hsapiens
454809: ..................................................   Ptroglodytes
471761:                                  ..T.G.....TTGAT..   Ggorilla
506074: ...............................TG.A.......A......A   Pabelli
485009: ............................C..TG.......A.........   Mmulatta
431879: ............................C..TG.......A..A......   Panubis

                                   LINE1
        ------------------------------------------------------------>
              135156    135166    135176    135186    135196
                   :         :         :         :         :
135147: GGGGCTTGAGGACTAGCCTGGGACTCAGATACACAAAGACAATCCTGGAG   Hsapiens
454859: ..................................................   Ptroglodytes
471778: A.TC.CCA.A..TCTA.....AG.C...G.C..TG.G.....G....A..   Ggorilla
506124: ..A...............................................   Pabelli
485059: .......C.......A........C........T..G......A......   Mmulatta
431929: .......C.......A......G.C...........G......A......   Panubis

                                   LINE1
        ------------------------------------------------------------>
              135206    135216    135226    135236
                   :         :         :         :
135197: CCTGGGTTCATGGAAGCCAGTCCAACACTGGAGTCCAGAGGGACAGTT--   Hsapiens
454909: .............G..................................--   Ptroglodytes
471828: ....CAGC..CA.G-.....C.T..GC....GTC.ACC....C...CCAA   Ggorilla
506174: .............G..................................--   Pabelli
485109: ........T....G.............G.......-...A........--   Mmulatta
431979: ...A....T..................G...........A..G.....--   Panubis

                                   LINE1
        ------------------------------------------------------------>
              135254    135264    135274    135284    135294
                   :         :         :         :         :
135245: CTAGACCCTAGGTCTGTTACACAGGCTTTAAGCCTGCGGCTTCCAGATCT   Hsapiens
454957: .......A..................C.........G.............   Ptroglodytes
471877: .CT.G.A..G..--------.T..T..G.G...T..A.T....A.AGAA.   Ggorilla
506222: ..........................C.........G.............   Pabelli
485156: .........T................C..C......GA...A.....C..   Mmulatta
432027: .........T................C..C......GA...G.....C..   Panubis

                                   LINE1
        ------------------------------------------------------------>
              135304    135314    135324    135334    135344
                   :         :         :         :         :
135295: GGCCTGTCCCTTGGGCAAGTCTGGTTACTCAGTCCTCAGGTATTGGCCTG   Hsapiens
455007: .......................................T..........   Ptroglodytes
471919: ....AAA.A..GA.ATGG.A...ACAC..TG....A.T...T.G...T..   Ggorilla
506272: .A.................C..............................   Pabelli
485206: ............A.............................CA......   Mmulatta
432077: ............A..T..........................CA......   Panubis
```

LINE1

```
                135354    135364    135374    135384    135394
135345: GAATCTGGAGCAATGAGGGCCTGCGTAGTGCTGGGCTTTGCTATGGCAAG   Hsapiens
455057: ........................C.........................   Ptroglodytes
471969: ..GC..CA.T.TG..T...------..CAT...A..C...----------   Ggorilla
506322: ........................C...A.....................   Pabelli
485256: ........................C.........................   Mmulatta
432127: ........................C.........................   Panubis
```

LINE1

```
                135404    135414    135424    135434    135444
135395: CTAGTTGCACTGGGATGTCAGGTAAACTTCAATGCTTACTTCCGTCTCCT   Hsapiens
455107: ...........................................T......   Ptroglodytes
472003: --....C.CA.A.TT..--------G.C.GG..C..G.G.C.--------   Ggorilla
506372: .....CA...................T................C......   Pabelli
485306: A.......C..C..........C....................C.T....   Mmulatta
432177: A.......C..T..........C....................C......   Panubis
```

LINE1

```
                135454    135464    135474    135484    135494
135445: TCCTCCATGCATTATCTCTCTCTGCATTGTGCTGCCGAGGATTGGGGAAG   Hsapiens
455157: .........................................C........   Ptroglodytes
472035: ----------------------------------------.C........   Ggorilla
506422: ..................................................   Pabelli
485356: ..........................C........TA.......T....A   Mmulatta
432227: ........A.................C........T.............A   Panubis
```

LINE1

```
                          135503    135513    135523    135533
135495: -----------GGGTGATGTGGGTAATGTAAAACTGTCTTTCCTATACTC   Hsapiens
455207: -----------.......................................   Ptroglodytes
472045: GTCTTGGTCCT.......................................   Ggorilla
506472: -----------.............................C.........   Pabelli
485406: -----------.A...........G.........................   Mmulatta
432277: -----------.............G.........................   Panubis
```

LINE1

```
                135543    135553    135563    135573    135583
135534: TTTAATGTGTCTTTTCTCATTTCTGTGCTACATCCAAGTGTTGAAATCTC   Hsapiens
455246: ..................................................   Ptroglodytes
472095: ..C...............................................   Ggorilla
506511: ...................C.................A.....T......   Pabelli
485445: .C.........................................T......   Mmulatta
432316: .C.........................................T......   Panubis
```

```
                           LINE1
        ------------------------------------------------->
            135593    135603    135613    135623    135633
                 :         :         :         :         :
135584: TTACCTGGATTCCTTAATCCTTGTGAAGGTAGTTTCATGAATTGGTGGTT  Hsapiens
455296: .................................A................  Ptroglodytes
472145: ..................................................  Ggorilla
506561: ......A.T......................T..................  Pabelli
485495: .......TT.A....................T......T...G.......  Mmulatta
432366: .......TT.A....................T......T...G.......  Panubis

                           LINE1
        ------------------------------------------------->
            135643    135653    135663    135673    135683
                 :         :         :         :         :
135634: ATTCAAATTGATGTTTCTGTAGGGGGATAAATACTGGAGAGTCCTATTTC  Hsapiens
455346: .........................................C........  Ptroglodytes
472195: .........................................C........  Ggorilla
506611: ............A............................C......C.  Pabelli
485545: .......C.............A...........G..............C.  Mmulatta
432416: .......C.............A...........G..............C.  Panubis

             LINE1
        --------------->
            135693    135703    135713    135723    135733
                 :         :         :         :         :
135684: ACCATCTTGCTGATGTGCATCCTAAACATGTTTTTAAGAGCAACATTATT  Hsapiens
455396: ..................................................  Ptroglodytes
472245: ..................T..............................C  Ggorilla
506661: ..................................................  Pabelli
485595: ................A.................................  Mmulatta
432466: ................A.................................  Panubis

                                                     LTR
                                                -------->
            135743    135753    135760              135773
                 :         :         :                   :
135734: CTTACTGAATAATGGTATGGTT---ATTCAGTAA-------GTATGGTTG  Hsapiens
455446: ......................---.........-------.........  Ptroglodytes
472295: ......................---.........-------.....A...  Ggorilla
506711: ........T.TT..ACCAATC.TCA...T.T.T.CATACTA.........  Pabelli
485645: ........T.GT..ACCA.TC.TCA...T.T.T.CATACTG........T  Mmulatta
432516: ........T.GT..ACCA.TC.TCA...T.T.T.CATACTG........T  Panubis

                           LTR
        ------------------------------------------------->
            135783    135793    135803    135813    135823
                 :         :         :         :         :
135774: GCCTCCATGTCCCCACCCAAATCTCATGTTGAATTGCGATCCCAAGTGTT  Hsapiens
455486: ....T...............................T.............  Ptroglodytes
472335: ....T.....T.........................T.............  Ggorilla
506761: .G..T...............................T.............  Pabelli
485695: .G..T................C.....C........T.............  Mmulatta
432566: .G..T................C.....C........T.............  Panubis
```

```
                               LTR
        ──────────────────────────────────────────────────►
             135833    135843    135853    135863    135873
135824: GGAGGTGGTGCCTGGTGGGAGGTGATTGTGTCATAGAGATGGTTTCTAAT  Hsapiens
455536: ..................................................  Ptroglodytes
472385: ....T.............................................  Ggorilla
506811: ....A...............A...............G.............  Pabelli
485745: ................A.................G.G.............  Mmulatta
432616: ................A.................G.G.............  Panubis
```

```
                               LTR
        ──────────────────────────────────────────────────►
             135883    135893    135902    135912    135922
135874: GGCTTAGTGCCATCCCCCCA-TGCTGTCTTGTGATAGAATTCTCATAAGA  Hsapiens
455586: ....................-.............................  Ptroglodytes
472435: ...................G-.............................  Ggorilla
506861: ..T.......T.......AG-.............................  Pabelli
485795: .......C.T........T.G....AAA......................  Mmulatta
432666: .......C..........T.G....AAA......................  Panubis
```

```
                               LTR
        ──────────────────────────────────────────────────►
             135932    135942    135952    135962    135972
135923: TCTGATTGTTTGAAAGTGTGTAGCACCTCCCACTTTCCTCTCTCTCTTCC  Hsapiens
455635: ..................................................  Ptroglodytes
472484: .......................................T..........  Ggorilla
506910: ..................................................  Pabelli
485845: ..A..C..A..A..................TG......C...........  Mmulatta
432716: C.......A..A.....A..........T.TG..................  Panubis
```

```
                               LTR
        ──────────────────────────────────────────────────►
             135982    135992    136002    136012    136022
135973: TCCTGCTTTAGTCATGTAGGACATGCCAGCTTCCCCATAGACTTCCACCA  Hsapiens
455685: ..................................................  Ptroglodytes
472534: ..................................................  Ggorilla
506960: ................................T.................  Pabelli
485895: ......A.....G...................A.......C.........  Mmulatta
432766: ......A...A.G...................A.......C.........  Panubis
```

```
                               LTR
        ──────────────────────────────────────────────────►
             136032    136042    136052    136062    136072
136023: TGACTGTAGGTTTCCTGAAGCCTCCCCATTCATAGATCCTGTGCAGCCTG  Hsapiens
455735: ........................................A.........  Ptroglodytes
472584: ..................................................  Ggorilla
507010: ...................................G..............  Pabelli
485945: ...T..............G.......TC.....G........A......A  Mmulatta
432816: ...T..............G.......T...............A.......  Panubis
```

```
                                     LTR
        ------------------------------------------------->
                  136082     136092     136102     136112     136122
136073: TGGAAATATGAGCCAATTAAATATGACTTTTAAATAAACTACCAGTCTCA  Hsapiens
455785: ......C...........................................  Ptroglodytes
472634: ......C...........................................  Ggorilla
507060: .....CC...............................T...........  Pabelli
485995: .....CC.................A.............T...........  Mmulatta
432866: .....CC.................A.............T.....A.....  Panubis

                               LTR                      LTR
        -------------------------------------> ---------->
                  136132     136142     136152     136162     136172
136123: GGTAGTTCTTTATATCAATGTGAGAACAGACTAATACAGAAAATTGGTAC  Hsapiens
455835: ..................................................  Ptroglodytes
472684: ...G...................C..........................  Ggorilla
507110: ..................................................  Pabelli
486045: .........C....G......T......A..C..................  Mmulatta
432916: .........C....G.............A..C..................  Panubis

                                     LTR
        ------------------------------------------------->
                  136182     136192     136202     136212     136222
136173: AAGAGAAGTGGGGCATTGCTATAAAAATACCTGAAAATGTGGAAGCAGCT  Hsapiens
455885: ..............G......................C............  Ptroglodytes
472734: ..............G...................................  Ggorilla
507160: .................T................................  Pabelli
486095: .................C.......G..............A......A..  Mmulatta
432966: .................C.......G..............A.........  Panubis

                                     LTR
        ------------------------------------------------->
                  136232     136242     136252     136262     136272
136223: TTGGAACTAGGTAACAGGCAGAGGCTGGAACAGGTTGGAGGCCTCAGTAG  Hsapiens
455935: ...............................G........A.........  Ptroglodytes
472784: ...............................G........A.........  Ggorilla
507210: ..................................................  Pabelli
486145: ........................T.................T....A..  Mmulatta
433016: ........................T.................T....A..  Panubis

                                     LTR
        ------------------------------------------------->
                  136282     136292     136302     136312     136322
136273: AAGACATGAAGATAAGGGAAAGTTTGGAACTTCCTAGAGACTTGTTGAAT  Hsapiens
455985: ..................................................  Ptroglodytes
472834: .............................T....................  Ggorilla
507260: ...................................G..............  Pabelli
486195: .................A..................C.............  Mmulatta
433066: .................A..................C.............  Panubis
```

```
                              LTR
         ------------------------------------------------------>
                  136332    136342    136352    136362    136372
                       :         :         :         :         :
136323: GATTTTGACCAAATGCTGAAAGTGATATGGACAGTGAAGTCCAAGCTAAG  Hsapiens
456035: ...................T..............................  Ptroglodytes
472884: ...................T..............................  Ggorilla
507310: ...................T.......................G...G..  Pabelli
486245: ...................T.......................G...G..  Mmulatta
433116: ...................T.......................G...G..  Panubis

                              LTR
         ------------------------------------------------------>
                  136382    136392    136402    136412    136422
                       :         :         :         :         :
136373: GAGGTTTCAGATGGAGATGAGGAACTTATTGTGAACTTAATAAAGGTCAA  Hsapiens
456085: ..................................................  Ptroglodytes
472934: ..................................................  Ggorilla
507360: ...............................G..................  Pabelli
486295: A...G..........................G.................C  Mmulatta
433166: A..............................G.................C  Panubis

                              LTR
         ------------------------------------------------------>
                  136432    136442    136452    136461    136471
                       :         :         :         :         :
136423: TCTTGCTATGCTTTAGCAAAGAGACTGGTG-CATTGTGTCCTGTTCTAGG  Hsapiens
456135: ..............................G...................  Ptroglodytes
472984: ..............................G...................  Ggorilla
507410: ..............................G...................  Pabelli
486345: ..............................G...................  Mmulatta
433216: ..............................G...................  Panubis

                              LTR
         ------------------------------------------------------>
                  136481    136491    136501    136511    136521
                       :         :         :         :         :
136472: GATCTGTAGAACTTTGAACTTGAGAGAGATGATTTAGGGTATCTGGTGAA  Hsapiens
456185: ..................................................  Ptroglodytes
473034: ..................................................  Ggorilla
507460: ........C.........................................  Pabelli
486395: ........C-.....A......................T...........  Mmulatta
433266: ........C.............................T...........  Panubis

                              LTR
         ------------------------------------------------------>
                  136531    136541    136551    136561    136571
                       :         :         :         :         :
136522: CAAATTTCTAAGCAGCAAAGTATTCAAGATGTGGCCTGGCCTAGCCGCCT  Hsapiens
456235: T................................T...........T..T.  Ptroglodytes
473084: .................................T...........T..T.  Ggorilla
507510: .............................................T..T.  Pabelli
486444: .............................CA..............T..T.  Mmulatta
433316: .................................A...........T..T.  Panubis
```

```
                                LTR
         ------------------------------------------------->
                  136581     136591     136601     136611     136621
136572: CTAACAGCATACACTGATATGCATGCACAAAGAGATTATCTGAAACTGGA  Hsapiens
456285: ............................G.....................  Ptroglodytes
473134: ...........................GG.....................  Ggorilla
507560: ..................................................  Pabelli
486494: ................................T..............T..  Mmulatta
433366: ................................T..............T..  Panubis
```

```
                                LTR
         ------------------------------------------------->
                  136631     136641     136651     136661     136671
136622: ATTTACACTTAAAAGGGAAGCACAGCATAAAAGTTTGGAAAATTTGCAGC  Hsapiens
456335: ......................G...........................  Ptroglodytes
473184: ......................G...........................  Ggorilla
507610: .......T..............G...........................  Pabelli
486544: .......T..G...........G...........................  Mmulatta
433416: ...C...T..G...........G...........................  Panubis
```

```
                                LTR
         ------------------------------------------------->
                  136681     136691     136701     136711     136721
136672: CTGTCCATGTTGTATAAAAGACAAACCCATTTCTGGGAAGGAATTAAAGC  Hsapiens
456385: ..................................................  Ptroglodytes
473234: ..................................................  Ggorilla
507660: ........A.....G...................................  Pabelli
486594: .C............GG....GA............................  Mmulatta
433466: .C............G......A............................  Panubis
```

```
                                LTR
         ------------------------------------------------->
                  136731     136741     136751     136761     136771
136722: TGCCTGCAGAAATTTGCATAAGTAAAGAAGAGTCAAATATTTATAGCCAA  Hsapiens
456435: .........................................A........  Ptroglodytes
473284: .........................................A........  Ggorilla
507710: .........................................A........  Pabelli
486644: ............................G............A........  Mmulatta
433516: .................G..........G............A........  Panubis
```

```
                                LTR
         ------------------------------------------------->
                  136781     136791     136801     136811     136821
136772: GACAATAGGGAAAATGCCTCCAAGTCATTTTAAAGACCATTACAGCAGCC  Hsapiens
456485: ..................................................  Ptroglodytes
473334: ..................................................  Ggorilla
507760: ......G...........................................  Pabelli
486694: ......G.A.......................G.................  Mmulatta
433566: ..A...G.A.......................G.................  Panubis
```

```
                          LTR                          Simple
          136831    136841    136851    136861    136871
136822: CCTCCCGTCACAGGCCTGGATGCCTAGGAGGGAAAAATGGTTTTGTGGGC  Hsapiens
456535: ......A...........................................  Ptroglodytes
473384: ......A...........................................  Ggorilla
507810: ....A.AC.......................C.....C............  Pabelli
486744: ......A..........C....................A...........  Mmulatta
433616: ......A..........T....................A...........  Panubis

              Simple                    LTR
          136881    136891    136901    136911    136921
136872: CAGGGCCAGGGCCAGGGCCAGGGCCCTGCTGCTCTGTGCAGCCTCAAGAC  Hsapiens
456585: .............------...............................  Ptroglodytes
473434: .............------...............................  Ggorilla
507860: .............------------.........................  Pabelli
486794: ....C........------------.........................  Mmulatta
433666: ....C........------------.........................  Panubis

                            LTR
          136931    136941    136951    136961    136970
136922: AAGGTGCCCTGCATCCCAGCTGCTCTAGCTCCAGCCATGGTTAAG-GAAC  Hsapiens
456629: .............................................-....  Ptroglodytes
473478: ....G.T......................................-....  Ggorilla
507898: ............................................AA....  Pabelli
486832: .....T.............A....................C...AA....  Mmulatta
433704: .....T.............A....................C...AA....  Panubis

                            LTR
          136980    136990    137000    137010    137020
136971: CCCAGATACATCGCAGGCCACTGCTCCAAAGAGTGCAAGGTATAAGCCTT  Hsapiens
456678: ..................................................  Ptroglodytes
473527: ..................................................  Ggorilla
507948: ............T.........................A...........  Pabelli
486882: A...........T.........................T...........  Mmulatta
433754: ............T.........................T...........  Panubis

                            LTR
          137030    137040    137050    137060    137070
137021: GGCAGCTTCTGCATGGTTTTAAGCCTACGGGTACACAGAGGGCAAGAGTT  Hsapiens
456728: ............................A.....................  Ptroglodytes
473577: ............................A.....................  Ggorilla
507998: ..................................................  Pabelli
486932: ....A...CAT.................A.....................  Mmulatta
433804: ....A...CAT.................A.......T.............  Panubis
```

```
                                  LTR
        ------------------------------------------------------->
                 137080     137090     137100     137110     137120
137071: GAGGCTTGGAAGTCTCCACCTAGACTTCAGAGGAAGTATGGAAACACCTG Hsapiens
456778: .................................................. Ptroglodytes
473627: .................................................. Ggorilla
508048: .................................................. Pabelli
486982: .........G......T....................G............ Mmulatta
433854: .........G......T....................G............ Panubis
```

```
                                  LTR
        ------------------------------------------------------->
                 137130     137140     137150     137160     137170
137121: GATGTCCAGGCAGAAGTCTACTGCAGGAATGGAGCCCTTATGAAAAACTT Hsapiens
456828: .................................................. Ptroglodytes
473677: .................................................. Ggorilla
508098: .......................................C.......... Pabelli
487032: ..........A........G........G.........C...GG...... Mmulatta
433904: ..........A........G........G.........C...G....... Panubis
```

```
                                  LTR
        ------------------------------------------------------->
                 137180     137190     137200     137210     137220
137171: CTACAAGGGCAGTGCAGAAGGCAAACGTGGGGTTGGAGCCCTCACACAAA Hsapiens
456878: .................................................. Ptroglodytes
473727: .........................T........................ Ggorilla
508148: .................................................. Pabelli
487082: ............G.....G......T....................A... Mmulatta
433954: ............G.....G......T....................A... Panubis
```

```
                                  LTR
        ------------------------------------------------------->
                 137230     137240     137250     137260     137270
137221: GTGCCCATTGGGGCACTGCCTAGTGAAGCTGTGAGAGAAGAGCCACTGTG Hsapiens
456928: ..C............................................... Ptroglodytes
473777: ..C...C........................................... Ggorilla
508198: ..C....C..........................A...G........... Pabelli
487132: ..C....C.............CA....T...................... Mmulatta
434004: ..C....C.............C.....T..A................... Panubis
```

```
                                  LTR
        ------------------------------------------------------->
                 137280     137290     137300     137310     137320
137271: CTCCAGACCCCAGAATGGTAGATCTACCAACAGCTTGCACCATATGCCTG Hsapiens
456978: .................................................. Ptroglodytes
473827: .................................................. Ggorilla
508248: ............................................G..... Pabelli
487182: ..........T.....T......................T.......... Mmulatta
434054: ..........T.....T.......................T......... Panubis
```

LTR

```
                137330    137340    137350    137360    137370
                     :         :         :         :         :
137321: GAAAAGCAATAGGCACTCAATATCACCCTGTCAAAGCAGCCATAGGAGGC Hsapiens
457028: .................................................. Ptroglodytes
473877: .................................................. Ggorilla
508298: .......C...........C..........T.................T Pabelli
487232: A......C.................G.....T.......T..C....A.T Mmulatta
434104: A......C.................G.....T..........C....A.T Panubis
```

LTR

```
                137380    137390    137400    137410    137420
                     :         :         :         :         :
137371: ATACCTTTCAGAGGCACAGGGCAGAGCTGCCCAAGGCCTTAGACTCCCAC Hsapiens
457078: ..................A............................... Ptroglodytes
473927: .................................................. Ggorilla
508348: G......G...........A........................A..... Pabelli
487282: G....C.G....................................A..... Mmulatta
434154: G....C.G....................................A...G. Panubis
```

LTR

```
                137430    137440    137450    137460    137470
                     :         :         :         :         :
137421: CCCTTGTGTCAGTGAGGCCTGGACGTGAGACATGGAGTCAAAGGAGATTA Hsapiens
457128: .................................................. Ptroglodytes
473977: .................................................. Ggorilla
508398: T.............T................................... Pabelli
487332: ....A.........TA........A...T.....A............... Mmulatta
434204: ....G.........TA........A...T.....A............... Panubis
```

LTR

```
                137480    137490    137500    137510    137520
                     :         :         :         :         :
137471: TTTTGCAGCTTTAAGATTTAATGACTTCCCCACTGGGTTTCAGACTTACA Hsapiens
457178: G..............................G.................. Ptroglodytes
474027: ...............................G.................. Ggorilla
508448: ..............................TG.................. Pabelli
487382: ...........C..C...............TG.................. Mmulatta
434254: ...........C..C...............TG.................. Panubis
```

LTR

```
                137530    137540    137550    137560    137570
                     :         :         :         :         :
137521: TGGGACCTGTACCCCTTTGTTTTGGCAATTTCTTCCTTTTGGAATGGGAT Hsapiens
457228: .................................................. Ptroglodytes
474077: .................................................. Ggorilla
508498: ........A......................................... Pabelli
487432: .................................CT............... Mmulatta
434304: ...........T.....................C................ Panubis
```

```
                              LTR
          137580    137590    137600    137610    137619
137571: AATTCACACAATGCCTGTACCCCCATTGTATCTTCAAAGAAACCAACT-T  Hsapiens
457278: ...........A....................................-.  Ptroglodytes
474127: ................................................-.  Ggorilla
508548: ....T...........A........C......................A.  Pabelli
487482: ....T.......T.A.................................-.  Mmulatta
434354: ....T.........A.................................-.  Panubis

                              LTR
          137629    137639    137649    137659    137669
137620: TTTTTTTATTTTACAGGTTCATAGGCAGGAGGAACTTGCTTTGCCTCAGA  Hsapiens
457327: ..................................................  Ptroglodytes
474176: ..................................................  Ggorilla
508598: ..................................A...............  Pabelli
487531: ......-.........T..T............................C.  Mmulatta
434403: ......-.........T..T............................C.  Panubis

                              LTR
          137679    137689    137699    137709    137719
137670: TGAAACTTTGGACTTGGACGTTTGAGTTAATGCTGGAATGAGTTAAGACT  Hsapiens
457377: ..................................................  Ptroglodytes
474226: ..................................................  Ggorilla
508648: ..................T..............C................  Pabelli
487580: ...................A..............................  Mmulatta
434452: ..................................................  Panubis

                              LTR
          137729    137739    137749    137759    137769
137720: TTGGGAGACTGCTGGGAAGGCATGATTGTGTTTTGAAATGTAAGAAGGAC  Hsapiens
457427: ...........T......................................  Ptroglodytes
474276: ...........T..........................C...........  Ggorilla
508698: C..........T......................................  Pabelli
487630: ...........T......A......................G........  Mmulatta
434502: ...........T......A......................G........  Panubis

                   LTR                       LTR
          137779    137789    137799    137809    137819
137770: ATGAGATTTGGGAGGGGCTGGGGTAGAATGATATGGTTTGGTTCTATGTT  Hsapiens
457477: ..................................................  Ptroglodytes
474326: ..................................................  Ggorilla
508748: .........................................C........  Pabelli
487680: ..............A...CA...C...............A.C........  Mmulatta
434552: ..............A...CA...C.................C........  Panubis
```

```
                                 LTR
        ---------------------------------------------------->
             137829    137839    137849    137859    137869
                  :         :         :         :         :
137820: CCCAACCAAATCTCATGTTGAATTGTGATCCTGAGTGTTGGAGGTGGGGC   Hsapiens
457527: ..T...............................................   Ptroglodytes
474376: ..................................................   Ggorilla
508798: ....C.............................................   Pabelli
487730: ....C......................................A...A..   Mmulatta
434602: ....C......................................A...A..   Panubis

                                 LTR
        ---------------------------------------------------->
             137879    137889    137899    137909    137919
                  :         :         :         :         :
137870: CTGGTGGAAGATAATGGTATCATGGGGGCAGTTTCTAATGGTTTAGCACC   Hsapiens
457577: .......G..........................................   Ptroglodytes
474426: .......G..........................................   Ggorilla
508848: .......G..........................................   Pabelli
487780: .......G............TG.....................C......   Mmulatta
434652: .......G............TG.....................C.T....   Panubis

                                 LTR
        ---------------------------------------------------->
             137929    137939    137949    137959    137969
                  :         :         :         :         :
137920: ATCCCTCCAGTCCTGTCTCATGATGGAATTCTCACAAGACCTGGTTGTTT   Hsapiens
457627: .....C....................C.......................   Ptroglodytes
474476: .....C....................C.......................   Ggorilla
508898: ...T.C......G.....................................   Pabelli
487830: .....-.....GT.........................------......   Mmulatta
434702: .....-.....GT.........................------......   Panubis

                                 LTR
        ---------------------------------------------------->
             137979    137989    137999              138015
                  :         :         :                   :
137970: GAAAGTGTATAGCACCTTTCCCTTTGCTCTCTCTCTTC----TGCTCCAG   Hsapiens
457677: ......................................----........   Ptroglodytes
474526: ................................A.....----........   Ggorilla
508948: ......................................CTCC....GT..   Pabelli
487873: ..............G.......................CTCC........   Mmulatta
434745: ..............G.......................CTCC........   Panubis

                                 LTR
        ---------------------------------------------------->
             138025    138035    138045    138055    138065
                  :         :         :         :         :
138016: CCACGTAGGACATGCTGTCTTCCCTCTCATGTTCTGCAGGATTGTAAGTT   Hsapiens
457723: ..................................................   Ptroglodytes
474572: ....A.............................................   Ggorilla
508998: ..................................................   Pabelli
487923: ...........G....C.................................   Mmulatta
434795: ................C.................................   Panubis
```

```
                                    LTR
         ---------------------------------------------------->
                  138075    138085    138095    138105    138115
                       :         :         :         :         :
138066: TCTGTGGCCTCCCAAGCCATGCTTCCCATATAGCCTGCAGAACCATGAGC  Hsapiens
457773: ..................................................  Ptroglodytes
474622: ............................................G.....  Ggorilla
509048: ..........................TG......................  Pabelli
487973: ....A.....................TG......................  Mmulatta
434845: ....A.....................TG......................  Panubis

                                    LTR
         ---------------------------------------------------->
                  138125    138135    138145    138155    138165
                       :         :         :         :         :
138116: CAATTAAACTTCTTTTTTTAATAAATTACCCAGCCTCAGATAGCTCTTTA  Hsapiens
457823: ..................................................  Ptroglodytes
474672: ..................................................  Ggorilla
509098: ..................................................  Pabelli
488023: .......................C......A..T.........T......  Mmulatta
434895: ..................A........................T......  Panubis

                      LTR
         ------------------------->
                  138175    138185    138195    138205    138215
                       :         :         :         :         :
138166: TAGCAATGTGAGAACAGATTAACATACAAACAGTTAACCATATGTATGAT  Hsapiens
457873: ..................................................  Ptroglodytes
474722: ..................................................  Ggorilla
509148: .....................................T............  Pabelli
488073: ......................T...T.----................C.  Mmulatta
434945: ......................T.....----................C.  Panubis

                  138225    138235    138245    138255    138265
                       :         :         :         :         :
138216: AGTATTCCTGTCATACAGAGCAAGAAACATCATGAAACTAAAAATTGAAA  Hsapiens
457923: ....................................G.............  Ptroglodytes
474772: ..................................................  Ggorilla
509198: ..................................................  Pabelli
488119: ...............A.............C....................  Mmulatta
434991: ...............A.............C....................  Panubis

                  138275    138285    138295    138305    138315
                       :         :         :         :         :
138266: AATAAAATTCTGTTATTTGTGACTTACCAATTTGCCTTAACTGATCATCA  Hsapiens
457973: ..................................................  Ptroglodytes
474822: .................................T........A.......  Ggorilla
509248: ..................................................  Pabelli
488169: ............-........C............................  Mmulatta
435041: .....................C............................  Panubis

                  138325    138335    138345    138355    138365
                       :         :         :         :         :
138316: GGAGGTTAAAAGTGATTGTATTTAAAATTTTGTTTGAAATTTTGAAGAGT  Hsapiens
458023: ..................................................  Ptroglodytes
474872: ..................................................  Ggorilla
509298: .................................................A  Pabelli
488218: ..................................................  Mmulatta
435091: ..................................................  Panubis
```

```
            138375    138385    138395    138405    138415
138366: TTAAAGAGGAATTTTGTTTCTAGATAATTTTCCATTAAATGTGATTGGAT   Hsapiens
458073: ...............A..................................   Ptroglodytes
474922: ...............A..................................   Ggorilla
509348: ........A......A..................................   Pabelli
488268: ........A..C...A..................................   Mmulatta
435141: ........A..C...A..................................   Panubis

            138425    138435    138444    138454    138464
138416: AAAATTCGGTATTTACGTCAG-TTTTTTATACAATTTCCAATAGTCAAAG   Hsapiens
458123: .....................-............................   Ptroglodytes
474972: .....................-...................C........   Ggorilla
509398: ......T.........A....T.........................T..   Pabelli
488318: ......T........T.....-............................   Mmulatta
435191: ......T........T.....-.....G......................   Panubis

            138474    138484    138494    138504    138514
138465: ACGGAAGTTAAGACTTACAAACTACTTTTGCTGTATAAATGGAAGGATAC   Hsapiens
458172: .T................................................   Ptroglodytes
475021: .T................................................   Ggorilla
509448: .T................................................   Pabelli
488367: .T..........C...........................A.........   Mmulatta
435240: ............C...........................A.........   Panubis

            138524    138534    138544    138554    138564
138515: ACAACTTCCTTTCTTATAAATGATACAGTACTCTTCTGAGAAAAACCCTT   Hsapiens
458222: ..................................................   Ptroglodytes
475071: ..................................................   Ggorilla
509498: ....A.............................................   Pabelli
488417: .A..................C.............................   Mmulatta
435290: .T..................C........................T....   Panubis

            138574    138584    138594    138604    138614
138565: ACTGTATAATCTTGTCTGCTCTATCAAAATGGGGGCTTTGTAAGTATCAT   Hsapiens
458272: ..................................................   Ptroglodytes
475121: ..................................T.........----....   Ggorilla
509548: ..................................................   Pabelli
488467: .................A.................T......----.A..   Mmulatta
435340: .................A..G...............T......----.A..   Panubis

            138624    138634    138644    138654    138664
138615: TTATGTTGAATACCTAGAACCCACACAGATGGTCAGCATCTATGTGGTAA   Hsapiens
458322: ..................................................   Ptroglodytes
475167: ..................................................   Ggorilla
509598: .................................................G   Pabelli
488513: .......T...................A......................   Mmulatta
435386: C......T...................A......................   Panubis

            138674    138684    138694    138704    138714
138665: ATGAATGACTGAATCATTTCCCTTAAGTCACTCAGGAACCCATCATTTGT   Hsapiens
458372: ......A...........................................   Ptroglodytes
475217: ..................................................   Ggorilla
509648: ....................A........G........G...........   Pabelli
488563: .....A..............T........T....................   Mmulatta
435436: ....................T........T....................   Panubis
```

```
            138724    138734    138744    138754    138764
138715: TATATGAGAACACATCTATATTTCTATTATAAAATATATTATAAACACCA  Hsapiens
458422: .....A............................................  Ptroglodytes
475267: ...............................................A..  Ggorilla
509698: ........................................G.........  Pabelli
488613: .......A....T..T....................C.........C...  Mmulatta
435486: ....C.......T..T..............................C...  Panubis

                                                 LINE2
                                                ---------->
            138774    138784    138794    138804    138814
138765: AAAATGTAGTATCACTGTGTATAGTGTTCAGTCTTATCTGCCACTCAACA  Hsapiens
458472: ..................................................  Ptroglodytes
475317: ..................................................  Ggorilla
509748: ...........................................T......  Pabelli
488663: ....G....A........................C........T......  Mmulatta
435536: ....G....A........................CG.......T......  Panubis

                           LINE2
        ------------------------------------------>
            138824    138834    138844    138854    138864
138815: ATTATTCTGCACCTACTATGTGCCACACTGGATTAGGCAATGGATTCTCA  Hsapiens
458522: ..................................................  Ptroglodytes
475367: ..................................................  Ggorilla
509798: ..................................................  Pabelli
488713: ...................................A..............  Mmulatta
435586: ..................................................  Panubis

            138874    138884    138894    138904    138914
138865: GATGTAGGCCAACAAAAAATTAGCCAAGCATGCTGAAGGCTAGAAGGGGG  Hsapiens
458572: ..................................T...............  Ptroglodytes
475417: ..................................T...............  Ggorilla
509848: ........T.......T.....A...........................  Pabelli
488763: ......................A...................C.......  Mmulatta
435636: ......................A...................C.......  Panubis
213108:            ...............G...C.G..                 Cjacchus
205888:            ...............G.T...                    Sboliviensis

            138924    138934    138944    138954    138964
138915: AACCAAATTCAGAGCACAAGTGGACCGGAACCTGGAAAAATGTCCAAATG  Hsapiens
458622: ..........................A.......................  Ptroglodytes
475467: ..........................A.......................  Ggorilla
509898: ..........G...............A.......................  Pabelli
488813: .......................G..A.....A.A...............  Mmulatta
435686: .......................G..A.....A.A...............  Panubis

            138974    138984    138994    139004    139014
138965: GTCAAAGCAGAGTCAAAGGTCTAAAACTACAATAGCAGTATAAACACAAT  Hsapiens
458672: ..................................................  Ptroglodytes
475517: ..................................................  Ggorilla
509948: ..................................................  Pabelli
488863: .....G......C.T..............T...GA...............  Mmulatta
435736: .....G......C.T..................GA...............  Panubis
```

```
                 139024    139034    139044    139054    139064
                    :         :         :         :         :
  139015: ATGTGGATGTAAGTACCTGAGATCAGGCAGAAAAGAGTTGGTGAGCAGTC  Hsapiens
  458722: ..................................................  Ptroglodytes
  475567: .......................T..........................  Ggorilla
  509998: .......................GG.........................  Pabelli
  488913: .G...............................G....G........A..  Mmulatta
  435786: .G...............................G....G........A..  Panubis

                 139074    139084    139094    139104    139114
                    :         :         :         :         :
  139065: AAAAAATCTATAAATCCACAAAACCCGGGGATTTCATAACAGAGAAATAC  Hsapiens
  458772: ..........................A.......................  Ptroglodytes
  475617: ..........................A.......................  Ggorilla
  510048: ........G.................A.......................  Pabelli
  488963: ..........................A.A..........A.......A..  Mmulatta
  435836: ..........................A.A..........A.......A..  Panubis

                 139124    139134    139144    139154    139164
                    :         :         :         :         :
  139115: TCAAAAATTCAGGAAGACATTTTGTAATAGACACAAGAGAGCAGAATTCA  Hsapiens
  458822: ..........................................G.......  Ptroglodytes
  475667: ..................................................  Ggorilla
  510098: ..................................................  Pabelli
  489013: ......G..................G........................  Mmulatta
  435886: ......G.........................GT..............T.  Panubis

                 139174    139184    139194    139204    139214
                    :         :         :         :         :
  139165: CTGAAGCAAACTTCTGTCCACCCAAGCAGCTATTTGTATGATTCCATCAA  Hsapiens
  458872: ..................................................  Ptroglodytes
  475717: ..................................................  Ggorilla
  510148: ....................A.............................  Pabelli
  489063: .....A....G.......TG..............................  Mmulatta
  435936: .....A.............G..............................  Panubis
  450192:               .A..TG.....A....................CTG.  Cjacchus
 1089493:               .A..TT...............A.....AA...CTG.  Sboliviensis

                 139224    139234    139244    139254    139264
                    :         :         :         :         :
  139215: GGATAGGGCCATCCAAGGGTATAAGAAACCCAAGGCCAAACTTTGCGTTC  Hsapiens
  458922: ..................................................  Ptroglodytes
  475767: ..................................................  Ggorilla
  510198: ..............................................C...  Pabelli
  489113: .......AT...........G...........G.............C...  Mmulatta
  435986: ........T...........G.......T...G.............C...  Panubis
  450228: ...C.......G..---------------------------.....C...  Cjacchus
 1089529: ...........G..---------------------------.....C...  Sboliviensis

                 139274    139284    139294    139304    139314
                    :         :         :         :         :
  139265: TAGGAGGTTAAAGTTTTAAGTTTGTATTTTTTCTGTTGCTGTTAATTATC  Hsapiens
  458972: ..................................................  Ptroglodytes
  475817: ..................................................  Ggorilla
  510248: .......................A..C.......................  Pabelli
  489163: ......A...........................................  Mmulatta
  436036: ......A...........................................  Panubis
  450251: .GA...........C.....G....G...C.......A........A..T  Cjacchus
 1089552: ..A.G.........C.....G....GG..........A........A..T  Sboliviensis
```

```
                                                      Simple
                                                 ---------->
             139324    139334    139344    139354    139364
  139315: TGCCATAATGGGAAGAAGCCAGAAAGACACATAATTTAAAATATTACATA  Hsapiens
  459022: .............G.............T......................  Ptroglodytes
  475867: .............GA..........A..............T.........  Ggorilla
  510298: .............G....................................  Pabelli
  489213: ....C........G............G..................G.T..  Mmulatta
  436086: ....C........G............G..................G....  Panubis
  450301: .T..C........G....AG.......T......................  Cjacchus
 1089602: ....CC.......G.C..TG....C..T............-.........  Sboliviensis

                   Simple
          -------------------------->
             139374    139384    139394              139405
  139365: AGATAATTCATATTCATATTTCATGTTAGACAACA---------AATGCA  Hsapiens
  459072: ...................................---------......  Ptroglodytes
  475917: ...................................---------......  Ggorilla
  510348: .........G......C..................---------......  Pabelli
  489263: ...................................---------......  Mmulatta
  436136: ...................................---------......  Panubis
  450351: .-----C.T.....T...A--A..A...C.T....TATTACTCA......  Cjacchus
 1089651: .-----C.T.........A--A..A...TGT....TATTATTCA......  Sboliviensis

             139415    139425    139435    139444    139454
  139406: ATTGCTTCCTCCTTAAGAACTTTGTATCTGTA-TTTTTTCTCTAATAATA  Hsapiens
  459113: ..........................C..A..-.................  Ptroglodytes
  475958: ..........................C..A..-.................  Ggorilla
  510389: .............................A..-.................  Pabelli
  489304: ..............C.......G...C..A..-.................  Mmulatta
  436177: ..............C.......G...C..A..-.................  Panubis
  450394: ....T...........T.........C..A..-.........C.......  Cjacchus
 1089694: ....T.....................AG.A..T.........C.......  Sboliviensis

             139464    139474    139484    139494    139504
  139455: ATTTTAAGAAAAACCAAAGCTTGCATTTTTTAGTGTCTTTTAAGTTGGAC  Hsapiens
  459162: ..................................................  Ptroglodytes
  476007: ..................................................  Ggorilla
  510438: ..................................................  Pabelli
  489353: ..................................................  Mmulatta
  436226: ..................................................  Panubis
  450443: .......................TG.....G..................T  Cjacchus
 1089744: ........................G.....G..................T  Sboliviensis

             139514    139524    139534    139544    139554
  139505: AGTGGTCAGACTAAAGTGAGGACCACCTTCAGATAAACTGAGCATGACAA  Hsapiens
  459212: ..................................................  Ptroglodytes
  476057: ..................................................  Ggorilla
  510488: ..................................................  Pabelli
  489403: .......................A........G..C..............  Mmulatta
  436276: .........................T......G.................  Panubis
  450493: ....A.......................C.......G.............  Cjacchus
 1089794: ....A.......................C.......G.........G...  Sboliviensis
```

```
             139564              139571    139581    139591
                  :                   :         :         :
 139555: ATGGGGCATTGTATGC-------------ACAGCCATGTTTATGACTGTT   Hsapiens
 459262: ................-------------.....................   Ptroglodytes
 476107: ................-------------.....................   Ggorilla
 510538: ................-------------.....................   Pabelli
 489453: ...CA.........T.-------------.A.T...C......C......   Mmulatta
 436326: ...CA.........T.-------------...T...C......C......   Panubis
 450543: ...A...G...G....TTTTCCCTTCAGC...................C.   Cjacchus
1089844: ...A.......G....TTTTCCCTTCAGC.............A.....C.   Sboliviensis

             139601    139611    139621    139631    139641
                  :         :         :         :         :
 139592: CCAGCAGTTGCACATCAATGACCTCCAAGGCAATGCTGAGACAGCTAGTG   Hsapiens
 459299: ..................................................   Ptroglodytes
 476144: ..................................................   Ggorilla
 510575: ..................................................   Pabelli
 489490: ............T.....................................   Mmulatta
 436363: ............T.....................................   Panubis
 450593: .......CG........GG....C.................T...C..CA   Cjacchus
1089894: ........G........G...........................C..CC   Sboliviensis

             139651    139661    139671    139681    139691
                  :         :         :         :         :
 139642: AAGATGATCCTAATGTCTCCTGTCTCCATCATAGGAAAGCTCTGCATCCT   Hsapiens
 459349: ..................................................   Ptroglodytes
 476194: ..................................................   Ggorilla
 510625: .......C..........................................   Pabelli
 489540: ...........G........................G..A..........   Mmulatta
 436413: .....T.....G.................T.........A..........   Panubis
 450643: ...........G......G....A.T..A..C..................   Cjacchus
1089944: .........................T..A.....................   Sboliviensis

             139701    139711    139721    139731    139741
                  :         :         :         :         :
 139692: GATACAGCCTATCACTGCATCTCTGATCCCATATCATCCAGAGCATGTTC   Hsapiens
 459399: ..................................................   Ptroglodytes
 476244: ..................................................   Ggorilla
 510675: A.................................T...............   Pabelli
 489590: ..................................................   Mmulatta
 436463: ..................................................   Panubis
 450693: ....TGA........................-..TC............CG   Cjacchus
1089994: ......A...........................GC............C.   Sboliviensis

             139751    139761    139771    139781    139791
                  :         :         :         :         :
 139742: TGGATCACACAAATGGTGGCTCTGGGGATAGGGAGGTTGTCCTCTCCATC   Hsapiens
 459449: ..................................................   Ptroglodytes
 476294: ..................................................   Ggorilla
 510725: ..................................................   Pabelli
 489640: .............CA...T.........A..............A......   Mmulatta
 436513: .............CA.............A..............A......   Panubis
 450742: ...T.T...........A.....T..-..G.A.......C..A.......   Cjacchus
1090044: ...T.T..G..............T.....G......G.............   Sboliviensis
```

```
                  139801    139811    139821    139831    139841
                       :         :         :         :         :
  139792: CCAAGCCCCCACCCTCCAAATACACACCTGCACAAATAAAGCAGAGTTAT  Hsapiens
  459499: ..................................................  Ptroglodytes
  476344: ..................................................  Ggorilla
  510775: ......................T...........................  Pabelli
  489690: .A......................T.........C...............  Mmulatta
  436563: .A..............A.......T.........................  Panubis
  450791: .......TT...T......T.T................C....A.....G  Cjacchus
 1090094: .......TT...T......T.T......CC.G......C.A.........  Sboliviensis

                  139851    139861    139871    139881    139891
                       :         :         :         :         :
  139842: ACAAGTGCTCAGGCATCTTAGGACCTTCTGCTGACCCCCAGAGAGTGAGC  Hsapiens
  459549: ..................................................  Ptroglodytes
  476394: ..................................................  Ggorilla
  510825: .........................................T........  Pabelli
  489740: .............TG..........................T........  Mmulatta
  436613: .............TG..........................T........  Panubis
  450841: .................C..A...........A.T......T....A.A.  Cjacchus
 1090144: ......A..........G..............A.T......T........  Sboliviensis

                  139901    139911    139920    139930    139940
                       :         :         :         :         :
  139892: TGGAAAGGAAGCAGGAAGTTTGC-ATATATGTAAATTTTGGAGCAGCTTG  Hsapiens
  459599: .......................-..........................  Ptroglodytes
  476444: .......................-..........................  Ggorilla
  510875: .......................-..........................  Pabelli
  489790: .................T....T-.........................T  Mmulatta
  436663: .....................---G..A.................A...T  Panubis
  450891: ..A.............C......-.........................T  Cjacchus
 1090194: ..A.............CT...T.A..............G..G.......T  Sboliviensis

                  139950    139960    139970    139980    139990
                       :         :         :         :         :
  139941: TTCTGCCAACACTTCTTGCAGTGACAGGTTTTGGTTGTGGTGATGACATA  Hsapiens
  459648: ..................................G...............  Ptroglodytes
  476493: ..................................G.............C.  Ggorilla
  510924: ...........................A......G...............  Pabelli
  489839: ..................................G...........T...  Mmulatta
  436710: ..................................G...............  Panubis
  450940: ................G...A...T.........G..A..-......CC.  Cjacchus
 1090244: ................G...A.............G.....G......CC.  Sboliviensis

                  140000    140010    140020    140030    140040
                       :         :         :         :         :
  139991: AAATCTACTTGGAGATGGAAAGTGCATCATAAATACAAGATCATAAGTTA  Hsapiens
  459698: ..................................................  Ptroglodytes
  476543: ..................................................  Ggorilla
  510974: ........................TG........................  Pabelli
  489889: ...C................................T.........A...  Mmulatta
  436760: ..................................................  Panubis
  450989: ........G.............C...........................  Cjacchus
 1090294: .......................A.........C................  Sboliviensis
```

```
             140050    140060    140070    140080    140090
  140041: ACATTTTCTAGACTCAAGGAATTAATCTGTGTAAGCTTAGTGAGCCAGAA  Hsapiens
  459748: ..................................................  Ptroglodytes
  476593: ..................................................  Ggorilla
  511024: ........C.........................................  Pabelli
  489939: ..................................................  Mmulatta
  436810: ..................................................  Panubis
  451039: ....A............T...-.............T...T...A......  Cjacchus
 1090344: .T..........T........-.............T....G..A......  Sboliviensis

             140100    140110    140120    140130    140140
  140091: ATATTAAACACACTTAATAAGCTTAATACGAACATTGGTGTACAGCTTCC  Hsapiens
  459798: ..................................................  Ptroglodytes
  476643: ..................................................  Ggorilla
  511074: .......G.....................A....................  Pabelli
  489989: .............................A..........G.........  Mmulatta
  436860: .............................A.........T..........  Panubis
  451088: ............T............C...A.........T......C...  Cjacchus
 1090393: ............T............C...A.........T..........  Sboliviensis

             140150              140169    140175    140185
  140141: AAGAACACTCACTTTCAAA-GCTGATGTGGAG----CTACAAATCAAACA  Hsapiens
  459848: ...................-............----..............  Ptroglodytes
  476693: ...................-............----..............  Ggorilla
  511124: ...................-............----...T..........  Pabelli
  490039: ...........T.......T............----...T....G...TG  Mmulatta
  436910: ...........T.......T............----T..T....G....G  Panubis
  451138: ..T......GG........T............ACATAC.T..........  Cjacchus
 1090443: ..T.....----.......T.....CA.....ACATAC.T.........T  Sboliviensis

             140195    140205    140215    140225    140235
  140186: GGGAAGAGAAATACCTCTTTGACCTGGTATCCACCAACATTTTAGAACTG  Hsapiens
  459893: ..................................................  Ptroglodytes
  476738: ..................................................  Ggorilla
  511169: ..................................................  Pabelli
  490085: .A................................................  Mmulatta
  436956: .AA............................................A..  Panubis
  451188: .............T.............A......................  Cjacchus
 1090489: ......................T....A......................  Sboliviensis

             140242    140252    140262    140272    140282
  140236: AT---AAGTTAACACTGATAGTCTAAGAAGGTGGCTGAGGACAGGATGGC  Hsapiens
  459943: ..---.............................................  Ptroglodytes
  476788: ..---.............................................  Ggorilla
  511219: ..---.............................................  Pabelli
  490135: .CAGA..A.......................................A..  Mmulatta
  437006: ..---..A......T...................................  Panubis
  451238: ..---..A......G....G.-..........A.............C...  Cjacchus
 1090539: ..---..AG.............-.........A.................  Sboliviensis
```

```
                                                       Simple
                                                    -------->
              140292    140302    140312    140322    140332
  140283: CATGATTTAGTTTCCAAGTAAAGTTAAAAGATTCTGCCACCACTGCTGCT  Hsapiens
  459990: ..................................................  Ptroglodytes
  476835: .......................................G..........  Ggorilla
  511266: T.................................................  Pabelli
  490185: ........G.C........G..............................  Mmulatta
  437053: ........G.C........G..............................  Panubis
  451284: ........G.................G.............T.T.......  Cjacchus
1090585: ........G.................G.............T......C..  Sboliviensis

            Simple
          -------->
              140342                                  140355
  140333: GCTGCTGCTAATGGTGGTA---------------------------GTGG  Hsapiens
  460040: ..................G---------------------------....  Ptroglodytes
  476885: ..................G---------------------------....  Ggorilla
  511316: ..................G------------------------GTG....  Pabelli
  490235: ......A.............---------GTGGTGGTGGTGATGGTG....  Mmulatta
  437103: .....C.............ATGGTGGTGATGGTGGTGGTGGTGGTG....  Panubis
  451334: ...AA..                                             Cjacchus
1090635: .........GC.AA....                                  Sboliviensis

                                          Simple
                                --------------------------->
              140365    140375                        140387
  140356: AGGTGATGGCGGCGGTCATAAAATGATG------------------ATGA  Hsapiens
  460063: .....................G......ATG---------------....  Ptroglodytes
  476908: .............A.......G......------------------....  Ggorilla
  511342: T...........T........G......ACG------ATCATGATC.C..  Pabelli
  490276: T....G...T..T.......T.......ATGATGATAATGATGATG....  Mmulatta
  437153: T....G..TT..T.......T.......ATG------ATGATAATG....  Panubis

            Simple                      MIR
          --------->      --------------------------------->
              140397    140407    140417    140427    140437
  140388: TGATGATGATAGCAAAAAAAATAGTAGTTAACATCAATTGAATACACACT  Hsapiens
  460098: ....................................G.............  Ptroglodytes
  476940: ..................................................  Ggorilla
  511386: ...G.................-........T...................  Pabelli
  490326: ......................TA............G.............  Mmulatta
  437197: ......................T...........................  Panubis

                                  MIR
          ------------------------------------------------->
              140447    140457    140467    140477    140487
  140438: GTGTCCCAGATTCTTTAATGAGCACATGCTCTATTTATTATAACTCAATA  Hsapiens
  460148: ..................................................  Ptroglodytes
  476990: ..................................................  Ggorilla
  511435: .C....................................C.......T...  Pabelli
  490376: ........T............A............................  Mmulatta
  437247: ........T............A............................  Panubis
```

```
                            MIR
        -------------------------------------------------->
                 140497    140507    140517    140527    140537
                      :         :         :         :         :
140488: AAGTAGAAATTCTTATAAAACCAATTTAAATGTTTTTAAACTGAAGCTCA  Hsapiens
460198: ......................C...........................  Ptroglodytes
477040: ......................C...........................  Ggorilla
511485: ......................C...............T...........  Pabelli
490426: .................T....C......................T....  Mmulatta
437297: .................T....C......................T....  Panubis

                  MIR
        ----------------------------->
                 140547    140557    140567    140577    140587
                      :         :         :         :         :
140538: GAAAGATAAACACAACAGCCCAAGGTCGTTCCAATTGTCTGACCCTAAAC  Hsapiens
460248: ..................................................  Ptroglodytes
477090: ..................................................  Ggorilla
511535: ...........T...............A......................  Pabelli
490476: ...........T...............A...........C..........  Mmulatta
437347: ..---......T...............A...........C..........  Panubis

                 140597    140607    140617    140627    140637
                      :         :         :         :         :
140588: TTGGCACATAGGATTTAGAGATGAACATGAACAAGATTTGTTTTTATTGC  Hsapiens
460298: ..................................................  Ptroglodytes
477140: ..................................................  Ggorilla
511585: .......................C..........................  Pabelli
490526: ..................................................  Mmulatta
437394: ..................................................  Panubis

                 140647    140657    140667    140677    140687
                      :         :         :         :         :
140638: TAAAGTAGTTGGCCATAATTTCATCTTTATGTCTGAATTAATTGTTGATT  Hsapiens
460348: ..................................................  Ptroglodytes
477190: .............AT.........T.........................  Ggorilla
511635: A....C....A..............................C........  Pabelli
490576: ........C.........................................  Mmulatta
437444: ........C.........................................  Panubis

                                                      MIR
                                                      ---->
                 140697    140707    140717    140727    140737
                      :         :         :         :         :
140688: ATTCATTTTTCATTAAAATTAGATAGCGAATAATTTTAAAGGGGGTGTTA  Hsapiens
460398: ..........................T.......................  Ptroglodytes
477240: ..........................T.......................  Ggorilla
511685: ......................G...T.........C.T...........  Pabelli
490626: .......................C..T...........T...........  Mmulatta
437494: .......................C..T...........T...........  Panubis

                            MIR
        -------------------------------------------------->
                 140747    140757    140767    140777    140787
                      :         :         :         :         :
140738: AAAGTGGTATTAGAAATCATTTATACAATTTTCTTCATTTTTGCAATTAA  Hsapiens
460448: ............................................C.....  Ptroglodytes
477290: ............................................C.....  Ggorilla
511735: .....A.......................................G....  Pabelli
490676: ...AG....................A........................  Mmulatta
437544: ...AG....................A........................  Panubis
```

```
                                       MIR
        ------------------------------------------------->
            140797    140807    140817    140827    140837
140788: AGTCCAAATAACTTAATTGCCTTCTGAGTTCGCAGTTGTCAGGGCAGAAC Hsapiens
460498: .................................................. Ptroglodytes
477340: .................................................. Ggorilla
511785: ................................................G. Pabelli
490726: ........G.T.A..................A.................. Mmulatta
437594: ........G.T.A..................A.................. Panubis

                                       MIR
        ------------------------------------------------->
            140847    140857    140867    140877    140887
140838: TGGAATAGAAACTCTAGCTTCCTGACTTCCAGCCTGGTGCTCTTCACTTG Hsapiens
460548: ...............T.................................. Ptroglodytes
477390: .................................................. Ggorilla
511835: ...........T...............C...................... Pabelli
490776: .............T.............C...................... Mmulatta
437644: .....C.......T.............C...................... Panubis

            MIR
        ---------->
            140897    140907    140917    140927    140937
140888: GCTTTCTACCCTCCACTGGGTTGATCAATTTAATGTTACAGATTCATGCT Hsapiens
460598: .......T.................................G........ Ptroglodytes
477440: .........................................G........ Ggorilla
511885: .........................................G...TA... Pabelli
490826: ..............G..................A......TG........ Mmulatta
437694: .................................A......TG........ Panubis

            140947    140957    140967    140977    140987
140938: GTCCACTTAGGAACTGATCTGTCATCCTCCAGAAGCACTGACTGCATGGG Hsapiens
460648: ......................T........................... Ptroglodytes
477490: ......................T........................... Ggorilla
511935: ...............A......T........................... Pabelli
490876: ..T...................T.......................C... Mmulatta
437744: ..T...........G.......T.......................C... Panubis

            140997    141007    141017    141027    141037
140988: CCTTGGAATTCACTCCTCTTTTGCCATGATCAACAAGTCAAATGCTGATT Hsapiens
460698: .....................A............................ Ptroglodytes
477540: .................................................. Ggorilla
511985: ...........................-.C.......C............ Pabelli
490926: .....................................C............ Mmulatta
437794: .................................................. Panubis

            141047    141057    141067    141077    141087
141038: ACACTGTGGTTCATGAATAACAGTCTATGTGGGAGTTAAATATTTCTGTT Hsapiens
460748: .................................................. Ptroglodytes
477590: .................................................. Ggorilla
512034: .............AT...................C........A...... Pabelli
490976: .......................A.......................... Mmulatta
437844: .......................A.......................... Panubis
```

```
            141097    141107    141117    141127    141137
141088: TCATTTAGGACACATATTCTAATATTCTAACATATAATTCTAACATTCTA  Hsapiens
460798: ..............C...................................  Ptroglodytes
477640: ..................................................  Ggorilla
512084: ..............................................G...  Pabelli
491026: ......................A...A....T..................  Mmulatta
437894: ......................A........T..................  Panubis
207397:                                                  .  Sboliviensis
```

```
                                                  LINE1
                                              ---------->
            141147    141157    141163    141173    141182
141138: ATATGTAATCATGTATCTATATAT----ACACACATTCTTATATATA-AT  Hsapiens
460848: ........................----...................-..  Ptroglodytes
477690: ...........C............----...................-..  Ggorilla
512134: ........................----...................-..  Pabelli
491076: ...CA...................ATAC...................-..  Mmulatta
437944: ...CA...................ATACG..................-..  Panubis
207398: G.G....TAT..AC..A.......----.......CATA...G....T..  Sboliviensis
```

```
                                 LINE1
        ------------------------------------------------->
            141192    141202    141212    141222    141232
141183: TAATATATTAAATGGTGAAAAGTCTTGACAATGTTCAAACCATATTGGAA  Hsapiens
460893: ........................................T.........  Ptroglodytes
477735: ........................................T.........  Ggorilla
512179: ........................................T....G...G  Pabelli
491125: ................................A.......T....G....  Mmulatta
437993: ........................................T....A....  Panubis
207444: AT......                                            Sboliviensis
```

```
                                 LINE1
        ------------------------------------------------->
            141242    141252    141262    141272    141282
141233: ATTTCAATAGAAAGATGGAAAGTGCAAAAGAGAAATGGTAGAAATAAAAA  Hsapiens
460943: .........A........................................  Ptroglodytes
477785: ..................................................  Ggorilla
512229: .......C..........................................  Pabelli
491175: .......C.T..........................A.C...C..-....  Mmulatta
438043: .......C.T..........................A.C...C.......  Panubis
```

```
                                 LINE1
        ------------------------------------------------->
            141292    141302    141312    141322    141332
141283: ACATAATAACAGAGATGAAAATGCCTTTGAAGCATTTACTGGTAGATTTT  Hsapiens
460993: ..................................................  Ptroglodytes
477835: ..................................................  Ggorilla
512279: .......................T...............C.........G  Pabelli
491224: ...............................A.................G  Mmulatta
438093: ...............................A.................G  Panubis
```

```
                                    LINE1
         ------------------------------------------------------>
                   141342    141352    141362    141372    141382
                        :         :         :         :         :
141333: CCATGGAGAGAATGATCTGTGACCTGAATATAGGTTCATAAAAATTACCC  Hsapiens
461043: ...A..............................................  Ptroglodytes
477885: ..................................................  Ggorilla
512329: ..................................................  Pabelli
491274: .......A.........................A................  Mmulatta
438143: .......A.........................A................  Panubis
```

```
                                    LINE1
         ------------------------------------------------------>
                   141392    141402    141412    141422    141432
                        :         :         :         :         :
141383: AAACTTCAAAACAAAGATTTTAAGAAAAGAGTAAGTGAAAATACTCACTG  Hsapiens
461093: ..................................................  Ptroglodytes
477935: ..................................................  Ggorilla
512379: .................C............A...................  Pabelli
491324: ............................A...........G.........  Mmulatta
438193: ............................A.....................  Panubis
```

```
                                    LINE1
         ------------------------------------------------------>
                   141442    141452    141462    141472    141482
                        :         :         :         :         :
141433: AGTGCCCAGGACAATAAGTGAAAATAAAGCCACATCAAAGCCCATATTGT  Hsapiens
461143: ..................................................  Ptroglodytes
477985: ..................................................  Ggorilla
512429: .A................................................  Pabelli
491374: ...........G.......A..............................  Mmulatta
438243: ...........G......................................  Panubis
```

```
                                    LINE1
         ------------------------------------------------------>
                   141492    141502    141512    141522    141532
                        :         :         :         :         :
141483: ACAATTTGAGAATACTGATGTAAAGTTTCTGAAAGCTTCAAGTGAGGGGA  Hsapiens
461193: ..................................................  Ptroglodytes
478035: ..................................................  Ggorilla
512479: ...................A.........C.C.........A........  Pabelli
491424: .A.............................CG.........CA......  Mmulatta
438293: .A.............................C..........CA......  Panubis
```

```
                                    LINE1
         ------------------------------------------------------>
                   141542    141552    141562    141572    141582
                        :         :         :         :         :
141533: AAAAATGCAAATCACACATAAGGAACCTGAGTGGATGCAAAATTCTCCAC  Hsapiens
461243: ..................................................  Ptroglodytes
478085: ..................A......T........C...............  Ggorilla
512529: .....G............................................  Pabelli
491474: .....GAG..............A...T......A................  Mmulatta
438343: .....GAG..............A...T......A................  Panubis
```

```
                                    LINE1
         ---------------------------------------------------------->
              141592    141602    141612    141622    141632
141583: GGCAAGAGAGAAAGAGGACAATAAAGCAACATCTTTTAAAAATTGTGAAG Hsapiens
461293: ....................................C............. Ptroglodytes
478135: .................................................. Ggorilla
512579: A......................G.......................... Pabelli
491524: T......................G.A......T....TTT.......... Mmulatta
438393: T......................G.A......T....-TT.......... Panubis

                                    LINE1
         ---------------------------------------------------------->
              141641    141651    141661    141671    141681
141633: G-AAAAAATTATTTTCCACTTAGAATTCCAAACTCAGAGAAATACCAATC Hsapiens
461343: .-......A......................................... Ptroglodytes
478185: .-................................................ Ggorilla
512629: .A.......G..................A..................... Pabelli
491574: .--......G.....................................G.. Mmulatta
438442: .--......G..........................A.......T..G.. Panubis

                                    LINE1
         ---------------------------------------------------------->
              141691    141701    141711    141721    141731
141682: AAATGTGAGGGTAGAATATAGACATATATAGACATATATAGACATGCAAA Hsapiens
461392: ...............----------...............C.....G... Ptroglodytes
478234: ...............----------......G.G............G... Ggorilla
512679: ...............----------.............C.......G... Pabelli
491622: ...............----------............TC.A.....G... Mmulatta
438490: .........A.....----------............TC.A.....G... Panubis

                                    LINE1
         ---------------------------------------------------------->
              141741    141751    141761    141771    141781
141732: ATCTCAAAAATGTATCTCATATCCATTCTTCCTCAGGAGGCTACTGAAGG Hsapiens
461432: .........................C.T...................... Ptroglodytes
478274: .....G............................................ Ggorilla
512719: G................................................. Pabelli
491662: G.T.....................T......................... Mmulatta
438530: G.T.....................T...............T......... Panubis

                                    LINE1
         ---------------------------------------------------------->
              141791    141801    141811    141821    141831
141782: ACATGTTCTATCAAATAAGAGAGTAAATCAAGACACAGGAGACAACAGAA Hsapiens
461482: ........C....................................T.... Ptroglodytes
478324: ........C....................................T.... Ggorilla
512769: ........C.........A..........................T.... Pabelli
491712: ........C.......G.A..........................T.AG. Mmulatta
438580: ........C.......G.A..........................T.AG. Panubis
```

```
                          LINE1
        ------------------------------------------------->
            141841    141851    141861    141871    141881
141832: AAGAGGATAGATACCCAGTATAGGAGTAAGGCAAAGGAAATCTCCAAAAG  Hsapiens
461532: ..................................................  Ptroglodytes
478374: ..................................................  Ggorilla
512819: ..................................................  Pabelli
491762: .................A................................  Mmulatta
438630: .................A................................  Panubis

                          LINE1
        ------------------------------------------------->
            141891    141901    141911    141921    141931
141882: GCAGTAAAAAGAGATCCTAAGATGACAACTATTGACCAGAGAGTGTACCA  Hsapiens
461582: ..........................C.......................  Ptroglodytes
478424: ..................................................  Ggorilla
512869: ..................................................  Pabelli
491812: .....-G......T..............................A.....  Mmulatta
438680: ..G..-G......T..............................A.....  Panubis

      LINE1
        --->
            141941    141951    141961    141971    141981
141932: ATCAGCGTATATCAGAGAATATGATTTAAGGAAAAAGCAACACATGGATG  Hsapiens
461632: ......A...........................................  Ptroglodytes
478474: ......A...........................................  Ggorilla
512919: ......A...................................A.......  Pabelli
491861: ......A..C......A......................GT....-....  Mmulatta
438729: ......A..C......A......................G.....-....  Panubis

            141991    142001    142011    142021    142031
141982: ACTATCATTTCCAAGATCACTGCTGCAATAGTAACATGCTTTGATCTTCA  Hsapiens
461682: ..................................................  Ptroglodytes
478524: ..................................................  Ggorilla
512969: ................................................G.  Pabelli
491910: ............................C....C....T.........G.  Mmulatta
438778: .................................CTG..T.........G.  Panubis

            142041    142051    142061    142071    142081
142032: GGACAGGATGGCCAGCTGATATTGACTCAGAGTCTTATCTACACTTGGCT  Hsapiens
461732: ..................................................  Ptroglodytes
478574: ................................................T.  Ggorilla
513019: .....................C...................G........  Pabelli
491960: .........A..........T.........C................AT.  Mmulatta
438828: ....................T....T....CA...............AT.  Panubis

            142091    142101    142111    142121    142131
142082: TGTCTTGACCGCGTGCTGACAAAATTCCTGCTCTTCATACCCTGATGCCT  Hsapiens
461782: ..................................................  Ptroglodytes
478624: ...........T................................C.....  Ggorilla
513069: G............................A....................  Pabelli
492010: .........T.T.................A..........T.........  Mmulatta
438878: ...........T.................A..........G.........  Panubis
```

```
                142141     142151     142161     142171     142181
                     :          :          :          :          :
142132: GGATCAGCTAAGAATACTCATTTCCCCACAGATGCTTCTGTGAGTTGGAA  Hsapiens
461832: ..................................................  Ptroglodytes
478674: ................................C.................  Ggorilla
513119: -...............................A..........A......  Pabelli
492060: .....G.TC.......................A..........A......  Mmulatta
438928: .......TC..T...........T........A..........A......  Panubis

                142191     142201     142211     142221     142231
                     :          :          :          :          :
142182: GTAGGCCAAGGTGAGCTTAAAAGCCAAAATCCCAGAGCAGCTCCATCTGT  Hsapiens
461882: ..............A...................................  Ptroglodytes
478724: ..................................................  Ggorilla
513168: ...........C......................................  Pabelli
492110: ......T................TTG............G...........  Mmulatta
438978: ......T................TTG.C......C...............  Panubis

                142241     142251     142261     142271     142281
                     :          :          :          :          :
142232: AACCTACTCTACCAAATAATCAGTCCCTTGGACACACTAAACACTTGACT  Hsapiens
461932: ..................G...............................  Ptroglodytes
478774: ..................G.......T.......................  Ggorilla
513218: ....C.............G....................C..G....C..  Pabelli
492160: ......T...........G....................T..G....C..  Mmulatta
439028: ..................G....................T.......C..  Panubis

                142291     142301     142311     142321     142331
                     :          :          :          :          :
142282: GATGCTGCAACTGTAGAGAAACCTACACGTCCCAAGATCCTTCATTACTT  Hsapiens
461982: .....C.........C............T.....................  Ptroglodytes
478824: .....C......................T.....................  Ggorilla
513268: .....C....A..............TG.T........G............  Pabelli
492210: .....CA................C.TG.T.....................  Mmulatta
439078: .....C.................C.TG.T.....................  Panubis

                142341     142351     142361     142371     142381
                     :          :          :          :          :
142332: CAGCCACTGACTACTTCAAAGGGGCCTCTTGGAAATGCTCAGATAGGCTC  Hsapiens
462032: ...........................................A......  Ptroglodytes
478874: ...........................................A......  Ggorilla
513318: ...........................................C......  Pabelli
492260: ............G...........T..................A......  Mmulatta
439128: ........................T..................A......  Panubis

                142391     142401     142411     142421     142431
                     :          :          :          :          :
142382: AGGGCCCTGCAATTCTGCGTACGTCATTTACCCTGTGAAGGTTACCCAGG  Hsapiens
462082: ..................................................  Ptroglodytes
478924: ..................................................  Ggorilla
513368: -A................................................  Pabelli
492310: -....T............T.....................A.........  Mmulatta
439178: -.C..T............T............G........A.........  Panubis

                142441     142451     142461     142471     142481
                     :          :          :          :          :
142432: AATCTTCATCTAATAGTGGAGACAGTGGCCTTACCTTATATGGCTAGGTT  Hsapiens
462132: ........................................C.........  Ptroglodytes
478974: .....C............................................  Ggorilla
513417: ..C...............................................  Pabelli
492359: ..C...............................A..G............  Mmulatta
439227: ..C..........C....................A...............  Panubis
```

```
            142491    142501    142511    142521    142531
                 :         :         :         :         :
142482: TGTGCTTACTACTCAGTGTTTTCAACTAATTGGCACATTATTTAGGAGTA  Hsapiens
462182: ..................................................  Ptroglodytes
479024: ........................G.........................  Ggorilla
513467: ..CT...............................G..............  Pabelli
492409: ..........................C.......................  Mmulatta
439277: ..........................C.......................  Panubis

            142541    142551    142561
                 :         :         :
142532: CATTCGTAGTTGGTAAAACTATTTTTAAAAATAGG---------------  Hsapiens
462232: ...................................---------------  Ptroglodytes
479074: ...................................---------------  Ggorilla
513517: .....A......A.....GC...............AATCCCCT-------  Pabelli
492459: .....A......A......................AATCGATTAAATCAG  Mmulatta
439327: .....A......A......................AATCGATTAAATCAG  Panubis

                                                Alu
                                             ------------->
                                        142569    142579
                                             :         :
142567: -------------------------------------AATCCCAGCACTT  Hsapiens
462267: -------------------------------------.............  Ptroglodytes
479109: -------------------------------------.............  Ggorilla
513560: -----------GGCCGGGCGCGGTGGCTCACGCCTGT.............  Pabelli
492509: AAGTCAATATAGGCTGGGTGCAGTGGCTCATGCCTTT.....T.......  Mmulatta
439377: AAGTCAATATAGGCTGGGTGCAGTGGCTCATGCCTTT.....T.......  Panubis

                              Alu
        ------------------------------------------------->
            142589    142599    142607    142617    142627
                 :         :         :         :         :
142580: TGGGAGGCCGACGCGGGCGGATCAC--GAGATCAGGAGATCGAGACCATC  Hsapiens
462280: ...........G.............--.......................  Ptroglodytes
479122: ...........G.....T.......--.......................  Ggorilla
513599: ...........G.....T.......--...G...................  Pabelli
492559: C........A.G..A---....TG.TT.......A.T.T..A....T.-.  Mmulatta
439427: C........A.G..A---....TG.TT.......A.T.T..A....T.-.  Panubis

                              Alu
        ------------------------------------------------->
            142637    142647    142657    142667    142677
                 :         :         :         :         :
142628: CTGGCTAACACGGTGAAACCCCGTCTCTACTAAAAATACAAAAAATTAGC  Hsapiens
462328: ....................................C.............  Ptroglodytes
479170: ...................................-C.....-.......  Ggorilla
513647: ..........T........................G..............  Pabelli
492605: A...GC....TA.CA.G..TTTC...............A...T..       Mmulatta
439473: A...GC....TA.CA.G..TTTC...............A...T..       Panubis

                              Alu
        ------------------------------------------------->
            142687    142697    142707    142717    142727
                 :         :         :         :         :
142678: CGGGCGTGGTGGCGAGCGCCTGCAGTCAGTCGCAGCTACTCGGCAGGCTG  Hsapiens
462378: ..................................................  Ptroglodytes
479218: ........                                            Ggorilla
513697: ......CA......G.......----T....T........G..G......  Pabelli
```

```
                               Alu
            142737    142747    142757    142767    142777
142728: AGGCGGTAGAATGGCGTGAACCCGGGAGGCAGAGCTTGCAGTGAACCGAG  Hsapiens
462428: ....A.............................................  Ptroglodytes
513743: ...AA.G.......................G.............G.....  Pabelli
```

```
                               Alu
            142787    142797    142807    142817    142827
142778: ATTGCGCCACTGCACTCCAGCCTGGGGGACAGAGCAAGACTCCGTCTCAA  Hsapiens
462478: ..........................A.......................  Ptroglodytes
479356:                                   ................  Ggorilla
513793: ...................T.T....C................AA.....  Pabelli
```

```
          Alu                                   Alu
            142837    142846    142856    142866    142876
142828: AAAAAAACTAGG-AATCGATTAAATCAAAAGTCAACATAGGCTGGGTGCA  Hsapiens
462528: ............-.....................................  Ptroglodytes
479372: ............-.....................................  Ggorilla
513843: .......A..AAT..ATA.A.....A....                      Pabelli
518395:                               .....T..............  Pabelli
492485:    ....A....-..............G.......T..............  Mmulatta
439353:    ....A....-..............G.......T..............  Panubis
```

```
                               Alu
            142886    142896    142906    142916    142926
142877: GTGGCTCATGCCTATAATCCCAGCACTTCGGGGGGCCAAGGCAGGATTGC  Hsapiens
462577: ..................................................  Ptroglodytes
479421: ..................................................  Ggorilla
518415: ................................A.................  Pabelli
492531: .............T......T...........A.................  Mmulatta
439399: .............T......T...........A.................  Panubis
```

```
                               Alu
            142936    142946    142956    142966    142976
142927: TTGAGCCCAAGTGTTCAAGACCACCTGGGCAACATAGCAAGACTTTCTCT  Hsapiens
462627: ......G...........................................  Ptroglodytes
479471: ......G...........................................  Ggorilla
518465: ......G...........................................  Pabelli
492581: .....AT..............T..A.........................  Mmulatta
439449: .....AT..............T..A.........................  Panubis
```

```
                 Alu
            142986    142996    143006    143016    143026
142977: CTACTAAAAATAAAAATAAAATGCATATAGTATATATCTGCAGGAAGCAA  Hsapiens
462677: ...............................G..................  Ptroglodytes
479521: ...............................G..................  Ggorilla
518515: .......................A......C....G..............  Pabelli
492631: .......................A......C.....C..A..........  Mmulatta
439499: .......................A......C.....C.............  Panubis
```

```
                 143036    143046    143056    143066    143076
                      :         :         :         :         :
143027: TGTATGGAGAGACAGATTCTGAGGCCAATTTATTGACCTGTTGGAAAGAT  Hsapiens
462727: ..................................................  Ptroglodytes
479571: ............................................T.....  Ggorilla
518565: ............G.....................................  Pabelli
492681: ............A........T............................  Mmulatta
439549: ............G........T............................  Panubis
```

```
                                                      Simple
                                                    -------->
                 143086    143096    143106    143116    143126
                      :         :         :         :         :
143077: ATATGGGAAATTATTTATTATCACTATTCTTATTAAACTGTATATATACA  Hsapiens
462777: ...........................A......................  Ptroglodytes
479621: ..................................................  Ggorilla
518615: ..................................................  Pabelli
492731: .A...A.................T........................T.  Mmulatta
439599: .....A....G............T........................T.  Panubis
```

```
                                Simple
        ------------------------------------------------->
                 143136    143146    143156    143166    143176
                      :         :         :         :         :
143127: CACACACATATATATACTTCTGTACATATTCATTGTATATATTTTTAAAT  Hsapiens
462827: ...T..............................................  Ptroglodytes
479671: .......--.........................................  Ggorilla
518665: ..................................................  Pabelli
492781: ..................................................  Mmulatta
439649: ........................T.........................  Panubis
```

```
                                     LINE1
                    ------------------------------------->
                 143186    143196    143206    143216    143226
                      :         :         :         :         :
143177: CAAGGAAAATTTAGAATACCACAGTATGATTAAATGGGCTTATTCCAGAT  Hsapiens
462877: ...A..............................................  Ptroglodytes
479719: ..................................................  Ggorilla
518715: ..........................C.................G.....  Pabelli
492831: ..............G........T..........................  Mmulatta
439699: ..............G........T..........................  Panubis
```

```
                                LINE1
        ------------------------------------------------->
                 143236    143246    143256    143266    143276
                      :         :         :         :         :
143227: AGCCATATTAGAAAATATATTAATATACTTTACCATATTAAAAGATCTCA  Hsapiens
462927: ..................................................  Ptroglodytes
479769: ..................................................  Ggorilla
518765: ........................................G.........  Pabelli
492881: ..................................................  Mmulatta
439749: ..................................................  Panubis
```

```
                  LINE1                   DNA
        -------------------->---------------------------->
                 143286    143296    143306    143316    143326
                      :         :         :         :         :
143277: GGGGGAAGTCTCTACGGAATTGCACTGTCCATACAGTAATCGTTATTCAC  Hsapiens
462977: ..............T...................................  Ptroglodytes
479819: ..................................................  Ggorilla
518815: ...............................................A.T  Pabelli
492931: ...............A..................................  Mmulatta
439799: ...............A..................................  Panubis
```

```
                            DNA
        --------------------------------------------------->
                 143336    143346    143356    143366    143376
143327: ATGTGGCTATTTAAATTTAAATAATTAACATTAAGCAAATTAAAAGTACA  Hsapiens
463027: .........C........................................  Ptroglodytes
479869: ..................................................  Ggorilla
518865: ........-......................................G..  Pabelli
492981: ......G...........................A...............  Mmulatta
439849: ..................................A...............  Panubis
```

```
                            DNA
        --------------------------------------------------->
                 143386    143396              143410    143420
143377: CTTCCTTTGTCTCACTAGTCACATGTCA------ATACCCACCTACGCCT  Hsapiens
463077: A.................C.........------................  Ptroglodytes
479919: A.................C.........------................  Ggorilla
518914: A.................C.........------................  Pabelli
493031: A.................C.........CTAGGTG...T.......A...  Mmulatta
439899: A.................C.........------G...T.......A...  Panubis
```

```
                            DNA
        --------------------------------------------------->
                 143430    143440    143450    143460    143470
143421: AGCAGCTATTGTACTGAACCATGTAATTATTGAACGTTTTCATCATCACA  Hsapiens
463121: ..................................T...............  Ptroglodytes
479963: ..................................................  Ggorilla
518958: ...................T..............................  Pabelli
493081: ...................T.........C.....A.........C....  Mmulatta
439943: .....T.............T.........C.....A.........C....  Panubis
```

```
                 DNA                         LINE1
        ----------------------->---------------------------->
                 143480    143490    143500    143510    143520
143471: GAGCATTCTATGAGACAGCACTGCTATATAGATCCTGGAAAATTATGTGA  Hsapiens
463171: ........................--........................  Ptroglodytes
480013: ........................--........................  Ggorilla
519008: ........................--....T.................A.  Pabelli
493131: ........................--........................  Mmulatta
439993: ........................--........................  Panubis
```

```
                            LINE1
        --------------------------------------------------->
                 143530    143540    143550    143560    143570
143521: CAAAATTTAATATATATGTTGTATAAAAGCTAGTAATGAAATAAAATAAA  Hsapiens
463219: ..................................................  Ptroglodytes
480061: .................A.............G..................  Ggorilla
519056: ..................................................  Pabelli
493179: ...................G.................A............  Mmulatta
440041: ...................G.................A............  Panubis
```

```
                                LINE1
        ------------------------------------------------->
            143580    143590    143600    143610    143620
143571: ATAGAAAGAAATTGACTCTTTAACATATTAAAGTGTAGCCATCTCAATTT  Hsapiens
463269: ..................................................  Ptroglodytes
480111: ................................C.................  Ggorilla
519106: .........C........................................  Pabelli
493229: .C.......G.....T.............G.........TG.........  Mmulatta
440091: .C.......G.....T.............G.........TG.........  Panubis
```

```
                    LINE1
        ------------------------->
            143630    143640    143650    143660    143670
143621: CAAAGCCAAAATCAGGTTTAATAGTTTCTAAAAGCAGTTAGAAACACAAG  Hsapiens
463319: ..............................G...................  Ptroglodytes
480161: ..............................C...................  Ggorilla
519156: ......................C.......G...................  Pabelli
493279: ...........................T..G.....T.............  Mmulatta
440141: ..............................G.....T.............  Panubis
```

```
            143680    143690    143700    143710    143720
143671: AAGCATTGAATTCAAAGGATAAAAAAGAACACTTGTATTACTATTATTTT  Hsapiens
463369: ..................................................  Ptroglodytes
480211: ........C.........................................  Ggorilla
519206: ..................................................  Pabelli
392285:                           .......A................  Nleucogenys
493329: .................................A.C.........G....  Mmulatta
440191: .................................A.C.........G....  Panubis
```

```
                                              LINE1
                                      ---------------------->
            143730    143740    143750    143760
143721: TATCAGTACCGAAGAATGAATTGGTAAAAAATAAAAAAATT---------  Hsapiens
463419: ........................................--AAAAAATT  Ptroglodytes
480261: .........................................TAAAAAATT  Ggorilla
519256: ..............G..........................TAAAAAATT  Pabelli
392309: .........T...............C...............AAAAAAAAG  Nleucogenys
493379: ..........A...........CA.......A......GA----------  Mmulatta
440241: ..........A...........CA.......A....G..AAGAAA-----  Panubis
```

```
                                LINE1
        ------------------------------------------------->
            143771    143781    143791    143801    143811
143762: AAAGAGGAGATTTGTTTATATGGTGTGATAACATTTTTGTTAAAAAGACA  Hsapiens
463467: ..G...............................................  Ptroglodytes
480311: ....................................C.............  Ggorilla
519306: ...A..........................C...................  Pabelli
392359: ..............................C................G..  Nleucogenys
493419: .....................T........C................G..  Mmulatta
440286: .....A...............T........C...............AG..  Panubis
```

```
                              LINE1
        ------------------------------------------------->
            143820    143830    143840    143850    143860
143812: AAAAAAA-TCTGTGCACATGTATTTGTGTGTACACATATAACATATCTCT  Hsapiens
463517: .......A..........................................  Ptroglodytes
480361: .......A..........................................  Ggorilla
519356: .......-.........G................................  Pabelli
392409: .......--.........................................  Nleucogenys
493469: .......A.....A....................T...............  Mmulatta
440336: .......-.....A....................................  Panubis

                              LINE1
        ------------------------------------------------->
            143870    143880    143890    143900    143910
143861: GAAGGAATATATAAGAAACAACACCATGTGGTCCAGGGCAGGGTAACTGA  Hsapiens
463567: ..................................................  Ptroglodytes
```
[truncated: 988,966 more chars]
