# Supplementary material for: Adaptive Evolution and Divergence of SERPINB3: A Young Duplicate in Great Apes
Source: PLoS One. 2014 Aug 18;9(8):e104935. doi: 10.1371/journal.pone.0104935 (PMC4136820; doi:10.1371/journal.pone.0104935)
Supplement: Table S1 — Genomic locations for the DNA sequences retrieved from the National Center for Biotechnology Information database (NCBI) and University of California Santa Cruz (USCS) Genomic Bioinformatics database for the nine primate species. (DOCX) [file pone.0104935.s007.docx]

Table S1: Genomic locations for the DNA sequences retrieved from the National Center for Biotechnology Information database (NCBI) and University of California Santa Cruz (USCS) Genomic Bioinformatics database for the nine primate species.

| Species | Gene | Source | Chromosomal region |
| --- | --- | --- | --- |
| *Homo sapiens* | *SERPINB3/B4* | NCBI | chr18: c61429197-61222431 |
|  | *CTSS/K* | NCBI | chr1: c150900000-150600000 |
|  | *CTSL1* | NCBI | chr9: 90330000-90470000 |
|  | *CTSL2* | NCBI | chr9: c99900000-99700000 |
|  | *CTSG/ CMA1* | NCBI | chr14: c25120000-24970000 |
| *Pan troglodytes* | *SERPINB3/B4* | NCBI | chr18: 59524289-60190903 |
|  | *CTSS/K* | NCBI | chr1: c129350000-128600000 |
|  | *CTSL1* | NCBI | chr9: 86550000-87150000 |
|  | *CTSL2* | NCBI | chr9: c96250000-95670000 |
|  | *CTSG/CMA1* | NCBI | chr14: c23700000-23300000 |
| *Gorilla gorilla* | *SERPINB3* | USCS | chr18: 61632792-62339554 |
|  | *CTSS/K* | USCS | chr1: c129600000-12970000 |
|  | *CTSL1* | NCBI | chr9: 69700000-70300000 |
|  | *CTSL2* | NCBI | chr9: c79400000-78900000 |
|  | *CTSG/CMA1* | NCBI | chr14: c5800000-5400000 |
| *Pongo abelli* | *SERPINB3/B4* | USCS | chr18: 76236327-76964735 |
|  | *CTSS/K* | NCBI | chr1: 100500000-101200000 |
|  | *CTSL1* | NCBI | chr9: 82950000-83140000 |
|  | *CTSL2* | NCBI | chr9: c93200000-92600000 |
|  | *CTSG/CMA1* | NCBI | chr14: c24400000-23900000 |
| *Nomascus leucogenys* | *SERPINB3/B4* | NCBI | chr4: c16670000-16050000 |
|  | *CTSS/K* | NCBI | chr12: 51150000-51800000 |
|  | *CTSL1* | NCBI | Chr1a: c47070000-46650000 |
|  | *CTSL2* | NCBI | chr1a: 38200000-38460000 |
|  | *CTSG/CMA1* | NCBI | Chr22a: 39500000-39900000 |
| *Macaca mulatta* | *SERPINB3/B4* | USCS | chr18: 57715350-58415478 |
|  | *CTSS/K* | NCBI | chr1: c129600000-128600000 |
|  | *CTSL1* | NCBI | chr15: 96220000-96950000 |
|  | *CTSL2* | NCBI | chr15: c110000000-108450000 |
|  | *CTSG/CMA1* | NCBI | chr7: c87800000-87200000 |
| *Papio anubis* | *SERPINB3/B4* | NCBI | chr18: 55670000-56330000 |
|  | *CTSS/K* | NCBI | chr1: c124850000-124000000 |
|  | *CTSL1* | NCBI | chr15: 94130000-94700000 |
|  | *CTSL2* | NCBI | chr15: c106930000-105900000 |
|  | *CTSG/CMA1* | NCBI | chr7: c81600000-81100000 |
| *Callithrix jacchus* | *SERPINB3/B4* | USCS | chr13: 101351650-102019314 |
|  | *CTSS/K* | NCBI | chr1: c5200000-4200000 |
|  | *CTSL1* | NCBI | chr1: 70570000-71450000 |
|  | *CTSL2* | NCBI | chr1: c77700000-76700000 |
|  | *CTSG/CMA1* | NCBI | chr10: c48800000-48400000 |
| *Saimiri boliviensis* | *SERPINB3/B4* | NCBI | Chromosome unknown (GI:395725132): c15500000-14200000 |
|  | *CTSS/K* | NCBI | Chromosome unknown (GI: 395721992): 2400000-3700000 |
|  | *CTSL1/L2* | NCBI | Chromosome unknown (GI:395722143): 3430000-3990000 |
|  | *CTSG/CMA1* | NCBI | Chromosome unknown (GI:395725602):13650000-14000000 |
